# A multi-level machine learning pipeline for prediction and prioritization of hybrid insulin peptides in type 1 diabetes

**DOI:** 10.3389/fimmu.2026.1917213

**Published:** 2026-08-07

**Authors:** Ilya Kandinov, Dmitry Trukhin, Dmitry Gryadunov, Elena Savvateeva

**Affiliations:** Center for Precision Genetic Technologies for Medicine, Engelhardt Institute of Molecular Biology, Russian Academy of Sciences, Moscow, Russia

**Keywords:** bioinformatics, hybrid insulin peptides, machine learning, neoepitopes, peptide prioritization, regression model, type 1 diabetes, XGBoost

## Abstract

Hybrid insulin peptides (HIPs) are neoepitopes involved in type 1 diabetes (T1D), but their complete repertoire remains unknown. The vast combinatorial space makes experimental screening unfeasible and requires bioinformatics-based prioritization. We developed a multi-level machine learning pipeline for ranking HIP candidates. First, 36 physicochemical and junction-specific features were computed for a reference library of 240 HIPs with known enzyme-linked immunospot (ELISPOT) reactivity, and a baseline Ridge regression model was trained. Next, all possible HIP candidates with 7–9 amino acid residues per fragment were generated from eight pancreatic β-cell secretory granule source proteins, including insulin chains and C-peptide, islet amyloid polypeptide, chromogranin A, neuropeptide Y, and two secretogranins, yielding 1,057,374 candidates. For each source protein, a local weighted XGBoost (Extreme Gradient Boosting) model was trained using Ridge-score-derived pseudo-labels together with weighted ELISPOT-derived and literature-derived reference HIPs. Finally, anchor-calibrated re-ranking was performed in the global model using cosine similarity to positive anchors (n = 46) and negative anchors (n = 210). The Ridge model achieved 5-fold out-of-fold R² = 0.711 and an area under the receiver operating characteristic curve (AUC) of 0.967. The global model produced a prioritized list of 40 HIP candidates, five per source protein. The highest ranks were observed for candidates with right fragments from neuropeptide Y, secretogranins 1 and 2, islet amyloid polypeptide, and chromogranin A. Candidates carrying the insulin fragment on the right side were systematically down-ranked, suggesting asymmetry in HIP formation. The proposed pipeline reduces the HIP search space from more than one million sequences to a limited set of candidates for experimental validation and provides a framework adaptable to other chimeric neoepitopes in autoimmunity.

## Introduction

Type 1 diabetes (T1D) is an autoimmune disease characterized by T-cell-mediated destruction of insulin-producing pancreatic β-cells. Hybrid insulin peptides (HIPs) are chimeric molecules formed in the secretory granules of β-cells through covalent linkage of fragments of insulin and its precursors with fragments of other granule proteins. The identification of HIPs as targets of autoreactive CD4+ T cells revealed a new component in the pathogenesis of T1D (1, 2). In a broader context, neoepitope formation in T1D, including HIPs, can be considered within the modern concept of immunometabolism. According to this concept, metabolic stress in β-cells and antigen-presenting cells is thought to create a self-sustaining cycle underlying autoimmune inflammation (3). Thus, HIP formation may represent not an isolated event but one manifestation of a fundamental process in which metabolic disturbances in β-cells and immune cells mutually reinforce each other, potentially forming a pathogenic axis of the disease.

HIPs were first described in 2016 by Delong et al. (4). Using the non-obese diabetic (NOD) mouse model and mass spectrometry analysis, the authors demonstrated that pathogenic CD4+ T cells recognize chimeric peptides formed from fragments of proinsulin and secretory granule proteins, such as chromogranin A (CHGA) (2.5HIP) and amylin (islet amyloid polypeptide, IAPP). The presence of HIPs was subsequently confirmed in pancreatic islets (5), and HIP-reactive T cells were detected in the peripheral blood of patients with T1D (6). Crystallization of the HLA-DQ8/HIP/TCR complex further confirmed the molecular feasibility of hybrid peptide presentation (7).

Over the decade since HIPs were identified, the field has progressed from their initial description to the recognition of HIPs as key contributors to the pathogenesis of T1D. Elucidating the molecular mechanisms of HIP formation has become an important area of research. Reed et al. demonstrated that cathepsin L can catalyze transpeptidation leading to the formation of chimeric epitopes under conditions that mimic the lysosomal environment (8). Subsequently, Crawford et al. identified cathepsin D as a key protease responsible for HIP formation in β-cells, thereby explaining how covalent linkage of proinsulin fragments with other proteins can occur in the acidic environment of secretory granules (pH 5.5) (9). In addition to enzymatic pathways, an alternative mechanism of spontaneous HIP formation through an aspartic anhydride intermediate was later identified. This process results in the formation of both conventional HIPs and their isomers. Both types of molecules were detected by mass spectrometry in islets from NOD mice, supporting the existence of this mechanism *in vivo* (10).

In parallel with studies of the mechanisms of HIP formation, considerable attention has been paid to the immune response to these neoantigens, including the characterization of HIP-reactive T cells (11). It is currently assumed that, in the acidic environment of secretory granules, HIPs are stabilized through binding to insulin and zinc. Owing to their unusual resistance to proteolysis, they do not degrade like most peptide fragments but instead accumulate within the granules. Upon exocytosis, HIPs are released, subsequently taken up by antigen-presenting cells, and presented on MHC-II (12). Thus, a key feature of HIPs is that, despite their intracellular origin, they behave as exogenous antigens. This property, together with the presumed absence of central tolerance to the peptide junction region (13), allows HIPs to initiate a CD4+ T-cell response directed against both insulin and other β-cell antigens.

Experimental approaches using peptide libraries containing more than one hundred HIP candidates, or combinatorial libraries of random peptides, are currently available. For example, Hohenstein et al. assessed T-cell reactivity in patients with T1D and at-risk individuals by IFN-γ ELISPOT using a library of 240 C-peptide-based hybrid peptides. This allowed the authors to identify immunogenic HIP variants formed at amino acid residues G15, A18, and L26 of C-peptide and, importantly, to demonstrate the presence of a T-cell response against HIPs at the preclinical stage (14). In a mouse model, Parras et al. developed an approach based on combinatorial libraries of random peptides to identify unknown antigens recognized by T-cell receptors. Using this approach, the authors showed that a single pathogenic T-cell receptor can recognize several distinct HIPs (15).

At the same time, the high dimensionality of possible HIP variants supports the use of bioinformatics approaches for *in silico* selection of the most likely HIP candidates for subsequent experimental validation. Additional support for bioinformatics approaches comes from data on the HLA specificity of HIP9, which is presented by HLA-DR11 rather than HLA-DQ8. This suggests that the HIP repertoire may depend on the HLA haplotype and highlights the need to assess peptide binding to different HLA alleles associated with disease risk (16).

Although only a few dozen HIPs have been characterized to date, the accumulated evidence suggests that they form a heterogeneous family of neoantigens whose spectrum is likely to expand during disease pathogenesis. Secretory granules of pancreatic β-cells contain more than 200 different proteins, each of which could theoretically participate in HIP formation (17). If all possible combinations of insulin fragments (A-chain, B-chain, and C-peptide) with fragments of other proteins are considered, together with variability in the length of the junction region, typically 7–12 amino acid residues on each side, the total number of potential HIPs exceeds one million. Experimental testing of all these candidates by mass spectrometry or T-cell assays is not feasible. Moreover, standard bioinformatic tools for predicting T-cell epitopes, such as NetMHCpan, IEDB, and MixMHCpred, were developed for linear peptides and do not account for the chimeric nature of HIPs, particularly the covalent junction point between the two fragments, which represents the key neoepitope recognized by autoreactive T cells (18, 19). Direct application of these methods to HIPs would lead to the loss of information about the structure of the junction region, which, as shown in the present study, is the most important feature of HIP formation.

Thus, given the large number of potential source proteins and their possible combinations, the identification of new clinically relevant HIPs is an extremely challenging task. It cannot be reduced to a simple immunogenic/non-immunogenic classification, because the number of experimentally characterized HIPs remains limited, whereas the number of possible candidates is much larger. Under these conditions, simple models, including linear regression or individual decision trees, can provide only a limited assessment and do not reliably identify the most promising sequences.

To address this task, we propose a multi-level machine learning pipeline in which the initial ranking of HIP candidates is performed using Ridge regression, then refined by local weighted XGBoost (Extreme Gradient Boosting) models, and finally integrated into an anchor-calibrated global ranking model. This strategy enables the use of known experimentally characterized HIPs, including both immunogenic and non-immunogenic variants, and allows the most probable signals to be progressively strengthened during candidate selection. The aim of this study was to develop a multi-level ranking model for HIP candidates based on known experimentally characterized HIPs. The resulting global model provides a ranked list of HIP candidates for subsequent experimental verification.

## Materials and methods

### Feature selection for the baseline regression model

This study used a set of 240 HIP candidates generated from a published peptide list and ELISPOT reactivity data for peptide pools containing 240 HIP candidates (14). For each peptide, a set of features was selected to capture physicochemical, structural, and other properties of HIPs. Features were selected based on experimental data, biological rationale, and published literature sources, including Calis et al. (20)Schaap-Johansen et al. (21)Farriol-Duran et al. (22), and Hohenstein et al. (14). Detailed feature definitions, calculation rules, and a worked example for an individual peptide are provided in Supplementary Methods S1. Feature selection was performed without the use of automated dimensionality reduction methods.

**Figure 1:**
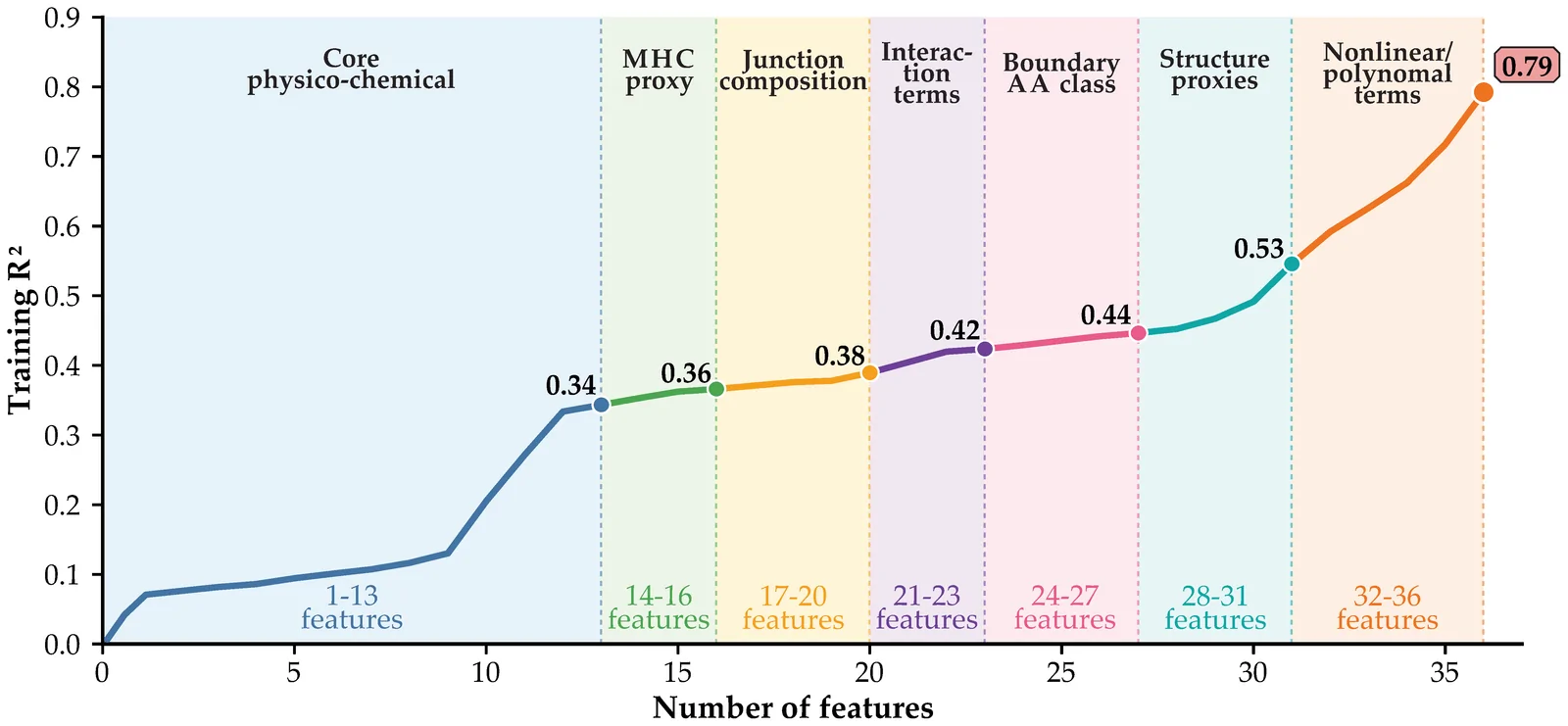
Exploratory training-fit analysis showing training R² during sequential addition of the seven feature groups. The X-axis shows the number of features included in the model. The Y-axis shows training R². Colors indicate the feature groups (n = 7) that were sequentially added to the model. These groups include basic physicochemical properties, MHC-proxy features, junction region features, interaction terms, the amino acid class at the junction boundary, structural proxy features, and nonlinear features. The baseline Ridge model uses 36 features and achieves training R² = 0.792.

In total, seven groups of features were generated. These groups were sequentially added to the baseline model, after which a Ridge regression model was trained for each cumulative feature set and its ability to predict the experimentally assigned signal for 240 HIP candidates was evaluated. Model performance was assessed using training R² and 5-fold out-of-fold (OOF) R². The cumulative contribution of the selected 36 features to the performance of the baseline model is shown in **Figure 1**.

Feature computation was performed using specialized Python scripts: feature_engineering.py, junction_features.py, mhc_features.py, structure_features.py, structure_proxy.py, aa_features.py, interactions.py, and nonlinear.py. The scikit-learn, pandas, and NumPy libraries were used for standardization and basic computational operations. All scripts used in this study are publicly available at https://github.com/1nsp1rer/HIP-. All features were standardized using Z-score transformation before model training. The complete list of selected features, their calculation methods, and an example calculation are presented in **Supplementary Table 1**.

### Construction of the baseline regression model

After selection, calculation, and standardization of the 36 features, a baseline regression model, Ridge regression, was built to test whether the selected features adequately described the experimental ELISPOT data obtained for the 240 HIPs reported by Hohenstein et al. (14). This model was used exclusively as the first step to assess the contribution of the features and to obtain the initial linear Ridge-score. The target variable (*y*) was defined as a conditional reactivity level, or group signal, assigned according to the membership of each HIP candidate in a peptide pool from the experimental data of 14 (14), based on the HIP junction region. In total, four groups were defined from the 24 pools: three positive groups, L26 (y = 1.00), A18 (y = 0.50), and G15 (y = 0.25), and one other group comprising all remaining ELISPOT-negative pools (y = 0.00).

The 240 × 36 feature matrix was subjected to Z-score standardization, after which Ridge regression was trained with a regularization parameter α = 0.1. The final model was fitted on the entire 240-HIP library, and its linear output was calculated as a weighted sum of the 36 standardized features. For convenience, the resulting Ridge-score was additionally normalized to the range from 0 to 1. The complete list of calculated features, Ridge-score values, and Ridge-ranks is presented in **Supplementary Table 2**.

The model was evaluated using 5-fold out-of-fold validation and leave-one-pool-out validation. Model performance was assessed using the coefficient of determination (R²) and the area under the receiver operating characteristic curve (AUC). All calculations were implemented in Python using the scikit-learn library.

### Generation of HIP candidates

FASTA sequences of pancreatic β-cell secretory granule proteins were used to generate HIP candidates: insulin/proinsulin, including insulin C-peptide (INSC), insulin A-chain (INSA), and insulin B-chain (INSB) (NP_000198.1); islet amyloid polypeptide/amylin (IAPP; NP_000406.1); chromogranin A (CHGA; NP_001266.1); neuropeptide Y (NPY; NP_000896.1); chromogranin B/secretogranin-1 (SCG1; NP_001810.2); and chromogranin C/secretogranin-2 (SCG2; NP_003460.2). Insulin-derived fragments from INSA, INSB, or INSC were placed on the left and combined in the native N-to-C orientation with 7–9-residue fragments from each predefined precursor protein; duplicate full-length sequences were removed. The resulting candidates derived from the eight source proteins were used to construct local XGBoost models.

### Construction of local XGBoost models

To refine the linear baseline Ridge ranking, local weighted XGBoost models were constructed for the eight HIP source proteins. Ridge-scores were calculated for all generated HIP candidates and then used to define pseudo-labels: the top 2% of candidates by Ridge-score were assigned as pseudo-positive, whereas the bottom 10% were assigned as pseudo-negative. In the local XGBoost models, these pseudo-labels corresponded to binary training labels, with pseudo-positive candidates assigned label = 1 and pseudo-negative candidates assigned label = 0. The sample weight of pseudo-positive candidates was set to 1.0, and the sample weight of pseudo-negative candidates was set to 1.0. Thus, the pseudo-label defined the target class, whereas the sample weight defined the relative contribution of each observation to model training. Ridge-score, Ridge-rank, and any derived score columns from the baseline model (**Supplementary Table 2**) were not used as XGBoost features, thereby preventing direct leakage of the Ridge signal into the XGBoost model. The complete dataset, including XGB-score values and the 36 calculated features for each of the eight local models, is presented in **Supplementary Table 3**.

Known HIPs from the literature (n = 16) were additionally included in each local model (**Supplementary Table 4**) as high-confidence positive observations and assigned label = 1 with a sample weight of 10,000 (4, 6, 18, 23–25). HIPs from the ELISPOT-positive pools L26, A18, and G15 were also assigned positive labels, with sample weights set to L26 = 8,000, A18 = 4,000, and G15 = 2,000. All remaining ELISPOT pools were treated as ELISPOT-derived weak-negative references and assigned label = 0 with a sample weight of 5,000. If a candidate overlapped with an experimentally derived HIP, the experimental label and its corresponding sample weight were used instead of the Ridge-derived pseudo-label. Local XGBoost models were trained using only the previously selected 36 features. The effect of XGBoost-based refinement was evaluated by rank changes, Ridge/XGBoost correlation, XGB boost, and exclusion of negative references from the top-k region; these comparisons characterize stage-wise candidate prioritization and are not interpreted as independent held-out validation beyond the Ridge baseline. As a result, the following parameters were empirically selected and used in subsequent analyses: n_estimators = 500, max_depth = 3, learning_rate = 0.03, subsample = 0.80, colsample_bytree = 0.80, min_child_weight = 5.0, reg_lambda = 10.0, reg_alpha = 0.1, and random_state = 42. All calculations were implemented in Python using pandas, NumPy, scikit-learn, and XGBoost.

### Construction of the global ranking model for HIP candidates

The final ranking was performed as anchor-calibrated reranking using the previously calculated local XGB-scores. After integration into the global model, the total dataset comprised 1,057,374 HIP candidates. Each candidate was then compared with two sets of anchors: 46 positive anchors and 210 negative anchors. Positive anchors included 16 known HIPs and 30 ELISPOT-derived positive HIPs; negative anchors corresponded to 210 ELISPOT-derived weak-negative HIPs from the original 240-HIP library reported by Hohenstein et al. (14). Similarity was calculated in the space of the 36 previously selected features using cosine similarity.

The global score for each HIP candidate was inherited from the local XGB-score. The following parameters were used: positive-similarity boost weight = 0.05, negative-similarity penalty weight = 0.25, and minimum margin between positive and negative similarity = 0.10. For the known negative HIPs, corresponding to 210 ELISPOT-derived weak-negative HIPs, an additional direct penalty of 5.0 was applied to exclude them from the upper experimental window. Known positive HIPs were used for top-1000 calibration: they were not all placed in the first positions but were instead distributed within the rank range of 50–1000. All 1,057,374 candidates were then reranked according to the global score. This model was considered not as an independent blind-validation predictor but as an anchor-calibrated prioritization score for candidate selection. The global model dataset, including the first 100,000 ranked candidates and Global XGB-score values, is presented in **Supplementary Table 5**.

## Results

### Multi-level ranking model for HIP candidates

**Figure 2:**
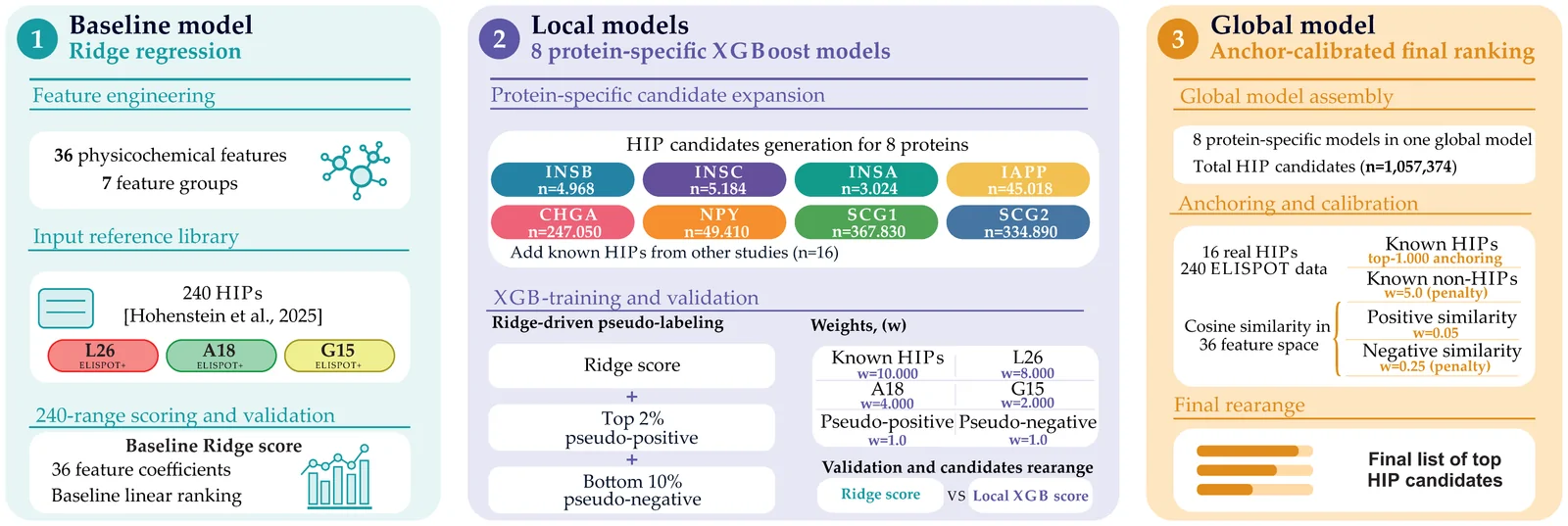
Multi-level approach for ranking HIP candidates. INS, insulin/proinsulin; INSC, insulin C-peptide; INSA, insulin A-chain; INSB, insulin B-chain; IAPP, islet amyloid polypeptide/amylin; CHGA, chromogranin A; NPY, neuropeptide Y; SCG1, secretogranin-1; SCG2, secretogranin-2. Pools L26, A18, and G15 are defined according to 14 (14).

Prediction of HIP candidates is a challenging task for machine learning. The space of possible sequences is vast, whereas the number of experimentally known HIPs is limited and is only partially represented by ELISPOT signals. Simple linear regression provides only an initial ranking, individual decision trees are unstable, and a complex model without additional constraints can easily overfit to the small number of real experimental signals. To address these challenges, this study developed a multi-level ranking approach for HIP candidates using multiple models. The concept of the proposed strategy is shown in **Figure 2**.

At the first stage, 36 features were calculated for the 240 HIPs reported by 14 (14), after which Ridge regression was used as the baseline linear model to test whether the selected features described the experimental ELISPOT data. This stage was not considered the final ranking step but served as the starting point for subsequent training. Thus, the Ridge model provided the initial ranking structure, whereas further improvement required nonlinear XGBoost-based refinement and the addition of known HIPs.

At the second stage, HIP candidates were generated for eight source proteins: insulin B-chain (INSB), insulin C-peptide (INSC), insulin A-chain (INSA), islet amyloid polypeptide (IAPP), chromogranin A (CHGA), neuropeptide Y (NPY), secretogranin-1 (SCG1), and secretogranin-2 (SCG2). Sixteen known HIPs from published studies were added to the training set (**Supplementary Table 4**). Known HIPs, as well as ELISPOT-derived positive and negative HIPs, were incorporated into training through a weighting scheme. For each source protein, a separate weighted XGBoost model was constructed and compared with the Ridge model by changes in rank, the magnitude of XGB boost, Pearson correlation, and the ability to move positive HIPs upward while excluding negative HIPs from the upper ranking regions. As a result, each of the eight local models learned features of HIP candidates specific to its corresponding source protein.

At the final stage, the eight local XGBoost models were integrated into a single global model. For this purpose, anchor calibration was performed using known HIPs and cosine similarity calculated across the 36 baseline features. An anchor-based system was also applied to increase the priority of candidates similar to confirmed positive HIPs and to further lower the priority of candidates similar to negative examples. The main result of this work is a prioritized set of HIP candidates generated by the multi-level ranking pipeline and intended for subsequent experimental validation. The global model was used as an anchor-calibrated candidate-prioritization framework that transfers information from experimentally supported positive and negative references into the final ranking and was not interpreted as an independent blind-validation predictor.

### Baseline ridge model

**Figure 3:**
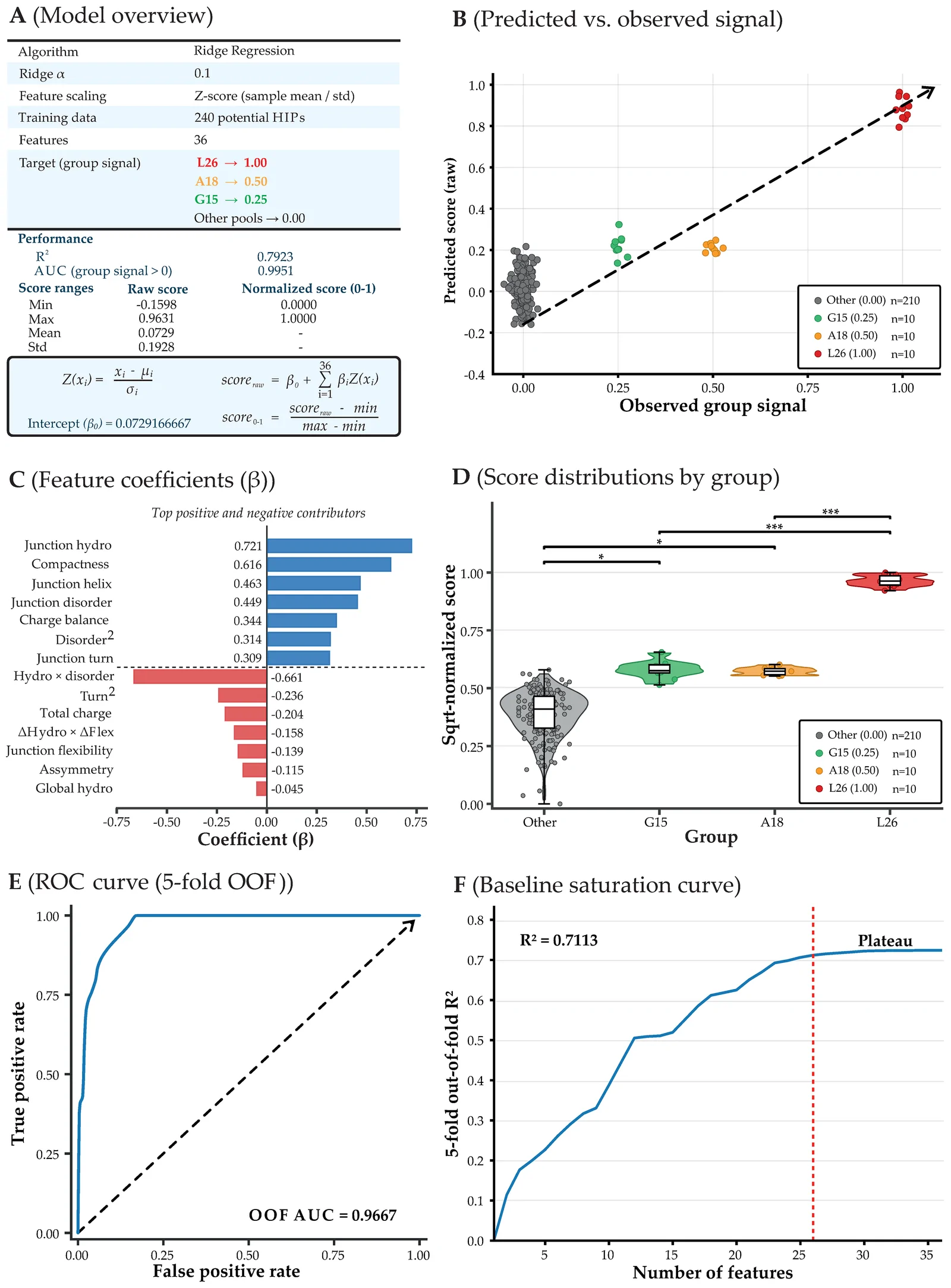
Characterization of the Ridge model for evaluating HIP candidates. **(A)** Model parameters, score calculation scheme, and main performance metrics; **(B)** relationship between the observed group signal on the X-axis and the predicted raw score on the Y-axis; the dashed line indicates theoretical ideal agreement; **(C)** feature coefficients β, with positive coefficients shown in blue and negative coefficients shown in red; **(D)** violin plot showing the distribution of the square-root-normalized score across groups; asterisks(*) indicate statistical significance between groups; **(E)** receiver operating characteristic (ROC) curve for 5-fold OOF validation, with the dashed line corresponding to random classification; **(F)** model saturation curve, with the number of features shown on the X-axis and 5-fold OOF R² on the Y-axis; the red dashed line marks the beginning of the plateau. **Figure 3D** shows the descriptive distribution of scores across the pool-derived surrogate groups. **Figure 3F** shows 5-fold out-of-fold validation performance.

At the first stage, we generated seven functional feature groups describing the main physicochemical properties of HIP candidates. The final model included 36 features. These features captured the hydrophobicity and flexibility of the junction region, total peptide charge and charge balance, fragment length and asymmetry, MHC-proxy characteristics of the central 9-mer region, the amino acid composition of the junction region, the amino acid class at the junction boundary, structural proxy features, interaction terms, and nonlinear combinations of the original parameters. This feature set made it possible to describe HIPs not only by their overall amino acid sequence but also by the properties of the junction region formed during transpeptidation, which appears to be critical for HIP formation, stability, and the development of an autoimmune response against them. The final characteristics of the resulting Ridge model are shown in **Figure 3A**.

As shown in **Figure 3B**, the predicted Ridge-score increased with the observed signal within each ELISPOT pool. Peptides that did not show a T-cell response, i.e., those without an ELISPOT signal, clustered predominantly at low score values, whereas peptides from the L26 pool formed a separate cluster with the highest predicted values. The A18 and G15 groups occupied intermediate positions, consistent with the predefined gradation of the target signal. This indicates that the model not only separated positive peptides from background peptides but also preserved the ordering of the experimental pools according to the conditional level of reactivity.

Analysis of the coefficients of the 36 model features (**Figure 3C**) showed that the largest positive contributions to the final Ridge-score were made by junction hydrophobicity (β = 0.721), local compactness (compactness proxy, β = 0.616), α-helical propensity of the junction region (junction helix, β = 0.463), local disorder (junction disorder, β = 0.449), charge balance (β = 0.344), squared disorder (disorder², β = 0.314), and turn propensity of the junction region (junction turn, β = 0.309). The largest negative contributions were associated with the interaction between hydrophobicity and disorder (hydro × disorder, β = −0.661), squared turn propensity (turn², β = −0.236), total peptide charge (total charge, β = −0.204), the interaction between hydrophobicity shift and flexibility shift (Δhydro × Δflex, β = −0.158), junction flexibility (β = −0.139), fragment asymmetry (asymmetry, β = −0.115), and global hydrophobicity (β = −0.045). Thus, the final Ridge-score of the baseline model was determined by a combination of local hydrophobicity, charge, structural proxy features, nonlinear interactions, and other feature groups.

The distribution of the square-root-normalized Ridge-score across groups (**Figure 3D**) confirmed peptide separation according to the group signal. The other group showed the widest and lowest range of values, whereas the L26 group demonstrated a compact distribution in the high-score region. The A18 and G15 groups were located between them and differed from the other group. The receiver operating characteristic curve for 5-fold OOF validation (**Figure 3E**) also showed a high ability of the model to distinguish peptides with a positive ELISPOT signal (L26, A18, and G15 groups) from negative peptides (other group), reaching an OOF AUC of 0.9667.

The 36 features were also ranked according to the absolute magnitude of their Ridge model coefficients. **Figure 3F** shows model saturation based on 5-fold OOF R² = 0.7113 during sequential addition of individual features in order of their contribution. R² increased rapidly when the most informative features were added and reached a plateau after approximately 26 features. This indicates that the main predictive capacity of the model is concentrated in a limited set of key physicochemical characteristics of HIPs, although this does not preclude the use of all 36 features.

Despite the high performance of the Ridge model on the training set and in 5-fold OOF validation, its linear structure limited its ability to capture more complex relationships between features. The target variable represented a pool-derived surrogate ELISPOT signal rather than peptide-level immunogenicity, and leave-one-pool-out validation showed limited transferability to new groups. Therefore, the next step was XGBoost-based refinement using eight local models, which preserved the physicochemical description of HIPs while capturing nonlinear relationships among the 36 features.

### XGBoost-based refinement of the baseline model and construction of local models

HIP candidates with left and right fragments of 7–9 amino acid residues were generated. For each combination of C-peptide with a fragment from a right source protein, a separate local XGBoost model was constructed. As a result, eight local models and eight independent ranked lists of HIP candidates were obtained (**Supplementary Table 3**). Each model captured the features specific to its corresponding source protein and re-ranked candidates based on the 36 physicochemical features. Ridge-score, Ridge-rank, and derived score features from the baseline model were not used as XGBoost input features in order to prevent leakage and model overfitting.

Sixteen known HIPs from experimental studies were added to each local XGBoost model (**Supplementary Table 4**). Previously reported ELISPOT data from Hohenstein et al. (14) for the L26, A18, and G15 pools were used as positive references, whereas the remaining pools with negative ELISPOT data were used as negative examples. Performance was evaluated by the ability of the model to move known HIPs and ELISPOT-derived positive HIPs into the top 5% of the ranked candidate list. The final peptide ranking for each local model and comparison with the baseline Ridge model are presented in **Table 1**.

**Table 1:** Comparison of known HIPs positions before and after XGBoost-based refinement.

№	Local model	Number of HIP candidates	Ridge rank			XGB rank				Top-k validation		Pearson (r)
			L26	A18	G15	L26	A18	G15	Known HIPs	Positives in XGB top 5%	Negatives in XGB top 10%	
1	INS_CHGA	247,050	13,798	54,937	52,124	2,300	14,617	54,992	27,139	Yes	No	0.635
2	INS_IAPP	45,018	2,189	6,836	6,580	393	1,159	18	3	Yes	No	0.242
3	INS_NPY	49,410	1,901	7,682	7,633	28	469	125	75	Yes	No	0.676
4	INS_SCG1	367,830	21,561	73,124	72,521	2,600	14,333	21,026	4,096	Yes	No	0.930
5	INS_SCG2	334,890	16,731	57,823	63,229	8,385	9,301	12,452	—	Yes	No	0.749
6	INSC_INSA	3,024	11	260	201	6	10	3	4	Yes	No	0.306
7	INSC_INSB	4,968	161	643	652	23	11	44	888	Yes	No	0.171
8	INSC_INSC	5,184	143	882	1,002	131	415	801	8	Yes	No	0.886

The table shows the number of candidates for each local model, the position of the best ELISPOT-derived positive HIPs (L26, A18, and G15) and known HIPs, the Pearson correlation coefficient (r) between Ridge- and XGBoost-based rankings, and the results of top-k validation. Extended data are presented in **Supplementary Table 3**.

As shown in **Table 1**, XGBoost-based refinement improved the ranking of ELISPOT-derived positive HIPs relative to the baseline Ridge model. The strongest effect was observed for HIPs formed from insulin and neuropeptide Y (INS_NPY), where L26 moved from rank 1,901 to rank 28, A18 from rank 7,682 to rank 469, and G15 from rank 7,633 to rank 125. For HIPs formed from insulin and islet amyloid polypeptide (INS_IAPP), G15 moved from rank 6,580 to rank 18. For HIPs formed from insulin C-peptide and insulin A-chain (INSC_INSA), all three positive HIPs entered the upper part of the list: L26 ranked 6th, A18 ranked 10th, and G15 ranked 3rd. The Pearson correlation between Ridge and XGBoost rankings ranged from 0.171 to 0.930, indicating that in some source proteins XGBoost largely preserved the overall Ridge-ranking structure, whereas in others it substantially reordered the candidates.

**Figure 4:**
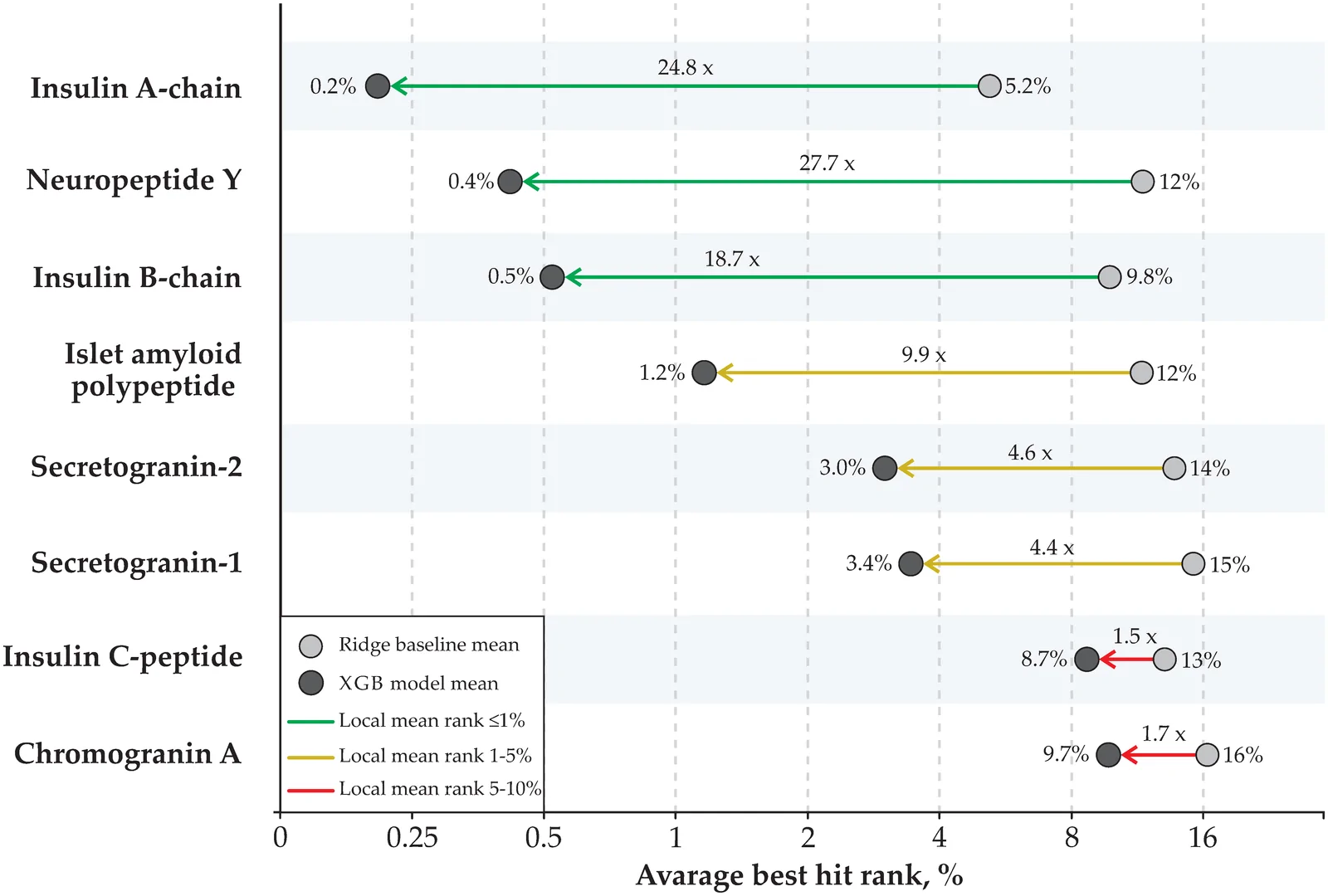
Comparison of the baseline Ridge model and the eight local XGBoost models by the average rank of ELISPOT-derived positive HIPs in the ranked candidate list. A lower average best-hit rank indicates that the model places positive HIPs higher in the ranked list. The light-gray point shows the average HIP position in the Ridge model, whereas the dark-gray point shows the average HIP position in the local XGBoost model. The number above each arrow indicates the fold decrease in the average rank of ELISPOT-derived positive HIPs relative to the Ridge model. The arrow color corresponds to the average HIP position in the XGBoost model: green indicates candidates ranked in the top 1%, yellow indicates candidates ranked in the top 1–5%, and red indicates candidates ranked in the top 5–10%.

Known HIPs also occupied high positions after XGBoost-based refinement: rank 3 for INS_IAPP, rank 75 for INS_NPY, rank 4 for INSC_INSA, and rank 8 for INSC_INSC. Top-k validation showed that, in all eight models, positive HIPs were ranked within the top 5% of candidates, whereas negative HIPs did not enter the top 10%. A more detailed comparison between the baseline Ridge model and the local XGBoost models is shown in **Figure 4**.

As shown in **Figure 4**, all eight local XGBoost models ranked ELISPOT-derived positive HIPs higher than the baseline Ridge model. The strongest improvement was observed for insulin A-chain (INSA), neuropeptide Y (NPY), and insulin B-chain (INSB). For insulin C-peptide (INSC) and chromogranin A (CHGA), the effect was weaker, but the direction remained the same. These results show that the local models more effectively identified experimentally supported HIPs among the large set of remaining candidates. Thus, the eight local XGBoost models refined the baseline Ridge model and improved the ranking positions of experimentally supported HIPs.

However, independent XGBoost-based refinement and the eight separate local models were not sufficient to generate a single ranked list. The total number of possible candidates was too large, and each model accounted only for its own source protein. This required the next stage of integrated candidate selection and the development of a global model.

### Global ranking of HIP candidates

We performed global ranking, which integrated the results of the eight local models into a single ranked list of HIP candidates. From the complete set of 1,057,374 candidates, five top-priority potential HIPs were selected for each source protein, yielding 40 peptides in total (**Table 2**). These candidates were highly ranked by the local XGBoost models, within the top 5%, and were similar to experimentally positive HIPs across the 36-feature space.

**Table 2:** Highest-priority HIP candidates identified by global ranking.

№	HIP sequence	Global model position from 1,057,374	Local XGB model (position/candidates)	Final score	Length	Left source	Right source
1	SHLVEALYLFRARAYGFR	36	1,828/247,050	0.894	18	INSB	CHGA
2	SHLVEALYLERAHQQKKH	54	4,543/247,050	0.893	18	INSB	
3	VCGERGFFYERAHQQKKH	61	2,368/247,050	0.892	18	INSB	
4	AGSLQPLALRAYGFRGPG	70	4,462/247,050	0.891	18	INSC	
5	SHLVEALYLARAYGFRGP	80	1,793/247,050	0.891	18	INSB	
6	SHLVEALYLHQVEKRKCN	16	467/45,018	0.898	18	INSB	IAPP
7	VCGERGFFYHQVEKRKCN	22	77/45,018	0.898	18	INSB	
8	AGSLQPLALHQVEKRKCN	32	381/45,018	0.895	18	INSC	
9	CGERGFFYTQVEKRKCNT	120	594/45,018	0.889	18	INSB	
10	AGSLQPLALNTYGKRNAV	168	84/45,018	0.887	18	INSC	
11	AGSLQPLALQRYGKRSSP	1	395/49,410	0.905	18	INSC	NPY
12	SHLVEALYLQRYGKRSSP	2	230/49,410	0.903	18	INSC	
13	QCCTSICSLQRYGKRSSP	4	1,191/49,410	0.902	18	INSA	
14	AGSLQPLALRYYSALRHY	5	267/49,410	0.902	18	INSC	
15	TSICSLYQLITRQRYGKR	6	576/49,410	0.901	18	INSA	
16	CGERGFFYTRQVLKTSRK	8	4,898/367,830	0.901	18	INSB	SCG1
17	CTSICSLYQWKSSHFERR	11	3,596/367,830	0.900	18	INSA	
18	AGSLQPLALVDKRRTRPR	18	2,165/367,830	0.898	18	INSC	
19	VCGERGFFYVDKRRTRPR	23	1,815/367,830	0.897	18	INSB	
20	CGERGFFYTVDKRRTRPR	24	7,457/367,830	0.897	18	INSB	
21	TSICSLYQLQQWPERKLK	3	812/334,890	0.903	18	INSA	SCG2
22	LCGSHLVEAQQWPERKLK	14	6,574/334,890	0.900	18	INSB	
23	CTSICSLYQQQWPERKLK	25	1,259/334,890	0.897	18	INSA	
24	ERGFFYTPKPERKLKHMQ	26	3,348/334,890	0.897	18	INSB	
25	RGFFYTPKTTQQWPERKL	37	2,475/334,890	0.894	18	INSB	
26	AGSLQPLALYQLENYCN	2,179	15/3,024	0.869	17	INSC	INSA
27	GSLQPLALLYQLENYCN	3,561	18/3,024	0.864	17	INSC	
28	SLQPLALYQLENYC	4,779	31/3,024	0.860	14	INSC	
29	GSLQPLALYQLENYC	4,879	40/3,024	0.860	15	INSC	
30	GSLQPLALYQLENYCN	4,979	36/3,024	0.860	16	INSC	
31	AGSLQPLALQHLCGSHL	1,611	64/4,968	0.872	17	INSC	INSB
32	GSLQPLALQHLCGSHLV	1,627	67/4,968	0.872	17	INSC	
33	GPGAGSLQERGFFYTPK	1,718	82/4,968	0.871	17	INSC	
34	GSLQPLALRGFFYTPKT	2,178	50/4,968	0.869	17	INSC	
35	AGSLQPLALQHLCGSHLV	2,183	61/4,968	0.869	18	INSC	
36	AGSLQPLALEDLQVGQV	15,975	52/5,184	0.842	17	INSC	INSC
37	AGSLQPLALEAEDLQVG	17,728	54/5,184	0.839	17	INSC	
38	AGSLQPLALEAEDLQVGQ	19,772	63/5,184	0.837	18	INSC	
39	GSLQPLALEDLQVGQV	21,493	78/5,184	0.835	16	INSC	
40	AGSLQPLALEDLQVGQVE	21,837	57/5,184	0.834	18	INSC	

For each candidate, the table shows its position in the global model and its position in the corresponding local XGBoost model.

As shown in **Table 2**, global ranking integrated the results of the eight local weighted XGBoost models into a single list of HIP candidates. The selected sequences occupied high positions both in the global ranking among 1,057,374 candidates and within their corresponding local models. This indicates that the final list was formed not only by global calibration but also by the consistently high positions of the candidates in the local models.

Potentially novel HIP candidates underwent sequential multi-level selection that included initial Ridge ranking, local nonlinear XGBoost-based refinement, and global similarity-based anchor calibration across the 36 features. This approach made it possible to identify candidates similar to positive anchors while simultaneously reducing the priority of sequences similar to negative examples. Thus, **Table 2** represents the main result of the multi-level computational selection and provides a list of HIP candidates for subsequent experimental validation.

## Discussion

One of the important unresolved challenges in type 1 diabetes research is the identification of new pancreatic β-cell autoantigens, among which HIPs occupy a special place; however, their systematic discovery remains difficult. To date, only a limited number of HIPs have been experimentally described, whereas the number of possible combinations of peptide fragments is substantially larger. Thus, there is a considerable gap between the theoretically possible diversity of HIPs and the experimentally feasible scale of their validation.

Machine learning offers a fundamentally different way to approach the ranking and discovery of new HIP candidates, as it can identify hidden patterns in settings where direct experimental testing of all variants is not feasible. However, the use of machine learning for HIPs discovery also presents several challenges. Simple Ridge regression provides an initial linear assessment of candidates but does not capture all possible nonlinear combinations of features. At the same time, applying a single complex model to a small number of experimentally characterized HIPs may lead to overfitting. Therefore, this study used a multi-level approach that included a baseline Ridge model, eight local weighted XGBoost models, and one anchor-calibrated global ranking model. This design enabled stepwise candidate ranking, reduced the contribution of less promising sequences, and strengthened real experimental signals.

A key element underlying the predictive performance of the model was the deliberate selection of 36 features without automated dimensionality reduction. These features were grouped into seven functional categories reflecting physicochemical properties, including hydrophobicity, charge, and flexibility; structural proxy features, including α-helix propensity, β-sheet propensity, and disorder; junction region features, including hydrophobicity and protease accessibility; MHC-proxy features, approximating HLA binding; and nonlinear interactions, such as hydrophobicity × disorder. Analysis of the Ridge model coefficients showed that junction hydrophobicity (β = 0.721) and local compactness (β = 0.616) made the largest positive contributions, which may influence the stability of HIPs in the acidic environment of secretory granules. The negative contribution of total peptide charge (β = −0.204) and fragment asymmetry (β = −0.115) supports the idea that excessive polarity or a strong imbalance in the lengths of the left and right fragments may hinder the formation of stable HIPs. Thus, this study proposes a computationally implementable set of predictors covering the presumed mechanisms of HIP formation.

For the first time, the total set of potential HIP candidates was generated and calculated for eight pancreatic β-cell secretory granule proteins. Candidate generation used a sliding window of 7–9 amino acid residues applied to eight source proteins: INSB, INSC, INSA, IAPP, CHGA, NPY, SCG1, and SCG2. For each of the 1,057,374 unique candidates, all 36 physicochemical features were calculated, followed by ranking first in eight local XGBoost models and then in a single global model.

Comparable large-scale systematic assessments of HIP repertoires have not been performed previously. Existing studies have either been limited to several dozen preselected hybrid peptides or have used random combinatorial libraries without accounting for biological plausibility. In contrast, our approach combines systematic enumeration of theoretically possible combinations, under defined constraints on fragment length and source proteins, with subsequent ranking based on selected features.

The practical result of this work was a list of 40 highest-priority HIP candidates. These candidates simultaneously satisfied three criteria: a high Ridge-score, representing the baseline linear assessment; a high position in the local XGBoost model, in some cases within the top 1% for the corresponding source protein; and positive anchor calibration, reflecting similarity to positive anchors and separation from negative examples. Importantly, the final list is not simply the top candidates according to a single score; it was generated through competition among different source proteins within the global model. Thus, the proposed multi-level approach identified the most plausible candidates in terms of structural stability and biological plausibility.

Analysis of global ranking showed that the highest positions, ranks 1–168, were occupied by candidates with right fragments derived from CHGA, IAPP, NPY, SCG1, and SCG2. Within these groups, the model identified several stable patterns rather than amplifying a single preferred sequence type. This may indicate that HIPs immunogenicity can be determined by different structural motifs depending on the right source protein.

A particularly interesting result was observed for candidates in which the insulin fragment was located on the right side of the sequence. Despite their high positions in local XGBoost models, these candidates shifted downward after global ranking with anchor calibration based on known HIPs, occupying ranks 2,179–21,837. Known HIPs are known to more often contain insulin, or an insulin fragment, on the left, N-terminal side, which may be related to the directionality of cathepsin-catalyzed transpeptidation (8). Thus, without being explicitly instructed to do so, the model independently captured this indirect feature, and HIP candidates with insulin on the right systematically received lower final scores. This supports the biological rationale of the selected weights and anchor calibration, although further studies and experimental confirmation are required.

**Figure 5:**
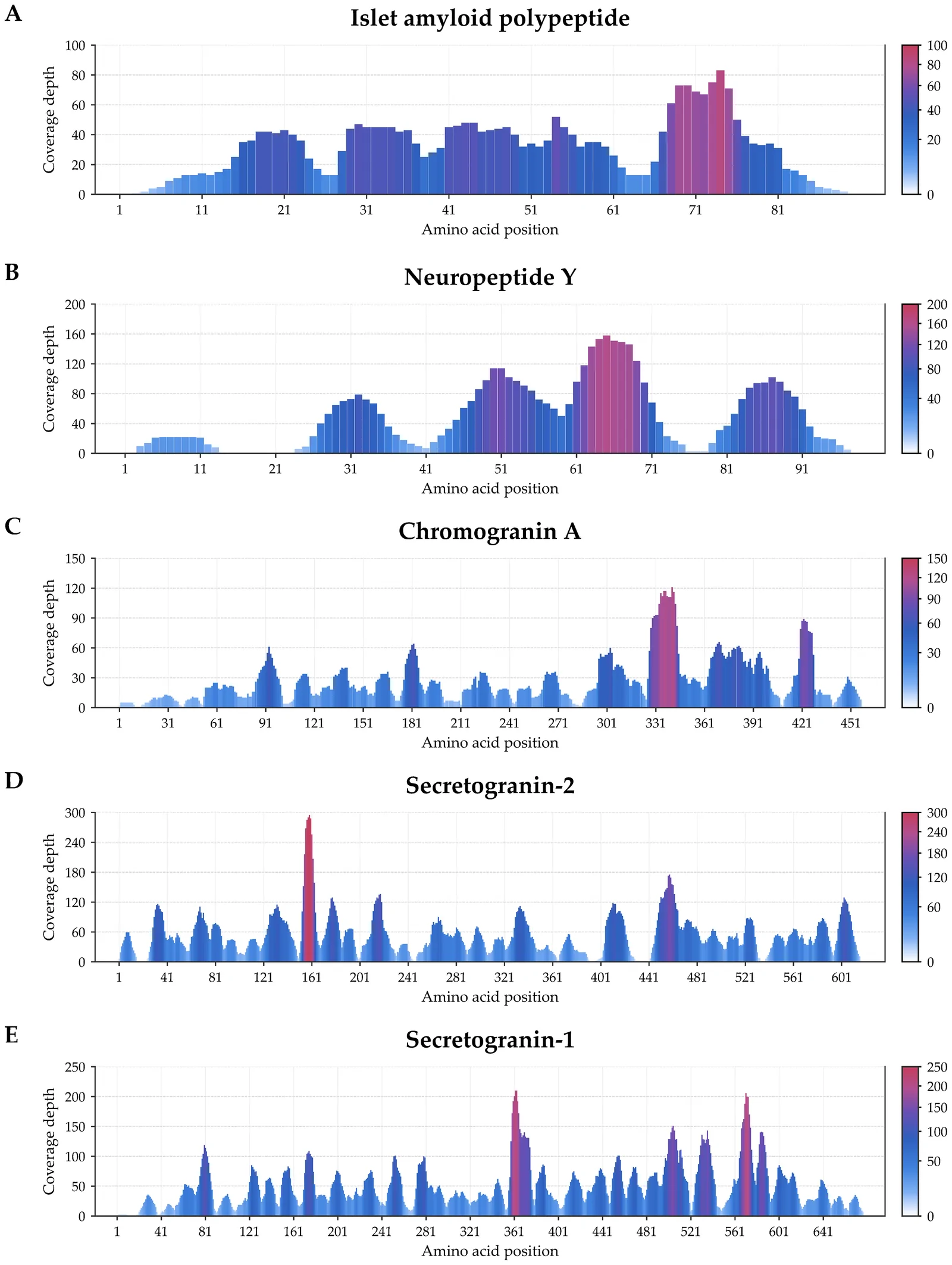
Peptide coverage landscape for five source proteins. Plots were generated only for peptides with a final score ≥ 0.85: **(A)** islet amyloid polypeptide; **(B)** neuropeptide Y; **(C)** chromogranin A; **(D)** secretogranin-2; and **(E)** secretogranin-1. The X-axis shows the amino acid residue position in the protein sequence, and the Y-axis shows coverage depth. Coverage depth was defined as the number of prioritized peptides overlapping each amino acid position. No denominator or normalization was applied, and protein-specific y-axis scales were used; therefore, coverage values should not be compared directly across proteins.

To determine which regions of the source proteins were most frequently selected by the model as potential HIP-forming regions, we additionally analyzed the peptide coverage landscape. To visualize this landscape and assess its relationship with the domain architecture of the proteins, coverage distribution plots were generated for five source proteins: CHGA, IAPP, NPY, SCG1, and SCG2 (**Figure 5**).

For IAPP, the highest coverage was observed at amino acid residues 68–76 (NTYGKRNAV) (**Figure 5A**). This region overlaps the C-terminal part of mature IAPP and the adjacent C-terminal processing region of IAPP. It is also relevant to IAPP/CGRP receptor binding and peptide function in glucose metabolism (26, 27). This region includes two of the three currently known HIPs with IAPP, namely GQVELGGGNAVEVLK (right IAPP_74–80) and SLQPLALNAVEVLK (right IAPP_74–80). Among the HIP candidates, only one top-priority candidate fell within this region: AGSLQPLALNTYGKRNAV (right IAPP_68–76). The remaining four HIP candidates fell within a lower peak around amino acid residues 28–36, corresponding to the HQVEKRKCN sequence: SHLVEALYLHQVEKRKCN (right IAPP_28–36), VCGERGFFYHQVEKRKCN (right IAPP_28–36), AGSLQPLALHQVEKRKCN (right IAPP_28–36), and CGERGFFYTQVEKRKCNT (right IAPP_29–37). Region 28–36 corresponds to the N-terminal processing region of IAPP and part of the junction between the propeptide and the mature hormone (26). Notably, the third of the three currently known HIPs with IAPP, GQVELGGGTPIESHQ (right IAPP_23–29), was located between the two peaks.

For NPY, the most pronounced peak with the maximum coverage was observed at amino acid residues 61–69 (RQRYGKRSS) (**Figure 5B**). This region includes two of the two currently known HIPs with NPY, namely GQVELGGGSSPETLI (right NPY_68–74) and SLQPLALSSPETLI (right NPY_68–74). Among the HIP candidates, four top-priority candidates fell within this region: TSICSLYQLITRQRYGKR (right NPY_58–67), AGSLQPLALQRYGKRSSP (right NPY_62–70), SHLVEALYLQRYGKRSSP (right NPY_62–70), and QCCTSICSLQRYGKRSSP (right NPY_62–70). The fifth HIPs candidate, AGSLQPLALRYYSALRHY (right NPY_47–55), fell within the neighboring peak at amino acid residues 47–55 (RYYSALRHY). Notably, both regions are located within the critically important functional mature region of neuropeptide Y (28).

For chromogranin A (CHGA), the highest coverage was observed for a peak at amino acid residues 337–345 (EDSKRWSKM) (**Figure 5C**), with a lower-intensity peak at amino acid residues 418–426 (KKEEEGSAN). The only currently known HIP with CHGA, SLQPLALWSKMDQL (right CHGA_342–348), lies within the highest-coverage peak (6). Three of the five identified top-priority candidates were located in another lower-coverage peak: SHLVEALYLFRARAYGFR (right CHGA_376–384), SHLVEALYLARAYGFRGP (right CHGA_378–386), and AGSLQPLALRAYGFRGPG (right CHGA_379–387). The remaining two candidates were located in another lower-coverage peak: SHLVEALYLERAHQQKKH (right CHGA_89–97) and VCGERGFFYERAHQQKKH (right CHGA_89–97).

For SCG2, the highest-coverage peak was identified at amino acid residues 155–163 (QWPERKLKH) (**Figure 5D**). Currently, no known HIPs with SCG2 have been reported in the literature, and all five identified top-priority candidates fell within the highest-coverage region at amino acid residues 155–163 (QWPERKLKH): TSICSLYQLQQWPERKLK (right SCG2_154–162), LCGSHLVEAQQWPERKLK (right SCG2_154–162), CTSICSLYQQQWPERKLK (right SCG2_154–162), RGFFYTPKTTQQWPERKL (right SCG2_153–161), and ERGFFYTPKPERKLKHMQ (right SCG2_157–165).

For SCG1, five highest-coverage peaks were identified at amino acid residues 358–366 (NENTKFEVR), 501–509 (FQDKQYSSH), 532–540 (KSSHFERRD), 567–575 (NYDWWEKKP), and 581–589 (NWGYEKRNL) (**Figure 5E**). Notably, two of the two currently known HIPs with SCG1 were located in a low-coverage region at amino acid residues 211–218, namely VCGERGFFEELVARSE (right SCG1_211–218) and HLVEALYLEELVARSE (right SCG1_211–218). Only one top-priority HIPs candidate fell within a high-coverage peak, CTSICSLYQWKSSHFERR (right SCG1_530–539), whereas the remaining candidates were located in lower-coverage regions: CGERGFFYTRQVLKTSRK (right SCG1_58–66), AGSLQPLALVDKRRTRPR (right SCG1_274–282), VCGERGFFYVDKRRTRPR (right SCG1_274–282), and CGERGFFYTVDKRRTRPR (right SCG1_274–282).

Thus, for the three major granin-family proteins, chromogranin A (CHGA), chromogranin B (SCG1), and chromogranin C (SCG2), the coverage peaks were located in different regions. This may correspond to functionally important domains and post-translational modification sites and may also reflect the high variability of the central regions of these proteins.

## Conclusions

The developed multi-level model enables *in silico* selection of the most likely HIP candidates for type 1 diabetes, reducing the search space from millions of possible sequences to several dozen prioritized candidates. The resulting list of 40 HIP candidates is intended for experimental validation using mass spectrometry and T-cell functional assays, including ELISPOT and HLA multimers. In a broader context, the proposed methodology, based on a linear baseline predictor, weighted XGBoost-based refinement, and anchor calibration, may be adapted to predict other classes of chimeric neoepitopes arising in autoimmune, cancer, and neurodegenerative diseases.

## Limitations

The multi-level approach depends on the quality of both the data and the features at each stage. Errors in the baseline Ridge model or insufficient feature representation may affect the local XGBoost models and subsequently the global ranking. Nevertheless, given the current shortage of experimental data, the construction of a single universal model is not yet feasible. Therefore, the proposed scheme is justified, although it is not the only possible approach.

As new experimental HIPs data become available, the model will require updating. The weights, as well as the pseudo-positive and pseudo-negative parameters in the local models, were selected empirically to achieve appropriate ranking of known HIPs and ELISPOT-derived HIPs. As the experimental dataset expands, these parameters should be reconsidered.

The selected HIPs generation window of 7–9 amino acid residues does not cover all possible variants of hybrid peptides. Although the precise length of immunogenic HIPs remains unknown and is likely dependent on the detection method employed, the immunologically relevant feature appears to be not the total number of amino acids, but rather the presence of the neoepitope at the splice junction. Both shorter and longer HIPs have been described in the literature; however, expanding the window would sharply increase the number of possible candidates to more than one billion, requiring a different computational strategy, additional filters, and a larger experimental dataset.

The global model relies on anchor calibration and cosine similarity across the 36-feature space to adjust the ranking of candidates relative to positive and negative reference sets. This step introduces an additional heuristic component into the pipeline and therefore does not represent an independent validation of candidate immunogenicity. However, under conditions of limited experimental data, such calibration is a necessary and justified strategy for transferring information from both ELISPOT-derived HIPs and known HIPs to a much larger candidate space.

## Data Availability

The original contributions presented in the study are included in the article/**Supplementary Material**. Further inquiries can be directed to the corresponding author.
