# Supplementary material for: A multi-level machine learning pipeline for prediction and prioritization of hybrid insulin peptides in type 1 diabetes
Source: Front Immunol. 2026 Aug 7;17:1917213. doi: 10.3389/fimmu.2026.1917213 (PMC13493391; doi:10.3389/fimmu.2026.1917213)
Supplement: Supplementary file 1 [file Table1.pdf]

Supplementary Table S1. Feature engineering strategy for 240-HIPs reference library

Feature	Feature explain	Python source block	Worked example on peptide GGPGAGSLFLGEGHH
junction_hydrophobicity	Mean hydrophobicity of the 6-residue junction window: last 3 residues of the left fragment plus first 3 residues of the right fragment.	feature_engineering.py / Kyte-Doolittle scale	left=GGPGAGSL, right=FLGEGHH. Junction window = GSL|FLG. Values: G=-0.4, S=-0.8, L=3.8, F=2.8, L=3.8, G=-0.4. Sum = 8.8. Mean = 8.8/6 = 1.466667. **Result in CSV: 1.466667**
junction_flexibility	Legacy local flexibility index for the junction. Here it is the fraction of flexible residues G/P/S in the 6-residue junction window.	feature_engineering.py	Junction = GSL|FLG. Flexible residue set = G, P, S. Present: G, S, G = 3 residues. Fraction = 3/6 = 0.500000. **Result in CSV: 0.5**
charge_balance	Simple balance between positively and negatively charged residues in the full peptide. Positive = K/R/H; negative = D/E.	feature_engineering.py	Sequence = GGPGAGSLFLGEGHH. Positive residues: H,H = 2. Negative residues: E = 1. Balance = 2 - 1 = 1. **Result in CSV: 1**
length	Number of amino acids in the full HIP sequence.	feature_engineering.py	Sequence = GGPGAGSLFLGEGHH. Count all letters: 15 residues. **Result in CSV: 15**
global_hydrophobicity	Mean Kyte-Doolittle hydrophobicity across the entire peptide, not just the junction.	feature_engineering.py / Kyte-Doolittle scale	Values for GGPGAGSLFLGEGHH: -0.4, -0.4, -1.6, -0.4, 1.8, -0.4, -0.8, 3.8, 2.8, 3.8, -0.4, -3.5, -0.4, -3.2, -3.2. Sum = -2.5. Mean = -2.5/15 = -0.166667. **Result in CSV: -0.166667**
total_charge	Approximate formal net charge at neutral pH. D/E = -1, K/R = +1, H treated as neutral in this feature.	feature_engineering.py	Sequence has E = 1, D = 0, K/R = 0. Histidines are treated as neutral here. Total charge = -1. **Result in CSV: -1**
anchor_residues	Count of strong aromatic/large anchor-like residues in the MHC core. In this legacy feature, F/W/Y are counted.	feature_engineering.py	MHC core = GAGSLFLGE. F/W/Y residues present: F = 1. Therefore anchor_residues = 1. **Result in CSV: 1**
asymmetry	Length asymmetry between left and right HIP fragments.	feature_engineering.py	left length = len(GGPGAGSL) = 8. right length = len(FLGEGHH) = 7. Asymmetry = |8 - 7| = 1. **Result in CSV: 1**
repeat_penalty	Count of overlapping 3-residue homopolymer windows. Each run of length n contributes (n−2).	feature_engineering.py	Sequence = GGPGAGSLFLGEGHH. No overlapping 3-residue homopolymer windows are present. Count = 0. **Result in CSV: 0**
junction_type_score	Categorical chemical score of the junction type. A hydrophobic junction transition receives score 1 in this dataset.	feature_engineering.py	Junction = GSL|FLG. Boundary residues include L|F, both hydrophobic. Junction type = hydrophobic-compatible. Score = 1. **Result in CSV: 1**
delta_hydro	Hydrophobicity jump across the junction: mean(right3) - mean(left3).	junction_features.py	left3=GSL: (-0.4 + -0.8 + 3.8)/3 = 0.866667. right3=FLG: (2.8 + 3.8 + -0.4)/3 = 2.066667. Delta = 2.066667 - 0.866667 = 1.200000. **Result in CSV: 1.2**
delta_charge	Charge jump across the junction: charge(right3) - charge(left3). D/E=-1, K/R=+1, H=0 here.	junction_features.py	left3=GSL: charge=0. right3=FLG: charge=0. Delta = 0 - 0 = 0. **Result in CSV: 0**
delta_flex	Flexibility jump across the junction: mean flexibility(right3) - mean flexibility(left3), using the local flexibility scale.	junction_features.py	left3=GSL: G=0.60, S=0.55, L=0.35 -> mean=0.500000. right3=FLG: F=0.35, L=0.35, G=0.60 -> mean=0.433333. Delta = 0.433333 - 0.500000 = -0.066667. **Result in CSV: -0.066667**
mhc_anchor_score	MHC-II proxy anchor score from a 9-mer core. Positions 1,4,6,9 are evaluated; only positive hydrophobicity contributes.	mhc_features.py	Core = GAGSLFLGE. Anchor positions 1,4,6,9 = ['G', 'S', 'F', 'E']. Hydrophobic contributions = [0, 0, 2.8, 0]. Score = sum/4 = 2.8/4 = 0.700000. **Result in CSV: 0.7**
mhc_charge_penalty	Penalty for charged residues in the MHC core: mean absolute charge across the 9-mer core.	mhc_features.py	Core = GAGSLFLGE. Only E contributes |charge|=1 here. Sum absolute charge = 1.0. Penalty = 1.0/9 = 0.111111. **Result in CSV: 0.111111**
mhc_binding_proxy	Simple MHC binding proxy: anchor score - charge penalty.	mhc_features.py	mhc_anchor_score = 0.700000. mhc_charge_penalty = 0.111111. Proxy = 0.700000 - 0.111111 = 0.588889. **Result in CSV: 0.588889**
proline_count_junction	Number of proline residues in the 6-residue junction window.	structure_features.py	Junction = GSL|FLG. P occurs 0 times. **Result in CSV: 0**
glycine_count_junction	Number of glycine residues in the 6-residue junction window.	structure_features.py	Junction = GSL|FLG. G occurs 2 times. **Result in CSV: 2**
has_proline_junction	Binary flag: 1 if the junction window contains at least one P, otherwise 0.	structure_features.py	Junction = GSL|FLG. P count = 0. Since count is 0, flag = 0. **Result in CSV: 0**
has_glycine_junction	Binary flag: 1 if the junction window contains at least one G, otherwise 0.	structure_features.py	Junction = GSL|FLG. G count = 2. Since count > 0, flag = 1. **Result in CSV: 1**
int_hydro_charge	Interaction term: delta_hydro multiplied by delta_charge.	interactions.py	delta_hydro = 1.200000. delta_charge = 0.000000. Product = 1.200000 * 0.000000 = 0.000000. **Result in CSV: 0**
int_hydro_mhc	Interaction term: junction_hydrophobicity multiplied by mhc_binding_proxy.	interactions.py	junction_hydrophobicity = 1.466667. mhc_binding_proxy = 0.588889. Product = 1.466667 * 0.588889 = 0.863704. **Result in CSV: 0.863704**
int_anchor_hydro	Interaction term: anchor_residues multiplied by delta_hydro.	interactions.py	anchor_residues = 1. delta_hydro = 1.200000. Product = 1 * 1.200000 = 1.200000. **Result in CSV: 1.2**
aa_0_class_hydrophobic	One-hot flag for the amino acid at the junction end of the left fragment. Hydrophobic class = A/I/L/M/F/W/V.	aa_features.py	aa_0 = last residue of left fragment GGPGAGSL = L. L is hydrophobic, so hydrophobic flag = 1. **Result in CSV: 1**
aa_0_class_polar	One-hot flag for polar aa_0. Polar class = S/T/N/Q/Y.	aa_features.py	aa_0 = L. L is not polar, so polar flag = 0. **Result in CSV: 0**
aa_0_class_charged	One-hot flag for charged aa_0. Charged class = D/E/K/R/H.	aa_features.py	aa_0 = L. L is not charged, so charged flag = 0. **Result in CSV: 0**
aa_0_class_special	One-hot flag for special aa_0. Special class = G/P.	aa_features.py	aa_0 = L. L is not G or P, so special flag = 0. **Result in CSV: 0**
junction_disorder	Average disorder propensity in the 6-residue junction window.	structure_proxy.py	Junction = GSL|FLG. Disorder values: G=0.7, S=0.6, L=0.2, F=0.2, L=0.2, G=0.7. Sum = 2.6. Mean = 2.6/6 = 0.433333. **Result in CSV: 0.433333**
junction_helix	Average alpha-helix propensity in the 6-residue junction window.	structure_proxy.py	Junction = GSL|FLG. Helix values: G=0.5, S=0.8, L=1.3, F=1.2, L=1.3, G=0.5. Sum = 5.6. Mean = 5.6/6 = 0.933333. **Result in CSV: 0.933333**
junction_turn	Average beta-turn / bend propensity in the 6-residue junction window.	structure_proxy.py	Junction = GSL|FLG. Turn values: G=1.5, S=1.2, L=0.5, F=0.6, L=0.5, G=1.5. Sum = 5.8. Mean = 5.8/6 = 0.966667. **Result in CSV: 0.966667**
compactness_proxy	Proxy for local compactness: hydrophobic fraction minus charged fraction in the 6-residue junction window.	structure_proxy.py	Junction = GSL|FLG. Hydrophobic residues = L,F,L = 3/6 = 0.5. Charged residues = none = 0/6 = 0.0. Compactness = 0.5 - 0.0 = 0.5. **Result in CSV: 0.5**
disorder_sq	Nonlinear term: square of junction_disorder.	nonlinear.py	junction_disorder = 0.433333. Square = 0.433333^2 = 0.187778. **Result in CSV: 0.187778**
turn_sq	Nonlinear term: square of junction_turn.	nonlinear.py	junction_turn = 0.966667. Square = 0.966667^2 = 0.934444. **Result in CSV: 0.934444**
hydro_disorder	Nonlinear interaction: junction_hydrophobicity multiplied by junction_disorder.	nonlinear.py	junction_hydrophobicity = 1.466667. junction_disorder = 0.433333. Product = 1.466667 * 0.433333 = 0.635556. **Result in CSV: 0.635556**
delta_flex_sq	Nonlinear flexibility-jump term	nonlinear.py	delta_flex_sq = -0.066667^2. Product = 0.004444 **Result in CSV: 0.004444**
delta_hydro_flex	Hydrophobicity jump x flexibility jump interaction.	nonlinear.py	delta_hydro = 1.200000. delta_flex = -0.066667. Product = 1.200000 * -0.066667 = -0.080000. **Result in CSV: -0.080000**
