# Supplementary material for: A multi-level machine learning pipeline for prediction and prioritization of hybrid insulin peptides in type 1 diabetes
Source: Front Immunol. 2026 Aug 7;17:1917213. doi: 10.3389/fimmu.2026.1917213 (PMC13493391; doi:10.3389/fimmu.2026.1917213)
Supplement: Supplementary file 2 [file Table5.pdf]

Supplementary Table S5. Anchor-Calibrated Global Ranking of HIPs Candidates

HIP ID	Pool according to Hohenstein et al.	Peptide information	Sequence	Left part	Right part	Right source protein	Local XGB position	Global XGB position from 1.057.374
HS_005	-	Real HIP	GQVELGGGTPIESHQ	GQVELGGG	TPIESHQ	IAPP	3	50
HS_006		Real HIP	GQVELGGGNAVEVLK	GQVELGGG	NAVEVLK	IAPP	31	134
HS_012		Real HIP	SLQPLALGIVEQCC	SLQPLAL	GIVEQCC	INSA	4	156
HS_014		Real HIP	SLQPLALNAVEVLK	SLQPLAL	NAVEVLK	IAPP	57	177
PNX24_L		Real HIP	EAEDLQVGLALEGSLQ	EAEDLQVG	LALEGSLQ	INSC	8	198
HS_016		Real HIP	SLQPLALSSPETLI	SLQPLAL	SSPETLI	NPY	75	219
HS_004		Real HIP	GQVELGGGGIVEQCC	GQVELGGG	GIVEQCC	INSA	7	261
HS_008		Real HIP	GQVELGGGSSPETLI	GQVELGGG	SSPETLI	NPY	184	346
DB190620_001		Real HIP	VCGERGFFEELVARSE	VCGERGFF	EELVARSE	SCG1	4096	493
DB190620_002		Real HIP	HLVEALYLEELVARSE	HLVEALYL	EELVARSE	SCG1	4646	536
HS_011		Real HIP	SLQPLALEAEDLQV	SLQPLAL	EAEDLQV	INSC	100	578
PNX24_A		Real HIP	VGQVELGGGLALEGSL	VGQVELGGG	LALEGSL	INSC	109	599
PNX24_F		Real HIP	VELGGGPGVELGGGP	VELGGGPG	VELGGGP	INSC	407	831
HS_009		Real HIP	SLQPLALWSKMDQL	SLQPLAL	WSKMDQL	CMGA	27139	916
PNX24_K		Real HIP	EAEDLQVGVCGERGF	EAEDLQVG	VCGERGF	INSB	888	958
PNX24_J		Real HIP	QPLALEGLQVGQVELG	QPLALEG	LQVGQVELG	INSC	3229	1000
HIP_1	G8	ELISPOT negative	EAEDLQVGFVNQHLCG	EAEDLQVG	FVNQHLCG	INSB	4774	1057357
HIP_2		ELISPOT negative	EAEDLQVGEAEDLQVG	EAEDLQVG	EAEDLQVG	INSC	5110	1057343
HIP_3		ELISPOT negative	EAEDLQVGGIVEQCCT	EAEDLQVG	GIVEQCCT	INSA	2602	1057263
HIP_4		ELISPOT negative	EAEDLQVGTPIESHQV	EAEDLQVG	TPIESHQV	IAPP	43397	1057363
HIP_5		ELISPOT negative	EAEDLQVGLEGQEEEE	EAEDLQVG	LEGQEEEE	CMGA	223103	1057286
HIP_6		ELISPOT negative	EAEDLQVGSSPETLIS	EAEDLQVG	SSPETLIS	NPY	32184	1057181
HIP_7		ELISPOT negative	EAEDLQVGMPVDNRNH	EAEDLQVG	MPVDNRNH	SCG1	322195	1057279
HIP_8		ELISPOT negative	EAEDLQVGFLGEGHH	EAEDLQVG	FLGEGHH	SCG1	332401	1057298
HIP_9		ELISPOT negative	EAEDLQVGSAEFPDFY	EAEDLQVG	SAEFPDFY	SCG1	293214	1057218
HIP_10		ELISPOT negative	EAEDLQVGTNEIVEEQ	EAEDLQVG	TNEIVEEQ	SCG2	323413	1057320
HIP_11	Q9	ELISPOT negative	AEDLQVGQFVNQHLCG	AEDLQVGQ	FVNQHLCG	INSB	4823	1057358
HIP_12		ELISPOT negative	AEDLQVGQEAEDLQVG	AEDLQVGQ	EAEDLQVG	INSC	5111	1057341
HIP_13		ELISPOT negative	AEDLQVGQGIVEQCCT	AEDLQVGQ	GIVEQCCT	INSA	1034	1057153
HIP_14		ELISPOT negative	AEDLQVGQTPIESHQV	AEDLQVGQ	TPIESHQV	IAPP	43252	1057356
HIP_15		ELISPOT negative	AEDLQVGQLEGQEEEE	AEDLQVGQ	LEGQEEEE	CMGA	223819	1057291
HIP_16		ELISPOT negative	AEDLQVGQSSPETLIS	AEDLQVGQ	SSPETLIS	NPY	32886	1057186
HIP_17		ELISPOT negative	AEDLQVGQMPVDNRNH	AEDLQVGQ	MPVDNRNH	SCG1	337910	1057311
HIP_18		ELISPOT negative	AEDLQVGQFLGEGHH	AEDLQVGQ	FLGEGHH	SCG1	339008	1057308
HIP_19		ELISPOT negative	AEDLQVGQSAEFPDFY	AEDLQVGQ	SAEFPDFY	SCG1	310602	1057253
HIP_20		ELISPOT negative	AEDLQVGQTNEIVEEQ	AEDLQVGQ	TNEIVEEQ	SCG2	324323	1057321
HIP_21	V10	ELISPOT negative	EDLQVGQVFVNQHLCG	EDLQVGQV	FVNQHLCG	INSB	4119	1057246
HIP_22		ELISPOT negative	EDLQVGQVEAEDLQVG	EDLQVGQVE	AEDLQVG	INSC	4858	1057259
HIP_23		ELISPOT negative	EDLQVGQVGIVEQCCT	EDLQVGQV	GIVEQCCT	INSA	916	1057151
HIP_24		ELISPOT negative	EDLQVGQVTPIESHQV	EDLQVGQV	TPIESHQV	IAPP	38240	1057255
HIP_25		ELISPOT negative	EDLQVGQVLEGQEEEE	EDLQVGQV	LEGQEEEE	CMGA	189104	1057205
HIP_26		ELISPOT negative	EDLQVGQVSSPETLIS	EDLQVGQV	SSPETLIS	NPY	29269	1057169
HIP_27		ELISPOT negative	EDLQVGQVMPVDNRNH	EDLQVGQV	MPVDNRNH	SCG1	232965	1057178
HIP_28		ELISPOT negative	EDLQVGQVFLGEGHH	EDLQVGQV	FLGEGHH	SCG1	248293	1057188
HIP_29		ELISPOT negative	EDLQVGQVSAEFPDFY	EDLQVGQV	SAEFPDFY	SCG1	220332	1057171
HIP_30		ELISPOT negative	EDLQVGQVTNEIVEEQ	EDLQVGQV	TNEIVEEQ	SCG2	251407	1057198
HIP_31	E11	ELISPOT negative	DLQVGQVEFVNQHLCG	DLQVGQVE	FVNQHLCG	INSB	4565	1057276
HIP_32		ELISPOT negative	DLQVGQVEEAEDLQVG	DLQVGQVE	EAEDLQVG	INSC	5014	1057285
HIP_33		ELISPOT negative	DLQVGQVEGIVEQCCT	DLQVGQVE	GIVEQCCT	INSA	3019	1057366
HIP_34		ELISPOT negative	DLQVGQVETPIESHQV	DLQVGQVE	TPIESHQV	IAPP	43783	1057334
HIP_35		ELISPOT negative	DLQVGQVELEGQEEEE	DLQVGQVEL	EGQEEEE	CMGA	108035	1057155
HIP_36		ELISPOT negative	DLQVGQVESSPETLIS	DLQVGQVE	SSPETLIS	NPY	48758	1057346
HIP_37		ELISPOT negative	DLQVGQVEMPVDNRNH	DLQVGQVE	MPVDNRNH	SCG1	340498	1057283
HIP_38		ELISPOT negative	DLQVGQVEFLGEGHH	DLQVGQVE	FLGEGHH	SCG1	351571	1057313
HIP_39		ELISPOT negative	DLQVGQVESAEFPDFY	DLQVGQVE	SAEFPDFY	SCG1	325407	1057245
HIP_40		ELISPOT negative	DLQVGQVETNEIVEEQ	DLQVGQVE	TNEIVEEQ	SCG2	315723	1057265
HIP_41	L12	ELISPOT negative	LQVGQVELFVNQHLCG	LQVGQVEL	FVNQHLCG	INSB	4419	1057240
HIP_42		ELISPOT negative	LQVGQVELEAEDLQVG	LQVGQVEL	EAEDLQVG	INSC	4608	1057238
HIP_43		ELISPOT negative	LQVGQVELGIVEQCCT	LQVGQVEL	GIVEQCCT	INSA	2459	1057214
HIP_44		ELISPOT negative	LQVGQVELTPIESHQV	LQVGQVEL	TPIESHQV	IAPP	36041	1057203
HIP_45		ELISPOT negative	LQVGQVELLEGQEEEE	LQVGQVEL	LEGQEEEE	CMGA	151710	1057172
HIP_46		ELISPOT negative	LQVGQVELSSPETLIS	LQVGQVEL	SSPETLIS	NPY	29353	1057162
HIP_47		ELISPOT negative	LQVGQVELMPVDNRNH	LQVGQVEL	MPVDNRNH	SCG1	281861	1057193
HIP_48		ELISPOT negative	LQVGQVELFLGEGHH	LQVGQVEL	FLGEGHH	SCG1	243851	1057177
HIP_49		ELISPOT negative	LQVGQVELSAEFPDFY	LQVGQVEL	SAEFPDFY	SCG1	109272	1057149
HIP_50		ELISPOT negative	LQVGQVELTNEIVEEQ	LQVGQVEL	TNEIVEEQ	SCG2	232284	1057180
HIP_51	G13	ELISPOT negative	QVGQVELGFVNQHLCG	QVGQVELG	FVNQHLCG	INSB	4811	1057347
HIP_52		ELISPOT negative	QVGQVELGEAEDLQVG	QVGQVELG	EAEDLQVG	INSC	5155	1057332
HIP_53		ELISPOT negative	QVGQVELGGIVEQCCT	QVGQVELGG	IVEQCCT	INSA	2922	1057350
HIP_54		ELISPOT negative	QVGQVELGTPIESHQV	QVGQVELG	TPIESHQV	IAPP	44932	1057370
HIP_55		ELISPOT negative	QVGQVELGLEGQEEEE	QVGQVELG	LEGQEEEE	CMGA	240535	1057352
HIP_56		ELISPOT negative	QVGQVELGSSPETLIS	QVGQVELG	SSPETLIS	NPY	48132	1057348
HIP_57		ELISPOT negative	QVGQVELGMPVDNRNH	QVGQVELG	MPVDNRNH	SCG1	333833	1057293
HIP_58		ELISPOT negative	QVGQVELGFLGEGHH	QVGQVELG	FLGEGHH	SCG1	348568	1057324
HIP_59		ELISPOT negative	QVGQVELGSAEFPDFY	QVGQVELG	SAEFPDFY	SCG1	344957	1057304
HIP_60		ELISPOT negative	QVGQVELGTNEIVEEQ	QVGQVELG	TNEIVEEQ	SCG2	332073	1057330
HIP_61	G14	ELISPOT negative	VGQVELGGFVNQHLCG	VGQVELGG	FVNQHLCG	INSB	4547	1057303
HIP_62		ELISPOT negative	VGQVELGGEAEDLQVG	VGQVELGG	EAEDLQVG	INSC	4997	1057331
HIP_63		ELISPOT negative	VGQVELGGGIVEQCCT	VGQVELGGG	IVEQCCT	INSA	2381	1057213
HIP_64		ELISPOT negative	VGQVELGGTPIESHQV	VGQVELGG	TPIESHQV	IAPP	44941	1057373
HIP_65		ELISPOT negative	VGQVELGGLEGQEEEE	VGQVELGG	LEGQEEEE	CMGA	223158	1057302
HIP_66		ELISPOT negative	VGQVELGGSSPETLIS	VGQVELGG	SSPETLIS	NPY	41540	1057250
HIP_67		ELISPOT negative	VGQVELGGMPVDNRNH	VGQVELGG	MPVDNRNH	SCG1	343998	1057327
HIP_68		ELISPOT negative	VGQVELGGFLGEGHH	VGQVELGG	FLGEGHH	SCG1	359285	1057365
HIP_69		ELISPOT negative	VGQVELGGSAEFPDFY	VGQVELGG	SAEFPDFY	SCG1	335926	1057306
HIP_70		ELISPOT negative	VGQVELGGTNEIVEEQ	VGQVELGG	TNEIVEEQ	SCG2	308688	1057296
HIP_71	G15	ELISPOT positive	GQVELGGGFVNQHLCG	GQVELGGG	FVNQHLCG	INSB	44	409
HIP_72		ELISPOT positive	GQVELGGGEAEDLQVG	GQVELGGG	EAEDLQVG	INSC	801	937
HIP_73		ELISPOT positive	GQVELGGGGIVEQCCT	GQVELGGG	GIVEQCCT	INSA	3	113
HIP_74		ELISPOT positive	GQVELGGGTPIESHQV	GQVELGGG	TPIESHQV	IAPP	18	71
HIP_75		ELISPOT positive	GQVELGGGLEGQEEEE	GQVELGGG	LEGQEEEE	CMGA	54992	979
HIP_76		ELISPOT positive	GQVELGGGSSPETLIS	GQVELGGG	SSPETLIS	NPY	125	303
HIP_77		ELISPOT positive	GQVELGGGMPVDNRNH	GQVELGGG	MPVDNRNH	SCG1	32163	873
HIP_78		ELISPOT positive	GQVELGGGFLGEGHH	GQVELGGG	FLGEGHH	SCG1	21026	768
HIP_79		ELISPOT positive	GQVELGGGSAEFPDFY	GQVELGGG	SAEFPDFY	SCG1	36538	894
HIP_80		ELISPOT positive	GQVELGGGTNEIVEEQ	GQVELGGG	TNEIVEEQ	SCG2	12452	704
HIP_81	P16	ELISPOT negative	QVELGGGPFVNQHLCG	QVELGGGP	FVNQHLCG	INSB	4384	1057264
HIP_82		ELISPOT negative	QVELGGGPEAEDLQVG	QVELGGGP	EAEDLQVG	INSC	4714	1057294
HIP_83		ELISPOT negative	QVELGGGPGIVEQCCT	QVELGGGPG	IVEQCCT	INSA	737	1057148
HIP_84		ELISPOT negative	QVELGGGPTPIESHQV	QVELGGGP	TPIESHQV	IAPP	26256	1057170
HIP_85		ELISPOT negative	QVELGGGPLEGQEEEE	QVELGGGP	LEGQEEEE	CMGA	185095	1057204
HIP_86		ELISPOT negative	QVELGGGPSSPETLIS	QVELGGGP	SSPETLIS	NPY	34059	1057187
HIP_87		ELISPOT negative	QVELGGGPMPVDNRNH	QVELGGGP	MPVDNRNH	SCG1	255589	1057190
HIP_88		ELISPOT negative	QVELGGGPFLGEGHH	QVELGGGP	FLGEGHH	SCG1	298385	1057237
HIP_89		ELISPOT negative	QVELGGGPSAEFPDFY	QVELGGGP	SAEFPDFY	SCG1	283159	1057215
HIP_90		ELISPOT negative	QVELGGGPTNEIVEEQ	QVELGGGP	TNEIVEEQ	SCG2	238951	1057191
HIP_91	G17	ELISPOT negative	VELGGGPGFVNQHLCG	VELGGGPG	FVNQHLCG	INSB	4517	1057288
HIP_92		ELISPOT negative	VELGGGPGEAEDLQVG	VELGGGPG	EAEDLQVG	INSC	4295	1057241
HIP_93		ELISPOT negative	VELGGGPGGIVEQCCT	VELGGGPG	GIVEQCCT	INSA	1743	1057168
HIP_94		ELISPOT negative	VELGGGPGTPIESHQV	VELGGGPG	TPIESHQV	IAPP	25487	1057166
HIP_95		ELISPOT negative	VELGGGPGLEGQEEEE	VELGGGPG	LEGQEEEE	CMGA	174583	1057196
HIP_96		ELISPOT negative	VELGGGPGSSPETLIS	VELGGGPG	SSPETLIS	NPY	32680	1057179
HIP_97		ELISPOT negative	VELGGGPGMPVDNRNH	VELGGGPG	MPVDNRNH	SCG1	187499	1057159
HIP_98		ELISPOT negative	VELGGGPGFLGEGHH	VELGGGPG	FLGEGHH	SCG1	240991	1057183
HIP_99		ELISPOT negative	VELGGGPGSAEFPDFY	VELGGGPG	SAEFPDFY	SCG1	291704	1057223
HIP_100		ELISPOT negative	VELGGGPGTNEIVEEQ	VELGGGPG	TNEIVEEQ	SCG2	261033	1057206
HIP_101	A18	ELISPOT positive	ELGGGPGAFVNQHLCG	ELGGGPGA	FVNQHLCG	INSB	11	282
HIP_102		ELISPOT positive	ELGGGPGAEAEDLQVG	ELGGGPGA	EAEDLQVG	INSC	415	852
HIP_103		ELISPOT positive	ELGGGPGAGIVEQCCT	ELGGGPGA	GIVEQCCT	INSA	10	324
HIP_104		ELISPOT positive	ELGGGPGATPIESHQV	ELGGGPGA	TPIESHQV	IAPP	1159	662
HIP_105		ELISPOT positive	ELGGGPGALEGQEEEE	ELGGGPGA	LEGQEEEE	CMGA	14617	789
HIP_106		ELISPOT positive	ELGGGPGASSPETLIS	ELGGGPGA	SSPETLIS	NPY	469	472
HIP_107		ELISPOT positive	ELGGGPGAMPVDNRNH	ELGGGPGA	MPVDNRNH	SCG1	14333	726
HIP_108		ELISPOT positive	ELGGGPGAFLGEGHH	ELGGGPGA	FLGEGHH	SCG1	20803	747
HIP_109		ELISPOT positive	ELGGGPGASAEFPDFY	ELGGGPGA	SAEFPDFY	SCG1	25466	810
HIP_110		ELISPOT positive	ELGGGPGATNEIVEEQ	ELGGGPGA	TNEIVEEQ	SCG2	9301	683
HIP_111	G19	ELISPOT negative	LGGGPGAGFVNQHLCG	LGGGPGAG	FVNQHLCG	INSB	3855	1057212
HIP_112		ELISPOT negative	LGGGPGAGEAEDLQVG	LGGGPGAG	EAEDLQVG	INSC	4927	1057317
HIP_113		ELISPOT negative	LGGGPGAGGIVEQCCT	LGGGPGAG	GIVEQCCT	INSA	1777	1057173
HIP_114		ELISPOT negative	LGGGPGAGTPIESHQV	LGGGPGAG	TPIESHQV	IAPP	40351	1057287
HIP_115		ELISPOT negative	LGGGPGAGLEGQEEEE	LGGGPGAG	LEGQEEEE	CMGA	196932	1057226
HIP_116		ELISPOT negative	LGGGPGAGSSPETLIS	LGGGPGAG	SSPETLIS	NPY	39373	1057216
HIP_117		ELISPOT negative	LGGGPGAGMPVDNRNH	LGGGPGAG	MPVDNRNH	SCG1	244064	1057185
HIP_118		ELISPOT negative	LGGGPGAGFLGEGHH	LGGGPGAG	FLGEGHH	SCG1	298738	1057236
HIP_119		ELISPOT negative	LGGGPGAGSAEFPDFY	LGGGPGAG	SAEFPDFY	SCG1	292006	1057221
HIP_120		ELISPOT negative	LGGGPGAGTNEIVEEQ	LGGGPGAG	TNEIVEEQ	SCG2	291766	1057257
HIP_121	S20	ELISPOT negative	GGGPGAGSFVNQHLCG	GGGPGAGS	FVNQHLCG	INSB	4102	1057235
HIP_122		ELISPOT negative	GGGPGAGSEAEDLQVG	GGGPGAGS	EAEDLQVG	INSC	4837	1057289
HIP_123		ELISPOT negative	GGGPGAGSGIVEQCCT	GGGPGAGS	GIVEQCCT	INSA	1677	1057163
HIP_124		ELISPOT negative	GGGPGAGSTPIESHQV	GGGPGAGS	TPIESHQV	IAPP	41980	1057307
HIP_125		ELISPOT negative	GGGPGAGSLEGQEEEE	GGGPGAGSL	EGQEEEE	CMGA	50156	1057147
HIP_126		ELISPOT negative	GGGPGAGSSSPETLIS	GGGPGAGS	SSPETLIS	NPY	31634	1057176
HIP_127		ELISPOT negative	GGGPGAGSMPVDNRNH	GGGPGAG	SMPVDNRNH	SCG1	177630	1057156
HIP_128		ELISPOT negative	GGGPGAGSFLGEGHH	GGGPGAGS	FLGEGHH	SCG1	297231	1057222
HIP_129		ELISPOT negative	GGGPGAGSSAEFPDFY	GGGPGAGS	SAEFPDFY	SCG1	267916	1057197
HIP_130		ELISPOT negative	GGGPGAGSTNEIVEEQ	GGGPGAGS	TNEIVEEQ	SCG2	258684	1057202
HIP_131	L21	ELISPOT negative	GGPGAGSLFVNQHLCG	GGPGAGSL	FVNQHLCG	INSB	4750	1057322
HIP_132		ELISPOT negative	GGPGAGSLEAEDLQVG	GGPGAGSL	EAEDLQVG	INSC	4808	1057282
HIP_133		ELISPOT negative	GGPGAGSLGIVEQCCT	GGPGAGSL	GIVEQCCT	INSA	1570	1057160
HIP_134		ELISPOT negative	GGPGAGSLTPIESHQV	GGPGAGSL	TPIESHQV	IAPP	43541	1057338
HIP_135		ELISPOT negative	GGPGAGSLLEGQEEEE	GGPGAGSL	LEGQEEEE	CMGA	214935	1057266
HIP_136		ELISPOT negative	GGPGAGSLSSPETLIS	GGPGAGSL	SSPETLIS	NPY	33505	1057182
HIP_137		ELISPOT negative	GGPGAGSLMPVDNRNH	GGPGAGSL	MPVDNRNH	SCG1	274798	1057200
HIP_138		ELISPOT negative	GGPGAGSLFLGEGHH	GGPGAGSL	FLGEGHH	SCG1	300912	1057232
HIP_139		ELISPOT negative	GGPGAGSLSAEFPDFY	GGPGAGSL	SAEFPDFY	SCG1	287921	1057210
HIP_140		ELISPOT negative	GGPGAGSLTNEIVEEQ	GGPGAGSL	TNEIVEEQ	SCG2	240979	1057192
HIP_141	Q22	ELISPOT negative	GPGAGSLQFVNQHLCG	GPGAGSLQ	FVNQHLCG	INSB	4681	1057290
HIP_142		ELISPOT negative	GPGAGSLQEAEDLQVG	GPGAGSLQ	EAEDLQVG	INSC	5062	1057305
HIP_143		ELISPOT negative	GPGAGSLQGIVEQCCT	GPGAGSLQ	GIVEQCCT	INSA	1737	1057164
HIP_144		ELISPOT negative	GPGAGSLQTPIESHQV	GPGAGSLQ	TPIESHQV	IAPP	42907	1057312
HIP_145		ELISPOT negative	GPGAGSLQLEGQEEEE	GPGAGSLQ	LEGQEEEE	CMGA	226465	1057295
HIP_146		ELISPOT negative	GPGAGSLQSSPETLIS	GPGAGSLQ	SSPETLIS	NPY	39171	1057211
HIP_147		ELISPOT negative	GPGAGSLQMPVDNRNH	GPGAGSLQ	MPVDNRNH	SCG1	337098	1057269
HIP_148		ELISPOT negative	GPGAGSLQFLGEGHH	GPGAGSLQ	FLGEGHH	SCG1	340126	1057284
HIP_149		ELISPOT negative	GPGAGSLQSAEFPDFY	GPGAGSLQ	SAEFPDFY	SCG1	287692	1057201
HIP_150		ELISPOT negative	GPGAGSLQTNEIVEEQ	GPGAGSLQ	TNEIVEEQ	SCG2	298400	1057249
HIP_151	P23	ELISPOT negative	PGAGSLQPFVNQHLCG	PGAGSLQP	FVNQHLCG	INSB	4272	1057243
HIP_152		ELISPOT negative	PGAGSLQPEAEDLQVG	PGAGSLQP	EAEDLQVG	INSC	4888	1057277
HIP_153		ELISPOT negative	PGAGSLQPGIVEQCCT	PGAGSLQP	GIVEQCCT	INSA	1522	1057157
HIP_154		ELISPOT negative	PGAGSLQPTPIESHQV	PGAGSLQP	TPIESHQV	IAPP	41377	1057272
HIP_155		ELISPOT negative	PGAGSLQPLEGQEEEE	PGAGSLQPL	EGQEEEE	CMGA	92891	1057154
HIP_156		ELISPOT negative	PGAGSLQPSSPETLIS	PGAGSLQP	SSPETLIS	NPY	39392	1057208
HIP_157		ELISPOT negative	PGAGSLQPMPVDNRNH	PGAGSLQP	MPVDNRNH	SCG1	308151	1057220
HIP_158		ELISPOT negative	PGAGSLQPFLGEGHH	PGAGSLQP	FLGEGHH	SCG1	334278	1057278
HIP_159		ELISPOT negative	PGAGSLQPSAEFPDFY	PGAGSLQP	SAEFPDFY	SCG1	314412	1057242
HIP_160		ELISPOT negative	PGAGSLQPTNEIVEEQ	PGAGSLQP	TNEIVEEQ	SCG2	270887	1057209
HIP_161	L24	ELISPOT negative	GAGSLQPLFVNQHLCG	GAGSLQPL	FVNQHLCG	INSB	4551	1057275
HIP_162		ELISPOT negative	GAGSLQPLEAEDLQVG	GAGSLQPL	EAEDLQVG	INSC	4835	1057268
HIP_163		ELISPOT negative	GAGSLQPLGIVEQCCT	GAGSLQPL	GIVEQCCT	INSA	1643	1057161
HIP_164		ELISPOT negative	GAGSLQPLTPIESHQV	GAGSLQPL	TPIESHQV	IAPP	41134	1057267
HIP_165		ELISPOT negative	GAGSLQPLLEGQEEEE	GAGSLQPL	LEGQEEEE	CMGA	221017	1057281
HIP_166		ELISPOT negative	GAGSLQPLSSPETLIS	GAGSLQPL	SSPETLIS	NPY	41531	1057231
HIP_167		ELISPOT negative	GAGSLQPLMPVDNRNH	GAGSLQPL	MPVDNRNH	SCG1	264052	1057189
HIP_168		ELISPOT negative	GAGSLQPLFLGEGHH	GAGSLQPL	FLGEGHH	SCG1	312417	1057244
HIP_169		ELISPOT negative	GAGSLQPLSAEFPDFY	GAGSLQPL	SAEFPDFY	SCG1	323811	1057256
HIP_170		ELISPOT negative	GAGSLQPLTNEIVEEQ	GAGSLQPL	TNEIVEEQ	SCG2	295888	1057254
HIP_171	A25	ELISPOT negative	AGSLQPLAFVNQHLCG	AGSLQPLA	FVNQHLCG	INSB	4513	1057260
HIP_172		ELISPOT negative	AGSLQPLAEAEDLQVG	AGSLQPLA	EAEDLQVG	INSC	5012	1057309
HIP_173		ELISPOT negative	AGSLQPLAGIVEQCCT	AGSLQPLA	GIVEQCCT	INSA	1589	1057158
HIP_174		ELISPOT negative	AGSLQPLATPIESHQV	AGSLQPLA	TPIESHQV	IAPP	37534	1057227
HIP_175		ELISPOT negative	AGSLQPLALEGQEEEE	AGSLQPLAL	EGQEEEE	CMGA	67367	1057150
HIP_176		ELISPOT negative	AGSLQPLASSPETLIS	AGSLQPLA	SSPETLIS	NPY	39651	1057217
HIP_177		ELISPOT negative	AGSLQPLAMPVDNRNH	AGSLQPLA	MPVDNRNH	SCG1	227652	1057167
HIP_178		ELISPOT negative	AGSLQPLAFLGEGHH	AGSLQPLA	FLGEGHH	SCG1	234169	1057174
HIP_179		ELISPOT negative	AGSLQPLASAEFPDFY	AGSLQPLA	SAEFPDFY	SCG1	275219	1057195
HIP_180		ELISPOT negative	AGSLQPLATNEIVEEQ	AGSLQPLA	TNEIVEEQ	SCG2	217515	1057175
HIP_181	L26	ELISPOT positive	GSLQPLALFVNQHLCG	GSLQPLAL	FVNQHLCG	INSB	23	367
HIP_182		ELISPOT positive	GSLQPLALEAEDLQVG	GSLQPLAL	EAEDLQVG	INSC	131	641
HIP_183		ELISPOT positive	GSLQPLALGIVEQCCT	GSLQPLAL	GIVEQCCT	INSA	6	240
HIP_184		ELISPOT positive	GSLQPLALTPIESHQV	GSLQPLAL	TPIESHQV	IAPP	393	430
HIP_185		ELISPOT positive	GSLQPLALLEGQEEEE	GSLQPLAL	LEGQEEEE	CMGA	2300	451
HIP_186		ELISPOT positive	GSLQPLALSSPETLIS	GSLQPLAL	SSPETLIS	NPY	28	92
HIP_187		ELISPOT positive	GSLQPLALMPVDNRNH	GSLQPLAL	MPVDNRNH	SCG1	4097	514
HIP_188		ELISPOT positive	GSLQPLALFLGEGHH	GSLQPLAL	FLGEGHH	SCG1	6697	557
HIP_189		ELISPOT positive	GSLQPLALSAEFPDFY	GSLQPLAL	SAEFPDFY	SCG1	2600	388
HIP_190		ELISPOT positive	GSLQPLALTNEIVEEQ	GSLQPLAL	TNEIVEEQ	SCG2	8385	620
HIP_191	E27	ELISPOT negative	SLQPLALEFVNQHLCG	SLQPLALE	FVNQHLCG	INSB	4809	1057292
HIP_192		ELISPOT negative	SLQPLALEEAEDLQVG	SLQPLALE	EAEDLQVG	INSC	4947	1057273
HIP_193		ELISPOT negative	SLQPLALEGIVEQCCT	SLQPLALE	GIVEQCCT	INSA	2967	1057333
HIP_194		ELISPOT negative	SLQPLALETPIESHQV	SLQPLALE	TPIESHQV	IAPP	43714	1057315
HIP_195		ELISPOT negative	SLQPLALELEGQEEEE	SLQPLALE	LEGQEEEE	CMGA	210424	1057248
HIP_196		ELISPOT negative	SLQPLALESSPETLIS	SLQPLALE	SSPETLIS	NPY	36620	1057194
HIP_197		ELISPOT negative	SLQPLALEMPVDNRNH	SLQPLALE	MPVDNRNH	SCG1	318721	1057230
HIP_198		ELISPOT negative	SLQPLALEFLGEGHH	SLQPLALE	FLGEGHH	SCG1	351111	1057301
HIP_199		ELISPOT negative	SLQPLALESAEFPDFY	SLQPLALE	SAEFPDFY	SCG1	262886	1057184
HIP_200		ELISPOT negative	SLQPLALETNEIVEEQ	SLQPLALE	TNEIVEEQ	SCG2	289469	1057229
HIP_201	G28	ELISPOT negative	LQPLALEGFVNQHLCG	LQPLALEG	FVNQHLCG	INSB	4827	1057349
HIP_202		ELISPOT negative	LQPLALEGEAEDLQVG	LQPLALEG	EAEDLQVG	INSC	5165	1057337
HIP_203		ELISPOT negative	LQPLALEGGIVEQCCT	LQPLALEG	GIVEQCCT	INSA	2956	1057364
HIP_204		ELISPOT negative	LQPLALEGTPIESHQV	LQPLALEG	TPIESHQV	IAPP	44937	1057372
HIP_205		ELISPOT negative	LQPLALEGLEGQEEEE	LQPLALEG	LEGQEEEE	CMGA	242733	1057362
HIP_206		ELISPOT negative	LQPLALEGSSPETLIS	LQPLALEGS	SPETLIS	NPY	48893	1057325
HIP_207		ELISPOT negative	LQPLALEGMPVDNRNH	LQPLALEG	MPVDNRNH	SCG1	335759	1057300
HIP_208		ELISPOT negative	LQPLALEGFLGEGHH	LQPLALEG	FLGEGHH	SCG1	360928	1057360
HIP_209		ELISPOT negative	LQPLALEGSAEFPDFY	LQPLALEGS	AEFPDFY	SCG1	350905	1057318
HIP_210		ELISPOT negative	LQPLALEGTNEIVEEQ	LQPLALEG	TNEIVEEQ	SCG2	333581	1057336
HIP_211	S29	ELISPOT negative	QPLALEGSFVNQHLCG	QPLALEGS	FVNQHLCG	INSB	4228	1057252
HIP_212		ELISPOT negative	QPLALEGSEAEDLQVG	QPLALEGS	EAEDLQVG	INSC	5025	1057316
HIP_213		ELISPOT negative	QPLALEGSGIVEQCCT	QPLALEGS	GIVEQCCT	INSA	3012	1057369
HIP_214		ELISPOT negative	QPLALEGSTPIESHQV	QPLALEGS	TPIESHQV	IAPP	43916	1057361
HIP_215		ELISPOT negative	QPLALEGSLEGQEEEE	QPLALEGSL	EGQEEEE	CMGA	85184	1057152
HIP_216		ELISPOT negative	QPLALEGSSSPETLIS	QPLALEGS	SSPETLIS	NPY	48043	1057328
HIP_217		ELISPOT negative	QPLALEGSMPVDNRNH	QPLALEGS	MPVDNRNH	SCG1	304067	1057239
HIP_218		ELISPOT negative	QPLALEGSFLGEGHH	QPLALEGS	FLGEGHH	SCG1	325818	1057270
HIP_219		ELISPOT negative	QPLALEGSSAEFPDFY	QPLALEGS	SAEFPDFY	SCG1	310900	1057251
HIP_220		ELISPOT negative	QPLALEGSTNEIVEEQ	QPLALEGS	TNEIVEEQ	SCG2	285526	1057225
HIP_221	L30	ELISPOT negative	PLALEGSLFVNQHLCG	PLALEGSL	FVNQHLCG	INSB	4944	1057368
HIP_222		ELISPOT negative	PLALEGSLEAEDLQVG	PLALEGSL	EAEDLQVG	INSC	5180	1057359
HIP_223		ELISPOT negative	PLALEGSLGIVEQCCT	PLALEGSL	GIVEQCCT	INSA	2553	1057258
HIP_224		ELISPOT negative	PLALEGSLTPIESHQV	PLALEGSL	TPIESHQV	IAPP	44813	1057371
HIP_225		ELISPOT negative	PLALEGSLLEGQEEEE	PLALEGSL	LEGQEEEE	CMGA	235232	1057344
HIP_226		ELISPOT negative	PLALEGSLSSPETLIS	PLALEGSL	SSPETLIS	NPY	36337	1057199
HIP_227		ELISPOT negative	PLALEGSLMPVDNRNH	PLALEGSL	MPVDNRNH	SCG1	292690	1057219
HIP_228		ELISPOT negative	PLALEGSLFLGEGHH	PLALEGSL	FLGEGHH	SCG1	344796	1057319
HIP_229		ELISPOT negative	PLALEGSLSAEFPDFY	PLALEGSL	SAEFPDFY	SCG1	320587	1057261
HIP_230		ELISPOT negative	PLALEGSLTNEIVEEQ	PLALEGSL	TNEIVEEQ	SCG2	327770	1057342
HIP_231	Q31	ELISPOT negative	LALEGSLQFVNQHLCG	LALEGSLQ	FVNQHLCG	INSB	4903	1057345
HIP_232		ELISPOT negative	LALEGSLQEAEDLQVG	LALEGSLQ	EAEDLQVG	INSC	5070	1057310
HIP_233		ELISPOT negative	LALEGSLQGIVEQCCT	LALEGSLQ	GIVEQCCT	INSA	3002	1057367
HIP_234		ELISPOT negative	LALEGSLQTPIESHQV	LALEGSLQ	TPIESHQV	IAPP	44218	1057355
HIP_235		ELISPOT negative	LALEGSLQLEGQEEEE	LALEGSLQ	LEGQEEEE	CMGA	234517	1057323
HIP_236		ELISPOT negative	LALEGSLQSSPETLIS	LALEGSLQ	SSPETLIS	NPY	40791	1057234
HIP_237		ELISPOT negative	LALEGSLQMPVDNRNH	LALEGSLQ	MPVDNRNH	SCG1	332695	1057274
HIP_238		ELISPOT negative	LALEGSLQFLGEGHH	LALEGSLQ	FLGEGHH	SCG1	357283	1057339
HIP_239		ELISPOT negative	LALEGSLQSAEFPDFY	LALEGSLQ	SAEFPDFY	SCG1	317544	1057247
HIP_240		ELISPOT negative	LALEGSLQTNEIVEEQ	LALEGSLQ	TNEIVEEQ	SCG2	319510	1057297

Candidate information						XGBoost		Global model		
HIP candidate ID	Left part	Right part	Sequence	Real or ELISPOT positive HIP?	Right source protein	XGB rank	XGB score normalised (0-1)	Global rank	Global score normalised (0-1)	Cosine similarity
HIP_1	AGSLQPLAL	QRYGKRSSP	AGSLQPLALQRYGKRSSP		NPY	395/49410	0.998	1	0.905	0.209700
HIP_2	SHLVEALYL	QRYGKRSSP	SHLVEALYLQRYGKRSSP		NPY	230/49410	0.999	2	0.903	0.188190
HIP_3	TSICSLYQL	QQWPERKLK	TSICSLYQLQQWPERKLK		SCG2	812/334890	1.000	3	0.903	0.116221
HIP_4	QCCTSICSL	QRYGKRSSP	QCCTSICSLQRYGKRSSP		NPY	1191/49410	0.994	4	0.902	0.139580
HIP_5	AGSLQPLAL	RYYSALRHY	AGSLQPLALRYYSALRHY		NPY	267/49410	0.999	5	0.902	0.134065
HIP_6	TSICSLYQL	ITRQRYGKR	TSICSLYQLITRQRYGKR		NPY	576/49410	0.997	6	0.901	0.069597
HIP_7	AGSLQPLAL	RQRYGKRSS	AGSLQPLALRQRYGKRSS		NPY	401/49410	0.998	7	0.901	0.222078
HIP_8	CGERGFFYT	RQVLKTSRK	CGERGFFYTRQVLKTSRK		SCG1	4898/367830	0.999	8	0.901	0.102790
HIP_9	SHLVEALYL	TRQRYGKRS	SHLVEALYLTRQRYGKRS		NPY	197/49410	0.999	9	0.901	0.265981
HIP_10	VCGERGFFY	QRYGKRSSP	VCGERGFFYQRYGKRSSP		NPY	238/49410	0.999	10	0.900	0.163481
HIP_11	CTSICSLYQ	WKSSHFERR	CTSICSLYQWKSSHFERR		SCG1	3596/367830	0.999	11	0.900	0.117318
HIP_12	CGERGFFYT	ITRQRYGKR	CGERGFFYTITRQRYGKR		NPY	471/49410	0.998	12	0.900	0.074331
HIP_13	LVCGERGFF	TRQRYGKRS	LVCGERGFFTRQRYGKRS		NPY	270/49410	0.999	13	0.900	0.139250
HIP_14	LCGSHLVEA	QQWPERKLK	LCGSHLVEAQQWPERKLK		SCG2	6574/334890	0.999	14	0.900	0.143379
HIP_15	CTSICSLYQ	ITRQRYGKR	CTSICSLYQITRQRYGKR		NPY	574/49410	0.997	15	0.899	0.039012
HIP_16	SHLVEALYL	HQVEKRKCN	SHLVEALYLHQVEKRKCN		IAPP	467/45018	0.998	16	0.898	0.150752
HIP_17	GSHLVEALY	QRYGKRSSP	GSHLVEALYQRYGKRSSP		NPY	245/49410	0.999	17	0.898	0.145994
HIP_18	AGSLQPLAL	VDKRRTRPR	AGSLQPLALVDKRRTRPR		SCG1	2165/367830	1.000	18	0.898	0.198707
HIP_19	LVCGERGFF	RQRYGKRSS	LVCGERGFFRQRYGKRSS		NPY	670/49410	0.997	19	0.898	0.128769
HIP_20	CGERGFFYT	KRLGLSGLT	CGERGFFYTKRLGLSGLT		NPY	860/49410	0.996	20	0.898	0.119828
HIP_21	HLVEALYLV	QRYGKRSSP	HLVEALYLVQRYGKRSSP		NPY	241/49410	0.999	21	0.898	0.164456
HIP_22	VCGERGFFY	HQVEKRKCN	VCGERGFFYHQVEKRKCN		IAPP	77/45018	1.000	22	0.898	0.108894
HIP_23	VCGERGFFY	VDKRRTRPR	VCGERGFFYVDKRRTRPR		SCG1	1815/367830	1.000	23	0.897	0.157875
HIP_24	CGERGFFYT	VDKRRTRPR	CGERGFFYTVDKRRTRPR		SCG1	7457/367830	0.999	24	0.897	0.104286
HIP_25	CTSICSLYQ	QQWPERKLK	CTSICSLYQQQWPERKLK		SCG2	1259/334890	1.000	25	0.897	0.086261
HIP_26	ERGFFYTPK	PERKLKHMQ	ERGFFYTPKPERKLKHMQ		SCG2	3348/334890	1.000	26	0.897	0.006716
HIP_27	SHLVEALYL	KRLGLSGLT	SHLVEALYLKRLGLSGLT		NPY	767/49410	0.996	27	0.896	0.198569
HIP_28	SHLVEALYL	ARYYSALRH	SHLVEALYLARYYSALRH		NPY	190/49410	0.999	28	0.896	0.116711
HIP_29	VCGERGFFY	TRQRYGKRS	VCGERGFFYTRQRYGKRS		NPY	232/49410	0.999	29	0.896	0.218897
HIP_30	LCGSHLVEA	RYGKRSSPE	LCGSHLVEARYGKRSSPE		NPY	573/49410	0.997	30	0.896	0.169128
HIP_31	CGERGFFYT	TAEKRKRLG	CGERGFFYTTAEKRKRLG		SCG1	4595/367830	0.999	31	0.896	0.110184
HIP_32	AGSLQPLAL	HQVEKRKCN	AGSLQPLALHQVEKRKCN		IAPP	381/45018	0.999	32	0.895	0.135862
HIP_33	HLVEALYLV	TRQRYGKRS	HLVEALYLVTRQRYGKRS		NPY	219/49410	0.999	33	0.894	0.241786
HIP_34	QHLCGSHLV	QRYGKRSSP	QHLCGSHLVQRYGKRSSP		NPY	1358/49410	0.993	34	0.894	0.205553
HIP_35	VCGERGFFY	SHHTAEKRK	VCGERGFFYSHHTAEKRK		SCG1	2246/367830	1.000	35	0.894	0.123477
HIP_36	SHLVEALYL	FRARAYGFR	SHLVEALYLFRARAYGFR		CMGA	1828/247050	0.999	36	0.894	0.123561
HIP_37	RGFFYTPKT	TQQWPERKL	RGFFYTPKTTQQWPERKL		SCG2	2475/334890	1.000	37	0.894	0.008563
HIP_38	GSHLVEALY	TRQRYGKRS	GSHLVEALYTRQRYGKRS		NPY	236/49410	0.999	38	0.894	0.223297
HIP_39	CTSICSLYQ	KQYSSHHTA	CTSICSLYQKQYSSHHTA		SCG1	8411/367830	0.998	39	0.894	0.101338
HIP_40	SLYQLENYC	QQWPERKLK	SLYQLENYCQQWPERKLK		SCG2	1457/334890	1.000	40	0.894	0.047469

HIP candidate ID	Ridge model coefficients (n=36)																																			
	junction_hydrophobicity	junction_flexibility	charge_balance	length	global_hydrophobicity	total_charge	anchor_residues	asymmetry	repeat_penalty	junction_type_score	delta_hydro	delta_charge	delta_flex	mhc_anchor_score	mhc_charge_penalty	mhc_binding_proxy	proline_count_junction	glycine_count_junction	has_proline_junction	has_glycine_junction	int_hydro_charge	int_hydro_mhc	int_anchor_hydro	aa_0_class_hydrophobic	aa_0_class_polar	aa_0_class_charged	aa_0_class_special	junction_disorder	junction_helix	junction_turn	compactness_proxy	disorder_sq	turn_sq	hydro_disorder	delta_flex_sq	delta_hydro_flex
HIP_1	0.0167	0	3	18	-0.7	3	1	0	0	0.000000	-6.2333	1	0.1	0.450000	0.1111	0.3389	0	0	0	0	-6.2333	0.0056	-6.2333	1	0	0	0	0.3436	1.2005	0.7423	0.3333	0.1181	0.551	0.0057	0.01	-0.6233
HIP_2	-0.5	0	3	18	-0.7056	2	2	0	0	0.000000	-5.2	1	0.1	0.000000	0.2222	-0.2222	0	0	0	0	-5.2	0.1111	-10.4	1	0	0	0	0.3821	1.1291	0.7835	0.1667	0.146	0.6138	-0.191	0.01	-0.52
HIP_3	-1.4833	0	2	18	-0.7778	2	2	0	0	0.000000	-2.3	0	0.05	0.000000	0	0	0	0	0	0	0	0	-4.6	1	0	0	0	0.4769	1.1122	0.8756	0.3333	0.2275	0.7667	-0.7074	0.0025	-0.115
HIP_4	-0.6333	0.1667	3	18	-0.6278	3	1	0	0	0.000000	-4.9333	1	0.0333	0.000000	0.1111	-0.1111	0	0	0	0	-4.9333	0.0704	-4.9333	1	0	0	0	0.4487	1.0458	0.9001	0	0.2013	0.8102	-0.2842	0.0011	-0.1644
HIP_5	0.3833	0	3	18	-0.1444	2	2	0	0	0.000000	-5.5	1	0.05	0.450000	0.1111	0.3389	0	0	0	0	-5.5	0.1299	-11	1	0	0	0	0.3154	1.1858	0.7301	0.3333	0.0995	0.5331	0.1209	0.0025	-0.275
HIP_6	-0.2833	0	4	18	-0.6278	4	1	0	0	1.000000	0.1	1	0.05	1.125000	0.1111	1.0139	0	0	0	0	0.1	-0.2873	0.1	1	0	0	0	0.4103	1.0605	0.7873	0.1667	0.1683	0.6199	-0.1162	0.0025	0.005
HIP_7	-0.5167	0	4	18	-0.8611	4	1	0	0	0.000000	-7.3	2	0.15	0.450000	0.2222	0.2278	0	0	0	0	-14.6	-0.1177	-7.3	1	0	0	0	0.3436	1.2005	0.7423	0.1667	0.1181	0.551	-0.1775	0.0225	-1.095
HIP_8	-0.5	0	4	18	-0.9167	4	3	0	0	0.000000	-1.5333	1	0.05	0.950000	0.1111	0.8389	0	0	0	0	-1.5333	-0.4194	-4.6	0	1	0	0	0.4103	1.0405	0.809	0.1667	0.1683	0.6544	-0.2051	0.0025	-0.0767
HIP_9	-0.4	0	4	18	-0.8611	3	1	0	0	0.000000	-5	1	0.15	0.000000	0.3333	-0.3333	0	0	0	0	-5	0.1333	-5	1	0	0	0	0.4103	1.0972	0.7923	0.1667	0.1683	0.6278	-0.1641	0.0225	-0.75
HIP_10	-0.8333	0	3	18	-1.0611	3	4	0	0	0.000000	-4.5333	1	0.1	0.700000	0.2222	0.4778	0	0	0	0	-4.5333	-0.3981	-18.1333	0	1	0	0	0.3821	1.0958	0.8168	0.1667	0.146	0.6672	-0.3184	0.01	-0.4533
HIP_11	-1.1	0.1667	3	18	-0.7278	2	2	0	0	0.000000	-1.5333	1	0.0667	0.625000	0.1111	0.5139	0	0	0	0	-1.5333	-0.5653	-3.0667	0	1	0	0	0.4487	1.0458	0.9001	0.1667	0.2013	0.8102	-0.4936	0.0044	-0.1022
HIP_12	0.0167	0	4	18	-1.1944	4	3	0	0	0.000000	-0.5	1	0.05	1.125000	0.1111	1.0139	0	0	0	0	-0.5	0.0169	-1.5	0	1	0	0	0.4103	0.9972	0.8006	0.1667	0.1683	0.641	0.0068	0.0025	-0.025
HIP_13	-0.5833	0.1667	4	18	-0.9333	4	2	0	0	0.000000	-4.6333	1	0.0667	0.700000	0.4444	0.2556	0	1	0	1	-4.6333	-0.1491	-9.2667	1	0	0	0	0.4551	0.9769	0.9245	0.1667	0.2071	0.8547	-0.2655	0.0044	-0.3089
HIP_14	-0.9	0	2	18	-0.7389	1	1	0	0	0.000000	-3.4667	1	0.05	0.000000	0.1111	-0.1111	0	0	0	0	-3.4667	0.1	-3.4667	1	0	0	0	0.4385	1.2103	0.7945	0.3333	0.1922	0.6312	-0.3946	0.0025	-0.1733
HIP_15	-0.2833	0	4	18	-0.7	4	1	0	0	0.000000	0.1	1	0.05	1.750000	0.1111	1.6389	0	0	0	0	0.1	-0.4644	0.1	0	1	0	0	0.4103	1.0605	0.7873	0.1667	0.1683	0.6199	-0.1162	0.0025	0.005
HIP_16	0.6333	0	3	18	-0.5944	1	1	0	0	0.000000	-2.9333	0	0.05	0.000000	0.2222	-0.2222	0	0	0	0	0	-0.1407	-2.9333	1	0	0	0	0.3436	1.1355	0.7156	0.3333	0.1181	0.5121	0.2176	0.0025	-0.1467
HIP_17	-0.8333	0	3	18	-0.9389	2	2	0	0	0.000000	-4.5333	1	0.1	2.000000	0.2222	1.7778	0	0	0	0	-4.5333	-1.4815	-9.0667	0	1	0	0	0.3821	1.1541	0.8101	0.1667	0.146	0.6563	-0.3184	0.01	-0.4533
HIP_18	1.0333	0	4	18	-0.8222	4	0	0	0	1.000000	-4.2	0	0.1	1.500000	0.3333	1.1667	0	0	0	0	0	1.2056	0	1	0	0	0	0.2769	1.1922	0.6623	0.3333	0.0767	0.4387	0.2862	0.01	-0.42
HIP_19	-1.2167	0.1667	4	18	-0.9389	4	3	0	0	0.000000	-5.9	2	0.0667	0.700000	0.4444	0.2556	0	1	0	1	-11.8	-0.3109	-17.7	1	0	0	0	0.4269	1.0088	0.9156	0	0.1823	0.8384	-0.5194	0.0044	-0.3933
HIP_20	-0.6333	0	2	18	-0.1111	2	3	0	0	0.000000	-1.8	2	0.05	0.000000	0.2222	-0.2222	0	0	0	0	-3.6	0.1407	-5.4	0	1	0	0	0.3821	1.0658	0.7968	0	0.146	0.6349	-0.242	0.0025	-0.09
HIP_21	-0.4333	0	3	18	-0.4278	2	2	0	0	0.000000	-5.3333	1	0.1	1.400000	0.1111	1.2889	0	0	0	0	-5.3333	-0.5585	-10.6667	1	0	0	0	0.3821	1.0891	0.7835	0.1667	0.146	0.6138	-0.1656	0.01	-0.5333
HIP_22	0.3	0	3	18	-0.95	2	3	0	0	0.000000	-2.2667	0	0.05	0.700000	0.2222	0.4778	0	0	0	0	0	0.1433	-6.8	0	1	0	0	0.3436	1.1022	0.749	0.3333	0.1181	0.561	0.1031	0.0025	-0.1133
HIP_23	0.1833	0	4	18	-1.1833	4	3	0	0	0.000000	-2.5	0	0.1	1.750000	0.4444	1.3056	0	0	0	0	0	0.2394	-7.5	0	1	0	0	0.3154	1.0874	0.7368	0.1667	0.0995	0.5429	0.0578	0.01	-0.25
HIP_24	-0.4	0	4	18	-1.4556	4	3	0	0	0.000000	-1.3333	0	0.05	1.050000	0.3333	0.7167	0	0	0	0	0	-0.2867	-4	0	1	0	0	0.3821	1.0258	0.7968	0	0.146	0.6349	-0.1528	0.0025	-0.0667
HIP_25	-1.4833	0	2	18	-0.85	2	2	0	1	0.000000	-2.3	0	0.05	0.625000	0	0.625	0	0	0	0	0	-0.9271	-4.6	0	1	0	0	0.4769	1.1122	0.8756	0.3333	0.2275	0.7667	-0.7074	0.0025	-0.115
HIP_26	-2.6333	0.3333	4	18	-1.5944	3	2	0	0	0.000000	-1.1333	-1	0	0.700000	0.4444	0.2556	2	0	1	0	1.1333	-0.673	-2.2667	0	0	1	0	0.5769	0.9205	1.0923	-0.5	0.3328	1.1931	-1.5192	0	0
HIP_27	0.2833	0	2	18	0.5167	1	1	0	0	0.000000	-3.6333	2	0.1	0.000000	0.3333	-0.3333	0	0	0	0	-7.2667	-0.0944	-3.6333	1	0	0	0	0.3154	1.1608	0.7035	0.1667	0.0995	0.4949	0.0894	0.01	-0.3633
HIP_28	0.3833	0	3	18	0.0222	1	3	0	0	1.000000	-3.4333	1	0.05	0.450000	0.2222	0.2278	0	0	0	0	-3.4333	0.0873	-10.3	1	0	0	0	0.3154	1.1858	0.7301	0.3333	0.0995	0.5331	0.1209	0.0025	-0.1717
HIP_29	-0.7333	0	4	18	-1.2167	4	3	0	0	0.000000	-4.3333	1	0.15	0.700000	0.3333	0.3667	0	0	0	0	-4.3333	-0.2689	-13	0	1	0	0	0.4103	1.0638	0.8256	0.1667	0.1683	0.6817	-0.3009	0.0225	-0.65
HIP_30	-0.6167	0.1667	2	18	-0.7278	1	1	0	0	0.000000	-2.9	2	0.0833	0.000000	0.3333	-0.3333	0	1	0	1	-5.8	0.2056	-2.9	1	0	0	0	0.4269	1.0938	0.869	0	0.1823	0.7551	-0.2633	0.0069	-0.2417
HIP_31	-0.2667	0	3	18	-1.0278	3	3	0	0	0.000000	-1.0667	-1	0.05	0.000000	0.2222	-0.2222	0	0	0	0	1.0667	0.0593	-3.2	0	1	0	0	0.4385	1.1403	0.8045	0.1667	0.1922	0.6472	-0.1169	0.0025	-0.0533
HIP_32	1.15	0	3	18	-0.5889	2	0	0	0	0.000000	-3.9667	0	0.05	0.450000	0.1111	0.3389	0	0	0	0	0	0.3897	0	1	0	0	0	0.3051	1.2069	0.6745	0.5	0.0931	0.4549	0.3509	0.0025	-0.1983

HIP_S01	CGERGFFYT	WPERKLKHM	CGERGFFYTWPERKLKHM		SCG2	1660/334890	1.000	S01
HIP_S02	TSICSLYQL	EKAEKGREH	TSICSLYQLEKAEKGREH		SCG2	13848/334890	0.998	S02
HIP_S03	VCGERGFFY	QNRMLSKSG	VCGERGFFYQNRMLSKSG		SCG2	3811/334890	1.000	S03
HIP_S04	SHLVEALYL	LRQQAHKEE	SHLVEALYLLRQQAHKEE		SCG2	3364/334890	1.000	S04
HIP_S05	VCGERGFFY	NSRDNPFKR	VCGERGFFYNSRDNPFKR		SCG2	1995/334890	1.000	S05
HIP_S06	AGSLQPLAL	RQYWDEDLL	AGSLQPLALRQYWDEDLL		SCG2	2161/334890	1.000	S06
HIP_S07	VCGERGFFY	RLQNGQNGE	VCGERGFFYRLQNGQNGE		SCG2	2714/334890	1.000	S07
HIP_S08	CGERGFFYT	PERKLKHMQ	CGERGFFYTPERKLKHMQ		SCG2	4540/334890	1.000	S08
HIP_S09	VCGERGFFY	NRMLSKSGY	VCGERGFFYNRMLSKSGY		SCG2	4220/334890	1.000	S09
HIP_S10	TSICSLYQL	NNQKRERMD	TSICSLYQLNNQKRERMD		SCG2	4589/334890	1.000	S10
HIP_S11	ERGFFYTPK	RDSSMKLSF	ERGFFYTPKRDSSMKLSF		CMGA	5805/247050	0.998	S11
HIP_S12	SHLVEALYL	KRSSPETLI	SHLVEALYLKRSSPETLI		NPY	563/49410	0.997	S12
HIP_S13	AGSLQPLAL	YPKTPGRAG	AGSLQPLALYPKTPGRAG		SCG2	4414/334890	1.000	S13
HIP_S14	GSLQPLAL	MPVDNRNH	GSLQPLALMPVDNRNH	ELISPOT positive	SCG1	4097/367830	0.999	S14
HIP_S15	CGERGFFYT	AEAKTHWLG	CGERGFFYTAEAKTHWLG		SCG2	1433/334890	1.000	S15
HIP_S16	CGSHLVEAL	WPERKLKHM	CGSHLVEALWPERKLKHM		SCG2	4648/334890	1.000	S16
HIP_S17	CTSICSLYQ	RESTENVPR	CTSICSLYQRESTENVPR		NPY	545/49410	0.997	S17
HIP_S18	SHLVEALYL	YLARMLVKY	SHLVEALYLYLARMLVKY		SCG2	844/334890	1.000	S18
HIP_S19	SICSLYQLE	TQQWPERKL	SICSLYQLETQQWPERKL		SCG2	5881/334890	0.999	S19
HIP_S20	VCGERGFFY	EVRDSKENI	VCGERGFFYEVRDSKENI		SCG2	730/334890	1.000	S20
HIP_S21	VCGERGFFY	RILSILRHQ	VCGERGFFYRILSILRHQ		CMGA	1835/247050	0.999	S21
HIP_S22	VCGERGFFY	RYGKRSSPE	VCGERGFFYRYGKRSSPE		NPY	179/49410	0.999	S22
HIP_S23	VCGERGFFY	HNEGMVTRC	VCGERGFFYHNEGMVTRC		SCG1	575/367830	1.000	S23
HIP_S24	SHLVEALYL	RQAENEPQS	SHLVEALYLRQAENEPQS		SCG2	3572/334890	1.000	S24
HIP_S25	QHLCGSHLV	NYDWWEKKP	QHLCGSHLVNYDWWEKKP		SCG1	1194/367830	1.000	S25
HIP_S26	GSHLVEALY	QWKSSHFER	GSHLVEALYQWKSSHFER		SCG1	4693/367830	0.999	S26
HIP_S27	AGSLQPLAL	EKAEKGREH	AGSLQPLALEKAEKGREH		SCG2	4518/334890	1.000	S27
HIP_S28	HLVEALYLV	RYYSALRHY	HLVEALYLVRYYSALRHY		NPY	977/49410	0.995	S28
HIP_S29	AGSLQPLAL	QKFPSPEMI	AGSLQPLALQKFPSPEMI		SCG2	3624/334890	1.000	S29
HIP_S30	SHLVEALYL	EAKTHWLGA	SHLVEALYLEAKTHWLGA		SCG2	4455/334890	1.000	S30
HIP_S31	LVCGERGFF	PERKLKHMQ	LVCGERGFFPERKLKHMQ		SCG2	2067/334890	1.000	S31
HIP_S32	GSHLVEALY	KEWEDSKRW	GSHLVEALYKEWEDSKRW		CMGA	1258/247050	1.000	S32
HIP_S33	GGPGAGSLQ	WERYRGRGS	GGPGAGSLQWERYRGRGS		SCG1	10490/367830	0.997	S33
HIP_S34	VCGERGFFY	DKQYSSHHT	VCGERGFFYDKQYSSHHT		SCG1	2147/367830	1.000	S34
HIP_S35	CGERGFFYT	RQQAHKEES	CGERGFFYTRQQAHKEES		SCG2	5984/334890	0.999	S35
HIP_S36	HLVEALYL	EELVARSE	HLVEALYLEELVARSE	Real HIP	SCG1	4646/367830	0.999	S36
HIP_S37	CTSICSLYQ	ENYQKGERG	CTSICSLYQENYQKGERG		SCG1	3972/367830	0.999	S37
HIP_S38	ERGFFYTPK	WPERKLKHM	ERGFFYTPKWPERKLKHM		SCG2	2659/334890	1.000	S38
HIP_S39	VCGERGFFY	HYRKKSAEF	VCGERGFFYHYRKKSAEF		SCG1	10859/367830	0.997	S39
HIP_S40	VCGERGFFY	NERKQASAI	VCGERGFFYNERKQASAI		SCG1	2569/367830	1.000	S40
HIP_S41	VCGERGFFY	NNQKRERM	VCGERGFFYNNQKRERM		SCG2	1365/334890	1.000	S41
HIP_S42	AGSLQPLAL	KEWEDSKRW	AGSLQPLALKEWEDSKRW		CMGA	3186/247050	0.999	S42
HIP_S43	VCGERGFFY	RMDEEQKLY	VCGERGFFYRMDEEQKLY		SCG2	1463/334890	1.000	S43
[illegible]	[illegible]	[illegible]	[illegible]	[illegible]	[illegible]	[illegible]	[illegible]	[illegible]

HIP_1012	CGERGFFYT	EKTHSREKS	CGERGFFYTEKTHSREKS		SCG1	9057/367830	0.998	1012	0.876	0.158277	-1.2167	0	2	18	-1.5278	1	3	0	0
HIP_1013	AGSLQPLAL	EVDKRRTRP	AGSLQPLALEVDKRRTRP		SCG1	555/367830	1.000	1013	0.876	0.150970	1.1	0	2	18	-0.7667	2	0	0	0
HIP_1014	VCGERGFFY	FEKPLDSQS	VCGERGFFYFEKPLDSQS		SCG2	1036/334890	1.000	1014	0.876	0.115368	-0.05	0	-1	18	-0.4889	-1	4	0	0
HIP_1015	LVCGERGFF	NLRQQAHKE	LVCGERGFFNLRQQAHKE		SCG2	4469/334890	1.000	1015	0.876	0.074880	0.1667	0.1667	2	18	-0.7056	1	2	0	0
HIP_1016	VCGERGFFY	ENQMDKARR	VCGERGFFYENQMDKARR		SCG1	40/367830	1.000	1016	0.876	0.184105	-1.0333	0	1	18	-1.1667	1	3	0	0
HIP_1017	VCGERGFFY	EEVKTRHS	VCGERGFFYEEVKTRHS		SCG1	1940/367830	1.000	1017	0.876	0.138834	0.25	0	1	17	-0.8059	0	3	1	0
HIP_1018	VCGERGFFY	KADTEKWAE	VCGERGFFYKADTEKWAE		SCG1	2891/367830	1.000	1018	0.876	0.136316	-0.2167	0	-1	18	-0.7833	-1	3	0	0
HIP_1019	LVCGERGFF	DKLAPVSKR	LVCGERGFFDKLAPVSKR		SCG2	4590/334890	1.000	1019	0.876	0.075390	0.2667	0.1667	2	18	-0.0611	2	2	0	0
HIP_1020	VCGERGFFY	QKFPSPEMI	VCGERGFFYQKFPSPEMI		SCG2	3418/334890	1.000	1020	0.876	0.134886	-0.05	0	0	18	-0.1944	0	4	0	0
HIP_1021	CGSHLVEAL	TQQWPERKL	CGSHLVEALTQQWPERKL		SCG2	7366/334890	0.999	1021	0.876	0.160262	-0.9333	0	1	18	-0.5611	0	1	0	0
HIP_1022	TSICSLYQL	ENLRQQAHK	TSICSLYQLENLRQQAHK		SCG2	11272/334890	0.999	1022	0.876	0.110720	-0.7	0	2	18	-0.6944	1	1	0	0
HIP_1023	VCGERGFFY	NYQKGERGE	VCGERGFFYNYQKGERGE		SCG1	2251/367830	1.000	1023	0.875	0.136668	-0.6667	0	0	18	-1.2389	0	4	0	0
HIP_1024	SHLVEALYL	SHFERRDNM	SHLVEALYLSHFERRDNM		SCG1	2522/367830	1.000	1024	0.875	0.130660	0.85	0.1667	1	18	-0.5667	-1	2	0	0
HIP_1025	VCGERGFFY	YLKRLVNAA	VCGERGFFYYLKRLVNAA		SCG2	6924/334890	0.999	1025	0.875	0.101452	0.4833	0	2	18	0.2444	2	4	0	0
HIP_1026	LVEALYLVC	QQWPERKLK	LVEALYLVCQQWPERKLK		SCG2	1515/334890	1.000	1026	0.875	0.148440	0.4333	0	1	18	-0.1222	1	2	0	0
HIP_1027	ERGFFYTPK	EKPNGSVEP	ERGFFYTPKEKPNGSVEP		SCG2	3353/334890	1.000	1027	0.875	-0.058910	-2.5333	0.3333	0	18	-1.3833	0	2	0	0
HIP_1028	SHLVEALYL	EKVLPRLPY	SHLVEALYLEKVLPRLPY		SCG2	4803/334890	1.000	1028	0.875	0.202162	0.5167	0	1	18	0.2222	0	1	0	0
HIP_1029	ERGFFYTPK	REDSKEAEK	ERGFFYTPKREDSKEAEK		CMGA	5474/247050	0.998	1029	0.875	-0.018524	-2.95	0.1667	0	18	-1.9778	0	2	0	0
HIP_1030	SHLVEALYL	LQWKSSHF	SHLVEALYLLQWKSSHF		SCG1	1971/367830	1.000	1030	0.875	0.102080	0.95	0	2	17	0.1235	0	2	1	0
HIP_1031	VCGERGFFY	KVLPRLPYG	VCGERGFFYKVLPRLPYG		SCG2	3009/334890	1.000	1031	0.875	0.060816	1.4	0	2	18	0.0389	2	3	0	0
HIP_1032	LVCGERGFF	RLENVQKFP	LVCGERGFFRLENVQKFP		SCG2	3873/334890	1.000	1032	0.875	0.073904	0.1667	0.1667	1	18	-0.1333	1	2	0	0
HIP_1033	VCGERGFFY	QVLKTSRKD	VCGERGFFYQVLKTSRKD		SCG1	1593/367830	1.000	1033	0.875	0.050400	1.4667	0	2	18	-0.5889	2	3	0	0
HIP_1034	SHLVEALYL	WEKKPFSED	SHLVEALYLWEKKPFSED		SCG1	3658/367830	0.999	1034	0.875	0.160763	-0.3333	0	-1	18	-0.5667	-2	2	0	0
HIP_1035	GSHLVEALY	YNQEKVLPR	GSHLVEALYYNQEKVLPR		SCG2	1849/334890	1.000	1035	0.875	0.128925	-0.6667	0	1	18	-0.5222	0	2	0	0
HIP_1036	AGSLQPLAL	NHLKATPIE	AGSLQPLALNHLKATPIE		IAPP	390/45018	0.999	1036	0.875	0.120363	1.0833	0	1	18	0.1333	0	0	0	0
HIP_1037	HLVEALYL	QQWPERKLK	HLVEALYLQQWPERKLK		SCG2	4153/334890	1.000	1037	0.875	0.158199	-0.2667	0	2	17	-0.7118	1	2	1	0
HIP_1038	SHLVEALY	EWERYRGRG	SHLVEALYEWERYRGRG		SCG1	2171/367830	1.000	1038	0.875	0.136989	-0.6	0	1	17	-1.1	0	3	1	0
HIP_1039	CGERGFFYT	NLRQQAHKE	CGERGFFYTNLRQQAHKE		SCG2	9302/334890	0.999	1039	0.875	0.104755	-0.5667	0	2	18	-1.2611	1	3	0	0
HIP_1040	SHLVEALYL	QYTPQSLAT	SHLVEALYLQYTPQSLAT		SCG2	1407/334890	1.000	1040	0.875	0.168708	0.1333	0	0	18	0.1167	-1	2	0	0
HIP_1041	CGERGFFYT	EEVKTRHSE	CGERGFFYTEEVKTRHSE		SCG1	4101/367830	0.999	1041	0.875	0.062791	-0.3333	0	0	18	-1.2278	-1	3	0	0
HIP_1042	VCGERGFFY	YGKRSSPET	VCGERGFFYYGKRSSPET		NPY	177/49410	0.999	1042	0.875	0.171134	-0.2167	0.1667	1	18	-0.85	1	4	0	0
HIP_1043	VCGERGFFY	YDRVAQLDQ	VCGERGFFYYDRVAQLDQ		SCG1	2269/367830	1.000	1043	0.875	0.117468	-0.8333	0	-1	18	-0.4333	-1	4	0	0
HIP_1044	GIVEQCCTS	TQQWPERKL	GIVEQCCTSTQQWPERKL		SCG2	4857/334890	1.000	1044	0.875	0.041789	-1.1167	0.1667	0	18	-0.75	0	1	0	0
HIP_1045	CGERGFFY	EWERYRGRG	CGERGFFYEWERYRGRG		SCG1	1955/367830	1.000	1045	0.875	0.130898	-0.6	0	1	17	-1.5	1	5	1	0
HIP_1046	TSICSLYQL	KLAPVSKRF	TSICSLYQLKLAPVSKRF		SCG2	8747/334890	0.999	1046	0.875	0.054908	0.1167	0	3	18	0.3	3	1	0	0
HIP_1047	AGSLQPLAL	FRARAYGFR	AGSLQPLALFRARAYGFR		CMGA	6372/247050	0.998	1047	0.875	0.125496	1.5833	0	3	18	0.15	3	1	0	0
HIP_1048	CGERGFFYT	ATQRLANFL	CGERGFFYTATQRLANFL		IAPP	491/45018	0.998	1048	0.875	0.072510	-0.2667	0	1	18	-0.05	1	3	0	0
HIP_1049	QCCTSICSL	QWPERKLKH	QCCTSICSLQWPERKLKH		SCG2	3970/334890	1.000	1049	0.875	0.123087	-0.0833	0.3333	3	18	-0.6222	2	1	0	0
HIP_1050	GERGFFYTP	EEEGSANRR	GERGFFYTPEEEGSANRR		CMGA	258/247050	1.000	1050	0.875	0.001776	-2.35	0.1667	-1	18	-1.6222	-1	3	0	1
HIP_1051	VCGERGFFY	RGRGSEEYR	VCGERGFFYRGRGSEEYR		SCG1	2750/367830	1.000	1051	0.875	0.213883	-0.85	0.1667	1	18	-1.1778	1	3	0	1
HIP_1052	CGERGFFYT	EKDRADQTV	CGERGFFYTEKDRADQTV		SCG1	4234/367830	0.999	1052	0.875	0.137174	-1.6833	0	-1	18	-1.1	-1	3	0	0
HIP_1053	CGERGFFYT	WEKKPFSED	CGERGFFYTWEKKPFSED		SCG1	5449/367830	0.999	1053	0.875	0.081554	-1.25	0	-1	18	-1.1944	-1	4	0	0
HIP_1054	AGSLQPLAL	ENLRQQAHK	AGSLQPLALENLRQQAHK		SCG2	432/334890	1.000	1054	0.875	0.185243	1.0333	0	2	18	-0.6278	1	0	0	0

HIP_1523	CSLYQLENY	EEEATNTHP	CSLYQLENYEEEATNTHP		CMGA	3328/247050	0.999	1523	0.872	-0.045412	-3.1333	0	-3	18	-1.2333	-4	1	0	1
HIP_1524	SHLVEALYL	KQYSSHHTA	SHLVEALYLKQYSSHHTA		SCG1	11031/367830	0.997	1524	0.872	0.179285	-0.4	0	3	18	-0.3889	0	2	0	0
HIP_1525	CGERGFFY	DQLLHYRKK	CGERGFFYDQLLHYRKK		SCG1	7586/367830	0.999	1525	0.872	0.171005	0.1833	0	3	17	-1.0706	2	3	1	0
HIP_1526	LVCGERGFF	AEKGREHIA	LVCGERGFFAEKGREHIA		SCG2	3542/334890	1.000	1526	0.872	0.058696	-0.0667	0.1667	1	18	-0.2	0	2	0	0
HIP_1527	AGSLQPLAL	NRMLSKSGY	AGSLQPLALNRMLSKSGY		SCG2	8187/334890	0.999	1527	0.872	0.188254	0.55	0	2	18	-0.0444	2	0	0	0
HIP_1528	CGERGFFYT	EAENEKDRA	CGERGFFYTEAENEKDRA		SCG1	1616/367830	1.000	1528	0.872	0.049943	-0.7333	0	-2	18	-1.3889	-2	3	0	0
HIP_1529	CGERGFFYT	LNQEKAEKG	CGERGFFYTLNQEKAEKG		SCG2	1926/334890	1.000	1529	0.872	0.041592	-0.4	0	0	18	-1.0722	0	3	0	0
HIP_1530	CGERGFFYT	TKADTEKWA	CGERGFFYTTKADTEKWA		SCG1	8412/367830	0.998	1530	0.872	0.085796	-0.3333	0	0	18	-0.9	0	3	0	0
HIP_1531	SHLVEALYL	LQKEPDLRL	SHLVEALYLLQKEPDLRL		SCG2	4618/334890	1.000	1531	0.872	0.183388	0.45	0	0	18	-0.0278	-1	1	0	0
HIP_1532	TSICSLYQL	QKENGDESH	TSICSLYQLQKENGDESH		SCG2	7266/334890	0.999	1532	0.872	0.185196	-1.9833	0	-1	18	-1.0167	-2	1	0	0
HIP_1533	CTSICSLYQ	GYEKRNLAR	CTSICSLYQGYEKRNLAR		SCG1	3452/367830	1.000	1533	0.872	0.117380	-1.0333	0.1667	2	18	-0.5444	2	2	0	0
HIP_1534	SICSLYQLE	RFQDKQYSS	SICSLYQLERFQDKQYSS		SCG1	12674/367830	0.996	1534	0.872	-0.060447	-1.4	0	0	18	-0.7944	0	2	0	0
HIP_1535	SHLVEALYL	DMARYYSAL	SHLVEALYLDMARYYSAL		NPY	108/49410	1.000	1535	0.872	0.090573	1.0833	0	0	18	0.3611	-1	1	0	0
HIP_1536	VCGERGFFY	KRLVNAAGS	VCGERGFFYKRLVNAAGS		SCG2	9266/334890	0.999	1536	0.872	0.141700	-0.05	0	2	18	0.0389	2	3	0	0
HIP_1537	SLYQLENYC	ENYQKGERG	SLYQLENYCENYQKGERG		SCG1	4365/367830	0.999	1537	0.872	0.014881	-1.7667	0	-1	18	-1.5722	-1	2	0	0
HIP_1538	CTSICSLYQ	KNFFPEYNY	CTSICSLYQKNFFPEYNY		SCG1	5667/367830	0.999	1538	0.872	0.026014	-0.9333	0	0	18	-0.3778	0	3	0	0
HIP_1539	VCGERGFFY	VSKRFPVGP	VCGERGFFYVSKRFPVGP		SCG2	2578/334890	1.000	1539	0.872	0.164435	0.6333	0.1667	2	18	0.0333	2	3	0	0
HIP_1540	CGERGFFYT	KNDDTPNRQ	CGERGFFYTKNDDTPNRQ		SCG2	7060/334890	0.999	1540	0.872	0.150442	-1.6833	0	0	18	-1.7167	0	3	0	0
HIP_1541	VCGERGFFY	LFQNRMLSK	VCGERGFFYLFQNRMLSK		SCG2	494/334890	1.000	1541	0.872	0.042556	1.2333	0	2	18	-0.0944	2	4	0	0
HIP_1542	GSLQPLAL	QKTSYFPNP	GSLQPLALQKTSYFPNP		SCG2	5890/334890	0.999	1542	0.872	0.239568	0.2167	0	1	17	-0.4235	1	1	1	0
HIP_1543	VCGERGFFY	HESSSRGEA	VCGERGFFYHESSSRGEA		SCG1	450/367830	1.000	1543	0.872	0.146416	-0.5333	0.1667	0	18	-0.75	-1	3	0	1
HIP_1544	SLYQLENYC	KWQQQGDLQ	SLYQLENYCKWQQQGDLQ		SCG1	7624/367830	0.999	1544	0.872	0.033081	-1.7667	0	-1	18	-1.2611	-1	2	0	1
HIP_1545	VCGERGFFY	HSEKSQRE	VCGERGFFYHSEKSQRE		SCG1	3631/367830	0.999	1545	0.872	0.159391	-0.5333	0.1667	1	17	-1.2647	0	3	1	0
HIP_1546	VCGERGFFY	YDWWEKKPF	VCGERGFFYYDWWEKKPF		SCG1	2168/367830	1.000	1546	0.872	0.068986	-0.2333	0	0	18	-0.8056	0	6	0	0
HIP_1547	GSHLVEALY	ENYQKGERG	GSHLVEALYENYQKGERG		SCG1	101/367830	1.000	1547	0.872	0.108681	-0.6667	0	0	18	-1.1167	-1	2	0	0
HIP_1548	VCGERGFFY	VRDSKENIE	VCGERGFFYVRDSKENIE		SCG2	1491/334890	1.000	1548	0.872	0.118194	0.0833	0	-1	18	-0.6833	-1	3	0	0
HIP_1549	AGSLQPLAL	NQKRERMDE	AGSLQPLALNQKRERMDE		SCG2	4580/334890	1.000	1549	0.872	0.228898	-0.25	0	0	18	-1.1	0	0	0	0
HIP_1550	TSICSLYQL	SNQVKRVPG	TSICSLYQLSNQVKRVPG		SCG2	3199/334890	1.000	1550	0.872	0.176050	-1.4667	0.1667	2	18	-0.1278	2	1	0	0
HIP_1551	TSICSLYQL	ERYRGRGSE	TSICSLYQLERYRGRGSE		SCG1	16352/367830	0.994	1551	0.872	0.129084	-1.7167	0	1	18	-0.8833	1	2	0	0
HIP_1552	LYQLENYCN	FQDKQYSSH	LYQLENYCNFQDKQYSSH		SCG1	3940/367830	0.999	1552	0.872	-0.032064	-1.0833	0	0	18	-1.3444	-1	2	0	0
HIP_1553	CTSICSLYQ	RQQAHKEES	CTSICSLYQRQQAHKEES		SCG2	11426/334890	0.999	1553	0.872	0.147209	-2.0833	0	1	18	-1.0222	0	1	0	0
HIP_1554	GERGFFYTP	EEKKEEEGS	GERGFFYTPEEKKEEEGS		CMGA	1069/247050	1.000	1554	0.872	-0.004373	-2.4167	0.1667	-3	18	-1.85	-3	3	0	1
HIP_1555	SHLVEALYL	PRLPYGAGR	SHLVEALYLPRLPYGAGR		SCG2	4445/334890	1.000	1555	0.872	0.226647	0.6667	0.1667	2	18	-0.0056	1	1	0	0
HIP_1556	TSICSLYQL	SRDNPFKRT	TSICSLYQLSRDNPFKRT		SCG2	11950/334890	0.998	1556	0.872	0.253054	-1.6333	0.1667	2	18	-0.7056	2	1	0	0
HIP_1557	VCGERGFFY	PGKGRHHRG	VCGERGFFYPGKGRHHRG		SCG1	1003/367830	1.000	1557	0.872	0.219268	-0.2667	0.3333	5	18	-1.1056	3	3	0	0
HIP_1558	ERGFFYTPK	SEEVKTRHS	ERGFFYTPKSEEVKTRHS		SCG1	7405/367830	0.999	1558	0.872	-0.073216	-2.3333	0.3333	2	18	-1.5	1	2	0	0
HIP_1559	ERGFFYTPK	ESRSEALAV	ERGFFYTPKESRSEALAV		CMGA	4518/247050	0.999	1559	0.872	-0.055522	-2.5	0.3333	0	18	-0.6556	0	2	0	0
HIP_1560	GSHLVEALY	WKELDRNYL	GSHLVEALYWKELDRNYL		SCG1	2696/367830	1.000	1560	0.872	0.099951	-0.6667	0	0	18	-0.5056	-1	2	0	0
HIP_1561	VCGERGFFY	THQEAENEK	VCGERGFFYTHQEAENEK		SCG1	2378/367830	1.000	1561	0.872	0.127329	-0.5167	0	-1	18	-1.1833	-2	3	0	0
HIP_1562	SHLVEALYL	NNQKRERM	SHLVEALYLNNQKRERM		SCG2	4087/334890	1.000	1562	0.872	0.260028	-0.7	0	2	17	-0.9647	1	1	1	0
HIP_1563	VCGERGFFY	VKDKETTEN	VCGERGFFYVKDKETTEN		SCG1	1816/367830	1.000	1563	0.872	0.119188	0.1833	0	-1	18	-0.9333	-1	3	0	0
HIP_1564	LVEALYLVC	TAEKRKRLG	LVEALYLVCTAEKRKRLG		SCG1	1080/367830	1.000	1564	0.872	0.161356	1.35	0	2	18	0.1944	2	1	0	0
HIP_1565	SHLVEALYL	AHQQKKHSG	SHLVEALYLAHQQKKHSG		CMGA	8988/247050	0.996	1565	0.872	0.144479	0.2333	0	4	18	-0.6667	1	1	0	0
HIP_1566	VCGERGFFY	RGLEPGKGR	VCGERGFFYRGLEPGKGR		SCG1	1139/367830	1.000	1566	0.872	0.195345	0.5333	0.1667	2	18	-0.7333	2	3	0	0
HIP_1567	LVCGERGFF	FQNRMLSKS	LVCGERGFFFQNRMLSKS		SCG2	1154/334890	1.000	1567	0.872	0.059544	0.1667	0.1667	2	18	-0.0667	2	3	0	0
HIP_1568	SLYQLENYC	KQYSSHHTA	SLYQLENYCKQYSSHHTA		SCG1	15040/367830	0.995	1568	0.872	0.066885	-1.8333	0	2	18	-1.0778	0	2	0	0
HIP_1569	SHLVEALYL	PFKRTNEIV	SHLVEALYLPFKRTNEIV		SCG2	2799/334890	1.000	1569	0.872	0.196479	0.6	0.1667	1	18	0.1333	0	2	0	0
HIP_1570	AGSLQPLAL	TKFEVRLLR	AGSLQPLALTKFEVRLLR		SCG1	9939/367830	0.998	1570	0.872	0.208839	1.2667	0	2	18	0.3444	2	1	0	0
HIP_1571	AGSLQPLAL	RKESKDQLS	AGSLQPLALRKESKDQLS		SCG2	8715/334890	0.999	1571	0.872	0.236798	-0.4167	0	1	18	-0.6611	1	0	0	0
HIP_1572	SLYQLENYC	WERYRGRGS	SLYQLENYCWERYRGRGS		SCG1	12177/367830	0.997	1572	0.872	0.071858	-1.8667	0	1	18	-1.3667	1	3	0	0
HIP_1573	SHLVEALYL	LQWKSSHFE	SHLVEALYLLQWKSSHFE		SCG1	223/367830	1.000	1573	0.872	0.082461	0.95	0	1	18	-0.0778	-1	2	0	0
HIP_1574	RGFFYTPKT	EKPNGSVEP	RGFFYTPKTEKPNGSVEP		SCG2	7144/334890	0.999	1574	0.872	-0.017849	-2.5333	0.3333	1	18	-1.2278	1	1	0	0
HIP_1575	SHLVEALYL	IKEHLNQGS	SHLVEALYLIKEHLNQGS		SCG2	3858/334890	1.000	1575	0.872	0.186361	0.5667	0	1	18	-0.1056	-1	1	0	0

HIP_2034	CTSICSLYQ	EQYTPQSLA	CTSICSLYQEQYTPQSLA		SCG2	4864/334890	1.000	2034	0.870	0.045874	-1.55	0	-1	18	-0.1722	-1	2	0	0
HIP_2035	SHLVEALYL	ERKLKHMQF	SHLVEALYLERKLKHMQF		SCG2	11187/334890	0.999	2035	0.870	0.240137	-0.9333	0	3	18	-0.3	1	1	0	0
HIP_2036	GSHLVEALY	RMLSKSGYP	GSHLVEALYRMLSKSGYP		SCG2	5364/334890	0.999	2036	0.870	0.051295	0.9167	0	2	18	-0.1778	1	1	0	0
HIP_2037	CGERGFFYT	STHYRASEE	CGERGFFYTSTHYRASEE		SCG1	3376/367830	1.000	2037	0.870	0.077265	-0.65	0.1667	0	18	-1.0667	-1	4	0	0
HIP_2038	VCGERGFFY	ELEKVAHQL	VCGERGFFYELEKVAHQL		CMGA	823/247050	1.000	2038	0.870	0.123139	0.1833	0	0	18	-0.1	-1	3	0	0
HIP_2039	CGERGFFYT	RAYFMSDTR	CGERGFFYTRAYFMSDTR		SCG1	8810/367830	0.998	2039	0.870	0.048949	-0.5333	0	1	18	-0.6389	1	5	0	0
HIP_2040	SHLVEALYL	DVNWGYEKR	SHLVEALYLDVNWGYEKR		SCG1	787/367830	1.000	2040	0.870	0.166634	0.5833	0	0	18	-0.4833	-1	2	0	0
HIP_2041	VCGERGFFY	HNEGMVTR	VCGERGFFYHNEGMVTR		SCG1	5082/367830	0.999	2041	0.870	0.144010	-0.9833	0	1	17	-0.4412	0	3	1	0
HIP_2042	AGSLQPLAL	WGYEKRNLA	AGSLQPLALWGYEKRNLA		SCG1	213/367830	1.000	2042	0.870	0.140580	1.1333	0.1667	1	18	-0.2056	1	2	0	0
HIP_2043	CTSICSLYQ	YYDPLQWKS	CTSICSLYQYYDPLQWKS		SCG1	7701/367830	0.998	2043	0.870	0.022455	-1.1833	0	0	18	-0.3778	0	3	0	0
HIP_2044	HLCGSHLVE	YNYDWWEKK	HLCGSHLVEYNYDWWEKK		SCG1	11121/367830	0.997	2044	0.870	0.048784	-0.2667	0	1	18	-1.0833	-1	2	0	0
HIP_2045	AGSLQPLAL	HWLGAALSL	AGSLQPLALHWLGAALSL		SCG2	3025/334890	1.000	2045	0.870	0.061970	1.5167	0	1	18	1.0222	0	1	0	0
HIP_2046	HLVEALYLV	QEKAEKGRE	HLVEALYLVQEKAEKGRE		SCG2	2726/334890	1.000	2046	0.870	0.208951	-0.7	0	0	18	-0.6278	-1	1	0	0
HIP_2047	LVCGERGFF	LKTSRKDVK	LVCGERGFFLKTSRKDVK		SCG1	6543/367830	0.999	2047	0.870	0.060895	0.7333	0.1667	3	18	-0.3278	3	2	0	0
HIP_2048	TSICSLYQL	YEKRNLARV	TSICSLYQLYEKRNLARV		SCG1	18949/367830	0.991	2048	0.870	0.139724	-1.6167	0	2	18	-0.2167	2	2	0	0
HIP_2049	GERGFFYTP	QQKENGDES	GERGFFYTPQQKENGDES		SCG2	4700/334890	1.000	2049	0.870	0.003492	-2.4167	0.1667	-2	18	-1.8278	-2	3	0	0
HIP_2050	GSHLVEALY	FRARAYGFR	GSHLVEALYFRARAYGFR		CMGA	5107/247050	0.998	2050	0.870	0.058342	0.7333	0	3	18	-0.0889	2	2	0	0
HIP_2051	VCGERGFFY	ERILSILRH	VCGERGFFYERILSILRH		CMGA	4675/247050	0.999	2051	0.870	0.142018	0.1333	0	2	18	0.1278	1	3	0	0
HIP_2052	VCGERGFFY	NALSKSSAP	VCGERGFFYNALSKSSAP		SCG1	1180/367830	1.000	2052	0.870	0.064579	1.0667	0	1	18	-0.1	1	3	0	0
HIP_2053	CTSICSLYQ	QSAPKENKP	CTSICSLYQQSAPKENKP		SCG2	4783/334890	1.000	2053	0.870	0.064854	-0.5833	0.1667	1	18	-0.7944	1	1	0	0
HIP_2054	VCGERGFFY	DKARRHPQ	VCGERGFFYDKARRHPQ		SCG1	11609/367830	0.997	2054	0.870	0.166763	-0.2167	0	3	17	-1.2176	2	3	1	0
HIP_2055	CGERGFFYT	DKQYSSHHT	CGERGFFYTDKQYSSHHT		SCG1	9696/367830	0.998	2055	0.870	0.151381	-1.6833	0	2	18	-1.3111	0	4	0	0
HIP_2056	GERGFFYTP	ENSRDNPFK	GERGFFYTPENSRDNPFK		SCG2	764/334890	1.000	2056	0.870	-0.003136	-1.9	0.3333	0	18	-1.6	0	3	0	0
HIP_2057	CGERGFFYT	MQESKAEGN	CGERGFFYTMQESKAEGN		CMGA	2444/247050	0.999	2057	0.870	0.054065	-0.7167	0	-1	18	-1.0056	-1	3	0	0
HIP_2058	ERGFFYTPK	EEKKEEEGS	ERGFFYTPKEEKKEEEGS		CMGA	3643/247050	0.999	2058	0.870	-0.046847	-2.85	0.1667	-2	18	-2.0444	-2	2	0	1
HIP_2059	LVCGERGFF	RNAVEVLKR	LVCGERGFFRNAVEVLKR		IAPP	885/45018	0.997	2059	0.870	0.061645	-0.1667	0.1667	2	18	0.0778	2	2	0	0
HIP_2060	LVEALYLVC	KRQYDRVAQ	LVEALYLVCKRQYDRVAQ		SCG1	9562/367830	0.998	2060	0.870	0.185267	-0.2333	0	1	18	0.0333	1	2	0	0
HIP_2061	VCGERGFFY	RALEYIENL	VCGERGFFYRALEYIENL		SCG2	1840/334890	1.000	2061	0.870	0.055868	0.9	0	-1	18	-0.0111	-1	3	0	0
HIP_2062	LCGSHLVEA	QSAPKENKP	LCGSHLVEAQSAPKENKP		SCG2	9212/334890	0.999	2062	0.870	0.132177	0	0.1667	1	18	-0.6833	0	0	0	0
HIP_2063	TSICSLYQL	NYQKGERGE	TSICSLYQLNYQKGERGE		SCG1	8893/367830	0.998	2063	0.870	0.098416	-1.55	0	0	18	-0.9444	0	2	0	0
HIP_2064	CGERGFFYT	YEKRNLARV	CGERGFFYTYEKRNLARV		SCG1	12898/367830	0.996	2064	0.870	0.109626	-1.3167	0	2	18	-0.7833	2	4	0	0
HIP_2065	VCGERGFFY	PRTRLEDPA	VCGERGFFYPRTRLEDPA		NPY	213/49410	0.999	2065	0.870	0.200842	-0.4167	0.1667	0	18	-0.6722	0	3	0	0
HIP_2066	SHLVEALYL	RMDEEQKLY	SHLVEALYLRMDEEQKLY		SCG2	5526/334890	0.999	2066	0.870	0.200433	0.0333	0	-1	18	-0.5222	-2	1	0	0
HIP_2067	VCGERGFFY	EQINDEMKR	VCGERGFFYEQINDEMKR		SCG2	225/334890	1.000	2067	0.870	0.126311	0.3	0	-1	18	-0.9611	-1	3	0	0
HIP_2068	GSHLVEALY	EWERYRGR	GSHLVEALYEWERYRGR		SCG1	2252/367830	1.000	2068	0.870	0.108476	-0.6	0	1	17	-1.1	0	2	1	0
HIP_2069	GSHLVEALY	MRESTENVP	GSHLVEALYMRESTENVP		NPY	79/49410	1.000	2069	0.870	0.109585	-0.3	0	-1	18	-0.4222	-2	1	0	0
HIP_2070	VCGERGFFY	DTKENREEA	VCGERGFFYDTKENREEA		SCG1	2043/367830	1.000	2070	0.870	0.184971	-0.6333	0	-2	18	-1.2556	-2	3	0	0
HIP_2071	HLVEALYLV	HFERRDNM	HLVEALYLVHFERRDNM		SCG1	4284/367830	0.999	2071	0.870	0.086652	0.4667	0	1	17	-0.2588	-1	2	1	0
HIP_2072	ERGFFYTPK	TQEEVRDSK	ERGFFYTPKTQEEVRDSK		SCG2	6192/334890	0.999	2072	0.870	-0.066230	-2.3167	0.1667	0	18	-1.6667	0	2	0	0
HIP_2073	AGSLQPLAL	NNFGAILS	AGSLQPLALNNFGAILS		IAPP	44/45018	1.000	2073	0.870	0.197009	0.8667	0	0	17	0.7882	0	1	1	0
HIP_2074	VCGERGFFY	QEHSQQKEE	VCGERGFFYQEHSQQKEE		CMGA	1794/247050	1.000	2074	0.870	0.121688	-0.9833	0	-1	18	-1.4833	-2	3	0	0
HIP_2075	SHLVEALYL	DLRLENVQK	SHLVEALYLDLRLENVQK		SCG2	4916/334890	1.000	2075	0.870	0.194631	0.35	0	0	18	-0.1111	-1	1	0	0
HIP_2076	LYQLENYCN	TQQWPERKL	LYQLENYCNTQQWPERKL		SCG2	3911/334890	1.000	2076	0.870	0.120531	-1.6667	0	0	18	-1.3778	0	2	0	0

HIP_2545	LVCGERGFF	WEDSKRWSK	LVCGERGFFWEDSKRWSK	CMGA	1323/247050	1.000	2545	0.868	0.050262
HIP_2546	VCGERGFFY	QDTKENRE	VCGERGFFYQDTKENRE	SCG1	1368/367830	1.000	2546	0.868	0.183601
HIP_2547	VCGERGFFY	TNTHPPASL	VCGERGFFYTNTHPPASL	CMGA	825/247050	1.000	2547	0.868	0.192097
HIP_2548	LVCGERGFF	PKENKPYAL	LVCGERGFFPKENKPYAL	SCG2	3565/334890	1.000	2548	0.868	0.126567
HIP_2549	ERGFFYTPK	EDKRNYPSL	ERGFFYTPKEDKRNYPSL	SCG1	13454/367830	0.996	2549	0.868	-0.001815
HIP_2550	CTSICSLYQ	YINLITRQR	CTSICSLYQYINLITRQR	NPY	728/49410	0.996	2550	0.868	-0.041001
HIP_2551	ERGFFYTPK	SREDSLEAG	ERGFFYTPKSREDSLEAG	CMGA	4276/247050	0.999	2551	0.868	-0.068357
HIP_2552	CGERGFFYT	ENREEARFQ	CGERGFFYTENREEARFQ	SCG1	7997/367830	0.998	2552	0.868	0.142568
HIP_2553	CGERGFFYT	NREEARFQD	CGERGFFYTNREEARFQD	SCG1	7998/367830	0.998	2553	0.868	0.142568
HIP_2554	VCGERGFFY	ETHAAGHS	VCGERGFFYETHAAGHS	SCG1	4664/367830	0.999	2554	0.868	0.128477
HIP_2555	CTSICSLYQ	HFERRDNMN	CTSICSLYQHFERRDNMN	SCG1	14250/367830	0.995	2555	0.868	0.013017
HIP_2556	GERGFFYTP	ERATRLFEK	GERGFFYTPERATRLFEK	SCG2	9310/334890	0.999	2556	0.868	-0.014220
HIP_2557	CGERGFFYT	MLSKSGYPK	CGERGFFYTMLSKSGYPK	SCG2	1137/334890	1.000	2557	0.868	-0.029977
HIP_2558	AGSLQPLAL	FRGGKSGEL	AGSLQPLALFRGGKSGEL	CMGA	245/247050	1.000	2558	0.868	0.212405
HIP_2559	HLVEALYL	ATQRLANF	HLVEALYLATQRLANF	IAPP	419/45018	0.999	2559	0.868	0.207561
HIP_2560	CTSICSLYQ	EYNYDWWEK	CTSICSLYQEYNYDWWEK	SCG1	3208/367830	1.000	2560	0.868	0.034847
HIP_2561	CGERGFFYT	QAENEPQSA	CGERGFFYTQAENEPQSA	SCG2	2513/334890	1.000	2561	0.868	0.052854
HIP_2562	GERGFFYTP	EEKRFLGEG	GERGFFYTPEEKRFLGEG	SCG1	7241/367830	0.999	2562	0.868	0.018664
HIP_2563	ERGFFYTPK	QETDKLAPV	ERGFFYTPKQETDKLAPV	SCG2	6193/334890	0.999	2563	0.868	-0.067971
HIP_2564	HLCGSHLVE	DIYKANNIA	HLCGSHLVEDIYKANNIA	SCG2	8105/334890	0.999	2564	0.868	0.022252
HIP_2565	HLVEALYLV	EARFQDKQY	HLVEALYLVEARFQDKQY	SCG1	4778/367830	0.999	2565	0.868	0.134589
HIP_2566	AGSLQPLAL	LRKESKDQL	AGSLQPLALLRKESKDQL	SCG2	11444/334890	0.999	2566	0.868	0.155096
HIP_2567	GERGFFYTP	QEKVLPRLP	GERGFFYTPQEKVLPRLP	SCG2	8716/334890	0.999	2567	0.868	0.002822
HIP_2568	AGSLQPLAL	ERAHQQKKH	AGSLQPLALERAHQQKKH	CMGA	9832/247050	0.995	2568	0.868	0.217580
HIP_2569	LVCGERGFF	TENVPRTRL	LVCGERGFFTENVPRTRL	NPY	50/49410	1.000	2569	0.868	0.078738
HIP_2570	LVEALYLVC	RYRGRGSEE	LVEALYLVCRYRGRGSEE	SCG1	11925/367830	0.997	2570	0.868	0.134771
HIP_2571	VCGERGFFY	AVDGAGKPG	VCGERGFFYAVDGAGKPG	CMGA	152/247050	1.000	2571	0.868	0.040887
HIP_2572	VCGERGFFY	NRQYWDEDL	VCGERGFFYNRQYWDEDL	SCG2	3518/334890	1.000	2572	0.867	0.179912
HIP_2573	CGERGFFYT	NPYQGVSVP	CGERGFFYTNPYQGVSVP	SCG2	2011/334890	1.000	2573	0.867	0.075414
HIP_2574	CTSICSLYQ	QAHKEESSP	CTSICSLYQQAHKEESSP	SCG2	10440/334890	0.999	2574	0.867	-0.026944
HIP_2575	RGFFYTPKT	TGPNNQKRE	RGFFYTPKTTGPNNQKRE	SCG2	1163/334890	1.000	2575	0.867	-0.035972
HIP_2576	CGERGFFYT	AHQQKKHSG	CGERGFFYTAHQQKKHSG	CMGA	9785/247050	0.995	2576	0.867	0.068819
HIP_2577	CTSICSLYQ	DVNWGYEKR	CTSICSLYQDVNWGYEKR	SCG1	5388/367830	0.999	2577	0.867	0.029941
HIP_2578	VCGERGFFY	ENQMDKAR	VCGERGFFYENQMDKAR	SCG1	1895/367830	1.000	2578	0.867	0.181425
HIP_2579	CGERGFFYT	TRHSEKSQR	CGERGFFYTTRHSEKSQR	SCG1	16440/367830	0.994	2579	0.867	0.113777
HIP_2580	VCGERGFFY	YSSHNTAEK	VCGERGFFYYSSHNTAEK	SCG1	1230/367830	1.000	2580	0.867	0.161387
HIP_2581	SHLVEALYL	REEKRFLGE	SHLVEALYLREEKRFLGE	SCG1	6351/367830	0.999	2581	0.867	0.223761
HIP_2582	VEALYLVC	EWERYRGRG	VEALYLVCEWERYRGRG	SCG1	1216/367830	1.000	2582	0.867	0.124147
HIP_2583	HLVEALYLV	ARAYGFRGP	HLVEALYLVARAYGFRGP	CMGA	4981/247050	0.998	2583	0.867	0.090352
HIP_2584	VCGERGFFY	AELEKVAHQ	VCGERGFFYAELEKVAHQ	CMGA	198/247050	1.000	2584	0.867	0.035405
HIP_2585	LVCGERGFF	RSETHAAGH	LVCGERGFFRSETHAAGH	SCG1	5614/367830	0.999	2585	0.867	0.134525
HIP_2586	VCGERGFFY	QQAHKEES	VCGERGFFYQQAHKEES	SCG2	3933/334890	1.000	2586	0.867	0.134923
HIP_2587	LVCGERGFF	ARAYGFRGP	LVCGERGFFARAYGFRGP	CMGA	3160/247050	0.999	2587	0.867	0.058168
[illegible]									

HIP_3056	VCGERGFFY	EKNEQINDE	VCGERGFFYEKNEQINDE		SCG2	2192/334890	1.000	3056	0.866	0.179121	-1.1	0	-3	18	-1.2056	-3	3	0	0	0.000000	-5.0667	0	0.15	0.700000	0.4444	0.2556	0	0	0	0	0	-0.2811	-15.2	0	1	0	0	0.4103	1.1038	0.8856	0	0.1683	0.7844	-0.4513	0.0225	-0.76
HIP_3057	HLVEALYLV	AEKRKRLGE	HLVEALYLVAEKRKRLGE		SCG1	10628/367830	0.997	3057	0.866	0.186898	0.1833	0	2	18	-0.2778	1	1	0	0	1.000000	-4.1	0	0.1	1.850000	0.3333	1.5167	0	0	0	0	0	0.2781	-4.1	1	0	0	0	0.3436	1.2272	0.7023	0.1667	0.1181	0.4932	0.063	0.01	-0.41
HIP_3058	LVCGERGFF	LKRQYDRVA	LVCGERGFFLKRQYDRVA		SCG1	12323/367830	0.997	3058	0.866	0.067121	0.1	0.1667	2	18	-0.2278	2	2	0	0	1.000000	-3.2667	2	0.0167	1.650000	0.4444	1.2056	0	1	0	1	-6.5333	0.1206	-6.5333	1	0	0	0	0.3603	1.0405	0.8356	0.1667	0.1298	0.6983	0.036	0.0003	-0.0544
HIP_3059	SHLVEALYL	QINDEMKRS	SHLVEALYLQINDEMKRS		SCG2	2256/334890	1.000	3059	0.866	0.204190	0.6333	0	0	18	-0.4556	-1	1	0	0	0.000000	-2.9333	0	0.1	0.000000	0.2222	-0.2222	0	0	0	0	0	-0.1407	-2.9333	1	0	0	0	0.3718	1.0803	0.8195	0.5	0.1382	0.6716	0.2355	0.01	-0.2933
HIP_3060	AGSLQPLAL	NTATCATQ	AGSLQPLALNTATCATQ		IAPP	12/45018	1.000	3060	0.866	0.195836	1.1667	0	0	17	0.3353	0	0	1	0	0.000000	-3.9333	0	0.1	0.450000	0	0.45	0	0	0	0	0	0.525	0	1	0	0	0	0.3333	1.1667	0.8067	0.6667	0.1111	0.6507	0.3889	0.01	-0.3933
HIP_3061	SHLVEALYL	PRTRLEDPA	SHLVEALYLPRTRLEDPA		NPY	305/49410	0.999	3061	0.866	0.243678	-0.0833	0.1667	0	18	-0.3167	-1	1	0	0	0.000000	-4.3667	1	0.1667	0.000000	0.3333	-0.3333	1	0	1	0	-4.3667	0.0278	-4.3667	1	0	0	0	0.4269	1.0072	0.8823	0.1667	0.1823	0.7785	-0.0356	0.0278	-0.7278
HIP_3062	LVCGERGFF	QYTPQSLAT	LVCGERGFFQYTPQSLAT		SCG2	2613/334890	1.000	3062	0.866	0.051924	-0.05	0.1667	0	18	0.0444	0	3	0	0	0.000000	-3.5667	0	0.0167	0.700000	0.2222	0.4778	0	1	0	1	0	-0.0239	-10.7	1	0	0	0	0.4551	0.9769	0.9245	0.3333	0.2071	0.8547	-0.0228	0.0003	-0.0594
HIP_3063	SHLVEALY	ETQQWPERK	SHLVEALYETQQWPERK		SCG2	2931/334890	1.000	3063	0.866	0.209730	-0.5667	0	0	17	-1.2235	-1	2	1	0	0.000000	-4	-1	0.15	0.000000	0.2222	-0.2222	0	0	0	0	4	0.1259	-8	0	1	0	0	0.4385	1.2036	0.7912	0.1667	0.1922	0.6259	-0.2485	0.0225	-0.6
HIP_3064	SHLVEALYL	EKIESQTQE	SHLVEALYLEKIESQTQE		SCG2	2897/334890	1.000	3064	0.866	0.169481	0.5667	0	-2	18	-0.5444	-3	1	0	0	0.000000	-3.0667	0	0.1	0.000000	0.4444	-0.4444	0	0	0	0	0	-0.2519	-3.0667	1	0	0	0	0.3436	1.2055	0.6706	0.1667	0.1181	0.4498	0.1947	0.01	-0.3067
HIP_3065	HLVEALYLV	NYQKGERGE	HLVEALYLVNYQKGERGE		SCG1	4302/367830	0.999	3065	0.866	0.138427	-0.2667	0	0	18	-0.6056	-1	2	0	0	0.000000	-5	0	0.1	1.400000	0.1111	1.2889	0	0	0	0	0	-0.3437	-10	1	0	0	0	0.4103	1.0305	0.8923	0.3333	0.1683	0.7962	-0.1094	0.01	-0.5
HIP_3066	ERGFFYTPK	KESKDQLSD	ERGFFYTPKKESKDQLSD		SCG2	9388/334890	0.999	3066	0.866	-0.054062	-2.4	0.3333	0	18	-1.6611	0	2	0	0	0.000000	-0.6667	-1	0	0.700000	0.4444	0.2556	1	0	1	0	0.6667	-0.6133	-1.3333	0	0	1	0	0.5603	0.9588	1.039	-0.5	0.3139	1.0795	-1.3446	0	0
HIP_3067	VCGERGFFY	DREKGLSAE	VCGERGFFYDREKGLSAE		CMGA	2774/247050	0.999	3067	0.866	0.187673	-1.2	0	-1	18	-0.6833	-1	3	0	0	0.000000	-5.2667	-1	0.15	0.700000	0.5556	0.1444	0	0	0	0	5.2667	-0.1733	-15.8	0	1	0	0	0.3821	1.1624	0.7768	-0.1667	0.146	0.6034	-0.4585	0.0225	-0.79
HIP_3068	GSHLVEALY	YLARMLVKY	GSHLVEALYYLARMLVKY		SCG2	1717/334890	1.000	3068	0.866	0.002700	1.4333	0	2	18	0.4944	1	2	0	0	0.000000	0	0	0	2.000000	0.2222	1.7778	0	0	0	0	0	2.5481	0	0	1	0	0	0.2769	1.2572	0.689	0.6667	0.0767	0.4747	0.3969	0	0
HIP_3069	CGERGFFYT	EKNEQINDE	CGERGFFYTEKNEQINDE		SCG2	4665/334890	1.000	3069	0.866	0.120269	-1.6833	0	-3	18	-1.4778	-3	3	0	0	0.000000	-3.9	0	0.1	0.000000	0.3333	-0.3333	0	0	0	0	0	0.5611	-11.7	0	1	0	0	0.4769	1.0422	0.9456	-0.1667	0.2275	0.8942	-0.8028	0.01	-0.39
HIP_3070	CGERGFFYT	QWPERKLK	CGERGFFYTQWPERKLK		SCG2	6611/334890	0.999	3070	0.866	0.099622	-0.8667	0.1667	2	17	-1.2176	2	4	1	0	0.000000	-2.2667	0	0.0667	0.000000	0.1111	-0.1111	1	0	1	0	0	0.0963	-9.0667	0	1	0	0	0.4936	0.9588	0.979	0.3333	0.2436	0.9584	-0.4278	0.0044	-0.1511
HIP_3071	LVCGERGFF	KTPGRAGTE	LVCGERGFFKTPGRAGTE		SCG2	3474/334890	1.000	3071	0.866	0.123159	-0.1667	0.3333	1	18	-0.3667	1	2	0	0	0.000000	-3.8	1	0.0833	0.700000	0.3333	0.3667	1	1	1	1	-3.8	-0.0611	-7.6	1	0	0	0	0.4718	0.8869	1.0145	0.1667	0.2226	1.0292	-0.0786	0.0069	-0.3167
HIP_3072	VEALYLVC	NYDWWEKKP	VEALYLVCNYDWWEKKP		SCG1	1618/367830	1.000	3072	0.866	0.138056	0.3667	0	-1	17	-0.4412	-1	4	1	0	0.000000	-6.2667	-1	0.1	0.625000	0.1111	0.5139	0	0	0	0	6.2667	0.1884	-25.0667	0	0	0	0	0.3821	1.0158	0.8801	0.1667	0.146	0.7746	0.1401	0.01	-0.6267
HIP_3073	SLYQLENYC	GYEKRNLAR	SLYQLENYCGYEKRNLAR		SCG1	2391/367830	1.000	3073	0.866	0.067992	-1.25	0.1667	1	18	-1.1	1	2	0	0	0.000000	-0.9667	-1	0.0833	0.950000	0.3333	0.6167	0	1	0	1	0.9667	-0.7708	-1.9333	0	0	0	0	0.5321	0.9574	1.0868	-0.1667	0.2831	1.1811	-0.6651	0.0069	-0.0806
HIP_3074	LVCGERGFF	QSAPKENKP	LVCGERGFFQSAPKENKP		SCG2	622/334890	1.000	3074	0.866	0.064369	0.45	0.3333	1	18	-0.7333	1	2	0	0	0.000000	-2.5667	0	0.0333	0.700000	0.2222	0.4778	0	1	0	1	0	0.215	-5.1333	1	0	0	0	0.4167	1.0433	0.9233	0.5	0.1736	0.8525	0.1875	0.0011	-0.0856
HIP_3075	GSLQPLAL	FQDKQYSS	GSLQPLALFQDKQYSS		SCG1	1215/367830	1.000	3075	0.866	0.168063	0.8667	0	0	16	-0.475	0	1	0	0	1.000000	-4.5333	-1	0.1	1.150000	0.2222	0.9278	0	0	0	0	4.5333	0.8041	-4.5333	1	0	0	0	0.3051	1.2303	0.6912	0.5	0.0931	0.4777	0.2644	0.01	-0.4533
HIP_3076	HLVEALYL	DLEWERYR	HLVEALYLDLEWERYR		SCG1	805/367830	1.000	3076	0.866	0.138385	0.5167	0	-1	16	-0.5312	-2	2	0	0	0.000000	-3.1667	-2	0.1	0.000000	0.3333	-0.3333	0	0	0	0	6.3333	-0.1722	-6.3333	1	0	0	0	0.3436	1.2422	0.6756	0.1667	0.1181	0.4565	0.1775	0.01	-0.3167
HIP_3077	VCGERGFFY	QECFETLRG	VCGERGFFYQECFETLRG		CMGA	1098/247050	1.000	3077	0.866	0.103862	-0.0333	0	-1	18	-0.2667	-1	4	0	0	0.000000	-2.9333	-1	0.1	1.400000	0.2222	1.1778	0	0	0	0	2.9333	-0.0393	-11.7333	0	1	0	0	0.4103	1.1772	0.789	0.1667	0.1683	0.6225	-0.0137	0.01	-0.2933
HIP_3078	AGSLQPLAL	RAYFMSDTR	AGSLQPLALRAYFMSDTR		SCG1	10961/367830	0.997	3078	0.866	0.112603	0.9	0	1	18	-0.0056	1	2	0	0	0.000000	-4.4667	1	0.05	1.150000	0.1111	1.0389	0	0	0	0	-4.4667	0.935	-8.9333	1	0	0	0	0.2769	1.2572	0.689	0.5	0.0767	0.4747	0.2492	0.0025	-0.2233
HIP_3079	VCGERGFFY	TENENTKF	VCGERGFFYTENENTKF		SCG1	1312/367830	1.000	3079	0.866	0.182895	-0.5667	0	-1	17	-0.8412	-1	3	1	0	0.000000	-4	-1	0.15	0.700000	0.3333	0.3667	0	0	0	0	4	-0.2078	-12	0	1	0	0	0.4385	1.0719	0.8945	0.1667	0.1922	0.8001	-0.2485	0.0225	-0.6
HIP_3080	VCGERGFFY	NPYQGVSVP	VCGERGFFYNPYQGVSVP		SCG2	636/334890	1.000	3080	0.866	0.138155	-0.35	0.1667	0	18	-0.1167	0	4	0	0	0.000000	-3.5667	0	0.1167	0.700000	0.1111	0.5889	1	0	1	0	0	-0.2061	-14.2667	0	1	0	0	0.4269	0.9472	1.0156	0.3333	0.1823	1.0315	-0.1494	0.0136	-0.4161
HIP_3081	ERGFFYTPK	GQNGERATR	ERGFFYTPKGQNGERATR		SCG2	2765/334890	1.000	3081	0.866	-0.051687	-2.2667	0.3333	2	18	-1.6389	2	2	0	0	0.000000	-0.4	-1	0.0167	0.700000	0.1111	0.5889	1	1	1	1	0.4	-1.3348	-0.8	0	0	1	0	0.6051	0.7836	1.2378	-0.1667	0.3662	1.5322	-1.3716	0.0003	-0.0067
HIP_3082	SHLVEALYL	YQKGERGED	SHLVEALYLYQKGERGED		SCG1	9460/367830	0.998	3082	0.866	0.157772	-0.4	0	-1	18	-0.8833	-2	2	0	0	0.000000	-5	1	0.1	0.000000	0.2222	-0.2222	0	0	0	0	-5	0.0889	-10	1	0	0	0	0.3821	1.1291	0.7835	0.1667	0.146	0.6138	-0.1528	0.01	-0.5
HIP_3083	HLVEALYLV	WRPSSREDS	HLVEALYLVWRPSSREDS		CMGA	2274/247050	0.999	3083	0.866	0.151283	-0.05	0.1667	0	18	-0.4056	-1	2	0	0	1.000000	-4.5667	1	0.1167	1.400000	0.1111	1.2889	1	0	1	0	-4.5667	-0.0644	-9.1333	1	0	0	0	0.3987	0.9991	0.8735	0.3333	0.159	0.7629	-0.0199	0.0136	-0.5328
HIP_3084	AGSLQPLAL	SMASLGEKR	AGSLQPLALSMASLGEKR		SCG1	987/367830	1.000	3084	0.866	0.103188	2.05	0.1667	1	18	0.1278	1	0	0	0	0.000000	-2.1667	0	0.0667	0.450000	0	0.45	0	0	0	0	0	0.9225	0	1	0	0	0	0.2667	1.25	0.6867	0.8333	0.0711	0.4715	0.5467	0.0044	-0.1444
HIP_3085	CGERGFFYT	QESKGQPR	CGERGFFYTQESKGQPR		SCG1	7301/367830	0.999	3085	0.866	0.167231	-1.1667	0.1667	1	17	-1.4353	1	3	1	0	0.000000	-2.8667	-1	0.1167	0.000000	0.2222	-0.2222	0	0	0	0	2.8667	0.2593	-8.6	0	1	0	0	0.5051	1.0786	0.8978	0	0.2552	0.8061	-0.5893	0.0136	-0.3344
HIP_3086	ERGFFYTPK	KGERGEDSS	ERGFFYTPKKGERGEDSS		SCG1	4555/367830	0.999	3086	0.866	-0.064843	-2.3333	0.3333	0	18	-1.7556	0	2	0	0	0.000000	-0.5333	-1	0.0167	0.700000	0.4444	0.2556	1	1	1	1	0.5333	-0.5963	-1.0667	0	0	1	0	0.5769	0.9088	1.089	-0.5	0.3328	1.1859	-1.3462	0.0003	-0.0089
HIP_3087	GERGFFYTP	ENEPQSAPK	GERGFFYTPENEPQSAPK		SCG2	3250/334890	1.000	3087	0.866	-0.002180	-2.35	0.1667	-1	18	-1.4944	-1	3	0	0	0.000000	-2.3	-2	0.0333	0.700000	0.2222	0.4778	1	0	1	0	4.6	-1.1228	-6.9	0	0	0	1	0.5885	1.0186	1.0712	-0.3333	0.3463	1.1474	-1.3829	0.0011	-0.0767
HIP_3088	VCGERGFFY	QTQEEVRD	VCGERGFFYQTQEEVRD		SCG2	1215/334890	1.000	3088	0.866	0.180723	-0.5667	0	-2	17	-0.9588	-2	3	1	0	0.000000	-4	0	0.15	0.700000	0.2222	0.4778	0	0	0	0	0	-0.2707	-12	0	1	0	0	0.4385	1.0786	0.8378	0.3333	0.1922	0.7019	-0.2485	0.0225	-0.6
HIP_3089	LVCGERGFF	ENKPYALNS	LVCGERGFFENKPYALNS		SCG2	5842/334890	0.999	3089	0.866	0.112340	-0.95	0.1667	0	18	-0.2889	0	2	0	0	0.000000	-5.3667	0	0.0667	0.700000	0.4444	0.2556	0	1	0	1	0	-0.2428	-10.7333	1	0	0	0	0.4551	1.0169	0.9845	0	0.2071	0.9692	-0.4324	0.0044	-0.3578
HIP_3090	GERGFFYTP	KWQQQGDLQ	GERGFFYTPKWQQQGDLQ		SCG1	6097/367830	0.999	3090	0.866	-0.004564	-1.9833	0.1667	0	18	-1.4278	0	4	0	1	0.000000	-1.5667	1	-0.0167	0.700000	0.1111	0.5889	1	0	1	0	-1.5667	-1.168	-6.2667	1	0	0	1	0.5321	0.9291	1.0301	0	0.2831	1.0612	-1.0552	0.0003	0.0261
HIP_3091	LVCGERGFF	EKVLPRLPY	LVCGERGFFEKVLPRLPY		SCG2	4832/334890	1.000	3091	0.866	0.047410	0.3333	0.1667	1	18	0.15	1	2	0	0	0.000000	-2.8	0	0.0167	1.650000	0.4444	1.2056	0	1	0	1	0	0.4019	-5.6	1	0	0	0	0.3885	1.0819	0.8078	0.1667	0.1509	0.6526	0.1295	0.0003	-0.0467
HIP_3092	SHLVEALYL	NPFKRTNEI	SHLVEALYLNPFKRTNEI		SCG2	258/334890	1.000	3092	0.866	0.191176	0.6667	0.1667	1	18	-0.2944	0	2	0	0	0.000000	-2.8667	0	0.1167	0.000000	0.2222	-0.2222	1	0	1	0	0	-0.1481	-5.7333	1	0	0	0	0.3885	1.0103	0.9312	0.5	0.1509	0.867	0.259	0.0136	-0.3344
HIP_3093	QHLCGSHLV	LFQNRMLSK	QHLCGSHLVLFQNRMLSK		SCG2	5609/334890	0.999	3093	0.866	0.059089	1.3167	0	4	18	-0.0389	2	1	0	0	1.000000	-0.5667	0	0.05	1.900000	0	1.9	0	0	0	0	0	2.5017	-0.5667	1	0	0	0	0.3051	1.1653	0.6645	0.5	0.0931	0.4415	0.4018	0.0025	-0.0283
HIP_3094	HLVEALYLV	RQAENEPQS	HLVEALYLVRQAENEPQS		SCG2	5519/334890	0.999	3094	0.866	0.158670	0.0833	0	-1	18	-0.5	-2	1	0	0	0.000000	-4.3	1	0.1	1.400000	0.2222	1.1778	0	0	0	0	-4.3	0.0981	-4.3	1	0	0	0	0.3436	1.1605	0.7423	0.3333	0.1181	0.551	0.0286	0.01	-0.43
HIP_3095	SHLVEALYL	CATQRLANF	SHLVEALYLCATQRLANF		IAPP	156/45018	1.000	3095	0.866	0.071143	1.65	0	1	18	0.5056	0	1	0	0	0.000000	-0.9	0	0.05	0.625000	0.1111	0.5139	0	0	0	0	0	0.8479	-0.9	1	0	0	0	0.3436	1.1538	0.739	0.5	0.1181	0.5461	0.5669	0.0025	-0.045
HIP_3096	VCGERGFFY	RSSPETLIS	VCGERGFFYRSSPETLIS		NPY	283/49410	0.999	3096	0.866	0.214073	-0.3	0.3333	0	18	-0.1222	0	3	0	0	0.000000	-3.4667	1	0.1833	0.700000	0.2222	0.4778	0	0	0	0	-3.4667	-0.1433	-10.4	0	1	0	0	0.4103	1.0072	0.9023	0.1667	0.1683	0.8142	-0.1231	0.0336	-0.6356
HIP_3097	CGERGFFYT	DWWEKKPFS	CGERGFFYTDWWEKKPFS		SCG1	6190/367830	0.999	3097	0.865	0.050327	-0.75	0	0	18	-1.05	0	5	0	0	0.000000	-2.0333	-1	0	0.000000	0.2222	-0.2222	0	0	0	0	2.0333	0.1667	-10.1667	0	1	0	0	0.4205	1.0194	0.8646	0.3333	0.1768	0.7476	-0.3154	0	0
HIP_3098	DLQVGQVEL	YYSALRHYI	DLQVGQVELYYSALRHYI		NPY	1195/49410	0.994	3098	0.865	0.058126	0.1833	0.1667	0	18	-0.0389	-1	2	0	0	0.000000	-2.6333	1	0.0167	0.450000	0.1111	0.3389	0	0	0	0	-2.6333	0.0621	-5.2667	1	0	0	0	0.4103	1.1188	0.7923	0.1667	0.1683	0.6278	0.0752	0.0003	-0.049

HIP_3567	LVCGERGFF	NKPYALNSE	LVCGERGFFNKPYALNSE		SCG2	3220/334890	1.000	3567	0.864	0.107159	-0.6333	0.3333	0	18	-0.2889	0	3	0	0
HIP_3568	VCGERGFFY	YWDEDLLMK	VCGERGFFYYWDEDLLMK		SCG2	2337/334890	1.000	3568	0.864	0.056962	-0.2333	0	-2	18	-0.2722	-2	5	0	0
HIP_3569	VCGERGFFY	DTLSKPSPM	VCGERGFFYDTLSKPSPM		CMGA	1577/247050	1.000	3569	0.864	0.142323	0.65	0	0	18	-0.2778	0	3	0	0
HIP_3570	CGERGFFY	NQEKVLPRL	CGERGFFYNQEKVLPRL		SCG2	4151/334890	1.000	3570	0.864	0.190621	-1.0333	0	1	17	-0.6294	1	3	1	0
HIP_3571	VCGERGFFY	RPQSEESWD	VCGERGFFYRPQSEESWD		SCG1	2222/367830	1.000	3571	0.864	0.188218	-0.8833	0.1667	-2	18	-1.1333	-2	3	0	0
HIP_3572	VCGERGFFY	IAEKFSQRG	VCGERGFFYIAEKFSQRG		SCG1	3931/367830	0.999	3572	0.864	0.036922	1.1833	0	1	18	-0.2944	1	3	0	0
HIP_3573	LVCGERGFF	KAEGNNQAP	LVCGERGFFKAEGNNQAP		CMGA	4844/247050	0.998	3573	0.864	0.064453	-0.0667	0.1667	0	18	-0.5	0	2	0	0
HIP_3574	CTSICSLYQ	AYLKRLVNA	CTSICSLYQAYLKRLVNA		SCG2	1786/334890	1.000	3574	0.864	0.001496	0.55	0	2	18	0.4667	2	2	0	0
HIP_3575	CTSICSLYQ	LHYRKKSAE	CTSICSLYQLHYRKKSAE		SCG1	11183/367830	0.997	3575	0.864	0.012212	-0.2833	0	3	18	-0.5167	2	2	0	0
HIP_3576	VCGERGFFY	SETHAAGHS	VCGERGFFYSETHAAGHS		SCG1	484/367830	1.000	3576	0.864	0.196572	-0.1167	0.1667	1	18	-0.3778	-1	3	0	0
HIP_3577	CTSICSLYQ	RLPYGAGRS	CTSICSLYQRLPYGAGRS		SCG2	7797/334890	0.999	3577	0.864	0.070532	-0.55	0.1667	2	18	-0.0944	2	2	0	0
HIP_3578	IVEQCCTSI	QQWPERKLK	IVEQCCTSIQQWPERKLK		SCG2	9200/334890	0.999	3578	0.864	0.023587	-0.8167	0.1667	1	18	-0.6556	1	1	0	0
HIP_3579	HLVEALYLV	YLNQEKAEK	HLVEALYLVYLNQEKAEK		SCG2	945/334890	1.000	3579	0.864	0.068275	0.95	0	0	18	-0.2167	-1	2	0	0
HIP_3580	LYQLENYCN	MRESTENVP	LYQLENYCNMRESTENVP		NPY	1206/49410	0.993	3580	0.864	-0.040638	-1.4	0	-2	18	-1.0278	-2	1	0	0
HIP_3581	VCGERGFFY	NQLLQKEPD	VCGERGFFYNQLLQKEPD		SCG2	310/334890	1.000	3581	0.864	0.108771	0.1833	0	-1	18	-0.7333	-1	3	0	0
HIP_3582	CGERGFFYT	EEQYTPQSL	CGERGFFYTEEQYTPQSL		SCG2	2401/334890	1.000	3582	0.864	0.101387	-1.6167	0	-2	18	-0.9611	-2	4	0	0
HIP_3583	CSLYQLENY	EEDNRDSSM	CSLYQLENYEEDNRDSSM		CMGA	5121/247050	0.998	3583	0.864	-0.027032	-3.1333	0	-4	18	-1.4167	-4	1	0	0
HIP_3584	GSLQPLAL	WAEGGGHSR	GSLQPLALWAEGGGHSR		SCG1	2336/367830	1.000	3584	0.864	0.089841	1.1333	0	1	17	-0.3176	0	1	1	1
HIP_3585	VCGERGFFY	RGSEEYRAP	VCGERGFFYRGSEEYRAP		SCG1	820/367830	1.000	3585	0.864	0.210543	-0.2333	0.3333	0	18	-0.8944	0	3	0	0
HIP_3586	VCGERGFFY	KEELVARS	VCGERGFFYKEELVARS		SCG1	5400/367830	0.999	3586	0.864	0.178614	-1.1	0	0	17	-0.2471	0	3	1	0
HIP_3587	VCGERGFFY	GQNGERATR	VCGERGFFYGQNGERATR		SCG2	93/334890	1.000	3587	0.864	0.210532	-0.5167	0.1667	1	18	-0.9444	1	3	0	0
HIP_3588	CGERGFFYT	EWERYRGR	CGERGFFYTEWERYRGR		SCG1	9569/367830	0.998	3588	0.864	0.085817	-1.1833	0	1	17	-1.5176	1	4	1	0
HIP_3589	GSLQPLAL	GFRGPGPQ	GSLQPLALGFRGPGPQ		CMGA	2213/247050	0.999	3589	0.864	0.230612	1.2167	0.1667	1	16	-0.1688	1	1	0	0
HIP_3590	LVEALYLVC	KNFFPEYNY	LVEALYLVCKNFFPEYNY		SCG1	3967/367830	0.999	3590	0.864	0.119204	0.9833	0	-1	18	0.35	-1	3	0	0
HIP_3591	LVCGERGFF	IKEHLNQGS	LVCGERGFFIKEHLNQGS		SCG2	3717/334890	1.000	3591	0.864	0.032883	0.3833	0.1667	1	18	-0.1778	0	2	0	0
HIP_3592	VCGERGFFY	ETQNAFLNE	VCGERGFFYETQNAFLNE		SCG1	234/367830	1.000	3592	0.864	0.169728	-0.5667	0	-2	18	-0.4222	-2	3	0	0
HIP_3593	ICSLYQLEN	QEKAEKGRE	ICSLYQLENQEKAEKGRE		SCG2	19851/334890	0.996	3593	0.864	0.060649	-2.35	0	-1	18	-1.2722	-1	1	0	0
HIP_3594	LVCGERGFF	NNFGAILSS	LVCGERGFFNNFGAILSS		IAPP	94/45018	1.000	3594	0.864	0.048415	0.1667	0.1667	0	18	0.6222	0	3	0	0
HIP_3595	GSHLVEALY	QKEPDLRLE	GSHLVEALYQKEPDLRLE		SCG2	3285/334890	1.000	3595	0.864	0.187750	-1.1	0	-1	18	-0.6667	-2	1	0	0
HIP_3596	GSLQPLALE	YGKRSSPET	GSLQPLALEYGKRSSPET		NPY	1470/49410	0.992	3596	0.864	0.123175	-0.5833	0.1667	0	18	-0.7833	0	1	0	0
HIP_3597	CGERGFFYT	KEPDLRLEN	CGERGFFYTKEPDLRLEN		SCG2	4968/334890	1.000	3597	0.864	0.145879	-1.3667	0.1667	-1	18	-1.0611	-1	3	0	0
HIP_3598	LYQLENYCN	GYEKRNLAR	LYQLENYCNGYEKRNLAR		SCG1	3097/367830	1.000	3598	0.864	0.074859	-1.25	0.1667	1	18	-1.25	1	2	0	0
HIP_3599	ERGFFYTPK	EEPSSKDVM	ERGFFYTPKEEPSSKDVM		CMGA	2132/247050	0.999	3599	0.864	-0.080833	-2.4667	0.3333	-1	18	-1.2111	-1	2	0	0
HIP_3600	CGSHLVEAL	ENLRQQAHK	CGSHLVEALENLRQQAHK		SCG2	14228/334890	0.998	3600	0.864	0.061588	-0.1833	0	2	18	-0.6556	0	0	0	0
HIP_3601	VCGERGFFY	SSMKLSFRA	VCGERGFFYSSMKLSFRA		CMGA	777/247050	1.000	3601	0.864	0.194364	0.7667	0.3333	2	18	0.0944	2	3	0	0
HIP_3602	VCGERGFFY	QASAKKEE	VCGERGFFYQASAKKEE		SCG1	1192/367830	1.000	3602	0.864	0.151087	0.3	0.1667	0	18	-0.4889	0	3	0	0
HIP_3603	LCGSHLVEA	DLFQNRMLS	LCGSHLVEADLFQNRMLS		SCG2	5380/334890	0.999	3603	0.864	0.016921	0.9333	0	0	18	0.2611	-1	1	0	0
HIP_3604	GERGFFYTP	GNKRLGLSG	GERGFFYTPGNKRLGLSG		NPY	566/49410	0.997	3604	0.864	0.037584	-1.9	0.3333	2	18	-0.7278	2	3	0	0
HIP_3605	VCGERGFFY	SMKLSFRAR	VCGERGFFYSMKLSFRAR		CMGA	6452/247050	0.998	3605	0.864	0.170201	0.25	0.1667	3	18	-0.1111	3	3	0	0
HIP_3606	ERGFFYTPK	QDKQYSSHH	ERGFFYTPKQDKQYSSHH		SCG1	16414/367830	0.994	3606	0.864	0.001035	-2.85	0.1667	1	18	-1.8889	1	2	0	0
HIP_3607	SHLVEALYL	PKAAWIPHV	SHLVEALYLPKAAWIPHV		SCG2	5500/334890	0.999	3607	0.864	0.213706	0.4333	0.1667	2	18	0.5389	0	1	0	0
HIP_3608	CGERGFFY	GYPKTPGRA	CGERGFFYGYPKTPGRA		SCG2	754/334890	1.000	3608	0.864	0.180574	0.1667	0.3333	2	17	-0.8588	2	4	1	0
HIP_3609	VCGERGFFY	NQEKAEKG	VCGERGFFYNQEKAEKG		SCG2	3628/334890	1.000	3609	0.864	0.191464	-1.0333	0	0	17	-1.0706	0	3	1	0

HIP_4078	GERGFFYTP	QQQGDLQDT	GERGFFYTPQQQGDLQDT		SCG1	2930/367830	1.000	4078	0.862	-0.014857	-2.35	0.1667	-2	18	-1.3944	-2	3	0	1	0.000000	-2.3	0	0.0333	0.700000	0	0.7	1	0	1	0	0	-1.645	-6.9	0	0	0	1	0.5885	0.9586	1.0545	0	0.3463	1.1119	-1.3829	0.0011	-0.0767
HIP_4079	ERGFFYTPK	SEEKGHPQE	ERGFFYTPKSEEKGHPQE		SCG1	8197/367830	0.998	4079	0.862	-0.068468	-2.3333	0.3333	0	18	-1.9	-1	2	0	0	0.000000	-0.5333	-1	0	0.700000	0.4444	0.2556	1	0	1	0	1.6	-0.5963	-1.0667	0	0	1	0	0.5885	1.0403	1.0112	-0.5	0.3463	1.0224	-1.3731	0	0
HIP_4080	CGERGFFYT	QYWDEDLLM	CGERGFFYTQYWDEDLLM		SCG2	2993/334890	1.000	4080	0.862	0.021204	-0.8167	0	-3	18	-0.5222	-3	5	0	0	0.000000	-2.1667	0	0	0.000000	0.1111	-0.1111	0	0	0	0	0	0.0907	-10.8333	0	1	0	0	0.4487	1.0341	0.8768	0.3333	0.2013	0.7688	-0.3665	0	0
HIP_4081	GSLQPLAL	FERRDNMN	GSLQPLALFERRDNMN		SCG1	9236/367830	0.998	4081	0.862	0.193923	0.7	0	0	16	-0.7125	0	1	0	0	1.000000	-4.8667	0	0.1	1.150000	0.3333	0.8167	0	0	0	0	0	0.5717	-4.8667	1	0	0	0	0.3051	1.2969	0.6512	0.3333	0.0931	0.424	0.2136	0.01	-0.4867
HIP_4082	SHLVEALYL	KVAHQLQAL	SHLVEALYLKVAHQLQAL		CMGA	7019/247050	0.997	4082	0.862	0.104356	1.4	0	2	18	0.55	0	1	0	0	0.000000	-1.4	1	0.05	0.000000	0.2222	-0.2222	0	0	0	0	-1.4	-0.3111	-1.4	1	0	0	0	0.2769	1.1922	0.6623	0.5	0.0767	0.4387	0.3877	0.0025	-0.07
HIP_4083	CGSHLVEAL	YYDPLQWKS	CGSHLVEALYYDPLQWKS		SCG1	11994/367830	0.997	4083	0.862	0.023118	-0.6667	0	0	18	-0.2667	-1	2	0	0	0.000000	-2.7333	0	0	1.400000	0.2222	1.1778	0	0	0	0	0	-0.7852	-5.4667	1	0	0	0	0.3821	1.2208	0.7701	0	0.146	0.5931	-0.2547	0	0
HIP_4084	ICSLYQLEN	EEEGPTVVL	ICSLYQLENEEEGPTVVL		CMGA	2192/247050	0.999	4084	0.862	0.004148	-2.2833	0	-4	18	0.0556	-4	1	0	1	0.000000	-2.4333	-2	0.05	0.000000	0.4444	-0.4444	0	0	0	0	4.8667	1.0148	-2.4333	0	1	0	0	0.5333	1.335	0.8367	-0.5	0.2844	0.7	-1.2178	0.0025	-0.1217
HIP_4085	HLVEALYLV	NQKTSYFPN	HLVEALYLVNQKTSYFPN		SCG2	7768/334890	0.999	4085	0.862	0.218557	-0.7	0	1	18	-0.1333	0	1	0	0	0.000000	-5.8667	1	0.15	1.400000	0.1111	1.2889	0	0	0	0	-5.8667	-0.9022	-5.8667	1	0	0	0	0.4103	1.0305	0.8923	0.1667	0.1683	0.7962	-0.2872	0.0225	-0.88
HIP_4086	ERGFFYTPK	RQVLKTSRK	ERGFFYTPKRQVLKTSRK		SCG1	13673/367830	0.996	4086	0.862	0.010417	-1.6667	0.1667	5	18	-1.3389	5	2	0	0	0.000000	0.8	0	-0.0667	1.650000	0.2222	1.4278	1	0	1	0	0	-2.3796	1.6	0	0	1	0	0.4936	0.9355	0.9623	-0.1667	0.2436	0.926	-0.8226	0.0044	-0.0533
HIP_4087	TSICSLYQL	EKNFPMDMS	TSICSLYQLEKNFPMDMS		SCG2	8455/334890	0.999	4087	0.862	0.142925	-1.9833	0	-1	18	-0.15	-1	2	0	0	0.000000	-3.3	0	0.1	0.700000	0.2222	0.4778	0	0	0	0	0	-0.9476	-6.6	1	0	0	0	0.4769	1.1055	0.9323	-0.1667	0.2275	0.8692	-0.9459	0.01	-0.33
HIP_4088	CGERGFFY	LNQEKAEKG	CGERGFFYLNQEKAEKG		SCG2	6360/334890	0.999	4088	0.862	0.158799	0.1833	0	0	17	-1.0941	0	3	1	0	0.000000	-2.5	0	0.1	0.000000	0.2222	-0.2222	0	0	0	0	0	-0.0407	-7.5	0	1	0	0	0.2718	1.0836	0.8578	0.5	0.1382	0.7359	0.0682	0.01	-0.25
HIP_4089	VCGERGFFY	SWDEEDKRN	VCGERGFFYSWDEEDKRN		SCG1	515/367830	1.000	4089	0.862	0.124286	-0.15	0.1667	-2	18	-1.4111	-2	4	0	0	0.000000	-3.1667	-1	0.1167	0.700000	0.3333	0.3667	0	0	0	0	3.1667	-0.055	-12.6667	0	1	0	0	0.3821	1.0441	0.8535	0.3333	0.146	0.7284	-0.0573	0.0136	-0.3694
HIP_4090	ERGFFYTPK	DSEGLSQGL	ERGFFYTPKDSEGLSQGL		CMGA	3380/247050	0.999	4090	0.862	-0.083918	-2.3333	0.3333	-1	18	-0.8667	-1	2	0	0	0.000000	-0.5333	-1	0	0.700000	0.3333	0.3667	1	0	1	0	1.6	-0.8556	-1.0667	0	0	1	0	0.5603	0.9588	1.039	-0.5	0.3139	1.0795	-1.3073	0	0
HIP_4091	TSICSLYQL	RFQDKQYSS	TSICSLYQLRFQDKQYSS		SCG1	19170/367830	0.991	4091	0.862	0.123987	-1.0333	0	1	18	-0.6389	1	2	0	0	0.000000	-1.4	1	0.05	0.000000	0.2222	-0.2222	0	0	0	0	-1.4	0.2296	-2.8	1	0	0	0	0.4103	1.1272	0.8123	0.1667	0.1683	0.6598	-0.4239	0.0025	-0.07
HIP_4092	VCGERGFFY	GYEKRNLA	VCGERGFFYGYEKRNLA		SCG1	2774/367830	1.000	4092	0.862	0.165299	-0.15	0.1667	1	17	-0.5471	1	4	1	0	0.000000	-3.1667	-1	0.1333	0.700000	0.3333	0.3667	0	1	0	1	3.1667	-0.055	-12.6667	0	1	0	0	0.4269	1.0755	0.8756	0.1667	0.1823	0.7667	-0.064	0.0178	-0.4222
HIP_4093	AGSLQPLAL	THWLGAALS	AGSLQPLALTHWLGAALS		SCG2	5562/334890	0.999	4093	0.862	0.105095	0.7667	0	1	18	0.7722	0	1	0	0	0.000000	-4.7333	0	0.05	1.400000	0	1.4	0	0	0	0	0	1.0733	-4.7333	1	0	0	0	0.3436	1.1518	0.729	0.5	0.1181	0.5461	0.2634	0.0025	-0.2367
HIP_4094	HLVEALYLV	NNQKRERM	HLVEALYLVNNQKRERM		SCG2	5337/334890	0.999	4094	0.862	0.230995	-0.6333	0	2	17	-0.6706	1	1	1	0	0.000000	-5.7333	0	0.15	1.400000	0.1111	1.2889	0	0	0	0	0	-0.8163	-5.7333	1	0	0	0	0.4385	0.9719	1.0012	0.3333	0.1922	1.0023	-0.2777	0.0225	-0.86
HIP_4095	LVEALYLVC	ARAYGFRGP	LVEALYLVCARAYGFRGP		CMGA	5363/247050	0.998	4095	0.862	0.087609	1.6	0	1	18	0.7222	1	2	0	0	0.000000	-3.8	1	0.05	2.450000	0.1111	2.3389	0	0	0	0	-3.8	3.7422	-7.6	0	0	0	0	0.2769	1.2172	0.689	0.5	0.0767	0.4747	0.4431	0.0025	-0.19
HIP_4096	GSLQPLAL	QWPERKLKH	GSLQPLALQWPERKLKH		SCG2	6333/334890	0.999	4096	0.862	0.217147	0.5667	0.1667	3	17	-0.8412	2	1	1	0	0.000000	-5.1333	0	0.1167	0.950000	0.2222	0.7278	1	0	1	0	0	0.4124	-5.1333	1	0	0	0	0.3885	1.1253	0.8445	0.6667	0.1509	0.7132	0.2201	0.0136	-0.5989
HIP_4097	DLQVGQVE	ERGFFYTPK	DLQVGQVEERGFFYTPK		INSB	130/4968	0.987	4097	0.862	0.089416	-1.8667	0.1667	-1	17	-0.7353	-1	2	1	0	0.000000	-1.8667	1	0.0833	0.700000	0.3333	0.3667	0	1	0	1	-1.8667	-0.6844	-3.7333	0	0	1	0	0.5218	1.1186	0.8945	-0.3333	0.2723	0.8001	-0.974	0.0069	-0.1556
HIP_4098	GSLQPLALE	ENLRQQAHK	GSLQPLALEENLRQQAHK		SCG2	16167/334890	0.997	4098	0.862	0.072525	-0.1833	0	1	18	-0.9222	0	0	0	0	0.000000	-1.7667	0	0.05	0.950000	0.3333	0.6167	0	0	0	0	0	-0.1131	0	0	0	1	0	0.4	1.29	0.7833	0.1667	0.16	0.6136	-0.0733	0.0025	-0.0883
HIP_4099	AGSLQPLAL	PKENKPYAL	AGSLQPLALPKENKPYAL		SCG2	3058/334890	1.000	4099	0.862	0.245876	0.0667	0.1667	1	18	-0.2778	1	0	0	0	0.000000	-6.1333	0	0.1667	0.450000	0.2222	0.2278	1	0	1	0	0	0.0152	0	1	0	0	0	0.3885	1.1919	0.8045	0.1667	0.1509	0.6472	0.0259	0.0278	-1.0222
HIP_4100	SHLVEALYL	FERRDNMN	SHLVEALYLFERRDNMN		SCG1	9851/367830	0.998	4100	0.862	0.171226	0.1833	0	0	17	-0.5706	-1	2	1	0	1.000000	-3.8333	0	0.1	0.700000	0.4444	0.2556	0	0	0	0	0	0.0469	-7.6667	1	0	0	0	0.3436	1.2255	0.6923	0.1667	0.1181	0.4793	0.063	0.01	-0.3833
HIP_4101	ERGFFYTPK	EEDNRDSSM	ERGFFYTPKEEDNRDSSM		CMGA	3592/247050	0.999	4101	0.862	-0.055067	-2.7833	0.1667	-2	18	-1.7778	-2	2	0	0	0.000000	-1.4333	-4	-0.0167	0.700000	0.4444	0.2556	1	0	1	0	5.7333	-0.7113	-2.8667	0	0	1	0	0.5603	1.0772	0.9623	-0.6667	0.3139	0.926	-1.5584	0.0003	0.0239
HIP_4102	CGERGFFYT	NEKNFFPEY	CGERGFFYTNEKNFFPEY		SCG1	9300/367830	0.998	4102	0.862	0.139855	-1.6833	0	-1	18	-0.9944	-1	3	0	0	0.000000	-3.9	0	0.1	0.000000	0.2222	-0.2222	0	0	0	0	0	0.3741	-11.7	0	1	0	0	0.4769	1.0422	0.9456	-0.1667	0.2275	0.8942	-0.8028	0.01	-0.39
HIP_4103	SHLVEALYL	SGYPKTPGR	SHLVEALYLSGYPKTPGR		SCG2	151/334890	1.000	4103	0.862	0.225180	0.6333	0.3333	2	18	-0.3667	1	2	0	0	0.000000	-2.9333	0	0.15	0.000000	0.1111	-0.1111	0	1	0	1	0	-0.0704	-5.8667	1	0	0	0	0.4269	0.9905	0.919	0.3333	0.1823	0.8445	0.2704	0.0225	-0.44
HIP_4104	CGERGFFYT	QEESEESNV	CGERGFFYTQEESEESNV		SCG1	883/367830	1.000	4104	0.862	0.085961	-1.6167	0	-4	18	-1.1722	-4	3	0	0	0.000000	-3.7667	-2	0.1	0.000000	0.2222	-0.2222	0	0	0	0	7.5333	0.3593	-11.3	0	1	0	0	0.5051	1.1969	0.8212	-0.1667	0.2552	0.6743	-0.8166	0.01	-0.3767
HIP_4105	CGERGFFYT	DWMRIILEA	CGERGFFYTDWMRIILEA		SCG2	3529/334890	1.000	4105	0.862	0.024079	-0.2833	0	-1	18	0.0778	-1	4	0	0	0.000000	-1.1	-1	0	0.000000	0.2222	-0.2222	0	0	0	0	1.1	0.063	-4.4	0	1	0	0	0.3821	1.0491	0.8135	0.3333	0.146	0.6617	-0.1082	0	0
HIP_4106	LYQLENYCN	KFEVRLLRD	LYQLENYCNKFEVRLLRD		SCG1	13416/367830	0.996	4106	0.862	-0.021375	-1.15	0	0	18	-0.6556	0	2	0	0	0.000000	-0.7667	0	0.05	1.675000	0.3333	1.3417	0	0	0	0	0	-1.5429	-1.5333	0	1	0	0	0.4487	1.0741	0.9368	-0.1667	0.2013	0.8776	-0.516	0.0025	-0.0383
HIP_4107	CGERGFFY	RQAENEPQS	CGERGFFYRQAENEPQS		SCG2	9370/334890	0.999	4107	0.862	0.148657	-0.3167	0	-1	17	-1.4471	-1	3	1	0	0.000000	-3.5	1	0.1	0.000000	0.2222	-0.2222	0	0	0	0	-3.5	0.0704	-10.5	0	1	0	0	0.3436	1.1672	0.7756	0.3333	0.1181	0.6016	-0.1088	0.01	-0.35
HIP_4108	ERGFFYTPK	RSSPETLIS	ERGFFYTPKRSSPETLIS		NPY	949/49410	0.995	4108	0.862	-0.069639	-2.05	0.5	1	18	-0.8167	1	2	0	0	0.000000	0.0333	0	0.0167	0.700000	0.2222	0.4778	1	0	1	0	0	-0.9794	0.0667	0	0	1	0	0.5603	0.8405	1.1156	-0.3333	0.3139	1.2447	-1.1485	0.0003	0.0006
HIP_4109	CGERGFFYT	AENEKDRAD	CGERGFFYTAENEKDRAD		SCG1	3747/367830	0.999	4109	0.862	0.041428	-0.7333	0	-2	18	-1.3889	-2	3	0	0	0.000000	-2	-1	0.05	0.000000	0.2222	-0.2222	0	0	0	0	2	-0.167	-6	0	1	0	0	0.4385	1.1136	0.9045	0.1667	0.1922	0.8181	-0.3215	0.0025	-0.1
HIP_4110	VEALYLVC	EEARFQDKQ	VEALYLVCEEARFQDKQ		SCG1	513/367830	1.000	4110	0.862	0.108557	0.8833	0	-2	17	-0.3412	-2	2	1	0	0.000000	-5.2333	-2	0.1	1.325000	0.3333	0.9917	0	0	0	0	10.4667	0.876	-10.4667	0	0	0	0	0.3718	1.3086	0.6745	0.1667	0.1382	0.4549	0.3284	0.01	-0.5233
HIP_4111	CTSICSLYQ	TENVPRTRL	CTSICSLYQTENVPRTRL		NPY	867/49410	0.995	4111	0.862	0.101132	-1.45	0	1	18	-0.2667	1	1	0	0	0.000000	-2.2333	1	0.1	1.675000	0.1111	1.5639	0	0	0	0	2.2333	-2.2676	-2.2333	0	1	0	0	0.5051	1.0716	0.9412	0	0.2552	0.8858	-0.7324	0.01	-0.2233
HIP_4112	CCTSICSLY	WDEEDKRNY	CCTSICSLYWDEEDKRNY		SCG1	1423/367830	1.000	4112	0.862	-0.017370	-1.0333	0.1667	-2	18	-0.8833	-2	2	0	0	0.000000	-3.2	-2	0.0333	2.075000	0.3333	1.7417	0	0	0	0	6.4	-1.7997	-6.4	0	1	0	0	0.4487	1.1124	0.8601	0	0.2013	0.7398	-0.4637	0.0011	-0.1067
HIP_4113	HLCGSHLVE	WWEKKPFSE	HLCGSHLVEWWEKKPFSE		SCG1	11243/367830	0.997	4113	0.862	0.044886	-0.1333	0	1	18	-0.7222	-1	2	0	0	0.000000	-3.2667	0	0	1.050000	0.3333	0.7167	0	0	0	0	0	-0.0956	-6.5333	0	1	0	0	0.4103	1.2372	0.7156	0.3333	0.1683	0.5121	-0.0547	0.01	0
HIP_4114	CGERGFFYT	RMLVKYPEI	CGERGFFYTRMLVKYPEI		SCG2	6013/334890	0.999	4114	0.862	-0.017444	0.3333	0	1	18	-0.1722	1	3	0	0	0.000000	0.1333	1	0	1.050000	0.1111	0.9389	0	0	0	0	0.1333	0.313	0.4	0	1	0	0	0.3436	1.0955	0.7456	0.3333	0.1181	0.556	0.1145	0	0
HIP_4115	VCGERGFFY	QEEDLRKES	VCGERGFFYQEEDLRKES		SCG2	872/334890	1.000	4115	0.862	0.154774	-1.0333	0	-2	18	-1.15	-2	3	0	0	[illegible]	[illegible]	[illegible]	[illegible]	[illegible]	[illegible]	[illegible]	[illegible]	[illegible]	[illegible]	[illegible]	[illegible]	[illegible]	[illegible]	[illegible]	[illegible]	[illegible]	[illegible]	[illegible]	[illegible]	[illegible]	[illegible]	[illegible]	[illegible]	[illegible]	[illegible]	[illegible]
HIP_4116	AGSLQPLAL	DLEWERYRG	AGSLQPLALDLEWERYRG		SCG1	199/367830	1.000	4116	0.862	0.133096	1.0333	0	-1	18	-0.5333	-1	1	0	0	[illegible]	[illegible]	[illegible]	[illegible]	[illegible]	[illegible]	[illegible]	[illegible]	[illegible]	[illegible]	[illegible]	[illegible]	[illegible]	[illegible]	[illegible]	[illegible]	[illegible]	[illegible]	[illegible]	[illegible]	[illegible]	[illegible]	[illegible]	[illegible]	[illegible]	[illegible]	[illegible]
HIP_4117	HLVEALYLV	SFQRNQLLQ	HLVEALYLVSFQRNQLLQ		SCG2	740/334890	1.000	4117	0.862	0.174164	0.8667	0.1667	1	18	0.2611	0	2	0	0	[illegible]	[illegible]	[illegible]	[illegible]	[illegible]	[illegible]	[illegible]	[illegible]	[illegible]	[illegible]	[illegible]	[illegible]	[illegible]	[illegible]	[illegible]	[illegible]	[illegible]	[illegible]	[illegible]	[illegible]	[illegible]	[illegible]	[illegible]	[illegible]	[illegible]	[illegible]	[illegible]
HIP_4118	GERGFFYTP	REHIAKRAM	GERGFFYTPREHIAKRAM		SCG2	13232/334890	0.998	4118	0.862	0.002345	-2.4667	0.1667	3	18	-0.9111	2	3	0	0	[illegible]	[illegible]	[illegible]	[illegible]	[illegible]	[illegible]	[illegible]	[illegible]	[illegible]	[illegible]	[illegible]	[illegible]	[illegible]	[illegible]	[illegible]	[illegible]	[illegible]	[illegible]	[illegible]	[illegible]	[illegible]	[illegible]	[illegible]	[illegible]	[illegible]	[illegible]	[illegible]
HIP_4119	AGSLQPLAL	WEKKPFSED	AGSLQPLALWEKKPFSED		SCG1	8693/367830	0.998	4119	0.862	0.191922	0.1833	0	-1	18	-0.5611	-1	1	0	0	[illegible]	[illegible]	[illegible]	[illegible]	[illegible]	[illegible]	[illegible]	[illegible]	[illegible]	[illegible]	[illegible]	[illegible]	[illegible]	[illegible]	[illegible]	[illegible]	[illegible]	[illegible]	[illegible]	[illegible]	[illegible]	[illegible]	[illegible]	[illegible]	[illegible]	[illegible]	[illegible]
HIP_4120	CGERGFFY	PQLRRGWRP	CGERGFFYPQLRRGWRP		CMGA	5643/247050	0.998	4120	0.862	0.190664	0.5	0.1667	3	17	-1.1588	3	3	1	0	[illegible]	[illegible]	[illegible]	[illegible]	[illegible]	[illegible]	[illegible]	[illegible]	[illegible]	[illegible]	[illegible]	[illegible]	[illegible]	[illegible]	[illegible]	[illegible]	[illegible]	[illegible]	[illegible]	[illegible]	[illegible]	[illegible]	[illegible]	[illegible]	[illegible]	[illegible]	[illegible]
[illegible]	[illegible]	[illegible]	[illegible]		[illegible]	[illegible]	[illegible]	[illegible]	[illegible]	[illegible]	[illegible]	[illegible]	[illegible]	[illegible]	[illegible]	[illegible]	[illegible]	[illegible]	[illegible]	[illegible]	[illegible]	[illegible]	[illegible]	[illegible]	[illegible]	[illegible]	[illegible]	[illegible]	[illegible]	[illegible]	[illegible]	[illegible]	[illegible]	[illegible]	[illegible]	[illegible]	[illegible]	[illegible]	[illegible]	[illegible]	[illegible]	[illegible]	[illegible]	[illegible]	[illegible]	[illegible]

HIP_4589	LVCGERGFF	SEVDKRRTR	LVCGERGFFSEVDKRRTR		SCG1	4194/367830	0.999	4589	0.861	0.067198	0.85	0.3333	2	18	-0.8	2	2	0	0
HIP_4590	AGSLQPLAL	RSRSNQLPK	AGSLQPLALRSRSNQLPK		SCG2	12380/334890	0.998	4590	0.861	0.225631	-0.0667	0.1667	3	18	-0.5889	3	0	0	0
HIP_4591	TSICSLYQL	GKGRHHRGR	TSICSLYQLGKGRHHRGR		SCG1	5410/367830	0.999	4591	0.861	0.219977	-0.95	0.3333	6	18	-0.9722	4	1	0	0
HIP_4592	LCGSHLVEA	ALRHYINLI	LCGSHLVEAALRHYINLI		NPY	1708/49410	0.990	4592	0.861	0.041067	0.6	0	2	18	0.7833	0	0	0	0
HIP_4593	ERGFFYTPK	TPQSLATLE	ERGFFYTPKTPQSLATLE		SCG2	782/334890	1.000	4593	0.861	-0.105615	-2	0.3333	0	18	-0.65	0	2	0	0
HIP_4594	ERGFFYTPK	KTSYFPNPY	ERGFFYTPKKTSYFPNPY		SCG2	9853/334890	0.999	4594	0.861	-0.055421	-1.9333	0.3333	2	18	-1.2333	2	3	0	0
HIP_4595	VCGERGFFY	DPLQWKSSH	VCGERGFFYDPLQWKSSH		SCG1	600/367830	1.000	4595	0.861	0.154864	0.5	0.1667	1	18	-0.6778	0	3	0	0
HIP_4596	VCGERGFFY	NPFKRTNE	VCGERGFFYNPFKRTNE		SCG2	3128/334890	1.000	4596	0.861	0.146417	0.3333	0.1667	1	17	-0.9529	1	4	1	0
HIP_4597	LVEALYLVC	STHYRASEE	LVEALYLVCSTHYRASEE		SCG1	1366/367830	1.000	4597	0.861	0.140980	0.9667	0.1667	-1	18	0.1556	-2	2	0	0
HIP_4598	CGERGFFYT	AENEPQSAP	CGERGFFYTAENEPQSAP		SCG2	3477/334890	1.000	4598	0.861	0.042288	-0.7333	0	-2	18	-0.95	-2	3	0	0
HIP_4599	LCGSHLVEA	YLARMLVKY	LCGSHLVEAYLARMLVKY		SCG2	6380/334890	0.999	4599	0.861	0.063811	1.1333	0	2	18	0.7056	1	1	0	0
HIP_4600	TSICSLYQL	AASFQRNQL	TSICSLYQLAASFQRNQL		SCG2	542/334890	1.000	4600	0.861	-0.051619	0.3	0.1667	1	18	0.1056	1	2	0	0
HIP_4601	GGPGAGSLQ	YNQEKVLPR	GGPGAGSLQYNQEKVLPR		SCG2	11269/334890	0.999	4601	0.861	-0.040334	-1.4667	0.1667	1	18	-0.8722	1	1	0	0
HIP_4602	GAGSLQPLA	YGKRSSPET	GAGSLQPLAYGKRSSPET		NPY	376/49410	0.998	4602	0.861	0.064049	-0.2667	0.3333	1	18	-0.7222	1	1	0	0
HIP_4603	CGERGFFYT	PNPYNQEKV	CGERGFFYTPNPYNQEKV		SCG2	290/334890	1.000	4603	0.861	0.132762	-0.9833	0.3333	0	18	-1.1611	0	4	0	0
HIP_4604	SHLVEALYL	MDKARRHPQ	SHLVEALYLMDKARRHPQ		SCG1	15485/367830	0.994	4604	0.861	0.215095	0.1333	0	3	18	-0.6889	1	1	0	0
HIP_4605	ERGFFYTPK	EEERGLEPG	ERGFFYTPKEEERGLEPG		SCG1	5582/367830	0.999	4605	0.861	-0.050464	-2.7833	0.1667	-2	18	-1.5222	-2	2	0	1
HIP_4606	HLVEALYLV	RESTENVPR	HLVEALYLVRESTENVPR		NPY	462/49410	0.998	4606	0.861	0.231096	-0.35	0.1667	0	18	-0.2667	-1	1	0	0
HIP_4607	SHLVEALYL	KMDQLAKEL	SHLVEALYLKMDQLAKEL		CMGA	7221/247050	0.997	4607	0.861	0.214843	0.1333	0	0	18	0.0889	-1	1	0	0
HIP_4608	AGSLQPLAL	RYGKRSSP	AGSLQPLALRYGKRSSP		NPY	1613/49410	0.990	4608	0.861	0.242976	0.5333	0.1667	3	17	-0.5353	3	1	1	0
HIP_4609	LVEALYLVC	NAFLNERKQ	LVEALYLVCNAFLNERKQ		SCG1	1012/367830	1.000	4609	0.861	0.053620	1.9333	0	0	18	0.2944	0	2	0	0
HIP_4610	LVEALYLVC	TAEKRKRL	LVEALYLVCTAEKRKRL		SCG1	5830/367830	0.999	4610	0.861	0.167292	1.35	0	2	17	0.2294	2	1	1	0
HIP_4611	VCGERGFFY	EWEDSKRW	VCGERGFFYEWEDSKRW		CMGA	2801/247050	0.999	4611	0.861	0.111821	-0.6	0	-1	17	-1.1353	-1	4	1	0
HIP_4612	SHLVEALYL	WDEEDKRN	SHLVEALYLWDEEDKRN		SCG1	2232/367830	1.000	4612	0.861	0.120127	-0.2667	0	-2	17	-1.0706	-3	2	1	0
HIP_4613	SHLVEALYL	RNYLNYGEE	SHLVEALYLRNYLNYGEE		SCG1	9528/367830	0.998	4613	0.861	0.143401	-0.5	0	-1	18	-0.5056	-2	2	0	0
HIP_4614	CGERGFFYT	KPNGSVEPE	CGERGFFYTKPNGSVEPE		SCG2	5267/334890	1.000	4614	0.861	0.138608	-1.3667	0.1667	-1	18	-0.9611	-1	3	0	0
HIP_4615	GSHLVEALY	RGWRPSSRE	GSHLVEALYRGWRPSSRE		CMGA	3691/247050	0.999	4615	0.861	0.174920	-0.25	0.1667	2	18	-0.95	1	2	0	0
HIP_4616	SHLVEALYL	KEELVARSE	SHLVEALYLKEELVARSE		SCG1	3647/367830	0.999	4616	0.861	0.201067	-0.7667	0	-1	18	-0.0722	-2	1	0	0
HIP_4617	HLVEALYLV	YETQQWPER	HLVEALYLVYETQQWPER		SCG2	1493/334890	1.000	4617	0.861	0.121432	0.2	0	-1	18	-0.5222	-2	2	0	0
HIP_4618	SHLVEALYL	KNEQINDEM	SHLVEALYLKNEQINDEM		SCG2	4104/334890	1.000	4618	0.861	0.228130	-0.7667	0	-2	18	-0.55	-3	1	0	0
HIP_4619	SHLVEALYL	QSAPKENK	SHLVEALYLQSAPKENK		SCG2	5370/334890	0.999	4619	0.861	0.237864	0.6333	0.1667	1	17	-0.6059	0	1	1	0
HIP_4620	VCGERGFFY	PYGAGRSRS	VCGERGFFYPYGAGRSRS		SCG2	53/334890	1.000	4620	0.861	0.162503	0.1667	0.3333	2	18	-0.5722	2	4	0	0
HIP_4621	CGERGFFYT	ESEESNVSM	CGERGFFYTESEESNVSM		SCG1	689/367830	1.000	4621	0.861	0.115328	-1.1667	0.1667	-3	18	-0.7222	-3	3	0	0
HIP_4622	TSICSLYQL	GQNGERATR	TSICSLYQLGQNGERATR		SCG2	236/334890	1.000	4622	0.861	0.205608	-1.4	0.1667	1	18	-0.65	1	1	0	0
HIP_4623	CGERGFFYT	NRDSSMKLS	CGERGFFYTNRDSSMKLS		CMGA	8754/247050	0.996	4623	0.861	0.159851	-1.7833	0	1	18	-0.8222	1	3	0	0
HIP_4624	HLVEALYL	NNQKRERMD	HLVEALYLNNQKRERMD		SCG2	4946/334890	1.000	4624	0.861	0.226736	-0.7	0	1	17	-1.1235	0	1	1	0
HIP_4625	TSICSLYQ	SEEYRAPRP	TSICSLYQSEEYRAPRP		SCG1	7375/367830	0.999	4625	0.861	0.173356	-1.4667	0.1667	0	17	-0.9294	0	2	1	0
HIP_4626	GERGFFYTP	SEVDKRRTR	GERGFFYTPSEVDKRRTR		SCG1	5710/367830	0.999	4626	0.861	-0.069791	-0.6167	0.3333	2	18	-1.5833	2	3	0	0
HIP_4627	AGSLQPLAL	SGYPKTPGR	AGSLQPLALSGYPKTPGR		SCG2	270/334890	1.000	4627	0.861	0.239188	1.15	0.3333	2	18	-0.3611	2	1	0	0
HIP_4628	CGERGFFY	QSAPKENK	CGERGFFYQSAPKENK		SCG2	5231/334890	1.000	4628	0.861	0.171335	0.3	0.1667	1	16	-1.3062	1	3	0	0
HIP_4629	VCGERGFFY	GRGGEPRAY	VCGERGFFYGRGGEPRAY		SCG1	1262/367830	1.000	4629	0.861	0.212677	-0.1667	0.3333	1	18	-0.7	1	3	0	0
HIP_4630	CGERGFFYT	GHSQEKTHS	CGERGFFYTGHSQEKTHS		SCG1	2347/367830	1.000	4630	0.861	0.126411	-0.6	0.3333	2	18	-1.2611	0	3	0	0
HIP_4631	CGERGFFYT	EEPSSKDVM	CGERGFFYTEEPSSKDVM		CMGA	338/247050	1.000	4631	0.861	0.113967	-1.3	0.1667	-2	18	-0.7889	-2	3	0	0

HIP_S100	HLVEALYLV	NPYNQEKVL	HLVEALYLVNPYNQEKVL		SCG2	659/334890	1.000	S100	0.860	0.145814	0.05	0.1667	0	18	0.0444
HIP_S101	VCGERGFFY	EPQWSLYPS	VCGERGFFYEPQWSLYPS		SCG1	117/367830	1.000	S101	0.860	0.180388	-0.7167	0.1667	-1	18	-0.4444
HIP_S102	LVCGERGFF	RVPKLDLKR	LVCGERGFFRVPKLDLKR		SCG1	8800/367830	0.998	S102	0.860	0.082972	0.55	0.3333	3	18	-0.1556
HIP_S103	CTSICSLYQ	AEAASFQRN	CTSICSLYQAEAASFQRN		SCG2	6553/334890	0.999	S103	0.860	-0.061362	-0.15	0	0	18	-0.0778
HIP_S104	SHLVEALYL	KPDNPGEDA	SHLVEALYLKPDNPGEDA		NPY	115/49410	1.000	S104	0.860	0.228599	-0.45	0.1667	-2	18	-0.6167
HIP_S105	HLVEALYLV	WDEEDKRNY	HLVEALYLVWDEEDKRNY		SCG1	350/367830	1.000	S105	0.860	0.095233	-0.2	0	-2	18	-0.8056
HIP_S106	ERGFFYTPK	NYQKGERGE	ERGFFYTPKNYQKGERGE		SCG1	7133/367830	0.999	S106	0.860	-0.010024	-2.4167	0.1667	1	18	-1.9333
HIP_S107	VCGERGFFY	KLDLKRQY	VCGERGFFYKLDLKRQY		SCG1	12862/367830	0.996	S107	0.860	0.153315	0.1167	0	2	17	-0.6353
HIP_S108	VCGERGFFY	ESQTQEEVR	VCGERGFFYESQTQEEVR		SCG2	590/334890	1.000	S108	0.860	0.182625	-0.5833	0.1667	-2	18	-0.95
HIP_S109	AGSLQPLAL	SRSNQLPKA	AGSLQPLALSRSNQLPKA		SCG2	5507/334890	0.999	S109	0.860	0.253402	0.55	0.3333	2	18	-0.2389
HIP_S110	ICSLYQLEN	WERYRGRGS	ICSLYQLENWERYRGRGS		SCG1	20663/367830	0.988	S110	0.860	0.067478	-2.0167	0	1	18	-1.0444
HIP_S111	AGSLQPLAL	LDQLLHYRK	AGSLQPLALLDQLLHYRK		SCG1	1698/367830	1.000	S111	0.860	0.129141	1.0333	0	2	18	0.0111
HIP_S112	VCGERGFFY	RILEALRQ	VCGERGFFYRILEALRQ		SCG2	3231/334890	1.000	S112	0.860	0.021278	1.4667	0	1	18	0.2556
HIP_S113	CSLYQLENY	EEEEEATNT	CSLYQLENYEEEEEATNT		CMGA	2004/247050	0.999	S113	0.860	-0.041242	-3.1333	0	-6	18	-1.3556
HIP_S114	VCGERGFFY	LVDREKGLS	VCGERGFFYLVDREKGLS		CMGA	1048/247050	1.000	S114	0.860	0.008725	1.4667	0	0	18	-0.1444
HIP_S115	GSHLVEALY	LFQNRMLSK	GSHLVEALYLFQNRMLSK		SCG2	2344/334890	1.000	S115	0.860	0.010693	1.2333	0	2	18	0.0278
HIP_S116	VCGERGFFY	KGHPQEESE	VCGERGFFYKGHPQEESE		SCG1	5042/367830	0.999	S116	0.860	0.158696	-0.5333	0.1667	-1	18	-1.2056
HIP_S117	ERGFFYTPK	QKEPDLRLE	ERGFFYTPKQKEPDLRLE		SCG2	12959/334890	0.998	S117	0.860	-0.031461	-2.85	0.1667	0	18	-1.4833
HIP_S118	AGSLQPLAL	KFEVRLLR	AGSLQPLALKFEVRLLR		SCG1	13107/367830	0.996	S118	0.860	0.201747	0.8	0	2	17	0.4059
HIP_S119	VCGERGFFY	IQEEDLRKE	VCGERGFFYIQEEDLRKE		SCG2	313/334890	1.000	S119	0.860	0.092094	0.3	0	-2	18	-0.8556
HIP_S120	HLVEALYLV	TEKWAEGGG	HLVEALYLVTEKWAEGGG		SCG1	4467/367830	0.999	S120	0.860	0.188578	-0.2333	0	-1	18	0.0944
HIP_S121	SHLVEALYL	KNFFPEYNY	SHLVEALYLKNFFPEYNY		SCG1	8997/367830	0.998	S121	0.860	0.144666	0.2833	0	0	18	-0.2444
HIP_S122	AGSLQPLAL	TNVGSNTY	AGSLQPLALTNVGSNTY		IAPP	114/45018	1.000	S122	0.860	0.187052	1.5667	0	0	17	0.1176
HIP_S123	CGERGFFY	QELGEYLAR	CGERGFFYQELGEYLAR		SCG2	5344/334890	0.999	S123	0.860	0.143139	0.1833	0	-1	17	-0.5471
HIP_S124	GERGFFYTP	HSQEKTHSR	GERGFFYTPHSQEKTHSR		SCG1	11397/367830	0.997	S124	0.860	-0.007532	-1.85	0.3333	3	18	-1.7167
HIP_S125	GSHLVEALY	ETQQWPERK	GSHLVEALYETQQWPERK		SCG2	1070/334890	1.000	S125	0.860	0.182030	-0.5667	0	0	18	-1.1778
HIP_S126	CGERGFFYT	SNNFGAILS	CGERGFFYTSNNFGAILS		IAPP	450/45018	0.999	S126	0.860	0.123340	-1.1667	0.1667	0	18	0.0667
HIP_S127	LVCGERGFF	EQEHSQQKE	LVCGERGFFEQEHSQQKE		CMGA	1160/247050	1.000	S127	0.860	0.064671	-0.8833	0.1667	-1	18	-1.2
HIP_S128	VCGERGFFY	DLELQKIAE	VCGERGFFYDLELQKIAE		SCG1	411/367830	1.000	S128	0.860	0.091371	0.1833	0	-2	18	-0.1
HIP_S129	CGERGFFY	EERGLEPGK	CGERGFFYEERGLEPGK		SCG1	6674/367830	0.999	S129	0.860	0.182406	-1.2	0	-1	17	-1.1471
HIP_S130	HLCGSHLVE	WGYEKRNLA	HLCGSHLVEWGYEKRNLA		SCG1	7845/367830	0.998	S130	0.860	0.056260	0.3167	0.1667	2	18	-0.5111
HIP_S131	SHLVEALYL	KEIRKGESR	SHLVEALYLKEIRKGESR		CMGA	11425/247050	0.993	S131	0.860	0.225974	0.5667	0	2	18	-0.6611
HIP_S132	TSICSLYQ	VALNHLKAT	TSICSLYQVALNHLKAT		IAPP	145/45018	1.000	S132	0.860	0.023843	1.4667	0	2	17	0.4588
HIP_S133	AGSLQPLAL	PNNQKRERM	AGSLQPLALPNNQKRERM		SCG2	1844/334890	1.000	S133	0.860	0.237858	0.1333	0.1667	2	18	-0.9944
HIP_S134	ERGFFYTPK	NEKNFFPEY	ERGFFYTPKNEKNFFPEY		SCG1	12590/367830	0.996	S134	0.860	-0.033181	-2.85	0.1667	0	18	-1.4167
HIP_S135	HLVEALYLV	KFPSPEMIR	HLVEALYLVKFPSPEMIR		SCG2	3857/334890	1.000	S135	0.860	0.167962	0.6667	0.1667	1	18	0.3833
HIP_S136	TSICSLYQL	PYQGVSVPL	TSICSLYQLPYQGVSVPL		SCG2	3309/334890	1.000	S136	0.859	0.087040	-1.2333	0.1667	0	18	0.5833
HIP_S137	PLALEGSLQ	NYDWWEKKP	PLALEGSLQNYDWWEKKP		SCG1	6506/367830	0.999	S137	0.859	-0.020211	-1.4667	0.1667	-1	18	-1.0889
HIP_S138	CTSICSLYQ	WAEGGGHSR	CTSICSLYQWAEGGGHSR		SCG1	9365/367830	0.998	S138	0.859	-0.006180	-0.6	0	1	18	-0.3389
HIP_S139	VCGERGFFY	FQDKQYSS	VCGERGFFYFQDKQYSS		SCG1	6231/367830	0.999	S139	0.859	0.120052	0.0167	0	0	17	-0.7235
HIP_S140	VCGERGFFY	KMAHGYGEE	VCGERGFFYKMAHGYGEE		SCG1	8138/367830	0.998	S140	0.859	0.070726	0.6833	0	0	18	-0.5722
HIP_S141	GIVEQCCTS	PNNQKRERM	GIVEQCCTSPNNQKRERM		SCG2	3814/334890	1.000	S141	0.859	0.020355	-1.2667	0.3333	1	18	-1.2111
HIP_S142	AGSLQPLAL	SKSGYPKTP	AGSLQPLALSKSGYPKTP		SCG2	5372/334890	0.999	S142	0.859	0.253994	0.65	0.3333	2	18	-0.35

HIP_5611	CTSICSLYQ	SFRARAYGF	CTSICSLYQSFRARAYGF		CMGA	9099/247050	0.996	5611	0.858	0.072754	-0.5833	0.1667	2	18	0.2167	2	2	0	0	0.000000	-0.5	1	0.0667	1.075000	0.1111	0.9639	0	0	0	0	-0.5	-0.5623	-1	0	1	0	0	0.4103	1.0755	0.849	0.1667	0.1683	0.7208	-0.2393	0.0044	-0.0333
HIP_5612	CTSICSLYQ	DREKGLSAE	CTSICSLYQDREKGLSAE		CMGA	8007/247050	0.997	5612	0.858	0.155456	-2.0833	0	-1	18	-0.4611	-1	1	0	0	0.000000	-3.5	-1	0.1	0.625000	0.4444	0.1806	0	0	0	0	3.5	-0.3762	-3.5	0	1	0	0	0.4487	1.1641	0.8235	-0.3333	0.2013	0.6781	-0.9348	0.01	-0.35
HIP_5613	LVEALYLVC	EWEDSKRWS	LVEALYLVCEWEDSKRWS		CMGA	287/247050	1.000	5613	0.858	0.092931	0.4333	0	-2	18	-0.1667	-2	2	0	0	0.000000	-6.1333	-2	0.1	2.000000	0.3333	1.6667	0	0	0	0	12.2667	0.7222	-12.2667	0	1	0	0	0.4103	1.2372	0.7156	0.1667	0.1683	0.5121	0.1778	0.01	-0.6133
HIP_5614	ERGFFYTPK	ENQMDKARR	ERGFFYTPKENQMDKARR		SCG1	13329/367830	0.996	5614	0.858	-0.033837	-2.7833	0.1667	2	18	-1.8611	2	2	0	0	0.000000	-1.4333	-2	-0.0167	1.175000	0.2222	0.9528	1	0	1	0	2.8667	-2.6519	-2.8667	0	0	1	0	0.5885	0.9519	1.1112	-0.3333	0.3463	1.2347	-1.6379	0.0003	0.0239
HIP_5615	VCGERGFFY	KPFSEDVNW	VCGERGFFYKPFSEDVNW		SCG1	3799/367830	0.999	5615	0.858	0.130879	0.2667	0.1667	-1	18	-0.4722	-1	4	0	0	0.000000	-2.3333	1	0.1167	0.700000	0.2222	0.4778	1	0	1	0	-2.3333	0.1274	-9.3333	0	1	0	0	0.3603	1.0355	0.8556	0.3333	0.1298	0.7321	0.0961	0.0136	-0.2722
HIP_5616	VCGERGFFY	SEEKHLEEP	VCGERGFFYSEEKHLEEP		SCG1	86/367830	1.000	5616	0.858	0.166458	-0.5833	0.1667	-2	18	-0.9722	-3	3	0	0	0.000000	-4.0333	-2	0.1667	0.700000	0.4444	0.2556	0	0	0	0	8.0667	-0.1491	-12.1	0	1	0	0	0.4385	1.2069	0.7978	0	0.1922	0.6365	-0.2558	0.0278	-0.6722
HIP_5617	LVCGERGFF	SSKDVMEKR	LVCGERGFFSSKDVMEKR		CMGA	4569/247050	0.999	5617	0.858	0.153950	-0.05	0.5	1	18	-0.4167	1	2	0	0	0.000000	-3.5667	1	0.1	0.700000	0.4444	0.2556	0	1	0	1	-3.5667	-0.0128	-7.1333	1	0	0	0	0.4551	0.9203	1.0012	0.1667	0.2071	1.0023	-0.0228	0.01	-0.3567
HIP_5618	GSHLVEALY	SNTYGKRNA	GSHLVEALYSNTYGKRNA		IAPP	314/45018	0.999	5618	0.858	0.206288	-0.1167	0.1667	2	18	-0.6889	1	2	0	0	0.000000	-3.1	0	0.1667	2.000000	0.1111	1.8889	0	0	0	0	0	-0.2204	-6.2	0	1	0	0	0.4385	1.0119	0.9645	0.3333	0.1922	0.9302	-0.0512	0.0278	-0.5167
HIP_5619	CGERGFFY	GYPKTPGR	CGERGFFYGYPKTPGR		SCG2	1740/334890	1.000	5619	0.858	0.172263	0.1667	0.3333	2	16	-1.025	2	4	0	0	0.000000	-2.5333	0	0.15	0.700000	0.2222	0.4778	1	1	1	1	0	0.0796	-10.1333	0	1	0	0	0.4436	0.9188	1.0056	0.3333	0.1968	1.0113	0.0739	0.0225	-0.38
HIP_5620	SHLVEALYL	NFLVHSSNN	SHLVEALYLNFLVHSSNN		IAPP	96/45018	1.000	5620	0.858	0.055900	1.5667	0	1	18	0.2278	-1	2	0	0	0.000000	-1.0667	0	0.05	1.050000	0.1111	0.9389	0	0	0	0	0	1.4709	-2.1333	1	0	0	0	0.3051	1.1319	0.7612	0.6667	0.0931	0.5794	0.478	0.0025	-0.0533
HIP_5621	GSLQPLAL	SYFPNPYNQ	GSLQPLALSYFPNPYNQ		SCG2	4035/334890	1.000	5621	0.858	0.112924	1.6833	0.1667	0	17	-0.4353	0	2	1	0	0.000000	-2.9	0	0.0667	0.950000	0	0.95	0	0	0	0	0	1.5992	-5.8	1	0	0	0	0.3051	1.1786	0.7278	0.6667	0.0931	0.5297	0.5136	0.0044	-0.1933
HIP_5622	CGERGFFY	NHNEGMVTR	CGERGFFYNHNEGMVTR		SCG1	11028/367830	0.997	5622	0.858	0.163762	-0.9833	0	1	17	-0.8941	0	3	1	0	0.000000	-4.8333	0	0.1	0.000000	0.1111	-0.1111	0	0	0	0	0	0.1093	-14.5	0	1	0	0	0.4103	0.9638	1.0223	0.1667	0.1683	1.0451	-0.4034	0.01	-0.4833
HIP_5623	HLVEALYLV	ELQKIAEKF	HLVEALYLVELQKIAEKF		SCG1	1273/367830	1.000	5623	0.858	0.150204	0.5833	0	0	18	0.4556	-1	1	0	0	0.000000	-3.3	-1	0.1	1.400000	0.2222	1.1778	0	0	0	0	3.3	0.687	-3.3	1	0	0	0	0.3718	1.2169	0.6878	0.3333	0.1382	0.4731	0.2169	0.01	-0.33
HIP_5624	GSHLVEALY	WAEGGGHSR	GSHLVEALYWAEGGGHSR		SCG1	4199/367830	0.999	5624	0.858	0.042560	0.2833	0	1	18	-0.4389	-1	2	0	1	0.000000	-2.3	-1	0.05	2.000000	0.2222	1.7778	0	0	0	0	2.3	0.5037	-4.6	0	1	0	0	0.3436	1.2922	0.729	0.5	0.1181	0.5314	0.0974	0.0025	-0.115
HIP_5625	AGSLQPLAL	STENVPRTR	AGSLQPLALSTENVPRTR		NPY	130/49410	1.000	5625	0.858	0.233564	0.7333	0.1667	1	18	-0.3833	1	0	0	0	0.000000	-4.8	-1	0.1667	0.450000	0.1111	0.3389	0	0	0	0	4.8	0.2485	0	1	0	0	0	0.4	1.1983	0.76	0.3333	0.16	0.5776	0.2933	0.0278	-0.8
HIP_5626	VCGERGFFY	VKRVPGQGS	VCGERGFFYVKRVPGQGS		SCG2	12446/334890	0.998	5626	0.858	0.121551	0.0167	0	2	18	-0.25	2	3	0	0	0.000000	-2.8333	2	0.1	2.800000	0.3333	2.4667	0	0	0	0	-5.6667	0.0411	-8.5	0	1	0	0	0.3154	1.0874	0.7368	0.1667	0.0995	0.5429	0.0053	0.01	-0.2833
HIP_5627	VCGERGFFY	WKELDRNY	VCGERGFFYWKELDRNY		SCG1	10193/367830	0.998	5627	0.858	0.128447	-0.6667	0	0	17	-0.8882	0	4	1	0	0.000000	-4.2	0	0.1	1.650000	0.3333	1.3167	0	0	0	0	0	-0.8778	-16.8	0	1	0	0	0.3821	1.1624	0.7768	0.1667	0.146	0.6034	-0.2547	0.01	-0.42
HIP_5628	SICSLYQLE	GENYQKGER	SICSLYQLEGENYQKGER		SCG1	2775/367830	1.000	5628	0.858	-0.011279	-1.7667	0.1667	-1	18	-1.1	-1	2	0	0	0.000000	-1.4	0	0.0833	1.900000	0.2222	1.6778	0	1	0	1	0	-2.9641	-2.8	0	0	1	0	0.55	1.1	1.0033	-0.1667	0.3025	1.0067	-0.9717	0.0069	-0.1167
HIP_5629	CGERGFFYT	RDSSMKLSF	CGERGFFYTRDSSMKLSF		CMGA	8017/247050	0.997	5629	0.858	0.173414	-1.3333	0.1667	1	18	-0.4722	1	3	0	0	0.000000	-3.2	0	0.1167	0.000000	0.2222	-0.2222	0	0	0	0	0	0.2963	-9.6	0	1	0	0	0.4487	0.9824	0.9135	-0.1667	0.2013	0.8344	-0.5983	0.0136	-0.3733
HIP_5630	SHLVEALYL	KRNYPSLEL	SHLVEALYLKRNYPSLEL		SCG1	14460/367830	0.995	5630	0.858	0.207020	-0.9333	0	1	18	-0.1611	0	2	0	0	0.000000	-6.0667	2	0.15	0.000000	0.3333	-0.3333	0	0	0	0	-12.1333	0.3111	-12.1333	0	1	0	0	0.3821	1.0558	0.8801	0	0.146	0.7746	-0.3566	0.0225	-0.91
HIP_5631	AGSLQPLAL	DEMKRSGQL	AGSLQPLALDEMKRSGQL		SCG2	1907/334890	1.000	5631	0.858	0.127277	0.7167	0	0	18	-0.3167	0	0	0	0	0.000000	-4.8333	-2	0.1	0.450000	0.3333	0.1167	0	0	0	0	9.6667	0.0836	0	1	0	0	0	0.3051	1.2969	0.6512	0.3333	0.0931	0.424	0.2187	0.01	-0.4833
HIP_5632	VCGERGFFY	RFLGEGHHR	VCGERGFFYRFLGEGHHR		SCG1	11354/367830	0.997	5632	0.858	0.077858	1.0667	0	3	18	-0.6056	1	4	0	0	0.000000	-0.7333	1	0.05	0.700000	0.2222	0.4778	0	0	0	0	-0.7333	0.5096	-2.9333	0	1	0	0	0.2769	1.1572	0.6856	0.5	0.0767	0.4701	0.2954	0.0025	-0.0367
HIP_5633	SHLVEALYL	ENHPQESKG	SHLVEALYLENHPQESKG		SCG1	6711/367830	0.999	5633	0.858	0.172301	-0.65	0	0	18	-0.85	-2	1	0	0	0.000000	-5.5	-1	0.1	0.000000	0.2222	-0.2222	0	0	0	0	5.5	0.1444	-5.5	1	0	0	0	0.4103	1.1372	0.8523	0	0.1683	0.7264	-0.2667	0.01	-0.55
HIP_5634	CGERGFFYT	EELVARSET	CGERGFFYTEELVARSET		SCG1	3711/367830	0.999	5634	0.858	0.005923	-0.4	0	-2	18	-0.5222	-2	3	0	0	0.000000	-1.3333	-2	0.05	1.050000	0.2222	0.8278	0	0	0	0	2.6667	-0.3311	-4	0	1	0	0	0.4385	1.2286	0.7412	0	0.1922	0.5493	-0.1754	0.0025	-0.0667
HIP_5635	LYQLENYCN	EQEEERLSK	LYQLENYCNEQEEERLSK		CMGA	988/247050	1.000	5635	0.858	0.070315	-2.1333	0	-3	18	-1.6333	-3	1	0	1	0.000000	-2.7333	-2	0.1	0.625000	0.4444	0.1806	0	0	0	0	5.4667	-0.3852	-2.7333	0	1	0	0	0.5436	1.1405	0.9723	-0.3333	0.2955	0.9454	-1.1597	0.01	-0.2733
HIP_5636	LYQLENYCN	QEEERLSKE	LYQLENYCNQEEERLSKE		CMGA	989/247050	1.000	5636	0.858	0.070315	-2.1333	0	-3	18	-1.6333	-3	1	0	1	0.000000	-2.7333	-2	0.1	0.625000	0.4444	0.1806	0	0	0	0	5.4667	-0.3852	-2.7333	0	1	0	0	0.5436	1.1405	0.9723	-0.3333	0.2955	0.9454	-1.1597	0.01	-0.2733
HIP_5637	HLVEALYLV	YRGRGSEEY	HLVEALYLVYRGRGSEEY		SCG1	4833/367830	0.999	5637	0.858	0.175329	0.0833	0.1667	0	18	-0.3667	-1	2	0	0	0.000000	-4.3	1	0.1333	1.400000	0.2222	1.1778	0	1	0	1	-4.3	0.0981	-8.6	1	0	0	0	0.3987	0.9874	0.8701	0.1667	0.159	0.7571	0.0332	0.0178	-0.5733
HIP_5638	TSICSLYQL	QTQEEVRDS	TSICSLYQLQTQEEVRDS		SCG2	2875/334890	1.000	5638	0.858	0.159396	-1.45	0	-2	18	-0.6556	-2	1	0	0	0.000000	-2.2333	0	0.1	0.000000	0.1111	-0.1111	0	0	0	0	0	0.1611	-2.2333	1	0	0	0	0.5051	1.0803	0.8845	0.1667	0.2552	0.7823	-0.7324	0.01	-0.2233
HIP_5639	SHLVEALYL	HQVEKRKC	SHLVEALYLHQVEKRKC		IAPP	2405/45018	0.986	5639	0.858	0.161185	0.6333	0	3	17	-0.4235	1	1	1	0	0.000000	-2.9333	0	0.05	0.000000	0.2222	-0.2222	0	0	0	0	0	-0.1407	-2.9333	1	0	0	0	0.3436	1.1355	0.7156	0.3333	0.1181	0.5121	0.2176	0.0025	-0.1467
HIP_5640	GERGFFYTP	NKRLGLSGL	GERGFFYTPNKRLGLSGL		NPY	2165/49410	0.985	5640	0.858	0.027985	-2.5833	0.1667	2	18	-0.4944	2	3	0	0	0.000000	-2.7667	2	0.0333	1.650000	0.2222	1.4278	1	0	1	0	-5.5333	-3.6884	-8.3	0	0	0	1	0.5321	0.8558	1.1268	-0.3333	0.2831	1.2697	-1.3745	0.0011	-0.0922
HIP_5641	ERGFFYTPK	SQTQEEVRD	ERGFFYTPKSQTQEEVRD		SCG2	1309/334890	1.000	5641	0.858	-0.130431	-1.8667	0.3333	-1	18	-1.6444	-1	2	0	0	0.000000	0.4	-1	0	0.700000	0.1111	0.5889	1	0	1	0	-0.4	-1.0993	0.8	0	0	1	0	0.5885	0.8603	1.0878	-0.1667	0.3463	1.1834	-1.0985	0	0
HIP_5642	SHLVEALYL	KDQELGEYL	SHLVEALYLKDQELGEYL		SCG2	5495/334890	0.999	5642	0.858	0.229816	-0.7667	0	-2	18	-0.1889	-3	1	0	0	0.000000	-5.7333	0	0.15	0.000000	0.4444	-0.4444	0	0	0	0	0	0.3407	-5.7333	1	0	0	0	0.3821	1.1291	0.7835	0	0.146	0.6138	-0.2929	0.0225	-0.86
HIP_5643	VCGERGFFY	PERDSLSEE	VCGERGFFYPERDSLSEE		SCG2	1836/334890	1.000	5643	0.858	0.183702	-0.8833	0.1667	-3	18	-0.8722	-3	3	0	0	0.000000	-4.6333	0	0.1667	0.700000	0.4444	0.2556	1	0	1	0	0	-0.2257	-13.9	0	1	0	0	0.4269	1.0872	0.879	0	0.1823	0.7726	-0.3771	0.0278	-0.7722
HIP_5644	CGERGFFYT	YRKKSAEFP	CGERGFFYTYRKKSAEFP		SCG1	20075/367830	0.989	5644	0.858	0.112138	-1.4833	0	2	18	-0.9778	2	4	0	0	0.000000	-3.5	2	0.05	0.000000	0.3333	-0.3333	0	0	0	0	-7	0.4944	-14	0	1	0	0	0.4205	1.0194	0.8646	-0.1667	0.1768	0.7476	-0.6238	0.0025	-0.175
HIP_5645	VCGERGFFY	EKPLDSQSI	VCGERGFFYEKPLDSQSI		SCG2	2957/334890	1.000	5645	0.858	0.182640	-0.7833	0.1667	-1	18	-0.3944	-1	3	0	0	0.000000	-4.4333	0	0.1667	1.650000	0.3333	1.3167	1	0	1	0	0	-1.0314	-13.3	0	1	0	0	0.4269	1.0872	0.879	0	0.1823	0.7726	-0.3344	0.0278	-0.7389
HIP_5646	VCGERGFFY	APKENKPY	VCGERGFFYAPKENKPY		SCG2	7970/334890	0.999	5646	0.858	0.162420	0.1	0.1667	1	17	-0.9	1	3	1	0	0.000000	-2.6667	1	0.1167	1.150000	0.3333	0.8167	1	0	1	0	-2.6667	0.0817	-8	0	1	0	0	0.3603	1.0772	0.8656	0.3333	0.1298	0.7493	0.036	0.0136	-0.3111
HIP_5647	SHLVEALYL	KRVPGQGSS	SHLVEALYLKRVPGQGSS		SCG2	18259/334890	0.997	5647	0.858	0.201470	0.35	0	2	18	-0.1722	1	1	0	0	0.000000	-3.5	2	0.1	0.000000	0.3333	-0.3333	0	0	0	0	-7	-0.1167	-3.5	1	0	0	0	0.3154	1.1208	0.7035	0.1667	0.0995	0.4949	0.1104	0.01	-0.35
HIP_5648	SHLVEALYL	WQQQGDLQ	SHLVEALYLWQQQGDLQ		SCG1	6424/367830	0.999	5648	0.858	0.156345	-0.2667	0	-1	17	-0.3765	-2	2	1	1	1.000000	-4.7333	0	0.1	0.000000	0.1111	-0.1111	0	0	0	0	0	0.0296	-9.4667	1	0	0	0	0.4103	1.1438	0.7956	0.5	0.1683	0.633	-0.1094	0.01	-0.4733
HIP_5649	SHLVEALYL	HESSSRGEA	SHLVEALYLHESSSRGEA		SCG1	3595/367830	0.999	5649	0.858	0.195273	-0.2	0.1667	0	18	-0.3944	-2	1	0	1	0.000000	-4.6	-1	0.1167	0.000000	0.2222	-0.2222	0	0	0	0	4.6	0.0444	-4.6	1	0	0	0	0.4103	1.1588	0.7923	0	0.1683	0.6278	-0.0821	0.0136	-0.5367
HIP_5650	SHLVEALYL	QEEDLRKES	SHLVEALYLQEEDLRKES		SCG2	591/334890	1.000	5650	0.858	0.170980	-0.7	0	-2	18	-0.7944	-3	1	0	0	0.000000	-5.6	-2	0.15	0.000000	0.4444	-0.4444	0	0	0	0	11.2	0.3111	-5.6	1	0	0	0	0.4385	1.2919	0.7278	0	0.1922	0.5297	-0.3069	0.0225	-0.84
HIP_5651	TSICSLYQL	NENTKFEVR	TSICSLYQLNENTKFEVR		SCG1	7927/367830	0.998	5651	0.858	0.177008	-1.9167	0	0	18	-0.4778	0	1	0	0	0.000000	-3.1667	-1	0.1	0.000000	0.1111	-0.1111	0	0	0	0	3.1667	0.213	-3.1667	1	0	0	0	0.5051	1.0469	1.0412	0	0.2552	1.084	-0.9682	0.01	-0.3167
HIP_5652	GSHLVEALY	QENQMDKAR	GSHLVEALYQENQMDKAR		SCG1	2106/367830	1.000	5652	0.858	0.175532	-1.0333	0	0	18	-0.9889	-1	1	0	0	0.000000	-4.9333	-1	0.15	2.000000	0.2222	1.7778	0	0	0	0	4.9333	-1.837	-4.9333	0	1	0	0	0.4385	1.1769	0.8912	0.1667	0.1922	0.7942	-0.4531	0.0225	-0.74
HIP_5653	LVCGERGFF	PQESKGQP	LVCGERGFFPQESKGQP		SCG1	1457/367830	1.000	5653	0.858	0.103899	-0.5667	0.3333	0	17	-0.6765	0	2	1	0	0.000000	-4.6	-1	0.0833	0.700000	0.3333	0.3667	1	1	1	1	4.6	-0.2078	-9.2	1	0	0	0	0.5	1.015	0.99	0.1667	0.25	0.9801	-0.2833	0.0069	-0.3833
HIP_5654	TSICSLYQL	DREKGLSAE	TSICSLYQLDREKGLSAE		CMGA	8336/247050	0.996	5654	0.858	0.180853	-2.0833	0	-1	18	-0.3889	-1	1	0	0	0.000000	-3.5	-1	0.1	0.000000	0.4444	-0.4444	0	0	0	0	3.5	0.9259	-3.5	1	0	0	0	0.4487	1.1641	0.8235	-0.3333	0.2013	0.6781	-0.9348	0.01	-0.35
HIP_5655	AGSLQPLAL	NQVKRVPGQ	AGSLQPLALNQVKRVPGQ		SCG2	8657/334890	0.999	5655	0.858	0.211281	1.1	0	2	18	-0.2111	2	0	0	0	0.000000	-4.0667	0	0.1	0.450000	0.1111	0.3389	0	0	0	0	0	0.3728	0	1	0	0	0	0.3333	1.1483	0.7833	0.6667	0.1111	0.6136	0.3667	0.01	-0.4067
HIP_5656	SHLVEALYL	SHQVEKRKC	SHLVEALYLSHQVEKRKC		IAPP	1423/45018	0.994	5656	0.858	0.223578	-0.2	0.1667	3	18	-0.4444	1	1	0	0	0.000000	-4.6	0	0.1167	1.050000	0.1111	0.9389	0	0	0	0	0	-0.1878	-4.6	1	0	0	0	0.4103	1.0922	0.8323	0.1667	0.1683	0.6927	-0.0821	0.0136	-0.5367
HIP_5657	GERGFFYTP	SQECFETLR	GERGFFYTPSQECFETLR		CMGA	1473/247050	1.000	5657	0.858	-0.043206	-1.9	0.3333	-1	18	-0.7889	-1	3	0	0	0.000000	-1.4	-1	0.05	1.325000	0.1111	1.2139	1	0	1	0	1.4	-2.3064	-4.2	0	0	0	1	0.5885	0.9736	1.0512	-0.1667	0.3463	1.1049	-1.1181	0.0025	-0.07
HIP_5658	VCGERGFFY	QLQALRRG	VCGERGFFYQLQALRRG		CMGA	7104/247050	0.997	5658	0.858	0.153958	0.1833	0	2	17	-0.2824	2	3	1	0	0.000000	-2.5	0	0.1	1.150000	0.1111	1.0389	0	0	0	0	0	0.1905	-7.5	0	1	0	0	0.3718	1.1569	0.7612	0.5	0.1382	0.5794	0.0682	0.01	-0.25

[illegible]

HIP_6122	AGSLQPLAL	MKLSFRARA	AGSLQPLALMKLSFRARA		CMGA	9683/247050	0.995	6122	0.857	0.108543	1.8667	0	3	18	0.3944	3	0
HIP_6123	CGERGFFY	QETDKLAPV	CGERGFFYQETDKLAPV		SCG2	5057/334890	1.000	6123	0.857	0.193326	-0.5667	0	-1	17	-0.5235	-1	3
HIP_6124	AGSLQPLAL	RPRHHHGRS	AGSLQPLALRPRHHHGRS		SCG1	14771/367830	0.995	6124	0.857	0.219515	-0.2	0.1667	6	18	-0.9556	3	0
HIP_6125	VCGERGFFY	QPTLLLSLL	VCGERGFFYQPTLLLSLL		SCG1	521/367830	1.000	6125	0.857	0.176688	-0.25	0.1667	0	18	0.8111	0	3
HIP_6126	GSLQPLAL	ENKPYALNS	GSLQPLALENKPYALNS		SCG2	8284/334890	0.999	6126	0.857	0.236603	-0.25	0	0	17	-0.3294	0	1
HIP_6127	LVCGERGFF	VEKRKCNTA	LVCGERGFFVEKRKCNTA		IAPP	1352/45018	0.996	6127	0.857	0.058659	0.3333	0.1667	2	18	-0.2333	2	2
HIP_6128	AGSLQPLAL	REEARFQDK	AGSLQPLALREEARFQDK		SCG1	5369/367830	0.999	6128	0.857	0.202752	-0.35	0	0	18	-0.7556	0	0
HIP_6129	CGERGFFYT	EEPVSTHQE	CGERGFFYTEEPVSTHQE		SCG1	247/367830	1.000	6129	0.857	0.086649	-1.3	0.1667	-2	18	-1.0444	-3	3
HIP_6130	HLVEALYLV	PKNDDTPNR	HLVEALYLVPKNDDTPNR		SCG2	4039/334890	1.000	6130	0.857	0.215850	-0.3833	0.1667	0	18	-0.7056	-1	1
HIP_6131	VEALYLVC	WDEEDKRNY	VEALYLVCWDEEDKRNY		SCG1	1577/367830	1.000	6131	0.857	0.106370	0.4333	0	-3	17	-0.7412	-3	2
HIP_6132	VCGERGFFY	GQNGERAT	VCGERGFFYGQNGERAT		SCG2	1347/334890	1.000	6132	0.857	0.208111	-0.5167	0.1667	0	17	-0.7353	0	3
HIP_6133	LVEALYLVC	DLEWERYRG	LVEALYLVCDLEWERYRG		SCG1	162/367830	1.000	6133	0.857	0.098552	1.2167	0	-2	18	0.0556	-2	2
HIP_6134	GERGFFYTP	KENIEKNEQ	GERGFFYTPKENIEKNEQ		SCG2	8678/334890	0.999	6134	0.857	-0.022830	-2.4167	0.1667	-1	18	-1.7278	-1	3
HIP_6135	GERGFFYTP	SQENHPQES	GERGFFYTPSQENHPQES		SCG1	2401/367830	1.000	6135	0.857	-0.029718	-1.9	0.3333	-1	18	-1.7056	-2	3
HIP_6136	LVCGERGFF	TENENTKFE	LVCGERGFFTENENTKFE		SCG1	464/367830	1.000	6136	0.857	0.071191	-0.4167	0.1667	-2	18	-0.7056	-2	2
HIP_6137	CGERGFFYT	KHMQFPPMY	CGERGFFYTKHMQFPPMY		SCG2	17440/334890	0.997	6137	0.857	0.078599	-0.7333	0	2	18	-0.6222	1	3
HIP_6138	LVCGERGFF	EEDIQGPTK	LVCGERGFFEEDIQGPTK		SCG1	1004/367830	1.000	6138	0.857	0.037830	-0.8833	0.1667	-2	18	-0.4889	-2	2
HIP_6139	AGSLQPLAL	ELDKMAHGY	AGSLQPLALELDKMAHGY		SCG1	1133/367830	1.000	6139	0.857	0.124742	1.0333	0	0	18	0.0222	-1	0
HIP_6140	CTSICSLYQ	EEERLSKEW	CTSICSLYQEEERLSKEW		CMGA	2330/247050	0.999	6140	0.857	0.077924	-1.9167	0	-2	18	-0.7833	-2	1
HIP_6141	SHLVEALYL	YDPLQWKS	SHLVEALYLYDPLQWKS		SCG1	2794/367830	1.000	6141	0.857	0.157080	-0.0167	0.1667	0	17	-0.1824	-1	2
HIP_6142	GSHLVEALY	AYLKRLVNA	GSHLVEALYAYLKRLVNA		SCG2	3953/334890	1.000	6142	0.857	-0.004410	1.4333	0	2	18	0.3667	1	2
HIP_6143	GSLQPLAL	NYLNYGEEG	GSLQPLALNYLNYGEEG		SCG1	3040/367830	1.000	6143	0.857	0.076748	1.4	0	-2	17	-0.3941	-2	2
HIP_6144	AGSLQPLAL	DREKGLSAE	AGSLQPLALDREKGLSAE		CMGA	2777/247050	0.999	6144	0.857	0.205034	-0.35	0	-1	18	-0.3222	-1	0
HIP_6145	AGSLQPLAL	AYENLNDKD	AGSLQPLALAYENLNDKD		SCG2	1535/334890	1.000	6145	0.857	0.065505	1.0667	0	-2	18	-0.4667	-2	1
HIP_6146	SHLVEALYL	ALNSEKNFP	SHLVEALYLALNSEKNFP		SCG2	1761/334890	1.000	6146	0.857	0.067614	1.4	0	0	18	0.0111	-1	1
HIP_6147	GSHLVEALY	NRQYWDEDL	GSHLVEALYNRQYWDEDL		SCG2	5975/334890	0.999	6147	0.857	0.157274	-1.2	0	-2	18	-0.8889	-2	2
HIP_6148	VCGERGFFY	EEKIESQTQ	VCGERGFFYEEKIESQTQ		SCG2	4451/334890	1.000	6148	0.857	0.156339	-1.1	0	-2	18	-0.9	-2	3
HIP_6149	SHLVEALYL	KELENLAAM	SHLVEALYLKELENLAAM		SCG1	8814/367830	0.998	6149	0.857	0.188747	0.45	0	-1	18	0.4056	-2	1
HIP_6150	ERGFFYTPK	GEQEHSQQK	ERGFFYTPKGEQEHSQQK		CMGA	4003/247050	0.999	6150	0.857	-0.070631	-2.2667	0.3333	1	18	-2.0056	0	2
HIP_6151	HLVEALYLV	DWMRIILEA	HLVEALYLVDWMRIILEA		SCG2	1170/334890	1.000	6151	0.857	0.058372	0.7	0	-1	18	0.9833	-2	2
HIP_6152	CGERGFFY	THSREKSSQ	CGERGFFYTHSREKSSQ		SCG1	11857/367830	0.997	6152	0.857	0.183385	-0.0667	0.1667	2	17	-1.3941	1	3
HIP_6153	GSLQPLAL	KFEVRLLRD	GSLQPLALKFEVRLLRD		SCG1	12351/367830	0.997	6153	0.857	0.194001	0.8	0	1	17	0.0941	1	1
HIP_6154	LVCGERGFF	SQETDKLAP	LVCGERGFFSQETDKLAP		SCG2	667/334890	1.000	6154	0.857	0.100018	-0.4333	0.3333	-1	18	-0.2556	-1	2
HIP_6155	VCGERGFFY	GWQAKREE	VCGERGFFYGWQAKREE		CMGA	1341/247050	1.000	6155	0.857	0.149022	-0.0833	0.1667	0	17	-0.9529	0	4
HIP_6156	SHLVEALYL	TAEKRLEGQ	SHLVEALYLTAEKRLEGQ		CMGA	3998/247050	0.999	6156	0.857	0.184510	0.65	0	0	18	-0.3222	-1	1
HIP_6157	AGSLQPLAL	GYPKTPGRA	AGSLQPLALGYPKTPGRA		SCG2	229/334890	1.000	6157	0.857	0.232268	1.0167	0.3333	2	18	-0.2167	2	1
HIP_6158	LVCGERGFF	DRSSQGGSL	LVCGERGFFDRSSQGGSL		SCG1	7615/367830	0.999	6158	0.857	0.129346	-0.6	0.3333	0	18	-0.2	0	2
HIP_6159	HLVEALYLV	APKENKPYA	HLVEALYLVAPKENKPYA		SCG2	4503/334890	1.000	6159	0.857	0.178114	0.5	0.1667	1	18	-0.1167	0	1
HIP_6160	CGERGFFY	EDSSEEKHL	CGERGFFYEDSSEEKHL		SCG1	1232/367830	1.000	6160	0.857	0.178516	-0.5833	0.1667	-2	17	-1.2294	-3	3
HIP_6161	SHLVEALY	KFEVRLLRD	SHLVEALYKFEVRLLRD		SCG1	13957/367830	0.996	6161	0.857	0.182334	-0.05	0	1	17	-0.0294	0	2
HIP_6162	SHLVEALYL	VFQELGKLT	SHLVEALYLVFQELGKLT		SCG2	789/334890	1.000	6162	0.857	0.047946	1.6333	0	0	18	0.6222	-1	2
HIP_6163	CGERGFFY	EKDRADQTV	CGERGFFYEKDRADQTV		SCG1	7793/367830	0.998	6163	0.857	0.193626	-1.1	0	-1	17	-1.1235	-1	3
HIP_6164	HLVEALYLV	TENVPRTRL	HLVEALYLVTENVPRTRL		NPY	157/49410	0.999	6164	0.857	0.186502	-0.1667	0	1	18	0.1444	0	1
HIP_6165	DLQVGQVEL	DLFQNRMLS	DLQVGQVELDLFQNRMLS		SCG2	4257/334890	1.000	6165	0.857	0.000260	1.2667	0	-2	18	-0.1056	-2	1
HIP_6166	TSICSLYQL	YPKTPGRAG	TSICSLYQLYPKTPGRAG		SCG2	15472/334890	0.998	6166	0.857	0.132513	-1.3	0.1667	2	18	-0.2833	2	2
HIP_6167	VCGERGFF	GENYQKGER	VCGERGFFGENYQKGER		SCG1	592/367830	1.000	6167	0.857	0.115149	-0.3667	0.3333	0	17	-1.2353	0	3
HIP_6168	CSLYQLENY	SKEWEDSKR	CSLYQLENYSKEWEDSKR		CMGA	9793/247050	0.995	6168	0.857	0.008063	-2.75	0.1667	-1	18	-1.6167	-1	2
HIP_6169	CGERGFFYT	GLEPGKGRH	CGERGFFYTGLEPGKGRH		SCG1	3065/367830	1.000	6169	0.857	0.091614	0.1167	0.1667	2	18	-0.9333	1	3
HIP_6170	VCGERGFFY	HSQQKEEEE	VCGERGFFYHSQQKEEEE		CMGA	2355/247050	0.999	6170	0.857	0.131170	-0.5333	0.1667	-2	18	-1.4833	-3	3
HIP_6171	GSLQPLAL	ATQRLANF	GSLQPLALATQRLANF		IAPP	717/45018	0.997	6171	0.857	0.190640	1.1667	0	1	16	0.3062	1	0
HIP_6172	SHLVEALYL	QGLFRGGKS	SHLVEALYLQGLFRGGKS		CMGA	376/247050	1.000	6172	0.857	0.203705	1.0333	0.1667	2	18	0.0722	1	2
HIP_6173	CGERGFFYT	YDRVAQLDQ	CGERGFFYTYDRVAQLDQ		SCG1	11825/367830	0.997	6173	0.857	0.060332	-1.4167	0	-1	18	-0.7056	-1	4
HIP_6174	CGERGFFY	TTENENTKF	CGERGFFYTTENENTKF		SCG1	4968/367830	0.999	6174	0.857	0.191375	-0.1	0	-1	17	-1.1294	-1	3
HIP_6175	AGSLQPLAL	EEDWMRIL	AGSLQPLALEEDWMRIL		SCG2	889/334890	1.000	6175	0.857	0.161409	-0.1833	0	-2	18	0.4167	-2	1
HIP_6176	ERGFFYTPK	EEEEDNRDS	ERGFFYTPKEEEEDNRDS		CMGA	2031/247050	0.999	6176	0.857	-0.069307	-2.7833	0.1667	-4	18	-2.2278	-4	2
HIP_6177	SHLVEALYL	SFQRNQLL	SHLVEALYLSFQRNQLL		SCG2	6137/334890	0.999	6177	0.857	0.20097	0.8	0.1667	1	17	0.1882	0	2
[illegible]	[illegible]	[illegible]	[illegible]		[illegible]	[illegible]	[illegible]	[illegible]	[illegible]	[illegible]	[illegible]	[illegible]	[illegible]	[illegible]	[illegible]	[illegible]	[illegible]

HIP_6633	CGERGFFYT	AQDPEGKGE	CGERGFFYTAQDPEGKGE		CMGA	4164/247050	0.999	6633	0.856	0.042524	-0.7333	0	-2	18	-1.1778	-2	3	0	0
HIP_6634	CGERGFFYT	KLKHMQFPP	CGERGFFYTKLKHMQFPP		SCG2	20814/334890	0.996	6634	0.856	0.121576	-0.5333	0	3	18	-0.6611	2	3	0	0
HIP_6635	SHLVEALY	EDVNWGYEK	SHLVEALYEDVNWGYEK		SCG1	2585/367830	1.000	6635	0.856	0.126623	0.25	0	-2	17	-0.6765	-3	2	1	0
HIP_6636	LVCGERGFF	PSQKYPGPQ	LVCGERGFFPSQKYPGPQ		CMGA	820/247050	1.000	6636	0.856	0.133628	-0.1167	0.5	1	18	-0.6056	1	2	0	0
HIP_6637	LVCGERGFF	EEEATNTHP	LVCGERGFFEEEATNTHP		CMGA	616/247050	1.000	6637	0.856	0.043002	-0.8833	0.1667	-2	18	-0.6167	-3	2	0	1
HIP_6638	VCGERGFFY	AVEEPSSKD	VCGERGFFYAVEEPSSKD		CMGA	200/247050	1.000	6638	0.856	0.006424	1.1333	0	-2	18	-0.5222	-2	3	0	0
HIP_6639	QHLCGSHLV	AASFQRNQL	QHLCGSHLVAASFQRNQL		SCG2	4353/334890	1.000	6639	0.856	0.057985	1.2667	0.1667	3	18	-0.1333	1	1	0	0
HIP_6640	CGERGFFYT	RALEYIENL	CGERGFFYTRALEYIENL		SCG2	5107/334890	1.000	6640	0.856	-0.019384	0.3167	0	-1	18	-0.2833	-1	3	0	0
HIP_6641	VCGERGFFY	GPNNQKRE	VCGERGFFYGPNNQKRE		SCG2	483/334890	1.000	6641	0.856	0.203003	-0.2	0.3333	1	17	-1.3059	1	3	1	0
HIP_6642	GSHLVEALY	HSQEKTHSR	GSHLVEALYHSQEKTHSR		SCG1	10428/367830	0.998	6642	0.856	0.178921	-0.5333	0.1667	3	18	-1.0944	0	1	0	0
HIP_6643	VCGERGFFY	KDVKDKET	VCGERGFFYKDVKDKET		SCG1	12175/367830	0.997	6643	0.856	0.156719	0.1833	0	0	17	-0.9706	0	3	1	0
HIP_6644	VCGERGFFY	SVALNHLKA	VCGERGFFYSVALNHLKA		IAPP	76/45018	1.000	6644	0.856	0.023806	1.5833	0.1667	2	18	0.3444	1	3	0	0
HIP_6645	CSLYQLENY	EQAIKEHLN	CSLYQLENYEQAIKEHLN		SCG2	10408/334890	0.999	6645	0.856	-0.143903	-2.25	0	-1	18	-0.8222	-2	1	0	0
HIP_6646	VEALYLVCG	SEEYRAPRP	VEALYLVCGSEEYRAPRP		SCG1	538/367830	1.000	6646	0.856	0.078386	-0.25	0.3333	-1	18	-0.2444	-1	2	0	0
HIP_6647	HLVEALYL	FERRDNMND	HLVEALYLFERRDNMND		SCG1	9081/367830	0.998	6647	0.856	0.146329	0.1833	0	-1	17	-0.7294	-2	2	1	0
HIP_6648	SHLVEALYL	YQGVSVPLQ	SHLVEALYLYQGVSVPLQ		SCG2	465/334890	1.000	6648	0.856	0.177730	0.1833	0.1667	0	18	0.5389	-1	2	0	0
HIP_6649	HLVEALYLV	ELDKMAHGY	HLVEALYLVELDKMAHGY		SCG1	300/367830	1.000	6649	0.856	0.119740	0.5833	0	0	18	0.2944	-2	1	0	0
HIP_6650	GSHLVEALY	ERMDEEQKL	GSHLVEALYERMDEEQKL		SCG2	4208/334890	1.000	6650	0.856	0.111310	-0.3	0	-2	18	-0.8778	-3	1	0	0
HIP_6651	VCGERGFFY	ETQQWPER	VCGERGFFYETQQWPER		SCG2	4862/334890	1.000	6651	0.856	0.181207	-0.5667	0	-1	17	-1.1471	-1	3	1	0
HIP_6652	LVEALYLVC	KEIRKGESR	LVEALYLVCKEIRKGESR		CMGA	7453/247050	0.997	6652	0.856	0.180457	1.2667	0	1	18	-0.0667	1	1	0	0
HIP_6653	VCGERGFFY	TENVPRTR	VCGERGFFYTENVPRTR		NPY	859/49410	0.995	6653	0.856	0.177758	-0.5667	0	1	17	-0.7412	1	3	1	0
HIP_6654	CGERGFFY	STENVPRTR	CGERGFFYSTENVPRTR		NPY	701/49410	0.996	6654	0.856	0.201553	-0.1167	0.1667	1	17	-1.0353	1	3	1	0
HIP_6655	SHLVEALYL	HYRASEEEP	SHLVEALYLHYRASEEEP		SCG1	13450/367830	0.996	6655	0.856	0.095042	-0.45	0	-1	18	-0.6389	-3	2	0	1
HIP_6656	LVCGERGFF	MQESKAEGN	LVCGERGFFMQESKAEGN		CMGA	1265/247050	1.000	6656	0.856	0.023077	0.0167	0.1667	-1	18	-0.45	-1	2	0	0
HIP_6657	VEALYLVCG	EEYRAPRPQ	VEALYLVCGEEYRAPRPQ		SCG1	3801/367830	0.999	6657	0.856	0.028597	-0.3333	0.1667	-1	18	-0.3944	-1	2	0	0
HIP_6658	CGERGFFYT	EKSSQESGE	CGERGFFYTEKSSQESGE		SCG1	7950/367830	0.998	6658	0.856	0.144206	-1.2333	0.1667	-2	18	-1.3	-2	3	0	0
HIP_6659	ERGFFYTPK	DEKKELENL	ERGFFYTPKDEKKELENL		SCG1	13014/367830	0.996	6659	0.856	-0.032212	-2.85	0.1667	-1	18	-1.5556	-1	2	0	0
HIP_6660	VCGERGFFY	SSQAELKEA	VCGERGFFYSSQAELKEA		CMGA	420/247050	1.000	6660	0.856	0.196530	-0.1333	0.3333	-1	18	-0.3556	-1	3	0	0
HIP_6661	SHLVEALYL	NSEKNFPMD	SHLVEALYLNSEKNFPMD		SCG2	544/334890	1.000	6661	0.856	0.227763	-0.25	0.1667	-1	18	-0.3889	-2	1	0	0
HIP_6662	CTSICSLYQ	PRAYFMSDT	CTSICSLYQPRAYFMSDT		SCG1	6661/367830	0.999	6662	0.856	0.059117	-0.8833	0.1667	0	18	0.0167	0	2	0	0
HIP_6663	CTSICSLYQ	KKPFSEDVN	CTSICSLYQKKPFSEDVN		SCG1	12014/367830	0.997	6663	0.856	0.131273	-1.7333	0.1667	0	18	-0.4167	0	2	0	0
HIP_6664	VCGERGFFY	WRPSSREDS	VCGERGFFYWRPSSREDS		CMGA	5623/247050	0.998	6664	0.856	0.146106	-0.45	0.1667	0	18	-1.0389	0	4	0	0
HIP_6665	VCGERGFFY	NFLVHSSNN	VCGERGFFYNFLVHSSNN		IAPP	151/45018	1.000	6665	0.856	0.017539	1.2333	0	1	18	-0.1278	0	4	0	0
HIP_6666	HLVEALYL	NNFGAIL	HLVEALYLNNFGAIL		IAPP	704/45018	0.998	6666	0.856	0.174096	0.35	0	0	15	0.9933	-1	2	1	0
HIP_6667	AGSLQPLAL	YINLITRQR	AGSLQPLALYINLITRQR		NPY	1185/49410	0.994	6667	0.856	0.107293	1.5167	0	2	18	0.1944	2	1	0	0
HIP_6668	VCGERGFFY	ELTAEKRLE	VCGERGFFYELTAEKRLE		CMGA	1313/247050	1.000	6668	0.856	0.115909	0.65	0	-1	18	-0.4444	-1	3	0	0
HIP_6669	SHLVEALYL	KPLDSQSIY	SHLVEALYLKPLDSQSIY		SCG2	4764/334890	1.000	6669	0.856	0.206109	0.7667	0.1667	0	18	0.0833	-1	1	0	0
HIP_6670	VCGERGFFY	GWQAKREEE	VCGERGFFYGWQAKREEE		CMGA	5/247050	1.000	6670	0.856	0.136686	-0.0833	0.1667	-1	18	-1.0944	-1	4	0	1
HIP_6671	VCGERGFFY	EYNYDWWE	VCGERGFFYEYNYDWWE		SCG1	2995/367830	1.000	6671	0.856	0.099888	-0.6667	0	-3	17	-0.9529	-3	5	1	0
HIP_6672	VCGERGFFY	STNVGSNTY	VCGERGFFYSTNVGSNTY		IAPP	258/45018	0.999	6672	0.856	0.183783	-0.1167	0.1667	0	18	-0.2944	0	3	0	0
HIP_6673	VCGERGFFY	QDPEGKGEQ	VCGERGFFYQDPEGKGEQ		CMGA	547/247050	1.000	6673	0.856	0.178707	-0.7167	0.1667	-2	18	-1.2	-2	3	0	0
HIP_6674	GERGFFYTP	KTHSREKSS	GERGFFYTPKTHSREKSS		SCG1	18052/367830	0.992	6674	0.856	0.015054	-1.9	0.1667	3	18	-1.6056	2	3	0	0
HIP_6675	HLCGSHLVE	EEQYTPQSL	HLCGSHLVEEEQYTPQSL		SCG2	5810/334890	0.999	6675	0.856	0.125887	-1	0	-1	18	-0.6333	-3	1	0	1
HIP_6676	LVCGERGFF	SKVIAYLKR	LVCGERGFFSKVIAYLKR		SCG2	7713/334890	0.999	6676	0.856	0.061972	0.7833	0.3333	3	18	0.4	3	2	0	0
HIP_6677	LYQLENYCN	QNRMLSKSG	LYQLENYCNQNRMLSKSG		SCG2	15568/334890	0.998	6677	0.856	0.131624	-2.3	0	1	18	-1.0111	1	1	0	0
HIP_6678	HLVEALYLV	WQAKREEEE	HLVEALYLVWQAKREEEE		CMGA	196/247050	1.000	6678	0.856	0.047048	0.6833	0	-2	18	-0.6333	-3	2	0	2
HIP_6679	CGERGFFY	PSEEKGHPQ	CGERGFFYPSEEKGHPQ		SCG1	2093/367830	1.000	6679	0.856	0.206072	-0.2667	0.3333	0	17	-1.4118	-1	3	1	0
HIP_6680	CCTSICSLY	SSHFERRDN	CCTSICSLYSSHFERRDN		SCG1	3792/367830	0.999	6680	0.856	0.001385	-0.5167	0.5	1	18	-0.5167	0	2	0	0
HIP_6681	GSHLVEALY	AEKGREHIA	GSHLVEALYAEKGREHIA		SCG2	8534/334890	0.999	6681	0.856	0.129834	-0.2167	0	1	18	-0.3611	-1	1	0	0
HIP_6682	HLVEALYLV	GFRGPGPQL	HLVEALYLVGFRGPGPQL		CMGA	1809/247050	1.000	6682	0.856	0.187044	0.7667	0.1667	1	18	0.4333	0	2	0	0
HIP_6683	ERGFFYTPK	EPERELDLP	ERGFFYTPKEPERELDLP		SCG2	3623/334890	1.000	6683	0.856	-0.089980	-2.4667	0.3333	-2	18	-1.3556	-2	2	0	0
HIP_6684	VCGERGFFY	DNPFKRTNE	VCGERGFFYDNPFKRTNE		SCG2	1581/334890	1.000	6684	0.856	0.175328	-0.7167	0.1667	0	18	-1.0944	0	4	0	0
HIP_6685	VCGERGFFY	IEMLKTGEK	VCGERGFFYIEMLKTGEK		SCG2	1848/334890	1.000	6685	0.856	-0.000344	1.2	0	0	18	-0.1944	0	3	0	0

[illegible]

HIP_7144	ERGFFYTPK	NRQYWDEDL	ERGFFYTPKNRQYWDEDL		SCG2	10077/334890	0.999	7144	0.855	-0.029599	-2.95	0.1667	-1	18	-1.7056	-1	3	0	0
HIP_7145	CGERGFFYT	YQGVSVPLQ	CGERGFFYTYQGVSVPLQ		SCG2	3064/334890	1.000	7145	0.855	0.068636	-0.7333	0.1667	0	18	-0.0889	0	4	0	0
HIP_7146	LVCGERGFF	ERYRGRGSE	LVCGERGFFERYRGRGSE		SCG1	13555/367830	0.996	7146	0.855	0.075070	-0.6833	0.1667	1	18	-0.8944	1	3	0	0
HIP_7147	GSHLVEALY	PKNDDTPNR	GSHLVEALYPKNDDTPNR		SCG2	4155/334890	1.000	7147	0.855	0.196661	-0.7833	0.1667	0	18	-1.2167	-1	1	0	0
HIP_7148	CGERGFFY	KSSHFERRD	CGERGFFYKSSHFERRD		SCG1	7902/367830	0.998	7148	0.855	0.216788	-0.2	0.3333	2	17	-1.4059	1	4	1	0
HIP_7149	ERGFFYTPK	TPNRQYWDE	ERGFFYTPKTPNRQYWDE		SCG2	3435/334890	1.000	7149	0.855	-0.092642	-2	0.3333	0	18	-1.85	0	2	0	0
HIP_7150	SICSLYQLE	QYSSHHTAE	SICSLYQLEQYSSHHTAE		SCG1	9411/367830	0.998	7150	0.855	-0.108197	-1.4667	0.1667	0	18	-0.5833	-2	2	0	0
HIP_7151	CGERGFFYT	LRQAENEPQ	CGERGFFYTLRQAENEPQ		SCG2	9976/334890	0.999	7151	0.855	0.028913	-0.5667	0	-1	18	-1.15	-1	3	0	0
HIP_7152	VCGERGFFY	EADLDHPDL	VCGERGFFYEADLDHPDL		SCG2	1068/334890	1.000	7152	0.855	0.075340	-0.15	0	-3	18	-0.4	-4	3	0	0
HIP_7153	VEALYLVC	EPQWSLYPS	VEALYLVCEPQWSLYPS		SCG1	709/367830	1.000	7153	0.855	0.190513	0.3167	0.1667	-2	17	0.3118	-2	2	1	0
HIP_7154	LYQLENYCN	FQRNQLLQK	LYQLENYCNFQRNQLLQK		SCG2	17542/334890	0.997	7154	0.855	0.023711	-1.25	0	1	18	-1.0278	1	2	0	0
HIP_7155	GSHLVEALY	QAIKEHLNQ	GSHLVEALYQAIKEHLNQ		SCG2	2497/334890	1.000	7155	0.855	0.008974	1.1833	0	1	18	-0.3667	-1	1	0	0
HIP_7156	LVCGERGFF	QKLYTDDED	LVCGERGFFQKLYTDDED		SCG2	6092/334890	1.000	7156	0.855	0.026728	0.2667	0.1667	-3	18	-0.6833	-3	3	0	0
HIP_7157	VEALYLVCG	GYEKRNLAR	VEALYLVCGGYEKRNLAR		SCG1	2710/367830	1.000	7157	0.855	0.069459	0.1833	0.3333	1	18	-0.05	1	2	0	0
HIP_7158	CTSICSLYQ	QINDEMKRS	CTSICSLYQQINDEMKRS		SCG2	8100/334890	0.999	7158	0.855	0.016777	-0.5833	0	0	18	-0.5889	0	1	0	0
HIP_7159	LVEALYLV	TAEKRKRL	LVEALYLVTAEKRKRL		SCG1	9452/367830	0.998	7159	0.855	0.186223	0.7167	0	2	16	0.0875	2	1	0	0
HIP_7160	LVCGERGFF	SEKNFPMDM	LVCGERGFFSEKNFPMDM		SCG2	5752/334890	0.999	7160	0.855	0.118203	-0.5	0.3333	-1	18	-0.1611	-1	2	0	0
HIP_7161	YLVCGERGF	NRQYWDEDL	YLVCGERGFNRQYWDEDL		SCG2	7235/334890	0.999	7161	0.855	0.028141	-2.2667	0.1667	-2	18	-0.9556	-2	2	0	0
HIP_7162	LVCGERGFF	GERATRLFE	LVCGERGFFGERATRLFE		SCG2	4496/334890	1.000	7162	0.855	0.133236	-0.5333	0.3333	0	18	-0.0778	0	2	0	0
HIP_7163	ERGFFYTPK	ETHAAGHSQ	ERGFFYTPKETHAAGHSQ		SCG1	11365/367830	0.997	7163	0.855	-0.035413	-2.2667	0.1667	2	18	-1.2222	0	2	0	0
HIP_7164	CGERGFFYT	HQQKKHSGF	CGERGFFYTHQQKKHSGF		CMGA	12610/247050	0.991	7164	0.855	0.113094	-1.5667	0	4	18	-1.2389	2	3	0	0
HIP_7165	HLVEALYLV	RDSKENIEK	HLVEALYLVRDSKENIEK		SCG2	5320/334890	0.999	7165	0.855	0.233929	-0.35	0.1667	0	18	-0.5	-1	1	0	0
HIP_7166	SHLVEALYL	EEDWMRIIL	SHLVEALYLEEDWMRIIL		SCG2	2295/334890	1.000	7166	0.855	0.153133	-0.7	0	-2	18	0.4111	-3	2	0	0
HIP_7167	VEALYLVC	GENYQKGER	VEALYLVCGENYQKGER		SCG1	379/367830	1.000	7167	0.855	0.207849	0.5167	0.1667	-1	17	-0.5294	-1	2	1	0
HIP_7168	CTSICSLYQ	PYQGVSVPL	CTSICSLYQPYQGVSVPL		SCG2	4275/334890	1.000	7168	0.855	0.052994	-1.2333	0.1667	0	18	0.5111	0	2	0	0
HIP_7169	RGFFYTPKT	QESKGQPRS	RGFFYTPKTQESKGQPRS		SCG1	18600/367830	0.991	7169	0.855	0.012814	-2.3333	0.3333	3	18	-1.6667	3	1	0	0
HIP_7170	LYQLENYCN	QNAFLNERK	LYQLENYCNQNAFLNERK		SCG1	8732/367830	0.998	7170	0.855	-0.021167	-1.25	0	0	18	-1.1389	0	2	0	0
HIP_7171	LVCGERGFF	WAEGGGHSR	LVCGERGFFWAEGGGHSR		SCG1	3630/367830	0.999	7171	0.855	0.040028	0.4333	0.1667	1	18	-0.2778	0	3	0	1
HIP_7172	LVCGERGFF	KRSGQLGIQ	LVCGERGFFKRSGQLGIQ		SCG2	14755/334890	0.998	7172	0.855	0.134852	-0.6667	0.3333	2	18	-0.0778	2	2	0	0
HIP_7173	GERGFFYTP	EEEEEMAVV	GERGFFYTPEEEEEMAVV		CMGA	81/247050	1.000	7173	0.855	-0.031936	-2.35	0.1667	-5	18	-0.6778	-5	3	0	3
HIP_7174	QHLCGSHLV	DLFQNRMLS	QHLCGSHLVDLFQNRMLS		SCG2	8295/334890	0.999	7174	0.855	0.066297	1.3167	0	-2	18	-0.0167	0	1	0	0
HIP_7175	VCGERGFFY	KKELENLAA	VCGERGFFYKKELENLAA		SCG1	13155/367830	0.996	7175	0.855	0.188430	-1.1667	0	0	18	-0.2722	0	3	0	0
HIP_7176	RGFFYTPKT	DNPFKRTNE	RGFFYTPKTDNPFKRTNE		SCG2	9764/334890	0.999	7176	0.855	-0.037352	-2.4667	0.3333	2	18	-1.6333	2	2	0	0
HIP_7177	LVCGERGFF	RGWRPSSRE	LVCGERGFFRGWRPSSRE		CMGA	6766/247050	0.998	7177	0.855	0.092443	-0.1	0.3333	2	18	-0.7889	2	3	0	0
HIP_7178	CGERGFFY	AENEPQSAP	CGERGFFYAENEPQSAP		SCG2	5414/334890	0.999	7178	0.855	0.128607	-0.15	0	-2	17	-0.9647	-2	3	1	0
HIP_7179	GSLQPLAL	NHLKATPI	GSLQPLALNHLKATPI		IAPP	1696/45018	0.993	7179	0.855	0.140082	1.0833	0	2	16	0.2562	1	0	0	0
HIP_7180	CGERGFFYT	LNHLKATPI	CGERGFFYTLNHLKATPI		IAPP	1398/45018	0.994	7180	0.855	-0.006855	-0.35	0	2	18	-0.0944	1	3	0	0
HIP_7181	AGSLQPLAL	ALEYIENLR	AGSLQPLALALEYIENLR		SCG2	2107/334890	1.000	7181	0.855	0.062334	1.9167	0	-1	18	0.35	-1	1	0	0
HIP_7182	SHLVEALYL	NENTKFEVR	SHLVEALYLNENTKFEVR		SCG1	4327/367830	0.999	7182	0.855	0.222172	-0.7	0	0	18	-0.4167	-1	1	0	0
HIP_7183	VEALYLVC	EEVKTRHSE	VEALYLVCEEVKTRHSE		SCG1	2131/367830	1.000	7183	0.855	0.128859	1.2833	0	-1	17	-0.2294	-2	1	1	0
HIP_7184	CSLYQLENY	PQSAPKENK	CSLYQLENYPQSAPKENK		SCG2	3294/334890	1.000	7184	0.855	-0.036394	-2.3667	0.3333	0	18	-1.35	0	1	0	0
HIP_7185	VEALYLVC	LQWKSSHFE	VEALYLVCLQWKSSHFE		SCG1	7336/367830	0.999	7185	0.855	0.100676	1.65	0	0	17	0.3235	-1	2	1	0
HIP_7186	ERGFFYTPK	DNRNHNEGM	ERGFFYTPKDNRNHNEGM		SCG1	15106/367830	0.995	7186	0.855	-0.019275	-2.95	0.1667	1	18	-1.8889	0	2	0	0
HIP_7187	VEALYLVCG	FQNRMLSKS	VEALYLVCGFQNRMLSKS		SCG2	4343/334890	1.000	7187	0.855	0.040627	0.35	0.1667	1	18	0.3667	1	2	0	0
HIP_7188	LVEALYLVC	NWGYEKRNL	LVEALYLVCNWGYEKRNL		SCG1	618/367830	1.000	7188	0.855	0.156768	0.95	0.1667	0	18	0.0889	0	3	0	0
HIP_7189	VCGERGFFY	YPEEKKEEE	VCGERGFFYYPEEKKEEE		CMGA	370/247050	1.000	7189	0.855	0.109042	-0.35	0.1667	-3	18	-1.4444	-3	4	0	1
HIP_7190	VCGERGFFY	ERADEPQW	VCGERGFFYERADEPQW		SCG1	10014/367830	0.998	7190	0.855	0.131281	-0.3167	0	-2	17	-1	-2	3	1	0
HIP_7191	SHLVEALYL	QDLALQGAK	SHLVEALYLQDLALQGAK		CMGA	3232/247050	0.999	7191	0.855	0.177503	0.5167	0	0	18	0.2778	-1	1	0	0
HIP_7192	AGSLQPLAL	EALYLVCGE	AGSLQPLALEALYLVCGE		INSB	10/4968	0.999	7192	0.855	0.054245	1.9167	0	-2	18	0.8944	-2	1	0	0
HIP_7193	HLVEALYLV	SGYPKTPGR	HLVEALYLVSGYPKTPGR		SCG2	208/334890	1.000	7193	0.855	0.200015	0.7	0.3333	2	18	-0.0889	1	2	0	0
HIP_7194	CGERGFFY	LPYGAGRSR	CGERGFFYLPYGAGRSR		SCG2	6730/334890	0.999	7194	0.855	0.093881	0.8667	0.1667	2	17	-0.5824	2	4	1	0
HIP_7195	AGSLQPLAL	EQKLYTDDE	AGSLQPLALEQKLYTDDE		SCG2	2470/334890	1.000	7195	0.855	0.191138	-0.25	0	-3	18	-0.6056	-3	0	0	0
HIP_7196	ICSLYQLEN	EDSKRWSKM	ICSLYQLENEDSKRWSKM		CMGA	12404/247050	0.992	7196	0.855	0.102756	-1.8333	0.1667	0	18	-0.9944	0	1	0	0
HIP_7197	VCGERGFFY	QYSSHHTA	VCGERGFFYQYSSHHTA		SCG1	9031/367830	0.998	7197	0.855	0.156303	-0.2167	0.1667	2	17	-0.5588	0	4	1	0
HIP_7198	LYQLENYCN	EEEATNTHP	LYQLENYCNEEEATNTHP		CMGA	946/247050	1.000	7198	0.855	0.034085	-2.1333	0	-3	18	-1.3833	-4	1	0	1
HIP_7199	CGERGFFYT	SKDVMEKRE	CGERGFFYTSKDVMEKRE		CMGA	7130/247050	0.997	7199	0.855	0.143323	-1.2333	0.1667	0	18	-1.1222	0	3	0	0

HIP_7655	LVEALYLVC	QQAHKEESS	LVEALYLVCQQAHKEESS		SCG2	2597/334890	1.000	7655	0.854	0.140597	0.8833	0	-1	18	-0.0889	-2	1
HIP_7656	SHLVEALYL	NEQINDEMK	SHLVEALYLNEQINDEMK		SCG2	814/334890	1.000	7656	0.854	0.183491	-0.7	0	-2	18	-0.55	-3	1
HIP_7657	SLYQLENYC	SQEKTHSRE	SLYQLENYCSQEKTHSRE		SCG1	6067/367830	0.999	7657	0.854	0.136238	-1.6833	0.1667	0	18	-1.5667	-1	1
HIP_7658	TSICSLYQL	NWGYEKRNL	TSICSLYQLNWGYEKRNL		SCG1	2792/367830	1.000	7658	0.854	0.104145	-0.9667	0.1667	1	18	-0.5667	1	3
HIP_7659	CGERGFFYT	YEENSRDNP	CGERGFFYTYEENSRDNP		SCG2	8037/334890	0.999	7659	0.854	0.054441	-1.25	0	-2	18	-1.5778	-2	4
HIP_7660	HLVEALYLV	RKESKDQLS	HLVEALYLVRKESKDQLS		SCG2	13934/334890	0.998	7660	0.854	0.222580	-0.8667	0	1	18	-0.3889	0	1
HIP_7661	ERGFFYTPK	YGKRSSPET	ERGFFYTPKYGKRSSPET		NPY	1125/49410	0.994	7661	0.854	-0.048054	-1.9667	0.3333	2	18	-1.5444	2	3
HIP_7662	AGSLQPLAL	MLSKSGYPK	AGSLQPLALMLSKSGYPK		SCG2	5681/334890	0.999	7662	0.854	0.097801	2.3833	0.1667	2	18	0.0944	2	0
HIP_7663	CGERGFFYT	VEEPSSKDV	CGERGFFYTVEEPSSKDV		CMGA	3288/247050	0.999	7663	0.854	0.000769	-0.3333	0	-2	18	-0.6611	-2	3
HIP_7664	CTSICSLYQ	PFKRTNEIV	CTSICSLYQPFKRTNEIV		SCG2	8476/334890	0.999	7664	0.854	0.064847	-0.6167	0.1667	1	18	0	1	2
HIP_7665	TSICSLYQL	WMRILEAL	TSICSLYQLWMRILEAL		SCG2	9109/334890	0.999	7665	0.854	-0.012486	-0.75	0	0	18	1.05	0	2
HIP_7666	GSHLVEALY	YSSHHTAEK	GSHLVEALYYSSHHTAEK		SCG1	1284/367830	1.000	7666	0.854	0.164962	0.2333	0.3333	2	18	-0.6222	-1	2
HIP_7667	VCGERGFFY	DYETQQWPE	VCGERGFFYDYETQQWPE		SCG2	3320/334890	1.000	7667	0.854	0.091966	-0.6667	0	-3	18	-1.1	-3	4
HIP_7668	VCGERGFFY	SQEESEEG	VCGERGFFYSQEESEEG		SCG1	660/367830	1.000	7668	0.854	0.169452	-0.5833	0.1667	-4	17	-1.0176	-4	3
HIP_7669	QHLCGSHLV	EEARFQDKQ	QHLCGSHLVEEARFQDKQ		SCG1	6609/367830	0.999	7669	0.854	0.125773	-0.0667	0	1	18	-1.0056	-1	0
HIP_7670	VCGERGFFY	AENEPQSA	VCGERGFFYAENEPQSA		SCG2	4491/334890	1.000	7670	0.854	0.111600	-0.15	0	-2	17	-0.6235	-2	3
HIP_7671	LVCGERGFF	EDLRKESKD	LVCGERGFFEDLRKESKD		SCG2	2820/334890	1.000	7671	0.854	0.026100	0.3333	0.1667	-1	18	-0.8889	-1	2
HIP_7672	LYQLENYCN	AEAKTHWLG	LYQLENYCNAEAKTHWLG		SCG2	8853/334890	0.999	7672	0.854	-0.092353	-0.3667	0	0	18	-0.65	-1	1
HIP_7673	CGERGFFY	HPQESKGQP	CGERGFFYHPQESKGQP		SCG1	10196/367830	0.998	7673	0.854	0.168631	-0.6667	0.1667	1	17	-1.4118	0	3
HIP_7674	LVCGERGFF	EEQIEQAIK	LVCGERGFFEEQIEQAIK		SCG2	1800/334890	1.000	7674	0.854	0.041912	-0.8833	0.1667	-2	18	-0.1833	-2	2
HIP_7675	AGSLQPLAL	ESKDQLSDD	AGSLQPLALESKDQLSDD		SCG2	1602/334890	1.000	7675	0.854	0.218240	0.2	0.1667	-3	18	-0.5833	-3	0
HIP_7676	VCGERGFFY	PVDNRNHNE	VCGERGFFYPVDNRNHNE		SCG1	457/367830	1.000	7676	0.854	0.141950	0.5667	0.1667	0	18	-1.1333	-1	3
HIP_7677	TSICSLYQL	RNAVEVLKR	TSICSLYQLRNAVEVLKR		IAPP	2809/45018	0.981	7677	0.854	0.090772	-1.2	0	2	18	0.0889	2	1
HIP_7678	VCGERGFFY	LVARSETHA	VCGERGFFYLVARSETHA		SCG1	957/367830	1.000	7678	0.854	-0.038198	2.35	0	1	18	0.0611	0	3
HIP_7679	LYQLENYCN	EDMARYYSA	LYQLENYCNEDMARYYSA		NPY	885/49410	0.995	7679	0.854	-0.054447	-1.2333	0	-2	18	-0.8833	-2	1
HIP_7680	PLALEGSLQ	QEEERLSKE	PLALEGSLQQEEERLSKE		CMGA	1693/247050	1.000	7680	0.854	-0.065626	-1.8333	0.1667	-3	18	-1.0833	-3	0
HIP_7681	PLALEGSLQ	EQEEERLSK	PLALEGSLQEQEEERLSK		CMGA	1694/247050	1.000	7681	0.854	-0.065626	-1.8333	0.1667	-3	18	-1.0833	-3	0
HIP_7682	LVCGERGFF	GWQAKREEE	LVCGERGFFGWQAKREEE		CMGA	335/247050	1.000	7682	0.854	0.046749	0.0667	0.3333	-1	18	-0.8111	-1	3
HIP_7683	LVCGERGFF	THSREKSSQ	LVCGERGFFTHSREKSSQ		SCG1	9488/367830	0.998	7683	0.854	0.076578	0.0833	0.3333	2	18	-0.8	1	2
HIP_7684	GERGFFYTP	RSNQLPKAA	GERGFFYTPRSNQLPKAA		SCG2	12207/334890	0.998	7684	0.854	0.038717	-2.0667	0.3333	2	18	-0.9556	2	3
HIP_7685	SHLVEALYL	LQDTKENR	SHLVEALYLLQDTKENR		SCG1	5535/367830	0.999	7685	0.854	0.175009	0.5167	0	0	17	-0.6294	-1	1
HIP_7686	SLQPLAL	EAYPSKP	SLQPLALEAYPSKP		NPY	1090/49410	0.994	7686	0.854	0.108115	1.0667	0	0	14	-0.2571	0	1
HIP_7687	GSHLVEALY	YDPLQWKSS	GSHLVEALYYDPLQWKSS		SCG1	634/367830	1.000	7687	0.854	0.099398	-0.35	0.1667	0	18	-0.45	-1	2
HIP_7688	HLVEALYL	SHFERRDNM	HLVEALYLSHFERRDNM		SCG1	7914/367830	0.998	7688	0.854	0.108359	0.85	0.1667	1	17	-0.5529	-1	2
HIP_7689	SICSLYQLE	EEEEEEATN	SICSLYQLEEEEEEEATN		CMGA	634/247050	1.000	7689	0.854	-0.069544	-2.2833	0	-7	18	-1.0389	-7	1
HIP_7690	LVCGERGFF	SEEPVSTHQ	LVCGERGFFSEEPVSTHQ		SCG1	710/367830	1.000	7690	0.854	0.079421	-0.4333	0.3333	-1	18	-0.3389	-2	2
HIP_7691	CTSICSLYQ	KRNYPSLEL	CTSICSLYQKRNYPSLEL		SCG1	20337/367830	0.989	7691	0.854	0.151312	-2.15	0	1	18	-0.2944	1	2
HIP_7692	AGSLQPLAL	KLYTDDEDD	AGSLQPLALKLYTDDEDD		SCG2	4632/334890	1.000	7692	0.854	0.054867	1.3333	0	-4	18	-0.6056	-4	1
HIP_7693	VCGERGFFY	PEYNYDWWE	VCGERGFFYPEYNYDWWE		SCG1	762/367830	1.000	7693	0.854	0.107887	-0.35	0.1667	-3	18	-0.9889	-3	4
HIP_7694	CGERGFFY	EEGENYQKG	CGERGFFYEEGENYQKG		SCG1	731/367830	1.000	7694	0.854	0.188045	-0.5167	0.1667	-2	17	-1.5	-2	3
HIP_7695	HLVEALYLV	ELDRNYLNY	HLVEALYLVELDRNYLNY		SCG1	279/367830	1.000	7695	0.854	0.115116	0.5833	0	-1	18	0.0056	-2	1
HIP_7696	VCGERGFFY	YNPYQGVS	VCGERGFFYYNPYQGVS		SCG2	4695/334890	1.000	7696	0.854	0.136356	-0.35	0.1667	0	17	-0.3529	0	5
HIP_7697	VCGERGFFY	EALAVDGAG	VCGERGFFYEALAVDGAG		CMGA	160/247050	1.000	7697	0.854	0.009802	1.0667	0	-2	18	0.4333	-2	3

[illegible]

HIP_8166	LVEALYLVC	WQAKREEEE	LVEALYLVCWQAKREEEE		CMGA	1432/247050	1.000	8166	0.853	0.052585	1.3167	0	-3	18	-0.3167	-3	2	0	2	0.000000	-4.3667	0	0.05	2.000000	0.1111	1.8889	0	0	0	0	0	2.487	-8.7333	0	0	0	0	0.3436	1.1605	0.7423	0.6667	0.1181	0.551	0.4524	0.0025	-0.2183
HIP_8167	VCGERGFFY	NGERATRL	VCGERGFFYNGERATRL		SCG2	3496/334890	1.000	8167	0.853	0.211677	-0.5167	0.1667	1	17	-0.5471	1	3	1	0	0.000000	-3.9	-1	0.1833	0.700000	0.3333	0.3667	0	1	0	1	3.9	-0.1894	-11.7	0	1	0	0	0.4551	1.0169	0.9845	0.1667	0.2071	0.9692	-0.2351	0.0336	-0.715
HIP_8168	CGERGFFY	PQSAPKEN	CGERGFFYPQSAPKEN		SCG2	2242/334890	1.000	8168	0.853	0.196835	-0.2667	0.3333	0	16	-1.1625	0	3	0	0	0.000000	-3.4	0	0.1833	1.150000	0.1111	1.0389	1	0	1	0	0	-0.277	-10.2	0	1	0	0	0.4551	0.9836	0.9678	0.3333	0.2071	0.9367	-0.1214	0.0336	-0.6233
HIP_8169	VCGERGFFY	EEENELTLN	VCGERGFFYEEENELTLN		SCG1	892/367830	1.000	8169	0.853	0.123892	-1.0333	0	-4	18	-0.6611	-4	3	0	1	0.000000	-4.9333	-3	0.15	0.700000	0.4444	0.2556	0	0	0	0	14.8	-0.2641	-14.8	0	1	0	0	0.4385	1.3253	0.7212	-0.1667	0.1922	0.5201	-0.4531	0.0225	-0.74
HIP_8170	AGSLQPLAL	DSKENIEKN	AGSLQPLALDSKENIEKN		SCG2	4890/334890	1.000	8170	0.853	0.241402	0.2	0.1667	-1	18	-0.7167	-1	0	0	0	0.000000	-5.8667	0	0.1667	0.450000	0.3333	0.1167	0	0	0	0	0	0.0233	0	1	0	0	0	0.3436	1.1488	0.779	0.1667	0.1181	0.6068	0.0687	0.0278	-0.9778
HIP_8171	RGFFYTPKT	FQNRMLSKS	RGFFYTPKTFQNRMLSKS		SCG2	12379/334890	0.998	8171	0.853	0.026189	-1.7333	0.1667	4	18	-0.8889	4	2	0	0	0.000000	0.6667	-1	-0.0667	0.700000	0.2222	0.4778	1	0	1	0	-0.6667	-0.8281	1.3333	0	1	0	0	0.5218	0.9003	1.0878	0	0.2723	1.1834	-0.9044	0.0044	-0.0444
HIP_8172	VCGERGFFY	EEDLRKESK	VCGERGFFYEEDLRKESK		SCG2	3683/334890	1.000	8172	0.853	0.131733	-1.0333	0	-1	18	-1.1722	-1	3	0	0	0.000000	-4.9333	-3	0.15	1.650000	0.4444	1.2056	0	0	0	0	14.8	-1.2457	-14.8	0	1	0	0	0.4103	1.2438	0.749	-0.1667	0.1683	0.561	-0.4239	0.0225	-0.74
HIP_8173	TSICSLYQL	QECFETLRG	TSICSLYQLQECFETLRG		CMGA	6113/247050	0.998	8173	0.853	0.022010	-0.9167	0	-1	18	0.0278	-1	2	0	0	0.000000	-1.1667	-1	0.05	0.700000	0.1111	0.5889	0	0	0	0	1.1667	-0.5398	-2.3333	1	0	0	0	0.4769	1.1788	0.8356	0	0.2275	0.6983	-0.4372	0.0025	-0.0583
HIP_8174	QCCTSICSL	AEAYPSKPD	QCCTSICSLAEAYPSKPD		NPY	345/49410	0.999	8174	0.853	0.001814	0.9333	0.1667	-1	18	-0.1444	-1	1	0	0	1.000000	-1.8	-1	-0.0167	0.450000	0.1111	0.3389	0	0	0	0	1.8	0.3163	-1.8	1	0	0	0	0.3718	1.2553	0.7778	0.3333	0.1382	0.605	0.347	0.0003	0.03
HIP_8175	QHLCGSHLV	MYEENSRDN	QHLCGSHLVMYEENSRDN		SCG2	6612/334890	0.999	8175	0.853	0.047400	0.3167	0	0	18	-1.0556	-2	1	0	0	1.000000	-2.5667	-1	0.05	1.425000	0.2222	1.2028	0	0	0	0	2.5667	0.3809	-2.5667	1	0	0	0	0.3436	1.1855	0.6923	0.1667	0.1181	0.4793	0.1088	0.0025	-0.1283
HIP_8176	CTSICSLYQ	KFEVRLLRD	CTSICSLYQKFEVRLLRD		SCG1	18794/367830	0.991	8176	0.853	0.031713	-0.9333	0	1	18	0.05	1	2	0	0	0.000000	-1.2	0	0.05	1.675000	0.2222	1.4528	0	0	0	0	0	-1.3559	-2.4	0	1	0	0	0.4103	1.1938	0.7723	0	0.1683	0.5965	-0.3829	0.0025	-0.06
HIP_8177	LVEALYLVC	REDSKEAEK	LVEALYLVCREDSKEAEK		CMGA	3131/247050	0.999	8177	0.853	0.176850	-0.1667	0	-2	18	-0.3333	-2	1	0	0	0.000000	-7.3333	-1	0.15	2.000000	0.3333	1.6667	0	0	0	0	7.3333	-0.2778	-7.3333	0	0	0	0	0.3821	1.1558	0.7435	-0.1667	0.146	0.5527	-0.0637	0.0225	-1.1
HIP_8178	VCGERGFFY	QALPEPMQE	VCGERGFFYQALPEPMQE		CMGA	373/247050	1.000	8178	0.853	0.015496	1.0667	0	-2	18	-0.4167	-2	3	0	0	0.000000	-0.7333	0	0.05	0.700000	0.1111	0.5889	0	0	0	0	0	0.6281	-2.2	0	1	0	0	0.3051	1.2136	0.7078	0.6667	0.0931	0.501	0.3255	0.0025	-0.0367
HIP_8179	SICSLYQLE	ENVPRTRLE	SICSLYQLEENVPRTRLE		NPY	1468/49410	0.992	8179	0.853	-0.147878	-1	0	-1	18	-0.5056	-1	1	0	0	0.000000	0.1333	0	0	1.900000	0.2222	1.6778	0	0	0	0	0	-1.6778	0.1333	0	0	1	0	0.4667	1.1933	0.8367	0	0.2178	0.7	-0.4667	0	0
HIP_8180	VCGERGFFY	GRLQNGQN	VCGERGFFYGRLQNGQN		SCG2	5638/334890	0.999	8180	0.853	0.193316	0.5333	0.1667	1	17	-0.7824	1	3	1	0	0.000000	-1.8	1	0.1333	0.700000	0.2222	0.4778	0	1	0	1	-1.8	0.2548	-5.4	0	1	0	0	0.3603	1.0405	0.8356	0.3333	0.1298	0.6983	0.1921	0.0178	-0.24
HIP_8181	GSHLVEALY	QEEERLSKE	GSHLVEALYQEEERLSKE		CMGA	341/247050	1.000	8181	0.853	0.134314	-1.0333	0	-2	18	-1.0278	-3	1	0	1	0.000000	-4.9333	-2	0.15	2.000000	0.4444	1.5556	0	0	0	0	9.8667	-1.6074	-4.9333	0	1	0	0	0.4385	1.3169	0.7545	0	0.1922	0.5693	-0.4531	0.0225	-0.74
HIP_8182	GSHLVEALY	EQEEERLSK	GSHLVEALYEQEEERLSK		CMGA	342/247050	1.000	8182	0.853	0.134314	-1.0333	0	-2	18	-1.0278	-3	1	0	1	0.000000	-4.9333	-2	0.15	2.000000	0.4444	1.5556	0	0	0	0	9.8667	-1.6074	-4.9333	0	1	0	0	0.4385	1.3169	0.7545	0	0.1922	0.5693	-0.4531	0.0225	-0.74
HIP_8183	VCGERGFFY	FNPYYDPLQ	VCGERGFFYFNPYYDPLQ		SCG1	1150/367830	1.000	8183	0.853	0.103716	0.3333	0.1667	-1	18	-0.4167	-1	5	0	0	0.000000	-2.2	0	0.1167	1.400000	0.1111	1.2889	1	0	1	0	0	0.4296	-11	0	1	0	0	0.3885	0.9769	0.9645	0.5	0.1509	0.9302	0.1295	0.0136	-0.2567
HIP_8184	VEQCCTSIC	KNFFPEYNY	VEQCCTSICKNFFPEYNY		SCG1	9256/367830	0.998	8184	0.853	0.029414	0.2667	0.1667	-1	18	-0.2944	-1	2	0	0	0.000000	-3.6	1	0.0333	2.450000	0.1111	2.3389	0	0	0	0	-3.6	0.6237	-7.2	0	0	0	0	0.4103	0.9655	0.9406	0.1667	0.1683	0.8848	0.1094	0.0011	-0.12
HIP_8185	QHLCGSHLV	GYEKRNLAR	QHLCGSHLVGYEKRNLAR		SCG1	8309/367830	0.998	8185	0.853	0.219780	-0.0667	0.1667	4	18	-0.7111	2	1	0	0	0.000000	-3.3333	-1	0.1333	0.950000	0.2222	0.7278	0	1	0	1	3.3333	-0.0485	-3.3333	1	0	0	0	0.4269	1.0688	0.8423	0	0.1823	0.7095	-0.0285	0.0178	-0.4444
HIP_8186	SHLVEALY	GYPKTPGRA	SHLVEALYGYPKTPGRA		SCG2	1795/334890	1.000	8186	0.853	0.218222	0.1667	0.3333	2	17	-0.4588	1	2	1	0	0.000000	-2.5333	0	0.15	0.000000	0.2222	-0.2222	1	1	1	1	0	-0.037	-5.0667	0	1	0	0	0.4436	0.9772	0.999	0.3333	0.1968	0.9979	0.0739	0.0225	-0.38
HIP_8187	VCGERGFFY	FPEYNYDWW	VCGERGFFYFPEYNYDWW		SCG1	657/367830	1.000	8187	0.853	0.095738	0.3333	0.1667	-2	18	-0.6389	-2	5	0	0	0.000000	-2.2	-1	0.1167	1.400000	0.2222	1.1778	1	0	1	0	2.2	0.3926	-11	0	1	0	0	0.3885	1.1169	0.8278	0.3333	0.1509	0.6853	0.1295	0.0136	-0.2567
HIP_8188	ICSLYQLEN	FQELGKLTG	ICSLYQLENFQELGKLTG		SCG2	9762/334890	0.999	8188	0.853	-0.117943	-1.2333	0	-1	18	0	-1	2	0	0	0.000000	-0.3333	0	0	1.650000	0.2222	1.4278	0	0	0	0	0	-1.7609	-0.6667	0	1	0	0	0.4667	1.2167	0.8533	0	0.2178	0.7282	-0.5756	0	0
HIP_8189	GERGFFYTP	KRFLGEGHH	GERGFFYTPKRFLGEGHH		SCG1	15138/367830	0.995	8189	0.853	-0.030358	-1.5333	0.1667	3	18	-1.0722	1	4	0	0	0.000000	-0.6667	2	-0.0167	1.650000	0.2222	1.4278	1	0	1	0	-1.3333	-2.1893	-2.6667	0	0	0	1	0.4654	0.9441	0.9668	-0.1667	0.2166	0.9347	-0.7136	0.0003	0.0111
HIP_8190	CGERGFFY	GSEEYRAPR	CGERGFFYGSEEYRAPR		SCG1	2343/367830	1.000	8190	0.853	0.206424	-0.0667	0.3333	0	17	-1.1941	0	4	1	0	0.000000	-3	-1	0.2	0.000000	0.2222	-0.2222	0	1	0	1	3	0.0148	-12	0	1	0	0	0.4551	1.0386	0.9245	0.1667	0.2071	0.8547	-0.0303	0.04	-0.6
HIP_8191	SLYQLENYC	QRNQLLQKE	SLYQLENYCQRNQLLQKE		SCG2	15386/334890	0.998	8191	0.853	0.134137	-2.3	0	0	18	-1.2278	0	1	0	0	0.000000	-3.0667	1	0.1	0.950000	0.2222	0.7278	0	0	0	0	-3.0667	-1.6739	-3.0667	0	0	0	0	0.5154	0.9191	1.1368	-0.1667	0.2656	1.2923	-1.1854	0.01	-0.3067
HIP_8192	HLVEALYLV	RQYWDEDLL	HLVEALYLVRQYWDEDLL		SCG2	8855/334890	0.999	8192	0.853	0.121214	-0.4333	0	-2	18	0.0278	-3	3	0	0	0.000000	-5.3333	1	0.1	1.400000	0.1111	1.2889	0	0	0	0	-5.3333	-0.5585	-16	1	0	0	0	0.3821	1.0891	0.7835	0.1667	0.146	0.6138	-0.1656	0.01	-0.5333
HIP_8193	VEALYLVC	WEKKPFSED	VEALYLVCWEKKPFSED		SCG1	10622/367830	0.997	8193	0.853	0.159309	0.3667	0	-2	17	-0.1941	-2	2	1	0	0.000000	-6.2667	0	0.1	0.625000	0.3333	0.2917	0	0	0	0	0	0.1069	-12.5333	0	0	0	0	0.3821	1.1558	0.7435	0.1667	0.146	0.5527	0.1401	0.01	-0.6267
HIP_8194	CGERGFFYT	QSAPKENK	CGERGFFYTQSAPKENK		SCG2	11034/334890	0.999	8194	0.853	0.107103	-0.2833	0.1667	1	17	-1.2706	1	3	1	0	0.000000	-1.1	0	0.0667	0.000000	0	0	0	0	0	0	0	0	-3.3	0	1	0	0	0.4385	1.0686	0.8845	0.3333	0.1922	0.7823	-0.1242	0.0044	-0.0733
HIP_8195	VEQCCTSIC	WWEKKPFSE	VEQCCTSICWWEKKPFSE		SCG1	5107/367830	0.999	8195	0.853	0.003033	0.15	0.1667	-1	18	-0.4722	-1	2	0	0	0.000000	-3.8333	-1	-0.0167	1.750000	0.2222	1.5278	0	0	0	0	3.8333	0.2292	-7.6667	0	0	0	0	0.4487	1.0758	0.8551	0.3333	0.2013	0.7312	0.0673	0.0003	0.0639
HIP_8196	VCGERGFF	TSEVDKRRT	VCGERGFFTSEVDKRRT		SCG1	5542/367830	0.999	8196	0.853	0.127250	0.0333	0.3333	1	17	-0.8471	1	2	1	0	0.000000	-3.4	-1	0.0833	0.700000	0.3333	0.3667	0	1	0	1	3.4	0.0122	-6.8	1	0	0	0	0.4833	1.0067	0.9333	0.1667	0.2336	0.8711	0.0161	0.0069	-0.2833
HIP_8197	TSICSLYQL	SEEKGHPQE	TSICSLYQLSEEKGHPQE		SCG1	4538/367830	0.999	8197	0.853	0.158248	-1.4667	0.1667	-1	18	-0.9111	-2	1	0	0	0.000000	-2.2667	-2	0.1167	0.000000	0.3333	-0.3333	0	0	0	0	4.5333	0.4889	-2.2667	1	0	0	0	0.5051	1.2086	0.8445	-0.1667	0.2552	0.7132	-0.7409	0.0136	-0.2644
HIP_8198	CGERGFFYT	AEEAQDPEG	CGERGFFYTAEEAQDPEG		CMGA	2135/247050	0.999	8198	0.853	-0.000842	-0.7333	0	-4	18	-1.0333	-4	3	0	0	0.000000	-2	-2	0.05	0.900000	0.2222	0.6778	0	0	0	0	4	-0.497	-6	0	1	0	0	0.4385	1.2536	0.7678	0	0.1922	0.5895	-0.3215	0.0025	-0.1
HIP_8199	GSLQPLAL	EVDKRRTRP	GSLQPLALEVDKRRTRP		SCG1	7090/367830	0.999	8199	0.853	0.128760	1.1	0	2	17	-0.9176	2	0	1	0	0.000000	-4.0667	-2	0.1	2.000000	0.4444	1.5556	0	0	0	0	8.1333	1.7111	0	1	0	0	0	0.3051	1.2736	0.6345	0.3333	0.0931	0.4026	0.3356	0.01	-0.4067
HIP_8200	AGSLQPLAL	QASAIKKEE	AGSLQPLALQASAIKKEE		SCG1	2300/367830	1.000	8200	0.853	0.191974	1.15	0.1667	0	18	-0.1278	0	0	0	0	0.000000	-3.9667	0	0.1167	0.900000	0	0.9	0	0	0	0	0	1.035	0	1	0	0	0	0.3333	1.225	0.75	0.6667	0.1111	0.5625	0.3833	0.0136	-0.4628
HIP_8201	SHLVEALY	PNPYNQEKV	SHLVEALYPNPYNQEKV		SCG2	724/334890	1.000	8201	0.853	0.216528	-0.4	0.3333	0	17	-0.7882	-1	2	1	0	0.000000	-3.6667	0	0.1833	0.000000	0.1111	-0.1111	2	0	1	0	0	0.0444	-7.3333	0	1	0	0	0.4718	0.9303	1.1112	0.3333	0.2226	1.2347	-0.1887	0.0336	-0.6722
HIP_8202	RGFFYTPKT	ESRSEALAV	RGFFYTPKTESRSEALAV		CMGA	11427/247050	0.993	8202	0.853	-0.008570	-2.5	0.3333	1	18	-0.5	1	1	0	0	0.000000	-0.8667	-1	0	0.000000	0.3333	-0.3333	1	0	1	0	0.8667	0.8333	-0.8667	0	1	0	0	0.5603	0.9588	1.039	-0.5	0.3139	1.0795	-1.4006	0	0
HIP_8203	SLYQLENYC	NQKRERMDE	SLYQLENYCNQKRERMDE		SCG2	13261/334890	0.998	8203	0.853	0.119983	-2.2	0	-1	18	-1.7944	-1	1	0	0	0.000000	-2.8667	1	0.1	0.950000	0.3333	0.6167	0	0	0	0	-2.8667	-1.3567	-2.8667	0	0	0	0	0.5154	0.9191	1.1368	-0.1667	0.2656	1.2923	-1.1338	0.01	-0.2867
HIP_8204	CTSICSLYQ	AYENLNDKD	CTSICSLYQAYENLNDKD		SCG2	4789/334890	1.000	8204	0.853	-0.087413	-0.6667	0	-2	18	-0.6056	-2	2	0	0	0.000000	-0.6667	-1	0	1.075000	0.1111	0.9639	0	0	0	0	0.6667	-0.6426	-1.3333	0	1	0	0	0.4103	1.2355	0.7823	0.1667	0.1683	0.612	-0.2735	0	0
[illegible]																																														

HIP_8677	CGERGFFYT	LGEKRDHHS	CGERGFFYTLGEKRDHHS		SCG1	1405/367830	1.000	8677	0.852	0.053396	0.1167	0.1667	2	18	-1.2167	0	3	0	0
HIP_8678	CGERGFFY	VDNRNHNEG	CGERGFFYVDNRNHNEG		SCG1	8603/367830	0.998	8678	0.852	0.148684	0.25	0	0	17	-1.3765	-1	3	1	0
HIP_8679	QHLCGSHLV	QKFPSPEMI	QHLCGSHLVQKFPSPEMI		SCG2	15845/334890	0.997	8679	0.852	0.198760	0.0333	0	2	18	-0.1389	0	1	0	0
HIP_8680	CTSICSLYQ	PQSAPKENK	CTSICSLYQPQSAPKENK		SCG2	1466/334890	1.000	8680	0.852	0.152699	-1.15	0.3333	1	18	-0.7944	1	1	0	0
HIP_8681	LVCGERGFF	EEEEMAVVP	LVCGERGFFEEEEMAVVP		CMGA	364/247050	1.000	8681	0.852	0.041070	-0.8833	0.1667	-4	18	0.2111	-4	2	0	2
HIP_8682	VCGERGFFY	QEESEESN	VCGERGFFYQEESEESN		SCG1	2267/367830	1.000	8682	0.852	0.140365	-1.0333	0	-4	17	-1.2	-4	3	1	0
HIP_8683	VCGERGFFY	EPERELDLP	VCGERGFFYEPERELDLP		SCG2	318/334890	1.000	8683	0.852	0.156986	-0.7167	0.1667	-3	18	-0.6611	-3	3	0	0
HIP_8684	SHLVEALYL	SPDYNPYQG	SHLVEALYLSPDYNPYQG		SCG2	349/334890	1.000	8684	0.852	0.212607	0.0667	0.3333	-1	18	-0.4944	-2	2	0	0
HIP_8685	GERGFFYT	TAEKRKRLG	GERGFFYTTAEKRKRLG		SCG1	20513/367830	0.989	8685	0.852	0.087284	-0.2667	0	3	17	-1.2353	3	3	1	0
HIP_8686	SHLVEALYL	INDEMKRSG	SHLVEALYLINDEMKRSG		SCG2	2925/334890	1.000	8686	0.852	0.166664	0.6333	0	0	18	-0.2833	-1	1	0	0
HIP_8687	LVEALYLVC	EYIENLRQQ	LVEALYLVCEYIENLRQQ		SCG2	2890/334890	1.000	8687	0.852	0.053342	1.7	0	-2	18	0.2389	-2	2	0	0
HIP_8688	HLVEALYLV	ELDKMAHG	HLVEALYLVELDKMAHG		SCG1	2253/367830	1.000	8688	0.852	0.125889	0.5833	0	0	17	0.3882	-2	1	1	0
HIP_8689	CGERGFFYT	EMIRALEYI	CGERGFFYTEMIRALEYI		SCG2	4654/334890	1.000	8689	0.852	-0.041212	0.6167	0	-1	18	0.0556	-1	3	0	0
HIP_8690	CGERGFFY	GPNNQKRE	CGERGFFYGPNNQKRE		SCG2	1566/334890	1.000	8690	0.852	0.201165	-0.2	0.3333	1	16	-1.65	1	3	0	0
HIP_8691	LVEALYLVC	NPYYDPLQW	LVEALYLVCNPYYDPLQW		SCG1	1055/367830	1.000	8691	0.852	0.126261	0.6833	0.1667	-2	18	0.3278	-2	3	0	0
HIP_8692	AGSLQPLAL	EDSKRWSKM	AGSLQPLALEDSKRWSKM		CMGA	2027/247050	0.999	8692	0.852	0.195508	0.2667	0.1667	1	18	-0.6222	1	0	0	0
HIP_8693	TSICSLYQL	PAEDMARYY	TSICSLYQLPAEDMARYY		NPY	123/49410	1.000	8693	0.852	0.058974	-0.7167	0.1667	-1	18	-0.15	-1	1	0	0
HIP_8694	AGSLQPLAL	SLYQLENYC	AGSLQPLALSLYQLENYC		INSA	14/3024	0.999	8694	0.852	0.099460	1.85	0.1667	-1	18	0.2722	-1	1	0	0
HIP_8695	ICSLYQLEN	PERKLKHMQ	ICSLYQLENPERKLKHMQ		SCG2	21869/334890	0.995	8695	0.852	0.118200	-2.1333	0.1667	2	18	-0.9111	1	1	0	0
HIP_8696	GSHLVEALY	PLQWKSSHF	GSHLVEALYPLQWKSSHF		SCG1	889/367830	1.000	8696	0.852	0.136071	0.5	0.1667	1	18	-0.2056	0	2	0	0
HIP_8697	CGERGFFYT	EKSQREDEE	CGERGFFYTEKSQREDEE		SCG1	7839/367830	0.998	8697	0.852	0.130969	-1.2333	0.1667	-3	18	-1.8278	-3	3	0	0
HIP_8698	GSHLVEALY	WDEDLLMKV	GSHLVEALYWDEDLLMKV		SCG2	2268/334890	1.000	8698	0.852	0.079262	-0.6	0	-2	18	0.1556	-3	2	0	0
HIP_8699	LVEALYLVC	KESKDQLSD	LVEALYLVCKESKDQLSD		SCG2	3962/334890	1.000	8699	0.852	0.210735	0.3833	0.1667	-2	18	-0.0167	-2	1	0	0
HIP_8700	GERGFFYTP	RSETHAAGH	GERGFFYTPRSETHAAGH		SCG1	13786/367830	0.996	8700	0.852	0.031017	-2.0667	0.3333	2	18	-1.0833	0	3	0	0
HIP_8701	ERGFFYTPK	AKTHWLGAA	ERGFFYTPKAKTHWLGAA		SCG2	9724/334890	0.999	8701	0.852	-0.015158	-1.5	0.1667	3	18	-0.5667	2	2	0	0
HIP_8702	CCTSICSLY	KRFLGEGHH	CCTSICSLYKRFLGEGHH		SCG1	18774/367830	0.991	8702	0.852	0.018821	-0.65	0.1667	3	18	-0.0167	1	2	0	0
HIP_8703	TSICSLYQL	SGYPKTPGR	TSICSLYQLSGYPKTPGR		SCG2	2973/334890	1.000	8703	0.852	0.133211	-0.5833	0.3333	2	18	-0.4278	2	2	0	0
HIP_8704	VCGERGFFY	DASEAHESS	VCGERGFFYDASEAHESS		SCG1	1027/367830	1.000	8704	0.852	0.129717	0.3	0.1667	-2	18	-0.5722	-3	3	0	0
HIP_8705	GSLQPLALE	EEEGSANRR	GSLQPLALEEEEGSANRR		CMGA	3932/247050	0.999	8705	0.852	0.079107	-1.4	0	-2	18	-1.0556	-2	0	0	2
HIP_8706	TSICSLYQL	EDSKRWSKM	TSICSLYQLEDSKRWSKM		CMGA	7947/247050	0.997	8706	0.852	0.201208	-1.4667	0.1667	1	18	-0.6889	1	1	0	0
HIP_8707	ERGFFYTPK	SKSSAPPIT	ERGFFYTPKSKSSAPPIT		SCG1	12993/367830	0.996	8707	0.852	-0.052643	-1.95	0.5	2	18	-0.7889	2	2	0	0
HIP_8708	LVCGERGFF	KAAWIPHVE	LVCGERGFFKAAWIPHVE		SCG2	6302/334890	0.999	8708	0.852	0.046112	0.8167	0.1667	1	18	0.3611	0	3	0	0
HIP_8709	SHLVEALYL	QGAKERAH	SHLVEALYLQGAKERAH		CMGA	2828/247050	0.999	8709	0.852	0.240758	0.7	0.1667	2	17	-0.4	0	1	1	0
HIP_8710	LVCGERGFF	KGERGEDSS	LVCGERGFFKGERGEDSS		SCG1	5050/367830	0.999	8710	0.852	0.133127	-0.4333	0.3333	-1	18	-0.7778	-1	2	0	0
HIP_8711	RGFFYTPKT	EEEGPTVVL	RGFFYTPKTEEEGPTVVL		CMGA	6513/247050	0.998	8711	0.852	-0.027788	-2.7833	0.1667	-1	18	-0.4722	-1	1	0	1
HIP_8712	LVCGERGFF	SEEKGHPQ	LVCGERGFFSEEKGHPQ		SCG1	3890/367830	0.999	8712	0.852	0.093621	-0.4333	0.3333	0	17	-0.7706	-1	2	1	0
HIP_8713	LYQLENYCN	WDEEDKRNY	LYQLENYCNWDEEDKRNY		SCG1	6465/367830	0.999	8713	0.852	-0.003930	-1.7	0	-3	18	-1.9222	-3	2	0	0
HIP_8714	LVEALYLVC	KRFLGEGHH	LVEALYLVCKRFLGEGHH		SCG1	15082/367830	0.995	8714	0.852	0.128915	0.8167	0	2	18	0.3778	0	2	0	0
HIP_8715	CGERGFFY	THQEAENEK	CGERGFFYTHQEAENEK		SCG1	10389/367830	0.998	8715	0.852	0.133123	-0.5167	0	-1	17	-1.5	-2	3	1	0
HIP_8716	SHLVEALYL	AQDPEGKGE	SHLVEALYLAQDPEGKGE		CMGA	1242/247050	1.000	8716	0.852	0.144844	0.1833	0	-2	18	-0.55	-3	1	0	0
HIP_8717	HLVEALYLV	TSYFPNPYN	HLVEALYLVTSYFPNPYN		SCG2	3439/334890	1.000	8717	0.852	0.127720	0.65	0.1667	0	18	0.1167	-1	3	0	0
HIP_8718	HLVEALYLV	RAHQQKKHS	HLVEALYLVRAHQQKKHS		CMGA	16740/247050	0.983	8718	0.852	0.132769	0.1333	0	5	18	-0.6167	2	1	0	0
HIP_8719	HLVEALYLV	HQLQALRRG	HLVEALYLVHQLQALRRG		CMGA	10022/247050	0.995	8719	0.852	0.109369	0.6333	0	3	18	0.1889	1	1	0	0

HIP_9188	CGERGFFY	GRSRPQRSS	CGERGFFYGRSRPQRSS		SCG1	8837/367830	0.998	9188	0.851	0.230238	-0.2333	0.3333	2	17	-1.3765	2	3	1	0
HIP_9189	CSLYQLENY	EYRAPRPQS	CSLYQLENYEYRAPRPQS		SCG1	25897/367830	0.978	9189	0.851	-0.027455	-2.9333	0	0	18	-1.2944	0	2	0	0
HIP_9190	SICSLYQLE	SSHFERRDN	SICSLYQLESSHFERRDN		SCG1	9969/367830	0.998	9190	0.851	-0.112522	-1.3333	0.3333	0	18	-0.9333	-1	2	0	0
HIP_9191	VCGERGFFY	QSIYQLIEI	VCGERGFFYQSIYQLIEI		SCG2	810/334890	1.000	9191	0.851	0.131487	0.75	0.1667	-1	18	0.3833	-1	4	0	0
HIP_9192	GSHLVEALY	GREHIAKRA	GSHLVEALYGREHIAKRA		SCG2	5855/334890	0.999	9192	0.851	0.225968	-0.6833	0.1667	3	18	-0.4167	1	1	0	0
HIP_9193	AGSLQPLAL	FMSDTREEK	AGSLQPLALFMSDTREEK		SCG1	194/367830	1.000	9193	0.851	0.069574	2.2167	0.1667	-1	18	-0.3889	-1	1	0	0
HIP_9194	LVEALYLVC	HSQEKTHSR	LVEALYLVCHSQEKTHSR		SCG1	9089/367830	0.998	9194	0.851	0.191995	0.5	0.1667	2	18	-0.2667	0	1	0	0
HIP_9195	VCGERGFFY	VLNPHPSLG	VCGERGFFYVLNPHPSLG		CMGA	932/247050	1.000	9195	0.851	0.002213	1.4667	0	1	18	0.1611	0	3	0	0
HIP_9196	RGFFYTPKT	QWKSSHFER	RGFFYTPKTQWKSSHFER		SCG1	18932/367830	0.991	9196	0.851	0.061994	-2.4167	0.1667	4	18	-1.4333	3	2	0	0
HIP_9197	AGSLQPLAL	GQNGERATR	AGSLQPLALGQNGERATR		SCG2	1719/334890	1.000	9197	0.851	0.239449	0.3333	0.1667	1	18	-0.5833	1	0	0	0
HIP_9198	VCGERGFFY	GEEIKGYP	VCGERGFFYGEEIKGYP		SCG1	918/367830	1.000	9198	0.851	0.172381	-0.5167	0.1667	-1	17	-0.4647	-1	3	1	0
HIP_9199	CGERGFFYT	QDTKENRE	CGERGFFYTQDTKENRE		SCG1	10617/367830	0.997	9199	0.851	0.133018	-1.15	0	-1	17	-1.7235	-1	3	1	0
HIP_9200	HLVEALYLV	DMARYYSAL	HLVEALYLVDMARYYSAL		NPY	547/49410	0.997	9200	0.851	0.059067	1.15	0	0	18	0.6389	-1	1	0	0
HIP_9201	AGSLQPLAL	GKGRHHRGR	AGSLQPLALGKGRHHRGR		SCG1	8759/367830	0.998	9201	0.851	0.248950	0.7833	0.3333	6	18	-0.9056	4	0	0	0
HIP_9202	QHLCGSHLV	PNPYNQEKV	QHLCGSHLVPNPYNQEKV		SCG2	1026/334890	1.000	9202	0.851	0.221219	-0.3167	0.3333	2	18	-0.8333	0	1	0	0
HIP_9203	LVCGERGFF	MKRSGQLGI	LVCGERGFFMKRSGQLGI		SCG2	16668/334890	0.997	9203	0.851	0.078212	-0.2167	0.1667	2	18	0.2222	2	2	0	0
HIP_9204	CGERGFFY	QEEERLSK	CGERGFFYQEEERLSK		CMGA	3958/247050	0.999	9204	0.851	0.163858	-1.0333	0	-1	16	-1.3375	-1	3	0	1
HIP_9205	CSLYQLENY	GYEKRNLAR	CSLYQLENYGYEKRNLAR		SCG1	12915/367830	0.996	9205	0.851	-0.041357	-2.25	0.1667	1	18	-1.1	1	2	0	0
HIP_9206	NQHLCGSHL	YDWWEKKPF	NQHLCGSHLYDWWEKKPF		SCG1	14397/367830	0.995	9206	0.851	0.035206	-0.9833	0.1667	2	18	-1.1778	0	3	0	0
HIP_9207	LVCGERGFF	WKSSHFERR	LVCGERGFFWKSSHFERR		SCG1	13732/367830	0.996	9207	0.851	0.086492	-0.0667	0.3333	3	18	-0.6667	2	3	0	0
HIP_9208	LVEALYLVC	NPYNQEKVL	LVEALYLVCNPYNQEKVL		SCG2	2918/334890	1.000	9208	0.851	0.138761	0.6833	0.1667	-1	18	0.3611	-1	2	0	0
HIP_9209	AGSLQPLAL	ECFETLRGD	AGSLQPLALECFETLRGD		CMGA	1685/247050	1.000	9209	0.851	0.060248	1.8667	0	-2	18	0.0944	-2	1	0	0
HIP_9210	AGSLQPLAL	FPNPYNQEK	AGSLQPLALFPNPYNQEK		SCG2	601/334890	1.000	9210	0.851	0.180416	1.1833	0.1667	0	18	-0.6056	0	1	0	0
HIP_9211	SHLVEALYL	YYDPLQWK	SHLVEALYLYYDPLQWK		SCG1	11373/367830	0.997	9211	0.851	0.100047	0.0333	0	0	17	-0.2118	-1	3	1	0
HIP_9212	RGFFYTPKT	EEEDNRDSS	RGFFYTPKTEEEDNRDSS		CMGA	6276/247050	0.998	9212	0.851	-0.035736	-2.7833	0.1667	-2	18	-1.9222	-2	1	0	1
HIP_9213	HLVEALYL	PNPYNQEK	HLVEALYLPNPYNQEK		SCG2	1687/334890	1.000	9213	0.851	0.217497	-0.0667	0.3333	0	16	-0.8125	-1	2	0	0
HIP_9214	GSHLVEALY	EERLSKEWE	GSHLVEALYEERLSKEWE		CMGA	2952/247050	0.999	9214	0.851	0.133862	-1.2	0	-2	18	-0.8833	-3	1	0	0
HIP_9215	TSICSLYQL	DVNWGYEKR	TSICSLYQLDVNWGYEKR		SCG1	12173/367830	0.997	9215	0.851	0.074169	-0.6333	0	0	18	-0.5444	0	2	0	0
HIP_9216	GERGFFYTP	EEDWMRIIL	GERGFFYTPEEDWMRIIL		SCG2	6794/334890	0.999	9216	0.851	0.005062	-2.35	0.1667	-2	18	-0.4444	-2	4	0	0
HIP_9217	LVEALYLVC	QSAPKENKP	LVEALYLVCQSAPKENKP		SCG2	3147/334890	1.000	9217	0.851	0.172240	1.3333	0.1667	0	18	-0.0667	0	1	0	0
HIP_9218	GSHLVEALY	GRSRSNQLP	GSHLVEALYGRSRSNQLP		SCG2	3869/334890	1.000	9218	0.851	0.221794	-0.2333	0.3333	2	18	-0.6333	1	1	0	0
HIP_9219	SLQPLAL	YQLENYCN	SLQPLALYQLENYCN		INSA	58/3024	0.993	9219	0.851	0.096539	1.4	0	-1	15	-0.2	-1	1	1	0
HIP_9220	LVEALYLVC	YRAPRPQSE	LVEALYLVCYRAPRPQSE		SCG1	13750/367830	0.996	9220	0.851	0.092710	1.0833	0	0	18	-0.0111	0	2	0	0
HIP_9221	GSHLVEALY	EEERLSKEW	GSHLVEALYEEERLSKEW		CMGA	743/247050	1.000	9221	0.851	0.115609	-1.0333	0	-2	18	-0.8833	-3	1	0	1
HIP_9222	VCGERGFFY	DSQVSEEV	VCGERGFFYDSQVSEEV		SCG1	1255/367830	1.000	9222	0.851	0.162318	-0.5833	0.1667	-3	17	-0.2941	-3	3	1	0
HIP_9223	VCGERGFFY	LELDKMAHG	VCGERGFFYLELDKMAHG		SCG1	3695/367830	0.999	9223	0.851	0.002079	1.4	0	0	18	-0.0556	-1	3	0	0
HIP_9224	AGSLQPLAL	ANRRPEDQE	AGSLQPLALANRRPEDQE		CMGA	7976/247050	0.997	9224	0.851	0.170747	0.5333	0	-1	18	-0.9778	-1	0	0	0
HIP_9225	CGERGFFYT	ESRSEALAV	CGERGFFYTESRSEALAV		CMGA	7458/247050	0.997	9225	0.851	0.154764	-1.3333	0.1667	-1	18	-0.2333	-1	3	0	0
HIP_9226	SHLVEALY	QKTSYFPNP	SHLVEALYQKTSYFPNP		SCG2	13113/334890	0.998	9226	0.851	0.230337	-0.6333	0	1	17	-0.5471	0	2	1	0
HIP_9227	VCGERGFFY	PEMIRALEY	VCGERGFFYPEMIRALEY		SCG2	626/334890	1.000	9227	0.851	0.114109	0.1833	0.1667	-1	18	-0.0111	-1	3	0	0
HIP_9228	LVEALYLVC	EEVKTRHSE	LVEALYLVCEEVKTRHSE		SCG1	677/367830	1.000	9228	0.851	0.101169	1.2833	0	-1	18	-0.0056	-2	1	0	0
HIP_9229	VCGERGFFY	ENELTLNE	VCGERGFFYENELTLNE		SCG1	2193/367830	1.000	9229	0.851	0.133581	-1.0333	0	-3	17	-0.4941	-3	3	1	0
HIP_9230	AGSLQPLAL	LVKYPEIIN	AGSLQPLALLVKYPEIIN		SCG2	6401/334890	0.999	9230	0.851	0.066896	2.25	0	0	18	0.6611	0	1	0	0
HIP_9231	TSICSLYQL	QETDKLAPV	TSICSLYQLQETDKLAPV		SCG2	8092/334890	0.999	9231	0.851	0.174095	-1.45	0	-1	18	0.0333	-1	1	0	0
HIP_9232	VCGERGFFY	NWGYEKRN	VCGERGFFYNWGYEKRN		SCG1	5296/367830	0.999	9232	0.851	0.155291	-0.0833	0.1667	1	17	-1.1353	1	5	1	0
HIP_9233	CGERGFFY	PKTPGRAGT	CGERGFFYPKTPGRAGT		SCG2	10385/334890	0.999	9233	0.851	0.220549	-0.3167	0.1667	2	17	-0.8235	2	3	1	0
HIP_9234	CTSICSLYQ	NQLPKAAWI	CTSICSLYQNQLPKAAWI		SCG2	12086/334890	0.998	9234	0.851	0.045187	-0.7	0	1	18	0.2611	1	1	0	0
HIP_9235	LVCGERGFF	WSKMDQLAK	LVCGERGFFWSKMDQLAK		CMGA	7273/247050	0.997	9235	0.851	0.067571	-0.0667	0.3333	1	18	-0.0944	1	3	0	0

HIP_9699	HLVEALYLV	ELQKIAEK	HLVEALYLVELQKIAEK		SCG1	5014/367830	0.999	9699	0.851	0.159345	0.5833	0	0	17	0.3176	-1	1	1	0	0.000000	-3.3	-1	0.1	1.400000	0.2222	1.1778	0	0	0	0	3.3	0.687	-3.3	1	0	0	0	0.3718	1.2169	0.6878	0.3333	0.1382	0.4731	0.2169	0.01	-0.33
HIP_9700	VCGERGFFY	YNQEKVLP	VCGERGFFYYNQEKVLP		SCG2	10406/334890	0.999	9700	0.851	0.141913	-0.6667	0	0	17	-0.4176	0	4	1	0	0.000000	-4.2	0	0.1	0.700000	0.2222	0.4778	0	0	0	0	0	-0.3185	-16.8	0	1	0	0	0.4103	1.0372	0.9256	0.3333	0.1683	0.8568	-0.2735	0.01	-0.42
HIP_9701	CGERGFFY	LNEKNFFPE	CGERGFFYLNEKNFFPE		SCG1	8437/367830	0.998	9701	0.851	0.144101	0.1833	0	-1	17	-0.7118	-1	3	1	0	0.000000	-2.5	-1	0.1	0.000000	0.2222	-0.2222	0	0	0	0	2.5	-0.0407	-7.5	0	1	0	0	0.3718	1.1503	0.8178	0.3333	0.1382	0.6688	0.0682	0.01	-0.25
HIP_9702	LVCGERGFF	EEEEEATNT	LVCGERGFFEEEEEATNT		CMGA	176/247050	1.000	9702	0.851	0.033397	-0.8833	0.1667	-5	18	-0.7389	-5	2	0	3	0.000000	-5.2333	-3	0.0667	0.700000	0.6667	0.0333	0	1	0	1	15.7	-0.0294	-10.4667	1	0	0	0	0.4833	1.2383	0.82	-0.1667	0.2336	0.6724	-0.4269	0.0044	-0.3489
HIP_9703	GERGFFYTP	NRDSSMKLS	GERGFFYTPNRDSSMKLS		CMGA	10626/247050	0.994	9703	0.851	0.040284	-2.5167	0.1667	1	18	-1.05	1	3	0	0	0.000000	-2.6333	0	0.0333	0.700000	0.2222	0.4778	1	0	1	0	0	-1.2024	-7.9	0	0	0	1	0.5321	0.8558	1.1268	-0.3333	0.2831	1.2697	-1.339	0.0011	-0.0878
HIP_9704	CGERGFFYT	ADQTVLTED	CGERGFFYTADQTVLTED		SCG1	5185/367830	0.999	9704	0.851	0.028051	-0.7333	0	-3	18	-0.4611	-3	3	0	0	0.000000	-2	-1	0.05	0.450000	0.1111	0.3389	0	0	0	0	2	-0.2485	-6	0	1	0	0	0.4103	1.1055	0.8356	0.1667	0.1683	0.6983	-0.3009	0.0025	-0.1
HIP_9705	CTSICSLYQ	KFPSPEMIR	CTSICSLYQKFPSPEMIR		SCG2	8997/334890	0.999	9705	0.851	0.058414	-0.6167	0.1667	1	18	-0.0278	1	2	0	0	0.000000	-0.5667	1	0.0667	0.625000	0.1111	0.5139	1	0	1	0	-0.5667	-0.3169	-1.1333	0	1	0	0	0.4269	1.0769	0.9023	0.1667	0.1823	0.8142	-0.2633	0.0044	-0.0378
HIP_9706	CGERGFFYT	DNMNDNFLE	CGERGFFYTDNMNDNFLE		SCG1	4782/367830	0.999	9706	0.851	0.037499	-0.7167	0	-3	18	-0.8444	-3	3	0	0	0.000000	-1.9667	-1	0.05	0.000000	0.1111	-0.1111	0	0	0	0	1.9667	0.0796	-5.9	0	1	0	0	0.4103	0.9905	0.9223	0.1667	0.1683	0.8507	-0.294	0.0025	-0.0983
HIP_9707	HLVEALYLV	AANQKTSYF	HLVEALYLVAANQKTSYF		SCG2	4419/334890	1.000	9707	0.851	0.057577	1.1333	0	1	18	0.35	0	1	0	0	1.000000	-2.2	0	0.05	1.850000	0	1.85	0	0	0	0	0	2.0967	-2.2	1	0	0	0	0.3051	1.1586	0.7978	0.6667	0.0931	0.6365	0.3458	0.0025	-0.11
HIP_9708	VEALYLVC	WKELDRNYL	VEALYLVCWKELDRNYL		SCG1	13042/367830	0.996	9708	0.851	0.168789	0.3667	0	-1	17	0.1176	-1	2	1	0	0.000000	-6.2667	0	0.1	0.625000	0.3333	0.2917	0	0	0	0	0	0.1069	-12.5333	0	0	0	0	0.3821	1.1558	0.7435	0.1667	0.146	0.5527	0.1401	0.01	-0.6267
HIP_9709	SHLVEALYL	EEMAVVPQG	SHLVEALYLEEMAVVPQG		CMGA	1165/247050	1.000	9709	0.851	0.114780	0.2	0	-2	18	0.4556	-3	1	0	0	0.000000	-3.8	-2	0.1	0.450000	0.3333	0.1167	0	0	0	0	7.6	0.0233	-3.8	1	0	0	0	0.3718	1.3069	0.6645	0.1667	0.1382	0.4415	0.0744	0.01	-0.38
HIP_9710	HLCGSHLVE	NSNQVKRVP	HLCGSHLVENSNQVKRVP		SCG2	14078/334890	0.998	9710	0.851	0.202365	-0.55	0.1667	3	18	-0.5389	1	0	0	0	0.000000	-4.1	1	0.1167	1.050000	0.1111	0.9389	0	0	0	0	-4.1	-0.5164	0	0	0	1	0	0.4667	1.0017	1.01	0.1667	0.2178	1.0201	-0.2567	0.0136	-0.4783
HIP_9711	LVEALYLVC	FLVHSSNNF	LVEALYLVCFLVHSSNNF		IAPP	105/45018	1.000	9711	0.851	-0.003421	3.55	0	0	18	1.1722	-1	2	0	0	0.000000	0.1	0	0	2.700000	0	2.7	0	0	0	0	0	9.585	0.2	0	0	0	0	0.2385	1.1569	0.5845	0.8333	0.0569	0.3416	0.8465	0	0
HIP_9712	CGERGFFY	DSSEEKHLE	CGERGFFYDSSEEKHLE		SCG1	2322/367830	1.000	9712	0.851	0.200001	-0.1333	0.3333	-2	17	-1.2294	-3	3	1	0	0.000000	-3.1333	-1	0.1833	0.000000	0.3333	-0.3333	0	0	0	0	3.1333	0.0444	-9.4	0	1	0	0	0.4103	1.0072	0.9023	0.1667	0.1683	0.8142	-0.0547	0.0336	-0.5744
HIP_9713	SHLVEALYL	NRQYWDED	SHLVEALYLNRQYWDED		SCG2	10314/334890	0.999	9713	0.851	0.202726	-0.8667	0	-2	17	-0.9176	-3	2	1	0	0.000000	-5.9333	1	0.15	0.000000	0.2222	-0.2222	0	0	0	0	-5.9333	0.1926	-11.8667	1	0	0	0	0.4103	1.0705	0.8923	0.1667	0.1683	0.7962	-0.3556	0.0225	-0.89
HIP_9714	LVCGERGFF	SAEPGWQAK	LVCGERGFFSAEPGWQAK		CMGA	1948/247050	0.999	9714	0.851	0.048114	0.45	0.3333	0	18	-0.2056	0	2	0	0	0.000000	-2.5667	-1	0.0333	0.700000	0.3333	0.3667	0	1	0	1	2.5667	0.165	-5.1333	1	0	0	0	0.4167	1.11	0.8833	0.3333	0.1736	0.7803	0.1875	0.0011	-0.0856
HIP_9715	TSICSLYQL	QQQGDLQDT	TSICSLYQLQQQGDLQDT		SCG1	7423/367830	0.999	9715	0.851	0.156530	-1.9167	0	-2	18	-0.6	-2	1	0	1	0.000000	-3.1667	0	0.1	0.000000	0	0	0	0	0	0	0	0	-3.1667	1	0	0	0	0.5051	1.1269	0.8878	0.1667	0.2552	0.7882	-0.9682	0.01	-0.3167
HIP_9716	VCGERGFFY	DNPGEDAPA	VCGERGFFYDNPGEDAPA		NPY	54/49410	1.000	9716	0.851	0.167797	-0.7167	0.1667	-3	18	-0.6556	-3	3	0	0	0.000000	-4.3	-1	0.1667	0.700000	0.2222	0.4778	1	0	1	0	4.3	-0.3424	-12.9	0	1	0	0	0.4269	0.9472	1.0156	0.1667	0.1823	1.0315	-0.306	0.0278	-0.7167
HIP_9717	LYQLENYCN	KTHWLGAAL	LYQLENYCNKTHWLGAAL		SCG2	20064/334890	0.996	9717	0.851	0.066912	-1.6833	0	1	18	-0.2444	0	2	0	0	0.000000	-1.8333	1	0.05	0.625000	0.2222	0.4028	0	0	0	0	-1.8333	-0.678	-3.6667	0	1	0	0	0.4872	0.931	1.0246	-0.3333	0.2373	1.0498	-0.8201	0.0025	-0.0917
HIP_9718	CGERGFFY	GGHSRERAD	CGERGFFYGGHSRERAD		SCG1	3112/367830	1.000	9718	0.851	0.203973	0.05	0.3333	1	17	-1.2353	0	3	1	0	0.000000	-2.7667	0	0.1667	0.000000	0.1111	-0.1111	0	2	0	1	0	-0.0056	-8.3	0	1	0	0	0.4436	0.9072	1.0023	0.1667	0.1968	1.0046	0.0222	0.0278	-0.4611
HIP_9719	CGERGFFY	DKETTENEN	CGERGFFYDKETTENEN		SCG1	7842/367830	0.998	9719	0.851	0.181112	-1.1	0	-3	17	-1.6647	-3	3	1	0	0.000000	-5.0667	-1	0.15	0.000000	0.3333	-0.3333	0	0	0	0	5.0667	0.3667	-15.2	0	1	0	0	0.3821	1.1624	0.7768	-0.1667	0.146	0.6034	-0.4203	0.0225	-0.76
HIP_9720	GSHLVEALY	GWRPSSRED	GSHLVEALYGWRPSSRED		CMGA	3340/247050	0.999	9720	0.851	0.155777	-0.25	0.1667	0	18	-0.8944	-1	2	0	0	0.000000	-3.3667	1	0.1333	2.000000	0.2222	1.7778	0	1	0	1	-3.3667	-0.4444	-6.7333	0	1	0	0	0.3987	1.0524	0.8968	0.3333	0.159	0.8042	-0.0997	0.0178	-0.4489
HIP_9721	HLVEALYLV	EIINSNQVK	HLVEALYLVEIINSNQVK		SCG2	2424/334890	1.000	9721	0.851	0.027014	2.0333	0	0	18	0.45	-1	1	0	0	0.000000	-0.4	-1	0.05	1.400000	0.1111	1.2889	0	0	0	0	0.4	2.6207	-0.4	1	0	0	0	0.3051	1.1753	0.5978	0.5	0.0931	0.3574	0.6204	0.0025	-0.02
HIP_9722	AGSLQPLAL	EDNRDSSMK	AGSLQPLALEDNRDSSMK		CMGA	1260/247050	1.000	9722	0.850	0.160729	-0.1833	0	-1	18	-0.7444	-1	0	0	0	0.000000	-6.6333	-2	0.15	0.450000	0.3333	0.1167	0	0	0	0	13.2667	-0.0214	0	0	1	0	0	0.3718	1.2086	0.8112	0.1667	0.1382	0.658	-0.0682	0.0225	-0.995
HIP_9723	SLYQLENYC	RQQAHKEES	SLYQLENYCRQQAHKEES		SCG2	15332/334890	0.998	9723	0.850	0.102555	-2.3	0	0	18	-1.5778	-1	1	0	0	0.000000	-3.0667	1	0.1	1.400000	0.2222	1.1778	0	0	0	0	-3.0667	-2.7089	-3.0667	0	0	0	0	0.5154	0.9924	1.0401	-0.1667	0.2656	1.0819	-1.1854	0.01	-0.3067
HIP_9724	LVCGERGFF	HESSSRGE	LVCGERGFFHESSSRGE		SCG1	6724/367830	0.999	9724	0.850	0.057372	-0.3833	0.3333	0	17	-0.6	-1	2	1	1	0.000000	-4.2333	-1	0.0333	0.700000	0.3333	0.3667	0	1	0	1	4.2333	-0.1406	-8.4667	1	0	0	0	0.4551	1.0386	0.9245	0	0.2071	0.8547	-0.1745	0.0011	-0.1411
HIP_9725	CGERGFFYT	LRKESKDQL	CGERGFFYTLRKESKDQL		SCG2	19936/334890	0.996	9725	0.850	0.086535	-0.6333	0	1	18	-1.0389	1	3	0	0	0.000000	-1.8	2	0.05	0.950000	0.3333	0.6167	0	0	0	0	-3.6	-0.3906	-5.4	0	1	0	0	0.3821	1.0658	0.7968	0	0.146	0.6349	-0.242	0.0025	-0.09
HIP_9726	VCGERGFFY	SSSRGEAGA	VCGERGFFYSSSRGEAGA		SCG1	1166/367830	1.000	9726	0.850	0.213928	0.3167	0.5	0	18	-0.3	0	3	0	1	0.000000	-2.2333	0	0.2	0.700000	0.2222	0.4778	0	0	0	0	0	0.1513	-6.7	0	1	0	0	0.4385	0.9703	0.9512	0.3333	0.1922	0.9047	0.1388	0.04	-0.4467
HIP_9727	SLYQLENYC	AWKELDRNY	SLYQLENYCAWKELDRNY		SCG1	10053/367830	0.998	9727	0.850	-0.105225	-0.8833	0	-1	18	-1.0722	-1	2	0	0	0.000000	-0.2333	1	0	1.400000	0.3333	1.0667	0	0	0	0	-0.2333	-0.9422	-0.4667	0	0	0	0	0.4205	1.0344	0.9746	0.1667	0.1768	0.9499	-0.3715	0	0
HIP_9728	SHLVEALYL	TYGKRNAVE	SHLVEALYLTYGKRNAVE		IAPP	553/45018	0.998	9728	0.850	0.210514	0.65	0.1667	1	18	-0.1778	0	2	0	0	0.000000	-2.9	0	0.1333	0.000000	0.2222	-0.2222	0	1	0	1	0	-0.1644	-5.8	1	0	0	0	0.4269	0.9955	0.879	0.3333	0.1823	0.7726	0.2775	0.0178	-0.3867
HIP_9729	SHLVEALYL	ATDGARPQA	SHLVEALYLATDGARPQA		CMGA	3901/247050	0.999	9729	0.850	0.177848	0.65	0	0	18	-0.0111	-1	1	0	0	1.000000	-2.9	-1	0.1	0.450000	0.2222	0.2278	0	0	0	0	2.9	0.1481	-2.9	1	0	0	0	0.3436	1.1538	0.739	0.3333	0.1181	0.5461	0.2233	0.01	-0.29
HIP_9730	HLVEALYLV	EEATNTHPP	HLVEALYLVEEATNTHPP		CMGA	688/247050	1.000	9730	0.850	0.100167	0.25	0	-1	18	-0.1611	-3	1	0	0	0.000000	-3.9667	-2	0.1	1.400000	0.2222	1.1778	0	0	0	0	7.9333	0.2944	-3.9667	1	0	0	0	0.3718	1.3086	0.6745	0.1667	0.1382	0.4549	0.0929	0.01	-0.3967
HIP_9731	QHLCGSHLV	WIPHVENRQ	QHLCGSHLVWIPHVENRQ		SCG2	3148/334890	1.000	9731	0.850	0.070743	1.1333	0.1667	3	18	-0.4889	0	1	0	0	1.000000	-0.9333	0	0.0667	0.950000	0	0.95	1	0	1	0	0	1.0767	-0.9333	1	0	0	0	0.3603	1.0088	0.8006	0.5	0.1298	0.641	0.4083	0.0044	-0.0622
HIP_9732	CSLYQLENY	FQRNQLLQK	CSLYQLENYFQRNQLLQK		SCG2	16367/334890	0.997	9732	0.850	-0.070013	-2.25	0	1	18	-0.8778	1	2	0	0	0.000000	1.0333	2	0	0.700000	0.2222	0.4778	0	0	0	0	2.0667	-1.075	2.0667	0	1	0	0	0.4769	1.0888	0.949	-0.1667	0.2275	0.9006	-1.0731	0	0
HIP_9733	LVEALYLVC	QKTSYFFNP	LVEALYLVCQKTSYFFNP		SCG2	10151/334890	0.999	9733	0.850	0.209895	0.4	0	0	18	0.2889	0	1	0	0	0.000000	-6.2	1	0.15	2.000000	0.1111	1.8889	0	0	0	0	-6.2	0.7556	-6.2	0	0	0	0	0.4103	1.0572	0.7923	0.1667	0.1683	0.6278	0.1641	0.0225	-0.93
HIP_9734	HLVEALYL	PYNQEKVLP	HLVEALYLPYNQEKVLP		SCG2	4529/334890	1.000	9734	0.850	0.153884	-0.0167	0.1667	0	17	-0.0882	-1	2	1	0	0.000000	-4.2333	0	0.1167	1.400000	0.1111	1.2889	1	0	1	0	0	-0.0215	-8.4667	1	0	0	0	0.4269	0.9805	0.9823	0.3333	0.1823	0.9649	-0.0071	0.0136	-0.4939
HIP_9735	VEALYLVCG	EYRAPRPQS	VEALYLVCGEYRAPRPQS		SCG1	12617/367830	0.996	9735	0.850	0.052720	-0.5	0.1667	0	18	-0.2444	0	2	0	0	0.000000	-5.2	0	0.0167	1.075000	0.2222	0.8528	0	1	0	1	0	-0.4264	-10.4	0	0	0	1	0.4654	1.0224	0.9101	-0.1667	0.2166	0.8283	-0.2327	0.0003	-0.0867
HIP_9736	ERGFFYTPK	HGRSRPDRS	ERGFFYTPKHGRSRPDRS		SCG1	16931/367830	0.993	9736	0.850	-0.007829	-2.3833	0.3333	4	18	-1.8944	3	2	0	0	0.000000	-0.6333	0	-0.0333	0.700000	0.2222	0.4778	1	1	1	1	0	-1.1387	-1.2667	0	0	1	0	0.5487	0.8274	1.1168	-0.5	0.3011	1.2472	-1.3078	0.0011	0.0211
HIP_9737	HLVEALYL	WDEEDKRN	HLVEALYLWDEEDKRN		SCG1	5574/367830	0.999	9737	0.850	0.118763	-0.2667	0	-2	16	-1.0875	-3	2	0	0	1.000000	-4.7333	-2	0.1	0.000000	0.4444	-0.4444	0	0	0	0	9.4667	0.1185	-9.4667	1	0	0	0	0.3821	1.1958	0.7435	0.1667	0.146	0.5527	-0.1019	0.01	-0.4733
HIP_9738	LVEALYLVC	QECFETLRG	LVEALYLVCQECFETLRG		CMGA	2021/247050	0.999	9738	0.850	0.098852	1	0	-2	18	0.6833	-2	2	0	0	0.000000	-5	-1	0.1	2.700000	0.1111	2.5889	0	0	0	0	5	2.5889	-10	0	0	0	0	0.4103	1.1705	0.7556	0.1667	0.1683	0.571	0.4103	0.01	-0.5
HIP_9739	SHLVEALYL	FPEYNYDW	SHLVEALYLFPEYNYDW		SCG1	870/367830	1.000	9739	0.850	0.126772	0.6667	0.1667	-2	17	-0.2471	-3	3	1	0	1.000000	-2.8667	-1	0.1167	0.700000	0.2222	0.4778	1	0	1	0	2.8667	0.3185	-8.6	1	0	0	0	0.3885	1.1503	0.7945	0.3333	0.1509	0.6312	0.259	0.0136	-0.3344
HIP_9740	CSLYQLENY	RQYDRVAQL	CSLYQLENYRQYDRVAQL		SCG1	23374/367830	0.984	9740	0.850	-0.001967	-2.9333	0	0	18	-0.8222	0	2	0	0	0.000000	-0.3333	2	0	0.000000	0.3333	-0.3333	0	0	0	0	-0.6667	0.9778	-0.6667	0	1	0	0	0.5154	1.0591	1.0001	-0.3333	0.2656	1.0003	-1.5118	0	0
HIP_9741	VCGERGFFY	SHFERRDN	VCGERGFFYSHFERRDN		SCG1	11597/367830	0.997	9741	0.850	0.097888	0.5167	0.1667	1	17	-1.0882	0	4	1	0	0.000000	-1.8333	0	0.0667	0.700000	0.2222	0.4778	0	0	0	0	0	0.2469	-7.3333	0	1	0	0	0.3436	1.0738	0.8023	0.3333	0.1181	0.6437	0.1775	0.0044	-0.1222
HIP_9742	ERGFFYTPK	KNDDTPNRQ	ERGFFYTPKKNDDTPNRQ		SCG2	16840/334890	0.997	9742	0.850	-0.008953	-2.85	0.1667	1	18	-2.1389	1	2	0	0	0.000000	-1.5667	-1	-0.0167	0.700000	0.4444	0.2556	1	0	1	0	1.5667	-0.7283	-3.1333	0	0	0	1	0.5321	0.8558	1.1268	-0.5	0.2831	1.2697	-1.5163	0.0003	0.0261
HIP_9743	GERGFFYTP	KKELENLAA	GERGFFYTPKKELENLAA		SCG1	17431/367830	0.993	9743	0.850	0.009129	-2.4833	0.1667	0	18	-0.7722	0	3	0	0	0.000000	-2.5667	1	0.0333	1.650000	0.3333	1.3167	1	0	1	0	-2.5667	-3.2697	-7.7	0	0	0	1	0.5321	0.9958	0.9901	-0.5	0.2831	0.9804	-1.3213	0.0011	-0.0856
HIP_9744	ERGFFYTPK	EGKGEQDHS	ERGFFYTPKEGKGEQDHS		CMGA	5408/247050	0.998	9744	0.850	-0.081668	-2.3333	0.3333	0	18	-1.8333	-1	2	0	0	0.000000	-0.5333	-1	0.0167	0.700000	0.3333	0.3667	1	1	1	1	0.5333	-0.8556	-1.0667	0	0	1	0	0.5769	0.9088	1.089	-0.5	0.3328	1.1859	-1.3462	0.0003	-0.0089
HIP_9745	SLYQLENYC	EEEGPTVVL	SLYQLENYCEEEGPTVVL		CMGA	1172/247050	1.000	9745	0.850	0.038190	-2.1333	0	-4	18	-0.2667	-4	1	0	1	0.000000	-2.7333	-3	0.1	0.950000	0.4444	0.5056	0	0	0	0	8.2	-1.0785	-2.7333	0	0	0	0	0.5436	1.2072	0.9323	-0.5	0.2955	0.8692	-1.1597	0.01	-0.2733
HIP_9746	LVCGERGFF	RSQEESEEG	LVCGERGFFRSQEESEEG		SCG1	6943/367830	0.999	9746	0.850	0.095432	-0.6	0.3333	-3	18	-0.9278	-3	2	0	0	0.000000	-4.6667	1	0.0833	0.700000	0.4444	0.2556	0	1	0	1	-4.6667	-0.1533	-9.3333	1	0	0	0	0.4551	0.9719	0.9645	0.1667	0.2071	0.9302	-0.2731	0.0069	-0.3889
[illegible]																																														

HIP_10210	LVCGERGFF	AENEPQSAP	LVCGERGFFAENEPQSAP		SCG2	1510/334890	1.000	10210	0.850	0.011414	0	0.1667	-2	18	-0.3944	-2	2	0	0
HIP_10211	GERGFFYTP	PEEEGPTVV	GERGFFYTPPEEEGPTVV		CMGA	620/247050	1.000	10211	0.850	-0.035327	-2.0333	0.3333	-3	18	-0.7333	-3	3	0	1
HIP_10212	AGSLQPLAL	RNLARVPKL	AGSLQPLALRNLARVPKL		SCG1	19277/367830	0.990	10212	0.850	0.203042	0.8667	0	3	18	0.2389	3	0	0	0
HIP_10213	HLVEALYLV	LPYGAGRSR	HLVEALYLVLPYGAGRSR		SCG2	3246/334890	1.000	10213	0.850	0.063404	1.2667	0.1667	2	18	0.3167	1	2	0	0
HIP_10214	HLVEALYL	WGYEKRNLA	HLVEALYLWGYEKRNLA		SCG1	5874/367830	0.999	10214	0.850	0.108688	0.6167	0.1667	1	17	-0.1765	0	3	1	0
HIP_10215	VCGERGFFY	NELTLNEK	VCGERGFFYNELTLNEK		SCG1	7471/367830	0.999	10215	0.850	0.132567	0.1833	0	-1	17	-0.5176	-1	3	1	0
HIP_10216	GSHLVEALY	ELDKMAHGY	GSHLVEALYELDKMAHGY		SCG1	3130/367830	1.000	10216	0.850	0.096355	0.1833	0	0	18	-0.2167	-2	1	0	0
HIP_10217	VCGERGFFY	LSDDVSKVI	VCGERGFFYLSDDVSKVI		SCG2	768/334890	1.000	10217	0.850	0.129075	0.6333	0.1667	-1	18	0.3556	-1	3	0	0
HIP_10218	CCTSICSLY	NRQYWDEDL	CCTSICSLYNRQYWDEDL		SCG2	10517/334890	0.999	10218	0.850	0.059844	-1.6333	0.1667	-2	18	-0.4556	-2	2	0	0
HIP_10219	HLCGSHLVE	YLNQEKAEK	HLCGSHLVEYLNQEKAEK		SCG2	13642/334890	0.998	10219	0.850	0.013882	0.5833	0	1	18	-0.7944	-1	1	0	0
HIP_10220	QHLCGSHLV	NSNQVKRVP	QHLCGSHLVNSNQVKRVP		SCG2	7842/334890	0.999	10220	0.850	0.241750	-0.5	0.1667	4	18	-0.5389	2	0	0	0
HIP_10221	LVEALYLVC	KFPSPEMIR	LVEALYLVCKFPSPEMIR		SCG2	7188/334890	0.999	10221	0.850	0.151492	1.3	0.1667	0	18	0.7	0	2	0	0
HIP_10222	CTSICSLYQ	ENEPQSAPK	CTSICSLYQENEPQSAPK		SCG2	7176/334890	0.999	10222	0.850	0.109021	-1.9167	0	-1	18	-0.7722	-1	1	0	0
HIP_10223	AGSLQPLAL	NGERATRLF	AGSLQPLALNGERATRLF		SCG2	1044/334890	1.000	10223	0.850	0.228559	0.3333	0.1667	1	18	0	1	0	0	0
HIP_10224	HLVEALYLV	YGEEGAPGK	HLVEALYLVYGEEGAPGK		SCG1	192/367830	1.000	10224	0.850	0.154512	0.25	0.1667	-1	18	0.0222	-2	2	0	0
HIP_10225	AGSLQPLAL	LELQKIAEK	AGSLQPLALLELQKIAEK		SCG1	4965/367830	0.999	10225	0.850	0.047976	2.25	0	0	18	0.2389	0	0	0	0
HIP_10226	GERGFFYTP	SKGQPRSQE	GERGFFYTPSKGQPRSQE		SCG1	2141/367830	1.000	10226	0.850	0.006496	-1.45	0.5	1	18	-1.6278	1	3	0	0
HIP_10227	SHLVEALYL	EHSQQKEEE	SHLVEALYLEHSQQKEEE		CMGA	1926/247050	0.999	10227	0.850	0.164540	-0.2	0.1667	-2	18	-1.1278	-4	1	0	1
HIP_10228	CSLYQLENY	EEEEEEAEA	CSLYQLENYEEEEEEAEA		CMGA	2005/247050	0.999	10228	0.850	-0.055828	-3.1333	0	-8	18	-1.3722	-8	1	0	4
HIP_10229	HLVEALYLV	FQELGKLTG	HLVEALYLVFQELGKLTG		SCG2	3408/334890	1.000	10229	0.850	0.096417	0.4167	0	0	18	0.6444	-1	2	0	0
HIP_10230	AGSLQPLAL	KPDNPGEDA	AGSLQPLALKPDNPGEDA		NPY	403/49410	0.998	10230	0.850	0.221930	0.0667	0.1667	-2	18	-0.6111	-2	0	0	0
HIP_10231	SICSLYQLE	PYQGVSVPL	SICSLYQLEPYQGVSVPL		SCG2	7471/334890	0.999	10231	0.850	0.101519	-1.6	0.1667	-1	18	0.4278	-1	2	0	0
HIP_10232	VCGERGFFY	SKDVMEKRE	VCGERGFFYSKDVMEKRE		CMGA	6087/247050	0.998	10232	0.850	0.194776	-0.65	0.1667	0	18	-0.85	0	3	0	0
HIP_10233	VCGERGFFY	SSMKLSFR	VCGERGFFYSSMKLSFR		CMGA	4747/247050	0.999	10233	0.850	0.201731	0.7667	0.3333	2	17	-0.0059	2	3	1	0
HIP_10234	VCGERGFFY	LIVLSVALN	VCGERGFFYLIVLSVALN		IAPP	64/45018	1.000	10234	0.850	-0.045216	2.8	0	0	18	1.3333	0	3	0	0
HIP_10235	TSICSLYQL	AILSSTNVG	TSICSLYQLAILSSTNVG		IAPP	6/45018	1.000	10235	0.850	0.004240	1.5167	0	0	18	0.8667	0	1	0	0
HIP_10236	SHLVEALYL	QNGERATR	SHLVEALYLQNGERATR		SCG2	3894/334890	1.000	10236	0.850	0.254576	-0.1833	0.1667	1	17	-0.6	0	1	1	0
HIP_10237	GERGFFYTP	WQQQGDLQD	GERGFFYTPWQQQGDLQD		SCG1	4224/367830	0.999	10237	0.850	-0.046122	-1.9167	0.1667	-2	18	-1.4056	-2	4	0	1
HIP_10238	ERGFFYTPK	RRTRPRHHH	ERGFFYTPKRRTRPRHHH		SCG1	27863/367830	0.972	10238	0.850	0.053329	-2.65	0.1667	8	18	-2.2333	5	2	0	1
HIP_10239	VCGERGFFY	KLAPVSKR	VCGERGFFYKLAPVSKR		SCG2	17133/334890	0.997	10239	0.850	0.105858	1	0	3	17	-0.1588	3	3	1	0
HIP_10240	GERGFFYTP	EQKLYTDDE	GERGFFYTPEQKLYTDDE		SCG2	8750/334890	0.999	10240	0.850	-0.037895	-2.4167	0.1667	-3	18	-1.4667	-3	3	0	0
HIP_10241	AGSLQPLAL	SKPDNPGED	AGSLQPLALSKPDNPGED		NPY	368/49410	0.999	10241	0.850	0.219459	0.5167	0.3333	-2	18	-0.7556	-2	0	0	0
HIP_10242	TSICSLYQL	LLHYRKKSA	TSICSLYQLLLHYRKKSA		SCG1	11043/367830	0.997	10242	0.850	0.029308	0.5667	0	4	18	-0.0389	3	2	0	1
HIP_10243	VCGERGFFY	SGEATDGAR	VCGERGFFYSGEATDGAR		CMGA	129/247050	1.000	10243	0.850	0.191497	-0.0667	0.3333	-1	18	-0.4444	-1	3	0	0
HIP_10244	GSLQPLAL	PRLPYGAGR	GSLQPLALPRLPYGAGR		SCG2	12058/334890	0.998	10244	0.850	0.214388	1.1833	0.1667	2	17	-0.1059	2	1	1	0
HIP_10245	CGERGFFY	GEDSSEEKH	CGERGFFYGEDSSEEKH		SCG1	1250/367830	1.000	10245	0.850	0.183803	-0.5167	0.1667	-2	17	-1.4765	-3	3	1	0
HIP_10246	SHLVEALYL	ENENTKFEV	SHLVEALYLENENTKFEV		SCG1	2676/367830	1.000	10246	0.850	0.170822	-0.7	0	-2	18	-0.3611	-3	1	0	0
HIP_10247	SHLVEALYL	ENQSSQAE	SHLVEALYLENQSSQAE		CMGA	2822/247050	0.999	10247	0.850	0.207688	-0.7	0	-2	17	-0.5118	-3	1	1	0
HIP_10248	VEALYLVC	YGEEIKGYP	VEALYLVCYGEEIKGYP		SCG1	1017/367830	1.000	10248	0.850	0.148539	0.8833	0.1667	-2	17	0.2412	-2	2	1	0
HIP_10249	CTSICSLYQ	NVQKFPSPE	CTSICSLYQNVQKFPSPE		SCG2	9516/334890	0.999	10249	0.850	0.021219	-0.6333	0	0	18	-0.2889	0	1	0	0
HIP_10250	LYQLENYCN	YYDPLQWKS	LYQLENYCNYYDPLQWKS		SCG1	12816/367830	0.996	10250	0.850	-0.016022	-1.4	0	-1	18	-1.0833	-1	3	0	0
HIP_10251	CGERGFFYT	LAVDGAGKP	CGERGFFYTLAVDGAGKP		CMGA	571/247050	1.000	10251	0.850	-0.041255	1.7667	0	0	18	-0.05	0	3	0	0
HIP_10252	ERGFFYTPK	QKGERGEDS	ERGFFYTPKQKGERGEDS		SCG1	8634/367830	0.998	10252	0.850	-0.072568	-2.3333	0.3333	0	18	-1.9056	0	2	0	0

HIP_10721	GSHLVEALY	QINDEMKRS	GSHLVEALYQINDEMKRS		SCG2	2563/334890	1.000	10721	0.849	0.125475	0.3	0	0	18	-0.6889	-1	1	0	0	[illegible]
HIP_10722	LVCGERGFF	SSQETDKLA	LVCGERGFFSSQETDKLA		SCG2	420/334890	1.000	10722	0.849	0.125649	0.0167	0.5	-1	18	-0.2111	-1	2	0	0	[illegible]
HIP_10723	LVEALYLVC	EERLSKEWE	LVEALYLVCEERLSKEWE		CMGA	2704/247050	0.999	10723	0.849	0.151144	-0.1667	0	-3	18	-0.0556	-3	1	0	0	[illegible]
HIP_10724	TSICSLYQL	SSHFERRDN	TSICSLYQLSSHFERRDN		SCG1	9915/367830	0.998	10724	0.849	0.104331	-0.9667	0.3333	1	18	-0.7778	0	2	0	0	[illegible]
HIP_10725	TSICSLYQL	DERILSILR	TSICSLYQLDERILSILR		CMGA	10607/247050	0.994	10725	0.849	0.146014	-2.0833	0	0	18	0.4056	0	1	0	0	[illegible]
HIP_10726	TSICSLYQL	EDLRKESKD	TSICSLYQLEDLRKESKD		SCG2	14234/334890	0.998	10726	0.849	0.061110	-0.7	0	-1	18	-0.8778	-1	1	0	0	[illegible]
HIP_10727	GPGAGSLQP	EDNRDSSMK	GPGAGSLQPEDNRDSSMK		CMGA	4503/247050	0.999	10727	0.849	-0.012938	-1.9667	0.1667	-1	18	-1.4	-1	0	0	0	[illegible]
HIP_10728	ERGFFYTPK	QESGEETGS	ERGFFYTPKQESGEETGS		SCG1	6178/367830	0.999	10728	0.849	-0.113944	-2.3333	0.3333	-2	18	-1.5222	-2	2	0	0	[illegible]
HIP_10729	ERGFFYTPK	SQESGEETG	ERGFFYTPKSQESGEETG		SCG1	6179/367830	0.999	10729	0.849	-0.113944	-2.3333	0.3333	-2	18	-1.5222	-2	2	0	0	[illegible]
HIP_10730	CGERGFFY	DEEDKRNYP	CGERGFFYDEEDKRNYP		SCG1	6411/367830	0.999	10730	0.849	0.150558	-1.0333	0	-2	17	-1.8118	-2	3	1	0	[illegible]
HIP_10731	HLVEALYLV	REDSLEAGL	HLVEALYLVREDSLEAGL		CMGA	3567/247050	0.999	10731	0.849	0.181727	-0.8	0	-2	18	0.3778	-3	1	0	0	[illegible]
HIP_10732	CGERGFFYT	ENLNDKDQE	CGERGFFYTENLNDKDQE		SCG2	4242/334890	1.000	10732	0.849	0.016451	-0.4	0	-3	18	-1.5167	-3	3	0	0	[illegible]
HIP_10733	VCGERGFFY	RGSEEYRA	VCGERGFFYRGSEEYRA		SCG1	6634/367830	0.999	10733	0.849	0.213788	-0.2333	0.3333	0	17	-0.8529	0	3	1	0	[illegible]
HIP_10734	CCTSICSLY	EEEGSANRR	CCTSICSLYEEEGSANRR		CMGA	3796/247050	0.999	10734	0.849	0.025902	-1.4667	0.1667	-1	18	-0.5667	-1	1	0	1	[illegible]
HIP_10735	VCGERGFFY	LQVRGYPEE	VCGERGFFYLQVRGYPEE		CMGA	672/247050	1.000	10735	0.849	-0.014946	1.4667	0	-1	18	-0.45	-1	3	0	0	[illegible]
HIP_10736	SLYQLENYC	NQVKRVPGQ	SLYQLENYCNQVKRVPGQ		SCG2	14242/334890	0.998	10736	0.849	0.008278	-0.85	0	1	18	-0.9056	1	1	0	0	[illegible]
HIP_10737	LCGSHLVEA	PERKLKHMQ	LCGSHLVEAPERKLKHMQ		SCG2	20181/334890	0.996	10737	0.849	0.228918	-1.1833	0.1667	3	18	-0.5667	1	0	0	0	[illegible]
HIP_10738	VCGERGFFY	SDSQVSEEV	VCGERGFFYSDSQVSEEV		SCG1	625/367830	1.000	10738	0.849	0.184630	-0.1333	0.3333	-3	18	-0.3222	-3	3	0	0	[illegible]
HIP_10739	AGSLQPLAL	EEERLSKEW	AGSLQPLALEEERLSKEW		CMGA	1689/247050	1.000	10739	0.849	0.147810	-0.1833	0	-2	18	-0.6444	-2	0	0	1	[illegible]
HIP_10740	AGSLQPLAL	SDTREEKRF	AGSLQPLALSDTREEKRF		SCG1	3037/367830	1.000	10740	0.849	0.229803	0.7333	0.1667	0	18	-0.7444	0	0	0	0	[illegible]
HIP_10741	RGFFYTPKT	SSPDYNPYQ	RGFFYTPKTSSPDYNPYQ		SCG2	5533/334890	0.999	10741	0.849	-0.056506	-1.5667	0.6667	1	18	-1.4111	1	1	0	0	[illegible]
HIP_10742	VCGERGFFY	GEEDIQGP	VCGERGFFYGEEDIQGP		SCG1	639/367830	1.000	10742	0.849	0.174360	-0.5167	0.1667	-3	17	-0.5706	-3	3	1	0	[illegible]
HIP_10743	SHLVEALYL	FSEDVNWGY	SHLVEALYLFSEDVNWGY		SCG1	274/367830	1.000	10743	0.849	0.146081	0.8	0.1667	-2	18	0.0944	-3	2	0	0	[illegible]
HIP_10744	AGSLQPLAL	ETQQWPER	AGSLQPLALETQQWPER		SCG2	3574/334890	1.000	10744	0.849	0.209571	0.2833	0	-1	17	-0.7647	-1	0	1	0	[illegible]
HIP_10745	SHLVEALYL	EMAWPQGL	SHLVEALYLEMAWPQGL		CMGA	2263/247050	0.999	10745	0.849	0.045151	1.0833	0	-1	18	0.8611	-2	1	0	0	[illegible]
HIP_10746	VCGERGFFY	QQQGDLQD	VCGERGFFYQQQGDLQD		SCG1	7703/367830	0.998	10746	0.849	0.183147	-1.0333	0	-2	17	-0.9059	-2	3	1	1	[illegible]
HIP_10747	SHLVEALY	VNWGYEKRN	SHLVEALYVNWGYEKRN		SCG1	11233/367830	0.997	10747	0.849	0.092825	0.6833	0	1	17	-0.7353	0	3	1	0	[illegible]
HIP_10748	GERGFFYT	EEGSANRRP	GERGFFYTEEGSANRRP		CMGA	3798/247050	0.999	10748	0.849	0.133858	-1.1	0.1667	0	17	-1.5118	0	3	1	0	[illegible]
HIP_10749	LVCGERGFF	EEYRAPRP	LVCGERGFFEEYRAPRP		SCG1	8982/367830	0.998	10749	0.849	0.046031	-0.5167	0.1667	0	17	-0.6706	0	3	1	0	[illegible]
HIP_10750	LVCGERGFF	SWDEEDKRN	LVCGERGFFSWDEEDKRN		SCG1	4078/367830	0.999	10750	0.849	0.037375	0	0.3333	-2	18	-1.1278	-2	3	0	0	[illegible]
HIP_10751	SHLVEALYL	WSLYPSDS	SHLVEALYLWSLYPSDS		SCG1	2657/367830	1.000	10751	0.849	0.081594	1.4	0.1667	-1	17	0.1588	-2	3	1	0	[illegible]
HIP_10752	VCGERGFFY	LSQGLVDRE	VCGERGFFYLSQGLVDRE		CMGA	349/247050	1.000	10752	0.849	0.124810	0.6333	0.1667	-1	18	-0.1222	-1	3	0	0	[illegible]
HIP_10753	GSHLVEALY	DLRLENVQK	GSHLVEALYDLRLENVQK		SCG2	7564/334890	0.999	10753	0.849	0.103959	0.0167	0	0	18	-0.3444	-1	1	0	0	[illegible]
HIP_10754	ERGFFYTPK	NRDSSMKLS	ERGFFYTPKNRDSSMKLS		CMGA	13649/247050	0.990	10754	0.849	0.004839	-2.95	0.1667	2	18	-1.2444	2	2	0	0	[illegible]
HIP_10755	CGERGFFYT	PEDQELESL	CGERGFFYTPEDQELESL		CMGA	1108/247050	1.000	10755	0.849	0.095634	-1.3	0.1667	-4	18	-0.8333	-4	3	0	0	[illegible]
HIP_10756	VEALYLVC	YDWWEKKPF	VEALYLVCYDWWEKKPF		SCG1	8923/367830	0.998	10756	0.849	0.086293	0.8	0	-1	17	-0.0706	-1	4	1	0	[illegible]
HIP_10757	HLCGSHLVE	QMAYENLND	HLCGSHLVEQMAYENLND		SCG2	6289/334890	0.999	10757	0.849	-0.024833	0.7833	0	-1	18	-0.45	-3	1	0	0	[illegible]
HIP_10758	GERGFFYT	PSEEKGHPQ	GERGFFYTPSEEKGHPQ		SCG1	4531/367830	0.999	10758	0.849	0.149226	-0.85	0.3333	0	17	-1.6	-1	3	1	0	[illegible]
HIP_10759	CGERGFFY	FSEDVNWGY	CGERGFFYFSEDVNWGY		SCG1	3347/367830	1.000	10759	0.849	0.121132	0.4667	0.1667	-2	17	-0.5235	-2	4	1	0	[illegible]
HIP_10760	HLVEALYL	PYGAGRSRS	HLVEALYLPYGAGRSRS		SCG2	1470/334890	1.000	10760	0.849	0.194709	0.5	0.3333	2	17	-0.1824	1	2	1	0	[illegible]
HIP_10761	RGFFYTPKT	WQQQGDLQD	RGFFYTPKTWQQQGDLQD		SCG1	6240/367830	0.999	10761	0.849	-0.006613	-2.35	0.1667	0	18	-1.4444	0	2	0	1	[illegible]
HIP_10762	CGERGFFYT	NMNDNFLEG	CGERGFFYTNMNDNFLEG		SCG1	6706/367830	0.999	10762	0.849	0.050549	-0.7167	0	-2	18	-0.6722	-2	3	0	0	[illegible]
HIP_10763	VCGERGFFY	MASLGEKR	VCGERGFFYMASLGEKR		SCG1	4493/367830	0.999	10763	0.849	0.041573	1.2	0.1667	1	17	-0.2	1	3	1	0	[illegible]
HIP_10764	HLVEALYLV	PKAAWIPHV	HLVEALYLVPKAAWIPHV		SCG2	7167/334890	0.999	10764	0.849	0.174611	0.5	0.1667	2	18	0.8167	0	1	0	0	[illegible]
HIP_10765	SHLVEALYL	EATDGARPQ	SHLVEALYLEATDGARPQ		CMGA	3999/247050	0.999	10765	0.849	0.167928	0.65	0	-1	18	-0.3056	-2	1	0	0	[illegible]
HIP_10766	YLVCGERGF	YPKTPGRAG	YLVCGERGFYPKTPGRAG		SCG2	12078/334890	0.998	10766	0.849	0.036306	-1.4833	0.3333	2	18	-0.5222	2	2	0	0	[illegible]
HIP_10767	CTSICSLYQ	AIKEHLNQG	CTSICSLYQAIKEHLNQG		SCG2	9144/334890	0.999	10767	0.849	-0.041412	0.2333	0	1	18	-0.0944	0	1	0	0	[illegible]
HIP_10768	GSHLVEALY	FERRDNMN	GSHLVEALYFERRDNMN		SCG1	11886/367830	0.997	10768	0.849	0.116178	-0.15	0	0	17	-0.8176	-1	2	1	0	[illegible]
HIP_10769	SLYQLENYC	QESKAEGNN	SLYQLENYCQESKAEGNN		CMGA	1946/247050	0.999	10769	0.849	0.110724	-1.6833	0.1667	-2	18	-1.3667	-2	1	0	0	[illegible]
HIP_10770	SHLVEALYL	MNKGDTEVM	SHLVEALYLMNKGDTEVM		CMGA	7775/247050	0.997	10770	0.849	0.181308	0.1333	0	-1	18	0.0611	-2	1	0	0	[illegible]
HIP_10771	ERGFFYTPK	RNQLLQKEP	ERGFFYTPKRNQLLQKEP		SCG2	16856/334890	0.997	10771	0.849	-0.036227	-2.95	0.1667	2	18	-1.4833	2	2	0	0	[illegible]
HIP_10772	GERGFFYTP	NDEMKRSGQ	GERGFFYTPNDEMKRSGQ		SCG2	10105/334890	0.998	10772	0.849	-0.003460	-2.35	0.1667	0	18	-1.5833	0	3	0	0	[illegible]
HIP_10773	SHLVEALYL	SLGYKEIRK	SHLVEALYLSLGYKEIRK		CMGA	345/247050	1.000	10773	0.849	0.209439	1.4833	0.3333	2	18	-0.0778	1	2	0	0	[illegible]
HIP_10774	LVCGERGFF	WNPVEEKIE	LVCGERGFFWNPVEEKIE		SCG2	454/334890	1.000	10774	0.849	0.008543	-0.1333	0.3333	-2	18	-0.2444	-2	3	0	0	[illegible]
HIP_10775	GPGAGSLQP	EEEATNTHP	GPGAGSLQPEEEATNTHP		CMGA	2286/247050	0.999	10775	0.849	-0.048851	-1.9667	0.1667	-2	18	-1.1944	-3	0	0	1	[illegible]
HIP_10776	AGSLQPLAL	SSNNFGAIL	AGSLQPLALSSNNFGAIL		IAPP	154/45018	1.000	10776	0.849	0.237654	0.7167	0.3333	0	18	0.7	0	0	0	0	[illegible]
[illegible]																				

HIP_11232	SHLVEALYL	SEEPVSTHQ	SHLVEALYLSEEPVSTHQ		SCG1	2342/367830	1.000	11232	0.848	0.193312	-0.25	0.1667	-1	18	-0.2667	-3	1	0	0
HIP_11233	DLQVGQVEL	ALAEAYPSK	DLQVGQVELALAEAYPSK		NPY	140/49410	1.000	11233	0.848	0.012385	1.9833	0	-2	18	-0.0167	-2	0	0	0
HIP_11234	CGERGFFY	EVRLLRDPA	CGERGFFYEVRLLRDPA		SCG1	12290/367830	0.997	11234	0.848	0.125118	0.0833	0	0	17	-0.3529	0	3	1	0
HIP_11235	GERGFFYT	EETGSQENH	GERGFFYTEETGSQENH		SCG1	7633/367830	0.999	11235	0.848	0.087939	-1.15	0	-2	17	-1.6353	-3	3	1	0
HIP_11236	AGSLQPLAL	GPNNQKRER	AGSLQPLALGPNNQKRER		SCG2	1600/334890	1.000	11236	0.848	0.232489	0.65	0.3333	2	18	-1.1222	2	0	0	0
HIP_11237	SHLVEALYL	QDTKENREE	SHLVEALYLQDTKENREE		SCG1	4402/367830	0.999	11237	0.848	0.209604	-0.2333	0	-2	18	-1.1944	-3	1	0	0
HIP_11238	CGERGFFY	ALSKSSAPP	CGERGFFYALSKSSAPP		SCG1	5182/367830	0.999	11238	0.848	0.044840	1.5167	0.1667	1	17	-0.2412	1	3	1	0
HIP_11239	CTSICSLYQ	HQQKKHSGF	CTSICSLYQHQQKKHSGF		CMGA	19443/247050	0.974	11239	0.848	0.113283	-1.8667	0	4	18	-0.7444	2	1	0	0
HIP_11240	GERGFFYT	SEEKHLEEP	GERGFFYTSEEKHLEEP		SCG1	6043/367830	0.999	11240	0.848	0.108846	-1.1667	0.1667	-2	17	-1.4647	-3	3	1	0
HIP_11241	VCGERGFF	SDTREEKRF	VCGERGFFSDTREEKRF		SCG1	5394/367830	0.999	11241	0.848	0.118709	0.0333	0.3333	0	17	-1.0941	0	2	1	0
HIP_11242	EDLQVGQVE	EEEEATNTH	EDLQVGQVEEEEEATNTH		CMGA	2528/247050	0.999	11242	0.848	-0.092957	-2.2167	0	-6	18	-1.4444	-7	0	0	3
HIP_11243	LVEALYLVC	RAYFMSDTR	LVEALYLVCRAYFMSDTR		SCG1	13165/367830	0.996	11243	0.848	0.078270	1.0833	0	0	18	0.5833	0	3	0	0
HIP_11244	GSLQPLAL	LDQLLHYRK	GSLQPLALLDQLLHYRK		SCG1	7493/367830	0.999	11244	0.848	0.149766	1.0333	0	2	17	-0.0941	1	0	1	0
HIP_11245	SHLVEALYL	LVDREKGLS	SHLVEALYLLVDREKGLS		CMGA	3952/247050	0.999	11245	0.848	0.046202	1.8	0	0	18	0.2111	-1	1	0	0
HIP_11246	CGERGFFYT	AWIPHVENR	CGERGFFYTAWIPHVENR		SCG2	2176/334890	1.000	11246	0.848	-0.034141	1.0333	0	1	18	-0.5222	0	4	0	0
HIP_11247	AGSLQPLAL	LELDKMANG	AGSLQPLALLELDKMANG		SCG1	4533/367830	0.999	11247	0.848	0.041980	2.25	0	0	18	0.3056	-1	0	0	0
HIP_11248	GERGFFYTP	GEEIKGYPG	GERGFFYTPGEEIKGYPG		SCG1	2000/367830	1.000	11248	0.848	-0.051374	-1.8333	0.3333	-1	18	-0.9611	-1	3	0	0
HIP_11249	SLYQLENYC	EEDKRNYPS	SLYQLENYCEEDKRNYPS		SCG1	8226/367830	0.998	11249	0.848	0.068721	-2.1333	0	-2	18	-1.6611	-2	1	0	0
HIP_11250	TSICSLYQL	YTPQSLATL	TSICSLYQLYTPQSLATL		SCG2	3479/334890	1.000	11250	0.848	0.061249	-0.7667	0.1667	0	18	0.4611	0	2	0	0
HIP_11251	ICSLYQLEN	GKGRHHRGR	ICSLYQLENGKGRHHRGR		SCG1	12825/367830	0.996	11251	0.848	0.143214	-1.3167	0.3333	5	18	-1.2778	3	1	0	0
HIP_11252	RGFFYTPKT	SWDEEDKRN	RGFFYTPKTSWDEEDKRN		SCG1	8808/367830	0.998	11252	0.848	-0.027796	-1.9	0.3333	0	18	-1.95	0	2	0	0
HIP_11253	LYQLENYCN	ENTKFEVRL	LYQLENYCNENTKFEVRL		SCG1	7323/367830	0.999	11253	0.848	0.109712	-1.6667	0	-1	18	-0.85	-1	1	0	0
HIP_11254	SHLVEALYL	IVLSVALNH	SHLVEALYLIVLSVALNH		IAPP	211/45018	0.999	11254	0.848	-0.006648	3.1333	0	1	18	1.3	-1	1	0	0
HIP_11255	ERGFFYTPK	QQKEEEEEM	ERGFFYTPKQQKEEEEEM		CMGA	5430/247050	0.998	11255	0.848	-0.068920	-2.85	0.1667	-3	18	-2.0444	-3	2	0	3
HIP_11256	GSHLVEALY	KTGEKPNGS	GSHLVEALYKTGEKPNGS		SCG2	3882/334890	1.000	11256	0.848	0.215602	-0.1167	0.1667	1	18	-0.7944	0	1	0	0
HIP_11257	VCGERGFFY	PQGAWKELD	VCGERGFFYPQGAWKELD		SCG1	331/367830	1.000	11257	0.848	0.184978	-0.2	0.3333	-1	18	-0.5278	-1	3	0	0
HIP_11258	CSLYQLENY	ENYQKGERG	CSLYQLENYENYQKGERG		SCG1	18123/367830	0.992	11258	0.848	-0.048078	-2.7667	0	-1	18	-1.5722	-1	2	0	0
HIP_11259	GSHLVEALY	NRDSSMKLS	GSHLVEALYNRDSSMKLS		CMGA	8119/247050	0.997	11259	0.848	0.207028	-1.2	0	1	18	-0.4278	0	1	0	0
HIP_11260	SLQPLAL	GFFYTPK	SLQPLALGFFYTPK		INSB	30/4968	0.999	11260	0.848	0.113284	2.4333	0.1667	1	14	0.3571	1	3	0	0
HIP_11261	CTSICSLYQ	VFQELGKLT	CTSICSLYQVFQELGKLT		SCG2	2553/334890	1.000	11261	0.848	-0.086901	0.4167	0	0	18	0.4889	0	2	0	0
HIP_11262	LVCGERGFF	EMLKTGEKP	LVCGERGFFEMLKTGEKP		SCG2	1668/334890	1.000	11262	0.848	0.014527	1.2333	0.1667	0	18	-0.25	0	2	0	0
HIP_11263	VCGERGFFY	QIEQAIKE	VCGERGFFYQIEQAIKE		SCG2	7496/334890	0.999	11263	0.848	0.132939	0.3	0	-1	17	-0.2882	-1	3	1	0
HIP_11264	CGERGFFY	YDSEEPVST	CGERGFFYYDSEEPVST		SCG1	5850/367830	0.999	11264	0.848	0.131530	-0.2167	0.1667	-3	17	-0.7941	-3	4	1	0
HIP_11265	ERGFFYTPK	KPNGSVEPE	ERGFFYTPKKPNGSVEPE		SCG2	9755/334890	0.999	11265	0.848	-0.062995	-2.5333	0.3333	0	18	-1.3833	0	2	0	0
HIP_11266	AGSLQPLAL	NDKDQELGE	AGSLQPLALNDKDQELGE		SCG2	5311/334890	0.999	11266	0.848	0.213118	-0.25	0	-3	18	-0.7111	-3	0	0	0
HIP_11267	VCGERGFFY	RDSSMKLSF	VCGERGFFYRDSSMKLSF		CMGA	7932/247050	0.997	11267	0.848	0.219537	-0.75	0.1667	1	18	-0.2	1	3	0	0
HIP_11268	VCGERGFFY	SEALAVDGA	VCGERGFFYSEALAVDGA		CMGA	925/247050	1.000	11268	0.848	0.114741	0.3	0.1667	-2	18	0.4111	-2	3	0	0
HIP_11269	CCTSICSLY	ENEKDRADQ	CCTSICSLYENEKDRADQ		SCG1	4369/367830	0.999	11269	0.848	0.023075	-1.4667	0.1667	-2	18	-0.8556	-2	1	0	0
HIP_11270	VCGERGFFY	EEEPEYGEE	VCGERGFFYEEEPEYGEE		SCG1	864/367830	1.000	11270	0.848	0.108471	-1.0333	0	-6	18	-1.2278	-6	3	0	1
HIP_11271	VCGERGFFY	PASLPSQKY	VCGERGFFYPASLPSQKY		CMGA	888/247050	1.000	11271	0.848	0.154623	0.6167	0.3333	1	18	-0.3167	1	3	0	0
HIP_11272	GPGAGSLQP	PNPYNQEKV	GPGAGSLQPPNPYNQEKV		SCG2	51/334890	1.000	11272	0.848	0.009647	-1.3333	0.5	0	18	-1.1833	0	1	0	0
HIP_11273	AGSLQPLAL	SDDYETQQW	AGSLQPLALSDDYETQQW		SCG2	578/334890	1.000	11273	0.848	0.176375	0.2667	0.1667	-3	18	-0.6944	-3	1	0	0
HIP_11274	LVCGERGFF	PRPQSEESW	LVCGERGFFPRPQSEESW		SCG1	4859/367830	0.999	11274	0.848	0.112197	-0.4167	0.5	-1	18	-0.7444	-1	2	0	0

HIP_11743	CGERGFFYT	LARVPKLDL	CGERGFFYTLARVPKLDL		SCG1	9968/367830	0.998	11743	0.847	-0.043565	0.3167	0	1	18	0.0667	1	3	0
HIP_11744	VCGERGFFY	NLITRQRY	VCGERGFFYNLITRQRY		NPY	1410/49410	0.992	11744	0.847	0.050280	1.5167	0	2	17	-0.4412	2	3	1
HIP_11745	SHLVEALYL	TLALSLLVC	SHLVEALYLTLALSLLVC		NPY	375/49410	0.999	11745	0.847	0.033011	1.8667	0	0	18	1.7111	-1	1	0
HIP_11746	VCGERGFF	QTQEEVRDS	VCGERGFFQTQEEVRDS		SCG2	5981/334890	0.999	11746	0.847	0.084629	-0.4167	0.1667	-2	17	-0.9294	-2	2	1
HIP_11747	CGERGFFYT	MNDNFLEGE	CGERGFFYTMNDNFLEGE		SCG1	5136/367830	0.999	11747	0.847	0.023998	-0.7167	0	-3	18	-0.6722	-3	3	0
HIP_11748	CGERGFFY	VMEKREDSK	CGERGFFYVMEKREDSK		CMGA	8063/247050	0.997	11748	0.847	0.060733	1.15	0	0	17	-1.1471	0	3	1
HIP_11749	VEALYLVC	MDKARRHPQ	VEALYLVCMDKARRHPQ		SCG1	20109/367830	0.989	11749	0.847	0.227385	0.8333	0	2	17	-0.3235	1	1	1
HIP_11750	HLVEALYLV	EPEYGEEIK	HLVEALYLVEPEYGEEIK		SCG1	202/367830	1.000	11750	0.847	0.146765	-0.3167	0.1667	-3	18	-0.1722	-4	2	0
HIP_11751	CGERGFFYT	EKSGEATDG	CGERGFFYTEKSGEATDG		CMGA	7358/247050	0.997	11751	0.847	0.144937	-1.2333	0.1667	-2	18	-0.9778	-2	3	0
HIP_11752	GERGFFYT	QNGQNGERA	GERGFFYTQNGQNGERA		SCG2	5570/334890	0.999	11752	0.847	0.148709	-1.1	0.1667	0	17	-1.5412	0	3	1
HIP_11753	VEALYLVC	QWSLYPSDS	VEALYLVCQWSLYPSDS		SCG1	6246/367830	0.999	11753	0.847	0.155270	0.8833	0.1667	-2	17	0.3588	-2	3	1
HIP_11754	GSLQPLAL	VKDKETTEN	GSLQPLALVKDKETTEN		SCG1	11500/367830	0.997	11754	0.847	0.187626	1.0333	0	-1	17	-0.7118	-1	0	1
HIP_11755	GERGFFYTP	NEGMVTRCI	GERGFFYTPNEGMVTRCI		SCG1	1921/367830	1.000	11755	0.847	-0.022154	-1.8333	0.3333	0	18	-0.35	0	3	0
HIP_11756	LVCGERGFF	EEPGETQNA	LVCGERGFFEEPGETQNA		SCG1	206/367830	1.000	11756	0.847	0.058136	-0.5667	0.3333	-3	18	-0.6167	-3	2	0
HIP_11757	VCGERGFFY	VEVLKREPL	VCGERGFFYVEVLKREPL		IAPP	498/45018	0.998	11757	0.847	-0.014679	1.5333	0	0	18	0.0667	0	3	0
HIP_11758	AGSLQPLAL	YDSEEPVST	AGSLQPLALYDSEEPVST		SCG1	1437/367830	1.000	11758	0.847	0.160515	0.6333	0.1667	-3	18	-0.1556	-3	1	0
HIP_11759	AGSLQPLAL	NEPQSAPKE	AGSLQPLALNEPQSAPKE		SCG2	324/334890	1.000	11759	0.847	0.203732	0.1333	0.1667	-1	18	-0.6333	-1	0	0
HIP_11760	VCGERGFFY	ILSILRHQN	VCGERGFFYILSILRHQN		CMGA	1657/247050	1.000	11760	0.847	-0.013248	1.9667	0.1667	2	18	0.1833	1	3	0
HIP_11761	SHLVEALYL	WNPVEEKI	SHLVEALYLWNPVEEKI		SCG2	2948/334890	1.000	11761	0.847	0.147598	0.05	0.1667	-1	17	0.0235	-2	2	1
HIP_11762	CGERGFFYT	SWDEEDKR	CGERGFFYTSWDEEDKR		SCG1	8702/367830	0.998	11762	0.847	0.069649	-0.7333	0.1667	-2	17	-1.5765	-2	4	1
HIP_11763	CGERGFFY	LDKMAHGYG	CGERGFFYLDKMAHGYG		SCG1	15697/367830	0.994	11763	0.847	0.161769	0.1167	0	1	17	-0.4235	0	3	1
HIP_11764	QHLCGSHLV	ENQMDKARR	QHLCGSHLVENQMDKARR		SCG1	10017/367830	0.998	11764	0.847	0.204662	-0.95	0	3	18	-1.1111	1	0	0
HIP_11765	LYQLENYCN	EPQWSLYPS	LYQLENYCNEPQWSLYPS		SCG1	1648/367830	1.000	11765	0.847	0.098169	-1.8167	0.1667	-2	18	-0.9278	-2	2	0
HIP_11766	SHLVEALYL	ESHLPERDS	SHLVEALYLESHLPERDS		SCG2	4033/334890	1.000	11766	0.847	0.162692	-0.2	0.1667	-1	18	-0.5	-3	1	0
HIP_11767	GSHLVEALY	RALEYIENL	GSHLVEALYRALEYIENL		SCG2	5523/334890	0.999	11767	0.847	0.004576	0.9	0	-1	18	0.1111	-2	1	0
HIP_11768	HLVEALYLV	DDIYKANNI	HLVEALYLVDDIYKANNI		SCG2	3214/334890	1.000	11768	0.847	0.113068	0.7	0	-1	18	0.2889	-2	2	0
HIP_11769	CGERGFFYT	DWNPVEEKI	CGERGFFYTDWNPVEEKI		SCG2	8544/334890	0.999	11769	0.847	0.057789	-1.1833	0	-2	18	-0.8	-2	4	0
HIP_11770	RGFFYTPKT	EEQKLYTDD	RGFFYTPKTEEQKLYTDD		SCG2	11875/334890	0.998	11770	0.847	-0.032521	-2.7833	0.1667	-1	18	-1.5056	-1	1	0
HIP_11771	GSHLVEALY	KPFSEDVNW	GSHLVEALYKPFSEDVNW		SCG1	2264/367830	1.000	11771	0.847	0.121633	0.2667	0.1667	-1	18	-0.35	-2	2	0
HIP_11772	CTSICSLYQ	SGYPKTPGR	CTSICSLYQSGYPKTPGR		SCG2	3239/334890	1.000	11772	0.847	0.103951	-0.5833	0.3333	2	18	-0.5	2	2	0
HIP_11773	TSICSLYQL	FLVHSSNN	TSICSLYQLFLVHSSNN		IAPP	284/45018	0.999	11773	0.847	-0.019871	1.6333	0	1	17	0.3824	0	2	1
HIP_11774	SHLVEALYL	YTPQSLAT	SHLVEALYLYTPQSLAT		SCG2	5846/334890	0.999	11774	0.847	0.184398	0.45	0.1667	0	17	0.3294	-1	2	1
HIP_11775	VCGERGFFY	RGGEPRAY	VCGERGFFYRGGEPRAY		SCG1	6804/367830	0.999	11775	0.847	0.217008	-0.1667	0.3333	1	17	-0.7176	1	3	1
HIP_11776	CGERGFFYT	EEEQIEQAI	CGERGFFYTEEEQIEQAI		SCG2	5051/334890	1.000	11776	0.847	0.076634	-1.6167	0	-4	18	-0.7167	-4	3	0
HIP_11777	LVCGERGFF	PEYNYDWWE	LVCGERGFFPEYNYDWWE		SCG1	1891/367830	1.000	11777	0.847	0.017777	-0.2	0.3333	-3	18	-0.7056	-3	3	0
HIP_11778	LVEALYLVC	RGYPEEKKE	LVEALYLVCRGYPEEKKE		CMGA	5707/247050	0.998	11778	0.847	0.163117	0.7167	0.1667	-1	18	-0.3778	-1	2	0
HIP_11779	SLYQLENYC	HYRKKSAEF	SLYQLENYCHYRKKSAEF		SCG1	28233/367830	0.971	11779	0.847	0.043849	-1.8833	0	2	18	-1.1278	1	2	0
HIP_11780	CGERGFFYT	EGENYQKG	CGERGFFYTEGENYQKG		SCG1	5594/367830	0.999	11780	0.847	0.148843	-1.1	0.1667	-1	17	-1.3353	-1	3	1
HIP_11781	LVCGERGFF	EGENYQKGE	LVCGERGFFEGENYQKGE		SCG1	156/367830	1.000	11781	0.847	0.089787	-0.3667	0.3333	-2	18	-0.9	-2	2	0
HIP_11782	VCGERGFFY	ESEEGEEDA	VCGERGFFYESEEGEEDA		SCG1	119/367830	1.000	11782	0.847	0.136896	-0.5833	0.1667	-6	18	-1.0111	-6	3	0
HIP_11783	LVEALYLVC	GENYQKGE	LVEALYLVCGENYQKGE		SCG1	662/367830	1.000	11783	0.847	0.186476	0.5167	0.1667	-2	17	-0.0412	-2	2	1
HIP_11784	VCGERGFFY	SKEAEKSGE	VCGERGFFYSKEAEKSGE		CMGA	6247/247050	0.998	11784	0.847	0.196861	-0.65	0.1667	-1	18	-0.9056	-1	3	0
HIP_11785	HLVEALYLV	RAYFMSDTR	HLVEALYLVRAYFMSDTR		SCG1	14735/367830	0.995	11785	0.847	0.080239	0.45	0	1	18	0.2667	0	3	0
HIP_11786	LVEALYLVC	WEKKPFSE	LVEALYLVCWEKKPFSE		SCG1	12339/367830	0.997	11786	0.847	0.147149	0.3667	0	-1	17	0.2353	-1	2	1
HIP_11787	VCGERGFFY	EEEEEGENY	VCGERGFFYEEEEEGENY		SCG1	862/367830	1.000	11787	0.847	0.108933	-1.0333	0	-6	18	-1.3333	-6	3	0
HIP_11788	LVCGERGFF	QQWPERKL	LVCGERGFFQQWPERKL		SCG2	11128/334890	0.999	11788	0.847	0.066019	-0.45	0.1667	1	17	-0.6059	1	3	1
HIP_11789	LVCGERGFF	GDERILSIL	LVCGERGFFGDERILSIL		CMGA	646/247050	1.000	11789	0.847	0.102094	-0.3667	0.3333	-1	18	0.6222	-1	2	0
HIP_11790	AGSLQPLAL	PEYGEEIKG	AGSLQPLALPEYGEEIKG		SCG1	1527/367830	1.000	11790	0.847	0.160458	0.5	0.1667	-2	18	-0.2722	-2	1	0

HIP_12254	GSHLVEALY	GRHHRGRGG	GSHLVEALYGRHHRGRGG		SCG1	16772/367830	0.993	12254	0.847	0.195552	-0.6333	0.1667	5	18	-0.95	2	1	0	0	0.000000	-4.1333	1	0.1333	2.000000	0.2222	1.7778	0	1	0	1	-4.1333	-1.1259	-4.1333	0	1	0	0	0.3987	1.0524	0.8968	0	0.159	0.8042	-0.2525	0.0178	-0.5511
HIP_12255	SHLVEALYL	EGGSANRRPE	SHLVEALYLEGGSANRRPE		CMGA	271/247050	1.000	12255	0.847	0.227756	0.2667	0.3333	0	18	-0.6611	-1	1	0	0	0.000000	-3.6667	-1	0.2	0.450000	0.2222	0.2278	0	1	0	1	3.6667	0.0607	-3.6667	1	0	0	1	0.4551	1.0719	0.8912	0.1667	0.2071	0.7942	0.1214	0.04	-0.7333
HIP_12256	GERGFFYTP	RQYDRVAQL	GERGFFYTPRQYDRVAQL		SCG1	17208/367830	0.993	12256	0.847	0.020457	-2.15	0.1667	1	18	-0.9889	1	4	0	0	0.000000	-1.9	1	-0.0167	0.700000	0.2222	0.4778	1	0	1	0	-1.9	-1.0272	-7.6	0	0	0	1	0.5321	0.9291	1.0301	-0.1667	0.2831	1.0612	-1.1439	0.0003	0.0317
HIP_12257	RGFFYTPKT	EEEEEMAVV	RGFFYTPKTEEEEEMAVV		CMGA	4532/247050	0.999	12257	0.847	-0.044077	-2.7833	0.1667	-3	18	-0.7167	-3	1	0	3	0.000000	-1.4333	-4	-0.0167	0.000000	0.5556	-0.5556	1	0	1	0	5.7333	1.5463	-1.4333	0	1	0	0	0.5885	1.1586	0.9345	-0.6667	0.3463	0.8733	-1.6379	0.0003	0.0239
HIP_12258	GSHLVEALY	EHLNQGSSQ	GSHLVEALYEHLNQGSSQ		SCG2	8152/334890	0.999	12258	0.847	0.016685	0.2333	0	0	18	-0.6111	-2	1	0	0	0.000000	-2.4	-1	0.05	2.000000	0.2222	1.7778	0	0	0	0	2.4	0.4148	-2.4	0	1	0	0	0.3436	1.2672	0.7023	0.1667	0.1181	0.4932	0.0802	0.0025	-0.12
HIP_12259	SICSLYQLE	EEGGSANRRP	SICSLYQLEEEGGSANRRP		CMGA	5431/247050	0.998	12259	0.847	-0.014890	-1.7667	0.1667	-1	18	-0.8778	-1	1	0	1	0.000000	-1.4	-1	0.0833	1.900000	0.3333	1.5667	0	1	0	1	1.4	-2.7678	-1.4	0	0	1	0	0.55	1.24	0.8667	-0.3333	0.3025	0.7511	-0.9717	0.0069	-0.1167
HIP_12260	SHLVEALYL	LIEMLKTGE	SHLVEALYLLIEMLKTGE		SCG2	2023/334890	1.000	12260	0.847	0.013441	1.85	0	-1	18	0.5889	-2	1	0	0	1.000000	-0.5	-1	0.05	1.425000	0.2222	1.2028	0	0	0	0	0.5	2.2251	-0.5	1	0	0	0	0.3051	1.2519	0.6028	0.5	0.0931	0.3634	0.5645	0.0025	-0.025
HIP_12261	LVCGERGFF	MDEEQKLYT	LVCGERGFFMDEEQKLYT		SCG2	3255/334890	1.000	12261	0.847	0.010647	0.0167	0.1667	-2	18	-0.3833	-2	2	0	0	1.000000	-3.4333	-2	0.0167	1.175000	0.5556	0.6194	0	1	0	1	6.8667	0.0103	-6.8667	1	0	0	0	0.3885	1.1053	0.8245	0.1667	0.1509	0.6798	0.0065	0.0003	-0.0572
HIP_12262	VCGERGFFY	RLQNGQNG	VCGERGFFYRLQNGQNG		SCG2	16191/334890	0.997	12262	0.847	0.159062	0.0167	0	1	17	-0.7824	1	3	1	0	0.000000	-2.8333	1	0.1	0.700000	0.2222	0.4778	0	0	0	0	-2.8333	0.008	-8.5	0	1	0	0	0.3436	1.1422	0.749	0.3333	0.1181	0.561	0.0057	0.01	-0.2833
HIP_12263	HLVEALYL	KELDRNYLN	HLVEALYLKELDRNYLN		SCG1	14448/367830	0.995	12263	0.847	0.187107	0.45	0	0	17	-0.3941	-1	1	1	0	0.000000	-3.3	0	0.1	1.400000	0.4444	0.9556	0	0	0	0	0	0.43	-3.3	1	0	0	0	0.3436	1.2422	0.6756	0.1667	0.1181	0.4565	0.1546	0.01	-0.33
HIP_12264	TSICSLYQL	RQYWDEDLL	TSICSLYQLRQYWDEDLL		SCG2	14423/334890	0.998	12264	0.847	0.075348	-1.7167	0	-2	18	-0.3111	-2	3	0	0	0.000000	-2.7667	1	0.05	0.000000	0.1111	-0.1111	0	0	0	0	-2.7667	0.1907	-8.3	1	0	0	0	0.4487	1.0974	0.8635	0	0.2013	0.7456	-0.7703	0.0025	-0.1383
HIP_12265	CCTSICSLY	YSSHHTAEK	CCTSICSLYYSSHHTAEK		SCG1	4733/367830	0.999	12265	0.847	0.015678	-0.2	0.5	2	18	-0.1889	0	2	0	0	0.000000	-1.5333	0	0.0667	2.075000	0	2.075	0	0	0	0	0	-0.415	-3.0667	0	1	0	0	0.4769	0.9572	0.9856	0.1667	0.2275	0.9715	-0.0954	0.0044	-0.1022
HIP_12266	HLVEALYL	SWDEEDKRN	HLVEALYLSWDEEDKRN		SCG1	3673/367830	0.999	12266	0.847	0.137299	0.1833	0.1667	-2	17	-1.0706	-3	2	1	0	0.000000	-3.8333	-1	0.1167	1.400000	0.3333	1.0667	0	0	0	0	3.8333	0.1956	-7.6667	1	0	0	0	0.3821	1.0774	0.8201	0.3333	0.146	0.6726	0.07	0.0136	-0.4472
HIP_12267	GSHLVEALY	AYFMSDTRE	GSHLVEALYAYFMSDTRE		SCG1	1419/367830	1.000	12267	0.847	-0.048209	1.2667	0	-1	18	-0.1889	-2	3	0	0	0.000000	-0.3333	0	0	2.925000	0.1111	2.8139	0	0	0	0	0	3.5643	-1	0	1	0	0	0.2769	1.2405	0.7056	0.6667	0.0767	0.4979	0.3508	0	0
HIP_12268	AGSLQPLAL	QCCTSICSL	AGSLQPLALQCCTSICSL		INSA	62/3024	0.990	12268	0.847	0.080590	1.8167	0	0	18	1.0389	0	0	0	0	0.000000	-2.6333	0	0.05	0.450000	0	0.45	0	0	0	0	0	0.8175	0	1	0	0	0	0.3436	1.2005	0.7423	0.5	0.1181	0.551	0.6242	0.0025	-0.1317
HIP_12269	SHLVEALYL	FSEDVNWG	SHLVEALYLFSEDVNWG		SCG1	1620/367830	1.000	12269	0.847	0.152479	0.8	0.1667	-2	17	0.1765	-3	2	1	0	1.000000	-2.6	-1	0.1167	0.700000	0.3333	0.3667	0	0	0	0	2.6	0.2933	-5.2	1	0	0	0	0.3718	1.1886	0.7412	0.3333	0.1382	0.5493	0.2974	0.0136	-0.3033
HIP_12270	RGFFYTPKT	HQQKKHSGF	RGFFYTPKTHQQKKHSGF		CMGA	15633/247050	0.985	12270	0.847	0.064900	-2.7333	0.1667	6	18	-1.5056	4	1	0	0	0.000000	-1.3333	-1	-0.0667	0.000000	0.2222	-0.2222	1	0	1	0	1.3333	0.6074	-1.3333	0	1	0	0	0.5603	0.9438	1.0423	-0.3333	0.3139	1.0864	-1.5314	0.0044	0.0889
HIP_12271	TSICSLYQL	QDLALQGAK	TSICSLYQLQDLALQGAK		CMGA	10229/247050	0.995	12271	0.847	0.058452	-0.7	0	0	18	0.2167	0	1	0	0	0.000000	-0.7333	-1	0.05	0.450000	0.1111	0.3389	0	0	0	0	0.7333	-0.2372	-0.7333	1	0	0	0	0.4103	1.1438	0.7956	0.1667	0.1683	0.633	-0.2872	0.0025	-0.0367
HIP_12272	SLYQLENYC	NPYYDPLQW	SLYQLENYCNPYYDPLQW		SCG1	2485/367830	1.000	12272	0.847	-0.010763	-1.45	0.1667	-2	18	-0.9556	-2	3	0	0	0.000000	-1.3667	0	0.0667	0.950000	0.1111	0.8389	1	0	1	0	0	-1.2164	-4.1	0	0	0	0	0.5321	0.8291	1.2268	0	0.2831	1.505	-0.7715	0.0044	-0.0911
HIP_12273	CGERGFFYT	ESHLPERDS	CGERGFFYTESHLPERDS		SCG2	9874/334890	0.999	12273	0.847	0.054222	-1.1167	0.1667	-1	18	-1.1278	-2	3	0	0	0.000000	-2.7667	-1	0.0667	0.950000	0.1111	0.8389	0	0	0	0	2.7667	-0.9368	-8.3	0	1	0	0	0.4769	1.0638	0.8856	-0.1667	0.2275	0.7844	-0.5326	0.0044	-0.1844
HIP_12274	VCGERGFFY	PADASEAHE	VCGERGFFYPADASEAHE		SCG1	588/367830	1.000	12274	0.847	0.110361	0.1667	0.1667	-2	18	-0.4722	-3	3	0	0	0.000000	-2.5333	-1	0.1167	1.150000	0.2222	0.9278	1	0	1	0	2.5333	0.1546	-7.6	0	1	0	0	0.3603	1.0772	0.8656	0.3333	0.1298	0.7493	0.06	0.0136	-0.2956
HIP_12275	LYQLENYCN	RNYLNYGEE	LYQLENYCNRNYLNYGEE		SCG1	14253/367830	0.995	12275	0.847	-0.010793	-1.9333	0	-2	18	-1.3444	-2	2	0	0	0.000000	-2.3333	1	0.05	1.575000	0.2222	1.3528	0	0	0	0	-2.3333	-2.6154	-4.6667	0	1	0	0	0.4872	0.9044	1.1246	-0.1667	0.2373	1.2648	-0.9419	0.0025	-0.1167
HIP_12276	LVEALYLVC	EEQKLYTDD	LVEALYLVCEEQKLYTDD		SCG2	967/334890	1.000	12276	0.847	0.128036	0	0	-4	18	-0.0167	-4	1	0	0	0.000000	-7	-2	0.15	2.000000	0.3333	1.6667	0	0	0	0	14	0	-7	0	0	0	0	0.4385	1.2519	0.7278	0	0.1922	0.5297	0	0.0225	-1.05
HIP_12277	LVCGERGFF	ESNVSMAS	LVCGERGFFESNVSMAS		SCG1	2857/367830	1.000	12277	0.847	0.079756	-0.4333	0.3333	-1	17	0.3412	-1	2	1	0	0.000000	-4.3333	-1	0.0833	1.750000	0.3333	1.4167	0	1	0	1	4.3333	-0.6139	-8.6667	1	0	0	0	0.4833	0.98	1.0333	0.1667	0.2336	1.0678	-0.2094	0.0069	-0.3611
HIP_12278	VCGERGFFY	LGEYLARML	VCGERGFFYLGEYLARML		SCG2	370/334890	1.000	12278	0.847	0.137473	0.7	0.1667	0	18	0.4222	0	4	0	0	0.000000	-1.4667	-1	0.1333	1.650000	0.2222	1.4278	0	1	0	1	1.4667	0.9994	-5.8667	0	1	0	0	0.3885	1.1219	0.8078	0.3333	0.1509	0.6526	0.2719	0.0178	-0.1956
HIP_12279	GSHLVEALY	VIAYLKRLV	GSHLVEALYVIAYLKRLV		SCG2	3909/334890	1.000	12279	0.847	-0.020940	2.4667	0	2	18	0.9444	1	2	0	0	0.000000	2.0667	0	0	3.050000	0.1111	2.9389	0	0	0	0	0	7.2493	4.1333	0	1	0	0	0.2385	1.2269	0.6162	0.8333	0.0569	0.3796	0.5882	0	0
HIP_12280	LVEALYLV	EEKRFLGEG	LVEALYLVEEKRFLGEG		SCG1	7821/367830	0.998	12280	0.847	0.154791	-0.7	0	-2	17	0.2176	-2	2	1	0	0.000000	-5.8667	-1	0.15	2.700000	0.4444	2.2556	0	0	0	0	5.8667	-1.5789	-11.7333	1	0	0	0	0.4103	1.2372	0.7156	-0.1667	0.1683	0.5121	-0.2872	0.0225	-0.88
HIP_12281	SHLVEALYL	NQGSSQETD	SHLVEALYLNQGSSQETD		SCG2	148/334890	1.000	12281	0.847	0.223287	-0.1833	0.1667	-2	18	-0.6444	-3	1	0	0	0.000000	-4.5667	0	0.1833	0.000000	0.1111	-0.1111	0	1	0	1	0	0.0204	-4.5667	1	0	0	0	0.4551	0.9836	0.9912	0.3333	0.2071	0.9824	-0.0834	0.0336	-0.8372
HIP_12282	CCTSICSLY	LSFRARAYG	CCTSICSLYLSFRARAYG		CMGA	2147/247050	0.999	12282	0.847	-0.053340	1.25	0.3333	2	18	0.6056	2	2	0	0	0.000000	1.3667	0	0	3.025000	0.1111	2.9139	0	0	0	0	0	3.6424	2.7333	0	1	0	0	0.3718	1.0703	0.8178	0.5	0.1382	0.6688	0.4647	0	0
HIP_12283	AGSLQPLAL	EPQWSLYPS	AGSLQPLALEPQWSLYPS		SCG1	3441/367830	1.000	12283	0.847	0.210810	0.1333	0.1667	-1	18	-0.0833	-1	1	0	0	0.000000	-6	-1	0.1667	0.450000	0.1111	0.3389	1	0	1	0	6	0.0452	-6	1	0	0	0	0.4167	1.2067	0.8167	0.3333	0.1736	0.6669	0.0556	0.0278	-1
HIP_12284	CGERGFFYT	IVLSVALNH	CGERGFFYTIVLSVALNH		IAPP	178/45018	0.999	12284	0.847	-0.036600	2.2167	0	1	18	0.6722	0	3	0	0	0.000000	3.9	0	-0.05	1.125000	0	1.125	0	0	0	0	0	2.4938	11.7	0	1	0	0	0.3051	1.0819	0.6562	0.6667	0.0931	0.4305	0.6764	0.0025	-0.195
HIP_12285	CTSICSLYQ	SEKNFPMDM	CTSICSLYQSEKNFPMDM		SCG2	10950/334890	0.999	12285	0.847	0.189124	-1.5333	0.1667	-1	18	-0.2222	-1	1	0	0	0.000000	-2.4	0	0.1167	0.625000	0.2222	0.4028	0	0	0	0	0	-0.6176	-2.4	0	1	0	0	0.4769	1.1272	0.8723	-0.1667	0.2275	0.7609	-0.7313	0.0136	-0.28
HIP_12286	GPGAGSLQP	NGERATRLF	GPGAGSLQPNGERATRLF		SCG2	2668/334890	1.000	12286	0.847	0.040811	-1.45	0.3333	1	18	-0.6556	1	0	0	0	0.000000	-2.0333	-1	0.0667	0.000000	0.2222	-0.2222	1	1	1	1	2.0333	0.3222	0	0	0	0	1	0.5667	0.9433	1.1333	0	0.3211	1.2844	-0.8217	0.0044	-0.1356
HIP_12287	SHLVEALYL	SREKSSQES	SHLVEALYLSREKSSQES		SCG1	11273/367830	0.997	12287	0.847	0.255444	-0.4167	0.1667	0	18	-0.75	-1	1	0	0	0.000000	-5.0333	0	0.1667	0.000000	0.4444	-0.4444	0	0	0	0	0	0.1852	-5.0333	1	0	0	0	0.4103	1.1588	0.7923	0	0.1683	0.6278	-0.1709	0.0278	-0.8389
HIP_12288	GSHLVEALY	SEEKGHPQE	GSHLVEALYSEEKGHPQE		SCG1	1548/367830	1.000	12288	0.847	0.159745	-0.5833	0.1667	-1	18	-1.0833	-3	1	0	0	0.000000	-4.0333	-2	0.1667	2.000000	0.4444	1.5556	0	0	0	0	8.0667	-0.9074	-4.0333	0	1	0	0	0.4385	1.2653	0.7912	0	0.1922	0.6259	-0.2558	0.0278	-0.6722
HIP_12289	CGERGFFY	EDNRDSSMK	CGERGFFYEDNRDSSMK		CMGA	5597/247050	0.998	12289	0.847	0.175779	-1.0333	0	-1	17	-1.4176	-1	3	1	0	0.000000	-4.9333	-2	0.15	0.000000	0.4444	-0.4444	0	0	0	0	9.8667	0.4593	-14.8	0	1	0	0	0.4103	1.1038	0.8856	0	0.1683	0.7844	-0.4239	0.0225	-0.74
HIP_12290	LVCGERGFF	AHQLQALRR	LVCGERGFFAHQLQALRR		CMGA	8338/247050	0.996	12290	0.847	0.033501	0.05	0.1667	3	18	-0.0389	2	2	0	0	1.000000	-3.3667	0	-0.0333	2.100000	0.2222	1.8778	0	1	0	1	0	0.0939	-6.7333	1	0	0	0	0.3885	1.0803	0.8745	0.3333	0.1509	0.7647	0.0194	0.0011	0.1122
HIP_12291	CGERGFFYT	EEGENYQK	CGERGFFYTEEGENYQK		SCG1	4581/367830	0.999	12291	0.847	0.140219	-1.1	0.1667	-2	17	-1.5176	-2	3	1	0	0.000000	-2.7333	-2	0.1333	0.000000	0.3333	-0.3333	0	1	0	1	5.4667	0.3667	-8.2	0	1	0	0	0.5218	1.0953	0.9078	-0.1667	0.2723	0.8241	-0.574	0.0178	-0.3644
HIP_12292	CGERGFFYT	NLQIPPEDL	CGERGFFYTNLQIPPEDL		SCG2	3334/334890	1.000	12292	0.847	-0.006509	-0.4	0	-2	18	-0.4333	-2	3	0	0	0.000000	-1.3333	0	0.05	1.125000	0	1.125	0	0	0	0	0	-0.45	-4	0	1	0	0	0.4385	1.0219	0.9178	0.3333	0.1922	0.8424	-0.1754	0.0025	-0.0667
HIP_12293	ERGFFYTPK	KRSSPETLI	ERGFFYTPKKRSSPETLI		NPY	3188/49410	0.973	12293	0.847	-0.005125	-2.5667	0.3333	2	18	-0.9889	2	2	0	0	0.000000	-1	1	0	0.700000	0.3333	0.3667	1	0	1	0	-1	-0.9411	-2	0	0	1	0	0.5321	0.8774	1.0668	-0.5	0.2831	1.1381	-1.3656	0	0
HIP_12294	GERGFFYT	EEGENYQKG	GERGFFYTEEGENYQKG		SCG1	3916/367830	0.999	12294	0.847	0.126404	-1.1	0.1667	-2	17	-1.6882	-2	3	1	0	0.000000	-2.7333	-2	0.1333	0.700000	0.3333	0.3667	0	1	0	1	5.4667	-0.4033	-8.2	0	1	0	0	0.5218	1.0953	0.9078	-0.1667	0.2723	0.8241	-0.574	0.0178	-0.3644
HIP_12295	AGSLQPLAL	EEQIEQAIK	AGSLQPLALEEQIEQAIK		SCG2	1011/334890	1.000	12295	0.847	0.133987	-0.1833	0	-2	18	-0.1056	-2	0	0	0	0.000000	-6.6333	-2	0.15	1.575000	0.2222	1.3528	0	0	0	0	13.2667	-0.248	0	1	0	0	0	0.4	1.3633	0.6867	0.1667	0.16	0.4715	-0.0733	0.0225	-0.995
HIP_12296	SHLVEALYL	DGARPQALP	SHLVEALYLDGARPQALP		CMGA	731/247050	1.000	12296	0.847	0.211907	0.7	0.1667	0	18	0.05	-1	1	0	0	0.000000	-2.8	-1	0.1333	0.000000	0.3333	-0.3333	0	1	0	1	2.8	-0.2333	-2.8	1	0	0	0	0.3603	1.0988	0.829	0.3333	0.1298	0.6872	0.2522	0.0178	-0.3733
HIP_12297	HLCGSHLVE	YETQQWPER	HLCGSHLVEYETQQWPER		SCG2	16320/334890	0.997	12297	0.847	0.072420	-0.1667	0	0	18	-1.1	-2	1	0	0	0.000000	-3.3333	0	0.05	1.050000	0.2222	0.8278	0	0	0	0	0	-0.138	-3.3333	0	0	0	1	0.4385	1.2053	0.7245	0	0.1922	0.5249	-0.0731	0.0025	-0.1667
HIP_12298	CGERGFFYT	IENLRQQAH	CGERGFFYTIENLRQQAH		SCG2	9486/334890	0.999	12298	0.847	-0.000767	-0.2833	0	1	18	-0.7944	0	3	0	0	0.000000	-1.1	-1	0.05	2.075000	0.1111	1.9639	0	0	0	0	1.1	-0.5564	-3.3	0	1	0	0	0.4385	1.0519	0.8728	0.1667	0.1922	0.7618	-0.1242	0.0025	-0.055
HIP_12299	GSHLVEALY	EPEYGEEIK	GSHLVEALYEPEYGEEIK		SCG1	57/367830	1.000	12299	0.847	0.135296	-0.7167	0.1667	-3	18	-0.6833	-4	2	0	0	0.000000	-4.3	-2	0.1667	2.000000	0.3333	1.6667	1	0	1	0	8.6	-1.1944	-8.6	0	1	0	0	0.4551	1.2269	0.8445	0	0.2071	0.7132	-0.3262	0.0278	-0.7167
HIP_12300	LVEALYLVC	KWAEGGGHS	LVEALYLVCKWAEGGGHS		SCG1	12775/367830	0.996	12300	0.847	0.086414	1.25	0	0	18	0.4222	-1	2	0	1	0.000000	-4.5	1	0.05	2.000000	0.2222	1.7778	0	0	0	0	-4.5	2.2222	-9	0	1	0	0	0.3154	1.1458	0.7301	0.5	0.0995	0.5331	0.3942	0.0025	-0.225
HIP_12301	GERGFFYTP	QDKQYSSHH	GERGFFYTPQDKQYSSHH		SCG1	18400/367830	0.992	12301	0.847	0.036770	-2.4167	0.1667	2	18	-1.6944	0	3	0	0	0.000000	-2.4333	0	0.0333	0.700000	0.2222	0.4778	1	0	1	0	0	-1.1546	-7.3	0	0	0	1	0.5321	0.9291	1.0301	-0.3333	0.2831	1.0612	-1.2858	0.0011	-0.0811
HIP_12302	AGSLQPLAL	SEEVKTRHS	AGSLQPLALSEEVKTRHS		SCG1	2959/367830	1.000	12302	0.847	0.175523	0.2667	0.1667	1	18	-0.4444	0	0	0	0	0.000000	-5.7333	-2	0.1667	1.500000	0.2222	1.2778	0	0	0	0	11.4667	0.3407	0	1	0	0	0	0.4	1.3117	0.7233	0.1667	0.16	0.5232	0.1067	0.0278	-0.9556
HIP_12303	GERGFFYT	HPQESKGQP	GERGFFYTHPQESKGQP		SCG1	14421/367830	0.995	12303	0.847	0.081883	-1.25	0.1667	1	17	-1.6	0	3	1	0	0.000000	-3.0333	0	0.0667	0.700000	0.1111	0.5889	1	0	1	0	0	-0.7361	-9.1	0	1	0	0	0.4936	0.9588	0.979	0	0.2436	0.9584	-0.617	0.0044	-0.2022
HIP_12304	CGERGFFYT	DSKEAEKSG	CGERGFFYTDSKEAEKSG		CMGA	8958/247050	0.996	12304	0.847	0.158918	-1.2333	0.1667	-1	18	-1.1778	-1	3	0	0	0.000000	-3	0	0.1167	0.000000	0.3333	-0.3333	0	0	0	0	0	0.4111	-9	0	1	0	0	0.4487	0.9824	0.9135	-0.1667	0.2013	0.8344	-0.5534	0.0136	-0.35
HIP_12305	VCGERGFFY	DMSDDYETQ	VCGERGFFYDMSDDYETQ		SCG2	368/334890	1.000	12305	0.847	0.108891	0.3167	0.1667	-4	18	-0.9	-4	3	0	0	0.000000	-2.2333	-1	0.1167	0.700000	0.3333	0.3667	0	0	0	0	2.2333	0.1161	-6.7	0	1	0	0	0.3436	1.0738	0.8023	0.3333	0.1181	0.6437	0.1088	0.0136	-0.2606
HIP_12306	GSHLVEALY	YGEEGAPGK	GSHLVEALYYGEEGAPGK		SCG1	171/367830	1.000	12306	0.847	0.137322	-0.15	0.1667	-1	18	-0.4889	-2	2	0	0	0.000000	-3.1667	-1	0.1333	2.000000	0.3333	1.6667	0	1	0	1	3.1667	-0.25	-6.3333	0	1	0	0	0.4269	1.1338	0.869	0.1667	0.1823	0.7551	-0.064	0.0178	-0.4222
HIP_12307	VCGERGFFY	CFETLRGDE	VCGERGFFYCFETLRGDE		CMGA	2409/247050	0.999	12307	0.847	0.008547	1.0167	0	-2	18	-0.2667	-2	4	0	0	0.000000	-0.8333	-1	0.05	1.325000	0.2222	1.1028	0	0	0	0	0.8333	1.1212	-3.3333	0	1	0	0	0.3436	1.1922	0.7256	0.3333	0.1181	0.5266	0.3493	0.0025	-0.0417
HIP_12308	GERGFFYTP	PRPQSEESW	GERGFFYTPPRPQSEESW		SCG1	4579/367830	0.999	12308	0.847	-0.013898	-1.8833	0.5	-1	18	-1.5278	-1	3	0	0	0.000000	-1.3667	1	0.0667	0.700000	0.1111	0.5889	1	0	1	0	-1.3667	-1.1091	-4.1	0	0	0	1	0.5936	0.7638	1.2223	-0.1667	0.3523	1.494	-1.1179	0.0044	-0.0911
HIP_12309	CGERGFFY	WSLYPSDSQ	CGERGFFYWSLYPSDSQ		SCG1	4434/367830	0.999	12309	0.847	0.056654	1.0667	0.1667	-1	17	-0.6706	-1	5	1	0	0.000000	-0.7333	0	0.0667	0.000000	0	0	0	0	0	0	0	0	-3.6667	0	1	0	0	0.3436	1.0905	0.7856	0.6667	0.1181	0.6172	0.3665	0.0044	-0.0889

HIP_12765	HLVEALYLV	VIAYLKRLV	HLVEALYLVVIAYLKRLV		SCG2	4028/334890	1.000	12765	0.846	-0.001004
HIP_12766	HLVEALYLV	EMIRALEYI	HLVEALYLVEMIRALEYI		SCG2	2118/334890	1.000	12766	0.846	0.026073
HIP_12767	TSICSLYQL	DYETQQWPE	TSICSLYQLDYETQQWPE		SCG2	8541/334890	0.999	12767	0.846	0.040427
HIP_12768	AGSLQPLAL	ALRQAENEP	AGSLQPLALALRQAENEP		SCG2	9719/334890	0.999	12768	0.846	0.064264
HIP_12769	VCGERGFFY	SEGLSQGLV	VCGERGFFYSEGLSQGLV		CMGA	555/247050	1.000	12769	0.846	0.184290
HIP_12770	LVEALYLVC	EEEEATNTH	LVEALYLVCEEEEATNTH		CMGA	758/247050	1.000	12770	0.846	0.124466
HIP_12771	GERGFFYTP	RQAENEPQS	GERGFFYTPRQAENEPQS		SCG2	10093/334890	0.999	12771	0.846	-0.057175
HIP_12772	SHLVEALYL	LEEPGETQN	SHLVEALYLLEEPGETQN		SCG1	469/367830	1.000	12772	0.846	0.089767
HIP_12773	DLQVGQVEL	EWEDSKRWS	DLQVGQVELEWEDSKRWS		CMGA	7829/247050	0.997	12773	0.846	0.068297
HIP_12774	VCGERGFFY	DLLMRESTE	VCGERGFFYDLLMRESTE		NPY	265/49410	0.999	12774	0.846	-0.012908
HIP_12775	HLVEALYLV	SEEDWMRII	HLVEALYLVSEEDWMRII		SCG2	616/334890	1.000	12775	0.846	0.169373
HIP_12776	GSLQPLAL	TCATQRLAN	GSLQPLALTCATQRLAN		IAPP	1026/45018	0.996	12776	0.846	0.064920
HIP_12777	VEALYLVC	FSEDVNWGY	VEALYLVCFSEDVNWGY		SCG1	1580/367830	1.000	12777	0.846	0.129159
HIP_12778	LVCGERGFF	ESWDEEDKR	LVCGERGFFESWDEEDKR		SCG1	3870/367830	0.999	12778	0.846	0.025923
HIP_12779	GERGFFYTP	YEKRNLARV	GERGFFYTPYEKRNLARV		SCG1	19650/367830	0.990	12779	0.846	0.030070
HIP_12780	CGERGFFYT	VLKTSRKDV	CGERGFFYTVLKTSRKDV		SCG1	11258/367830	0.997	12780	0.846	-0.028460
HIP_12781	VCGERGFFY	EDEKKELE	VCGERGFFYEDEKKELE		SCG1	5600/367830	0.999	12781	0.846	0.135718
HIP_12782	TSICSLYQL	EEEEATNTH	TSICSLYQLEEEEATNTH		CMGA	3088/247050	0.999	12782	0.846	0.078550
HIP_12783	VCGERGFFY	QTVLTEDEK	VCGERGFFYQTVLTEDEK		SCG1	701/367830	1.000	12783	0.846	0.083639
HIP_12784	LVCGERGFF	NHLKATPIE	LVCGERGFFNHLKATPIE		IAPP	990/45018	0.996	12784	0.846	0.038423
HIP_12785	VCGERGFFY	NIEKNEQI	VCGERGFFYNIEKNEQI		SCG2	8018/334890	0.999	12785	0.846	0.135611
HIP_12786	AGSLQPLAL	NRDSSMKLS	AGSLQPLALNRDSSMKLS		CMGA	8995/247050	0.996	12786	0.846	0.250214
HIP_12787	VCGERGFFY	DNPFKRTN	VCGERGFFYDNPFKRTN		SCG2	6487/334890	0.999	12787	0.846	0.184990
HIP_12788	GERGFFYT	PNPYNQEK	GERGFFYTPNPYNQEK		SCG2	5564/334890	0.999	12788	0.846	0.133910
HIP_12789	GERGFFYTP	ESHQVEKRK	GERGFFYTPESHQVEKRK		IAPP	1973/45018	0.991	12789	0.846	-0.013924
HIP_12790	VCGERGFFY	PEDLIEMLK	VCGERGFFYPEDLIEMLK		SCG2	2278/334890	1.000	12790	0.846	0.150935
HIP_12791	RGFFYTPKT	SKRWSKMDQ	RGFFYTPKTSKRWSKMDQ		CMGA	18103/247050	0.979	12791	0.846	0.048827
HIP_12792	HLCGSHLVE	PQSAPKENK	HLCGSHLVEPQSAPKENK		SCG2	7742/334890	0.999	12792	0.846	0.181847
HIP_12793	CSLYQLENY	QESKAEGNN	CSLYQLENYQESKAEGNN		CMGA	9185/247050	0.996	12793	0.846	-0.018377
HIP_12794	AGSLQPLAL	VENRQMAYE	AGSLQPLALVENRQMAYE		SCG2	984/334890	1.000	12794	0.846	0.131667
HIP_12795	CGERGFFY	DNMNDNFLE	CGERGFFYDNMNDNFLE		SCG1	7141/367830	0.999	12795	0.846	0.118847
HIP_12796	GSLQPLAL	QNAFLNERK	GSLQPLALQNAFLNERK		SCG1	11444/367830	0.997	12796	0.846	0.174844
HIP_12797	LVEALYLVC	FSEDVNWGY	LVEALYLVCFSEDVNWGY		SCG1	150/367830	1.000	12797	0.846	0.105576
HIP_12798	TSICSLYQL	LGNKRLGLS	TSICSLYQLLGNKRLGLS		NPY	327/49410	0.999	12798	0.846	0.058992
HIP_12799	CGERGFFY	GAEAASFQR	CGERGFFYGAEAASFQR		SCG2	3310/334890	1.000	12799	0.846	0.169027
HIP_12800	CGERGFFYT	EENELTLN	CGERGFFYTEENELTLN		SCG1	7992/367830	0.998	12800	0.846	0.100741
HIP_12801	SHLVEALYL	HLPERDSLS	SHLVEALYLHLPERDSLS		SCG2	6543/334890	1.000	12801	0.846	0.108598
HIP_12802	LVCGERGFF	NGQNGERA	LVCGERGFFNGQNGERA		SCG2	4381/334890	1.000	12802	0.846	0.132569
HIP_12803	CGERGFFY	EGMVTRQI	CGERGFFYEGMVTRQI		SCG1	4417/367830	0.999	12803	0.846	0.182506
HIP_12804	GSLQPLALE	SEEYRAPRP	GSLQPLALESEEYRAPRP		SCG1	11514/367830	0.997	12804	0.846	0.167141
HIP_12805	AGSLQPLAL	ETLRGDERI	AGSLQPLALETLRGDERI		CMGA	2912/247050	0.999	12805	0.846	0.149807
HIP_12806	VEALYLVC	LDRNYLNYG	VEALYLVCLDRNYLNYG		SCG1	13749/367830	0.996	12806	0.846	0.162955
HIP_12807	VEALYLVCG	PNNQKRERM	VEALYLVCGPNNQKRERM		SCG2	3308/334890	1.000	12807	0.846	0.106422
HIP_12808	GSHLVEALY	KEIRKGESR	GSHLVEALYKEIRKGESR		CMGA	12165/247050	0.992	12808	0.846	0.161507
HIP_12809	RGFFYTPKT	NPYQGVSVP	RGFFYTPKTNPYQGVSVP		SCG2	8871/334890	0.999	12809	0.846	-0.010864
HIP_12810	RGFFYTPKT	QGSSQETDK	RGFFYTPKTQGSSQETDK		SCG2	5697/334890	0.999	12810	0.846	-0.056865
HIP_12811	AGSLQPLAL	FSEDVNWGY	AGSLQPLALFSEDVNWGY		SCG1	275/367830	1.000	12811	0.846	0.154998
HIP_12812	LVCGERGFF	KRTNEIVEE	LVCGERGFFKRTNEIVEE		SCG2	15200/334890	0.998	12812	0.846	0.098363
[illegible]										

HIP_13276	VCGERGFF	QNGQNGERA	VCGERGFFQNGQNGERA		SCG2	4567/334890	1.000	13276	0.845	0.129466	-0.3667	0.3333	0	17	-1.0294	0	2
HIP_13277	CGERGFFYT	EEGAPGKW	CGERGFFYTEEGAPGKW		SCG1	5595/367830	0.999	13277	0.845	0.141294	-1.1	0.1667	-1	17	-0.8882	-1	3
HIP_13278	CSLYQLENY	SGYPKTPGR	CSLYQLENYSGYPKTPGR		SCG2	5286/334890	1.000	13278	0.845	-0.073849	-1.8	0.3333	1	18	-1.0556	1	2
HIP_13279	QCCTSICSL	WPERKLKHM	QCCTSICSLWPERKLKHM		SCG2	11954/334890	0.998	13279	0.845	0.111027	-0.0833	0.3333	3	18	-0.3222	2	1
HIP_13280	ICSLYQLEN	NPYYDPLQW	ICSLYQLENNPYYDPLQW		SCG1	6682/367830	0.999	13280	0.845	-0.036632	-1.6	0.1667	-2	18	-0.6333	-2	3
HIP_13281	LVCGERGFF	SRSEALAVD	LVCGERGFFSRSEALAVD		CMGA	5020/247050	0.998	13281	0.845	0.132963	-0.15	0.5	-1	18	0.3222	-1	2
HIP_13282	GSHLVEALY	VLSVALNHL	GSHLVEALYVLSVALNHL		IAPP	197/45018	0.999	13282	0.845	-0.018384	1.9167	0.1667	1	18	1.0278	-1	1
HIP_13283	HLVEALYLV	DSQSIYQLI	HLVEALYLVDSQSIYQLI		SCG2	2039/334890	1.000	13283	0.845	0.202213	-0.1833	0.1667	-1	18	0.7222	-2	1
HIP_13284	VCGERGFFY	LAPVSKRFP	VCGERGFFYLAPVSKRFP		SCG2	3438/334890	1.000	13284	0.845	0.014019	1.3833	0.1667	2	18	0.1333	2	3
HIP_13285	SHLVEALY	RFPVGPPKN	SHLVEALYRFPVGPPKN		SCG2	13229/334890	0.998	13285	0.845	0.197951	0.1667	0.1667	2	17	-0.3118	1	2
HIP_13286	ERGFFYTPK	QGAWKELDR	ERGFFYTPKQGAWKELDR		SCG1	3643/367830	0.999	13286	0.845	-0.094693	-1.3833	0.3333	1	18	-1.3833	1	3
HIP_13287	CGERGFFYT	SLIPLIFLI	CGERGFFYTSLIPLIFLI		SCG2	2698/334890	1.000	13287	0.845	-0.062715	1.3833	0.1667	0	18	1.2556	0	3
HIP_13288	SHLVEALYL	DEDLLMKVL	SHLVEALYLDEDLLMKVL		SCG2	3335/334890	1.000	13288	0.845	0.145245	-0.7	0	-2	18	0.65	-3	1
HIP_13289	VEALYLVC	EAENEKDRA	VEALYLVCEAENEKDRA		SCG1	3416/367830	1.000	13289	0.845	0.103250	0.8833	0	-3	17	-0.4	-3	1
HIP_13290	CGERGFFY	EEERLSKEW	CGERGFFYEEERLSKEW		CMGA	4368/247050	0.999	13290	0.845	0.136685	-1.0333	0	-2	17	-1.3118	-2	3
HIP_13291	GERGFFYTP	DSQVSEEVK	GERGFFYTPDSQVSEEVK		SCG1	4572/367830	0.999	13291	0.845	-0.066806	-1.9	0.3333	-2	18	-0.9944	-2	3
HIP_13292	CGERGFFY	QDKQYSSHH	CGERGFFYQDKQYSSHH		SCG1	16051/367830	0.994	13292	0.845	0.215758	-1.1	0	2	17	-1.5118	0	4
HIP_13293	AGSLQPLAL	PQWSLYPSD	AGSLQPLALPQWSLYPSD		SCG1	4705/367830	0.999	13293	0.845	0.186447	0.5667	0.1667	-1	18	-0.0833	-1	1
HIP_13294	SLYQLENYC	KNFFPEYNY	SLYQLENYCKNFFPEYNY		SCG1	11465/367830	0.997	13294	0.845	-0.035311	-1.15	0	-1	18	-0.9333	-1	3
HIP_13295	CGERGFFYT	EPMQESKAE	CGERGFFYTEPMQESKAE		CMGA	2148/247050	0.999	13295	0.845	0.039353	-0.4	0.1667	-2	18	-1.0722	-2	3
HIP_13296	LVCGERGFF	ESSSRGEAG	LVCGERGFFESSSRGEAG		SCG1	392/367830	1.000	13296	0.845	0.118135	0.0167	0.5	-1	18	-0.3111	-1	2
HIP_13297	SHLVEALYL	PGKWQQQG	SHLVEALYLPGKWQQQG		SCG1	7296/367830	0.999	13297	0.845	0.233748	0.0667	0.3333	1	17	-0.5353	0	2
HIP_13298	SHLVEALYL	DILNLLGME	SHLVEALYLDILNLLGME		SCG2	2675/334890	1.000	13298	0.845	0.022162	1.85	0	-2	18	0.8611	-3	1
HIP_13299	SHLVEALYL	LLMKVLEYL	SHLVEALYLLLMKVLEYL		SCG2	2725/334890	1.000	13299	0.845	-0.001484	2.6333	0	0	18	1.1778	-1	1
HIP_13300	GSHLVEALY	QELGEYLAR	GSHLVEALYQELGEYLAR		SCG2	2331/334890	1.000	13300	0.845	0.098501	0.1833	0	-1	18	-0.1611	-2	1
HIP_13301	ERGFFYTPK	EEIKGYPGV	ERGFFYTPKEEIKGYPGV		SCG1	13754/367830	0.996	13301	0.845	-0.019014	-1.45	0.1667	0	18	-0.9	0	2
HIP_13302	AGSLQPLAL	PRAYFMSDT	AGSLQPLALPRAYFMSDT		SCG1	12037/367830	0.997	13302	0.845	0.200755	0.85	0.1667	0	18	0.1556	0	1
HIP_13303	SICSLYQLE	ERYRGRGSE	SICSLYQLEERYRGRGSE		SCG1	26235/367830	0.977	13303	0.845	-0.017761	-2.0833	0	0	18	-1.0389	0	2
HIP_13304	TSICSLYQL	MESAANQKT	TSICSLYQLMESAANQKT		SCG2	8802/334890	0.999	13304	0.845	0.044042	-0.5667	0.1667	0	18	-0.1611	0	1
HIP_13305	GGPGAGSLQ	SKEWEDSKR	GGPGAGSLQSKEWEDSKR		CMGA	13509/247050	0.990	13305	0.845	0.010019	-1.45	0.3333	0	18	-1.5111	0	1
HIP_13306	VCGERGFFY	RGDERILSI	VCGERGFFYRGDERILSI		CMGA	5673/247050	0.998	13306	0.845	0.216982	-0.6833	0.1667	0	18	-0.1222	0	3
HIP_13307	QHLCGSHLV	QEAENEKDR	QHLCGSHLVQEAENEKDR		SCG1	8154/367830	0.998	13307	0.845	0.144212	-0.0667	0	0	18	-1.3556	-2	0
HIP_13308	LVCGERGFF	SNQVKRVP	LVCGERGFFSNQVKRVP		SCG2	9454/334890	0.999	13308	0.845	0.114260	-0.4333	0.3333	2	17	-0.1235	2	2
HIP_13309	VCGERGFFY	DEEEEEGEN	VCGERGFFYDEEEEEGEN		SCG1	602/367830	1.000	13309	0.845	0.102610	-1.0333	0	-7	18	-1.4556	-7	3
HIP_13310	CGERGFFYT	EEPVSTHQ	CGERGFFYTEEPVSTHQ		SCG1	6425/367830	0.999	13310	0.845	0.100164	-1.3	0.1667	-1	17	-0.9	-2	3
HIP_13311	LVCGERGFF	KTHWLGAAL	LVCGERGFFKTHWLGAAL		SCG2	16147/334890	0.997	13311	0.845	0.076461	-0.4333	0.1667	2	18	0.5222	1	2
HIP_13312	VCGERGFFY	SKSGYPKT	VCGERGFFYSKSGYPKT		SCG2	11417/334890	0.999	13312	0.845	0.228128	-0.2	0.3333	2	17	-0.6588	2	3
HIP_13313	CGERGFFYT	EETGSQEN	CGERGFFYTEETGSQEN		SCG1	8279/367830	0.998	13313	0.845	0.098866	-1.15	0	-3	17	-1.3	-3	3
HIP_13314	HLVEALYLV	GENYQKGE	HLVEALYLVGENYQKGE		SCG1	2170/367830	1.000	13314	0.845	0.194568	-0.1167	0.1667	-1	17	-0.3765	-2	2
HIP_13315	GSLQPLAL	VDKRRTRP	GSLQPLALVDKRRTRP		SCG1	19891/367830	0.990	13315	0.845	0.203419	1.0333	0	3	16	-0.7562	3	0
HIP_13316	LVCGERGFF	SSHFERRDN	LVCGERGFFSSHFERRDN		SCG1	8892/367830	0.998	13316	0.845	0.059751	0.0667	0.5	1	18	-0.7889	0	3
HIP_13317	FVNQHLCGS	SGYPKTPGR	FVNQHLCGSSGYPKTPGR		SCG2	4344/334890	1.000	13317	0.845	-0.013315	-0.2	0.6667	3	18	-0.7389	2	1
HIP_13318	HLVEALYLV	SNQLPKAAW	HLVEALYLVSNQLPKAAW		SCG2	4855/334890	1.000	13318	0.845	0.208217	-0.1833	0.1667	1	18	0.3778	0	1

HIP_13787	VCGERGFF	QDTKENRE	VCGERGFFQDTKENRE		SCG1	7977/367830	0.998	13787	0.844	0.087790	-0.4167	0.1667	-1	16	-1.4438	-1	2	0	0	0.000000	-4.3	-1	0.0667	0.700000	0.4444	0.2556	0	1	0	1	4.3	-0.1065	-8.6	1	0	0	0	0.4551	0.9769	0.9245	0.1667	0.2071	0.8547	-0.1896	0.0044	-0.2867
HIP_13788	LVCGERGFF	QFPPMYEEN	LVCGERGFFQFPPMYEEN		SCG2	569/334890	1.000	13788	0.844	0.016163	0.4833	0.3333	-2	18	-0.3611	-2	3	0	0	0.000000	-2.5	0	0.0333	0.700000	0.2222	0.4778	1	1	1	1	0	0.2309	-7.5	1	0	0	0	0.4333	0.9633	0.9667	0.5	0.1878	0.9344	0.2094	0.0011	-0.0833
HIP_13789	QHLCGSHLV	VIAYLKRLV	QHLCGSHLVVIAYLKRLV		SCG2	8297/334890	0.999	13789	0.844	0.017109	2.55	0	4	18	0.8778	2	1	0	0	1.000000	1.9	0	0	2.000000	0	2	0	0	0	0	0	5.1	1.9	1	0	0	0	0.2385	1.1619	0.5895	0.6667	0.0569	0.3475	0.6081	0	0
HIP_13790	CGERGFFY	AGQVTALPV	CGERGFFYAGQVTALPV		CMGA	3234/247050	0.999	13790	0.844	0.182105	0.3667	0.1667	0	17	0.4471	0	3	1	0	0.000000	-2.1333	0	0.1333	0.000000	0	0	0	1	0	1	0	0	-6.4	0	1	0	0	0.3885	1.0803	0.8745	0.5	0.1509	0.7647	0.1424	0.0178	-0.2844
HIP_13791	VCGERGFFY	NLNDKDQE	VCGERGFFYNLNDKDQE		SCG2	6511/334890	0.999	13791	0.844	0.121705	0.1833	0	-2	17	-1.1118	-2	3	1	0	0.000000	-2.5	0	0.1	0.700000	0.2222	0.4778	0	0	0	0	0	0.0876	-7.5	0	1	0	0	0.3718	1.0103	0.9545	0.5	0.1382	0.911	0.0682	0.01	-0.25
HIP_13792	SHLVEALYL	GPNNQKRE	SHLVEALYLGPNNQKRE		SCG2	2302/334890	1.000	13792	0.844	0.238097	0.1333	0.3333	1	17	-0.9294	0	1	1	0	0.000000	-3.9333	0	0.2	0.000000	0.1111	-0.1111	1	1	1	1	0	-0.0148	-3.9333	1	0	0	0	0.4718	0.8936	1.0812	0.3333	0.2226	1.1689	0.0629	0.04	-0.7867

[illegible]

HIP_14298	LYQLENYCN	PRAYFMSDT	LYQLENYCNPRAYFMSDT		SCG1	5504/367830	0.999	14298	0.844	0.000143	-1.1	0.1667	-1	18	-0.6889	-1	2	0	0	0.000000	-0.6667	1	0.0667	0.625000	0.2222	0.4028	1	0	1	0	-0.6667	-0.4431	-1.3333	0	1	0	0	0.4654	0.9591	1.0768	0	0.2166	1.1595	-0.5119	0.0044	-0.0444
HIP_14299	LYQLENYCN	EEEEEMAVV	LYQLENYCNEEEEEMAVV		CMGA	564/247050	1.000	14299	0.844	0.022349	-2.1333	0	-6	18	-0.6611	-6	1	0	3	0.000000	-2.7333	-3	0.1	0.625000	0.5556	0.0694	0	0	0	0	8.2	-0.1481	-2.7333	0	1	0	0	0.5436	1.2072	0.9323	-0.5	0.2955	0.8692	-1.1597	0.01	-0.2733
HIP_14300	LVEALYLVC	SEEDWMRII	LVEALYLVCSEEDWMRII		SCG2	861/334890	1.000	14300	0.844	0.154112	0.45	0.1667	-3	18	0.75	-3	1	0	0	0.000000	-6.1	-2	0.1667	2.000000	0.3333	1.6667	0	0	0	0	12.2	0.75	-6.1	0	0	0	0	0.4385	1.2003	0.7645	0	0.1922	0.5846	0.1972	0.0278	-1.0167
HIP_14301	QHLCGSHLV	HYINLITRQ	QHLCGSHLVHYINLITRQ		NPY	2816/49410	0.978	14301	0.844	0.062070	0.8	0	4	18	-0.0389	1	1	0	0	0.000000	-1.6	0	0	0.950000	0	0.95	0	0	0	0	0	0.76	-1.6	1	0	0	0	0.3154	1.0841	0.6985	0.1667	0.0995	0.4878	0.2523	0	0
HIP_14302	SHLVEALY	EPRAYFMSD	SHLVEALYEPRAYFMSD		SCG1	9999/367830	0.998	14302	0.844	0.215898	-0.8833	0.1667	-1	17	-0.2294	-2	2	1	0	0.000000	-4.6333	0	0.1667	0.000000	0.3333	-0.3333	1	0	1	0	0	0.2944	-9.2667	0	1	0	0	0.4269	1.1455	0.8723	0	0.1823	0.7609	-0.3771	0.0278	-0.7722
HIP_14303	CGERGFFY	RQVLKTSRK	CGERGFFYRQVLKTSRK		SCG1	24256/367830	0.982	14303	0.844	0.187071	0.0833	0	4	17	-0.9294	4	3	1	0	0.000000	-2.7	1	0.1	0.000000	0.2222	-0.2222	0	0	0	0	-2.7	-0.0185	-8.1	0	1	0	0	0.3436	1.1022	0.749	0.3333	0.1181	0.561	0.0286	0.01	-0.27
HIP_14304	TSICSLYQL	SKEWEDSKR	TSICSLYQLSKEWEDSKR		CMGA	11754/247050	0.993	14304	0.844	0.203635	-1.5333	0.1667	0	18	-0.9889	0	2	0	0	0.000000	-2.4	0	0.1167	0.000000	0.2222	-0.2222	0	0	0	0	0	0.3407	-4.8	1	0	0	0	0.4769	1.1272	0.8723	-0.1667	0.2275	0.7609	-0.7313	0.0136	-0.28
HIP_14305	LVCGERGFF	FLNERKQAS	LVCGERGFFFLNERKQAS		SCG1	693/367830	1.000	14305	0.844	0.010243	1.3833	0.1667	1	18	-0.2222	1	3	0	1	1.000000	-0.7	0	-0.0333	1.400000	0.3333	1.0667	0	1	0	1	0	1.4756	-2.1	1	0	0	0	0.35	1.0117	0.8933	0.6667	0.1225	0.798	0.4842	0.0011	0.0233
HIP_14306	TSICSLYQL	LQQKENGDE	TSICSLYQLLQQKENGDE		SCG2	4603/334890	1.000	14306	0.844	-0.042137	-0.7	0	-2	18	-0.7778	-2	1	0	0	1.000000	-0.7333	0	0.05	0.950000	0.1111	0.8389	0	0	0	0	0	-0.5872	-0.7333	1	0	0	0	0.4385	1.1586	0.8078	0.3333	0.1922	0.6526	-0.3069	0.0025	-0.0367
HIP_14307	VCGERGFFY	GKLTGPNN	VCGERGFFYGKLTGPNN		SCG2	7850/334890	0.999	14307	0.844	0.193916	0.6333	0.1667	1	17	-0.4706	1	3	1	0	0.000000	-1.6	1	0.1333	0.700000	0.2222	0.4778	0	1	0	1	-1.6	0.3026	-4.8	0	1	0	0	0.3603	1.0405	0.8356	0.3333	0.1298	0.6983	0.2282	0.0178	-0.2133
HIP_14308	GSHLVEALY	PEYNYDWWE	GSHLVEALYPEYNYDWWE		SCG1	397/367830	1.000	14308	0.844	0.086109	-0.35	0.1667	-3	18	-0.8667	-4	2	0	0	0.000000	-3.5667	-1	0.1167	2.000000	0.2222	1.7778	1	0	1	0	3.5667	-0.6222	-7.1333	0	1	0	0	0.4269	1.1455	0.8723	0.1667	0.1823	0.7609	-0.1494	0.0136	-0.4161
HIP_14309	VCGERGFFY	PSPMPVSQE	VCGERGFFYPSPMPVSQE		CMGA	542/247050	1.000	14309	0.844	0.183831	0.05	0.5	-1	18	-0.2833	-1	3	0	0	0.000000	-2.7667	0	0.2	1.175000	0.1111	1.0639	2	0	1	0	0	0.0532	-8.3	0	1	0	0	0.4718	0.8936	1.0578	0.3333	0.2226	1.119	0.0236	0.04	-0.5533
HIP_14310	LYQLENYCN	FERRDNMND	LYQLENYCNFERRDNMND		SCG1	15202/367830	0.995	14310	0.844	-0.036869	-1.25	0	-2	18	-1.5722	-2	2	0	0	0.000000	-0.9667	0	0.05	1.325000	0.4444	0.8806	0	0	0	0	0	-1.1007	-1.9333	0	1	0	0	0.4487	1.0741	0.9368	-0.1667	0.2013	0.8776	-0.5609	0.0025	-0.0483
HIP_14311	HLVEALYLV	QGAWKELDR	HLVEALYLVQGAWKELDR		SCG1	1467/367830	1.000	14311	0.844	0.170497	0.7667	0.1667	0	18	-0.0556	-1	2	0	0	0.000000	-2.9333	0	0.1333	1.400000	0	1.4	0	1	0	1	0	1.0733	-5.8667	1	0	0	0	0.3885	1.0736	0.8412	0.5	0.1509	0.7075	0.2978	0.0178	-0.3911
HIP_14312	GSLQPLAL	TATCATQRL	GSLQPLALTATCATQRL		IAPP	881/45018	0.997	14312	0.844	0.141481	1.6333	0	1	17	0.3941	1	0	1	0	0.000000	-3	0	0.1	1.850000	0	1.85	0	0	0	0	0	3.0217	0	1	0	0	0	0.3333	1.1933	0.7067	0.6667	0.1111	0.4994	0.5444	0.01	-0.3
HIP_14313	SHLVEALY	LGNKRLGLS	SHLVEALYLGNKRLGLS		NPY	756/49410	0.996	14313	0.844	0.222939	0.7	0.1667	2	17	0.1588	1	1	1	0	0.000000	-1.4667	0	0.1333	0.000000	0.3333	-0.3333	0	1	0	1	0	-0.2333	-1.4667	0	1	0	0	0.3885	1.0403	0.9378	0.5	0.1509	0.8795	0.2719	0.0178	-0.1956
HIP_14314	SHLVEALYL	EQYTPQSL	SHLVEALYLEQYTPQSL		SCG2	10729/334890	0.999	14314	0.844	0.147656	-0.3333	0	-1	17	-0.1471	-2	2	1	0	0.000000	-4.8667	-1	0.1	0.000000	0.2222	-0.2222	0	0	0	0	4.8667	0.0741	-9.7333	1	0	0	0	0.4103	1.2105	0.7556	0.1667	0.1683	0.571	-0.1368	0.01	-0.4867
HIP_14315	TSICSLYQL	NDEMKRSGQ	TSICSLYQLNDEMKRSGQ		SCG2	11525/334890	0.999	14315	0.844	0.139137	-1.9167	0	0	18	-0.7889	0	1	0	0	0.000000	-3.1667	-2	0.1	0.475000	0.2222	0.2528	0	0	0	0	6.3333	-0.4845	-3.1667	1	0	0	0	0.4769	1.1055	0.9323	-0.1667	0.2275	0.8692	-0.9141	0.01	-0.3167
HIP_14316	LVEALYLVC	EGHHRVQEN	LVEALYLVCEGHHRVQEN		SCG1	2407/367830	1.000	14316	0.844	0.160285	0.5667	0.1667	0	18	-0.1	-2	1	0	0	0.000000	-5.8667	-1	0.1333	2.000000	0.1111	1.8889	0	1	0	1	5.8667	1.0704	-5.8667	0	0	0	0	0.4269	1.0688	0.8423	0	0.1823	0.7095	0.2419	0.0178	-0.7822
HIP_14317	LVCGERGFF	SEEKHLEE	LVCGERGFFSEEKHLEE		SCG1	3891/367830	0.999	14317	0.844	0.074520	-0.4333	0.3333	-2	17	-0.6353	-3	2	1	0	0.000000	-4.3333	-2	0.0833	0.700000	0.5556	0.1444	0	1	0	1	8.6667	-0.0626	-8.6667	1	0	0	0	0.4833	1.12	0.8967	0	0.2336	0.804	-0.2094	0.0069	-0.3611
HIP_14318	CGERGFFY	NQVKRVPG	CGERGFFYNQVKRVPG		SCG2	14176/334890	0.998	14318	0.844	0.164281	0.25	0	2	16	-0.6875	2	3	0	0	0.000000	-2.3667	0	0.1	0.700000	0.2222	0.4778	0	0	0	0	0	0.1194	-7.1	0	1	0	0	0.3718	1.0436	0.8578	0.5	0.1382	0.7359	0.0929	0.01	-0.2367
HIP_14319	VCGERGFFY	ADTEKWAE	VCGERGFFYADTEKWAE		SCG1	7024/367830	0.999	14319	0.844	0.114599	0.3167	0	-2	17	-0.6	-2	3	1	0	0.000000	-2.2333	-1	0.1	1.150000	0.3333	0.8167	0	0	0	0	2.2333	0.2586	-6.7	0	1	0	0	0.3436	1.1205	0.7723	0.3333	0.1181	0.5965	0.1088	0.01	-0.2233
HIP_14320	VCGERGFFY	LKELQDLAL	VCGERGFFYLKELQDLAL		CMGA	6970/247050	0.997	14320	0.844	0.097665	0.1167	0	-1	18	0.2667	-1	3	0	0	0.000000	-2.6333	0	0.1	2.600000	0.3333	2.2667	0	0	0	0	0	0.2644	-7.9	0	1	0	0	0.3436	1.2088	0.709	0.1667	0.1181	0.5026	0.0401	0.01	-0.2633
HIP_14321	CGERGFFYT	TQNAFLNE	CGERGFFYTTQNAFLNE		SCG1	11353/367830	0.997	14321	0.844	0.120419	-1.15	0	-1	17	-0.5294	-1	3	1	0	0.000000	-2.8333	0	0.1	0.450000	0	0.45	0	0	0	0	0	-0.5175	-8.5	0	1	0	0	0.5051	0.9436	0.9945	0.1667	0.2552	0.989	-0.5809	0.01	-0.2833
HIP_14322	LVCGERGFF	SQESGEET	LVCGERGFFSQESGEET		SCG1	2894/367830	1.000	14322	0.844	0.081514	-0.4333	0.3333	-3	17	-0.5529	-3	2	1	0	0.000000	-4.3333	-2	0.0833	0.700000	0.3333	0.3667	0	1	0	1	4.3333	-0.1589	-8.6667	1	0	0	0	0.4833	1.0533	0.9367	0.1667	0.2336	0.8773	-0.2094	0.0069	-0.3611
HIP_14323	VCGERGFFY	LALSLLVCL	VCGERGFFYLALSLLVCL		NPY	426/49410	0.998	14323	0.844	-0.038708	2.2833	0	0	18	1.6056	0	3	0	0	0.000000	1.7	0	0	1.650000	0.1111	1.5389	0	0	0	0	0	3.5138	5.1	0	1	0	0	0.2385	1.2453	0.6278	0.8333	0.0569	0.3942	0.5445	0	0
HIP_14324	LCGSHLVEA	YGFRGPGPQ	LCGSHLVEAYGFRGPGPQ		CMGA	5577/247050	0.998	14324	0.844	0.035970	0.6	0.1667	1	18	-0.15	0	2	0	0	0.000000	-0.4667	1	0.0333	0.000000	0.2222	-0.2222	0	1	0	1	-0.4667	-0.1333	-0.9333	1	0	0	0	0.3885	1.1236	0.8178	0.3333	0.1509	0.6688	0.2331	0.0011	-0.0156
HIP_14325	AGSLQPLAL	ENIEKNEQI	AGSLQPLALENIEKNEQI		SCG2	1514/334890	1.000	14325	0.844	0.140980	1.15	0	-2	18	-0.4	-2	0	0	0	0.000000	-3.9667	-1	0.1	0.450000	0.2222	0.2278	0	0	0	0	3.9667	0.2619	0	1	0	0	0	0.3333	1.2183	0.7383	0.5	0.1111	0.5451	0.3833	0.01	-0.3967
HIP_14326	LVEALYLVC	MARYYSALR	LVEALYLVCMARYYSALR		NPY	1953/49410	0.987	14326	0.844	0.086617	1.6167	0	1	18	0.9	1	2	0	0	0.000000	-3.7667	1	0.05	2.475000	0.1111	2.3639	0	0	0	0	-3.7667	3.8216	-7.5333	0	0	0	0	0.2769	1.1755	0.679	0.5	0.0767	0.461	0.4477	0.0025	-0.1883
HIP_14327	LCGSHLVEA	WGYEKRNLA	LCGSHLVEAWGYEKRNLA		SCG1	12289/367830	0.997	14327	0.844	0.069387	-0.0167	0.1667	1	18	-0.2333	0	2	0	0	1.000000	-1.7	1	0.0333	0.000000	0.2222	-0.2222	0	1	0	1	-1.7	0.0037	-3.4	1	0	0	0	0.4269	1.0938	0.869	0.3333	0.1823	0.7551	-0.0071	0.0011	-0.0567
HIP_14328	YLVCGERGF	EEEEEEATN	YLVCGERGFEEEEEEATN		CMGA	577/247050	1.000	14328	0.844	-0.037767	-2.1	0.1667	-6	18	-1.1222	-6	1	0	4	0.000000	-2.8	-4	0.0167	0.000000	0.6667	-0.6667	0	1	0	1	11.2	1.4	-2.8	1	0	0	0	0.5218	1.2086	0.8712	-0.5	0.2723	0.7589	-1.0958	0.0003	-0.0467
HIP_14329	SHLVEALYL	EEEGSANR	SHLVEALYLEEEGSANR		CMGA	3937/247050	0.999	14329	0.844	0.158024	-0.7	0	-2	17	-0.5471	-3	1	1	1	0.000000	-5.6	-3	0.15	0.000000	0.4444	-0.4444	0	0	0	0	16.8	0.3111	-5.6	1	0	0	0	0.4385	1.3586	0.6878	-0.1667	0.1922	0.4731	-0.3069	0.0225	-0.84
HIP_14330	VCGERGFFY	EPGETQNA	VCGERGFFYEPGETQNA		SCG1	1477/367830	1.000	14330	0.844	0.185658	-0.2	0.3333	-2	17	-0.7471	-2	3	1	0	0.000000	-3.2667	-1	0.2	0.700000	0.3333	0.3667	1	1	1	1	3.2667	-0.0733	-9.8	0	1	0	0	0.4718	1.0003	0.9778	0.1667	0.2226	0.9561	-0.0944	0.04	-0.6533
HIP_14331	HLVEALYLV	HNEGMVTRC	HLVEALYLVHNEGMVTRC		SCG1	10656/367830	0.997	14331	0.844	0.147414	-0.5833	0	1	18	0.3556	-1	1	0	0	0.000000	-5.6333	-1	0.1	1.400000	0.1111	1.2889	0	0	0	0	5.6333	-0.7519	-5.6333	1	0	0	0	0.4103	1.0972	0.8523	0	0.1683	0.7264	-0.2393	0.01	-0.5633
HIP_14332	LVCGERGFF	RNYLNYGEE	LVCGERGFFRNYLNYGEE		SCG1	13740/367830	0.996	14332	0.844	0.034828	-0.6833	0.1667	-1	18	-0.5778	-1	3	0	0	0.000000	-4.8333	1	0.0167	1.650000	0.3333	1.3167	0	1	0	1	-4.8333	-0.8997	-14.5	1	0	0	0	0.4269	0.9355	1.0123	0.1667	0.1823	1.0248	-0.2917	0.0003	-0.0806
HIP_14333	VCGERGFF	EEPGETQNA	VCGERGFFEEPGETQNA		SCG1	2036/367830	1.000	14333	0.844	0.061386	-0.5667	0.3333	-3	17	-0.8765	-3	2	1	0	0.000000	-4.6	-2	0.0833	0.700000	0.4444	0.2556	1	1	1	1	9.2	-0.1448	-9.2	1	0	0	0	0.5	1.0817	0.95	0	0.25	0.9025	-0.2833	0.0069	-0.3833
HIP_14334	FVNQHLCGS	NQEKAEKGR	FVNQHLCGSNQEKAEKGR		SCG2	10897/334890	0.999	14334	0.844	-0.017270	-1.5333	0.3333	2	18	-1.2778	1	0	0	0	0.000000	-3.9333	-1	0	0.000000	0.2222	-0.2222	0	1	0	1	3.9333	0.3407	0	0	1	0	0	0.5885	0.9353	1.1478	-0.1667	0.3463	1.3175	-0.9023	0	0
HIP_14335	CGERGFFYT	MAYENLNDK	CGERGFFYTMAYENLNDK		SCG2	2556/334890	1.000	14335	0.844	-0.034004	0.5333	0	-1	18	-0.8	-1	4	0	0	0.000000	0.5333	0	-0.05	0.475000	0.1111	0.3639	0	0	0	0	0	0.1941	2.1333	0	1	0	0	0.3436	1.1205	0.7723	0.5	0.1181	0.5965	0.1832	0.0025	-0.0267
HIP_14336	SHLVEALYL	EKVAHQLQA	SHLVEALYLEKVAHQLQA		CMGA	11040/247050	0.993	14336	0.844	0.204839	0.5167	0	1	18	0.1444	-1	1	0	0	0.000000	-3.1667	0	0.1	0.450000	0.3333	0.1167	0	0	0	0	0	0.0603	-3.1667	1	0	0	0	0.3436	1.2022	0.6756	0.1667	0.1181	0.4565	0.1775	0.01	-0.3167
HIP_14337	VCGERGFFY	REDSKEAE	VCGERGFFYREDSKEAE		CMGA	7511/247050	0.997	14337	0.844	0.181801	-1.2	0	-2	17	-1.1294	-2	3	1	0	0.000000	-5.2667	-1	0.15	0.700000	0.4444	0.2556	0	0	0	0	5.2667	-0.3067	-15.8	0	1	0	0	0.3821	1.1624	0.7768	-0.1667	0.146	0.6034	-0.4585	0.0225	-0.79
HIP_14338	VCGERGFFY	FPSPEMIRA	VCGERGFFYFPSPEMIRA		SCG2	220/334890	1.000	14338	0.844	0.136162	0.7833	0.3333	0	18	0.0667	0	4	0	0	0.000000	-1.3	0	0.1333	1.400000	0.1111	1.2889	1	0	1	0	0	1.0096	-5.2	0	1	0	0	0.3885	0.9986	0.9045	0.5	0.1509	0.8181	0.3043	0.0178	-0.1733
HIP_14339	CGERGFFYT	TEALPDGLS	CGERGFFYTTEALPDGLS		SCG2	5383/334890	0.999	14339	0.844	-0.005307	-0.2667	0	-2	18	-0.2111	-2	3	0	0	0.000000	-1.0667	-1	0.05	0.950000	0.1111	0.8389	0	0	0	0	1.0667	-0.2237	-3.2	0	1	0	0	0.4385	1.1403	0.8045	0.1667	0.1922	0.6472	-0.1169	0.0025	-0.0533
HIP_14340	CGERGFFY	RVPGQGSSE	CGERGFFYRVPGQGSSE		SCG2	8690/334890	0.999	14340	0.844	0.154112	0.4	0.1667	0	17	-0.7824	0	3	1	0	0.000000	-2.0667	1	0.1167	1.050000	0.1111	0.9389	1	0	1	0	-2.0667	0.2756	-6.2	0	1	0	0	0.3603	1.0122	0.839	0.3333	0.1298	0.7039	-0.1441	0.0136	-0.241

HIP_14809	LVCGERGFF	PYALNSEKN	LVCGERGFFPYALNSEKN		SCG2	2116/334890	1.000	14809	0.843	-0.003721	0.6833	0.3333	0	18	-0.2889	0	3	0	0
HIP_14810	QCCTSICSL	AILSSTNVG	QCCTSICSLAILSSTNVG		IAPP	28/45018	1.000	14810	0.843	0.056305	2.6	0.1667	0	18	1.0056	0	0	0	0
HIP_14811	AGSLQPLAL	LNEKNFFPE	AGSLQPLALLNEKNFFPE		SCG1	2605/367830	1.000	14811	0.843	0.134190	1.0333	0	-1	18	-0.0778	-1	0	0	0
HIP_14812	CGERGFFY	PKAAWIPHV	CGERGFFYPKAAWIPHV		SCG2	13574/334890	0.998	14812	0.843	0.162500	0.1	0.1667	2	17	-0.0529	1	4	1	0
HIP_14813	TSICSLYQL	ESTENVPRT	TSICSLYQLESTENVPRT		NPY	937/49410	0.995	14813	0.843	0.191004	-1	0.1667	-1	18	-0.3944	-1	1	0	0
HIP_14814	GSHLVEALY	PRHHHGRSR	GSHLVEALYPRHHHGRSR		SCG1	21463/367830	0.987	14814	0.843	0.173913	-0.8333	0.1667	6	18	-1.1944	2	1	0	1
HIP_14815	CGERGFFYT	NNIAYEDVV	CGERGFFYTNNIAYEDVV		SCG2	2557/334890	1.000	14815	0.843	0.004518	-0.2833	0	-2	18	-0.1833	-2	3	0	0
HIP_14816	VEALYLVCG	SFQRNQLLQ	VEALYLVCGSFQRNQLLQ		SCG2	3614/334890	1.000	14816	0.843	0.042306	0.8	0.3333	0	18	0.3444	0	2	0	0
HIP_14817	CTSICSLYQ	RMLVKYPEI	CTSICSLYQRMLVKYPEI		SCG2	12149/334890	0.998	14817	0.843	-0.045325	0.0333	0	1	18	0.3222	1	1	0	0
HIP_14818	AGSLQPLAL	PDFYDSEEP	AGSLQPLALPDFYDSEEP		SCG1	276/367830	1.000	14818	0.843	0.122202	1.1833	0.1667	-4	18	-0.4333	-4	2	0	0
HIP_14819	SHLVEALY	QGLFRGGKS	SHLVEALYQGLFRGGKS		CMGA	4966/247050	0.998	14819	0.843	0.214923	0.7	0.1667	2	17	-0.1471	1	2	1	0
HIP_14820	CGERGFFYT	GGEPRAYFM	CGERGFFYTGGEPRAYFM		SCG1	781/367830	1.000	14820	0.843	0.184935	-0.5833	0.3333	0	18	-0.4389	0	3	0	0
HIP_14821	CTSICSLYQ	EDKRNYPSL	CTSICSLYQEDKRNYPSL		SCG1	20787/367830	0.988	14821	0.843	0.170998	-1.9833	0	0	18	-0.7	0	1	0	0
HIP_14822	RGFFYTPKT	RQQAHKEES	RGFFYTPKTRQQAHKEES		SCG2	24224/334890	0.994	14822	0.843	0.001918	-2.95	0.1667	3	18	-1.7833	2	1	0	0
HIP_14823	CGERGFFY	EPQSAPKE	CGERGFFYEPQSAPKE		SCG2	6151/334890	0.999	14823	0.843	0.186119	-0.7167	0.1667	-1	16	-1.1625	-1	3	0	0
HIP_14824	SHLVEALYL	GDTEVMKCI	SHLVEALYLGDTEVMKCI		CMGA	558/247050	1.000	14824	0.843	0.226564	0.2833	0.1667	-1	18	0.5389	-2	1	0	0
HIP_14825	VEALYLVC	ERYRGRGSE	VEALYLVCERYRGRGSE		SCG1	18843/367830	0.991	14825	0.843	0.180015	0.2	0	0	17	-0.4647	0	2	1	0
HIP_14826	LVEALYLVC	EKGHPQEES	LVEALYLVCEKGHPQEES		SCG1	3704/367830	0.999	14826	0.843	0.206154	0.45	0.1667	-2	18	-0.2556	-3	1	0	0
HIP_14827	ICSLYQLEN	YNPYQGVSV	ICSLYQLENYNPYQGVSV		SCG2	8887/334890	0.999	14827	0.843	-0.018203	-1.6	0.1667	-1	18	-0.1111	-1	3	0	0
HIP_14828	LVCGERGFF	QSEESWDEE	LVCGERGFFQSEESWDEE		SCG1	472/367830	1.000	14828	0.843	0.054939	-0.4333	0.3333	-5	18	-0.9	-5	2	0	0
HIP_14829	LVCGERGFF	DKRRTRPRH	LVCGERGFFDKRRTRPRH		SCG1	26444/367830	0.976	14829	0.843	0.148434	-1.1167	0.1667	5	18	-1.3111	4	2	0	0
HIP_14830	GERGFFYTP	EGENYQKGE	GERGFFYTPEGENYQKGE		SCG1	2311/367830	1.000	14830	0.843	-0.029304	-1.8333	0.3333	-2	18	-1.6833	-2	3	0	0
HIP_14831	ICSLYQLEN	QSAPKENKP	ICSLYQLENQSAPKENKP		SCG2	15193/334890	0.998	14831	0.843	-0.033306	-0.95	0.1667	0	18	-1.0278	0	1	0	0
HIP_14832	AGSLQPLAL	AGRSRSNQ	AGSLQPLALAGRSRSNQ		SCG2	11358/334890	0.999	14832	0.843	0.226079	1.05	0.1667	2	17	-0.4412	2	0	1	0
HIP_14833	LVCGERGFF	EDDIYKANN	LVCGERGFFEDDIYKANN		SCG2	5492/334890	0.999	14833	0.843	0.039031	-0.8833	0.1667	-2	18	-0.5056	-2	2	0	0
HIP_14834	CGERGFFYT	EKRDHHSTH	CGERGFFYTEKRDHHSTH		SCG1	24605/367830	0.981	14834	0.843	0.164488	-1.85	0	3	18	-1.6222	0	3	0	0
HIP_14835	LVEALYLVC	FFPEYNYDW	LVEALYLVCFFPEYNYDW		SCG1	255/367830	1.000	14835	0.843	0.028432	2.4167	0.1667	-3	18	0.5167	-3	3	0	0
HIP_14836	SHLVEALY	PEYGEEIKG	SHLVEALYPEYGEEIKG		SCG1	5715/367830	0.999	14836	0.843	0.149445	-0.35	0.1667	-2	17	-0.5176	-3	2	1	0
HIP_14837	SHLVEALYL	EGAPGKWQQ	SHLVEALYLEGAPGKWQQ		SCG1	2491/367830	1.000	14837	0.843	0.209383	0.7	0.1667	0	18	-0.4056	-1	1	0	0
HIP_14838	GERGFFYTP	RERADEPQW	GERGFFYTPRERADEPQW		SCG1	19820/367830	0.990	14838	0.843	0.021243	-2.6833	0.1667	-1	18	-1.6944	-1	3	0	0
HIP_14839	QHLCGSHLV	LLQKEPDLR	QHLCGSHLVLLQKEPDLR		SCG2	9405/334890	0.999	14839	0.843	0.031805	1.4833	0	2	18	-0.3278	0	0	0	0
HIP_14840	CGERGFFYT	WDEDLLMKV	CGERGFFYTWDEDLLMKV		SCG2	10359/334890	0.999	14840	0.843	0.058628	-1.1833	0	-2	18	-0.2389	-2	4	0	0
HIP_14841	VCGERGFFY	EEGEEDAT	VCGERGFFYEEGEEDAT		SCG1	684/367830	1.000	14841	0.843	0.157883	-0.5167	0.1667	-5	17	-0.8588	-5	3	1	0
HIP_14842	GSHLVEALY	EYRAPRPQ	GSHLVEALYEYRAPRPQ		SCG1	17919/367830	0.992	14842	0.843	0.126594	-0.8333	0	1	17	-0.8412	0	2	1	0
HIP_14843	AGSLQPLAL	FSEDVNWG	AGSLQPLALFSEDVNWG		SCG1	1772/367830	1.000	14843	0.843	0.161391	1.3167	0.1667	-2	17	0.1824	-2	1	1	0
HIP_14844	VCGERGFFY	ILEALRQAE	VCGERGFFYILEALRQAE		SCG2	3503/334890	1.000	14844	0.843	-0.022249	1.5167	0	-1	18	0.1611	-1	3	0	0
HIP_14845	CCTSICSLY	EESWDEEDK	CCTSICSLYEESWDEEDK		SCG1	316/367830	1.000	14845	0.843	0.017414	-1.0167	0.3333	-5	18	-0.8	-5	2	0	0
HIP_14846	LVCGERGFF	NAFLNERKQ	LVCGERGFFNAFLNERKQ		SCG1	3987/367830	0.999	14846	0.843	0.015878	1.05	0.1667	1	18	-0.3722	1	3	0	0
HIP_14847	AGSLQPLAL	EEEEATNTH	AGSLQPLALEEEEATNTH		CMGA	1561/247050	1.000	14847	0.843	0.134535	-0.1833	0	-3	18	-0.6444	-4	0	0	2
HIP_14848	CGERGFFY	EPGETQNAF	CGERGFFYEPGETQNAF		SCG1	2201/367830	1.000	14848	0.843	0.192364	-0.2	0.3333	-2	17	-0.8294	-2	3	1	0
HIP_14849	SHLVEALYL	FLNERKQA	SHLVEALYLFLNERKQA		SCG1	10412/367830	0.998	14849	0.843	0.084664	1.5667	0	1	17	-0.1118	0	2	1	0
HIP_14850	AGSLQPLAL	EELVARSET	AGSLQPLALEELVARSET		SCG1	1506/367830	1.000	14850	0.843	0.085483	1.0333	0	-2	18	0.1111	-2	0	0	0
HIP_14851	SHLVEALYL	LTEDEKKEL	SHLVEALYLLTEDEKKEL		SCG1	1174/367830	1.000	14851	0.843	0.120138	0.9833	0	-2	18	-0.35	-3	1	0	0

HIP_15320	LVCGERGFF	ESRSEALAV	LVCGERGFFESRSEALAV		CMGA	7393/247050	0.997	15320	0.842	0.117857	-0.6	0.3333	-1	18	0.3222	-1	2	0	0	0.000000	-4.6667	0	0.0833	0.700000	0.4444	0.2556	0	1	0	1	0	-0.1533	-9.3333	1	0	0	0	0.4551	1.0386	0.9245	0	0.2071	0.8547	-0.2731	0.0069	-0.3889
HIP_15321	VEQCCTSIC	YNPYQGVSV	VEQCCTSICYNPYQGVSV		SCG2	6226/334890	0.999	15321	0.842	0.036508	-0.0333	0.3333	-1	18	0.2056	-1	2	0	0	0.000000	-4.2	0	0.05	1.750000	0	1.75	1	0	1	0	0	-0.0583	-8.4	0	0	0	0	0.4936	0.8605	1.094	0.1667	0.2436	1.1968	-0.0165	0.0025	-0.21
HIP_15322	GSLQPLAL	KWAEGGGHS	GSLQPLALKWAEGGGHS		SCG1	19100/367830	0.991	15322	0.842	0.123248	1.0667	0	1	17	-0.2824	0	1	1	1	0.000000	-4.1333	1	0.05	0.950000	0.2222	0.7278	0	0	0	0	-4.1333	0.7763	-4.1333	1	0	0	0	0.2769	1.2572	0.689	0.6667	0.0767	0.4747	0.2954	0.0025	-0.2067
HIP_15323	GERGFFYTP	LDLKRQYDR	GERGFFYTPLDLKRQYDR		SCG1	5623/367830	0.999	15323	0.842	-0.050859	0.0833	0.1667	1	18	-1.3278	1	3	0	0	0.000000	2.5667	-1	-0.0667	1.650000	0.2222	1.4278	1	0	1	0	-2.5667	0.119	7.7	0	0	0	1	0.4269	1.0072	0.8823	0.1667	0.1823	0.7785	0.0356	0.0044	-0.1711
HIP_15324	LVCGERGFF	YRGRGSEEY	LVCGERGFFYRGRGSEEY		SCG1	12606/367830	0.996	15324	0.842	0.077856	-0.1667	0.3333	0	18	-0.7167	0	3	0	0	0.000000	-3.8	1	0.05	0.700000	0.4444	0.2556	0	2	0	1	-3.8	-0.0426	-11.4	1	0	0	0	0.4436	0.9072	1.0023	0.1667	0.1968	1.0046	-0.0739	0.0025	-0.19
HIP_15325	SHLVEALYL	ELQDLALQG	SHLVEALYLELQDLALQG		CMGA	3747/247050	0.999	15325	0.842	0.147455	0.5167	0	-2	18	0.4111	-3	1	0	0	0.000000	-3.1667	-1	0.1	0.000000	0.3333	-0.3333	0	0	0	0	3.1667	-0.1722	-3.1667	1	0	0	0	0.3718	1.2569	0.6878	0.3333	0.1382	0.4731	0.1921	0.01	-0.3167
HIP_15326	CGERGFFYT	NHLKATPIE	CGERGFFYTNHLKATPIE		IAPP	2169/45018	0.989	15326	0.842	0.045190	-0.35	0	1	18	-0.5	0	3	0	0	0.000000	-1.2333	0	0	0.000000	0.1111	-0.1111	0	0	0	0	0	0.0389	-3.7	0	1	0	0	0.4103	1.0072	0.9056	0.1667	0.1683	0.8202	-0.1436	0	0
HIP_15327	AGSLQPLAL	IAYEDVVGG	AGSLQPLALIAYEDVVGG		SCG2	3016/334890	1.000	15327	0.842	-0.000134	2.4	0	-2	18	0.7944	-2	1	0	0	1.000000	-1.4667	0	0	1.575000	0.1111	1.4639	0	0	0	0	0	3.5133	-1.4667	1	0	0	0	0.2385	1.2669	0.6162	0.8333	0.0569	0.3796	0.5723	0	0
[illegible]																																														

HIP_15831	LVCGERGFF	ESSPDYNPY	LVCGERGFFESSPDYNPY		SCG2	233/334890	1.000	15831	0.842	0.104864	0.0167	0.5	-2	18	-0.5889	-2	2	0
HIP_15832	SHLVEALYL	FPNPYNQE	SHLVEALYLFPNPYNQE		SCG2	2609/334890	1.000	15832	0.842	0.159781	0.6667	0.1667	-1	17	-0.4176	-2	2	1
HIP_15833	SLYQLENYC	TSRKDVKDK	SLYQLENYCTSRKDVKDK		SCG1	17722/367830	0.992	15833	0.842	0.164627	-1.3833	0.1667	1	18	-1.35	1	1	0
HIP_15834	QHLCGSHLV	QFPPMYEEN	QHLCGSHLVQFPPMYEEN		SCG2	791/334890	1.000	15834	0.842	0.165439	0.4167	0.1667	0	18	-0.5889	-2	1	0
HIP_15835	GERGFFYTP	EAELEKVAH	GERGFFYTPEAELEKVAH		CMGA	4756/247050	0.998	15835	0.842	-0.124705	-1.4667	0.1667	-1	18	-0.7111	-2	3	0
HIP_15836	VCGERGFFY	EISRNLQI	VCGERGFFYEISRNLQI		SCG2	7903/334890	0.999	15836	0.842	0.170145	0.75	0.1667	0	17	-0.0471	0	3	1
HIP_15837	AGSLQPLAL	KMDQLAKEL	AGSLQPLALKMDQLAKEL		CMGA	10875/247050	0.994	15837	0.842	0.208412	0.65	0	0	18	0.0944	0	0	0
HIP_15838	HLVEALYLV	LQVFLIVLS	HLVEALYLVLQVFLIVLS		IAPP	427/45018	0.999	15838	0.842	0.006204	1.8667	0	0	18	2.0222	-1	2	0
HIP_15839	CGERGFFY	EESNVSMA	CGERGFFYEESNVSMA		SCG1	6380/367830	0.999	15839	0.842	0.172466	-0.5833	0.1667	-2	16	-0.3875	-2	3	0
HIP_15840	LVCGERGFF	KGESRSEAL	LVCGERGFFKGESRSEAL		CMGA	6802/247050	0.997	15840	0.842	0.142785	-0.4333	0.3333	0	18	-0.25	0	2	0
HIP_15841	CSLYQLENY	QNGQNGERA	CSLYQLENYQNGQNGERA		SCG2	5594/334890	0.999	15841	0.842	-0.012365	-2.6167	0.1667	-1	18	-1.3778	-1	1	0
HIP_15842	VCGERGFFY	DSQSIYQL	VCGERGFFYDSQSIYQL		SCG2	7673/334890	0.999	15842	0.842	0.195797	-0.5833	0.1667	-1	17	-0.1706	-1	3	1
HIP_15843	HLVEALYLV	DFYDSEEPV	HLVEALYLVDFYDSEEPV		SCG1	4905/367830	0.999	15843	0.842	0.029004	0.7833	0	-4	18	0.1611	-5	3	0
HIP_15844	QHLCGSHLV	YKANNIAYE	QHLCGSHLVYKANNIAYE		SCG2	22248/334890	0.995	15844	0.842	0.116544	0.2333	0	2	18	-0.3167	0	1	0
HIP_15845	SHLVEALYL	REDSLEAG	SHLVEALYLREDSLEAG		CMGA	7045/247050	0.997	15845	0.842	0.211762	-0.8667	0	-2	17	-0.1176	-3	1	1
HIP_15846	GERGFFYTP	EEERLSKE	GERGFFYTPEEERLSKE		CMGA	7858/247050	0.997	15846	0.842	-0.001764	-2.35	0.1667	-2	17	-1.5412	-2	3	1
HIP_15847	ICSLYQLEN	EAASFQRNQ	ICSLYQLENEAASFQRNQ		SCG2	10771/334890	0.999	15847	0.842	0.001890	-0.5167	0	-1	18	-0.6056	-1	1	0
HIP_15848	CGERGFFY	EEEEEAEA	CGERGFFYEEEEEAEA		CMGA	1887/247050	0.999	15848	0.842	0.110482	-1.0333	0	-6	16	-1.2125	-6	3	0
HIP_15849	DLQVGQVEL	FPEYNYDWW	DLQVGQVELFPEYNYDWW		SCG1	2107/367830	1.000	15849	0.842	0.028953	0.3667	0.1667	-4	18	-0.6722	-4	2	0
HIP_15850	LVCGERGFF	EHSQQKEEE	LVCGERGFFEHSQQKEEE		CMGA	4243/247050	0.999	15850	0.842	0.029303	-0.3833	0.3333	-2	18	-1.2	-3	2	0
HIP_15851	AGSLQPLAL	KTSRKDVKD	AGSLQPLALKTSRKDVKD		SCG1	15232/367830	0.995	15851	0.842	0.255906	0.6667	0.1667	2	18	-0.6556	2	0	0
HIP_15852	CTSICSLYQ	APVSKRFPV	CTSICSLYQAPVSKRFPV		SCG2	6824/334890	0.999	15852	0.842	-0.043861	0.5667	0.1667	2	18	0.3778	2	1	0
HIP_15853	GERGFFYTP	DVNWGYEKR	GERGFFYTPDVNWGYEKR		SCG1	6605/367830	0.999	15853	0.842	-0.090509	-1.0667	0.1667	0	18	-1.3389	0	4	0
HIP_15854	GSHLVEALY	LQKEPDLRL	GSHLVEALYLQKEPDLRL		SCG2	10383/334890	0.999	15854	0.842	0.099526	0.1167	0	0	18	-0.2611	-1	1	0
HIP_15855	AGSLQPLAL	KRSSPETLI	AGSLQPLALKRSSPETLI		NPY	2394/49410	0.983	15855	0.842	0.218185	0.0333	0.1667	1	18	0.0667	1	0	0
HIP_15856	CGERGFFY	FPPMYEENS	CGERGFFYFPPMYEENS		SCG2	1360/334890	1.000	15856	0.842	0.123277	0.65	0.3333	-2	17	-0.7706	-2	5	1
HIP_15857	SHLVEALYL	KEEEEEMA	SHLVEALYLKEEEEEMA		CMGA	4496/247050	0.999	15857	0.842	0.184908	-0.7667	0	-4	17	-0.5353	-5	1	1
HIP_15858	SICSLYQLE	YNYDWWEKK	SICSLYQLEYNYDWWEKK		SCG1	14621/367830	0.995	15858	0.842	-0.060184	-1.55	0	-1	18	-1	-1	3	0
HIP_15859	CGERGFFYT	DELSEVLEN	CGERGFFYTDELSEVLEN		CMGA	4034/247050	0.999	15859	0.842	0.007985	-0.4	0	-4	18	-0.5111	-4	3	0
HIP_15860	SHLVEALYL	ESNVSMASL	SHLVEALYLESNVSMASL		SCG1	3731/367830	0.999	15860	0.842	0.209314	-0.25	0.1667	-1	18	0.6056	-2	1	0
HIP_15861	TSICSLYQL	VNAAGSGRL	TSICSLYQLVNAAGSGRL		SCG2	7342/334890	0.999	15861	0.842	-0.045046	0.25	0	1	18	0.5278	1	1	0
HIP_15862	GSLQPLAL	YQGVSVPLQ	GSLQPLALYQGVSVPLQ		SCG2	5422/334890	0.999	15862	0.842	0.214274	0.7	0.1667	0	17	0.4706	0	1	1
HIP_15863	GERGFFYTP	YGEEGAPGK	GERGFFYTPYGEEGAPGK		SCG1	2469/367830	1.000	15863	0.842	-0.085250	-1.4667	0.3333	-1	18	-1.1111	-1	4	0
HIP_15864	AGSLQPLAL	SSQAELKEA	AGSLQPLALSSQAELKEA		CMGA	1872/247050	0.999	15864	0.842	0.225943	0.7167	0.3333	-1	18	0.0056	-1	0	0
HIP_15865	CGERGFFYT	REEARFQD	CGERGFFYTREEARFQD		SCG1	16816/367830	0.993	15865	0.842	0.128906	-1.7833	0	-1	17	-1.2412	-1	3	1
HIP_15866	CGERGFFYT	TLNEKNFFP	CGERGFFYTTLNEKNFFP		SCG1	10580/367830	0.997	15866	0.842	0.039476	0.0667	0	0	18	-0.5556	0	3	0
HIP_15867	CGERGFFY	LNDKDQELG	CGERGFFYLNDKDQELG		SCG2	9042/334890	0.999	15867	0.842	0.134139	0.1833	0	-2	17	-0.9529	-2	3	1
HIP_15868	LYQLENYCN	DEEDKRNYP	LYQLENYCNDEEDKRNYP		SCG1	8714/367830	0.998	15868	0.842	0.060760	-2.1333	0	-3	18	-1.9611	-3	1	0
HIP_15869	GERGFFYT	QQGDLQDTK	GERGFFYTQQGDLQDTK		SCG1	6406/367830	0.999	15869	0.842	0.122235	-1.1	0.1667	-1	17	-1.4059	-1	3	1
HIP_15870	GERGFFYT	EESEESNVS	GERGFFYTEESEESNVS		SCG1	6278/367830	0.999	15870	0.842	0.095381	-1.1667	0.1667	-4	17	-1.2294	-4	3	1
HIP_15871	HLVEALYL	ELDRNYLNY	HLVEALYLELDRNYLNY		SCG1	4353/367830	0.999	15871	0.842	0.096431	0.5167	0	-1	17	-0.2412	-2	1	1
HIP_15872	GERGFFYTP	SEDVNWGYE	GERGFFYTPSEDVNWGYE		SCG1	4742/367830	0.999	15872	0.842	-0.074696	-1.9	0.3333	-3	18	-1.1111	-3	3	0
HIP_15873	AGSLQPLAL	NNQAPGEEE	AGSLQPLALNNQAPGEEE		CMGA	1942/247050	0.999	15873	0.842	0.191064	-0.1833	0	-3	18	-0.6944	-3	0	0
HIP_15874	HLVEALYLV	MLKTGEKPN	HLVEALYLVMLKTGEKPN		SCG2	11224/334890	0.999	15874	0.842	0.058846	1.4167	0	1	18	0.1	0	1	0
HIP_15875	SHLVEALYL	TEALPDGLS	SHLVEALYLTEALPDGLS		SCG2	1749/334890	1.000	15875	0.842	0.125853	0.65	0	-2	18	0.4167	-3	1	0
HIP_15876	GIVEQCCTS	EWEDSKRWS	GIVEQCCTSEWEDSKRWS		CMGA	6497/247050	0.998	15876	0.842	-0.027420	-1.15	0.1667	-2	18	-0.9722	-2	1	0
HIP_15877	LYQLENYCN	EMKRSGQLG	LYQLENYCNEMKRSGQLG		SCG2	26332/334890	0.993	15877	0.842	0.060760	-1.3	0	0	18	-0.9889	0	1	0
HIP_15878	EDLQVGQVE	QWPERKLKH	EDLQVGQVEQWPERKLKH		SCG2	11882/334890	0.998	15878	0.842	-0.118852	-1.4667	0.1667	0	18	-1.4944	-1	1	0
HIP_15879	LVEALYLVC	NAAGSGRLQ	LVEALYLVCNAAGSGRLQ		SCG2	7196/334890	0.999	15879	0.842	0.068862	1.7667	0	0	18	0.7556	0	1	0
HIP_15880	RGFFYTPKT	GAWKELDRN	RGFFYTPKTGAWKELDRN		SCG1	3734/367830	0.999	15880	0.842	0.013159	-0.95	0.3333	2	18	-1.2278	2	2	0
HIP_15881	CGERGFFY	QLSDDVSKV	CGERGFFYQLSDDVSKV		SCG2	5271/334890	1.000	15881	0.842	0.152614	0.6333	0.1667	-1	17	-0.3412	-1	3	1
HIP_15882	HLVEALYLV	YGEEGAPG	HLVEALYLVYGEEGAPG		SCG1	1717/367830	1.000	15882	0.842	0.147895	0.25	0.1667	-2	17	0.2529	-3	2	1
HIP_15883	VEALYLVCG	NPFKRTNEI	VEALYLVCGNPFKRTNEI		SCG2	1959/334890	1.000	15883	0.842	0.028668	0.6667	0.3333	0	18	0.0667	0	2	0

HIP_16342	SLYQLENYC	DWNPVEEKI	SLYQLENYCDWNPVEEKI		SCG2	6074/334890	0.999	16342	0.841	-0.023171	-1.7	0	-3	18	-0.8611	-3	2	0	0
HIP_16343	VCGERGFF	PSEEKGHPQ	VCGERGFFPSEEKGHPQ		SCG1	3555/367830	1.000	16343	0.841	0.115750	-0.1167	0.5	0	17	-1.0882	-1	2	1	0
HIP_16344	CTSICSLYQ	EEQKLYTDD	CTSICSLYQEEQKLYTDD		SCG2	7095/334890	0.999	16344	0.841	0.081274	-1.9167	0	-3	18	-0.7444	-3	1	0	0
HIP_16345	GSHLVEALY	TPNRQYWDE	GSHLVEALYTPNRQYWDE		SCG2	747/334890	1.000	16345	0.841	0.181305	-0.25	0.1667	-1	18	-1.0333	-2	1	0	0
HIP_16346	ERGFFYTPK	KDQELGEYL	ERGFFYTPKKDQELGEYL		SCG2	16481/334890	0.997	16346	0.841	-0.040314	-2.85	0.1667	-1	18	-1.2389	-1	2	0	0
HIP_16347	CGERGFFY	FPEYNYDWW	CGERGFFYFPEYNYDWW		SCG1	7058/367830	0.999	16347	0.841	0.116754	0.3333	0.1667	-2	17	-0.9235	-2	5	1	0
HIP_16348	RGFFYTPKT	NWGYEKRNL	RGFFYTPKTNWGYEKRNL		SCG1	10274/367830	0.998	16348	0.841	-0.026060	-1.8333	0.3333	3	18	-1.4	3	3	0	0
HIP_16349	VCGERGFFY	RSQEESEE	VCGERGFFYRSQEESEE		SCG1	11104/367830	0.997	16349	0.841	0.190234	-0.75	0.1667	-3	17	-1.2588	-3	3	1	0
HIP_16350	VCGERGFFY	EALRQAEN	VCGERGFFYEALRQAEN		SCG2	9931/334890	0.999	16350	0.841	0.045582	1.0667	0	-1	17	-0.5235	-1	3	1	0
HIP_16351	SHLVEALYL	DSQVSEEVK	SHLVEALYLDSQVSEEVK		SCG1	3326/367830	1.000	16351	0.841	0.201923	-0.25	0.1667	-2	18	-0.1389	-3	1	0	0
HIP_16352	SICSLYQLE	KENGDESHL	SICSLYQLEKENGDESHL		SCG2	18659/334890	0.997	16352	0.841	-0.038412	-2.35	0	-2	18	-0.7667	-3	1	0	0
HIP_16353	VNQHLCG	GSNTYGKRN	VNQHLCGGSNTYGKRN		IAPP	884/45018	0.997	16353	0.841	0.182224	0.2	0.5	3	16	-1.1938	2	1	2	0
HIP_16354	VCGERGFFY	SEAHESSS	VCGERGFFYSEAHESSS		SCG1	7001/367830	0.999	16354	0.841	0.145184	0.3	0.1667	-1	17	-0.5529	-2	3	1	1
HIP_16355	TSICSLYQL	RLGLSGLTL	TSICSLYQLRLGLSGLTL		NPY	635/49410	0.997	16355	0.841	0.064464	-0.35	0.1667	1	18	0.8833	1	1	0	0
HIP_16356	SLYQLENYC	PDYNPYQGV	SLYQLENYCPDYNPYQGV		SCG2	4036/334890	1.000	16356	0.841	-0.015070	-1.45	0.1667	-2	18	-0.9056	-2	2	0	0
HIP_16357	VEALYLVC	TQQWPERK	VEALYLVCTQQWPERK		SCG2	10121/334890	0.999	16357	0.841	0.193563	0.4667	0	0	16	-0.4125	0	2	0	0
HIP_16358	GERGFFYTP	RGSEEYRAP	GERGFFYTPRGSEEYRAP		SCG1	3487/367830	1.000	16358	0.841	0.003970	-1.55	0.5	0	18	-1.3944	0	3	0	0
HIP_16359	LVEALYLV	EGENYQKGE	LVEALYLVEGENYQKGE		SCG1	1665/367830	1.000	16359	0.841	0.162824	-0.1167	0.1667	-3	17	-0.3941	-3	2	1	0
HIP_16360	VCGERGFFY	KSSAPPIT	VCGERGFFYKSSAPPIT		SCG1	12033/367830	0.997	16360	0.841	0.217271	-0.2	0.3333	1	17	-0.0529	1	3	1	0
HIP_16361	VCGERGFF	QEKTHSREK	VCGERGFFQEKTHSREK		SCG1	19639/367830	0.990	16361	0.841	0.126728	-0.95	0.1667	2	17	-1.4118	1	2	1	0
HIP_16362	GERGFFYT	YSSHHTAEK	GERGFFYTYSSHHTAEK		SCG1	11858/367830	0.997	16362	0.841	0.089554	-0.35	0.3333	2	17	-1.2235	0	4	1	0
HIP_16363	AGSLQPLAL	KTSYFPNP	AGSLQPLALKTSYFPNP		SCG2	13468/334890	0.998	16363	0.841	0.257350	0.6667	0.1667	1	17	-0.1118	1	1	1	0
HIP_16364	CGERGFFY	LPRLPYGAG	CGERGFFYLPRLPYGAG		SCG2	13249/334890	0.998	16364	0.841	0.181630	0.3333	0.1667	1	17	-0.1412	1	3	1	0
HIP_16365	GSHLVEALY	NEPQSAPKE	GSHLVEALYNEPQSAPKE		SCG2	1924/334890	1.000	16365	0.841	0.173032	-0.7167	0.1667	-1	18	-0.8722	-2	1	0	0
HIP_16366	CGERGFFY	LEYIENLRQ	CGERGFFYLEYIENLRQ		SCG2	12621/334890	0.998	16366	0.841	0.076682	0.55	0	-1	17	-0.5706	-1	4	1	0
HIP_16367	QHLCGSHLV	QNGERATRL	QHLCGSHLVQNGERATRL		SCG2	6425/334890	0.999	16367	0.841	0.235831	-0.4333	0.1667	3	18	-0.6556	1	0	0	0
HIP_16368	ICSLYQLEN	EPQSAPKEN	ICSLYQLENEPQSAPKEN		SCG2	10155/334890	0.999	16368	0.841	0.028525	-1.9667	0.1667	-2	18	-1.0056	-2	1	0	0
HIP_16369	GSHLVEALY	VLKREPLNY	GSHLVEALYVLKREPLNY		IAPP	1149/45018	0.995	16369	0.841	-0.000569	1.4	0	1	18	-0.1167	0	1	0	0
HIP_16370	QHLCGSHLV	STENVPRTR	QHLCGSHLVSTENVPRTR		NPY	1336/49410	0.993	16370	0.841	0.234671	-0.0333	0.1667	3	18	-0.6889	1	0	0	0
HIP_16371	AGSLQPLAL	LEYLNQEKA	AGSLQPLALLEYLNQEKA		SCG2	6127/334890	0.999	16371	0.841	0.043667	1.4	0	-1	18	-0.0611	-1	1	0	0
HIP_16372	LQPLALEGS	QQWPERKLK	LQPLALEGSQQWPERKLK		SCG2	7173/334890	0.999	16372	0.841	0.030969	-2.1	0.3333	1	18	-1.0056	1	1	0	0
HIP_16373	DLQVGQVEL	QNGERATRL	DLQVGQVELQNGERATRL		SCG2	7502/334890	0.999	16373	0.841	0.211959	-0.4833	0.1667	-1	18	-0.7444	-1	0	0	0
HIP_16374	LVEALYLVC	CATQRLANF	LVEALYLVCCATQRLANF		IAPP	810/45018	0.997	16374	0.841	0.038384	2.35	0	0	18	1.1	0	1	0	0
HIP_16375	GPGAGSLQP	EEEMAVVPQ	GPGAGSLQPEEEMAVVPQ		CMGA	3142/247050	0.999	16375	0.841	-0.054306	-1.9667	0.1667	-3	18	-0.3667	-3	0	0	1
HIP_16376	GSHLVEALY	EKSQREDEE	GSHLVEALYEKSQREDEE		SCG1	5360/367830	0.999	16376	0.841	0.176668	-0.65	0.1667	-3	18	-1.4333	-4	1	0	0
HIP_16377	ICSLYQLEN	ANQKTSYFP	ICSLYQLENANQKTSYFP		SCG2	20624/334890	0.996	16377	0.841	-0.030463	-1.4	0	0	18	-0.4833	0	1	0	0
HIP_16378	SHLVEALYL	EEEATNTH	SHLVEALYLEEEATNTH		CMGA	3811/247050	0.999	16378	0.841	0.147389	-0.7	0	-2	17	-0.4824	-4	1	1	1
HIP_16379	CGERGFFYT	SQKYPGPQA	CGERGFFYTSQKYPGPQA		CMGA	12237/247050	0.992	16379	0.841	0.158911	-1.2333	0.1667	1	18	-0.9722	1	4	0	0
HIP_16380	CGERGFFYT	EGPTVVLNP	CGERGFFYTEGPTVVLNP		CMGA	631/247050	1.000	16380	0.841	0.158103	-0.7833	0.3333	-1	18	-0.1	-1	3	0	0
HIP_16381	LVEALYLVC	QESKGQPR	LVEALYLVCQESKGQPR		SCG1	7483/367830	0.999	16381	0.841	0.208978	0.45	0.1667	0	17	-0.1412	0	1	1	0
HIP_16382	ERGFFYTPK	TRHSEKSQR	ERGFFYTPKTRHSEKSQR		SCG1	25086/367830	0.980	16382	0.841	0.034045	-2.4333	0.1667	4	18	-1.9833	3	2	0	0
HIP_16383	CGERGFFY	RSSPETLIS	CGERGFFYRSSPETLIS		NPY	1644/49410	0.990	16383	0.841	0.225580	-0.3	0.3333	0	17	-0.3765	0	3	1	0
HIP_16384	PLALEGSLQ	EEEGPTVVL	PLALEGSLQEEEGPTVVL		CMGA	3199/247050	0.999	16384	0.841	-0.063037	-1.8333	0.1667	-4	18	0.1333	-4	0	0	1
HIP_16385	LCGSHLVEA	QNGQNGERA	LCGSHLVEAQNGQNGERA		SCG2	7245/334890	0.999	16385	0.841	0.212283	-0.8167	0.1667	0	18	-0.7111	-1	0	0	0
HIP_16386	LVCGERGFF	EDSLEAGLP	LVCGERGFFEDSLEAGLP		CMGA	1454/247050	1.000	16386	0.841	0.050035	-0.4333	0.3333	-3	18	0.1889	-3	2	0	0
HIP_16387	HLVEALYL	QDKQYSSHH	HLVEALYLQDKQYSSHH		SCG1	16061/367830	0.994	16387	0.841	0.205673	-0.7667	0	2	17	-0.8412	-1	2	1	0
HIP_16388	CCTSICSLY	YGEEGAPGK	CCTSICSLYYGEEGAPGK		SCG1	1987/367830	1.000	16388	0.841	-0.001654	-0.5833	0.3333	-1	18	-0.0556	-1	2	0	0
HIP_16389	LVEALYLVC	SAPKENKPY	LVEALYLVCSAPKENKPY		SCG2	1782/334890	1.000	16389	0.841	0.174265	1.65	0.3333	0	18	0.0556	0	1	0	0

HIP_16853	CGERGFFY	WKSSHFERR	CGERGFFYWKSSHFERR		SCG1	19986/367830	0.990	16853	0.840	0.178404	-0.2167	0.1667	3	17	-1.2529	2	4	1	0
HIP_16854	VCGERGFF	ESEESNVSM	VCGERGFFESEESNVSM		SCG1	4039/367830	0.999	16854	0.840	0.069162	-0.4333	0.3333	-3	17	-0.4	-3	2	1	0
HIP_16855	GSHLVEALY	FMSDTREEK	GSHLVEALYFMSDTREEK		SCG1	609/367830	1.000	16855	0.840	-0.020202	1.3667	0.1667	-1	18	-0.6278	-2	2	0	0
HIP_16856	ERGFFYTPK	NELTLNEKN	ERGFFYTPKNELTLNEKN		SCG1	10320/367830	0.998	16856	0.840	-0.042635	-1.5667	0.1667	0	18	-1.3778	0	2	0	0
HIP_16857	SLYQLENYC	ENKPYALNS	SLYQLENYCENKPYALNS		SCG2	16464/334890	0.997	16857	0.840	0.130278	-2.2	0	-1	18	-0.9056	-1	1	0	0
HIP_16858	AGSLQPLAL	LTGPNNQKR	AGSLQPLALLTGPNNQKR		SCG2	3536/334890	1.000	16858	0.840	0.176646	2.0167	0.1667	2	18	-0.5056	2	0	0	0
HIP_16859	LVEALYLVC	EDQELESLS	LVEALYLVCEDQELESLS		CMGA	1298/247050	1.000	16859	0.840	0.121446	0	0	-5	18	0.4333	-5	1	0	0
HIP_16860	VCGERGFFY	EGMVTRQ	VCGERGFFYEGMVTRQ		SCG1	3976/367830	0.999	16860	0.840	0.153285	0.3833	0.1667	0	17	0.3647	0	3	1	0
HIP_16861	CGERGFFY	SRKDVKDKE	CGERGFFYSRKDVKDKE		SCG1	19030/367830	0.991	16861	0.840	0.206800	-0.8167	0.1667	1	17	-1.4882	1	3	1	0
HIP_16862	GERGFFYTP	LNEKNFFPE	GERGFFYTPLNEKNFFPE		SCG1	3512/367830	1.000	16862	0.840	-0.156823	-1.1333	0.1667	-1	18	-0.9389	-1	3	0	0
HIP_16863	LVEALYLVC	HQVEKRKCN	LVEALYLVCHQVEKRKCN		IAPP	2047/45018	0.990	16863	0.840	0.104606	1.3333	0	2	18	0	1	1	0	0
HIP_16864	CGERGFFY	FPNPYNQE	CGERGFFYFPNPYNQE		SCG2	5010/334890	1.000	16864	0.840	0.112886	0.3333	0.1667	-1	16	-1.1062	-1	4	0	0
HIP_16865	AGSLQPLAL	DKARRHPQ	AGSLQPLALDKARRHPQ		SCG1	24963/367830	0.980	16865	0.840	0.233028	0.6333	0	3	17	-0.8353	2	0	1	0
HIP_16866	CGERGFFYT	DDTPNRQYW	CGERGFFYTDDTPNRQYW		SCG2	11783/334890	0.998	16866	0.840	0.120212	-1.15	0	-1	18	-1.4278	-1	3	0	0
HIP_16867	HLVEALYL	EQEHSQQK	HLVEALYLEQEHSQQK		CMGA	6048/247050	0.998	16867	0.840	0.183577	-0.7	0	0	16	-1	-2	1	0	0
HIP_16868	GSHLVEALY	HQNLLKELQ	GSHLVEALYHQNLLKELQ		CMGA	8780/247050	0.996	16868	0.840	0.096753	-0.9833	0	1	18	-0.2944	-1	1	0	0
HIP_16869	VCGERGFFY	GGEDWNPVE	VCGERGFFYGGEDWNPVE		SCG2	149/334890	1.000	16869	0.840	0.179440	0	0.3333	-3	18	-0.6056	-3	3	0	0
HIP_16870	VCGERGFFY	AQLDQLLH	VCGERGFFYAQLDQLLH		SCG1	8830/367830	0.998	16870	0.840	0.038858	1.0667	0	0	17	0.1	-1	3	1	0
HIP_16871	VCGERGFFY	IAYEDVVGG	VCGERGFFYIAYEDVVGG		SCG2	1398/334890	1.000	16871	0.840	-0.055771	1.55	0	-2	18	0.4333	-2	4	0	0
HIP_16872	LVCGERGFF	DNPFKRTN	LVCGERGFFDNPFKRTN		SCG2	7523/334890	0.999	16872	0.840	0.092673	-0.5667	0.3333	1	17	-0.6529	1	3	1	0
HIP_16873	ICSLYQLEN	WEKKPFSED	ICSLYQLENWEKKPFSED		SCG1	22731/367830	0.985	16873	0.840	0.015200	-1.9167	0	-2	18	-0.9333	-2	2	0	0
HIP_16874	LVEALYLVC	KSQREDEEE	LVEALYLVCKSQREDEEE		SCG1	7754/367830	0.998	16874	0.840	0.186353	0.3833	0.1667	-4	18	-0.6056	-4	1	0	1
HIP_16875	GSLQPLAL	GYEKRNLA	GSLQPLALGYEKRNLA		SCG1	8158/367830	0.998	16875	0.840	0.216244	0.7	0.1667	1	16	-0.2875	1	1	0	0
HIP_16876	VCGERGFF	SSPDYNPYQ	VCGERGFFSSPDYNPYQ		SCG2	975/334890	1.000	16876	0.840	0.115793	0.3333	0.6667	-1	17	-0.8471	-1	3	1	0
HIP_16877	CTSICSLYQ	FPNPYNQEK	CTSICSLYQFPNPYNQEK		SCG2	1469/334890	1.000	16877	0.840	-0.031868	-0.55	0.1667	0	18	-0.7444	0	2	0	0
HIP_16878	SHLVEALYL	EKSGEATDG	SHLVEALYLEKSGEATDG		CMGA	6304/247050	0.998	16878	0.840	0.235790	-0.3167	0.1667	-2	18	-0.35	-3	1	0	0
HIP_16879	GERGFFYTP	EESEESNVS	GERGFFYTPEESEESNVS		SCG1	6068/367830	0.999	16879	0.840	-0.053381	-1.9	0.3333	-4	18	-1.25	-4	3	0	0
HIP_16880	GERGFFYTP	RAYFMSDTR	GERGFFYTPRAYFMSDTR		SCG1	9304/367830	0.998	16880	0.840	-0.033894	-1.2667	0.1667	1	18	-0.8667	1	5	0	0
HIP_16881	LVCGERGFF	SQEESEEG	LVCGERGFFSQEESEEG		SCG1	3079/367830	1.000	16881	0.840	0.071039	-0.4333	0.3333	-4	17	-0.7176	-4	2	1	0
HIP_16882	VCGERGFFY	EEEEEMAV	VCGERGFFYEEEEEMAV		CMGA	2921/247050	0.999	16882	0.840	0.119434	-1.0333	0	-5	17	-0.4353	-5	3	1	3
HIP_16883	GSHLVEALY	WKELDRNY	GSHLVEALYWKELDRNY		SCG1	15111/367830	0.995	16883	0.840	0.104126	-0.6667	0	0	17	-0.7588	-1	2	1	0
HIP_16884	QHLCGSHLV	AAWIPHVEN	QHLCGSHLVAAWIPHVEN		SCG2	7280/334890	0.999	16884	0.840	-0.012218	1.25	0	2	18	0.1556	-1	1	0	0
HIP_16885	HLVEALYLV	MDEEQKLYT	HLVEALYLVMDEEQKLYT		SCG2	2857/334890	1.000	16885	0.840	0.088879	0.2667	0	-2	18	-0.0333	-3	1	0	0
HIP_16886	AGSLQPLAL	TNEIVEEQY	AGSLQPLALTNEIVEEQY		SCG2	135/334890	1.000	16886	0.840	0.164155	0.2833	0	-3	18	-0.1167	-3	0	0	0
HIP_16887	SLQPLALEG	EQEHSQQKE	SLQPLALEGEQEHSQQKE		CMGA	6164/247050	0.998	16887	0.840	-0.102334	-1.7667	0.1667	-2	18	-1.4167	-3	0	0	0
HIP_16888	GSHLVEALY	RGEDSSEEK	GSHLVEALYRGEDSSEEK		SCG1	4280/367830	0.999	16888	0.840	0.193252	-0.6833	0.1667	-2	18	-1.1111	-3	1	0	0
HIP_16889	GSHLVEALY	DSEEPVSTH	GSHLVEALYDSEEPVSTH		SCG1	1735/367830	1.000	16889	0.840	0.152489	-0.5833	0.1667	-2	18	-0.5	-4	1	0	0
HIP_16890	CGERGFFY	PNGSVEPE	CGERGFFYPNGSVEPE		SCG2	1647/334890	1.000	16890	0.840	0.182667	-0.2	0.3333	-2	16	-0.7938	-2	3	0	0
HIP_16891	TSICSLYQL	EDKRNYPSL	TSICSLYQLEDKRNYPSL		SCG1	22147/367830	0.986	16891	0.840	0.196135	-1.9833	0	0	18	-0.6278	0	1	0	0
HIP_16892	GSHLVEALY	QKGERGEDS	GSHLVEALYQKGERGEDS		SCG1	6388/367830	0.999	16892	0.840	0.200053	-0.5833	0.1667	-1	18	-1.0889	-2	1	0	0
HIP_16893	GPGAGSSLQP	EEEEEATNT	GPGAGSSLQPEEEEEATNT		CMGA	1578/247050	1.000	16893	0.840	-0.051289	-1.9667	0.1667	-5	18	-1.3167	-5	0	0	3
HIP_16894	LVEALYLVC	MQFPPMYEE	LVEALYLVCMQFPPMYEE		SCG2	4166/334890	1.000	16894	0.840	0.031940	1.95	0	-3	18	0.6056	-3	2	0	0
HIP_16895	HLCGSHLVE	MAYENLNDK	HLCGSHLVEMAYENLNDK		SCG2	7406/334890	0.999	16895	0.840	-0.004569	1.15	0	0	18	-0.4722	-2	1	0	0
HIP_16896	VEALYLVC	SYFPNPYNQ	VEALYLVCSYFPNPYNQ		SCG2	9939/334890	0.999	16896	0.840	0.099522	1.8667	0.1667	-1	17	0.0706	-1	3	1	0
HIP_16897	SHLVEALYL	KSGELEQEE	SHLVEALYLKSGELEQEE		CMGA	2197/247050	0.999	16897	0.840	0.216092	0.2	0.3333	-3	18	-0.5667	-4	1	0	0
HIP_16898	ERGFFYTPK	SEAHESSSR	ERGFFYTPKSEAHESSSR		SCG1	11472/367830	0.997	16898	0.840	-0.058540	-1.45	0.3333	1	18	-1.4667	0	2	0	1
HIP_16899	HLVEALYL	SGYPKTPG	HLVEALYLSGYPKTPG		SCG2	5366/334890	0.999	16899	0.840	0.222061	0.6333	0.3333	1	16	-0.0813	0	2	0	0
HIP_16900	AGSLQPLAL	TVLTEDEKK	AGSLQPLALTVLTEDEKK		SCG1	5925/367830	0.999	16900	0.840	0.038745	2.7833	0	-1	18	-0.1667	-1	0	0	0

HIP_17364	HLCGSHLV	EEARFQDKQ	HLCGSHLVEEARFQDKQ		SCG1	12211/367830	0.997	17364	0.840	0.124636	-0.0667	0	1	17	-0.8588	-1	1	1	0	0.000000	-3.3333	-2	0.1	1.750000	0.3333	1.4167	0	0	0	0	6.6667	-0.0944	-3.3333	1	0	0	0	0.3718	1.3086	0.6745	0	0.1382	0.4549	-0.0248	0.01	-0.3333
HIP_17365	SLQPLALEG	ERGFFYTPK	SLQPLALEGERGFFYTPK		INSB	204/4968	0.971	17365	0.840	-0.004542	-1.4167	0.3333	0	18	-0.3833	0	1	0	0	0.000000	-2.7667	1	0.05	1.650000	0.3333	1.3167	0	2	0	1	-2.7667	-1.8653	-2.7667	0	0	0	1	0.5385	1.0569	0.9812	-0.3333	0.2899	0.9627	-0.7628	0.0025	-0.1383
HIP_17366	SLYQLENYC	QNGQNGERA	SLYQLENYCQNGQNGERA		SCG2	1332/334890	1.000	17366	0.840	0.113143	-1.6167	0.1667	-1	18	-1.3778	-1	1	0	0	0.000000	-1.7	0	0.1333	0.950000	0.1111	0.8389	0	1	0	1	0	-1.3562	-1.7	0	0	0	0	0.5603	0.8322	1.2356	0	0.3139	1.5268	-0.9057	0.0178	-0.2267
HIP_17367	HLVEALYL	QSAPKENK	HLVEALYLQSAPKENK		SCG2	11566/334890	0.999	17367	0.840	0.235245	0.6333	0.1667	1	16	-0.5938	0	1	0	0	0.000000	-2.9333	0	0.1167	0.000000	0.1111	-0.1111	0	0	0	0	0	-0.0704	-2.9333	1	0	0	0	0.3718	1.1636	0.7912	0.5	0.1382	0.6259	0.2355	0.0136	-0.3422
HIP_17368	AGSLQPLAL	EEDIQGPTK	AGSLQPLALEEDIQGPTK		SCG1	4658/367830	0.999	17368	0.840	0.133872	-0.1833	0	-2	18	-0.4111	-2	0	0	0	0.000000	-6.6333	-3	0.15	1.575000	0.3333	1.2417	0	0	0	0	19.9	-0.2276	0	1	0	0	0	0.3718	1.3486	0.6745	0	0.1382	0.4549	-0.0682	0.0225	-0.995
HIP_17369	GSHLVEALY	AAWIPHVEN	GSHLVEALYAAWIPHVEN		SCG2	3747/334890	1.000	17369	0.840	-0.047185	1.1667	0	0	18	0.2222	-2	2	0	0	0.000000	-0.5333	0	0	3.575000	0.1111	3.4639	0	0	0	0	0	4.0412	-1.0667	0	1	0	0	0.2769	1.2822	0.7156	0.8333	0.0767	0.5121	0.3231	0	0
HIP_17370	CCTSICSLY	VALNHLKAT	CCTSICSLYVALNHLKAT		IAPP	409/45018	0.999	17370	0.840	0.004140	1.9167	0.1667	2	18	0.9056	1	1	0	0	0.000000	2.7	0	-0.0667	3.125000	0	3.125	0	0	0	0	0	5.9896	2.7	0	1	0	0	0.3051	1.1553	0.7112	0.6667	0.0931	0.5057	0.5848	0.0044	-0.18
HIP_17371	LVEALYLVC	PVSKRFPVG	LVEALYLVCPVSKRFPVG		SCG2	2315/334890	1.000	17371	0.840	0.165652	2.05	0.3333	1	18	0.9833	1	1	0	0	0.000000	-2.9	0	0.1333	2.000000	0.1111	1.8889	1	0	1	0	0	3.8722	-2.9	0	0	0	0	0.3885	0.9686	0.8545	0.5	0.1509	0.7301	0.7963	0.0178	-0.3867
HIP_17372	GERGFFYT	EEPVSTHQE	GERGFFYTEEPVSTHQE		SCG1	7367/367830	0.999	17372	0.840	0.097626	-1.3	0.1667	-2	17	-1.2529	-3	3	1	0	0.000000	-3.1333	-2	0.1167	0.700000	0.2222	0.4778	1	0	1	0	6.2667	-0.6211	-9.4	0	1	0	0	0.5218	1.1069	0.9112	-0.1667	0.2723	0.8302	-0.6783	0.0136	-0.3656
HIP_17373	CGERGFFYT	EEERGLEP	CGERGFFYTEEERGLEP		SCG1	10294/367830	0.998	17373	0.840	0.089268	-1.6167	0	-3	17	-1.1412	-3	3	1	1	0.000000	-3.7667	-3	0.1	0.000000	0.4444	-0.4444	0	0	0	0	11.3	0.7185	-11.3	0	1	0	0	0.5051	1.2636	0.7812	-0.3333	0.2552	0.6102	-0.8166	0.01	-0.3767
HIP_17374	HLVEALYLV	EVKTRHSE	HLVEALYLVEVKTRHSE		SCG1	16079/367830	0.994	17374	0.840	0.190681	0.5833	0	1	17	-0.1353	-1	1	1	0	0.000000	-3.3	0	0.1	1.400000	0.2222	1.1778	0	0	0	0	0	0.687	-3.3	1	0	0	0	0.3436	1.1622	0.6756	0.1667	0.1181	0.4565	0.2004	0.01	-0.33
HIP_17375	SLYQLENYC	YETQQWPER	SLYQLENYCYETQQWPER		SCG2	11565/334890	0.999	17375	0.840	-0.037472	-1.3	0	-2	18	-1.4889	-2	2	0	0	0.000000	-1.0667	-1	0.05	0.950000	0.2222	0.7278	0	0	0	0	1.0667	-0.9461	-2.1333	0	0	0	0	0.5154	1.0124	0.9968	-0.1667	0.2656	0.9936	-0.67	0.0025	-0.0533
HIP_17376	ERGFFYTPK	SMKLSFRAR	ERGFFYTPKSMKLSFRAR		CMGA	12284/247050	0.992	17376	0.840	-0.029040	-1.5	0.3333	4	18	-0.8056	4	2	0	0	0.000000	1.1333	0	-0.05	1.650000	0.2222	1.4278	1	0	1	0	0	-2.1417	2.2667	0	0	1	0	0.4936	0.9072	1.0156	-0.1667	0.2436	1.0315	-0.7404	0.0025	-0.0567
HIP_17377	QHLCGSHLV	WSLYPSDSQ	QHLCGSHLVWSLYPSDSQ		SCG1	6194/367830	0.999	17377	0.840	0.066693	1.15	0.1667	1	18	-0.3444	-1	2	0	0	1.000000	-0.9	0	0.0667	0.950000	0	0.95	0	0	0	0	0	1.0925	-1.8	1	0	0	0	0.3436	1.0838	0.7523	0.5	0.1181	0.566	0.3951	0.0044	-0.06
HIP_17378	YLVCGERGF	DRNYLNYGE	YLVCGERGFDRNYLNYGE		SCG1	14629/367830	0.995	17378	0.840	0.042216	-2.2667	0.1667	-1	18	-0.8056	-1	2	0	0	0.000000	-3.1333	-1	0.0167	0.000000	0.4444	-0.4444	0	1	0	1	3.1333	1.0074	-6.2667	1	0	0	0	0.4654	0.9058	1.0635	-0.3333	0.2166	1.131	-1.0549	0.0003	-0.0522
HIP_17379	AGSLQPLAL	QLLQKEPDL	AGSLQPLALQLLQKEPDL		SCG2	6981/334890	0.999	17379	0.840	0.050244	2.25	0	-1	18	0.0333	-1	0	0	0	0.000000	-1.7667	0	0.05	0.450000	0	0.45	0	0	0	0	0	1.0125	0	1	0	0	0	0.2667	1.2933	0.6067	0.8333	0.0711	0.368	0.6	0.0025	-0.0883
HIP_17380	LCGSHLVEA	QKFPSPEMI	LCGSHLVEAQKFPSPEMI		SCG2	22736/334890	0.995	17380	0.840	0.107594	-0.35	0	0	18	0.1389	-1	1	0	0	0.000000	-2.3667	2	0.05	0.000000	0.2222	-0.2222	0	0	0	0	-4.7333	0.0778	-2.3667	1	0	0	0	0.3718	1.2253	0.7312	0.1667	0.1382	0.5346	-0.1301	0.0025	-0.1183
HIP_17381	CGERGFFY	INSNQVKR	CGERGFFYINSNQVKR		SCG2	8785/334890	0.999	17381	0.840	0.153126	0.75	0.1667	2	16	-0.8125	2	3	0	0	0.000000	-1.3667	0	0.1167	1.825000	0.1111	1.7139	0	0	0	0	0	1.2854	-4.1	0	1	0	0	0.3718	0.9953	0.8895	0.5	0.1382	0.7912	0.2788	0.0136	-0.1594
HIP_17382	CGERGFFY	RAGTEALP	CGERGFFYRAGTEALP		SCG2	9328/334890	0.999	17382	0.840	0.179519	0.2	0.1667	0	16	-0.3313	0	3	0	0	0.000000	-2.4667	1	0.1333	0.700000	0.2222	0.4778	0	1	0	1	-2.4667	0.0956	-7.4	0	1	0	0	0.3603	1.0655	0.8623	0.3333	0.1298	0.7436	0.0721	0.0178	-0.3289
HIP_17383	LVEALYLVC	EEGENYQKG	LVEALYLVCEEGENYQKG		SCG1	590/367830	1.000	17383	0.840	0.163716	0.5167	0.1667	-3	18	-0.2333	-3	1	0	0	0.000000	-5.9667	-2	0.1833	2.000000	0.3333	1.6667	0	1	0	1	11.9333	0.8611	-5.9667	0	0	0	0	0.4551	1.1503	0.8145	0	0.2071	0.6634	0.2351	0.0336	-1.0939
HIP_17384	VCGERGFFY	EYLNQEKA	VCGERGFFYEYLNQEKA		SCG2	12254/334890	0.998	17384	0.840	0.064910	0.55	0	-1	17	-0.6706	-1	4	1	0	0.000000	-1.7667	-1	0.05	0.700000	0.2222	0.4778	0	0	0	0	1.7667	0.2628	-7.0667	0	1	0	0	0.3436	1.2088	0.709	0.3333	0.1181	0.5026	0.189	0.0025	-0.0883
HIP_17385	AGSLQPLAL	QAKREEEEE	AGSLQPLALQAKREEEEE		CMGA	7086/247050	0.997	17385	0.840	0.137220	0.6333	0	-3	18	-1.05	-3	0	0	3	0.000000	-5	1	0.1	0.450000	0.2222	0.2278	0	0	0	0	-5	0.1443	0	1	0	0	0	0.3051	1.2719	0.7012	0.5	0.0931	0.4916	0.1932	0.01	-0.5
HIP_17386	GSHLVEALY	EPEYGEEI	GSHLVEALYEPEYGEEI		SCG1	1607/367830	1.000	17386	0.840	0.129701	-0.7167	0.1667	-4	17	-0.4941	-5	2	1	0	0.000000	-4.3	-2	0.1667	2.000000	0.3333	1.6667	1	0	1	0	8.6	-1.1944	-8.6	0	1	0	0	0.4551	1.2269	0.8445	0	0.2071	0.7132	-0.3262	0.0278	-0.7167
HIP_17387	HLVEALYLV	VQKFPSPEM	HLVEALYLVVQKFPSPEM		SCG2	10190/334890	0.999	17387	0.840	0.105231	0.5833	0	0	18	0.4222	-1	2	0	0	1.000000	-3.3	1	0.1	3.150000	0.1111	3.0389	0	0	0	0	-3.3	1.7727	-6.6	1	0	0	0	0.3436	1.0955	0.7156	0.3333	0.1181	0.5121	0.2004	0.01	-0.33
HIP_17388	CSLYQLENY	SNQVKRVPG	CSLYQLENYSNQVKRVPG		SCG2	16819/334890	0.997	17388	0.840	-0.034134	-2.6833	0.1667	1	18	-0.7556	1	1	0	0	0.000000	0.1667	1	0.0667	1.050000	0.1111	0.9389	0	0	0	0	0.1667	-2.5194	0.1667	0	1	0	0	0.5718	0.9636	1.1578	-0.1667	0.3269	1.3405	-1.5343	0.0044	0.0111
HIP_17389	AGSLQPLAL	EEEEEATNT	AGSLQPLALEEEEEATNT		CMGA	1092/247050	1.000	17389	0.840	0.121661	-0.1833	0	-5	18	-0.6611	-5	0	0	3	0.000000	-6.6333	-3	0.15	0.450000	0.4444	0.0056	0	0	0	0	19.9	-0.001	0	1	0	0	0	0.4	1.43	0.6467	0	0.16	0.4182	-0.0733	0.0225	-0.995
HIP_17390	LVCGERGFF	LGEKRDHHS	LVCGERGFFLGEKRDHHS		SCG1	2461/367830	1.000	17390	0.840	0.034583	0.85	0.3333	2	18	-0.6611	0	2	0	0	1.000000	-1.7667	-1	0.05	1.650000	0.4444	1.2056	0	2	0	1	1.7667	1.0247	-3.5333	1	0	0	0	0.4333	1.035	0.9067	0.3333	0.1878	0.822	0.3683	0.0025	-0.0883
HIP_17391	AGSLQPLAL	ADTEKWAE	AGSLQPLALADTEKWAE		SCG1	3360/367830	1.000	17391	0.840	0.137851	1.1667	0	-2	17	-0.2176	-2	0	1	0	1.000000	-3.9333	-1	0.1	0.900000	0.2222	0.6778	0	0	0	0	3.9333	0.7907	0	1	0	0	0	0.3051	1.2253	0.6978	0.5	0.0931	0.487	0.356	0.01	-0.3933
HIP_17392	YLVCGERGF	NPFKRTNEI	YLVCGERGFNPFKRTNEI		SCG2	3777/334890	1.000	17392	0.840	-0.024206	-0.7333	0.3333	1	18	-0.5944	1	2	0	0	0.000000	-0.0667	-1	-0.0167	0.000000	0.3333	-0.3333	1	1	1	1	0.0667	0.2444	-0.1333	1	0	0	0	0.4718	0.8603	1.1145	0.1667	0.2226	1.2421	-0.346	0.0003	0.0011
HIP_17393	LVEALYLVC	EISRNLQIP	LVEALYLVCEISRNLQIP		SCG2	2477/334890	1.000	17393	0.840	0.143437	1.7833	0.1667	-1	18	0.8167	-1	1	0	0	0.000000	-3.4333	-1	0.1167	2.000000	0.2222	1.7778	0	0	0	0	3.4333	3.1704	-3.4333	0	0	0	0	0.3718	1.1286	0.7195	0.3333	0.1382	0.5177	0.663	0.0136	-0.4006
HIP_17394	SHLVEALYL	QGVSVPLQQ	SHLVEALYLQGVSVPLQQ		SCG2	728/334890	1.000	17394	0.840	0.202032	1.1	0.1667	0	18	0.4167	-1	1	0	0	0.000000	-2	0	0.1333	0.000000	0.1111	-0.1111	0	1	0	1	0	-0.1222	-2	1	0	0	0	0.3885	1.0486	0.8145	0.5	0.1509	0.6634	0.4273	0.0178	-0.2667
HIP_17395	GSHLVEALY	NKGDTEVMK	GSHLVEALYNKGDTEVMK		CMGA	4797/247050	0.998	17395	0.840	0.207663	-0.5833	0.1667	0	18	-0.4944	-1	1	0	0	0.000000	-4.0333	1	0.1833	2.000000	0.3333	1.6667	0	1	0	1	-4.0333	-0.9722	-4.0333	0	1	0	0	0.4269	0.9938	1.0056	0.1667	0.1823	1.0113	-0.249	0.0336	-0.7394
HIP_17396	VEALYLVC	NQEKAEKGR	VEALYLVCNQEKAEKGR		SCG2	9983/334890	0.999	17396	0.840	0.206968	0	0	0	17	-0.5529	0	1	1	0	0.000000	-7	-1	0.15	1.075000	0.2222	0.8528	0	0	0	0	7	0	-7	0	0	0	0	0.4385	1.1119	0.8645	0.1667	0.1922	0.7473	0	0.0225	-1.05
HIP_17397	SHLVEALYL	NGDESHLPE	SHLVEALYLNGDESHLPE		SCG2	983/334890	1.000	17397	0.840	0.210989	-0.1833	0.1667	-2	18	-0.4222	-4	1	0	0	0.000000	-4.5667	-1	0.1833	0.000000	0.3333	-0.3333	0	1	0	1	4.5667	0.0611	-4.5667	1	0	0	0	0.4269	0.9688	0.979	0.1667	0.1823	0.9584	-0.0783	0.0336	-0.8372
HIP_17398	TSICSLYQL	ERELDLPVD	TSICSLYQLERELDLPVD		SCG2	10706/334890	0.999	17398	0.840	0.101562	-2.0833	0	-3	18	-0.0444	-3	1	0	0	0.000000	-3.5	-1	0.1	0.950000	0.3333	0.6167	0	0	0	0	3.5	-1.2847	-3.5	1	0	0	0	0.4769	1.2455	0.7956	-0.3333	0.2275	0.633	-0.9936	0.01	-0.35
HIP_17399	EQCCTSICS	EWEDSKRWS	EQCCTSICSEWEDSKRWS		CMGA	5751/247050	0.998	17399	0.840	0.015737	-0.2833	0.1667	-2	18	-1.0889	-2	1	0	0	0.000000	-4.7	-2	0.0333	0.625000	0.3333	0.2917	0	0	0	0	9.4	-0.0826	-4.7	0	1	0	0	0.4769	1.1572	0.8273	0	0.2275	0.6844	-0.1351	0.0011	-0.1567
HIP_17400	SHLVEALYL	YLNYGEEGA	SHLVEALYLYLNYGEEGA		SCG1	9476/367830	0.998	17400	0.840	0.066983	0.8833	0	-2	18	0.0167	-3	3	0	0	0.000000	-2.4333	0	0.05	0.000000	0.1111	-0.1111	0	0	0	0	0	-0.0981	-7.3	1	0	0	0	0.3436	1.1022	0.8123	0.5	0.1181	0.6598	0.3035	0.0025	-0.1217
HIP_17401	SLYQLENYC	GPQLRRGWR	SLYQLENYCGPQLRRGWR		CMGA	2255/247050	0.999	17401	0.840	0.106905	-1.3	0.3333	2	18	-1.1278	2	1	0	0	0.000000	-1.0667	0	0.15	1.900000	0.1111	1.7889	1	1	1	1	0	-2.3256	-1.0667	0	0	0	0	0.5769	0.8155	1.229	0	0.3328	1.5104	-0.75	0.0225	-0.16
HIP_17402	ICSLYQLEN	EYRAPRPQS	ICSLYQLENEYRAPRPQS		SCG1	30604/367830	0.963	17402	0.840	0.035731	-2.0833	0	0	18	-0.9722	0	2	0	0	0.000000	-2.0333	1	0	0.450000	0.3333	0.1167	0	0	0	0	-2.0333	-0.2431	-4.0667	0	1	0	0	0.4769	1.1722	0.8923	-0.3333	0.2275	0.7962	-0.9936	0	0
HIP_17403	EDLQVGQVE	EEEEMAVVP	EDLQVGQVEEEEEMAVVP		CMGA	3752/247050	0.999	17403	0.840	-0.095402	-2.2167	0	-7	18	-0.5111	-7	0	0	3	0.000000	-2.5667	-2	0.05	2.100000	0.5556	1.5444	0	0	0	0	5.1333	-3.4235	0	0	0	1	0	0.5333	1.3683	0.74	-0.5	0.2844	0.5476	-1.1822	0.0025	-0.1283
HIP_17404	CGSHLVEAL	EYLNQEKAE	CGSHLVEALEYLNQEKAE		SCG2	9257/334890	0.999	17404	0.840	-0.063926	0.1833	0	-2	18	-0.4944	-3	1	0	0	0.000000	-1.0333	0	0	1.400000	0.2222	1.1778	0	0	0	0	0	0.2159	-1.0333	1	0	0	0	0.3718	1.3486	0.6745	0.1667	0.1382	0.4549	0.0682	0	0
HIP_17405	VCGERGFF	APGKWQQQG	VCGERGFFAPGKWQQQG		SCG1	655/367830	1.000	17405	0.840	0.066099	0.8333	0.5	1	17	-0.7294	1	3	1	1	1.000000	-1.8	0	0.0667	0.700000	0.2222	0.4778	1	2	1	1	0	0.3981	-5.4	1	0	0	0	0.45	0.9033	1.0633	0.5	0.2025	1.1307	0.375	0.0044	-0.12
HIP_17406	GERGFFYTP	DQTVLTEDE	GERGFFYTPDQTVLTEDE		SCG1	4086/367830	0.999	17406	0.840	-0.102035	-1.8833	0.1667	-4	18	-0.9833	-4	3	0	0	0.000000	-1.3667	-1	0.0333	1.750000	0.1111	1.6389	1	0	1	0	1.3667	-3.0866	-4.1	0	0	0	1	0.5603	0.8972	1.039	-0.1667	0.3139	1.0795	-1.0551	0.0011	-0.0456
HIP_17407	LVEALYLVC	QDKQYSSHH	LVEALYLVCQDKQYSSHH		SCG1	14348/367830	0.995	17407	0.840	0.212261	-0.0667	0	1	18	-0.2444	-1	1	0	0	0.000000	-7.1333	0	0.15	2.000000	0.2222	1.7778	0	0	0	0	0	-0.1185	-7.1333	0	0	0	0	0.3821	1.0891	0.7835	0	0.146	0.6138	-0.0255	0.0225	-1.07
HIP_17408	AGSLQPLAL	SEAHESSS	AGSLQPLALSEAHESSS		SCG1	2884/367830	1.000	17408	0.840	0.170855	1.15	0.1667	-1	17	-0.1706	-2	0	1	1	0.000000	-3.9667	-1	0.1167	0.450000	0.1111	0.3389	0	0	0	0	3.9667	0.3897	0	1	0	0	0	0.3333	1.3017	0.71	0.5	0.1111	0.5041	0.3833	0.0136	-0.4628
HIP_17409	VCGERGFF	PEDLEWERY	VCGERGFFPEDLEWERY		SCG1	3322/367830	1.000	17409	0.840	0.062909	-0.5667	0.3333	-3	17	-0.8824	-3	2	1	0	0.000000	-4.6	-2	0.0833	0.700000	0.4444	0.2556	1	1	1	1	9.2	-0.1448	-9.2	1	0	0	0	0.4718	1.0003	0.9778	0	0.2226	0.9561	-0.2674	0.0069	-0.3833
HIP_17410	LYLVCGERG	EKGREHIAK	LYLVCGERGEKGREHIAK		SCG2	18595/334890	0.997	17410	0.840	-0.032471	-2.7	0.3333	2	18	-0.6889	1	0	0	0	0.000000	0.2	0	0	0.625000	0.5556	0.0694	0	2	0	1	0	-0.1875	0	0	0	0	1	0.5769	1.0105	1.049	-0.6667	0.3328	1.1003	-1.5577	0	0
HIP_17411	GERGFFYT	QGDLQDTKE	GERGFFYTQGDLQDTKE		SCG1	5642/367830	0.999	17411	0.840	0.133414	-1.1	0.1667	-2	17	-1.4050	-2	3	1	0	0.000000	-2.7333	-1	0.1333	0.700000	0.1111	0.5889	0	1	0	1	2.7333	-0.6478	-8.2	0	1	0	0	0.4936	0.9472	0.9756	0	0.2436	0.9519	-0.5429	0.0178	-0.3644
[illegible]																																														

HIP_17875	VCGERGFFY	PRLPYGAG	VCGERGFFYPRLPYGAG		SCG2	13247/334890	0.998	17875	0.839	0.173138	0.3333	0.1667	1	17	-0.1176	1	3	1	0	0.000000	-2.2	1	0.1167	0.700000	0.2222	0.4778	1	0	1	0	-2.2	0.1593	-6.6	0	1	0	0	0.3603	1.0522	0.839	0.3333	0.1298	0.7039	0.1201	0.0136	-0.2567
HIP_17876	GERGFFYTP	TLSKPSPMP	GERGFFYTPTLSKPSPMP		CMGA	3417/247050	0.999	17876	0.839	-0.102554	-0.2167	0.3333	1	18	-0.6722	1	3	0	0	0.000000	1.9667	0	0	0.700000	0.1111	0.5889	1	0	1	0	0	-0.1276	5.9	0	0	0	1	0.5218	0.8919	1.0078	0.1667	0.2723	1.0157	-0.1131	0	0
HIP_17877	CGERGFFYT	DQLLHYRK	CGERGFFYTDQLLHYRK		SCG1	20611/367830	0.989	17877	0.839	0.076019	-0.4	0	2	17	-0.8824	1	3	1	0	0.000000	-1.3333	-1	0.05	0.950000	0.1111	0.8389	0	0	0	0	1.3333	-0.3356	-4	0	1	0	0	0.4103	1.0805	0.809	0.1667	0.1683	0.6544	-0.1641	0.0025	-0.0667
HIP_17878	VCGERGFF	QQAHKEESS	VCGERGFFQQAHKEESS		SCG2	10768/334890	0.999	17878	0.839	0.048615	0	0.1667	0	17	-1.0235	-1	2	1	0	0.000000	-3.4667	0	0.0167	0.700000	0.2222	0.4778	0	1	0	1	0	0	-6.9333	1	0	0	0	0.4167	1.095	0.8867	0.5	0.1736	0.7862	0	0.0003	-0.0578
HIP_17879	HLVEALYLV	EPEYGEEI	HLVEALYLVEPEYGEEI		SCG1	2349/367830	1.000	17879	0.839	0.141598	-0.3167	0.1667	-4	17	0.0471	-5	2	1	0	0.000000	-5.1	-2	0.1667	1.400000	0.2222	1.1778	1	0	1	0	10.2	-0.373	-10.2	1	0	0	0	0.4551	1.1619	0.8178	0	0.2071	0.6688	-0.1441	0.0278	-0.85
HIP_17880	GSHLVEALY	SEEERGLEP	GSHLVEALYSEEERGLEP		SCG1	1533/367830	1.000	17880	0.839	0.146194	-0.5833	0.1667	-3	18	-0.7278	-4	1	0	1	0.000000	-4.0333	-2	0.1667	2.000000	0.4444	1.5556	0	0	0	0	8.0667	-0.9074	-4.0333	0	1	0	0	0.4385	1.2653	0.7912	0	0.1922	0.6259	-0.2558	0.0278	-0.6722
HIP_17881	GSHLVEALY	NRQMAYENL	GSHLVEALYNRQMAYENL		SCG2	13481/334890	0.998	17881	0.839	0.175046	-1.2	0	0	18	-0.4389	-1	1	0	0	0.000000	-5.2667	1	0.15	2.475000	0.2222	2.2528	0	0	0	0	-5.2667	-2.7033	-5.2667	0	1	0	0	0.4103	1.0955	0.919	0.1667	0.1683	0.8445	-0.4923	0.0225	-0.79
HIP_17882	GERGFFYTP	NPFKRTNEI	GERGFFYTPNPFKRTNEI		SCG2	4596/334890	1.000	17882	0.839	-0.089404	-0.9833	0.3333	1	18	-1.15	1	4	0	0	0.000000	0.4333	0	0	0.700000	0.1111	0.5889	2	0	1	0	0	-0.5791	1.7333	0	0	0	1	0.5385	0.8103	1.1778	0.1667	0.2899	1.3873	-0.5295	0	0
HIP_17883	LVCGERGFF	QVEKRKCN	LVCGERGFFQVEKRKCN		IAPP	1945/45018	0.991	17883	0.839	0.074659	0.4	0.1667	2	17	-0.5176	2	2	1	0	0.000000	-2.6667	-1	0.0167	0.700000	0.4444	0.2556	0	1	0	1	2.6667	0.1022	-5.3333	1	0	0	0	0.4167	1.0967	0.82	0.3333	0.1736	0.6724	0.1667	0.0003	-0.0444
HIP_17884	CGERGFFY	LSKSGYPK	CGERGFFYLSKSGYPK		SCG2	14512/334890	0.998	17884	0.839	0.170153	0.5667	0.1667	2	16	-0.6813	2	3	0	0	0.000000	-1.7333	1	0.1167	1.650000	0.2222	1.4278	0	0	0	0	-1.7333	0.8091	-5.2	0	1	0	0	0.3436	1.0905	0.7856	0.3333	0.1181	0.6172	0.1947	0.0136	-0.2022
HIP_17885	CGERGFFYT	GEEAVPEEE	CGERGFFYTGEEAVPEEE		CMGA	397/247050	1.000	17885	0.839	0.093855	-1.1	0.1667	-5	18	-0.9	-5	3	0	1	0.000000	-2.7333	-2	0.1333	0.450000	0.2222	0.2278	0	1	0	1	5.4667	-0.2506	-8.2	0	1	0	0	0.5218	1.0953	0.9078	-0.1667	0.2723	0.8241	-0.574	0.0178	-0.3644
HIP_17886	AGSLQPLAL	DTEVMKCIV	AGSLQPLALDTEVMKCIV		CMGA	2618/247050	0.999	17886	0.839	0.152716	0.2833	0	-1	18	0.8	-1	0	0	0	0.000000	-5.7	-2	0.15	1.500000	0.2222	1.2778	0	0	0	0	11.4	0.362	0	1	0	0	0	0.3718	1.2353	0.7112	0.1667	0.1382	0.5057	0.1053	0.0225	-0.855
HIP_17887	GERGFFYTP	ATSEVDKRR	GERGFFYTPATSEVDKRR		SCG1	5540/367830	0.999	17887	0.839	-0.117976	-0.55	0.3333	1	18	-1.2333	1	3	0	0	0.000000	1.3	0	0	1.150000	0.1111	1.0389	1	0	1	0	0	-0.5714	3.9	0	0	0	1	0.5218	0.9169	1.0345	0.1667	0.2723	1.0702	-0.287	0	0
HIP_17888	GSHLVEALY	VEKRKCNTA	GSHLVEALYVEKRKCNTA		IAPP	2099/45018	0.990	17888	0.839	0.121286	0.1833	0	2	18	-0.3944	1	1	0	0	0.000000	-2.5	0	0.1	3.050000	0.4444	2.6056	0	0	0	0	0	0.4777	-2.5	0	1	0	0	0.3436	1.2272	0.7023	0.1667	0.1181	0.4932	0.063	0.01	-0.25
HIP_17889	CTSICSLYQ	NEPQSAPKE	CTSICSLYQNEPQSAPKE		SCG2	4422/334890	1.000	17889	0.839	0.133932	-1.6	0.1667	-1	18	-0.7722	-1	1	0	0	0.000000	-2.5333	-1	0.1167	0.625000	0.1111	0.5139	1	0	1	0	2.5333	-0.8222	-2.5333	0	1	0	0	0.5218	1.0303	1.0345	0	0.2723	1.0702	-0.8349	0.0136	-0.2956
HIP_17890	HLVEALYLV	EGNNQAPGE	HLVEALYLVEGNNQAPGE		CMGA	55/247050	1.000	17890	0.839	0.193731	-0.1167	0.1667	-2	18	-0.25	-3	1	0	0	0.000000	-4.7	-1	0.1833	1.400000	0.1111	1.2889	0	1	0	1	4.7	-0.1504	-4.7	1	0	0	0	0.4551	1.0103	0.9512	0.1667	0.2071	0.9047	-0.0531	0.0136	-0.8617
HIP_17891	GSHLVEALY	SDTLSKPSP	GSHLVEALYSDTLSKPSP		CMGA	2342/247050	0.999	17891	0.839	0.173598	-0.1167	0.1667	0	18	-0.3056	-1	1	0	0	0.000000	-3.1	-1	0.1667	2.950000	0.2222	2.7278	0	0	0	0	3.1	-0.3182	-3.1	0	1	0	0	0.4103	1.0705	0.8556	0.1667	0.1683	0.7321	-0.0479	0.0278	-0.5167
HIP_17892	CGERGFFYT	LENLAAMDL	CGERGFFYTLENLAAMDL		SCG1	5876/367830	0.999	17892	0.839	-0.028836	-0.4	0	-2	18	0.2056	-2	3	0	0	0.000000	-1.3333	-1	0.05	1.900000	0.1111	1.7889	0	0	0	0	1.3333	-0.7156	-4	0	1	0	0	0.4385	1.0886	0.8778	0.1667	0.1922	0.7706	-0.1754	0.0025	-0.0667
HIP_17893	CGERGFFY	LEYLNQEKA	CGERGFFYLEYLNQEKA		SCG2	13016/334890	0.998	17893	0.839	0.074516	0.55	0	-1	17	-0.6941	-1	4	1	0	0.000000	-1.7667	-1	0.05	0.000000	0.1111	-0.1111	0	0	0	0	1.7667	-0.0611	-7.0667	0	1	0	0	0.3436	1.2088	0.709	0.3333	0.1181	0.5026	0.189	0.0025	-0.0883
HIP_17894	SHLVEALYL	GGGHSRERA	SHLVEALYLGGGHSRERA		SCG1	314/367830	1.000	17894	0.839	0.220444	0.85	0.5	2	18	-0.4056	0	1	0	1	0.000000	-2.5	0	0.25	0.000000	0.1111	-0.1111	0	3	0	1	0	-0.0944	-2.5	1	0	0	0	0.4885	0.8536	1.0678	0.3333	0.2386	1.1402	0.4152	0.0625	-0.625
HIP_17895	SHLVEALYL	EFPDFYDS	SHLVEALYLEFPDFYDS		SCG1	2192/367830	1.000	17895	0.839	0.138111	0.6667	0.1667	-3	17	0	-4	2	1	0	0.000000	-2.8667	-1	0.1167	0.000000	0.3333	-0.3333	1	0	1	0	2.8667	-0.2222	-5.7333	1	0	0	0	0.3885	1.1503	0.7945	0.3333	0.1509	0.6312	0.259	0.0136	-0.3344
HIP_17896	VEALYLVCG	FQRNQLLQK	VEALYLVCGFQRNQLLQK		SCG2	15362/334890	0.998	17896	0.839	0.054861	0.1833	0.1667	1	18	0.1722	1	2	0	0	0.000000	-3.8333	1	0.0167	1.325000	0.1111	1.2139	0	1	0	1	-3.8333	0.2225	-7.6667	0	0	0	1	0.4269	0.9855	0.899	0.1667	0.1823	0.8082	0.0783	0.0003	-0.0639
HIP_17897	LVCGERGFF	GLFRGGKSG	LVCGERGFFGLFRGGKSG		CMGA	1192/247050	1.000	17897	0.839	-0.008710	1.9	0.3333	2	18	0.1722	2	3	0	0	0.000000	0.3333	0	0	0.700000	0.3333	0.3667	0	2	0	1	0	0.6967	1	1	0	0	0	0.3667	0.9833	0.8833	0.6667	0.1344	0.7803	0.6967	0	0
HIP_17898	SLYQLENYC	SRSNQLPKA	SLYQLENYCSRSNQLPKA		SCG2	12431/334890	0.998	17898	0.839	0.174180	-1.4	0.3333	1	18	-0.9333	1	1	0	0	0.000000	-1.2667	1	0.1333	0.950000	0.2222	0.7278	0	0	0	0	-1.2667	-1.0189	-1.2667	0	0	0	0	0.5154	0.8891	1.1135	-0.1667	0.2656	1.2398	-0.7215	0.0178	-0.1689
HIP_17899	HLVEALYL	EEPEYGEEI	HLVEALYLEEPEYGEEI		SCG1	1782/367830	1.000	17899	0.839	0.134401	-0.3833	0.1667	-5	17	-0.4059	-6	2	1	0	0.000000	-4.9667	-2	0.1667	1.400000	0.3333	1.0667	1	0	1	0	9.9333	-0.4089	-9.9333	1	0	0	0	0.4551	1.2019	0.8178	0	0.2071	0.6688	-0.1745	0.0278	-0.8278
HIP_17900	ERGFFYTPK	QSEESWDEE	ERGFFYTPKQSEESWDEE		SCG1	5470/367830	0.999	17900	0.839	-0.130501	-2.3333	0.3333	-4	18	-1.8778	-4	2	0	0	0.000000	-0.5333	-2	0	0.700000	0.3333	0.3667	1	0	1	0	1.0667	-0.8556	-1.0667	0	0	1	0	0.5885	0.9736	1.0512	-0.3333	0.3463	1.1049	-1.3731	0	0
HIP_17901	CGERGFFY	LENVQKFPS	CGERGFFYLENVQKFPS		SCG2	12937/334890	0.998	17901	0.839	0.152988	0.1833	0	0	17	-0.4706	0	3	1	0	0.000000	-2.5	-1	0.1	0.000000	0.1111	-0.1111	0	0	0	0	2.5	-0.0204	-7.5	0	1	0	0	0.3718	1.1503	0.8178	0.3333	0.1382	0.6688	0.0682	0.01	-0.25
HIP_17902	AGSLQPLAL	RHQNLLKEL	AGSLQPLALRHQNLLKEL		CMGA	17534/247050	0.980	17902	0.839	0.202043	-0.3	0	2	18	-0.1111	1	0	0	0	0.000000	-6.8667	1	0.1	0.450000	0.1111	0.3389	0	0	0	0	-6.8667	-0.1017	0	1	0	0	0	0.3436	1.2005	0.7423	0.1667	0.1181	0.551	-0.1031	0.01	-0.6867
HIP_17903	QHLCGSHLV	PDLFQNRML	QHLCGSHLVPDLFQNRML		SCG2	5427/334890	0.999	17903	0.839	0.163744	0.5833	0.1667	2	18	-0.0611	0	1	0	0	0.000000	-2.0333	-1	0.1167	1.650000	0.1111	1.5389	1	0	1	0	2.0333	0.8977	-2.0333	0	1	0	0	0.3603	1.0455	0.8056	0.1667	0.1298	0.6491	0.2101	0.0136	-0.2372
HIP_17904	AGSLQPLAL	EEAQDPEGK	AGSLQPLALEEAQDPEGK		CMGA	2501/247050	0.999	17904	0.839	0.087948	0.7	0	-3	18	-0.7167	-3	0	0	0	0.000000	-4.8667	-2	0.1	0.450000	0.2222	0.2278	0	0	0	0	9.7333	0.1594	0	1	0	0	0	0.3333	1.42	0.6333	0.3333	0.1111	0.4011	0.2333	0.01	-0.4867
HIP_17905	CGERGFFY	GRAGTEAL	CGERGFFYGRAGTEAL		SCG2	9499/334890	0.999	17905	0.839	0.179514	0.2	0.1667	0	16	-0.2563	0	3	0	0	0.000000	-2.4667	1	0.1333	0.700000	0.2222	0.4778	0	1	0	1	-2.4667	0.0956	-7.4	0	1	0	0	0.3603	1.0655	0.8623	0.3333	0.1298	0.7436	0.0721	0.0178	-0.3289
HIP_17906	HLVEALYL	ENQSSQAE	HLVEALYLENQSSQAE		CMGA	5082/247050	0.998	17906	0.839	0.206200	-0.7	0	-2	16	-0.4938	-3	1	0	0	0.000000	-5.6	-1	0.15	0.000000	0.2222	-0.2222	0	0	0	0	5.6	0.1556	-5.6	1	0	0	0	0.4385	1.1519	0.8645	0.1667	0.1922	0.7473	-0.3069	0.0225	-0.84
HIP_17907	SLYQLENYC	YPEEKKEEE	SLYQLENYCYPEEKKEEE		CMGA	1014/247050	1.000	17907	0.839	-0.046815	-1.45	0.1667	-4	18	-1.7778	-4	2	0	1	0.000000	-1.3667	-1	0.0667	0.950000	0.3333	0.6167	1	0	1	0	1.3667	-0.8942	-2.7333	0	0	0	0	0.5321	0.9691	1.0901	-0.1667	0.2831	1.1884	-0.7715	0.0044	-0.0911
HIP_17908	AGSLQPLAL	VNSMPVDNR	AGSLQPLALVNSMPVDNR		SCG1	2631/367830	1.000	17908	0.839	0.145333	1.55	0.1667	0	18	0.0889	0	0	0	0	1.000000	-3.1667	0	0.1167	1.975000	0	1.975	0	0	0	0	0	3.0612	0	1	0	0	0	0.3333	1.0967	0.82	0.6667	0.1111	0.6724	0.5167	0.0136	-0.3694
HIP_17909	AGSLQPLAL	PIESHQVEK	AGSLQPLALPIESHQVEK		IAPP	235/45018	0.999	17909	0.839	0.166658	1.4667	0.1667	0	18	-0.1444	-1	0	0	0	0.000000	-3.3333	-1	0.1167	0.450000	0.1111	0.3389	1	0	1	0	3.3333	0.497	0	1	0	0	0	0.35	1.2017	0.7317	0.5	0.1225	0.5353	0.5133	0.0136	-0.3889
HIP_17910	RGFFYTPKT	KWQQQGDLQ	RGFFYTPKTKWQQQGDLQ		SCG1	19600/367830	0.990	17910	0.839	0.045047	-2.4167	0.1667	2	18	-1.4667	2	2	0	1	0.000000	-0.7	0	-0.0667	0.000000	0.2222	-0.2222	1	0	1	0	0	0.537	-1.4	0	1	0	0	0.5321	0.9291	1.0301	-0.1667	0.2831	1.0612	-1.2858	0.0044	0.0467
HIP_17911	LVCGERGFF	DDVSKVIAY	LVCGERGFFDDVSKVIAY		SCG2	5650/334890	0.999	17911	0.839	0.025057	0.4	0.1667	-1	18	0.5	-1	2	0	0	0.000000	-2.6667	-2	0.0167	0.700000	0.4444	0.2556	0	1	0	1	5.3333	0.1022	-5.3333	1	0	0	0	0.3603	1.0005	0.8356	0.1667	0.1298	0.6983	0.1441	0.0003	-0.0444
HIP_17912	TSICSLYQL	WGYEKRNLA	TSICSLYQLWGYEKRNLA		SCG1	9331/367830	0.998	17912	0.839	0.026528	-0.6	0.1667	1	18	-0.2722	1	3	0	0	1.000000	-0.5333	0	0.0333	0.000000	0.1111	-0.1111	1	0	1	0	0	0.0667	-1.6	1	0	0	0	0.4654	0.9958	0.9501	0.3333	0.2166	0.9027	-0.2792	0.0011	-0.0178
HIP_17913	CGERGFFY	EKIESQTQE	CGERGFFYEKIESQTQE		SCG2	13811/334890	0.998	17913	0.839	0.141894	0.2333	0	-2	17	-1.2	-2	3	1	0	0.000000	-2.4	0	0.1	0.000000	0.3333	-0.3333	0	0	0	0	0	-0.0778	-7.2	0	1	0	0	0.3436	1.1722	0.704	0.1667	0.1181	0.4956	0.0802	0.01	-0.24
HIP_17914	VCGERGFF	NQVKRVPGQ	VCGERGFFNQVKRVPGQ		SCG2	13623/334890	0.998	17914	0.839	0.075907	0.4	0.1667	2	17	-0.5294	2	2	1	0	0.000000	-2.6667	0	0.0167	0.700000	0.3333	0.3667	0	1	0	1	0	0.1467	-5.3333	1	0	0	0	0.4167	0.9567	0.9567	0.5	0.1736	0.9152	0.1667	0.0003	-0.0444
HIP_17915	NQHLCGSHL	GENYQKGER	NQHLCGSHLGENYQKGER		SCG1	8390/367830	0.998	17915	0.839	0.153089	-1.2667	0.3333	2	18	-1.6111	0	1	0	0	0.000000	-2.4	-1	0.1167	0.625000	0.1111	0.5139	0	1	0	1	2.4	-0.6509	-2.4	1	0	0	0	0.5218	0.9669	1.0678	-0.1667	0.2723	1.1402	-0.6609	0.0136	-0.28
HIP_17916	SLQPLAL	SSNNFGA	SLQPLALSSNNFGA		IAPP	608/45018	0.998	17916	0.839	0.242162	0.7167	0.3333	0	14	0.2071	0	0	0	0	0.000000	-4.8333	0	0.1833	0.450000	0	0.45	0	0	0	0	0	0.3225	0	1	0	0	0	0.4	1.0533	0.9367	0.5	0.16	0.8773	0.2867	0.0136	-0.8861
HIP_17917	SLYQLENYC	QYWDEDLLM	SLYQLENYCQYWDEDLLM		SCG2	4354/334890	1.000	17917	0.839	-0.086850	-1.3333	0	-4	18	-0.5833	-4	3	0	0	0.000000	-1.1333	0	0	0.950000	0.2222	0.7278	0	0	0	0	0	-0.9704	-3.4	0	0	0	0	0.4872	0.9777	1.0279	0.1667	0.2373	1.0567	-0.6496	0	0
HIP_17918	SHLVEALYL	GSANRRPED	SHLVEALYLGSANRRPED		CMGA	186/247050	1.000	17918	0.839	0.210043	1.15	0.3333	0	18	-0.6611	-1	1	0	0	0.000000	-1.9	0	0.15	0.000000	0.1111	-0.1111	0	1	0	1	0	-0.1278	-1.9	1	0	0	0	0.3885	1.0619	0.8778	0.5	0.1509	0.7706	0.4467	0.0225	-0.285
HIP_17919	VCGERGFFY	GQGSSEDD	VCGERGFFYGQGSSEDD		SCG2	879/334890	1.000	17919	0.839	0.182545	0	0.3333	-3	17	-0.8353	-3	3	1	0	0.000000	-2.8667	0	0.2167	0.700000	0.1111	0.5889	0	2	0	1	0	0	-8.6	0	1	0	0	0.4718	0.9219	1.0145	0.3333	0.2226	1.0292	0	0.0469	-0.6211
HIP_17920	TSICSLYQL	GAGRSRSNQ	TSICSLYQLGAGRSRSNQ		SCG2	1449/334890	1.000	17920	0.839	0.115121	0	0.3333	2	18	-0.5056	2	1	0	0	0.000000	0.6667	0	0.1167	0.000000	0.1111	-0.1111	0	2	0	1	0	0	0.6667	1	0	0	0	0.4718	0.9803	1.0078	0.3333	0.2226	1.0157	0	0.0136	0.0778
HIP_17921	VCGERGFFY	VSQECFETL	VCGERGFFYVSQECFETL		CMGA	535/247050	1.000	17921	0.839	0.107822	0.7	0.1667	-2	18	0.1944	-2	3	0	0	0.000000	-1.4667	0	0.1167	1.750000	0.2222	1.5278	0	0	0	0	0	1.0694	-4.4	0	1	0	0	0.3718	1.0653	0.7978	0.5	0.1382	0.6365	0.2603	0.0136	-0.1711
HIP_17922	LVEALYLVC	QESGEETGS	LVEALYLVCQESGEETGS		SCG1	1928/367830	1.000	17922	0.839	0.169327	0.45	0.1667	-4	18	0.1222	-4	1	0	0	0.000000	-6.1	-1	0.1667	2.000000	0.1111	1.8889	0	0	0	0	6.1	0.85	-6.1	0	0	0	0	0.4385	1.1336	0.8045	0.1667	0.1922	0.6472	0.1973	0.0278	-1.0167
HIP_17923	LVEALYLVC	SQESGEETG	LVEALYLVCSQESGEETG		SCG1	1929/367830	1.000	17923	0.839	0.169327	0.45	0.1667	-4	18	0.1222	-4	1	0	0	0.000000	-6.1	-1	0.1667	2.000000	0.1111	1.8889	0	0	0	0	6.1	0.85	-6.1	0	0	0	0	0.4385	1.1336	0.8045	0.1667	0.1922	0.6472	0.1973	0.0278	-1.0167
HIP_17924	GSHLVEALY	SQTQEEVRD	GSHLVEALYSQTQEEVRD		SCG2	1339/334890	1.000	17924	0.839	0.178386	-0.1167	0.1667	-2	18	-0.8278	-3	1	0	0	0.000000	-3.1	0	0.1667	2.000000	0.1111	1.8889	0	0	0	0	0	-0.2204	-3.1	0	1	0	0	0.4385	1.0853	0.8678	0.3333	0.1922	0.7531	-0.0512	0.0278	-0.5167
HIP_17925	GSHLVEALY	KLSFRARAY	GSHLVEALYKLSFRARAY		CMGA	8756/247050	0.995	17925	0.839	0.146860	0.5667	0.1667	3	18	-0.0222	2	2	0	0	0.000000	-1.7333	1	0.1167	2.700000	0.2222	2.4778	0	0	0	0	-1.7333	1.4041	-3.4667	0	1	0	0	0.3436	1.1488	0.779	0.3333	0.1181	0.6068	0.1947	0.0136	-0.2022
HIP_17926	GSHLVEALY	QEEEQIEQA	GSHLVEALYQEEEQIEQA		SCG2	2466/334890	1.000	17926	0.839	0.114996	-1.0333	0	-4	18	-0.7667	-5	1	0	1	0.000000	-4.9333	-2	0.15	2.000000	0.4444	1.5556	0	0	0	0	9.8667	-1.6074	-4.9333	0	1	0	0	0.4385	1.3169	0.7545	0	0.1922	0.5693	-0.4531	0.0225	-0.74
HIP_17927	VCGERGFF	HSEKSQRED	VCGERGFFHSEKSQRED		SCG1	12189/367830	0.997	17927	0.839	0.055883	-0.3833	0.3333	0	17	-1.3941	-1	2	1	0	0.000000	-4.2333	-1	0.0333	0.700000	0.3333	0.3667	0	1	0	1	4.2333	-0.1406	-8.4667	1	0	0	0	0.4551	1.0386	0.9245	0	0.2071	0.8547	-0.1745	0.0011	-0.1411
HIP_17928	SHLVEALYL	QECFETLR	SHLVEALYLQECFETLR		CMGA	7338/247050	0.997	17928	0.839	0.143588	0.3	0	-1	17	0.1176	-2	2	1	0	0.000000	-3.6	-1	0.1	0.700000	0.2222	0.4778	0	0	0	0	3.6	0.1433	-7.2	1	0	0	0	0.4103	1.2105	0.7556	0.1667	0.1683	0.571	0.1231	0.01	-0.36
HIP_17929	LVEALYLVC	KSSAPPITP	LVEALYLVCKSSAPPITP		SCG1	9456/367830	0.998	17929	0.839	0.218167	0.8333	0.3333	0	18	0.8111	0	1	0	0	0.000000	-5.3333	1	0.1833	2.450000	0.1111	2.3389	0	0	0	0	-5.3333	1.9491	-5.3333	0	0	0	0	0.4103	1.0005	0.869	0.1667	0.1683	0.7551	0.3419	0.0136	-0.9778
HIP_17930	CGERGFFYT	SSHFERRD	CGERGFFYTSSHFERRD		SCG1	14619/367830	0.995	17930	0.839	0.093034	-0.6667	0.3333	1	17	-1.2176	0	4	1	0	0.000000	-1.8667	0	0.0833	0.700000	0	0.7	0	0	0	0	0	-0.4667	-7.4667	0	1	0	0	0.4769	0.9455	0.9623	0	0.2275	0.926	-0.3179	0.0069	-0.1556
HIP_17931	VCGERGFFY	YRGRGSEE	VCGERGFFYYRGRGSEE		SCG1	13948/367830	0.996	17931	0.839	0.169139	-0.3167	0.1667	0	17	-0.9824	0	4	1	0	0.000000	-3.5	1	0.1333	0.700000	0.3333	0.3667	0	1	0	1	-3.5	-0.1161	-14	0	1	0	0	0.3987	0.9941	0.9035	0.1667	0.159	0.8162	-0.1263	0.0178	-0.4667
HIP_17932	GSLQPLALE	NVQKFPSPE	GSLQPLALENVQKFPSPE		SCG2	15442/334890	0.998	17932	0.839	0.028943	-0.1167	0	-1	18	-0.4444	-1	0	0	0	0.000000	-1.6333	1	0.05	0.950000	0.2222	0.7278	0	0	0	0	-1.6333	-0.0849	0	0	0	1	0	0.4	1.1833	0.8233	0.3333	0.16	0.6779	-0.0467	0.0025	-0.0817

HIP_18386	QHLCGSHLV	RFQDKQYSS	QHLCGSHLVRFQDKQYSS		SCG1	26480/367830	0.976	18386	0.838	0.208158	-0.0667	0	3	18	-0.8778	1	1	0	0
HIP_18387	VCGERGFFY	QVSEEVKT	VCGERGFFYQVSEEVKT		SCG1	6664/367830	0.999	18387	0.838	0.157189	0.7	0.1667	-1	17	-0.3118	-1	3	1	0
HIP_18388	EQCCTSICS	NENTKFEVR	EQCCTSICSNENTKFEVR		SCG1	5475/367830	0.999	18388	0.838	0.093312	-0.7167	0.1667	-1	18	-0.7444	-1	0	0	0
HIP_18389	TSICSLYQL	FETLRGDER	TSICSLYQLFETLRGDER		CMGA	5855/247050	0.998	18389	0.838	-0.064184	-0.4	0	-1	18	-0.3611	-1	2	0	0
HIP_18390	SHLVEALYL	EDLLMKVLE	SHLVEALYLEDLLMKVLE		SCG2	3841/334890	1.000	18390	0.838	0.108174	0.5167	0	-2	18	0.65	-3	1	0	0
HIP_18391	HLVEALYLV	ENELTLNEK	HLVEALYLVENELTLNEK		SCG1	4185/367830	0.999	18391	0.838	0.130112	-0.6333	0	-2	18	-0.05	-3	1	0	0
HIP_18392	VCGERGFFY	VQAPEDLEW	VCGERGFFYVQAPEDLEW		SCG1	1063/367830	1.000	18392	0.838	-0.026946	1.1333	0	-3	18	-0.25	-3	3	0	0
HIP_18393	SHLVEALY	DWWEKKPFS	SHLVEALYDWWEKKPFS		SCG1	15798/367830	0.994	18393	0.838	0.093290	-0.1667	0	0	17	-0.6706	-1	3	1	0
HIP_18394	VCGERGFFY	EEEEEEAE	VCGERGFFYEEEEEEAE		CMGA	2114/247050	0.999	18394	0.838	0.103252	-1.0333	0	-7	17	-1.2059	-7	3	1	4
HIP_18395	AGSLQPLAL	RILEALRQ	AGSLQPLALRILEALRQ		SCG2	12476/334890	0.998	18395	0.838	0.068309	2.3167	0	1	18	0.6167	1	0	0	0
HIP_18396	GSHLVEALY	ESEESNVSM	GSHLVEALYESEESNVSM		SCG1	1961/367830	1.000	18396	0.838	0.146307	-0.5833	0.1667	-3	18	-0.3278	-4	1	0	0
HIP_18397	ERGFFYTPK	DLQDTKENR	ERGFFYTPKDLQDTKENR		SCG1	13428/367830	0.996	18397	0.838	-0.030232	-1.5667	0.1667	0	18	-1.8389	0	2	0	0
HIP_18398	GSHLVEALY	QEHSQQKEE	GSHLVEALYQEHSQQKEE		CMGA	7141/247050	0.997	18398	0.838	0.094160	-0.9833	0	-1	18	-1.3611	-3	1	0	0
HIP_18399	LCGSHLVEA	NDEMKRSGQ	LCGSHLVEANDEMKRSGQ		SCG2	15019/334890	0.998	18399	0.838	0.172900	-1.3333	0	0	18	-0.75	-1	0	0	0
HIP_18400	LVEALYLVC	PGKWQQQG	LVEALYLVCPGKWQQQG		SCG1	8434/367830	0.998	18400	0.838	0.206462	0.7667	0.3333	0	17	0.0941	0	2	1	1
HIP_18401	SHLVEALYL	EFPDFYDSE	SHLVEALYLEFPDFYDSE		SCG1	200/367830	1.000	18401	0.838	0.116500	0.6667	0.1667	-4	18	-0.1944	-5	2	0	0
HIP_18402	LVEALYLVC	AEAKTHWLG	LVEALYLVCAEAKTHWLG		SCG2	7441/334890	0.999	18402	0.838	0.049836	1.7667	0	0	18	0.7833	-1	1	0	0
HIP_18403	GERGFFYT	GEEDIQGPT	GERGFFYTGEEDIQGPT		SCG1	3897/367830	0.999	18403	0.838	0.091560	-1.1	0.1667	-3	17	-1.0471	-3	3	1	0
HIP_18404	LVEALYLVC	SSQGGSLPS	LVEALYLVCSSQGGSLPS		SCG1	4687/367830	0.999	18404	0.838	0.215101	0.9	0.3333	-1	18	0.7778	-1	1	0	0
HIP_18405	LVEALYLVC	EEEEEAEAG	LVEALYLVCEEEEEAEAG		CMGA	746/247050	1.000	18405	0.838	0.105073	0	0	-7	18	0.0833	-7	1	0	3
HIP_18406	GSHLVEALY	YQGVSVPLQ	GSHLVEALYYQGVSVPLQ		SCG2	3190/334890	1.000	18406	0.838	0.121977	-0.15	0.1667	0	18	0.3056	-1	2	0	0
HIP_18407	SHLVEALYL	KRFLGEGHH	SHLVEALYLKRFLGEGHH		SCG1	24201/367830	0.982	18407	0.838	0.164076	0.1167	0	3	18	-0.2167	0	2	0	0
HIP_18408	ERGFFYTPK	TQRLANFLV	ERGFFYTPKTQRLANFLV		IAPP	2657/45018	0.983	18408	0.838	-0.054446	-2.4833	0.1667	2	18	-0.3389	2	2	0	0
HIP_18409	AGSLQPLAL	FPVGPPKND	AGSLQPLALFPVGPPKND		SCG2	2999/334890	1.000	18409	0.838	0.059444	2.4667	0.1667	0	18	-0.0222	0	1	0	0
HIP_18410	HLVEALYLV	GEEGAPGKW	HLVEALYLVGEEGAPGKW		SCG1	2045/367830	1.000	18410	0.838	0.183910	-0.1167	0.1667	-1	18	0.0444	-2	1	0	0
HIP_18411	TSICSLYQL	AEPGWQAKR	TSICSLYQLAEPGWQAKR		CMGA	9442/247050	0.995	18411	0.838	0.077200	-0.7167	0.1667	1	18	-0.4	1	1	0	0
HIP_18412	LYQLENYCN	EEGSANRRP	LYQLENYCNEEGSANRRP		CMGA	3125/247050	0.999	18412	0.838	0.129939	-1.6167	0.1667	-1	18	-1.5	-1	1	0	0
HIP_18413	PGAGSLQPL	LITRQRYGK	PGAGSLQPLLITRQRYGK		NPY	1119/49410	0.994	18413	0.838	0.077042	1.05	0.1667	3	18	-0.5222	3	0	0	0
HIP_18414	LVCGERGFF	SDDYETQQW	LVCGERGFFSDDYETQQW		SCG2	4629/334890	1.000	18414	0.838	0.067115	-0.4333	0.3333	-3	18	-0.7722	-3	3	0	0
HIP_18415	CGSHLVEAL	SNTYGKRNA	CGSHLVEALSNTYGKRNA		IAPP	2109/45018	0.990	18415	0.838	0.195698	-0.4833	0.1667	2	18	-0.4778	1	1	0	0
HIP_18416	ERGFFYTPK	MESAANQKT	ERGFFYTPKMESAANQKT		SCG2	12232/334890	0.998	18416	0.838	-0.083389	-1.4333	0.3333	1	18	-1.15	1	2	0	0
HIP_18417	VCGERGFF	EEEGENYQK	VCGERGFFEEEGENYQK		SCG1	7898/367830	0.998	18417	0.838	0.049078	-0.8833	0.1667	-3	17	-1.3588	-3	2	1	1
HIP_18418	VCGERGFF	LEEPGETQN	VCGERGFFLEEPGETQN		SCG1	4148/367830	0.999	18418	0.838	-0.002202	0.3333	0.1667	-3	17	-0.7588	-3	2	1	0
HIP_18419	SICSLYQLE	DRNYLNYGE	SICSLYQLEDRNYLNYGE		SCG1	21843/367830	0.987	18419	0.838	-0.000018	-2.45	0	-2	18	-0.7222	-2	2	0	0
HIP_18420	CCTSICSLY	EEEMAVVPQ	CCTSICSLYEEEMAVVPQ		CMGA	3933/247050	0.999	18420	0.838	-0.003196	-1.4667	0.1667	-3	18	0.4833	-3	1	0	1
HIP_18421	LVEALYLVC	LTGPNNQKR	LVEALYLVCLTGPNNQKR		SCG2	2498/334890	1.000	18421	0.838	0.126279	2.2	0.1667	1	18	0.0833	1	1	0	0
HIP_18422	AGSLQPLAL	EAELEKVAH	AGSLQPLALEAELEKVAH		CMGA	3040/247050	0.999	18422	0.838	0.089553	0.7	0	-1	18	0.15	-2	0	0	0
HIP_18423	CSLYQLENY	TPNRQYWDE	CSLYQLENYTPNRQYWDE		SCG2	5677/334890	0.999	18423	0.838	-0.064163	-2.35	0.1667	-2	18	-1.4889	-2	1	0	0
HIP_18424	HLVEALYL	LNERKQASA	HLVEALYLLNERKQASA		SCG1	12369/367830	0.997	18424	0.838	0.178973	0.5167	0	1	17	-0.1706	0	1	1	0
HIP_18425	HLVEALYLV	TSEVDKRRT	HLVEALYLVTSEVDKRRT		SCG1	6071/367830	0.999	18425	0.838	0.201419	0.2833	0.1667	1	18	-0.2389	0	1	0	0
HIP_18426	SHLVEALYL	EEPGETQNA	SHLVEALYLEEPGETQNA		SCG1	2813/367830	1.000	18426	0.838	0.171772	-0.3833	0.1667	-3	18	-0.5444	-4	1	0	0
HIP_18427	LYQLENYCN	HYRKKSAEF	LYQLENYCNHYRKKSAEF		SCG1	31376/367830	0.960	18427	0.838	0.051668	-1.8833	0	2	18	-1.2778	1	2	0	0
HIP_18428	AGSLQPLAL	YPEIINSN	AGSLQPLALYPEIINSN		SCG2	4781/334890	1.000	18428	0.838	0.156834	0.5	0.1667	-1	17	0.2059	-1	1	1	0

HIP_18897	CCTSICSLY	EEEEDNRDS	CCTSICSLYEEEEDNRDS		CMGA	2728/247050	0.999	18897	0.838	-0.008856	-1.4667	0.1667	-5	18	-0.9778	-5	1
HIP_18898	GERGFFYT	EEEEDNRDS	GERGFFYTEEEEDNRDS		CMGA	5809/247050	0.998	18898	0.838	0.061134	-1.6167	0	-5	17	-2.0588	-5	3
HIP_18899	GSLQPLAL	YNYDWWEKK	GSLQPLALYNYDWWEKK		SCG1	16959/367830	0.993	18899	0.838	0.103240	0.55	0	0	17	-0.9294	0	3
HIP_18900	VCGERGFFY	EEPSSKDV	VCGERGFFYEEPSSKDV		CMGA	4689/247050	0.999	18900	0.838	0.166721	-0.7167	0.1667	-2	17	-0.6588	-2	3
HIP_18901	HLCGSHLV	LTEDEKKEL	HLCGSHLVLTEDEKKEL		SCG1	8818/367830	0.998	18901	0.838	0.148785	0.7333	0	0	17	-0.4824	-2	0
HIP_18902	VCGERGFF	DQTVLTEDE	VCGERGFFDQTVLTEDE		SCG1	3568/367830	0.999	18902	0.838	0.031770	-0.4167	0.1667	-4	17	-0.4353	-4	2
HIP_18903	GSLQPLAL	TQEEVRDSK	GSLQPLALTQEEVRDSK		SCG2	5657/334890	0.999	18903	0.838	0.183825	0.2833	0	-1	17	-0.7529	-1	0
HIP_18904	AGSLQPLAL	QINDEMKRS	AGSLQPLALQINDEMKRS		SCG2	10241/334890	0.999	18904	0.838	0.193531	1.15	0	0	18	-0.45	0	0
HIP_18905	CCTSICSLY	FRARAYGFR	CCTSICSLYFRARAYGFR		CMGA	12138/247050	0.992	18905	0.838	-0.005938	0.3	0.1667	3	18	0.3444	3	2
HIP_18906	GERGFFYTP	KRNLARVPK	GERGFFYTPKRNLARVPK		SCG1	26361/367830	0.977	18906	0.838	0.043729	-2.5833	0.1667	4	18	-1.05	4	3
HIP_18907	HLVEALYLV	PGWQAKRE	HLVEALYLVPGWQAKRE		CMGA	1752/247050	1.000	18907	0.838	0.184327	0.6333	0.3333	1	17	-0.1706	0	2
HIP_18908	QCCTSICSL	LSFRARAYG	QCCTSICSLLSFRARAYG		CMGA	6599/247050	0.998	18908	0.838	0.020800	1.8833	0.3333	2	18	0.4833	2	1
HIP_18909	ERGFFYTPK	LENLAAMDL	ERGFFYTPKLENLAAMDL		SCG1	6590/367830	0.999	18909	0.838	-0.114554	-1.5667	0.1667	-1	18	-0.2167	-1	2
HIP_18910	TSICSLYQL	ALAEAYPS	TSICSLYQLALAEAYPS		NPY	511/49410	0.998	18910	0.838	0.000468	1.0667	0	-1	17	0.5588	-1	1
HIP_18911	VEALYLVC	PYQGVSVPL	VEALYLVCPYQGVSVPL		SCG2	8505/334890	0.999	18911	0.838	0.148267	0.6833	0.1667	-1	17	1.0882	-1	2
HIP_18912	LVEALYLVC	LEYLNQEKA	LVEALYLVCLEYLNQEKA		SCG2	6609/334890	0.999	18912	0.838	0.026130	1.5833	0	-2	18	0.5278	-2	2
HIP_18913	HLVEALYLV	ERDSLSEED	HLVEALYLVERDSLSEED		SCG2	6186/334890	0.999	18913	0.838	0.160738	-0.8	0	-4	18	-0.3444	-5	1
HIP_18914	AGSLQPLAL	SPETLISDL	AGSLQPLALSPETLISDL		NPY	86/49410	1.000	18914	0.838	0.203404	0.5833	0.3333	-2	18	0.55	-2	0
HIP_18915	RGFFYTPKT	STHYRASEE	RGFFYTPKTSTHYRASEE		SCG1	15769/367830	0.994	18915	0.838	-0.017875	-1.8167	0.3333	2	18	-1.3333	1	2
HIP_18916	LVEALYLVC	ELDKMAHG	LVEALYLVCELDKMAHG		SCG1	5812/367830	0.999	18916	0.838	0.106679	1.2167	0	-1	17	0.7235	-2	1
HIP_18917	CGERGFFY	QEEVRDSKE	CGERGFFYQEEVRDSKE		SCG2	9812/334890	0.999	18917	0.838	0.165856	-1.0333	0	-2	17	-1.4412	-2	3
HIP_18918	HLVEALYLV	PSSKDVMEK	HLVEALYLVPSSKDVMEK		CMGA	1061/247050	1.000	18918	0.838	0.214044	0.5833	0.5	0	18	0.0944	-1	1
HIP_18919	VEALYLVC	PKAAWIPHV	VEALYLVCPKAAWIPHV		SCG2	14522/334890	0.998	18919	0.838	0.178016	1.1333	0.1667	1	17	0.9765	0	2
HIP_18920	LVEALYLVC	YPEIINSNQ	LVEALYLVCYPEIINSNQ		SCG2	4324/334890	1.000	18920	0.838	0.103812	0.6833	0.1667	-2	18	0.5889	-2	2
HIP_18921	LVEALYLVC	WNPVEEKIE	LVEALYLVCWNPVEEKIE		SCG2	3013/334890	1.000	18921	0.838	0.100829	0.75	0.1667	-3	18	0.4222	-3	2
HIP_18922	GERGFFYTP	GEDSSEEKH	GERGFFYTPGEDSSEEKH		SCG1	2410/367830	1.000	18922	0.838	-0.035154	-1.8333	0.3333	-2	18	-1.6611	-3	3
HIP_18923	ERGFFYTPK	ERATRLFEK	ERGFFYTPKERATRLFEK		SCG2	21399/334890	0.995	18923	0.838	0.023668	-2.0667	0.1667	2	18	-1.25	2	2
HIP_18924	CGERGFFYT	SQVSEEVKT	CGERGFFYTSQVSEEVKT		SCG1	5086/367830	0.999	18924	0.838	0.027908	0.1167	0.1667	-1	18	-0.6111	-1	3
HIP_18925	CTSICSLYQ	SEEERGLEP	CTSICSLYQSEEERGLEP		SCG1	3968/367830	0.999	18925	0.838	0.105838	-1.4667	0.1667	-3	18	-0.6278	-3	1
HIP_18926	QHLCGSHLV	QELGEYLAR	QHLCGSHLVQELGEYLAR		SCG2	10697/334890	0.999	18926	0.838	0.160009	0.2667	0	1	18	-0.2278	-1	0
HIP_18927	GPGAGSLQ	RGFFYTPKT	GPGAGSLQRGFFYTPKT		INS8	219/4968	0.968	18927	0.838	0.032662	-0.4333	0.3333	2	17	-0.5294	2	3
HIP_18928	CGERGFFY	ENYQKGER	CGERGFFYENYQKGER		SCG1	14883/367830	0.995	18928	0.838	0.130128	-0.6667	0	0	16	-1.6312	0	4
HIP_18929	CGERGFFY	REDEEEEEG	CGERGFFYREDEEEEEG		SCG1	8153/367830	0.998	18929	0.838	0.153022	-1.2	0	-6	17	-1.8471	-6	3
HIP_18930	AGSLQPLAL	KGRHHRGRG	AGSLQPLALKGRHHRGRG		SCG1	20283/367830	0.989	18930	0.838	0.234731	0.1	0.1667	6	18	-0.9056	4	0
HIP_18931	SLYQLENYC	RLENVQKFP	SLYQLENYCRLENVQKFP		SCG2	28145/334890	0.992	18931	0.838	0.029319	-1.0833	0	0	18	-0.75	0	1
HIP_18932	IVEQCCTSI	TQQWPERKL	IVEQCCTSITQQWPERKL		SCG2	15714/334890	0.997	18932	0.838	0.048898	-0.7833	0.1667	0	18	-0.4778	0	1
HIP_18933	SICSLYQLE	PGKWQQQGD	SICSLYQLEPGKWQQQGD		SCG1	4454/367830	0.999	18933	0.838	-0.004297	-1.5167	0.3333	-1	18	-0.9167	-1	2
HIP_18934	SLQPLALEG	EEEGPTVVL	SLQPLALEGEEEGPTVVL		CMGA	3192/247050	0.999	18934	0.838	-0.089794	-1.7667	0.1667	-4	18	0.1333	-4	0
HIP_18935	HLCGSHLVE	EEEEMAVVP	HLCGSHLVEEEEEMAVVP		CMGA	3694/247050	0.999	18935	0.838	0.054667	-1	0	-3	18	-0.0167	-5	0
HIP_18936	TSICSLYQL	PLIFLISGA	TSICSLYQLPLIFLISGA		SCG2	1880/334890	1.000	18936	0.838	-0.079466	0.95	0.1667	0	18	1.4389	0	2
HIP_18937	SHLVEALYL	SDTLSKPS	SHLVEALYLSDTLSKPS		CMGA	4630/247050	0.999	18937	0.838	0.233035	0.2167	0.1667	0	17	0.0176	-1	1
HIP_18938	LVCGERGFF	EEERGLEP	LVCGERGFFEEERGLEP		SCG1	7954/367830	0.998	18938	0.838	0.050905	-0.8833	0.1667	-3	17	-0.5529	-3	2
HIP_18939	LVEALYLVC	AVEVLKREP	LVEALYLVCAVEVLKREP		IAPP	797/45018	0.997	18939	0.838	0.020621	2.1667	0	-1	18	0.9056	-1	1
[illegible]																	

HIP_19408	AGSLQPLAL	DASEAHESS	AGSLQPLALDASEAHESS		SCG1	1591/367830	1.000	19408	0.837	0.152794	1.15	0.1667	-2	18	-0.2111	-3	0	0	0
HIP_19409	LYQLENYCN	NYPSLELDK	LYQLENYCNNYPSLELDK		SCG1	4198/367830	0.999	19409	0.837	-0.016389	-1.45	0.1667	-2	18	-0.9444	-2	2	0	0
HIP_19410	LYQLENYCN	YGAGRSRSN	LYQLENYCNYGAGRSRSN		SCG2	6326/334890	0.999	19410	0.837	-0.070421	-0.3667	0.1667	1	18	-1.1611	1	2	0	0
HIP_19411	GERGFFYTP	YSALRHYIN	GERGFFYTPYSALRHYIN		NPY	624/49410	0.997	19411	0.837	-0.091425	-0.65	0.3333	2	18	-0.6278	1	4	0	0
HIP_19412	GSHLVEALY	RQVLKTSRK	GSHLVEALYRQVLKTSRK		SCG1	22582/367830	0.985	19412	0.837	0.137276	0.0833	0	4	18	-0.5222	3	1	0	0
HIP_19413	LGGGPGAGS	ERGFFYTP	LGGGPGAGSERGFFYTP		INSB	119/4968	0.989	19413	0.837	0.002703	-1.3	0.5	0	17	-0.3059	0	1	1	1
HIP_19414	LVEALYLVC	NDDTPNRQY	LVEALYLVCNDDTPNRQY		SCG2	5295/334890	0.999	19414	0.837	0.143648	0	0	-2	18	-0.35	-2	1	0	0
HIP_19415	EAEDLQVGQ	EEEGSANRR	EAEDLQVGQEEEGSANRR		CMGA	2456/247050	0.999	19415	0.837	-0.085943	-1.7	0.1667	-4	18	-1.6944	-4	0	0	1
HIP_19416	GERGFFYT	EPGWQAKR	GERGFFYTEPGWQAKR		CMGA	5432/247050	0.998	19416	0.837	0.163841	-0.7833	0.3333	1	16	-1.3562	1	4	0	0
HIP_19417	VCGERGFFY	PTVVLNPHP	VCGERGFFYPTVVLNPHP		CMGA	782/247050	1.000	19417	0.837	0.108583	1.0333	0.1667	1	18	0.1222	0	3	0	0
HIP_19418	CGERGFFY	SSAPPITPE	CGERGFFYSSAPPITPE		SCG1	5165/367830	0.999	19418	0.837	0.177843	0.75	0.3333	-1	17	-0.3706	-1	3	1	0
HIP_19419	CGERGFFY	GEETGSQE	CGERGFFYGEETGSQE		SCG1	4573/367830	0.999	19419	0.837	0.171812	-0.5167	0.1667	-3	16	-1.1438	-3	3	0	0
HIP_19420	LVCGERGFF	GEPRAYFM	LVCGERGFFGEPRAYFM		SCG1	2367/367830	1.000	19420	0.837	0.136488	-0.05	0.5	0	17	0.1471	0	2	1	0
HIP_19421	YLVCGERGF	EEEAEAGEE	YLVCGERGFEEEAEAGEE		CMGA	2166/247050	0.999	19421	0.837	-0.057425	-2.1	0.1667	-6	18	-0.8111	-6	1	0	1
HIP_19422	QHLCGSHLV	LGEKRDHHS	QHLCGSHLVLGEKRDHHS		SCG1	6647/367830	0.999	19422	0.837	0.164576	0.7833	0.1667	4	18	-0.8889	0	0	0	0
HIP_19423	VNQHLCGSH	EEEEEAEAG	VNQHLCGSHEEEEEAEAG		CMGA	2108/247050	0.999	19423	0.837	-0.116370	-2.4833	0.3333	-4	18	-1.2167	-6	0	0	3
HIP_19424	LVEALYLV	ELDKMAHGY	LVEALYLVELDKMAHGY		SCG1	4509/367830	0.999	19424	0.837	0.069280	0.5833	0	-1	17	0.5	-2	1	1	0
HIP_19425	LCGSHLVEA	NLRQQAHKE	LCGSHLVEANLRQQAHKE		SCG2	28183/334890	0.992	19425	0.837	0.118209	-0.2833	0	2	18	-0.6556	0	0	0	0
HIP_19426	HLVEALYLV	HKEESSPDY	HLVEALYLVHKEESSPDY		SCG2	15709/334890	0.997	19426	0.837	0.157269	-0.65	0	-1	18	-0.4722	-3	1	0	0
HIP_19427	VCGERGFF	PGKGRHHRG	VCGERGFFPGKGRHHRG		SCG1	15337/367830	0.995	19427	0.837	0.170582	-0.1167	0.5	5	17	-1.0941	3	2	1	0
HIP_19428	CGERGFFY	GEEDIQGP	CGERGFFYGEEDIQGP		SCG1	4688/367830	0.999	19428	0.837	0.173078	-0.5167	0.1667	-3	16	-0.8688	-3	3	0	0
HIP_19429	LCGSHLVEA	LLQKEPDLR	LCGSHLVEALLQKEPDLR		SCG2	10435/334890	0.999	19429	0.837	-0.010319	1.1	0	0	18	-0.05	-1	0	0	0
HIP_19430	HLVEALYL	ETQQWPER	HLVEALYLETQQWPER		SCG2	8852/334890	0.999	19430	0.837	0.222377	-0.2333	0	-1	16	-0.7688	-2	1	0	0
HIP_19431	AGSLQPLAL	AELEKVAHQ	AGSLQPLALAELEKVAHQ		CMGA	6546/247050	0.998	19431	0.837	0.055242	1.9167	0	0	18	0.15	-1	0	0	0
HIP_19432	LVCGERGFF	PEDLEWER	LVCGERGFFPEDLEWER		SCG1	3366/367830	1.000	19432	0.837	0.046314	-0.5667	0.3333	-3	17	-0.5824	-3	2	1	0
HIP_19433	SLYQLENYC	HNEGMVTRC	SLYQLENYCHNEGMVTRC		SCG1	20062/367830	0.989	19433	0.837	0.019900	-2.0833	0	0	18	-0.6111	-1	1	0	0
HIP_19434	CGERGFFYT	KTHWLGAAL	CGERGFFYTKTHWLGAAL		SCG2	21554/334890	0.995	19434	0.837	0.100335	-1.1667	0	2	18	-0.0333	1	4	0	0
HIP_19435	SHLVEALYL	TEKWAEGG	SHLVEALYLTEKWAEGG		SCG1	15488/367830	0.994	19435	0.837	0.214686	-0.3	0	-1	17	-0.1706	-2	2	1	0
HIP_19436	LVCGERGFF	RKDVKDKET	LVCGERGFFRKDVKDKET		SCG1	22726/367830	0.985	19436	0.837	0.106705	-1.1167	0.1667	1	18	-0.8833	1	2	0	0
HIP_19437	GERGFFYTP	ELTLNEKNF	GERGFFYTPELTLNEKNF		SCG1	3720/367830	0.999	19437	0.837	-0.164146	-0.6667	0.1667	-1	18	-0.8333	-1	3	0	0
HIP_19438	AGSLQPLAL	KKPFSEDVN	AGSLQPLALKKPFSEDVN		SCG1	17421/367830	0.993	19438	0.837	0.206055	0	0.1667	0	18	-0.2778	0	1	0	0
HIP_19439	GSHLVEALY	PEEEGPTVV	GSHLVEALYPEEEGPTVV		CMGA	425/247050	1.000	19439	0.837	0.138025	-0.7167	0.1667	-3	18	-0.1111	-4	1	0	1
HIP_19440	TSICSLYQL	LFRGGKSGE	TSICSLYQLLFRGGKSGE		CMGA	9537/247050	0.995	19440	0.837	-0.029435	0.1833	0	1	18	0.0111	1	2	0	0
HIP_19441	GSHLVEALY	SFRARAYGF	GSHLVEALYSFRARAYGF		CMGA	9957/247050	0.995	19441	0.837	0.153558	0.3	0.1667	2	18	0.1167	1	2	0	0
HIP_19442	CGSHLVEAL	SRDNPFKRT	CGSHLVEALSRDNPFKRT		SCG2	23900/334890	0.994	19442	0.837	0.201382	-1.1167	0.1667	2	18	-0.6667	1	0	0	0
HIP_19443	YLVCGERGF	QWKSSHFER	YLVCGERGFQWKSSHFER		SCG1	20591/367830	0.989	19443	0.837	0.057443	-1.7333	0.1667	2	18	-0.8389	1	2	0	0
HIP_19444	AGSLQPLAL	PYNQEKVL	AGSLQPLALPYNQEKVL		SCG2	9720/334890	0.999	19444	0.837	0.208085	0.5	0.1667	0	17	-0.0353	0	1	1	0
HIP_19445	GAGSLQPLA	DWWEKKPFS	GAGSLQPLADWWEKKPFS		SCG1	9689/367830	0.998	19445	0.837	-0.006709	-0.2167	0.1667	0	18	-0.65	0	2	0	0
HIP_19446	NQHLCGSHL	AWIPHVENR	NQHLCGSHLAWIPHVENR		SCG2	5966/334890	0.999	19446	0.837	0.032274	0.8667	0.1667	3	18	-0.6222	0	1	0	0
HIP_19447	GSHLVEALY	DQLSDDVSK	GSHLVEALYDQLSDDVSK		SCG2	2328/334890	1.000	19447	0.837	0.082747	0.1833	0	-2	18	-0.3944	-3	1	0	0
HIP_19448	CGERGFFY	AYGFRGPGP	CGERGFFYAYGFRGPGP		CMGA	7801/247050	0.997	19448	0.837	0.107742	0.7333	0.1667	1	17	-0.4471	1	5	1	0
HIP_19449	HLVEALYLV	DLELQKIA	HLVEALYLVDLELQKIA		SCG1	4661/367830	0.999	19449	0.837	0.093919	0.5833	0	-1	17	0.7706	-2	1	1	0
HIP_19450	IVEQCCTSI	EQEEERLSK	IVEQCCTSIEQEEERLSK		CMGA	4041/247050	0.999	19450	0.837	-0.037612	-1.25	0.1667	-3	18	-0.7333	-3	0	0	1
HIP_19451	IVEQCCTSI	QEEERLSKE	IVEQCCTSIQEEERLSKE		CMGA	4042/247050	0.999	19451	0.837	-0.037612	-1.25	0.1667	-3	18	-0.7333	-3	0	0	1
HIP_19452	VCGERGFF	SQENHPQE	VCGERGFFSQENHPQE		SCG1	7596/367830	0.999	19452	0.837	0.095236	-0.4333	0.3333	-1	16	-1.225	-2	2	0	0
HIP_19453	GERGFFYT	EPGWQAKRE	GERGFFYTEPGWQAKRE		CMGA	4186/247050	0.999	19453	0.837	0.145285	-0.7833	0.3333	0	17	-1.4824	0	4	1	0
HIP_19454	AGSLQPLAL	EGSANRRPE	AGSLQPLALEGSANRRPE		CMGA	1961/247050	0.999	19454	0.837	0.217626	0.7833	0.3333	0	18	-0.6556	0	0	0	0
HIP_19455	RGFFYTPKT	FPEYNYDWW	RGFFYTPKTFPEYNYDWW		SCG1	8596/367830	0.998	19455	0.837	-0.084133	-1.4167	0.3333	0	18	-1.1778	0	3	0	0

HIP_19919	TSICSLYQL	EIVEEQYTP	TSICSLYQLEIVEEQYTP		SCG2	6423/334890	0.999	19919	0.837	-0.065760	0.7	0	-3	18	-0.0778	-3	1	0	0	[illegible]
HIP_19920	CGERGFFY	NGSVEPER	CGERGFFYNGSVEPER		SCG2	3839/334890	1.000	19920	0.837	0.184186	-0.0667	0.3333	-1	16	-0.975	-1	3	0	0	[illegible]
HIP_19921	GERGFFYTP	SLGYKEIRK	GERGFFYTPSLGYKEIRK		CMGA	2937/247050	0.999	19921	0.837	-0.094683	-0.1667	0.5	2	18	-0.9333	2	4	0	0	[illegible]
HIP_19922	IVEQCCTSI	GKGRHHRGR	IVEQCCTSIGKGRHHRGR		SCG1	14741/367830	0.995	19922	0.837	0.138782	-0.2833	0.5	5	18	-0.85	3	0	0	0	[illegible]
HIP_19923	LVCGERGFF	SKEAEKSGE	LVCGERGFFSKEAEKSGE		CMGA	8614/247050	0.996	19923	0.837	0.108490	-0.5	0.3333	-1	18	-0.6222	-1	2	0	0	[illegible]
HIP_19924	CGERGFFYT	ERRDNMNDN	CGERGFFYTERRDNMNDN		SCG1	22929/367830	0.983	19924	0.837	0.149619	-1.95	0	-1	18	-1.7111	-1	3	0	0	[illegible]
HIP_19925	VCGERGFF	LGNKRLGLS	VCGERGFFLGNKRLGLS		NPY	906/49410	0.995	19925	0.837	0.078629	0.85	0.3333	2	17	0.0824	2	2	0	0	[illegible]
HIP_19926	ERGFFYTPK	INLITRQRY	ERGFFYTPKINLITRQRY		NPY	924/49410	0.995	19926	0.837	0.018111	-0.2333	0.1667	3	18	-0.8611	3	2	1	0	[illegible]
HIP_19927	GERGFFYT	PECRQVLKT	GERGFFYTPECRQVLKT		SCG1	14919/367830	0.995	19927	0.837	0.072957	-0.3	0.1667	1	17	-0.7294	1	3	1	0	[illegible]
HIP_19928	LYQLENYCN	LQWKSSHFE	LYQLENYCNLQWKSSHFE		SCG1	7174/367830	0.999	19928	0.837	-0.146666	-0.4833	0	0	18	-0.9167	-1	2	0	0	[illegible]
HIP_19929	AGSLQPLAL	GAVGLAAVN	AGSLQPLALGAVGLAAVN		SCG1	1411/367830	1.000	19929	0.837	0.084904	2.5	0.1667	0	18	1.2222	0	0	0	0	[illegible]
HIP_19930	CGERGFFYT	LAKELTAEK	CGERGFFYTLAKELTAEK		CMGA	8820/247050	0.996	19930	0.837	-0.039622	0.4167	0	0	18	-0.3889	0	3	0	0	[illegible]
HIP_19931	SHLVEALYL	QGSSEDDLQ	SHLVEALYLQGSSEDDLQ		SCG2	424/334890	1.000	19931	0.837	0.209419	0.2667	0.3333	-3	18	-0.3944	-4	1	0	0	[illegible]
HIP_19932	GSLQPLAL	EQYTPQSLA	GSLQPLALEQYTPQSLA		SCG2	11025/334890	0.999	19932	0.837	0.156614	0.1833	0	-1	17	-0.1412	-1	2	0	0	[illegible]
HIP_19933	LVEALYLVC	DTKENREEA	LVEALYLVCDTKENREEA		SCG1	10021/367830	0.998	19933	0.837	0.192765	0.4	0	-3	18	-0.3056	-3	1	0	0	[illegible]
HIP_19934	SHLVEALYL	QYSSHHTA	SHLVEALYLQYSSHHTA		SCG1	16258/367830	0.994	19934	0.837	0.197419	0.1167	0.1667	2	17	-0.1824	-1	2	1	0	[illegible]
HIP_19935	CGERGFFY	DGARPQALP	CGERGFFYDGARPQALP		CMGA	5950/247050	0.998	19935	0.837	0.186566	0.3667	0.1667	0	17	-0.5706	0	3	1	0	[illegible]
HIP_19936	SLYQLENYC	EDEKKELEN	SLYQLENYCEDEKKELEN		SCG1	8563/367830	0.998	19936	0.837	0.042035	-2.1333	0	-4	18	-1.6	-4	1	0	0	[illegible]
HIP_19937	AGSLQPLAL	VKDKETTE	AGSLQPLALVKDKETTE		SCG1	13046/367830	0.996	19937	0.837	0.171143	1.0333	0	-1	17	-0.4	-1	0	1	0	[illegible]
HIP_19938	SHLVEALYL	GFRGPGPQ	SHLVEALYLGFRGPGPQ		CMGA	9081/247050	0.996	19938	0.837	0.219989	0.7	0.1667	1	17	-0.0588	0	2	1	0	[illegible]
HIP_19939	LVCGERGFF	PETLISDLL	LVCGERGFFPETLISDLL		NPY	98/49410	1.000	19939	0.837	0.053028	-0.1	0.3333	-2	18	0.7278	-2	2	0	0	[illegible]
HIP_19940	SHLVEALY	EEERLSKEW	SHLVEALYEEERLSKEW		CMGA	5180/247050	0.998	19940	0.837	0.136930	-1.0333	0	-2	17	-0.9118	-3	1	1	1	[illegible]
HIP_19941	ICSLYQLEN	EDLRKESKD	ICSLYQLENEDLRKESKD		SCG2	19489/334890	0.996	19941	0.837	-0.036119	-1.0667	0	-2	18	-1.1833	-2	1	0	0	[illegible]
HIP_19942	GSHLVEALY	EDVNWGYEK	GSHLVEALYEDVNWGYEK		SCG1	3919/367830	0.999	19942	0.837	0.071292	0.25	0	-2	18	-0.6611	-3	1	0	0	[illegible]
HIP_19943	LVEALYLV	GYEKRNLA	LVEALYLVGYEKRNLA		SCG1	7601/367830	0.999	19943	0.837	0.172642	0.25	0.1667	0	16	0.3312	0	2	0	0	[illegible]
HIP_19944	LVCGERGFF	EMAVVPQGL	LVCGERGFFEMAVVPQGL		CMGA	1064/247050	1.000	19944	0.837	-0.018829	0.9	0.1667	-1	18	0.7889	-1	2	0	0	[illegible]
HIP_19945	HLVEALYLV	EENELTLNE	HLVEALYLVEENELTLNE		SCG1	3237/367830	1.000	19945	0.837	0.130457	-0.6333	0	-4	18	-0.0278	-5	1	0	0	[illegible]
HIP_19946	LVCGERGFF	PASLPSQKY	LVCGERGFFPASLPSQKY		CMGA	790/247050	1.000	19946	0.837	0.025905	0.7667	0.5	1	18	-0.0333	1	2	0	0	[illegible]
HIP_19947	VEALYLVC	EFPDFYDSE	VEALYLVCEFPDFYDSE		SCG1	1900/367830	1.000	19947	0.837	0.091303	1.3667	0.1667	-5	17	0.2	-5	3	1	0	[illegible]
HIP_19948	CGSHLVEAL	NQLPKAAWI	CGSHLVEALNQLPKAAWI		SCG2	18222/334890	0.997	19948	0.837	0.043165	-0.1833	0	1	18	0.3722	0	0	0	0	[illegible]
HIP_19949	LVCGERGFF	TREEKRFL	LVCGERGFFTREEKRFL		SCG1	19869/367830	0.990	19949	0.837	0.126810	-0.5833	0.1667	1	17	-0.3941	1	2	1	0	[illegible]
HIP_19950	LVCGERGFF	GDTEVMKCI	LVCGERGFFGDTEVMKCI		CMGA	1427/247050	1.000	19950	0.837	0.115777	0.1	0.3333	-1	18	0.4667	-1	2	0	0	[illegible]
HIP_19951	LVEALYLVC	AHESSSRGE	LVEALYLVCAHESSSRGE		SCG1	9019/367830	0.998	19951	0.837	0.048340	0.9333	0	-1	18	0.2	-2	1	0	1	[illegible]
HIP_19952	CGERGFFY	NYGEEGAPG	CGERGFFYNYGEEGAPG		SCG1	8098/367830	0.998	19952	0.837	0.151398	-0.15	0.1667	-2	17	-0.8706	-2	4	1	0	[illegible]
HIP_19953	LVEALYLVC	EEPGETQNA	LVEALYLVCEEPGETQNA		SCG1	1494/367830	1.000	19953	0.837	0.138499	0.3167	0.1667	-4	18	0.05	-4	1	0	0	[illegible]
HIP_19954	GERGFFYTP	LSKSSAPPI	GERGFFYTPLSKSSAPPI		SCG1	7012/367830	0.999	19954	0.837	-0.130684	-0.75	0.3333	1	18	-0.3444	1	3	0	0	[illegible]
HIP_19955	HLCGSHLVE	EEEATNTHP	HLCGSHLVEEEEATNTHP		CMGA	5745/247050	0.998	19955	0.837	0.058145	-1	0	-1	18	-0.8444	-4	0	0	2	[illegible]
HIP_19956	GSHLVEALY	EEAQDPEGK	GSHLVEALYEEAQDPEGK		CMGA	2893/247050	0.999	19956	0.837	0.055486	-0.15	0	-3	18	-0.9556	-4	1	0	0	[illegible]
HIP_19957	CCTSICSLY	NPYNQEKVL	CCTSICSLYNPYNQEKVL		SCG2	8359/334890	0.999	19957	0.837	-0.005472	-0.7833	0.3333	0	18	-0.0333	0	2	0	0	[illegible]
HIP_19958	GSHLVEALY	QQKEEEEEM	GSHLVEALYQQKEEEEEM		CMGA	6227/247050	0.998	19958	0.837	0.146525	-1.1	0	-4	18	-1.2278	-5	1	0	3	[illegible]
HIP_19959	CGERGFFYT	LQIPPEDLI	CGERGFFYTLQIPPEDLI		SCG2	1662/334890	1.000	19959	0.837	-0.100459	0.9333	0	-2	18	0.0111	-2	3	0	0	[illegible]
HIP_19960	GERGFFYTP	SEKSQREDE	GERGFFYTPSEKSQREDE		SCG1	13091/367830	0.996	19960	0.837	-0.017866	-1.9667	0.3333	-2	18	-1.9056	-2	3	0	0	[illegible]
HIP_19961	SLYQLENYC	NEPQSAPKE	SLYQLENYCNEPQSAPKE		SCG2	3373/334890	1.000	19961	0.837	0.079244	-1.8167	0.1667	-2	18	-1.3278	-2	1	0	0	[illegible]
HIP_19962	RGFFYTPKT	RPEDQELES	RGFFYTPKTRPEDQELES		CMGA	10916/247050	0.994	19962	0.837	-0.043620	-2.6333	0.3333	-1	18	-1.5611	-1	3	0	0	[illegible]
HIP_19963	GERGFFYTP	PGKWQQQGD	GERGFFYTPPGKWQQQGD		SCG1	4162/367830	0.999	19963	0.837	-0.001517	-1.5833	0.5	0	18	-1.5556	0	4	0	1	[illegible]
HIP_19964	LYQLENYCN	WDEDLLMKV	LYQLENYCNWDEDLLMKV		SCG2	9465/334890	0.999	19964	0.837	-0.002638	-1.7	0	-3	18	-0.45	-3	2	0	0	[illegible]
HIP_19965	CGERGFFYT	QVFLIVLSV	CGERGFFYTQVFLIVLSV		IAPP	780/45018	0.997	19965	0.837	-0.063072	0.7167	0	0	18	1.1389	0	4	0	0	[illegible]
HIP_19966	VEALYLVC	EEERLSKEW	VEALYLVCEEERLSKEW		CMGA	4965/247050	0.998	19966	0.837	0.144647	0	0	-3	17	-0.2824	-3	1	1	1	[illegible]
HIP_19967	CGERGFFYT	DEDLLMKVL	CGERGFFYTDEDLLMKVL		SCG2	10139/334890	0.999	19967	0.837	0.079327	-1.6167	0	-2	18	0.0222	-2	3	0	0	[illegible]
HIP_19968	GSLQPLAL	PQGLFRGGK	GSLQPLALPQGLFRGGK		CMGA	4563/247050	0.999	19968	0.837	0.231055	0.65	0.3333	2	17	-0.0706	2	1	1	0	[illegible]
HIP_19969	ERGFFYTPK	DKRNYPSLE	ERGFFYTPKDKRNYPSLE		SCG1	28461/367830	0.971	19969	0.837	0.008832	-3.0167	0.1667	1	18	-1.6167	1	2	0	0	[illegible]
HIP_19970	GERGFFYTP	KKPFSEDVN	GERGFFYTPKKPFSEDVN		SCG1	17944/367830	0.992	19970	0.837	0.010817	-2.1667	0.3333	0	18	-1.1389	0	4	0	0	[illegible]
HIP_19971	SHLVEALY	STENVPRTR	SHLVEALYSTENVPRTR		NPY	1306/49410	0.993	19971	0.837	0.233811	-0.1167	0.1667	1	17	-0.6353	0	1	1	0	[illegible]
HIP_19972	GSHLVEALY	RSQEESEEG	GSHLVEALYRSQEESEEG		SCG1	8755/367830	0.998	19972	0.837	0.176308	-0.75	0.1667	-3	18	-1.0889	-4	1	0	0	[illegible]
HIP_19973	CGERGFFYT	SMASLGEKR	CGERGFFYTSMASLGEKR		SCG1	11875/367830	0.997	19973	0.837	-0.018946	0.6167	0.1667	1	18	-0.5056	1	3	0	0	[illegible]
HIP_19974	LCGSHLVEA	LEYLNQEKA	LCGSHLVEALEYLNQEKA		SCG2	11477/334890	0.999	19974	0.837	-0.054998	0.25	0	-1	18	-0.0889	-2	1	0	0	[illegible]
HIP_19975	CGERGFFY	PEPMQESK	CGERGFFYPEPMQESK		CMGA	4084/247050	0.999	19975	0.837	0.182549	-0.4	0.3333	-1	16	-1.1562	-1	3	0	0	[illegible]
HIP_19976	CTSICSLYQ	DSSMKLSFR	CTSICSLYQDSSMKLSFR		CMGA	6489/247050	0.998	19976	0.837	0.178292	-1.0167	0.3333	-1	18	0.0222	1	1	0	0	[illegible]
[illegible]	[illegible]	[illegible]	[illegible]		[illegible]	[illegible]	[illegible]	[illegible]	[illegible]	[illegible]	[illegible]	[illegible]	[illegible]	[illegible]	[illegible]	[illegible]	[illegible]	[illegible]	[illegible]	[illegible]

HIP_20430	GSHLVEALY	GEYLARMLV	GSHLVEALYGEYLARMLV	SCG2	3674/334890	1.000	20430	0.836	0.120802
HIP_20431	HLCGSHLVE	FPDFYDSEE	HLCGSHLVEFPDFYDSEE	SCG1	1613/367830	1.000	20431	0.836	-0.018435
HIP_20432	VEALYLVCG	QYSSHHTAE	VEALYLVCGQYSSHHTAE	SCG1	8889/367830	0.998	20432	0.836	0.037834
HIP_20433	HLVEALYLV	LSKSGYPKT	HLVEALYLVLSKSGYPKT	SCG2	12219/334890	0.998	20433	0.836	0.166058
HIP_20434	CGERGFFY	AQDPEGKGE	CGERGFFYAQDPEGKGE	CMGA	9176/247050	0.996	20434	0.836	0.129597
HIP_20435	VEALYLVC	GEEIKGYPG	VEALYLVCGEEIKGYPG	SCG1	4378/367830	0.999	20435	0.836	0.188055
HIP_20436	CSLYQLENY	KEHLNQGSS	CSLYQLENYKEHLNQGSS	SCG2	28791/334890	0.991	20436	0.836	-0.056035
HIP_20437	ALYLVCGER	SKEWEDSKR	ALYLVCGERSKEWEDSKR	CMGA	14470/247050	0.988	20437	0.836	-0.082464
HIP_20438	HLVEALYL	EPEYGEEI	HLVEALYLEPEYGEEI	SCG1	4726/367830	0.999	20438	0.836	0.158615
HIP_20439	AGSLQPLAL	ESKGQPRSQ	AGSLQPLALESKGQPRSQ	SCG1	14973/367830	0.995	20439	0.836	0.253858

[illegible]

HIP_20941	GERGFFYT	QSEESWDEE	GERGFFYTQSEESWDEE		SCG1	7063/367830	0.999	20941	0.835	0.086280	[illegible]
HIP_20942	HLVEALYLV	EGGGHSRER	HLVEALYLVEGGGHSRER		SCG1	1685/367830	1.000	20942	0.835	0.210514	[illegible]
HIP_20943	HLVEALYLV	SHLPERDSL	HLVEALYLVSHLPERDSL		SCG2	6018/334890	0.999	20943	0.835	0.072413	[illegible]
HIP_20944	HLVEALYL	ALAEAYPS	HLVEALYLALAEAYPS		NPY	922/49410	0.995	20944	0.835	-0.002370	[illegible]
HIP_20945	HLVEALYLV	QEEVRDSKE	HLVEALYLVQEEVRDSKE		SCG2	5558/334890	0.999	20945	0.835	0.128156	[illegible]
HIP_20946	TSICSLYQL	EGENYQKGE	TSICSLYQLEGENYQKGE		SCG1	3838/367830	0.999	20946	0.835	0.156183	[illegible]
HIP_20947	GAGSLQPLA	REEKRFLGE	GAGSLQPLAREEKRFLGE		SCG1	14173/367830	0.995	20947	0.835	0.070796	[illegible]
HIP_20948	LVEALYLVC	WKELDRNY	LVEALYLVCWKELDRNY		SCG1	15199/367830	0.995	20948	0.835	0.128053	[illegible]
HIP_20949	GAGSLQPLA	EPGKGRHHR	GAGSLQPLAEPGKGRHHR		SCG1	3524/367830	1.000	20949	0.835	0.136670	[illegible]
HIP_20950	LVEALYLVC	GERGEDSSE	LVEALYLVCGERGEDSSE		SCG1	5041/367830	0.999	20950	0.835	0.193277	[illegible]
HIP_20951	VEALYLVC	LDQLLHYRK	VEALYLVCLDQLLHYRK		SCG1	12241/367830	0.997	20951	0.835	0.164466	[illegible]
HIP_20952	LVEALYLVC	VLSNALSKS	LVEALYLVCVLSNALSKS		SCG1	6309/367830	0.999	20952	0.835	0.029367	[illegible]
HIP_20953	CTSICSLYQ	YFPNPYNQE	CTSICSLYQYFPNPYNQE		SCG2	1912/334890	1.000	20953	0.835	-0.108173	[illegible]
HIP_20954	LYQLENYCN	KPQNPGEDA	LYQLENYCNKPQNPGEDA		NPY	887/49410	0.995	20954	0.835	0.099485	[illegible]
HIP_20955	LVCGERGFF	NFLVHSSNN	LVCGERGFFNFLVHSSNN		IAPP	218/45018	0.999	20955	0.835	-0.008716	[illegible]
HIP_20956	DLQVGQVEL	WMRIILEAL	DLQVGQVELWMRIILEAL		SCG2	16743/334890	0.997	20956	0.835	0.008387	[illegible]
HIP_20957	SHLVEALYL	QGDLQDTKE	SHLVEALYLQGDLQDTKE		SCG1	2854/367830	1.000	20957	0.835	0.204735	[illegible]
HIP_20958	GSHLVEALY	EEGPTVVLN	GSHLVEALYEEGPTVVLN		CMGA	835/247050	1.000	20958	0.835	0.163198	[illegible]
HIP_20959	LYQLENYCN	PEYGEEKG	LYQLENYCNPEYGEEKG		SCG1	4153/367830	0.999	20959	0.835	-0.029522	[illegible]
HIP_20960	VCGERGFF	GEEDIQGPT	VCGERGFFGEEDIQGPT		SCG1	2351/367830	1.000	20960	0.835	0.063629	[illegible]
HIP_20961	CGERGFFY	LPSQKYPGP	CGERGFFYLPSQKYPGP		CMGA	5083/247050	0.998	20961	0.835	0.184362	[illegible]
HIP_20962	LVEALYLVC	QSSQAELKE	LVEALYLVCQSSQAELKE		CMGA	3099/247050	0.999	20962	0.835	0.207731	[illegible]
HIP_20963	TSICSLYQL	AAGSGRLQN	TSICSLYQLAAGSGRLQN		SCG2	5237/334890	1.000	20963	0.835	-0.029009	[illegible]
HIP_20964	CSLYQLENY	YRKKSAEFP	CSLYQLENYYRKKSAEFP		SCG1	33431/367830	0.952	20964	0.835	0.032194	[illegible]
HIP_20965	GSHLVEALY	FYDSEEPVS	GSHLVEALYFYDSEEPVS		SCG1	6483/367830	0.999	20965	0.835	-0.008427	[illegible]
HIP_20966	CGERGFFYT	ARPQALPEP	CGERGFFYTARPQALPEP		CMGA	10727/247050	0.994	20966	0.835	0.079716	[illegible]
HIP_20967	VEALYLVC	EWERYRGR	VEALYLVCEWERYRGR		SCG1	11503/367830	0.997	20967	0.835	0.109177	[illegible]
HIP_20968	CCTSICSLY	DERILSILR	CCTSICSLYDERILSILR		CMGA	10867/247050	0.994	20968	0.835	0.031810	[illegible]
HIP_20969	CGERGFFY	EEGAPGKW	CGERGFFYEEGAPGKW		SCG1	7163/367830	0.999	20969	0.835	0.180673	[illegible]
HIP_20970	SHLVEALYL	DASEAHESS	SHLVEALYLDASEAHESS		SCG1	3977/367830	0.999	20970	0.835	0.162051	[illegible]
HIP_20971	LVCGERGFF	NAVEVLKRE	LVCGERGFFNAVEVLKRE		IAPP	254/45018	0.999	20971	0.835	0.006781	[illegible]
HIP_20972	LVCGERGFF	PYGAGRSR	LVCGERGFFPYGAGRSR		SCG2	7998/334890	0.999	20972	0.835	0.077045	[illegible]
HIP_20973	GAGSLQPL	EWERYRGRG	GAGSLQPLEWERYRGRG		SCG1	17481/367830	0.993	20973	0.835	-0.030287	[illegible]
HIP_20974	VCGERGFFY	KPNGSVEP	VCGERGFFYKPNGSVEP		SCG2	13305/334890	0.998	20974	0.835	0.204628	[illegible]
HIP_20975	ERGFFYTPK	GEESEEERG	ERGFFYTPKGEESEEERG		SCG1	5557/367830	0.999	20975	0.835	-0.118251	[illegible]
HIP_20976	GSHLVEALY	PPKNDDTPN	GSHLVEALYPPKNDDTPN		SCG2	5632/334890	0.999	20976	0.835	0.188413	[illegible]
HIP_20977	AGSLQPLAL	LLGAVGLAA	AGSLQPLALLLGAVGLAA		SCG1	1339/367830	1.000	20977	0.835	0.049369	[illegible]
HIP_20978	VCGERGFF	YNQEKVLPR	VCGERGFFYNQEKVLPR		SCG2	15549/334890	0.998	20978	0.835	0.067865	[illegible]
HIP_20979	CGERGFFYT	DFYDSEEPV	CGERGFFYTDFYDSEEPV		SCG1	8631/367830	0.998	20979	0.835	-0.020031	[illegible]
HIP_20980	VEALYLVC	ASLGEKRDH	VEALYLVCASLGEKRDH		SCG1	8285/367830	0.998	20980	0.835	0.100310	[illegible]
HIP_20981	CGERGFFY	QWKSSHFER	CGERGFFYQWKSSHFER		SCG1	23221/367830	0.984	20981	0.835	0.163804	[illegible]
HIP_20982	CGERGFFY	SNVSMASLG	CGERGFFYSNVSMASLG		SCG1	8357/367830	0.998	20982	0.835	0.166486	[illegible]
HIP_20983	CTSICSLYQ	SEVDKRRTR	CTSICSLYQSEVDKRRTR		SCG1	24089/367830	0.982	20983	0.835	0.072877	[illegible]
[illegible]	[illegible]	[illegible]	[illegible]	[illegible]	[illegible]	[illegible]	[illegible]	[illegible]	[illegible]	[illegible]	[illegible]

HIP_21452	GGGPGAGSL	TQQWPERKL	GGGPGAGSLTQQWPERKL		SCG2	12929/334890	0.998	21452	0.835
HIP_21453	HLVEALYLV	IEMLKTGEK	HLVEALYLVIEMLKTGEK		SCG2	6891/334890	0.999	21453	0.835
HIP_21454	LVEALYLVC	EAELEKVAH	LVEALYLVCEAELEKVAH		CMGA	3284/247050	0.999	21454	0.835
HIP_21455	HLVEALYL	DLELQKIA	HLVEALYLDLELQKIA		SCG1	7824/367830	0.998	21455	0.835
HIP_21456	HLVEALYLV	GAWKELDR	HLVEALYLVGAWKELDR		SCG1	2512/367830	1.000	21456	0.835
HIP_21457	GSHLVEALY	THQEAENEK	GSHLVEALYTHQEAENEK		SCG1	11458/367830	0.997	21457	0.835
HIP_21458	CCTSICSLY	FQRNQLLQK	CCTSICSLYFQRNQLLQK		SCG2	20709/334890	0.996	21458	0.835
HIP_21459	HLVEALYLV	PLQWKSSH	HLVEALYLVPLQWKSSH		SCG1	9577/367830	0.998	21459	0.835
HIP_21460	VEALYLVCG	YEENSRDNP	VEALYLVCGYEENSRDNP		SCG2	7893/334890	0.999	21460	0.835
HIP_21461	AGSLQPLAL	EEEEGENYQ	AGSLQPLALEEEEGENYQ		SCG1	3852/367830	0.999	21461	0.835
HIP_21462	LGGGPGAGS	PNPYNQEKV	LGGGPGAGSPNPYNQEKV		SCG2	1478/334890	1.000	21462	0.835
HIP_21463	GERGFFYT	EEPEYGEEI	GERGFFYTEEPEYGEEI		SCG1	6471/367830	0.999	21463	0.835
HIP_21464	SHLVEALYL	FPDFYDSE	SHLVEALYLFPDFYDSE		SCG1	3629/367830	0.999	21464	0.835
HIP_21465	CGERGFFYT	EEKGHPQE	CGERGFFYTEEKGHPQE		SCG1	18812/367830	0.991	21465	0.835
HIP_21466	HLVEALYLV	APVSKRFPV	HLVEALYLVAPVSKRFPV		SCG2	5921/334890	0.999	21466	0.835
HIP_21467	TSICSLYQL	LLMKVLEYL	TSICSLYQLLLMKVLEYL		SCG2	3989/334890	1.000	21467	0.835
HIP_21468	AGSLQPLAL	QDKQYSSHH	AGSLQPLALQDKQYSSHH		SCG1	18102/367830	0.992	21468	0.835
HIP_21469	CSLYQLENY	EYLNQEKAE	CSLYQLENYEYLNQEKAE		SCG2	7771/334890	0.999	21469	0.835
HIP_21470	LVEALYLVC	EEEPEYGEE	LVEALYLVCEEEPEYGEE		SCG1	2880/367830	1.000	21470	0.835
HIP_21471	AGSLQPLAL	YPGPQAEGD	AGSLQPLALYPGPQAEGD		CMGA	46/247050	1.000	21471	0.835
HIP_21472	VEALYLVC	ESSSRGEAG	VEALYLVCESSSRGEAG		SCG1	5523/367830	0.999	21472	0.835
HIP_21473	QHLCGSHLV	RGWRPSSR	QHLCGSHLVRGWRPSSR		CMGA	14957/247050	0.987	21473	0.835
HIP_21474	ICSLYQLEN	AYLKRLVNA	ICSLYQLENAYLKRLVNA		SCG2	5444/334890	0.999	21474	0.835
HIP_21475	TSICSLYQL	PNNQKRER	TSICSLYQLPNNQKRER		SCG2	15048/334890	0.998	21475	0.835
HIP_21476	CGERGFFYT	KDVMEKRED	CGERGFFYTKDVMEKRED		CMGA	13172/247050	0.990	21476	0.835
HIP_21477	CGERGFFY	PQALPEPMQ	CGERGFFYPQALPEPMQ		CMGA	6943/247050	0.997	21477	0.835
HIP_21478	SHLVEALYL	GILKLQVFL	SHLVEALYLGILKLQVFL		IAPP	435/45018	0.999	21478	0.835
HIP_21479	SHLVEALYL	SPMPVSQEC	SHLVEALYLSPMPVSQEC		CMGA	386/247050	1.000	21479	0.835
HIP_21480	GGGPGAGSL	NWGYEKRNL	GGGPGAGSLNWGYEKRNL		SCG1	6377/367830	0.999	21480	0.835
HIP_21481	LVCGERGFF	EKDRADQT	LVCGERGFFEKDRADQT		SCG1	15689/367830	0.994	21481	0.835
HIP_21482	LVCGERGFF	YYDPLQWKS	LVCGERGFFYYDPLQWKS		SCG1	13164/367830	0.996	21482	0.835
HIP_21483	PGAGSLQPL	QEEEEDNRD	PGAGSLQPLQEEEEDNRD		CMGA	2461/247050	0.999	21483	0.835
HIP_21484	GSLQPLALE	RQYWDEDLL	GSLQPLALERQYWDEDLL		SCG2	20412/334890	0.996	21484	0.835
HIP_21485	LVEALYLVC	SSHFERRD	LVEALYLVCSSHFERRD		SCG1	10768/367830	0.997	21485	0.835
HIP_21486	GSHLVEALY	GERGEDSSE	GSHLVEALYGERGEDSSE		SCG1	6446/367830	0.999	21486	0.835
HIP_21487	CGERGFFY	TAEKRKRL	CGERGFFYTAEKRKRL		SCG1	21701/367830	0.987	21487	0.835
HIP_21488	TSICSLYQL	AASFQRNQ	TSICSLYQLAASFQRNQ		SCG2	10188/334890	0.999	21488	0.835
HIP_21489	LVCGERGFF	KRDHHSTHY	LVCGERGFFKRDHHSTHY		SCG1	26445/367830	0.976	21489	0.835
HIP_21490	LVEALYLVC	EEEAEAGEE	LVEALYLVCEEEAEAGEE		CMGA	1646/247050	1.000	21490	0.835
HIP_21491	GSHLVEALY	ESSSRGEAG	GSHLVEALYESSSRGEAG		SCG1	3139/367830	1.000	21491	0.835
HIP_21492	NQHLCGSHL	SQEKTHSRE	NQHLCGSHLSQEKTHSRE		SCG1	19885/367830	0.990	21492	0.835
HIP_21493	GSLQPLAL	EDLQVGQV	GSLQPLALEDLQVGQV		INSC	78/5184	0.999	21493	0.835
HIP_21494	GSLQPLAL	WKELDRNYL	GSLQPLALWKELDRNYL		SCG1	20730/367830	0.988	21494	0.835
HIP_21495	LVCGERGFF	EGGGHSRE	LVCGERGFFEGGGHSRE		SCG1	160/367830	1.000	21495	0.835
HIP_21496	ERGFFYTPK	KELTAEKRL	ERGFFYTPKKELTAEKRL		CMGA	15111/247050	0.987	21496	0.835
HIP_21497	TSICSLYQL	LQVFLIVLS	TSICSLYQLLQVFLIVLS		IAPP	768/45018	0.997	21497	0.835
HIP_21498	GSHLVEALY	ESSPDYNPY	GSHLVEALYESSPDYNPY		SCG2	1947/334890	1.000	21498	0.835
HIP_21499	YLVCGERGF	ALAEAYPSK	YLVCGERGFALAEAYPSK		NPY	103/49410	1.000	21499	0.835
HIP_21500	CTSICSLYQ	EKDRADQTV	CTSICSLYQEKDRADQTV		SCG1	20788/367830	0.988	21500	0.835
HIP_21501	QCCTSICS	EYNYDWWEK	QCCTSICSEYNYDWWEK		SCG1	14652/367830	0.995	21501	0.835
HIP_21502	RGFFYTPKT	QKTSYFPNP	RGFFYTPKTQKTSYFPNP		SCG2	26198/334890	0.993	21502	0.835
HIP_21503	RGFFYTPKT	ALAEAYPSK	RGFFYTPKTALAEAYPSK		NPY	895/49410	0.995	21503	0.835
HIP_21504	LVCGERGFF	THAAGHSQE	LVCGERGFFTHAAGHSQE		SCG1	9500/367830	0.998	21504	0.835
HIP_21505	GERGFFYT	HESSSRGEA	GERGFFYTHESSSRGEA		SCG1	17285/367830	0.993	21505	0.835
HIP_21506	SHLVEALYL	LDQLLHYR	SHLVEALYLLDQLLHYR		SCG1	10790/367830	0.997	21506	0.835
HIP_21507	CGERGFFY	QMDKARRH	CGERGFFYQMDKARRH		SCG1	20537/367830	0.989	21507	0.835

HIP_21963	CCTSICSLY	SQENHPQES	CCTSICSLYSQENHPQES		SCG1	6056/367830	0.999	21963	0.834	0.056718	-1.0167	0.3333	-1	18	-0.65	-2	1	0	0
HIP_21964	YLVCGERGF	RSNQLPKAA	YLVCGERGFRSNQLPKAA		SCG2	20202/334890	0.996	21964	0.834	0.075843	-1.8167	0.3333	2	18	-0.4	2	1	0	0
HIP_21965	GERGFFYT	GKWQQQGDL	GERGFFYTGKWQQQGDL		SCG1	14226/367830	0.995	21965	0.834	0.089498	-0.7333	0.1667	0	17	-1.2353	0	4	1	1
HIP_21966	RGFFYTPKT	LLHYRKKSA	RGFFYTPKTLLHYRKKSA		SCG1	11685/367830	0.997	21966	0.834	0.137335	-0.3	0.1667	6	18	-0.8722	5	2	0	0
HIP_21967	GERGFFYT	DEEDKRNYP	GERGFFYTDEEDKRNYP		SCG1	14118/367830	0.995	21967	0.834	0.087243	-1.6167	0	-2	17	-2	-2	3	1	0
HIP_21968	GSHLVEALY	HSREKSSQE	GSHLVEALYHSREKSSQE		SCG1	20935/367830	0.988	21968	0.834	0.168219	-0.7	0.1667	1	18	-1.1167	-1	1	0	0
HIP_21969	ERGFFYTPK	RYYSALRHY	ERGFFYTPKRYYSALRHY		NPY	2438/49410	0.982	21969	0.834	0.091573	-2.2167	0.1667	4	18	-1.2	3	4	0	0
HIP_21970	AEDLQVGQV	KEEEGSANR	AEDLQVGQVKEEEGSANR		CMGA	6619/247050	0.998	21970	0.834	-0.042656	-1.7667	0.1667	-3	18	-1.2333	-3	0	0	1
HIP_21971	CGERGFFY	GEEENELT	CGERGFFYGEEENELT		SCG1	4419/367830	0.999	21971	0.834	0.165090	-0.5167	0.1667	-4	16	-1.05	-4	3	0	1
HIP_21972	LVEALYLVC	RMLSKSGYP	LVEALYLVCRMLSKSGYP		SCG2	16012/334890	0.997	21972	0.834	0.085826	1.95	0	1	18	0.65	1	1	0	0
HIP_21973	CTSICSLYQ	IKEHLNQGS	CTSICSLYQIKEHLNQGS		SCG2	22457/334890	0.995	21973	0.834	-0.009625	-0.65	0	1	18	-0.2389	0	1	0	0
HIP_21974	VEALYLVC	AGQVTALPV	VEALYLVCAGQVTALPV		CMGA	3213/247050	0.999	21974	0.834	0.201593	1.4	0.1667	-1	17	1.4765	-1	1	1	0
HIP_21975	AGSLQPLAL	REKGLSAEP	AGSLQPLALREKGLSAEP		CMGA	14627/247050	0.988	21975	0.834	0.230235	-0.4167	0	0	18	-0.2167	0	0	0	0
HIP_21976	GERGFFYTP	SNQLPKAAW	GERGFFYTPSNQLPKAAW		SCG2	12511/334890	0.998	21976	0.834	-0.027650	-1.9	0.3333	1	18	-0.7556	1	3	0	0
HIP_21977	GERGFFYTP	NSPMNKGDT	GERGFFYTPNSPMNKGDT		CMGA	4122/247050	0.999	21977	0.834	-0.043491	-1.5833	0.5	0	18	-1.2667	0	3	0	0
HIP_21978	ICSLYQLEN	EESSPDYNP	ICSLYQLENEESSPDYNP		SCG2	10363/334890	0.999	21978	0.834	-0.000761	-1.8333	0.1667	-4	18	-1.0056	-4	1	0	0
HIP_21979	AGSLQPLAL	EGNNQAPGE	AGSLQPLALEGNNQAPGE		CMGA	1641/247050	1.000	21979	0.834	0.204677	0.3333	0.1667	-2	18	-0.5222	-2	0	0	0
HIP_21980	GAGSLQPLA	FRARAYGFR	GAGSLQPLAFRARAYGFR		CMGA	11682/247050	0.993	21980	0.834	0.010899	0.6833	0.1667	3	18	-0.0833	3	1	0	0
HIP_21981	CGERGFFYT	EDDIYKANN	CGERGFFYTEDDIYKANN		SCG2	11391/334890	0.999	21981	0.834	0.075337	-1.6167	0	-2	18	-1.0611	-2	3	0	0
HIP_21982	QCCTSICSL	PGKGRHHRG	QCCTSICSLPGKGRHHRG		SCG1	15957/367830	0.994	21982	0.834	0.205354	-0.0667	0.5	5	18	-0.6722	3	0	0	0
HIP_21983	SHLVEALY	FLVHSSNNF	SHLVEALYFLVHSSNNF		IAPP	978/45018	0.996	21983	0.834	-0.030298	2.5167	0	1	17	0.3882	-1	2	1	0
HIP_21984	LGGGPGAGS	NKPYALNSE	LGGGPGAGSNKPYALNSE		SCG2	10594/334890	0.999	21984	0.834	0.001581	-1.4	0.5	0	18	-0.6278	0	1	0	1
HIP_21985	QHLCGSHLV	NIAYEDVVG	QHLCGSHLVNIAYEDVVG		SCG2	5980/334890	0.999	21985	0.834	0.023997	1.2667	0	0	18	0.3167	-2	1	0	0
HIP_21986	FVNQHLCGS	PYGAGRSRS	FVNQHLCGSPYGAGRSRS		SCG2	5503/334890	0.999	21986	0.834	-0.035189	-0.3333	0.6667	3	18	-0.5889	2	1	0	0
HIP_21987	CSLYQLENY	NEQINDEMK	CSLYQLENYNEQINDEMK		SCG2	15509/334890	0.998	21987	0.834	-0.090514	-3.1333	0	-3	18	-1.2389	-3	1	0	0
HIP_21988	NQHLCGSHL	NRQYWDEDL	NQHLCGSHLNRQYWDEDL		SCG2	21001/334890	0.996	21988	0.834	0.121627	-1.95	0.1667	0	18	-1.3833	-2	1	0	0
HIP_21989	GSLQPLAL	YFMSDTREE	GSLQPLALYFMSDTREE		SCG1	9130/367830	0.998	21989	0.834	0.017762	2.1333	0	-2	17	-0.3647	-2	2	1	0
HIP_21990	HLCGSHLVE	EISRNLQIP	HLCGSHLVEEISRNLQIP		SCG2	14900/334890	0.998	21990	0.834	0.079662	0.7833	0.1667	1	18	-0.0778	-1	0	0	0
HIP_21991	GSHLVEALY	PSPEMIRAL	GSHLVEALYPSPEMIRAL		SCG2	1066/334890	1.000	21991	0.834	0.186968	0.05	0.5	0	18	0.2444	-1	1	0	0
HIP_21992	TSICSLYQL	SKRFPVGPP	TSICSLYQLSKRFPVGPP		SCG2	24658/334890	0.994	21992	0.834	0.188511	-1.7	0.1667	2	18	0.0056	2	2	0	0
HIP_21993	LVEALYLVC	VMEKREDSK	LVEALYLVCVMEKREDSK		CMGA	5638/247050	0.998	21993	0.834	0.027383	2.1833	0	-1	18	0.1	-1	1	0	0
HIP_21994	AGSLQPLAL	FGAILSSTN	AGSLQPLALFGAILSSTN		IAPP	373/45018	0.999	21994	0.834	0.060654	2.2667	0.1667	0	18	0.8556	0	1	0	0
HIP_21995	LYQLENYCN	LSFRARAYG	LYQLENYCNLSFRARAYG		CMGA	5730/247050	0.998	21995	0.834	-0.094283	0.5833	0.1667	1	18	-0.4333	1	2	0	0
HIP_21996	VCGERGFFY	KQYSSHHT	VCGERGFFYKQYSSHHT		SCG1	24652/367830	0.981	21996	0.834	0.159208	-0.7333	0	3	17	-0.8941	1	4	1	0
HIP_21997	GIVEQCCTS	ETQQWPERK	GIVEQCCTSETQQWPERK		SCG2	14386/334890	0.998	21997	0.834	-0.019370	-1.1167	0.1667	-1	18	-1.1556	-1	0	0	0
HIP_21998	LVEALYLVC	QAENEPQSA	LVEALYLVCQAENEPQSA		SCG2	4942/334890	1.000	21998	0.834	0.106879	0.8833	0	-3	18	0.1667	-3	1	0	0
HIP_21999	HLCGSHLVE	GLEPGKGRH	HLCGSHLVEGLEPGKGRH		SCG1	13799/367830	0.996	21999	0.834	0.114462	0.7333	0.1667	3	18	-0.6056	0	0	0	0
HIP_22000	TSICSLYQL	FLIVLSVA	TSICSLYQLFLIVLSVA		IAPP	588/45018	0.998	22000	0.834	-0.031695	1.6833	0	0	17	1.8706	0	2	1	0
HIP_22001	LVCGERGFF	GPGPQLRRG	LVCGERGFFGPGPQLRRG		CMGA	632/247050	1.000	22001	0.834	0.165538	0.4667	0.6667	2	18	-0.3222	2	2	0	0
HIP_22002	AGSLQPLAL	DVMEKREDS	AGSLQPLALDVMEKREDS		CMGA	5471/247050	0.998	22002	0.834	0.041806	2	0	-2	18	-0.4667	-2	0	0	0
HIP_22003	CGERGFFYT	AGSGRLQNG	CGERGFFYTAGSGRLQNG		SCG2	1248/334890	1.000	22003	0.834	0.075013	0.2333	0.3333	1	18	-0.5889	1	3	0	0
HIP_22004	LVCGERGFF	NERKQASA	LVCGERGFFNERKQASA		SCG1	20853/367830	0.988	22004	0.834	0.127503	-1.05	0.1667	1	17	-0.5176	1	2	1	0
HIP_22005	LVCGERGFF	VPKLDLKRQ	LVCGERGFFVPKLDLKRQ		SCG1	11115/367830	0.997	22005	0.834	0.019245	0.65	0.3333	2	18	-0.1	2	2	0	0
HIP_22006	LVCGERGFF	RFLGEGHHR	LVCGERGFFRFLGEGHHR		SCG1	14583/367830	0.995	22006	0.834	0.051153	1.2167	0.1667	3	18	-0.3222	1	3	0	0
HIP_22007	ICSLYQLEN	KRNYPSLEL	ICSLYQLENKRNYPSLEL		SCG1	29854/367830	0.966	22007	0.834	0.088547	-2.5167	0	0	18	-0.5278	0	2	0	0
HIP_22008	EQCCTSICS	NPFKRTNEI	EQCCTSICSNPFKRTNEI		SCG2	5587/334890	0.999	22008	0.834	0.053805	0.65	0.3333	0	18	-0.6222	0	1	0	0
HIP_22009	SICSLYQLE	KTHWLGAAL	SICSLYQLEKTHWLGAAL		SCG2	26187/334890	0.993	22009	0.834	-0.027886	-1.8333	0	1	18	0.3778	0	2	0	0
HIP_22010	SHLVEALYL	LSKSSAPPI	SHLVEALYLLSKSSAPPI		SCG1	15034/367830	0.995	22010	0.834	0.205949	0.9	0.1667	1	18	0.5111	0	1	0	0
HIP_22011	CSLYQLENY	ENQSSQAEL	CSLYQLENYENQSSQAEL		CMGA	11574/247050	0.993	22011	0.834	-0.035769	-3.1333	0	-3	18	-0.9611	-3	1	0	0
HIP_22012	HLVEALYLV	SYFPNPYN	HLVEALYLVSYFPNPYN		SCG2	9425/334890	0.999	22012	0.834	0.081290	1.2333	0.1667	0	17	0.1647	-1	3	1	0
HIP_22013	VCGERGFFY	DSEGLSQG	VCGERGFFYDSEGLSQG		CMGA	6341/247050	0.998	22013	0.834	0.175099	-0.5833	0.1667	-2	17	-0.4059	-2	3	1	0
HIP_22014	SHLVEALYL	ENQMDKAR	SHLVEALYLENQMDKAR		SCG1	12817/367830	0.996	22014	0.834	0.220124	-0.7	0	0	17	-0.5941	-1	1	1	0
HIP_22015	HLCGSHLVE	EEEEEMAVV	HLCGSHLVEEEEEEMAVV		CMGA	3513/247050	0.999	22015	0.834	0.042768	-1	0	-4	18	-0.1222	-6	0	0	4
HIP_22016	CSLYQLENY	MYEENSRDN	CSLYQLENYMYEENSRDN		SCG2	8943/334890	0.999	22016	0.834	-0.097709	-1.8667	0	-3	18	-1.4444	-3	2	0	0
HIP_22017	QHLCGSHLV	SYFPNPYN	QHLCGSHLVSYFPNPYN		SCG2	14292/334890	0.998	22017	0.834	0.110917	0.9167	0.1667	2	17	-0.4471	0	2	1	0
HIP_22018	SHLVEALYL	EEEEEEAT	SHLVEALYLEEEEEEAT		CMGA	2779/247050	0.999	22018	0.834	0.122234	-0.7	0	-6	17	-0.8647	-7	1	1	4

HIP_22474	CGERGFFY	ELQKIAEK	CGERGFFYELQKIAEK		SCG1	16216/367830	0.994	22474	0.834	0.142212	0.1833	0	0	16	-0.6375	0	3	0	0
HIP_22475	LCGSHLVEA	QVKRVPGQG	LCGSHLVEAQVKRVPGQG		SCG2	30347/334890	0.990	22475	0.834	0.125128	-0.1167	0	2	18	-0.0667	1	0	0	0
HIP_22476	CGERGFFY	GQGSSEDD	CGERGFFYGQGSSEDD		SCG2	2447/334890	1.000	22476	0.834	0.180947	0	0.3333	-3	16	-1.15	-3	3	0	0
HIP_22477	VNQHLCGSH	WWEKIPFSE	VNQHLCGSHWWEKIPFSE		SCG1	5648/367830	0.999	22477	0.834	0.025113	-1.6167	0.3333	2	18	-1.1278	0	2	0	0
HIP_22478	GERGFFYTP	KELDRNYLN	GERGFFYTPKELDRNYLN		SCG1	17333/367830	0.993	22478	0.834	-0.073668	-1.2	0.1667	0	18	-1.2722	0	3	0	0
HIP_22479	LVCGERGFF	QEEEEDNR	LVCGERGFFQEEEEDNR		CMGA	5376/247050	0.998	22479	0.834	0.049114	-0.8833	0.1667	-4	17	-1.2765	-4	2	1	2
HIP_22480	VCGERGFFY	LPDGLSVED	VCGERGFFYLPDGLSVED		SCG2	614/334890	1.000	22480	0.834	0.089827	0.5	0.1667	-3	18	0.0389	-3	3	0	0
HIP_22481	TSICSLYQ	NPFKRTNEI	TSICSLYQNPFKRTNEI		SCG2	12547/334890	0.998	22481	0.834	0.082429	-0.55	0.1667	1	17	-0.6	1	2	1	0
HIP_22482	CGERGFFY	ENEKDRAD	CGERGFFYENEKDRAD		SCG1	11098/367830	0.997	22482	0.834	0.155896	-1.0333	0	-2	16	-1.6312	-2	3	0	0
HIP_22483	GERGFFYT	EGEEDATSE	GERGFFYTEGEEDATSE		SCG1	4075/367830	0.999	22483	0.834	0.095667	-1.1	0.1667	-5	17	-1.3412	-5	3	1	0
HIP_22484	GERGFFYT	EEGEEDATS	GERGFFYTEEGEEDATS		SCG1	4076/367830	0.999	22484	0.834	0.095667	-1.1	0.1667	-5	17	-1.3412	-5	3	1	0
HIP_22485	CGERGFFY	QENQMDKA	CGERGFFYQENQMDKA		SCG1	13034/367830	0.996	22485	0.834	0.180879	-1.0333	0	-1	16	-1.2312	-1	3	0	0
HIP_22486	AGSLQPLAL	SREKSSQES	AGSLQPLALSREKSSQES		SCG1	14633/367830	0.995	22486	0.834	0.247357	0.1	0.1667	0	18	-0.7444	0	0	0	0
HIP_22487	SHLVEALYL	SQENHPQE	SHLVEALYLSQENHPQE		SCG1	9060/367830	0.998	22487	0.834	0.223999	-0.25	0.1667	-1	17	-0.8529	-3	1	1	0
HIP_22488	VCGERGFFY	DLFQNRML	VCGERGFFYDLFQNRML		SCG2	12515/334890	0.998	22488	0.834	0.047691	1.2333	0	0	17	-0.0294	0	4	1	0
HIP_22489	GSHLVEALY	PEYNYDWW	GSHLVEALYPEYNYDWW		SCG1	6102/367830	0.999	22489	0.834	0.103966	-0.35	0.1667	-2	17	-0.7118	-3	2	1	0
HIP_22490	LVCGERGFF	GHSRERAD	LVCGERGFFGHSRERAD		SCG1	9426/367830	0.998	22490	0.834	0.094231	0.1333	0.5	1	17	-0.6647	0	2	1	0
HIP_22491	GSLQPLAL	YPGPQAEG	GSLQPLALYPGPQAEG		CMGA	1474/247050	1.000	22491	0.834	0.209395	1.0167	0.3333	-1	16	-0.225	-1	1	0	0
HIP_22492	HLVEALYL	APGKWQQQG	HLVEALYLAPGKWQQQG		SCG1	1598/367830	1.000	22492	0.834	0.186562	1.0167	0.3333	1	17	-0.3824	0	2	1	1
HIP_22493	EQCCTSICS	EEEGPTVVL	EQCCTSICSEEEGPTVVL		CMGA	3324/247050	0.999	22493	0.834	0.022132	-0.7167	0.1667	-4	18	0.0944	-4	0	0	1
HIP_22494	LVEALYLVC	RPQALPEPM	LVEALYLVCRPQALPEPM		CMGA	8571/247050	0.996	22494	0.834	0.194576	0.15	0.1667	-1	18	0.5833	-1	1	0	0
HIP_22495	CTSICSLYQ	PEPMQESKA	CTSICSLYQPEPMQESKA		CMGA	1010/247050	1.000	22495	0.834	0.116711	-1.2833	0.3333	-1	18	-0.4722	-1	1	0	0
HIP_22496	CTSICSLYQ	TKENREEAR	CTSICSLYQTKENREEAR		SCG1	26123/367830	0.977	22496	0.834	0.183528	-1.5167	0	0	18	-1.0889	0	1	0	0
HIP_22497	GSHLVEALY	PKAAWIPHV	GSHLVEALYPKAAWIPHV		SCG2	11293/334890	0.999	22497	0.834	0.144557	0.1	0.1667	2	18	0.3056	0	1	0	0
HIP_22498	ERGFFYTPK	PTKADTEKW	ERGFFYTPKPTKADTEKW		SCG1	17303/367830	0.993	22498	0.834	-0.088880	-2.0667	0.3333	1	18	-1.5111	1	2	0	0
HIP_22499	AGSLQPLAL	GKSGELEQE	AGSLQPLALGKSGELEQE		CMGA	4507/247050	0.999	22499	0.834	0.219305	0.7167	0.3333	-2	18	-0.3889	-2	0	0	0
HIP_22500	VCGERGFFY	GHPQEESE	VCGERGFFYGHPQEESE		SCG1	5401/367830	0.999	22500	0.834	0.149701	-0.15	0.3333	-2	17	-1.0471	-3	3	1	0
HIP_22501	LCGSHLVEA	WDEDLLMKV	LCGSHLVEAWDEDLLMKV		SCG2	14048/334890	0.998	22501	0.834	0.074853	-0.9	0	-2	18	0.3667	-3	1	0	0
HIP_22502	SHLVEALYL	EGKGEQEHS	SHLVEALYLEGKGEQEHS		CMGA	6153/247050	0.998	22502	0.834	0.236153	-0.25	0.1667	-1	18	-0.7833	-3	1	0	0
HIP_22503	AGSLQPLAL	VRLLRDPAD	AGSLQPLALVRLLRDPAD		SCG1	12397/367830	0.997	22503	0.834	0.037972	2.15	0	0	18	0.2611	0	0	0	0
HIP_22504	LVEALYLVC	SEDVNWGYE	LVEALYLVCSEDVNWGYE		SCG1	2526/367830	1.000	22504	0.834	0.132912	0.45	0.1667	-4	18	0.3389	-4	1	0	0
HIP_22505	HLVEALYLV	QYTPQSLA	HLVEALYLVQYTPQSLA		SCG2	13283/334890	0.998	22505	0.834	0.144629	0.2	0	0	17	0.4588	-1	2	1	0
HIP_22506	ERGFFYTPK	SPETLISDL	ERGFFYTPKSPETLISDL		NPY	1189/49410	0.994	22506	0.834	-0.114706	-2.0167	0.5	-1	18	-0.5056	-1	2	0	0
HIP_22507	LCGSHLVEA	NIEKNEQIN	LCGSHLVEANIEKNEQIN		SCG2	16802/334890	0.997	22507	0.834	0.069344	0	0	-1	18	-0.4278	-2	0	0	0
HIP_22508	LVEALYLVC	ALNHLKAT	LVEALYLVCALNHLKAT		IAPP	1302/45018	0.995	22508	0.834	0.057603	2.1	0	1	17	1.1294	0	1	1	0
HIP_22509	SICSLYQLE	QRNQLLQKE	SICSLYQLEQRNQLLQKE		SCG2	26208/334890	0.993	22509	0.834	0.003720	-2.45	0	0	18	-0.7556	0	1	0	0
HIP_22510	SHLVEALYL	NFPMDMSD	SHLVEALYLNFPMDMSD		SCG2	3362/334890	1.000	22510	0.834	0.149108	0.6667	0.1667	-2	17	0.1353	-3	2	1	0
HIP_22511	VELGGGPGA	SNTYGKRNA	VELGGGPGASNTYGKRNA		IAPP	631/45018	0.998	22511	0.834	-0.021657	-0.8667	0.5	1	18	-0.7611	1	1	0	1
HIP_22512	ICSLYQL	FLVHSSN	ICSLYQLFLVHSSN		IAPP	796/45018	0.997	22512	0.834	-0.018982	1.6333	0	1	14	0.8214	0	2	0	0
HIP_22513	VCGERGFF	NWGYEKRNL	VCGERGFFNWGYEKRNL		SCG1	11631/367830	0.997	22513	0.834	0.070145	0.0667	0.3333	1	17	-0.8353	1	4	1	0
HIP_22514	LVCGERGFF	NWGYEKRN	LVCGERGFFNWGYEKRN		SCG1	11632/367830	0.997	22514	0.834	0.070145	0.0667	0.3333	1	17	-0.8353	1	4	1	0
HIP_22515	GSHLVEALY	PQEESEESN	GSHLVEALYPQEESEESN		SCG1	1416/367830	1.000	22515	0.834	0.142194	-0.7167	0.1667	-4	18	-1.1	-5	1	0	0
HIP_22516	LVCGERGFF	ESKGQPRS	LVCGERGFFESKGQPRS		SCG1	19088/367830	0.991	22516	0.834	0.142418	-0.5	0.3333	1	17	-0.6882	1	2	1	0

HIP_22985	HLVEALYL	EDNRDSSMK	HLVEALYLEDNRDSSMK		CMGA	6414/247050	0.998	22985	0.833	0.164006	-0.7	0	-1	17	-0.7471	-2
HIP_22986	CCTSICSLY	QEESEEGEE	CCTSICSLYQEESEEGEE		SCG1	4063/367830	0.999	22986	0.833	-0.023252	-1.4667	0.1667	-6	18	-0.75	-6
HIP_22987	SHLVEALYL	LQVRGYPEE	SHLVEALYLLQVRGYPEE		CMGA	4793/247050	0.998	22987	0.833	0.022193	1.8	0	-1	18	-0.0944	-2
HIP_22988	VCGERGFF	GEEDATSEV	VCGERGFFGEEDATSEV		SCG1	2277/367830	1.000	22988	0.833	0.065744	-0.3667	0.3333	-4	17	-0.3765	-4
HIP_22989	CTSICSLYQ	VDKRRTRPR	CTSICSLYQVDKRRTRPR		SCG1	34077/367830	0.949	22989	0.833	0.070569	-0.7	0	4	18	-0.9611	4
HIP_22990	TSICSLYQL	PGPQLRRGW	TSICSLYQLPGPQLRRGW		CMGA	2251/247050	0.999	22990	0.833	0.171973	-0.7667	0.5	2	18	-0.3389	2
HIP_22991	QHLCGSHLV	RMLSKSGYP	QHLCGSHLVRMLSKSGYP		SCG2	24124/334890	0.994	22991	0.833	0.100619	1	0	4	18	-0.2444	2
HIP_22992	GERGFFYTP	GLFRGGKSG	GERGFFYTPGLFRGGKSG		CMGA	3654/247050	0.999	22992	0.833	-0.072647	0.4333	0.3333	2	18	-0.6111	2
HIP_22993	ERGFFYTPK	QAELKEAVE	ERGFFYTPKQAELKEAVE		CMGA	7144/247050	0.997	22993	0.833	-0.098665	-1.9	0.1667	-1	18	-0.9222	-1
HIP_22994	CGERGFFYT	RPQALPEPM	CGERGFFYTRPQALPEPM		CMGA	12236/247050	0.992	22994	0.833	0.142363	-1.4667	0.1667	0	18	-0.6389	0
HIP_22995	CGSHLVEAL	EEQKLYTDD	CGSHLVEALEEQKLYTDD		SCG2	9717/334890	0.999	22995	0.833	0.068229	-1.4	0	-3	18	-0.6333	-4
HIP_22996	GGPGAGSLQ	PNPYNQEKV	GGPGAGSLQPNPYNQEKV		SCG2	5091/334890	1.000	22996	0.833	-0.029073	-1.2	0.5	0	18	-1.1167	0
HIP_22997	RGFFYTPKT	NQMDKARRH	RGFFYTPKTNQMDKARRH		SCG1	24426/367830	0.981	22997	0.833	0.067120	-1.8833	0.1667	5	18	-1.6889	4
HIP_22998	LVCGERGFF	FQNRMLSK	LVCGERGFFFQNRMLSK		SCG2	14632/334890	0.998	22998	0.833	0.063584	0.1667	0.1667	2	17	-0.0235	2
HIP_22999	PLALEGSLQ	GPNNQKRER	PLALEGSLQGPNNQKRER		SCG2	3087/334890	1.000	22999	0.833	0.011390	-1	0.5	1	18	-1.4167	1
HIP_23000	EQCCTSICS	EEEEEATNT	EQCCTSICSEEEEEATNT		CMGA	2044/247050	0.999	23000	0.833	0.005730	-0.7167	0.1667	-6	18	-0.9944	-6
HIP_23001	HLCGSHLVE	KLAPVSKRF	HLCGSHLVEKLAPVSKRF		SCG2	25634/334890	0.993	23001	0.833	0.057952	1.0333	0	4	18	0.0611	2
HIP_23002	ERGFFYTPK	SSEDDLQEE	ERGFFYTPKSSEDDLQEE		SCG2	4147/334890	1.000	23002	0.833	-0.152590	-1.8833	0.5	-4	18	-1.6167	-4
HIP_23003	GERGFFYTP	NHPQESKGQ	GERGFFYTPNHPQESKGQ		SCG1	14976/367830	0.995	23003	0.833	-0.031907	-1.9833	0.3333	1	18	-1.7056	0
HIP_23004	SHLVEALY	EEEEEMAVV	SHLVEALYEEEEEMAVV		CMGA	3607/247050	0.999	23004	0.833	0.124666	-1.0333	0	-5	17	-0.0353	-6
HIP_23005	AGSLQPLAL	ADQTVLTE	AGSLQPLALADQTVLTE		SCG1	6239/367830	0.999	23005	0.833	0.134145	0.7	0	-2	17	0.3882	-2
HIP_23006	LVEALYLVC	ALQGAKERA	LVEALYLVCALQGAKERA		CMGA	7250/247050	0.997	23006	0.833	0.049787	2.1	0	0	18	0.7056	0
HIP_23007	PGAGSLQPL	NRQYWDEDL	PGAGSLQPLNRQYWDEDL		SCG2	16957/334890	0.997	23007	0.833	0.022454	-2.1333	0.1667	-2	18	-1.0722	-2
HIP_23008	VCGERGFFY	EGQEEEED	VCGERGFFYEGQEEEED		CMGA	2036/247050	0.999	23008	0.833	0.157111	-0.5167	0.1667	-6	17	-1.3353	-6
HIP_23009	VCGERGFFY	EENSRDNP	VCGERGFFYEENSRDNP		SCG2	10327/334890	0.999	23009	0.833	0.156358	-1.0333	0	-2	17	-1.3059	-2
HIP_23010	SLYQLENYC	QQQGDLQDT	SLYQLENYCQQQGDLQDT		SCG1	9924/367830	0.998	23010	0.833	0.068806	-2.1333	0	-3	18	-1.2278	-3
HIP_23011	LVEALYLVC	VKYPEINS	LVEALYLVCVKYPEINS		SCG2	13460/334890	0.998	23011	0.833	0.058248	1.5833	0	-1	18	0.9944	-1
HIP_23012	VCGERGFFY	QNLLKELQ	VCGERGFFYQNLLKELQ		CMGA	9444/247050	0.995	23012	0.833	0.125711	0.1833	0	0	17	-0.2529	0
HIP_23013	LVEALYLVC	NAVEVLKR	LVEALYLVCNAVEVLKR		IAPP	1268/45018	0.995	23013	0.833	0.065223	2.1667	0	0	17	1.0529	0
HIP_23014	CGERGFFYT	ANQKTSYF	CGERGFFYTANQKTSYF		SCG2	19577/334890	0.996	23014	0.833	0.091582	-0.7333	0	1	17	-0.6941	1
HIP_23015	GSHLVEALY	EDSLEAGLP	GSHLVEALYEDSLEAGLP		CMGA	1731/247050	1.000	23015	0.833	0.126364	-0.5833	0.1667	-3	18	0.0278	-4
HIP_23016	LVCGERGFF	LQKIAEKFS	LVCGERGFFLQKIAEKFS		SCG1	15962/367830	0.994	23016	0.833	0.021134	0.2667	0.1667	1	18	0.2556	1
HIP_23017	SHLVEALY	PKAAWIPHV	SHLVEALYPKAAWIPHV		SCG2	16885/334890	0.997	23017	0.833	0.201865	0.1	0.1667	2	17	0.3471	0
HIP_23018	CGERGFFY	EEEEMAVV	CGERGFFYEEEEMAVV		CMGA	5353/247050	0.998	23018	0.833	0.126419	-1.0333	0	-4	16	-0.2437	-4
HIP_23019	LVEALYLVC	RTRPRHHHG	LVEALYLVCRTRPRHHHG		SCG1	25099/367830	0.980	23019	0.833	0.201512	0.1333	0	5	18	-0.3611	2
HIP_23020	LCGSHLVEA	GYPKTPGRA	LCGSHLVEAGYPKTPGRA		SCG2	11149/334890	0.999	23020	0.833	0.174241	-0.1333	0.3333	2	18	-0.2444	1
HIP_23021	GERGFFYTP	QASAIKKEE	GERGFFYTPQASAIKKEE		SCG1	5711/367830	0.999	23021	0.833	-0.137691	-1.0167	0.3333	0	18	-0.9889	0
HIP_23022	GSHLVEALY	MKRSGQLGI	GSHLVEALYMKRSGQLGI		SCG2	23530/334890	0.994	23022	0.833	0.135970	-0.3667	0	2	18	0.0611	1
HIP_23023	VCGERGFFY	LSLIPLIFL	VCGERGFFYLSLIPLIFL		SCG2	4240/334890	1.000	23023	0.833	-0.021526	1.85	0.1667	0	18	1.4889	0
HIP_23024	SHLVEALY	EQKLYTDDE	SHLVEALYEQKLYTDDE		SCG2	13932/334890	0.998	23024	0.833	0.190855	-1.1	0	-3	17	-0.8706	-4
HIP_23025	LVEALYLVC	EEDATSEVD	LVEALYLVCEEDATSEVD		SCG1	4131/367830	0.999	23025	0.833	0.108158	0	0	-6	18	0.35	-6
HIP_23026	LYQLENYCN	YGEEIKGYP	LYQLENYCNYGEEIKGYP		SCG1	3000/367830	1.000	23026	0.833	0.013429	-1.25	0.1667	-2	18	-0.9944	-2
HIP_23027	AGSLQPLAL	ELGKLTGP	AGSLQPLALELGKLTGP		SCG2	4276/334890	1.000	23027	0.833	0.201458	1.55	0.1667	0	17	0.3412	0
HIP_23028	SHLVEALYL	GPTVVLNPH	SHLVEALYLGPTVVLNPH		CMGA	1327/247050	1.000	23028	0.833	0.213280	0.6	0.3333	1	18	0.5444	-1
HIP_23029	LYQLENYCN	YALNSEKNF	LYQLENYCNYALNSEKNF		SCG2	4399/334890	1.000	23029	0.833	-0.060326	0.3333	0	-1	18	-0.8111	-1
HIP_23030	CGERGFFY	WQAKREEEE	CGERGFFYWQAKREEEE		CMGA	9850/247050	0.995	23030	0.833	0.068824	0.2833	0	-2	17	-1.5882	-2
HIP_23031	DLQVGQVEL	RMLSKSGYP	DLQVGQVELRMLSKSGYP		SCG2	22546/334890	0.995	23031	0.833	0.045861	0.95	0	0	18	-0.3333	0
HIP_23032	LCGSHLVEA	EEDNRDSSM	LCGSHLVEAEEDNRDSSM		CMGA	7103/247050	0.997	23032	0.833	0.090165	-1.3333	0	-3	18	-0.75	-4
HIP_23033	GSHLVEALY	ENGDESHLP	GSHLVEALYENGDESHLP		SCG2	1060/334890	1.000	23033	0.833	0.171901	-0.5167	0.1667	-2	18	-0.6556	-4
HIP_23034	HLVEALYL	EEKKEEEG	HLVEALYLEEKKEEEG		CMGA	8061/247050	0.997	23034	0.833	0.197744	-0.7667	0	-3	16	-1.0188	-4
HIP_23035	AGSLQPLAL	GGKSGELEQ	AGSLQPLALGGKSGELEQ		CMGA	4529/247050	0.999	23035	0.833	0.222525	0.7833	0.3333	-1	18	-0.2167	-1
HIP_23036	GSHLVEALY	LALQGAKER	GSHLVEALYLALQGAKER		CMGA	6282/247050	0.998	23036	0.833	-0.018485	2.2833	0	1	18	-0.0111	0
HIP_23037	HLVEALYLV	FLISGAEAA	HLVEALYLVFLISGAEAA		SCG2	4029/334890	1.000	23037	0.833	-0.029192	2.9667	0	-1	18	1.4111	-2

[illegible]

HIP_23496	GSLQPLALE	EIINSNQVK	GSLQPLALEEIINSNQVK		SCG2	13360/334890	0.998	23496	0.832	-0.019117	1.2667	0	-1	18	-0.1167	-1	0	0	0	0.000000	1.1333	0	0	0.950000	0.2222	0.7278	0	0	0	0	0	0.9219	0	0	0	1	0	0.3333	1.3217	0.5967	0.3333	0.1111	0.356	0.4222	0	0
HIP_23497	ERGFFYTPK	PQLRRGWRP	ERGFFYTPKPQLRRGWRP		CMGA	12740/247050	0.991	23497	0.832	-0.010693	-1.25	0.3333	4	18	-1.5556	4	2	0	0	0.000000	1.6333	-1	-0.05	0.700000	0.2222	0.4778	2	0	1	0	-1.6333	-0.5972	3.2667	0	0	1	0	0.5385	0.9003	1.0645	0	0.2899	1.1331	-0.6731	0.0025	-0.0817
HIP_23498	LVEALYLVC	ESEEGEEDA	LVEALYLVCESEEGEEDA		SCG1	1119/367830	1.000	23498	0.832	0.121010	0.45	0.1667	-7	18	-0.0611	-7	1	0	0	0.000000	-6.1	-2	0.1667	2.000000	0.3333	1.6667	0	0	0	0	12.2	0.75	-6.1	0	0	0	0	0.4385	1.2003	0.7645	0	0.1922	0.5844	0.1973	0.0278	-1.0167
HIP_23499	VEALYLVC	SLGYKEIRK	VEALYLVCSLGYKEIRK		CMGA	4115/247050	0.999	23499	0.832	0.194089	2.1833	0.3333	1	17	0.3235	1	2	1	0	0.000000	-2.6333	0	0.15	1.575000	0.1111	1.4639	0	1	0	1	0	3.1962	-5.2667	0	0	0	0	0.3885	0.9969	0.8512	0.5	0.1509	0.7245	0.8481	0.0225	-0.395
HIP_23500	LVEALYLVC	ARMLVKYPE	LVEALYLVCARMLVKYPE		SCG2	13727/334890	0.998	23500	0.832	0.051282	1.6167	0	0	18	0.9	0	1	0	0	0.000000	-3.7667	1	0.05	3.400000	0.1111	3.2889	0	0	0	0	-3.7667	5.317	-3.7667	0	0	0	0	0.2769	1.1755	0.679	0.5	0.0767	0.461	0.4477	0.0025	-0.1883
HIP_23501	SICSLYQLE	QEKAEKGRE	SICSLYQLEQEKAEKGRE		SCG2	24761/334890	0.994	23501	0.832	-0.026473	-2.35	0	-1	18	-1.1222	-1	1	0	0	0.000000	-2.5667	1	0.05	2.350000	0.3333	2.0167	0	0	0	0	-2.5667	-4.7392	-2.5667	0	0	1	0	0.5051	1.2603	0.8078	-0.3333	0.2552	0.6526	-1.1871	0.0025	-0.1283
HIP_23502	SLYQLENYC	LQWKSSHFE	SLYQLENYCLQWKSSHFE		SCG1	8613/367830	0.998	23502	0.832	-0.151761	-0.4833	0	0	18	-0.7667	-1	2	0	0	0.000000	0.5667	0	0	1.900000	0.2222	1.6778	0	0	0	0	0	-0.8109	1.1333	0	0	0	0	0.4487	1.0241	0.9601	0.3333	0.2013	0.9218	-0.2169	0	0
HIP_23503	LVEALYLVC	EVMKCIVEV	LVEALYLVCEVMKCIVEV		CMGA	4338/247050	0.999	23503	0.832	0.032040	2.1833	0	-2	18	1.6611	-2	1	0	0	0.000000	-2.6333	-1	0.05	2.000000	0.2222	1.7778	0	0	0	0	2.6333	3.8815	-2.6333	0	0	0	0	0.3051	1.1919	0.6245	0.5	0.0931	0.39	0.6662	0.0025	-0.1317
HIP_23504	GSLQPLALE	SGYPKTPGR	GSLQPLALESGYPKTPGR		SCG2	10426/334890	0.999	23504	0.832	0.151432	-0.0667	0.3333	1	18	-0.6556	1	1	0	0	0.000000	-1.5333	1	0.1	0.950000	0.1111	0.8389	0	1	0	1	-1.5333	-0.0559	-1.5333	0	0	1	0	0.4551	1.0969	0.9178	0.1667	0.2071	0.8424	-0.0303	0.01	-0.1533
HIP_23505	HLVEALYL	GPQLRRGWR	HLVEALYLGPQLRRGWR		CMGA	5968/247050	0.998	23505	0.832	0.234027	0.1333	0.3333	3	17	-0.4176	2	1	1	0	0.000000	-3.9333	0	0.2	1.400000	0.1111	1.2889	1	1	1	1	0	0.1719	-3.9333	1	0	0	0	0.4718	0.9669	0.9845	0.3333	0.2226	0.9692	0.0629	0.04	-0.7867
HIP_23506	LVEALYLVC	GGHSRERAD	LVEALYLVCGGHSRERAD		SCG1	3716/367830	0.999	23506	0.832	0.197884	1.0833	0.3333	0	18	0.0167	-1	1	0	0	0.000000	-4.8333	0	0.1667	2.000000	0	2	0	2	0	1	0	2.1667	-4.8333	0	0	0	0	0.4436	0.9005	0.969	0.1667	0.1968	0.9389	0.4806	0.0278	-0.8056
HIP_23507	QCCTSICSL	RGWRPSSRE	QCCTSICSLRGWRPSSRE		CMGA	15238/247050	0.986	23507	0.832	0.153807	-0.05	0.3333	2	18	-0.6389	2	1	0	0	0.000000	-3.7667	1	0.0667	0.000000	0.2222	-0.2222	0	1	0	1	-3.7667	0.0111	-3.7667	1	0	0	0	0.4654	0.9441	0.9868	0.1667	0.2166	0.9738	-0.0233	0.0044	-0.2511
HIP_23508	HLCGSHLVE	EEEEEEEAT	HLCGSHLVEEEEEEEEAT		CMGA	2610/247050	0.999	23508	0.832	0.021219	-1	0	-6	18	-1.1222	-8	0	0	6	0.000000	-5	-2	0.1	1.050000	0.5556	0.4944	0	0	0	0	10	-0.4944	0	0	0	1	0	0.4667	1.4	0.66	-0.3333	0.2178	0.4356	-0.4667	0.01	-0.5
HIP_23509	SHLVEALYL	QEKAEKGR	SHLVEALYLQEKAEKGR		SCG2	19173/334890	0.996	23509	0.832	0.253363	-0.7667	0	1	17	-0.7529	0	1	1	0	0.000000	-5.7333	0	0.15	0.450000	0.3333	0.1167	0	0	0	0	0	-0.0894	-5.7333	1	0	0	0	0.4103	1.2105	0.7556	0	0.1683	0.571	-0.3145	0.0225	-0.86
HIP_23510	PLALEGSLQ	RAYFMSDTR	PLALEGSLQRAYFMSDTR		SCG1	16063/367830	0.994	23510	0.832	-0.009704	-0.75	0.1667	0	18	-0.3	0	2	0	0	0.000000	-1.1667	1	-0.0667	1.650000	0.2222	1.4278	0	0	0	0	-1.1667	-1.0708	-2.3333	1	0	1	0	0.4103	1.1172	0.859	0.1667	0.1683	0.7378	-0.3077	0.0044	0.0778
HIP_23511	VCGERGFF	GHSRERADE	VCGERGFFGHSRERADE		SCG1	7892/367830	0.998	23511	0.832	0.080645	0.1333	0.5	0	17	-1.0941	-1	2	1	0	0.000000	-3.2	0	0.0667	0.700000	0.3333	0.3667	0	2	0	1	0	0.0489	-6.4	1	0	0	0	0.4718	0.8703	1.0512	0.1667	0.2226	1.1049	0.0629	0.0044	-0.2133
HIP_23512	LVCGERGFF	PSSKDVMEK	LVCGERGFFPSSKDVMEK		CMGA	1551/247050	1.000	23512	0.832	0.135780	0.3333	0.6667	0	18	-0.2556	0	2	0	0	0.000000	-2.8	0	0.1167	0.700000	0.3333	0.3667	1	1	1	1	0	0.1222	-5.6	1	0	0	0	0.5	0.845	1.1033	0.3333	0.25	1.2173	0.1667	0.0136	-0.3267
HIP_23513	TSICSLYQL	DEEDKRNYP	TSICSLYQLDEEDKRNYP		SCG1	13462/367830	0.996	23513	0.832	0.101604	-1.9167	0	-2	18	-1.1833	-2	1	0	0	0.000000	-3.1667	-3	0.1	0.000000	0.4444	-0.4444	0	0	0	0	9.5	0.8519	-3.1667	1	0	0	0	0.4769	1.2455	0.7956	-0.3333	0.2275	0.633	-0.9141	0.01	-0.3167
HIP_23514	HLVEALYL	EPGWQAKR	HLVEALYLEPGWQAKR		CMGA	5352/247050	0.998	23514	0.832	0.223646	0.1333	0.3333	1	16	-0.4438	0	2	0	0	0.000000	-3.9333	-1	0.2	0.000000	0.2222	-0.2222	1	1	1	1	3.9333	-0.0296	-7.8667	1	0	0	0	0.4718	1.0336	0.9445	0.1667	0.2226	0.8921	0.0629	0.04	-0.7867
HIP_23515	ERGFFYTPK	PEDLEWERY	ERGFFYTPKPEDLEWERY		SCG1	12103/367830	0.997	23515	0.832	-0.118278	-2.4667	0.3333	-2	18	-1.6	-2	2	0	0	0.000000	-0.8	-3	0	1.650000	0.3333	1.3167	2	0	1	0	2.4	-3.2478	-1.6	0	0	1	0	0.5769	0.9205	1.0923	-0.5	0.3328	1.1931	-1.4231	0	0
HIP_23516	GSHLVEALY	PLQWKSSH	GSHLVEALYPLQWKSSH		SCG1	8991/367830	0.998	23516	0.832	0.143603	0.5	0.1667	2	17	-0.3824	0	2	1	0	0.000000	-1.8667	0	0.1167	2.000000	0.1111	1.8889	1	0	1	0	0	0.9444	-3.7333	0	1	0	0	0.3885	1.1253	0.8445	0.5	0.1509	0.7132	0.1942	0.0136	-0.2178
HIP_23517	HLVEALYLV	DHPDLFQNR	HLVEALYLVDHPDLFQNR		SCG2	8009/334890	0.999	23517	0.832	0.154253	-0.2667	0.1667	0	18	-0.1722	-2	1	0	0	0.000000	-5	-1	0.1167	1.400000	0.2222	1.1778	1	0	1	0	5	-0.3141	-5	1	0	0	0	0.3987	0.9991	0.8735	0	0.159	0.7629	-0.1063	0.0136	-0.5833
HIP_23518	TSICSLYQL	ETTENENTK	TSICSLYQLETTENENTK		SCG1	10938/367830	0.997	23518	0.832	0.140148	-0.9833	0	-2	18	-0.8889	-2	1	0	0	0.000000	-1.3	-1	0.1	0.000000	0.2222	-0.2222	0	0	0	0	1.3	0.2185	-1.3	1	0	0	0	0.5051	1.1003	0.8412	0	0.2552	0.7075	-0.4967	0.01	-0.13
HIP_23519	EALYLVCGE	EEEEEEEEA	EALYLVCGEEEEEEEEEA		CMGA	814/247050	1.000	23519	0.832	-0.101822	-1.9833	0.1667	-10	18	-1.0444	-10	0	0	7	0.000000	-3.0333	-2	0.0167	0.950000	0.5556	0.3944	0	1	0	1	6.0667	-0.7823	0	0	0	1	0	0.5885	1.2603	0.8945	-0.6667	0.3463	0.8001	-1.1671	0.0003	-0.0506
HIP_23520	EALYLVCGE	EEEEEEEEA	EALYLVCGEEEEEEEEEA		CMGA	815/247050	1.000	23520	0.832	-0.101822	-1.9833	0.1667	-10	18	-1.0444	-10	0	0	7	0.000000	-3.0333	-2	0.0167	0.950000	0.5556	0.3944	0	1	0	1	6.0667	-0.7823	0	0	0	1	0	0.5885	1.2603	0.8945	-0.6667	0.3463	0.8001	-1.1671	0.0003	-0.0506
HIP_23521	LVCGERGFF	INLITRQRY	LVCGERGFFINLITRQRY		NPY	788/49410	0.996	23521	0.832	-0.010303	1.6667	0.1667	2	18	0.1167	2	2	0	0	1.000000	-0.1333	0	-0.0333	2.950000	0.2222	2.7278	0	1	0	1	0	4.5463	-0.2667	1	0	0	0	0.35	0.9917	0.8717	0.6667	0.1225	0.7598	0.5833	0.0011	0.0044
HIP_23522	ICSLYQLEN	YNYDWWEKK	ICSLYQLENYNYDWWEKK		SCG1	18733/367830	0.991	23522	0.832	0.016322	-1.55	0	-1	18	-1.15	-1	3	0	0	0.000000	-0.9667	1	-0.05	0.000000	0.2222	-0.2222	0	0	0	0	-0.9667	0.3444	-2.9	0	1	0	0	0.4769	1.0322	1.029	0	0.2275	1.0588	-0.7392	0.0025	0.0483
HIP_23523	CTSICSLYQ	SREKSSQES	CTSICSLYQSREKSSQES		SCG1	22495/367830	0.985	23523	0.832	0.208986	-1.6333	0.1667	0	18	-0.8833	0	1	0	0	0.000000	-2.6	0	0.1167	0.625000	0.3333	0.2917	0	0	0	0	0	-0.4764	-2.6	0	1	0	0	0.4769	1.1272	0.8723	-0.1667	0.2275	0.7609	-0.779	0.0136	-0.3033
HIP_23524	ERGFFYTPK	EMKRSGQLG	ERGFFYTPKEMKRSGQLG		SCG2	22889/334890	0.995	23524	0.832	0.026206	-1.95	0.1667	2	18	-1.2	2	2	0	0	0.000000	0.2333	-1	-0.0667	0.700000	0.4444	0.2556	1	0	1	0	-0.2333	-0.4983	0.4667	0	0	1	0	0.4936	1.0255	0.939	-0.3333	0.2436	0.8817	-0.9625	0.0044	-0.0156
HIP_23525	CGERGFFYT	SQGGSLPS	CGERGFFYTSQGGSLPS		SCG1	7559/367830	0.999	23525	0.832	0.184692	-0.65	0.3333	0	17	-0.4235	0	3	1	0	0.000000	-1.8333	0	0.15	0.000000	0	0	0	1	0	1	0	0	-5.5	0	1	0	0	0.5218	0.9103	1.0245	0.1667	0.2723	1.0496	-0.3392	0.0225	-0.275
HIP_23526	ERGFFYTPK	ITPECRQVL	ERGFFYTPKITPECRQVL		SCG1	7777/367830	0.998	23526	0.832	-0.092673	-0.6667	0.3333	1	18	-0.5056	1	2	0	0	0.000000	2.8	-1	-0.05	1.825000	0.2222	1.6028	2	0	1	0	-2.8	-1.0685	5.6	0	0	1	0	0.5385	0.8169	1.0562	0	0.2899	1.1155	-0.359	0.0025	-0.14
HIP_23527	RGFFYTPKT	RNQLLQKEP	RGFFYTPKTRNQLLQKEP		SCG2	26011/334890	0.993	23527	0.832	-0.013947	-2.95	0.1667	3	18	-1.3278	3	1	0	0	0.000000	-1.7667	0	-0.0167	0.950000	0.2222	0.7278	1	0	1	0	0	-2.1469	-1.7667	0	1	0	0	0.5603	0.8705	1.139	-0.3333	0.3139	1.2973	-1.6528	0.0003	0.0294
HIP_23528	RGFFYTPKT	RSSPETLIS	RGFFYTPKTRSSPETLIS		NPY	2929/49410	0.976	23528	0.832	-0.013621	-2.05	0.5	2	18	-0.6611	2	1	0	0	0.000000	0.0333	0	0.0167	0.000000	0.2222	-0.2222	1	0	1	0	0	0.4556	0.0333	0	1	0	0	0.5603	0.8405	1.1156	-0.3333	0.3139	1.2447	-1.1485	0.0003	0.0006
HIP_23529	VCGERGFFY	GDERILSI	VCGERGFFYGDERILSI		CMGA	5804/247050	0.998	23529	0.832	0.190106	-0.5167	0.1667	-1	17	0.1353	-1	3	1	0	0.000000	-3.9	-2	0.1833	0.700000	0.4444	0.2556	0	1	0	1	7.8	-0.132	-11.7	0	1	0	0	0.4269	1.0755	0.8756	0	0.1823	0.7667	-0.2206	0.0136	-0.715
HIP_23530	VCGERGFFY	TRLEDPAMW	VCGERGFFYTRLEDPAMW		NPY	2181/49410	0.985	23530	0.832	0.138761	0.4833	0	-1	18	-0.2778	-1	3	0	0	0.000000	-1.9	1	0.1	0.700000	0.3333	0.3667	0	0	0	0	-1.9	0.1772	-5.7	0	1	0	0	0.3436	1.0955	0.7456	0.3333	0.1181	0.556	0.1661	0.01	-0.19
HIP_23531	HLCGSHLVE	QFPPMYEEN	HLCGSHLVEQFPPMYEEN		SCG2	6651/334890	0.999	23531	0.832	0.037611	0.3667	0.1667	-1	18	-0.5889	-3	1	0	0	0.000000	-2.2667	1	0.0667	1.050000	0.1111	0.9389	1	0	1	0	-2.2667	0.3443	-2.2667	0	0	1	0	0.4167	1.125	0.8067	0.3333	0.1736	0.6507	0.1528	0.0044	-0.1511
HIP_23532	SICSLYQLE	RNYPSLELD	SICSLYQLERNYPSLELD		SCG1	23242/367830	0.984	23532	0.832	-0.053247	-2.0833	0	-2	18	-0.3556	-2	2	0	0	0.000000	-2.0333	2	0	1.900000	0.2222	1.6778	0	0	0	0	-4.0667	-3.4954	-4.0667	0	0	1	0	0.4769	1.1055	0.9323	-0.1667	0.2275	0.8692	-0.9936	0	0
HIP_23533	AGSLQPLAL	EESEESNVS	AGSLQPLALEESEESNVS		SCG1	3509/367830	1.000	23533	0.832	0.154093	0.2667	0.1667	-4	18	-0.3889	-4	0	0	0	0.000000	-5.7333	-2	0.1667	0.450000	0.3333	0.1167	0	0	0	0	11.4667	0.0311	0	1	0	0	0	0.4	1.3117	0.7233	0.1667	0.16	0.5232	0.1067	0.0278	-0.9556
HIP_23534	VCGERGFFY	SLSEEDWMR	VCGERGFFYSLSEEDWMR		SCG2	112/334890	1.000	23534	0.832	0.135069	1.0833	0.3333	-2	18	-0.5333	-2	3	0	0	0.000000	-0.7	0	0.1333	0.700000	0.2222	0.4778	0	0	0	0	0	0.5176	-2.1	0	1	0	0	0.3718	1.0536	0.8345	0.5	0.1382	0.6964	0.4028	0.0178	-0.0933
HIP_23535	GERGFFYTP	LAEAYPSKP	GERGFFYTPLAEAYPSKP		NPY	737/49410	0.996	23535	0.832	-0.084273	-0.25	0.1667	0	18	-0.6722	0	3	0	0	0.000000	1.9	-1	-0.0667	2.100000	0.1111	1.9889	1	0	1	0	-1.9	-0.4972	5.7	0	0	0	1	0.4551	1.1136	0.8812	0.1667	0.2071	0.7764	-0.1138	0.0044	-0.1267
HIP_23536	GSHLVEALY	SSREDSLEA	GSHLVEALYSSREDSLEA		CMGA	5130/247050	0.998	23536	0.832	0.195820	-0.3	0.3333	-2	18	-0.4111	-3	1	0	0	0.000000	-3.4667	1	0.1833	2.000000	0.3333	1.6667	0	0	0	0	-3.4667	-0.5	-3.4667	0	1	0	0	0.4103	1.0655	0.8956	0.1667	0.1683	0.8022	-0.1231	0.0136	-0.6356
HIP_23537	GERGFFYTP	EAQDPEGKG	GERGFFYTPEAQDPEGKG		CMGA	5852/247050	0.998	23537	0.832	-0.104942	-1.4667	0.1667	-2	18	-1.4056	-2	3	0	0	0.000000	-0.5333	-1	-0.0167	0.700000	0.2222	0.4778	1	0	1	0	0.5333	-0.7007	-1.6	0	1	0	1	0.5218	1.0819	0.9612	0	0.2723	0.9238	-0.7653	0.0003	0.0089
HIP_23538	PGAGSLQPL	NENTKFEVR	PGAGSLQPLNENTKFEVR		SCG1	14091/367830	0.996	23538	0.832	-0.001897	-1.9667	0.1667	0	18	-0.8333	0	0	0	0	0.000000	-3.0667	-1	0.0333	0.000000	0.1111	-0.1111	1	0	1	0	3.0667	0.2185	0	1	0	0	0	0.55	0.9717	1.1433	0	0.3025	1.3072	-1.0817	0.0011	-0.1022
HIP_23539	SHLVEALY	PDLFQNRML	SHLVEALYPDLFQNRML		SCG2	8281/334890	0.999	23539	0.832	0.180149	0.5	0.1667	0	17	0.0294	-1	2	1	0	0.000000	-1.8667	-1	0.1167	0.000000	0.2222	-0.2222	1	0	1	0	1.8667	0.1802	-3.7333	0	1	0	0	0.3603	1.1105	0.8323	0.3333	0.1298	0.6927	0.1801	0.0136	-0.2178
HIP_23540	VCGERGFFY	KQASAIKK	VCGERGFFYKQASAIKK		SCG1	25921/367830	0.978	23540	0.832	0.167566	-0.2167	0	3	17	-0.3353	3	3	1	0	0.000000	-3.3	1	0.1	0.700000	0.2222	0.4778	0	0	0	0	-3.3	-0.1035	-9.9	0	1	0	0	0.3436	1.1672	0.7756	0.3333	0.1181	0.6016	-0.0744	0.01	-0.33
HIP_23541	ICSLYQLEN	NQKTSYFPN	ICSLYQLENNQKTSYFPN		SCG2	29001/334890	0.991	23541	0.832	0.086153	-2.35	0	0	18	-0.7778	0	1	0	0	0.000000	-2.5667	2	0.05	0.000000	0.2222	-0.2222	0	0	0	0	-5.1333	0.5222	-2.5667	0	1	0	0	0.5051	1.0469	1.0412	-0.1667	0.2552	1.084	-1.1871	0.0025	-0.1283
HIP_23542	CGERGFFYT	GQEEEEDN	CGERGFFYTGQEEEEDN		CMGA	2662/247050	0.999	23542	0.832	0.111469	-1.1	0.1667	-5	17	-1.6235	-5	3	1	2	0.000000	-2.7333	-1	0.1333	0.000000	0.2222	-0.2222	0	1	0	1	2.7333	0.2444	-8.2	0	1	0	0	0.5218	1.0286	0.9478	0	0.2723	0.8984	-0.574	0.0178	-0.3644
HIP_23543	CGERGFFYT	GEDSSEEK	CGERGFFYTGEDSSEEK		SCG1	7448/367830	0.999	23543	0.832	0.130205	-1.1	0.1667	-3	17	-1.3294	-3	3	1	0	0.000000	-2.7333	-2	0.1333	0.000000	0.2222	-0.2222	0	1	0	1	5.4667	0.2444	-8.2	0	1	0	0	0.4936	1.0138	0.9356	-0.1667	0.2436	0.8754	-0.5429	0.0178	-0.3644
[illegible]																																														

HIP_24007	TSICSLYQL	DNRDSSMKL	TSICSLYQLDNRDSSMKL		CMGA	17616/247050	0.980	24007	0.832	0.221214	-2.0833	0	0	18	-0.4056	0	1	0	0	0.000000	-3.5	0	0.1	0.000000	0.3333	-0.3333	0	0	0	0	0	0.6944	-3.5	1	0	0	0	0.4487	1.0241	0.9601	-0.1667	0.2013	0.9218	-0.9348	0.01	-0.35
HIP_24008	CCTSICSLY	KEELVARSE	CCTSICSLYKEELVARSE		SCG1	16246/367830	0.994	24008	0.832	0.023221	-1.5333	0.1667	-1	18	0.1278	-1	1	0	0	0.000000	-4.2	-1	0.0833	3.025000	0.3333	2.6917	0	0	0	0	4.2	-4.1272	-4.2	0	1	0	0	0.4769	1.1938	0.8323	-0.3333	0.2275	0.6927	-0.7313	0.0069	-0.35
HIP_24009	AGSLQPLAL	LQEEEQIEQ	AGSLQPLALLQEEEQIEQ		SCG2	961/334890	1.000	24009	0.832	0.080654	1.0333	0	-4	18	-0.4167	-4	0	0	1	1.000000	-4.2	-1	0.1	1.400000	0.2222	1.1778	0	0	0	0	4.2	1.217	0	1	0	0	0	0.3333	1.3283	0.6467	0.5	0.1111	0.4182	0.3444	0.01	-0.42
HIP_24010	LVCGERGFF	EKRKRLGEL	LVCGERGFFEKRKRLGEL		SCG1	27485/367830	0.974	24010	0.832	0.136076	-1.1167	0.1667	2	18	-0.5167	2	2	0	0	0.000000	-5.7	1	0.0667	0.700000	0.6667	0.0333	0	1	0	1	-5.7	-0.0372	-11.4	1	0	0	0	0.4269	1.0755	0.8756	-0.1667	0.1823	0.7667	-0.4767	0.0044	-0.38
HIP_24011	GSLQPLALE	THSREKSSQ	GSLQPLALETHSREKSSQ		SCG1	31275/367830	0.961	24011	0.832	0.114332	-0.4333	0.1667	1	18	-1.0167	0	0	0	0	0.000000	-2.2667	1	0.0667	0.950000	0.2222	0.7278	0	0	0	0	-2.2667	-0.3154	0	0	0	1	0	0.4385	1.1519	0.8278	0	0.1922	0.6853	-0.19	0.0044	-0.1511
HIP_24012	HLVEALYL	QEAENEKDR	HLVEALYLQEAENEKDR		SCG1	9096/367830	0.998	24012	0.832	0.125389	0.1833	0	-2	17	-1.0706	-3	1	1	0	0.000000	-3.8333	-1	0.1	1.400000	0.2222	1.1778	0	0	0	0	3.8333	0.2159	-3.8333	1	0	0	0	0.3718	1.2819	0.7145	0.3333	0.1382	0.5105	0.0682	0.01	-0.3833
HIP_24013	CSLYQLENY	DYETQQWPE	CSLYQLENYDYETQQWPE		SCG2	16021/334890	0.997	24013	0.832	-0.080389	-2.7667	0	-4	18	-1.4333	-4	2	0	0	0.000000	0	-1	0	0.000000	0.3333	-0.3333	0	0	0	0	0	0.9222	0	0	1	0	0	0.5154	1.1258	0.9601	-0.5	0.2656	0.9218	-1.4259	0	0
HIP_24014	CGERGFFY	GNNQAPGEE	CGERGFFYGNNQAPGEE		CMGA	5269/247050	0.998	24014	0.832	0.191987	-0.5167	0.1667	-2	17	-1.1824	-2	3	1	0	0.000000	-3.9	0	0.1833	0.450000	0	0.45	0	1	0	1	0	-0.2325	-11.7	0	1	0	0	0.4551	0.8769	1.1212	0.3333	0.2071	1.257	-0.2351	0.0136	-0.715
HIP_24015	CGERGFFYT	EESWDEED	CGERGFFYTEESWDEED		SCG1	8166/367830	0.998	24015	0.832	0.079474	-1.1667	0.1667	-6	17	-1.4941	-6	4	1	0	0.000000	-2.8667	-2	0.1167	0.000000	0.2222	-0.2222	0	0	0	0	5.7333	0.2593	-11.4667	0	1	0	0	0.5051	1.1453	0.8578	-0.1667	0.2552	0.7359	-0.5893	0.0136	-0.3344
HIP_24016	TSICSLYQL	LFQNRMLS	TSICSLYQLLFQNRMLS		SCG2	12506/334890	0.998	24016	0.832	-0.033878	0.35	0	1	17	0.4412	1	2	1	0	1.000000	1.3667	0	0	0.950000	0	0.95	0	0	0	0	0	0.3325	2.7333	1	0	0	0	0.3718	1.1736	0.7445	0.5	0.1382	0.5543	0.1301	0	0
HIP_24017	CTSICSLYQ	PERELDLPV	CTSICSLYQPERELDLPV		SCG2	10730/334890	0.999	24017	0.832	0.153548	-1.7667	0.1667	-2	18	-0.0111	-2	1	0	0	0.000000	-2.8667	0	0.1167	0.625000	0.3333	0.2917	1	0	1	0	0	-0.5153	-2.8667	0	1	0	0	0.4936	1.0888	0.9256	-0.1667	0.2436	0.8568	-0.872	0.0136	-0.3344
HIP_24018	HLVEALYLV	GILKLQVFL	HLVEALYLVGILKLQVFL		IAPP	231/45018	0.999	24018	0.832	0.041712	2.4333	0.1667	1	18	1.5944	0	1	0	0	0.000000	0.4	0	0.0833	1.400000	0.1111	1.2889	0	1	0	1	0	3.1363	0.4	1	0	0	0	0.3218	1.0436	0.7295	0.6667	0.1036	0.5322	0.783	0.0069	0.0333
HIP_24019	LVCGERGFF	LSKPSPMPV	LVCGERGFFLSKPSPMPV		CMGA	8660/247050	0.996	24019	0.832	0.043271	0.7167	0.3333	1	18	0.3833	1	2	0	0	1.000000	-2.0333	1	0.0333	1.650000	0.3333	1.3167	0	1	0	1	-2.0333	0.9436	-4.0667	1	0	0	0	0.3885	1.0036	0.8845	0.3333	0.1509	0.7823	0.2784	0.0011	-0.0678
HIP_24020	AGSLQPLAL	MGILKLQVF	AGSLQPLALMGILKLQVF		IAPP	610/45018	0.998	24020	0.832	0.063852	2.5667	0.1667	1	18	1.2167	1	0	0	0	1.000000	-1.1333	0	0.0833	1.875000	0	1.875	0	1	0	1	0	4.8125	0	1	0	0	0	0.2833	1.1383	0.705	0.8333	0.0803	0.497	0.7272	0.0069	-0.0944
HIP_24021	DLQVGQVEL	YEENSRDNP	DLQVGQVELYEENSRDNP		SCG2	14075/334890	0.998	24021	0.832	0.050668	-0.6333	0	-4	18	-1.3389	-4	1	0	0	0.000000	-4.2667	-1	0.05	0.000000	0.3333	-0.3333	0	0	0	0	4.2667	0.2111	-4.2667	1	0	0	0	0.4385	1.3186	0.6878	-0.1667	0.1922	0.4731	-0.2777	0.0025	-0.2133
HIP_24022	LVEALYLV	PGKWQQQGD	LVEALYLVPGKWQQQGD		SCG1	8130/367830	0.998	24022	0.832	0.198233	0.1333	0.3333	-1	17	-0.2588	-1	2	1	1	0.000000	-4.2	1	0.2	2.000000	0.1111	1.8889	1	1	1	1	-4.2	0.2519	-8.4	1	0	0	0	0.4436	0.9122	0.9723	0.1667	0.1968	0.9454	0.0591	0.04	-0.84
HIP_24023	GERGFFYTP	ENGDESHLP	GERGFFYTPENGDESHLP		SCG2	2643/334890	1.000	24023	0.832	-0.039462	-1.8333	0.3333	-2	18	-1.2778	-3	3	0	0	0.000000	-1.2667	-1	0.0667	0.700000	0.2222	0.4778	1	1	1	1	1.2667	-0.8759	-3.8	0	0	0	1	0.6051	0.8503	1.1978	-0.1667	0.3662	1.4348	-1.1094	0.0044	-0.0844
HIP_24024	HLVEALYLV	YGEESEEE	HLVEALYLVYGEESEEE		SCG1	626/367830	1.000	24024	0.832	0.111672	0.25	0.1667	-5	17	-0.3765	-6	2	1	1	0.000000	-3.9667	-1	0.1333	1.400000	0.2222	1.1778	0	1	0	1	3.9667	0.2944	-7.9333	1	0	0	0	0.4269	1.0688	0.8423	0.1667	0.1823	0.7095	0.1067	0.0178	-0.5289
HIP_24025	LYQLENYCN	SKRFPVGPP	LYQLENYCNSKRFPVGPP		SCG2	22421/334890	0.995	24025	0.832	0.142156	-1.9167	0.1667	1	18	-0.7722	1	2	0	0	0.000000	-2.3	2	0.1167	1.325000	0.3333	0.9917	0	0	0	0	-4.6	-1.9007	-4.6	0	1	0	0	0.4872	0.926	1.0646	-0.3333	0.2373	1.1334	-0.9338	0.0136	-0.2683
HIP_24026	SHLVEALYL	GPQAEGDSE	SHLVEALYLGPQAEGDSE		CMGA	387/247050	1.000	24026	0.832	0.107059	0.1333	0.3333	-3	18	-0.3778	-4	1	0	0	0.000000	-3.9333	0	0.2	0.450000	0.1111	0.3389	1	1	1	1	0	0.0452	-3.9333	1	0	0	0	0.4718	0.9669	0.9845	0.3333	0.2226	0.9692	0.0629	0.04	-0.7867
HIP_24027	VCGERGFF	GEESEEER	VCGERGFFGEESEEER		SCG1	2709/367830	1.000	24027	0.832	0.071615	-0.3667	0.3333	-4	16	-1.2312	-4	2	0	1	0.000000	-4.2	-2	0.1	0.700000	0.4444	0.2556	0	2	0	1	8.4	-0.0937	-8.4	1	0	0	0	0.5	1.07	0.9467	0	0.25	0.8962	-0.1833	0.01	-0.42
HIP_24028	QCCTSICSL	EEEEMAVVP	QCCTSICSLEEEEMAVVP		CMGA	4004/247050	0.999	24028	0.832	0.037574	-0.8333	0.1667	-4	18	0.3611	-4	0	0	2	0.000000	-5.3333	-3	0.0833	0.000000	0.4444	-0.4444	0	0	0	0	16	0.3704	0	1	0	0	0	0.5051	1.2753	0.8045	-0.3333	0.2552	0.6472	-0.4209	0.0069	-0.4444
HIP_24029	NQHLCGSHL	SEEPVSTHQ	NQHLCGSHLSEEPVSTHQ		SCG1	12150/367830	0.997	24029	0.832	0.099730	-1.3333	0.3333	1	18	-0.9944	-2	0	0	0	0.000000	-2.5333	-2	0.1	0.625000	0.2222	0.4028	0	0	0	0	5.0667	-0.537	0	1	0	0	0	0.5051	1.1569	0.8812	-0.3333	0.2552	0.7764	-0.6735	0.01	-0.2533
HIP_24030	CTSICSLYQ	VEKRKCNTA	CTSICSLYQVEKRKCNTA		IAPP	3868/45018	0.961	24030	0.832	0.040823	-0.7	0	2	18	-0.2944	2	1	0	0	0.000000	-0.7333	0	0.05	1.675000	0.3333	1.3417	0	0	0	0	0	-0.9392	-0.7333	0	1	0	0	0.4103	1.1705	0.7556	0	0.1683	0.571	-0.2872	0.0025	-0.0367
HIP_24031	VCGERGFFY	REDSLEAG	VCGERGFFYREDSLEAG		CMGA	10221/247050	0.995	24031	0.832	0.182449	-1.2	0	-2	17	-0.4941	-2	3	1	0	0.000000	-5.2667	-1	0.15	0.700000	0.4444	0.2556	0	0	0	0	5.2667	-0.3067	-15.8	0	1	0	0	0.3821	1.1624	0.7768	-0.1667	0.146	0.6034	-0.4585	0.0225	-0.79
HIP_24032	GGGPGAGSL	PQESKGQPR	GGGPGAGSLPQESKGQPR		SCG1	7913/367830	0.998	24032	0.832	-0.020403	-1	0.5	1	18	-1.2278	1	0	0	1	0.000000	-3.7333	-1	0.0167	0.000000	0.1111	-0.1111	1	1	1	1	3.7333	0.1111	0	1	0	0	0	0.5667	0.965	1.0733	0	0.3211	1.152	-0.5667	0.0003	-0.0622
HIP_24033	LVCGERGFF	ESEEGEED	LVCGERGFFESEEGEED		SCG1	4022/367830	0.999	24033	0.832	0.039867	-0.4333	0.3333	-6	17	-0.8765	-6	2	1	0	0.000000	-4.3333	-2	0.0833	0.700000	0.5556	0.1444	0	1	0	1	8.6667	-0.0626	-8.6667	1	0	0	0	0.4833	1.12	0.8967	0	0.2336	0.804	-0.2094	0.0069	-0.3611
HIP_24034	LVCGERGFF	EESEEGEE	LVCGERGFFEESEEGEE		SCG1	4023/367830	0.999	24034	0.832	0.039867	-0.4333	0.3333	-6	17	-0.8765	-6	2	1	0	0.000000	-4.3333	-2	0.0833	0.700000	0.5556	0.1444	0	1	0	1	8.6667	-0.0626	-8.6667	1	0	0	0	0.4833	1.12	0.8967	0	0.2336	0.804	-0.2094	0.0069	-0.3611
HIP_24035	HLVEALYLV	YTDDEDDIY	HLVEALYLVYTDDEDDIY		SCG2	5002/334890	1.000	24035	0.832	0.075999	0.2	0	-5	18	-0.15	-6	2	0	0	0.000000	-4.0667	-1	0.1	1.400000	0.2222	1.1778	0	0	0	0	4.0667	0.2356	-8.1333	1	0	0	0	0.3821	1.0424	0.7801	0.1667	0.146	0.6086	0.0764	0.01	-0.4067
HIP_24036	VNQHLCGSH	EKGREHIAK	VNQHLCGSHEKGREHIAK		SCG2	18869/334890	0.996	24036	0.832	-0.024716	-2.0333	0.5	4	18	-1.15	1	0	0	0	0.000000	-1.1333	0	0.0333	0.950000	0.3333	0.6167	0	2	0	1	0	-1.2539	0	0	0	1	0	0.5769	0.8022	1.1256	-0.5	0.3328	1.2671	-1.1731	0.0011	-0.0378
HIP_24037	VCGERGFFY	LPSEEKGHP	VCGERGFFYLPSEEKGHP		SCG1	956/367830	1.000	24037	0.832	0.127806	0.95	0.3333	0	18	-0.6944	-1	3	0	0	0.000000	-0.9667	0	0.1333	1.650000	0.2222	1.4278	1	0	1	0	0	1.3564	-2.9	0	1	0	0	0.3885	1.0153	0.8878	0.5	0.1509	0.7882	0.369	0.0178	-0.1289
HIP_24038	LYLVCGERG	SPDYNPYQG	LYLVCGERGSPDYNPYQG		SCG2	8975/334890	0.999	24038	0.832	-0.121579	-2.3833	0.5	-1	18	-0.7389	-1	1	0	0	0.000000	0.8333	-1	0	0.625000	0.3333	0.2917	1	1	1	1	-0.8333	-0.6951	0.8333	0	0	0	1	0.5769	0.9038	1.129	-0.5	0.3328	1.2746	-1.375	0	0
HIP_24039	CCTSICSLY	PNPYNQEKV	CCTSICSLYPNPYNQEKV		SCG2	4887/334890	1.000	24039	0.832	0.077169	-0.8333	0.5	0	18	-0.3333	0	2	0	0	0.000000	-2.8	0	0.1167	2.075000	0	2.075	2	0	1	0	0	-1.7292	-5.6	0	1	0	0	0.5385	0.8219	1.2012	0.1667	0.2899	1.4428	-0.4487	0.0136	-0.3267
HIP_24040	GSHLVEALY	DVMEKREDS	GSHLVEALYDVMEKREDS		CMGA	4197/247050	0.999	24040	0.832	-0.026325	1.15	0	-2	18	-0.7056	-3	1	0	0	0.000000	-0.5667	-1	0.05	2.000000	0.3333	1.6667	0	0	0	0	0.5667	1.9167	-0.5667	0	1	0	0	0.2769	1.1755	0.679	0.5	0.0767	0.461	0.3185	0.0025	-0.0283
HIP_24041	VEALYLVC	FYDSEEPVS	VEALYLVCFYDSEEPVS		SCG1	11008/367830	0.997	24041	0.832	0.059497	1.4167	0	-4	17	0.4412	-4	3	1	0	0.000000	-4.1667	-1	0.05	0.625000	0.2222	0.4028	0	0	0	0	4.1667	0.5706	-12.5	0	1	0	0	0.3154	1.1041	0.7201	0.3333	0.0995	0.5186	0.4468	0.0025	-0.2083
HIP_24042	CGERGFFY	EDELSEVLE	CGERGFFYEDELSEVLE		CMGA	5595/247050	0.998	24042	0.832	0.128052	-1.0333	0	-5	17	-0.5	-5	3	1	0	0.000000	-4.9333	-3	0.15	0.000000	0.3333	-0.3333	0	0	0	0	14.8	0.3444	-14.8	0	1	0	0	0.4103	1.2438	0.749	-0.1667	0.1683	0.561	-0.4239	0.0225	-0.74
HIP_24043	CGERGFFY	LPERDSLS	CGERGFFYLPERDSLS		SCG2	5958/334890	0.999	24043	0.832	0.122521	0.5	0.1667	-1	16	-0.5688	-1	3	0	0	0.000000	-1.8667	-1	0.1167	1.650000	0.3333	1.3167	1	0	1	0	1.8667	0.6583	-5.6	0	1	0	0	0.3885	1.1336	0.8112	0.3333	0.1509	0.658	0.1942	0.0136	-0.2178
HIP_24044	LCGSHLVEA	RYYSALRHY	LCGSHLVEARYYSALRHY		NPY	4334/49410	0.950	24044	0.832	0.096497	-0.7667	0	3	18	-0.1722	1	2	0	0	0.000000	-3.2	2	0	0.000000	0.2222	-0.2222	0	0	0	0	-6.4	0.1704	-6.4	1	0	0	0	0.3821	1.1808	0.7701	0	0.146	0.5931	-0.2929	0	0
HIP_24045	HLVEALYL	EFPDFYDSE	HLVEALYLEFPDFYDSE		SCG1	2665/367830	1.000	24045	0.832	0.069606	0.6667	0.1667	-4	17	-0.1588	-5	3	1	0	0.000000	-2.8667	-1	0.1167	2.800000	0.2222	2.5778	1	0	1	0	2.8667	1.7185	-8.6	1	0	0	0	0.3885	1.1503	0.7945	0.3333	0.1509	0.6312	0.259	0.0136	-0.3344
HIP_24046	TSICSLYQL	NQLLQKEPD	TSICSLYQLNQLLQKEPD		SCG2	9483/334890	0.999	24046	0.832	-0.025197	-0.7	0	-1	18	-0.4389	-1	1	0	0	0.000000	-0.7333	0	0.05	0.950000	0	0.95	0	0	0	0	0	-0.665	-0.7333	1	0	0	0	0.4385	1.0853	0.9045	0.3333	0.1922	0.8181	-0.3069	0.0025	-0.0367
HIP_24047	CGERGFFY	SLPSQKYP	CGERGFFYSLPSQKYP		CMGA	4290/247050	0.999	24047	0.832	0.171467	0.95	0.3333	1	16	-0.7313	1	3	0	0	0.000000	-0.9667	0	0.1333	0.700000	0.1111	0.5889	1	0	1	0	0	0.5594	-2.9	0	1	0	0	0.3885	1.0153	0.8878	0.5	0.1509	0.7882	0.369	0.0178	-0.1289
HIP_24048	CCTSICSLY	NPYQGVSVP	CCTSICSLYNPYQGVSVP		SCG2	9562/334890	0.999	24048	0.832	-0.005002	-0.7833	0.3333	0	18	0.4389	0	2	0	0	0.000000	-2.7	0	0.05	2.075000	0	2.075	1	0	1	0	0	-1.6254	-5.4	0	1	0	0	0.4936	0.8972	1.099	0.1667	0.2436	1.2077	-0.3866	0.0025	-0.135
HIP_24049	LVCGERGFF	IEQAIKEHL	LVCGERGFFIEQAIKEHL		SCG2	6632/334890	0.999	24049	0.832	-0.008678	0.45	0.1667	0	18	0.2389	-1	2	0	0	1.000000	-2.5667	-1	0.0167	2.275000	0.3333	1.9417	0	1	0	1	2.5667	0.8737	-5.1333	1	0	0	0	0.4167	1.1	0.815	0.3333	0.1736	0.6642	0.1875	0.0003	-0.048

HIP_24518	VEALYLVC	EEEEEAEAG	VEALYLVCEEEEEAEAG		CMGA	3635/247050	0.999	24518	0.831	0.123319	0	0	-7	17	-0.1353	-7	1	1
HIP_24519	EQCCTSICS	EEEEEMAVV	EQCCTSICSEEEEEMAVV		CMGA	2121/247050	0.999	24519	0.831	0.006160	-0.7167	0.1667	-6	18	-0.15	-6	0	0
HIP_24520	CTSICSLY	GSEEYRAPR	CTSICSLYGSEEYRAPR		SCG1	6019/367830	0.999	24520	0.831	0.170801	-0.5	0.5	0	17	-0.5059	0	2	1
HIP_24521	LYQLENYCN	HSTHYRASE	LYQLENYCNHSTHYRASE		SCG1	18241/367830	0.992	24521	0.831	0.018900	-1.1667	0.1667	1	18	-1.2611	-1	1	0
HIP_24522	SICSLYQLE	YINLITRQR	SICSLYQLEYINLITRQR		NPY	1680/49410	0.990	24522	0.831	-0.092599	-0.5833	0	1	18	-0.0278	1	2	0
HIP_24523	GSLQPLAL	AHESSSRGE	GSLQPLALAHESSSRGE		SCG1	14237/367830	0.995	24523	0.831	0.074334	0.75	0	0	17	-0.5176	-1	0	1
HIP_24524	GPGAGSLQP	EEEEEEEAE	GPGAGSLQPEEEEEEEAE		CMGA	1465/247050	1.000	24524	0.831	-0.068708	-1.9667	0.1667	-8	18	-1.6278	-8	0	0
HIP_24525	LVEALYLVC	PQSLATLES	LVEALYLVCPQSLATLES		SCG2	3675/334890	1.000	24525	0.831	0.183557	0.7667	0.3333	-2	18	0.9889	-2	1	0
HIP_24526	GSLQPLAL	ESSSRGEAG	GSLQPLALESSSRGEAG		SCG1	5964/367830	0.999	24526	0.831	0.220802	0.7167	0.3333	-1	17	-0.3529	-1	0	1
HIP_24527	LVCGERGFF	ALSKSSAPP	LVCGERGFFALSKSSAPP		SCG1	4152/367830	0.999	24527	0.831	-0.007380	1.6667	0.3333	1	18	0.2889	1	2	0
HIP_24528	LVEALYLV	WEDSKRWSK	LVEALYLVWEDSKRWSK		CMGA	9431/247050	0.995	24528	0.831	0.110706	-0.2	0	0	17	-0.3471	0	2	1
HIP_24529	VEALYLVC	QFPPMYEEN	VEALYLVCQFPPMYEEN		SCG2	5680/334890	0.999	24529	0.831	0.123841	1.3667	0.1667	-3	17	0.1	-3	2	1
HIP_24530	CGSHLVEAL	MQFPPMYEE	CGSHLVEALMQFPPMYEE		SCG2	6141/334890	0.999	24530	0.831	-0.075404	0.55	0	-2	18	-0.0111	-3	1	0
HIP_24531	FVNQHLCGS	EQEHSQQKE	FVNQHLCGSEQEHSQQKE		CMGA	6743/247050	0.998	24531	0.831	-0.055816	-1.5333	0.3333	0	18	-1.5	-2	0	0
HIP_24532	HLVEALYLV	EKTHSREKS	HLVEALYLVEKTHSREKS		SCG1	18971/367830	0.991	24532	0.831	0.236956	-0.2333	0	2	18	-0.6222	0	1	0
HIP_24533	LVEALYLVC	SQGLVDREK	LVEALYLVCSQGLVDREK		CMGA	2940/247050	0.999	24533	0.831	0.193456	0.9667	0.3333	-1	18	0.4	-1	1	0
HIP_24534	RGFFYTPKT	YETQQWPER	RGFFYTPKTYETQQWPER		SCG2	18182/334890	0.997	24534	0.831	0.011714	-1.95	0.1667	1	18	-1.6944	1	2	0
HIP_24535	HLVEALYL	MESAANQK	HLVEALYLMESAANQK		SCG2	8336/334890	0.999	24535	0.831	0.192641	0.65	0.1667	0	16	-0.0188	-1	1	0
HIP_24536	LVEALYLVC	KVAHQLQAL	LVEALYLVCKVAHQLQAL		CMGA	10880/247050	0.994	24536	0.831	0.073722	2.1	0	1	18	1.1444	0	1	0
HIP_24537	SHLVEALYL	GLSQGLVDR	SHLVEALYLGLSQGLVDR		CMGA	574/247050	1.000	24537	0.831	0.192800	1.4833	0.3333	0	18	0.4056	-1	1	0
HIP_24538	CGERGFFY	SKPDNPGED	CGERGFFYSKPDNPGED		NPY	1658/49410	0.990	24538	0.831	0.203673	-0.3333	0.3333	-2	17	-1.4294	-2	3	1
HIP_24539	LVCGERGFF	EKGLSAEPG	LVCGERGFFEKGLSAEPG		CMGA	7134/247050	0.997	24539	0.831	0.112610	-0.4333	0.3333	-1	18	-0.0667	-1	2	0
HIP_24540	QHLCGSHLV	KYPEIINSN	QHLCGSHLVKYPEIINSN		SCG2	19419/334890	0.996	24540	0.831	0.207959	-0.3333	0.1667	2	18	-0.3278	0	1	0
HIP_24541	SHLVEALY	VALNHLKAT	SHLVEALYVALNHLKAT		IAPP	1653/45018	0.993	24541	0.831	0.008874	2.35	0	2	17	0.5235	0	1	1
HIP_24542	CGERGFFY	SQREDEEEE	CGERGFFYSQREDEEEE		SCG1	12201/367830	0.997	24542	0.831	0.178862	-0.75	0.1667	-5	17	-1.8706	-5	3	1
HIP_24543	CGERGFFYT	NPVEEKIES	CGERGFFYTNPVEEKIES		SCG2	762/334890	1.000	24543	0.831	0.000392	-0.0167	0.1667	-2	18	-0.7944	-2	3	0
HIP_24544	CSLYQLENY	REDSKEAEK	CSLYQLENYREDSKEAEK		CMGA	18932/247050	0.976	24544	0.831	0.019101	-3.3	0	-2	18	-1.6167	-2	1	0
HIP_24545	LVEALYLV	EPGWQAKR	LVEALYLVEPGWQAKR		CMGA	4021/247050	0.999	24545	0.831	0.202986	0.2	0.3333	0	16	0.0187	0	2	0
HIP_24546	LVEALYLV	YNPYQGVS	LVEALYLVYNPYQGVS		SCG2	9540/334890	0.999	24546	0.831	0.134917	0.05	0.1667	-1	16	0.5375	-1	3	0
HIP_24547	VEALYLVC	VSKRFPVGP	VEALYLVCVSKRFPVGP		SCG2	17015/334890	0.997	24547	0.831	0.187712	1.6667	0.1667	1	17	0.8176	1	2	1
HIP_24548	QHLCGSHLV	ENEPQSAPK	QHLCGSHLVENEPQSAPK		SCG2	10193/334890	0.999	24548	0.831	0.166986	-0.95	0	1	18	-0.9389	-1	0	0
HIP_24549	VCGERGFF	EDSKRWSKM	VCGERGFFEDSKRWSKM		CMGA	9489/247050	0.995	24549	0.831	0.108834	-0.4333	0.3333	1	17	-0.9647	1	2	1
HIP_24550	YLVCGERGF	SQKYPGPQA	YLVCGERGFSQKYPGPQA		CMGA	14452/247050	0.988	24550	0.831	0.068952	-1.7167	0.3333	1	18	-0.6444	1	2	0
HIP_24551	LVEALYLVC	KERAHQQKK	LVEALYLVCKERAHQQKK		CMGA	16830/247050	0.982	24551	0.831	0.212650	-0.2333	0	3	18	-0.4889	2	1	0
HIP_24552	AGSLQPLAL	ENELTLNEK	AGSLQPLALENELTLNEK		SCG1	6318/367830	0.999	24552	0.831	0.137076	-0.1833	0	-2	18	-0.3222	-2	0	0
HIP_24553	GERGFFYT	GEEEEEEE	GERGFFYTGEEEEEEE		CMGA	1103/247050	1.000	24553	0.831	0.078916	-1.1	0.1667	-7	16	-1.8813	-7	3	0
HIP_24554	VCGERGFFY	DGAGKPGA	VCGERGFFYDGAGKPGA		CMGA	6301/247050	0.998	24554	0.831	0.176353	0.3667	0.1667	0	17	-0.2588	0	3	1
HIP_24555	GSHLVEALY	NGDESHLPE	GSHLVEALYNGDESHLPE		SCG2	1481/334890	1.000	24555	0.831	0.172846	-0.5167	0.1667	-2	18	-0.6556	-4	1	0
HIP_24556	LYQLENYCN	ESNVSMASL	LYQLENYCNESNVSMASL		SCG1	5894/367830	0.999	24556	0.831	0.055280	-1.6833	0.1667	-2	18	-0.2333	-2	1	0
HIP_24557	HLVEALYL	LDQLLHYRK	HLVEALYLLDQLLHYRK		SCG1	13579/367830	0.996	24557	0.831	0.140890	0.5167	0	2	17	0.0529	0	1	1
HIP_24558	GSHLVEALY	DWNPVEEK	GSHLVEALYDWNPVEEK		SCG2	9398/334890	0.999	24558	0.831	0.092194	-0.6	0	-2	17	-0.6941	-3	2	1
HIP_24559	CCTSICSLY	RGWRPSSRE	CCTSICSLYRGWRPSSRE		CMGA	12290/247050	0.992	24559	0.831	0.059288	-0.6833	0.3333	2	18	-0.5167	2	2	0
HIP_24560	LGGGPGAGS	TQQWPERKL	LGGGPGAGSTQQWPERKL		SCG2	13993/334890	0.998	24560	0.831	0.006058	-1.1833	0.3333	1	18	-0.95	1	1	0
HIP_24561	LVCGERGFF	GRSRPDRS	LVCGERGFFGRSRPDRS		SCG1	16470/367830	0.994	24561	0.831	0.177276	-0.0833	0.5	2	17	-0.7824	2	2	1
HIP_24562	LVEALYLVC	ELENLAAM	LVEALYLVCELENLAAM		SCG1	5543/367830	0.999	24562	0.831	0.086480	1.2167	0	-3	17	1.2882	-3	1	1
HIP_24563	SHLVEALYL	ENREEARFQ	SHLVEALYLENREEARFQ		SCG1	17744/367830	0.992	24563	0.831	0.238559	-0.8667	0	-1	18	-0.7389	-2	1	0
HIP_24564	SHLVEALYL	NREEARFQD	SHLVEALYLNREEARFQD		SCG1	17745/367830	0.992	24564	0.831	0.238559	-0.8667	0	-1	18	-0.7389	-2	1	0
HIP_24565	HLCGSHLVE	WSLYPSDSQ	HLCGSHLVEWSLYPSDSQ		SCG1	10284/367830	0.998	24565	0.831	-0.016846	1.1	0.1667	0	18	-0.3444	-2	2	0

HIP_25029	SICSLYQLE	EESSPDYNP	SICSLYQLEEESSPDYNP		SCG2	11132/334890	0.999	25029	0.831	-0.072979	-1.8333	0.1667	-4	18	-0.8556	-4	1	0	1	[illegible]
HIP_25030	VEQCCTSIC	EPGKGRHHR	VEQCCTSICEPGKGRHHR		SCG1	4245/367830	0.999	25030	0.831	0.147040	0.1167	0.5	3	18	-0.9722	1	0	0	0	[illegible]
HIP_25031	VEALYLVCG	QKFPSPEMI	VEALYLVCGQKFPSPEMI		SCG2	14603/334890	0.998	25031	0.831	0.043657	0.2833	0.1667	-1	18	0.5222	-1	2	0	0	[illegible]
HIP_25032	AGSLQPLAL	ITPECRQVL	AGSLQPLALITPECRQVL		SCG1	3297/367830	1.000	25032	0.831	0.137264	1.9333	0.1667	0	18	0.55	0	0	0	0	[illegible]
HIP_25033	QCCTSICSL	LFEKPLDSQ	QCCTSICSLLFEKPLDSQ		SCG2	7036/334890	0.999	25033	0.831	-0.017776	1.4333	0.1667	-1	18	0.2	-1	1	0	0	[illegible]
HIP_25034	HLVEALYL	NQSSQAELK	HLVEALYLNQSSQAELK		CMGA	7255/247050	0.997	25034	0.831	0.226385	-0.25	0.1667	0	17	-0.2647	-1	1	1	0	[illegible]
HIP_25035	VCGERGFFY	CAGQVTALP	VCGERGFFYCAGQVTALP		CMGA	134/247050	1.000	25035	0.831	0.033340	1.3667	0.1667	0	18	0.5611	0	3	0	0	[illegible]
HIP_25036	HLVEALYLV	SRKDVKDKE	HLVEALYLVSRKDVKDKE		SCG1	21451/367830	0.987	25036	0.831	0.216067	-0.4167	0.1667	1	18	-0.5389	0	1	0	0	[illegible]
HIP_25037	AGSLQPLAL	NEGMVTRCI	AGSLQPLALNEGMVTRCI		SCG1	5567/367830	0.999	25037	0.831	0.214181	0.3333	0.1667	0	18	0.5111	0	0	0	0	[illegible]
HIP_25038	AGSLQPLAL	GSNTYGKR	AGSLQPLALGSNTYGKR		IAPP	1154/45018	0.995	25038	0.831	0.246081	0.7833	0.3333	2	17	-0.4	2	0	1	0	[illegible]
HIP_25039	YLVCGERGF	WGYEKRNLA	YLVCGERGFWGYEKRNLA		SCG1	5647/367830	0.999	25039	0.831	0.006244	-0.7833	0.3333	1	18	-0.5111	1	3	0	0	[illegible]
HIP_25040	SLYQLENYC	PFKRTNEIV	SLYQLENYCPFKRTNEIV		SCG2	10844/334890	0.999	25040	0.831	-0.006926	-0.8333	0.1667	0	18	-0.5556	0	2	0	0	[illegible]
HIP_25041	CGERGFFY	DGAGKPGAE	CGERGFFYDGAGKPGAE		CMGA	6046/247050	0.998	25041	0.831	0.175815	0.3667	0.1667	-1	17	-0.7118	-1	3	1	0	[illegible]
HIP_25042	LVEALYLVC	EGMVTRCII	LVEALYLVCEGMVTRCII		SCG1	2340/367830	1.000	25042	0.831	0.128310	1.4167	0.1667	-1	18	1.5444	-1	1	0	0	[illegible]
HIP_25043	LYQLENYCN	EDWMRIILE	LYQLENYCNEDWMRIILE		SCG2	10588/334890	0.999	25043	0.831	-0.027716	-1.7	0	-3	18	-0.4278	-3	2	0	0	[illegible]
HIP_25044	GSLQPLAL	QVFLIVLSV	GSLQPLALQVFLIVLSV		IAPP	1239/45018	0.995	25044	0.831	0.032516	2.15	0	0	17	1.7706	0	1	1	0	[illegible]
HIP_25045	GSLQPLAL	NPYNQEKV	GSLQPLALNPYNQEKV		SCG2	11639/334890	0.998	25045	0.831	0.206182	0.5	0.1667	0	16	-0.6062	0	1	0	0	[illegible]
HIP_25046	NQHLCGSHL	YWDEDLLMK	NQHLCGSHLYWDEDLLMK		SCG2	15307/334890	0.998	25046	0.831	-0.006801	-0.9833	0.1667	0	18	-0.6444	-2	2	0	0	[illegible]
HIP_25047	GERGFFYTP	HSRERADEP	GERGFFYTPHSRERADEP		SCG1	20566/367830	0.989	25047	0.831	-0.020483	-2.0167	0.3333	0	18	-1.6722	-1	3	0	0	[illegible]
HIP_25048	AGSLQPLAL	LPERDSLSE	AGSLQPLALLPERDSLSE		SCG2	204/334890	1.000	25048	0.831	0.126194	1.35	0.1667	-2	18	-0.1056	-2	0	0	0	[illegible]
HIP_25049	TSICSLYQL	EHLNQGSSQ	TSICSLYQLEHLNQGSSQ		SCG2	22446/334890	0.995	25049	0.831	0.012389	-0.65	0	0	18	-0.4389	-1	1	0	0	[illegible]
HIP_25050	ERGFFYTPK	PNGSVEPER	ERGFFYTPKPNGSVEPER		SCG2	5778/334890	0.999	25050	0.831	-0.095682	-1.95	0.5	0	18	-1.4167	0	2	0	0	[illegible]
HIP_25051	CTSICSLYQ	EYGEEIKGY	CTSICSLYQEYGEEIKGY		SCG1	3495/367830	1.000	25051	0.831	0.043194	-1.0333	0.1667	-2	18	-0.3944	-2	2	0	0	[illegible]
HIP_25052	VCGERGFFY	IEKNEQIN	VCGERGFFYIEKNEQIN		SCG2	15440/334890	0.998	25052	0.831	0.120500	0.2333	0	-1	17	-0.6	-1	3	1	0	[illegible]
HIP_25053	SHLVEALY	VSKRFPVGP	SHLVEALYVSKRFPVGP		SCG2	18438/334890	0.997	25053	0.831	0.209282	0.6333	0.1667	2	17	0.1882	1	2	1	0	[illegible]
HIP_25054	CGERGFFY	EAELEKVAH	CGERGFFYEAELEKVAH		CMGA	10067/247050	0.995	25054	0.831	0.116681	-0.15	0	-1	17	-0.4706	-2	3	1	0	[illegible]
HIP_25055	DLQVGQVEL	WGYEKRNLA	DLQVGQVELWGYEKRNLA		SCG1	14751/367830	0.995	25055	0.831	0.058011	0.3167	0.1667	-1	18	-0.6	-1	2	0	0	[illegible]
HIP_25056	VCGERGFF	ELDRNYLNY	VCGERGFFELDRNYLNY		SCG1	9996/367830	0.998	25056	0.831	0.008744	0.3333	0.1667	-1	17	-0.5882	-1	2	1	0	[illegible]
HIP_25057	RGFFYTPKT	TQNAFLNER	RGFFYTPKTTQNAFLNER		SCG1	22241/367830	0.986	25057	0.831	-0.034964	-2.3167	0.1667	2	18	-1.0167	2	1	0	0	[illegible]
HIP_25058	CTSICSLYQ	GESRSEALA	CTSICSLYQGESRSEALA		CMGA	1252/247050	1.000	25058	0.831	0.165146	-0.95	0.3333	-1	18	0.0056	-1	1	0	0	[illegible]
HIP_25059	AGSLQPLAL	EEAEAGEEA	AGSLQPLALEEAEAGEEA		CMGA	2594/247050	0.999	25059	0.831	0.064795	0.7	0	-5	18	-0.2111	-5	0	0	0	[illegible]
HIP_25060	ICSLYQLEN	YDPLQWKSS	ICSLYQLENYDPLQWKSS		SCG1	16661/367830	0.993	25060	0.831	-0.078330	-1.6	0.1667	-1	18	-0.5833	-1	2	0	0	[illegible]
HIP_25061	SHLVEALYL	RGRGGEPRA	SHLVEALYLRGRGGEPRA		SCG1	20376/367830	0.989	25061	0.831	0.242078	-0.5167	0.1667	2	18	-0.5222	1	1	0	0	[illegible]
HIP_25062	GSHLVEALY	RGGKSGELE	GSHLVEALYRGGKSGELE		CMGA	4512/247050	0.999	25062	0.831	0.202578	-0.1667	0.3333	0	18	-0.5111	-1	1	0	0	[illegible]
HIP_25063	LVCGERGFF	EEERLSKE	LVCGERGFFEEERLSKE		CMGA	7915/247050	0.997	25063	0.831	0.058359	-0.8833	0.1667	-2	17	-0.7118	-2	2	1	1	[illegible]
HIP_25064	SHLVEALY	EEDNRDSSM	SHLVEALYEEDNRDSSM		CMGA	6466/247050	0.998	25064	0.831	0.144320	-1.0333	0	-3	17	-0.9941	-4	1	1	0	[illegible]
HIP_25065	CGSHLVEAL	QNGQNGERA	CGSHLVEALQNGQNGERA		SCG2	8411/334890	0.999	25065	0.831	0.165180	-0.8833	0.1667	0	18	-0.7111	-1	0	0	0	[illegible]
HIP_25066	CGSHLVEAL	QENQMDKAR	CGSHLVEALQENQMDKAR		SCG1	21084/367830	0.988	25066	0.831	0.147124	-1.4	0	0	18	-0.7778	-1	0	0	0	[illegible]
HIP_25067	VCGERGFF	YGEESEEER	VCGERGFFYGEESEEER		SCG1	6168/367830	0.999	25067	0.831	0.027065	0	0.3333	-4	17	-1.2353	-4	3	1	1	[illegible]
HIP_25068	LVEALYLVC	AALSLIPLI	LVEALYLVCAALSLIPLI		SCG2	7806/334890	0.999	25068	0.831	0.005175	2.9833	0	-1	18	2.2722	-1	1	0	0	[illegible]
HIP_25069	SLYQLENYC	ENLRQQAHK	SLYQLENYCENLRQQAHK		SCG2	23396/334890	0.995	25069	0.831	0.006843	-0.9167	0	1	18	-1.3222	0	1	0	0	[illegible]
HIP_25070	VEQCCTSIC	FPEYNYDWW	VEQCCTSICFPEYNYDWW		SCG1	2228/367830	1.000	25070	0.831	-0.021060	0.65	0.3333	-3	18	-0.3333	-3	2	0	0	[illegible]
HIP_25071	LVEALYLVC	LLMRESTEN	LVEALYLVCLLMRESTEN		NPY	978/49410	0.995	25071	0.831	-0.012309	3.3333	0	-2	18	0.6833	-2	1	0	0	[illegible]
[illegible]																				

HIP_25540	GSLQPLAL	DGARPQALP	GSLQPLALDGARPQALP		CMGA	3627/247050	0.999	25540	0.830	0.198115	1.2167	0.1667	0	17	-0.0471	0	0	1	[illegible]	
HIP_25541	VELGGGPGA	EDSKRWSKM	VELGGGPGAEDSKRWSKM		CMGA	4435/247050	0.999	25541	0.830	-0.034647	-1.3333	0.5	0	18	-0.9333	0	0	0	[illegible]	
HIP_25542	SHLVEALYL	GRSRPDRSS	SHLVEALYLGRSRPDRSS		SCG1	14945/367830	0.995	25542	0.830	0.257273	0.1	0.3333	2	18	-0.7111	1	1	0	[illegible]	
HIP_25543	GIVEQCCTS	EEEAEAGEE	GIVEQCCTSEEEAEAGEE		CMGA	3167/247050	0.999	25543	0.830	-0.075520	-1.5833	0.1667	-7	18	-0.7222	-7	0	0	[illegible]	
HIP_25544	CGERGFFYT	IAYEDVVGG	CGERGFFYTIAYEDVVGG		SCG2	3142/334890	1.000	25544	0.830	-0.072774	0.9667	0	-2	18	0.1611	-2	4	0	[illegible]	
HIP_25545	CGERGFFY	ENENTKFE	CGERGFFYENENTKFE		SCG1	11980/367830	0.997	25545	0.830	0.155166	-1.0333	0	-2	16	-1.3312	-2	3	0	[illegible]	
HIP_25546	HLCGSHLVE	NENTKFEVR	HLCGSHLVENENTKFEVR		SCG1	21999/367830	0.986	25546	0.830	0.188734	-1	0	1	18	-0.7167	-1	0	0	[illegible]	
HIP_25547	EAEDLQVGQ	QEEERLSKE	EAEDLQVGQQEEERLSKE		CMGA	1984/247050	0.999	25547	0.830	-0.114816	-1.7	0.1667	-5	18	-1.7222	-5	0	0	[illegible]	
HIP_25548	EAEDLQVGQ	EQEEERLSK	EAEDLQVGQEQEEERLSK		CMGA	1985/247050	0.999	25548	0.830	-0.114816	-1.7	0.1667	-5	18	-1.7222	-5	0	0	[illegible]	
HIP_25549	LCGSHLVEA	LVKYPEIIN	LCGSHLVEALVKYPEIIN		SCG2	12466/334890	0.998	25549	0.830	-0.015367	1.1	0	0	18	0.6333	-1	1	0	[illegible]	
HIP_25550	GIVEQCCTS	EEEEEEEEE	GIVEQCCTSEEEEEEEEE		CMGA	1537/247050	1.000	25550	0.830	-0.066571	-1.5833	0.1667	-10	18	-1.4833	-10	0	0	[illegible]	
HIP_25551	HLVEALYL	EEEEEATN	HLVEALYLEEEEEATN		CMGA	4370/247050	0.999	25551	0.830	0.131014	-0.7	0	-5	16	-0.6563	-6	1	0	[illegible]	
HIP_25552	GSLQPLAL	FLNERKQA	GSLQPLALFLNERKQA		SCG1	14588/367830	0.995	25552	0.830	0.089822	2.0833	0	1	16	-0.225	1	1	0	[illegible]	
HIP_25553	LCGSHLVEA	EEEEEEATN	LCGSHLVEAEEEEEEATN		CMGA	4171/247050	0.999	25553	0.830	0.047443	-1.3333	0	-6	18	-0.8444	-7	0	0	[illegible]	
HIP_25554	SHLVEALYL	PIESHQVEK	SHLVEALYLPIESHQVEK		IAPP	772/45018	0.997	25554	0.830	0.177161	0.95	0.1667	0	18	-0.15	-2	1	0	[illegible]	
HIP_25555	QCCTSICSL	RQYWDEDLL	QCCTSICSLRQYWDEDLL		SCG2	20059/334890	0.996	25555	0.830	0.077068	-0.6333	0.1667	-2	18	-0.1722	-2	2	0	[illegible]	
HIP_25556	SLYQLENYC	SDTREEKRF	SLYQLENYCSDTREEKRF		SCG1	10453/367830	0.998	25556	0.830	0.118669	-1.2167	0.1667	-1	18	-1.4389	-1	1	0	[illegible]	
HIP_25557	PGAGSLQPL	QRYGKRSSP	PGAGSLQPLQRYGKRSSP		NPY	4463/49410	0.946	25557	0.830	0.067503	-1.7667	0.1667	3	18	-1.1222	3	1	0	[illegible]	
HIP_25558	VCGERGFFY	QREDEEEE	VCGERGFFYQREDEEEE		SCG1	13204/367830	0.996	25558	0.830	0.162528	-1.2	0	-5	17	-1.5765	-5	3	1	[illegible]	
HIP_25559	CGERGFFY	RTRPRHHHG	CGERGFFYRTRPRHHHG		SCG1	28844/367830	0.969	25559	0.830	0.220977	-0.9	0	6	17	-1.6353	3	3	1	[illegible]	
HIP_25560	RGFFYTPKT	VNWGYEKRN	RGFFYTPKTVNWGYEKRN		SCG1	10801/367830	0.997	25560	0.830	0.062382	-1.0667	0.1667	3	18	-1.3778	3	2	0	[illegible]	
HIP_25561	GERGFFYTP	PSDSQVSEE	GERGFFYTPPSDSQVSEE		SCG1	1487/367830	1.000	25561	0.830	-0.067273	-1.5833	0.5	-3	18	-1.1444	-3	3	0	[illegible]	
HIP_25562	LVCGERGFF	KELENLAAM	LVCGERGFFKELENLAAM		SCG1	15741/367830	0.994	25562	0.830	0.045762	0.2667	0.1667	-1	18	0.3333	-1	2	0	[illegible]	
HIP_25563	SHLVEALYL	FYDSEEPV	SHLVEALYLFYDSEEPV		SCG1	11813/367830	0.997	25563	0.830	0.052726	0.7167	0	-3	17	0.0824	-4	3	1	[illegible]	
HIP_25564	SHLVEALYL	MQFPPMYE	SHLVEALYLMQFPPMYE		SCG2	10896/334890	0.999	25564	0.830	0.066986	1.25	0	-1	17	0.2176	-2	2	1	[illegible]	
HIP_25565	RGFFYTPKT	GEEEEEEEE	RGFFYTPKTGEEEEEEEE		CMGA	1900/247050	0.999	25565	0.830	-0.088070	-2.2667	0.3333	-6	18	-1.9944	-6	1	0	[illegible]	
HIP_25566	TSICSLYQ	AASFQRNQL	TSICSLYQAASFQRNQL		SCG2	13532/334890	0.998	25566	0.830	-0.032866	0.3	0.1667	1	17	-0.1118	1	2	1	[illegible]	
HIP_25567	LYQLENYCN	THSREKSSQ	LYQLENYCNTHSREKSSQ		SCG1	22585/367830	0.985	25567	0.830	0.040796	-1.1667	0.1667	1	18	-1.5667	0	1	0	[illegible]	
HIP_25568	LVEALYLVC	LDRNYLNYG	LVEALYLVCLDRNYLNYG		SCG1	13855/367830	0.996	25568	0.830	0.130471	1.05	0	-1	18	0.4944	-1	1	0	[illegible]	
HIP_25569	LCGSHLVEA	EEEEMAVVP	LCGSHLVEAEEEEMAVVP		CMGA	5965/247050	0.998	25569	0.830	0.074392	-1.3333	0	-4	18	0.2611	-5	0	0	[illegible]	
HIP_25570	ICSLYQLEN	EDSKEAEKS	ICSLYQLENEDSKEAEKS		CMGA	10906/247050	0.994	25570	0.830	0.030930	-1.8333	0.1667	-3	18	-1.0889	-3	1	0	[illegible]	
HIP_25571	SHLVEALYL	RVQENQMDK	SHLVEALYLRVQENQMDK		SCG1	23437/367830	0.984	25571	0.830	0.204055	0.4167	0	0	18	-0.6222	-1	1	0	[illegible]	
HIP_25572	VCGERGFF	NNQKRERM	VCGERGFFNNQKRERM		SCG2	17898/334890	0.997	25572	0.830	0.117871	-0.8833	0.1667	2	16	-1.3438	2	2	0	[illegible]	
HIP_25573	SLYQLENYC	KSGYPKTPG	SLYQLENYCKSGYPKTPG		SCG2	9132/334890	0.999	25573	0.830	0.158696	-1.2333	0.3333	1	18	-1.0222	1	2	0	[illegible]	
HIP_25574	SHLVEALY	LKRQYDRVA	SHLVEALYLKRQYDRVA		SCG1	30067/367830	0.965	25574	0.830	0.191203	-0.05	0	2	17	-0.3882	1	2	1	[illegible]	
HIP_25575	LVEALYLVC	ELESLSAIE	LVEALYLVCELESLSAIE		CMGA	2854/247050	0.999	25575	0.830	0.071439	1.2167	0	-4	18	1.1722	-4	1	0	[illegible]	
HIP_25576	LYLVCGERG	QENQMDKAR	LYLVCGERGQENQMDKAR		SCG1	21862/367830	0.987	25576	0.830	-0.062286	-3.15	0.1667	0	18	-1	0	0	0	[illegible]	
HIP_25577	VCGERGFF	EESEEGEED	VCGERGFFEESEEGEED		SCG1	3902/367830	0.999	25577	0.830	0.029950	-0.4333	0.3333	-7	17	-1.3059	-7	2	1	[illegible]	
HIP_25578	TSICSLYQL	EKTHSREKS	TSICSLYQLEKTHSREKS		SCG1	32032/367830	0.958	25578	0.830	0.227315	-1.5167	0	2	18	-0.9611	1	1	0	[illegible]	
HIP_25579	CGERGFFY	VLSVALNHL	CGERGFFYVLSVALNHL		IAPP	860/45018	0.997	25579	0.830	-0.005523	1.9167	0.1667	1	17	0.7118	0	3	1	[illegible]	
HIP_25580	LVEALYLVC	HQNLLKELQ	LVEALYLVCHQNLLKELQ		CMGA	10242/247050	0.995	25580	0.830	0.131573	0.05	0	0	18	0.5333	-1	1	0	[illegible]	
HIP_25581	LYQLENYCN	ENHPQESKG	LYQLENYCNENHPQESKG		SCG1	20295/367830	0.989	25581	0.830	0.008531	-2.0833	0	-1	18	-1.6889	-2	1	0	[illegible]	
HIP_25582	TSICSLYQL	NDDTPNRQY	TSICSLYQLNDDTPNRQY		SCG2	14060/334890	0.998	25582	0.830	0.143071	-1.9167	0	-1	18	-1.0056	-1	1	0	[illegible]	

[illegible]

HIP_26051	LVCGERGFF	IVLSVALNH	LVCGERGFFIVLSVALNH		IAPP	113/45018	1.000	26051	0.830	0.014436	2.95	0.1667	1	18	1.2278	0	2	0	0
HIP_26052	QCCTSICSL	YALNSEKNF	QCCTSICSLYALNSEKNF		SCG2	7778/334890	0.999	26052	0.830	0.056963	1.6333	0.1667	0	18	0.1056	0	1	0	0
HIP_26053	LVEALYLVC	HPPASLPSQ	LVEALYLVCHPPASLPSQ		CMGA	5041/247050	0.998	26053	0.830	0.158818	0.6833	0.3333	0	18	0.6556	-1	1	0	0
HIP_26054	VEALYLVC	DLQDTKENR	VEALYLVCDLQDTKENR		SCG1	9862/367830	0.998	26054	0.830	0.139333	1.2167	0	-2	17	-0.4294	-2	1	1	0
HIP_26055	VNQHLCGSH	WERYRGRGS	VNQHLCGSHWERYRGRGS		SCG1	25585/367830	0.979	26055	0.830	0.010679	-2.2167	0.3333	4	18	-1.3833	2	2	0	0
HIP_26056	CSLYQLENY	EYNYDWWEK	CSLYQLENYEYNYDWWEK		SCG1	17234/367830	0.993	26056	0.830	-0.081912	-2.7667	0	-3	18	-1.45	-3	3	0	0
HIP_26057	ALYLVCGER	EEEGPTVVL	ALYLVCGEREEEGPTVVL		CMGA	10852/247050	0.994	26057	0.830	-0.092554	-3.15	0.1667	-3	18	0.3	-3	0	0	1
HIP_26058	HLVEALYLV	KARRHPQGA	HLVEALYLVKARRHPQGA		SCG1	31160/367830	0.961	26058	0.830	0.178177	0.0167	0	4	18	-0.2444	2	1	0	0
HIP_26059	CTSICSLYQ	IVLSVALN	CTSICSLYQIVLSVALN		IAPP	721/45018	0.997	26059	0.830	-0.013319	1.9167	0	0	17	1.4235	0	1	1	0
HIP_26060	CCTSICSLY	KENREEARF	CCTSICSLYKENREEARF		SCG1	22265/367830	0.986	26060	0.830	0.086551	-1.5333	0.1667	0	18	-0.5611	0	1	0	0
HIP_26061	AGSLQPLAL	ESGEETGSQ	AGSLQPLALESGEETGSQ		SCG1	2085/367830	1.000	26061	0.830	0.198573	0.7833	0.3333	-3	18	-0.4667	-3	0	0	0
HIP_26062	AGSLQPLAL	SGEETGSQE	AGSLQPLALSGEETGSQE		SCG1	2086/367830	1.000	26062	0.830	0.198573	0.7833	0.3333	-3	18	-0.4667	-3	0	0	0
HIP_26063	TSICSLYQL	GGGHSRERA	TSICSLYQLGGGHSRERA		SCG1	1675/367830	1.000	26063	0.830	0.164691	-0.3667	0.5	2	18	-0.4667	1	1	0	1
HIP_26064	LQVGQVELG	EEEEEEEEE	LQVGQVELGEEEEEEEEE		CMGA	1131/247050	1.000	26064	0.830	-0.097755	-1.7667	0.1667	-10	18	-1.4889	-10	0	0	7
HIP_26065	CGERGFFY	ADEPQWSL	CGERGFFYADEPQWSL		SCG1	13278/367830	0.996	26065	0.830	0.101805	-0.15	0	-2	16	-0.6375	-2	3	0	0
HIP_26066	NQHLCGSHL	ALAEAYPSK	NQHLCGSHLALAEAYPSK		NPY	1381/49410	0.992	26066	0.830	0.066689	1.2	0.1667	2	18	-0.3556	0	0	0	0
HIP_26067	LVCGERGFF	NREEARFQ	LVCGERGFFNREEARFQ		SCG1	20871/367830	0.988	26067	0.830	0.118846	-1.05	0.1667	0	17	-0.6529	0	2	1	0
HIP_26068	YLVCGERGF	NVPRTRLED	YLVCGERGFNVPRTRLED		NPY	408/49410	0.998	26068	0.830	-0.054211	-0.5	0.3333	0	18	-0.5889	0	1	0	0
HIP_26069	VCGERGFF	GGGHSRER	VCGERGFFGGGHSRER		SCG1	374/367830	1.000	26069	0.830	0.175387	0.6667	0.6667	2	16	-0.8875	1	2	0	1
HIP_26070	LVCGERGFF	GQPRSQEE	LVCGERGFFGQPRSQEE		SCG1	4269/367830	0.999	26070	0.830	0.130708	-0.05	0.5	-1	17	-0.8235	-1	2	1	0
HIP_26071	PGAGSLQPL	EPERELDLP	PGAGSLQPLEPERELDLP		SCG2	4158/334890	1.000	26071	0.830	-0.042646	-1.65	0.3333	-3	18	-0.7222	-3	0	0	0
HIP_26072	SHLVEALYL	AEGNNQAP	SHLVEALYLAEGNNQAP		CMGA	2694/247050	0.999	26072	0.830	0.195545	0.7	0.1667	-1	17	-0.2235	-2	1	1	0
HIP_26073	AGSLQPLAL	LPRLPYGAG	AGSLQPLALLPRLPYGAG		SCG2	11751/334890	0.998	26073	0.830	0.150566	1.1833	0.1667	1	18	0.4611	1	0	0	0
HIP_26074	GERGFFYT	EEKKEEEGS	GERGFFYTEEKKEEEGS		CMGA	12060/247050	0.992	26074	0.830	0.105396	-1.6833	0	-3	17	-1.8647	-3	3	1	1
HIP_26075	GGPGAGSLQ	ENEKDRADQ	GGPGAGSLQENEKDRADQ		SCG1	13640/367830	0.996	26075	0.830	-0.076622	-1.8333	0.1667	-2	18	-1.6389	-2	0	0	0
HIP_26076	GSHLVEALY	RPEDQELES	GSHLVEALYRPEDQELES		CMGA	5797/247050	0.998	26076	0.830	0.171440	-0.8833	0.1667	-3	18	-0.9	-4	1	0	0
HIP_26077	HLVEALYL	SFQRNQLLQ	HLVEALYLSFQRNQLLQ		SCG2	11268/334890	0.999	26077	0.830	0.161476	0.8	0.1667	1	17	0.0294	0	2	1	0
HIP_26078	LVEALYLVC	LGEGHHRVQ	LVEALYLVCLGEGHHRVQ		SCG1	4218/367830	0.999	26078	0.830	0.132535	1.7333	0.1667	1	18	0.4778	-1	1	0	0
HIP_26079	HLVEALYLV	EEEEEMAV	HLVEALYLVEEEEEMAV		CMGA	3551/247050	0.999	26079	0.830	0.114162	-0.6333	0	-5	17	0.2353	-6	1	1	3
HIP_26080	ERGFFYTPK	PQSEESWDE	ERGFFYTPKPQSEESWDE		SCG1	4873/367830	0.999	26080	0.830	-0.141897	-2.0167	0.5	-3	18	-1.7722	-3	2	0	0
HIP_26081	SHLVEALY	HMQFPPMYE	SHLVEALYHMQFPPMYE		SCG2	18136/334890	0.997	26081	0.830	0.090398	-0.0833	0	0	17	-0.1941	-2	2	1	0
HIP_26082	CTSICSLYQ	KDQELGEYL	CTSICSLYQKDQELGEYL		SCG2	19039/334890	0.996	26082	0.830	0.157674	-1.9833	0	-2	18	-0.3222	-2	1	0	0
HIP_26083	HLVEALYLV	EDSKRWSK	HLVEALYLVEDSKRWSK		CMGA	7878/247050	0.997	26083	0.830	0.199419	-0.1833	0.1667	1	17	-0.4824	0	1	1	0
HIP_26084	LVEALYLVC	TATCATQRL	LVEALYLVCTATCATQRL		IAPP	1182/45018	0.995	26084	0.830	0.111703	1.8167	0	0	18	1.0611	0	1	0	0
HIP_26085	CTSICSLYQ	GEYLARMLV	CTSICSLYQGEYLARMLV		SCG2	7965/334890	0.999	26085	0.830	0.032691	-1.0333	0.1667	0	18	0.6667	0	2	0	0
HIP_26086	SICSLYQLE	ESKDQLSDD	SICSLYQLEESKDQLSDD		SCG2	15872/334890	0.997	26086	0.830	-0.058716	-1.9	0.1667	-4	18	-0.8056	-4	1	0	0
HIP_26087	CGERGFFY	KWQQQGDLQ	CGERGFFYKWQQQGDLQ		SCG1	22739/367830	0.985	26087	0.830	0.153895	-0.6667	0	0	17	-1.2294	0	4	1	1
HIP_26088	LVEALYLVC	FLVHSSNN	LVEALYLVCFLVHSSNN		IAPP	1092/45018	0.996	26088	0.830	-0.000530	3.55	0	0	17	1.0765	-1	2	1	0
HIP_26089	CGERGFFYT	EALAVDGAG	CGERGFFYTEALAVDGAG		CMGA	6151/247050	0.998	26089	0.830	-0.069421	0.4833	0	-2	18	0.1611	-2	3	0	0
HIP_26090	QHLCGSHLV	GWQAKREE	QHLCGSHLVGWQAKREE		CMGA	7224/247050	0.997	26090	0.830	0.207586	0	0.1667	2	17	-0.8941	0	1	1	0
HIP_26091	SICSLYQL	EESWDEEDK	SICSLYQLEESWDEEDK		SCG1	6183/367830	0.999	26091	0.830	0.041655	-1.4667	0.1667	-5	17	-1.0824	-5	2	1	0
HIP_26092	SHLVEALYL	GPQLRRGW	SHLVEALYLGPQLRRGW		CMGA	5862/247050	0.998	26092	0.830	0.236103	0.1333	0.3333	2	17	-0.2	1	1	1	0
HIP_26093	CCTSICSLY	DKETTENEN	CCTSICSLYDKETTENEN		SCG1	13872/367830	0.996	26093	0.830	0.032441	-1.5333	0.1667	-3	18	-0.7833	-3	1	0	0
[illegible]																			

HIP_26562	GERGFFYT	EEEMAVVPQ	GERGFFYTEEEMAVVPQ		CMGA	8851/247050	0.996	26562	0.829	0.067057	-1.6167	0	-3	17	-0.5118	-3	3	1
HIP_26563	HLVEALYL	YPEEKKEEE	HLVEALYLYPEEKKEEE		CMGA	5270/247050	0.998	26563	0.829	0.115500	-0.0167	0.1667	-3	17	-1.1059	-4	2	1
HIP_26564	EDLQVGQVE	DWWEKKPFS	EDLQVGQVEDWWEKKPFS		SCG1	11571/367830	0.997	26564	0.829	-0.113091	-1.35	0	-3	18	-1.2167	-3	2	0
HIP_26565	GSHLVEALY	WGYEKRNL	GSHLVEALYWGYEKRNL		SCG1	11909/367830	0.997	26565	0.829	0.085657	0.2833	0.1667	1	17	-0.5765	0	3	1
HIP_26566	VEALYLVC	ESGEETGSQ	VEALYLVCESGEETGSQ		SCG1	3581/367830	0.999	26566	0.829	0.199250	0.9667	0.3333	-4	17	-0.0941	-4	1	1
HIP_26567	LVEALYLV	SAEFPDFYD	LVEALYLVSAEFPDFYD		SCG1	3229/367830	1.000	26567	0.829	0.092094	0.7	0.1667	-4	17	0.5882	-4	2	1
HIP_26568	VCGERGFF	DRNYLNYGE	VCGERGFFDRNYLNYGE		SCG1	17322/367830	0.993	26568	0.829	0.081224	-1.05	0.1667	-1	17	-0.8353	-1	3	1
HIP_26569	ICSLYQLEN	TDKLAPVSK	ICSLYQLENTDKLAPVSK		SCG2	32063/334890	0.988	26569	0.829	0.039929	-1.8833	0	0	18	-0.1444	0	1	0
HIP_26570	GIVEQCCTS	GEQEHSQQK	GIVEQCCTSGEQEHSQQK		CMGA	3350/247050	0.999	26570	0.829	0.008518	-1.0667	0.3333	-1	18	-1.1667	-2	0	0
HIP_26571	LVCGERGFF	NGSVEPERE	LVCGERGFFNGSVEPERE		SCG2	105/334890	1.000	26571	0.829	0.096765	0.0833	0.5	-2	18	-0.5444	-2	2	0
HIP_26572	AGSLQPLAL	GFEDELSE	AGSLQPLALGFEDELSE		CMGA	69/247050	1.000	26572	0.829	0.153835	1.3833	0.1667	-4	17	0.0059	-4	1	1
HIP_26573	TSICSLYQL	EDAPAEDMA	TSICSLYQLEDAPAEDMA		NPY	1296/49410	0.993	26573	0.829	-0.003908	-1.0333	0	-4	18	-0.0444	-4	1	0
HIP_26574	GSLQPLALE	TRPRHHHGR	GSLQPLALETRPRHHHGR		SCG1	28784/367830	0.970	26574	0.829	0.179682	-0.7833	0.1667	5	18	-1.2444	2	0	0
HIP_26575	GSHLVEALY	EEEGPTVV	GSHLVEALYEEEGPTVV		CMGA	5191/247050	0.998	26575	0.829	0.109448	-1.0333	0	-3	17	-0.0235	-4	1	1
HIP_26576	GERGFFYTP	VGSNTYGKR	GERGFFYTPVGSNTYGKR		IAPP	649/45018	0.998	26576	0.829	-0.117721	-0.1	0.5	2	18	-1.0056	2	3	0
HIP_26577	AGSLQPLAL	DPADASEAH	AGSLQPLALDPADASEAH		SCG1	1959/367830	1.000	26577	0.829	0.142990	1.0167	0.1667	-2	18	-0.1111	-3	0	0
HIP_26578	GSHLVEALY	SEESWDEED	GSHLVEALYSEESWDEED		SCG1	1664/367830	1.000	26578	0.829	0.111079	-0.5833	0.1667	-6	18	-1.0611	-7	1	0
HIP_26579	QCCTSICSL	EGGGHSRER	QCCTSICSLEGGGHSRER		SCG1	2182/367830	1.000	26579	0.829	0.191192	0.2	0.5	1	18	-0.6222	0	0	0
HIP_26580	AGSLQPLAL	LPSEEKGHP	AGSLQPLALLPSEEKGHP		SCG1	2208/367830	1.000	26580	0.829	0.161917	1.8	0.3333	0	18	-0.3333	-1	0	0
HIP_26581	PGAGSLQPL	ALAEAYPSK	PGAGSLQPLALAEAYPSK		NPY	67/49410	1.000	26581	0.829	0.136084	1.0167	0.1667	0	18	-0.0444	0	0	0
HIP_26582	VCGERGFF	EEPVSTHQ	VCGERGFFEEPVSTHQ		SCG1	7966/367830	0.998	26582	0.829	0.054438	-0.5667	0.3333	-1	16	-0.5688	-2	2	0
HIP_26583	CGERGFFYT	APPITPECR	CGERGFFYTAPPITPECR		SCG1	1905/367830	1.000	26583	0.829	0.005052	-0.1	0.3333	0	18	-0.4111	0	3	0
HIP_26584	CCTSICSLY	TYGKRNAVE	CCTSICSLYTYGKRNAVE		IAPP	866/45018	0.997	26584	0.829	0.006752	-0.1167	0.3333	1	18	0.0222	1	2	0
HIP_26585	LVCGERGFF	SLEAGLPLQ	LVCGERGFFSLEAGLPLQ		CMGA	2580/247050	0.999	26585	0.829	0.007333	0.7833	0.3333	-1	18	0.5944	-1	2	0
HIP_26586	LVEALYLVC	LNSEKNFPM	LVEALYLVCLNSEKNFPM		SCG2	5740/334890	0.999	26586	0.829	0.121195	1.6667	0.1667	-1	18	0.6111	-1	1	0
HIP_26587	SHLVEALYL	LAVDGAGKP	SHLVEALYLLAVDGAGKP		CMGA	6551/247050	0.998	26587	0.829	-0.003985	2.6833	0	0	18	0.5778	-1	1	0
HIP_26588	LVEALYLVC	KADTEKWAE	LVEALYLVCKADTEKWAE		SCG1	14880/367830	0.995	26588	0.829	0.149925	0.8167	0	-2	18	0.1667	-2	1	0
HIP_26589	AGSLQPLAL	GDSEGLSQG	AGSLQPLALGDSEGLSQG		CMGA	2239/247050	0.999	26589	0.829	0.209253	0.7833	0.3333	-2	18	-0.0444	-2	0	0
HIP_26590	GERGFFYTP	LARVPKLDL	GERGFFYTPLARVPKLDL		SCG1	5836/367830	0.999	26590	0.829	-0.088797	-0.4167	0.1667	1	18	-0.1611	1	3	0
HIP_26591	AGSLQPLAL	SLQPLALEG	AGSLQPLALSLQPLALEG		INSC	108/5184	0.998	26591	0.829	0.177131	1.4833	0.1667	-1	18	0.6722	-1	0	0
HIP_26592	CGERGFFY	GDTEVMKC	CGERGFFYGDTEVMKC		CMGA	5927/247050	0.998	26592	0.829	0.200554	-0.05	0.1667	-1	16	-0.3375	-1	3	0
HIP_26593	SICSLYQL	DTEKWAEGG	SICSLYQLDTEKWAEGG		SCG1	13355/367830	0.996	26593	0.829	0.065019	-1.45	0	-2	17	-0.4	-2	2	1
HIP_26594	SICSLYQLE	KSSHFERRD	SICSLYQLEKSSHFERRD		SCG1	23105/367830	0.984	26594	0.829	0.009581	-1.45	0.3333	1	18	-0.9556	0	1	0
HIP_26595	VEALYLVC	DLEWERYR	VEALYLVCDLEWERYR		SCG1	10519/367830	0.997	26595	0.829	0.100627	1.2167	0	-2	16	-0.15	-2	2	0
HIP_26596	LVCGERGFF	FPSPEMIRA	LVCGERGFFFPSPEMIRA		SCG2	1315/334890	1.000	26596	0.829	0.017730	0.9333	0.5	0	18	0.35	0	3	0
HIP_26597	PGAGSLQPL	EEEEEEEEE	PGAGSLQPLEEEEEEEEE		CMGA	1637/247050	1.000	26597	0.829	-0.066236	-1.9667	0.1667	-9	18	-1.6889	-9	0	0
HIP_26598	HLVEALYLV	QSEESWDEE	HLVEALYLVQSEESWDEE		SCG1	3393/367830	1.000	26598	0.829	0.158537	-0.1833	0.1667	-5	18	-0.55	-6	1	0
HIP_26599	SLYQLENYC	NNQAPGEEE	SLYQLENYCNNQAPGEEE		CMGA	5161/247050	0.998	26599	0.829	0.019390	-2.1333	0	-4	18	-1.3889	-4	1	0
HIP_26600	GSHLVEALY	LTEDEKKEL	GSHLVEALYLTEDEKKEL		SCG1	4283/367830	0.999	26600	0.829	0.036212	0.65	0	-2	18	-0.5833	-3	1	0
HIP_26601	TSICSLYQL	QWSLYPSDS	TSICSLYQLQWSLYPSDS		SCG1	10977/367830	0.997	26601	0.829	0.031644	-1.0333	0.1667	-1	18	-1.1056	-1	2	0
HIP_26602	VCGERGFF	PGETQNAFL	VCGERGFFPGETQNAFL		SCG1	2607/367830	1.000	26602	0.829	0.123320	-0.05	0.5	-1	17	-0.0765	-1	2	1
HIP_26603	GSLQPLAL	PRAYFMSDT	GSLQPLALPRAYFMSDT		SCG1	17437/367830	0.993	26603	0.829	0.162019	0.85	0.1667	0	17	0.0588	0	2	1
HIP_26604	TSICSLYQL	SEEEPEYGE	TSICSLYQLSEEEPEYGE		SCG1	4183/367830	0.999	26604	0.829	0.091511	-1.4667	0.1667	-5	18	-0.7833	-5	1	0

HIP_27073	GERGFFYTP	AAGSGRLQN	GERGFFYTPAAGSGRLQN		SCG2	3208/334890	1.000	27073	0.828	-0.100739	-0.0667	0.3333	1	18	-0.6944	1	3	0	0
HIP_27074	QCCTSICSL	QSAPKENKP	QCCTSICSLQSAPKENKP		SCG2	17131/334890	0.997	27074	0.828	0.109179	0.5	0.3333	1	18	-0.5833	1	0	0	0
HIP_27075	AGSLQPLAL	HRVQENQMD	AGSLQPLALHRVQENQMD		SCG1	25589/367830	0.979	27075	0.828	0.104209	0.9833	0	0	18	-0.5778	-1	0	0	0
HIP_27076	HLVEALYLV	ENREEARFQ	HLVEALYLVENREEARFQ		SCG1	16702/367830	0.993	27076	0.828	0.211707	-0.8	0	-1	18	-0.4611	-2	1	0	0
HIP_27077	HLVEALYLV	NREEARFQD	HLVEALYLVNREEARFQD		SCG1	16703/367830	0.993	27077	0.828	0.211707	-0.8	0	-1	18	-0.4611	-2	1	0	0
HIP_27078	AEDLQVGQV	EDNRDSSMK	AEDLQVGQVEDNRDSSMK		CMGA	4321/247050	0.999	27078	0.828	-0.054375	-1.7	0.1667	-3	18	-1.25	-3	0	0	0
HIP_27079	VCGERGFF	EDATSEVDK	VCGERGFFEDATSEVDK		SCG1	9058/367830	0.998	27079	0.828	0.008821	0	0.1667	-3	17	-0.5824	-3	2	1	0
HIP_27080	YLVCGERGF	QNAFLNERK	YLVCGERGFQNAFLNERK		SCG1	14249/367830	0.995	27080	0.828	-0.008185	-1.2167	0.1667	1	18	-0.6	1	2	0	0
HIP_27081	GSLQPLAL	FLVHSSNNF	GSLQPLALFLVHSSNNF		IAPP	1389/45018	0.994	27081	0.828	0.017281	3.3667	0	1	17	0.5118	0	1	1	0
HIP_27082	AGSLQPLAL	EAEAGEEAV	AGSLQPLALEAEAGEEAV		CMGA	3255/247050	0.999	27082	0.828	0.069006	0.7	0	-4	18	0.2167	-4	0	0	0
HIP_27083	CTSICSLYQ	SGEATDGAR	CTSICSLYQSGEATDGAR		CMGA	1187/247050	1.000	27083	0.828	0.145667	-0.95	0.3333	-1	18	-0.2222	-1	1	0	0
HIP_27084	LCGSHLVEA	MESAANQKT	LCGSHLVEAMESAANQKT		SCG2	16082/334890	0.997	27084	0.828	0.079848	0.0167	0.1667	0	18	-0.1222	-1	0	0	0
HIP_27085	CSLYQLENY	NQSSQAELK	CSLYQLENYNQSSQAELK		CMGA	11825/247050	0.992	27085	0.828	-0.018470	-2.6833	0.1667	-1	18	-0.9833	-1	1	0	0
HIP_27086	AGSLQPLAL	EGPTVVLNP	AGSLQPLALEGPTVVLNP		CMGA	2233/247050	0.999	27086	0.828	0.206601	0.65	0.3333	-1	18	0.5333	-1	0	0	0
HIP_27087	VCGERGFF	EEEPEYGEE	VCGERGFFEEEPEYGEE		SCG1	8266/367830	0.998	27087	0.828	0.026441	-0.8833	0.1667	-6	17	-1.2235	-6	2	1	1
HIP_27088	QHLCGSHLV	GAPGKWQQQ	QHLCGSHLVGAPGKWQQQ		SCG1	5239/367830	0.999	27088	0.828	0.192138	0.7667	0.3333	3	18	-0.7056	1	0	0	1
HIP_27089	GPGAGSLQP	NTKFEVRLL	GPGAGSLQPNTKFEVRLL		SCG1	24594/367830	0.981	27089	0.828	0.018532	-1.5667	0.1667	1	18	-0.2556	1	1	0	0
HIP_27090	GAGSLQPLA	KEWEDSKRW	GAGSLQPLAKEWEDSKRW		CMGA	14596/247050	0.988	27090	0.828	0.025228	-0.7167	0.1667	0	18	-1.1611	0	1	0	0
HIP_27091	HLCGSHLVE	PKLDLKRQY	HLCGSHLVEPKLDLKRQY		SCG1	27841/367830	0.973	27091	0.828	0.102634	0.4667	0.1667	3	18	-0.6333	1	0	0	0
HIP_27092	VCGERGFF	SQETDKLAP	VCGERGFFSQETDKLAP		SCG2	10891/334890	0.999	27092	0.828	0.104603	-0.4333	0.3333	-1	17	-0.4941	-1	2	1	0
HIP_27093	IVEQCCTSI	EEEEEAEAG	IVEQCCTSIEEEEEAEAG		CMGA	2806/247050	0.999	27093	0.828	-0.059686	-1.25	0.1667	-7	18	-0.45	-7	0	0	3
HIP_27094	IVEQCCTSI	EEEEEEATN	IVEQCCTSIEEEEEEATN		CMGA	2803/247050	0.999	27094	0.828	-0.057959	-1.25	0.1667	-7	18	-0.7611	-7	0	0	4
HIP_27095	SHLVEALYL	VDLDDISEA	SHLVEALYLVDLDDISEA		SCG2	2635/334890	1.000	27095	0.828	-0.023651	1.8	0	-4	18	0.45	-5	1	0	0
HIP_27096	VCGERGFFY	WIPHVENR	VCGERGFFYWIPHVENR		SCG2	9097/334890	0.999	27096	0.828	0.058645	1.05	0.1667	1	17	-0.3706	0	4	1	0
HIP_27097	LVEALYLVC	KKEEEGSAN	LVEALYLVCKKEEEGSAN		CMGA	13666/247050	0.990	27097	0.828	0.194940	-0.1333	0	-2	18	-0.1056	-2	1	0	1
HIP_27098	VEALYLVCG	RNYLNYGEE	VEALYLVCGRNYLNYGEE		SCG1	17569/367830	0.992	27098	0.828	0.022500	-0.5	0.1667	-2	18	-0.1444	-2	2	0	0
HIP_27099	VCGERGFF	EEEEDNRDS	VCGERGFFEEEEDNRDS		CMGA	6295/247050	0.998	27099	0.828	0.035371	-0.8833	0.1667	-5	17	-1.5471	-5	2	1	2
HIP_27100	PGAGSLQPL	LSFRARAYG	PGAGSLQPLLSFRARAYG		CMGA	4498/247050	0.999	27100	0.828	-0.005039	0.75	0.3333	2	18	-0.0111	2	1	0	0
HIP_27101	GERGFFYTP	ELVARSETH	GERGFFYTPELVARSETH		SCG1	7434/367830	0.999	27101	0.828	-0.064372	0.15	0.1667	0	18	-0.7333	-1	3	0	0
HIP_27102	HLVEALYLV	GEEENELTL	HLVEALYLVGEEENELTL		SCG1	1818/367830	1.000	27102	0.828	0.158214	-0.1167	0.1667	-4	18	0.1444	-5	1	0	1
HIP_27103	FVNQHLCGS	EEEDNRDSS	FVNQHLCGSEEEDNRDSS		CMGA	4917/247050	0.998	27103	0.828	-0.063590	-1.5333	0.3333	-3	18	-1.4	-4	0	0	1
HIP_27104	LVCGERGFF	LNERKQAS	LVCGERGFFLNERKQAS		SCG1	14677/367830	0.995	27104	0.828	0.038568	0.3333	0.1667	1	17	-0.4	1	2	1	0
HIP_27105	AGSLQPLAL	LIFLISGAE	AGSLQPLALLIFLISGAE		SCG2	7821/334890	0.999	27105	0.828	-0.000165	3.4167	0	-1	18	1.4	-1	1	0	0
HIP_27106	HLVEALYL	DLELQKIAE	HLVEALYLDLELQKIAE		SCG1	6331/367830	0.999	27106	0.828	0.079536	0.5167	0	-2	17	0.3176	-3	1	1	0
HIP_27107	YLVCGERGF	FLVHSSNN	YLVCGERGFFLVHSSNN		IAPP	602/45018	0.998	27107	0.828	0.074762	1.45	0.1667	1	17	0.1294	0	2	1	0
HIP_27108	AGSLQPLAL	GLAAVNSM	AGSLQPLALGLAAVNSM		SCG1	4730/367830	0.999	27108	0.828	0.085698	2.4333	0.1667	0	17	1.0294	0	0	1	0
HIP_27109	VCGERGFFY	SMPVDNRN	VCGERGFFYSMPVDNRN		SCG1	6428/367830	0.999	27109	0.828	0.147674	0.6333	0.3333	0	17	-0.5353	0	3	1	0
HIP_27110	GSHLVEALY	NLNDKDQEL	GSHLVEALYNLNDKDQEL		SCG2	2837/334890	1.000	27110	0.828	0.086069	0.1833	0	-2	18	-0.7167	-3	1	0	0
HIP_27111	LVCGERGFF	APGKWQQQ	LVCGERGFFAPGKWQQQ		SCG1	1323/367830	1.000	27111	0.828	0.057419	0.8333	0.5	1	17	-0.4824	1	2	1	1
HIP_27112	CGSHLVEAL	EEGSANRRP	CGSHLVEALEEGSANRRP		CMGA	6796/247050	0.997	27112	0.828	0.159936	-0.8833	0.1667	0	18	-0.6833	-1	0	0	0
HIP_27113	CGERGFFYT	WLGAALSLI	CGERGFFYTWLGAALSLI		SCG2	2523/334890	1.000	27113	0.828	-0.064225	0.55	0.1667	0	18	0.8167	0	4	0	0
HIP_27114	VELGGGPGA	SKEWEDSKR	VELGGGPGASKEWEDSKR		CMGA	6902/247050	0.997	27114	0.828	-0.019681	-1.4	0.5	-1	18	-1.2333	-1	1	0	1
HIP_27115	VCGERGFF	SRPDRSSQG	VCGERGFFSRPDRSSQG		SCG1	17563/367830	0.992	27115	0.828	0.144968	-0.2833	0.5	1	17	-0.9941	1	2	1	0
HIP_27116	CCTSICSLY	EDSSEEKHL	CCTSICSLYEDSSEEKHL		SCG1	5983/367830	0.999	27116	0.828	0.035707	-1.0167	0.3333	-2	18	-0.3722	-3	1	0	0
HIP_27117	DLQVGQVEL	PYALNSEKN	DLQVGQVELPYALNSEKN		SCG2	9048/334890	0.999	27117	0.828	-0.033071	0.5667	0.1667	-2	18	-0.6056	-2	1	0	0
HIP_27118	ICSLYQLEN	EEERGLEPG	ICSLYQLENEEERGLEPG		SCG1	16964/367830	0.993	27118	0.828	0.007512	-2.2833	0	-4	18	-0.8389	-4	1	0	1
HIP_27119	CGERGFFY	LTLNEKNFF	CGERGFFYLTLNEKNFF		SCG1	12834/367830	0.996	27119	0.828	0.002346	1.8667	0	0	17	-0.2294	0	3	1	0
HIP_27120	ERGFFYTPK	QREDEEEEE	ERGFFYTPKQREDEEEEE		SCG1	12591/367830	0.996	27120	0.828	-0.078153	-2.95	0.1667	-5	18	-2.3778	-5	2	0	3
HIP_27121	SLYQLENYC	ALAEAYPSK	SLYQLENYCALAEAYPSK		NPY	543/49410	0.997	27121	0.828	-0.043683	0.85	0	-1	18	-0.3167	-1	1	0	0
HIP_27122	RGFFYTPKT	KENREEARF	RGFFYTPKTKENREEARF		SCG1	29284/367830	0.968	27122	0.828	0.021132	-2.85	0.1667	2	18	-1.6556	2	1	0	0
HIP_27123	SHLVEALYL	GRHHRGRGG	SHLVEALYLGRHHRGRGG		SCG1	26041/367830	0.978	27123	0.828	0.244279	-0.3	0.1667	5	18	-0.7167	2	1	0	0
HIP_27124	QHLCGSHLV	EEGAPGKWQ	QHLCGSHLVEEGAPGKWQ		SCG1	7270/367830	0.999	27124	0.828	0.186428	-0.4333	0.1667	1	18	-0.7056	-1	0	0	0
HIP_27125	SHLVEALYL	RGEDSSEEK	SHLVEALYLRGEDSSEEK		SCG1	11112/367830	0.997	27125	0.828	0.233090	-0.35	0.1667	-2	18	-0.8778	-3	1	0	0
HIP_27126	GSHLVEALY	YRGRGSEE	GSHLVEALYYRGRGSEE		SCG1	15292/367830	0.995	27126	0.828	0.164533	-0.3167	0.1667	0	17	-0.8529	-1	2	1	0
HIP_27127	VCGERGFFY	LSVALNHL	VCGERGFFYLSVALNHL		IAPP	930/45018	0.996	27127	0.828	-0.011785	1.9167	0.1667	1	17	0.7118	0	3	1	0
HIP_27128	VCGERGFFY	NNIAYEDV	VCGERGFFYNNIAYEDV		SCG2	9128/334890	0.999	27128	0.828	0.110259	0.3	0	-2	17	-0.1529	-2	3	1	0
HIP_27129	LYQLENYCN	EEYRAPRP	LYQLENYCNEEYRAPRP		SCG1	22932/367830	0.985	27129	0.828	0.038244	-1.7667	0	-1	17	-1.4824	-1	2	1	0
HIP_27130	LVEALYLVC	ESKAEGNNQ	LVEALYLVCESKAEGNNQ		CMGA	8310/247050	0.996	27130	0.828	0.201596	0.3833	0.1667	-2	18	-0.0833	-2	1	0	0

HIP_27584	LCGSHLVEA	WDEEDKRNY	LCGSHLVEAWDEEDKRNY		SCG1	10228/367830	0.991	27584	0.828	0.074033	-0.9	0	-2	18	-1.1056	-3	[illegible]
HIP_27585	ERGFFYTPK	APEDLEWER	ERGFFYTPKAPEDLEWER		SCG1	7344/367830	0.999	27585	0.828	-0.128866	-1.5833	0.3333	-2	18	-1.4278	-2	[illegible]
HIP_27586	AGSLQPLAL	QWSLYPSD	AGSLQPLALQWSLYPSD		SCG1	11743/367830	0.997	27586	0.828	0.183887	0.7	0.1667	-1	17	0.0059	-1	[illegible]
HIP_27587	VEALYLVC	LFQNRMLSK	VEALYLVCLFQNRMLSK		SCG2	15771/334890	0.997	27587	0.828	0.096437	2.2667	0	1	17	0.6824	1	[illegible]
HIP_27588	SHLVEALYL	DGARFQAL	SHLVEALYLDGARFQAL		CMGA	5692/247050	0.998	27588	0.828	0.219709	0.7	0.1667	0	17	0.1471	-1	[illegible]
HIP_27589	CCTSICSLY	QRNQLLQKE	CCTSICSLYQRNQLLQKE		SCG2	23229/334890	0.995	27589	0.828	0.087950	-1.6333	0.1667	1	18	-0.3389	1	[illegible]
HIP_27590	LVCGERGFF	NPYYDPLQ	LVCGERGFFNPYYDPLQ		SCG1	11933/367830	0.997	27590	0.828	0.039378	-0.2	0.3333	-1	17	-0.3059	-1	[illegible]
HIP_27591	VEALYLVCG	RQQAHKEES	VEALYLVCGRQQAHKEES		SCG2	19585/334890	0.996	27591	0.828	0.098301	-0.8667	0.1667	0	18	-0.5278	-1	[illegible]
HIP_27592	QHLCGSHLV	NVQKFPSPE	QHLCGSHLVNVQKFPSPE		SCG2	16889/334890	0.997	27592	0.828	0.198998	0.3333	0	2	18	-0.4556	0	[illegible]
HIP_27593	CTSICSLYQ	IFLISGAEA	CTSICSLYQIFLISGAEA		SCG2	4863/334890	1.000	27593	0.828	-0.054499	1.6833	0	-1	18	1.15	-1	[illegible]
HIP_27594	HLVEALYL	SEEKGHPQE	HLVEALYLSEEKGHPQE		SCG1	8993/367830	0.998	27594	0.828	0.176888	-0.25	0.1667	-1	17	-0.8529	-3	[illegible]
HIP_27595	HLCGSHLV	PDFYDSEEP	HLCGSHLVPDFYDSEEP		SCG1	4390/367830	0.999	27595	0.828	0.126014	0.4167	0.1667	-2	17	-0.5765	-4	[illegible]
HIP_27596	CTSICSLYQ	GFRGPGPQL	CTSICSLYQGFRGPGPQL		CMGA	8344/247050	0.996	27596	0.828	0.082617	-0.5167	0.1667	1	18	0.0222	1	[illegible]
HIP_27597	SHLVEALYL	YPSDSQVS	SHLVEALYLYPSDSQVS		SCG1	10262/367830	0.998	27597	0.828	0.190300	0.4333	0.3333	-1	17	0.0294	-2	[illegible]
HIP_27598	LVEALYLV	DWWEKKPFS	LVEALYLVDWWEKKPFS		SCG1	15574/367830	0.994	27598	0.828	0.062944	0.2333	0	-1	17	0.0353	-1	[illegible]
HIP_27599	SHLVEALYL	HGRSRPDRS	SHLVEALYLHGRSRPDRS		SCG1	24113/367830	0.982	27599	0.828	0.241652	-0.3	0.1667	3	18	-0.8444	1	[illegible]
HIP_27600	TSICSLYQL	RRGWRPSSR	TSICSLYQLRRGWRPSSR		CMGA	10090/247050	0.975	27600	0.828	0.217574	-1.7333	0.1667	4	18	-0.8333	4	[illegible]
HIP_27601	PLALEGSLQ	EEKKEEEGS	PLALEGSLQEEKKEEEGS		CMGA	10096/247050	0.995	27601	0.828	-0.072254	-1.9	0.1667	-4	18	-1.2833	-4	[illegible]
HIP_27602	LVEALYLVC	GAILSSTNV	LVEALYLVCGAILSSTNV		IAPP	580/45018	0.998	27602	0.828	0.034369	2.7333	0.1667	-1	18	1.5222	-1	[illegible]
HIP_27603	LVCGERGFF	QGLVDREKG	LVCGERGFFQGLVDREKG		CMGA	1335/247050	1.000	27603	0.828	0.015771	0.85	0.3333	0	18	-0.2444	0	[illegible]
HIP_27604	NQHLCGSHL	ENELTLNEK	NQHLCGSHLENELTLNEK		SCG1	14137/367830	0.995	27604	0.828	0.018010	-1.7833	0.1667	0	18	-1.0556	-2	[illegible]
HIP_27605	CGERGFFYT	FKRTNEIVE	CGERGFFYTFKRTNEIVE		SCG2	23561/334890	0.994	27605	0.828	0.077348	-0.8	0	0	18	-0.6	0	[illegible]
HIP_27606	AGSLQPLAL	RGEDSSEEK	AGSLQPLALRGEDSSEEK		SCG1	10111/367830	0.998	27606	0.828	0.226181	0.1667	0.1667	-2	18	-0.8722	-2	[illegible]
HIP_27607	GIVEQCCTS	DWWEKKPFS	GIVEQCCTSDWWEKKPFS		SCG1	12214/367830	0.997	27607	0.828	0.026108	-0.7167	0.1667	-1	18	-0.6333	-1	[illegible]
HIP_27608	HLVEALYL	SAPKENKPY	HLVEALYLSAPKENKPY		SCG2	6248/334890	0.999	27608	0.828	0.185727	0.95	0.3333	1	17	-0.5235	0	[illegible]
HIP_27609	RGFFYTPKT	DWNPVEEKI	RGFFYTPKTDWNPVEEKI		SCG2	13207/334890	0.998	27609	0.828	0.000967	-2.35	0.1667	0	18	-1.0667	0	[illegible]
HIP_27610	CTSICSLYQ	PDYNPYQGV	CTSICSLYQPDYNPYQGV		SCG2	11400/334890	0.999	27610	0.828	0.040852	-1.2333	0.1667	-1	18	-0.35	-1	[illegible]
HIP_27611	LYQLENYCN	EDAPAEDMA	LYQLENYCNEDAPAEDMA		NPY	910/49410	0.995	27611	0.828	-0.079698	-1.25	0	-5	18	-0.8222	-5	[illegible]
HIP_27612	LVCGERGF	FLVHSSNN	LVCGERGFFLVHSSNN		IAPP	564/45018	0.998	27612	0.828	0.073343	1.45	0.1667	1	16	0.2188	0	[illegible]
HIP_27613	LYLVCGERG	GRSRSNQLP	LYLVCGERGGRSRSNQLP		SCG2	18262/334890	0.997	27613	0.828	-0.039607	-2.35	0.5	2	18	-0.6444	2	[illegible]
HIP_27614	GSHLVEALY	AHKEESSPD	GSHLVEALYAHKEESSPD		SCG2	18898/334890	0.996	27614	0.828	0.015085	-0.1667	0	-1	18	-0.8111	-3	[illegible]
HIP_27615	CGERGFFYT	GNNQAPGE	CGERGFFYTGNNQAPGE		CMGA	7429/247050	0.997	27615	0.828	0.150107	-1.1	0.1667	-1	17	-1.0176	-1	[illegible]
HIP_27616	QHLCGSHLV	LITRQRYGK	QHLCGSHLVLITRQRYGK		NPY	2764/49410	0.979	27616	0.828	0.056607	2.0667	0	5	18	-0.4056	3	[illegible]
HIP_27617	VEALYLVCG	SFRARAYGF	VEALYLVCGSFRARAYGF		CMGA	11286/247050	0.993	27617	0.828	0.060173	0.6333	0.3333	1	18	0.7111	1	[illegible]
HIP_27618	VEALYLVC	EAGLPLQVR	VEALYLVCEAGLPLQVR		CMGA	3825/247050	0.999	27618	0.828	0.186001	1.6	0.1667	-1	17	0.9176	-1	[illegible]
HIP_27619	VEQCCTSIC	EPEYGEEIK	VEQCCTSICEPEYGEEIK		SCG1	3650/367830	0.999	27619	0.828	0.042509	-0.4	0.3333	-4	18	-0.5	-4	[illegible]
HIP_27620	NQHLCGSHL	NNFGAILSS	NQHLCGSHLNNFGAILSS		IAPP	2418/45018	0.986	27620	0.828	0.029413	-0.7333	0.1667	2	18	-0.0333	0	[illegible]
HIP_27621	CGERGFFY	EWEDSKRW	CGERGFFYEWEDSKRW		CMGA	10748/247050	0.994	27621	0.828	0.109965	-0.6	0	-1	16	-1.4688	-1	[illegible]
HIP_27622	CGSHLVEAL	QENHPQESK	CGSHLVEALQENHPQESK		SCG1	20228/367830	0.989	27622	0.828	0.132781	-1.4	0	0	18	-1.0444	-2	[illegible]
HIP_27623	GERGFFYT	TGPNNQKRE	GERGFFYTTGPNNQKRE		SCG2	5935/334890	0.999	27623	0.828	0.153009	-0.3167	0.3333	1	17	-1.7824	1	[illegible]
HIP_27624	SLYQLENYC	SEEYRAPR	SLYQLENYCSEEYRAPR		SCG1	15609/367830	0.994	27624	0.828	0.123909	-1.6833	0.1667	-1	17	-1.2765	-1	[illegible]
HIP_27625	GSHLVEALY	STENVPRT	GSHLVEALYSTENVPRT		NPY	1263/49410	0.993	27625	0.828	0.202767	-0.1167	0.1667	0	17	-0.3941	-1	[illegible]
HIP_27626	HLVEALYL	SVALNHLKA	HLVEALYLSVALNHLKA		IAPP	1276/45018	0.995	27626	0.828	0.036944	1.9167	0.1667	2	17	0.7882	0	[illegible]
HIP_27627	CGERGFFYT	QPTLLLSL	CGERGFFYTQPTLLLSL		SCG1	11930/367830	0.997	27627	0.828	0.127603	-0.8333	0.1667	0	17	0.3471	0	[illegible]
HIP_27628	AGSLQPLAL	IESQTQEEV	AGSLQPLALIESQTQEEV		SCG2	727/334890	1.000	27628	0.828	0.108029	1.6	0.1667	-3	18	-0.0889	-3	[illegible]
HIP_27629	CGSHLVEAL	RATRLFEKP	CGSHLVEALRATRLFEKP		SCG2	30241/334890	0.980	27629	0.828	0.076744	-0.2167	0	2	18	-0.1167	1	[illegible]
HIP_27630	ERGFFYTPK	GKWQQQGDL	ERGFFYTPKGKWQQQGDL		SCG1	11684/367830	0.997	27630	0.828	-0.077820	-1.9	0.3333	1	18	-1.45	1	[illegible]
HIP_27631	HLVEALYL	REDSLEAG	HLVEALYLREDSLEAG		CMGA	10106/247050	0.995	27631	0.828	0.210334	-0.8667	0	-2	16	-0.075	-3	[illegible]
HIP_27632	SLYQLENYC	EDKRNYPSL	SLYQLENYCEDKRNYPSL		SCG1	23611/367830	0.983	27632	0.828	0.131191	-2.2	0	-1	18	-1.2556	-1	[illegible]
HIP_27633	VCGERGFFY	APVSKRFP	VCGERGFFYAPVSKRFP		SCG2	10979/334890	0.999	27633	0.828	0.046763	1.45	0.1667	2	17	-0.0824	2	[illegible]
HIP_27634	LYQLENYCN	GRSRSNQLP	LYQLENYCNGRSRSNQLP		SCG2	10957/334890	0.999	27634	0.828	0.171746	-1.3333	0.3333	1	18	-1.2389	1	[illegible]
HIP_27635	TSICSLYQL	EEEEEGENY	TSICSLYQLEEEEEGENY		SCG1	7581/367830	0.999	27635	0.828	0.048349	-1.9167	0	-6	18	-1.0389	-6	[illegible]
HIP_27636	DLQVGQVEL	EEEEEMAVV	DLQVGQVELEEEEEMAVV		CMGA	4214/247050	0.999	27636	0.828	0.046055	-1	0	-7	18	-0.2111	-7	[illegible]
HIP_27637	GAGSLQPLA	PGKWQQQGD	GAGSLQPLAPGKWQQQGD		SCG1	5115/367830	0.999	27637	0.828	0.137228	-0.3167	0.5	0	18	-0.9278	0	[illegible]
HIP_27638	LYQLENYCN	SSHHTAEKR	LYQLENYCNSSHHTAEKR		SCG1	19338/367830	0.990	27638	0.828	0.068832	-1.1833	0.3333	2	18	-1.4056	0	[illegible]
HIP_27639	VCGERGFF	GEEDIQGP	VCGERGFFGEEDIQGP		SCG1	5839/367830	0.999	27639	0.828	0.08537	-0.3667	0.3333	-3	16	-0.525	-3	[illegible]
[illegible]	[illegible]	[illegible]	[illegible]		[illegible]	[illegible]	[illegible]	[illegible]	[illegible]	[illegible]	[illegible]	[illegible]	[illegible]	[illegible]	[illegible]	[illegible]	[illegible]

HIP_28095	GERGFFYTP	EATNTHPPA	GERGFFYTPEATNTHPPA		CMGA	7251/247050	0.997	28095	0.827	-0.121675	-1	0.1667	0	18	-1	-1	3	0	0	[illegible]
HIP_28096	SHLVEALYL	PVDNRNHNE	SHLVEALYLPVDNRNHNE		SCG1	7889/367830	0.998	28096	0.827	0.179931	0.9	0.1667	0	18	-0.7778	-2	1	0	0	[illegible]
HIP_28097	QHLCGSHLV	SSQETDKLA	QHLCGSHLVSSQETDKLA		SCG2	9037/334890	0.999	28097	0.827	0.230351	-0.05	0.3333	1	18	-0.4389	-1	0	0	0	[illegible]
HIP_28098	GSLQPLALE	EQYTPQSLA	GSLQPLALEEQYTPQSLA		SCG2	21697/334890	0.995	28098	0.827	0.052686	-1.0333	0	-2	18	-0.3278	-2	1	0	0	[illegible]
HIP_28099	EQCCTSICS	EEEEEEAEA	EQCCTSICSEEEEEEAEA		CMGA	2045/247050	0.999	28099	0.827	-0.010120	-0.7167	0.1667	-8	18	-1.0111	-8	0	0	4	[illegible]
HIP_28100	SICSLYQLE	KEWEDSKRW	SICSLYQLEKEWEDSKRW		CMGA	19597/247050	0.973	28100	0.827	-0.044418	-1.9167	0	-1	18	-1.15	-1	2	0	0	[illegible]
HIP_28101	VCGERGFFY	AGTEALPDG	VCGERGFFYAGTEALPDG		SCG2	137/334890	1.000	28101	0.827	0.129965	0.8333	0.1667	-2	18	-0.0278	-2	3	0	0	[illegible]
HIP_28102	LVEALYLVC	NKRLGLSGL	LVEALYLVCNKRLGLSGL		NPY	3172/49410	0.973	28102	0.827	0.175674	-0.2333	0	1	18	0.9556	1	1	0	0	[illegible]
HIP_28103	LVEALYLVC	PGQGSSEDD	LVEALYLVCPGQGSSEDD		SCG2	1822/334890	1.000	28103	0.827	0.177936	0.8333	0.3333	-4	18	0.0722	-4	1	0	0	[illegible]
HIP_28104	CCTSICSLY	EGENYQKGE	CCTSICSLYEGENYQKGE		SCG1	3226/367830	1.000	28104	0.827	0.058480	-0.95	0.3333	-2	18	-0.6278	-2	1	0	0	[illegible]
HIP_28105	AGSLQPLAL	GIQEEDLRK	AGSLQPLALGIQEEDLRK		SCG2	3375/334890	1.000	28105	0.827	0.189181	1.6667	0.1667	-1	18	-0.3222	-1	0	0	0	[illegible]
HIP_28106	CGERGFFYT	GIQEEDLRK	CGERGFFYTGIQEEDLRK		SCG2	1283/334890	1.000	28106	0.827	0.028185	0.2333	0.1667	-1	18	-0.9556	-1	3	0	0	[illegible]
HIP_28107	HLVEALYLV	ENVPRTRLE	HLVEALYLVENVPRTRLE		NPY	1433/49410	0.992	28107	0.827	0.152199	0.65	0	0	18	-0.0111	-1	1	0	0	[illegible]
HIP_28108	CTSICSLY	NDNFLEGEE	CTSICSLYNDNFLEGEE		SCG1	10137/367830	0.998	28108	0.827	0.038332	-1.4667	0.1667	-4	17	-0.3	-4	2	1	0	[illegible]
HIP_28109	LCGSHLVEA	LNQGSSQET	LCGSHLVEALNQGSSQET		SCG2	14725/334890	0.998	28109	0.827	0.015955	-0.1167	0	-1	18	-0.2611	-2	0	0	0	[illegible]
HIP_28110	QHLCGSHLV	YPGPQAEGD	QHLCGSHLVYPGPQAEGD		CMGA	1089/247050	1.000	28110	0.827	0.183096	0.25	0.3333	0	18	-0.6	-2	1	0	0	[illegible]
HIP_28111	FVNQHLCGS	PQSAPKENK	FVNQHLCGSPQSAPKENK		SCG2	8407/334890	0.999	28111	0.827	-0.040786	-0.7667	0.6667	2	18	-1.0333	1	0	0	0	[illegible]
HIP_28112	GERGFFYTP	SLPSEEKGH	GERGFFYTPSLPSEEKGH		SCG1	3048/367830	1.000	28112	0.827	-0.137045	-0.3667	0.5	0	18	-1.15	-1	3	0	0	[illegible]
HIP_28113	GSLQPLAL	CGERGFFYT	GSLQPLALCGERGFFYT		INSB	79/4968	0.995	28113	0.827	0.199957	1.3333	0.1667	0	17	0.2471	0	0	1	0	[illegible]
HIP_28114	HLVEALYL	EPQSAPKEN	HLVEALYLEPQSAPKEN		SCG2	7611/334890	0.999	28114	0.827	0.187483	-0.3833	0.1667	-1	17	-0.6294	-2	1	1	0	[illegible]
HIP_28115	GSHLVEALY	LNSEKNFPM	GSHLVEALYLNSEKNFPM		SCG2	4110/334890	1.000	28115	0.827	0.096397	0.6333	0.1667	0	18	-0.2167	-1	1	0	0	[illegible]
HIP_28116	HLVEALYLV	PLDSQSIYQ	HLVEALYLVPLDSQSIYQ		SCG2	1940/334890	1.000	28116	0.827	0.140987	0.9	0.1667	-1	18	0.3833	-2	1	0	0	[illegible]
HIP_28117	LCGSHLVEA	APKENKPYA	LCGSHLVEAAPKENKPYA		SCG2	23795/334890	0.994	28117	0.827	0.106525	-0.2	0.1667	1	18	-0.4167	0	0	0	0	[illegible]
HIP_28118	VCGERGFF	EEEEEEEAE	VCGERGFFEEEEEEEAE		CMGA	4159/247050	0.999	28118	0.827	0.014558	-0.8833	0.1667	-8	17	-1.3353	-8	2	1	5	[illegible]
HIP_28119	GGPGAGSLQ	GERGFFYTP	GGPGAGSLQGERGFFYTP		INSB	214/4968	0.970	28119	0.827	-0.007167	-1.4833	0.3333	0	18	-0.4833	0	0	0	0	[illegible]
HIP_28120	VNQHLCGSH	EEEAEAGEE	VNQHLCGSHEEEAEAGEE		CMGA	5094/247050	0.998	28120	0.827	-0.138056	-2.4833	0.3333	-4	18	-1.2167	-6	0	0	1	[illegible]
HIP_28121	CGERGFFYT	LAEAYPSKP	CGERGFFYTLAEAYPSKP		NPY	2043/49410	0.987	28121	0.827	-0.059348	0.4833	0	0	18	-0.4444	0	3	0	0	[illegible]
HIP_28122	HLVEALYL	EDSKEAEKS	HLVEALYLEDSKEAEKS		CMGA	5800/247050	0.998	28122	0.827	0.176507	-0.25	0.1667	-2	17	-0.7176	-3	1	1	0	[illegible]
HIP_28123	LVEALYLVC	EEEGPTVV	LVEALYLVCEEEGPTVV		CMGA	5781/247050	0.998	28123	0.827	0.130440	0	0	-4	17	0.8529	-4	1	1	1	[illegible]
HIP_28124	HLVEALYLV	ASEAHESSS	HLVEALYLVASEAHESSS		SCG1	3111/367830	1.000	28124	0.827	0.116625	0.7	0.1667	-1	18	0.2111	-3	1	0	1	[illegible]
HIP_28125	GSHLVEALY	GEDAPAEDM	GSHLVEALYGEDAPAEDM		NPY	183/49410	0.999	28125	0.827	0.137299	-0.5167	0.1667	-4	18	-0.3389	-5	1	0	0	[illegible]
HIP_28126	CCTSICSLY	RRTRPRHHH	CCTSICSLYRRTRPRHHH		SCG1	35009/367830	0.945	28126	0.827	0.121363	-1.3333	0.1667	7	18	-0.9833	4	1	0	1	[illegible]
HIP_28127	VCGERGFFY	GPQAEGDS	VCGERGFFYGPQAEGDS		CMGA	4732/247050	0.999	28127	0.827	0.180730	-0.2	0.3333	-2	17	-0.5706	-2	3	1	0	[illegible]
HIP_28128	VCGERGFF	GEETGSQE	VCGERGFFGEETGSQE		SCG1	5840/367830	0.999	28128	0.827	0.082109	-0.3667	0.3333	-3	16	-0.8	-3	2	0	0	[illegible]
HIP_28129	LVCGERGFF	ASEEEPEYG	LVCGERGFFASEEEPEYG		SCG1	1364/367830	1.000	28129	0.827	-0.022117	0.45	0.3333	-4	18	-0.5	-4	2	0	1	[illegible]
HIP_28130	VEALYLVC	LGEKRDHHS	VEALYLVCLGEKRDHHS		SCG1	8979/367830	0.998	28130	0.827	0.209167	1.7333	0.1667	1	17	-0.2176	-1	1	1	0	[illegible]
HIP_28131	GSHLVEALY	EEGSANRR	GSHLVEALYEEGSANRR		CMGA	5366/247050	0.998	28131	0.827	0.183596	-0.5167	0.1667	0	17	-0.8529	-1	1	1	0	[illegible]
HIP_28132	LVEALYLVC	SPMPVSQEC	LVEALYLVCSPMPVSQEC		CMGA	1346/247050	1.000	28132	0.827	0.144032	1.6667	0.3333	-2	18	0.8944	-2	1	0	0	[illegible]
HIP_28133	LVEALYLVC	PEPMQESKA	LVEALYLVCPEPMQESKA		CMGA	3073/247050	0.999	28133	0.827	0.178593	0.6333	0.3333	-2	18	0.2556	-2	1	0	0	[illegible]
HIP_28134	LVCGERGFF	KVLEYLNQE	LVCGERGFFKVLEYLNQE		SCG2	4941/334890	1.000	28134	0.827	-0.011760	1.55	0.1667	-1	18	-0.0056	-1	2	0	0	[illegible]
HIP_28135	HLCGSHLVE	FSEDVNWGY	HLCGSHLVEFSEDVNWGY		SCG1	11137/367830	0.997	28135	0.827	0.030203	0.5	0.1667	-1	18	-0.2056	-3	1	0	0	[illegible]
HIP_28136	CGSHLVEAL	WIPHVENRQ	CGSHLVEALWIPHVENRQ		SCG2	7062/334890	0.999	28136	0.827	-0.054642	0.6833	0.1667	1	18	-0.2111	-1	1	0	0	[illegible]
HIP_28137	GSHLVEALY	TLESVFQEL	GSHLVEALYTLESVFQEL		SCG2	3341/334890	1.000	28137	0.827	0.063744	0.65	0	-2	18	0.3889	-3	1	0	0	[illegible]

HIP_28606	CGERGFFY	EADLDHPDL	CGERGFFYEADLDHPDL		SCG2	11935/334890	0.998	28606	0.827	0.098327	-0.15	0	-3	17	-0.6706	-4	3	1	0	0.000000	-3.1667	-2	0.1	0.450000	0.3333	0.1167	0	0	0	0	6.3333	-0.0175	-9.5	0	1	0	0	0.3436	1.2338	0.7356	0.1667	0.1181	0.5412	-0.0515	0.01	-0.3167
HIP_28607	GSLQPLALE	LQNGQNGER	GSLQPLALELQNGQNGER		SCG2	17470/334890	0.997	28607	0.827	-0.026403	-0.1833	0	-1	18	-0.8667	-1	0	0	0	0.000000	-1.7667	1	0.05	1.900000	0.1111	1.7889	0	0	0	0	-1.7667	-0.328	0	0	0	1	0	0.4	1.2233	0.8233	0.3333	0.16	0.6779	-0.0733	0.0025	-0.0883
HIP_28608	GSLQPLAL	GGEDWNPVE	GSLQPLALGGEDWNPVE		SCG2	3030/334890	1.000	28608	0.827	0.202155	0.85	0.3333	-3	17	-0.3647	-3	1	1	0	0.000000	-4.5667	-1	0.2167	0.950000	0.2222	0.7278	0	2	0	1	4.5667	0.6186	-4.5667	1	0	0	0	0.4333	1.0933	0.9	0.3333	0.1878	0.81	0.3683	0.0469	-0.9894
HIP_28609	VEALYLVC	EGEEEENELT	VEALYLVCEGEEEENELT		SCG1	3585/367830	0.999	28609	0.827	0.157568	0.5167	0.1667	-6	17	-0.1647	-6	1	1	1	0.000000	-5.9667	-2	0.1833	0.625000	0.4444	0.1806	0	1	0	1	11.9333	0.0933	-5.9667	1	0	0	0	0.4551	1.1503	0.8145	0	0.2071	0.6634	0.2351	0.0336	-1.0939
HIP_28610	QCCTSICSL	QNGERATRL	QCCTSICSLQNGERATRL		SCG2	10030/334890	0.999	28610	0.827	0.202412	-0.3167	0.3333	1	18	-0.2778	1	0	0	0	0.000000	-4.3	0	0.1167	0.000000	0.1111	-0.1111	0	1	0	1	0	0.0352	0	1	0	0	0	0.5218	0.9003	1.1078	0.1667	0.2723	1.2273	-0.1652	0.0136	-0.5017
HIP_28611	CGSHLVEAL	EIINSNQVK	CGSHLVEALEIINSNQVK		SCG2	14599/334890	0.998	28611	0.827	-0.016607	1.2667	0	0	18	0.15	-1	0	0	0	0.000000	1.1333	0	0	1.400000	0.2222	1.1778	0	0	0	0	0	1.4919	0	1	0	0	0	0.3333	1.3217	0.5967	0.3333	0.1111	0.356	0.4222	0	0
HIP_28612	SLYQLENYC	KENGDESHL	SLYQLENYCKENGDESHL		SCG2	16462/334890	0.997	28612	0.827	0.096477	-2.2	0	-2	18	-1.2389	-3	1	0	0	0.000000	-2.8667	0	0.1	0.950000	0.3333	0.6167	0	0	0	0	0	-1.3567	-2.8667	0	0	0	0	0.5154	0.9858	1.0968	-0.3333	0.2656	1.203	-1.1338	0.01	-0.2867
HIP_28613	AGSLQPLAL	QEEEEDNR	AGSLQPLALQEEEEDNR		CMGA	4908/247050	0.998	28613	0.827	0.135807	-0.1833	0	-4	17	-1.1941	-4	0	1	2	0.000000	-6.6333	-2	0.15	0.450000	0.3333	0.1167	0	0	0	0	13.2667	-0.0214	0	1	0	0	0	0.4	1.3633	0.6867	0.1667	0.16	0.4715	-0.0733	0.0225	-0.995
HIP_28614	VCGERGFF	REDEEEEEG	VCGERGFFREDEEEEEG		SCG1	10780/367830	0.997	28614	0.827	0.049734	-1.05	0.1667	-6	17	-1.5235	-6	2	1	3	0.000000	-5.5667	-1	0.0667	0.700000	0.6667	0.0333	0	1	0	1	5.5667	-0.035	-11.1333	1	0	0	0	0.4269	1.0755	0.8756	-0.1667	0.1823	0.7667	-0.4483	0.0044	-0.3711
HIP_28615	QCCTSICSL	EEEAEAGEE	QCCTSICSLEEEAEAGEE		CMGA	3682/247050	0.999	28615	0.827	0.007595	-0.8333	0.1667	-6	18	-0.4333	-6	0	0	1	0.000000	-5.3333	-3	0.0833	0.450000	0.3333	0.1167	0	0	0	0	16	-0.0972	0	1	0	0	0	0.5051	1.2753	0.8045	-0.3333	0.2552	0.6472	-0.4209	0.0069	-0.4444
HIP_28616	QHLCGSHLV	KEEEGSANR	QHLCGSHLVKEEEGSANR		CMGA	12145/247050	0.992	28616	0.827	0.208394	-1.0167	0	1	18	-1.0333	-1	0	0	1	0.000000	-5.2333	-1	0.15	0.950000	0.4444	0.5056	0	0	0	0	5.2333	-0.514	0	1	0	0	0	0.4103	1.2372	0.7156	-0.3333	0.1683	0.5121	-0.4171	0.0225	-0.785
HIP_28617	QHLCGSHLV	EENSRDNPF	QHLCGSHLVEENSRDNPF		SCG2	10194/334890	0.999	28617	0.827	0.158102	-0.95	0	0	18	-1.0222	-2	0	0	0	0.000000	-5.1	-2	0.15	0.950000	0.2222	0.7278	0	0	0	0	10.2	-0.6914	0	1	0	0	0	0.4385	1.1786	0.8245	-0.1667	0.1922	0.6798	-0.4165	0.0225	-0.765
HIP_28618	LYLVCGERG	NYDWWEKKP	LYLVCGERGNYDWWEKKP		SCG1	16198/367830	0.994	28618	0.827	-0.002654	-2.7833	0.1667	0	18	-1.0444	0	2	0	0	0.000000	0.0333	-1	-0.0833	0.625000	0.3333	0.2917	0	1	0	1	-0.0333	-0.8118	0.0667	0	0	0	1	0.5321	0.9574	1.0868	-0.5	0.2831	1.1811	-1.4809	0.0069	-0.0028
HIP_28619	VCGERGFF	KTSRKDVKD	VCGERGFFKTSRKDVKD		SCG1	23572/367830	0.983	28619	0.827	0.150394	-0.0333	0.3333	2	17	-1	2	2	1	0	0.000000	-3.5333	1	0.0833	0.700000	0.4444	0.2556	0	1	0	1	-3.5333	-0.0085	-7.0667	1	0	0	0	0.4551	0.9253	0.9612	0.1667	0.2071	0.9238	-0.0152	0.0069	-0.2944
HIP_28620	GERGFFYTP	EEAEAGEEA	GERGFFYTPEEAEAGEEA		CMGA	3507/247050	0.999	28620	0.827	-0.127712	-1.4667	0.1667	-5	18	-1.0722	-5	3	0	0	0.000000	-0.5333	-2	-0.0167	0.700000	0.3333	0.3667	1	0	1	0	1.0667	-0.5378	-1.6	0	0	0	1	0.5218	1.1486	0.9212	-0.1667	0.2723	0.8485	-0.7653	0.0003	0.0089
HIP_28621	HLVEALYLV	RGYPEEKK	HLVEALYLVRGYPEEKK		CMGA	12339/247050	0.992	28621	0.827	0.185745	0.0833	0.1667	1	17	-0.5294	0	2	1	0	0.000000	-4.3	1	0.1333	1.400000	0.1111	1.2889	0	1	0	1	-4.3	0.1074	-8.6	1	0	0	0	0.3987	0.9874	0.8701	0.1667	0.159	0.7571	0.0332	0.0178	-0.5733
HIP_28622	SLYQLENYC	GLFRGGKSG	SLYQLENYCGLFRGGKSG		CMGA	5409/247050	0.998	28622	0.827	-0.087629	0.65	0.1667	1	18	-0.4444	1	2	0	0	0.000000	2.8333	0	0.0333	0.950000	0.2222	0.7278	0	1	0	1	0	0.4731	5.6667	0	0	0	0	0.4269	0.9522	0.9956	0.3333	0.1823	0.9913	0.2775	0.0011	0.0944
HIP_28623	VCGERGFF	DTEKWAEG	VCGERGFFDTEKWAEG		SCG1	11761/367830	0.997	28623	0.827	0.065099	-0.4167	0.1667	-2	16	-0.6937	-2	2	0	0	0.000000	-4.3	-2	0.0667	0.700000	0.5556	0.1444	0	1	0	1	8.6	-0.0602	-8.6	1	0	0	0	0.4551	1.0436	0.8845	0	0.2071	0.7823	-0.1896	0.0044	-0.2867
HIP_28624	SICSLYQLE	EYIENLRQQ	SICSLYQLEEYIENLRQQ		SCG2	10814/334890	0.999	28624	0.827	-0.115167	-0.5833	0	-2	18	-0.5722	-2	2	0	0	0.000000	0.9667	0	-0.05	1.900000	0.3333	1.5667	0	0	0	0	0	-0.9139	1.9333	0	0	1	0	0.4385	1.2553	0.7228	0	0.1922	0.5225	-0.2558	0.0025	-0.0483
HIP_28625	LVEALYLVC	VNSPMNKGD	LVEALYLVCVNSPMNKGD		CMGA	4476/247050	0.999	28625	0.827	0.111594	1.7333	0.1667	-1	18	0.4556	-1	1	0	0	0.000000	-3.5333	0	0.1167	3.050000	0	3.05	0	0	0	0	0	5.2867	-3.5333	0	0	0	0	0.3718	0.9853	0.8612	0.5	0.1382	0.7416	0.6444	0.0136	-0.4122
HIP_28626	GPGAGSLQP	RAYFMSDTR	GPGAGSLQPRAYFMSDTR		SCG1	18234/367830	0.992	28626	0.827	0.029283	-0.8833	0.1667	1	18	-0.6611	1	2	0	0	0.000000	-0.9	1	-0.0667	0.700000	0.1111	0.5889	1	0	1	0	-0.9	-0.5202	-1.8	0	0	0	1	0.4269	1.0788	0.9123	0.1667	0.1823	0.8323	-0.3771	0.0044	0.06
HIP_28627	GERGFFYTP	FPEYNYDWW	GERGFFYTPFPEYNYDWW		SCG1	3978/367830	0.999	28627	0.827	-0.158196	-0.9833	0.3333	-2	18	-1.1389	-2	5	0	0	0.000000	0.4333	-1	0	1.400000	0.1111	1.2889	2	0	1	0	-0.4333	-1.2674	2.1667	0	0	0	1	0.5385	0.9503	1.0412	0	0.2899	1.084	-0.5295	0	0
HIP_28628	TSICSLYQL	DKLAPVSKR	TSICSLYQLDKLAPVSKR		SCG2	32607/334890	0.988	28628	0.827	0.118518	-0.7667	0	2	18	-0.05	2	1	0	0	0.000000	-0.8667	0	0.05	0.450000	0.2222	0.2278	0	0	0	0	0	-0.1746	-0.8667	1	0	0	0	0.3821	1.1291	0.7835	0	0.146	0.6138	-0.2929	0	-0.0433
HIP_28629	CTSICSLYQ	NYGEEGAPG	CTSICSLYQNYGEEGAPG		SCG1	2137/367830	1.000	28629	0.827	0.033442	-1.0333	0.1667	-2	18	-0.3667	-2	2	0	0	0.000000	-1.4	0	0.0833	0.625000	0.1111	0.5139	0	1	0	1	0	-0.531	-2.8	0	1	0	0	0.4936	0.9372	1.059	0.1667	0.2436	1.1214	-0.51	0.0069	-0.1167
HIP_28630	LVEALYLVC	LKRQYDRVA	LVEALYLVCLKRQYDRVA		SCG1	24718/367830	0.981	28630	0.827	0.134966	0.9833	0	1	18	0.4389	1	1	0	0	0.000000	-5.0333	2	0.1	2.950000	0.2222	2.7278	0	0	0	0	-10.0667	2.6823	-5.0333	0	0	0	0	0.3154	1.1208	0.7035	0.1667	0.0995	0.4949	0.3101	0.01	-0.5033
HIP_28631	VCGERGFF	EEEEEMAVV	VCGERGFFEEEEEMAVV		CMGA	6033/247050	0.998	28631	0.827	0.036200	-0.8833	0.1667	-5	17	-0.1118	-5	2	1	3	0.000000	-5.2333	-3	0.0667	0.700000	0.6667	0.0333	0	1	0	1	15.7	-0.0294	-10.4667	1	0	0	0	0.4833	1.2383	0.82	-0.1667	0.2336	0.6724	-0.4269	0.0044	-0.3489
HIP_28632	LVCGERGFF	ATNTHPPA	LVCGERGFFATNTHPPA		CMGA	8437/247050	0.996	28632	0.827	0.038274	0.4667	0.1667	1	17	-0.0235	0	2	1	0	1.000000	-2.5333	0	0.0167	1.150000	0.2222	0.9278	0	1	0	1	0	0.433	-5.0667	1	0	0	0	0.4167	0.975	0.98	0.5	0.1736	0.9604	0.1944	0.0003	-0.0422
HIP_28633	VCGERGFFY	QEEEQIEQ	VCGERGFFYQEEEQIEQ		SCG2	10411/334890	0.999	28633	0.827	0.141993	-1.0333	0	-4	17	-1.0471	-4	3	1	1	0.000000	-4.9333	-2	0.15	0.700000	0.4444	0.2556	0	0	0	0	9.8667	-0.2641	-14.8	0	1	0	0	0.4385	1.2586	0.7612	0	0.1922	0.5794	-0.4531	0.0225	-0.74
HIP_28634	LYQLENYCN	RAYGFRGPG	LYQLENYCNRAYGFRGPG		CMGA	21215/247050	0.968	28634	0.827	0.007013	-1.05	0	1	18	-0.8333	1	2	0	0	0.000000	-0.5667	1	0	0.625000	0.2222	0.4028	0	0	0	0	-0.5667	-0.4229	-1.1333	0	1	0	0	0.4205	1.0344	0.9746	0	0.1768	0.9499	-0.4415	0	0
HIP_28635	VEALYLVC	GYGEEEEEE	VEALYLVCGYGEEEEEE		SCG1	1444/367830	1.000	28635	0.827	0.155570	1.4	0.3333	-6	17	-0.2882	-6	2	1	1	0.000000	-4.2	0	0.1667	0.625000	0.2222	0.4028	0	2	0	1	0	0.5639	-8.4	0	0	0	0	0.4436	0.9005	0.969	0.3333	0.1968	0.9389	0.621	0.0278	-0.7
HIP_28636	SHLVEALY	GPQLRRGWR	SHLVEALYGPQLRRGWR		CMGA	8416/247050	0.996	28636	0.827	0.248168	-0.2	0.3333	3	17	-0.6882	2	1	1	0	0.000000	-3.2667	0	0.2	0.000000	0.2222	-0.2222	1	1	1	1	0	0.0444	-3.2667	0	1	0	0	0.4718	0.9919	1.0112	0.3333	0.2226	1.0224	-0.0944	0.04	-0.6533
HIP_28637	RGFFYTPKT	AEAYPSKPD	RGFFYTPKTAEAYPSKPD		NPY	1793/49410	0.989	28637	0.827	0.074696	-1.0167	0.1667	1	18	-1.1167	1	2	0	0	0.000000	2.1	-2	-0.1167	0.450000	0.2222	0.2278	1	0	1	0	-4.2	-0.2316	4.2	0	1	0	0	0.4551	1.1386	0.9078	0	0.2071	0.8241	-0.4627	0.0136	-0.345
HIP_28638	SHLVEALYL	FEDELSEV	SHLVEALYLFEDELSEV		CMGA	6132/247050	0.998	28638	0.827	0.092744	0.35	0	-4	17	0.2706	-5	2	1	0	1.000000	-3.5	-2	0.1	0.700000	0.4444	0.2556	0	0	0	0	7	0.0894	-7	1	0	0	0	0.3436	1.2255	0.6923	0.1667	0.1181	0.4793	0.1203	0.01	-0.35
HIP_28639	LVCGERGFF	VLENQSSQA	LVCGERGFFVLENQSSQA		CMGA	1491/247050	1.000	28639	0.827	-0.036978	1.6167	0.1667	-1	18	0.0833	-1	2	0	0	1.000000	-0.2333	-1	-0.0333	1.750000	0.3333	1.4167	0	1	0	1	0.2333	2.2903	-0.4667	1	0	0	0	0.35	1.1283	0.74	0.5	0.1225	0.5476	0.5658	0.0011	0.0078
HIP_28640	RGFFYTPKT	PYYDPLQWK	RGFFYTPKTPYYDPLQWK		SCG1	12693/367830	0.996	28640	0.827	0.044601	-1.7333	0.3333	2	18	-1.1833	2	3	0	0	0.000000	0.6667	-1	-0.1	0.000000	0.2222	-0.2222	2	0	1	0	-0.6667	0.3852	2	0	1	0	0	0.5487	0.8391	1.1201	-0.1667	0.3011	1.2547	-0.9511	0.01	-0.0667
HIP_28641	VEALYLVC	SPMNKGDTE	VEALYLVCSPMNKGDTE		CMGA	3227/247050	0.999	28641	0.827	0.192832	1.6667	0.3333	-2	17	-0.0294	-2	1	1	0	0.000000	-3.6667	0	0.1333	0.625000	0.1111	0.5139	1	0	1	0	0	0.8565	-3.6667	0	0	0	0	0.3885	0.9919	0.8712	0.5	0.1509	0.7589	0.6474	0.0178	-0.4889
HIP_28642	QCCTSICSL	PEEKKEEEG	QCCTSICSLPEEKKEEEG		CMGA	2889/247050	0.999	28642	0.827	0.070463	-0.5167	0.3333	-3	18	-0.9611	-3	0	0	1	0.000000	-4.7	-2	0.1	0.000000	0.3333	-0.3333	1	0	1	0	9.4	0.1722	0	1	0	0	0	0.5218	1.1186	0.9345	-0.1667	0.2723	0.8733	-0.2696	0.01	-0.47
HIP_28643	SICSLYQLE	QGAWKELDR	SICSLYQLEQGAWKELDR		SCG1	6066/367830	0.999	28643	0.827	-0.164669	-0.8833	0.1667	-1	18	-0.55	-1	2	0	0	0.000000	0.3667	1	0.0333	1.900000	0.1111	1.7889	0	1	0	1	0.3667	-1.5802	0.7333	0	0	1	0	0.4833	1.1633	0.8933	0.1667	0.2336	0.798	-0.4269	0.0011	0.0122
HIP_28644	EDLQVGQVE	EQYTPQSLA	EDLQVGQVEEQYTPQSLA		SCG2	15876/334890	0.997	28644	0.827	-0.129589	-1.85	0	-4	18	-0.8333	-4	1	0	0	0.000000	-1.8333	0	0	2.100000	0.2222	1.8778	0	0	0	0	0	-3.4739	-1.8333	0	0	1	0	0.5051	1.2203	0.8078	-0.1667	0.2552	0.6526	-0.9345	0	0
HIP_28645	LVCGERGFF	KKELENLAA	LVCGERGFFKKELENLAA		SCG1	24564/367830	0.981	28645	0.827	0.099647	-1.0167	0.1667	0	18	0.0111	0	2	0	0	0.000000	-5.5	1	0.0667	1.650000	0.5556	1.0944	0	1	0	1	-5.5	-1.1127	-11	1	0	0	0	0.4269	1.0755	0.8756	-0.1667	0.1823	0.7667	-0.434	0.0044	-0.3667
HIP_28646	VEALYLVC	FLIVLSVAL	VEALYLVCFLIVLSVAL		IAPP	1093/45018	0.996	28646	0.827	-0.001739	3.6	0	-1	17	2.5647	-1	2	1	0	0.000000	0.2	0	0	2.525000	0	2.525	0	0	0	0	0	9.09	0.4	0	1	0	0	0.2385	1.1603	0.5795	0.8333	0.0569	0.3358	0.8585	0	0
HIP_28647	VCGERGFFY	QYWDEDLL	VCGERGFFYQYWDEDLL		SCG2	14421/334890	0.998	28647	0.827	0.051743	-0.2333	0	-3	17	-0.3765	-3	5	1	0	0.000000	-3.3333	0	0.05	0.700000	0.2222	0.4778	0	0	0	0	0	-0.1115	-16.6667	0	1	0	0	0.3821	1.0958	0.8168	0.5	0.146	0.6672	-0.0891	0.0025	-0.1667
HIP_28648	CGERGFFY	KENIEKNEQ	CGERGFFYKENIEKNEQ		SCG2	18702/334890	0.997	28648	0.827	0.209158	-1.1	0	-1	17	-1.5471	-3	3	1	0	0.000000	-5.0667	0	0.15	0.000000	0.3333	-0.3333	0	0	0	0	0	0.3667	-15.2	0	1	0	0	0.4103	1.1038	0.8856	0	0.1683	0.7844	-0.4513	0.0225	-0.76
[illegible]																																														

HIP_29117	GAGSLQPLA	MARYYSALR	GAGSLQPLAMARYYSALR		NPY	2491/49410	0.982	29117	0.826	-0.000093	0.5333	0.1667	2	18	0.0778	2	1	0
HIP_29118	GSHLVEALY	KELENLAAM	GSHLVEALYKELENLAAM		SCG1	15500/367830	0.994	29118	0.826	0.121206	0.1167	0	-1	18	0.1722	-2	1	0
HIP_29119	AGSLQPLAL	SEESWDEED	AGSLQPLALSEESWDEED		SCG1	3386/367830	1.000	29119	0.826	0.133270	0.2667	0.1667	-6	18	-0.8222	-6	0	0
HIP_29120	HLVEALYL	EEEEMAVVP	HLVEALYLEEEEMAVVP		CMGA	5059/247050	0.998	29120	0.826	0.114439	-0.7	0	-4	17	0.3471	-5	1	1
HIP_29121	CGERGFFYT	SVPLQQKEN	CGERGFFYTSVPLQQKEN		SCG2	1378/334890	1.000	29121	0.826	-0.017624	0.4333	0.3333	0	18	-0.8333	0	3	0
HIP_29122	HLVEALYL	MNDNFLEGE	HLVEALYLMNDNFLEGE		SCG1	8476/367830	0.998	29122	0.826	0.088673	0.2	0	-3	17	0	-4	2	1
HIP_29123	QCCTSICSL	EEEEEEEAE	QCCTSICSLEEEEEEEAE		CMGA	2555/247050	0.999	29123	0.826	0.002017	-0.8333	0.1667	-8	18	-0.9	-8	0	0
HIP_29124	LVCGERGFF	MGILKLQVF	LVCGERGFFMGILKLQVF		IAPP	252/45018	0.999	29124	0.826	-0.033322	1.8667	0.3333	1	18	1.1389	1	2	0
HIP_29125	HLVEALYLV	VEVLKREPL	HLVEALYLVVEVLKREPL		IAPP	1285/45018	0.995	29125	0.826	0.000527	1.9333	0	0	18	0.7	-1	1	0
HIP_29126	HLCGSHLVE	HSQEKTHSR	HLCGSHLVEHSQEKTHSR		SCG1	35301/367830	0.943	29126	0.826	0.126601	-0.5	0.1667	4	18	-1.1611	0	0	0
HIP_29127	PGAGSLQPL	EEEAEAGEE	PGAGSLQPLEEEAEAGEE		CMGA	3907/247050	0.999	29127	0.826	-0.079683	-1.9667	0.1667	-6	18	-0.9278	-6	0	0
HIP_29128	GSHLVEALY	GPGPQLRRG	GSHLVEALYGPGPQLRRG		CMGA	2505/247050	0.999	29128	0.826	0.187163	0.3167	0.5	2	18	-0.4833	1	1	0
HIP_29129	GSLQPLAL	QKLYTDDED	GSLQPLALQKLYTDDED		SCG2	15381/334890	0.998	29129	0.826	0.147195	0.9667	0	-3	17	-0.7471	-3	1	1
HIP_29130	LVCGERGFF	SQGGSLPS	LVCGERGFFSQGGSLPS		SCG1	7610/367830	0.999	29130	0.826	0.153626	0.0833	0.5	0	17	0.1647	0	2	1
HIP_29131	SHLVEALY	EEEEEEEEE	SHLVEALYEEEEEEEEE		CMGA	2867/247050	0.999	29131	0.826	0.084712	-1.0333	0	-9	17	-1.5706	-10	1	1
HIP_29132	GSLQPLAL	QSAPKENKP	GSLQPLALQSAPKENKP		SCG2	15274/334890	0.998	29132	0.826	0.214374	1.15	0.1667	1	17	-0.8	1	0	1
HIP_29133	CSLYQLENY	LAEAYPSKP	CSLYQLENYLAEAYPSKP		NPY	1762/49410	0.989	29133	0.826	-0.088307	-1.0333	0	-1	18	-0.5056	-1	1	0
HIP_29134	AGSLQPLAL	GPTKADTEK	AGSLQPLALGPTKADTEK		SCG1	5700/367830	0.999	29134	0.826	0.223972	1.1167	0.3333	0	18	-0.4278	0	0	0
HIP_29135	GSHLVEALY	ETGSQENHP	GSHLVEALYETGSQENHP		SCG1	3756/367830	0.999	29135	0.826	0.179432	-0.05	0.1667	-1	18	-0.9056	-3	1	0
HIP_29136	HLCGSHLVE	LEYLNQEKA	HLCGSHLVELEYLNQEKA		SCG2	15327/334890	0.998	29136	0.826	-0.052039	0.5833	0	0	18	-0.3667	-2	1	0
HIP_29137	GERGFFYT	FLVHSSNN	GERGFFYTFLVHSSNN		IAPP	1218/45018	0.995	29137	0.826	-0.037171	1.9333	0	1	16	-0.3875	0	4	0
HIP_29138	CCTSICSLY	VNWGYEKRN	CCTSICSLYVNWGYEKRN		SCG1	10778/367830	0.997	29138	0.826	-0.074993	0.25	0.1667	1	18	-0.2833	1	2	0
HIP_29139	EQCCTSICS	EEEEEEEEE	EQCCTSICSEEEEEEEEE		CMGA	1521/247050	1.000	29139	0.826	-0.021464	-0.7167	0.1667	-10	18	-1.6	-10	0	0
HIP_29140	LVEALYLVC	GEEGAPGK	LVEALYLVCGEEGAPGK		SCG1	6459/367830	0.999	29140	0.826	0.172341	0.5167	0.1667	-2	17	0.4353	-2	1	1
HIP_29141	VCGERGFF	SKGQPRSQE	VCGERGFFSKGQPRSQE		SCG1	15316/367830	0.995	29141	0.826	0.167634	0.0167	0.5	1	17	-1.1176	1	2	1
HIP_29142	RGFFYTPKT	QEEVRDSKE	RGFFYTPKTQEEVRDSKE		SCG2	20703/334890	0.996	29142	0.826	-0.069096	-2.7833	0.1667	0	18	-1.6667	0	1	0
HIP_29143	GSLQPLAL	GEATDGARP	GSLQPLALGEATDGARP		CMGA	3208/247050	0.999	29143	0.826	0.183742	1.2167	0.1667	-1	17	-0.2412	-1	0	1
HIP_29144	GSLQPLAL	LANFLVHSS	GSLQPLALLANFLVHSS		IAPP	1603/45018	0.993	29144	0.826	0.055721	1.9167	0	1	17	0.8824	0	1	1
HIP_29145	SHLVEALYL	QEEDLRKE	SHLVEALYLQEEDLRKE		SCG2	11668/334890	0.998	29145	0.826	0.174486	-0.7	0	-2	17	-0.7941	-3	1	1
HIP_29146	GSHLVEALY	RNHNEGMVT	GSHLVEALYRNHNEGMVT		SCG1	26016/367830	0.978	29146	0.826	0.139960	-1.15	0	1	18	-0.4889	-1	1	0
HIP_29147	SLYQLENYC	EEGAPGKWQ	SLYQLENYCEEGAPGKWQ		SCG1	5801/367830	0.999	29147	0.826	0.090196	-1.6167	0.1667	-2	18	-1.0944	-2	1	0
HIP_29148	SHLVEALYL	NPYYDPLQ	SHLVEALYLNPYYDPLQ		SCG1	14475/367830	0.995	29148	0.826	0.154246	-0.0167	0.1667	-1	17	-0.2294	-2	3	1
HIP_29149	NQHLCGSHL	EEEMAVVPQ	NQHLCGSHLEEEMAVVPQ		CMGA	8624/247050	0.996	29149	0.826	0.018655	-1.7833	0.1667	-1	18	-0.4444	-3	0	0
HIP_29150	ICSLYQLEN	RNYPSLELD	ICSLYQLENRNYPSLELD		SCG1	26700/367830	0.976	29150	0.826	0.014162	-2.0833	0	-2	18	-0.5056	-2	2	0
HIP_29151	QHLCGSHLV	EEYRAPRP	QHLCGSHLVEEYRAPRP		SCG1	20759/367830	0.988	29151	0.826	0.144700	-0.5833	0	2	17	-0.9118	0	1	1
HIP_29152	LQPLALEGS	TGPNNQKRE	LQPLALEGSTGPNNQKRE		SCG2	4025/334890	1.000	29152	0.826	-0.106743	-1.2333	0.6667	0	18	-1.2056	0	0	0
HIP_29153	DLQVGQVEL	LLLSLLGAV	DLQVGQVELLLLSLLGAV		SCG1	4163/367830	0.999	29153	0.826	0.010281	2.65	0	-2	18	1.4111	-2	0	0
HIP_29154	LVCGERGFF	GSSQETDKL	LVCGERGFFGSSQETDKL		SCG2	34/334890	1.000	29154	0.826	0.145680	0.5333	0.6667	-1	18	-0.3333	-1	2	0
HIP_29155	LCGSHLVEA	ENELTLNEK	LCGSHLVEAENELTLNEK		SCG1	13475/367830	0.996	29155	0.826	0.108540	-1.3333	0	-2	18	-0.35	-3	0	0
HIP_29156	CGERGFFY	HESSSRGE	CGERGFFYHESSSRGE		SCG1	17594/367830	0.992	29156	0.826	0.148298	-0.5333	0.1667	0	16	-1.2188	-1	3	0
HIP_29157	HLVEALYLV	PDRSSQGGS	HLVEALYLVPDRSSQGGS		SCG1	14517/367830	0.995	29157	0.826	0.222644	-0.4833	0.1667	0	18	-0.15	-1	1	0
HIP_29158	RGFFYTPKT	KENGDESHL	RGFFYTPKTKENGDESHL		SCG2	23959/334890	0.994	29158	0.826	-0.009265	-2.85	0.1667	1	18	-1.4444	0	1	0
HIP_29159	TSICSLYQL	AAVLALLLC	TSICSLYQLAAVLALLLC		CMGA	4514/247050	0.999	29159	0.826	-0.007805	1.1333	0	0	18	1.9333	0	1	0

[illegible]

HIP_29628	CGERGFFY	SPMPVSQEC	CGERGFFYSPMPVSQEC		CMGA	3740/247050	0.999	29628	0.826	0.148701	0.6333	0.3333	-1	17	-0.3059	-1	3	1
HIP_29629	TSICSLYQL	EPEYGEEI	TSICSLYQLEPEYGEEI		SCG1	7697/367830	0.998	29629	0.826	0.104006	-1.6	0.1667	-4	17	-0.3118	-4	2	1
HIP_29630	SLYQLENYC	QKFPSPEMI	SLYQLENYCQKFPSPEMI		SCG2	20681/334890	0.996	29630	0.826	-0.005635	-1.15	0	-1	18	-0.5278	-1	2	0
HIP_29631	AGSLQPLAL	RGGEPRAYF	AGSLQPLALRGGEPRAYF		SCG1	12884/367830	0.996	29631	0.826	0.237010	0.6833	0.3333	1	18	-0.1611	1	0	0
HIP_29632	ALYLVCGER	EEGSANRRP	ALYLVCGEREEGSANRRP		CMGA	14933/247050	0.987	29632	0.826	-0.082300	-2.6333	0.3333	0	18	-0.7833	0	0	0
HIP_29633	VEALYLVC	EPGETQNAF	VEALYLVCEPGETQNAF		SCG1	4637/367830	0.999	29633	0.826	0.199983	0.8333	0.3333	-3	17	0.2	-3	1	1
HIP_29634	AGSLQPLAL	IEVLSNALS	AGSLQPLALIEVLSNALS		SCG1	10228/367830	0.998	29634	0.826	0.016827	2.4333	0	-1	18	1.0111	-1	0	0
HIP_29635	LYQLENYCN	EMLKTGEKP	LYQLENYCNEMLKTGEKP		SCG2	16848/334890	0.997	29635	0.826	-0.081786	-0.0167	0	-1	18	-1.0167	-1	1	0
HIP_29636	ICSLYQLEN	AFLNERKQA	ICSLYQLENAFLNERKQA		SCG1	6565/367830	0.999	29636	0.826	0.020655	0.8667	0	0	18	-0.3722	0	2	0
HIP_29637	SLYQLENYC	HSTHYRASE	SLYQLENYCHSTHYRASE		SCG1	20315/367830	0.989	29637	0.826	0.008087	-1.1667	0.1667	1	18	-1.1111	-1	1	0
HIP_29638	LVEALYLVC	SSEEKHLE	LVEALYLVCSSEEKHLE		SCG1	6947/367830	0.999	29638	0.826	0.198734	0.9	0.3333	-2	17	0.2294	-3	1	1
HIP_29639	LVEALYLVC	SKEAEKSGE	LVEALYLVCSKEAEKSGE		CMGA	8940/247050	0.996	29639	0.826	0.201523	0.3833	0.1667	-2	18	0.0444	-2	1	0
HIP_29640	SLYQLENYC	HPQGAWKEL	SLYQLENYCHPQGAWKEL		SCG1	17524/367830	0.992	29640	0.826	0.011458	-1.7667	0.1667	0	18	-0.8444	-1	1	0
HIP_29641	DLQVGQVEL	EDWMRILE	DLQVGQVELEDWMRILE		SCG2	12639/334890	0.998	29641	0.826	0.039823	-0.5667	0	-4	18	0.0222	-4	1	0
HIP_29642	LVCGERGFF	EEEEEAEA	LVCGERGFFEEEEEAEA		CMGA	5789/247050	0.998	29642	0.826	0.028645	-0.8833	0.1667	-6	17	-0.5941	-6	2	1
HIP_29643	HLVEALYLV	PEDLEWERY	HLVEALYLVPEDLEWERY		SCG1	4659/367830	0.999	29643	0.826	0.136447	-0.3167	0.1667	-3	18	-0.2722	-4	1	0
HIP_29644	CGERGFFY	AEAKTHWL	CGERGFFYAEAKTHWL		SCG2	17839/334890	0.997	29644	0.826	0.070471	0.7333	0	1	16	-0.425	0	3	0
HIP_29645	VCGERGFF	QDPEGKGEQ	VCGERGFFQDPEGKGEQ		CMGA	6868/247050	0.997	29645	0.826	0.084543	-0.5667	0.3333	-2	17	-1.1941	-2	2	1
HIP_29646	RGFFYTPKT	AWIPHVENR	RGFFYTPKTAWIPHVENR		SCG2	8160/334890	0.999	29646	0.826	0.123014	-0.1333	0.1667	3	18	-0.7889	2	2	0
HIP_29647	QHLCGSHLV	AEGGGHSRE	QHLCGSHLVAEGGGHSRE		SCG1	6444/367830	0.999	29647	0.826	0.175319	0.45	0.1667	2	18	-0.65	-1	0	0
HIP_29648	IVEQCCTSI	EEEEEEAEA	IVEQCCTSIEEEEEEAEA		CMGA	2805/247050	0.999	29648	0.826	-0.064298	-1.25	0.1667	-8	18	-0.6222	-8	0	0
HIP_29649	HLVEALYLV	IVEEQYTPQ	HLVEALYLVIVEEQYTPQ		SCG2	6626/334890	0.999	29649	0.826	-0.014273	1.9833	0	-2	18	0.2611	-3	1	0
HIP_29650	LGGGPGAGS	PYGAGRSRS	LGGGPGAGSPYGAGRSRS		SCG2	5586/334890	0.999	29650	0.826	-0.042406	-0.45	0.6667	2	18	-0.6278	2	1	0
HIP_29651	QHLCGSHLV	LIFLISGAE	QHLCGSHLVLIFLISGAE		SCG2	9478/334890	0.999	29651	0.826	-0.016475	2.65	0	1	18	1.0944	-1	1	0
HIP_29652	VEALYLVC	SEEEPEYGE	VEALYLVCSEEEPEYGE		SCG1	5899/367830	0.999	29652	0.826	0.147493	0.45	0.1667	-6	17	-0.3588	-6	1	1
HIP_29653	CGERGFFY	SLGYKEIR	CGERGFFYSLGYKEIR		CMGA	3749/247050	0.999	29653	0.826	0.167202	1.15	0.3333	1	16	-0.5063	1	4	0
HIP_29654	LVEALYLVC	LEQEEERLS	LVEALYLVCLEQEEERLS		CMGA	3477/247050	0.999	29654	0.826	0.071294	1.2167	0	-4	18	0.2278	-4	1	0
HIP_29655	QHLCGSHLV	IFLISGAEA	QHLCGSHLVIFLISGAEA		SCG2	9479/334890	0.999	29655	0.826	-0.019114	2.65	0	1	18	0.9833	-1	1	0
HIP_29656	GSHLVEALY	QAEGDSEGL	GSHLVEALYQAEGDSEGL		CMGA	4441/247050	0.999	29656	0.826	0.067186	-0.15	0	-3	18	-0.3111	-4	1	0
HIP_29657	HLVEALYLV	QGAWKELD	HLVEALYLVQGAWKELD		SCG1	6285/367830	0.999	29657	0.826	0.164217	0.7667	0.1667	-1	17	0.2059	-2	2	1
HIP_29658	CGERGFFY	GRHHRGRGG	CGERGFFYGRHHRGRGG		SCG1	26724/367830	0.976	29658	0.826	0.206421	-0.6333	0.1667	5	17	-1.3824	3	3	1
HIP_29659	TSICSLYQ	ENYQKGERG	TSICSLYQENYQKGERG		SCG1	26114/367830	0.977	29659	0.826	0.096368	-1.55	0	0	17	-1.2235	0	2	1
HIP_29660	GSLQPLAL	ATRLFEKPL	GSLQPLALATRLFEKPL		SCG2	21277/334890	0.996	29660	0.826	0.180587	1	0	1	17	0.2882	1	1	1
HIP_29661	LVEALYLV	ELQKIAEKF	LVEALYLVELQKIAEKF		SCG1	9465/367830	0.998	29661	0.826	0.066907	0.5833	0	-1	17	0.6706	-1	1	1
HIP_29662	AGSLQPLAL	MDMSDDYET	AGSLQPLALMDMSDDYET		SCG2	4922/334890	1.000	29662	0.826	0.004701	1.6167	0	-4	18	-0.2389	-4	0	0
HIP_29663	LVCGERGFF	DSLEAGLPL	LVCGERGFFDSLEAGLPL		CMGA	2544/247050	0.999	29663	0.826	0.005850	0.7833	0.3333	-2	18	0.5944	-2	2	0
HIP_29664	SHLVEALYL	ESEEERGL	SHLVEALYLESEEERGL		SCG1	8963/367830	0.998	29664	0.826	0.184487	-0.25	0.1667	-3	17	-0.4294	-4	1	1
HIP_29665	SHLVEALYL	SEEERGLE	SHLVEALYLSEEERGLE		SCG1	8964/367830	0.998	29665	0.826	0.184487	-0.25	0.1667	-3	17	-0.4294	-4	1	1
HIP_29666	SHLVEALYL	KSAEFPDFY	SHLVEALYLKSAEFPDFY		SCG1	17803/367830	0.992	29666	0.826	0.206631	0.5667	0.1667	-1	18	0.0778	-2	1	0
HIP_29667	TSICSLYQL	QRLANFLVH	TSICSLYQLQRLANFLVH		IAPP	3864/45018	0.961	29667	0.826	0.099108	-0.8667	0	2	18	0.5111	1	1	0
HIP_29668	GERGFFYTP	PGWQAKREE	GERGFFYTPPGWQAKREE		CMGA	1895/247050	0.999	29668	0.826	-0.106290	-1.0833	0.5	0	18	-1.4889	0	4	0
HIP_29669	GSHLVEALY	LNHLKATPI	GSHLVEALYLNHLKATPI		IAPP	2053/45018	0.990	29669	0.826	-0.005573	0.2333	0	2	18	0.3	0	1	0
HIP_29670	GERGFFYTP	SQGGSLPSE	GERGFFYTPSQGGSLPSE		SCG1	860/367830	1.000	29670	0.826	-0.043414	-1.3833	0.5	-1	18	-0.8222	-1	3	0

HIP_30139	AGSLQPLAL	QESKAEGN	AGSLQPLALQESKAEGN		CMGA	7682/247050	0.997	30139	0.825	0.218762	0.2667	0.1667	-1	17	-0.5059	-1	0	1	0	0.000000	-5.7333	-1	0.1667	0.450000	0.2222	0.2278	0	0	0	0	5.7333	0.0607	0	1	0	0	0	0.4	1.245	0.7633	0.3333	0.16	0.5827	0.1067	0.0278	-0.9556
HIP_30140	LVEALYLVC	FLIVLSVA	LVEALYLVCFLIVLSVA		IAPP	1095/45018	0.996	30140	0.825	-0.018199	3.6	0	-1	17	2.5647	-1	2	1	0	0.000000	0.2	0	0	3.750000	0	3.75	0	0	0	0	0	13.5	0.4	0	0	0	0	0.2385	1.1603	0.5795	0.8333	0.0569	0.3358	0.8585	0	0
HIP_30141	GERGFFYTP	SSEDDLQEE	GERGFFYTPSSEDDLQEE		SCG2	561/334890	1.000	30141	0.825	-0.099126	-1.45	0.5	-5	18	-1.4222	-5	3	0	0	0.000000	-0.5	-1	0.0667	0.700000	0.2222	0.4778	1	0	1	0	0.5	-0.6928	-1.5	0	0	0	1	0.5885	0.9219	1.0878	-0.1667	0.3463	1.1834	-0.8533	0.0044	-0.0333
HIP_30142	GERGFFYTP	GSGRLQNGQ	GERGFFYTPGSGRLQNGQ		SCG2	811/334890	1.000	30142	0.825	-0.004800	-0.8667	0.6667	1	18	-1.1111	1	3	0	0	0.000000	0.6667	0	0.1167	0.700000	0.1111	0.5889	1	2	1	1	0	-0.5104	2	0	0	0	1	0.6218	0.7036	1.2645	0	0.3866	1.5989	-0.5389	0.0136	0.0778
HIP_30143	LYQLENYCN	EDKRNYPSL	LYQLENYCNEDKRNYPSL		SCG1	24532/367830	0.981	30143	0.825	0.138412	-2.2	0	-1	18	-1.4056	-1	1	0	0	0.000000	-2.8667	-1	0.1	0.625000	0.5556	0.0694	0	0	0	0	2.8667	-0.1528	-2.8667	0	1	0	0	0.4872	1.0444	0.9879	-0.5	0.2373	0.976	-1.0718	0.01	-0.2867
HIP_30144	SHLVEALYL	EERGLEPG	SHLVEALYLEERGLEPG		SCG1	16846/367830	0.993	30144	0.825	0.209488	-0.8667	0	-2	17	-0.2941	-3	1	1	0	0.000000	-5.9333	-1	0.15	0.000000	0.4444	-0.4444	0	0	0	0	5.9333	0.3852	-5.9333	1	0	0	0	0.4103	1.2772	0.7156	-0.1667	0.1683	0.5121	-0.3556	0.0225	-0.89
HIP_30145	HLCGSHLVE	EEEEEEEAE	HLCGSHLVEEEEEEEEAE		CMGA	3834/247050	0.999	30145	0.825	0.012216	-1	0	-7	18	-1.2778	-9	0	0	6	0.000000	-5	-2	0.1	1.050000	0.5556	0.4944	0	0	0	0	10	-0.4944	0	0	0	1	0	0.4667	1.4	0.66	-0.3333	0.2178	0.4356	-0.4667	0.01	-0.5
HIP_30146	RGFFYTPKT	HPQESKGQP	RGFFYTPKTHPQESKGQP		SCG1	23911/367830	0.983	30146	0.825	0.008346	-2.4167	0.3333	3	18	-1.6389	2	1	0	0	0.000000	-0.7	-1	-0.05	0.000000	0.2222	-0.2222	2	0	1	0	0.7	0.537	-0.7	0	1	0	0	0.5769	0.8538	1.1323	-0.3333	0.3328	1.2821	-1.3942	0.0025	0.035
HIP_30147	GSHLVEALY	ALLLCAGQV	GSHLVEALYALLLCAGQV		CMGA	6475/247050	0.998	30147	0.825	-0.026846	2.2833	0	0	18	1.2333	-1	1	0	1	0.000000	1.7	0	0	3.400000	0.1111	3.2889	0	0	0	0	0	7.5096	1.7	0	1	0	0	0.2385	1.3036	0.6212	0.8333	0.0569	0.3858	0.5445	0	0
HIP_30148	CSLYQLENY	QQAHKEESS	CSLYQLENYQQAHKEESS		SCG2	18286/334890	0.997	30148	0.825	-0.092517	-2.25	0	-1	18	-1.3722	-2	1	0	0	0.000000	1.0333	1	0	0.000000	0.1111	-0.1111	0	0	0	0	1.0333	0.25	1.0333	0	1	0	0	0.5051	1.1453	0.9712	0	0.2552	0.9431	-1.1365	0	0
HIP_30149	HLCGSHLVE	EEEEEEEEA	HLCGSHLVEEEEEEEEEA		CMGA	3832/247050	0.999	30149	0.825	0.012320	-1	0	-7	18	-1.2778	-9	0	0	7	0.000000	-5	-2	0.1	1.050000	0.5556	0.4944	0	0	0	0	10	-0.4944	0	0	0	1	0	0.4667	1.4	0.66	-0.3333	0.2178	0.4356	-0.4667	0.01	-0.5
HIP_30150	HLCGSHLVE	EEEEEEEEA	HLCGSHLVEEEEEEEEEA		CMGA	3833/247050	0.999	30150	0.825	0.012320	-1	0	-7	18	-1.2778	-9	0	0	7	0.000000	-5	-2	0.1	1.050000	0.5556	0.4944	0	0	0	0	10	-0.4944	0	0	0	1	0	0.4667	1.4	0.66	-0.3333	0.2178	0.4356	-0.4667	0.01	-0.5
HIP_30151	LVEALYLVC	SEGLSQGLV	LVEALYLVCSEGLSQGLV		CMGA	2814/247050	0.999	30151	0.825	0.177978	0.9667	0.3333	-2	18	1.2056	-2	1	0	0	0.000000	-5.0667	-1	0.2	2.950000	0.1111	2.8389	0	1	0	1	5.0667	2.7443	-5.0667	0	0	0	0	0.4551	1.0319	0.8912	0.1667	0.2071	0.7942	0.44	0.04	-1.0133
HIP_30152	GSHLVEALY	GEGHHRVQE	GSHLVEALYGEGHHRVQE		SCG1	4252/367830	0.999	30152	0.825	0.187809	0	0.3333	1	18	-0.7556	-2	1	0	0	0.000000	-2.8667	-1	0.2167	2.000000	0.2222	1.7778	0	2	0	1	2.8667	0	-2.8667	0	1	0	0	0.4718	1.0469	0.9678	0.1667	0.2226	0.9367	0	0.0469	-0.6211
HIP_30153	GPGAGSLQP	DPEGKGEQE	GPGAGSLQPDPEGKGEQE		CMGA	3996/247050	0.999	30153	0.825	-0.049848	-1.65	0.3333	-3	18	-1.4944	-3	0	0	0	0.000000	-2.4333	-2	0.05	0.000000	0.2222	-0.2222	2	0	1	0	4.8667	0.3667	0	0	0	0	1	0.5385	1.0136	1.0278	-0.1667	0.2899	1.0564	-0.8885	0.0025	-0.1217
HIP_30154	QHLCGSHLV	AENEKDRAD	QHLCGSHLVAENEKDRAD		SCG1	12029/367830	0.997	30154	0.825	0.133213	-0.0667	0	0	18	-1.0611	-2	0	0	0	1.000000	-3.3333	-1	0.1	1.400000	0.2222	1.1778	0	0	0	0	3.3333	-0.0785	0	1	0	0	0	0.3718	1.1686	0.8112	0.1667	0.1382	0.658	-0.0248	0.01	-0.3333
HIP_30155	EQCCTSICS	LPYGAGRSR	EQCCTSICSLPYGAGRSR		SCG2	9681/334890	0.999	30155	0.825	-0.021825	1.1833	0.3333	1	18	-0.2889	1	1	0	0	0.000000	-1.7667	0	0	1.575000	0	1.575	1	0	1	0	0	1.8638	-1.7667	0	1	0	0	0.4269	0.9655	0.9173	0.3333	0.1823	0.8415	0.5052	0	0
HIP_30156	SHLVEALYL	ENLAAMDLE	SHLVEALYLENLAAMDLE		SCG1	6054/367830	0.999	30156	0.825	0.120903	0.5167	0	-3	18	0.4278	-4	1	0	0	0.000000	-3.1667	-1	0.1	0.450000	0.2222	0.2278	0	0	0	0	3.1667	0.1177	-3.1667	1	0	0	0	0.3718	1.1836	0.7845	0.3333	0.1382	0.6154	0.1921	0.01	-0.3167
HIP_30157	AGSLQPLAL	SPETLISD	AGSLQPLALSPETLISD		NPY	770/49410	0.996	30157	0.825	0.207693	0.5833	0.3333	-2	17	0.3588	-2	0	1	0	0.000000	-5.1	-1	0.1833	0.450000	0.1111	0.3389	1	0	1	0	5.1	0.1977	0	1	0	0	0	0.4167	1.155	0.8533	0.3333	0.1736	0.7282	0.2431	0.0336	-0.935
HIP_30158	GERGFFYT	LDQLLHYRK	GERGFFYTLDQLLHYRK		SCG1	24352/367830	0.982	30158	0.825	0.050785	-0.4	0	2	17	-0.8059	1	3	1	0	0.000000	-1.3333	-1	0.05	1.650000	0.1111	1.5389	0	0	0	0	1.3333	-0.6156	-4	0	1	0	0	0.4103	1.0805	0.809	0.1667	0.1683	0.6544	-0.1641	0.0025	-0.0667
HIP_30159	LVCGERGFF	RDNMNDNF	LVCGERGFFRDNMNDNF		SCG1	19613/367830	0.990	30159	0.825	0.098404	-1.05	0.1667	-1	17	-0.5882	-1	2	1	0	0.000000	-5.5667	0	0.0667	1.175000	0.4444	0.7306	0	1	0	1	0	-0.7671	-11.1333	1	0	0	0	0.4269	0.9355	1.0123	0	0.1823	1.0248	-0.4483	0.0044	-0.3711
HIP_30160	QCCTSICSL	YQLIEISRN	QCCTSICSLYQLIEISRN		SCG2	13239/334890	0.998	30160	0.825	-0.000092	0.75	0.1667	0	18	0.3167	0	1	0	0	0.000000	-2.1667	0	-0.0167	1.125000	0	1.125	0	0	0	0	0	0.8438	-2.1667	1	0	0	0	0.4103	1.0922	0.8323	0.3333	0.1683	0.6927	0.3077	0.0003	0.0361
HIP_30161	CGERGFFYT	GAGRSRSN	CGERGFFYTGAGRSRSN		SCG2	6415/334890	0.999	30161	0.825	0.116845	0.3	0.3333	2	17	-0.9294	2	3	1	0	0.000000	0.0667	0	0.1167	0.000000	0.1111	-0.1111	0	2	0	1	0	-0.0333	0.2	0	1	0	0	0.4718	0.9169	1.0212	0.3333	0.2226	1.0428	0.1415	0.0136	0.0078
HIP_30162	GGPGAGS	VALNHLKA	GGPGAGSVALNHLKA		IAPP	261/45018	0.999	30162	0.825	0.182553	1.7333	0.3333	2	15	0.1733	1	0	1	0	0.000000	3.0667	0	-0.15	0.450000	0	0.45	0	1	0	1	0	0.78	0	0	1	0	0	0.35	1.0933	0.8367	0.6667	0.1225	0.7	0.6067	0.0225	-0.46
HIP_30163	CTSICSLYQ	GSEEYRAPR	CTSICSLYQGSEEYRAPR		SCG1	7398/367830	0.999	30163	0.825	0.178878	-0.95	0.3333	0	18	-0.6722	0	1	0	0	0.000000	-1.2333	-1	0.15	0.625000	0.2222	0.4028	0	1	0	1	1.2333	-0.3826	-1.2333	0	1	0	0	0.5218	1.0403	0.9712	0	0.2723	0.9431	-0.4957	0.0225	-0.185
HIP_30164	LVEALYLVC	EEEEEMAV	LVEALYLVCEEEEEMAV		CMGA	4746/247050	0.999	30164	0.825	0.115296	0	0	-6	17	0.5706	-6	1	1	3	0.000000	-7	-3	0.15	2.000000	0.4444	1.5556	0	0	0	0	21	0	-7	0	0	0	0	0.4385	1.3186	0.6878	-0.1667	0.1922	0.4731	0	0.0225	-1.05
HIP_30165	GERGFFYTP	LNQGSSQET	GERGFFYTPLNQGSSQET		SCG2	6005/334890	0.999	30165	0.825	-0.156266	-1.1333	0.1667	-1	18	-1.0944	-1	3	0	0	0.000000	0.1333	0	-0.0167	1.650000	0	1.65	1	0	1	0	0	-1.87	0.4	0	0	0	1	0.5218	0.9169	1.0712	0.1667	0.2723	1.1474	-0.5914	0.0003	-0.0022
HIP_30166	SLYQLENYC	DEPQWSLYP	SLYQLENYCDEPQWSLYP		SCG1	6544/367830	0.999	30166	0.825	0.069260	-1.8167	0.1667	-3	18	-0.9278	-3	1	0	0	0.000000	-2.1	-2	0.1167	0.950000	0.3333	0.6167	1	0	1	0	4.2	-1.1203	-2.1	0	0	0	0	0.5321	0.9691	1.0901	-0.3333	0.2831	1.1884	-0.9666	0.0136	-0.245
HIP_30167	DLQVGQVEL	QFPPMYEEN	DLQVGQVELQFPPMYEEN		SCG2	6570/334890	0.999	30167	0.825	0.032949	0.3667	0.1667	-4	18	-0.6778	-4	1	0	0	0.000000	-2.2667	1	0.0667	0.000000	0.1111	-0.1111	1	0	1	0	-2.2667	-0.0407	-2.2667	1	0	0	0	0.4167	1.125	0.8067	0.3333	0.1736	0.6507	0.1528	0.0044	-0.1511
HIP_30168	CTSICSLYQ	RPDRSSQGG	CTSICSLYQRPDRSSQGG		SCG1	24244/367830	0.982	30168	0.825	0.203862	-1.7667	0.1667	1	18	-0.7667	1	1	0	0	0.000000	-2.8667	0	0.1167	0.625000	0.3333	0.2917	1	0	1	0	0	-0.5153	-2.8667	0	1	0	0	0.4654	1.0074	0.9526	-0.1667	0.2166	0.9091	-0.8222	0.0136	-0.3344
HIP_30169	SLYQLENYC	EEPGETQNA	SLYQLENYCEEPGETQNA		SCG1	5468/367830	0.999	30169	0.825	0.048001	-1.8167	0.1667	-4	18	-1.2333	-4	1	0	0	0.000000	-2.1	-2	0.1167	0.950000	0.3333	0.6167	1	0	1	0	4.2	-1.1203	-2.1	0	1	0	0	0.5603	1.0505	1.0623	-0.3333	0.3139	1.1285	-1.0178	0.0136	-0.245
HIP_30170	SLQPLALEG	EEEAEAGEE	SLQPLALEGEEEAEAGEE		CMGA	3731/247050	0.999	30170	0.825	-0.123956	-1.7667	0.1667	-7	18	-0.8	-7	0	0	1	0.000000	-3.4667	-2	0.0167	1.400000	0.4444	0.9556	0	1	0	1	6.9333	-1.6881	0	0	0	0	1	0.55	1.3067	0.8267	-0.5	0.3025	0.6834	-0.9717	0.0003	-0.0578
HIP_30171	SICSLYQLE	DTPNRQYWD	SICSLYQLEDTPNRQYWD		SCG2	12210/334890	0.998	30171	0.825	-0.092927	-1.5	0.1667	-2	18	-1.0167	-2	1	0	0	0.000000	-0.8667	0	0.0667	1.900000	0.2222	1.6778	1	0	1	0	0	-2.5167	-0.8667	0	0	0	1	0.5218	1.0569	0.9345	-0.1667	0.2723	0.8733	-0.7827	0.0044	-0.0578
HIP_30172	SHLVEALYL	VSMASLGEK	SHLVEALYLVSMASLGEK		SCG1	7647/367830	0.999	30172	0.825	0.034508	1.9333	0.1667	0	18	0.6056	-1	1	0	0	1.000000	-0.3333	0	0.0667	1.500000	0.1111	1.3889	0	0	0	0	0	2.6852	-0.3333	1	0	0	0	0.3051	1.1136	0.7012	0.6667	0.0931	0.4916	0.5899	0.0044	-0.0222
HIP_30173	CCTSICSLY	WNPVEEKIE	CCTSICSLYWNPVEEKIE		SCG2	6222/334890	0.999	30173	0.825	-0.064006	-0.7167	0.3333	-2	18	0.0278	-2	2	0	0	0.000000	-2.5667	0	0.05	3.125000	0	3.125	1	0	1	0	0	-2.2396	-5.1333	0	1	0	0	0.4936	0.8972	1.099	0.3333	0.2436	1.2077	-0.3537	0.0025	-0.1283
HIP_30174	CCTSICSLY	SEEEPEYGE	CCTSICSLYSEEEPEYGE		SCG1	3341/367830	1.000	30174	0.825	0.003039	-1.0167	0.3333	-5	18	-0.5222	-5	1	0	1	0.000000	-3.1667	-2	0.1	2.075000	0.3333	1.7417	0	0	0	0	6.3333	-1.7707	-3.1667	0	1	0	0	0.5051	1.1569	0.8812	-0.1667	0.2552	0.7764	-0.5135	0.01	-0.3167
HIP_30175	CGERGFFYT	DKETTENE	CGERGFFYTDKETTENE		SCG1	19779/367830	0.990	30175	0.825	0.119033	-1.6833	0	-3	17	-1.5	-3	3	1	0	0.000000	-3.9	-1	0.1	0.000000	0.3333	-0.3333	0	0	0	0	3.9	0.5611	-11.7	0	1	0	0	0.4487	1.1008	0.8368	-0.3333	0.2013	0.7002	-0.7553	0.01	-0.39
HIP_30176	GERGFFYT	EGHHRVQEN	GERGFFYTEGHHRVQEN		SCG1	18244/367830	0.992	30176	0.825	0.091609	-1.05	0.1667	1	17	-1.5471	-1	3	1	0	0.000000	-2.6333	-1	0.0833	0.700000	0.2222	0.4778	0	1	0	1	2.6333	-0.5017	-7.9	0	1	0	0	0.4936	1.0138	0.9356	-0.1667	0.2436	0.8754	-0.5183	0.0069	-0.2194
HIP_30177	GIVEQCCTS	EEGSANRRP	GIVEQCCTSEEGSANRRP		CMGA	6169/247050	0.998	30177	0.825	0.013649	-1.0667	0.3333	-1	18	-0.8722	-1	0	0	0	0.000000	-2.8	-2	0.0667	0.000000	0.2222	-0.2222	0	1	0	1	5.6	0.237	0	0	1	0	0	0.5885	1.0286	1.0078	-0.3333	0.3463	1.0157	-0.6277	0.0044	-0.1867
HIP_30178	CSLYQLENY	DEPQWSLYP	CSLYQLENYDEPQWSLYP		SCG1	16062/367830	0.994	30178	0.825	-0.036883	-2.8167	0.1667	-3	18	-0.9278	-3	1	0	0	0.000000	-0.1	-1	0.0667	0.000000	0.3333	-0.3333	1	0	1	0	0.1	0.9389	-0.1	0	1	0	0	0.5603	1.0505	1.0623	-0.5	0.3139	1.1285	-1.5781	0.0044	-0.0067
HIP_30179	GERGFFYT	MDKARRHPQ	GERGFFYTMDKARRHPQ		SCG1	32271/367830	0.957	30179	0.825	0.112475	-0.7833	0	3	17	-1.5412	2	3	1	0	0.000000	-2.1	0	0.05	0.700000	0.3333	0.3667	0	0	0	0	0	-0.2872	-6.3	0	1	0	0	0.3821	1.0491	0.8125	0	0.146	0.6617	-0.2993	0.0025	-0.105
HIP_30180	AGSLQPLAL	EEPGETQNA	AGSLQPLALEEPGETQNA		SCG1	5988/367830	0.999	30180	0.825	0.154301	0.1333	0.1667	-3	18	-0.5389	-3	0	0	0	0.000000	-6	-2	0.1667	0.450000	0.2222	0.2278	1	0	1	0	12	0.0304	0	1	0	0	0	0.4167	1.2733	0.7767	0.1667	0.1736	0.6032	0.0556	0.0278	-1
HIP_30181	SHLVEALYL	AENEPQSA	SHLVEALYLAENEPQSA		SCG2	10665/334890	0.999	30181	0.825	0.154474	0.1833	0	-2	17	-0.2471	-3	1	1	0	1.000000	-3.8333	-1	0.1	0.450000	0.3333	0.1167	0	0	0	0	3.8333	0.0214	-3.8333	1	0	0	0	0.3718	1.2086	0.8112	0.3333	0.1382	0.658	0.0682	0.01	-0.3833
HIP_30182	ICSLYQLEN	LANFLVHSS	ICSLYQLENLANFLVHSS		IAPP	658/45018	0.998	30182	0.825	-0.091021	-0.1833	0	0	18	0.5611	-1	2	0	0	0.000000	1.7667	1	-0.05	1.650000	0.1111	1.5389	0	0	0	0	1.7667	-0.2821	3.5333	0	1	0	0	0.4	1.15	0.92	0.3333	0.16	0.8464	-0.0733	0.0025	-0.0883
HIP_30183	GERGFFYTP	FEKPLDSQS	GERGFFYTPFEKPLDSQS		SCG2	14017/334890	0.998	30183	0.825	-0.105191	-1.3667	0.1667	-1	18	-0.9889	-1	4	0	0	0.000000	-0.3333	0	-0.0167	1.400000	0.2222	1.1778	1	0	1	0	0	-1.6096	-1.3333	0	0	0	1	0.4936	1.0255	0.939	-0.1667	0.2436	0.8817	-0.6746	0.0003	0.0056
HIP_30184	VCGERGFFY	PEDQELES	VCGERGFFYPEDQELES		CMGA	6130/247050	0.998	30184	0.825	0.151790	-0.7167	0.1667	-4	17	-0.8176	-4	3	1	0	0.000000	-4.3	-2	0.1667	0.700000	0.3333	0.3667	1	0	1	0	8.6	-0.2628	-12.9	0	1	0	0	0.4269	1.0872	0.879	0	0.1823	0.7726	-0.306	0.0278	-0.7167
HIP_30185	YLVCGERGF	IVLSVALNH	YLVCGERGFIVLSVALNH		IAPP	549/45018	0.998	30185	0.825	0.107056	1.7333	0.1667	1	18	1	0	1	0	0	1.000000	4.8667	-1	-0.1333	1.125000	0.2222	0.9028	0	1	0	1	-4.8667	1.5648	4.8667	1	0	0	0	0.3218	1.0269	0.7462	0.5	0.1036	0.5567	0.5578	0.0178	-0.6489
HIP_30186	VCGERGFFY	ALPDGLSVE	VCGERGFFYALPDGLSVE		SCG2	1065/334890	1.000	30186	0.825	-0.017521	1.3833	0.1667	-2	18	0.3333	-2	3	0	0	0.000000	-0.1	0	0.0667	1.150000	0.2222	0.9278	1	0	1	0	0	1.2834	-0.3	0	1	0	0	0.3218	1.1236	0.7978	0.6667	0.1036	0.6365	0.4451	0.0044	-0.0067
HIP_30187	QHLCGSHLV	LAAVNSMPV	QHLCGSHLVLAAVNSMPV		SCG1	13812/367830	0.996	30187	0.825	0.000067	2.0333	0	2	18	0.8333	0	0	0	0	1.000000	0.8667	0	0	2.950000	0	2.95	0	0	0	0	0	5.9983	0	1	0	0	0	0.2385	1.2636	0.6212	0.6667	0.0569	0.3858	0.4849	0	0
HIP_30188	AGSLQPLAL	PEDLEWERY	AGSLQPLALPEDLEWERY		SCG1	4858/367830	0.999	30188	0.825	0.144035	0.1333	0.1667	-3	18	-0.5444	-3	0	0	0	0.000000	-6	-2	0.1667	1.400000	0.2222	1.1778	1	0	1	0	12	0.157	0	1	0	0	0	0.3885	1.1919	0.8045	0.1667	0.1509	0.6472	0.0518	0.0278	-1
HIP_30189	TSICSLYQ	QEEERLSKE	TSICSLYQQEEERLSKE		CMGA	11701/247050	0.993	30189	0.825	0.124988	-1.9167	0	-2	17	-1.1294	-2	1	1	1	0.000000	-3.1667	-2	0.1	0.000000	0.4444	-0.4444	0	0	0	0	6.3333	0.8519	-3.1667	0	1	0	0	0.5051	1.2603	0.8078	-0.1667	0.2552	0.6526	-0.9682	0.01	-0.3167
HIP_30190	TSICSLYQ	EQEEERLSK	TSICSLYQEQEEERLSK		CMGA	11702/247050	0.993	30190	0.825	0.124988	-1.9167	0	-2	17	-1.1294	-2	1	1	1	0.000000	-3.1667	-2	0.1	0.000000	0.4444	-0.4444	0	0	0	0	6.3333	0.8519	-3.1667	0	1	0	0	0.5051	1.2603	0.8078	-0.1667	0.2552	0.6526	-0.9682	0.01	-0.3167
HIP_30191	VEALYLVC	VGSNTYGKR	VEALYLVCVGSNTYGKR		IAPP	943/45018	0.996	30191	0.825	0.220995	2.25	0.3333	1	17	0.2471	1	1	1	0	0.000000	-2.5	0	0.15	0.625000	0	0.625	0	1	0	1	0	1.4062	-2.5	0	0	0	0	0.3885	0.9569	0.8512	0.5	0.1509	0.7245	0.874	0.0225	-0.375

HIP_30650	GSHLVEALY	RRHPQGAWK	GSHLVEALYRRHPQGAWK		SCG1	34592/367830	0.947	30650	0.825	0.145406	-1.3167	0	4	18	-0.9056	2	1	0	0
HIP_30651	GSLQPLALE	LLQKEPDLR	GSLQPLALELLQKEPDLR		SCG2	12792/334890	0.998	30651	0.825	-0.044423	1.0333	0	-1	18	-0.3167	-1	0	0	0
HIP_30652	SICSLYQLE	PEDQELESL	SICSLYQLEPEDQELESL		CMGA	6527/247050	0.998	30652	0.825	-0.091006	-1.9667	0.1667	-5	18	-0.4222	-5	1	0	0
HIP_30653	QHLCGSHLV	SEEERGLEP	QHLCGSHLVSEEERGLEP		SCG1	8783/367830	0.998	30653	0.825	0.173075	-0.5	0.1667	-1	18	-0.7944	-3	0	0	1
HIP_30654	LVEALYLVC	LNPHPSLGY	LVEALYLVCLNPHPSLGY		CMGA	4475/247050	0.999	30654	0.825	0.116183	1.5333	0.1667	0	18	0.8056	-1	1	0	0
HIP_30655	SHLVEALY	AYFMSDTRE	SHLVEALYAYFMSDTRE		SCG1	14115/367830	0.995	30655	0.825	0.001560	1.2667	0	-1	17	-0.1765	-2	3	1	0
HIP_30656	GSLQPLALE	REEARFQDK	GSLQPLALEREEARFQDK		SCG1	28766/367830	0.970	30656	0.825	0.137771	-1.5667	0	-1	18	-1.05	-1	0	0	0
HIP_30657	VCGERGFF	GAPGKWQQQ	VCGERGFFGAPGKWQQQ		SCG1	2493/367830	1.000	30657	0.825	0.055209	0.8333	0.5	1	17	-0.7294	1	2	1	1
HIP_30658	GERGFFYT	NAAGSGRLQ	GERGFFYTNAAGSGRLQ		SCG2	17104/334890	0.997	30658	0.825	-0.013675	0.15	0	1	17	-0.6412	1	3	1	0
HIP_30659	ALYLVCGER	EEEEEEEAE	ALYLVCGEREEEEEEEAE		CMGA	6075/247050	0.998	30659	0.825	-0.108035	-3.15	0.1667	-8	18	-1.1	-8	0	0	5
HIP_30660	VEQCCTSIC	NTYGKRNAV	VEQCCTSICNTYGKRNAV		IAPP	2529/45018	0.985	30660	0.825	0.046563	0.1167	0.1667	1	18	-0.2278	1	1	0	0
HIP_30661	CTSICSLYQ	LNHLKATPI	CTSICSLYQLNHLKATPI		IAPP	2737/45018	0.982	30661	0.825	-0.070974	-0.65	0	2	18	0.4	1	1	0	0
HIP_30662	LVEALYLVC	IEKNEQIND	LVEALYLVCIEKNEQIND		SCG2	10104/334890	0.999	30662	0.825	0.093579	1.2667	0	-3	18	0.1889	-3	1	0	0
HIP_30663	LVEALYLVC	SPEMIRALE	LVEALYLVCSPEMIRALE		SCG2	5275/334890	1.000	30663	0.825	0.183034	0.7667	0.3333	-2	18	0.9667	-2	1	0	0
HIP_30664	LVEALYLV	TSYFPNPYN	LVEALYLVTSYFPNPYN		SCG2	12444/334890	0.998	30664	0.825	0.129952	0.65	0.1667	-1	17	0.3118	-1	3	1	0
HIP_30665	LVCGERGFF	KSGYPKTP	LVCGERGFFKSGYPKTP		SCG2	16697/334890	0.997	30665	0.825	0.156553	0.0167	0.5	2	17	-0.4059	2	3	1	0
HIP_30666	NQHLCGSHL	STENVPRTR	NQHLCGSHLSTENVPRTR		NPY	3218/49410	0.972	30666	0.825	0.158605	-0.8667	0.3333	3	18	-1.1167	1	0	0	0
HIP_30667	ERGFFYTPK	RSQEESEEG	ERGFFYTPKRSQEESEEG		SCG1	18277/367830	0.992	30667	0.825	-0.092428	-2.5	0.3333	-2	18	-1.9056	-2	2	0	0
HIP_30668	GSLQPLAL	EGLSQGLV	GSLQPLALEGLSQGLV		CMGA	2518/247050	0.999	30668	0.825	0.182847	1.55	0.1667	-1	16	0.6312	-1	0	0	0
HIP_30669	HLVEALYLV	RSETHAAGH	HLVEALYLVRSETHAAGH		SCG1	17917/367830	0.992	30669	0.825	0.235413	-0.35	0.1667	2	18	0.05	-1	1	0	0
HIP_30670	EQCCTSICS	GPNNQKRER	EQCCTSICSGPNNQKRER		SCG2	5292/334890	1.000	30670	0.825	0.157110	0.1167	0.5	1	18	-1.4556	1	0	0	0
HIP_30671	HLVEALYLV	TNTHPPAS	HLVEALYLVTNTHPPAS		CMGA	8773/247050	0.996	30671	0.825	0.226049	0.3	0	1	17	0.1941	-1	1	1	0
HIP_30672	HLVEALYL	WKELDRNY	HLVEALYLWKELDRNY		SCG1	22641/367830	0.985	30672	0.825	0.156958	-0.3333	0	0	16	-0.4938	-1	2	0	0
HIP_30673	SLYQLENYC	EEGENYQKG	SLYQLENYCEEGENYQKG		SCG1	5357/367830	0.999	30673	0.825	0.093718	-1.6167	0.1667	-3	18	-1.5167	-3	1	0	0
HIP_30674	HLVEALYLV	KRTNEIVEE	HLVEALYLVKRTNEIVEE		SCG2	20400/334890	0.996	30674	0.825	0.189215	-0.4	0	-1	18	-0.0444	-2	1	0	0
HIP_30675	SLYQLENYC	AILSSTNVG	SLYQLENYCAILSSTNVG		IAPP	566/45018	0.998	30675	0.825	-0.039594	1.3	0	-1	18	0.2389	-1	1	0	0
HIP_30676	LVEALYLVC	ELKEAVEEP	LVEALYLVCELKEAVEEP		CMGA	7474/247050	0.997	30676	0.825	0.113119	1.15	0	-4	18	0.5333	-4	1	0	0
HIP_30677	HLVEALYL	GAWKELDR	HLVEALYLGAWKELDR		SCG1	9228/367830	0.998	30677	0.825	0.126231	1.1333	0.1667	0	16	-0.1063	-1	2	0	0
HIP_30678	EAEDLQVG	ERGFFYTPK	EAEDLQVGERGFFYTPK		INSB	165/4968	0.982	30678	0.825	-0.014672	-1.35	0.3333	-2	17	-0.8765	-2	2	1	0
HIP_30679	GSLQPLAL	NFLVHSSN	GSLQPLALNFLVHSSN		IAPP	1687/45018	0.993	30679	0.825	0.069702	2.0833	0	1	16	0.3687	0	1	0	0
HIP_30680	ERGFFYTPK	GSLPSEEKG	ERGFFYTPKGSLPSEEKG		SCG1	7613/367830	0.999	30680	0.825	-0.115663	-0.6	0.5	0	18	-1.1889	0	2	0	0
HIP_30681	LCGSHLVEA	GERATRLFE	LCGSHLVEAGERATRLFE		SCG2	17579/334890	0.997	30681	0.825	0.215176	-0.9833	0.1667	0	18	-0.0278	-1	0	0	0
HIP_30682	GSLQPLAL	AMDLELQKI	GSLQPLALAMDLELQKI		SCG1	12328/367830	0.997	30682	0.825	0.050735	1.6	0	-1	17	0.4882	-1	0	1	0
HIP_30683	SHLVEALYL	RLQNGQNG	SHLVEALYLRLQNGQNG		SCG2	26195/334890	0.993	30683	0.825	0.223541	0.35	0	1	17	-0.4059	0	1	1	0
HIP_30684	VCGERGFFY	WAEGGGHS	VCGERGFFYWAEGGGHS		SCG1	18741/367830	0.991	30684	0.825	0.072717	0.2833	0	0	17	-0.3294	-1	4	1	1
HIP_30685	LVEALYLVC	SVALNHLK	LVEALYLVCSVALNHLK		IAPP	1442/45018	0.994	30685	0.825	0.046058	2.6167	0.1667	1	17	1.2647	0	1	1	0
HIP_30686	ERGFFYTPK	LAEAYPSKP	ERGFFYTPKLAEAYPSKP		NPY	1744/49410	0.989	30686	0.825	0.026043	-0.6833	0.1667	1	18	-0.8667	1	2	0	0
HIP_30687	CGERGFFYT	DSLSEEDWM	CGERGFFYTDSLSEEDWM		SCG2	3658/334890	1.000	30687	0.825	-0.020374	0.05	0.1667	-4	18	-0.75	-4	3	0	0
HIP_30688	CCTSICSLY	ESTENVPRT	CCTSICSLYESTENVPRT		NPY	955/49410	0.995	30688	0.825	0.066622	-0.55	0.3333	-1	18	-0.1333	-1	1	0	0
HIP_30689	LYLVCGERG	EEGSANRRP	LYLVCGERGEEGSANRRP		CMGA	15083/247050	0.987	30689	0.825	-0.064929	-2.6333	0.3333	0	18	-0.9056	0	0	0	0
HIP_30690	ICSLYQLEN	FLVHSSNNF	ICSLYQLENFLVHSSNNF		IAPP	653/45018	0.998	30690	0.825	0.005701	1.2667	0	0	18	0.2111	-1	2	0	0
HIP_30691	ICSLYQLEN	MYEENSRDN	ICSLYQLENMYEENSRDN		SCG2	11476/334890	0.999	30691	0.825	-0.076614	-1.0167	0	-3	18	-1.1222	-3	2	0	0
HIP_30692	LVCGERGFF	QWSLYPSDS	LVCGERGFFQWSLYPSDS		SCG1	13346/367830	0.996	30692	0.825	0.030551	0	0.3333	-1	18	-0.1167	-1	3	0	0
[illegible]																			

HIP_31161	SICSLYQLE	YDPLQWKSS	SICSLYQLEYDPLQWKSS		SCG1	18692/367830	0.991	31161	0.824	-0.111029	-1.6	0.1667	-1	18	-0.4333	-1	2	0	0
HIP_31162	GSHLVEALY	NGSVEPERE	GSHLVEALYNGSVEPERE		SCG2	1863/334890	1.000	31162	0.824	0.155003	-0.0667	0.3333	-2	18	-0.7056	-3	1	0	0
HIP_31163	ICSLYQLEN	MRESTENVP	ICSLYQLENMRESTENVP		NPY	3967/49410	0.958	31163	0.824	-0.052278	-1.55	0	-2	18	-0.5556	-2	1	0	0
HIP_31164	HLCGSHLVE	RQAENEPQS	HLCGSHLVERQAENEPQS		SCG2	28276/334890	0.991	31164	0.824	0.054464	-0.2833	0	0	18	-1.0778	-2	0	0	0
HIP_31165	HLVEALYLV	SSHFERRD	HLVEALYLVSSHFERRD		SCG1	14930/367830	0.995	31165	0.824	0.171424	0.3167	0.3333	1	17	-0.2588	-1	2	1	0
HIP_31166	GSHLVEALY	VTALPVNSP	GSHLVEALYVTALPVNSP		CMGA	6833/247050	0.997	31166	0.824	-0.028232	1.6	0	0	18	0.5667	-1	1	0	0
HIP_31167	DLQVGQVEL	EALRQAENE	DLQVGQVELEALRQAENE		SCG2	12783/334890	0.998	31167	0.824	-0.034869	1.1	0	-4	18	-0.7222	-4	0	0	0
HIP_31168	HLCGSHLVE	APVSKRFPV	HLCGSHLVEAPVSKRFPV		SCG2	15404/334890	0.998	31168	0.824	0.006798	1.4833	0.1667	3	18	0.2111	1	0	0	0
HIP_31169	SHLVEALY	QEKAEKGRE	SHLVEALYQEKAEKGRE		SCG2	21443/334890	0.995	31169	0.824	0.251596	-1.1	0	0	17	-1.1824	-1	1	1	0
HIP_31170	SHLVEALYL	ENEKDRAD	SHLVEALYLENEKDRAD		SCG1	13030/367830	0.996	31170	0.824	0.174977	-0.7	0	-2	17	-0.9118	-3	1	1	0
HIP_31171	ERGFFYTPK	NFLVHSSNN	ERGFFYTPKNFLVHSSNN		IAPP	1200/45018	0.995	31171	0.824	0.003072	-0.5167	0.1667	2	18	-0.8222	1	3	0	0
HIP_31172	LVCGERGFF	EALAVDGAG	LVCGERGFFEALAVDGAG		CMGA	1589/247050	1.000	31172	0.824	-0.032938	1.2167	0.1667	-2	18	0.7167	-2	2	0	0
HIP_31173	SICSLYQLE	GRSRPQRSS	SICSLYQLEGRSRPQRSS		SCG1	19534/367830	0.990	31173	0.824	0.044030	-1.4833	0.3333	1	18	-0.9278	1	1	0	0
HIP_31174	AGSLQPLAL	APKENKPY	AGSLQPLALAPKENKPY		SCG2	19106/334890	0.996	31174	0.824	0.207936	0.95	0.1667	1	17	-0.5176	1	0	1	0
HIP_31175	CGERGFFYT	PYNQEKVL	CGERGFFYTPYNQEKVL		SCG2	17144/334890	0.997	31175	0.824	0.079294	-0.9333	0.1667	0	17	-0.7059	0	4	1	0
HIP_31176	HLVEALYLV	FPDFYDSE	HLVEALYLVFPDFYDSE		SCG1	6296/367830	0.999	31176	0.824	0.081142	0.7333	0.1667	-3	17	0.2941	-4	3	1	0
HIP_31177	LVEALYLV	EEEDNRDSS	LVEALYLVEEEDNRDSS		CMGA	5700/247050	0.998	31177	0.824	0.110855	-0.6333	0	-5	17	-0.6059	-5	1	1	1
HIP_31178	CSLYQLENY	YIENLRQQA	CSLYQLENYYIENLRQQA		SCG2	15137/334890	0.998	31178	0.824	-0.050498	-1.4333	0	-1	18	-0.75	-1	2	0	0
HIP_31179	ERGFFYTPK	ASLGEKRDH	ERGFFYTPKASLGEKRDH		SCG1	11059/367830	0.997	31179	0.824	0.010349	-0.2333	0.3333	2	18	-1.3611	1	2	0	0
HIP_31180	GSLQPLAL	GAEAASFQR	GSLQPLALGAEAASFQR		SCG2	5547/334890	0.999	31180	0.824	0.168527	1.2167	0.1667	0	17	0.1412	0	0	1	0
HIP_31181	GERGFFYTP	EEKKEEEG	GERGFFYTPEEKKEEEG		CMGA	13332/247050	0.990	31181	0.824	-0.000445	-2.4167	0.1667	-3	17	-1.9118	-3	3	1	1
HIP_31182	GERGFFYTP	KAEGNNQAP	GERGFFYTPKAEGNNQAP		CMGA	15313/247050	0.986	31182	0.824	-0.045925	-1.5333	0.1667	0	18	-1.2833	0	3	0	0
HIP_31183	SHLVEALYL	RADQTVLTE	SHLVEALYLRADQTVLTE		SCG1	21897/367830	0.986	31183	0.824	0.199606	0.0167	0	-1	18	0.1111	-2	1	0	0
HIP_31184	GGPGAGSLQ	GEQEHSQQK	GGPGAGSLQGEQEHSQQK		CMGA	6411/247050	0.998	31184	0.824	-0.043527	-1.3167	0.3333	0	18	-1.5389	-1	0	0	0
HIP_31185	VCGERGFF	ASEAHESSS	VCGERGFFASEAHESSS		SCG1	7524/367830	0.999	31185	0.824	0.026556	0.45	0.3333	-1	17	-0.3706	-2	2	1	1
HIP_31186	EQCCTSICS	DFYDSEEPV	EQCCTSICSDFYDSEEPV		SCG1	7691/367830	0.999	31186	0.824	-0.037594	0.7	0.1667	-5	18	-0.4444	-5	2	0	0
HIP_31187	LVEALYLV	QEEEEDNRD	LVEALYLVQEEEEDNRD		CMGA	4508/247050	0.999	31187	0.824	0.111147	-0.6333	0	-6	17	-0.9235	-6	1	1	2
HIP_31188	AGSLQPLAL	GGHSRERAD	AGSLQPLALGGHSRERAD		SCG1	8125/367830	0.998	31188	0.824	0.227567	0.9	0.3333	1	18	-0.5722	0	0	0	0
HIP_31189	GSLQPLALE	EEEGENYQK	GSLQPLALEEEEGENYQK		SCG1	12731/367830	0.996	31189	0.824	0.052572	-1.4	0	-4	18	-1.2889	-4	0	0	2
HIP_31190	SHLVEALYL	DSLEAGLPL	SHLVEALYLDSLEAGLPL		CMGA	5157/247050	0.998	31190	0.824	0.163508	0.9667	0.1667	-2	18	0.6667	-3	1	0	0
HIP_31191	GERGFFYTP	MLVKYPEII	GERGFFYTPMLVKYPEII		SCG2	3555/334890	1.000	31191	0.824	0.014876	1.05	0.1667	0	18	0.1	0	3	0	0
HIP_31192	LVEALYLVC	QASAIKKEE	LVEALYLVCQASAIKKEE		SCG1	10348/367830	0.998	31192	0.824	0.145460	1.3333	0.1667	-1	18	0.4611	-1	1	0	0
HIP_31193	VCGERGFFY	KHSGFEDEL	VCGERGFFYKHSGFEDEL		CMGA	14464/247050	0.988	31193	0.824	0.150165	-0.6	0.1667	-1	18	-0.5556	-2	3	0	0
HIP_31194	GERGFFYTP	YPSDSQVSE	GERGFFYTPYPSDSQVSE		SCG1	3662/367830	0.999	31194	0.824	-0.127385	-1.2167	0.5	-2	18	-1.0222	-2	4	0	0
HIP_31195	CTSICSLYQ	WLGAALSLI	CTSICSLYQWLGAALSLI		SCG2	2688/334890	1.000	31195	0.824	-0.092283	0.25	0.1667	0	18	1.3111	0	2	0	0
HIP_31196	SLYQLENYC	PNRQYWDED	SLYQLENYCPNRQYWDED		SCG2	10631/334890	0.999	31196	0.824	0.077758	-1.9833	0.1667	-3	18	-1.6444	-3	1	0	0
HIP_31197	GERGFFYT	GQNGERAT	GERGFFYTGQNGERAT		SCG2	13380/334890	0.998	31197	0.824	0.162608	-1.1	0.1667	0	16	-1.2437	0	3	0	0
HIP_31198	TSICSLYQL	DKDQELGEY	TSICSLYQLDKDQELGEY		SCG2	16898/334890	0.997	31198	0.824	0.146854	-1.9833	0	-3	18	-0.6556	-3	1	0	0
HIP_31199	CCTSICSLY	SQGLVDREK	CCTSICSLYSQGLVDREK		CMGA	1836/247050	0.999	31199	0.824	0.077782	-0.5	0.5	0	18	0.0056	0	1	0	0
HIP_31200	HLVEALYLV	AVEEPSSKD	HLVEALYLVAVEEPSSKD		CMGA	5445/247050	0.998	31200	0.824	0.002483	1.5333	0	-2	18	0.1111	-3	1	0	0
HIP_31201	LVCGERGFF	EEGSANRR	LVCGERGFFEEGSANRR		CMGA	8208/247050	0.996	31201	0.824	0.112572	-0.3667	0.3333	0	17	-0.6824	0	2	1	0
HIP_31202	ERGFFYTPK	SLYPSDSQV	ERGFFYTPKSLYPSDSQV		SCG1	8640/367830	0.998	31202	0.824	-0.016726	-0.75	0.3333	0	18	-0.8111	0	3	0	0
HIP_31203	LYQLENYCN	QDTKENREE	LYQLENYCNQDTKENREE		SCG1	12532/367830	0.996	31203	0.824	0.067339	-1.6667	0	-3	18	-2.0333	-3	1	0	0

[illegible]

HIP_31672	VEALYLVCG	PGPQLRRGW	VEALYLVCGPGPQLRRGW		CMGA	1220/247050	1.000	31672	0.824	0.136731	0.45	0.6667	1	18	0.0833
HIP_31673	YLVCGERGF	DLFQNRMLS	YLVCGERGFDLFQNRMLS		SCG2	9941/334890	0.999	31673	0.824	0.044508	0.1667	0.1667	0	18	-0.0167
HIP_31674	VEALYLVC	PEEKKEEEG	VEALYLVCPEEKKEEEG		CMGA	5535/247050	0.998	31674	0.824	0.158466	0.3167	0.1667	-4	17	-0.6941
HIP_31675	CGERGFFYT	LRGDERILS	CGERGFFYTLRGDERILS		CMGA	6873/247050	0.997	31675	0.824	0.064027	-0.05	0.1667	0	18	-0.4333
HIP_31676	AGSLQPLAL	EISRNLQI	AGSLQPLALEISRNLQI		SCG2	11807/334890	0.998	31676	0.824	0.193722	1.6	0.1667	0	17	0.3353
HIP_31677	TSICSLYQL	LAAMDLELQ	TSICSLYQLLAAMDLELQ		SCG1	4084/367830	0.999	31677	0.824	-0.040909	1.0667	0	-2	18	0.7722
HIP_31678	SHLVEALY	QVFLIVLSV	SHLVEALYQVFLIVLSV		IAPP	1310/45018	0.995	31678	0.824	-0.000184	1.3	0	0	17	1.6471
HIP_31679	LYQLENYCN	EEPGETQNA	LYQLENYCNEEPGETQNA		SCG1	6050/367830	0.999	31679	0.824	0.054723	-1.8167	0.1667	-4	18	-1.3833
HIP_31680	SHLVEALYL	QGSSQETD	SHLVEALYLQGSSQETD		SCG2	6483/334890	0.999	31680	0.824	0.224500	0.2667	0.3333	-2	17	-0.4765
HIP_31681	GSLQPLAL	AHESSSRG	GSLQPLALAHESSSRG		SCG1	18816/367830	0.991	31681	0.824	0.087976	0.75	0	1	16	-0.3313
HIP_31682	HLCGSHLVE	FGAILSSTN	HLCGSHLVEFGAILSSTN		IAPP	965/45018	0.996	31682	0.824	-0.037890	1.45	0.1667	1	18	0.55
HIP_31683	HLVEALYL	GPPKNDDTP	HLVEALYLGPPKNDDTP		SCG2	2653/334890	1.000	31683	0.824	0.192852	0.45	0.5	-1	17	-0.6412
HIP_31684	HLVEALYL	RFPVGPPKN	HLVEALYLRFPVGPPKN		SCG2	18033/334890	0.997	31684	0.824	0.166131	0.5	0.1667	2	17	-0.0412
HIP_31685	VCGERGFFY	HHTAEKRK	VCGERGFFYHHTAEKRK		SCG1	29568/367830	0.967	31685	0.824	0.106388	-0.4667	0	4	17	-1.1118
HIP_31686	EQCCTSICS	SQENHPQES	EQCCTSICSSQENHPQES		SCG1	5427/367830	0.999	31686	0.824	0.097498	-0.2667	0.3333	-2	18	-1.1778
HIP_31687	LVCGERGFF	DQLLHYRK	LVCGERGFFDQLLHYRK		SCG1	17969/367830	0.992	31687	0.824	0.045824	0.3333	0.1667	2	17	-0.2941
HIP_31688	QHLCGSHLV	SKEWEDSKR	QHLCGSHLVSKEWEDSKR		CMGA	14224/247050	0.988	31688	0.824	0.249105	-0.5667	0.1667	2	18	-1.2278
HIP_31689	HLVEALYLV	STHYRASE	HLVEALYLVSTHYRASE		SCG1	17780/367830	0.992	31689	0.824	0.170640	0.3333	0.1667	1	17	0.0353
HIP_31690	CCTSICSLY	EMKRSGQLG	CCTSICSLYEMKRSGQLG		SCG2	26781/334890	0.993	31690	0.824	0.014828	-0.6333	0.1667	1	18	0.05
HIP_31691	HLVEALYLV	QSIYQLIE	HLVEALYLVQSIYQLIE		SCG2	7309/334890	0.999	31691	0.824	0.145088	1.15	0.1667	-1	17	0.8118
HIP_31692	HLVEALYL	LANFLVHSS	HLVEALYLLANFLVHSS		IAPP	1474/45018	0.994	31692	0.824	0.026126	1.4	0	1	17	1.0294
HIP_31693	SLYQLENYC	FPEYNYDWW	SLYQLENYCFPEYNYDWW		SCG1	1468/367830	1.000	31693	0.824	-0.124987	-0.7667	0.1667	-3	18	-0.9722
HIP_31694	GSHLVEALY	EEEEAEAG	GSHLVEALYEEEEAEAG		CMGA	5144/247050	0.998	31694	0.824	0.093614	-1.0333	0	-5	17	-0.5824
HIP_31695	QHLCGSHLV	GPGPQLRRG	QHLCGSHLVGPGPQLRRG		CMGA	5603/247050	0.998	31695	0.824	0.208713	0.4	0.5	4	18	-0.55
HIP_31696	TSICSLYQL	GEEENELTL	TSICSLYQLGEEENELTL		SCG1	2945/367830	1.000	31696	0.824	0.123131	-1.4	0.1667	-4	18	-0.1944
HIP_31697	GSLQPLAL	SLQPLAL	GSLQPLALSLQPLAL		INSC	181/5184	0.998	31697	0.824	0.199835	1.4833	0.1667	0	15	0.9467
HIP_31698	VCGERGFFY	RELDLPVD	VCGERGFFYRELDLPVD		SCG2	18425/334890	0.997	31698	0.824	0.131952	0.0167	0	-2	17	-0.1529
HIP_31699	VELGGGPGA	QNGQNGERA	VELGGGPGAQNGQNGERA		SCG2	1242/334890	1.000	31699	0.824	-0.057157	-1.2667	0.5	-1	18	-0.9944
HIP_31700	CCTSICSLY	ERATRLFEK	CCTSICSLYERATRLFEK		SCG2	26636/334890	0.993	31700	0.824	0.013256	-0.75	0.1667	1	18	0
HIP_31701	GSLQPLAL	ELQKIAEKF	GSLQPLALELQKIAEKF		SCG1	11924/367830	0.997	31701	0.824	0.101224	1.0333	0	0	17	0.0882
HIP_31702	AGSLQPLAL	PPMYEENS	AGSLQPLALPPMYEENS		SCG2	4330/334890	1.000	31702	0.824	0.180692	1.35	0.3333	-2	17	-0.3059
HIP_31703	CSLYQLENY	ENENTKFEV	CSLYQLENYENENTKFEV		SCG1	22782/367830	0.985	31703	0.824	-0.026268	-3.1333	0	-3	18	-1.05
HIP_31704	GERGFFYTP	AALSLIPLI	GERGFFYTPAALSLIPLI		SCG2	3754/334890	1.000	31704	0.824	0.006924	0.6333	0.1667	0	18	0.8222
HIP_31705	SHLVEALYL	PNGSVEPE	SHLVEALYLPNGSVEPE		SCG2	4737/334890	1.000	31705	0.824	0.213637	0.1333	0.3333	-2	17	-0.1235
HIP_31706	LCGSHLVEA	ENKPYALNS	LCGSHLVEAENKPYALNS		SCG2	29187/334890	0.991	31706	0.824	0.208937	-1.4	0	0	18	-0.2389
HIP_31707	LVCGERGFF	FEVRLLRDP	LVCGERGFFFEVRLLRDP		SCG1	7170/367830	0.999	31707	0.824	-0.003208	1.45	0.1667	0	18	0.2389
HIP_31708	CTSICSLYQ	DLIEMLKTG	CTSICSLYQDLIEMLKTG		SCG2	14528/334890	0.998	31708	0.824	-0.051622	0.6333	0	-1	18	0.4556
HIP_31709	PGAGSLQP	RGFFYTPKT	PGAGSLQPRGFFYTPKT		INSB	224/4968	0.967	31709	0.824	0.003934	-0.5667	0.3333	2	17	-0.6
HIP_31710	CTSICSLYQ	EGGGHSRER	CTSICSLYQEGGGHSRER		SCG1	5791/367830	0.999	31710	0.824	0.174507	-0.8833	0.3333	1	18	-0.8333
HIP_31711	DLQVGQVEL	QLLQKEPDL	DLQVGQVELQLLQKEPDL		SCG2	11285/334890	0.999	31711	0.824	-0.029055	1.4333	0	-3	18	-0.3611
HIP_31712	LYQLENYCN	SKENIEKNE	LYQLENYCNSKENIEKNE		SCG2	15704/334890	0.997	31712	0.824	0.136241	-1.75	0.1667	-2	18	-1.5611
HIP_31713	GSLQPLALE	EEEEEEEEE	GSLQPLALEEEEEEEEEE		CMGA	3771/247050	0.999	31713	0.824	-0.005836	-1.4	0	-10	18	-1.5611
HIP_31714	RGFFYTPKT	KEESSPDYN	RGFFYTPKTKEESSPDYN		SCG2	25369/334890	0.993	31714	0.824	-0.008135	-2.85	0.1667	0	18	-1.6611
HIP_31715	GERGFFYT	QGSSQETDK	GERGFFYTQGSSQETDK		SCG2	7826/334890	0.999	31715	0.824	0.157054	-0.65	0.3333	-1	17	-1.5176
HIP_31716	CGERGFFY	QRNQLLQK	CGERGFFYQRNQLLQK		SCG2	23512/334890	0.994	31716	0.824	0.213403	-1.2	0	2	16	-1.05
HIP_31717	LVEALYLV	EQEHSQQKE	LVEALYLVEQEHSQQKE		CMGA	7574/247050	0.997	31717	0.824	0.139613	-0.6333	0	-2	17	-0.7118
HIP_31718	AGSLQPLAL	GAEEAQDPE	AGSLQPLALGAEEAQDPE		CMGA	79/247050	1.000	31718	0.824	0.137927	1.2167	0.1667	-4	18	-0.4
HIP_31719	CGERGFFY	ARAYGFRGP	CGERGFFYARAYGFRGP		CMGA	18494/247050	0.977	31719	0.824	0.111769	0.5667	0	2	17	-0.4882

HIP_32183	SLYQLENYC	NLITRQRYG	SLYQLENYCNLITRQRYG		NPY	2147/49410	0.985	32183	0.823	-0.061547	0.4167	0	1	18	-0.7722	1	1	0	0
HIP_32184	GERGFFYT	NSNQVKRV	GERGFFYTNSNQVKRV		SCG2	20019/334890	0.996	32184	0.823	0.166188	-1.1667	0.1667	2	16	-1.0312	2	3	0	0
HIP_32185	TSICSLYQ	EEPEYGEEI	TSICSLYQEEPEYGEEI		SCG1	7883/367830	0.998	32185	0.823	0.086040	-1.6	0.1667	-5	17	-0.7412	-5	2	1	0
HIP_32186	CTSICSLYQ	ETDKLAPVS	CTSICSLYQETDKLAPVS		SCG2	16053/334890	0.997	32186	0.823	0.125341	-1.45	0	-1	18	0.1111	-1	1	0	0
HIP_32187	CGERGFFY	QGDLQDTK	CGERGFFYQGDLQDTK		SCG1	11569/367830	0.997	32187	0.823	0.184577	-0.5167	0.1667	-1	16	-1.075	-1	3	0	0
HIP_32188	GIVEQCCTS	GKGRHHRGR	GIVEQCCTSGKGRHHRGR		SCG1	20478/367830	0.989	32188	0.823	0.124334	-0.6167	0.5	5	18	-1.1222	3	0	0	0
HIP_32189	LVEALYLVC	EWEDSKRW	LVEALYLVCEWEDSKRW		CMGA	9205/247050	0.996	32189	0.823	0.095871	0.4333	0	-2	17	-0.1294	-2	2	1	0
HIP_32190	SHLVEALYL	LKEAVEEPS	SHLVEALYLLKEAVEEPS		CMGA	9915/247050	0.995	32190	0.823	0.138226	0.45	0	-2	18	0.0889	-3	1	0	0
HIP_32191	CGERGFFYT	GEEAVPEE	CGERGFFYTGEEAVPEE		CMGA	5770/247050	0.998	32191	0.823	0.109127	-1.1	0.1667	-4	17	-0.7471	-4	3	1	0
HIP_32192	VEQCCTSIC	SKEWEDSKR	VEQCCTSICSKEWEDSKR		CMGA	12898/247050	0.991	32192	0.823	0.147134	-0.3333	0.3333	-1	18	-0.9778	-1	1	0	0
HIP_32193	ERGFFYTPK	ALQGAKERA	ERGFFYTPKALQGAKERA		CMGA	8285/247050	0.996	32193	0.823	0.054431	-0.6833	0.1667	2	18	-0.9389	2	2	0	0
HIP_32194	GSHLVEALY	PGEDAPAED	GSHLVEALYPGEDAPAED		NPY	10/49410	1.000	32194	0.823	0.149470	-0.2	0.3333	-4	18	-0.5333	-5	1	0	0
HIP_32195	GSHLVEALY	AVEEPSSKD	GSHLVEALYAVEEPSSKD		CMGA	6393/247050	0.998	32195	0.823	-0.034068	1.1333	0	-2	18	-0.4	-3	1	0	0
HIP_32196	AGSLQPLAL	PQAEGDSEG	AGSLQPLALPQAEGDSEG		CMGA	2959/247050	0.999	32196	0.823	0.148181	1.0167	0.1667	-3	18	-0.3722	-3	0	0	0
HIP_32197	QHLCGSHLV	PVDNRNHNE	QHLCGSHLVPVDNRNHNE		SCG1	11253/367830	0.997	32197	0.823	0.166920	0.65	0.1667	2	18	-1.0778	-1	0	0	0
HIP_32198	CGERGFFY	AEGDSEGLS	CGERGFFYAEGDSEGLS		CMGA	5309/247050	0.998	32198	0.823	0.148839	0.3667	0.1667	-3	17	-0.5471	-3	3	1	0
HIP_32199	SICSLYQLE	YEDVVGGED	SICSLYQLEYEDVVGGED		SCG2	15041/334890	0.998	32199	0.823	-0.113978	-1.9167	0	-5	18	-0.1667	-5	2	0	0
HIP_32200	VEALYLVC	DERILSILR	VEALYLVCDERILSILR		CMGA	11598/247050	0.993	32200	0.823	0.193593	-0.1667	0	-1	17	0.9	-1	1	1	0
HIP_32201	YLVCGERGF	REDSLEAGL	YLVCGERGFREDSLEAGL		CMGA	11896/247050	0.992	32201	0.823	0.010426	-2.2667	0.1667	-2	18	-0.2	-2	1	0	0
HIP_32202	VEALYLVCG	YFPNPYNQE	VEALYLVCGYFPNPYNQE		SCG2	4950/334890	1.000	32202	0.823	-0.019660	1.0333	0.3333	-2	18	-0.1056	-2	3	0	0
HIP_32203	GPGAGSLQP	YYDPLQWKS	GPGAGSLQPYYDPLQWKS		SCG1	16669/367830	0.993	32203	0.823	0.032594	-1.2333	0.1667	0	18	-0.8944	0	2	0	0
HIP_32204	HLCGSHLVE	NVQKFPSPE	HLCGSHLVENVQKFPSPE		SCG2	23875/334890	0.994	32204	0.823	0.066825	0.2833	0	1	18	-0.4556	-1	0	0	0
HIP_32205	VEALYLVC	AEGGGHSRE	VEALYLVCAEGGGHSRE		SCG1	6901/367830	0.999	32205	0.823	0.189485	1.4	0.1667	-1	17	0.0353	-2	1	1	1
HIP_32206	LYQLENYCN	FKRTNEIVE	LYQLENYCNFKRTNEIVE		SCG2	25670/334890	0.993	32206	0.823	-0.000600	-1.3167	0	-1	18	-0.8111	-1	2	0	0
HIP_32207	VCGERGFFY	MPVSQECFE	VCGERGFFYMPVSQECFE		CMGA	670/247050	1.000	32207	0.823	-0.027795	1.4667	0.1667	-2	18	0.0389	-2	3	0	0
HIP_32208	ERGFFYTPK	GRAGTEALP	ERGFFYTPKGRAGTEALP		SCG2	11610/334890	0.998	32208	0.823	-0.088856	-1.55	0.3333	1	18	-0.7778	1	2	0	0
HIP_32209	LVCGERGFF	EEEEEEEA	LVCGERGFFEEEEEEEA		CMGA	5649/247050	0.998	32209	0.823	0.021544	-0.8833	0.1667	-7	17	-0.9059	-7	2	1	5
HIP_32210	LVCGERGFF	EEEEEEEA	LVCGERGFFEEEEEEEA		CMGA	5650/247050	0.998	32210	0.823	0.021544	-0.8833	0.1667	-7	17	-0.9059	-7	2	1	5
HIP_32211	ERGFFYTPK	AFLNERKQA	ERGFFYTPKAFLNERKQA		SCG1	9493/367830	0.998	32211	0.823	0.098870	0.3667	0.1667	2	18	-1.0556	2	3	0	0
HIP_32212	LYQLENYCN	KEPDLRLEN	LYQLENYCNKEPDLRLEN		SCG2	13464/334890	0.998	32212	0.823	0.122041	-1.8833	0.1667	-2	18	-1.2722	-2	1	0	0
HIP_32213	HLVEALYL	EEEEMAVV	HLVEALYLEEEEMAVV		CMGA	6775/247050	0.998	32213	0.823	0.140451	-0.7	0	-4	16	0.4687	-5	1	0	2
HIP_32214	GSLQPLAL	KELDRNYL	GSLQPLALKELDRNYL		SCG1	23738/367830	0.983	32214	0.823	0.208169	0.9667	0	0	16	-0.3563	0	0	0	0
HIP_32215	LVEALYLVC	HGYGEESE	LVEALYLVCHGYGEESE		SCG1	11207/367830	0.997	32215	0.823	0.078799	0.9333	0.1667	-3	17	0.1588	-4	2	1	0
HIP_32216	VNQHLCGSH	REEEEEEEE	VNQHLCGSHREEEEEEEE		CMGA	6184/247050	0.998	32216	0.823	-0.106429	-2.65	0.3333	-5	18	-2.0333	-7	0	0	6
HIP_32217	VCGERGFF	EDSKEAEKS	VCGERGFFEDSKEAEKS		CMGA	8361/247050	0.996	32217	0.823	0.081700	-0.4333	0.3333	-2	17	-1.0647	-2	2	1	0
HIP_32218	LVEALYLV	FLIVLSVAL	LVEALYLVFLIVLSVAL		IAPP	1088/45018	0.996	32218	0.823	-0.033767	2.9667	0	-1	17	2.6412	-1	2	1	0
HIP_32219	SICSLYQLE	TNTHPPASL	SICSLYQLETNTHPPASL		CMGA	14050/247050	0.989	32219	0.823	-0.088031	-1.35	0	0	18	-0.1	-1	1	0	0
HIP_32220	CCTSICSLY	EEATNTHPP	CCTSICSLYEEATNTHPP		CMGA	9477/247050	0.995	32220	0.823	-0.058730	-0.5833	0.1667	-1	18	-0.2389	-2	1	0	0
HIP_32221	CTSICSLY	LIVLSVALN	CTSICSLYLIVLSVALN		IAPP	746/45018	0.997	32221	0.823	0.009148	2.3667	0.1667	0	17	1.8529	0	1	1	0
HIP_32222	SHLVEALY	QENQMDKAR	SHLVEALYQENQMDKAR		SCG1	17279/367830	0.992	32222	0.823	0.219405	-1.0333	0	0	17	-1.0235	-1	1	1	0
HIP_32223	CCTSICSLY	NEKDRADQT	CCTSICSLYNEKDRADQT		SCG1	22266/367830	0.986	32223	0.823	0.071231	-1.5333	0.1667	-1	18	-0.7	-1	1	0	0
HIP_32224	CGERGFFYT	EEDNRDSS	CGERGFFYTEEDNRDSS		CMGA	11178/247050	0.993	32224	0.823	0.088825	-1.6167	0	-3	17	-1.5471	-3	3	1	0
HIP_32225	LCGSHLVEA	QFPPMYEEN	LCGSHLVEAQFPPMYEEN		SCG2	8280/334890	0.999	32225	0.823	0.048055	0.0333	0.1667	-2	18	-0.3111	-3	1	0	0

HIP_32694	LCGSHLVEA	PDLFQNRML	LCGSHLVEAPDLFQNRML		SCG2	12582/334890	0.998	32694	0.823	0.076006	0.2	0.1667	0	18	0.2167	-1
HIP_32695	CGERGFFY	KRLGELFNP	CGERGFFYKRLGELFNP		SCG1	29992/367830	0.965	32695	0.823	0.167216	-0.05	0	1	17	-0.5294	1
HIP_32696	CCTSICSLY	ALQGAKERA	CCTSICSLYALQGAKERA		CMGA	9100/247050	0.996	32696	0.823	-0.053102	0.6333	0.1667	1	18	0.3111	1
HIP_32697	CGERGFFY	QDLALQGAK	CGERGFFYQDLALQGAK		CMGA	12979/247050	0.991	32697	0.823	0.137519	0.1833	0	0	17	-0.3294	0
HIP_32698	ERGFFYTPK	AEFPDFYDS	ERGFFYTPKAEFPDFYDS		SCG1	8548/367830	0.998	32698	0.823	-0.006029	-0.85	0.1667	-2	18	-0.95	-2
HIP_32699	HLVEALYLV	GLEPGKGRH	HLVEALYLVGLEPGKGRH		SCG1	8863/367830	0.998	32699	0.823	0.187195	1.1	0.1667	2	18	-0.0278	0
HIP_32700	ERGFFYTPK	GALAEAYPS	ERGFFYTPKGALAEAYPS		NPY	792/49410	0.996	32700	0.823	-0.040593	-0.1667	0.3333	0	18	-0.4833	0
HIP_32701	PGAGSLQPL	QYWDEDLLM	PGAGSLQPLQYWDEDLLM		SCG2	4021/334890	1.000	32701	0.823	-0.028156	-1.1667	0.1667	-3	18	-0.3111	-3
HIP_32702	LVEALYL	ALRHYIN	LVEALYLALRHYIN		NPY	3124/49410	0.974	32702	0.823	0.104496	1.2333	0	1	14	0.7286	0
HIP_32703	EQCCTSICS	FPDFYDSEE	EQCCTSICSFPDFYDSEE		SCG1	1911/367830	1.000	32703	0.823	-0.040277	0.65	0.3333	-5	18	-0.5222	-5
HIP_32704	HLVEALYLV	VKDKETTEN	HLVEALYLVVKDKETTEN		SCG1	14987/367830	0.995	32704	0.823	0.125263	0.5833	0	-1	18	-0.3	-2
HIP_32705	GSHLVEALY	LNLLGMESA	GSHLVEALYLNLLGMESA		SCG2	7691/334890	0.999	32705	0.823	-0.039818	1.4	0	-1	18	0.6278	-2
HIP_32706	SHLVEALYL	SNALSKSSA	SHLVEALYLSNALSKSSA		SCG1	14602/367830	0.995	32706	0.823	0.210219	0.6333	0.1667	1	18	0.3	0
HIP_32707	RGFFYTPKT	GEDSSEEKH	RGFFYTPKTGEDSSEEKH		SCG1	17274/367830	0.993	32707	0.823	-0.055445	-2.2667	0.3333	0	18	-1.7	-1
HIP_32708	CGERGFFY	DWMRIILEA	CGERGFFYDWMRIILEA		SCG2	16599/334890	0.997	32708	0.823	0.062544	0.3	0	-1	17	0.1235	-1
HIP_32709	AGSLQPLAL	SSQESGEET	AGSLQPLALSSQESGEET		SCG1	7866/367830	0.998	32709	0.823	0.214065	0.7167	0.3333	-3	18	-0.4889	-3
HIP_32710	SHLVEALYL	GALAEAYP	SHLVEALYLGALAEAYP		NPY	194/49410	0.999	32710	0.823	0.058529	1.9167	0.1667	-1	17	0.6471	-2
HIP_32711	GGPGAGSLQ	EEDWMRIIL	GGPGAGSLQEEDWMRIIL		SCG2	14870/334890	0.998	32711	0.823	-0.032403	-1.8333	0.1667	-2	18	-0.1722	-2
HIP_32712	CCTSICSLY	DWMRIILEA	CCTSICSLYDWMRIILEA		SCG2	10171/334890	0.999	32712	0.823	-0.061977	-0.1333	0.1667	-1	18	0.9056	-1
HIP_32713	VCGERGFF	NSPMNKGDT	VCGERGFFNSPMNKGDT		CMGA	6945/247050	0.997	32713	0.823	0.119129	-0.1167	0.5	0	17	-0.7353	0
HIP_32714	GSLQPLAL	ESAANQKT	GSLQPLALESAANQKT		SCG2	11325/334890	0.999	32714	0.823	0.182665	1.15	0.1667	0	16	-0.3375	0
HIP_32715	CGERGFFYT	GPPKNDDT	CGERGFFYTGPPKNDDT		SCG2	3948/334890	1.000	32715	0.823	0.155902	-0.4667	0.5	-1	17	-1.2588	-1
HIP_32716	AGSLQPLAL	SGAEAASFQ	AGSLQPLALSGAEAASFQ		SCG2	3562/334890	1.000	32716	0.823	0.198469	1.6667	0.3333	-1	18	0.4389	-1
HIP_32717	LVEALYLVC	KGDTEVMKC	LVEALYLVCKGDTEVMKC		CMGA	8610/247050	0.996	32717	0.823	0.218811	0.45	0.1667	-1	18	0.6667	-1
HIP_32718	GERGFFYTP	AYPSKPDNP	GERGFFYTPAYPSKPDNP		NPY	780/49410	0.996	32718	0.823	-0.107943	-0.7833	0.3333	0	18	-1.2667	0
HIP_32719	ERGFFYTPK	LPLQVRGYP	ERGFFYTPKLPLQVRGYP		CMGA	7161/247050	0.997	32719	0.822	0.000667	-0.0333	0.3333	2	18	-0.6333	2
HIP_32720	CTSICSLYQ	GYEKRNLA	CTSICSLYQGYEKRNLA		SCG1	18970/367830	0.991	32720	0.822	0.117407	-1.0333	0.1667	1	17	-0.3118	1
HIP_32721	CGSHLVEAL	ESAANQKTS	CGSHLVEALESAANQKTS		SCG2	17066/334890	0.997	32721	0.822	0.025233	-0.0667	0.1667	0	18	-0.2722	-1
HIP_32722	TSICSLYQL	QNGQNGER	TSICSLYQLQNGQNGER		SCG2	11239/334890	0.999	32722	0.822	0.203940	-1.4	0.1667	0	17	-0.9	0
HIP_32723	TSICSLYQ	ITRQRYGKR	TSICSLYQITRQRYGKR		NPY	5083/49410	0.930	32723	0.822	0.112872	-0.2833	0	4	17	-0.8882	4
HIP_32724	SLYQLENYC	FNPYYDPLQ	SLYQLENYCFNPYYDPLQ		SCG1	1621/367830	1.000	32724	0.822	-0.105667	-0.7667	0.1667	-2	18	-0.75	-2
HIP_32725	TSICSLYQL	LALSLLVCL	TSICSLYQLLALSLLVCL		NPY	1240/49410	0.993	32725	0.822	-0.001736	1.4	0	0	18	1.9	0
HIP_32726	ERGFFYTPK	VARSETHAA	ERGFFYTPKVARSETHAA		SCG1	10049/367830	0.998	32726	0.822	0.035181	-0.7833	0.1667	2	18	-0.7444	1
HIP_32727	SHLVEALYL	VLKREPLNY	SHLVEALYLVLKREPLNY		IAPP	2485/45018	0.985	32727	0.822	0.066435	1.7333	0	1	18	0.1167	0
HIP_32728	GSHLVEALY	KSGELEQEE	GSHLVEALYKSGELEQEE		CMGA	4409/247050	0.999	32728	0.822	0.177500	-0.1333	0.3333	-3	18	-0.8	-4
HIP_32729	CTSICSLYQ	QAELKEAVE	CTSICSLYQQAELKEAVE		CMGA	10160/247050	0.995	32729	0.822	-0.059280	-1.0333	0	-2	18	-0.0056	-2
HIP_32730	CTSICSLYQ	EEKGHPQEE	CTSICSLYQEEKGHPQEE		SCG1	20709/367830	0.988	32730	0.822	0.115742	-1.9833	0	-2	18	-1.1333	-3
HIP_32731	SHLVEALY	FPDFYDSEE	SHLVEALYFPDFYDSEE		SCG1	9124/367830	0.998	32731	0.822	0.099395	0.3333	0.1667	-4	17	-0.4294	-5
HIP_32732	ERGFFYTPK	SHFERRDNM	ERGFFYTPKSHFERRDNM		SCG1	14092/367830	0.996	32732	0.822	0.013598	-1.2333	0.3333	2	18	-1.6167	1
HIP_32733	AGSLQPLAL	NLAAMDLEL	AGSLQPLALNLAAMDLEL		SCG1	9078/367830	0.998	32733	0.822	0.032639	1.9167	0	-2	18	0.8389	-2
HIP_32734	GSHLVEALY	EEEQIEQAI	GSHLVEALYEEEQIEQAI		SCG2	7964/334890	0.999	32734	0.822	0.098086	-1.0333	0	-4	18	-0.3222	-5
HIP_32735	LVCGERGFF	EKKELENLA	LVCGERGFFEKKELENLA		SCG1	25924/367830	0.978	32735	0.822	0.111581	-1.0167	0.1667	-1	18	-0.2833	-1
HIP_32736	SLYQLENYC	SEESNVSMA	SLYQLENYCSEESNVSMA		SCG1	9486/367830	0.998	32736	0.822	0.081824	-1.6833	0.1667	-3	18	-0.4889	-3
HIP_32737	SLYQLENYC	EESNVSMAS	SLYQLENYCEESNVSMAS		SCG1	9487/367830	0.998	32737	0.822	0.081824	-1.6833	0.1667	-3	18	-0.4889	-3
HIP_32738	SHLVEALYL	KTRHSEKSQ	SHLVEALYLKTRHSEKSQ		SCG1	30315/367830	0.964	32738	0.822	0.235255	-0.4667	0	3	18	-0.9	1
HIP_32739	AGSLQPLAL	GQVELGGGP	AGSLQPLALGQVELGGGP		INSC	64/5184	0.999	32739	0.822	0.188269	1.6167	0.1667	-1	18	0.3611	-1
HIP_32740	QHLCGSHLV	MESAANQKT	QHLCGSHLVMESAANQKT		SCG2	13344/334890	0.998	32740	0.822	0.161288	0.4	0.1667	2	18	-0.4	0
HIP_32741	GSLQPLALE	PDFYDSEEP	GSLQPLALEPDFYDSEEP		SCG1	4780/367830	0.999	32741	0.822	-0.026738	-0.0333	0.1667	-5	18	-0.7278	-5

HIP_33205	GIVEQCCTS	RQYWDEDLL	GIVEQCCTSRQYWDEDLL		SCG2	20600/334890	0.996	33205	0.822	0.007590	-1.3833	0.1667	-3	18	-0.4611	-3	2	0	0
HIP_33206	HLVEALYLV	SVEPERELD	HLVEALYLVSVEPERELD		SCG2	1461/334890	1.000	33206	0.822	0.109254	1.1	0.1667	-3	18	0.0389	-4	1	0	0
HIP_33207	GSHLVEALY	LGEGHHRVQ	GSHLVEALYLGEGHHRVQ		SCG1	5993/367830	0.999	33207	0.822	0.114989	0.7	0.1667	2	18	-0.35	-1	1	0	0
HIP_33208	HLVEALYL	DLQDTKEN	HLVEALYLDLQDTKEN		SCG1	13451/367830	0.996	33208	0.822	0.158654	0.5167	0	-2	16	-0.5563	-3	1	0	0
HIP_33209	CGERGFFY	GQLGIQEED	CGERGFFYGQLGIQEED		SCG2	2020/334890	1.000	33209	0.822	0.119844	0.7	0.1667	-3	17	-0.7059	-3	3	1	0
HIP_33210	LVEALYLVC	GGEDWNPVE	LVEALYLVCGGEDWNPVE		SCG2	2647/334890	1.000	33210	0.822	0.175035	1.0333	0.3333	-4	18	0.3444	-4	1	0	0
HIP_33211	SLYQLENYC	PGKGRHHRG	SLYQLENYCPGKGRHHRG		SCG1	16042/367830	0.994	33211	0.822	0.155980	-1.3667	0.3333	4	18	-1.4389	2	1	0	0
HIP_33212	LVEALYLVC	EGGGHSRE	LVEALYLVCEGGGHSRE		SCG1	5622/367830	0.999	33212	0.822	0.196847	1.0333	0.3333	-1	17	0.1529	-2	1	1	1
HIP_33213	CGERGFFYT	EPSSKDVM	CGERGFFYTEPSSKDVM		CMGA	8735/247050	0.996	33213	0.822	0.157502	-0.85	0.3333	-1	17	-0.6294	-1	3	1	0
HIP_33214	QCCTSICSL	QEEDLRKES	QCCTSICSLQEEDLRKES		SCG2	11987/334890	0.998	33214	0.822	0.070984	-0.8333	0.1667	-2	18	-0.7167	-2	0	0	0
HIP_33215	VCGERGFFY	SLGYKEIR	VCGERGFFYSLGYKEIR		CMGA	4999/247050	0.998	33215	0.822	0.169260	1.15	0.3333	1	17	-0.2294	1	4	1	0
HIP_33216	CGERGFFYT	PTVVLNPHP	CGERGFFYTPTVVLNPHP		CMGA	4400/247050	0.999	33216	0.822	-0.036070	0.45	0.1667	1	18	-0.15	0	3	0	0
HIP_33217	HLVEALYLV	FETLRGDER	HLVEALYLVFETLRGDER		CMGA	6946/247050	0.997	33217	0.822	0.079185	0.8833	0	-1	18	-0.0222	-2	2	0	0
HIP_33218	FVNQHLCGS	YNQEKVLPR	FVNQHLCGSYNQEKVLPR		SCG2	17182/334890	0.997	33218	0.822	0.012779	-1.1667	0.3333	2	18	-0.6611	1	1	0	0
HIP_33219	SHLVEALYL	SEEEPEYG	SHLVEALYLSEEEPEYG		SCG1	9045/367830	0.998	33219	0.822	0.173881	-0.25	0.1667	-4	17	-0.5588	-5	1	1	1
HIP_33220	EQCCTSICS	GGGHSRERA	EQCCTSICSGGGHSRERA		SCG1	1475/367830	1.000	33220	0.822	0.175341	0.8333	0.6667	1	18	-0.7333	0	0	0	1
HIP_33221	CCTSICSLY	KENIEKNEQ	CCTSICSLYKENIEKNEQ		SCG2	18915/334890	0.996	33221	0.822	0.018680	-1.5333	0.1667	-1	18	-0.6722	-1	1	0	0
HIP_33222	FVNQHLCGS	PPKNDDTPN	FVNQHLCGSPPKNDDTPN		SCG2	9945/334890	0.999	33222	0.822	-0.044737	-0.9667	0.6667	0	18	-1.1944	-1	0	0	0
HIP_33223	SHLVEALYL	YINLITRQ	SHLVEALYLYINLITRQ		NPY	2654/49410	0.980	33223	0.822	0.099477	1	0	1	17	0.4647	0	2	1	0
HIP_33224	CGERGFFYT	SLPSEEKGH	CGERGFFYTSLPSEEKGH		SCG1	4242/367830	0.999	33224	0.822	0.009559	0.3667	0.3333	0	18	-0.9222	-1	3	0	0
HIP_33225	EQCCTSICS	EESWDEEDK	EQCCTSICSEESWDEEDK		SCG1	5846/367830	0.999	33225	0.822	0.044881	-0.2667	0.3333	-6	18	-1.3278	-6	1	0	0
HIP_33226	TSICSLYQL	LVHSSNNFG	TSICSLYQLLVHSSNNFG		IAPP	1699/45018	0.993	33226	0.822	0.004478	0.6333	0	1	18	0.3389	0	1	0	0
HIP_33227	GERGFFYTP	PSLELDKMA	GERGFFYTPPSLELDKMA		SCG1	3434/367830	1.000	33227	0.822	-0.142852	-0.3667	0.5	-1	18	-0.4889	-1	3	0	0
HIP_33228	RGFFYTPKT	GYKEIRKGE	RGFFYTPKTGYKEIRKGE		CMGA	15817/247050	0.985	33228	0.822	0.000682	-1.9667	0.3333	3	18	-1.3556	3	2	0	0
HIP_33229	HLVEALYL	EEPSSKDV	HLVEALYLEEPSSKDV		CMGA	6677/247050	0.998	33229	0.822	0.183557	-0.3833	0.1667	-2	16	-0.25	-3	1	0	0
HIP_33230	HLVEALYL	EEEAEAGE	HLVEALYLEEEAEAGE		CMGA	6239/247050	0.998	33230	0.822	0.125665	-0.7	0	-5	16	-0.3063	-6	1	0	1
HIP_33231	GERGFFYTP	ALLLCAGQV	GERGFFYTPALLLCAGQV		CMGA	2328/247050	0.999	33231	0.822	0.002631	0.9667	0.1667	0	18	0.6111	0	3	0	1
HIP_33232	SHLVEALYL	ENENTKFE	SHLVEALYLENENTKFE		SCG1	13371/367830	0.996	33232	0.822	0.174265	-0.7	0	-2	17	-0.6294	-3	1	1	0
HIP_33233	LCGSHLVEA	NVQKFPSPE	LCGSHLVEANVQKFPSPE		SCG2	23577/334890	0.994	33233	0.822	0.095666	-0.05	0	0	18	-0.1778	-1	0	0	0
HIP_33234	GERGFFYTP	ELQDLALQG	GERGFFYTPELQDLALQG		CMGA	5287/247050	0.998	33234	0.822	-0.142861	-1.1333	0.1667	-2	18	-0.4444	-2	3	0	0
HIP_33235	NQHLCGSHL	EEEEAEAGE	NQHLCGSHLEEEEAEAGE		CMGA	6734/247050	0.998	33235	0.822	0.000623	-1.7833	0.1667	-4	18	-1.2389	-6	0	0	2
HIP_33236	VEQCCTSIC	EEERGLEPG	VEQCCTSICEEERGLEPG		SCG1	9373/367830	0.998	33236	0.822	0.005449	-0.7167	0.1667	-4	18	-0.5222	-4	0	0	1
HIP_33237	ERGFFYTPK	NFFPEYNYD	ERGFFYTPKNFFPEYNYD		SCG1	7903/367830	0.998	33237	0.822	-0.009487	-0.6833	0.1667	-1	18	-1.2722	-1	4	0	0
HIP_33238	CGERGFFYT	SSSRGEAG	CGERGFFYTSSSRGEAG		SCG1	10716/367830	0.997	33238	0.822	0.190192	-0.2667	0.5	0	17	-0.7118	0	3	1	1
HIP_33239	VCGERGFF	PSEEKGHP	VCGERGFFPSEEKGHP		SCG1	9762/367830	0.998	33239	0.822	0.114191	-0.1167	0.5	0	16	-0.9375	-1	2	0	0
HIP_33240	HLVEALYL	SEEKHLEEP	HLVEALYLSEEKHLEEP		SCG1	9588/367830	0.998	33240	0.822	0.167692	-0.25	0.1667	-2	17	-0.6059	-4	1	1	0
HIP_33241	GPGAGSLQP	PEEEGPTVV	GPGAGSLQPPEEEGPTVV		CMGA	4129/247050	0.999	33241	0.822	-0.045280	-1.65	0.3333	-3	18	-0.5278	-3	0	0	1
HIP_33242	ERGFFYTPK	RSAAVLALL	ERGFFYTPKRSAAVLALL		CMGA	13358/247050	0.990	33242	0.822	-0.047992	-1.6167	0.3333	2	18	0.3	2	2	0	0
HIP_33243	AGSLQPLAL	AELKEAVEE	AGSLQPLALAELKEAVEE		CMGA	6429/247050	0.998	33243	0.822	0.017728	1.9167	0	-3	18	0.1333	-3	0	0	0
HIP_33244	VEALYLVC	TENENTKFE	VEALYLVCTENENTKFE		SCG1	12151/367830	0.997	33244	0.822	0.192917	0.4667	0	-3	17	-0.2647	-3	1	1	0
HIP_33245	TSICSLYQL	GEEEEEEEE	TSICSLYQLGEEEEEEEE		CMGA	6/247050	1.000	33245	0.822	0.050460	-1.4	0.1667	-8	18	-1.1611	-8	1	0	6
HIP_33246	SHLVEALYL	QVSEEVKTR	SHLVEALYLQVSEEVKTR		SCG1	14366/367830	0.995	33246	0.822	0.207752	1.0333	0.1667	0	18	-0.1889	-1	1	0	0
HIP_33247	HLVEALYL	NAFLNERK	HLVEALYLNAFLNERK		SCG1	17929/367830	0.992	33247	0.822	0.088344	1.2333	0	1	16	-0.0688	0	2	0	0
HIP_33248	AEDLQVGQV	QNGERATRL	AEDLQVGQVQNGERATRL		SCG2	4233/334890	1.000	33248	0.822	-0.012712	-1.1833	0.3333	-1	18	-0.8556	-1	0	0	0
HIP_33249	SHLVEALYL	QDTKENRE	SHLVEALYLQDTKENRE		SCG1	16279/367830	0.994	33249	0.822	0.227217	-0.2333	0	-1	17	-1.0588	-2	1	1	0
HIP_33250	HLVEALYL	EEEEAEAGE	HLVEALYLEEEEAEAGE		CMGA	4921/247050	0.998	33250	0.822	0.097909	-0.7	0	-6	17	-0.4941	-7	1	1	2
HIP_33251	AGSLQPLAL	LAKELTAEK	AGSLQPLALLAKELTAEK		CMGA	13692/247050	0.989	33251	0.822	0.068335	1.85	0	0	18	0.2444	0	0	0	0
HIP_33252	ALYLVCGER	DWWEKKPFS	ALYLVCGERDWWEKKPFS		SCG1	9386/367830	0.998	33252	0.822	0.076837	-2.2833	0.1667	0	18	-0.5444	0	2	0	0
HIP_33253	CGERGFFY	QMAYENLN	CGERGFFYQMAYENLN		SCG2	15648/334890	0.998	33253	0.822	0.055571	0.75	0	-1	16	-0.6125	-1	4	0	0
HIP_33254	VEALYLVCG	RPQSEESWD	VEALYLVCGRPQSEESWD		SCG1	12605/367830	0.996	33254	0.822	0.080653	-0.55	0.3333	-3	18	-0.4167	-3	1	0	0
HIP_33255	SICSLYQLE	YPSDSQVSE	SICSLYQLEYPSDSQVSE		SCG1	5090/367830	0.999	33255	0.822	-0.185381	-1.15	0.3333	-3	18	-0.3833	-3	2	0	0
HIP_33256	DLQVGQVEL	SQEKTHSRE	DLQVGQVELSQEKTHSRE		SCG1	20422/367830	0.989	33256	0.822	0.214047	-0.55	0.1667	-1	18	-1.2667	-2	0	0	0
HIP_33257	VEALYLVCG	DSEGLSQGL	VEALYLVCGDSEGLSQGL		CMGA	3699/247050	0.999	33257	0.822	0.051210	-0.25	0.3333	-3	18	0.5444	-3	1	0	0
[illegible]	[illegible]	[illegible]	[illegible]		[illegible]	[illegible]	[illegible]	[illegible]	[illegible]	[illegible]	[illegible]	[illegible]	[illegible]	[illegible]	[illegible]	[illegible]	[illegible]	[illegible]	[illegible]

HIP_33716	SICSLYQLE	QAIKEHLNQ	SICSLYQLEQAIKEHLNQ		SCG2	10046/334890	0.999	33716	0.821	-0.071811	-0.0667	0	0	18	-0.35	-1	1	0	0	0.000000	2	1	-0.05	1.900000	0.2222	1.6778	0	0	0	0	2	-0.1119	2	0	0	1	0	0.4	1.26	0.7217	0.3333	0.16	0.5208	-0.0267	0.0025	-0.1
HIP_33717	RGFFYTPKT	SNNFGAILS	RGFFYTPKTSNNFGAILS		IAPP	2558/45018	0.984	33717	0.821	-0.044359	-2.3333	0.3333	2	18	-0.2	2	2	0	0	0.000000	-0.5333	-1	0	0.700000	0.1111	0.5889	1	0	1	0	0.5333	-1.3741	-1.0667	0	1	0	0	0.5885	0.7603	1.2845	-0.1667	0.3463	1.6499	-1.3731	0	0
HIP_33718	VCGERGFFY	IESQTQEE	VCGERGFFYIESQTQEE		SCG2	5729/334890	0.999	33718	0.821	0.093004	0.75	0.1667	-3	17	-0.7235	-3	3	1	0	0.000000	-1.3667	-1	0.1167	1.825000	0.2222	1.6028	0	0	0	0	1.3667	1.2021	-4.1	0	1	0	0	0.3718	1.1353	0.7528	0.3333	0.1382	0.5667	0.2788	0.0136	-0.1594
HIP_33719	CGERGFFYT	QAIKEHLN	CGERGFFYTQAIKEHLN		SCG2	17517/334890	0.997	33719	0.821	-0.015614	0.6	0	1	17	-0.6	0	3	1	0	0.000000	0.6667	0	0	0.000000	0.1111	-0.1111	0	0	0	0	0	-0.0667	2	0	1	0	0	0.3718	1.1153	0.7628	0.5	0.1382	0.5819	0.2231	0	0
HIP_33720	TSICSLYQL	KKPFSEDVN	TSICSLYQLKKPFSEDVN		SCG1	25598/367830	0.979	33720	0.821	0.158706	-1.7333	0.1667	0	18	-0.3444	0	2	0	0	0.000000	-2.8	2	0.1167	0.700000	0.2222	0.4778	1	0	1	0	-5.6	-0.8281	-5.6	1	0	0	0	0.4654	1.0074	0.9535	-0.1667	0.2166	0.9091	-0.8067	0.0136	-0.3267
[illegible]																																														

HIP_34227	LVCGERGFF	ELSEVLENQ	LVCGERGFFELSEVLENQ		CMGA	2472/247050	0.999	34227	0.821	-0.015157	0.7833	0.3333	-3	18	0.0444	-3	2	0	0	0.000000	-1.9	-1	0.0333	0.700000	0.4444	0.2556	0	1	0	1	1.9	0.2002	-3.8	1	0	0	0	0.4167	1.085	0.8567	0.3333	0.1736	0.7339	0.3264	0.0011	-0.0633
HIP_34228	GSLQPLAL	SAKKEELV	GSLQPLALSAKKEELV		SCG1	13319/367830	0.996	34228	0.821	0.069275	2.4833	0.1667	0	17	0.3294	0	0	1	0	0.000000	-1.3	0	0.0667	1.400000	0.2222	1.1778	0	0	0	0	0	2.9248	0	1	0	0	0	0.2667	1.23	0.665	0.8333	0.0711	0.4422	0.6622	0.0044	-0.0867
HIP_34229	CGERGFFY	DQLAKELTA	CGERGFFYDQLAKELTA		CMGA	13241/247050	0.990	34229	0.821	0.145303	0.1833	0	-1	17	-0.3471	-1	3	1	0	0.000000	-2.5	-1	0.1	0.000000	0.2222	-0.2222	0	0	0	0	2.5	-0.0407	-7.5	0	1	0	0	0.3436	1.1422	0.749	0.3333	0.1181	0.561	0.063	0.01	-0.25
HIP_34230	LVEALYLVC	VSQECFETL	LVEALYLVCVSQECFETL		CMGA	4120/247050	0.999	34230	0.821	0.092307	1.7333	0.1667	-3	18	1.1444	-3	1	0	0	0.000000	-3.5333	0	0.1167	3.050000	0.1111	2.9389	0	0	0	0	0	5.0941	-3.5333	0	0	0	0	0.3718	1.0586	0.7645	0.5	0.1382	0.5844	0.6444	0.0136	-0.4122
HIP_34231	HLVEALYL	NEQINDEMK	HLVEALYLNEQINDEMK		SCG2	11782/334890	0.998	34231	0.821	0.179589	-0.7	0	-2	17	-0.5353	-3	1	1	0	0.000000	-5.6	-1	0.15	1.400000	0.1111	1.2889	0	0	0	0	5.6	-0.9022	-5.6	1	0	0	0	0.4385	1.1519	0.8645	0.1667	0.1922	0.7473	-0.3069	0.0225	-0.84
HIP_34232	CGERGFFY	GEATDGAR	CGERGFFYGEATDGAR		CMGA	7344/247050	0.997	34232	0.821	0.162073	0.3667	0.1667	-1	16	-0.7125	-1	3	0	0	0.000000	-2.1333	-1	0.1333	0.700000	0.2222	0.4778	0	1	0	1	2.1333	0.1752	-6.4	0	1	0	0	0.3885	1.1469	0.8345	0.3333	0.1509	0.6964	0.1424	0.0178	-0.2844
HIP_34233	GSLQPLAL	GAWKELDRN	GSLQPLALGAWKELDRN		SCG1	10532/367830	0.997	34233	0.821	0.121661	1.65	0.1667	0	17	-0.4529	0	1	1	0	0.000000	-2.9667	0	0.0833	1.400000	0.2222	1.1778	0	1	0	1	0	1.9433	-2.9667	1	0	0	0	0.3218	1.1703	0.7878	0.8333	0.1036	0.6207	0.531	0.0069	-0.2472
HIP_34234	QCCTSICSL	SSSRGEAGA	QCCTSICSLSSSRGEAGA		SCG1	7653/367830	0.999	34234	0.821	0.200802	0.5167	0.6667	0	18	0.1333	0	0	0	1	0.000000	-2.6333	0	0.1333	0.000000	0.1111	-0.1111	0	0	0	0	0	-0.0574	0	1	0	0	0	0.5051	0.9203	1.0345	0.1667	0.2552	1.0702	0.261	0.0178	-0.3511
HIP_34235	VCGERGFF	SSPDYNPY	VCGERGFFSSPDYNPY		SCG2	3835/334890	1.000	34235	0.821	0.127478	0.3333	0.6667	-1	16	-0.6813	-1	2	0	0	0.000000	-2.8	0	0.1167	0.700000	0.3333	0.3667	1	1	1	1	0	0.1222	-5.6	1	0	0	0	0.5	0.845	1.1033	0.3333	0.25	1.2173	0.1667	0.0136	-0.3267
HIP_34236	VCGERGFFY	LLQKEPDL	VCGERGFFYLLQKEPDL		SCG2	11039/334890	0.999	34236	0.821	-0.003887	1.4	0	-1	17	-0.1412	-1	3	1	0	0.000000	-0.0667	0	0.05	1.650000	0.2222	1.4278	0	0	0	0	0	1.9989	-0.2	0	1	0	0	0.3051	1.1886	0.6812	0.6667	0.0931	0.464	0.4272	0.0025	-0.0033
HIP_34237	LYQLENYCN	APVSKRFPV	LYQLENYCNAPVSKRFPV		SCG2	9600/334890	0.999	34237	0.821	-0.095775	0.35	0.1667	1	18	-0.3278	1	1	0	0	0.000000	2.2333	0	0.0167	1.075000	0.1111	0.9639	1	0	1	0	0	0.3374	2.2333	0	1	0	0	0.4269	0.9655	1.009	0.3333	0.1823	1.018	0.1494	0.0003	0.0372
HIP_34238	ERGFFYTPK	ILRHQNLLK	ERGFFYTPKILRHQNLLK		CMGA	9673/247050	0.995	34238	0.821	0.048787	-0.4	0.1667	4	18	-0.7222	3	2	0	0	0.000000	3.3333	0	-0.1167	1.825000	0.2222	1.6028	1	0	1	0	0	-0.6411	6.6667	0	0	1	0	0.4269	0.9705	0.8773	0	0.1823	0.7697	-0.1708	0.0136	-0.3889
HIP_34239	CGERGFFYT	LNQEKAEK	CGERGFFYTLNQEKAEK		SCG2	19999/334890	0.996	34239	0.821	0.049630	-0.4	0	0	17	-1.1118	0	3	1	0	0.000000	-1.3333	0	0.05	0.950000	0.1111	0.8389	0	0	0	0	0	-0.3356	-4	0	1	0	0	0.4385	1.0219	0.9178	0.3333	0.1922	0.8424	-0.1754	0.0025	-0.0667
HIP_34240	RGFFYTPKT	NPHPSLGYK	RGFFYTPKTNPHPSLGYK		CMGA	16875/247050	0.982	34240	0.821	0.015291	-2.4167	0.3333	4	18	-1.1111	3	1	0	0	0.000000	-0.7	-1	-0.05	0.000000	0.1111	-0.1111	2	0	1	0	0.7	0.2685	-0.7	0	1	0	0	0.5769	0.7805	1.229	-0.3333	0.3328	1.5104	-1.3942	0.0025	0.035
HIP_34241	VEALYLVC	ENQMDKARR	VEALYLVCENQMDKARR		SCG1	18291/367830	0.992	34241	0.821	0.213086	0	0	0	17	-0.4529	0	1	1	0	0.000000	-7	-1	0.15	0.625000	0.2222	0.4028	0	0	0	0	7	0	-7	0	0	0	0	0.4385	1.1119	0.8645	0.1667	0.1922	0.7473	0	0.0225	-1.05
HIP_34242	HLVEALYL	NSPMNKGDT	HLVEALYLNSPMNKGDT		CMGA	6733/247050	0.998	34242	0.821	0.218971	0.0667	0.3333	0	17	-0.3882	-1	1	1	0	0.000000	-4.0667	0	0.1833	1.400000	0	1.4	1	0	1	0	0	0.0933	-4.0667	1	0	0	0	0.4551	0.9436	1.0312	0.3333	0.2071	1.0633	0.0303	0.0336	-0.7456
HIP_34243	GSLQPLALE	LANFLVHSS	GSLQPLALELANFLVHSS		IAPP	1656/45018	0.993	34243	0.821	-0.060954	0.7	0	0	18	0.6389	-1	1	0	0	0.000000	0	1	0	2.600000	0.1111	2.4889	0	0	0	0	0	1.7422	0	0	0	1	0	0.3333	1.28	0.77	0.5	0.1111	0.5929	0.2333	0	0
HIP_34244	CSLYQLENY	EARFQDKQY	CSLYQLENYEARFQDKQY		SCG1	29274/367830	0.968	34244	0.821	-0.096167	-2.4167	0	-1	18	-1.2722	-1	2	0	0	0.000000	0.7	1	0	0.700000	0.3333	0.3667	0	0	0	0	0.7	-0.8861	1.4	0	1	0	0	0.4769	1.1072	0.919	-0.3333	0.2275	0.8445	-1.1526	0	0
HIP_34245	LYQLENYCN	ATQRLANFL	LYQLENYCNATQRLANFL		IAPP	2438/45018	0.986	34245	0.821	-0.049731	-0.7833	0	0	18	-0.2611	0	1	0	0	0.000000	-0.0333	0	0.05	1.075000	0.2222	0.8528	0	0	0	0	0	-0.668	-0.0333	0	1	0	0	0.4769	1.0172	0.9956	0.1667	0.2275	0.9913	-0.3736	0.0025	-0.0017
HIP_34246	VCGERGFFY	IAKRAMEN	VCGERGFFYIAKRAMEN		SCG2	19964/334890	0.996	34246	0.821	0.051976	1.1167	0	1	17	-0.1882	1	3	1	0	0.000000	-0.6333	1	0.05	1.825000	0.3333	1.4917	0	0	0	0	-0.6333	1.6657	-1.9	0	1	0	0	0.2769	1.1622	0.6906	0.5	0.0767	0.477	0.3092	0.0025	-0.0317
[illegible]																																														

HIP_34738	LVEALYLVC	ISGAEAASF	LVEALYLVCISGAEAASF		SCG2	3068/334890	1.000	34738	0.820	0.096369	2.3	0.3333	-2	18	1.4722	-2	1	0	0
HIP_34739	VCGERGFF	GAGRSRSNQ	VCGERGFFGAGRSRSNQ		SCG2	5442/334890	0.999	34739	0.820	0.087986	1.0333	0.5	2	17	-0.7706	2	2	1	0
HIP_34740	HLVEALYLV	WDEDLLMK	HLVEALYLVWDEDLLMK		SCG2	13137/334890	0.998	34740	0.820	0.098729	-0.2	0	-2	17	0.4588	-3	2	1	0
HIP_34741	CGERGFFY	KTSRKDVK	CGERGFFYKTSRKDVK		SCG1	24945/367830	0.980	34741	0.820	0.224843	-0.1833	0.1667	3	16	-1.1875	3	3	0	0
HIP_34742	VEALYLVC	NTKFEVRLL	VEALYLVCNTKFEVRLL		SCG1	23706/367830	0.983	34742	0.820	0.215071	0.4	0	0	17	0.8235	0	2	1	0
HIP_34743	VCGERGFFY	FQELGKLT	VCGERGFFYFQELGKLT		SCG2	17145/334890	0.997	34743	0.820	0.100200	0.0167	0	0	17	0.0353	0	4	1	0
HIP_34744	GSHLVEALY	SQGGSLPSE	GSHLVEALYSQGGSLPSE		SCG1	6126/367830	0.999	34744	0.820	0.189712	-0.0667	0.3333	-1	18	-0.2	-2	1	0	0
HIP_34745	LVEALYLVC	GEEAVPEEE	LVEALYLVCGEEAVPEEE		CMGA	2415/247050	0.999	34745	0.820	0.134321	0.5167	0.1667	-6	18	0.3222	-6	1	0	0
HIP_34746	GSLQPLALE	EKPNGSVEP	GSLQPLALEEKPNGSVEP		SCG2	19078/334890	0.996	34746	0.820	0.151592	-1.15	0.1667	-2	18	-0.6222	-2	0	0	0
HIP_34747	RGFFYTPKT	VPRTRLEDP	RGFFYTPKTVPRTRLEDP		NPY	2586/49410	0.981	34747	0.820	-0.048771	-1.35	0.3333	2	18	-1.0778	2	1	0	0
HIP_34748	GSHLVEALY	GIQEEDLRK	GSHLVEALYGIQEEDLRK		SCG2	714/334890	1.000	34748	0.820	0.119959	0.8167	0.1667	-1	18	-0.5611	-2	1	0	0
HIP_34749	SHLVEALYL	RRPEDQELE	SHLVEALYLRRPEDQELE		CMGA	14777/247050	0.987	34749	0.820	0.204852	-0.7167	0.1667	-2	18	-0.8722	-3	1	0	0
HIP_34750	CSLYQLENY	SLGYKEIRK	CSLYQLENYSLGYKEIRK		CMGA	7887/247050	0.997	34750	0.820	-0.091485	-0.95	0.3333	1	18	-0.7667	1	2	0	0
HIP_34751	QHLCGSHLV	EEEEATNTH	QHLCGSHLVEEEEATNTH		CMGA	7744/247050	0.997	34751	0.820	0.121847	-0.95	0	-1	18	-0.95	-4	0	0	2
HIP_34752	GPGAGSLQP	NQEKVLPRL	GPGAGSLQPNQEKVLPRL		SCG2	21862/334890	0.995	34752	0.820	0.024373	-1.9667	0.1667	1	18	-0.6556	1	0	0	0
HIP_34753	CGERGFFY	NTYGKRNAV	CGERGFFYNTYGKRNAV		IAPP	3127/65018	0.976	34753	0.820	0.158351	-0.2	0	2	17	-0.8118	2	4	1	0
HIP_34754	PGAGSLQPL	EEYRAPRPQ	PGAGSLQPLEEYRAPRPQ		SCG1	23037/367830	0.984	34754	0.820	0.001825	-1.6	0.1667	0	18	-1.1722	0	1	0	0
HIP_34755	QCCTSICSL	EYNYDWWE	QCCTSICSLEYNYDWWE		SCG1	17590/367830	0.992	34755	0.820	0.050145	-0.4667	0.1667	-3	17	-0.4941	-3	2	1	0
HIP_34756	GSHLVEALY	LPVNSPMNK	GSHLVEALYLPVNSPMNK		CMGA	6486/247050	0.998	34756	0.820	-0.013768	1.7833	0.1667	1	18	-0.0333	0	1	0	0
HIP_34757	QHLCGSHLV	EENELTLNE	QHLCGSHLVEENELTLNE		SCG1	10308/367830	0.998	34757	0.820	0.140343	-0.95	0	-2	18	-0.6056	-4	0	0	0
HIP_34758	HLVEALYL	KLDLKRQY	HLVEALYLKLDLKRQY		SCG1	28942/367830	0.969	34758	0.820	0.222156	0.45	0	2	16	-0.225	1	1	0	0
HIP_34759	LVCGERGFF	MLVKYPEII	LVCGERGFFMLVKYPEII		SCG2	2439/334890	1.000	34759	0.820	0.023566	2.5167	0.1667	0	18	0.8833	0	2	0	0
HIP_34760	QCCTSICSL	QKFPSPEMI	QCCTSICSLQKFPSPEMI		SCG2	26622/334890	0.993	34760	0.820	0.105984	0.15	0.1667	0	18	0.2389	0	1	0	0
HIP_34761	GSHLVEALY	LQEEEQIEQ	GSHLVEALYLQEEEQIEQ		SCG2	4498/334890	1.000	34761	0.820	0.018149	0.1833	0	-4	18	-0.6556	-5	1	0	1
HIP_34762	TSICSLYQL	DQLSDDVSK	TSICSLYQLDQLSDDVSK		SCG2	15427/334890	0.998	34762	0.820	0.024677	-0.7	0	-2	18	-0.2222	-2	1	0	0
HIP_34763	CGERGFFY	KPNGSVEP	CGERGFFYKPNGSVEP		SCG2	18060/334890	0.997	34763	0.820	0.203193	-0.7833	0.1667	0	16	-0.8187	0	3	0	0
HIP_34764	QHLCGSHLV	VPKLDLKRQ	QHLCGSHLVVPKLDLKRQ		SCG1	19381/367830	0.990	34764	0.820	0.129243	0.5833	0.1667	4	18	-0.3278	2	0	0	0
HIP_34765	HLCGSHLVE	AYGFRGPGP	HLCGSHLVEAYGFRGPGP		CMGA	11254/247050	0.993	34765	0.820	0.008381	0.7667	0.1667	2	18	-0.1333	0	2	0	0
HIP_34766	CGERGFFY	SANRRPED	CGERGFFYSANRRPED		CMGA	12062/247050	0.992	34766	0.820	0.163733	0.3	0.1667	0	16	-1.3813	0	3	0	0
HIP_34767	LYQLENYCN	RWSKMDQLA	LYQLENYCNRWSKMDQLA		CMGA	17252/247050	0.981	34767	0.820	0.070710	-1.4167	0.1667	0	18	-0.8944	0	2	0	0
HIP_34768	CGERGFFY	GPTVVLNPH	CGERGFFYGPTVVLNPH		CMGA	6417/247050	0.998	34768	0.820	0.197747	0.2667	0.3333	1	17	-0.0471	0	3	1	0
HIP_34769	VCGERGFFY	RSGQLGIQ	VCGERGFFYRSGQLGIQ		SCG2	16251/334890	0.997	34769	0.820	0.218702	-0.2333	0.3333	1	17	-0.1529	1	3	1	0
HIP_34770	GGGPGAGSL	EEEEEEATN	GGGPGAGSLEEEEEEATN		CMGA	3861/247050	0.999	34770	0.820	-0.065094	-1.3167	0.3333	-6	18	-1.2333	-6	0	0	5
HIP_34771	SLYQLENYC	ETTENENTK	SLYQLENYCETTENENTK		SCG1	11025/367830	0.997	34771	0.820	0.034165	-1.2	0	-3	18	-1.5167	-3	1	0	0
HIP_34772	PGAGSLQPL	GEQEHSQQK	PGAGSLQPLGEQEHSQQK		CMGA	6070/247050	0.998	34772	0.820	0.017074	-1.45	0.3333	0	18	-1.3722	-1	0	0	0
HIP_34773	SLQPLAL	FLGEGHH	SLQPLALFLGEGHH		SCG1	6708/367830	0.999	34773	0.820	0.080332	2.6	0.1667	1	14	0.2286	-1	1	0	0
HIP_34774	GERGFFYT	GPPKNDDTP	GERGFFYTGPPKNDDTP		SCG2	3947/334890	1.000	34774	0.820	0.142938	-0.4667	0.5	-1	17	-1.5	-1	3	1	0
HIP_34775	LVCGERGFF	GELFNPYYD	LVCGERGFFGELFNPYYD		SCG1	3921/367830	0.999	34775	0.820	0.004668	0.85	0.3333	-2	18	-0.0667	-2	3	0	0
HIP_34776	TSICSLYQL	TQNAFLNER	TSICSLYQLTQNAFLNER		SCG1	20970/367830	0.988	34776	0.820	0.171462	-1.45	0	0	18	-0.1833	0	1	0	0
HIP_34777	SLYQLENYC	RWSKMDQLA	SLYQLENYCRWSKMDQLA		CMGA	17327/247050	0.981	34777	0.820	0.061913	-1.4167	0.1667	0	18	-0.7444	0	2	0	0
HIP_34778	SLYQLENYC	MQFPPMYEE	SLYQLENYCMQFPPMYEE		SCG2	3453/334890	1.000	34778	0.820	-0.175042	-0.1833	0	-3	18	-0.6778	-3	2	0	0
HIP_34779	VCGERGFFY	FLGEGHHR	VCGERGFFYFLGEGHHR		SCG1	8283/367830	0.998	34779	0.820	0.011081	1.75	0.1667	2	17	-0.3765	0	4	1	0
HIP_34780	ICSLYQLEN	EEENELTLN	ICSLYQLENEEENELTLN		SCG1	17197/367830	0.993	34780	0.820	-0.010388	-2.2833	0	-5	18	-0.6722	-5	1	0	1
HIP_34781	CGERGFFYT	PPITPECRQ	CGERGFFYTPPITPECRQ		SCG1	4972/367830	0.999	34781	0.820	0.010899	0.35	0.3333	0	18	-0.7056	0	3	0	0
HIP_34782	GSHLVEALY	SEEERGLE	GSHLVEALYSEEERGLE		SCG1	8760/367830	0.998	34782	0.820	0.150107	-0.5833	0.1667	-3	17	-0.6765	-4	1	1	1
HIP_34783	GSHLVEALY	ESEEERGL	GSHLVEALYESEEERGL		SCG1	8761/367830	0.998	34783	0.820	0.150107	-0.5833	0.1667	-3	17	-0.6765	-4	1	1	1
HIP_34784	VCGERGFF	EKSSQESGE	VCGERGFFEKSSQESGE		SCG1	17874/367830	0.992	34784	0.820	0.109326	-0.5	0.3333	-2	17	-1.0118	-2	2	1	0
HIP_34785	HLVEALYL	NTATCATQ	HLVEALYLNTATCATQ		IAPP	2079/65018	0.990	34785	0.820	0.205866	0.65	0	0	16	0.4	-1	1	0	0
HIP_34786	SHLVEALYL	LSKEWEDSK	SHLVEALYLLSKEWEDSK		CMGA	10585/247050	0.994	34786	0.820	0.182464	0.9	0.1667	-1	18	-0.4667	-2	1	0	0
HIP_34787	GGGPGAGSL	NRQYWDEDL	GGGPGAGSLNRQYWDEDL		SCG2	18311/334890	0.997	34787	0.820	0.008193	-1.4833	0.3333	-2	18	-1.0667	-2	1	0	1
HIP_34788	CGSHLVEAL	SEEDWMRII	CGSHLVEALSEEDWMRII		SCG2	11738/334890	0.998	34788	0.820	0.115481	-0.95	0.1667	-2	18	0.1333	-3	0	0	0
HIP_34789	CCTSICSLY	FLNERKQAS	CCTSICSLYFLNERKQAS		SCG1	11779/367830	0.997	34789	0.820	-0.065027	0.8	0.1667	1	18	0.05	1	2	0	0
HIP_34790	SLYQLENYC	EEDLRKESK	SLYQLENYCEEDLRKESK		SCG2	16116/334890	0.997	34790	0.820	0.031373	-2.1333	0	-2	18	-1.5056	-2	1	0	0
HIP_34791	AGSLQPLAL	PSDSQVSEE	AGSLQPLALPSDSQVSEE		SCG1	5875/367830	0.999	34791	0.820	0.195733	0.5833	0.3333	-3	18	-0.2833	-3	0	0	0
HIP_34792	LVEALYLV	LANFLVHSS	LVEALYLVLANFLVHSS		IAPP	1476/65018	0.994	34792	0.820	0.014888	1.4667	0	0	17	1.4647	-1	2	1	0
HIP_34793	ERGFFYTPK	LRHQNLLKE	ERGFFYTPKLRHQNLLKE		CMGA	13133/247050	0.990	34793	0.820	0.055800	-1.6833	0.1667	3	18	-1.1667	2	2	0	0

HIP_35249	LVCGERGFF	LAEAYPSKP	LVCGERGFFLAEAYPSKP		NPY	1232/49410	0.993	35249	0.820	-0.016418
HIP_35250	SHLVEALYL	QNAFLNER	SHLVEALYLQNAFLNER		SCG1	20626/367830	0.989	35250	0.820	0.163077
HIP_35251	SLYQLENYC	EEKIESQTQ	SLYQLENYCEEKIESQTQ		SCG2	18011/334890	0.997	35251	0.820	0.030397
HIP_35252	GSHLVEALY	QALPEPMQE	GSHLVEALYQALPEPMQE		CMGA	6103/247050	0.998	35252	0.820	-0.030271
HIP_35253	AGSLQPLAL	CRQVLKTSR	AGSLQPLALCRQVLKTSR		SCG1	29468/367830	0.967	35253	0.820	0.174290
HIP_35254	LVEALYLVC	MSDDYETQQ	LVEALYLVCMSDDYETQQ		SCG2	4895/334890	1.000	35254	0.820	0.102430
HIP_35255	GERGFFYT	PLQWKSSHF	GERGFFYTPLQWKSSHF		SCG1	17349/367830	0.993	35255	0.820	0.028831
HIP_35256	LYQLENYCN	HSEKSQRED	LYQLENYCNHSEKSQRED		SCG1	22316/367830	0.986	35256	0.820	0.022238
HIP_35257	SLQPLAL	VDKRRTRPR	SLQPLALVDKRRTRPR		SCG1	34413/367830	0.947	35257	0.820	0.223896
HIP_35258	GSLQPLAL	LQGAKERAH	GSLQPLALLQGAKERAH		CMGA	8706/247050	0.996	35258	0.820	0.212184
HIP_35259	LYQLENYC	ENYQKGERG	LYQLENYCENYQKGERG		SCG1	25455/367830	0.979	35259	0.820	0.042553
HIP_35260	AGSLQPLAL	DRSSQGGSL	AGSLQPLALDRSSQGGSL		SCG1	18815/367830	0.991	35260	0.820	0.248121
HIP_35261	GERGFFYT	SDSQVSEEV	GERGFFYTSDSQVSEEV		SCG1	8410/367830	0.998	35261	0.820	0.101384
HIP_35262	AGSLQPLAL	EEERLSKE	AGSLQPLALEEERLSKE		CMGA	9090/247050	0.996	35262	0.820	0.150045
HIP_35263	CCTSICSLY	DPEGKGEQE	CCTSICSLYDPEGKGEQE		CMGA	4779/247050	0.998	35263	0.820	0.019303
HIP_35264	LVCGERGFF	LTLALSLLV	LVCGERGFFLTLALSLLV		NPY	674/49410	0.997	35264	0.820	-0.029343
HIP_35265	LVEALYLVC	GEEIKGYP	LVEALYLVCGEEIKGYP		SCG1	7968/367830	0.998	35265	0.820	0.157911
HIP_35266	CTSICSLYQ	AVLALLLCA	CTSICSLYQAVLALLLCA		CMGA	5962/247050	0.998	35266	0.820	-0.014806
HIP_35267	LCGSHLVEA	QEESEESNV	LCGSHLVEAQEESEESNV		SCG1	13772/367830	0.996	35267	0.820	0.096852
HIP_35268	RGFFYTPKT	ANFLVHSSN	RGFFYTPKTANFLVHSSN		IAPP	1531/45018	0.994	35268	0.820	0.049618
HIP_35269	GSLQPLAL	SETHAAGHS	GSLQPLALSETHAAGHS		SCG1	11975/367830	0.997	35269	0.820	0.214294
HIP_35270	LVEALYLVC	ATLESVFQE	LVEALYLVCATLESVFQE		SCG2	8488/334890	0.999	35270	0.820	0.007972
HIP_35271	SHLVEALY	PSPEMIRAL	SHLVEALYPSPEMIRAL		SCG2	7794/334890	0.999	35271	0.820	0.216994
HIP_35272	CGERGFFYT	DPEGKGEQ	CGERGFFYTDPEGKGEQ		CMGA	10851/247050	0.994	35272	0.820	0.119055
HIP_35273	NQHLCGSHL	PGKGRHHRG	NQHLCGSHLPGKGRHHRG		SCG1	22638/367830	0.985	35273	0.820	0.166339
HIP_35274	QCCTSICSL	EAYPSKPDN	QCCTSICSLEAYPSKPDN		NPY	2495/49410	0.982	35274	0.820	0.019462
HIP_35275	SLYQLENYC	RPEDQELES	SLYQLENYCRPEDQELES		CMGA	7797/247050	0.997	35275	0.820	0.076795
HIP_35276	SHLVEALYL	LGAALSLIP	SHLVEALYLLGAALSLIP		SCG2	3794/334890	1.000	35276	0.820	0.045585
HIP_35277	GSLQPLALE	REEKRFLGE	GSLQPLALEREEKRFLGE		SCG1	31703/367830	0.959	35277	0.820	0.163202
HIP_35278	CCTSICSLY	AWIPHVENR	CCTSICSLYAWIPHVENR		SCG2	6590/334890	0.999	35278	0.820	-0.026085
HIP_35279	VEALYLVC	EGQEEEEDN	VEALYLVCEGQEEEEDN		CMGA	3549/247050	0.999	35279	0.820	0.167723
HIP_35280	LVCGERGFF	LSAEPGWQA	LVCGERGFFLSAEPGWQA		CMGA	1159/247050	1.000	35280	0.820	-0.051156
HIP_35281	VCGERGFFY	GLAAVNSM	VCGERGFFYGLAAVNSM		SCG1	5213/367830	0.999	35281	0.820	0.026645
HIP_35282	CGERGFFYT	NHNEGMVT	CGERGFFYTNHNEGMVT		SCG1	24903/367830	0.980	35282	0.820	0.086137
HIP_35283	CTSICSLYQ	QMAYENLN	CTSICSLYQQMAYENLN		SCG2	14123/334890	0.998	35283	0.820	-0.060787
HIP_35284	VEQCCTSIC	SNTYGKRNA	VEQCCTSICSNTYGKRNA		IAPP	1992/45018	0.991	35284	0.820	0.148235
HIP_35285	TSICSLYQ	PEYGEEIKG	TSICSLYQPEYGEEIKG		SCG1	15338/367830	0.995	35285	0.820	0.057406
HIP_35286	SLYQLENYC	RPDRSSQGG	SLYQLENYCRPDRSSQGG		SCG1	22384/367830	0.986	35286	0.820	0.151879
HIP_35287	GIVEQCCTS	ENTKFEVRL	GIVEQCCTSENTKFEVRL		SCG1	18024/367830	0.992	35287	0.820	-0.015445
HIP_35288	RGFFYTPKT	KREEEEEEE	RGFFYTPKTKREEEEEEE		CMGA	14381/247050	0.988	35288	0.820	-0.024955
HIP_35289	VCGERGFFY	DLLMKVLE	VCGERGFFYDLLMKVLE		SCG2	12867/334890	0.998	35289	0.820	0.011128
HIP_35290	VCGERGFF	DNPGEDAPA	VCGERGFFDNPGEDAPA		NPY	1307/49410	0.993	35290	0.820	0.071578
HIP_35291	AGSLQPLAL	CGSHLVEAL	AGSLQPLALCGSHLVEAL		INSB	48/4968	0.997	35291	0.820	0.184276
HIP_35292	GSLQPLAL	ADEPQWSL	GSLQPLALADEPQWSL		SCG1	10723/367830	0.997	35292	0.820	0.098603
HIP_35293	LVEALYLVC	EERLSKEW	LVEALYLVCEERLSKEW		CMGA	10949/247050	0.994	35293	0.820	0.162479
HIP_35294	HLVEALYLV	PGQGSSED	HLVEALYLVPGQGSSED		SCG2	4885/334890	1.000	35294	0.820	0.196123
HIP_35295	HLVEALYLV	EGQEEEED	HLVEALYLVEGQEEEED		CMGA	2041/247050	0.999	35295	0.820	0.156821
HIP_35296	GERGFFYTP	DLFQNRMLS	GERGFFYTPDLFQNRMLS		SCG2	11096/334890	0.999	35296	0.820	-0.053413
HIP_35297	PGAGSLQPL	YSSHHTAEK	PGAGSLQPLYSSHHTAEK		SCG1	15652/367830	0.994	35297	0.820	-0.027028
HIP_35298	GGGPGAGS	ERGFFYTP	GGGPGAGSERGFFYTP		INSB	194/4968	0.976	35298	0.820	-0.001036
HIP_35299	SHLVEALY	QGSSEDDLQ	SHLVEALYQGSSEDDLQ		SCG2	5945/334890	0.999	35299	0.820	0.206899
HIP_35300	HLVEALYL	YFPNPYNQ	HLVEALYLYFPNPYNQ		SCG2	14904/334890	0.998	35300	0.820	0.097378
HIP_35301	CGSHLVEAL	REEARFQDK	CGSHLVEALREEARFQDK		SCG1	29621/367830	0.967	35301	0.820	0.122318
HIP_35302	CCTSICSLY	RDSSMKLSF	CCTSICSLYRDSSMKLSF		CMGA	15865/247050	0.985	35302	0.820	0.124315
HIP_35303	VEALYLVC	MYEENSRDN	VEALYLVCMYEENSRDN		SCG2	15893/334890	0.997	35303	0.820	0.071681
HIP_35304	FVNQHLCGS	NSRDNPFKR	FVNQHLCGSNSRDNPFKR		SCG2	25099/334890	0.994	35304	0.820	0.025209
HIP_35305	LYQLENYCN	RPEDQELES	LYQLENYCNRPEDQELES		CMGA	7798/247050	0.997	35305	0.820	0.083876

[illegible]

HIP_35760	CGERGFFYT	FPDFYDSE	CGERGFFYTFPDFYDSE		SCG1	9478/367830	0.998	35760	0.819	-0.010631	-0.25	0.1667	-3	17	-0.6647	-3	5	1	0	0.000000	-1.0333	-1	0.0667	1.400000	0.1111	1.2889	1	0	1	0	1.0333	-0.3222	-5.1667	0	1	0	0	0.4269	0.9738	0.9156	0.1667	0.1823	0.8384	-0.1067	0.0044	-0.0689
HIP_35761	QCCTSICSL	FRGPGPQLR	QCCTSICSLFRGPGPQLR		CMGA	14770/247050	0.987	35761	0.819	0.129295	0.5667	0.3333	2	18	0.0056	2	1	0	0	1.000000	-2.5333	1	0.0667	0.700000	0.1111	0.5889	0	1	0	1	-2.5333	0.3337	-2.5333	1	0	0	0	0.4269	0.9738	0.9356	0.1667	0.1823	0.8754	0.2419	0.0044	-0.1689
HIP_35762	PLALEGSLQ	EEGENYQKG	PLALEGSLQEEGENYQKG		SCG1	5799/367830	0.999	35762	0.819	-0.055414	-1.3167	0.3333	-3	18	-1.1167	-3	0	0	0	0.000000	-2.3	-2	0.0667	0.950000	0.4444	0.5056	0	1	0	1	4.6	-0.6656	0	0	1	0	0	0.55	1.1217	0.9433	-0.1667	0.3025	0.8899	-0.7242	0.0044	-0.1533
HIP_35763	RGFFYTPKT	YLARMLVKY	RGFFYTPKTYLARMLVKY		SCG2	13752/334890	0.998	35763	0.819	0.147071	-0.3167	0.1667	4	18	-0.1667	4	2	0	0	0.000000	3.5	-1	-0.1667	0.000000	0.2222	-0.2222	1	0	1	0	-3.5	0.0704	7	0	1	0	0	0.4269	1.0322	0.909	0.1667	0.1823	0.8262	-0.1352	0.0278	-0.5833
HIP_35764	LYQLENYCN	ENRQMAYEN	LYQLENYCNENRQMAYEN		SCG2	21180/334890	0.996	35764	0.819	0.120181	-2.3	0	-2	18	-1.45	-2	1	0	0	0.000000	-3.0667	0	0.1	0.625000	0.3333	0.2917	0	0	0	0	0	-0.6708	-3.0667	0	1	0	0	0.5154	0.9858	1.0968	-0.3333	0.2656	1.203	-1.1854	0.01	-0.3067
HIP_35765	GSHLVEALY	GEEEEEEEE	GSHLVEALYGEEEEEEEE		CMGA	409/247050	1.000	35765	0.819	0.099110	-0.5167	0.1667	-8	18	-1.3333	-9	1	0	6	0.000000	-3.9	-2	0.1833	2.000000	0.4444	1.5556	0	1	0	1	7.8	-0.8037	-3.9	0	1	0	0	0.4551	1.2153	0.8412	0	0.2071	0.7075	-0.2351	0.0136	-0.715
HIP_35766	VCGERGFF	AEAKTHWLG	VCGERGFFAEAKTHWLG		SCG2	13912/334890	0.998	35766	0.819	0.035296	0.8833	0.1667	1	17	-0.1	0	2	1	0	1.000000	-1.7	-1	-0.0333	0.700000	0.3333	0.3667	0	1	0	1	1.7	0.3239	-3.4	1	0	0	0	0.35	1.2183	0.7932	0.5	0.1225	0.6294	0.3092	0.0011	0.0567
HIP_35767	HLVEALYLV	SEEERGLE	HLVEALYLVSEEERGLE		SCG1	9266/367830	0.998	35767	0.819	0.163914	-0.1833	0.1667	-3	17	-0.1353	-4	1	1	1	0.000000	-4.8333	-2	0.1667	1.400000	0.3333	1.0667	0	0	0	0	9.6667	-0.1956	-4.8333	1	0	0	0	0.4385	1.2003	0.7645	0	0.1922	0.5844	-0.0804	0.0278	-0.8056
HIP_35768	HLVEALYLV	ESEEERGL	HLVEALYLVESEEERGL		SCG1	9267/367830	0.998	35768	0.819	0.163914	-0.1833	0.1667	-3	17	-0.1353	-4	1	1	1	0.000000	-4.8333	-2	0.1667	1.400000	0.3333	1.0667	0	0	0	0	9.6667	-0.1956	-4.8333	1	0	0	0	0.4385	1.2003	0.7645	0	0.1922	0.5844	-0.0804	0.0278	-0.8056
HIP_35769	GPGAGSLQP	RFFVGPPKN	GPGAGSLQPRFFVGPPKN		SCG2	20131/334890	0.996	35769	0.819	-0.041948	-0.7667	0.3333	2	18	-0.7333	2	1	0	0	0.000000	-0.6667	1	0	1.050000	0.1111	0.9389	2	0	1	0	-0.6667	-0.7198	-0.6667	0	0	0	1	0.4718	0.9619	1.0045	0.1667	0.2226	1.009	-0.3617	0	0
HIP_35770	EQCCTSICS	PEEEGPTVV	EQCCTSICSPEEEGPTVV		CMGA	2753/247050	0.999	35770	0.819	0.040138	-0.4	0.3333	-4	18	-0.2056	-4	0	0	1	0.000000	-4.9333	-2	0.1	0.625000	0.3333	0.2917	1	0	1	0	9.8667	-0.1167	0	0	1	0	0	0.5218	1.0819	0.9295	-0.1667	0.2723	0.8639	-0.2087	0.01	-0.4933
HIP_35771	SLYQLENYC	LITRQRYGK	SLYQLENYCLITRQRYGK		NPY	2489/49410	0.982	35771	0.819	-0.056301	0.8833	0	2	18	-0.7944	2	1	0	0	0.000000	3.3	0	0	1.900000	0.2222	1.6778	0	0	0	0	0	1.482	3.3	0	0	0	0	0.4103	0.9872	0.884	0.3333	0.1683	0.7814	0.3624	0	0
HIP_35772	CTSICSLYQ	VVLNPHPSL	CTSICSLYQVVLNPHPSL		CMGA	7026/247050	0.997	35772	0.819	-0.012943	1.8667	0	1	18	0.6389	0	1	0	0	0.000000	4.4	0	-0.05	1.675000	0	1.675	0	0	0	0	0	3.1267	4.4	0	1	0	0	0.3051	1.1419	0.6478	0.6667	0.0931	0.4197	0.5696	0.0025	-0.22
HIP_35773	LVCGERGFF	NSPMNKGD	LVCGERGFFNSPMNKGD		CMGA	7360/247050	0.997	35773	0.819	0.112087	-0.1167	0.5	0	17	-0.4706	0	2	1	0	0.000000	-3.7	0	0.1	1.175000	0.2222	0.9528	1	1	1	1	0	-0.1112	-7.4	1	0	0	0	0.5	0.8233	1.1633	0.3333	0.25	1.3533	-0.0583	0.01	-0.37
HIP_35774	LYQLENYCN	EEKIESQTQ	LYQLENYCNEEKIESQTQ		SCG2	18065/334890	0.997	35774	0.819	0.038855	-2.2	0	-3	18	-1.3833	-3	1	0	0	0.000000	-2.8667	-1	0.1	1.750000	0.4444	1.3056	0	0	0	0	2.8667	-2.8722	-2.8667	0	1	0	0	0.5154	1.1258	0.9601	-0.5	0.2656	0.9218	-1.1338	0.01	-0.2867
HIP_35775	ERGFFYTPK	EKVAHQLQA	ERGFFYTPKEKVAHQLQA		CMGA	16643/247050	0.983	35775	0.819	-0.022412	-1.5667	0.1667	2	18	-0.9056	1	2	0	0	0.000000	1	-1	-0.0667	1.150000	0.3333	0.8167	1	0	1	0	-1	-1.2794	2	0	0	1	0	0.4936	1.0022	0.9223	-0.3333	0.2436	0.8507	-0.7733	0.0044	-0.0667
HIP_35776	VEQCCTSIC	EETGSQENH	VEQCCTSICEETGSQENH		SCG1	7743/367830	0.998	35776	0.819	0.004645	-0.25	0.1667	-3	18	-0.8278	-4	0	0	0	0.000000	-4.6333	-2	0.0833	1.750000	0.2222	1.5278	0	0	0	0	9.2667	-0.3819	0	0	0	0	0	0.5051	1.1253	0.8362	-0.1667	0.2552	0.6992	-0.1263	0.0069	-0.3861
HIP_35777	NQHLCGSHL	NQEKAEKGR	NQHLCGSHLNQEKAEKGR		SCG2	27152/334890	0.992	35777	0.819	0.131622	-1.7833	0.1667	3	18	-1.6333	1	0	0	0	0.000000	-3.4333	-1	0.0833	0.625000	0.2222	0.4028	0	0	0	0	3.4333	-0.7183	0	1	0	0	0	0.5051	1.0686	0.9812	-0.1667	0.2552	0.9627	-0.9008	0.0069	-0.2861
HIP_35778	VCGERGFF	FLVHSSNNF	VCGERGFFFLVHSSNNF		IAPP	581/45018	0.998	35778	0.819	0.007343	2.6667	0.1667	1	17	0.3118	0	3	1	1	1.000000	1.8667	0	-0.0833	1.650000	0.1111	1.5389	0	1	0	1	0	4.1037	5.6	1	0	0	0	0.2833	1.0767	0.7167	0.8333	0.0803	0.5136	0.7556	0.0069	-0.1556
HIP_35779	HLVEALYL	NKPYALNSE	HLVEALYLNKPYALNSE		SCG2	17334/334890	0.997	35779	0.819	0.191877	-0.45	0.1667	0	17	-0.1824	-1	2	1	0	0.000000	-5.1	1	0.1667	1.850000	0.1111	1.7389	1	0	1	0	-5.1	-0.7825	-10.2	1	0	0	0	0.4269	0.9805	0.9823	0.1667	0.1823	0.9649	-0.1021	0.0278	-0.85
HIP_35780	CGERGFFY	NQKTSYFP	CGERGFFYNQKTSYFP		SCG2	23514/334890	0.994	35780	0.819	0.208079	-1.1	0	1	16	-0.9063	1	3	0	0	0.000000	-5.0667	1	0.15	0.700000	0.2222	0.4778	0	0	0	0	-5.0667	-0.5256	-15.2	0	1	0	0	0.4103	1.0372	0.9256	0.1667	0.1683	0.8568	-0.4513	0.0225	-0.76
HIP_35781	QHLCGSHLV	KEEEEEMAV	QHLCGSHLVKEEEEEMAV		CMGA	9347/247050	0.995	35781	0.819	0.171341	-1.0167	0	-2	18	-0.5722	-4	0	0	3	0.000000	-5.2333	-1	0.15	0.950000	0.4444	0.5056	0	0	0	0	5.2333	-0.514	0	1	0	0	0	0.4103	1.2372	0.7156	-0.3333	0.1683	0.5121	-0.4171	0.0225	-0.785
HIP_35782	LVEALYLVC	HHTAEKRKR	LVEALYLVCHHTAEKRKR		SCG1	30033/367830	0.965	35782	0.819	0.111965	0.5667	0	4	18	-0.35	2	1	0	0	0.000000	-5.8667	0	0.05	2.450000	0	2.45	0	0	0	0	0	1.3883	-5.8667	0	1	0	0	0.3821	1.0424	0.7801	0	0.146	0.6086	0.2165	0.0025	-0.2933
HIP_35783	SHLVEALY	GEDAPAEDM	SHLVEALYGEDAPAEDM		NPY	919/49410	0.995	35783	0.819	0.180481	-0.5167	0.1667	-4	17	-0.3353	-5	1	1	0	0.000000	-3.9	-2	0.1833	0.000000	0.3333	-0.3333	0	1	0	1	7.8	0.1722	-3.9	0	1	0	0	0.4269	1.1338	0.869	0	0.1823	0.7551	-0.2206	0.0136	-0.715
HIP_35784	LCGSHLVEA	MLVKYPEII	LCGSHLVEAMLVKYPEII		SCG2	10801/334890	0.999	35784	0.819	0.033326	2.0667	0	0	18	0.9333	-1	0	0	0	1.000000	2.4667	1	-0.05	0.475000	0.2222	0.2528	0	0	0	0	2.4667	0.5224	0	1	0	0	0	0.2667	1.2633	0.5833	0.6667	0.0711	0.3403	0.5511	0.0025	-0.1233
HIP_35785	HLVEALYLV	QGLVDREKG	HLVEALYLVQGLVDREKG		CMGA	3312/247050	0.999	35785	0.819	0.137685	1.1	0.1667	0	18	0.1056	-1	1	0	0	0.000000	-2.2667	0	0.1333	2.450000	0	2.45	0	1	0	1	0	2.695	-2.2667	1	0	0	0	0.3885	1.0486	0.8145	0.5	0.1509	0.6634	0.4273	0.0178	-0.3022
HIP_35786	VEALYLVC	TSRKDVKDK	VEALYLVCTSRKDVKDK		SCG1	23991/367830	0.982	35786	0.819	0.255068	0.75	0.1667	1	17	-0.2941	1	1	1	0	0.000000	-5.5	1	0.1667	0.625000	0.3333	0.2917	0	0	0	0	-5.5	0.2188	-5.5	0	0	0	0	0.4103	1.0055	0.829	0.1667	0.1683	0.6872	0.3077	0.0278	-0.9167
HIP_35787	VCGERGFF	GPGPQLRRG	VCGERGFFGPGPQLRRG		CMGA	4447/247050	0.999	35787	0.819	0.168216	0.4667	0.6667	2	17	-0.5647	2	2	1	0	0.000000	-2.5333	0	0.15	0.700000	0.1111	0.5889	1	3	1	1	0	0.2748	-5.0667	1	0	0	0	0.5333	0.745	1.2033	0.3333	0.2844	1.448	0.2489	0.0225	-0.38
HIP_35788	SICSLYQL	ALAEAYPS	SICSLYQLALAEAYPS		NPY	1295/49410	0.993	35788	0.819	-0.002147	1.0667	0	-1	16	0.6375	-1	1	0	0	1.000000	2.8	0	-0.05	0.450000	0.1111	0.3389	0	0	0	0	0	0.3615	2.8	1	0	0	0	0.3051	1.2719	0.7012	0.6667	0.0931	0.4916	0.3255	0.0025	-0.14
HIP_35789	LYQLENYCN	PLIFLISGA	LYQLENYCNPLIFLISGA		SCG2	4915/334890	1.000	35789	0.819	-0.129080	0.7333	0.1667	-1	18	0.6611	-1	2	0	0	0.000000	3	0	0.0167	1.325000	0.1111	1.2139	1	0	1	0	0	0.8902	6	0	1	0	0	0.4269	0.9438	0.9773	0.3333	0.1823	0.9551	0.3131	0.0003	0.05
HIP_35790	LCGSHLVEA	IVEEQYTPQ	LCGSHLVEAIVEEQYTPQ		SCG2	12438/334890	0.998	35790	0.819	-0.045621	1.2833	0	-2	18	-0.0389	-3	0	0	0	1.000000	0.9	0	0	1.125000	0.3333	0.7917	0	0	0	0	0	1.066	0	1	0	0	0	0.3333	1.2783	0.6017	0.3333	0.1111	0.362	0.4278	0	0
HIP_35791	ERGFFYTPK	NAVEVLKRE	ERGFFYTPKNAVEVLKRE		IAPP	1345/45018	0.994	35791	0.819	0.053762	-0.6167	0.1667	1	18	-0.8444	1	2	0	0	0.000000	2.9	-1	-0.1167	0.700000	0.2222	0.4778	1	0	1	0	-2.9	-0.2946	5.8	0	0	0	1	0.4551	0.9336	1.0178	0.1667	0.2071	1.036	-0.2807	0.0136	-0.3383
HIP_35792	QCCTSICSL	EEGPTVVLN	QCCTSICSLEEGPTVVLN		CMGA	3724/247050	0.999	35792	0.819	0.109277	-0.3167	0.3333	-2	18	0.5	-2	0	0	0	0.000000	-4.3	-2	0.1167	0.000000	0.2222	-0.2222	0	1	0	1	8.6	0.0704	0	1	0	0	0	0.5218	1.1069	0.9312	-0.1667	0.2723	0.867	-0.1652	0.0136	-0.5017
HIP_35793	CGERGFFY	FRGPGPQLR	CGERGFFYFRGPGPQLR		CMGA	14824/247050	0.987	35793	0.819	0.185593	0.3667	0.1667	2	17	-0.7	2	4	1	0	0.000000	-2.1333	1	0.1333	0.000000	0.1111	-0.1111	0	1	0	1	-2.1333	-0.0407	-8.5333	0	1	0	0	0.3603	1.0238	0.8523	0.3333	0.1298	0.7264	0.1321	0.0178	-0.2844
HIP_35794	GSHLVEALY	LSAEPGWQA	GSHLVEALYLSAEPGWQA		CMGA	5704/247050	0.998	35794	0.819	-0.023050	1.5167	0.1667	-1	18	0.0611	-2	1	0	0	0.000000	0.1667	0	0.0667	2.950000	0.2222	2.7278	0	0	0	0	0	4.1371	0.1667	0	1	0	0	0.3051	1.2203	0.7278	0.6667	0.0931	0.5444	0.4628	0.0044	0.0111
HIP_35795	YLVCGERGF	QEKAEKGRE	YLVCGERGFQEKAEKGRE		SCG2	24731/334890	0.994	35795	0.819	0.021612	-2.1667	0.1667	0	18	-1.2056	0	1	0	0	0.000000	-2.9333	-1	0.0167	0.450000	0.4444	0.0056	0	1	0	1	2.9333	-0.012	-2.9333	1	0	0	0	0.4936	1.0605	0.939	-0.3333	0.2436	0.8817	-1.0684	0.0003	-0.0489
HIP_35796	GERGFFYTP	ISDLLMRES	GERGFFYTPISDLLMRES		NPY	1077/49410	0.994	35796	0.819	-0.180116	-0.5667	0.3333	-1	18	-0.3278	-1	3	0	0	0.000000	1.2667	-1	0	2.775000	0.1111	2.6639	1	0	1	0	-1.2667	-1.5095	3.8	0	0	0	1	0.4936	0.8872	0.994	0	0.2436	0.988	-0.2797	0	0
HIP_35797	RGFFYTPKT	EENELTLNE	RGFFYTPKTEENELTLNE		SCG1	19287/367830	0.990	35797	0.819	-0.049275	-2.7833	0.1667	-2	18	-1.2	-2	1	0	0	0.000000	-1.4333	-3	-0.0167	0.000000	0.4444	-0.4444	1	0	1	0	4.3	1.237	-1.4333	0	1	0	0	0.5885	1.0186	1.0712	-0.5	0.3463	1.1474	-1.6379	0.0003	0.0239
HIP_35798	CGERGFFY	PRTRLEDPA	CGERGFFYPRTRLEDPA		NPY	2705/49410	0.979	35798	0.819	0.199122	-0.4167	0.1667	0	17	-0.9588	0	3	1	0	0.000000	-3.7	1	0.1667	0.950000	0.2222	0.7278	1	0	1	0	-3.7	-0.3032	-11.1	0	1	0	0	0.4269	0.9738	0.9156	0.1667	0.1823	0.8384	-0.1779	0.0278	-0.6167
HIP_35799	HLVEALYL	QGLVDREK	HLVEALYLQGLVDREK		CMGA	4275/247050	0.999	35799	0.819	0.194025	1.0333	0.1667	0	16	-0.1188	-1	1	0	0	0.000000	-2.1333	0	0.1333	1.050000	0.1111	0.9389	0	1	0	1	0	0.9702	-2.1333	1	0	0	0	0.3885	1.0886	0.8145	0.5	0.1509	0.6634	0.4014	0.0178	-0.2844
HIP_35800	LVEALYLVC	GGKSGELEQ	LVEALYLVCGGKSGELEQ		CMGA	7240/247050	0.997	35800	0.819	0.201784	0.9667	0.3333	-2	18	0.3722	-2	1	0	0	0.000000	-5.0667	1	0.2167	2.000000	0.1111	1.8889	0	2	0	1	-5.0667	1.8259	-5.0667	1	0	0	0	0.4436	0.9005	0.969	0.1667	0.1968	0.9389	0.4288	0.0469	-1.0978
HIP_35801	GAGSLQPLA	PFKRTNEIV	GAGSLQPLAPFKRTNEIV		SCG2	15759/334890	0.997	35801	0.819	0.031508	0.2167	0.3333	1	18	-0.0944	1	1	0	0	0.000000	-2.2333	1	0.05	1.900000	0.2222	1.6778	2	0	1	0	-2.2333	0.3635	-2.2333	1	0	0	0	0.4051	1.0186	0.9512	0.3333	0.1641	0.9047	0.0878	0.0025	-0.1117
HIP_35802	HLCGSHLVE	ERATRLFEK	HLCGSHLVEERATRLFEK		SCG2	36068/334890	0.983	35802	0.819	0.101167	-0.2833	0	2	18	-0.5	0	0	0	0	0.000000	-3.5667	1	0.05	1.050000	0.3333	0.7167	0	0	0	0	-3.5667	-0.2031	0	0	0	1	0	0.3718	1.3086	0.6745	0	0.1382	0.4549	-0.1053	0.0025	-0.1783
HIP_35803	QHLCGSHLV	GEESEEERG	QHLCGSHLVGEESEEERG		SCG1	6443/367830	0.999	35803	0.819	0.167250	-0.4333	0.1667	-2	18	-1.1333	-4	0	0	1	0.000000	-4.0667	-2	0.1833	0.950000	0.2222	0.7278	0	1	0	1	8.1333	-0.3154	0	1	0	0	0	0.4551	1.1503	0.8145	-0.1667	0.2071	0.6634	-0.1972	0.0136	-0.7456
HIP_35804	GERGFFYTP	EKGLSAEPG	GERGFFYTPEKGLSAEPG		CMGA	9938/247050	0.995	35804	0.819	-0.035215	-1.9	0.3333	-1	18	-0.85	-1	2	0	0	0.000000	-1.4	0	0.0667	1.650000	0.2222	1.4278	1	1	1	1	0	-2.7128	-4.2	0	0	0	1	0.5769	0.9088	1.089	-0.3333	0.3328	1.1859	-1.0962	0.0044	-0.0933
HIP_35805	CSLYQLENY	TAEKRLEGQ	CSLYQLENYTAEKRLEGQ		CMGA	17645/247050	0.980	35805	0.819	-0.074167	-1.7833	0	-1	18	-1.0111	-1	1	0	0	0.000000	1.9667	0	0	0.000000	0.3333	-0.3333	0	0	0	0	0	0.5944	1.9667	0	1	0	0	0.5051	1.1653	0.9278	-0.1667	0.2552	0.8609	-0.9008	0	0
HIP_35806	TSICSLYQL	ENIEKNEQI	TSICSLYQLENIEKNEQI		SCG2	14532/334890	0.998	35806	0.819	0.014593	-0.5833	0	-2	18	-0.4667	-2	1	0	0	0.000000	-0.5	-1	0.05	0.000000	0.2222	-0.2222	0	0	0	0	0.5	0.1286	-0.5	1	0	0	0	0.4385	1.1153	0.8595	0.1667	0.1922	0.7387	-0.2558	0.0025	-0.025
HIP_35807	EQCCTSICS	QNGERATRL	EQCCTSICSQNGERATRL		SCG2	8707/334890	0.999	35807	0.819	0.170288	-0.2	0.3333	0	18	-0.6833	0	0	0	0	0.000000	-4.5333	0	0.1167	0.625000	0.1111	0.5139	0	1	0	1	0	-0.1028	0	0	1	0	0	0.5218	0.8636	1.1028	0.1667	0.2723	1.2162	-0.1044	0.036	-0.5289

HIP_36271	ERGFFYTPK	RLLRDPADA	ERGFFYTPKRLLRDPADA		SCG1	11837/367830	0.997	36271	0.819	0.056302	-0.5167	0.1667	1	18	-0.9278	1	2	0	0
HIP_36272	HLCGSHLVE	DESHLPERD	HLCGSHLVEDESHLPERD		SCG2	12218/334890	0.998	36272	0.819	0.132487	-0.55	0.1667	-1	18	-0.95	-4	0	0	0
HIP_36273	ERGFFYTPK	GEKRDHHS	ERGFFYTPKGEKRDHHS		SCG1	27390/367830	0.974	36273	0.819	-0.024673	-2.3333	0.3333	3	17	-1.9588	1	2	1	0
HIP_36274	GPGAGSSLQP	SQECFETLR	GPGAGSSLQPSQECFETLR		CMGA	7922/247050	0.997	36274	0.819	-0.057662	-1.5167	0.3333	-1	18	-0.5833	-1	0	0	0
HIP_36275	HLVEALYL	YDWWEKKP	HLVEALYLYDWWEKKP		SCG1	21839/367830	0.987	36275	0.819	0.092274	0.1	0	0	16	-0.6313	-1	4	0	0
HIP_36276	GSLQPLAL	QECFETLRG	GSLQPLALQECFETLRG		CMGA	11749/247050	0.993	36276	0.819	0.164314	0.8167	0	-1	17	-0.0059	-1	1	1	0
HIP_36277	HLVEALYLV	QKKHSGFED	HLVEALYLVQKKHSGFED		CMGA	18609/247050	0.977	36277	0.819	0.193593	-0.7667	0	1	18	-0.35	-1	1	0	0
HIP_36278	ICSLYQLEN	EEGPTVVLN	ICSLYQLENEEGPTVVLN		CMGA	7433/247050	0.997	36278	0.819	0.031901	-1.7667	0.1667	-3	18	0.0556	-3	1	0	0
HIP_36279	GAGSLQPLA	DLFQNRMLS	GAGSLQPLADLFQNRMLS		SCG2	9664/334890	0.999	36279	0.819	-0.035535	1.1833	0.1667	0	18	0.0556	0	1	0	0
HIP_36280	GSHLVEALY	APAEDMARY	GSHLVEALYAPAEDMARY		NPY	774/49410	0.996	36280	0.819	-0.020072	1.05	0.1667	-1	18	-0.15	-2	1	0	0
HIP_36281	VEQCCTSIC	EEEEEEEEE	VEQCCTSICEEEEEEEEE		CMGA	2991/247050	0.999	36281	0.819	-0.034965	-0.7167	0.1667	-10	18	-1.3222	-10	0	0	7
HIP_36282	YLVCGERGF	NKPYALNSE	YLVCGERGFNKPYALNSE		SCG2	17352/334890	0.997	36282	0.819	0.035766	-1.85	0.3333	0	18	-0.5167	0	2	0	0
HIP_36283	LYQLENYCN	PYGAGRSRS	LYQLENYCNPYGAGRSRS		SCG2	7470/334890	0.999	36283	0.819	0.023657	-0.9333	0.3333	1	18	-1.0556	1	2	0	0
HIP_36284	RGFFYTPKT	AYPSKPDNP	RGFFYTPKTAYPSKPDNP		NPY	1853/49410	0.988	36284	0.819	0.034379	-1.2167	0.3333	2	18	-1.3056	2	2	0	0
HIP_36285	GSHLVEALY	MDMSDDHET	GSHLVEALYMDMSDDHET		SCG2	5839/334890	0.999	36285	0.819	-0.057204	0.7667	0	-4	18	-0.4778	-5	1	0	0
HIP_36286	ERGFFYTPK	YGEESEEER	ERGFFYTPKYGEESEEER		SCG1	7486/367830	0.999	36286	0.819	-0.133975	-1.9	0.3333	-3	18	-1.9333	-3	3	0	1
HIP_36287	GERGFFYTP	LPLQVRGYP	GERGFFYTPLPLQVRGYP		CMGA	5913/247050	0.998	36287	0.819	-0.083451	0.4	0.3333	1	18	-0.4389	1	3	0	0
HIP_36288	SHLVEALY	GEEENELTL	SHLVEALYGEEENELTL		SCG1	7160/367830	0.999	36288	0.819	0.179657	-0.5167	0.1667	-4	17	-0.3647	-5	1	1	1
HIP_36289	GPGAGSSLQP	FQRNQLLQK	GPGAGSSLQPFQRNQLLQK		SCG2	28570/334890	0.991	36289	0.819	0.006635	-1.0833	0.1667	2	18	-0.8389	2	1	0	0
HIP_36290	LCGSHLVE	NWGYEKRNL	LCGSHLVENWGYEKRNL		SCG1	23244/367830	0.984	36290	0.819	0.148639	-0.05	0.1667	1	17	-0.6647	0	2	1	0
HIP_36291	CGERGFFY	EEKRFLGE	CGERGFFYEEKRFLGE		SCG1	21529/367830	0.987	36291	0.819	0.187878	-1.1	0	-1	16	-0.9187	-1	3	0	0
HIP_36292	HLVEALYL	ATCATQRLA	HLVEALYLATCATQRLA		IAPP	2004/45018	0.991	36292	0.819	0.052546	1.65	0	1	17	0.6882	0	1	1	0
HIP_36293	LVEALYLV	EEERLSKEW	LVEALYLVEEERLSKEW		CMGA	8038/247050	0.997	36293	0.819	0.111668	-0.6333	0	-3	17	-0.2059	-3	1	1	1
HIP_36294	GERGFFYTP	KCNTATCAT	GERGFFYTPKCNTATCAT		IAPP	2660/45018	0.983	36294	0.819	-0.046140	-1.4167	0.1667	1	18	-0.4278	1	3	0	0
HIP_36295	HLCGSHLVE	EALPDGLSV	HLCGSHLVEEALPDGLSV		SCG2	15512/334890	0.998	36295	0.819	-0.034178	1.1	0	-1	18	0.3889	-3	0	0	0
HIP_36296	ERGFFYTPK	EAGLPLQVR	ERGFFYTPKEAGLPLQVR		CMGA	10713/247050	0.994	36296	0.819	-0.110809	-1.3833	0.3333	1	18	-0.5667	1	2	0	0
HIP_36297	GSHLVEALY	PGEEDIQGP	GSHLVEALYPGEEDIQGP		SCG1	2904/367830	1.000	36297	0.819	0.160489	-0.2	0.3333	-3	18	-0.5056	-4	1	0	0
HIP_36298	QHLCGSHLV	DMARYYSAL	QHLCGSHLVDMARYYSAL		NPY	3241/49410	0.972	36298	0.819	0.077546	0.8333	0	2	18	0.0611	0	0	0	0
HIP_36299	GERGFFYTP	ANNIAYEDV	GERGFFYTPANNIAYEDV		SCG2	8293/334890	0.999	36299	0.819	-0.139919	-1.4667	0.1667	-2	18	-0.5444	-2	3	0	0
HIP_36300	VEALYLVCG	ALNHLKATP	VEALYLVCGALNHLKATP		IAPP	812/45018	0.997	36300	0.819	-0.010955	1.4	0.1667	1	18	0.7444	0	1	0	0
HIP_36301	SLYQLENYC	EAELEKVAH	SLYQLENYCEAELEKVAH		CMGA	11533/247050	0.993	36301	0.819	-0.095513	-1.25	0	-2	18	-0.5444	-3	1	0	0
HIP_36302	ICSLYQLEN	YLNQEKAEK	ICSLYQLENYLNQEKAEK		SCG2	12401/334890	0.998	36302	0.819	-0.038712	-0.7	0	-1	18	-0.8611	-1	2	0	0
HIP_36303	CCTSICSLY	NRQMAYENL	CCTSICSLYNRQMAYENL		SCG2	23407/334890	0.995	36303	0.819	0.055922	-1.6333	0.1667	0	18	-0.0056	0	1	0	0
HIP_36304	ERGFFYTPK	KRFPVGPPK	ERGFFYTPKKRFPVGPPK		SCG2	29021/334890	0.991	36304	0.819	0.044411	-1.9667	0.1667	4	18	-1.1556	4	3	0	0
HIP_36305	ELGGGPGA	ERGFFYTPK	ELGGGPGAERGFFYTPK		INSB	201/4968	0.973	36305	0.819	-0.005128	-1.4333	0.5	0	17	-0.6706	0	2	1	1
HIP_36306	SLYQLENYC	DSQSIYQLI	SLYQLENYCDSQSIYQLI		SCG2	10372/334890	0.999	36306	0.819	0.112822	-1.6833	0.1667	-2	18	-0.2444	-2	1	0	0
HIP_36307	GSHLVEALY	LYTDDEDDI	GSHLVEALYLYTDDEDDI		SCG2	3721/334890	1.000	36307	0.819	-0.077702	1.0167	0	-5	18	-0.3778	-6	2	0	0
HIP_36308	YLVCGERGF	ALAEAYPS	YLVCGERGFALAEAYPS		NPY	618/49410	0.997	36308	0.819	0.123751	0.8833	0.1667	-1	17	0.3059	-1	1	1	0
HIP_36309	CGERGFFYT	SGLTLALSL	CGERGFFYTSGLTLALSL		NPY	381/49410	0.998	36309	0.819	0.029468	0.5667	0.3333	0	18	0.6444	0	3	0	0
HIP_36310	CGERGFFYT	VMKCVEVI	CGERGFFYTVMKCVEVI		CMGA	9991/247050	0.995	36310	0.819	-0.064067	0.5	0	0	18	0.8833	0	3	0	0
HIP_36311	QCCTSICSL	EKNFPMDMS	QCCTSICSLEKNFPMDMS		SCG2	22430/334890	0.995	36311	0.819	0.156304	-0.9	0.1667	-1	18	-0.0111	-1	1	0	0
HIP_36312	VEALYLVC	TPQSLATLE	VEALYLVCTPQSLATLE		SCG2	9965/334890	0.999	36312	0.819	0.203516	0.7833	0.1667	-2	17	0.8294	-2	1	1	0
HIP_36313	LVEALYLV	ENQMDKARR	LVEALYLVENQMDKARR		SCG1	17499/367830	0.993	36313	0.819	0.193416	-0.6333	0	0	17	-0.3765	0	1	1	0
[illegible]																			

HIP_36782	RGFFYTPKT	QQQGDLQDT	RGFFYTPKTQQQGDLQDT		SCG1	23892/367830	0.983	36782	0.818	-0.036740	-2.7833	0.1667	0	18	-1.4333	0	1	0	1
HIP_36783	LVCGERGFF	YRAPRPQSE	LVCGERGFFYRAPRPQSE		SCG1	24740/367830	0.981	36783	0.818	0.059829	0.2	0.1667	1	18	-0.6778	1	3	0	0
HIP_36784	CCTSICSLY	AHQLQALRR	CCTSICSLYAHQLQALRR		CMGA	17156/247050	0.981	36784	0.818	-0.050060	-0.5333	0.1667	3	18	0.2333	2	1	0	0
HIP_36785	LVCGERGFF	RSETHAAG	LVCGERGFFRSETHAAG		SCG1	23409/367830	0.984	36785	0.818	0.136384	-0.6	0.3333	1	17	-0.1294	0	2	1	0
HIP_36786	HLVEALYLV	GAKERAHQ	HLVEALYLVGAKERAHQ		CMGA	12221/247050	0.992	36786	0.818	0.218381	0.7	0.1667	2	17	-0.1059	0	1	1	0
HIP_36787	FVNQHLCGS	KEWEDSKRW	FVNQHLCGSKEWEDSKRW		CMGA	15788/247050	0.985	36787	0.818	0.016004	-1.1667	0.3333	1	18	-1.3056	0	1	0	0
HIP_36788	GSHLVEALY	DVVGGEDWN	GSHLVEALYDVVGGEDWN		SCG2	7163/334890	0.999	36788	0.818	-0.048906	1.5333	0	-3	18	-0.1611	-4	1	0	0
HIP_36789	YLVCGERGF	YENLNDKDQ	YLVCGERGFYENLNDKDQ		SCG2	12350/334890	0.998	36789	0.818	-0.053464	-1.7333	0.1667	-2	18	-1.0667	-2	2	0	0
HIP_36790	GERGFFYTP	NPGEDAPAE	GERGFFYTPNPGEDAPAE		NPY	153/49410	0.999	36790	0.818	-0.058936	-1.5167	0.5	-3	18	-1.1556	-3	3	0	0
HIP_36791	NQHLCGSHL	GEPRAYFMS	NQHLCGSHLGEPRAYFMS		SCG1	8906/367830	0.998	36791	0.818	0.149778	-0.95	0.5	2	18	-0.5611	0	0	0	0
HIP_36792	QHLCGSHLV	ALSKSSAPP	QHLCGSHLVALSKSSAPP		SCG1	19808/367830	0.990	36792	0.818	0.070112	1.6	0.1667	3	18	0.0611	1	0	0	0
HIP_36793	VELGGGPGA	QEEERLSKE	VELGGGPGAQEEERLSKE		CMGA	4493/247050	0.999	36793	0.818	-0.078414	-1.7833	0.3333	-3	18	-1.1	-3	0	0	2
HIP_36794	VELGGGPGA	EQEEERLSK	VELGGGPGAEQEEERLSK		CMGA	4494/247050	0.999	36794	0.818	-0.078414	-1.7833	0.3333	-3	18	-1.1	-3	0	0	2
HIP_36795	PGAGSLQPL	WNPVEEKIE	PGAGSLQPLWNPVEEKIE		SCG2	4222/334890	1.000	36795	0.818	-0.124799	-1.2167	0.3333	-2	18	-0.5889	-2	1	0	0
HIP_36796	LYQLENYCN	LALSLLVCL	LYQLENYCNLALSLLVCL		NPY	831/49410	0.996	36796	0.818	-0.045240	1.1833	0	-1	18	1.1222	-1	1	0	0
HIP_36797	CGERGFFY	TPECRQVL	CGERGFFYTPECRQVL		SCG1	15642/367830	0.994	36797	0.818	0.182168	-0.25	0.1667	0	16	-0.3313	0	3	0	0
HIP_36798	GSHLVEALY	LQIPPEDLI	GSHLVEALYLQIPPEDLI		SCG2	7860/334890	0.999	36798	0.818	-0.044898	1.5167	0	-2	18	0.4056	-3	1	0	0
HIP_36799	GERGFFYTP	QTVLTEDEK	GERGFFYTPQTVLTEDEK		SCG1	4348/367830	0.999	36799	0.818	-0.183282	-0.6	0.1667	-2	18	-1.0056	-2	3	0	0
HIP_36800	TSICSLYQL	GPTKADTEK	TSICSLYQLGPTKADTEK		SCG1	5398/367830	0.999	36800	0.818	0.158960	-0.6167	0.3333	0	18	-0.4944	0	1	0	0
HIP_36801	LCGSHLVEA	REEARFQDK	LCGSHLVEAREEARFQDK		SCG1	33631/367830	0.951	36801	0.818	0.196961	-1.5	0	0	18	-0.7833	-1	0	0	0
HIP_36802	SICSLYQLE	AASFQRNQL	SICSLYQLEAASFQRNQL		SCG2	10404/334890	0.999	36802	0.818	-0.118297	-0.0667	0.1667	0	18	-0.05	0	2	0	0
HIP_36803	HLVEALYLV	LAVDGAGKP	HLVEALYLVLAVDGAGKP		CMGA	8440/247050	0.996	36803	0.818	-0.009886	2.75	0	0	18	0.8556	-1	1	0	0
HIP_36804	VCGERGFF	NQSSQAELK	VCGERGFFNQSSQAELK		CMGA	11887/247050	0.992	36804	0.818	0.117112	-0.4333	0.3333	0	17	-0.6118	0	2	1	0
HIP_36805	LYQLENYCN	PYALNSEKN	LYQLENYCNPYALNSEKN		SCG2	6481/334890	0.999	36805	0.818	-0.173852	-0.5667	0.1667	-1	18	-1.0556	-1	2	0	0
HIP_36806	LVEALYLVC	SLGEKRDHH	LVEALYLVCSLGEKRDHH		SCG1	6873/367830	0.999	36806	0.818	0.169514	2.1833	0.3333	1	18	0.0056	-1	1	0	0
HIP_36807	LYQLENYCN	QQGDLQDTK	LYQLENYCNQQGDLQDTK		SCG1	7710/367830	0.998	36807	0.818	0.119378	-1.6167	0.1667	-2	18	-1.4	-2	1	0	0
HIP_36808	SICSLYQLE	AYPSKPDNP	SICSLYQLEAYPSKPDNP		NPY	1268/49410	0.993	36808	0.818	-0.168256	-0.7167	0.1667	-1	18	-0.6278	-1	2	0	0
HIP_36809	VCGERGFF	VALNHLKA	VCGERGFFVALNHLKA		IAPP	820/45018	0.997	36809	0.818	0.034601	2.5	0.1667	2	16	0.5188	1	2	0	0
HIP_36810	AGSLQPLAL	QGDLQDTKE	AGSLQPLALQGDLQDTKE		SCG1	7803/367830	0.998	36810	0.818	0.193978	0.3333	0.1667	-2	18	-0.5556	-2	0	0	0
HIP_36811	SHLVEALYL	LELDKMANG	SHLVEALYLLELDKMANG		SCG1	15865/367830	0.994	36811	0.818	0.035290	1.7333	0	0	18	0.3	-2	1	0	0
HIP_36812	SHLVEALY	AFLNERKQA	SHLVEALYAFLNERKQA		SCG1	17671/367830	0.992	36812	0.818	-0.008640	2.1167	0	1	17	-0.2294	0	2	1	0
HIP_36813	CCTSICSLY	PFKRTNEIV	CCTSICSLYPFKRTNEIV		SCG2	17490/334890	0.997	36813	0.818	0.006528	-0.1667	0.3333	1	18	0.3333	1	2	0	0
HIP_36814	NQHLCGSHL	AALSLIPLI	NQHLCGSHLAALSLIPLI		SCG2	11715/334890	0.998	36814	0.818	0.060619	1.2	0.1667	2	18	0.95	0	1	0	0
HIP_36815	LVCGERGFF	ALSLLVCLG	LVCGERGFFALSLLVCLG		NPY	693/49410	0.996	36815	0.818	-0.034616	1.6667	0.3333	0	18	1.6556	0	2	0	0
HIP_36816	LVEALYLV	EEEEEEEEA	LVEALYLVEEEEEEEEA		CMGA	4247/247050	0.999	36816	0.818	0.076164	-0.6333	0	-9	17	-0.5529	-9	1	1	6
HIP_36817	LVEALYLV	EEEEEEEEA	LVEALYLVEEEEEEEEA		CMGA	4248/247050	0.999	36817	0.818	0.076164	-0.6333	0	-9	17	-0.5529	-9	1	1	6
HIP_36818	GERGFFYTP	SPMNKGDTE	GERGFFYTPSPMNKGDTE		CMGA	3290/247050	0.999	36818	0.818	-0.145182	-0.6833	0.5	-1	18	-1.2667	-1	3	0	0
HIP_36819	HLVEALYLV	QGLFRGGK	HLVEALYLVQGLFRGGK		CMGA	8434/247050	0.996	36819	0.818	0.170628	1.1	0.1667	2	17	0.4176	1	2	1	0
HIP_36820	HLVEALYL	RLPYGAGRS	HLVEALYLRLPYGAGRS		SCG2	18629/334890	0.997	36820	0.818	0.158981	0.6667	0.1667	2	17	0.0882	1	2	1	0
HIP_36821	CGERGFFY	QAIKEHLN	CGERGFFYQAIKEHLN		SCG2	17975/334890	0.997	36821	0.818	0.054143	1.1833	0	1	16	-0.5938	0	3	0	0
HIP_36822	AGSLQPLAL	EEEDNRDS	AGSLQPLALEEEDNRDS		CMGA	7924/247050	0.997	36822	0.818	0.134066	-0.1833	0	-4	17	-1.0353	-4	0	1	1
HIP_36823	SHLVEALYL	YKANNIAY	SHLVEALYLYKANNIAY		SCG2	28006/334890	0.992	36823	0.818	0.131589	0.4833	0	1	17	0.1882	0	2	1	0
HIP_36824	VEALYLVC	VKDKETTEN	VEALYLVCVKDKETTEN		SCG1	22153/367830	0.986	36824	0.818	0.189084	1.2167	0	-2	17	-0.2059	-2	1	1	0
HIP_36825	DLQVGQVEL	EEERGLEPG	DLQVGQVELEEERGLEPG		SCG1	13538/367830	0.996	36825	0.818	0.069389	-1	0	-5	18	-0.8611	-5	0	0	1
HIP_36826	GSHLVEALY	YQKGERGE	GSHLVEALYYQKGERGE		SCG1	26834/367830	0.975	36826	0.818	0.128165	-0.7333	0	0	17	-0.9765	-1	2	1	0
HIP_36827	VCGERGFFY	QAPEDLEW	VCGERGFFYQAPEDLEW		SCG1	11983/367830	0.997	36827	0.818	0.120666	0.1667	0.1667	-3	17	-0.5118	-3	3	1	0
HIP_36828	VCGERGFF	TYGKRNAVE	VCGERGFFTYGKRNAVE		IAPP	2071/45018	0.990	36828	0.818	0.074358	0.4667	0.3333	1	17	-0.4882	1	3	1	0
HIP_36829	QHLCGSHLV	DPEGKGEQE	QHLCGSHLVDPEGKGEQE		CMGA	6536/247050	0.998	36829	0.818	0.162494	-0.6333	0.1667	-1	18	-1.1444	-3	0	0	0
HIP_36830	AGSLQPLAL	GLPLQVRGY	AGSLQPLALGLPLQVRGY		CMGA	3810/247050	0.999	36830	0.818	0.177719	1.8667	0.3333	1	18	0.4889	1	0	0	0
HIP_36831	RGFFYTPKT	AFLNERKQA	RGFFYTPKTAFLNERKQA		SCG1	13298/367830	0.996	36831	0.818	0.115529	0.3667	0.1667	3	18	-0.9	3	2	0	0
HIP_36832	QCCTSICSL	GPTKADTEK	QCCTSICSLGPTKADTEK		SCG1	4093/367830	0.999	36832	0.818	0.169208	0.4667	0.5	0	18	-0.3556	0	0	0	0
HIP_36833	GSHLVEALY	TKADTEKWA	GSHLVEALYTKADTEKWA		SCG1	21394/367830	0.987	36833	0.818	0.135623	0.25	0	0	18	-0.5056	-1	1	0	0
HIP_36834	GSLQPLALE	KSSHFERRD	GSLQPLALEKSSHFERRD		SCG1	25664/367830	0.978	36834	0.818	0.198068	-0.5667	0.3333	1	18	-1.0278	0	0	0	0
HIP_36835	CTSICSLYQ	DRSSQGGSL	CTSICSLYQDRSSQGGSL		SCG1	26063/367830	0.977	36835	0.818	0.211713	-1.6333	0.1667	0	18	-0.2611	0	1	0	0
HIP_36836	VEQCCTSIC	AYGFRGPGP	VEQCCTSICAYGFRGPGP		CMGA	6737/247050	0.998	36836	0.818	-0.025105	1.05	0.3333	0	18	0.1167	0	2	0	0
HIP_36837	LCGSHLVEA	GEYLARMLV	LCGSHLVEAGEYLARMLV		SCG2	14059/334890	0.998	36837	0.818	0.121060	-0.45	0.1667	0	18	0.7778	-1	1	0	0
HIP_36838	SLYQLENYC	NYGEEGAPG	SLYQLENYCNYGEEGAPG		SCG1	3888/367830	0.999	36838	0.818	-0.024056	-1.25	0.1667	-3	18	-0.9222	-3	2	0	0
HIP_36839	GERGFFYTP	SAPPITPEC	GERGFFYTPSAPPITPEC		SCG1	3701/367830	0.999	36839	0.818	-0.147092	-0.7	0.5	-1	18	-0.4333	-1	3	0	0

HIP_37293	CCTSICSLY	RDNPFKRTN	CCTSICSLYRDNPFKRTN		SCG2	27689/334890	0.992	37293	0.818	0.106560	-1.6333	0.1667	2	18	-0.5944	2	1	0
HIP_37294	GERGFFYTP	LGMESAANQ	GERGFFYTPLGMESAANQ		SCG2	3829/334890	1.000	37294	0.818	-0.123775	0.2833	0.3333	-1	18	-0.5111	-1	3	0
HIP_37295	LVEALYLV	DREKGLSAE	LVEALYLVDREKGLSAE		CMGA	12054/247050	0.992	37295	0.818	0.180452	-0.8	0	-2	17	0.1353	-2	1	1
HIP_37296	RGFFYTPKT	DSKENIEKN	RGFFYTPKTDSKENIEKN		SCG2	27856/334890	0.992	37296	0.818	-0.003304	-2.4	0.3333	1	18	-1.6167	1	1	0
HIP_37297	AGSLQPLAL	EEEEEEAT	AGSLQPLALEEEEEEAT		CMGA	5833/247050	0.998	37297	0.818	0.114712	-0.1833	0	-6	17	-0.6588	-6	0	1
HIP_37298	VEALYLVC	YPEEKKEEE	VEALYLVCYPEEKKEEE		CMGA	8814/247050	0.996	37298	0.818	0.127496	0.6833	0.1667	-4	17	-0.7471	-4	2	1
HIP_37299	DLQVGQVEL	EEEGENYQK	DLQVGQVELEEEGENYQK		SCG1	13758/367830	0.996	37299	0.818	0.067051	-1	0	-5	18	-1.3889	-5	0	0
HIP_37300	GERGFFYTP	QAEGDSEGL	GERGFFYTPQAEGDSEGL		CMGA	6490/247050	0.998	37300	0.818	-0.126975	-1.4667	0.1667	-3	18	-0.9333	-3	3	0
HIP_37301	LVEALYLVC	ESTENVPRT	LVEALYLVCESTENVPRT		NPY	1489/49410	0.992	37301	0.818	0.192189	0.9167	0.1667	-2	18	0.2611	-2	1	0
HIP_37302	CGERGFFY	LLMRESTEN	CGERGFFYLLMRESTEN		NPY	1798/49410	0.989	37302	0.818	-0.047424	2.3	0	-1	17	-0.5294	-1	3	1
HIP_37303	HLCGSHLVE	PDFYDSEE	HLCGSHLVEPDFYDSEE		SCG1	10572/367830	0.997	37303	0.818	0.022446	0.3667	0.1667	-5	17	-0.6882	-5	2	1
HIP_37304	ICSLYQLEN	YKEIRKGES	ICSLYQLENYKEIRKGES		CMGA	25207/247050	0.949	37304	0.818	-0.001487	-1.9833	0	0	18	-0.85	0	2	0
HIP_37305	VEALYLVCG	EEEEEGENY	VEALYLVCGEEEEEGENY		SCG1	7424/367830	0.999	37305	0.818	-0.001197	-0.7	0.1667	-7	18	-0.6167	-7	1	0
HIP_37306	CSLYQLENY	WRPSSREDS	CSLYQLENYWRPSSREDS		CMGA	13864/247050	0.989	37306	0.818	-0.079592	-2.55	0.1667	-1	18	-1.3722	-1	2	0
HIP_37307	LVEALYLV	PRLPYGAGR	LVEALYLVPRLPYGAGR		SCG2	18317/334890	0.997	37307	0.818	0.164986	0.7333	0.1667	1	17	0.4765	1	2	1
HIP_37308	YLVCGERGF	GAGRSRSNQ	YLVCGERGFGAGRSRSNQ		SCG2	11178/334890	0.999	37308	0.818	-0.013090	-0.1833	0.5	2	18	-0.7444	2	1	0
HIP_37309	VCGERGFFY	KDQELGEY	VCGERGFFYKDQELGEY		SCG2	20147/334890	0.996	37309	0.818	0.194052	-1.1	0	-2	17	-0.8	-2	3	1
HIP_37310	GSHLVEALY	NLLKELQDL	GSHLVEALYNLLKELQDL		CMGA	6647/247050	0.998	37310	0.818	-0.023517	1.4	0	-1	18	0.0944	-2	1	0
HIP_37311	NQHLCGSHL	PEPMQESKA	NQHLCGSHLPEPMQESKA		CMGA	6104/247050	0.998	37311	0.818	0.090626	-1.15	0.5	1	18	-1.0667	-1	0	0
HIP_37312	GPGAGSLQP	FRARAYGFR	GPGAGSLQPFRARAYGFR		CMGA	15412/247050	0.986	37312	0.818	0.063448	-0.2	0.1667	3	18	-0.5056	3	1	0
HIP_37313	NQHLCGSHL	EEEEEGENY	NQHLCGSHLEEEEEGENY		SCG1	11661/367830	0.997	37313	0.818	0.001001	-1.7833	0.1667	-4	18	-1.7056	-6	0	0
HIP_37314	QHLCGSHLV	ENVQKFPSP	QHLCGSHLVENVQKFPSP		SCG2	19364/334890	0.996	37314	0.818	0.181809	0.3333	0	2	18	-0.4556	0	0	0
HIP_37315	SHLVEALYL	LIPLIFLIS	SHLVEALYLLIPLIFLIS		SCG2	6797/334890	0.999	37315	0.818	0.007393	2.1667	0.1667	0	18	1.8833	-1	1	0
HIP_37316	ERGFFYTPK	PGEDAPAED	ERGFFYTPKPGEDAPAED		NPY	898/49410	0.995	37316	0.818	-0.136663	-1.95	0.5	-3	18	-1.35	-3	2	0
HIP_37317	QHLCGSHLV	EEMAVVPQG	QHLCGSHLVEEMAVVPQG		CMGA	9790/247050	0.995	37317	0.818	0.098457	-0.05	0	0	18	0.1556	-2	0	0
HIP_37318	HLVEALYLV	QKLYTDDE	HLVEALYLVQKLYTDDE		SCG2	17961/334890	0.997	37318	0.818	0.129215	0.5167	0	-2	17	-0.1471	-3	2	1
HIP_37319	LCGSHLVEA	DTEKWAEGG	LCGSHLVEADTEKWAEGG		SCG1	19062/367830	0.991	37319	0.818	0.148199	-0.8667	0	-2	18	-0.3778	-3	0	0
HIP_37320	VEALYLVCG	KRFPVGPPK	VEALYLVCGKRFPVGPPK		SCG2	27944/334890	0.992	37320	0.818	0.081388	0.1167	0.1667	2	18	0.2556	2	2	0
HIP_37321	TSICSLYQL	AAMDLELQK	TSICSLYQLAAMDLELQK		SCG1	11205/367830	0.997	37321	0.818	-0.003077	0.75	0	-1	18	0.3444	-1	1	0
HIP_37322	IVEQCCTSI	NDNFLEGEE	IVEQCCTSINDNFLEGEE		SCG1	8585/367830	0.998	37322	0.818	-0.053971	-1.25	0.1667	-5	18	-0.2833	-5	1	0
HIP_37323	DLQVGQVEL	LISDLLMRE	DLQVGQVELLISDLLMRE		NPY	925/49410	0.995	37323	0.818	-0.052698	2	0.1667	-3	18	0.3944	-3	0	0
HIP_37324	LVEALYLVC	LPERDSLSE	LVEALYLVCLPERDSLSE		SCG2	4970/334890	1.000	37324	0.818	0.089045	1.5333	0.1667	-3	18	0.4833	-3	1	0
HIP_37325	VCGERGFF	PDNPGEDAP	VCGERGFFPDNPGEDAP		NPY	1391/49410	0.992	37325	0.818	0.068419	-0.5667	0.3333	-3	17	-0.8176	-3	2	1
HIP_37326	RGFFYTPKT	NYGEEGAPG	RGFFYTPKTNYGEEGAPG		SCG1	12582/367830	0.996	37326	0.818	-0.066647	-1.9	0.3333	0	18	-1.1278	0	2	0
HIP_37327	VEALYLVCG	VFLIVLSVA	VEALYLVCGVFLIVLSVA		IAPP	542/45018	0.998	37327	0.818	-0.003475	2.85	0.1667	-1	18	2.4222	-1	2	0
HIP_37328	HLVEALYLV	RSQEESEEG	HLVEALYLVRSQEESEEG		SCG1	16508/367830	0.994	37328	0.818	0.196708	-0.35	0.1667	-3	18	-0.5778	-4	1	0
HIP_37329	HLCGSHLVE	QWSLYPSDS	HLCGSHLVEQWSLYPSDS		SCG1	20881/367830	0.988	37329	0.818	0.053655	-0.1167	0.1667	0	18	-0.3444	-2	1	0
HIP_37330	ICSLYQLEN	YPSDSQVSE	ICSLYQLENYPSDSQVSE		SCG1	6564/367830	0.999	37330	0.818	-0.106456	-1.15	0.3333	-3	18	-0.5333	-3	2	0
HIP_37331	DLQVGQVEL	WQAKREEEE	DLQVGQVELWQAKREEEE		CMGA	11238/247050	0.993	37331	0.818	-0.009627	0.3167	0	-4	18	-1.3	-4	1	0
HIP_37332	VCGERGFFY	EEAEAGEE	VCGERGFFYEEAEAGEE		CMGA	10005/247050	0.995	37332	0.818	0.079365	-0.15	0	-5	17	-0.7118	-5	3	1
HIP_37333	ERGFFYTPK	QESKGQPR	ERGFFYTPKQESKGQPR		SCG1	30759/367830	0.963	37333	0.818	-0.024760	-2.3333	0.3333	2	17	-1.8824	2	2	1
HIP_37334	ERGFFYTPK	LYPSDSQVS	ERGFFYTPKLYPSDSQVS		SCG1	8876/367830	0.998	37334	0.818	-0.044365	-0.8833	0.3333	0	18	-0.8111	0	3	0
HIP_37335	VCGERGFF	EEKGHPQE	VCGERGFFEEKGHPQE		SCG1	20765/367830	0.988	37335	0.818	0.088960	-0.95	0.1667	-1	16	-1.225	-2	2	0
HIP_37336	SHLVEALY	FLGEGHHRV	SHLVEALYFLGEGHHRV		SCG1	8066/367830	0.998	37336	0.818	0.031802	1.75	0.1667	2	17	0.0235	-1	2	1
HIP_37337	GSLQPLALE	RVPKLDLKR	GSLQPLALERVPKLDLKR		SCG1	32514/367830	0.955	37337	0.818	0.102537	0.0333	0.1667	2	18	-0.3722	2	0	0
HIP_37338	LVCGERGFF	DMARYYSA	LVCGERGFFDMARYYSA		NPY	1973/49410	0.987	37338	0.818	0.032848	0.9	0.1667	0	17	0.0824	0	2	1
HIP_37339	GAGSLQPLA	RRTRPRHHH	GAGSLQPLARRTRPRHHH		SCG1	36616/367830	0.937	37339	0.818	0.116846	-0.95	0.1667	7	18	-1.4111	4	0	0
HIP_37340	NQHLCGSHL	VLEYLNQEK	NQHLCGSHLVLEYLNQEK		SCG2	16052/334890	0.997	37340	0.818	-0.058526	0.7167	0.1667	1	18	-0.6611	-1	1	0
[illegible]																		

HIP_37804	HLVEALYLV	AAVNSMPVD	HLVEALYLVAAVNSMPVD		SCG1	11142/367830	0.997	37804	0.817	-0.017291	2.4167	0	-1	18	1.0056	-2	1	0	0	1.000000	0.3667	0	0	1.850000	0	1.85	0	0	0	0	0	4.4708	0.3667	1	0	0	0	0.2385	1.2236	0.6212	0.8333	0.0569	0.3858	0.5763	0	0
HIP_37805	ERGFFYTPK	QAPEDLEWE	ERGFFYTPKQAPEDLEWE		SCG1	4499/367830	0.999	37805	0.817	-0.140812	-1.5833	0.3333	-3	18	-1.3722	-3	2	0	0	0.000000	0.9667	-1	-0.05	0.700000	0.2222	0.4778	2	0	1	0	-0.9667	-0.7565	1.9333	0	0	1	0	0.5385	0.9253	1.0912	0	0.2899	1.1906	-0.8526	0.0025	-0.0483
HIP_37806	QHLCGSHLV	EEEEEMAVV	QHLCGSHLVEEEEEMAVV		CMGA	6792/247050	0.997	37806	0.817	0.109351	-0.95	0	-3	18	-0.1222	-5	0	0	3	0.000000	-5.1	-3	0.15	0.950000	0.4444	0.5056	0	0	0	0	15.3	-0.4803	0	1	0	0	0	0.4385	1.3186	0.6878	-0.3333	0.1922	0.4731	-0.4165	0.0225	-0.765
HIP_37807	DLQVGQVEL	EEGENYQKG	DLQVGQVELEEGENYQKG		SCG1	9217/367830	0.998	37807	0.817	0.146111	-0.4833	0.1667	-4	18	-1.2167	-4	0	0	0	0.000000	-3.9667	-1	0.1333	0.000000	0.4444	-0.4444	0	1	0	1	3.9667	0.2148	0	1	0	0	0	0.4833	1.2317	0.7867	-0.1667	0.2336	0.6188	-0.2336	0.0178	-0.5289
HIP_37808	CTSICSLYQ	EGEEENELT	CTSICSLYQEGEEENELT		SCG1	3137/367830	1.000	37808	0.817	0.082036	-1.4	0.1667	-5	18	-0.6722	-5	1	0	1	0.000000	-2.1333	-2	0.1333	0.625000	0.3333	0.2917	0	1	0	1	4.2667	-0.4083	-2.1333	0	1	0	0	0.5218	1.1586	0.8945	-0.1667	0.2723	0.8001	-0.7305	0.0178	-0.2844
HIP_37809	VEALYLVC	KSAEFPDFY	VEALYLVCKSAEFPDFY		SCG1	20524/367830	0.989	37809	0.817	0.159240	1.2667	0.1667	-2	17	0.4882	-2	2	1	0	0.000000	-4.4667	1	0.1167	1.325000	0.2222	1.1028	0	0	0	0	-4.4667	1.3969	-8.9333	0	1	0	0	0.3436	1.1088	0.779	0.3333	0.1181	0.6068	0.4352	0.0136	-0.5211
HIP_37810	GSLQPLALE	SSHFERRDN	GSLQPLALESSHFERRDN		SCG1	23531/367830	0.983	37810	0.817	0.089230	-0.45	0.3333	0	18	-1.0056	-1	1	0	0	0.000000	-2.3	1	0.0833	1.650000	0.1111	1.5389	0	0	0	0	-2.3	-0.6925	-2.3	0	0	1	0	0.4385	1.1469	0.8678	0	0.1922	0.7531	-0.1973	0.0069	-0.1917
HIP_37811	CTSICSLYQ	KGEQEHSQQ	CTSICSLYQKGEQEHSQQ		CMGA	11771/247050	0.992	37811	0.817	0.189029	-1.4667	0.1667	0	18	-1.0889	-1	1	0	0	0.000000	-2.2667	1	0.1333	0.625000	0.2222	0.4028	0	1	0	1	0	-0.5907	-2.2667	0	1	0	0	0.4936	1.0772	0.9223	-0.1667	0.2436	0.8507	-0.7239	0.0178	-0.3022
HIP_37812	AGSLQPLAL	ERGEDSSEE	AGSLQPLALERGEDSSEE		SCG1	11224/367830	0.997	37812	0.817	0.206041	0.1667	0.1667	-4	18	-0.85	-4	0	0	0	0.000000	-5.9333	0	0.1833	0.450000	0.3333	0.1167	0	1	0	1	0	0.0194	0	1	0	0	0	0.3885	1.1803	0.8012	0.1667	0.1509	0.6418	0.0647	0.0136	-1.0878
HIP_37813	SICSLYQLE	DSSMKLSFR	SICSLYQLEDSSMKLSFR		CMGA	14280/247050	0.988	37813	0.817	-0.027391	-1.3833	0.3333	0	18	-0.0611	0	1	0	0	0.000000	-0.6333	0	0.0833	2.375000	0.2222	2.1528	0	0	0	0	0	-2.978	-0.6333	0	0	1	0	0.5051	1.0903	0.9212	-0.1667	0.2552	0.8485	-0.6988	0.0069	-0.0528
HIP_37814	CGERGFFY	PGPQAEGD	CGERGFFYPGPQAEGD		CMGA	4969/247050	0.998	37814	0.817	0.176461	0.1167	0.5	-2	16	-0.9188	-2	3	0	0	0.000000	-2.6333	0	0.2167	0.700000	0.1111	0.5889	2	1	1	1	0	0.0687	-7.9	0	1	0	0	0.4885	0.8436	1.1078	0.3333	0.2386	1.2273	0.057	0.0469	-0.5706
HIP_37815	CCTSICSLY	GWRPSSRED	CCTSICSLYGWRPSSRED		CMGA	12039/247050	0.992	37815	0.817	0.027914	-0.6833	0.3333	0	18	-0.4611	0	2	0	0	0.000000	-2.5	1	0.0667	2.075000	0.1111	1.9639	0	1	0	1	-2.5	-1.342	-5	0	1	0	0	0.4654	0.9441	0.9868	0.1667	0.2166	0.9738	-0.318	0.0044	-0.1667
HIP_37816	VCGERGFF	NWGYEKRN	VCGERGFFNWGYEKRN		SCG1	17330/367830	0.993	37816	0.817	0.068304	0.0667	0.3333	1	16	-1.125	1	4	0	0	0.000000	-3.3333	0	0.05	0.700000	0.2222	0.4778	0	2	0	1	0	0.0319	-13.3333	1	0	0	0	0.4718	0.8486	1.1112	0.5	0.2226	1.2347	0.0315	0.0025	-0.1667
HIP_37817	CGERGFFYT	LVHSSNNFG	CGERGFFYTLVHSSNNFG		IAPP	1826/45018	0.992	37817	0.817	-0.029427	0.9333	0	1	18	-0.2278	0	3	0	0	0.000000	1.3333	0	-0.05	0.950000	0	0.95	0	0	0	0	0	0.8867	4	0	1	0	0	0.3436	1.0722	0.729	0.3333	0.1181	0.5314	0.3207	0.0025	-0.0667
HIP_37818	HLVEALYL	YPGPQAEG	HLVEALYLYPGPQAEG		CMGA	6146/247050	0.998	37818	0.817	0.190858	0.5	0.3333	-1	16	-0.0688	-2	2	0	0	0.000000	-3.2	0	0.15	0.000000	0.1111	-0.1111	1	1	1	1	0	-0.0556	-6.4	1	0	0	0	0.4436	0.9522	0.9723	0.3333	0.1968	0.9454	0.2218	0.0225	-0.48
HIP_37819	GERGFFYTP	SAEFPDFYD	GERGFFYTPSAEFPDFYD		SCG1	5155/367830	0.999	37819	0.817	-0.183318	-1.0167	0.3333	-3	18	-0.7556	-3	4	0	0	0.000000	0.3667	-1	0	1.400000	0.1111	1.2889	1	0	1	0	-0.3667	-1.3104	1.4667	0	0	0	1	0.5218	1.0303	0.9978	0	0.2723	0.9956	-0.5305	0	0
HIP_37820	CGERGFFYT	GSGRLQNG	CGERGFFYTGSGRLQNG		SCG2	7961/334890	0.999	37820	0.817	0.186408	-0.1333	0.5	1	17	-0.7294	1	3	1	0	0.000000	-0.8	0	0.1833	0.000000	0.1111	-0.1111	0	2	0	1	0	0.0148	-2.4	0	1	0	0	0.5385	0.8086	1.1112	0.1667	0.2899	1.2347	-0.0718	0.0136	-0.1467
HIP_37821	HLVEALYL	QAENEPQSA	HLVEALYLQAENEPQSA		SCG2	11847/334890	0.998	37821	0.817	0.113704	0.1833	0	-2	17	-0.4059	-3	1	1	0	0.000000	-3.8333	-1	0.1	1.850000	0.2222	1.6278	0	0	0	0	3.8333	0.2984	-3.8333	1	0	0	0	0.3718	1.2819	0.7145	0.3333	0.1382	0.5105	0.0682	0.01	-0.3833
HIP_37822	GERGFFYTP	QGSSEDDLQ	GERGFFYTPQGSSEDDLQ		SCG2	47/334890	1.000	37822	0.817	-0.073538	-1.3833	0.5	-3	18	-1.25	-3	3	0	0	0.000000	-0.3667	0	0.0833	0.700000	0	0.7	1	1	1	1	0	-0.9683	-1.1	0	0	0	1	0.6051	0.8053	1.1778	0	0.3662	1.3873	-0.8371	0.0069	-0.0306
HIP_37823	LYQLENYCN	QGDLQDTKE	LYQLENYCNQGDLQDTKE		SCG1	5726/367830	0.999	37823	0.817	0.063016	-1.6167	0.1667	-3	18	-1.4	-3	1	0	0	0.000000	-1.7	-1	0.1333	1.575000	0.2222	1.3528	0	1	0	1	1.7	-2.187	-1.7	0	1	0	0	0.5321	0.8908	1.1268	-0.1667	0.2831	1.2697	-0.8601	0.0178	-0.2267
HIP_37824	GSHLVEALY	MSDDYETQQ	GSHLVEALYMSDDYETQQ		SCG2	4371/334890	1.000	37824	0.817	0.074619	0.3167	0.1667	-3	18	-0.7778	-4	1	0	0	0.000000	-2.2333	-1	0.1167	2.475000	0.3333	2.1417	0	0	0	0	2.2333	0.6782	-2.2333	0	1	0	0	0.3436	1.1322	0.7956	0.3333	0.1181	0.633	0.1088	0.0136	-0.2606
HIP_37825	ICSLYQLEN	GERATRLFE	ICSLYQLENGERATRLFE		SCG2	21367/334890	0.995	37825	0.817	0.082686	-1.9333	0.1667	-1	18	-0.3722	-1	1	0	0	0.000000	-1.7333	1	0.0833	0.450000	0.3333	0.1167	0	1	0	1	-1.7333	-0.2256	-1.7333	0	1	0	0	0.5218	1.0853	0.9912	-0.3333	0.2723	0.9824	-1.0088	0.0069	-0.1444
HIP_37826	HLVEALYL	IVLSVALNH	HLVEALYLIVLSVALNH		IAPP	1565/45018	0.993	37826	0.817	-0.019434	3.1333	0	1	17	1.4235	-1	1	1	0	1.000000	2.0667	0	0	3.500000	0	3.5	0	0	0	0	0	10.9667	2.0667	1	0	0	0	0.2385	1.1769	0.5628	0.8333	0.0569	0.3168	0.7472	0	0
HIP_37827	AGSLQPLA	FNPYYDPLQ	AGSLQPLAFNPYYDPLQ		SCG1	12405/367830	0.997	37827	0.817	0.041773	0.2833	0.3333	-1	17	-0.2824	-1	3	1	0	1.000000	-2.1	0	0.05	0.450000	0	0.45	2	0	1	0	0	0.1275	-6.3	1	0	0	0	0.4333	0.96	1.06	0.5	0.1878	1.1236	0.1228	0.0025	-0.105
HIP_37828	CGERGFFYT	PQGLFRGG	CGERGFFYTPQGLFRGG		CMGA	8651/247050	0.996	37828	0.817	0.160258	-0.7833	0.3333	1	17	-0.4059	1	3	1	0	0.000000	-2.1	0	0.15	0.950000	0	0.95	1	1	1	1	0	-0.7442	-6.3	0	1	0	0	0.5385	0.8719	1.0778	0.1667	0.2899	1.1617	-0.4218	0.0225	-0.315
HIP_37829	LVEALYLVC	NPVEEKIES	LVEALYLVCNPVEEKIES		SCG2	4186/334890	1.000	37829	0.817	0.116565	1.6	0.1667	-3	18	0.4278	-3	1	0	0	0.000000	-3.8	0	0.1167	2.000000	0.1111	1.8889	1	0	1	0	0	3.0222	-3.8	0	0	0	0	0.3885	0.9469	0.9145	0.5	0.1509	0.8363	0.6215	0.0136	-0.4433
HIP_37830	DLQVGQVEL	PQESKGQPR	DLQVGQVELPQESKGQPR		SCG1	20358/367830	0.989	37830	0.817	0.208597	-0.6833	0.1667	-1	18	-1.2056	-1	0	0	0	0.000000	-4.3667	0	0.1167	0.000000	0.2222	-0.2222	1	0	1	0	0	0.1519	0	1	0	0	0	0.4833	1.1767	0.83	0	0.2336	0.6889	-0.3303	0.0136	-0.5094
HIP_37831	VCGERGFFY	YFMSDTRE	VCGERGFFYYFMSDTRE		SCG1	16266/367830	0.994	37831	0.817	-0.004632	1.2833	0	-1	17	-0.4353	-1	5	1	0	0.000000	-0.3	0	0	0.700000	0.1111	0.5889	0	0	0	0	0	0.7557	-1.5	0	1	0	0	0.2769	1.1405	0.7023	0.6667	0.0767	0.4932	0.3554	0	0
HIP_37832	CCTSICSLY	SETHAAGHS	CCTSICSLYSETHAAGHS		SCG1	12186/367830	0.997	37832	0.817	0.075100	-0.55	0.3333	1	18	0.1778	-1	1	0	0	0.000000	-2.2333	-1	0.1	2.075000	0.1111	1.9639	0	0	0	0	2.2333	-1.0801	-2.2333	0	1	0	0	0.5051	1.0436	0.9178	0	0.2552	0.8424	-0.2778	0.01	-0.2233
HIP_37833	GSHLVEALY	KEAVEEPSS	GSHLVEALYKEAVEEPSS		CMGA	10943/247050	0.994	37833	0.817	0.069115	-0.2167	0	-2	18	-0.4	-3	1	0	0	0.000000	-3.3	0	0.1	3.050000	0.3333	2.7167	0	0	0	0	0	-0.5886	-3.3	0	1	0	0	0.3436	1.2922	0.729	0.1667	0.1181	0.5314	-0.0744	0.01	-0.33
HIP_37834	VCGERGFF	GSGRLQNG	VCGERGFFGSGRLQNG		SCG2	5622/334890	0.999	37834	0.817	0.174925	0.6	0.6667	1	16	-0.3875	1	2	0	0	0.000000	-2.2667	0	0.15	0.700000	0.3333	0.3667	0	3	0	1	0	0.22	-4.5333	1	0	0	0	0.5167	0.7833	1.15	0.3333	0.2669	1.3225	0.31	0.0225	-0.34
HIP_37835	SHLVEALYL	PFSEDVNW	SHLVEALYLPFSEDVNW		SCG1	7013/367830	0.999	37835	0.817	0.168872	1.1167	0.3333	-2	17	0.1059	-3	2	1	0	0.000000	-1.9667	0	0.1333	0.000000	0.2222	-0.2222	1	0	1	0	0	-0.2481	-3.9333	1	0	0	0	0.3885	1.0319	0.8712	0.5	0.1509	0.7589	0.4338	0.0178	-0.2622
HIP_37836	QHLCGSHLV	EVDKRRTR	QHLCGSHLVEVDKRRTR		SCG1	29052/367830	0.969	37836	0.817	0.172099	0.3333	0	4	17	-1.0412	2	0	1	0	0.000000	-2.5333	-2	0.1	0.950000	0.3333	0.6167	0	0	0	0	5.0667	0.2056	0	0	1	0	0	0.3436	1.1622	0.6756	0	0.1181	0.4565	0.1145	0.01	-0.2533
HIP_37837	TSICSLYQL	DTREEKRFL	TSICSLYQLDTREEKRFL		SCG1	32713/367830	0.954	37837	0.817	0.228680	-1.6167	0	0	18	-0.5556	0	1	0	0	0.000000	-2.5667	0	0.1	0.000000	0.3333	-0.3333	0	0	0	0	0	0.5389	-2.5667	1	0	0	0	0.4487	1.0508	0.8601	-0.1667	0.2013	0.7398	-0.7254	0.01	-0.2567
HIP_37838	VEALYLVC	EEKHLEEPG	VEALYLVCEEKHLEEPG		SCG1	16391/367830	0.994	37838	0.817	0.172292	-0.0667	0	-3	17	-0.2235	-4	1	1	0	0.000000	-7.1333	-1	0.15	1.575000	0.3333	1.2417	0	0	0	0	7.1333	-0.0828	-7.1333	0	0	0	0	0.4103	1.2372	0.7156	-0.1667	0.1683	0.5121	-0.0274	0.0225	-1.07
HIP_37839	GSHLVEALY	DLFQNRML	GSHLVEALYDLFQNRML		SCG2	14837/334890	0.998	37839	0.817	0.011876	1.2333	0	0	17	0.1	-1	2	1	0	0.000000	-0.4	-1	0.05	2.000000	0.2222	1.7778	0	0	0	0	0.4	2.1926	-0.8	0	1	0	0	0.2769	1.2155	0.679	0.5	0.0767	0.461	0.3415	0.0025	-0.02
HIP_37840	DLQVGQVEL	IVLSVALNH	DLQVGQVELIVLSVALNH		IAPP	1054/45018	0.996	37840	0.817	0.013004	2.8333	0	-1	18	0.9111	-2	0	0	0	1.000000	2.6667	1	-0.05	1.125000	0.1111	1.0139	0	0	0	0	2.6667	2.8727	0	1	0	0	0	0.2667	1.2183	0.535	0.6667	0.0711	0.2862	0.7556	0.0025	-0.1333
HIP_37841	GAGSLQPLA	WERYRGRG	GAGSLQPLAWERYRGRG		SCG1	28720/367830	0.970	37841	0.817	0.066577	-0.8167	0.1667	2	17	-0.9118	2	2	1	0	1.000000	-4.3	0	0.0333	1.900000	0.2222	1.6778	1	0	1	0	0	-1.3702	-8.6	1	0	0	0	0.4269	1.1455	0.8723	0.1667	0.1823	0.7609	-0.3487	0.0011	-0.1433
HIP_37842	LCGSHLVEA	EPQWSLYPS	LCGSHLVEAEPQWSLYPS		SCG1	19089/367830	0.991	37842	0.817	0.196818	-1.0167	0.1667	-1	18	-0.1111	-2	1	0	0	0.000000	-3.7	0	0.1167	0.000000	0.2222	-0.2222	1	0	1	0	0	0.2259	-3.7	1	0	0	0	0.4833	1.2017	0.8567	0	0.2336	0.7339	-0.4914	0.0136	-0.4317
HIP_37843	VEALYLVC	SGFEDELSE	VEALYLVCSGFEDELSE		CMGA	2366/247050	0.999	37843	0.817	0.149053	2.0167	0.3333	-5	17	0.3588	-5	2	1	0	0.000000	-2.9667	0	0.15	0.625000	0.2222	0.4028	0	1	0	1	0	0.8123	-5.9333	0	0	0	0	0.3885	0.9803	0.8678	0.5	0.1509	0.7531	0.7834	0.0225	-0.445
HIP_37844	LCGSHLVEA	ADLDHPDLF	LCGSHLVEAADLDHPDLF		SCG2	14254/334890	0.998	37844	0.817	-0.034508	0.7667	0	-2	18	0.2833	-4	0	0	0	1.000000	-0.1333	0	0	0.450000	0.3333	0.1167	0	0	0	0	0	0.0894	0	1	0	0	0	0.3051	1.2986	0.6612	0.3333	0.0931	0.4371	0.2339	0	0
HIP_37845	SICSLYQLE	EGDSEGLSQ	SICSLYQLEEGDSEGLSQ		CMGA	7071/247050	0.997	37845	0.817	-0.063427	-1.7667	0.1667	-4	18	-0.4389	-4	1	0	0	0.000000	-1.4	-1	0.0833	1.900000	0.3333	1.5667	0	1	0	1	1.4	-2.7678	-1.4	0	0	1	0	0.5218	1.1586	0.8945	-0.3333	0.2723	0.8001	-0.9218	0.0069	-0.1167
HIP_37846	EQCCTSICS	GQNGERATR	EQCCTSICSGQNGERATR		SCG2	9303/334890	0.999	37846	0.817	0.163722	-0.2	0.3333	0	18	-0.9167	0	0	0	0	0.000000	-4.5333	0	0.1167	0.625000	0	0.625	0	1	0	1	0	-0.125	0	0	1	0	0	0.5218	0.8636	1.1026	0.1667	0.2723	1.2162	-0.1044	0.0136	-0.5289

HIP_38315	NQHLCGSHL	PYALNSEKN	NQHLCGSHLPYALNSEKN		SCG2	15212/334890	0.998	38315	0.817	-0.084512	-0.2167	0.3333	2	18	-0.9444	0	1	0
HIP_38316	AEDLQVGQV	EEEEAEAGE	AEDLQVGQVEEEEAEAGE		CMGA	4242/247050	0.999	38316	0.817	-0.080177	-1.7	0.1667	-8	18	-1.0111	-8	0	0
HIP_38317	NQHLCGSHL	EDSKRWSKM	NQHLCGSHLEDSKRWSKM		CMGA	17784/247050	0.980	38317	0.817	0.142212	-1.3333	0.3333	3	18	-1.3556	1	0	0
HIP_38318	GERGFFYTP	VLTEDEKKE	GERGFFYTPVLTEDEKKE		SCG1	6404/367830	0.999	38318	0.817	-0.087571	0.6167	0.1667	-2	18	-1.1833	-2	3	0
HIP_38319	AGSLQPLAL	PQGAWKELD	AGSLQPLALPQGAWKELD		SCG1	8355/367830	0.998	38319	0.817	0.210908	0.65	0.3333	-1	18	-0.1667	-1	0	0
HIP_38320	CGSHLVEAL	EEEAEAGEE	CGSHLVEALEEEAEAGEE		CMGA	7260/247050	0.997	38320	0.817	0.000556	-1.4	0	-6	18	-0.5333	-7	0	0
HIP_38321	SHLVEALYL	NEQINDEM	SHLVEALYLNEQINDEM		SCG2	12150/334890	0.998	38321	0.817	0.176498	-0.7	0	-3	17	-0.3529	-4	1	1
HIP_38322	HLVEALYLV	TEALPDGLS	HLVEALYLVTEALPDGLS		SCG2	7624/334890	0.999	38322	0.817	0.082948	0.7167	0	-2	18	0.6944	-3	1	0
HIP_38323	HLVEALYL	ENEPQSAPK	HLVEALYLENEPQSAPK		SCG2	13787/334890	0.998	38323	0.817	0.158417	-0.7	0	-1	17	-0.6294	-2	1	1
HIP_38324	HLVEALYLV	ALSLIPLIF	HLVEALYLVALSLIPLIF		SCG2	9907/334890	0.999	38324	0.817	0.015185	1.9167	0.1667	0	18	2.0111	-1	1	0
HIP_38325	CGERGFFYT	WIPHVENR	CGERGFFYTWIPHVENR		SCG2	12499/334890	0.998	38325	0.817	-0.042407	0.4667	0.1667	1	17	-0.6588	0	4	1
HIP_38326	VCGERGFF	EKSQREDEE	VCGERGFFEKSQREDEE		SCG1	18076/367830	0.992	38326	0.817	0.097395	-0.5	0.3333	-3	17	-1.5706	-3	2	1
HIP_38327	HLVEALYLV	EESNVSMA	HLVEALYLVEESNVSMA		SCG1	11428/367830	0.997	38327	0.817	0.171400	-0.1833	0.1667	-2	17	0.5529	-3	1	1
HIP_38328	RGFFYTPKT	ESTENVPRT	RGFFYTPKTESTENVPRT		NPY	3724/49410	0.963	38328	0.817	-0.033780	-1.8667	0.3333	1	18	-1.2278	1	1	0
HIP_38329	GERGFFYTP	FLISGAEAA	GERGFFYTPFLISGAEAA		SCG2	4218/334890	1.000	38329	0.817	0.004713	1.25	0.1667	-1	18	0.2778	-1	4	0
HIP_38330	SHLVEALYL	KRLEGQEEE	SHLVEALYLKRLEGQEEE		CMGA	18123/247050	0.979	38330	0.817	0.155130	0.2833	0	-2	18	-0.7722	-3	1	0
HIP_38331	VCGERGFFY	RLGLSGLTL	VCGERGFFYRLGLSGLTL		NPY	2192/49410	0.985	38331	0.817	0.166112	0.5333	0.1667	1	18	0.5889	1	3	0
HIP_38332	HLVEALYL	QFPPMYEEN	HLVEALYLQFPPMYEEN		SCG2	7250/334890	0.999	38332	0.817	0.099000	0.6667	0.1667	-2	17	-0.2588	-3	2	1
HIP_38333	SHLVEALYL	DKARRHPQ	SHLVEALYLDKARRHPQ		SCG1	35013/367830	0.945	38333	0.817	0.240966	0.1167	0	3	17	-0.8412	1	1	1
HIP_38334	VCGERGFFY	LEAGLPLQ	VCGERGFFYLEAGLPLQ		CMGA	11012/247050	0.994	38334	0.817	0.021378	1.0667	0	-1	17	0.3765	-1	3	1
HIP_38335	LYQLENYCN	EYIENLRQQ	LYQLENYCNEYIENLRQQ		SCG2	16118/334890	0.997	38335	0.817	-0.123279	-0.4333	0	-2	18	-1.1944	-2	2	0
HIP_38336	QHLCGSHLV	LAAMDLELQ	QHLCGSHLVLAAMDLELQ		SCG1	13696/367830	0.996	38336	0.817	-0.017491	2.0333	0	0	18	0.5333	-2	0	0
HIP_38337	GSHLVEALY	VDNRNHNEG	GSHLVEALYVDNRNHNEG		SCG1	12157/367830	0.997	38337	0.817	0.070059	0.25	0	0	18	-0.9444	-2	1	0
HIP_38338	GERGFFYTP	APEDLEWER	GERGFFYTPAPEDLEWER		SCG1	4857/367830	0.999	38338	0.817	-0.177279	-1.15	0.3333	-3	18	-1.2333	-3	3	0
HIP_38339	GSHLVEALY	EEAVPEEEG	GSHLVEALYEEAVPEEEG		CMGA	4739/247050	0.999	38339	0.817	0.005295	-0.15	0	-5	18	-0.5056	-6	1	0
HIP_38340	CGERGFFY	AYENLNDK	CGERGFFYAYENLNDK		SCG2	19998/334890	0.996	38340	0.817	0.061442	0.2167	0	-1	16	-0.975	-1	4	0
HIP_38341	SHLVEALYL	QEEEQIEQ	SHLVEALYLQEEEQIEQ		SCG2	11999/334890	0.998	38341	0.817	0.155729	-0.7	0	-4	17	-0.6706	-5	1	1
HIP_38342	LQVGQVELG	GQNGERATR	LQVGQVELGGQNGERATR		SCG2	13709/334890	0.998	38342	0.817	-0.033883	-1.25	0.3333	0	18	-0.8056	0	0	0
HIP_38343	ICSLYQLEN	AAWIPHVEN	ICSLYQLENAAWIPHVEN		SCG2	2921/334890	1.000	38343	0.817	-0.012580	-0.0833	0	-1	18	0.0889	-2	2	0
HIP_38344	SHLVEALYL	DIQGPTKA	SHLVEALYLDIQGPTKA		SCG1	20579/367830	0.989	38344	0.817	0.195315	0.6333	0	0	17	0.0765	-1	1	1
HIP_38345	HLVEALYLV	EGMVTRCII	HLVEALYLVEGMVTRCII		SCG1	6389/367830	0.999	38345	0.817	0.142891	0.7833	0.1667	0	18	1.2278	-1	1	0
HIP_38346	LVCGERGFF	KGRHHRGR	LVCGERGFFKGRHHRGR		SCG1	32075/367830	0.957	38346	0.817	0.162550	-0.6	0.3333	6	17	-1.0176	4	2	1
HIP_38347	PGAGSLQPL	NQSSQAELK	PGAGSLQPLNQSSQAELK		CMGA	11240/247050	0.993	38347	0.817	0.010285	-1.5167	0.3333	0	18	-0.7111	0	0	0
HIP_38348	VCGERGFFY	SEKNFPMD	VCGERGFFYSEKNFPMD		SCG2	19319/334890	0.996	38348	0.817	0.207802	-0.65	0.1667	-1	17	-0.5824	-1	3	1
HIP_38349	VEALYLVC	GRLQNGQNG	VEALYLVCGRLQNGQNG		SCG2	17158/334890	0.997	38349	0.817	0.224529	1.5667	0.1667	0	17	-0.0235	0	1	1
HIP_38350	VNQHLCGSH	DERILSILR	VNQHLCGSHDERILSILR		CMGA	17875/247050	0.979	38350	0.817	-0.083021	-2.65	0.3333	2	18	-0.2389	0	0	0
HIP_38351	GGGPGAGSL	EEEEAEAGE	GGGPGAGSLEEEEAEAGE		CMGA	4777/247050	0.998	38351	0.817	-0.069740	-1.3167	0.3333	-6	18	-0.9222	-6	0	0
HIP_38352	RGFFYTPKT	LMRESTENV	RGFFYTPKTLMRESTENV		NPY	1592/49410	0.991	38352	0.817	0.099520	-0.8333	0.1667	1	18	-0.7833	1	1	0
HIP_38353	LVEALYLV	SVALNHLKA	LVEALYLVSVALNHLKA		IAPP	1638/45018	0.993	38353	0.817	0.024601	1.9833	0.1667	1	17	1.2235	0	1	1
HIP_38354	ERGFFYTPK	VAQLDQLLH	ERGFFYTPKVAQLDQLLH		SCG1	9665/367830	0.998	38354	0.817	-0.004285	-0.6167	0.1667	1	18	-0.3667	0	2	0
HIP_38355	CGERGFFYT	AEGGGHSR	CGERGFFYTAEGGGHSR		SCG1	14867/367830	0.995	38355	0.817	0.097717	-0.2167	0.1667	1	17	-0.8294	0	3	1
HIP_38356	YLVCGERGF	PSQKYPGPQ	YLVCGERGFPSQKYPGPQ		CMGA	9903/247050	0.995	38356	0.817	0.023677	-1.3333	0.5	1	18	-0.8333	1	1	0
HIP_38357	VEALYLVCG	SQQKEEEEE	VEALYLVCGSQQKEEEEE		CMGA	2453/247050	0.999	38357	0.817	0.044804	-0.25	0.3333	-5	18	-0.7833	-5	1	0
HIP_38358	ICSLYQLEN	KPNGSVEPE	ICSLYQLENKPNGSVEPE		SCG2	20926/334890	0.996	38358	0.817	0.037963	-2.0333	0.1667	-2	18	-0.7	-2	1	0
HIP_38359	CGERGFFY	FEVRLLRDP	CGERGFFYFEVRLLRDP		SCG1	19473/367830	0.990	38359	0.817	0.034508	1.3	0	0	17	-0.2941	0	4	1
HIP_38360	TSICSLYQL	SREKSSQES	TSICSLYQLSREKSSQES		SCG1	28984/367830	0.969	38360	0.817	0.236090	-1.6333	0.1667	0	18	-0.8111	0	1	0
HIP_38361	DLQVGQVEL	VNAAGSGRL	DLQVGQVELVNAAGSGRL		SCG2	14167/334890	0.998	38361	0.817	-0.027716	1.1667	0	-1	18	0.2	-1	0	0
HIP_38362	AGSLQPLAL	EAGEEAVP	AGSLQPLALEAGEEAVP		CMGA	3035/247050	0.999	38362	0.817	0.160782	1.2167	0.1667	-3	17	0.2353	-3	0	1
HIP_38363	CGSHLVEAL	NFLVHSSNN	CGSHLVEALNFLVHSSNN		IAPP	1863/45018	0.992	38363	0.817	-0.050167	0.8667	0	1	18	0.2056	-1	1	0
HIP_38364	ICSLYQLEN	SAPKENKPY	ICSLYQLENSAPKENKPY		SCG2	13198/334890	0.998	38364	0.817	-0.066704	-0.6333	0.3333	0	18	-0.9056	0	1	0
HIP_38365	ERGFFYTP	NQMDKARRH	ERGFFYTPNQMDKARRH		SCG1	27743/367830	0.973	38365	0.817	-0.008942	-1.45	0.1667	3	17	-1.7235	2	2	1
HIP_38366	HLCGSHLVE	EDELSEVLE	HLCGSHLVEEDELSEVLE		CMGA	8075/247050	0.997	38366	0.817	0.020924	-1	0	-4	18	-0.1833	-6	0	0
HIP_38367	LCGSHLVEA	LLVCLGALA	LCGSHLVEALLVCLGALA		NPY	1386/49410	0.992	38367	0.817	0.017965	2.3833	0	0	18	1.85	-1	0	0
HIP_38368	SLYQLENYC	GEETGSQEN	SLYQLENYCGEETGSQEN		SCG1	5738/367830	0.999	38368	0.817	0.074026	-1.6167	0.1667	-4	18	-1.3111	-4	1	0
HIP_38369	GERGFFYTP	QLAKELTAE	GERGFFYTPQLAKELTAE		CMGA	4460/247050	0.999	38369	0.817	-0.072864	-0.25	0.1667	-1	18	-0.5944	-1	3	0
HIP_38370	VCGERGFFY	KEESSPDY	VCGERGFFYKEESSPDY		SCG2	19284/334890	0.996	38370	0.817	0.180226	-1.1	0	-2	17	-0.9824	-2	3	1
HIP_38371	LVEALYLVC	EDLIEMLKT	LVEALYLVCEDLIEMLKT		SCG2	9061/334890	0.999	38371	0.817	0.065213	1.2167	0	-3	18	1.0111	-3	1	0
HIP_38372	QCCTSICSL	QSIYQLIEI	QCCTSICSLQSIYQLIEI		SCG2	9978/334890	0.999	38372	0.817	0.054923	0.95	0.3333	-1	18	0.8167	-1	1	0
[illegible]	[illegible]	[illegible]	[illegible]	[illegible]	[illegible]	[illegible]	[illegible]	[illegible]	[illegible]	[illegible]	[illegible]	[illegible]	[illegible]	[illegible]	[illegible]	[illegible]	[illegible]	[illegible]

HIP_38826	AGSLQPLAL	PSQKYPGP	AGSLQPLALPSQKYPGP		CMGA	10389/247050	0.984	38826	0.816	0.245173	0.5833	0.3333	1	17	-0.3529	1	0	1	0	0.000000	-5.1	0	0.1833	0.450000	0.1111	0.3389	1	0	1	0	0	0.1977	0	1	0	0	0	0.4167	1.0883	0.8933	0.5	0.1736	0.798	0.2431	0.0036	-0.935
HIP_38827	AGSLQPLAL	KADTEKWA	AGSLQPLALKADTEKWA		SCG1	27067/367830	0.975	38827	0.816	0.218365	0.6333	0	0	17	-0.2412	0	0	1	0	0.000000	-5	0	0.1	0.450000	0.2222	0.2278	0	0	0	0	0	0.1443	0	1	0	0	0	0.2769	1.2572	0.689	0.3333	0.0767	0.4747	0.1754	0.01	-0.5
HIP_38828	CGERGFFY	AGTEALPD	CGERGFFYAGTEALPD		SCG2	3744/334890	1.000	38828	0.816	0.134738	0.8333	0.1667	-2	16	-0.2688	-2	3	0	0	0.000000	-1.2	0	0.1333	1.150000	0.2222	0.9278	0	1	0	1	0	0.7731	-3.6	0	1	0	0	0.3885	1.0336	0.8712	0.5	0.1509	0.7589	0.3237	0.0178	-0.16
HIP_38829	GERGFFYTP	PDFYDSEEP	GERGFFYTPPDFYDSEEP		SCG1	4349/367830	0.999	38829	0.816	-0.154121	-0.9833	0.3333	-4	18	-1.2944	-4	5	0	0	0.000000	0.4333	-1	0	0.700000	0.1111	0.5889	2	0	1	0	-0.4333	-0.5791	2.1667	0	0	0	1	0.5103	0.8688	1.069	0	0.2604	1.1427	-0.5018	0	0
HIP_38830	LGGGPGAGS	SGYPKTPGR	LGGGPGAGSSGYPKTPGR		SCG2	11418/334890	0.999	38830	0.816	-0.017999	-0.3167	0.6667	2	18	-0.7778	2	1	0	1	0.000000	-1.0333	0	0	0.000000	0	0	0	2	0	1	0	0	-1.0333	0	1	0	0	0.5385	0.8453	1.1612	0.1667	0.2899	1.3483	-0.1705	0	0
HIP_38831	HLVEALYL	QLDQLLHYR	HLVEALYLQLDQLLHYR		SCG1	15105/367830	0.995	38831	0.816	0.097283	0.5167	0	1	17	0.0765	-1	1	1	0	0.000000	-3.1667	-1	0.1	3.300000	0.1111	3.1889	0	0	0	0	3.1667	1.6476	-3.1667	1	0	0	0	0.3436	1.1755	0.7156	0.3333	0.1181	0.5121	0.1775	0.01	-0.3167
HIP_38832	CGSHLVEAL	TSRKDVKDK	CGSHLVEALTSRKDVKDK		SCG1	32160/367830	0.957	38832	0.816	0.182332	-0.65	0.1667	2	18	-0.6833	1	0	0	0	0.000000	-2.7	2	0.1167	1.400000	0.3333	1.0667	0	0	0	0	-5.4	-0.6933	0	1	0	0	0	0.4385	1.1519	0.8278	0	0.1922	0.6853	-0.285	0.0136	-0.315
HIP_38833	FVNQHLCGS	EDNRDSSMK	FVNQHLCGSEDNRDSSMK		CMGA	10421/247050	0.984	38833	0.816	-0.042276	-1.5333	0.3333	0	18	-1.1222	-1	0	0	0	0.000000	-3.9333	-2	0	0.000000	0.3333	-0.3333	0	1	0	1	7.8667	0.5111	0	0	1	0	0	0.5603	0.9205	1.1356	-0.3333	0.3139	1.2897	-0.8581	0	0
HIP_38834	SLYQLENYC	SGEATDGAR	SLYQLENYCSGEATDGAR		CMGA	2996/247050	0.999	38834	0.816	0.097976	-1.1667	0.3333	-2	18	-0.7778	-2	1	0	0	0.000000	-0.8	-1	0.15	1.400000	0.2222	1.1778	0	1	0	1	0.8	-1.3741	-0.8	0	0	0	0	0.5603	0.9205	1.1356	-0.1667	0.3139	1.2897	-0.6536	0.0025	-0.12
HIP_38835	ERGFFYTPK	EDVNWGYEK	ERGFFYTPKEDVNWGYEK		SCG1	21134/367830	0.988	38835	0.816	-0.040702	-1.5	0.1667	-1	18	-1.4778	-1	2	0	0	0.000000	1.1333	-1	-0.0667	0.700000	0.3333	0.3667	1	0	1	0	-3.4	-0.55	2.3667	0	0	1	0	0.4936	1.0022	0.9223	-0.3333	0.2436	0.8507	-0.7404	0.0044	-0.0756
HIP_38836	VELGGGPGA	EENSRDNPF	VELGGGPGAEENSRDNPF		SCG2	5769/334890	0.999	38836	0.816	-0.087135	-1.7833	0.3333	-3	18	-1.0278	-3	0	0	1	0.000000	-3.4333	-2	0	0.000000	0.2222	-0.2222	1	1	1	1	6.8667	0.3963	0	1	0	0	0	0.5667	1.035	1.12	-0.1667	0.3211	1.2544	-1.0106	0	0
HIP_38837	FVNQHLCGS	SQEKTHSRE	FVNQHLCGSSQEKTHSRE		SCG1	18936/367830	0.991	38837	0.816	-0.023149	-1.0833	0.5	2	18	-1.25	0	0	0	0	0.000000	-3.0333	-1	0.0167	0.000000	0.2222	-0.2222	0	1	0	1	3.0333	0.2407	0	0	1	0	0	0.5885	0.9569	1.0878	-0.1667	0.3463	1.1834	-0.6375	0.0003	-0.0506
HIP_38838	QHLCGSHLV	EEEEEAEAG	QHLCGSHLVEEEEEAEAG		CMGA	6465/247050	0.998	38838	0.816	0.100100	-0.95	0	-4	18	-0.8111	-6	0	0	3	0.000000	-5.1	-3	0.15	0.950000	0.4444	0.5056	0	0	0	0	15.3	-0.4803	0	1	0	0	0	0.4385	1.3186	0.6878	-0.3333	0.1922	0.4731	-0.4165	0.0225	-0.765
HIP_38839	SHLVEALYL	LIVLSVAL	SHLVEALYLLIVLSVAL		IAPP	1558/45018	0.993	38839	0.816	-0.008674	3.1333	0	0	17	1.9941	-1	1	1	0	1.000000	2.0667	0	0	1.900000	0.1111	1.7889	0	0	0	0	0	5.6052	2.0667	1	0	0	0	0.2385	1.1769	0.5628	0.8333	0.0569	0.3168	0.7472	0	0
HIP_38840	GERGFFYT	EWEDSKRWS	GERGFFYTEWEDSKRWS		CMGA	15999/247050	0.984	38840	0.816	0.054623	-1.1833	0	-1	17	-1.6176	-1	4	1	0	0.000000	-2.9	-2	0.05	0.700000	0.3333	0.3667	0	0	0	0	5.8	-0.4339	-11.6	0	1	0	0	0.4769	1.1822	0.809	0	0.2275	0.6544	-0.5644	0.0025	-0.145
HIP_38841	AGSLQPLAL	SHLVEALYL	AGSLQPLALSHLVEALYL		INSB	195/4968	0.975	38841	0.816	0.071888	1.5333	0.1667	0	18	0.9611	-1	0	0	0	0.000000	-3.2	0	0.0667	1.500000	0	1.5	0	0	0	0	0	2.3	0	1	0	0	0	0.3051	1.1953	0.7112	0.5	0.0931	0.5057	0.4679	0.0044	-0.2133
HIP_38842	VGQVELGGG	QWPERKLKH	VGQVELGGGQWPERKLKH		SCG2	3367/334890	1.000	38842	0.816	0.049214	-1.2	0.6667	2	18	-0.9778	1	1	0	1	0.000000	-1.6	0	-0.1333	0.000000	0.2222	-0.2222	1	3	1	1	0	0.2667	-1.6	0	0	0	1	0.6385	0.7003	1.3178	0.1667	0.4076	1.7367	-0.7662	0.0178	0.2133
HIP_38843	QHLCGSHLV	EEENELTLN	QHLCGSHLVEEENELTLN		SCG1	12435/367830	0.997	38843	0.816	0.117766	-0.95	0	-2	18	-0.6056	-4	0	0	1	0.000000	-5.1	-3	0.15	0.950000	0.3333	0.6167	0	0	0	0	15.3	-0.5858	0	1	0	0	0	0.4385	1.3186	0.6878	-0.3333	0.1922	0.4731	-0.4165	0.0225	-0.765
HIP_38844	CTSICSLYQ	PEDLIEMLK	CTSICSLYQPEDLIEMLK		SCG2	9798/334890	0.999	38844	0.816	0.069559	-1.6	0.1667	-2	18	0.2333	-2	1	0	0	0.000000	-2.5333	-2	0.1167	1.575000	0.2222	1.3528	1	0	1	0	5.0667	-2.1644	-2.5333	0	1	0	0	0.4936	1.0888	0.9256	-0.1667	0.2436	0.8568	-0.7897	0.0136	-0.2956
HIP_38845	EALYLVCGE	EDELSEVLE	EALYLVCGEEDELSEVLE		CMGA	6644/247050	0.998	38845	0.816	-0.139639	-1.9833	0.1667	-7	18	0.05	-7	0	0	0	0.000000	-3.0333	-2	0.0167	1.900000	0.4444	1.4556	0	1	0	1	6.0667	-2.8869	0	0	0	1	0	0.5603	1.1788	0.9223	-0.6667	0.3139	0.8507	-1.1112	0.0003	-0.0506
HIP_38846	GSLQPLAL	YDSEEPVS	GSLQPLALYDSEEPVS		SCG1	12822/367830	0.996	38846	0.816	0.164369	0.6333	0.1667	-3	16	-0.2438	-3	1	0	0	0.000000	-5	0	0.1167	0.450000	0.2222	0.2278	0	0	0	0	5	0.1443	-5	1	0	0	0	0.3436	1.1488	0.779	0.3333	0.1181	0.6068	0.2176	0.0136	-0.5833
HIP_38847	VEALYLVC	EPQWSLYP	VEALYLVCEPQWSLYP		SCG1	13364/367830	0.996	38847	0.816	0.173288	0.3167	0.1667	-2	16	0.3812	-2	2	0	0	0.000000	-6.3667	-1	0.1667	2.000000	0.1111	1.8889	1	0	1	0	6.3667	0.5981	-12.7333	0	0	0	0	0.4551	1.0953	0.8578	0.1667	0.2071	0.7359	0.1441	0.0278	-1.0611
HIP_38848	RGFFYTPKT	DEPQWSLYP	RGFFYTPKTDEPQWSLYP		SCG1	23217/367830	0.984	38848	0.816	-0.039076	-2.4667	0.3333	0	18	-1.1333	0	1	0	0	0.000000	-0.8	-3	0	0.000000	0.3333	-0.3333	2	0	1	0	2.4	0.8222	-0.8	0	1	0	0	0.5769	0.9205	1.0923	-0.5	0.3328	1.1931	-1.4231	0	0
HIP_38849	LVCGERGFF	GERGEDSS	LVCGERGFFGERGEDSS		SCG1	16916/367830	0.993	38849	0.816	0.126217	-0.5333	0.3333	-2	17	-0.5941	-2	2	1	0	0.000000	-4.5333	0	0.1	0.700000	0.4444	0.2556	0	2	0	1	0	-0.1363	-9.0667	1	0	0	0	0.4718	0.9886	0.9745	0	0.2226	0.9496	-0.2516	0.01	-0.4533
HIP_38850	QCCTSICSL	QECFETLRG	QCCTSICSLQECFETLRG		CMGA	12036/247050	0.992	38850	0.816	0.035124	0.1667	0.1667	-1	18	0.1667	-1	1	0	0	0.000000	-3.3333	-1	0.0333	0.700000	0.1111	0.5889	0	0	0	0	3.3333	0.0981	-3.3333	1	0	0	0	0.4769	1.1272	0.8723	0	0.2275	0.7609	0.0795	0.0011	-0.1111
HIP_38851	LVEALYL	NHLKATP	LVEALYLNHLKATP		IAPP	3431/45018	0.970	38851	0.816	0.149381	0.5667	0	1	14	0.3786	0	1	0	0	0.000000	-3.0667	0	0.05	0.000000	0.2222	-0.2222	0	0	0	0	0	-0.1259	-3.0667	1	0	0	0	0.3436	1.1022	0.8123	0.3333	0.1181	0.6598	0.1947	0.0025	-0.1533
HIP_38852	LVEALYLVC	EDNRDSSM	LVEALYLVCEDNRDSSM		CMGA	8729/247050	0.996	38852	0.816	0.139512	0	0	-3	17	0.0647	-3	1	1	0	0.000000	-7	-2	0.15	2.000000	0.3333	1.6667	0	0	0	0	14	0	-7	0	0	0	0	0.4103	1.0972	0.8523	0	0.1683	0.7264	0	0.0225	-1.05
HIP_38853	SLYQLENYC	LIFLISGAE	SLYQLENYCLIFLISGAE		SCG2	7197/334890	0.999	38853	0.816	-0.072367	1.4667	0	-2	18	0.7056	-2	2	0	0	0.000000	4.4667	0	-0.05	2.850000	0.1111	2.7389	0	0	0	0	0	4.017	8.9333	0	0	0	0	0.3436	1.0488	0.824	0.5	0.1181	0.6789	0.5039	0.0025	-0.2233
HIP_38854	AGSLQPLAL	VAHQLQALR	AGSLQPLALVAHQLQALR		CMGA	14688/247050	0.987	38854	0.816	0.035865	2.0333	0	2	18	0.5222	1	0	0	0	1.000000	-2.2	0	0	1.500000	0	1.5	0	0	0	0	0	3.05	0	1	0	0	0	0.2385	1.2636	0.6212	0.6667	0.0569	0.3858	0.4849	0	0
HIP_38855	LYQLENYCN	PGQGSSEDD	LYQLENYCNPGQGSSEDD		SCG2	41/334890	1.000	38855	0.816	0.070073	-1.3	0.3333	-4	18	-1.3611	-4	1	0	0	0.000000	-1.0667	0	0.15	0.625000	0.1111	0.5139	1	1	1	1	0	-0.6681	-1.0667	0	1	0	0	0.5769	0.8155	1.229	0	0.3328	1.5104	-0.75	0.0225	-0.16
HIP_38856	AEDLQVGQV	PEEKKEEEG	AEDLQVGQVPEEKKEEEG		CMGA	2285/247050	0.999	38856	0.816	-0.085575	-1.3833	0.3333	-5	18	-1.5389	-5	0	0	1	0.000000	-2.9667	-2	0.0333	0.000000	0.3333	-0.3333	1	1	1	1	5.9333	0.4611	0	1	0	0	0	0.5667	1.0433	0.9967	-0.1667	0.3211	0.9933	-0.7839	0.0011	-0.0989
HIP_38857	GAGSLQPLA	YALNSEKNF	GAGSLQPLAYALNSEKNF		SCG2	7006/334890	0.999	38857	0.816	-0.002378	1.3833	0.1667	0	18	-0.2	0	1	0	0	0.000000	0.1	0	-0.0667	1.900000	0	1.9	1	0	1	0	0	2.6283	0.1	1	0	0	0	0.3218	1.1819	0.7912	0.6667	0.1036	0.6259	0.6451	0.0044	-0.0067
HIP_38858	HLCGSHLV	AASFQRNQL	HLCGSHLVAASFQRNQL		SCG2	19270/334890	0.996	38858	0.816	0.083085	1.2667	0.1667	3	17	0.0647	1	1	1	0	1.000000	-0.6667	0	0.0667	1.500000	0	1.5	0	0	0	0	0	1.9	-0.6667	1	0	0	0	0.3051	1.1803	0.7378	0.5	0.0931	0.5444	0.3865	0.0044	-0.0444
HIP_38859	HLVEALYLV	TQRLANFLV	HLVEALYLVTQRLANFLV		IAPP	2831/45018	0.980	38859	0.816	0.200328	-0.3333	0	1	18	0.9889	0	1	0	0	0.000000	-5.1333	1	0.15	2.350000	0.1111	2.2389	0	0	0	0	-5.1333	-0.7463	-5.1333	1	0	0	0	0.4103	1.0572	0.7923	0.1667	0.1683	0.6278	-0.1368	0.0225	-0.77
HIP_38860	CGERGFFY	VDGAGKPGA	CGERGFFYVDGAGKPGA		CMGA	8833/247050	0.996	38860	0.816	0.185350	0.7667	0.1667	0	17	-0.2588	0	3	1	0	0.000000	-1.3333	-1	0.1333	0.000000	0.1111	-0.1111	0	1	0	1	1.3333	-0.0852	-4	0	1	0	0	0.3603	1.0005	0.8356	0.3333	0.1298	0.6983	0.2762	0.0178	-0.1778
HIP_38861	PGAGSLQPL	ASFQRNQLL	PGAGSLQPLASFQRNQLL		SCG2	7925/334890	0.999	38861	0.816	-0.011953	0.4167	0.3333	1	18	-0.1389	1	1	0	0	1.000000	1.7	0	-0.05	0.450000	0	0.45	1	0	1	0	0	0.1875	1.7	1	0	0	0	0.4167	1.0717	0.91	0.5	0.1736	0.8281	0.1736	0.0025	-0.085
HIP_38862	HLVEALYL	QESKGQPR	HLVEALYLQESKGQPR		SCG1	19554/367830	0.990	38862	0.816	0.247181	-0.25	0.1667	1	16	-0.7688	0	1	0	0	0.000000	-4.7	-1	0.1667	0.000000	0.3333	-0.3333	0	0	0	0	4.7	0.0833	-4.7	1	0	0	0	0.4385	1.1736	0.8045	0.1667	0.1922	0.6472	-0.1096	0.0278	-0.7833
HIP_38863	HLVEALYLV	SLGYKEIR	HLVEALYLVSLGYKEIR		CMGA	5045/247050	0.998	38863	0.816	0.179684	1.55	0.3333	1	17	0.4412	0	2	1	0	0.000000	-1.3667	0	0.15	1.400000	0	1.4	0	1	0	1	0	2.17	-2.7333	1	0	0	0	0.3885	0.9969	0.8512	0.5	0.1509	0.7245	0.6021	0.0225	-0.205
HIP_38864	VEALYLVCG	QMDKARRHP	VEALYLVCGQMDKARRHP		SCG1	20791/367830	0.988	38864	0.816	0.053616	0.2	0.1667	2	18	-0.3278	1	1	0	0	0.000000	-3.8	-1	0.0167	0.625000	0.2222	0.4028	0	1	0	1	3.8	0.0806	-3.8	0	0	0	1	0.4269	0.9855	0.899	0.1667	0.1823	0.8082	0.0854	0.0003	-0.0633
HIP_38865	GERGFFYTP	QQGDLQDT	GERGFFYTPQQGDLQDT		SCG1	10305/367830	0.998	38865	0.816	-0.029202	-1.8333	0.3333	-2	17	-1.2706	-2	3	1	0	0.000000	-1.2667	0	0.0667	0.700000	0.1111	0.5889	1	1	1	1	0	-1.0796	-3.8	0	0	0	1	0.6051	0.8569	1.1412	0	0.3662	1.3022	-1.1094	0.0044	-0.0844
HIP_38866	PLALEGSLQ	GEEEEEEEE	PLALEGSLQGEEEEEEEE		CMGA	883/247050	1.000	38866	0.816	-0.084308	-1.3167	0.3333	-9	18	-1.3889	-9	0	0	6	0.000000	-2.3	-2	0.0667	0.950000	0.4444	0.5056	0	1	0	1	4.6	-0.6656	0	0	1	0	0	0.55	1.1217	0.9433	-0.1667	0.3025	0.8899	-0.7242	0.0044	-0.1533
HIP_38867	LYQLENYCN	DQTVLTEDE	LYQLENYCNDQTVLTEDE		SCG1	8785/367830	0.998	38867	0.816	-0.021172	-1.6667	0	-5	18	-0.9667	-5	1	0	0	0.000000	-1.8	-1	0.1	1.675000	0.2222	1.4528	0	1	0	0	1.8	-2.4213	-1.8	0	1	0	0	0.5154	0.9458	1.0368	-0.1667	0.2656	1.0749	-0.859	0.01	-0.18
HIP_38868	ERGFFYTPK	GRLQNGQNG	ERGFFYTPKGRLQNGQNG		SCG2	16558/334890	0.997	38868	0.816	-0.068837	-1.2167	0.3333	2	18	-1.4556	2	2	0	0	0.000000	1.7	0	-0.0333	0.700000	0.2222	0.4778	1	1	1	1	0	-0.5813	3.4	0	0	1	0	0.5103	0.8738	1.049	-0.1667	0.2604	1.1003	-0.6208	0.0011	-0.0567

[illegible]

HIP_39337	CSLYQLENY	DMARYYSAL	CSLYQLENYDMARYYSAL		NPY	2694/49410	0.980	39337	0.816	-0.013599	-1.35	0	-1	18	-0.3278	-1	1	0
HIP_39338	GGPGAGSLQ	QEESEESNV	GGPGAGSLQQEESEESNV		SCG1	14315/367830	0.995	39338	0.816	-0.102440	-1.8333	0.1667	-4	18	-1.1278	-4	0	0
HIP_39339	CTSICSLYQ	AEEAQDPEG	CTSICSLYQAEEAQDPEG		CMGA	9414/247050	0.995	39339	0.816	-0.080191	-1.0333	0	-4	18	-0.5389	-4	1	0
HIP_39340	GIVEQCCTS	PQLRRGWRP	GIVEQCCTSPQLRRGWRP		CMGA	16054/247050	0.984	39340	0.816	-0.003096	-0.05	0.3333	2	18	-0.7167	2	0	0
HIP_39341	HLVEALYL	NSRDNPFKR	HLVEALYLNSRDNPFKR		SCG2	22859/334890	0.995	39341	0.816	0.243518	-0.4167	0.1667	2	17	-0.8	1	1	1
HIP_39342	SHLVEALY	SEEGEEDAT	SHLVEALYSEEGEEDAT		SCG1	10276/367830	0.998	39342	0.816	0.161203	-0.5833	0.1667	-5	17	-0.7529	-6	1	1
HIP_39343	GERGFFYTP	RYRGRGSEE	GERGFFYTPRYRGRGSEE		SCG1	31396/367830	0.960	39343	0.816	0.031824	-2.3167	0.1667	1	18	-1.6778	1	4	0
HIP_39344	CGERGFFY	PVGPPKNDD	CGERGFFYPVGPPKNDD		SCG2	1826/334890	1.000	39344	0.816	0.134965	1.0833	0.3333	-1	17	-1.0235	-1	3	1
HIP_39345	GERGFFYT	EEEEEEEA	GERGFFYTEEEEEEEA		CMGA	9045/247050	0.996	39345	0.816	0.053192	-1.6167	0	-7	16	-1.7438	-7	3	0
HIP_39346	GERGFFYT	EEEEEEEA	GERGFFYTEEEEEEEA		CMGA	9046/247050	0.996	39346	0.816	0.053192	-1.6167	0	-7	16	-1.7438	-7	3	0
HIP_39347	CCTSICSLY	PGWQAKREE	CCTSICSLYPGWQAKREE		CMGA	2115/247050	0.999	39347	0.816	-0.004670	-0.2	0.5	0	18	-0.4333	0	2	0
HIP_39348	HLCGSHLVE	GDERILSIL	HLCGSHLVEGDERILSIL		CMGA	8636/247050	0.996	39348	0.816	0.171625	-0.4833	0.1667	0	18	0.3944	-2	0	0
HIP_39349	QHLCGSHLV	QQGDLQDTK	QHLCGSHLVQQGDLQDTK		SCG1	13771/367830	0.996	39349	0.816	0.224887	-0.4333	0.1667	1	18	-0.8611	-1	0	0
HIP_39350	SHLVEALYL	TRLFEKPL	SHLVEALYLTRLFEKPL		SCG2	24573/334890	0.994	39350	0.816	0.184593	0.8167	0	1	17	0.2824	0	2	1
HIP_39351	DLQVGQVEL	NGERATRLF	DLQVGQVELNGERATRLF		SCG2	14166/334890	0.998	39351	0.816	0.226179	-0.4833	0.1667	-1	18	-0.3944	-1	0	0
HIP_39352	GERGFFYTP	APAEDMARY	GERGFFYTPAPAEDMARY		NPY	617/49410	0.997	39352	0.816	-0.115284	-0.2667	0.3333	-1	18	-0.7722	-1	3	0
HIP_39353	EQCCTSICS	QWKSSHFER	EQCCTSICSQWKSSHFER		SCG1	33258/367830	0.952	39353	0.816	0.089737	-0.35	0.1667	1	18	-0.8667	0	1	0
HIP_39354	LYQLENYCN	GEETGSQEN	LYQLENYCNGEETGSQEN		SCG1	6196/367830	0.999	39354	0.816	0.079778	-1.6167	0.1667	-4	18	-1.4611	-4	1	0
HIP_39355	CGERGFFY	QEAENEKD	CGERGFFYQEAENEKD		SCG1	18710/367830	0.991	39355	0.816	0.105457	-0.15	0	-3	16	-1.5687	-3	3	0
HIP_39356	HLVEALYLV	ESEESNVS	HLVEALYLVESEESNVS		SCG1	10931/367830	0.997	39356	0.816	0.163740	-0.1833	0.1667	-3	17	0.0824	-4	1	1
HIP_39357	CGERGFFY	SKSGYPKT	CGERGFFYSKSGYPKT		SCG2	21037/334890	0.996	39357	0.816	0.226640	-0.2	0.3333	2	16	-0.9625	2	3	0
HIP_39358	YLVCGERGF	LANFLVHSS	YLVCGERGFLANFLVHSS		IAPP	606/45018	0.998	39358	0.816	-0.008471	0	0.1667	1	18	0.6278	0	2	0
HIP_39359	LVCGERGFF	VPLQQKENG	LVCGERGFFVPLQQKENG		SCG2	2666/334890	1.000	39359	0.816	-0.055620	1.9333	0.3333	0	18	-0.2556	0	2	0
HIP_39360	CGERGFFY	DSLSEEDW	CGERGFFYDSLSEEDW		SCG2	7792/334890	0.999	39360	0.816	0.104391	0.6333	0.1667	-4	16	-0.9188	-4	3	0
HIP_39361	GIVEQCCTS	TGPNNQKRE	GIVEQCCTSTGPNNQKRE		SCG2	5869/334890	0.999	39361	0.816	0.014750	-0.2833	0.5	0	18	-1.1278	0	0	0
HIP_39362	LVCGERGFF	RAYFMSDTR	LVCGERGFFRAYFMSDTR		SCG1	24171/367830	0.982	39362	0.816	0.046845	0.2	0.1667	1	18	-0.0833	1	4	0
HIP_39363	GSHLVEALY	YLARMLVK	GSHLVEALYYLARMLVK		SCG2	18926/334890	0.996	39363	0.816	0.007986	1.4333	0	2	17	0.6	1	2	1
HIP_39364	QPLALEGSL	EEEDNRDSS	QPLALEGSLEEEDNRDSS		CMGA	6174/247050	0.998	39364	0.816	-0.084582	-1.3167	0.3333	-5	18	-1.3167	-5	0	0
HIP_39365	VCGERGFFY	NDKDQELG	VCGERGFFYNDKDQELG		SCG2	20341/334890	0.996	39365	0.816	0.193272	-1.1	0	-2	17	-0.9294	-2	3	1
HIP_39366	VEQCCTSIC	YGEEGAPGK	VEQCCTSICYGEEGAPGK		SCG1	6164/367830	0.999	39366	0.816	0.022271	0.1667	0.3333	-2	18	-0.3056	-2	1	0
HIP_39367	GGGPGAGSL	GGGHSRERA	GGGPGAGSLGGGHSRERA		SCG1	2574/367830	1.000	39367	0.816	0.049337	0.2333	0.8333	2	18	-0.8167	1	0	0
HIP_39368	SHLVEALYL	YDRVAQLD	SHLVEALYLYDRVAQLD		SCG1	25327/367830	0.979	39368	0.816	0.150331	-0.5	0	-1	17	0.1235	-2	2	1
HIP_39369	QCCTSICS	FPNPYNQEK	QCCTSICSFPNPYNQEK		SCG2	16424/334890	0.997	39369	0.816	0.076291	0.65	0.3333	0	17	-0.7882	0	2	1
HIP_39370	GSHLVEALY	YFPNPYNQ	GSHLVEALYYFPNPYNQ		SCG2	12627/334890	0.998	39370	0.816	0.037251	0.7	0.1667	0	17	-0.5353	-1	3	1
HIP_39371	GSHLVEALY	PGPQAEGDS	GSHLVEALYPGPQAEGDS		CMGA	1559/247050	1.000	39371	0.816	0.156205	0.1167	0.5	-2	18	-0.5056	-3	1	0
HIP_39372	QCCTSICSL	FPDFYDSEE	QCCTSICSLFPDFYDSEE		SCG1	5096/367830	0.999	39372	0.816	-0.018128	0.5333	0.3333	-4	18	-0.1167	-4	2	0
HIP_39373	EQCCTSICS	SEEPVSTHQ	EQCCTSICSSEEPVSTHQ		SCG1	9842/367830	0.998	39373	0.816	0.067403	-0.2667	0.3333	-2	18	-0.5944	-3	0	0
HIP_39374	AGSLQPLAL	MQESKAEG	AGSLQPLALMQESKAEG		CMGA	10603/247050	0.994	39374	0.816	0.147490	0.7167	0	-1	17	-0.1882	-1	0	1
HIP_39375	QCCTSICSL	QEESEEGEE	QCCTSICSLQEESEEGEE		SCG1	9012/367830	0.998	39375	0.816	0.018819	-0.8333	0.1667	-6	18	-0.8722	-6	0	0
HIP_39376	HLVEALYL	VRGYPEEKK	HLVEALYLVRGYPEEKK		CMGA	11402/247050	0.993	39376	0.816	0.192649	0.9333	0.1667	1	17	-0.5294	0	2	1
HIP_39377	LVCGERGFF	LQWKSSHF	LVCGERGFFLQWKSSHF		SCG1	18079/367830	0.992	39377	0.816	0.034456	0.7667	0.1667	2	17	0.0471	1	3	1
HIP_39378	CGERGFFYT	HHRGRGGEP	CGERGFFYTHHRGRGGEP		SCG1	36264/367830	0.939	39378	0.816	0.083958	-1.6833	0	3	18	-1.3556	1	3	0
HIP_39379	CSLYQLENY	SSEDDLQEE	CSLYQLENYSSEDDLQEE		SCG2	8688/334890	0.999	39379	0.816	-0.080923	-2.2333	0.3333	-6	18	-1.2556	-6	1	0
HIP_39380	HLCGSHLVE	EEKRFLGEG	HLCGSHLVEEEKRFLGEG		SCG1	30318/367830	0.964	39380	0.816	0.165234	-1.0667	0	0	18	-0.55	-2	0	0
HIP_39381	VCGERGFFY	HSGFEDEL	VCGERGFFYHSGFEDEL		CMGA	8341/247050	0.996	39381	0.816	0.139623	-0.0167	0.3333	-2	17	-0.3588	-3	4	1
HIP_39382	HLCGSHLVE	YPKTPGRAG	HLCGSHLVEYPKTPGRAG		SCG2	32034/334890	0.988	39382	0.816	0.115800	-0.3833	0.1667	3	18	-0.5222	1	1	0
HIP_39383	CGERGFFY	ATNTHPPA	CGERGFFYATNTHPPA		CMGA	13982/247050	0.989	39383	0.816	0.139362	0.3167	0	1	16	-0.6062	0	3	0
HIP_39384	SHLVEALYL	RDPADASEA	SHLVEALYLRDPADASEA		SCG1	16598/367830	0.993	39384	0.816	0.222084	-0.55	0.1667	-2	18	-0.1889	-3	1	0
HIP_39385	YLVCGERGF	FPNPYNQEK	YLVCGERGFFPNPYNQEK		SCG2	5788/334890	0.999	39385	0.816	-0.090022	-0.7333	0.3333	0	18	-0.9111	0	2	0
HIP_39386	LVEALYLVC	RLPYGAGR	LVEALYLVCRLPYGAGR		SCG2	20370/334890	0.996	39386	0.816	0.163219	1.3667	0.1667	1	17	0.7176	1	2	1
HIP_39387	SHLVEALY	GDSEGLSQG	SHLVEALYGDSEGLSQG		CMGA	6115/247050	0.998	39387	0.816	0.215867	-0.0667	0.3333	-2	17	-0.2765	-3	1	1
HIP_39388	LVCGERGFF	GLEPGKGR	LVCGERGFFGLEPGKGR		SCG1	12950/367830	0.996	39388	0.816	0.070069	0.85	0.3333	1	17	-0.2118	1	2	1
HIP_39389	VCGERGFF	LLLSLLGAV	VCGERGFFLLLSLLGAV		SCG1	6347/367830	0.999	39389	0.816	0.010165	2.7667	0.1667	0	17	1.6059	0	2	1

HIP_39848	SHLVEALY	KYPEINSN	SHLVEALYKYPEINSN		SCG2	21636/334890	0.995	39848	0.815	0.165102	-0.4167	0.1667	0	17	-0.2529
HIP_39849	QCCTSICSL	WNPVEEKIE	QCCTSICSLWNPVEEKIE		SCG2	6982/334890	0.999	39849	0.815	0.010117	-0.0833	0.3333	-2	18	-0.0944
HIP_39850	GAGSLQPLA	ALEYIENLR	GAGSLQPLAALEYIENLR		SCG2	8651/334890	0.999	39850	0.815	-0.061426	1.0167	0.1667	-1	18	0.1167
HIP_39851	VEALYLVC	SEDVNWGYE	VEALYLVCSEDVNWGYE		SCG1	11767/367830	0.997	39851	0.815	0.165302	0.45	0.1667	-4	17	0.1353
HIP_39852	NQHLCGSHL	TPECRQVLK	NQHLCGSHLTPECRQVLK		SCG1	20887/367830	0.988	39852	0.815	0.104768	-1	0.3333	3	18	-0.65
HIP_39853	GAGSLQPLA	VLEYLNQEK	GAGSLQPLAVLEYLNQEK		SCG2	9095/334890	0.999	39853	0.815	-0.059294	1.4167	0.1667	-1	18	-0.1611
HIP_39854	GSLQPLALE	EMKRSGQLG	GSLQPLALEEMKRSGQLG		SCG2	36419/334890	0.983	39854	0.815	0.082059	-0.5667	0	0	18	-0.4389
HIP_39855	LYLVCGERG	KEWEDSKRW	LYLVCGERGKEWEDSKRW		CMGA	16835/247050	0.982	39855	0.815	0.018368	-2.7833	0.1667	0	18	-1.1778
HIP_39856	LVEALYLVC	MVTRCIIEV	LVEALYLVCMVTRCIIEV		SCG1	13777/367830	0.996	39856	0.815	0.026681	2.65	0	-1	18	1.8
HIP_39857	HLVEALYLV	KEAVEEPSS	HLVEALYLVKEAVEEPSS		CMGA	11315/247050	0.993	39857	0.815	0.112133	0.1833	0	-2	18	0.1111
HIP_39858	GSHLVEALY	DIQGPTKAD	GSHLVEALYDIQGPTKAD		SCG1	12560/367830	0.996	39858	0.815	0.100163	0.3	0	-1	18	-0.3556
HIP_39859	ERGFFYTPK	KEIRKGESR	ERGFFYTPKKEIRKGESR		CMGA	21406/247050	0.967	39859	0.815	0.029680	-1.5167	0.1667	3	18	-1.7111
HIP_39860	TSICSLYQL	SSEEKHLEE	TSICSLYQLSSEEKHLEE		SCG1	9825/367830	0.998	39860	0.815	0.165449	-1.0167	0.3333	-2	18	-0.6333
HIP_39861	SLQPLAL	ASLGEKRD	SLQPLALASLGEKRD		SCG1	16260/367830	0.994	39861	0.815	0.083271	2.3667	0.1667	0	15	-0.2467
HIP_39862	SHLVEALY	DYNPYQGVS	SHLVEALYDYNPYQGVS		SCG2	19671/334890	0.996	39862	0.815	0.137332	-0.6667	0	-1	17	-0.4059
HIP_39863	HLVEALYL	AGSGRLQNG	HLVEALYLAGSGRLQNG		SCG2	7923/334890	0.999	39863	0.815	0.200375	1.15	0.3333	1	17	0.0882
HIP_39864	HLVEALYLV	ALAVDGAGK	HLVEALYLVALAVDGAGK		CMGA	9487/247050	0.995	39864	0.815	-0.010869	2.35	0	0	18	1.0444
HIP_39865	HLCGSHLV	YYDPLQWKS	HLCGSHLVYYDPLQWKS		SCG1	26513/367830	0.976	39865	0.815	0.076432	-0.2167	0	2	17	-0.3706
HIP_39866	CGERGFFY	RGYPEEKKE	CGERGFFYRGYPEEKKE		CMGA	15833/247050	0.985	39866	0.815	0.176015	-0.3167	0.1667	0	17	-1.6529
HIP_39867	GERGFFYTP	GGGHSRERA	GERGFFYTPGGGHSRERA		SCG1	3541/367830	1.000	39867	0.815	0.012518	-0.8	0.6667	2	18	-1.2611
HIP_39868	PGAGSLQPL	QEEVRDSKE	PGAGSLQPLQEEVRDSKE		SCG2	15382/334890	0.998	39868	0.815	-0.077411	-1.9667	0.1667	-2	18	-1.1889
HIP_39869	RGFFYTPKT	AEFPDFYDS	RGFFYTPKTAEFPDFYDS		SCG1	12859/367830	0.996	39869	0.815	0.044898	-0.85	0.1667	-1	18	-0.7944
HIP_39870	QPLALEGSL	EWEDSKRWS	QPLALEGSLEWEDSKRWS		CMGA	7348/247050	0.997	39870	0.815	-0.053814	-0.8833	0.3333	-2	18	-1.05
HIP_39871	SLYQLENYC	DQLLHYRKK	SLYQLENYCDQLLHYRKK		SCG1	32116/367830	0.957	39871	0.815	-0.018697	-0.9167	0	2	18	-1.1111
HIP_39872	SLQPLAL	FLNERKQ	SLQPLALFLNERKQ		SCG1	20138/367830	0.989	39872	0.815	0.091145	2.0833	0	1	14	-0.3571
HIP_39873	HLVEALYLV	ASFQRNQL	HLVEALYLVASFQRNQL		SCG2	13764/334890	0.998	39873	0.815	0.060484	1.75	0.1667	1	17	0.3647
HIP_39874	AGSLQPLAL	LPERDSLS	AGSLQPLALLPERDSLS		SCG2	8127/334890	0.999	39874	0.815	0.148678	1.35	0.1667	-1	17	0.0941
HIP_39875	PGAGSLQPL	DNPFKRTNE	PGAGSLQPLDNPFKRTNE		SCG2	14985/334890	0.998	39875	0.815	-0.003600	-1.65	0.3333	0	18	-1.1556
HIP_39876	GSHLVEALY	DATSEVDKR	GSHLVEALYDATSEVDKR		SCG1	12471/367830	0.997	39876	0.815	0.099945	0.3167	0	-1	18	-0.5556
HIP_39877	QCCTSICSL	PEPMQESKA	QCCTSICSLPEPMQESKA		CMGA	3729/247050	0.999	39877	0.815	0.126357	-0.2	0.5	-1	18	-0.2611
HIP_39878	QHLCGSHLV	DTPNRQYWD	QHLCGSHLVDTPNRQYWD		SCG2	12260/334890	0.998	39878	0.815	0.210038	-0.1667	0.1667	1	18	-1.1
HIP_39879	VEALYLVCG	YWDEDLLMK	VEALYLVCGYWDEDLLMK		SCG2	13070/334890	0.998	39879	0.815	0.002667	0.1	0.1667	-3	18	0.4444
HIP_39880	CGERGFFY	KELENLAA	CGERGFFYKELENLAA		SCG1	24580/367830	0.981	39880	0.815	0.142460	0.1167	0	-1	16	-0.325
HIP_39881	YLVCGERGF	SLGYKEIRK	YLVCGERGFSLGYKEIRK		CMGA	9813/247050	0.995	39881	0.815	-0.039722	0.0833	0.5	2	18	-0.3778
HIP_39882	VEALYLVCG	RNQLLQKEP	VEALYLVCGRNQLLQKEP		SCG2	23137/334890	0.995	39882	0.815	0.087341	-0.8667	0.1667	0	18	-0.0722
HIP_39883	QHLCGSHLV	GEATDGARP	QHLCGSHLVGEATDGARP		CMGA	7052/247050	0.997	39883	0.815	0.189218	0.45	0.1667	1	18	-0.4333
HIP_39884	EQCCTSICS	EEEGENYQK	EQCCTSICSEEEGENYQK		SCG1	12700/367830	0.996	39884	0.815	0.021300	-0.7167	0.1667	-4	18	-1.3278
HIP_39885	GERGFFYTP	LLRDPADAS	GERGFFYTPLLRDPADAS		SCG1	7244/367830	0.999	39885	0.815	-0.089443	-0.0833	0.1667	-1	18	-0.5278
HIP_39886	CCTSICSLY	EEDLRKESK	CCTSICSLYEEDLRKESK		SCG2	16520/334890	0.997	39886	0.815	0.005051	-1.4667	0.1667	-1	18	-0.6167
HIP_39887	CTSICSLYQ	SHQVEKRKC	CTSICSLYQSHQVEKRKC		IAPP	4019/45018	0.957	39887	0.815	0.057537	-1.4167	0.1667	3	18	-0.5778
HIP_39888	HLCGSHLVE	SNQLPKAAW	HLCGSHLVESNQLPKAAW		SCG2	22604/334890	0.995	39888	0.815	0.160585	-0.55	0.1667	2	18	-0.2
HIP_39889	CSLYQLENY	VENRQMAYE	CSLYQLENYVENRQMAYE		SCG2	19109/334890	0.996	39889	0.815	-0.168269	-1.85	0	-2	18	-0.8722
HIP_39890	VCGERGFFY	EKPLDSQS	VCGERGFFYEKPLDSQS		SCG2	17929/334890	0.997	39890	0.815	0.185928	-0.7833	0.1667	-1	17	-0.6824
HIP_39891	VCGERGFF	EEQYTPQSL	VCGERGFFEEQYTPQSL		SCG2	15537/334890	0.998	39891	0.815	0.060804	-0.8833	0.1667	-2	17	-0.6529
HIP_39892	VEALYLVCG	VFQELGKLT	VEALYLVCGVFQELGKLT		SCG2	4403/334890	1.000	39892	0.815	-0.035204	1.6333	0.1667	-1	18	0.9833
HIP_39893	QCCTSICSL	SQESGEETG	QCCTSICSLSQESGEETG		SCG1	8109/367830	0.998	39893	0.815	0.114316	-0.3833	0.3333	-3	18	-0.3944
HIP_39894	QCCTSICSL	QESGEETGS	QCCTSICSLQESGEETGS		SCG1	8110/367830	0.998	39894	0.815	0.114316	-0.3833	0.3333	-3	18	-0.3944
HIP_39895	VCGERGFF	EESWDEED	VCGERGFFEESWDEED		SCG1	10629/367830	0.997	39895	0.815	0.038556	-0.4333	0.3333	-6	16	-1.2
HIP_39896	SHLVEALY	SKEWEDSKR	SHLVEALYSKEWEDSKR		CMGA	16023/247050	0.984	39896	0.815	0.234072	-0.65	0.1667	0	17	-1.2059
HIP_39897	YLVCGERGF	KGESRSEAL	YLVCGERGFKGESRSEAL		CMGA	13194/247050	0.990	39897	0.815	0.050965	-1.65	0.3333	0	18	-0.4778
HIP_39898	GERGFFYTP	VSKRFPVGP	GERGFFYTPVSKRFPVGP		SCG2	16450/334890	0.997	39898	0.815	-0.106154	-0.6833	0.3333	2	18	-0.4667
HIP_39899	YLVCGERGF	ANQKTSYFP	YLVCGERGFANQKTSYFP		SCG2	17778/334890	0.997	39899	0.815	0.001371	-1.2167	0.1667	1	18	-0.4167
HIP_39900	GAGSLQPLA	GQNGERATR	GAGSLQPLAGQNGERATR		SCG2	7864/334890	0.999	39900	0.815	0.114259	-0.5667	0.3333	1	18	-0.8167

HIP_40359	VEALYLVC	GSGRLQNGQ	VEALYLVCGSGRLQNGQ		SCG2	9206/334890	0.999	40359	0.815	0.217197	1.4833	0.5	0	17	0.1353	0	1	1	0
HIP_40360	GAGSLQPLA	EGGGHSRER	GAGSLQPLAEGGGHSRER		SCG1	2063/367830	1.000	40360	0.815	0.137554	-0.05	0.5	1	18	-0.9278	0	0	0	1
HIP_40361	GERGFFYTP	CATQRLANF	GERGFFYTPCATQRLANF		IAPP	1388/45018	0.994	40361	0.815	-0.063646	0	0.1667	1	18	-0.35	1	3	0	0
HIP_40362	RGFFYTPKT	REEARFQDK	RGFFYTPKTREEARFQDK		SCG1	35534/367830	0.942	40362	0.815	0.003331	-2.95	0.1667	2	18	-1.6556	2	1	0	0
HIP_40363	GSLQPLALE	TQNAFLNER	GSLQPLALETQNAFLNER		SCG1	24326/367830	0.982	40363	0.815	0.128100	-0.9333	0	-1	18	-0.4111	-1	0	0	0
HIP_40364	HLVEALYLV	ADEPQWSL	HLVEALYLVADEPQWSL		SCG1	12623/367830	0.996	40364	0.815	0.092207	0.25	0	-2	17	0.3176	-3	1	1	0
HIP_40365	SHLVEALY	NGERATRLF	SHLVEALYNGERATRLF		SCG2	13917/334890	0.998	40365	0.815	0.237264	-0.5167	0.1667	1	17	-0.2294	0	1	1	0
HIP_40366	VEALYLVC	TQEEVRDSK	VEALYLVCTQEEVRDSK		SCG2	14448/334890	0.998	40366	0.815	0.187879	0.4667	0	-2	17	-0.2471	-2	1	1	0
HIP_40367	GAGSLQPLA	EYIENLRQQ	GAGSLQPLAEYIENLRQQ		SCG2	9716/334890	0.999	40367	0.815	-0.070973	0.6167	0.1667	-1	18	-0.5833	-1	1	0	0
HIP_40368	CGERGFFYT	EYNYDWWE	CGERGFFYTEYNYDWWE		SCG1	21303/367830	0.987	40368	0.815	0.047468	-1.25	0	-3	17	-1.2412	-3	5	1	0
HIP_40369	RGFFYTPKT	NLRQQAHKE	RGFFYTPKTNLRQQAHKE		SCG2	27930/334890	0.992	40369	0.815	0.063266	-1.7333	0.1667	4	18	-1.5278	3	1	0	0
HIP_40370	CCTSICSLY	DEEQKLYTD	CCTSICSLYDEEQKLYTD		SCG2	13948/334890	0.998	40370	0.815	0.007927	-1.4667	0.1667	-3	18	-0.4111	-3	1	0	0
HIP_40371	VEALYLVCG	YPGVQAPED	VEALYLVCGYPGVQAPED		SCG1	2383/367830	1.000	40371	0.815	-0.004852	0.5	0.5	-3	18	0.3167	-3	2	0	0
HIP_40372	CGERGFFY	EKPNGSVEP	CGERGFFYEKPNGSVEP		SCG2	19333/334890	0.996	40372	0.815	0.209998	-0.7833	0.1667	-1	17	-0.9765	-1	3	1	0
HIP_40373	ICSLYQLEN	IKGYPGVQA	ICSLYQLENIKGYPGVQA		SCG1	11054/367830	0.997	40373	0.815	-0.122378	-0.5	0.1667	0	18	0.0778	0	2	0	0
HIP_40374	AGSLQPLAL	VLSNALSK	AGSLQPLALVLSNALSK		SCG1	16349/367830	0.994	40374	0.815	0.064901	2.7667	0.1667	1	17	0.7824	1	0	1	0
HIP_40375	QHLCGSHLV	GRSRPQRSS	QHLCGSHLVGRSRPQRSS		SCG1	21027/367830	0.988	40375	0.815	0.241976	-0.15	0.3333	4	18	-1.0111	2	0	0	0
HIP_40376	GSHLVEALY	GQEEEEDN	GSHLVEALYGQEEEEDN		CMGA	3688/247050	0.999	40376	0.815	0.148689	-0.5167	0.1667	-5	17	-1.2059	-6	1	1	2
HIP_40377	CTSICSLYQ	WEKKPFSE	CTSICSLYQWEKKPFSE		SCG1	34411/367830	0.947	40377	0.815	0.094914	-1.55	0	0	17	-0.5353	0	2	1	0
HIP_40378	CCTSICSLY	RSRPQRSSQ	CCTSICSLYRSRPQRSSQ		SCG1	33608/367830	0.951	40378	0.815	0.119992	-1.35	0.3333	2	18	-0.6833	2	1	0	0
HIP_40379	GSLQPLAL	AEGDSEGLS	GSLQPLALAEGDSEGLS		CMGA	2944/247050	0.999	40379	0.815	0.152024	1.2167	0.1667	-3	17	-0.0235	-3	0	1	0
HIP_40380	VCGERGFF	PPKNDDTPN	VCGERGFFPPKNDDTPN		SCG2	15787/334890	0.997	40380	0.815	0.115287	-0.3167	0.5	-1	17	-1.1706	-1	2	1	0
HIP_40381	LVEALYLV	NAVEVLKRE	LVEALYLVNAVEVLKRE		IAPP	1633/45018	0.993	40381	0.815	0.001197	1.5333	0	-1	17	0.7	-1	1	1	0
HIP_40382	LVEALYLV	EERGLEPGK	LVEALYLVEERGLEPGK		SCG1	18245/367830	0.992	40382	0.815	0.154724	-0.8	0	-2	17	-0.0412	-2	1	1	0
HIP_40383	GSLQPLAL	SNQVKRVPG	GSLQPLALSNQVKRVPG		SCG2	18710/334890	0.997	40383	0.815	0.251558	0.2667	0.1667	2	17	-0.1706	2	0	1	0
HIP_40384	CTSICSLYQ	QSLATLESV	CTSICSLYQQSLATLESV		SCG2	8526/334890	0.999	40384	0.815	-0.041190	-0.25	0.1667	-1	18	0.5833	-1	1	0	0
HIP_40385	NQHLCGSHL	PGWQAKREE	NQHLCGSHLPGWQAKREE		CMGA	5611/247050	0.998	40385	0.815	0.037113	-0.5167	0.5	2	18	-1.3611	0	1	0	0
HIP_40386	HLVEALYL	SGELEQEEE	HLVEALYLSGELEQEEE		CMGA	2691/247050	0.999	40386	0.815	0.167222	0.2667	0.3333	-5	17	-0.5294	-6	1	1	1
HIP_40387	GAGSLQPLA	LITRQRYGK	GAGSLQPLALITRQRYGK		NPY	2369/49410	0.983	40387	0.815	0.004338	1.9333	0.1667	3	18	-0.3333	3	0	0	0
HIP_40388	LGGGPGAGS	EEKKEEEGS	LGGGPGAGSEEKKEEEGS		CMGA	9910/247050	0.995	40388	0.815	-0.066178	-1.7167	0.3333	-3	18	-1.4056	-3	0	0	2
HIP_40389	LVEALYLVC	TPNRQYWD	LVEALYLVCTPNRQYWD		SCG2	12360/334890	0.998	40389	0.815	0.207354	0.7833	0.1667	-1	17	-0.0118	-1	1	1	0
HIP_40390	VCGERGFF	GERGEDSSE	VCGERGFFGERGEDSSE		SCG1	15965/367830	0.994	40390	0.815	0.114020	-0.5333	0.3333	-3	17	-1.0235	-3	2	1	0
HIP_40391	VEQCCTSIC	EEEEGENYQ	VEQCCTSICEEEEGENYQ		SCG1	9657/367830	0.998	40391	0.815	-0.012006	-0.7167	0.1667	-6	18	-1.0278	-6	0	0	2
HIP_40392	CGERGFFYT	KRKCNTATC	CGERGFFYTKRKCNTATC		IAPP	4598/45018	0.941	40392	0.815	0.144860	-1.9167	0	3	18	-0.7278	3	3	0	0
HIP_40393	TSICSLYQ	YDSEEPVST	TSICSLYQYDSEEPVST		SCG1	16832/367830	0.993	40393	0.815	0.056090	-1.1	0.1667	-3	17	-0.4588	-3	2	1	0
HIP_40394	AGSLQPLAL	KEHLNQGSS	AGSLQPLALKEHLNQGSS		SCG2	27231/334890	0.992	40394	0.815	0.105422	-0.2	0	1	18	-0.3944	0	0	0	0
HIP_40395	ERGFFYTP	EEEGPTVVL	ERGFFYTPEEEGPTVVL		CMGA	12085/247050	0.992	40395	0.815	-0.031953	-2.35	0.1667	-3	17	-0.4353	-3	2	1	1
HIP_40396	HLVEALYLV	GRGSEEYRA	HLVEALYLVGRGSEEYRA		SCG1	13653/367830	0.996	40396	0.815	0.218299	0.2333	0.3333	0	18	-0.1944	-1	1	0	0
HIP_40397	HLVEALYL	EESEESNVS	HLVEALYLEESEESNVS		SCG1	10595/367830	0.997	40397	0.815	0.154672	-0.25	0.1667	-4	17	-0.3706	-5	1	1	0
HIP_40398	LYQLENYCN	GGEDWNPVE	LYQLENYCNGGEDWNPVE		SCG2	1416/334890	1.000	40398	0.815	0.098834	-1.1	0.3333	-4	18	-1.0889	-4	1	0	0
HIP_40399	CGSHLVEAL	PAEDMARYY	CGSHLVEALPAEDMARYY		NPY	1903/49410	0.988	40399	0.815	0.006722	-0.2	0.1667	-1	18	-0.1111	-2	0	0	0
HIP_40400	VCGERGFFY	ALNSEKNF	VCGERGFFYALNSEKNF		SCG2	16780/334890	0.997	40400	0.815	0.045348	1.0667	0	0	17	-0.2706	0	3	1	0
HIP_40401	SHLVEALYL	TPECRQVL	SHLVEALYLTPECRQVL		SCG1	14285/367830	0.995	40401	0.815	0.225513	0.0833	0.1667	0	17	0.3118	-1	1	1	0
HIP_40402	GSLQPLAL	YFPNPYNQ	GSLQPLALYFPNPYNQ		SCG2	17206/334890	0.997	40402	0.815	0.111310	1.55	0.1667	0	16	-0.4125	0	2	0	0
HIP_40403	SHLVEALYL	LGELFNPYY	SHLVEALYLLGELFNPYY		SCG1	4665/367830	0.999	40403	0.815	0.142814	1.0333	0.1667	-1	18	0.4111	-2	1	0	0
HIP_40404	CSLYQLENY	SEESNVSMA	CSLYQLENYSEESNVSMA		SCG1	23127/367830	0.984	40404	0.815	-0.031174	-2.6833	0.1667	-3	18	-0.4889	-3	1	0	0
HIP_40405	CSLYQLENY	EESNVSMAS	CSLYQLENYEESNVSMAS		SCG1	23128/367830	0.984	40405	0.815	-0.031174	-2.6833	0.1667	-3	18	-0.4889	-3	1	0	0
HIP_40406	CTSICSLYQ	RDSSMKLSF	CTSICSLYQRDSSMKLSF		CMGA	20239/247050	0.971	40406	0.815	0.220666	-1.6333	0.1667	1	18	0.0222	1	1	0	0

HIP_40870	QHLCGSHLV	GDLQDTKEN	QHLCGSHLVGDLQDTKEN		SCG1	7221/367830	0.999	40870	0.814	0.167490	0.7833	0.1667	0	18	-0.8611	-2	0	0	0	0.000000	-1.6333	-1
HIP_40871	SLYQLENYC	MSDTREEKR	SLYQLENYCMSDTREEKR		SCG1	17501/367830	0.993	40871	0.814	-0.056056	-0.7833	0.1667	-1	18	-1.4889	-1	1	0	0	0.000000	-0.0333	-1
HIP_40872	HLCGSHLVE	PSPEMIRAL	HLCGSHLVEPSPEMIRAL		SCG2	10841/334890	0.999	40872	0.814	0.180804	0.0833	0.5	1	18	0.1778	-1	0	0	0	0.000000	-2.8333	1
HIP_40873	GERGFFYTP	LKATPIESH	GERGFFYTPLKATPIESH		IAPP	1282/45018	0.995	40873	0.814	-0.074901	-0.3167	0.1667	1	18	-0.5778	0	3	0	0	0.000000	1.7667	1
HIP_40874	GGPGAGSLQ	TAEKRKRLG	GGPGAGSLQTAEKRKRLG		SCG1	35327/367830	0.943	40874	0.814	-0.001032	-0.4833	0.1667	3	18	-0.9833	3	0	0	0	0.000000	-0.6333	-1
HIP_40875	HLCGSHLV	FPSPEMIRA	HLCGSHLVFPSPEMIRA		SCG2	11665/334890	0.999	40875	0.814	0.196332	0.8667	0.3333	2	17	0.3353	0	1	1	0	1.000000	-1.4667	0
HIP_40876	AEDLQVGQV	NGQNGERAT	AEDLQVGQVNGQNGERAT		SCG2	4067/334890	1.000	40876	0.814	-0.038288	-1.1833	0.3333	-2	18	-1.0333	-2	0	0	0	0.000000	-2.5667	0
HIP_40877	VCGERGFF	DKARRHPQG	VCGERGFFDKARRHPQG		SCG1	31328/367830	0.960	40877	0.814	0.096815	-0.0667	0.1667	3	17	-1.1647	2	2	1	0	0.000000	-3.6	0
HIP_40878	VELGGGPGA	EEEEMAVVP	VELGGGPGAEEEEMAVVP		CMGA	4511/247050	0.999	40878	0.814	-0.077074	-1.7833	0.3333	-5	18	-0.0222	-5	0	0	3	0.000000	-3.4333	-3
HIP_40879	SHLVEALY	YNPYQGVS	SHLVEALYYNPYQGVS		SCG2	16120/334890	0.997	40879	0.814	0.116622	-0.35	0.1667	0	16	-0.2125	-1	3	0	0	0.000000	-3.5667	0
HIP_40880	QHLCGSHLV	SLIPLIFLI	QHLCGSHLVSLIPLIFLI		SCG2	15156/334890	0.998	40880	0.814	0.047650	2.05	0.1667	2	18	1.5833	0	0	0	0	0.000000	0.9	0
HIP_40881	AGSLQPLAL	DSEGLSQG	AGSLQPLALDSEGLSQG		CMGA	8474/247050	0.996	40881	0.814	0.176215	0.2667	0.1667	-2	17	-0.0235	-2	0	1	0	0.000000	-5.7333	-2
HIP_40882	LCGSHLVE	EFPDFYDSE	LCGSHLVEEFPDFYDSE		SCG1	11140/367830	0.997	40882	0.814	0.006817	0.3667	0.1667	-4	17	-0.3353	-5	2	1	0	0.000000	-2.2667	0
HIP_40883	SHLVEALY	FRGPGPQLR	SHLVEALYFRGPGPQLR		CMGA	15586/247050	0.985	40883	0.814	0.226527	0.3667	0.1667	2	17	-0.3	1	2	1	0	0.000000	-2.1333	1
HIP_40884	CSLYQLENY	ENHPQESKG	CSLYQLENYENHPQESKG		SCG1	32387/367830	0.956	40884	0.814	-0.041516	-3.0833	0	-1	18	-1.5389	-2	1	0	0	0.000000	-0.6333	0
HIP_40885	TSICSLYQL	VEEPSSKDV	TSICSLYQLVEEPSSKDV		CMGA	13094/247050	0.990	40885	0.814	-0.034419	-0.6333	0	-2	18	-0.0944	-2	1	0	0	1.000000	-0.6	-2
HIP_40886	GSHLVEALY	SPMPVSQEC	GSHLVEALYSPMPVSQEC		CMGA	1978/247050	0.999	40886	0.814	0.120972	0.6333	0.3333	-1	18	0.0667	-2	1	0	0	0.000000	-1.6	0
HIP_40887	VCGERGFFY	LLMKVLEY	VCGERGFFYLLMKVLEY		SCG2	13736/334890	0.998	40887	0.814	-0.037350	2.3	0	0	17	0.6471	0	3	1	0	0.000000	1.7333	0
HIP_40888	GSHLVEALY	DLDDISEAD	GSHLVEALYDLDDISEAD		SCG2	6528/334890	0.999	40888	0.814	0.027380	0.1833	0	-5	18	-0.2111	-6	1	0	0	0.000000	-2.5	-2
HIP_40889	LVEALYLVC	HLPERDSLS	LVEALYLVCHLPERDSLS		SCG2	11559/334890	0.999	40889	0.814	0.065298	1.5833	0.1667	-1	18	0.5	-2	1	0	0	0.000000	-3.8333	0
HIP_40890	GERGFFYTP	KELQDLALQ	GERGFFYTPKELQDLALQ		CMGA	12447/247050	0.991	40890	0.814	-0.110522	-1.2	0.1667	-1	18	-0.6389	-1	3	0	0	0.000000	0	0
HIP_40891	AGSLQPLAL	GEQEHSQQ	AGSLQPLALGEQEHSQQ		CMGA	7988/247050	0.997	40891	0.814	0.213436	0.3333	0.1667	-1	17	-0.7765	-2	0	1	0	0.000000	-5.6	-1
HIP_40892	LYLVCGERG	YPSKPDNPG	LYLVCGERGYPSKPDNPG		NPY	1674/49410	0.990	40892	0.814	-0.085861	-2.0167	0.5	0	18	-0.7778	0	1	0	0	0.000000	1.5667	0
HIP_40893	SLYQLENYC	PSSKDVMEK	SLYQLENYCPSSKDVMEK		CMGA	4376/247050	0.999	40893	0.814	0.104912	-0.9167	0.5	-1	18	-0.8722	-1	1	0	0	0.000000	-0.3	0
HIP_40894	SHLVEALYL	LYPSDSQV	SHLVEALYLLYPSDSQV		SCG1	10986/367830	0.997	40894	0.814	0.064368	1.2	0.1667	-1	17	0.3	-2	2	1	0	1.000000	-1.8	0
HIP_40895	GSLQPLAL	LPERDSLSE	GSLQPLALLPERDSLSE		SCG2	7500/334890	0.999	40895	0.814	0.146782	1.35	0.1667	-2	17	-0.2176	-2	0	1	0	1.000000	-3.5667	-1
HIP_40896	LCGSHLVEA	PPMYEENSR	LCGSHLVEAPPMYEENSR		SCG2	7390/334890	0.999	40896	0.814	0.075132	0.2	0.3333	-1	18	-0.5667	-2	1	0	0	0.000000	-1.2667	1
HIP_40897	DLQVGQVEL	LRLENVQKF	DLQVGQVELLRLENVQKF		SCG2	21887/334890	0.995	40897	0.814	0.001153	1.2667	0	-1	18	-0.15	-1	0	0	0	1.000000	-0.4667	2
HIP_40898	SHLVEALY	QELGKLTGP	SHLVEALYQELGKLTGP		SCG2	21323/334890	0.996	40898	0.814	0.187702	0.1833	0	0	17	-0.0941	-1	1	1	0	0.000000	-2.5	-1
HIP_40899	AGSLQPLAL	GEEENELT	AGSLQPLALGEEENELT		SCG1	8029/367830	0.998	40899	0.814	0.166500	0.3333	0.1667	-4	17	-0.3588	-4	0	1	1	0.000000	-5.6	-2
HIP_40900	ERGFFYTPK	HFERRDNMN	ERGFFYTPKHFERRDNMN		SCG1	21329/367830	0.987	40900	0.814	0.071250	-1.6833	0.1667	2	18	-1.7667	1	3	0	0	0.000000	0.7667	-2
HIP_40901	GSLQPLAL	KELENLAAM	GSLQPLALKELENLAAM		SCG1	22946/367830	0.984	40901	0.814	0.183487	0.9667	0	-1	17	0.3294	-1	0	1	0	0.000000	-4.2333	0
HIP_40902	EQCCTSICS	ALAEAYPSK	EQCCTSICSALAEAYPSK		NPY	1226/49410	0.993	40902	0.814	0.020239	2.2667	0.1667	-1	18	0.0444	-1	0	0	0	0.000000	0.4	0
HIP_40903	GAGSLQPLA	SPDYNPYQG	GAGSLQPLASPDYNPYQG		SCG2	5295/334890	1.000	40903	0.814	0.101327	-0.3167	0.5	-1	18	-0.7222	-1	1	0	0	0.000000	-3.3	-1
HIP_40904	CGERGFFY	EKGHPQEE	CGERGFFYEKGHPQEE		SCG1	18198/367830	0.992	40904	0.814	0.203939	-0.5833	0.1667	-1	16	-1.5688	-2	3	0	0	0.000000	-4.0333	0
HIP_40905	GSHLVEALY	AILSSTNV	GSHLVEALYAILSSTNV		IAPP	1761/45018	0.992	40905	0.814	-0.023457	2.4	0	0	17	0.7588	-1	1	1	0	0.000000	1.9333	0
HIP_40906	VELGGGPGA	EPQWSLYPS	VELGGGPGAEPQWSLYPS		SCG1	5960/367830	0.999	40906	0.814	-0.057418	-1.4667	0.5	-2	18	-0.3944	-2	1	0	1	0.000000	-2.8	-1
HIP_40907	GSLQPLAL	GEEENELTL	GSLQPLALGEEENELTL		SCG1	7473/367830	0.999	40907	0.814	0.161210	0.3333	0.1667	-4	17	-0.2412	-4	0	1	1	0.000000	-5.6	-2
HIP_40908	CGERGFFY	AGKPGAEEA	CGERGFFYAGKPGAEEA		CMGA	12432/247050	0.991	40908	0.814	0.180436	0.3	0.1667	-1	17	-0.5824	-1	3	1	0	0.000000	-2.2667	1
HIP_40909	LVEALYLVC	LDQLLHYR	LVEALYLVCLDQLLHYR		SCG1	14824/367830	0.995	40909	0.814	0.089717	1.2167	0	0	17	0.8647	-1	1	1	0	0.000000	-4.5667	-1
HIP_40910	EDLQVGQVE	QRYGKRSSP	EDLQVGQVEQRYGKRSSP		NPY	4998/49410	0.933	40910	0.814	-0.045381	-2.0167	0	0	18	-1.5	0	1	0	0	0.000000	-2.1667	2
HIP_40911	VEALYLVC	QYWDEDLLM	VEALYLVCQYWDEDLLM		SCG2	17473/334890	0.997	40911	0.814	0.068546	0.8	0	-4	17	0.5176	-4	3	1	0	0.000000	-5.4	0
HIP_40912	DLQVGQVEL	DEMKRSGQL	DLQVGQVELDEMKRSGQL		SCG2	22852/334890	0.995	40912	0.814	0.066463	-0.1	0	-2	18	-0.7111	-2	0	0	0	0.000000	-3.2	-1

HIP_41381	GSLQPLALE	QQQGDLQDT	GSLQPLALEQQQGDLQDT		SCG1	22497/367830	0.985	41381	0.813	0.103526	-1.4	0	-3	18	-0.8278	-3	0	0
HIP_41382	CGSHLVEAL	QVFLIVLSV	CGSHLVEALQVFLIVLSV		IAPP	1865/45018	0.992	41382	0.813	-0.045792	0.9333	0	0	18	1.7444	-1	1	0
HIP_41383	GAGSLQPLA	EKTHSREKS	GAGSLQPLAEKTHSREKS		SCG1	27908/367830	0.972	41383	0.813	0.112467	-0.6833	0.1667	2	18	-1.1278	1	0	0
HIP_41384	SHLVEALYL	EEDIQGPT	SHLVEALYLEEDIQGPT		SCG1	14762/367830	0.995	41384	0.813	0.136364	-0.7	0	-3	17	-0.2118	-4	1	1
HIP_41385	VCGERGFFY	VTRCIIEV	VCGERGFFYVTRCIIEV		SCG1	20914/367830	0.988	41385	0.813	0.111377	0.55	0	0	17	0.7882	0	3	1
HIP_41386	LVEALYLVC	SEDVNWGY	LVEALYLVCSEDVNWGY		SCG1	11266/367830	0.997	41386	0.813	0.143317	0.45	0.1667	-3	17	0.5647	-3	1	1
HIP_41387	EALYLVCGE	NPYNQEKVL	EALYLVCGENPYNQEKVL		SCG2	9715/334890	0.999	41387	0.813	-0.123317	-1.3	0.3333	-2	18	-0.3	-2	1	0
HIP_41388	GGPGAGSLQ	TREEKRFLG	GGPGAGSLQTREEKRFLG		SCG1	40910/367830	0.913	41388	0.813	-0.013382	-1.5333	0.1667	1	18	-0.9056	1	0	0
HIP_41389	PGAGSLQPL	IVLSVALNH	PGAGSLQPLIVLSVALNH		IAPP	680/45018	0.998	41389	0.813	0.122306	1.8667	0.1667	1	18	0.8833	0	0	0
HIP_41390	SHLVEALYL	LGALAEAYP	SHLVEALYLLGALAEAYP		NPY	616/49410	0.997	41390	0.813	0.021269	1.9167	0.1667	-1	18	0.8222	-2	1	0
HIP_41391	CGERGFFY	EVMKCIVEV	CGERGFFYEVMKCIVEV		CMGA	11113/247050	0.993	41391	0.813	0.015117	1.15	0	-1	17	0.5059	-1	3	1
HIP_41392	SLYQLENYC	QYSSHHTA	SLYQLENYCQYSSHHTA		SCG1	24321/367830	0.982	41392	0.813	0.036158	-1.3167	0.1667	1	17	-0.9118	-1	2	1
HIP_41393	HLCGSHLVE	NLLGMESAA	HLCGSHLVENLLGMESAA		SCG2	15083/334890	0.998	41393	0.813	-0.024811	1.4333	0	0	18	0.45	-2	0	0
HIP_41394	TSICSLYQL	RGYPEEKKE	TSICSLYQLRGYPEEKKE		CMGA	16339/247050	0.984	41394	0.813	0.132288	-1.2	0.1667	0	18	-1.0333	0	2	0
HIP_41395	GERGFFYTP	MDQLAKELT	GERGFFYTPMDQLAKELT		CMGA	10924/247050	0.994	41395	0.813	-0.129533	-1.45	0.1667	-1	18	-0.5889	-1	3	0
HIP_41396	ICSLYQLEN	FLNERKQAS	ICSLYQLENFLNERKQAS		SCG1	13902/367830	0.996	41396	0.813	-0.049646	-0.0167	0	0	18	-0.5167	0	2	0
HIP_41397	SHLVEALY	ENGDESHLP	SHLVEALYENGDESHLP		SCG2	10146/334890	0.999	41397	0.813	0.211145	-0.5167	0.1667	-2	17	-0.6706	-4	1	1
HIP_41398	GERGFFYTP	EDEKKELE	GERGFFYTPEDEKKELE		SCG1	20736/367830	0.988	41398	0.813	-0.010958	-2.35	0.1667	-3	17	-1.6647	-3	3	1
HIP_41399	VCGERGFF	EERGLEPG	VCGERGFFEERGLEPG		SCG1	21383/367830	0.987	41399	0.813	0.086211	-1.05	0.1667	-2	16	-0.6313	-2	2	0
HIP_41400	LVEALYLV	EEEEATNTH	LVEALYLVEEEEATNTH		CMGA	8092/247050	0.997	41400	0.813	0.107190	-0.6333	0	-4	17	-0.2059	-5	1	1
HIP_41401	GGPGAGSLQ	NNFGAILSS	GGPGAGSLQNNFGAILSS		IAPP	2017/45018	0.990	41401	0.813	-0.091119	-0.7833	0.1667	0	18	0.1111	0	1	0
HIP_41402	SICSLYQL	KWQQQGDLQ	SICSLYQLKWQQQGDLQ		SCG1	30275/367830	0.964	41402	0.813	0.032841	-1.55	0	0	17	-0.6294	0	2	1
HIP_41403	GSLQPLAL	WDEDLLMKV	GSLQPLALWDEDLLMKV		SCG2	13618/334890	0.998	41403	0.813	0.098093	0.25	0	-2	17	0.3118	-2	1	1
HIP_41404	SICSLYQLE	LIFLISGAE	SICSLYQLELIFLISGAE		SCG2	6442/334890	0.999	41404	0.813	-0.029624	1.3167	0	-2	18	1.1778	-2	2	0
HIP_41405	NQHLCGSHL	GPTKADTEK	NQHLCGSHLGPTKADTEK		SCG1	8557/367830	0.998	41405	0.813	0.127358	-0.4833	0.5	2	18	-1.1611	0	0	0
HIP_41406	GERGFFYT	QWKSSHFER	GERGFFYTQWKSSHFER		SCG1	32034/367830	0.958	41406	0.813	0.088249	-1.25	0	2	17	-1.3824	1	4	1
HIP_41407	CGERGFFY	EEKHLEEP	CGERGFFYEEKHLEEP		SCG1	21238/367830	0.988	41407	0.813	0.173421	-1.1	0	-2	16	-1.3063	-3	3	0
HIP_41408	SICSLYQLE	GPQLRRGWR	SICSLYQLEGPQLRRGWR		CMGA	12537/247050	0.991	41408	0.813	-0.011511	-1.45	0.3333	2	18	-0.6556	2	1	0
HIP_41409	VCGERGFFY	GLSVEDILN	VCGERGFFYGLSVEDILN		SCG2	649/334890	1.000	41409	0.813	0.111624	1.15	0.3333	-2	18	0.3778	-2	3	0
HIP_41410	CCTSICSLY	LEYIENLRQ	CCTSICSLYLEYIENLRQ		SCG2	12823/334890	0.998	41410	0.813	-0.112090	0.1167	0.1667	-1	18	0.25	-1	2	0
HIP_41411	ERGFFYTPK	LPEPMQESK	ERGFFYTPKLPEPMQESK		CMGA	11961/247050	0.992	41411	0.813	-0.105125	-1.25	0.3333	0	18	-1.2778	0	2	0
HIP_41412	QHLCGSHLV	EEGPTVVLN	QHLCGSHLVEEGPTVVLN		CMGA	7547/247050	0.997	41412	0.813	0.186278	-0.4333	0.1667	0	18	0.1222	-2	0	0
HIP_41413	TSICSLYQL	GRSRPDRSS	TSICSLYQLGRSRPDRSS		SCG1	21686/367830	0.987	41413	0.813	0.230029	-1.1167	0.3333	2	18	-0.7722	2	1	0
HIP_41414	CGERGFFYT	KSGEATDGA	CGERGFFYTKSGEATDGA		CMGA	10856/247050	0.994	41414	0.813	0.180853	-0.7167	0.3333	-1	18	-0.6833	-1	3	0
HIP_41415	RGFFYTPKT	KPGAEEAQD	RGFFYTPKTKPGAEEAQD		CMGA	10366/247050	0.994	41415	0.813	-0.085345	-2.0167	0.5	0	18	-1.3222	0	1	0
HIP_41416	PLALEGSLQ	NLITRQRYG	PLALEGSLQNLITRQRYG		NPY	1771/49410	0.989	41416	0.813	0.052606	0.7167	0.1667	1	18	-0.3722	1	0	0
HIP_41417	LCGSHLVEA	KNEQINDEM	LCGSHLVEAKNEQINDEM		SCG2	27018/334890	0.992	41417	0.813	0.170739	-1.4	0	-2	18	-0.5722	-3	0	0
HIP_41418	GERGFFYTP	SGRLQNGQN	GERGFFYTPSGRLQNGQN		SCG2	14307/334890	0.998	41418	0.813	-0.019047	-1.55	0.5	1	18	-1.2833	1	3	0
HIP_41419	VCGERGFF	TPQSLATLE	VCGERGFFTPQSLATLE		SCG2	10966/334890	0.999	41419	0.813	0.072396	-0.1	0.3333	-1	17	0.1235	-1	2	1
HIP_41420	GSLQPLALE	KESKDQLSD	GSLQPLALEKESKDQLSD		SCG2	24667/334890	0.994	41420	0.813	0.167251	-1.0167	0.1667	-2	18	-0.9	-2	0	0
HIP_41421	VELGGGPGA	TSRKDVKDK	VELGGGPGATSRKDVKDK		SCG1	19447/367830	0.990	41421	0.813	-0.012415	-1.0333	0.5	1	18	-0.9667	1	0	0
HIP_41422	ICSLYQLEN	MESAANQKT	ICSLYQLENMESAANQKT		SCG2	21625/334890	0.995	41422	0.813	-0.143210	-0.9333	0.1667	-1	18	-0.4667	-1	1	0
HIP_41423	TSICSLYQ	GEEEEEEEE	TSICSLYQGEEEEEEEEE		CMGA	2792/247050	0.999	41423	0.813	0.052123	-1.4	0.1667	-8	17	-1.4529	-8	1	1

HIP_41892	ERGFFYTPK	AVDGAGKPG	ERGFFYTPKAVDGAGKPG		CMGA	11740/247050	0.993	41892	0.813	0.050446	-0.6167	0.1667	1	18	-0.7056	1	2	0	0	0.000000	2.9	-2	-0.1167	1.150000	0.2222	0.9278	1	0	1	0	-5.8	-0.5721	5.8	0	0	1	0	0.4269	0.9922	0.909	0	0.1823	0.8262	-0.2633	0.0136	-0.3383
HIP_41893	LCGSHLVEA	AVVPQGLFR	LCGSHLVEAAVVPQGLFR		CMGA	10814/247050	0.994	41893	0.813	0.046266	2.1167	0	1	18	0.8333	0	0	0	0	1.000000	2.5667	1	-0.05	0.450000	0.1111	0.3389	0	0	0	0	2.5667	0.7173	0	1	0	0	0	0.2667	1.265	0.5933	0.6667	0.0711	0.352	0.5644	0.0025	-0.1283
HIP_41894	VCGERGFFY	KVAHQLQA	VCGERGFFYKVAHQLQA		CMGA	18833/247050	0.976	41894	0.813	0.085752	1.0667	0	2	17	-0.0176	1	3	1	0	0.000000	-0.7333	1	0.05	0.700000	0.2222	0.4778	0	0	0	0	-0.7333	0.5096	-2.2	0	1	0	0	0.2769	1.1588	0.6956	0.5	0.0767	0.4839	0.2954	0.0025	-0.0367
HIP_41895	LVCGERGFF	RGEDSSEE	LVCGERGFFRGEDSSEE		SCG1	16729/367830	0.993	41895	0.813	0.115403	-0.5333	0.3333	-3	17	-0.7765	-3	2	1	0	0.000000	-4.5333	0	0.1	0.700000	0.5556	0.1444	0	2	0	1	0	-0.077	-9.0667	1	0	0	0	0.4718	0.9886	0.9745	0	0.2226	0.9496	-0.2516	0.01	-0.4533
HIP_41896	LVCGERGFF	ERGEDSSE	LVCGERGFFERGEDSSE		SCG1	16730/367830	0.993	41896	0.813	0.115403	-0.5333	0.3333	-3	17	-0.7765	-3	2	1	0	0.000000	-4.5333	0	0.1	0.700000	0.5556	0.1444	0	2	0	1	0	-0.077	-9.0667	1	0	0	0	0.4718	0.9886	0.9745	0	0.2226	0.9496	-0.2516	0.01	-0.4533
HIP_41897	GSLQPLAL	GPNNQKRER	GSLQPLALGPNNQKRER		SCG2	13267/334890	0.998	41897	0.813	0.230170	0.65	0.3333	2	17	-1.2941	2	0	1	0	0.000000	-4.9667	0	0.2	0.950000	0	0.95	1	1	1	1	0	0.6175	0	1	0	0	0	0.4333	0.965	1.04	0.5	0.1878	1.0816	0.2817	0.04	-0.9933
HIP_41898	TSICSLYQ	EYNYDWWEK	TSICSLYQEYNYDWWEK		SCG1	24794/367830	0.981	41898	0.813	0.061847	-1.55	0	-2	17	-1.0941	-2	3	1	0	0.000000	-2.4333	-1	0.05	0.000000	0.2222	-0.2222	0	0	0	0	2.4333	0.3444	-7.3	0	1	0	0	0.4769	1.1055	0.9323	0	0.2275	0.8692	-0.7392	0.0025	-0.1217
HIP_41899	PGAGSLQPL	KTHWLGAAL	PGAGSLQPLKTHWLGAAL		SCG2	32060/334890	0.988	41899	0.813	0.042058	-1.5167	0.1667	2	18	0.1778	1	1	0	0	0.000000	-2.1667	1	-0.0167	0.000000	0.1111	-0.1111	1	0	1	0	-2.1667	0.1685	-2.1667	1	0	0	0	0.4936	0.9755	0.9623	-0.1667	0.2436	0.926	-0.7486	0.0003	0.0361
HIP_41900	LCGSHLVEA	NSRDNPFKR	LCGSHLVEANSRDNPFKR		SCG2	32317/334890	0.988	41900	0.813	0.209949	-1.05	0.1667	2	18	-0.8222	1	0	0	0	0.000000	-3.7667	2	0.1167	0.000000	0.3333	-0.3333	0	0	0	0	-7.5333	0.35	0	1	0	0	0	0.4385	1.0853	0.9278	0	0.1922	0.8609	-0.4604	0.0136	-0.4394
HIP_41901	LYQLENYCN	RGPGPQLRR	LYQLENYCNRGPGPQLRR		CMGA	12007/247050	0.992	41901	0.813	0.162141	-1.4667	0.3333	2	18	-1.3167	2	1	0	0	0.000000	-1.4	1	0.15	0.625000	0.2222	0.4028	1	1	1	1	-1.4	-0.5907	-1.4	0	1	0	0	0.5487	0.8008	1.2168	-0.1667	0.3011	1.4806	-0.8048	0.0225	-0.21
HIP_41902	VEALYLVC	LGEGHHRVQ	VEALYLVCLGEGHHRVQ		SCG1	12946/367830	0.996	41902	0.813	0.204378	1.7333	0.1667	1	17	0.2824	-1	1	1	0	0.000000	-3.5333	-1	0.1333	0.625000	0.1111	0.5139	0	1	0	1	3.5333	0.8907	-3.5333	0	0	0	0	0.3885	1.1153	0.7745	0.3333	0.1509	0.5998	0.6733	0.0178	-0.4711
HIP_41903	SLQPLALEG	EEDWMRIIL	SLQPLALEGEEDWMRIIL		SCG2	16122/334890	0.997	41903	0.813	-0.045708	-1.7667	0.1667	-3	18	0.1222	-3	1	0	0	0.000000	-3.4667	-2	0.0167	0.950000	0.4444	0.5056	0	1	0	1	6.9333	-0.8931	-3.4667	0	0	0	1	0.5218	1.2253	0.8545	-0.5	0.2723	0.7301	-0.9218	0.0003	-0.0578
HIP_41904	ERGFFYTPK	NAAGSGRLQ	ERGFFYTPKNAAGSGRLQ		SCG2	14087/334890	0.998	41904	0.813	0.072819	-1.0167	0.1667	2	18	-0.8889	2	2	0	0	0.000000	2.1	-1	-0.1167	0.700000	0.1111	0.5889	1	0	1	0	-2.1	-0.5987	4.2	0	0	1	0	0.4551	0.9986	1.0445	0.1667	0.2071	1.091	-0.4627	0.0136	-0.345
HIP_41905	AGSLQPLAL	EEEGPTVV	AGSLQPLALEEEGPTVV		CMGA	9474/247050	0.995	41905	0.813	0.141959	-0.1833	0	-3	17	0.2294	-3	0	1	1	0.000000	-6.6333	-3	0.15	0.450000	0.3333	0.1167	0	0	0	0	19.9	-0.0214	0	1	0	0	0	0.4	1.43	0.6467	0	0.16	0.4182	-0.0733	0.0225	-0.995
HIP_41906	GSLQPLALE	EALPDGLSV	GSLQPLALEEALPDGLSV		SCG2	15843/334890	0.997	41906	0.813	-0.053476	0.7	0	-3	18	0.4	-3	0	0	0	0.000000	0	0	0	0.950000	0.2222	0.7278	0	0	0	0	0	0.5094	0	0	0	1	0	0.3333	1.42	0.6333	0.3333	0.1111	0.4011	0.2333	0	0
HIP_41907	NQHLCGSHL	REEKRFLGE	NQHLCGSHLREEKRFLGE		SCG1	35077/367830	0.944	41907	0.813	0.134989	-1.95	0.1667	2	18	-1.2056	0	0	0	0	0.000000	-3.7667	-1	0.0833	0.625000	0.4444	0.1806	0	0	0	0	3.7667	-0.3521	0	1	0	0	0	0.4769	1.1938	0.8323	-0.5	0.2275	0.6927	-0.93	0.0069	-0.3139
HIP_41908	DLQVGQVEL	EEENELTLN	DLQVGQVELEEENELTLN		SCG1	13595/367830	0.996	41908	0.813	0.055746	-1	0	-6	18	-0.6944	-6	0	0	1	0.000000	-5	-2	0.1	0.000000	0.4444	-0.4444	0	0	0	0	10	0.4444	0	0	1	0	0	0.4667	1.4	0.66	-0.3333	0.2178	0.4356	-0.4667	0.01	-0.5
HIP_41909	LVCGERGFF	QSSQAELK	LVCGERGFFQSSQAELK		CMGA	10839/247050	0.994	41909	0.813	0.141864	0.0167	0.5	0	17	-0.1824	0	2	1	0	0.000000	-3.4333	0	0.1	0.700000	0.2222	0.4778	0	1	0	1	0	0.008	-6.8667	1	0	0	0	0.4833	0.935	1.0133	0.3333	0.2336	1.0268	0.0081	0.01	-0.3433
HIP_41910	RGFFYTPKT	QGDLQDTKE	RGFFYTPKTQGDLQDTKE		SCG1	19346/367830	0.990	41910	0.813	-0.101828	-2.2667	0.3333	0	18	-1.4556	0	1	0	0	0.000000	-0.4	-2	0.0167	0.950000	0.2222	0.7278	1	1	1	1	0.8	-1.6496	-0.4	1	0	1	0	0.5769	0.8422	1.129	-0.3333	0.3328	1.2746	-1.3077	0.0003	-0.0067
HIP_41911	HLVEALYLV	SDTREEKR	HLVEALYLVSDTREEKR		SCG1	15743/367830	0.994	41911	0.813	0.224408	0.2833	0.1667	0	17	-0.6647	-1	1	1	0	0.000000	-3.9	-1	0.1667	1.400000	0.2222	1.1778	0	0	0	0	3.9	0.3337	-3.9	1	0	0	0	0.4103	1.0055	0.829	0.1667	0.1683	0.6872	0.1162	0.0278	-0.65
HIP_41912	SICSLYQLE	EDSLEAGLP	SICSLYQLEEDSLEAGLP		CMGA	12399/247050	0.992	41912	0.813	-0.089299	-1.8333	0.1667	-4	18	0.0444	-4	1	0	0	0.000000	-1.5333	-1	0.0667	2.850000	0.3333	2.5167	0	0	0	0	1.5333	-4.6139	-1.5333	0	0	1	0	0.5051	1.2086	0.8445	-0.3333	0.2552	0.7132	-0.9261	0.0044	-0.1022
HIP_41913	LVCGERGFF	NEQINDEM	LVCGERGFFNEQINDEM		SCG2	14607/334890	0.998	41913	0.813	0.044044	-0.8833	0.1667	-3	17	-0.4294	-3	2	1	0	0.000000	-5.2333	-1	0.0667	1.825000	0.3333	1.4917	0	1	0	1	5.2333	-1.3176	-10.4667	1	0	0	0	0.4833	1.0317	0.9967	0.1667	0.2336	0.9933	-0.4269	0.0044	-0.3489
HIP_41914	LVEALYLV	KEELVARSE	LVEALYLVKEELVARSE		SCG1	18246/367830	0.992	41914	0.813	0.153503	-0.7	0	-2	17	0.4059	-2	1	1	0	0.000000	-5.8667	-1	0.15	3.050000	0.3333	2.7167	0	0	0	0	5.8667	-1.9017	-5.8667	1	0	0	0	0.4103	1.2372	0.7156	-0.1667	0.1683	0.5121	-0.2872	0.0225	-0.88
HIP_41915	VCGERGFFY	KGHPQEES	VCGERGFFYKGHPQEES		SCG1	24117/367830	0.982	41915	0.813	0.171516	-0.5333	0.1667	0	17	-1.0706	-1	3	1	0	0.000000	-3.9333	1	0.1333	0.700000	0.2222	0.4778	0	1	0	1	-3.9333	-0.2548	-11.8	0	1	0	0	0.3987	0.9941	0.9035	0	0.159	0.8162	-0.2126	0.0178	-0.5244
HIP_41916	GAGSLQPLA	ENELTLNEK	GAGSLQPLAENELTLNEK		SCG1	11551/367830	0.997	41916	0.813	-0.017574	-1.0833	0.1667	-2	18	-0.5556	-2	0	0	0	0.000000	-4.8333	-2	0.0833	2.850000	0.2222	2.6278	1	0	1	0	9.6667	-2.8468	0	1	0	0	0	0.4833	1.1683	0.9533	0	0.2336	0.9088	-0.5236	0.0069	-0.4028
HIP_41917	ERGFFYTP	WPERKLKHM	ERGFFYTPWPERKLKHM		SCG2	24012/334890	0.994	41917	0.813	-0.010499	-1.6	0.3333	3	17	-1.3059	2	3	1	0	0.000000	-0.8	-1	0	0.700000	0.3333	0.3667	2	0	1	0	0.8	-0.5867	-2.4	0	0	0	1	0.5769	0.9205	1.0923	0	0.3328	1.1931	-0.9231	0	0
HIP_41918	GSLQPLALE	GEEEEEEEE	GSLQPLALEGEEEEEEEE		CMGA	2482/247050	0.999	41918	0.813	0.031672	-0.8833	0.1667	-9	18	-1.3889	-9	0	0	6	0.000000	-3.1667	-1	0.1333	0.950000	0.4444	0.5056	0	1	0	1	3.1667	-0.4466	0	0	0	1	0	0.4833	1.2967	0.8133	-0.1667	0.2336	0.6615	-0.4269	0.0178	-0.4222
HIP_41919	GSLQPLAL	NQSSQAEL	GSLQPLALNQSSQAEL		CMGA	10527/247050	0.994	41919	0.813	0.233298	0.2667	0.1667	-1	16	-0.1938	-1	0	0	0	0.000000	-5.7333	0	0.1667	0.450000	0	0.45	0	0	0	0	0	0.12	0	1	0	0	0	0.4	1.105	0.9	0.5	0.16	0.81	0.1067	0.0278	-0.9556
HIP_41920	CGERGFFY	ISGAEAASF	CGERGFFYISGAEAASF		SCG2	5040/334890	1.000	41920	0.813	0.155000	1.2667	0.3333	-1	17	0.3059	-1	3	1	0	0.000000	-0.3333	0	0.15	0.000000	0.1111	-0.1111	0	1	0	1	0	-0.1407	-1	0	1	0	0	0.3885	0.9669	0.8795	0.5	0.1509	0.7735	0.4921	0.0225	-0.05
HIP_41921	SICSLYQLE	SDLLMREST	SICSLYQLESDLLMREST		NPY	2102/49410	0.986	41921	0.813	-0.182475	-0.6167	0.1667	-2	18	0.0222	-2	1	0	0	0.000000	0.9	0	0.0167	2.850000	0.2222	2.6278	0	0	0	0	0	-1.6205	0.9	0	0	1	0	0.4385	1.1736	0.8045	0	0.1922	0.6472	-0.2704	0.0003	0.015
HIP_41922	ERGFFYTPK	SVFQELGKL	ERGFFYTPKSVFQELGKL		SCG2	13048/334890	0.998	41922	0.813	0.000129	0	0.3333	1	18	-0.4333	1	3	0	0	0.000000	4.1333	-1	-0.1	0.700000	0.1111	0.5889	1	0	1	0	-4.1333	0	12.4	0	0	1	0	0.4551	0.9136	0.9478	0.1667	0.2071	0.8984	0	0.01	-0.4133
HIP_41923	ICSLYQLEN	GEEGAPGKW	ICSLYQLENGEEGAPGKW		SCG1	17163/367830	0.993	41923	0.813	0.054875	-1.7667	0.1667	-2	18	-0.6	-2	1	0	0	0.000000	-1.4	-1	0.0833	0.000000	0.3333	-0.3333	0	1	0	1	1.4	0.5889	-1.4	0	1	0	0	0.55	1.1667	0.9633	-0.3333	0.3025	0.928	-0.9717	0.0069	-0.1167
HIP_41924	CCTSICSLY	YWDEDLLMK	CCTSICSLYYWDEDLLMK		SCG2	16955/334890	0.997	41924	0.813	-0.029094	-0.6667	0.1667	-2	18	0.2833	-2	3	0	0	0.000000	-2.4667	0	-0.0167	2.075000	0.2222	1.8528	0	0	0	0	2.4667	-1.2352	-7.4	0	1	0	0	0.4205	1.031	0.8879	0.1667	0.1768	0.7885	-0.2803	0.0003	0.0411
HIP_41925	CSLYQLENY	ASFQRNQLL	CSLYQLENYASFQRNQLL		SCG2	13539/334890	0.998	41925	0.813	-0.078502	-0.75	0.1667	0	18	-0.4111	0	2	0	0	0.000000	4.0333	1	-0.0333	0.450000	0.1111	0.3389	0	0	0	0	4.0333	-0.2542	8.0667	0	1	0	0	0.4385	1.1086	0.9445	0.1667	0.1922	0.8921	-0.3288	0.0011	-0.1344
HIP_41926	EQCCTSICS	EAASFQRNQ	EQCCTSICSEAASFQRNQ		SCG2	14909/334890	0.998	41926	0.813	-0.036152	1.05	0.1667	-1	18	-0.5667	-1	0	0	0	0.000000	-2.0333	-1	-0.0167	0.625000	0.1111	0.5139	0	0	0	0	2.0333	0.5396	0	0	1	0	0	0.3718	1.2186	0.7728	0.3333	0.1382	0.5973	0.3904	0.0003	0.0339
HIP_41927	TSICSLYQL	MSDDYETQQ	TSICSLYQLMSDDYETQQ		SCG2	8159/334890	0.999	41927	0.813	-0.012406	-0.5667	0.1667	-3	18	-0.6056	-3	1	0	0	1.000000	-0.4667	-1	0.0667	0.475000	0.2222	0.2528	0	0	0	0	0.4667	-0.1432	-0.4667	1	0	0	0	0.4103	1.0755	0.849	0.1667	0.1683	0.7208	-0.2325	0.0044	-0.0311
HIP_41928	HLVEALYLV	QVLKTSRK	HLVEALYLVQVLKTSRK		SCG1	24537/367830	0.981	41928	0.813	0.079076	1.8667	0	3	17	0.2529	2	1	1	0	0.000000	-0.7333	0	0.05	1.400000	0.1111	1.2889	0	0	0	0	0	2.4059	-0.7333	1	0	0	0	0.3051	1.1419	0.6478	0.6667	0.0931	0.4197	0.5696	0.0025	-0.0367
HIP_41929	ERGFFYTPK	KMAHGYGEE	ERGFFYTPKKMAHGYGEE		SCG1	12741/367830	0.996	41929	0.813	0.061840	-1.0667	0.1667	1	18	-1.2667	0	2	0	0	0.000000	2	0	-0.1167	0.700000	0.2222	0.4778	1	0	1	0	0	-0.5096	4	0	0	1	0	0.4269	1.0155	0.9256	0	0.1823	0.8568	-0.4554	0.0136	-0.2333
HIP_41930	SLYQLENYC	QKGERGEDS	SLYQLENYCQKGERGEDS		SCG1	14584/367830	0.995	41930	0.813	0.126882	-1.6833	0.1667	-2	18	-1.5444	-2	1	0	0	0.000000	-1.8333	1	0.1333	0.950000	0.3333	0.6167	0	1	0	1	-1.8333	-1.0381	-1.8333	0	0	0	0	0.5321	0.8908	1.1268	-0.1667	0.2831	1.2697	-0.8956	0.0178	-0.2444
HIP_41931	VEALYLVCG	ENRQMAYEN	VEALYLVCGENRQMAYEN		SCG2	18423/334890	0.997	41931	0.813	0.096738	-0.8667	0.1667	-2	18	-0.25	-2	1	0	0	0.000000	-5.9333	0	0.0667	0.625000	0.2222	0.4028	0	1	0	1	0	-0.3491	-5.9333	0	0	0	1	0.4936	0.9638	1.019	-0.1667	0.2436	1.0383	-0.4278	0.0044	-0.3956
HIP_41932	ERGFFYTPK	KSGELEQEE	ERGFFYTPKKSGELEQEE		CMGA	7430/247050	0.997	41932	0.813	-0.130331	-1.8833	0.5	-2	18	-1.6167	-2	2	0	0	0.000000	0.3667	0	0.0333	0.700000	0.3333	0.3667	1	1	1	1	0	-0.6906	0.7333	0	0	1	0	0.5769	0.7905	1.1656	-0.3333	0.3328	1.3587	-1.0865	0.0011	0.0122
HIP_41933	GGPGAGSLQ	TGPNNQKRE	GGPGAGSLQTGPNNQKRE		SCG2	7541/334890	0.999	41933	0.813	-0.022139	-0.5333	0.5	1	18	-1.5	1	0	0	0	0.000000	-0.7333	0	0.0833	1.400000	0	1.4	1	1	1	1	0	-0.7467	0	0	1	0	0	0.5667	0.8517	1.11	0.1667	0.3211	1.2321	-0.3022	0.0069	-0.0611
HIP_41934	VCGERGFFY	LYPSDSQV	VCGERGFFYLYPSDSQV		SCG1	13340/367830	0.996	41934	0.813	0.032514	0.8667	0.1667	-1	17	-0.0765	-3	4	1	0	0.000000	-1.1333	0	0.0667	1.650000	0.1111	1.5389	1	0	1	0	0	1.3337	-4.5333	0	1	0	0	0.3603	1.0522	0.839	0.5	0.1298	0.7039	0.3122	0.0044	-0.0756
[illegible]																																														

HIP_42403	HLVEALYL	QSEESWDEE	HLVEALYLQSEESWDEE	SCG1	10016/367830	0.998	42403	0.812	0.161394
HIP_42404	LVCGERGFF	HLKATPIES	LVCGERGFFHLKATPIES	IAPP	3098/45018	0.976	42404	0.812	0.040169
HIP_42405	GSHLVEALY	GQNGERAT	GSHLVEALYGQNGERAT	SCG2	12165/334890	0.998	42405	0.812	0.204290
HIP_42406	AGSLQPLAL	SEEKHLEE	AGSLQPLALSEEKHLEE	SCG1	13087/367830	0.996	42406	0.812	0.170304
HIP_42407	HLVEALYL	EGQEEEED	HLVEALYLEGQEEEED	CMGA	4813/247050	0.998	42407	0.812	0.174202
HIP_42408	GSLQPLALE	SKENIEKNE	GSLQPLALESKENIEKNE	SCG2	24668/334890	0.994	42408	0.812	0.165060
HIP_42409	ERGFFYTPK	YQKGERGED	ERGFFYTPKYQKGERGED	SCG1	26381/367830	0.977	42409	0.812	-0.014167
HIP_42410	GERGFFYTP	QSLATLESV	GERGFFYTPQSLATLESV	SCG2	6705/334890	0.999	42410	0.812	-0.165548
HIP_42411	VELGGGPGA	KGRHHRGRG	VELGGGPGAKGRHHRGRG	SCG1	30194/367830	0.965	42411	0.812	0.052161
HIP_42412	SHLVEALY	SEEDWMRII	SHLVEALYSEEDWMRII	SCG2	15546/334890	0.998	42412	0.812	0.187257
HIP_42413	HLVEALYLV	EENSRDNP	HLVEALYLVEENSRDNP	SCG2	13788/334890	0.998	42413	0.812	0.150524
HIP_42414	GSHLVEALY	AASFQRNQ	GSHLVEALYAASFQRNQ	SCG2	14811/334890	0.998	42414	0.812	0.011702
HIP_42415	QPLALEGSL	EEEEEEEAE	QPLALEGSLEEEEEEEAE	CMGA	3899/247050	0.999	42415	0.812	-0.098117
HIP_42416	LVEALYLV	TPNRQYWDE	LVEALYLVTPNRQYWDE	SCG2	10090/334890	0.999	42416	0.812	0.192745
HIP_42417	CTSICSLYQ	HPPASLPSQ	CTSICSLYQHPPASLPSQ	CMGA	11164/247050	0.993	42417	0.812	0.041935
HIP_42418	GERGFFYTP	VSQECFETL	GERGFFYTPVSQECFETL	CMGA	3066/247050	0.999	42418	0.812	-0.194800
HIP_42419	ERGFFYTPK	QFPPMYEEN	ERGFFYTPKQFPPMYEEN	SCG2	10720/334890	0.999	42419	0.812	-0.109890
HIP_42420	GSLQPLALE	KRNLARVPK	GSLQPLALEKRNLARVPK	SCG1	38808/367830	0.925	42420	0.812	0.112017
HIP_42421	PLALEGSLQ	PQESKGQPR	PLALEGSLQPQESKGQPR	SCG1	20448/367830	0.989	42421	0.812	-0.036046
HIP_42422	GSHLVEALY	PQSAPKEN	GSHLVEALYPQSAPKEN	SCG2	11499/334890	0.999	42422	0.812	0.192081
HIP_42423	GSLQPLAL	GQVELGG	GSLQPLALGQVELGG	INSC	130/5184	0.998	42423	0.812	0.199175
HIP_42424	CSLYQLENY	ESKGQPRSQ	CSLYQLENYESKGQPRSQ	SCG1	35309/367830	0.943	42424	0.812	0.033142
HIP_42425	VEALYLVCG	GKGEQEHSQ	VEALYLVCGGKGEQEHSQ	CMGA	6005/247050	0.998	42425	0.812	0.150081
HIP_42426	YLVCGERGF	NNFGAILSS	YLVCGERGFNNFGAILSS	IAPP	1636/45018	0.993	42426	0.812	-0.005091
HIP_42427	RGFFYTPKT	EEEEGENYQ	RGFFYTPKTEEEEGENYQ	SCG1	20905/367830	0.988	42427	0.812	-0.045989
HIP_42428	LVCGERGFF	DASEAHES	LVCGERGFFDASEAHES	SCG1	11095/367830	0.997	42428	0.812	0.016560
HIP_42429	HLVEALYL	PSSKDVMEK	HLVEALYLPSSKDVMEK	CMGA	7832/247050	0.997	42429	0.812	0.220056
HIP_42430	YLVCGERGF	VFQELGKLT	YLVCGERGFVFQELGKLT	SCG2	7386/334890	0.999	42430	0.812	0.005256
HIP_42431	SHLVEALYL	TPIESHQV	SHLVEALYLTPIESHQV	IAPP	1263/45018	0.995	42431	0.812	0.168662
HIP_42432	HLVEALYLV	GQGSSEDD	HLVEALYLVGQGSSEDD	SCG2	5653/334890	0.999	42432	0.812	0.187819
HIP_42433	GSHLVEALY	GEEENELT	GSHLVEALYGEEENELT	SCG1	7694/367830	0.998	42433	0.812	0.148706
HIP_42434	LVEALYLV	SNQLPKAAW	LVEALYLVSNQLPKAAW	SCG2	15770/334890	0.997	42434	0.812	0.217483
HIP_42435	LVEALYL	TYGKRNA	LVEALYLTYGKRNA	IAPP	2383/45018	0.987	42435	0.812	0.220196
HIP_42436	GSLQPLALE	QMDKARRHP	GSLQPLALEQMDKARRHP	SCG1	40540/367830	0.915	42436	0.812	0.100420
HIP_42437	QCCTSICSL	EGENYQKGE	QCCTSICSLEGENYQKGE	SCG1	8933/367830	0.998	42437	0.812	0.109672
HIP_42438	VEALYLVCG	PKENKPYAL	VEALYLVCGPKENKPYAL	SCG2	17705/334890	0.997	42438	0.812	0.131320
HIP_42439	LGGGPGAGS	EGGGHSRER	LGGGPGAGSEGGGHSRER	SCG1	5932/367830	0.999	42439	0.812	0.002850
HIP_42440	VNQHLCGSH	GEDWNPVEE	VNQHLCGSHGEDWNPVEE	SCG2	8681/334890	0.999	42440	0.812	-0.121036
HIP_42441	HLCGSHLV	SSPDYNPYQ	HLCGSHLVSSPDYNPYQ	SCG2	11805/334890	0.998	42441	0.812	0.222770
HIP_42442	HLVEALYLV	RDNMNDNFL	HLVEALYLVRDNMNDNFL	SCG1	21265/367830	0.987	42442	0.812	0.199024
HIP_42443	CGERGFFY	NNFGAILSS	CGERGFFYNNFGAILSS	IAPP	2753/45018	0.981	42443	0.812	0.130590
HIP_42444	LVCGERGFF	YNQEKVLP	LVCGERGFFYNQEKVLP	SCG2	21414/334890	0.995	42444	0.812	0.058715
HIP_42445	HLVEALYL	AEGGGHSRE	HLVEALYLAEGGGHSRE	SCG1	8509/367830	0.998	42445	0.812	0.175636
HIP_42446	SHLVEALY	TEDEKKELE	SHLVEALYTEDEKKELE	SCG1	15833/367830	0.994	42446	0.812	0.174434
HIP_42447	HLVEALYLV	HESSSRGE	HLVEALYLVHESSSRGE	SCG1	17982/367830	0.992	42447	0.812	0.168660
HIP_42448	LYQLENYCN	PPMYEENSR	LYQLENYCNPPMYEENSR	SCG2	2414/334890	1.000	42448	0.812	-0.065149
HIP_42449	SLQPLAL	SICSLYQLE	SLQPLALSICSLYQLE	INSA	117/3024	0.970	42449	0.812	0.023391
HIP_42450	AGSLQPLAL	LIVLSVALN	AGSLQPLALLIVLSVALN	IAPP	1946/45018	0.991	42450	0.812	0.011496

HIP_42914	AGSLQPLAL	LQDLALQGA	AGSLQPLALLQDLALQGA		CMGA	8556/247050	0.996	42914	0.812	0.101074	1.0333	0	-1	18	0.7111	-1	0	0	0	1.000000	-4.2	-1	0.1	2.350000	0.1111	2.2389	0	0	0	0	4.2	2.3135	0	1	0	0	0	0.3051	1.2469	0.6745	0.5	0.0931	0.4549	0.3153	0.01	-0.42
HIP_42915	VCGERGFFY	LISDLLMR	VCGERGFFYLISDLLMR		NPY	1765/49410	0.989	42915	0.812	-0.014551	1.9667	0.1667	0	17	0.6588	0	3	1	0	0.000000	1.0667	0	0.0667	1.650000	0.2222	1.4278	0	0	0	0	0	2.808	3.2	0	1	0	0	0.3051	1.1003	0.7128	0.6667	0.0931	0.5081	0.6001	0.0044	0.0711
HIP_42916	LVEALYLVC	IPLIFLISG	LVEALYLVCIPLIFLISG		SCG2	10346/334890	0.999	42916	0.812	-0.002131	2.8667	0.1667	-1	18	2.2444	-1	1	0	0	0.000000	-1.2667	0	0.0667	4.250000	0	4.25	1	0	1	0	0	12.1833	-1.2667	0	0	0	0	0.3218	1.0553	0.7328	0.6667	0.1036	0.537	0.9225	0.0044	-0.0844
HIP_42917	HLVEALYLV	DLQEEEQIE	HLVEALYLVDLQEEEQIE		SCG2	5983/334890	0.999	42917	0.812	0.064502	0.5833	0	-5	18	-0.1444	-6	1	0	1	0.000000	-3.3	-1	0.1	1.400000	0.2222	1.1778	0	0	0	0	3.3	0.687	-3.3	1	0	0	0	0.3436	1.1355	0.7156	0.3333	0.1181	0.5121	0.2004	0.01	-0.33
HIP_42918	CGSHLVEAL	SEEKGHPQE	CGSHLVEALSEEKGHPQE		SCG1	18236/367830	0.992	42918	0.812	0.122487	-0.95	0.1667	-1	18	-0.8722	-3	0	0	0	0.000000	-3.3	-1	0.1167	1.400000	0.4444	0.9556	0	0	0	0	3.3	-0.9078	0	1	0	0	0	0.4667	1.3467	0.7633	-0.1667	0.2178	0.5827	-0.4433	0.0136	-0.385
HIP_42919	HLVEALYLV	ATDGARPQA	HLVEALYLVATDGARPQA		CMGA	10543/247050	0.994	42919	0.812	0.134134	0.7167	0	0	18	0.2667	-1	1	0	0	1.000000	-3.0333	-1	0.1	1.850000	0.1111	1.7389	0	0	0	0	3.0333	1.2462	-3.0333	1	0	0	0	0.3436	1.1138	0.739	0.3333	0.1181	0.5461	0.2462	0.01	-0.3033
HIP_42920	SHLVEALYL	DLPVDLDDI	SHLVEALYLDLPVDLDDI		SCG2	1943/334890	1.000	42920	0.812	0.093480	0.8333	0.1667	-4	18	0.5167	-5	1	0	0	0.000000	-2.5333	-1	0.1167	1.050000	0.2222	0.8278	1	0	1	0	2.5333	0.6898	-2.5333	1	0	0	0	0.3603	1.0855	0.8056	0.3333	0.1298	0.6491	0.3002	0.0136	-0.2956
HIP_42921	VCGERGFFY	LSFRARAY	VCGERGFFYLSFRARAY		CMGA	12605/247050	0.991	42921	0.812	0.023223	1.6833	0.1667	2	17	0.0765	2	4	1	0	0.000000	0.5	0	0.0667	1.650000	0.2222	1.4278	0	0	0	0	0	2.4034	2	0	1	0	0	0.3051	1.1203	0.7345	0.6667	0.0931	0.5395	0.5136	0.0044	0.0333
HIP_42922	QHLCGSHLV	DGAGKPGAE	QHLCGSHLVDGAGKPGAE		CMGA	7569/247050	0.997	42922	0.812	0.191054	0.45	0.1667	1	18	-0.3833	-1	0	0	0	0.000000	-2.3	-1	0.1333	0.950000	0.1111	0.8389	0	1	0	1	2.3	0.3775	0	1	0	0	0	0.3603	1.0588	0.829	0.1667	0.1298	0.6872	0.1621	0.0178	-0.3067
HIP_42923	LVEALYLV	VLSVALNHL	LVEALYLVVLSVALNHL		IAPP	1621/45018	0.993	42923	0.812	0.005363	2.3167	0.1667	0	17	1.8176	-1	1	1	0	1.000000	0.1667	0	0.0667	3.400000	0	3.4	0	0	0	0	0	7.8767	0.1667	1	0	0	0	0.3051	1.0903	0.6845	0.6667	0.0931	0.4685	0.7069	0.0044	0.0111
HIP_42924	SICSLYQL	EEGSANRR	SICSLYQLEEGSANRR		CMGA	11646/247050	0.993	42924	0.812	0.191698	-1.4	0.1667	0	16	-0.6688	0	1	0	0	0.000000	-2.1333	-2	0.1333	0.000000	0.2222	-0.2222	0	1	0	1	4.2667	0.3111	-2.1333	1	0	0	0	0.5218	1.1586	0.8945	-0.1667	0.2723	0.8001	-0.7305	0.0178	-0.2844
HIP_42925	SHLVEALYL	RWSKMDQL	SHLVEALYLRWSKMDQL		CMGA	19716/247050	0.973	42925	0.812	0.202221	0.0167	0.1667	1	17	-0.1647	0	2	1	0	0.000000	-4.1667	1	0.1167	0.000000	0.3333	-0.3333	0	0	0	0	-4.1667	-0.0056	-8.3333	1	0	0	0	0.3821	1.0774	0.8201	0.3333	0.146	0.6726	0.0064	0.0136	-0.4861
HIP_42926	VEALYLVC	LDKMAHGYG	VEALYLVCLDKMAHGYG		SCG1	25635/367830	0.979	42926	0.812	0.196290	1.15	0	0	17	0.6059	-1	1	1	0	0.000000	-4.7	0	0.1	1.075000	0.2222	0.8528	0	0	0	0	0	0.9807	-4.7	0	0	0	0	0.3154	1.1208	0.7035	0.1667	0.0995	0.4949	0.3627	0.01	-0.47
HIP_42927	GERGFFYTP	NYLNYGEEG	GERGFFYTPNYLNYGEEG		SCG1	5291/367830	0.999	42927	0.812	-0.099983	-0.7667	0.1667	-2	18	-1.1333	-2	4	0	0	0.000000	0.8667	0	-0.0667	0.700000	0	0.7	1	0	1	0	0	-0.5367	3.4667	0	0	0	1	0.4936	0.9022	1.059	0.1667	0.2436	1.1214	-0.3784	0.0044	-0.0578
HIP_42928	SHLVEALY	RALEYIENL	SHLVEALYRALEYIENL		SCG2	21098/334890	0.996	42928	0.812	0.057314	0.9	0	-1	17	0.1412	-2	2	1	0	0.000000	-1.0667	1	0.05	0.450000	0.3333	0.1167	0	0	0	0	-1.0667	0.105	-2.1333	0	1	0	0	0.2769	1.2572	0.689	0.5	0.0767	0.4747	0.2492	0.0025	-0.0533
HIP_42929	ICSLYQLEN	SVFQELGKL	ICSLYQLENSVFQELGKL		SCG2	10535/334890	0.999	42929	0.812	-0.070752	0.5	0.1667	-1	18	0.25	-1	2	0	0	0.000000	3.1333	1	-0.0333	0.000000	0.1111	-0.1111	0	0	0	0	3.1333	-0.0556	6.2667	0	1	0	0	0.4	1.09	0.85	0.3333	0.16	0.7225	0.2	0.0011	-0.1044
HIP_42930	LVCGERGFF	VCLGALAEA	LVCGERGFFVCLGALAEA		NPY	453/49410	0.998	42930	0.812	0.000552	2.6167	0.1667	-1	18	1.2833	-1	2	0	0	1.000000	1.7667	0	-0.0833	1.750000	0.2222	1.5278	0	1	0	1	0	3.9977	3.5333	1	0	0	0	0.3218	1.0469	0.7678	0.6667	0.1036	0.5895	0.842	0.0069	-0.1472
HIP_42931	YLVCGERGF	PAEDMARYY	YLVCGERGFPAEDMARYY		NPY	1440/49410	0.992	42931	0.812	-0.057388	-0.9	0.3333	-1	18	-0.3889	-1	1	0	0	0.000000	-0.4	-2	-0.0167	0.000000	0.4444	-0.4444	1	1	1	1	0.8	0.4	-0.4	1	0	0	0	0.4718	1.0419	0.9878	0	0.2226	0.9758	-0.4246	0.0003	0.0067
HIP_42932	LVCGERGFF	EAVPEEEGP	LVCGERGFFEAVPEEEGP		CMGA	1609/247050	1.000	42932	0.812	-0.059621	1.2833	0.1667	-4	18	-0.2389	-4	2	0	1	0.000000	-0.9	-1	-0.0333	0.700000	0.3333	0.3667	0	1	0	1	0.9	0.4706	-1.8	1	0	0	0	0.35	1.1533	0.7667	0.5	0.1225	0.5878	0.4492	0.0011	0.03
HIP_42933	SHLVEALYL	TLNEKNFFP	SHLVEALYLTLNEKNFFP		SCG1	19590/367830	0.990	42933	0.812	0.102792	0.9833	0	0	18	0.0722	-1	1	0	0	0.000000	-2.2333	0	0.1	0.000000	0.2222	-0.2222	0	0	0	0	0	-0.2185	-2.2333	1	0	0	0	0.3718	1.0703	0.8212	0.5	0.1382	0.6743	0.3656	0.01	-0.2233
HIP_42934	GAGSLQPLA	EEEEEMAVV	GAGSLQPLAEEEEEMAVV		CMGA	6120/247050	0.998	42934	0.812	-0.017769	-1.0833	0.1667	-5	18	-0.05	-5	0	0	3	0.000000	-4.8333	-3	0.0833	1.900000	0.4444	1.4556	1	0	1	0	14.5	-1.5769	0	1	0	0	0	0.4833	1.3083	0.8167	-0.1667	0.2336	0.6669	-0.5236	0.0069	-0.4028
HIP_42935	LYQLENYCN	KGERGEDSS	LYQLENYCNKGERGEDSS		SCG1	15375/367830	0.995	42935	0.812	0.139197	-1.6833	0.1667	-2	18	-1.5444	-2	1	0	0	0.000000	-1.8333	0	0.1333	0.625000	0.4444	0.1806	0	1	0	1	0	-0.3039	-1.8333	0	1	0	0	0.5321	0.9574	1.0868	-0.3333	0.2831	1.1811	-0.8956	0.0178	-0.2444
HIP_42936	LYQLENYCN	KEIRKGESR	LYQLENYCNKEIRKGESR		CMGA	27467/247050	0.935	42936	0.812	0.051581	-0.8667	0	1	18	-1.5	1	1	0	0	0.000000	-0.2	0	0.05	0.625000	0.4444	0.1806	0	0	0	0	0	-0.1565	-0.2	0	1	0	0	0.4487	1.0541	0.9151	-0.1667	0.2013	0.8375	-0.3889	0.0025	-0.01
HIP_42937	LCGSHLVEA	EEEPEYGEE	LCGSHLVEAEEEPEYGEE		SCG1	13556/367830	0.996	42937	0.812	0.046334	-1.3333	0	-6	18	-0.8944	-7	0	0	1	0.000000	-4.3333	-2	0.1	0.000000	0.4444	-0.4444	0	0	0	0	8.6667	0.5926	0	1	0	0	0	0.4667	1.425	0.6867	-0.3333	0.2178	0.4715	-0.6222	0.01	-0.4333
HIP_42938	GSHLVEALY	LSAIEAELE	GSHLVEALYLSAIEAELE		CMGA	5840/247050	0.998	42938	0.812	-0.050154	1.5167	0.1667	-3	18	0.4889	-4	1	0	0	0.000000	0.1667	0	0.0667	4.075000	0.1111	3.9639	0	0	0	0	0	6.0119	0.1667	0	1	0	0	0.3051	1.2203	0.7378	0.6667	0.0931	0.5444	0.4628	0.0044	0.0111
HIP_42939	VCGERGFFY	NVGSNTYG	VCGERGFFYNVGSNTYG		IAPP	1432/45018	0.994	42939	0.812	0.171636	0.7667	0.1667	0	17	-0.2471	0	3	1	0	0.000000	-1.3333	0	0.1333	0.700000	0.1111	0.5889	0	1	0	1	0	0.4515	-4	0	1	0	0	0.3885	0.9419	0.9445	0.5	0.1509	0.8921	0.2978	0.0178	-0.1778
HIP_42940	LYQLENYCN	PSPMPVSQE	LYQLENYCNPSPMPVSQE		CMGA	2864/247050	0.999	42940	0.812	0.070997	-1.05	0.5	-2	18	-0.7667	-2	1	0	0	0.000000	-0.5667	0	0.15	1.100000	0.1111	0.9889	2	0	1	0	0	-1.0383	-0.5667	0	1	0	0	0.5769	0.7755	1.269	0	0.3328	1.6103	-0.6058	0.0225	-0.085
HIP_42941	LQVGQVELG	EYNYDWWEK	LQVGQVELGEYNYDWWEK		SCG1	18173/367830	0.992	42941	0.812	-0.053581	-1.4	0.1667	-3	18	-0.9778	-3	2	0	0	0.000000	-2.7333	0	-0.0333	0.950000	0.2222	0.7278	0	1	0	1	0	-1.0189	-5.4667	0	0	0	1	0.5218	1.0853	0.9912	-0.1667	0.2723	0.9824	-0.7305	0.0011	0.0911
HIP_42942	GSHLVEALY	TNEIVEEQY	GSHLVEALYTNEIVEEQY		SCG2	9510/334890	0.999	42942	0.812	0.115002	-0.5667	0	-3	18	-0.3556	-4	1	0	0	0.000000	-4	-1	0.15	3.125000	0.2222	2.9028	0	0	0	0	4	-1.6449	-4	0	1	0	0	0.4385	1.1303	0.8878	0.1667	0.1922	0.7882	-0.2485	0.0225	-0.6
HIP_42943	CCTSICSLY	EEEQIEQAI	CCTSICSLYEEEQIEQAI		SCG2	13080/334890	0.998	42943	0.812	-0.002299	-1.4667	0.1667	-4	18	0.1111	-4	1	0	1	0.000000	-4.0667	-3	0.0833	2.075000	0.3333	1.7417	0	0	0	0	12.2	-2.5544	-4.0667	0	1	0	0	0.5051	1.2753	0.8045	-0.3333	0.2552	0.6472	-0.7409	0.0069	-0.3389
HIP_42944	AGSLQPLA	NRQYWDEDL	AGSLQPLANRQYWDEDL		SCG2	23357/334890	0.995	42944	0.812	0.112182	-1.25	0.1667	-2	17	-0.9118	-2	2	1	0	0.000000	-5.1667	1	0.0833	0.450000	0.1111	0.3389	1	0	1	0	-5.1667	-0.4236	-10.3333	1	0	0	0	0.4551	1.0203	1.0212	0.1667	0.2071	1.0428	-0.5689	0.0069	-0.4306
HIP_42945	TSICSLYQ	PRAYFMSDT	TSICSLYQPRAYFMSDT		SCG1	23777/367830	0.983	42945	0.812	0.062603	-0.8833	0.1667	0	17	-0.1294	0	3	1	0	0.000000	-1.1	1	0.0667	0.700000	0.1111	0.5889	1	0	1	0	-1.1	-0.5202	-3.3	0	1	0	0	0.4269	1.0788	0.9123	0.1667	0.1823	0.8323	-0.3771	0.0044	-0.0733
HIP_42946	EQCCTSICS	QEESEESNV	EQCCTSICSQEESEESNV		SCG1	10937/367830	0.997	42946	0.812	0.013574	-0.7167	0.1667	-5	18	-0.8722	-5	0	0	0	0.000000	-5.5667	-2	0.0833	0.625000	0.2222	0.4028	0	0	0	0	11.1333	-0.2887	0	0	1	0	0	0.5051	1.1719	0.8395	-0.1667	0.2552	0.7047	-0.362	0.0069	-0.4639
HIP_42947	GERGFFYTP	GKRNAVEVL	GERGFFYTPGKRNAVEVL		IAPP	3185/45018	0.974	42947	0.812	0.045539	-2.0667	0.3333	1	18	-0.4778	1	3	0	0	0.000000	-1.7333	2	0.0667	0.700000	0.2222	0.4778	1	1	1	1	-3.4667	-0.9874	-5.2	0	0	0	1	0.5487	0.8274	1.1168	-0.3333	0.3011	1.2472	-1.134	0.0044	-0.1156
HIP_42948	CCTSICSLY	KEIRKGESR	CCTSICSLYKEIRKGESR		CMGA	23982/247050	0.956	42948	0.812	0.022821	-0.2	0.1667	2	18	-0.4611	2	1	0	0	0.000000	-1.5333	0	0.0333	2.075000	0.3333	1.7417	0	0	0	0	0	-0.3483	-1.5333	0	1	0	0	0.4103	1.1222	0.7873	0	0.1683	0.6199	-0.0821	0.0011	-0.0511
HIP_42949	QCCTSICS	EPGWQAKRE	QCCTSICSEPGWQAKRE		CMGA	7765/247050	0.997	42949	0.812	0.178530	0.1167	0.5	0	17	-0.8118	0	1	1	0	0.000000	-3.9	-1	0.1333	0.000000	0.1111	-0.1111	1	1	1	1	3.9	-0.013	-3.9	0	1	0	0	0.5385	0.9136	1.0562	0	0.2899	1.1155	0.0628	0.0178	-0.52
HIP_42950	AGSLQPLAL	VDGAGKPGA	AGSLQPLALVDGAGKPGA		CMGA	5848/247050	0.998	42950	0.812	0.153236	1.6167	0.1667	0	18	0.35	0	0	0	0	1.000000	-3.0333	-1	0.1333	1.950000	0.1111	1.8389	0	1	0	1	3.0333	2.9729	0	1	0	0	0	0.3218	1.1053	0.7612	0.5	0.1036	0.5794	0.5202	0.0178	-0.4044
HIP_42951	VELGGGPGA	EEEEEEEAE	VELGGGPGAEEEEEEEAE		CMGA	3295/247050	0.999	42951	0.812	-0.091894	-1.7833	0.3333	-9	18	-1.2833	-9	0	0	6	0.000000	-3.4333	-3	0	0.000000	0.4444	-0.4444	1	1	1	1	10.3	0.7926	0	1	0	0	0	0.5667	1.175	0.9833	-0.3333	0.3211	0.9669	-1.0106	0	0
HIP_42952	CGERGFFY	PMNKGDTE	CGERGFFYPMNKGDTE		CMGA	11365/247050	0.993	42952	0.812	0.141157	0.1833	0.1667	-1	16	-1.075	-1	3	0	0	0.000000	-2.5	0	0.1167	0.700000	0.2222	0.4778	1	0	1	0	0	0.0876	-7.5	0	1	0	0	0.3885	0.9769	0.9645	0.5	0.1509	0.9302	0.0712	0.0136	-0.2917
HIP_42953	VEALYLVC	SGQLGIQEE	VEALYLVCSGQLGIQEE		SCG2	10224/334890	0.999	42953	0.812	0.214457	0.9667	0.3333	-3	17	0.4824	-3	1	1	0	0.000000	-5.0667	0	0.2	0.625000	0	0.625	0	1	0	1	0	0.6042	-5.0667	0	1	0	0	0.4551	0.9653	0.9312	0.3333	0.2071	0.867	0.44	0.04	-1.0133
HIP_42954	GERGFFYT	SSQETDKL	GERGFFYTSSQETDKL		SCG2	16346/334890	0.997	42954	0.812	0.172167	-0.7167	0.3333	-1	16	-1.1312	-1	3	0	0	0.000000	-1.9667	0	0.1333	0.000000	0.1111	-0.1111	0	0	0	0	0	0.0796	-5.9	0	1	0	0	0.5051	0.9603	0.9745	0.1667	0.2552	0.9496	-0.362	0.0178	-0.2622
HIP_42955	VEALYLVCG	EEPEYGEEI	VEALYLVCGEEPEYGEEI		SCG1	5181/367830	0.999	42955	0.812	0.009396	-0.3833	0.3333	-6	18	-0.0667	-6	1	0	0	0.000000	-4.9667	-2	0.0833	0.625000	0.3333	0.2917	1	1	1	1	9.9333	-0.1118	-4.9667	0	0	0	1	0.5385	1.0286	0.9845	-0.1667	0.2899	0.9692	-0.2064	0.0069	-0.4139
HIP_42956	TSICSLYQ	WNPVEEKE	TSICSLYQWNPVEEKE		SCG2	12171/334890	0.998	42956	0.812	0.043533	-1.1667	0.1667	-2	17	-0.4706	-2	2	1	0	0.000000	-1.6667	0	0.0667	0.000000	0.1111	-0.1111	1	0	1	0	0	0.1296	-3.3333	0	1	0	0	0.4936	0.9488	1.0623	0.3333	0.2436	1.1285	-0.5759	0.0044	-0.1111
[illegible]	[illegible]	[illegible]	[illegible]		[illegible]	[illegible]	[illegible]	[illegible]	[illegible]	[illegible]	[illegible]	[illegible]	[illegible]	[illegible]	[illegible]	[illegible]	[illegible]	[illegible]	[illegible]	[illegible]	[illegible]	[illegible]	[illegible]	[illegible]	[illegible]	[illegible]	[illegible]	[illegible]	[illegible]	[illegible]	[illegible]	[illegible]	[illegible]	[illegible]	[illegible]	[illegible]	[illegible]	[illegible]	[illegible]	[illegible]	[illegible]	[illegible]	[illegible]	[illegible]	[illegible]	[illegible]

HIP_43425	GSHLVEALY	LEKVAHQLQ	GSHLVEALYLEKVAHQLQ		CMGA	14212/247050	0.989	43425	0.811	0.065311	0.1167	0	1	18	0.0222	-1	1	0	0	0.000000	-2.6333	0	0.1	4.000000	0.3333	3.6667	0	0	0	0	0	0.4278	-2.6333	0	1	0	0	0.3436	1.2672	0.7023	0.1667	0.1181	0.4932	0.0401	0.01	-0.2633
HIP_43426	GGPGAGSLQ	PQESKGQPR	GGPGAGSLQPQESKGQPR		SCG1	24029/367830	0.982	43426	0.811	-0.037342	-1.5167	0.3333	1	18	-1.4	1	0	0	0	0.000000	-2.7	-1	0.05	1.400000	0.1111	1.2889	1	0	1	0	2.7	-1.9548	0	0	1	0	0	0.55	1.0667	0.9867	0	0.3025	0.9735	-0.8342	0.0025	-0.135
HIP_43427	CSLYQLENY	EATNTHPPA	CSLYQLENYEATNTHPPA		CMGA	17436/247050	0.981	43427	0.811	-0.097006	-1.7833	0	-1	18	-0.8333	-2	1	0	0	0.000000	1.9667	0	0	0.000000	0.2222	-0.2222	0	0	0	0	0	0.3963	1.9667	0	1	0	0	0.5051	1.1653	0.9278	-0.1667	0.2552	0.8609	-0.9008	0	0
HIP_43428	DLQVGQVEL	RLQNGQNGE	DLQVGQVELRLQNGQNGE		SCG2	29749/334890	0.990	43428	0.811	0.067632	0.05	0	-2	18	-0.9667	-2	0	0	0	0.000000	-2.9	2	0.05	0.000000	0.2222	-0.2222	0	0	0	0	-5.8	-0.0111	0	1	0	0	0	0.3718	1.2169	0.6878	0.1667	0.1382	0.4731	0.0186	0.0025	-0.145
HIP_43429	VEALYLVC	ELFNPYYDP	VEALYLVCELFNPYYDP		SCG1	14904/367830	0.995	43429	0.811	0.039439	2.2667	0	-3	17	0.3412	-3	2	1	0	0.000000	-2.4667	-1	0.05	1.575000	0.1111	1.4639	0	0	0	0	2.4667	3.3181	-4.9333	0	0	0	0	0.3051	1.2319	0.6245	0.5	0.0931	0.39	0.6916	0.0025	-0.1233
HIP_43430	GERGFFYTP	LQIPPEDLI	GERGFFYTPLQIPPEDLI		SCG2	6199/334890	0.999	43430	0.811	-0.097070	0.2	0.1667	-2	18	-0.2167	-2	3	0	0	0.000000	2.8	0	-0.0667	1.650000	0	1.65	1	0	1	0	0	0.33	8.4	0	0	0	1	0.4551	0.9853	0.8895	0.3333	0.2071	0.7912	0.091	0.0044	-0.1867
HIP_43431	CTSICSLY	GPNNQKRER	CTSICSLYGPNNQKRER		SCG2	13087/334890	0.998	43431	0.811	0.163352	-0.6333	0.5	2	17	-1.1294	2	1	1	0	0.000000	-2.4	0	0.1333	0.625000	0	0.625	1	1	1	1	0	-0.3958	-2.4	0	1	0	0	0.5385	0.8103	1.1978	0.1667	0.2899	1.4348	-0.341	0.0178	-0.32
HIP_43432	SLYQLENYC	SSQGGSLPS	SLYQLENYCSSQGGSLPS		SCG1	11830/367830	0.997	43432	0.811	0.125786	-1.2333	0.3333	-1	18	-0.5056	-1	1	0	0	0.000000	-0.9333	0	0.1333	0.950000	0.1111	0.8389	0	0	0	0	0	-1.0346	-0.9333	0	0	0	0	0.5436	0.9038	1.1256	0	0.2955	1.2671	-0.6704	0.0178	-0.1244
HIP_43433	DLQVGQVEL	RMLVKYPEI	DLQVGQVELRMLVKYPEI		SCG2	24130/334890	0.994	43433	0.811	0.000254	0.95	0	-1	18	0.0667	-1	0	0	0	0.000000	-1.1	2	0	1.050000	0.2222	0.8278	0	0	0	0	-2.2	0.7864	0	1	0	0	0	0.3051	1.2319	0.6245	0.3333	0.0931	0.39	0.2899	0	0
HIP_43434	ERGFFYTP	QWPERKLK	ERGFFYTPQWPERKLK		SCG2	20954/334890	0.996	43434	0.811	-0.022292	-1.6	0.3333	2	16	-1.525	2	4	0	0	0.000000	-0.8	0	0	0.700000	0.1111	0.5889	2	0	1	0	0	-0.9422	-3.2	0	0	0	1	0.5769	0.8538	1.1323	0.1667	0.3328	1.2821	-0.9231	0	0
HIP_43435	LVCGERGFF	IRALEYIEN	LVCGERGFFIRALEYIEN		SCG2	10591/334890	0.999	43435	0.811	-0.034308	1.1667	0.1667	-1	18	0.3111	-1	2	0	0	1.000000	-1.1333	1	-0.0333	2.775000	0.3333	2.4417	0	1	0	1	-1.1333	2.8486	-2.2667	1	0	0	0	0.3218	1.0753	0.7895	0.5	0.1036	0.6233	0.3754	0.0011	0.0378
HIP_43436	AGSLQPLAL	GVQAPEDLE	AGSLQPLALGVQAPEDLE		SCG1	3520/367830	1.000	43436	0.811	0.144194	1.6167	0.1667	-3	18	0.1389	-3	0	0	0	0.000000	-3.0333	0	0.1333	0.900000	0	0.9	0	1	0	1	0	1.455	0	1	0	0	0	0.35	1.12	0.7733	0.6667	0.1225	0.598	0.5658	0.0178	-0.4044
HIP_43437	ICSLYQLEN	KRVPGQGSS	ICSLYQLENKRVPGQGSS		SCG2	39334/334890	0.978	43437	0.811	0.046633	-1.2333	0	1	18	-0.5389	1	1	0	0	0.000000	-0.3333	3	0	0.000000	0.3333	-0.3333	0	0	0	0	-1	0.4111	-0.3333	0	1	0	0	0.4103	1.0972	0.8523	-0.1667	0.1683	0.7264	-0.506	0	0
HIP_43438	CGERGFFY	MAHGYGEES	CGERGFFYMAHGYGEES		SCG1	22134/367830	0.986	43438	0.811	0.024457	0.8	0	-1	17	-0.6706	-2	4	1	0	0.000000	-1.2667	0	0	0.450000	0	0.45	0	0	0	0	0	0.36	-5.0667	0	1	0	0	0.2769	1.1822	0.7123	0.5	0.0767	0.5074	0.2215	0	0
HIP_43439	CCTSICSLY	EGQSEGLSQ	CCTSICSLYEGQSEGLSQ		CMGA	4205/247050	0.999	43439	0.811	0.047141	-0.95	0.3333	-3	18	-0.0222	-3	1	0	0	0.000000	-3.0333	-2	0.1167	2.075000	0.2222	1.8528	0	1	0	1	6.0667	-1.7601	-3.0333	0	1	0	0	0.4936	1.0255	0.959	-0.1667	0.2436	0.9196	-0.4689	0.0136	-0.3539
HIP_43440	LYQLENYCN	GEEDATSEV	LYQLENYCNGEEDATSEV		SCG1	6075/367830	0.999	43440	0.811	0.066917	-1.6167	0.1667	-5	18	-0.9111	-5	1	0	0	0.000000	-1.7	-2	0.1333	0.625000	0.4444	0.1806	0	1	0	1	3.4	-0.2919	-1.7	0	1	0	0	0.5603	1.0388	1.059	-0.3333	0.3139	1.1214	-0.9057	0.0178	-0.2267
HIP_43441	HLVEALYLV	LNDKDQELG	HLVEALYLVLNDKDQELG		SCG2	9191/334890	0.999	43441	0.811	0.092015	0.5833	0	-2	18	-0.0333	-3	1	0	0	1.000000	-3.3	-1	0.1	2.350000	0.2222	2.1278	0	0	0	0	3.3	1.2412	-3.3	1	0	0	0	0.3436	1.0622	0.8123	0.3333	0.1181	0.6598	0.2004	0.01	-0.33
HIP_43442	LVEALYLV	TGPNNQKRE	LVEALYLVTGPNNQKRE		SCG2	8960/334890	0.999	43442	0.811	0.202882	0.6667	0.3333	0	17	-0.4882	0	1	1	0	0.000000	-3.1333	0	0.2	2.000000	0	2	1	1	1	1	0	1.3333	-3.1333	1	0	0	0	0.4718	0.8803	0.9812	0.3333	0.2226	0.9627	0.3145	0.04	-0.6267
HIP_43443	SICSLYQLE	RKLKHMQFP	SICSLYQLERKLKHMQFP		SCG2	39070/334890	0.979	43443	0.811	-0.003915	-1.3	0	3	18	-0.4111	2	1	0	0	0.000000	-0.4667	3	0	1.900000	0.4444	1.4556	0	0	0	0	-1.4	-1.8922	-0.4667	0	0	1	0	0.4103	1.2105	0.7556	-0.1667	0.1683	0.571	-0.5333	0	0
HIP_43444	VELGGGPGA	QTQEEVRDS	VELGGGPGAQTQEEVRDS		SCG2	3183/334890	1.000	43444	0.811	-0.120427	-1.3167	0.3333	-3	18	-0.9	-3	0	0	1	0.000000	-2.5	0	0	0.000000	0.1111	-0.1111	1	1	1	1	0	0.1463	0	1	0	0	0	0.5667	0.9283	1.1	0.1667	0.3211	1.21	-0.7461	0	0
HIP_43445	GERGFFYTP	GAALSLIPL	GERGFFYTPGAALSLIPL		SCG2	5556/334890	0.999	43445	0.811	-0.122962	-0.0667	0.3333	0	18	0.55	0	3	0	0	0.000000	2.2667	0	-0.0333	1.650000	0	1.65	1	1	1	1	0	-0.11	6.8	0	0	0	1	0.4718	0.9703	1.0345	0.3333	0.2226	1.0702	-0.0315	0.0011	-0.0756
HIP_43446	VEALYLVC	GIQEEDLRK	VEALYLVCGIQEEDLRK		SCG2	7741/334890	0.999	43446	0.811	0.165327	1.85	0.1667	-2	17	0.0588	-2	1	1	0	0.000000	-3.3	0	0.1333	1.750000	0.2222	1.5278	0	1	0	1	0	2.8264	-3.3	0	0	0	0	0.3885	1.0119	0.8095	0.5	0.1509	0.6553	0.7187	0.0178	-0.44
HIP_43447	EQCCTSICS	SRSNQLPKA	EQCCTSICSSRSNQLPKA		SCG2	23356/334890	0.995	43447	0.811	0.200896	0.0167	0.5	1	18	-0.5722	1	0	0	0	0.000000	-4.1	1	0.1167	0.625000	0.1111	0.5139	0	0	0	0	-4.1	0.0086	0	0	1	0	0	0.4769	0.9205	0.9806	0	0.2275	0.9617	0.0079	0.0136	-0.4783
HIP_43448	PLALEGSLQ	GLFRGGKSG	PLALEGSLQGLFRGGKSG		CMGA	5581/247050	0.998	43448	0.811	-0.051337	0.95	0.3333	1	18	-0.0444	1	1	0	0	0.000000	2.2333	0	-0.0333	0.950000	0.2222	0.7278	0	1	0	1	0	0.6914	2.2333	0	1	0	0	0.4167	1.035	0.88	0.5	0.1736	0.7744	0.3958	0.0011	-0.0744
HIP_43449	VEALYLVCG	QGSSQETDK	VEALYLVCGQGSSQETDK		SCG2	3407/334890	1.000	43449	0.811	0.122446	0.2667	0.5	-2	18	-0.3056	-2	1	0	0	0.000000	-3.6667	0	0.1167	0.625000	0	0.625	0	2	0	1	0	0.1667	-3.6667	0	0	0	1	0.5385	0.8319	1.0978	0.1667	0.2899	1.2052	0.1436	0.0136	-0.4278
HIP_43450	VEQCCTSIC	SALRHYINL	VEQCCTSICSALRHYINL		NPY	2033/49410	0.987	43450	0.811	-0.015912	1.8333	0.3333	1	18	0.4611	0	0	0	0	0.000000	-0.4667	0	0	1.750000	0.1111	1.6389	0	0	0	0	0	3.0046	0	0	0	0	0	0.3718	1.0753	0.8228	0.5	0.1382	0.677	0.6816	0	0
HIP_43451	LYQLENYCN	ESKAEGNNQ	LYQLENYCNESKAEGNNQ		CMGA	13976/247050	0.989	43451	0.811	0.112754	-1.75	0.1667	-2	18	-1.5167	-2	1	0	0	0.000000	-1.9667	0	0.1167	1.075000	0.3333	0.7417	0	0	0	0	0	-1.2979	-1.9667	0	1	0	0	0.5154	1.0074	1.0368	-0.3333	0.2656	1.0749	-0.9019	0.0136	-0.2294
HIP_43452	HLCGSHLVE	PVDNRNHNE	HLCGSHLVEPVDNRNHNE		SCG1	18184/367830	0.992	43452	0.811	0.048298	0.6	0.1667	1	18	-1.0778	-2	0	0	0	0.000000	-1.8	0	0.0667	1.050000	0.2222	0.8278	1	0	1	0	0	0.4967	0	0	0	1	0	0.3885	1.0869	0.7778	0.1667	0.1509	0.605	0.2331	0.0044	-0.12
HIP_43453	LYQLENYCN	RGEAGAPGE	LYQLENYCNRGEAGAPGE		SCG1	14327/367830	0.995	43453	0.811	0.123437	-1.7833	0.1667	-2	18	-0.9556	-2	1	0	0	0.000000	-2.0333	0	0.1333	1.075000	0.3333	0.7417	0	1	0	1	0	-1.3226	-2.0333	0	1	0	0	0.5321	0.9574	1.0868	-0.3333	0.2831	1.1811	-0.9488	0.0178	-0.2711
HIP_43454	ERGFFYTPK	AEKSGEATD	ERGFFYTPKAEKSGEATD		CMGA	14802/247050	0.987	43454	0.811	-0.071919	-1.9667	0.1667	-1	18	-1.2778	-1	2	0	0	0.000000	0.2	-1	-0.0667	1.150000	0.3333	0.8167	1	0	1	0	-0.2	-1.6061	0.4	0	0	1	0	0.4936	1.0672	0.949	-0.3333	0.2436	0.9006	-0.9707	0.0044	-0.0133
HIP_43455	QHLCGSHLV	KIAEKFSQR	QHLCGSHLVKIAEKFSQR		SCG1	34208/367830	0.948	43455	0.811	0.098490	1.2	0	4	18	-0.4333	2	0	0	0	0.000000	-0.8	1	0.05	0.950000	0.2222	0.7278	0	0	0	0	-0.8	0.8733	0	1	0	0	0	0.2769	1.1555	0.6573	0.3333	0.0767	0.4321	0.3323	0.0025	-0.04
HIP_43456	GERGFFYTP	SGEATDGAR	GERGFFYTPSGEATDGAR		CMGA	5013/247050	0.998	43456	0.811	-0.055718	-1.3833	0.5	-1	18	-0.9444	-1	3	0	0	0.000000	-0.3667	-1	0.0833	1.150000	0.1111	1.0389	1	1	1	1	0.3667	-1.4371	-1.1	0	0	0	1	0.6051	0.8719	1.1378	-0.1667	0.3662	1.2946	-0.8371	0.0069	-0.0306
HIP_43457	GSHLVEALY	LRGDERILS	GSHLVEALYLRGDERILS		CMGA	7646/247050	0.997	43457	0.811	0.125096	0.5333	0.1667	0	18	-0.0389	-1	1	0	0	0.000000	-1.8	1	0.1333	2.950000	0.3333	2.6167	0	1	0	1	-1.8	1.3956	-1.8	0	1	0	0	0.3603	1.0988	0.829	0.3333	0.1298	0.6872	0.1921	0.0178	-0.24
HIP_43458	DLQVGQVEL	GERGFFYT	DLQVGQVELGERGFFYT		INSB	307/4968	0.947	43458	0.811	0.212346	-0.65	0.1667	-2	17	-0.2118	-2	0	1	0	0.000000	-4.3	1	0.1333	0.000000	0.3333	-0.3333	0	1	0	1	-4.3	0.2167	0	1	0	0	0	0.4551	1.1503	0.8145	-0.1667	0.2071	0.6634	-0.2958	0.0178	-0.5733
HIP_43459	LYQLENYCN	NYLNYGEEG	LYQLENYCNNYLNYGEEG		SCG1	8953/367830	0.998	43459	0.811	-0.153596	-0.55	0	-3	18	-1.1167	-3	2	0	0	0.000000	0.4333	0	0	0.625000	0.1111	0.5139	0	0	0	0	0	-0.2826	0.8667	0	1	0	0	0.4487	0.9508	1.0568	0.1667	0.2013	1.1168	-0.2468	0	0
HIP_43460	LQPLALEGS	GPPKNDQTP	LQPLALEGSGPPKNDQTP		SCG2	4477/334890	1.000	43460	0.811	-0.124534	-1.3833	0.8333	-2	18	-0.9389	-2	0	0	0	0.000000	0.3667	1	0.0167	0.450000	0.2222	0.2278	2	2	1	1	0.3667	-0.3151	0	0	1	0	0	0.6667	0.7417	1.33	-0.1667	0.4444	1.7689	-0.9222	0.0003	0.0061
HIP_43461	SLQPLAL	HYINLIT	SLQPLALHYINLIT		NPY	3445/49410	0.969	43461	0.811	0.070888	1.5667	0	1	14	0.8143	0	1	0	0	0.000000	-3.1333	0	0	0.450000	0	0.45	0	0	0	0	0	0.705	-3.1333	1	0	0	0	0.2769	1.1955	0.6573	0.5	0.0767	0.4321	0.4338	0	0
HIP_43462	RGFFYTPKT	PLIFLISGA	RGFFYTPKTPLIFLISGA		SCG2	13403/334890	0.998	43462	0.811	0.014666	0.0833	0.3333	2	18	0.6056	2	2	0	0	0.000000	4.3	-1	-0.1	0.700000	0.1111	0.5889	2	0	1	0	-4.3	0.0491	8.6	0	1	0	0	0.4718	0.8953	0.9795	0.1667	0.2226	0.9594	0.0393	0.01	-0.43
HIP_43463	GPGAGSLQP	QRNQLLQKE	GPGAGSLQPQRNQLLQKE		SCG2	32711/334890	0.987	43463	0.811	0.044267	-2.1333	0.1667	1	18	-1.1889	1	0	0	0	0.000000	-3.4	1	0.0333	0.000000	0.1111	-0.1111	1	0	1	0	-3.4	0.237	0	0	0	0	1	0.5218	0.9636	1.0745	0	0.2723	1.1545	-1.1132	0.0011	-0.1133
HIP_43464	CCTSICSLY	RGPGPQLRR	CCTSICSLYRGPGPQLRR		CMGA	13229/247050	0.990	43464	0.811	0.142460	-0.8	0.5	3	18	-0.2778	3	1	0	0	0.000000	-2.7333	1	0.1333	2.075000	0.1111	1.9639	1	1	1	1	-2.7333	-1.5711	-2.7333	0	1	0	0	0.5103	0.8688	1.089	0	0.2604	1.1859	-0.4082	0.0178	-0.3644
HIP_43465	VEALYLVC	NENTKFEVR	VEALYLVCNENTKFEVR		SCG1	19437/367830	0.990	43465	0.811	0.204659	0	0	-1	17	-0.0353	-1	1	1	0	0.000000	-7	-1	0.15	0.625000	0.2222	0.4028	0	0	0	0	7	0	-7	0	0	0	0	0.4385	1.0386	0.9612	0.1667	0.1922	0.9238	0	0.0225	-1.05
HIP_43466	HLVEALYL	GPQAEGDSE	HLVEALYLGPQAEGDSE		CMGA	4937/247050	0.998	43466	0.811	0.187582	0.1333	0.3333	-3	17	-0.3529	-4	1	1	0	0.000000	-3.9333	0	0.2	1.400000	0.1111	1.2889	1	1	1	1	0	0.1719	-3.9333	1	0	0	0	0.4718	0.9669	0.9845	0.3333	0.2226	0.9692	0.0629	0.04	-0.7867
HIP_43467	GAGSLQPLA	YEENSRDNP	GAGSLQPLAYEENSRDNP		SCG2	15084/334890	0.998	43467	0.811	-0.029059	-0.7167	0.1667	-2	18	-1.1778	-2	1	0	0	0.000000	-4.1	-2	0.0333	1.900000	0.2222	1.6778	1	0	1	0	8.2	-1.2024	-4.1	1	0	0	0	0.4551	1.2269	0.8445	0	0.2071	0.7132	-0.3263	0.0011	-0.1367

HIP_43936	EDLQVGQVE	SEESNVSMA	EDLQVGQVESEESNVSMA		SCG1	16952/367830	0.993	43936	0.811	-0.104005	-1.7667	0.1667	-5	18	-0.5944	-5	0	0	[illegible]
HIP_43937	QCCTSICSL	WKSSHFERR	QCCTSICSLWKSSHFERR		SCG1	36483/367830	0.938	43937	0.811	0.145296	-0.0167	0.3333	3	18	-0.5167	2	1	0	[illegible]
HIP_43938	ELGGGPGAG	NEPQSAPKE	ELGGGPGAGNEPQSAPKE		SCG2	2605/334890	1.000	43938	0.811	-0.117861	-1.2667	0.5	-2	18	-1.2	-2	0	0	[illegible]
HIP_43939	GSHLVEALY	VDREKGLSA	GSHLVEALYVDREKGLSA		CMGA	14640/247050	0.988	43939	0.811	0.100756	0.0833	0	0	18	-0.1333	-1	1	0	[illegible]
HIP_43940	LVEALYLVC	KRLGLSGLT	LVEALYLVCKRLGLSGLT		NPY	4282/49410	0.951	43940	0.811	0.152797	0.9833	0	1	18	1.1111	1	1	0	[illegible]
HIP_43941	GERGFFYTP	ELGKLTGPN	GERGFFYTPELGKLTGPN		SCG2	10786/334890	0.999	43941	0.811	-0.126648	-0.6167	0.3333	0	18	-0.7333	0	3	0	[illegible]
HIP_43942	VCGERGFF	WDEEDKRN	VCGERGFFWDEEDKRN		SCG1	19057/367830	0.991	43942	0.811	0.027659	-0.45	0.1667	-2	16	-1.4563	-2	3	0	[illegible]
HIP_43943	YLVCGERGF	KGREHIAKR	YLVCGERGFKGREHIAKR		SCG2	32261/334890	0.988	43943	0.811	0.111122	-1.8167	0.3333	4	18	-0.8	3	1	0	[illegible]
HIP_43944	HLCGSHLVE	WMRIILEAL	HLCGSHLVEWMRIILEAL		SCG2	27883/334890	0.992	43944	0.811	0.005913	0.1667	0	1	18	0.8111	-1	1	0	[illegible]
HIP_43945	GGPGAGSLQ	DSEGLSQGL	GGPGAGSLQDSEGLSQGL		CMGA	10282/247050	0.994	43945	0.811	-0.078649	-1.3833	0.3333	-2	18	-0.4	-2	0	0	[illegible]
HIP_43946	QCCTSICSL	GRGSEEYRA	QCCTSICSLGRGSEEYRA		SCG1	14294/367830	0.995	43946	0.811	0.203331	0.0333	0.5	0	18	-0.3944	0	0	0	[illegible]
HIP_43947	LCGSHLVEA	TTENENTKF	LCGSHLVEATTENENTKF		SCG1	24698/367830	0.981	43947	0.811	0.189375	-0.4	0	-1	18	-0.5	-2	0	0	[illegible]
HIP_43948	EQCCTSICS	PSSKDVMEK	EQCCTSICSPSSKDVMEK		CMGA	4057/247050	0.999	43948	0.811	0.153393	0.5	0.6667	-1	18	-0.5111	-1	0	0	[illegible]
HIP_43949	HLVEALYL	NTYGKRNAV	HLVEALYLNTYGKRNAV		IAPP	3319/45018	0.972	43949	0.811	0.166899	0.1333	0	2	17	-0.1412	1	2	1	[illegible]
HIP_43950	CGERGFFY	NYLNYGEEG	CGERGFFYNYLNYGEEG		SCG1	21920/367830	0.986	43950	0.811	0.059136	0.55	0	-2	17	-0.9176	-2	5	1	[illegible]
HIP_43951	GSLQPLAL	PYQGVSVP	GSLQPLALPYQGVSVP		SCG2	18479/334890	0.997	43951	0.811	0.205518	0.5	0.1667	0	16	0.3812	0	1	0	[illegible]
HIP_43952	LVCGERGFF	SLLGAVGLA	LVCGERGFFSLLGAVGLA		SCG1	7278/367830	0.999	43952	0.811	-0.030237	2	0.3333	0	18	1.3833	0	2	0	[illegible]
HIP_43953	SICSLYQLE	EESNVSMAS	SICSLYQLEEESNVSMAS		SCG1	21184/367830	0.988	43953	0.811	-0.058546	-1.8333	0.1667	-3	18	-0.0167	-3	1	0	[illegible]
HIP_43954	SLYQLENYC	APAEDMARY	SLYQLENYCAPAEDMARY		NPY	324/49410	0.999	43954	0.811	-0.189112	-0.05	0.1667	-2	18	-0.6056	-2	1	0	[illegible]
HIP_43955	ERGFFYTPK	LPYGAGRSR	ERGFFYTPKLPYGAGRSR		SCG2	16020/334890	0.997	43955	0.811	-0.009452	-0.8833	0.3333	3	18	-1.0111	3	3	0	[illegible]
HIP_43956	GSHLVEALY	LLGAVGLAA	GSHLVEALYLLGAVGLAA		SCG1	9497/367830	0.998	43956	0.811	-0.014340	1.9167	0.1667	0	18	1.3667	-1	1	0	[illegible]
HIP_43957	GPGAGSLQP	ALAEAYPS	GPGAGSLQPALAEAYPS		NPY	592/49410	0.997	43957	0.811	0.126656	1.0167	0.1667	-1	17	-0.0647	-1	0	1	[illegible]
HIP_43958	CGERGFFY	GLSQGLVD	CGERGFFYGLSQGLVD		CMGA	3323/247050	0.999	43958	0.811	0.153167	1.15	0.3333	-1	16	0.075	-1	3	0	[illegible]
HIP_43959	HLVEALYL	AWKELDRNY	HLVEALYLAWKELDRNY		SCG1	26052/367830	0.978	43959	0.811	0.067438	0.55	0	0	17	-0.3588	-1	2	1	[illegible]
HIP_43960	HLVEALYLV	QAPGEEEEE	HLVEALYLVQAPGEEEEE		CMGA	1666/247050	1.000	43960	0.811	0.076278	0.5667	0.1667	-5	18	-0.4222	-6	1	0	[illegible]
HIP_43961	SHLVEALY	ESSSRGEAG	SHLVEALYESSSRGEAG		SCG1	15078/367830	0.995	43961	0.811	0.232970	-0.1333	0.3333	-1	17	-0.4765	-2	1	1	[illegible]
HIP_43962	CTSICSLYQ	YLARMLVK	CTSICSLYQYLARMLVK		SCG2	20671/334890	0.996	43962	0.811	0.018308	0.55	0	2	17	0.7059	2	2	1	[illegible]
HIP_43963	GERGFFYT	LAAVNSMPV	GERGFFYTLAAVNSMPV		SCG1	14767/367830	0.995	43963	0.811	-0.035926	1.3667	0	0	17	0.3882	0	3	1	[illegible]
HIP_43964	VEALYLVCG	KGESRSEAL	VEALYLVCGKGESRSEAL		CMGA	10361/247050	0.994	43964	0.811	0.147201	-0.25	0.3333	-1	18	0.1833	-1	1	0	[illegible]
HIP_43965	CCTSICSL	FLVHSSNNF	CCTSICSLFLVHSSNNF		IAPP	1627/45018	0.993	43965	0.811	0.013430	2.7167	0.1667	1	17	0.9	0	1	1	[illegible]
HIP_43966	LGGGPGAGS	YGFRGPGPQ	LGGGPGAGSYGFRGPGPQ		CMGA	2841/247050	0.999	43966	0.811	0.001730	0.2833	0.5	1	18	-0.5389	1	2	0	[illegible]
HIP_43967	GERGFFYT	PGPQLRRG	GERGFFYTPGPQLRRG		CMGA	8994/247050	0.996	43967	0.811	0.176025	-0.4667	0.5	2	16	-1.1187	2	3	0	[illegible]
HIP_43968	VEQCCTSIC	EKNFFPEYN	VEQCCTSICEKNFFPEYN		SCG1	22825/367830	0.985	43968	0.811	0.079964	-0.7833	0.1667	-2	18	-0.4167	-2	1	0	[illegible]
HIP_43969	GERGFFYT	YQLIEISRN	GERGFFYTYQLIEISRN		SCG2	22668/334890	0.995	43969	0.811	0.000656	-0.0333	0	0	17	-0.5588	0	4	1	[illegible]
HIP_43970	AEDLQVGQV	NEPQSAPKE	AEDLQVGQVNEPQSAPKE		SCG2	4129/334890	1.000	43970	0.811	-0.084218	-1.3833	0.3333	-3	18	-1.1389	-3	0	0	[illegible]
HIP_43971	AGSLQPLAL	AEDLQVGQV	AGSLQPLALAEDLQVGQV		INSC	182/5184	0.998	43971	0.811	0.078584	0.7	0	-2	18	0.4611	-2	0	0	[illegible]
HIP_43972	PLALEGSLQ	NPYNQEKVL	PLALEGSLQNPYNQEKVL		SCG2	13179/334890	0.998	43972	0.811	-0.103108	-1.15	0.3333	-1	18	-0.5222	-1	1	0	[illegible]
HIP_43973	ERGFFYTPK	EEVRDSKEN	ERGFFYTPKEEVRDSKEN		SCG2	22212/334890	0.995	43973	0.811	-0.037332	-1.5	0.1667	-1	18	-1.8222	-1	2	0	[illegible]
HIP_43974	AGSLQPLA	TQQWPERKL	AGSLQPLATQQWPERKL		SCG2	23973/334890	0.994	43974	0.811	0.169000	-0.6167	0.1667	1	17	-0.7882	1	1	1	[illegible]
HIP_43975	QPLALEGSL	KEEEGSANR	QPLALEGSLKEEEGSANR		CMGA	11758/247050	0.993	43975	0.811	-0.083515	-1.3833	0.3333	-2	18	-1.0222	-2	0	0	[illegible]
HIP_43976	PGAGSLQPL	REEARFQDK	PGAGSLQPLREEARFQDK		SCG1	32325/367830	0.956	43976	0.811	0.006120	-2.1333	0.1667	0	18	-1.1778	0	0	0	[illegible]
HIP_43977	SLYQLENYC	YQLIEISRN	SLYQLENYCYQLIEISRN		SCG2	14443/334890	0.998	43977	0.811	-0.171815	-0.55	0	-1	18	-0.45	-1	2	0	[illegible]
HIP_43978	TSICSLYQL	RGDERILSI	TSICSLYQLRGDERILSI		CMGA	14710/247050	0.987	43978	0.811	0.230975	-1.5667	0.1667	0	18	0.1722	0	1	0	[illegible]

[illegible]

HIP_44447	GERGFFYTP	VEEQYTPQS	GERGFFYTPVEEQYTPQS		SCG2	14202/334890	0.998	44447	0.810	-0.168855	-1.0667	0.1667	-2	18	-1.1667	-2	3	0	0	0.000000	0.2667	-2	-0.0167	1.750000	0.2222	1.5278	1	0	1	0	-0.5333	-1.6296	0.8	0	0	0	1	0.5218	1.0836	0.8945	-0.1667	0.2723	0.8001	-0.5566	0.0003	-0.0044
HIP_44448	GERGFFYTP	VENRQMAYE	GERGFFYTPVENRQMAYE		SCG2	12805/334890	0.998	44448	0.810	-0.161851	-1.0667	0.1667	-1	18	-1.0389	-1	3	0	0	0.000000	0.2667	-1	-0.0167	1.750000	0.2222	1.5278	1	0	1	0	-0.2667	-1.6296	0.8	0	0	0	1	0.5218	0.9436	1.0312	0	0.2723	1.0633	-0.5566	0.0003	-0.0044
HIP_44449	SICSLYQL	WKELDRNYL	SICSLYQLWKELDRNYL		SCG1	32509/367830	0.955	44449	0.810	0.039761	-1.55	0	0	17	-0.3118	0	2	1	0	1.000000	-2.4333	0	0.05	1.900000	0.3333	1.5667	0	0	0	0	0	-2.4283	-4.8667	1	0	0	0	0.4487	1.1641	0.8235	0	0.2013	0.6781	-0.6955	0.0025	-0.1217
HIP_44450	LYQLENYCN	QGAKERAHQ	LYQLENYCNQGAKERAHQ		CMGA	9745/247050	0.995	44450	0.810	0.029691	-0.7333	0.1667	1	18	-1.4111	0	1	0	0	0.000000	0.0667	0	0.0833	0.625000	0.2222	0.4028	0	1	0	1	0	-0.2954	0.0667	0	1	0	0	0.4936	0.9622	1.0856	0.1667	0.2436	1.1786	-0.362	0.0069	0.0056
HIP_44451	LVCGERGFF	KSSHFERR	LVCGERGFFKSSHFERR		SCG1	27670/367830	0.973	44451	0.810	0.167962	-0.05	0.5	3	17	-0.6529	2	2	1	0	0.000000	-3.5667	1	0.1	0.700000	0.3333	0.3667	0	1	0	1	-3.5667	-0.0183	-7.1333	1	0	0	0	0.4551	0.9203	1.0012	0.1667	0.2071	1.0023	-0.0228	0.01	-0.3567
HIP_44452	VEALYLVC	QENQMDKAR	VEALYLVCQENQMDKAR		SCG1	19887/367830	0.990	44452	0.810	0.197439	0	0	-1	17	-0.3941	-1	1	1	0	0.000000	-7	-1	0.15	1.100000	0.1111	0.9889	0	0	0	0	7	0	-7	0	0	0	0	0.4385	1.1119	0.8645	0.1667	0.1922	0.7473	0	0.0225	-1.05
HIP_44453	TSICSLYQL	EGNNQAPG	TSICSLYQLEGNNQAPG		CMGA	9095/247050	0.996	44453	0.810	0.193009	-1.4	0.1667	-1	17	-0.4176	-1	1	1	0	0.000000	-2.1333	-1	0.1333	0.000000	0.1111	-0.1111	0	1	0	1	2.1333	0.1556	-2.1333	1	0	0	0	0.5218	1.0186	1.0312	0	0.2723	1.0633	-0.7305	0.0178	-0.2844
HIP_44454	GSLQPLAL	MYEENSRDN	GSLQPLALMYEENSRDN		SCG2	18244/334890	0.997	44454	0.810	0.072479	1.0833	0	-2	17	-0.9	-2	1	1	0	1.000000	-4.1	-1	0.05	0.950000	0.2222	0.7278	0	0	0	0	4.1	0.7884	-4.1	1	0	0	0	0.3051	1.2969	0.6512	0.5	0.0931	0.424	0.3306	0.0025	-0.205
HIP_44455	VEALYLVC	GRSRPDRSS	VEALYLVCGRSRPDRSS		SCG1	22175/367830	0.986	44455	0.810	0.248475	0.8	0.3333	1	17	-0.3471	1	1	1	0	0.000000	-5.4	1	0.2	0.625000	0.2222	0.4028	0	1	0	1	-5.4	0.3222	-5.4	0	0	0	0	0.4269	0.9505	0.919	0.1667	0.1823	0.8445	0.3415	0.04	-1.08
HIP_44456	GSHLVEALY	SGLTLALSL	GSHLVEALYSGLTLALSL		NPY	579/49410	0.997	44456	0.810	0.126105	1.15	0.3333	0	18	1.0389	-1	1	0	0	0.000000	-0.5667	0	0.15	2.000000	0.1111	1.8889	0	1	0	1	0	2.1722	-0.5667	0	1	0	0	0.3885	1.0619	0.8778	0.5	0.1509	0.7706	0.4467	0.0225	-0.085
HIP_44457	VELGGGPGA	WEDSKRWSK	VELGGGPGAWEDSKRWSK		CMGA	9151/247050	0.996	44457	0.810	-0.014860	-1.35	0.3333	0	18	-1.0889	0	1	0	1	1.000000	-2.5667	-2	-0.05	0.000000	0.2222	-0.2222	1	1	1	1	5.1333	0.3	-2.5667	1	0	0	0	0.5103	1.0122	1.039	0	0.2604	1.0795	-0.6888	0.0025	0.1283
HIP_44458	GIVEQCCTS	SGYPKTPGR	GIVEQCCTSSGYPKTPGR		SCG2	15246/334890	0.998	44458	0.810	-0.008806	-0.25	0.5	1	18	-0.5778	1	1	0	0	0.000000	-1.1667	0	0.0333	0.000000	0	0	0	1	0	1	0	0	-1.1667	0	1	0	0	0.5603	0.8288	1.1123	0	0.3139	1.2372	-0.1401	0.0011	-0.0389
HIP_44459	ICSLYQLEN	KNEQINDEM	ICSLYQLENKNEQINDEM		SCG2	29066/334890	0.991	44459	0.810	0.038224	-2.35	0	-3	18	-0.9167	-3	1	0	0	0.000000	-2.5667	1	0.05	0.000000	0.3333	-0.3333	0	0	0	0	-2.5667	0.7833	-2.5667	0	1	0	0	0.5051	1.1136	1.0012	-0.3333	0.2552	1.0023	-1.1871	0.0025	-0.1283
HIP_44460	GGGPGAGSL	PEEEGPTVV	GGGPGAGSLPEEEGPTVV		CMGA	3900/247050	0.999	44460	0.810	-0.070382	-1	0.5	-3	18	-0.2889	-3	0	0	2	0.000000	-3.7333	-2	0.0167	0.000000	0.3333	-0.3333	1	1	1	1	7.4667	0.3333	0	1	0	0	0	0.5667	1.0317	1.0333	-0.1667	0.3211	1.0678	-0.5667	0.0003	-0.0622
HIP_44461	GIVEQCCTS	GDERILSIL	GIVEQCCTSGDERILSIL		CMGA	7002/247050	0.997	44461	0.810	0.004696	-1.0667	0.3333	-2	18	0.4833	-2	0	0	0	0.000000	-2.8	-2	0.0667	0.000000	0.3333	-0.3333	0	1	0	1	5.6	0.3556	0	0	1	0	0	0.5603	0.9472	1.0356	-0.3333	0.3139	1.0726	-0.5976	0.0044	-0.1867
HIP_44462	GSHLVEALY	GLSGLTLAL	GSHLVEALYGLSGLTLAL		NPY	580/49410	0.997	44462	0.810	0.126000	1.15	0.3333	0	18	1.0611	-1	1	0	0	0.000000	-0.5667	0	0.15	2.000000	0.1111	1.8889	0	1	0	1	0	2.1722	-0.5667	0	1	0	0	0.3885	1.0619	0.8778	0.5	0.1509	0.7706	0.4467	0.0225	-0.085
HIP_44463	HLCGSHLV	WPERKLKH	HLCGSHLVWPERKLKH		SCG2	24092/334890	0.994	44463	0.810	0.202496	-0.2	0.1667	5	16	-0.6875	2	1	0	0	1.000000	-3.6	-1	0.1167	0.950000	0.2222	0.7278	1	0	1	0	3.6	-0.1456	-3.6	1	0	0	0	0.4269	1.0805	0.8456	0.1667	0.1823	0.7151	-0.0854	0.0136	-0.42
HIP_44464	HLVEALYL	NGQNGERA	HLVEALYLNGQNGERA		SCG2	14864/334890	0.998	44464	0.810	0.244304	-0.1833	0.1667	0	16	-0.5063	-1	1	0	0	0.000000	-4.5667	0	0.1833	0.000000	0.1111	-0.1111	0	1	0	1	0	0.0204	-4.5667	1	0	0	0	0.4551	0.9836	0.9912	0.3333	0.2071	0.9824	-0.0834	0.0136	-0.8372
HIP_44465	LCGSHLVEA	GLEPGKGRH	LCGSHLVEAGLEPGKGRH		SCG1	23340/367830	0.984	44465	0.810	0.135178	0.4	0.1667	2	18	-0.3278	0	0	0	0	0.000000	-0.8667	0	0.0833	0.000000	0.2222	-0.2222	0	1	0	1	0	-0.0889	0	1	0	0	0	0.4167	1.2217	0.7733	0.1667	0.1736	0.598	0.1667	0.0069	-0.0722
HIP_44466	HLVEALYLV	LNLLGMESA	HLVEALYLVLNLLGMESA		SCG2	11781/334890	0.998	44466	0.810	-0.010348	1.8	0	-1	18	1.1389	-2	1	0	0	1.000000	-0.8667	0	0.05	3.300000	0	3.3	0	0	0	0	0	5.94	-0.8667	1	0	0	0	0.3051	1.1086	0.7445	0.6667	0.0931	0.5543	0.5492	0.0025	-0.0433
HIP_44467	GERGFFYTP	VALNHLKAT	GERGFFYTPVALNHLKAT		IAPP	1304/45018	0.995	44467	0.810	0.018482	1.0333	0.1667	2	18	-0.15	1	3	0	0	0.000000	4.4667	0	-0.1167	1.750000	0	1.75	1	0	1	0	0	1.8083	13.4	0	0	0	1	0.3885	1.0386	0.8412	0.5	0.1509	0.7075	0.4014	0.0136	-0.5211
HIP_44468	LYQLENYCN	EEERLSKE	LYQLENYCNEEERLSKE		CMGA	13456/247050	0.990	44468	0.810	0.064083	-2.1333	0	-3	17	-1.5235	-3	1	1	1	0.000000	-2.7333	-3	0.1	0.625000	0.5556	0.0694	0	0	0	0	8.2	-0.1481	-2.7333	0	1	0	0	0.5436	1.2072	0.9323	-0.5	0.2955	0.8692	-1.1597	0.01	-0.2733
HIP_44469	LYQLENYCN	IAKRAMENM	LYQLENYCNIAKRAMENM		SCG2	17950/334890	0.997	44469	0.810	-0.111122	0.0167	0	0	18	-0.5556	0	1	0	0	0.000000	1.5667	1	0	1.750000	0.3333	1.4167	0	0	0	0	1.5667	0.0236	1.5667	0	1	0	0	0.3821	1.0441	0.9018	0.1667	0.146	0.8132	0.0064	0	0
HIP_44470	PGAGSLQPL	NNQKRERMD	PGAGSLQPLNNQKRERMD		SCG2	25339/334890	0.993	44470	0.810	0.023399	-1.9667	0.1667	1	18	-1.5222	1	0	0	0	0.000000	-3.0667	0	0.0333	0.000000	0.1111	-0.1111	1	0	1	0	0	0.2185	0	1	0	0	0	0.55	0.905	1.1833	0.1667	0.3025	1.4003	-1.0817	0.0011	-0.1022
HIP_44471	CGERGFFY	EAQDPEGK	CGERGFFYEAQDPEGK		CMGA	14896/247050	0.987	44471	0.810	0.116896	-0.15	0	-2	16	-1.2562	-2	3	0	0	0.000000	-3.1667	-1	0.1	0.700000	0.3333	0.3667	0	0	0	0	3.1667	-0.055	-9.5	0	1	0	0	0.3718	1.2486	0.7478	0.3333	0.1382	0.5592	-0.0558	0.01	-0.3167
HIP_44472	CCTSICSLY	HSEKSQRED	CCTSICSLYHSEKSQRED		SCG1	23411/367830	0.984	44472	0.810	-0.016293	-0.9667	0.3333	0	18	-0.8333	-1	1	0	0	0.000000	-3.0667	-1	0.05	2.075000	0.2222	1.8528	0	0	0	0	3.0667	-1.791	-3.0667	0	1	0	0	0.4769	1.0755	0.909	-0.1667	0.2275	0.8262	-0.461	0.0025	-0.1533
HIP_44473	CGERGFFYT	ILKLQVFLI	CGERGFFYTILKLQVFLI		IAPP	2242/45018	0.988	44473	0.810	-0.062246	0.8667	0	1	18	0.9611	1	3	0	0	0.000000	1.2	1	0	2.075000	0.1111	1.9639	0	0	0	0	1.2	1.702	3.6	0	1	0	0	0.3436	1.0755	0.724	0.3333	0.1181	0.5241	0.2978	0	0
HIP_44474	EQCCTSICS	LITRQRYGK	EQCCTSICSLITRQRYGK		NPY	2808/49410	0.978	44474	0.810	0.015931	2.3	0.1667	2	18	-0.4333	2	0	0	0	0.000000	0.4667	0	-0.0167	1.575000	0.1111	1.4639	0	0	0	0	0	3.3669	0	0	1	0	0	0.3718	1.0186	0.7512	0.5	0.1382	0.5642	0.8551	0.0003	-0.0078
HIP_44475	TSICSLYQ	DKQYSSHHT	TSICSLYQDKQYSSHHT		SCG1	35474/367830	0.943	44475	0.810	0.187856	-1.9833	0	2	17	-1.0118	0	2	1	0	0.000000	-3.3	0	0.1	0.000000	0.2222	-0.2222	0	0	0	0	0	0.4407	-6.6	0	1	0	0	0.4487	1.0974	0.8635	-0.1667	0.2013	0.7456	-0.89	0.01	-0.33
HIP_44476	VEALYLVC	DSEGLSQGL	VEALYLVCDSEGLSQGL		CMGA	9118/247050	0.996	44476	0.810	0.162718	0.45	0.1667	-3	17	0.6	-3	1	1	0	0.000000	-6.1	-2	0.1667	1.575000	0.2222	1.3528	0	0	0	0	12.2	0.6088	-6.1	0	0	0	0	0.4103	1.1188	0.7923	0	0.1683	0.6278	0.1846	0.0278	-1.0167
HIP_44477	VCGERGFFY	VAHQLQAL	VCGERGFFYVAHQLQAL		CMGA	14725/247050	0.987	44477	0.810	0.005735	1.1833	0	1	17	0.4353	0	3	1	0	0.000000	-0.5	0	0	1.750000	0.1111	1.6389	0	0	0	0	0	1.9394	-1.5	0	1	0	0	0.2769	1.1588	0.6956	0.5	0.0767	0.4839	0.3277	0	0
HIP_44478	SHLVEALYL	TSEVDKRR	SHLVEALYLTSEVDKRR		SCG1	19267/367830	0.990	44478	0.810	0.238053	0.2167	0.1667	1	17	-0.5059	0	1	1	0	0.000000	-3.7667	-1	0.1667	1.050000	0.2222	0.8278	0	0	0	0	3.7667	0.1794	-3.7667	1	0	0	0	0.4385	1.1269	0.8012	0.1667	0.1922	0.6418	0.095	0.0278	-0.6278
HIP_44479	CCTSICSLY	GSGRLQNGQ	CCTSICSLYGSGRLQNGQ		SCG2	5333/334890	0.999	44479	0.810	0.135798	0.0167	0.6667	1	18	-0.0556	1	1	0	0	0.000000	-1.1	0	0.1667	2.075000	0.1111	1.9639	0	2	0	1	0	0.0327	-1.1	0	1	0	0	0.5385	0.8203	1.1345	0.1667	0.2899	1.2871	0.009	0.0278	-0.1833
HIP_44480	GERGFFYTP	EDQELESL	GERGFFYTPEDQELESL		CMGA	12974/247050	0.991	44480	0.810	-0.024400	-2.35	0.1667	-4	17	-1.0294	-4	3	1	0	0.000000	-2.3	-2	0.0333	0.700000	0.3333	0.3667	1	0	1	0	4.6	-0.8617	-6.9	0	0	0	1	0.5603	1.0105	1.0023	-0.3333	0.3139	1.0046	-1.3166	0.0011	-0.0767
HIP_44481	CTSICSLYQ	LALLLCAGQ	CTSICSLYQLALLLCAGQ		CMGA	8330/247050	0.996	44481	0.810	-0.027129	1.4	0	0	18	1.3111	0	1	0	1	0.000000	3.4667	0	-0.05	2.525000	0	2.525	0	0	0	0	0	3.535	3.4667	0	1	0	0	0.3051	1.2469	0.6745	0.6667	0.0931	0.4549	0.4272	0.0025	-0.1733
HIP_44482	VCGERGFF	SKGQPRSQ	VCGERGFFSKGQPRSQ		SCG1	22809/367830	0.985	44482	0.810	0.175074	0.0167	0.5	2	16	-0.9688	2	2	0	0	0.000000	-3.4333	1	0.1167	0.700000	0.3333	0.3667	0	2	0	1	-3.4333	0.0061	-6.8667	1	0	0	0	0.4718	0.8703	1.0512	0.1667	0.2226	1.1049	0.0079	0.0136	-0.4006
HIP_44483	VCGERGFFY	MDMSDDYE	VCGERGFFYMDMSDDYE		SCG2	13093/334890	0.998	44483	0.810	0.003160	0.7667	0	-4	17	-0.5841	-4	3	1	0	0.000000	-1.3333	-1	0.05	1.175000	0.2222	0.9528	0	0	0	0	1.3333	0.7305	-4	0	1	0	0	0.2769	1.1405	0.7023	0.5	0.0767	0.4932	0.2123	0.0025	-0.0667
HIP_44484	LYQLENYC	DEPQWSLYP	LYQLENYCDEPQWSLYP		SCG1	14417/367830	0.995	44484	0.810	0.092523	-1.8167	0.1667	-3	17	-0.9353	-3	2	1	0	0.000000	-2.1	-2	0.1167	0.625000	0.3333	0.2917	1	0	1	0	4.2	-0.5299	-4.2	0	0	0	0	0.5321	0.9691	1.0901	-0.3333	0.2831	1.1884	-0.9666	0.0136	-0.245
HIP_44485	HLCGSHLVE	KQYSSHHTA	HLCGSHLVEKQYSSHHTA		SCG1	41076/367830	0.912	44485	0.810	0.104296	-0.7	0	4	18	-0.6889	0	1	0	0	0.000000	-4.4	2	0.05	1.050000	0.2222	0.8278	0	0	0	0	-8.8	-0.5794	-4.4	0	0	1	0	0.4103	1.1705	0.7556	0	0.1683	0.571	-0.2872	0.0025	-0.22
HIP_44486	VCGERGFFY	DDTPNRQY	VCGERGFFYDDTPNRQY		SCG2	19218/334890	0.996	44486	0.810	0.169887	-0.5667	0	-1	17	-1.1706	-1	3	1	0	0.000000	-4	-2	0.15	0.700000	0.3333	0.3667	0	0	0	0	8	-0.2078	-12	0	1	0	0	0.3821	1.0491	0.8135	0	0.146	0.6617	-0.2165	0.0225	-0.6
HIP_44487	HLVEALYLV	EKGLSAEPG	HLVEALYLVEKGLSAEPG		CMGA	10247/247050	0.994	44487	0.810	0.203398	-0.1833	0.1667	-1	18	0.2833	-2	1	0	0	0.000000	-4.8333	0	0.1833	2.350000	0.2222	2.1278	0	1	0	1	0	-0.3901	-4.8333	1	0	0	0	0.4269	1.0688	0.8423	0	0.1823	0.7095	-0.0783	0.0136	-0.8861
HIP_44488	LGGGPGAGS	GERGFFYT	LGGGPGAGSGERGFFYT		INSB	236/4968	0.964	44488	0.810	0.003146	-1.3	0.5	0	17	-0.2353	0	0	1	1	0.000000	-3	0	0.0333	0.000000	0.2222	-0.2222	0	2	0	1	0	0.2889	0	0	1	0	0	0.5385	0.9636	1.0845	-0.1667	0.2899	1.1761	-0.7	0.0011	-0.1
HIP_44489	SHLVEALYL	SVEPEREL	SHLVEALYLSVEPEREL		SCG2	11812/334890	0.998	44489	0.810	0.171124	1.0333	0.1667	-2	17	-0.0471	-3	1	1	0	0.000000	-2.1333	-1	0.1167	0.000000	0.2222	-0.2222	0	0	0	0	2.1333	-0.2296	-2.1333	1	0	0	0	0.3718	1.1653	0.7245	0.3333	0.1382	0.5249	0.3842	0.0136	-0.249
[illegible]																																														

HIP_44958	CGSHLVEAL	NLLGMESAA	CGSHLVEALNLLGMESAA		SCG2	13114/334890	0.998	44958	0.810	-0.047803	1.0333	0	-1	18	0.7278	-2	0	0	0	[illegible]
HIP_44959	HLVEALYL	LGEYLARML	HLVEALYLLGEYLARML		SCG2	9551/334890	0.999	44959	0.810	0.141532	1.0333	0.1667	0	17	0.8706	-1	2	1	0	[illegible]
HIP_44960	GPGAGSLQP	PSPEMIRAL	GPGAGSLQPPSPEMIRAL		SCG2	8259/334890	0.999	44960	0.810	0.011417	-0.8833	0.6667	0	18	-0.1722	0	0	0	0	[illegible]
HIP_44961	TSICSLYQL	EDSLEAGLP	TSICSLYQLEDSLEAGLP		CMGA	8677/247050	0.996	44961	0.810	0.099955	-1.4667	0.1667	-3	18	0.2	-3	1	0	0	[illegible]
HIP_44962	GERGFFYTP	NQAPGEEEE	GERGFFYTPNQAPGEEEE		CMGA	5090/247050	0.998	44962	0.810	-0.126162	-1.4667	0.1667	-4	18	-1.5556	-4	3	0	2	[illegible]
HIP_44963	ICSLYQLEN	EEGENYQKG	ICSLYQLENEEGENYQKG		SCG1	16898/367830	0.993	44963	0.810	0.034558	-1.7667	0.1667	-3	18	-1.1944	-3	1	0	0	[illegible]
HIP_44964	IVEQCCTSI	GLFRGGKSG	IVEQCCTSIGLFRGGKSG		CMGA	5475/247050	0.998	44964	0.810	-0.019495	1.5333	0.3333	1	18	0.3056	1	1	0	0	[illegible]
HIP_44965	CGERGFFY	LFRGGKSGE	CGERGFFYLFRGGKSGE		CMGA	18505/247050	0.977	44965	0.810	0.073521	1.0667	0	1	17	-0.5471	1	4	1	0	[illegible]
HIP_44966	GERGFFYT	PSSREDSLE	GERGFFYTPSSREDSLE		CMGA	6410/247050	0.998	44966	0.810	0.140708	-0.4	0.5	-2	17	-1.2	-2	3	1	0	[illegible]
HIP_44967	VEALYLVC	GLPLQVRGY	VEALYLVCGLPLQVRGY		CMGA	5698/247050	0.998	44967	0.810	0.179412	2.05	0.3333	0	17	0.9176	0	1	1	0	[illegible]
HIP_44968	LVCGERGFF	LGALAEAYP	LVCGERGFFLGALAEAYP		NPY	581/49410	0.997	44968	0.810	-0.061670	1.7333	0.3333	-1	18	0.75	-1	2	0	0	[illegible]
HIP_44969	SICSLYQLE	WIPHVENRQ	SICSLYQLEWIPHVENRQ		SCG2	8173/334890	0.999	44969	0.810	-0.142789	-0.2	0.1667	0	18	-0.4056	-1	2	0	0	[illegible]
HIP_44970	SICSLYQL	ENYQKGERG	SICSLYQLENYQKGERG		SCG1	28994/367830	0.969	44970	0.810	0.021754	-1.55	0	0	17	-0.9588	0	2	1	0	[illegible]
HIP_44971	GERGFFYTP	SDDYETQQW	GERGFFYTPSDDYETQQW		SCG2	16042/334890	0.997	44971	0.810	-0.025748	-1.9	0.3333	-3	18	-1.5556	-3	4	0	0	[illegible]
HIP_44972	EAEDLQVGQ	GQNGERATR	EAEDLQVGQGQNGERATR		SCG2	5354/334890	0.999	44972	0.810	-0.089693	-1.1833	0.3333	-2	18	-1.5167	-2	0	0	0	[illegible]
HIP_44973	CTSICSLYQ	SQEESEEGE	CTSICSLYQSQEESEEGE		SCG1	9777/367830	0.998	44973	0.810	0.072221	-1.4667	0.1667	-5	18	-0.9333	-5	1	0	0	[illegible]
HIP_44974	DLQVGQVEL	GEEDIQGPT	DLQVGQVELGEEDIQGPT		SCG1	9475/367830	0.998	44974	0.810	0.129135	-0.4833	0.1667	-5	18	-0.6111	-5	0	0	0	[illegible]
HIP_44975	SHLVEALYL	QLSDDVSK	SHLVEALYLQLSDDVSK		SCG2	15273/334890	0.998	44975	0.810	0.202282	0.9667	0.1667	-1	17	0.0353	-2	1	1	0	[illegible]
HIP_44976	GSHLVEALY	EESEEGEE	GSHLVEALYEESEEGEE		SCG1	9338/367830	0.998	44976	0.810	0.116005	-0.5833	0.1667	-6	17	-1.0471	-7	1	1	0	[illegible]
HIP_44977	GSHLVEALY	ESEEGEED	GSHLVEALYESEEGEED		SCG1	9339/367830	0.998	44977	0.810	0.116005	-0.5833	0.1667	-6	17	-1.0471	-7	1	1	0	[illegible]
HIP_44978	AGSLQPLAL	GEEDIQGP	AGSLQPLALGEEDIQGP		SCG1	10991/367830	0.997	44978	0.810	0.174955	0.3333	0.1667	-3	17	-0.1882	-3	0	1	0	[illegible]
HIP_44979	LYQLENYCN	NDEMKRSGQ	LYQLENYCNNDEMKRSGQ		SCG2	20473/334890	0.996	44979	0.810	0.077873	-2.1333	0	-1	18	-1.5667	-1	1	0	0	[illegible]
HIP_44980	RGFFYTPKT	HMQFPPMYE	RGFFYTPKTHMQFPPMYE		SCG2	17523/334890	0.997	44980	0.810	0.077877	-1.8333	0.1667	2	18	-0.8667	1	2	0	0	[illegible]
HIP_44981	GSLQPLAL	QVELGGG	GSLQPLALQVELGGG		INSC	211/5184	0.997	44981	0.810	0.136686	1.1	0	-1	15	0.4467	-1	0	1	1	[illegible]
HIP_44982	RGFFYTPKT	SRSEALAVD	RGFFYTPKTSRSEALAVD		CMGA	18452/247050	0.977	44982	0.810	-0.028043	-2.05	0.5	1	18	-0.5	1	1	0	0	[illegible]
HIP_44983	CCTSICSLY	TQNAFLNER	CCTSICSLYTQNAFLNER		SCG1	20475/367830	0.989	44983	0.810	0.033562	-1	0.1667	0	18	0.0778	0	1	0	0	[illegible]
HIP_44984	VELGGGPGA	WKSSHFERR	VELGGGPGAWKSSHFERR		SCG1	19185/367830	0.991	44984	0.810	0.000731	-0.9667	0.5	2	18	-0.9	1	1	0	1	[illegible]
HIP_44985	EQCCTSICS	QGLFRGGKS	EQCCTSICSQGLFRGGKS		CMGA	8398/247050	0.996	44985	0.810	0.064935	1.0167	0.3333	1	18	-0.2556	1	1	0	0	[illegible]
HIP_44986	HLCGSHLVE	ETQNAFLNE	HLCGSHLVEETQNAFLNE		SCG1	22386/367830	0.986	44986	0.810	0.143175	-0.5333	0	-1	18	-0.3667	-3	0	0	0	[illegible]
HIP_44987	SHLVEALYL	DEPQWSLY	SHLVEALYLDEPQWSLY		SCG1	14640/367830	0.995	44987	0.810	0.186623	-0.3833	0.1667	-2	17	-0.1588	-3	1	1	0	[illegible]
HIP_44988	TSICSLYQL	DGARPQALP	TSICSLYQLDGARPQALP		CMGA	10182/247050	0.995	44988	0.810	0.114553	-0.5167	0.1667	0	18	-0.0111	0	1	0	0	[illegible]
HIP_44989	QHLCGSHLV	SLPSQKYPG	QHLCGSHLVSLPSQKYPG		CMGA	11042/247050	0.993	44989	0.810	0.183374	1.0333	0.3333	3	18	-0.3833	1	0	0	0	[illegible]
HIP_44990	LVCGERGFF	EERLSKEW	LVCGERGFFEERLSKEW		CMGA	16166/247050	0.984	44990	0.810	0.077332	-1.05	0.1667	-1	17	-0.5588	-1	2	1	0	[illegible]
HIP_44991	SLYQLENYC	SKDQLSDDV	SLYQLENYCSKDQLSDDV		SCG2	16050/334890	0.997	44991	0.810	0.110942	-1.75	0.1667	-3	18	-0.85	-3	1	0	0	[illegible]
HIP_44992	EQCCTSIC	YDWWEKKPF	EQCCTSICYDWWEKKPF		SCG1	24383/367830	0.982	44992	0.810	0.038913	0.0833	0.1667	-1	17	-0.7765	-1	3	1	0	[illegible]
HIP_44993	LYQLENYCN	DSSEEKHLE	LYQLENYCNDSSEEKHLE		SCG1	8393/367830	0.998	44993	0.810	0.090668	-1.2333	0.3333	-3	18	-1.4111	-4	1	0	0	[illegible]
HIP_44994	GSLQPLAL	TGEKPNGSV	GSLQPLALTGEKPNGSV		SCG2	12558/334890	0.998	44994	0.810	0.224231	0.8	0.1667	0	17	-0.2176	0	0	1	0	[illegible]
HIP_44995	LQVGQVELG	WWEKKPFSE	LQVGQVELGWWEKKPFSE		SCG1	14733/367830	0.995	44995	0.810	0.005344	-0.9	0.1667	-1	18	-0.6389	-1	2	0	0	[illegible]
HIP_44996	SICSLYQLE	GERATRLFE	SICSLYQLEGERATRLFE		SCG2	23144/334890	0.995	44996	0.810	-0.015101	-1.9333	0.1667	-1	18	-0.2222	-1	1	0	0	[illegible]
HIP_44997	HLVEALYLV	EEPGETQN	HLVEALYLVEEPGETQN		SCG1	11822/367830	0.997	44997	0.810	0.153932	-0.3167	0.1667	-3	17	-0.3882	-4	1	1	0	[illegible]
HIP_44998	RGFFYTPKT	LNERKQASA	RGFFYTPKTLNERKQASA		SCG1	31818/367830	0.958	44998	0.810	0.022935	-1.5667	0.1667	3	18	-1.1	3	1	0	0	[illegible]
HIP_44999	SHLVEALYL	QKEPDLRL	SHLVEALYLQKEPDLRL		SCG2	25315/334890	0.993	44999	0.810	0.252613	-0.7667	0	0	17	-0.2529	-1	1	1	0	[illegible]
HIP_45000	DLQVGQVEL	LLVCLGALA	DLQVGQVELLLVCLGALA		NPY	1387/49410	0.992	45000	0.810	0.004589	2.7167	0	-2	18	1.4833	-2	0	0	1	[illegible]

[illegible]

HIP_45469	YLVCGERGF	DTEVMKCIV	YLVCGERGFDTEVMKCIV		CMGA	12639/247050	0.991	45469	0.809	-0.047265	-1.6333	0.1667	-1	18	0.4944	-1	1	0	0	[illegible]
HIP_45470	LVCGERGFF	HGYGEESEE	LVCGERGFFHGYGEESEE		SCG1	16131/367830	0.994	45470	0.809	0.002204	0.05	0.3333	-3	18	-0.7111	-4	3	0	0	[illegible]
HIP_45471	LYQLENYCN	ETGSQENHP	LYQLENYCNETGSQENHP		SCG1	7542/367830	0.999	45471	0.809	0.086659	-1.15	0.1667	-2	18	-1.5111	-3	1	0	0	[illegible]
HIP_45472	GERGFFYTP	AIKEHLNQG	GERGFFYTPAIKEHLNQG		SCG2	12005/334890	0.998	45472	0.809	-0.059317	-0.2	0.1667	1	18	-0.8167	0	3	0	0	[illegible]
HIP_45473	SLQPLAL	DLQVGQV	SLQPLALDLQVGQV		INSC	210/5184	0.997	45473	0.809	0.131931	1.0333	0	-1	14	0.6143	-1	0	0	0	[illegible]
HIP_45474	CGERGFFY	DTEVMKCI	CGERGFFYDTEVMKCI		CMGA	13200/247050	0.990	45474	0.809	0.151609	-0.5667	0	-1	16	-0.0313	-1	3	0	0	[illegible]
HIP_45475	GERGFFYT	EPVSTHQEA	GERGFFYTEPVSTHQEA		SCG1	11850/367830	0.997	45475	0.809	-0.004669	-0.0167	0.1667	-1	17	-0.9412	-2	3	1	0	[illegible]
HIP_45476	RGFFYTPKT	QEESEESNV	RGFFYTPKTQEESEESNV		SCG1	24102/367830	0.982	45476	0.809	-0.053613	-2.7833	0.1667	-2	18	-1.4389	-2	1	0	0	[illegible]
HIP_45477	LVCGERGFF	PMNKGDTE	LVCGERGFFPMNKGDTE		CMGA	9052/247050	0.996	45477	0.809	0.028593	0.3333	0.3333	-1	17	-0.4647	-1	2	1	0	[illegible]
HIP_45478	SHLVEALYL	LLGAVGLAA	SHLVEALYLLLGAVGLAA		SCG1	7247/367830	0.999	45478	0.809	0.030918	2.25	0.1667	0	18	1.6	-1	1	0	1	[illegible]
HIP_45479	VEQCCTSIC	PGKWQQQGD	VEQCCTSICPGKWQQQGD		SCG1	13159/367830	0.996	45479	0.809	0.145953	0.05	0.5	-1	18	-0.75	-1	1	0	1	[illegible]
HIP_45480	QPLALEGSL	EEEMAVVPQ	QPLALEGSLEEEMAVVPQ		CMGA	7590/247050	0.997	45480	0.809	-0.089763	-1.3167	0.3333	-4	18	-0.0056	-4	0	0	1	[illegible]
HIP_45481	GSLQPLAL	EPERELDLP	GSLQPLALEPERELDLP		SCG2	11005/334890	0.999	45481	0.809	0.153683	0.1333	0.1667	-3	17	-0.4235	-3	0	1	0	[illegible]
HIP_45482	HLVEALYL	SLSAIEAE	HLVEALYLSLSAIEAE		CMGA	4181/247050	0.999	45482	0.809	0.160478	1.4167	0.3333	-2	16	0.7937	-3	1	0	0	[illegible]
HIP_45483	TSICSLYQL	NFPMDMSDD	TSICSLYQLNFPMDMSDD		SCG2	1746/334890	1.000	45483	0.809	-0.052245	-0.55	0.1667	-3	18	-0.1278	-3	2	0	0	[illegible]
HIP_45484	SLYQLENYC	PSPMPVSQE	SLYQLENYCPSPMPVSQE		CMGA	3552/247050	0.999	45484	0.809	0.065962	-1.05	0.5	-2	18	-0.6167	-2	1	0	0	[illegible]
HIP_45485	LVEALYLVC	EIKGYPGV	LVEALYLVCEIKGYPGV		SCG1	22744/367830	0.985	45485	0.809	0.158458	1.2667	0	-1	17	0.9941	-1	1	1	0	[illegible]
HIP_45486	ERGFFYTPK	AYENLNDKD	ERGFFYTPKAYENLNDKD		SCG2	13060/334890	0.998	45486	0.809	0.010822	-1.5333	0.1667	-1	18	-1.5222	-1	3	0	0	[illegible]
HIP_45487	AGSLQPLAL	AQDPEGKG	AGSLQPLALAQDPEGKG		CMGA	12201/247050	0.992	45487	0.809	0.150896	0.7	0	-1	17	-0.3706	-1	0	1	0	[illegible]
HIP_45488	VEQCCTSIC	RGLEPGKGR	VEQCCTSICRGLEPGKGR		SCG1	19340/367830	0.990	45488	0.809	0.067843	0.85	0.3333	1	18	-0.4278	1	0	0	0	[illegible]
HIP_45489	CSLYQLENY	ESEESNVSM	CSLYQLENYESEESNVSM		SCG1	22818/367830	0.985	45489	0.809	-0.040340	-2.6833	0.1667	-4	18	-0.7833	-4	1	0	0	[illegible]
HIP_45490	SLYQLENYC	EGKGEQEHS	SLYQLENYCEGKGEQEHS		CMGA	9493/247050	0.995	45490	0.809	0.119072	-1.6833	0.1667	-2	18	-1.4722	-3	1	0	0	[illegible]
HIP_45491	VCGERGFF	AILSSTNV	VCGERGFFAILSSTNV		IAPP	767/45018	0.997	45491	0.809	0.025112	2.55	0.1667	0	16	0.75	0	2	0	0	[illegible]
HIP_45492	HLVEALYLV	LPERDSLSE	HLVEALYLVLPERDSLSE		SCG2	5582/334890	0.999	45492	0.809	0.095671	0.9	0.1667	-2	18	0.1667	-3	1	0	0	[illegible]
HIP_45493	ERGFFYTP	ENTKFEVRL	ERGFFYTPENTKFEVRL		SCG1	24405/367830	0.981	45493	0.809	-0.036078	-1.8833	0.1667	0	17	-0.8941	0	3	1	0	[illegible]
HIP_45494	CCTSICSLY	KPFSEDVNW	CCTSICSLYKPFSEDVNW		SCG1	15029/367830	0.995	45494	0.809	-0.040460	-0.1667	0.3333	-1	18	0.0833	-1	2	0	0	[illegible]
HIP_45495	LVCGERGFF	VLSVALNH	LVCGERGFFVLSVALNH		IAPP	1273/45018	0.995	45495	0.809	-0.034215	2.0667	0.3333	1	17	1.0353	0	2	1	0	[illegible]
HIP_45496	LYQLENYCN	IAYEDVVGG	LYQLENYCNIAYEDVVGG		SCG2	5796/334890	0.999	45496	0.809	-0.113379	0.45	0	-3	18	-0.05	-3	2	0	0	[illegible]
HIP_45497	LCGSHLVEA	NKPYALNSE	LCGSHLVEANKPYALNSE		SCG2	26815/334890	0.983	45497	0.809	0.182540	-1.0833	0.1667	0	18	-0.2389	-1	1	0	0	[illegible]
HIP_45498	CGERGFFY	FQDKQYSS	CGERGFFYFQDKQYSS		SCG1	24318/367830	0.982	45498	0.809	0.118174	0.0167	0	0	16	-1.0313	0	4	0	0	[illegible]
HIP_45499	HLCGSHLVE	GSSQETDKL	HLCGSHLVEGSSQETDKL		SCG2	9056/334890	0.999	45499	0.809	0.183727	0.4167	0.5	0	18	-0.5611	-2	0	0	0	[illegible]
HIP_45500	VEALYLVCG	EESEEGEED	VEALYLVCGEESEEGEED		SCG1	5764/367830	0.999	45500	0.809	-0.004852	-0.25	0.3333	-8	18	-0.5889	-8	1	0	0	[illegible]
HIP_45501	LYQLENYCN	PTKADTEKW	LYQLENYCNPTKADTEKW		SCG1	17561/367830	0.992	45501	0.809	0.088717	-1.4167	0.1667	-1	18	-1.3	-1	1	0	0	[illegible]
HIP_45502	GERGFFYTP	AAWIPHVEN	GERGFFYTPAAWIPHVEN		SCG2	6698/334890	0.999	45502	0.809	-0.032261	-0.15	0.1667	0	18	-0.4	-1	4	0	0	[illegible]
HIP_45503	DLQVGQVEL	ILEALRQAE	DLQVGQVELILEALRQAE		SCG2	14050/334890	0.998	45503	0.809	-0.050529	1.55	0	-3	18	0.1278	-3	0	0	0	[illegible]
HIP_45504	LVEALYLV	AHGYGEESE	LVEALYLVAHGYGEESE		SCG1	9608/367830	0.998	45504	0.809	0.050524	0.8167	0.1667	-3	17	0.1176	-4	2	1	0	[illegible]
HIP_45505	QCCTSICSL	DAPAEDMAR	QCCTSICSLDAPAEDMAR		NPY	515/49410	0.998	45505	0.809	-0.010070	0.3667	0.3333	-2	18	0.0389	-2	0	0	0	[illegible]
HIP_45506	GAGSLQPLA	YEKRNLARV	GAGSLQPLAYEKRNLARV		SCG1	34890/367830	0.945	45506	0.809	0.061492	-0.7833	0.1667	2	18	-0.3833	2	1	0	0	[illegible]
HIP_45507	CGSHLVEAL	PEEEGPTVV	CGSHLVEALPEEEGPTVV		CMGA	7554/247050	0.997	45507	0.809	0.083263	-1.0833	0.1667	-3	18	0.1	-4	0	0	1	[illegible]
HIP_45508	VELGGGPGA	EEEGPTVVL	VELGGGPGAEEEGPTVVL		CMGA	6365/247050	0.998	45508	0.809	-0.074635	-1.7833	0.3333	-4	18	0.1167	-4	0	0	2	[illegible]
HIP_45509	ERGFFYTPK	SEADLDHPD	ERGFFYTPKSEADLDHPD		SCG2	13225/334890	0.998	45509	0.809	-0.114971	-1.45	0.3333	-2	18	-1.35	-3	2	0	0	[illegible]
HIP_45510	GGPGAGSLQ	DTEVMKCIV	GGPGAGSLQDTEVMKCIV		CMGA	14025/247050	0.989	45510	0.809	-0.101725	-1.3667	0.1667	-1	18	0.2111	-1	0	0	0	[illegible]
HIP_45511	QHLCGSHLV	SEDVNWGYE	QHLCGSHLVSEDVNWGYE		SCG1	13116/367830	0.996	45511	0.809	0.152303	-0.5	0.1667	-1	18	-0.5556	-3	0	0	0	[illegible]
[illegible]																				

HIP_45980	RGFFYTPKT	SEKSQREDE	RGFFYTPKTSEKSQREDE	SCG1	31678/367830	0.959	45980	0.809	-0.031809	-2.4	0.3333	0	18	-1.9444	0	1	0
HIP_45981	GGGPGAGSL	RSQEESEEG	GGGPGAGSLRSQEESEEG	SCG1	16166/367830	0.994	45981	0.809	-0.053468	-1.0333	0.5	-3	18	-1.2667	-3	0	0
HIP_45982	CGSHLVEAL	DLLMKVLEY	CGSHLVEALDLLMKVLEY	SCG2	17195/334890	0.997	45982	0.809	-0.041064	1.0333	0	-1	18	0.75	-2	0	0
HIP_45983	CGERGFFY	LGEKRDHH	CGERGFFYLGEKRDHH	SCG1	16706/367830	0.993	45983	0.809	0.158885	0.7	0.1667	2	16	-1.275	0	3	0
HIP_45984	LVCGERGFF	NYQKGERG	LVCGERGFFNYQKGERG	SCG1	26966/367830	0.975	45984	0.809	0.067697	-0.5167	0.1667	1	17	-0.8059	1	3	1
HIP_45985	VEALYLVC	ERGLEPGKG	VEALYLVCERGLEPGKG	SCG1	20547/367830	0.989	45985	0.809	0.240241	0.35	0.1667	-1	17	0.0647	-1	1	1
HIP_45986	AGSLQPLAL	KKSAEFPDF	AGSLQPLALKKSAEFPDF	SCG1	28016/367830	0.972	45986	0.809	0.208620	0.1333	0.1667	0	18	-0.0611	0	0	0
HIP_45987	AEDLQVGQV	GERGFFYT	AEDLQVGQVGERGFFYT	INSB	161/4968	0.982	45987	0.809	-0.000168	-1.35	0.3333	-2	17	-0.3294	-2	0	1
HIP_45988	GAGSLQPLA	EEEEDNRDS	GAGSLQPLAEEEEDNRDS	CMGA	7665/247050	0.997	45988	0.809	-0.019251	-1.0833	0.1667	-5	18	-1.4056	-5	0	0
HIP_45989	VEQCCTSIC	EYGEEIKGY	VEQCCTSICEYGEEIKGY	SCG1	6310/367830	0.999	45989	0.809	0.003804	0.1667	0.3333	-3	18	-0.3111	-3	1	0
HIP_45990	VELGGGPGA	RRTRPRHHH	VELGGGPGARRTRPRHHH	SCG1	36827/367830	0.936	45990	0.809	0.051543	-1.65	0.3333	6	18	-1.4889	3	0	0
HIP_45991	CCTSICSLY	LQNGQNGER	CCTSICSLYLQNGQNGER	SCG2	16968/334890	0.997	45991	0.809	-0.084122	-0.25	0.1667	0	18	-0.3778	0	1	0
HIP_45992	HLCGSHLVE	EEKGHPQEE	HLCGSHLVEEEKGHPQEE	SCG1	28145/367830	0.972	45992	0.809	0.130781	-1.0667	0	-1	18	-1.3	-4	0	0
HIP_45993	HLVEALYLV	EDWNPVEE	HLVEALYLVEDWNPVEE	SCG2	14875/334890	0.998	45993	0.809	0.079195	-0.2	0	-4	17	-0.1294	-5	2	1
HIP_45994	VEALYLVC	ESKGQPRSQ	VEALYLVCESKGQPRSQ	SCG1	25231/367830	0.980	45994	0.809	0.249875	0.3833	0.1667	0	17	-0.4118	0	1	1
HIP_45995	VEALYLVCG	DKETTENEN	VEALYLVCGDKETTENEN	SCG1	16361/367830	0.994	45995	0.809	0.051971	-0.7667	0.1667	-4	18	-0.6222	-4	1	0
HIP_45996	HLVEALYL	EESNVSMAS	HLVEALYLEESNVSMAS	SCG1	13597/367830	0.996	45996	0.809	0.153428	-0.25	0.1667	-2	17	0.2588	-3	1	1
HIP_45997	ERGFFYTPK	LSILRHQNL	ERGFFYTPKLSILRHQNL	CMGA	11247/247050	0.993	45997	0.809	-0.004876	0.2167	0.3333	3	18	-0.55	2	2	0
HIP_45998	GERGFFYTP	EALRQAENE	GERGFFYTPEALRQAENE	SCG2	10812/334890	0.999	45998	0.809	-0.075951	-0.25	0.1667	-2	18	-1.1889	-2	3	0
HIP_45999	SHLVEALY	HPSLGYKEI	SHLVEALYHPSLGYKEI	CMGA	13363/247050	0.990	45999	0.809	0.230725	-0.2167	0.3333	1	17	-0.0941	-1	1	1
HIP_46000	LGGGPGAGS	GWQAKREEE	LGGGPGAGSGWQAKREEE	CMGA	3605/247050	0.999	46000	0.809	-0.082197	-0.7	0.5	-1	18	-1.15	-1	1	0
HIP_46001	LYQLENYCN	SRERADEPQ	LYQLENYCNSRERADEPQ	SCG1	23882/367830	0.983	46001	0.809	0.140825	-1.85	0.1667	-2	18	-1.6722	-2	1	0
HIP_46002	QHLCGSHLV	QEEVRDSKE	QHLCGSHLVQEEVRDSKE	SCG2	16059/334890	0.997	46002	0.809	0.133640	-0.95	0	0	18	-1.0722	-2	0	0
HIP_46003	AGSLQPLAL	QKGERGEDS	AGSLQPLALQKGERGEDS	SCG1	20127/367830	0.989	46003	0.809	0.229159	0.2667	0.1667	-1	18	-0.85	-1	0	0
HIP_46004	CSLYQLENY	ENRQMAYEN	CSLYQLENYENRQMAYEN	SCG2	31181/334890	0.989	46004	0.809	0.006973	-3.3	0	-2	18	-1.3	-2	1	0
HIP_46005	ICSLYQLEN	GKRSSPETL	ICSLYQLENGKRSSPETL	NPY	4490/49410	0.946	46005	0.809	0.109883	-2	0.1667	0	18	-0.5778	0	1	0
HIP_46006	HLCGSHLVE	YQKGERGED	HLCGSHLVEYQKGERGED	SCG1	37220/367830	0.934	46006	0.809	0.074885	-0.7	0	0	18	-1.1833	-2	1	0
HIP_46007	GERGFFYT	SEGLSQGLV	GERGFFYTSEGLSQGLV	CMGA	8931/247050	0.996	46007	0.809	0.162020	-0.65	0.3333	-1	17	-0.1647	-1	3	1
HIP_46008	VEALYLVCG	ETGSQENHP	VEALYLVCGETGSQENHP	SCG1	3507/367830	1.000	46008	0.809	0.083808	0.2833	0.3333	-2	18	-0.3111	-3	1	0
HIP_46009	LQPLALEGS	KENKPYALN	LQPLALEGSKENKPYALN	SCG2	27460/334890	0.992	46009	0.809	-0.039517	-2.6	0.3333	0	18	-0.6778	0	0	0
HIP_46010	LYQLENYCN	NPYYDPLQ	LYQLENYCNNPYYDPLQ	SCG1	16957/367830	0.993	46010	0.809	0.001527	-1.45	0.1667	-2	17	-1.1176	-2	3	1
HIP_46011	LCGSHLVEA	EPDLRLENV	LCGSHLVEAEPDLRLENV	SCG2	12372/334890	0.998	46011	0.809	0.125962	-1.0167	0.1667	-2	18	-0.0056	-3	0	0
HIP_46012	VCGERGFFY	KVLPRLPY	VCGERGFFYKVLPRLPY	SCG2	26018/334890	0.993	46012	0.809	0.067823	1.4	0	2	17	0.0647	2	3	1
HIP_46013	GSLQPLAL	EYIENLRQQ	GSLQPLALEYIENLRQQ	SCG2	19885/334890	0.996	46013	0.809	0.082777	1.5167	0	-1	17	-0.4765	-1	1	1
HIP_46014	GPGAGSLQ	AILSSTNVG	GPGAGSLQAILSSTNVG	IAPP	1160/45018	0.995	46014	0.809	0.130935	1.6	0.1667	0	17	0.3882	0	0	1
HIP_46015	CCTSICSLY	GPTVVLNPH	CCTSICSLYGPTVVLNPH	CMGA	3176/247050	0.999	46015	0.809	0.054762	-0.1667	0.5	1	18	0.7444	0	1	0
HIP_46016	LYQLENYCN	DTPNRQYWD	LYQLENYCNDTPNRQYWD	SCG2	11072/334890	0.999	46016	0.809	0.078531	-1.35	0.1667	-2	18	-1.6389	-2	1	0
HIP_46017	GERGFFYT	AEPGWQAKR	GERGFFYTAEPGWQAKR	CMGA	16957/247050	0.982	46017	0.809	0.071733	-0.4167	0.1667	1	17	-1.1706	1	4	1
HIP_46018	VCGERGFF	AYGFRGPGP	VCGERGFFAYGFRGPGP	CMGA	12533/247050	0.991	46018	0.809	0.038032	0.8833	0.3333	1	17	-0.1235	1	4	1
HIP_46019	GIVEQCCTS	HQQKKHSGF	GIVEQCCTSHQQKKHSGF	CMGA	27476/247050	0.935	46019	0.809	0.019923	-1.5333	0.1667	3	18	-0.8222	1	0	0
HIP_46020	VEALYLVC	SKGQPRSQE	VEALYLVCSKGQPRSQE	SCG1	20882/367830	0.988	46020	0.809	0.241975	0.9	0.3333	0	17	-0.4118	0	1	1
HIP_46021	NQHLCGSHL	PYNQEKVLP	NQHLCGSHLPYNQEKVLP	SCG2	22258/334890	0.995	46021	0.809	0.034123	-1.1	0.3333	2	18	-0.8556	0	1	0
HIP_46022	QCCTSICSL	TTENENTKF	QCCTSICSLTTENENTKF	SCG1	17069/367830	0.993	46022	0.809	0.134650	0.1	0.1667	-1	18	-0.4	-1	0	0
HIP_46023	AGSLQPLAL	AYENLNDK	AGSLQPLALAYENLNDK	SCG2	20200/334890	0.996	46023	0.809	0.086486	1.0667	0	-1	17	-0.2882	-1	1	1
HIP_46024	EQCCTSICS	KEEEEEMAV	EQCCTSICSKEEEEEMAV	CMGA	9133/247050	0.996	46024	0.809	0.043032	-0.7833	0.1667	-5	18	-0.6	-5	0	0
HIP_46025	HLVEALYLV	AGEEAVPEE	HLVEALYLVAGEEAVPEE	CMGA	1616/247050	1.000	46025	0.809	0.106006	0.7667	0.1667	-4	18	0.3	-5	1	0
HIP_46026	VCGERGFFY	LQKIAEKF	VCGERGFFYLQKIAEKF	SCG1	27587/367830	0.973	46026	0.809	0.116741	0.1167	0	1	17	0.0176	1	3	1
HIP_46027	VEALYLVC	HPSLGYKEI	VEALYLVCHPSLGYKEI	CMGA	12807/247050	0.991	46027	0.809	0.211860	0.8167	0.3333	0	17	0.5353	-1	1	1
HIP_46028	CSLYQLENY	PFSEDVNWG	CSLYQLENYPFSEDVNWG	SCG1	9617/367830	0.998	46028	0.809	-0.142825	-1.3167	0.3333	-3	18	-0.6111	-3	2	0
HIP_46029	GERGFFYTP	LKTGEKPNG	GERGFFYTPLKTGEKPNG	SCG2	17747/334890	0.997	46029	0.809	-0.121429	-0.7333	0.1667	1	18	-1.1611	1	3	0
HIP_46030	SICSLYQLE	ALAEAYPSK	SICSLYQLEALAEAYPSK	NPY	1221/49410	0.993	46030	0.809	0.017154	0.7	0	-1	18	0.1556	-1	1	0
HIP_46031	CCTSICSLY	EENSRDNPF	CCTSICSLYEENSRDNPF	SCG2	16634/334890	0.997	46031	0.809	0.020069	-1.4667	0.1667	-2	18	-0.5222	-3	1	0
HIP_46032	NQHLCGSHL	GEESEEERG	NQHLCGSHLGEESEEERG	SCG1	8235/367830	0.998	46032	0.809	0.069616	-1.2667	0.3333	-2	18	-1.5611	-4	0	0
HIP_46033	YLVCGERGF	QQQGDLQDT	YLVCGERGFQQQGDLQDT	SCG1	17603/367830	0.992	46033	0.809	-0.016819	-2.1	0.1667	-2	18	-0.8389	-2	1	0
HIP_46034	GERGFFYTP	AVEVLKREP	GERGFFYTPAVEVLKREP	IAPP	1702/45018	0.993	46034	0.809	-0.084880	-0.1833	0.1667	0	18	-0.5444	0	3	0
HIP_46035	SICSLYQL	PGKWQQQG	SICSLYQLPGKWQQQG	SCG1	17588/367830	0.992	46035	0.809	0.202786	-1.15	0.3333	1	16	-0.5938	1	2	0
HIP_46036	GPGAGSLQP	PEDQELESL	GPGAGSLQPPEDQELESL	CMGA	5895/247050	0.998	46036	0.809	-0.064935	-1.65	0.3333	-4	18	-0.8556	-4	0	0
HIP_46037	GSHLVEALY	MPVDNRNHN	GSHLVEALYMPVDNRNHN	SCG1	13088/367830	0.996	46037	0.809	-0.009875	1.4667	0.1667	1	18	-0.7111	-1	1	0

HIP_46491	LCGSHLVEA	VPLQQKENG	LCGSHLVEAVPLQQKENG		SCG2	11802/334890	0.998	46491	0.808	-0.041926
HIP_46492	CGSHLVEAL	DNPFKRTNE	CGSHLVEALDNPFKRTNE		SCG2	18893/334890	0.996	46492	0.808	0.131169
HIP_46493	VCGERGFF	YDSEEPVST	VCGERGFFYDSEEPVST		SCG1	18230/367830	0.992	46493	0.808	0.032792
HIP_46494	NQHLCGSHL	NVPRTRLED	NQHLCGSHLNVPRTRLED		NPY	2625/49410	0.981	46494	0.808	-0.000307
HIP_46495	VEQCCTSIC	EEDKRNYPS	VEQCCTSICEEDKRNYPS		SCG1	17809/367830	0.992	46495	0.808	0.025617
HIP_46496	HLVEALYL	FRARAYGF	HLVEALYLFRARAYGF		CMGA	21415/247050	0.967	46496	0.808	0.118380
HIP_46497	QHLCGSHLV	DVSKVIAYL	QHLCGSHLVDVSKVIAYL		SCG2	18888/334890	0.996	46497	0.808	0.189681
HIP_46498	TSICSLYQL	SRERADEPQ	TSICSLYQLSRERADEPQ		SCG1	28873/367830	0.969	46498	0.808	0.220113
HIP_46499	SLYQLENYC	ATDGARPQA	SLYQLENYCATDGARPQA		CMGA	15378/247050	0.986	46499	0.808	-0.093570
HIP_46500	GAGSLQPLA	WQQQGDLQD	GAGSLQPLAWQQQGDLQD		SCG1	15188/367830	0.995	46500	0.808	-0.033353
HIP_46501	GSLQPLAL	EQCCTSICS	GSLQPLALEQCCTSICS		INSA	146/3024	0.959	46501	0.808	0.151636
HIP_46502	CSLYQLENY	RGEAGAPGE	CSLYQLENYRGEAGAPGE		SCG1	21904/367830	0.986	46502	0.808	-0.020611
HIP_46503	SHLVEALYL	GGEPRAYF	SHLVEALYLGGEPRAYF		SCG1	13447/367830	0.996	46503	0.808	0.231110
HIP_46504	SLYQLENYC	DHPDLFQNR	SLYQLENYCDHPDLFQNR		SCG2	19398/334890	0.996	46504	0.808	0.002360
HIP_46505	AGSLQPLAL	LLKELQDLA	AGSLQPLALLLKELQDLA		CMGA	13788/247050	0.989	46505	0.808	0.047445
HIP_46506	LVCGERGFF	EEATNTHP	LVCGERGFFEEATNTHP		CMGA	13180/247050	0.990	46506	0.808	0.025769
HIP_46507	HLVEALYLV	EADLDHPDL	HLVEALYLVEADLDHPDL		SCG2	10649/334890	0.999	46507	0.808	0.055951
HIP_46508	SHLVEALYL	SFRARAYG	SHLVEALYLSFRARAYG		CMGA	20433/247050	0.970	46508	0.808	0.214117
HIP_46509	VCGERGFF	TAEKRKRL	VCGERGFFTAEKRKRL		SCG1	28615/367830	0.970	46509	0.808	0.081604
HIP_46510	GERGFFYT	EVKTRHSEK	GERGFFYTEVKTRHSEK		SCG1	35685/367830	0.942	46510	0.808	0.059193
HIP_46511	HLVEALYLV	QRYGKRSS	HLVEALYLVQRYGKRSS		NPY	4720/49410	0.960	46511	0.808	0.168447
HIP_46512	SLYQLENYC	SGRLQNGQN	SLYQLENYCSGRLQNGQN		SCG2	13789/334890	0.998	46512	0.808	0.115550
HIP_46513	VEALYLVCG	GDTEVMKCI	VEALYLVCGGDTEVMKCI		CMGA	3174/247050	0.999	46513	0.808	0.096504
HIP_46514	GERGFFYTP	SGAEAASFQ	GERGFFYTPSGAEAASFQ		SCG2	4380/334890	1.000	46514	0.808	-0.146588
HIP_46515	HLVEALYLV	APKENKPY	HLVEALYLVAPKENKPY		SCG2	21607/334890	0.995	46515	0.808	0.185995
HIP_46516	LVEALYLVC	LMKVLEYLN	LVEALYLVCLMKVLEYLN		SCG2	18353/334890	0.997	46516	0.808	0.024570
HIP_46517	LYLVCGERG	SQKYPGPQA	LYLVCGERGSQKYPGPQA		CMGA	21102/247050	0.968	46517	0.808	-0.054463
HIP_46518	SLYQLENYC	QFPPMYEEN	SLYQLENYCQFPPMYEEN		SCG2	3072/334890	1.000	46518	0.808	-0.111099
HIP_46519	LVCGERGFF	AQDPEGKG	LVCGERGFFAQDPEGKG		CMGA	13369/247050	0.990	46519	0.808	0.031173
HIP_46520	CGERGFFYT	DLDDISEAD	CGERGFFYTDLDDISEAD		SCG2	14960/334890	0.998	46520	0.808	-0.002124
HIP_46521	TSICSLYQL	EAVEEPSSK	TSICSLYQLEAVEEPSSK		CMGA	14368/247050	0.988	46521	0.808	-0.040700
HIP_46522	GSHLVEALY	WAEGGGHS	GSHLVEALYWAEGGGHS		SCG1	20470/367830	0.989	46522	0.808	0.033913
HIP_46523	RGFFYTPKT	EEMAVVPQG	RGFFYTPKTEEMAVVPQG		CMGA	18546/247050	0.977	46523	0.808	-0.004909
HIP_46524	PGAGSLQPL	WDEDLLMKV	PGAGSLQPLWDEDLLMKV		SCG2	15842/334890	0.997	46524	0.808	-0.034620
HIP_46525	HLVEALYLV	VGPPKNDDT	HLVEALYLVVGPPKNDDT		SCG2	2248/334890	1.000	46525	0.808	0.113105
HIP_46526	QHLCGSHLV	LQDTKENRE	QHLCGSHLVLQDTKENRE		SCG1	20569/367830	0.989	46526	0.808	0.122637
HIP_46527	GSHLVEALY	SDTREEKR	GSHLVEALYSDTREEKR		SCG1	17356/367830	0.993	46527	0.808	0.205227
HIP_46528	LCGSHLVEA	PQLRRGWRP	LCGSHLVEAPQLRRGWRP		CMGA	22722/247050	0.962	46528	0.808	0.130408
HIP_46529	ICSLYQLEN	RGSEEYRAP	ICSLYQLENRGSEEYRAP		SCG1	18655/367830	0.991	46529	0.808	0.096604
HIP_46530	GSLQPLALE	HLNQGSSQE	GSLQPLALEHLNQGSSQE		SCG2	26855/334890	0.993	46530	0.808	-0.056062
HIP_46531	VCGERGFFY	GPTVVLNP	VCGERGFFYGPTVVLNP		CMGA	8518/247050	0.996	46531	0.808	0.186625
HIP_46532	ERGFFYTPK	VPQGLFRGG	ERGFFYTPKVPQGLFRGG		CMGA	13486/247050	0.990	46532	0.808	-0.082829
HIP_46533	AEDLQVGQV	SNNFGAILS	AEDLQVGQVSNNFGAILS		IAPP	1212/45018	0.995	46533	0.808	-0.051241
HIP_46534	CGERGFFYT	TSYFPNPY	CGERGFFYTTSYFPNPY		SCG2	21618/334890	0.995	46534	0.808	0.057844
HIP_46535	CTSICSLYQ	DVVGGEDWN	CTSICSLYQDVVGGEDWN		SCG2	13461/334890	0.998	46535	0.808	-0.094354
HIP_46536	EQCCTSIC	FLVHSSNNF	EQCCTSICFLVHSSNNF		IAPP	1422/45018	0.994	46536	0.808	0.019059
HIP_46537	LYQLENYCN	MSDTREEKR	LYQLENYCNMSDTREEKR		SCG1	19910/367830	0.990	46537	0.808	-0.043591
HIP_46538	LVEALYLV	ENEKDRADQ	LVEALYLVENEKDRADQ		SCG1	16151/367830	0.994	46538	0.808	0.139287
[illegible]										

HIP_47002	CGERGFFYT	QQPEGKGE	CGERGFFYTQQPEGKGE		CMGA	14194/247050	0.989	47002	0.808	0.129025	-1.3	0.1667	-2	17	-1.3529	-2	3	1	0
HIP_47003	YLVCGERGF	QAIKEHLNQ	YLVCGERGFQAIKEHLNQ		SCG2	13062/334890	0.998	47003	0.808	0.079175	0.1167	0.1667	1	18	-0.4333	0	1	0	0
HIP_47004	CSLYQLENY	QEESEESNV	CSLYQLENYQEESEESNV		SCG1	24434/367830	0.981	47004	0.808	-0.051903	-3.1333	0	-5	18	-1.2333	-5	1	0	0
HIP_47005	GGGPGAG	GFFYTPK	GGGPGAGGFFYTPK		INSB	15/4968	0.999	47005	0.808	-0.011456	1.0333	0.5	1	14	-0.2929	1	3	0	1
HIP_47006	SLYQLENYC	MLVKYPEII	SLYQLENYCMLVKYPEII		SCG2	9775/334890	0.999	47006	0.808	-0.039142	1.2667	0	-1	18	0.2667	-1	1	0	0
HIP_47007	GERGFFYT	PQESKGQP	GERGFFYTPQESKGQP		SCG1	24071/367830	0.982	47007	0.808	0.141901	-1.3	0.1667	0	16	-1.5	0	3	0	0
HIP_47008	LVCGERGFF	ECFETLRGD	LVCGERGFFECFETLRGD		CMGA	7721/247050	0.997	47008	0.808	-0.016140	1.1667	0.1667	-2	18	0.0167	-2	3	0	0
HIP_47009	RGFFYTPKT	THSREKSSQ	RGFFYTPKTTHSREKSSQ		SCG1	33546/367830	0.951	47009	0.808	0.029258	-1.8167	0.3333	4	18	-1.6222	3	1	0	0
HIP_47010	EQCCTSICS	WKELDRNYL	EQCCTSICSWKELDRNYL		SCG1	29158/367830	0.968	47010	0.808	0.039308	-0.35	0.1667	-1	18	-0.6	-1	1	0	0
HIP_47011	HLCGSHLVE	LQKEPDLRL	HLCGSHLVELQKEPDLRL		SCG2	32129/334890	0.988	47011	0.808	0.037333	0.15	0	1	18	-0.3278	-1	0	0	0
HIP_47012	GSHLVEALY	KRFLGEGH	GSHLVEALYKRFLGEGH		SCG1	32508/367830	0.955	47012	0.808	0.119508	-0.2167	0	2	17	-0.2882	0	2	1	0
HIP_47013	LYQLENYCN	LRHYINLIT	LYQLENYCNLRHYINLIT		NPY	4632/49410	0.943	47013	0.808	-0.058840	-1.0333	0	1	18	-0.1722	0	2	0	0
HIP_47014	HLVEALYLV	LTLALSLLV	HLVEALYLVLTLALSLLV		NPY	2084/49410	0.986	47014	0.808	0.000554	2.2667	0	0	18	2.0611	-1	1	0	0
HIP_47015	GERGFFYTP	DVKDKETTE	GERGFFYTPDVKDKETTE		SCG1	17612/367830	0.992	47015	0.808	-0.122185	-1.1333	0.1667	-2	18	-1.4333	-2	3	0	0
HIP_47016	SLYQLENYC	QGAKERAHQ	SLYQLENYCQGAKERAHQ		CMGA	9967/247050	0.995	47016	0.808	0.022078	-0.7333	0.1667	1	18	-1.2611	0	1	0	0
HIP_47017	ALYLVCGER	SQKYPGPQA	ALYLVCGERSQKYPGPQA		CMGA	21169/247050	0.968	47017	0.808	-0.072749	-2.7667	0.3333	1	18	-0.4667	1	1	0	0
HIP_47018	RGFFYTPKT	EEPVSTHQE	RGFFYTPKTEEPVSTHQE		SCG1	24707/367830	0.981	47018	0.808	-0.099127	-2.4667	0.3333	0	18	-1.3111	-1	1	0	0
HIP_47019	CGERGFFY	KEELVARS	CGERGFFYKEELVARS		SCG1	25688/367830	0.978	47019	0.808	0.177313	-1.1	0	0	16	-0.525	0	3	0	0
HIP_47020	RGFFYTPKT	YYDPLQWKS	RGFFYTPKTYYDPLQWKS		SCG1	23981/367830	0.982	47020	0.808	0.105574	-2.05	0.1667	2	18	-1.1389	2	3	0	0
HIP_47021	LVCGERGFF	LARVPKLDL	LVCGERGFFLARVPKLDL		SCG1	18788/367830	0.991	47021	0.808	-0.000393	1.05	0.1667	1	18	0.6222	1	2	0	0
HIP_47022	QVELGGG	NAVEVLK	QVELGGGNAVEVLK		IAPP	19/45018	1.000	47022	0.808	0.150888	0.2167	0.5	-1	14	0.2071	-1	0	0	1
HIP_47023	ICSLYQLEN	SDLLMREST	ICSLYQLENSDLLMREST		NPY	2058/49410	0.986	47023	0.808	-0.181465	-0.6167	0.1667	-2	18	-0.1278	-2	1	0	0
HIP_47024	LQVGQVELG	EEDKRNYPS	LQVGQVELGEEDKRNYPS		SCG1	22274/367830	0.986	47024	0.808	-0.062363	-1.7667	0.1667	-2	18	-1.1889	-2	0	0	0
HIP_47025	GSLQPLALE	YFMSDTREE	GSLQPLALEYFMSDTREE		SCG1	12463/367830	0.997	47025	0.808	-0.048663	0.9167	0	-3	18	-0.5389	-3	2	0	0
HIP_47026	ICSLYQLEN	YQLIEISRN	ICSLYQLENYQLIEISRN		SCG2	14336/334890	0.998	47026	0.808	-0.097990	-0.7	0	-1	18	-0.1278	-1	2	0	0
HIP_47027	HLCGSHLVE	RKESKDQLS	HLCGSHLVERKESKDQLS		SCG2	37650/334890	0.981	47027	0.808	0.145582	-1.2333	0	2	18	-0.9667	0	0	0	0
HIP_47028	HLVEALYL	YFMSDTRE	HLVEALYLYFMSDTRE		SCG1	20255/367830	0.989	47028	0.808	0.025573	1.6167	0	-1	16	-0.0125	-2	3	0	0
HIP_47029	LVCGERGFF	IPLIFLISG	LVCGERGFFIPLIFLISG		SCG2	7157/334890	0.999	47029	0.808	-0.050497	1.9833	0.3333	0	18	1.5778	0	2	0	0
HIP_47030	VEALYLVCG	PSKPDNPGE	VEALYLVCGPSKPDNPGE		NPY	1826/49410	0.989	47030	0.808	0.106780	0	0.5	-2	18	-0.2944	-2	1	0	0
HIP_47031	GSLQPLAL	GGPGAGS	GSLQPLALGGPGAGS		INSC	147/5184	0.998	47031	0.808	0.216751	1.1667	0.5	0	15	0.3133	0	0	1	0
HIP_47032	GSHLVEALY	FPNPYNQE	GSHLVEALYFPNPYNQE		SCG2	9746/334890	0.999	47032	0.808	0.092544	0.3333	0.1667	-1	17	-0.6647	-2	2	1	0
HIP_47033	NQHLCGSHL	QQGDLQDTK	NQHLCGSHLQQGDLQDTK		SCG1	16184/367830	0.994	47033	0.808	0.151294	-1.2667	0.3333	1	18	-1.2889	-1	0	0	0
HIP_47034	GSHLVEALY	EKKPFSEDV	GSHLVEALYEKKPFSEDV		SCG1	28602/367830	0.970	47034	0.808	0.188540	-1.1667	0	-1	18	-0.5167	-2	1	0	0
HIP_47035	PGAGSLQPL	PPKNDDTPN	PGAGSLQPLPPKNDDTPN		SCG2	15222/334890	0.998	47035	0.808	0.002911	-1.4	0.5	-1	18	-1.2389	-1	0	0	0
HIP_47036	GERGFFYT	KDVKDKETT	GERGFFYTKDVKDKETT		SCG1	34220/367830	0.948	47036	0.808	0.081241	-0.4	0	0	17	-1.4471	0	3	1	0
HIP_47037	GERGFFYTP	GAEAASFQR	GERGFFYTPGAEAASFQR		SCG2	10498/334890	0.999	47037	0.808	-0.144288	-0.95	0.3333	0	18	-0.6278	0	3	0	0
HIP_47038	VEALYLVC	LGKLTGPNN	VEALYLVCLGKLTGPNN		SCG2	19209/334890	0.996	47038	0.808	0.222170	1.6667	0.1667	0	17	0.5353	0	1	1	0
HIP_47039	SLYQLENYC	THPPASLPS	SLYQLENYCTHPPASLPS		CMGA	13304/247050	0.990	47039	0.808	-0.016743	-1.3	0.1667	0	18	-0.4722	-1	1	0	0
HIP_47040	QVGQVELGG	EEEEATNTH	QVGQVELGGEEEEATNTH		CMGA	6689/247050	0.998	47040	0.808	-0.091883	-1.25	0.3333	-4	18	-1.1	-5	0	0	2
HIP_47041	VEALYLVCG	SEEGEEDAT	VEALYLVCGSEEGEEDAT		SCG1	7408/367830	0.999	47041	0.808	0.013657	-0.25	0.3333	-6	18	-0.1389	-6	1	0	0
HIP_47042	SLYQLENYC	ESKDQLSDD	SLYQLENYCESKDQLSDD		SCG2	15683/334890	0.997	47042	0.808	0.086615	-1.75	0.1667	-4	18	-1.2778	-4	1	0	0
HIP_47043	GERGFFYT	QINDEMKRS	GERGFFYTQINDEMKRS		SCG2	22705/334890	0.995	47043	0.808	0.010716	-0.2833	0	0	17	-1.2941	0	3	1	0
HIP_47044	HLVEALYLV	TRCIIEVLS	HLVEALYLVTRCIIEVLS		SCG1	21677/367830	0.987	47044	0.808	0.121984	0.6667	0	0	18	1.3111	-1	1	0	0
HIP_47045	GAGSLQPLA	QEESEEGEE	GAGSLQPLAQEESEEGEE		SCG1	8855/367830	0.998	47045	0.808	-0.036184	-1.0833	0.1667	-6	18	-1.1778	-6	0	0	0
HIP_47046	ERGFFYTP	SQEKTHSRE	ERGFFYTPSQEKTHSRE		SCG1	27167/367830	0.974	47046	0.808	0.001014	-1.9	0.3333	1	17	-1.8118	0	2	1	0
HIP_47047	GAGSLQPL	FLVHSSNN	GAGSLQPLFLVHSSNN		IAPP	882/45018	0.997	47047	0.808	0.089749	1.5833	0.1667	1	16	0.1062	0	1	0	0
HIP_47048	HLCGSHLVE	PLQQKENGD	HLCGSHLVEPLQQKENGD		SCG2	16452/334890	0.997	47048	0.808	0.032886	0.5333	0.1667	0	18	-0.9111	-2	0	0	0
HIP_47049	PGAGSLQ	ERGFFYTPK	PGAGSLQERGFFYTPK		INSB	407/4968	0.918	47049	0.808	0.096742	-1.4833	0.3333	1	16	-0.7125	1	2	2	0
HIP_47050	HLVEALYL	LNEKNFFPE	HLVEALYLLNEKNFFPE		SCG1	16623/367830	0.993	47050	0.808	0.145989	0.5167	0	-1	17	-0.0412	-2	1	1	0
HIP_47051	QHLCGSHLV	GEEEEEEEE	QHLCGSHLVGEEEEEEEEE		CMGA	3854/247050	0.999	47051	0.808	0.118706	-0.4333	0.1667	-6	18	-1.4	-8	0	0	6
HIP_47052	HLVEALYLV	IPLIFLISG	HLVEALYLVIPLIFLISG		SCG2	11814/334890	0.998	47052	0.808	-0.005882	2.2333	0.1667	0	18	1.9278	-1	1	0	0

HIP_47513	GSHLVEALY	DNPFKRTN	GSHLVEALYDNPFKRTN		SCG2	17710/334890	0.997	47513	0.807	0.168805	-0.7167	0.1667
HIP_47514	VEQCCTSIC	QSEESWDEE	VEQCCTSICQSEESWDEE		SCG1	4938/367830	0.999	47514	0.807	0.008897	-0.2667	0.3333
HIP_47515	QCCTSICSL	AYEDVVGGE	QCCTSICSLAYEDVVGGE		SCG2	13564/334890	0.998	47515	0.807	-0.031256	0.4167	0.1667
HIP_47516	GERGFFYT	VALNHLKA	GERGFFYTVALNHLKA		IAPP	2229/45018	0.988	47516	0.807	-0.026884	1.7667	0
HIP_47517	AGSLQPLAL	GLSVEDILN	AGSLQPLALGLSVEDILN		SCG2	4602/334890	1.000	47517	0.807	0.147440	2	0.3333
HIP_47518	VEALYLVC	EDKRNYPSL	VEALYLVCEDKRNYPSL		SCG1	24674/367830	0.981	47518	0.807	0.210397	-0.0667	0
HIP_47519	CGERGFFY	NRQYWDED	CGERGFFYNRQYWDED		SCG2	23888/334890	0.994	47519	0.807	0.180895	-1.2	0
HIP_47520	GPGAGSLQP	SAPKENKPY	GPGAGSLQPSAPKENKPY		SCG2	16270/334890	0.997	47520	0.807	-0.038004	-0.3167	0.5
HIP_47521	GSLQPLALE	DESHLPERD	GSLQPLALEDESHLPERD		SCG2	14633/334890	0.998	47521	0.807	0.112262	-0.95	0.1667
HIP_47522	HLVEALYL	KFPSPEMI	HLVEALYLKFPSPEMI		SCG2	21782/334890	0.995	47522	0.807	0.188753	0.6	0.1667
HIP_47523	GAGSLQPLA	EEEEAEAGE	GAGSLQPLAEEEEAEAGE		CMGA	6984/247050	0.997	47523	0.807	-0.027208	-1.0833	0.1667
HIP_47524	HLCGSHLVE	GQPRSQEES	HLCGSHLVEGQPRSQEES		SCG1	13498/367830	0.996	47524	0.807	0.174887	-0.1667	0.3333
HIP_47525	NQHLCGSHL	EEKKEEEGS	NQHLCGSHLEEKKEEEGS		CMGA	18191/247050	0.978	47525	0.807	0.079652	-1.85	0.1667
HIP_47526	SLQPLALEG	EEKKEEEGS	SLQPLALEGEEKKEEEGS		CMGA	15648/247050	0.985	47526	0.807	-0.094338	-1.8333	0.1667
HIP_47527	GGGPGAGSL	GLFRGGKSG	GGGPGAGSLGLFRGGKSG		CMGA	5096/247050	0.998	47527	0.807	0.034818	1.4667	0.5
HIP_47528	LVEALYLV	EEENELTLN	LVEALYLVEEENELTLN		SCG1	14307/367830	0.995	47528	0.807	0.110980	-0.6333	0
HIP_47529	CSLYQLENY	PEGKGEQEH	CSLYQLENYPEGKGEQEH		CMGA	8309/247050	0.996	47529	0.807	-0.030436	-2.3	0.3333
HIP_47530	SLQPLAL	QVELGGGPG	SLQPLALQVELGGGPG		INSC	224/5184	0.997	47530	0.807	0.121391	1.1	0
HIP_47531	LYQLENYCN	FPMDMSDDY	LYQLENYCNFPMDMSDDY		SCG2	2364/334890	1.000	47531	0.807	-0.187129	0.1333	0.1667
HIP_47532	SHLVEALYL	SNVSMASLG	SHLVEALYLSNVSMASLG		SCG1	16712/367830	0.993	47532	0.807	0.200424	1.0333	0.1667
HIP_47533	GAGSLQPLA	GEEENELTL	GAGSLQPLAGEEENELTL		SCG1	2144/367830	1.000	47533	0.807	0.029509	-0.5667	0.3333
HIP_47534	HLCGSHLVE	FPSPEMIRA	HLCGSHLVEFPSPEMIRA		SCG2	12409/334890	0.998	47534	0.807	0.058567	0.8167	0.3333
HIP_47535	GAGSLQPLA	RESTENVPR	GAGSLQPLARESTENVPR		NPY	2882/49410	0.977	47535	0.807	0.122563	-0.8	0.3333
HIP_47536	HLVEALYLV	LYPSDSQV	HLVEALYLVLYPSDSQV		SCG1	12308/367830	0.997	47536	0.807	0.029581	1.2667	0.1667
HIP_47537	SLQPLAL	AEKFSQRG	SLQPLALAEKFSQRG		SCG1	32168/367830	0.957	47537	0.807	0.209457	0.6333	0
HIP_47538	CGERGFFY	ITRQRYGKR	CGERGFFYITRQRYGKR		NPY	5058/49410	0.931	47538	0.807	0.191353	0.6	0
HIP_47539	CSLYQLENY	DSKRWSKMD	CSLYQLENYDSKRWSKMD		CMGA	24672/247050	0.952	47539	0.807	0.042996	-2.75	0.1667
HIP_47540	RGFFYTPKT	GGKSGELEQ	RGFFYTPKTGGKSGELEQ		CMGA	12228/247050	0.992	47540	0.807	-0.040393	-1.8167	0.5
HIP_47541	CCTSICSLY	RKESKDQLS	CCTSICSLYRKESKDQLS		SCG2	34860/334890	0.985	47541	0.807	0.105096	-1.7	0.1667
HIP_47542	NQHLCGSHL	ETQNAFLNE	NQHLCGSHLETQNAFLNE		SCG1	22552/367830	0.985	47542	0.807	0.086186	-1.3167	0.1667
HIP_47543	CGERGFFY	SNNFGAILS	CGERGFFYSNNFGAILS		IAPP	2695/45018	0.982	47543	0.807	0.205973	-0.5833	0.1667
HIP_47544	AGSLQPLAL	PGPQLRRG	AGSLQPLALPGPQLRRG		CMGA	9882/247050	0.995	47544	0.807	0.227798	0.9667	0.5
HIP_47545	QCCTSICSL	PSPEMIRAL	QCCTSICSLPSPEMIRAL		SCG2	9241/334890	0.999	47545	0.807	0.165583	0.25	0.6667
HIP_47546	LCGSHLVEA	GSGRLQNGQ	LCGSHLVEAGSGRLQNGQ		SCG2	11719/334890	0.998	47546	0.807	0.206580	0.15	0.5
HIP_47547	GSLQPLALE	SSPDYNPYQ	GSLQPLALESSPDYNPYQ		SCG2	9401/334890	0.999	47547	0.807	0.157307	-0.1833	0.5
HIP_47548	VCGERGFFY	FLISGAEA	VCGERGFFYFLISGAEA		SCG2	14377/334890	0.998	47548	0.807	-0.058585	2.5667	0
HIP_47549	YLVCGERGF	KNFPMDMSD	YLVCGERGFKNFPMDMSD		SCG2	16901/334890	0.997	47549	0.807	-0.007161	-1.1167	0.1667
HIP_47550	HLVEALYL	EHSQQKEEE	HLVEALYLEHSQQKEEE		CMGA	10945/247050	0.994	47550	0.807	0.137653	-0.2	0.1667
HIP_47551	QHLCGSHLV	KGRHHRGRG	QHLCGSHLVKGRHHRGRG		SCG1	32493/367830	0.955	47551	0.807	0.233600	-0.6667	0.1667
HIP_47552	CCTSICSL	VFLIVLSVA	CCTSICSLVFLIVLSVA		IAPP	1630/45018	0.993	47552	0.807	-0.001299	2.7167	0.1667
HIP_47553	HLVEALYLV	HPQGAWKEL	HLVEALYLVHPQGAWKEL		SCG1	21685/367830	0.987	47553	0.807	0.178472	-0.2667	0.1667
HIP_47554	LYQLENYCN	AAGSGRLQN	LYQLENYCNAAGSGRLQN		SCG2	8300/334890	0.999	47554	0.807	-0.121468	0.15	0.1667
HIP_47555	PGAGSLQPL	EEGPTVVLN	PGAGSLQPLEEGPTVVLN		CMGA	5772/247050	0.998	47555	0.807	-0.014291	-1.45	0.3333
HIP_47556	QCCTSICSL	GDERILSIL	QCCTSICSLGDERILSIL		CMGA	8179/247050	0.996	47556	0.807	0.126231	-0.3167	0.3333
HIP_47557	NQHLCGSHL	DFYDSEEPV	NQHLCGSHLDFYDSEEPV		SCG1	15775/367830	0.994	47557	0.807	-0.082630	-0.3667	0.1667
HIP_47558	QHLCGSHLV	SALRHYIN	QHLCGSHLVSALRHYIN		NPY	3651/49410	0.965	47558	0.807	0.094209	1.6	0.1667
HIP_47559	YLVCGERGF	WEKKPFSED	YLVCGERGFWEKKPFSED		SCG1	25271/367830	0.979	47559	0.807	0.022362	-1.7333	0.1667
HIP_47560	CGERGFFY	GLFRGGKSG	CGERGFFYGLFRGGKSG		CMGA	10912/247050	0.994	47560	0.807	0.032319	1.75	0.1667

[illegible]

HIP_48024	GSLQPLALE	DILNLLGME	GSLQPLALEDILNLLGME	SCG2	15844/334890	0.997	48024	0.807	-0.051656	1.15
HIP_48025	ERGFFYTPK	QLAKELTAE	ERGFFYTPKQLAKELTAE	CMGA	9575/247050	0.995	48025	0.807	0.046313	-0.6833
HIP_48026	CGSHLVEAL	EEGENYQKG	CGSHLVEALEEGENYQKG	SCG1	13457/367830	0.996	48026	0.807	0.129340	-0.8833
HIP_48027	VCGERGFF	TSEVDKRR	VCGERGFFTSEVDKRR	SCG1	20007/367830	0.989	48027	0.807	0.100580	0.0333
HIP_48028	SHLVEALY	NDEMKRSGQ	SHLVEALYNDEMKRSGQ	SCG2	20419/334890	0.996	48028	0.807	0.197042	-1.0333
HIP_48029	EAEDLQVGQ	ENQSSQAEL	EAEDLQVGQENQSSQAEL	CMGA	5169/247050	0.998	48029	0.807	-0.151483	-1.7
HIP_48030	VEALYLVCG	NLITRQRYG	VEALYLVCGNLITRQRYG	NPY	1638/49410	0.990	48030	0.807	0.003384	1.85
HIP_48031	GSHLVEALY	QMDKARRH	GSHLVEALYQMDKARRH	SCG1	29401/367830	0.968	48031	0.807	0.159322	-0.1333
HIP_48032	PGAGSLQP	VFLIVLSVA	PGAGSLQPVFLIVLSVA	IAPP	784/45018	0.997	48032	0.807	0.063912	1.5833
HIP_48033	GGGPGAGSL	YGEEIKGYP	GGGPGAGSLYGEEIKGYP	SCG1	9615/367830	0.998	48033	0.807	-0.038853	-0.4333
HIP_48034	ERGFFYTPK	PLIFLISGA	ERGFFYTPKPLIFLISGA	SCG2	13318/334890	0.998	48034	0.807	-0.013191	0.0833
HIP_48035	QHLCGSHLV	GQLGIQEED	QHLCGSHLVGQLGIQEED	SCG2	5191/334890	1.000	48035	0.807	0.146724	0.7833
HIP_48036	HLCGSHLVE	GNNQAPGEE	HLCGSHLVEGNNQAPGEE	CMGA	9829/247050	0.995	48036	0.807	0.154317	-0.4833
HIP_48037	SLYQLENYC	EPGETQNAF	SLYQLENYCEPGETQNAF	SCG1	5413/367830	0.999	48037	0.807	0.089608	-1.3
HIP_48038	FVNQHLCG	GGGHSRERA	FVNQHLCGGGGHSRERA	SCG1	8550/367830	0.998	48038	0.807	0.169910	0.7833
HIP_48039	GSLQPLAL	EEPSSKDVM	GSLQPLALEEPSSKDVM	CMGA	9208/247050	0.996	48039	0.807	0.160072	0.1333
HIP_48040	TSICSLYQ	PLQWKSSHF	TSICSLYQPLQWKSSHF	SCG1	22976/367830	0.984	48040	0.807	0.029133	-0.3833
HIP_48041	VCGERGFF	ELQKIAEK	VCGERGFFELQKIAEK	SCG1	22125/367830	0.986	48041	0.807	0.047516	0.3333
HIP_48042	SICSLYQLE	KSQREDEEE	SICSLYQLEKSQREDEEE	SCG1	24896/367830	0.980	48042	0.807	-0.057804	-1.9
HIP_48043	VEALYLVCG	DFYDSEEPV	VEALYLVCGDFYDSEEPV	SCG1	12846/367830	0.996	48043	0.807	-0.020690	0.7167
HIP_48044	GSHLVEALY	PEPMQESK	GSHLVEALYPEPMQESK	CMGA	8104/247050	0.997	48044	0.807	0.171732	-0.4
HIP_48045	CGERGFFY	DLQDTKEN	CGERGFFYDLQDTKEN	SCG1	22126/367830	0.986	48045	0.807	0.119876	0.1833
HIP_48046	SHLVEALYL	KEESSPDY	SHLVEALYLKEESSPDY	SCG2	21535/334890	0.995	48046	0.807	0.210502	-0.7667
HIP_48047	CSLYQLENY	SREKSSQES	CSLYQLENYSREKSSQES	SCG1	34808/367830	0.946	48047	0.807	0.025173	-2.85
HIP_48048	AGSLQPLAL	DTEKWAEG	AGSLQPLALDTEKWAEG	SCG1	16721/367830	0.993	48048	0.807	0.164373	0.2833
HIP_48049	GERGFFYTP	KVIAYLKRL	GERGFFYTPKVIAYLKRL	SCG2	16784/334890	0.997	48049	0.807	-0.029549	0.2
HIP_48050	LYQLENYCN	LNQGSSQET	LYQLENYCNLNQGSSQET	SCG2	13102/334890	0.998	48050	0.807	-0.130680	-0.9167
HIP_48051	CSLYQLENY	YGEESEEER	CSLYQLENYYGEESEEER	SCG1	12969/367830	0.996	48051	0.807	-0.128377	-2.25
HIP_48052	LVEALYLVC	HTAEKRKRL	LVEALYLVCHTAEKRKRL	SCG1	30952/367830	0.962	48052	0.807	0.112617	1.4
HIP_48053	LVEALYLV	NENTKFEVR	LVEALYLVNENTKFEVR	SCG1	19242/367830	0.990	48053	0.807	0.183729	-0.6333
HIP_48054	PLALEGSLQ	TNTHPPASL	PLALEGSLQTNTHPPASL	CMGA	13238/247050	0.990	48054	0.807	-0.095521	-0.9
HIP_48055	GPGAGSLQP	KTSYFPNPY	GPGAGSLQPKTSYFPNPY	SCG2	27285/334890	0.992	48055	0.807	0.054381	-1.1167
HIP_48056	GERGFFYT	YGAGRSRSN	GERGFFYTYGAGRSRSN	SCG2	21601/334890	0.995	48056	0.807	0.011743	0.15
HIP_48057	GSHLVEALY	GDLQDTKEN	GSHLVEALYGDLQDTKEN	SCG1	4540/367830	0.999	48057	0.807	0.108994	0.7
HIP_48058	YLVCGERGF	IILEALRQA	YLVCGERGFIILEALRQA	SCG2	10053/334890	0.999	48058	0.807	0.104829	1.7833
HIP_48059	QHLCGSHLV	LISDLLMRE	QHLCGSHLVLISDLLMRE	NPY	2225/49410	0.985	48059	0.807	0.018775	2.05
HIP_48060	VEALYLVCG	EEQKLYTDD	VEALYLVCGEEQKLYTDD	SCG2	12970/334890	0.998	48060	0.807	0.026221	-0.7
HIP_48061	PLALEGSLQ	KPYALNSEK	PLALEGSLQKPYALNSEK	SCG2	26023/334890	0.993	48061	0.807	-0.072087	-1.2167
HIP_48062	FVNQHLCGS	REDSLEAGL	FVNQHLCGSREDSLEAGL	CMGA	14238/247050	0.988	48062	0.807	-0.053375	-1.7
HIP_48063	CGERGFF	PGWQAKRE	CGERGFFPGWQAKRE	CMGA	10923/247050	0.994	48063	0.807	0.064028	0.3833
HIP_48064	GAGSLQPLA	RQAENEPQS	GAGSLQPLARQAENEPQS	SCG2	23502/334890	0.994	48064	0.807	-0.037166	-0.3667
HIP_48065	GAGSLQPLA	EESEESNVS	GAGSLQPLAEESEESNVS	SCG1	7330/367830	0.999	48065	0.807	0.008846	-0.6333
HIP_48066	LYQLENYCN	SSPETLISD	LYQLENYCNSSPETLISD	NPY	647/49410	0.997	48066	0.807	0.079887	-0.9167
HIP_48067	VELGGGPGA	EESWDEEDK	VELGGGPGAEESWDEEDK	SCG1	5247/367830	0.999	48067	0.807	-0.094935	-1.3333
HIP_48068	QHLCGSHLV	SNALSKSSA	QHLCGSHLVSNALSKSSA	SCG1	22106/367830	0.986	48068	0.807	0.175535	0.3833
HIP_48069	AGSLQPLAL	DIYKANNI	AGSLQPLALDIYKANNI	SCG2	23199/334890	0.995	48069	0.807	0.109815	1.5167
HIP_48070	GSLQPLALE	GGEPRAYFM	GSLQPLALEGGEPRAYFM	SCG1	11170/367830	0.997	48070	0.807	0.186595	-0.3667
HIP_48071	GSHLVEALY	ENVPRTRLE	GSHLVEALYENVPRTRLE	NPY	2419/49410	0.983	48071	0.807	0.119443	0.25
HIP_48072	GSHLVEALY	PTVVLNPHP	GSHLVEALYPTVVLNPHP	CMGA	6713/247050	0.998	48072	0.807	0.061476	1.0333
HIP_48073	GSLQPLAL	NFGAILSST	GSLQPLALNFGAILSST	IAPP	1542/45018	0.994	48073	0.807	0.150788	1.3833
HIP_48074	AGSLQPLAL	GEETGSQE	AGSLQPLALGEETGSQE	SCG1	11993/367830	0.997	48074	0.807	0.173897	0.3333
HIP_48075	QVGQVELGG	NGQNGERAT	QVGQVELGGNGQNGERAT	SCG2	5808/334890	0.999	48075	0.807	-0.097921	-0.7333
HIP_48076	YLVCGERGF	LLMKVLEYL	YLVCGERGFLLMKVLEYL	SCG2	9355/334890	0.999	48076	0.807	0.114865	1.2333
HIP_48077	YLVCGERGF	LTGPNNQKR	YLVCGERGFLTGPNNQKR	SCG2	16332/334890	0.997	48077	0.807	-0.052258	0.1
HIP_48078	VEALYLVC	GVSVPLQQK	VEALYLVCGVSVPLQQK	SCG2	9967/334890	0.999	48078	0.807	0.178903	2.25
HIP_48079	LYQLENYC	EEEEEEATN	LYQLENYCEEEEEEATN	CMGA	9877/247050	0.995	48079	0.807	0.00469	-2.1333

HIP_48535	SHLVEALYL	EPGETQNA	SHLVEALYLEPGETQNA		SCG1	12075/367830	0.997	48535	0.806	0.212090	0.1333	0.3333	-2	17	-0.3706	-3	1	1	0
HIP_48536	YQLENYCN	EGENYQKGE	YQLENYCNEGENYQKGE		SCG1	14470/367830	0.995	48536	0.806	0.118284	-1.6167	0.1667	-3	17	-1.9882	-3	2	1	0
HIP_48537	QCCTSICSL	QGPTKADTE	QCCTSICSLQGPTKADTE		SCG1	7685/367830	0.999	48537	0.806	0.163960	0	0.5	-1	18	-0.3333	-1	0	0	0
HIP_48538	GSHLVEALY	LGELFNPYY	GSHLVEALYLGELFNPYY		SCG1	7431/367830	0.999	48538	0.806	0.064483	0.7	0.1667	-1	18	0.1778	-2	1	0	0
HIP_48539	CSLYQLENY	SLYPSDSQV	CSLYQLENYSLYPSDSQV		SCG1	14579/367830	0.995	48539	0.806	-0.097409	-1.1	0.1667	-2	18	-0.45	-2	2	0	0
HIP_48540	CGERGFFY	KVIAYLKRL	CGERGFFYKVIAYLKRL		SCG2	25808/334890	0.993	48540	0.806	0.052066	1.5167	0	3	17	0.1471	3	4	1	0
HIP_48541	GPGAGSLQP	RGFFYTPK	GPGAGSLQPRGFFYTPK		INSB	295/4968	0.951	48541	0.806	-0.027509	-0.5667	0.3333	2	17	-0.5824	2	2	1	0
HIP_48542	VELGGGPGA	ALAEAYPSK	VELGGGPGAALAEAYPSK		NPY	30/49410	1.000	48542	0.806	0.145843	1.2	0.3333	-1	18	0.0667	-1	0	0	1
HIP_48543	HLCGSHLV	EEVKTRHSE	HLCGSHLVEEVKTRHSE		SCG1	25937/367830	0.978	48543	0.806	0.143932	0.3333	0	2	17	-0.7471	-1	0	1	0
HIP_48544	VEALYLVCG	ESVFQELGK	VEALYLVCGESVFQELGK		SCG2	7589/334890	0.999	48544	0.806	-0.020637	1.0333	0.3333	-2	18	0.5722	-2	2	0	0
HIP_48545	QHLCGSHLV	DQTVLTEDE	QHLCGSHLVDQTVLTEDE		SCG1	13150/367830	0.996	48545	0.806	0.146521	-0.4833	0	-2	18	-0.4278	-4	0	0	0
HIP_48546	TSICSLYQ	HSTHYRASE	TSICSLYQHSTHYRASE		SCG1	33823/367830	0.950	48546	0.806	0.118340	-0.95	0.1667	2	17	-0.7353	0	2	1	0
HIP_48547	LVEALYLV	EEEEEEEA	LVEALYLVEEEEEEEA		CMGA	7468/247050	0.997	48547	0.806	0.090852	-0.6333	0	-8	16	-0.3688	-8	1	0	5
HIP_48548	LVEALYLV	EEEEEEEA	LVEALYLVEEEEEEEA		CMGA	7469/247050	0.997	48548	0.806	0.090852	-0.6333	0	-8	16	-0.3688	-8	1	0	5
HIP_48549	PGAGSLQPL	KTSYFPNPY	PGAGSLQPLKTSYFPNPY		SCG2	27332/334890	0.992	48549	0.806	0.061340	-1.1167	0.3333	1	18	-0.6	1	1	0	0
HIP_48550	HLVEALYLV	RADEPQWSL	HLVEALYLVRADEPQWSL		SCG1	25574/367830	0.979	48550	0.806	0.168760	0.0833	0	-1	18	0.05	-2	1	0	0
HIP_48551	CSLYQLENY	KTPGRAGTE	CSLYQLENYKTPGRAGTE		SCG2	24354/334890	0.994	48551	0.806	-0.018862	-2.4167	0.1667	0	18	-0.9833	0	1	0	0
HIP_48552	QHLCGSHLV	EESEEGEED	QHLCGSHLVEESEEGEED		SCG1	10494/367830	0.997	48552	0.806	0.126087	-0.5	0.1667	-5	18	-1.25	-7	0	0	0
HIP_48553	VEALYLVCG	PGRAGTEAL	VEALYLVCGPGRAGTEAL		SCG2	11730/334890	0.998	48553	0.806	0.123786	-0.0333	0.5	-1	18	0.6333	-1	1	0	0
HIP_48554	PGAGSLQPL	GYPEEKKEE	PGAGSLQPLGYPEEKKEE		CMGA	3034/247050	0.999	48554	0.806	-0.092053	-0.7667	0.5	-2	18	-1.3333	-2	1	0	0
HIP_48555	VCGERGFFY	IPPEDLIE	VCGERGFFYIPPEDLIE		SCG2	2885/334890	1.000	48555	0.806	0.084825	0.9333	0.3333	-3	17	0.0765	-3	3	1	0
HIP_48556	CGERGFFYT	PVSKRFPV	CGERGFFYTPVSKRFPV		SCG2	17792/334890	0.997	48556	0.806	0.061845	0.4333	0.3333	2	17	-0.2294	2	3	1	0
HIP_48557	VCGERGFF	QEEDLRKES	VCGERGFFQEEDLRKES		SCG2	18663/334890	0.997	48557	0.806	0.047645	-0.8833	0.1667	-2	17	-1.1412	-2	1	0	0
HIP_48558	QHLCGSHLV	PPEDLIEML	QHLCGSHLVPPEDLIEML		SCG2	10043/334890	0.999	48558	0.806	0.188102	-0.3167	0.3333	-1	18	0.1944	-3	0	0	0
HIP_48559	VEALYLVCG	QKYPGPQAE	VEALYLVCGQKYPGPQAE		CMGA	19250/247050	0.975	48559	0.806	0.051956	-0.4	0.1667	-1	18	-0.1333	-1	2	0	0
HIP_48560	SLYQLENYC	LLMKVLEYL	SLYQLENYCLLMKVLEYL		SCG2	10142/334890	0.999	48560	0.806	-0.045766	1.2	0	-1	18	0.4889	-1	1	0	0
HIP_48561	GERGFFYTP	NFPMDMSDD	GERGFFYTPNFPMDMSDD		SCG2	5178/334890	1.000	48561	0.806	-0.170038	-0.9833	0.3333	-3	18	-0.9222	-3	4	0	0
HIP_48562	LVCGERGFF	RSNQLPKA	LVCGERGFFRSNQLPKA		SCG2	28123/334890	0.992	48562	0.806	0.142703	-0.6	0.3333	2	17	-0.2882	2	2	1	0
HIP_48563	FVNQHLCGS	RAYFMSDTR	FVNQHLCGSRAYFMSDTR		SCG1	20929/367830	0.988	48563	0.806	0.070650	-0.45	0.3333	2	18	-0.3833	1	2	0	0
HIP_48564	HLVEALYLV	KAEKGREH	HLVEALYLVKAEKGREH		SCG2	30406/334890	0.990	48564	0.806	0.204736	0.1833	0	2	17	-0.4412	0	1	1	0
HIP_48565	YLVCGERGF	QKENGDESH	YLVCGERGFQKENGDESH		SCG2	24730/334890	0.994	48565	0.806	0.013213	-2.1667	0.1667	-1	18	-1.2556	-2	1	0	0
HIP_48566	SLYQLENYC	SSSRGEAGA	SLYQLENYCSSSRGEAGA		SCG1	10041/367830	0.998	48566	0.806	0.116920	-0.7833	0.5	-1	18	-0.6333	-1	1	0	1
HIP_48567	SICSLYQLE	QESGEETGS	SICSLYQLEQESGEETGS		SCG1	20888/367830	0.988	48567	0.806	-0.082411	-1.8333	0.1667	-4	18	-0.6889	-4	1	0	0
HIP_48568	SICSLYQLE	SQESGEETG	SICSLYQLESQESGEETG		SCG1	20889/367830	0.988	48568	0.806	-0.082411	-1.8333	0.1667	-4	18	-0.6889	-4	1	0	0
HIP_48569	HLVEALYLV	EENELTLN	HLVEALYLVEENELTLN		SCG1	15927/367830	0.994	48569	0.806	0.143129	-0.6333	0	-3	17	0.1765	-4	1	1	0
HIP_48570	GSLQPLAL	EQEEERLS	GSLQPLALEQEEERLS		CMGA	10617/247050	0.994	48570	0.806	0.144311	-0.1833	0	-3	16	-0.7563	-3	0	0	1
HIP_48571	VNQHLCGSH	SPDYNPYQG	VNQHLCGSHSPDYNPYQG		SCG2	14849/334890	0.998	48571	0.806	-0.096694	-1.7167	0.6667	1	18	-1.2	-1	1	0	0
HIP_48572	HLVEALYLV	ALSLLVCLG	HLVEALYLVALSLLVCLG		NPY	1977/49410	0.987	48572	0.806	0.015185	1.9167	0.1667	0	18	2.0056	-1	1	0	0
HIP_48573	GSLQPLALE	KVIAYLKRL	GSLQPLALEKVIAYLKRL		SCG2	27114/334890	0.992	48573	0.806	0.022154	1.15	0	2	18	0.4389	2	0	0	0
HIP_48574	HLVEALYLV	AENEPQSA	HLVEALYLVAENEPQSA		SCG2	13581/334890	0.998	48574	0.806	0.116391	0.25	0	-2	17	0.0471	-3	1	1	0
HIP_48575	HLCGSHLVE	RLVNAAGSG	HLCGSHLVERLVNAAGSG		SCG2	28071/334890	0.992	48575	0.806	0.025039	1.3333	0	2	18	0.2889	0	0	0	0
HIP_48576	IVEQCCTSI	EEDNRDSSM	IVEQCCTSIEEDNRDSSM		CMGA	11241/247050	0.993	48576	0.806	-0.040807	-1.25	0.1667	-4	18	-0.6667	-4	0	0	0
HIP_48577	VCGERGFFY	GLSQGLVD	VCGERGFFYGLSQGLVD		CMGA	4913/247050	0.998	48577	0.806	0.154743	1.15	0.3333	-1	17	0.3176	-1	3	1	0
HIP_48578	HLCGSHLVE	KGREHIAKR	HLCGSHLVEKGREHIAKR		SCG2	33146/334890	0.987	48578	0.806	0.167963	-0.7167	0.1667	5	18	-0.8	2	0	0	0
HIP_48579	CSLYQLENY	PEDLIEMLK	CSLYQLENYPEDLIEMLK		SCG2	19868/334890	0.996	48579	0.806	-0.088709	-2.8167	0.1667	-3	18	-0.3222	-3	1	0	0
HIP_48580	LVEALYLVC	SPDYNPYQ	LVEALYLVCSPDYNPYQ		SCG2	14314/334890	0.998	48580	0.806	0.186275	0.7667	0.3333	-2	17	0.1294	-2	2	1	0
HIP_48581	RGFFYTPKT	FKRTNEIVE	RGFFYTPKTFKRTNEIVE		SCG2	30300/334890	0.990	48581	0.806	0.019130	-1.9667	0.1667	2	18	-0.8667	2	2	0	0
HIP_48582	LYQLENYCN	LIVLSVALN	LYQLENYCNLIVLSVALN		IAPP	1346/85018	0.994	48582	0.806	-0.046009	1.7	0	-1	18	0.85	-1	1	0	0
HIP_48583	PLALEGSLQ	YGFRGPGPQ	PLALEGSLQYGFRGPGPQ		CMGA	4362/247050	0.999	48583	0.806	-0.102871	0.1	0.3333	0	18	-0.4167	0	2	0	0
HIP_48584	VEALYLVC	NYDWWEKK	VEALYLVCNYDWWEKK		SCG1	21634/367830	0.987	48584	0.806	0.121109	0.3667	0	-1	16	-0.3688	-1	3	0	0
HIP_48585	LYLVCGERG	GRGGEPRAY	LYLVCGERGGRGGEPRAY		SCG1	21035/367830	0.988	48585	0.806	-0.062176	-2.2833	0.5	1	18	-0.5889	1	0	0	0
HIP_48586	CGSHLVEAL	KVIAYLKRL	CGSHLVEALKVIAYLKRL		SCG2	26211/334890	0.993	48586	0.806	0.021041	1.15	0	3	18	0.7056	2	0	0	0
HIP_48587	GAGSLQPLA	EESNVSMAS	GAGSLQPLAEESNVSMAS		SCG1	10218/367830	0.998	48587	0.806	0.034791	-0.6333	0.3333	-2	18	-0.0278	-2	0	0	0
[illegible]																			

HIP_49046	GPGAGSLQP	DWNPVEEKI	GPGAGSLQPDWNPVEEKI		SCG2	14333/334890	0.998	49046	0.806	-0.059471	-1.5333	0.1667	-2	18	-0.8222	-2	1	0	0
HIP_49047	CGERGFFY	KSGEATDGA	CGERGFFYKSGEATDGA		CMGA	13850/247050	0.989	49047	0.806	0.210257	-0.1333	0.3333	-1	17	-0.6824	-1	3	1	0
HIP_49048	GGPGAGSLQ	EEENELTLN	GGPGAGSLQEEENELTLN		SCG1	18089/367830	0.992	49048	0.806	-0.079423	-1.8333	0.1667	-4	18	-0.8889	-4	0	0	1
HIP_49049	CGERGFFY	LGNKRLGL	CGERGFFYLGNKRLGL		NPY	2405/49410	0.983	49049	0.806	0.170416	0.7	0.1667	2	16	-0.2063	2	3	0	0
HIP_49050	SHLVEALYL	RNQLLQKE	SHLVEALYLRNQLLQKE		SCG2	28327/334890	0.991	49050	0.806	0.233646	-0.8667	0	1	17	-0.3647	0	1	1	0
HIP_49051	GSLQPLALE	FRARAYGFR	GSLQPLALEFRARAYGFR		CMGA	24790/247050	0.951	49051	0.806	0.030630	0.3667	0	2	18	-0.1444	2	1	0	0
HIP_49052	PGAGSLQPL	KSGYPKTPG	PGAGSLQPLKSGYPKTPG		SCG2	20084/334890	0.996	49052	0.806	0.094848	-1.0667	0.5	2	18	-0.75	2	1	0	0
HIP_49053	ERGFFYTP	EEEEEEEA	ERGFFYTPEEEEEEEA		CMGA	10666/247050	0.994	49053	0.806	-0.043184	-2.35	0.1667	-7	16	-1.8187	-7	3	0	5
HIP_49054	ERGFFYTP	EEEEEEEA	ERGFFYTPEEEEEEEA		CMGA	10667/247050	0.994	49054	0.806	-0.043184	-2.35	0.1667	-7	16	-1.8187	-7	3	0	5
HIP_49055	CGERGFFY	RQYWDEDLL	CGERGFFYRQYWDEDLL		SCG2	26835/334890	0.993	49055	0.806	0.136618	-0.8333	0	-2	17	-0.8882	-2	5	1	0
HIP_49056	CTSICSLYQ	NPYNQEKV	CTSICSLYQNPYNQEKV		SCG2	21357/334890	0.995	49056	0.806	0.057242	-1.2333	0.1667	0	17	-0.6118	0	2	1	0
HIP_49057	TSICSLYQL	KMDQLAKEL	TSICSLYQLKMDQLAKEL		CMGA	26195/247050	0.943	49057	0.806	0.125215	-1.0833	0	0	18	0.0278	0	1	0	0
HIP_49058	EAEDLQVG	GERGFFYTP	EAEDLQVGGERGFFYTP		INSB	170/4968	0.981	49058	0.806	-0.069000	-1.35	0.3333	-3	17	-0.6706	-3	1	1	0
HIP_49059	GPGAGSLQP	NQVKRVPGQ	GPGAGSLQPNQVKRVPGQ		SCG2	26354/334890	0.993	49059	0.806	0.011478	-0.6833	0.1667	2	18	-0.8667	2	0	0	0
HIP_49060	GAGSLQPLA	DDIYKANNI	GAGSLQPLADDIYKANNI		SCG2	13670/334890	0.998	49060	0.806	-0.028042	0.25	0.1667	-1	18	-0.2167	-1	1	0	0
HIP_49061	HLCGSHLVE	TPQSLATLE	HLCGSHLVETPQSLATLE		SCG2	17718/334890	0.997	49061	0.806	0.156171	-0.2167	0.1667	0	18	0.1	-2	0	0	0
HIP_49062	VCGERGFFY	EGLSQGLV	VCGERGFFYEGLSQGLV		CMGA	9291/247050	0.996	49062	0.806	0.163572	0.7	0.1667	-1	17	0.3176	-1	3	1	0
HIP_49063	GSLQPLALE	ARSETHAAG	GSLQPLALEARSETHAAG		SCG1	33343/367830	0.952	49063	0.806	0.060864	-0.2333	0.1667	0	18	-0.2389	-1	0	0	0
HIP_49064	CCTSICSLY	ENYQKGER	CCTSICSLYENYQKGER		SCG1	27525/367830	0.973	49064	0.806	0.008875	-1.1	0.1667	0	17	-0.7	0	2	1	0
HIP_49065	LQPLALEGS	QEEDLRKES	LQPLALEGSQEEDLRKES		SCG2	18176/334890	0.997	49065	0.806	-0.100490	-2.5333	0.3333	-3	18	-1.0833	-3	0	0	0
HIP_49066	VEALYLVC	QFPPMYEE	VEALYLVCQFPPMYEE		SCG2	13448/334890	0.998	49066	0.806	0.118468	1.3667	0.1667	-3	16	0.325	-3	2	0	0
HIP_49067	CSLYQLENY	AEFPDFYDS	CSLYQLENYAEFPDFYDS		SCG1	15002/367830	0.995	49067	0.806	-0.099818	-1.2	0	-4	18	-0.5889	-4	2	0	0
HIP_49068	HLVEALYLV	GILKLQVF	HLVEALYLVGILKLQVF		IAPP	1456/45018	0.994	49068	0.806	0.047552	2.4333	0.1667	1	17	1.4647	0	1	1	0
HIP_49069	PLALEGSLQ	FLVHSSNN	PLALEGSLQFLVHSSNN		IAPP	1022/45018	0.996	49069	0.806	0.072907	1.7167	0.1667	0	17	0.1412	-1	1	1	0
HIP_49070	YLVCGERGF	PLIFLISGA	YLVCGERGFPLIFLISGA		SCG2	8923/334890	0.999	49070	0.806	-0.034425	0.7667	0.3333	0	18	1.2	0	2	0	0
HIP_49071	CSLYQLENY	RQYWDEDLL	CSLYQLENYRQYWDEDLL		SCG2	26142/334890	0.993	49071	0.806	-0.062024	-2.9333	0	-3	18	-0.9389	-3	3	0	0
HIP_49072	GERGFFYTP	LIPLIFLIS	GERGFFYTPLIPLIFLIS		SCG2	9660/334890	0.999	49072	0.806	-0.099922	0.5167	0.3333	0	18	1.0278	0	3	0	0
HIP_49073	CTSICSLYQ	NLQIPPEDL	CTSICSLYQNLQIPPEDL		SCG2	12725/334890	0.998	49073	0.806	-0.103953	-0.7	0	-2	18	0.0611	-2	1	0	0
HIP_49074	GERGFFYT	DNMNDNFLE	GERGFFYTDNMNDNFLE		SCG1	21518/367830	0.987	49074	0.806	0.025853	-0.7167	0	-3	17	-1.0412	-3	3	1	0
HIP_49075	LYQLENYCN	SEEEPEYGE	LYQLENYCNSEEEPEYGE		SCG1	11340/367830	0.997	49075	0.806	0.035042	-1.6833	0.1667	-6	18	-1.5611	-6	1	0	1
HIP_49076	LVEALYLV	EEKHLEEPG	LVEALYLVEEKHLEEPG		SCG1	18903/367830	0.991	49076	0.806	0.140842	-0.7	0	-3	17	-0.1471	-4	1	1	0
HIP_49077	GIVEQCCTS	EPERELDLP	GIVEQCCTSEPERELDLP		SCG2	11347/334890	0.999	49077	0.806	-0.050939	-1.2667	0.3333	-4	18	-0.5167	-4	0	0	0
HIP_49078	CTSICSLYQ	MDMSDDYET	CTSICSLYQMDMSDDYET		SCG2	11658/334890	0.998	49078	0.806	-0.126221	-0.1167	0	-4	18	-0.3778	-4	1	0	0
HIP_49079	GAGSLQPLA	QELGEYLAR	GAGSLQPLAQELGEYLAR		SCG2	11303/334890	0.999	49079	0.806	-0.072458	0.1333	0.1667	-1	18	-0.1556	-1	0	0	0
HIP_49080	DLQVGQVEL	DILNLLGME	DLQVGQVELDILNLLGME		SCG2	15638/334890	0.998	49080	0.806	-0.037571	1.55	0	-4	18	0.4722	-4	0	0	0
HIP_49081	CCTSICSLY	ALAVDGAGK	CCTSICSLYALAVDGAGK		CMGA	9086/247050	0.986	49081	0.806	-0.019117	1.5167	0.1667	0	18	0.9667	0	1	0	0
HIP_49082	PLALEGSLQ	GEEDATSEV	PLALEGSLQGEEDATSEV		SCG1	6171/367830	0.999	49082	0.806	-0.081831	-1.3167	0.3333	-5	18	-0.3611	-5	0	0	0
HIP_49083	AGSLQPLAL	AHGYGEES	AGSLQPLALAHGYGEES		SCG1	15325/367830	0.995	49083	0.806	0.125140	1.2667	0.1667	-1	17	-0.1529	-2	1	1	0
HIP_49084	GSHLVEALY	AEGDSEGLS	GSHLVEALYAEGDSEGLS		CMGA	2916/247050	0.999	49084	0.806	0.089901	0.3667	0.1667	-3	18	-0.1611	-4	1	0	0
HIP_49085	QCCTSICSL	SDTLSKPSP	QCCTSICSLSDTLSKPSP		CMGA	10500/247050	0.998	49085	0.806	0.154229	0.0833	0.3333	0	18	0.0056	0	0	0	0
HIP_49086	HLVEALYL	QELGEYLAR	HLVEALYLQELGEYLAR		SCG2	17262/334890	0.997	49086	0.806	0.142297	0.5167	0	-1	17	0.1235	-2	1	1	0
HIP_49087	PLALEGSLQ	EEEGENYQK	PLALEGSLQEEEGENYQK		SCG1	18896/367830	0.991	49087	0.806	-0.063829	-1.8333	0.1667	-4	18	-1.2889	-4	0	0	1
HIP_49088	CGERGFFY	AGEEAVPE	CGERGFFYAGEEAVPE		CMGA	8026/247050	0.997	49088	0.806	0.129858	0.3667	0.1667	-3	16	-0.4188	-3	3	0	0
HIP_49089	QHLCGSHLV	RGSEEYRAP	QHLCGSHLVRGSEEYRAP		SCG1	20920/367830	0.988	49089	0.806	0.232866	-0.15	0.3333	2	18	-0.8389	0	0	0	0
HIP_49090	VNQHLCGSH	PSPEMIRAL	VNQHLCGSHPSPEMIRAL		SCG2	12578/334890	0.998	49090	0.806	-0.076545	-1.4	0.8333	2	18	-0.2278	0	0	0	0
HIP_49091	NQHLCGSHL	LLMRESTEN	NQHLCGSHLLLMRESTEN		NPY	1877/49410	0.988	49091	0.806	0.041863	1.55	0.1667	1	18	-0.6389	-1	0	0	1
HIP_49092	GPGAGSLQP	EEDKRNYPS	GPGAGSLQPEEDKRNYPS		SCG1	24056/367830	0.982	49092	0.806	-0.007664	-1.9667	0.1667	-1	18	-1.6222	-1	0	0	0
HIP_49093	GERGFFYTP	FEDELSEVL	GERGFFYTPFEDELSEVL		CMGA	8274/247050	0.996	49093	0.806	-0.148257	-1.3	0.1667	-4	18	-0.3889	-4	4	0	0
HIP_49094	CGERGFFYT	DKQYSSHH	CGERGFFYTDKQYSSHH		SCG1	33746/367830	0.950	49094	0.806	0.155492	-1.6833	0	2	17	-1.3471	0	4	1	0
HIP_49095	NQHLCGSHL	QGLFRGGKS	NQHLCGSHLQGLFRGGKS		CMGA	15642/247050	0.985	49095	0.806	0.031149	-0.05	0.3333	4	18	-0.6556	2	1	0	0
HIP_49096	CGSHLVEAL	YQKGERGED	CGSHLVEALYQKGERGED		SCG1	37017/367830	0.935	49096	0.806	0.050496	-1.1	0	-1	18	-0.9056	-2	1	0	0
HIP_49097	DLQVGQVEL	YPEIINSNQ	DLQVGQVELYPEIINSNQ		SCG2	16777/334890	0.997	49097	0.806	0.047401	-0.3167	0.1667	-3	18	-0.3944	-3	1	0	0
HIP_49098	HLCGSHLVE	EGDSEGLSQ	HLCGSHLVEEGDSEGLSQ		CMGA	7913/247050	0.997	49098	0.806	0.138900	-0.4833	0.1667	-2	18	-0.5222	-4	0	0	0
HIP_49099	HLVEALYL	EEDATSEVD	HLVEALYLEEDATSEVD		SCG1	14761/367830	0.995	49099	0.806	0.113650	-0.7	0	-5	17	-0.2118	-6	1	1	0
HIP_49100	QCCTSICSL	ALQGAKERA	QCCTSICSLALQGAKERA		CMGA	16272/247050	0.984	49100	0.806	0.025551	1.2667	0.1667	1	18	0.1889	1	0	0	0
HIP_49101	VEALYLVC	ENGDESHLP	VEALYLVCENGDESHLP		SCG2	12668/334890	0.998	49101	0.806	0.200670	0.5167	0.1667	-3	17	-0.0412	-4	1	1	0
HIP_49102	HLVEALYLV	NTYGKRNA	HLVEALYLVNTYGKRNA		IAPP	3478/45018	0.969	49102	0.806	0.162803	0.2	0	2	17	-0.1412	1	2	1	0
HIP_49103	HLCGSHLVE	QEEEQIEQA	HLCGSHLVEQEEEQIEQA		SCG2	16982/334890	0.997	49103	0.806	0.077775	-1	0	-3	18	-0.8333	-5	0	0	1

HIP_49557	CCTSICSLY	FPDFYDSEE	CCTSICSLYFPDFYDSEE		SCG1	7942/367830	0.998	49557	0.805	-0.102985	-0.1	0.3333	-4	18	0.0056	-4	3	0	0	0.000000	-1.3333	-1	0.05	3.475000	0.1111	3.3639	1	0	1	0	1.3333	-0.3364	-4	0	1	0	0	0.4269	0.9855	0.939	0.1667	0.1823	0.8817	-0.0427	0.0025	-0.0667
HIP_49558	CCTSICSLY	LLLCAGQVT	CCTSICSLYLLLCAGQVT		CMGA	9217/247050	0.996	49558	0.805	-0.008414	2.1833	0.1667	0	18	1.5278	0	1	0	1	0.000000	3.2333	0	-0.0667	3.650000	0	3.65	0	0	0	0	0	7.9692	3.2333	0	1	0	0	0.3051	1.1703	0.6845	0.6667	0.0931	0.4685	0.6662	0.0044	-0.2156
HIP_49559	VNQHLCGSH	EEEGENYQK	VNQHLCGSHEEEGENYQK		SCG1	21926/367830	0.986	49559	0.805	-0.098068	-2.4833	0.3333	-1	18	-1.7056	-3	0	0	1	0.000000	-2.0333	-3	0	0.950000	0.3333	0.6167	0	1	0	1	6.1	-1.5314	0	0	0	1	0	0.5885	1.1419	0.9712	-0.6667	0.3463	0.9431	-1.4613	0	0
HIP_49560	GSLQPLA	YSALRHYI	GSLQPLAYSALRHYI		NPY	3144/49410	0.974	49560	0.805	0.038702	0.6167	0.3333	2	15	0.14	1	1	1	0	0.000000	-1.4333	0	0	0.450000	0.1111	0.3389	1	0	1	0	0	0.209	-1.4333	1	0	0	0	0.3885	1.0986	0.9078	0.5	0.1509	0.8241	0.2396	0	0
HIP_49561	PGAGSLQPL	TRLEDPAMW	PGAGSLQPLTRLEDPAMW		NPY	2581/49410	0.981	49561	0.805	-0.054242	-0.45	0.1667	-1	18	-0.3389	-1	0	0	0	0.000000	-0.0333	1	-0.0167	0.000000	0.2222	-0.2222	1	0	1	0	-0.0333	0.1	0	1	0	0	0	0.4551	1.0219	0.8945	0.1667	0.2071	0.8001	-0.2048	0.0003	0.0006
HIP_49562	SHLVEALY	SPDYNPYQ	SHLVEALYSPDYNPYQ		SCG2	13592/334890	0.998	49562	0.805	0.183474	-0.2667	0.3333	-1	16	-0.7688	-2	2	0	0	0.000000	-3.4	-1	0.1833	2.000000	0.2222	1.7778	1	0	1	0	3.4	-0.4741	-6.8	0	1	0	0	0.4269	1.0272	0.949	0.1667	0.1823	0.9006	-0.1138	0.0036	-0.6233
HIP_49563	CTSICSLYQ	SALRHYIN	CTSICSLYQSALRHYIN		NPY	3389/49410	0.970	49563	0.805	-0.019378	0.6333	0.1667	2	17	0.1765	1	1	1	0	0.000000	1.9333	0	0.0167	0.625000	0.1111	0.5139	0	0	0	0	0	0.3255	1.9333	0	1	0	0	0.3718	1.1636	0.7912	0.5	0.1382	0.6259	0.2355	0.0003	0.0322
HIP_49564	LVCGERGFF	GPQAEGDS	LVCGERGFFGPQAEGDS		CMGA	6526/247050	0.998	49564	0.805	0.108689	-0.05	0.5	-2	17	-0.2706	-2	2	1	0	0.000000	-3.5667	0	0.1167	1.150000	0.2222	0.9278	1	2	1	1	0	-0.0464	-7.1333	1	0	0	0	0.5167	0.8467	1.1167	0.3333	0.2669	1.2469	-0.0258	0.0036	-0.4161
HIP_49565	AGSLQPLA	GGGPGAGSL	AGSLQPLAGGGPGAGSL		INSC	67/5184	0.999	49565	0.805	0.167637	0.4667	0.6667	0	17	0.3588	0	0	1	1	0.000000	-1.7333	0	0.1833	0.450000	0	0.45	1	3	1	1	0	0.21	0	1	0	0	0	0.5333	0.8033	1.1967	0.3333	0.2844	1.432	0.2489	0.0036	-0.3178
HIP_49566	YLVCGERGF	YPEIINSNQ	YLVCGERGFYPEIINSNQ		SCG2	13529/334890	0.998	49566	0.805	-0.083160	-1.4167	0.3333	-1	18	-0.3056	-1	2	0	0	0.000000	-1.4333	-2	-0.0167	1.125000	0.3333	0.7917	1	1	1	1	2.8667	-1.1215	-2.8667	1	0	0	0	0.5103	0.9705	1.029	-0.1667	0.2604	1.0588	-0.7229	0.0003	0.0239
HIP_49567	SLYQLENYC	HSREKSSQE	SLYQLENYCHSREKSSQE		SCG1	37310/367830	0.933	49567	0.805	0.060440	-1.8	0.1667	0	18	-1.5722	-1	1	0	0	0.000000	-2.0667	1	0.0667	0.950000	0.3333	0.6167	0	0	0	0	-2.0667	-1.11	-2.0667	0	0	0	0	0.4872	0.926	1.0646	-0.3333	0.2373	1.1334	-0.8769	0.0044	-0.1378
HIP_49568	HLVEALYLV	LQVRGYPEE	HLVEALYLVLQVRGYPEE		CMGA	10605/247050	0.994	49568	0.805	0.000162	1.8667	0	-1	18	0.1833	-2	1	0	0	1.000000	-0.7333	0	0.05	2.350000	0.1111	2.2389	0	1	0	0	0	4.1793	-0.7333	1	0	0	0	0.3051	1.1419	0.6478	0.6667	0.0931	0.4197	0.5696	0.0025	-0.0367
HIP_49569	SLQPLALE	QWPERKLKH	SLQPLALEQWPERKLKH		SCG2	33180/334890	0.987	49569	0.805	0.125930	-0.65	0.1667	2	17	-1.0235	1	1	1	0	0.000000	-2.7	1	0.0667	0.950000	0.3333	0.6167	1	0	1	0	-2.7	-0.4008	-2.7	0	0	1	0	0.4551	1.1603	0.8845	0.3333	0.2071	0.7823	-0.2958	0.0044	-0.18
HIP_49570	CGERGFFY	KGQPRSQE	CGERGFFYKGQPRSQE		SCG1	25383/367830	0.979	49570	0.805	0.218792	-0.5833	0.1667	1	16	-1.4812	1	3	0	0	0.000000	-4.0333	1	0.1833	0.700000	0.2222	0.4778	0	1	0	1	-4.0333	-0.2787	-12.1	0	1	0	0	0.4269	1.0088	0.9156	0.1667	0.1823	0.8384	-0.249	0.0036	-0.7394
HIP_49571	VCGERGFF	MLGNKRLGL	VCGERGFFMLGNKRLGL		NPY	1822/49410	0.989	49571	0.805	-0.006662	1.75	0.3333	2	17	0.2412	2	2	1	0	1.000000	0.0333	0	0	1.650000	0.2222	1.4278	0	2	0	1	0	2.4986	0.0667	1	0	0	0	0.3667	0.9833	0.8833	0.6667	0.1344	0.7803	0.6417	0	0
HIP_49572	LYQLENYCN	VSVPLQQKE	LYQLENYCNVSVPLQQKE		SCG2	9958/334890	0.999	49572	0.805	-0.136416	0.8833	0.1667	-1	18	-0.6167	-1	1	0	0	0.000000	3.3	0	0.0167	1.675000	0.1111	1.5639	0	0	0	0	0	1.3814	3.3	0	1	0	0	0.4103	0.9388	0.929	0.3333	0.1683	0.863	0.3624	0.0003	0.055
HIP_49573	CTSICSLYQ	NFPMDMSDD	CTSICSLYQNFPMDMSDD		SCG2	2265/334890	1.000	49573	0.805	-0.092602	-0.55	0.1667	-3	18	-0.2	-3	2	0	0	0.000000	-0.4333	0	0.0667	1.100000	0	1.1	1	0	1	0	0	-0.605	-0.8667	0	1	0	0	0.4551	0.9786	1.0112	0.3333	0.2071	1.0224	-0.2503	0.0044	-0.0289
HIP_49574	TSICSLYQL	EVKTRHSEK	TSICSLYQLEVKTRHSEK		SCG1	43453/367830	0.898	49574	0.805	0.119138	-0.7	0	2	18	-0.6833	1	1	0	0	0.000000	-0.7333	0	0.05	0.000000	0.2222	-0.2222	0	0	0	0	0	0.1556	-0.7333	1	0	0	0	0.4103	1.1705	0.7556	0	0.1683	0.571	-0.2872	0.0025	-0.0367
HIP_49575	CGERGFFY	ALAVDGAGK	CGERGFFYALAVDGAGK		CMGA	13352/247050	0.990	49575	0.805	-0.022964	1.95	0	0	17	0.1882	0	3	1	0	0.000000	1.0333	0	0	0.950000	0.1111	0.8389	0	0	0	0	0	1.6358	3.1	0	1	0	0	0.2385	1.2703	0.6545	0.8333	0.0569	0.4284	0.465	0	0
HIP_49576	LVCGERGF	FLVHSSN	LVCGERGFFLVHSSN		IAPP	1615/45018	0.993	49576	0.805	0.076319	1.45	0.1667	1	15	0.4667	0	2	1	0	1.000000	4.3	-1	-0.1333	0.700000	0.2222	0.4778	0	1	0	1	-4.3	0.6928	8.6	1	0	0	0	0.3218	1.0469	0.7678	0.5	0.1036	0.5895	0.4666	0.0178	-0.5733
HIP_49577	VEQCCTSIC	YGEESEEER	VEQCCTSICYGEESEEER		SCG1	4653/367830	0.999	49577	0.805	-0.027100	0.1667	0.3333	-5	18	-0.9333	-5	1	0	1	0.000000	-3.8	-1	0.0667	1.750000	0.2222	1.5278	0	1	0	1	3.8	0.2546	-3.8	0	0	0	0	0.4936	0.9888	0.954	0	0.2436	0.9101	0.0823	0.0044	-0.2533
HIP_49578	PLALEGSLQ	EEERGLEPG	PLALEGSLQEEERGLEPG		SCG1	18987/367830	0.991	49578	0.805	-0.059893	-1.8333	0.1667	-4	18	-0.7611	-4	0	0	1	0.000000	-3.3333	-3	0.0333	0.950000	0.5556	0.3944	0	0	0	0	10	-0.7231	0	0	1	0	0	0.5333	1.29	0.8167	-0.3333	0.2844	0.6669	-0.9778	0.0011	-0.1111
HIP_49579	GSLQPLAL	DEEDKRNYP	GSLQPLALDEEDKRNYP		SCG1	19235/367830	0.991	49579	0.805	0.146565	-0.1833	0	-2	17	-1.2882	-2	0	1	0	0.000000	-6.6333	-3	0.15	0.950000	0.5556	0.3944	0	0	0	0	19.9	-0.0723	0	1	0	0	0	0.3718	1.3486	0.6745	0	0.1382	0.4549	-0.0682	0.0225	-0.995
HIP_49580	VEQCCTSIC	TRQRYGKRS	VEQCCTSICTRQRYGKRS		NPY	5506/49410	0.917	49580	0.805	0.150685	-0.4167	0.1667	3	18	-0.9111	3	0	0	0	0.000000	-4.9667	1	0.0833	1.750000	0.2222	1.5278	0	0	0	0	-4.9667	-0.6366	0	1	0	0	0	0.4769	0.9772	0.904	0	0.2275	0.8172	-0.1987	0.0069	-0.4139
HIP_49581	AGSLQPLAL	PTLLLSLL	AGSLQPLALPTLLLSLL		SCG1	12651/367830	0.996	49581	0.805	0.144853	1.8167	0.1667	0	17	1.4471	0	0	1	1	0.000000	-2.6333	0	0.1167	1.400000	0	1.4	1	0	1	0	0	2.5433	0	1	0	0	0	0.35	1.125	0.7733	0.6667	0.1225	0.598	0.6358	0.0136	-0.3072
HIP_49582	VELGGGPGA	TPECRQVLK	VELGGGPGATPECRQVLK		SCG1	9189/367830	0.998	49582	0.805	-0.092228	-1	0.5	0	18	-0.2278	0	0	0	1	0.000000	-1.8667	-1	0.0167	0.625000	0.1111	0.5139	2	1	1	1	1.8667	-0.5139	0	1	0	0	0	0.5833	0.905	1.15	0	0.3403	1.3225	-0.5833	0.0003	-0.0311
HIP_49583	HLVEALYL	NQVKRVPGQ	HLVEALYLNQVKRVPGQ		SCG2	25711/334890	0.993	49583	0.805	0.203579	0.5833	0	2	17	-0.1824	1	1	1	0	0.000000	-3.0333	0	0.1	1.400000	0.2222	1.1778	0	0	0	0	0	0.687	-3.0333	1	0	0	0	0.3718	1.0769	0.8245	0.5	0.1382	0.6798	0.2169	0.01	-0.3033
HIP_49584	GSLQPLALE	EAKTHWLGA	GSLQPLALEEAKTHWLGA		SCG2	36932/334890	0.982	49584	0.805	0.065073	-0.5833	0	0	18	-0.1	-1	0	0	0	0.000000	-2.5667	1	0.05	0.950000	0.3333	0.6167	0	0	0	0	-2.5667	-0.3597	0	0	0	1	0	0.3718	1.3736	0.7012	0	0.1382	0.4916	-0.2169	0.0025	-0.1283
HIP_49585	GPGAGSLQP	ENENTKFEV	GPGAGSLQPENENTKFEV		SCG1	19458/367830	0.990	49585	0.805	-0.038289	-1.9667	0.1667	-2	18	-1.0111	-2	0	0	0	0.000000	-3.0667	-2	0.0333	0.000000	0.2222	-0.2222	1	0	1	0	6.1333	0.437	0	0	0	0	1	0.55	1.1117	1.0067	-0.1667	0.3025	1.0134	-1.0817	0.0011	-0.1022
HIP_49586	RGFFYTPKT	WLGAALSLI	RGFFYTPKTWLGAALSLI		SCG2	13601/334890	0.998	49586	0.805	-0.005970	-0.6167	0.3333	2	18	0.55	2	2	0	0	0.000000	2.9	-1	-0.0833	0.450000	0.1111	0.3389	1	1	1	1	-2.9	-0.209	5.8	0	1	0	0	0.5103	0.8738	1.049	0.1667	0.2604	1.1003	-0.3147	0.0069	-0.2417
HIP_49587	RGFFYTPKT	PKAAWIPHV	RGFFYTPKTPKAAWIPHV		SCG2	27701/334890	0.992	49587	0.805	0.010850	-1.65	0.3333	4	18	-0.3556	3	1	0	0	0.000000	0.8333	0	-0.05	0.450000	0.2222	0.2278	2	0	1	0	0	-0.3758	0.8333	0	1	0	0	0.5103	0.9105	1.079	-0.1667	0.2604	1.1642	-0.8419	0.0025	-0.0417
HIP_49588	CTSICSLYQ	VHSSNNFGA	CTSICSLYQVHSSNNFGA		IAPP	2864/45018	0.980	49588	0.805	-0.092931	-0.1333	0.1667	1	18	0.1556	0	1	0	0	0.000000	0.4	0	0.0167	1.675000	0	1.675	0	0	0	0	0	-0.2233	0.4	0	1	0	0	0.4103	1.0522	0.8323	0.1667	0.1683	0.6927	-0.0547	0.0003	0.0067
HIP_49589	CGERGFFY	MLVKYPEII	CGERGFFYMLVKYPEII		SCG2	16965/334890	0.997	49589	0.805	-0.045344	2.3667	0	0	17	0.3882	0	4	1	0	0.000000	1.8667	0	0	0.950000	0.1111	0.8389	0	0	0	0	0	1.9854	7.4667	0	1	0	0	0.2385	1.1636	0.6178	0.8333	0.0569	0.3817	0.5644	0	0
HIP_49590	QCCTSICSL	NNFGAILS	QCCTSICSLNNFGAILS		IAPP	2944/45018	0.979	49590	0.805	0.088704	0.2167	0.1667	0	17	0.8647	0	1	1	0	0.000000	-3.2333	0	0.0333	0.000000	0	0	0	0	0	0	0	0	-3.2333	1	0	0	0	0.4385	0.9436	1.0545	0.3333	0.1922	1.1119	0.095	0.0011	-0.1078
HIP_49591	GERGFFYTP	SQKYPGPQA	GERGFFYTPSQKYPGPQA		CMGA	20016/247050	0.972	49591	0.805	0.029327	-1.9667	0.3333	1	18	-1.2	1	4	0	0	0.000000	-1.5333	1	0.05	0.700000	0.1111	0.5889	1	0	1	0	-1.5333	-1.1581	-6.1333	0	0	0	1	0.5603	0.8922	1.079	-0.1667	0.3139	1.1642	-1.1018	0.0025	-0.0767
HIP_49592	GSLQPLAL	MSDDYETQ	GSLQPLALMSDDYETQ		SCG2	10035/334890	0.999	49592	0.805	0.140597	1.1667	0.1667	-3	16	-0.5	-3	0	0	0	1.000000	-3.9333	-1	0.1167	0.925000	0.2222	0.7028	0	0	0	0	3.9333	0.8199	0	1	0	0	0	0.3051	1.1786	0.7278	0.5	0.0931	0.5297	0.356	0.0136	-0.4589
HIP_49593	QHLCGSHLV	MQPTLLLSL	QHLCGSHLVMQPTLLLSL		SCG1	16879/367830	0.993	49593	0.805	0.164987	0.2667	0.1667	2	18	0.7611	0	0	0	1	1.000000	-2.6667	0	0.1167	1.425000	0	1.425	1	0	1	0	0	0.38	0	1	0	0	0	0.3885	1.0436	0.8345	0.3333	0.1509	0.6964	0.1036	0.0136	-0.3111
HIP_49594	SLYQLENYC	RHPQGAWKE	SLYQLENYCRHPQGAWKE		SCG1	35280/367830	0.943	49594	0.805	0.056130	-1.9333	0.1667	1	18	-1.3056	0	1	0	0	0.000000	-2.3333	1	0.0667	0.950000	0.2222	0.7278	1	0	1	0	-2.3333	-1.407	-2.3333	0	0	0	0	0.5038	0.8877	1.1179	-0.3333	0.2539	1.2498	-0.9741	0.0044	-0.1556
HIP_49595	YLVCGERGF	KEESSPDYN	YLVCGERGFKEESSPDYN		SCG2	23210/334890	0.995	49595	0.805	0.005138	-2.1667	0.1667	-2	18	-1.0667	-2	1	0	0	0.000000	-2.9333	-2	0.0167	0.000000	0.5556	-0.5556	0	1	0	1	5.8667	1.2037	-2.9333	1	0	0	0	0.4936	1.1272	0.899	-0.5	0.2436	0.8082	-1.0684	0.0003	-0.0489
HIP_49596	LGGGPGAGS	GEEGAPGKW	LGGGPGAGSGEEGAPGKW		SCG1	11439/367830	0.997	49596	0.805	-0.043642	-1.1333	0.5	-1	18	-0.6444	-1	0	0	1	0.000000	-2.6667	-2	0.0333	0.000000	0.2222	-0.2222	0	2	0	1	5.3333	0.2519	0	0	1	0	0	0.5667	1.045	1.0567	-0.1667	0.3211	1.1165	-0.6422	0.0011	-0.0889
HIP_49597	SICSLYQLE	PLQQKENGD	SICSLYQLEPLQQKENGD		SCG2	11555/334890	0.999	49597	0.805	-0.207331	-0.75	0.1667	-2	18	-0.8278	-2	1	0	0	0.000000	0.6333	1	0.0167	1.900000	0.1111	1.7889	1	0	1	0	0.6333	-1.3417	0.6333	0	0	1	0	0.4833	1.15	0.87	0.1667	0.2336	0.7569	-0.3625	0.0003	0.0106
HIP_49598	HLCGSHLVE	KEESSPDYN	HLCGSHLVEKEESSPDYN		SCG2	29283/334890	0.991	49598	0.805	0.145665	-1.0667	0	-1	18	-1.0667	-3	0	0	0	0.000000	-5.1333	0	0.1	1.050000	0.4444	0.6056	0	0	0	0	0	-0.6459	0	0	0	1	0	0.4385	1.3186	0.6878	-0.3333	0.1922	0.4731	-0.4677	0.01	-0.5133
HIP_49599	VEALYLVCG	THYRASEEE	VEALYLVCGTHYRASEEE		SCG1	18997/367830	0.991	49599	0.805	-0.003019	0.1833	0.1667	-2	18	-0.2278	-3	2	0	1	0.000000	-3.8333	0	-0.0333	0.625000	0.1111	0.5139	0	1	0	1	0	0.0942	-7.6667	0	0	0	1	0.4654	0.9091	0.9468	0	0.2166	0.8964	0.0853	0.0011	0.1278
[illegible]																																														

HIP_S0068	LYQLENYCN	KSQREDEEE	LYQLENYCNKSQREDEEE		SCG1	18206/367830	0.992	S0068	0.805	0.085735	-1.75	0.1667	-4	18	-2.0389	-4	1	0	1	0.000000	-1.9667	1	0.1167	0.625000	0.3333	0.2917	0	0	0	0	-1.9667	-0.5104	-1.9667	0	1	0	0	0.5154	0.9408	1.0768	-0.1667	0.2656	1.1595	-0.9019	0.0136	-0.2294
HIP_S0069	YLVCGERGF	EDQELESLS	YLVCGERGFEDQELESLS		CMGA	11614/247050	0.993	S0069	0.805	-0.028615	-2.1	0.1667	-4	18	-0.4611	-4	1	0	0	0.000000	-2.8	-3	0.0167	0.000000	0.5556	-0.5556	0	1	0	1	8.4	1.1667	-2.8	1	0	0	0	0.4936	1.0605	0.939	-0.3333	0.2436	0.8817	-1.0365	0.0003	-0.0467
HIP_S0070	VCGERGFF	YGEESEEE	VCGERGFFYGEESEEE		SCG1	14094/367830	0.996	S0070	0.805	0.017490	0	0.3333	-5	16	-1.0312	-5	3	0	1	0.000000	-3.4667	-1	0.05	0.700000	0.4444	0.2556	0	2	0	1	3.4667	0	-10.4	1	0	0	0	0.4718	0.9886	0.9745	0.1667	0.2226	0.9496	0	0.0025	-0.1733
HIP_S0071	GSHLVEALY	VDGAGKPGA	GSHLVEALYVDGAGKPGA		CMGA	6196/247050	0.998	S0071	0.805	0.087795	0.7667	0.1667	0	18	0.1111	-1	1	0	0	0.000000	-1.3333	-1	0.1333	3.500000	0.2222	3.2778	0	1	0	1	1.3333	2.513	-1.3333	0	1	0	0	0.3603	1.0588	0.829	0.3333	0.1298	0.6872	0.2762	0.0178	-0.1778
HIP_S0072	LQPLALEG	LALEGSLQ	LQPLALEGLALEGSLQ		INSC	19/5184	1.000	S0072	0.805	0.094978	1.55	0.1667	-2	16	0.575	-2	0	0	0	0.000000	3.1667	1	-0.1333	1.900000	0.2222	1.6778	0	1	0	1	3.1667	2.6006	0	0	0	0	1	0.35	1.2267	0.7333	0.5	0.1225	0.5378	0.5425	0.0178	-0.4222
HIP_S0073	ERGFFYT	FLVHSSN	ERGFFYTFLVHSSN		IAPP	2162/45018	0.989	S0073	0.805	-0.035150	1.9333	0	1	14	-0.1643	0	4	0	0	0.000000	3.3333	0	-0.05	0.700000	0	0.7	0	0	0	0	0	1.3533	13.3333	0	1	0	0	0.3051	1.1019	0.6778	0.6667	0.0931	0.4594	0.5899	0.0025	-0.1667
HIP_S0074	SICSLYQL	VALNHLKA	SICSLYQLVALNHLKA		IAPP	2313/45018	0.988	S0074	0.805	0.017211	1.4667	0	2	16	0.8125	1	1	0	0	1.000000	3.6	0	-0.05	1.050000	0	1.05	0	0	0	0	0	1.54	3.6	1	0	0	0	0.3051	1.2069	0.6745	0.6667	0.0931	0.4549	0.4475	0.0025	-0.18
HIP_S0075	DLQVGQVEL	GEEDATSEV	DLQVGQVELGEEDATSEV		SCG1	9672/367830	0.998	S0075	0.805	0.111189	-0.4833	0.1667	-6	18	-0.4611	-6	0	0	0	0.000000	-3.9667	-1	0.1333	0.000000	0.4444	-0.4444	0	1	0	1	3.9667	0.2148	0	1	0	0	0	0.4833	1.2317	0.7867	-0.1667	0.2336	0.6188	-0.2336	0.0178	-0.5289
HIP_S0076	ELGGGPGAG	EEEEEEEAT	ELGGGPGAGEEEEEEEAT		CMGA	5139/247050	0.998	S0076	0.805	-0.096766	-1.5833	0.3333	-8	18	-1.3833	-8	0	0	6	0.000000	-3.8333	-3	-0.0167	0.450000	0.4444	0.0056	0	2	0	1	11.5	-0.0088	0	0	0	0	1	0.5667	1.1633	0.98	-0.3333	0.3211	0.9604	-0.8972	0.0003	0.0639
HIP_S0077	YQLENYCN	QYSSHHTAE	YQLENYCNQYSSHHTAE		SCG1	24585/367830	0.981	S0077	0.805	0.046250	-1.3167	0.1667	0	17	-1.5	-2	2	1	0	0.000000	-1.1	0	0.0667	0.000000	0	0	0	0	0	0	0	0	-2.2	0	1	0	0	0.5154	0.9408	1.0768	0	0.2656	1.1595	-0.6786	0.0044	-0.0733
HIP_S0078	FVNQHLCGS	EGGGHSRER	FVNQHLCGSEGGGHSRER		SCG1	11744/367830	0.997	S0078	0.805	0.004492	-0.5	0.6667	2	18	-1.0722	0	0	0	1	0.000000	-1.8667	-1	0.0667	0.000000	0.1111	-0.1111	0	3	0	1	1.8667	0.0556	0	0	1	0	0	0.6218	0.8053	1.2245	-0.1667	0.3866	1.4994	-0.3109	0.0044	-0.1244
HIP_S0079	AGSLQPLAL	VPGQGSSED	AGSLQPLALVPGQGSSED		SCG2	3566/334890	1.000	S0079	0.805	0.140468	1.9333	0.3333	-2	18	-0.0889	-2	0	0	0	1.000000	-2.4	0	0.15	1.500000	0	1.5	1	1	1	1	0	2.9	0	1	0	0	0	0.3667	1.03	0.8633	0.6667	0.1344	0.7453	0.7089	0.0225	-0.36
HIP_S0080	CSLYQLENY	SKDVMEKRE	CSLYQLENYSKDVMEKRE		CMGA	22584/247050	0.962	S0080	0.805	-0.046868	-2.75	0.1667	-1	18	-1.1833	-1	1	0	0	0.000000	0.0333	1	0.0667	1.050000	0.3333	0.7167	0	0	0	0	0.0333	-1.9708	0.0333	0	1	0	0	0.5154	1.0074	1.0368	-0.5	0.2656	1.0749	-1.4173	0.0044	0.0022
HIP_S0081	CSLYQLENY	RPEDQELES	CSLYQLENYRPEDQELES		CMGA	15836/247050	0.985	S0081	0.805	-0.034991	-2.9833	0.1667	-4	18	-1.3556	-4	1	0	0	0.000000	-0.4333	1	0.0667	0.000000	0.4444	-0.4444	1	0	1	0	-0.4333	1.3259	-0.4333	0	1	0	0	0.5603	1.0505	1.0623	-0.5	0.3139	1.1285	-1.6714	0.0044	-0.0289
HIP_S0082	CGSHLVEAL	AEAYPSKPD	CGSHLVEALAEAYPSKPD		NPY	3180/49410	0.973	S0082	0.805	-0.046677	0.3667	0	-1	18	-0.2444	-2	1	0	0	1.000000	-0.6667	0	0	1.850000	0.2222	1.6278	0	0	0	0	0	0.5969	-0.6667	1	0	0	0	0.3333	1.445	0.66	0.3333	0.1111	0.4356	0.1222	0	0
HIP_S0083	ERGFFYTPK	LSDDVSKVI	ERGFFYTPKLSDDVSKVI		SCG2	19804/334890	0.996	S0083	0.805	-0.090611	-1.1167	0.3333	0	18	-0.3389	0	2	0	0	0.000000	1.9	-2	-0.05	1.650000	0.3333	1.3167	1	0	1	0	-3.8	-1.4703	3.8	0	0	1	0	0.4936	0.9238	0.999	-0.1667	0.2436	0.9979	-0.5512	0.0025	-0.095
HIP_S0084	SHLVEALY	CNTATCATQ	SHLVEALYCNTATCATQ		IAPP	2764/45018	0.981	S0084	0.805	0.205720	0.4333	0	0	17	0.2529	-1	1	1	0	0.000000	-2	0	0.1	0.000000	0.1111	-0.1111	0	0	0	0	0	-0.0481	-2	0	1	0	0	0.4103	1.0488	0.9156	0.3333	0.1683	0.8384	0.1778	0.01	-0.2
HIP_S0085	LVEALYLVC	EAYPSKPDN	LVEALYLVCEAYPSKPDN		NPY	2953/49410	0.976	S0085	0.805	0.057884	1.25	0	-2	18	0.0778	-2	2	0	0	0.000000	-4.5	-1	0.05	2.000000	0.1111	1.8889	0	0	0	0	4.5	2.3611	-9	0	1	0	0	0.3436	1.2272	0.7023	0.3333	0.1181	0.4932	0.4295	0.0025	-0.225
HIP_S0086	CSLYQLENY	AEAASFQRN	CSLYQLENYAEAASFQRN		SCG2	21335/334890	0.996	S0086	0.805	-0.071443	-1.3667	0	-1	18	-0.6333	-1	1	0	0	0.000000	2.8	0	-0.05	0.900000	0.2222	0.6778	0	0	0	0	0	-0.9263	2.8	0	1	0	0	0.4385	1.2686	0.8778	0	0.1922	0.7706	-0.5992	0.0025	-0.14
HIP_S0087	EQCCTSICS	GRGGEPRAY	EQCCTSICSGRGGEPRAY		SCG1	15959/367830	0.994	S0087	0.805	0.188132	0.15	0.5	0	18	-0.6722	0	0	0	0	0.000000	-3.8333	1	0.15	0.625000	0.1111	0.5139	0	2	0	1	-3.8333	0.0771	0	0	1	0	0	0.5103	0.8205	1.0806	0	0.2604	1.1678	0.0765	0.0225	-0.575
HIP_S0088	PLALEGSLQ	FQELGKLTG	PLALEGSLQFQELGKLTG		SCG2	18920/334890	0.996	S0088	0.805	-0.136818	-0.7833	0.1667	-1	18	0.0778	-1	1	0	0	0.000000	-1.2333	-1	-0.0167	2.600000	0.2222	2.3778	0	0	0	0	1.2333	-1.8626	-1.2333	0	1	0	0	0.4667	1.1717	0.8333	0.1667	0.2178	0.6944	-0.3656	0.0003	0.0206
HIP_S0089	VEALYLVCG	SKENIEKNE	VEALYLVCGSKENIEKNE		SCG2	17585/334890	0.997	S0089	0.805	0.117052	-0.3167	0.3333	-2	18	-0.3611	-2	1	0	0	0.000000	-4.8333	0	0.0833	0.625000	0.2222	0.4028	0	1	0	1	0	-0.1275	-4.8333	0	0	0	1	0.4936	0.9855	0.959	-0.1667	0.2436	0.9196	-0.1563	0.0069	-0.4028
HIP_S0090	AGSLQPLAL	QDPEGKGE	AGSLQPLALQDPEGKGE		CMGA	10573/247050	0.984	S0090	0.805	0.202115	0.1333	0.1667	-2	17	-0.6824	-2	0	1	0	0.000000	-6	-1	0.1667	0.450000	0.2222	0.2278	1	0	1	0	6	0.0304	0	1	0	0	0	0.3885	1.1253	0.8445	0.3333	0.1509	0.7132	0.0518	0.0278	-1
HIP_S0091	GSLQPLALE	LLMKVLEYL	GSLQPLALELLMKVLEYL		SCG2	14320/334890	0.998	S0091	0.805	0.004437	1.9333	0	-1	18	0.8889	-1	0	0	0	0.000000	2.4667	1	-0.05	1.900000	0.2222	1.6778	0	0	0	0	2.4667	3.2437	0	0	0	1	0	0.2667	1.3433	0.5833	0.6667	0.0711	0.3403	0.5156	0.0025	-0.1233
HIP_S0092	ICSLYQLEN	LEALRQAEN	ICSLYQLENLEALRQAEN		SCG2	13560/334890	0.998	S0092	0.805	-0.109670	-0.1833	0	-2	18	-0.2944	-2	1	0	0	0.000000	1.7667	0	-0.05	1.900000	0.2222	1.6778	0	0	0	0	0	-0.3076	1.7667	0	1	0	0	0.4	1.29	0.7833	0.1667	0.16	0.6136	-0.0733	0.0025	-0.0883
HIP_S0093	LYQLENYC	PSQKYPGPQ	LYQLENYCPSQKYPGPQ		CMGA	12457/247050	0.991	S0093	0.805	0.145144	-1.3667	0.3333	0	17	-1.2471	0	2	1	0	0.000000	-1.2	0	0.1333	0.625000	0.2222	0.4028	1	0	1	0	0	-0.5505	-2.4	0	0	0	0	0.5603	0.8655	1.179	0	0.3139	1.39	-0.7657	0.0178	-0.16
HIP_S0094	RGFFYTPKT	SSQESGEET	RGFFYTPKTSSQESGEET		SCG1	20752/367830	0.988	S0094	0.805	-0.088474	-1.8833	0.5	-1	18	-1.3889	-1	1	0	0	0.000000	0.3667	-1	0.0167	0.000000	0.2222	-0.2222	1	0	1	0	-0.3667	0.4185	0.3667	0	1	0	0	0.5885	0.8553	1.1278	-0.1667	0.3463	1.272	-1.1083	0.0003	0.0061
HIP_S0095	GIVEQCCTS	NPGEDAPAE	GIVEQCCTSNPGEDAPAE		NPY	321/49410	0.999	S0095	0.805	-0.034599	-0.75	0.5	-4	18	-0.5111	-4	0	0	0	0.000000	-2.1667	0	0.0833	0.000000	0.1111	-0.1111	1	1	1	1	0	0.0833	0	0	1	0	0	0.6051	0.7319	1.2745	0	0.3662	1.6243	-0.4538	0.0069	-0.1806
HIP_S0096	CCTSICSLY	YPKTPGRAG	CCTSICSLYYPKTPGRAG		SCG2	28731/334890	0.991	S0096	0.805	0.040866	-0.85	0.3333	2	18	-0.0222	2	2	0	0	0.000000	-2.8333	1	0.05	2.075000	0.1111	1.9639	1	0	1	0	-2.8333	-1.6693	-5.6667	0	1	0	0	0.4654	0.9558	0.9901	0	0.2166	0.9804	-0.3956	0.0025	-0.1417
HIP_S0097	CCTSICSL	GENYQKGE	CCTSICSLGENYQKGE		SCG1	14936/367830	0.995	S0097	0.805	0.168901	-0.3167	0.3333	-1	16	-0.4062	-1	1	0	0	0.000000	-4.3	1	0.1167	0.000000	0.1111	-0.1111	0	1	0	1	4.3	0.0352	-4.3	1	0	0	0	0.5218	0.9669	1.0678	0	0.2723	1.1402	-0.1652	0.0136	-0.5017
HIP_S0098	GERGFFYTP	VTALPVNSP	GERGFFYTPVTALPVNSP		CMGA	8143/247050	0.997	S0098	0.805	-0.088156	0.2833	0.1667	0	18	-0.0556	0	3	0	0	0.000000	2.9667	0	-0.0667	2.700000	0	2.7	1	0	1	0	0	0.765	8.9	0	0	0	1	0.4551	0.9603	0.9178	0.3333	0.2071	0.8424	0.129	0.0044	-0.1978
HIP_S0099	VCGERGFFY	EDLIEMLK	VCGERGFFYEDLIEMLK		SCG2	18818/334890	0.996	S0099	0.805	0.092412	0.1833	0	-2	17	0.1059	-2	3	1	0	0.000000	-2.5	-2	0.1	1.825000	0.3333	1.4917	0	0	0	0	5	0.2735	-7.5	0	1	0	0	0.3436	1.2088	0.709	0.1667	0.1181	0.5026	0.062	0.01	-0.25
HIP_S0100	QHLCGSHLV	GGSLPSEEK	QHLCGSHLVGGSLPSEEK		SCG1	10003/367830	0.998	S0100	0.805	0.182680	0.5333	0.5	1	18	-0.4389	-1	0	0	0	0.000000	-2.1333	0	0.2333	1.900000	0	1.9	0	2	0	1	0	1.0133	0	1	0	0	0	0.4718	0.8636	1.0178	0.1667	0.2226	1.036	0.2516	0.0544	-0.4978
HIP_S0101	HLVEALYLV	ELGEYLAR	HLVEALYLVELGEYLAR		SCG2	8415/334890	0.999	S0101	0.805	0.167716	1.1	0.1667	-1	17	0.5765	-2	1	1	0	0.000000	-2.2667	-1	0.1333	1.400000	0.2222	1.1778	0	1	0	1	2.2667	1.2956	-2.2667	0	1	0	0	0.3885	1.1153	0.7745	0.3333	0.1509	0.5998	0.4273	0.0178	-0.3022
HIP_S0102	AGSLQPLAL	QAELKEAV	AGSLQPLALQAELKEAV		CMGA	12589/247050	0.991	S0102	0.805	0.134928	0.7	0	-1	17	0.3471	-1	0	1	0	0.000000	-4.8667	-1	0.1	1.400000	0.1111	1.2889	0	0	0	0	4.8667	0.9022	0	1	0	0	0	0.3333	1.3533	0.6733	0.5	0.1111	0.4534	0.2333	0.01	-0.4867
HIP_S0103	DLQVGQVEL	PVEEKIESQ	DLQVGQVELPVEEKIESQ		SCG2	11246/334890	0.999	S0103	0.805	0.006733	0.6	0.1667	-4	18	-0.5556	-4	0	0	0	0.000000	-1.8	0	0.0667	0.000000	0.3333	-0.3333	1	0	1	0	0	-0.2	0	1	0	0	0	0.4167	1.1683	0.75	0.1667	0.1736	0.5625	0.25	0.0044	-0.12
HIP_S0104	GSLQPLAL	NAFLNERKQ	GSLQPLALNAFLNERKQ		SCG1	25273/367830	0.979	S0104	0.805	0.093046	1.75	0	1	17	-0.4176	1	1	1	0	0.000000	-2.7667	0	0.05	1.400000	0	1.4	0	0	0	0	0	2.45	-2.7667	1	0	0	0	0.2667	1.2283	0.7467	0.8333	0.0711	0.5575	0.4667	0.0025	-0.1383
HIP_S0105	YLVCGERGF	SWDEEDKRN	YLVCGERGFSWDEEDKRN		SCG1	15702/367830	0.994	S0105	0.805	-0.039803	-1.2167	0.3333	-2	18	-1.3556	-2	2	0	0	0.000000	-1.0333	-2	-0.0167	0.000000	0.4444	-0.4444	0	1	0	1	2.0667	0.5407	-2.0667	1	0	0	0	0.4654	0.9274	1.0035	0	0.2166	1.0069	-0.5662	0.0003	0.0172
HIP_S0106	LQVGQVELG	EPERELDLP	LQVGQVELGEPERELDLP		SCG2	9442/334890	0.999	S0106	0.805	-0.127059	-1.45	0.3333	-4	18	-0.5222	-4	0	0	0	0.000000	-2.8333	-1	0.0333	0.950000	0.4444	0.5056	1	1	1	1	2.8333	-0.7331	0	0	0	0	1	0.5667	1.15	0.9567	-0.3333	0.3211	0.9152	-0.8217	0.0011	-0.0944
HIP_S0107	GERGFFYT	DRSSQGGSL	GERGFFYTDRSSQGGSL		SCG1	30110/367830	0.965	S0107	0.805	0.155105	-1.3333	0.1667	0	17	-0.9471	0	3	1	0	0.000000	-3.2	0	0.1167	0.700000	0.2222	0.4778	0	0	0	0	0	-0.637	-9.6	0	1	0	0	0.4487	0.9824	0.9135	-0.1667	0.2013	0.8344	-0.5983	0.0136	-0.3733
HIP_S0108	VEALYLVCG	RSGQLGIQE	VEALYLVCGRSGQLGIQE		SCG2	14220/334890	0.998	S0108	0.805	0.148962	0.1	0.5	-1	18	0.3778	-1	1	0	0	0.000000	-4	1	0.1167	0.625000	0.1111	0.5139	0	2	0	1	-4	0.0514	-4	0	0	0	1	0.5103	0.8172	1.0856	0	0.2604	1.1786	0.051	0.0136	-0.4667
HIP_S0109	HLVEALYL	TYGKRNA	HLVEALYLTYGKRNA		IAPP	2946/45018	0.979	S0109	0.805	0.226704	0.65	0.1667	2	15	-0.2067	1	2	1	0	0.000000	-2.9	0	0.1333	0.000000	0.2222	-0.2222	0	1	0	1	0	-0.1444	-5.8	1	0	0	0	0.4269	0.9955	0.879	0.3333	0.1823	0.7726	0.2775	0.0178	-0.3867
HIP_S0110	DLQVGQVEL	PGPQAEGDS	DLQVGQVELPGPQAEGDS		CMGA	4127/247050	0.999	S0110	0.805	0.147821	0.15	0.5	-4	18	-0.6611	-4	0	0	0	0.000000	-2.7	1	0.1667	0.000000	0.1111	-0.1111	2	1	1	1	-2.7	-0.0167	0	1	0	0	0	0.5167	0.9183	1.0467	0.1667	0.2669	1.0955	0.0775	0.0278	-0.45
HIP_S0111	YLVCGERGF	GSQENHPQE	YLVCGERGFGSQENHPQE		SCG1	8083/367830	0.998	S0111	0.805	-0.006714	-1.1333	0.5	-1	18	-1.1278	-2	1	0	0	0.000000	-0.8667	-1	0.0667	0.000000	0.3333	-0.3333	0	2	0	1	0.8667	0.3778	-0.8667	1	0	0	0	0.5385	0.8553	1.1145	0	0.2899	1.2421	-0.6103	0.0044	-0.0578
HIP_S0112	PLALEGSLQ	YPKTPGRAG	PLALEGSLQYPKTPGRAG		SCG2	30917/334890	0.989	S0112	0.805	-0.023203	-1.2167	0.3333	1	18	-0.5111	1	1	0	0	0.000000	-2.1	1	0	0.950000	0.2222	0.7278	1	0	1	0	-2.1	-0.8855	-2.1	0	1	0	0	0.4936	0.9705	1.0023	0	0.2436	1.0046	-0.6005	0	0
HIP_S0113	HLVEALYL	RNYLNYGEE	HLVEALYLRNYLNYGEE		SCG1	29811/367830	0.966	S0113	0.805	0.136795	-0.5	0	-1	17	-0.4882	-2	2	1	0	0.000000	-5.2	1	0.1	1.400000	0.1111	1.2889	0	0	0	0	-5.2	-0.6444	-10.4	1	0	0	0	0.3821	1.0558	0.8801	0.1667	0.146	0.7746	-0.191	0.01	-0.52
HIP_S0114	GAGSLQPLA	KENREEARF	GAGSLQPLAKENREEARF		SCG1	26981/367830	0.975	S0114	0.805	0.092506	-1.15	0.1667	0	18	-0.9889	0	0	0	0	0.000000	-4.9667	0	0.0833	1.900000	0.3333	1.5667	1	0	1	0	0	-1.8017	0	1	0	0	0	0.4551	1.0869	0.9812	0	0.2071	0.9627	-0.5234	0.0069	-0.4139
HIP_S0115	ERGFFYTPK	TNTHPPASL	ERGFFYTPKTNTHPPASL		CMGA	18502/247050	0.977	S0115	0.805	-0.069332	-1.85	0.1667	2	18	-0.9333	1	2	0	0	0.000000	0.4333	-1	-0.0167	0.700000	0.1111	0.5889	1	0	1	0	-0.4333	-1.0804	0.8667	0	0	1	0	0.5885	0.7919	1.1445	-0.1667	0.3463	1.3099	-1.0887	0.0003	-0.0072
HIP_S0116	YLVCGERGF	PQSAPKENK	YLVCGERGFPQSAPKENK		SCG2	16176/334890	0.997	S0116	0.805	-0.003230	-1.3333	0.5	1	18	-0.9611	1	1	0	0	0.000000	-1.2667	-1	0.05	0.450000	0.2222	0.2278	1	1	1	1	1.2667	-0.3037	-1.2667	1	0	0	0	0.5385	0.8669	1.1178	0	0.2899	1.2495	-0.7179	0.0025	-0.0633
HIP_S0117	DLQVGQVEL	EGDSEGLSQ	DLQVGQVELEGDSEGLSQ		CMGA	6985/247050	0.997	S0117	0.805	0.131730	-0.4833	0.1667	-5	18	-0.6111	-5	0	0	0	0.000000	-3.9667	-1	0.1333	0.000000	0.3333	-0.3333	0	1	0	1	3.9667	0.1611	0	1	0	0	0	0.4551	1.1503	0.8145	-0.1667	0.2071	0.6634	-0.22	0.0178	-0.5289
HIP_S0118	LVCGERGFF	LDRNYLNY	LVCGERGFFLDRNYLNY		SCG1	26607/367830	0.976	S0118	0.805	0.044958	0.1667	0.1667	0	17	-0.1588	0	2	1	0	1.000000	-3.1333	0	0.0167	1.650000	0.4444	1.2056	0	1	0	1	0	0.2009	-6.2667	1	0	0	0	0.3603	1.0405	0.8356	0.1667	0.1298	0.6983	0.06	0.0003	-0.0522
HIP_S0119	HLCGSHLVE	RPQSEESWD	HLCGSHLVERPQSEESWD		SCG1	28163/367830	0.972	S0119	0.805	0.121891	-0.85	0.1667	-1	18	-1.0778	-3	0	0	0	0.000000	-4.7	2	0.1167	1.050000	0.2222	0.8278	1	0	1	0	-9.4	-0.7036	0	0	0	1	0	0.4551	1.0953	0.8578	0	0.2071	0.7359	-0.3869	0.0136	-0.5483
HIP_S0120	CTSICSLYQ	PDLRLENVQ	CTSICSLYQPDLRLENVQ		SCG2	12430/334890	0.998	S0120	0.805	-0.012478	-0.3833	0.1667	-1	18	-0.1167	-1	1	0	0	0.000000	-0.1	-1	0.0667	0.625000	0.2222	0.4028	1	0	1	0	0.1	-0.1544	-0.1	0	1	0	0	0.4269	1.0538	0.8856	0.1667	0.1823	0.7844	-0.1637	0.0044	-0.0067
HIP_S0121	LVCGERGFF	GNNQAPGE	LVCGERGFFGNNQAPGE		CMGA	11447/247050	0.993	S0121	0.805	0.116910	-0.3667	0.3333	-1	17	-0.4294	-1	2	1	0	0.000000	-4.2	0	0.1	0.700000	0.2222	0.4778	0	2	0	1	0	-0.1752	-8.4	1	0	0	0	0.5	0.79	1.22	0.3333	0.25	1.4884	-0.1833	0.01	-0.42
HIP_S0122	SICSLYQLE	QPRSQEESE	SICSLYQLEQPRSQEESE		SCG1	27333/367830	0.974	S0122	0.805	-0.046983	-2.1333	0.1667	-3	18	-1.1389	-3	1	0	0	0.000000	-2.1333	2	0.0667	1.900000	0.2222	1.6778	1	0	1	0	-4.2667	-3.5793	-2.1333	0	0	1	0	0.5218	1.1036	0.9378	-0.1667	0.2723	0.8795	-1.1132	0.0044	-0.1422
HIP_S0123	VNQHLCGSH	EESNVSMAS	VNQHLCGSHEESNVSMAS		SCG1	20931/367830	0.988	S0123	0.805	-0.114625	-2.0333	0.5	0	18	-0.5056	-2	0	0	0	0.000000	-1.1333	-2	0.0167	0.950000	0.2222	0.7278	0	1	0	1	2.2667	-1.4798	0	0	0	1	0	0.5885	1.0236	1.0478	-0.5	0.3463	1.0979	-1.1965	0.0003	-0.0189
HIP_S0124	VNQHLCGSH	SEESNVSMA	VNQHLCGSHSEESNVSMA		SCG1	20932/367830	0.988	S0124	0.805	-0.114625	-2.0333	0.5	0	18	-0.5056	-2	0	0	0	0.000000	-1.1333	-2	0.0167	0.950000	0.2222	0.7278	0	1	0	1	2.2667	-1.4798	0	0	0	1	0	0.5885	1.0236	1.0478	-0.5	0.3463	1.0979	-1.1965	0.0003	-0.0189
HIP_S0125	SLQPLAL	TSICSLY	SLQPLALTSICSLY		INSA	127/3024	0.965	S0125	0.805	0.159231	2.0667	0.1667	0	14	1.0357	0	0	0	0	0.000000	-2.1333	0	0.1167	1.075000	0	1.075	0	0	0	0	0	2.2217	0	1	0	0	0	0.3333	1.1267	0.715	0.6667	0.1111	0.5112	0.6889	0.0136	-0.2489
[illegible]																																														

HIP_S0579	GGPGAGSLQ	VFLIVLSV	GGPGAGSLQVFLIVLSV		IAPP	1337/45018	0.995	S0579	0.804	0.040613	1.7167	0.1667	0	17	1.4588	0	1	1	0	[illegible]
HIP_S0580	SHLVEALYL	PQSEESWDE	SHLVEALYLPQSEESWDE		SCG1	12868/367830	0.996	S0580	0.804	0.199278	0.0667	0.3333	-4	18	-0.7222	-5	1	0	0	[illegible]
HIP_S0581	ERGFFYTPK	EVLENQSSQ	ERGFFYTPKEVLENQSSQ		CMGA	12737/247050	0.991	S0581	0.804	0.033499	-0.2833	0.1667	-1	18	-1.1889	-1	2	0	0	[illegible]
HIP_S0582	GSLQPLALE	YPKTPGRAG	GSLQPLALEYPKTPGRAG		SCG2	35251/334890	0.985	S0582	0.804	0.106186	-0.7833	0.1667	1	18	-0.5111	1	1	0	0	[illegible]
HIP_S0583	GSLQPLAL	KEEEEEEMA	GSLQPLALKEEEEEEMA		CMGA	11973/247050	0.992	S0583	0.804	0.172046	-0.25	0	-4	16	-0.675	-4	0	0	3	[illegible]
HIP_S0584	AGSLQPLAL	HMQFPPMY	AGSLQPLALHMQFPPMY		SCG2	27770/334890	0.992	S0584	0.804	0.120610	0.7667	0	1	17	0.2412	0	1	1	0	[illegible]
HIP_S0585	SLYQLENYC	EEEEEEEE	SLYQLENYCEEEEEEEE		CMGA	8874/247050	0.996	S0585	0.804	-0.007854	-2.1333	0	-9	17	-1.8706	-9	1	1	6	[illegible]
HIP_S0586	SLYQLENYC	EEEEEEEE	SLYQLENYCEEEEEEEE		CMGA	8875/247050	0.996	S0586	0.804	-0.007854	-2.1333	0	-9	17	-1.8706	-9	1	1	6	[illegible]
HIP_S0587	SLYQLENYC	EEEEEEEE	SLYQLENYCEEEEEEEE		CMGA	8876/247050	0.996	S0587	0.804	-0.007854	-2.1333	0	-9	17	-1.8706	-9	1	1	6	[illegible]
HIP_S0588	CGERGFFYT	QYWDEDLL	CGERGFFYTQYWDEDLL		SCG2	22901/334890	0.995	S0588	0.804	0.024604	-0.8167	0	-3	17	-0.6647	-3	5	1	0	[illegible]
HIP_S0589	CGERGFFY	PVSQECFET	CGERGFFYPVSQECFET		CMGA	4840/247050	0.998	S0589	0.804	0.122151	1.0167	0.3333	-2	17	-0.3588	-2	3	1	0	[illegible]
HIP_S0590	EALYLVCGE	EEPSSKDVM	EALYLVCGEEEPSSKDVM		CMGA	7751/247050	0.997	S0590	0.804	-0.130184	-1.6667	0.3333	-4	18	-0.2278	-4	0	0	1	[illegible]
HIP_S0591	CGERGFFY	NLQIPPED	CGERGFFYNLQIPPED		SCG2	17980/334890	0.997	S0591	0.804	0.095851	0.1833	0	-2	16	-0.6813	-2	3	0	0	[illegible]
HIP_S0592	VCGERGFF	FLVHSSNN	VCGERGFFFLVHSSNN		IAPP	1196/45018	0.995	S0592	0.804	0.008196	2.6667	0.1667	1	16	0.1562	0	3	0	1	[illegible]
HIP_S0593	TSICSLYQL	LPKAAWIPH	TSICSLYQLLPKAAWIPH		SCG2	22580/334890	0.995	S0593	0.804	0.024268	-0.45	0.1667	2	18	0.4556	1	1	0	0	[illegible]
HIP_S0594	YLVCGERGF	KVIAYLKRL	YLVCGERGFKVIAYLKRL		SCG2	20055/334890	0.996	S0594	0.804	0.094728	0.45	0.1667	3	18	0.4278	3	1	0	0	[illegible]
HIP_S0595	ELGGGPGAG	RQYWDEDLL	ELGGGPGAGRQYWDEDLL		SCG2	16861/334890	0.997	S0595	0.804	-0.014111	-1.3833	0.3333	-3	18	-0.8111	-3	2	0	1	[illegible]
HIP_S0596	GAGSLQPLA	GEEDATSEV	GAGSLQPLAGEEDATSEV		SCG1	3761/367830	0.999	S0596	0.804	0.028916	-0.5667	0.3333	-4	18	-0.3	-4	0	0	0	[illegible]
HIP_S0597	QCCTSICSL	SKSGYPKTP	QCCTSICSLSKSGYPKTP		SCG2	28697/334890	0.991	S0597	0.804	0.227380	0	0.5	2	18	-0.2778	2	0	0	0	[illegible]
HIP_S0598	VCGERGFF	QGGSLPSEE	VCGERGFFQGGSLPSEE		SCG1	7644/367830	0.999	S0598	0.804	0.119492	0.15	0.5	-2	17	-0.4235	-2	2	1	0	[illegible]
HIP_S0599	GERGFFY	TYGKRNAVE	GERGFFYTYGKRNAVE		IAPP	3027/45018	0.978	S0599	0.804	0.177276	0.3167	0.1667	1	16	-1.0188	1	4	2	0	[illegible]
HIP_S0600	LYQLENYCN	RGGEPRAYF	LYQLENYCNRGGEPRAYF		SCG1	14991/367830	0.995	S0600	0.804	0.158709	-1.2667	0.3333	0	18	-1.0056	0	1	0	0	[illegible]
HIP_S0601	GGGPGAGSL	EGSANRRPE	GGGPGAGSLEGSANRRPE		CMGA	5314/247050	0.998	S0601	0.804	-0.023239	-0.35	0.6667	0	18	-1.0722	0	0	0	1	[illegible]
HIP_S0602	CGERGFFY	DSKEAEKSG	CGERGFFYDSKEAEKSG		CMGA	17566/247050	0.980	S0602	0.804	0.211590	-0.65	0.1667	-1	17	-1.2059	-1	3	1	0	[illegible]
HIP_S0603	LCGSHLVEA	FETLRGDER	LCGSHLVEAFETLRGDER		CMGA	15846/247050	0.985	S0603	0.804	-0.003999	0.1833	0	-1	18	-0.3222	-2	1	0	0	[illegible]
HIP_S0604	GGGPGAGSL	SKSSAPPIT	GGGPGAGSLSKSSAPPIT		SCG1	23003/367830	0.984	S0604	0.804	0.025407	-0.4833	0.6667	1	18	-0.15	1	0	0	1	[illegible]
HIP_S0605	HLVEALYL	LESVFQELG	HLVEALYLLESVFQELG		SCG2	11768/334890	0.998	S0605	0.804	0.111251	0.9667	0.1667	-2	17	0.7235	-3	2	1	0	[illegible]
HIP_S0606	CSLYQLENY	QLPKAAWIP	CSLYQLENYQLPKAAWIP		SCG2	18970/334890	0.996	S0606	0.804	-0.095204	-1.6	0.1667	0	18	-0.1889	0	1	0	0	[illegible]
HIP_S0607	ERGFFYTPK	HPSLGYKEI	ERGFFYTPKHPSLGYKEI		CMGA	16416/247050	0.983	S0607	0.804	-0.093638	-1.9667	0.5	2	18	-0.9278	1	2	0	0	[illegible]
HIP_S0608	LVEALYLVC	PSPEMIRA	LVEALYLVCPSPEMIRA		SCG2	12773/334890	0.998	S0608	0.804	0.204282	1.0833	0.5	-1	17	0.9118	-1	1	1	0	[illegible]
HIP_S0609	YLVCGERGF	FNPYYDPLQ	YLVCGERGFFNPYYDPLQ		SCG1	6463/367830	0.999	S0609	0.804	-0.107694	-0.7333	0.3333	-1	18	-0.3611	-1	3	0	0	[illegible]
HIP_S0610	DLQVGQVEL	AYFMSDTRE	DLQVGQVELAYFMSDTRE		SCG1	14941/367830	0.995	S0610	0.804	-0.022479	1.3	0	-3	18	-0.3444	-3	2	0	0	[illegible]
HIP_S0611	QHLCGSHLV	KPFSEDVNW	QHLCGSHLVKPFSEDVNW		SCG1	23875/367830	0.983	S0611	0.804	0.192583	0.35	0.1667	1	18	-0.4167	-1	1	0	0	[illegible]
HIP_S0612	GERGFFYT	SGYPKTPGR	GERGFFYTSGYPKTPGR		SCG2	20741/334890	0.996	S0612	0.804	0.114359	-0.2833	0.3333	2	17	-1.2	2	4	1	0	[illegible]
HIP_S0613	VCGERGFFY	LEALRQAE	VCGERGFFYLEALRQAE		SCG2	18360/334890	0.997	S0613	0.804	0.000981	1.0667	0	-1	17	-0.0941	-1	3	1	0	[illegible]
HIP_S0614	CTSICSLY	QENQMDKAR	CTSICSLYQENQMDKAR		SCG1	26802/367830	0.975	S0614	0.804	0.108337	-1.4667	0.1667	0	17	-0.7353	0	1	1	0	[illegible]
HIP_S0615	CGERGFFY	VFQELGKLT	CGERGFFYVFQELGKLT		SCG2	18547/334890	0.997	S0615	0.804	0.015264	1.3	0	0	17	0.0353	0	4	1	0	[illegible]
HIP_S0616	LVEALYL	ASLGEKR	LVEALYLASLGEKR		SCG1	19149/367830	0.991	S0616	0.804	0.079510	1.85	0.1667	0	14	0.3643	0	1	0	0	[illegible]
HIP_S0617	GPGAGSLQP	VALNHLKAT	GPGAGSLQPVALNHLKAT		IAPP	1466/45018	0.994	S0617	0.804	0.133316	1.4167	0.1667	2	18	0.0556	1	0	0	0	[illegible]
HIP_S0618	GSLQPLAL	EEQKLYTD	GSLQPLALEEQKLYTD		SCG2	16541/334890	0.997	S0618	0.804	0.152688	-0.1833	0	-2	16	-0.575	-2	0	0	0	[illegible]
HIP_S0619	GSLQPLAL	WKSSHFERR	GSLQPLALWKSSHFERR		SCG1	34244/367830	0.948	S0619	0.804	0.221933	0.6333	0.1667	3	17	-0.7294	2	1	1	0	[illegible]
HIP_S0620	AGSLQPLAL	DLQVGQV	AGSLQPLALDLQVGQV		INSC	234/5184	0.997	S0620	0.804	0.097581	1.0333	0	-1	16	0.625	-1	0	2	0	[illegible]
HIP_S0621	YLVCGERGF	TYGKRNAVE	YLVCGERGFTYGKRNAVE		IAPP	2346/45018	0.987	S0621	0.804	-0.021015	-0.75	0.3333	1	18	-0.4778	1	2	0	0	[illegible]
[illegible]																				

HIP_S1090	SLYQLENYC	LRLENVQKF	SLYQLENYCLRLENVQKF		SCG2	19801/334890	0.996	S1090	0.804	-0.107217	0.1333	0	0	18	-0.45	0	1	0	0
HIP_S1091	EQCCTSICS	EKWAEGGGH	EQCCTSICSEKWAEGGGH		SCG1	28681/367830	0.970	S1091	0.804	0.047211	-0.35	0.1667	-1	18	-0.65	-2	1	0	1
HIP_S1092	VEQCCTSIC	GEQEHSQQK	VEQCCTSICGEQEHSQQK		CMGA	6303/247050	0.998	S1092	0.804	0.104648	-0.2	0.3333	-1	18	-1.0056	-2	0	0	0
HIP_S1093	LCGSHLVEA	PGQGSSEDD	LCGSHLVEAPGQGSSEDD		SCG2	8137/334890	0.999	S1093	0.804	0.153261	-0.5	0.3333	-3	18	-0.5444	-4	0	0	0
HIP_S1094	VCGERGFF	EDELSEVLE	VCGERGFFEDELSEVLE		CMGA	11939/247050	0.992	S1094	0.804	0.033892	-0.8833	0.1667	-5	17	-0.1765	-5	2	1	0
HIP_S1095	LCGSHLVEA	RPRHHHGRS	LCGSHLVEARPRHHHGRS		SCG1	40620/367830	0.915	S1095	0.804	0.168551	-1.35	0.1667	6	18	-0.9833	2	0	0	1
HIP_S1096	HLVEALYL	PNGSVEPER	HLVEALYLPNGSVEPER		SCG2	10223/334890	0.999	S1096	0.804	0.183601	0.1333	0.3333	-1	17	-0.3412	-2	1	1	0
HIP_S1097	IVEQCCTSI	FPEYNYDWW	IVEQCCTSIFPEYNYDWW		SCG1	8694/367830	0.998	S1097	0.804	-0.090962	0.1167	0.3333	-3	18	-0.2222	-3	2	0	0
HIP_S1098	QCCTSICSL	YPEEKKEEE	QCCTSICSLYPEEKKEEE		CMGA	9937/247050	0.995	S1098	0.804	0.030683	-0.15	0.3333	-3	18	-1.0111	-3	1	0	1
HIP_S1099	EALYLVCGE	ENSRDNPFK	EALYLVCGEENSRDNPFK		SCG2	17722/334890	0.997	S1099	0.804	-0.108392	-1.5333	0.3333	-2	18	-0.8111	-2	0	0	0
HIP_S1100	GERGFFYT	EALRQAENE	GERGFFYTEALRQAENE		SCG2	18298/334890	0.997	S1100	0.804	-0.085506	0.4833	0	-2	17	-1.1647	-2	3	1	0
HIP_S1101	LVCGERGFF	ALPEPMQES	LVCGERGFFALPEPMQES		CMGA	1355/247050	1.000	S1101	0.804	-0.075160	1.5333	0.3333	-2	18	0.0167	-2	2	0	0
HIP_S1102	LCGSHLVEA	SDTREEKRF	LCGSHLVEASDTREEKRF		SCG1	28083/367830	0.972	S1102	0.804	0.240885	-0.4167	0.1667	0	18	-0.7722	-1	0	0	0
HIP_S1103	ELGGGPGAG	EEEEEEAEA	ELGGGPGAGEEEEEEAEA		CMGA	5233/247050	0.998	S1103	0.804	-0.100522	-1.5833	0.3333	-8	18	-1.2444	-8	0	0	5
HIP_S1104	LVEALYLVC	ELGEYLAR	LVEALYLVCELGEYLAR		SCG2	9770/334890	0.999	S1104	0.804	0.149415	1.7333	0.1667	-2	17	0.9118	-2	1	1	0
HIP_S1105	GSLQPLAL	EERLSKEWE	GSLQPLALEERLSKEWE		CMGA	14913/247050	0.987	S1105	0.804	0.188491	-0.35	0	-2	17	-0.7882	-2	0	1	0
HIP_S1106	SHLVEALYL	RLENVQKF	SHLVEALYLRLENVQKF		SCG2	32270/334890	0.988	S1106	0.804	0.230755	0.35	0	1	17	0.0294	0	1	1	0
HIP_S1107	GGGPGAGSL	SQEKTHSRE	GGGPGAGSLSQEKTHSRE		SCG1	19701/367830	0.990	S1107	0.804	-0.012999	-0.8667	0.5	1	18	-1.2889	0	0	0	1
HIP_S1108	QPLALEGSL	NGERATRLF	QPLALEGSLNGERATRLF		SCG2	10184/334890	0.999	S1108	0.804	-0.049943	-0.8	0.5	0	18	-0.2944	0	0	0	0
HIP_S1109	SLYQLENYC	MLKTGEKPN	SLYQLENYCMLKTGEKPN		SCG2	22280/334890	0.995	S1109	0.804	-0.099315	-0.0833	0	0	18	-0.8667	0	1	0	0
HIP_S1110	ICSLYQLEN	MQESKAEGN	ICSLYQLENMQESKAEGN		CMGA	20618/247050	0.970	S1110	0.804	-0.090593	-1.3833	0	-2	18	-0.7444	-2	1	0	0
HIP_S1111	GPGAGSLQP	SRDNPFKRT	GPGAGSLQPSRDNPFKRT		SCG2	35384/334890	0.984	S1111	0.804	0.088055	-1.6833	0.3333	2	18	-1.2944	2	0	0	0
HIP_S1112	CSLYQLENY	TNEIVEEQY	CSLYQLENYTNEIVEEQY		SCG2	20390/334890	0.996	S1112	0.804	-0.130084	-2.6667	0	-4	18	-0.8111	-4	1	0	0
HIP_S1113	VEQCCTSIC	ESNVSMASL	VEQCCTSICESNVSMASL		SCG1	9995/367830	0.998	S1113	0.804	0.037139	-0.2667	0.3333	-2	18	0.5556	-2	0	0	0
HIP_S1114	CGERGFFYT	ENREEARF	CGERGFFYTENREEARF		SCG1	31932/367830	0.958	S1114	0.804	0.147179	-1.7833	0	-1	17	-1.2412	-1	3	1	0
HIP_S1115	GSHLVEALY	ENHPQESK	GSHLVEALYENHPQESK		SCG1	25020/367830	0.980	S1115	0.804	0.114137	-0.9833	0	0	17	-1.1235	-2	1	1	0
HIP_S1116	ERGFFYTPK	QAKREEEEE	ERGFFYTPKQAKREEEEE		CMGA	11969/247050	0.992	S1116	0.804	-0.058729	-1.9667	0.1667	-2	18	-2.1056	-2	2	0	3
HIP_S1117	DLQVGQVEL	SKEWEDSKR	DLQVGQVELSKEWEDSKR		CMGA	21843/247050	0.965	S1117	0.804	0.200748	-0.6167	0.1667	-2	18	-1.3167	-2	1	0	0
HIP_S1118	PLALEGSLQ	FPNPYNQEK	PLALEGSLQFPNPYNQEK		SCG2	8239/334890	0.999	S1118	0.804	-0.170475	-0.4667	0.3333	-1	18	-0.9	-1	1	0	0
HIP_S1119	HLVEALYL	ESSSRGEA	HLVEALYLESSSRGEA		SCG1	17047/367830	0.993	S1119	0.804	0.234275	0.2	0.3333	-1	16	-0.1938	-2	1	0	1
HIP_S1120	CSLYQLENY	LAVDGAGKP	CSLYQLENYLAVDGAGKP		CMGA	7614/247050	0.997	S1120	0.804	0.023005	0.25	0	-1	18	-0.1111	-1	1	0	0
HIP_S1121	GSLQPLALE	ATQRLANFL	GSLQPLALEATQRLANFL		IAPP	3954/45018	0.959	S1121	0.804	0.031078	-0.05	0	0	18	0.2889	0	0	0	0
HIP_S1122	ELGGGPGAG	TPNRQYWDE	ELGGGPGAGTPNRQYWDE		SCG2	4358/334890	1.000	S1122	0.804	-0.116573	-0.8	0.5	-2	18	-1.3611	-2	0	0	1
HIP_S1123	QVGQVELGG	LALEGSLQ	QVGQVELGGLALEGSLQ		INSC	12/5184	1.000	S1123	0.804	0.126084	2.0667	0.3333	-2	17	0.3235	-2	0	1	0
HIP_S1124	GGPGAGSLQ	EEDIQGPTK	GGPGAGSLQEEDIQGPTK		SCG1	22754/367830	0.985	S1124	0.804	-0.082132	-1.8333	0.1667	-2	18	-1	-2	0	0	0
HIP_S1125	VELGGGPGA	DSEGLSQGL	VELGGGPGADSEGLSQGL		CMGA	4262/247050	0.999	S1125	0.804	-0.090801	-1.3333	0.5	-3	18	-0.1222	-3	0	0	1
HIP_S1126	VCGERGFF	NPYYDPLQ	VCGERGFFNPYYDPLQ		SCG1	20445/367830	0.989	S1126	0.804	0.037665	-0.2	0.3333	-1	16	-0.5625	-1	4	0	0
HIP_S1127	CTSICSLYQ	LNPHPSLGY	CTSICSLYQLNPHPSLGY		CMGA	9618/247050	0.995	S1127	0.804	-0.044804	-0.3833	0.1667	1	18	0.0778	0	1	0	0
HIP_S1128	HLVEALYL	DTEVMKCI	HLVEALYLDTEVMKCI		CMGA	12650/247050	0.991	S1128	0.804	0.175768	-0.2333	0	-1	16	0.6812	-2	1	0	0
HIP_S1129	SLYQLENYC	PHPSLGYKE	SLYQLENYCPHPSLGYKE		CMGA	12112/247050	0.992	S1129	0.804	0.007906	-1.45	0.3333	0	18	-0.9056	-1	1	0	0
HIP_S1130	VCGERGFF	PQGAWKEL	VCGERGFFPQGAWKEL		SCG1	13734/367830	0.996	S1130	0.804	0.129302	-0.05	0.5	0	16	-0.2938	0	2	0	0
HIP_S1131	GERGFFYT	EEEGENYQ	GERGFFYTEEEGENYQ		SCG1	22022/367830	0.986	S1131	0.804	0.077359	-1.6167	0	-4	16	-1.7438	-4	3	0	1
HIP_S1132	GGGPGAGSL	KGERGEDSS	GGGPGAGSLKGERGEDSS		SCG1	16321/367830	0.994	S1132	0.804	-0.008664	-0.8667	0.5	-1	18	-1.1167	-1	0	0	1
HIP_S1133	VEALYLV	EVDKRRT	VEALYLVEVDKRRT		SCG1	22406/367830	0.986	S1133	0.804	0.142755	0.65	0	0	14	-0.2429	0	1	0	1
HIP_S1134	HLVEALYLV	VVPQGLFRG	HLVEALYLVVVPQGLFRG		CMGA	9780/247050	0.995	S1134	0.804	0.013837	2.25	0.1667	1	18	1.0111	0	1	0	1
HIP_S1135	HLVEALYLV	MIRALEYIE	HLVEALYLVMIRALEYIE		SCG2	17922/334890	0.997	S1135	0.804	0.018018	1.4333	0	-1	18	0.9611	-2	1	0	0
HIP_S1136	YQLENYCN	ESWDEEDKR	YQLENYCNESWDEEDKR		SCG1	17794/367830	0.992	S1136	0.804	-0.008225	-1.25	0.1667	-4	17	-2.2294	-4	2	1	0
HIP_S1137	LYQLENYCN	PGETQNAFL	LYQLENYCNPGETQNAFL		SCG1	7783/367830	0.998	S1137	0.804	0.107141	-1.3	0.3333	-2	18	-0.6278	-2	1	0	0

[illegible]

HIP_S1601	QVGQVELGG	EEEEEAEAG	QVGQVELGGEEEEEAEAG		CMGA	5977/247050	0.998	S1601	0.803	-0.104455	-1.25	0.3333	-7	18	-0.9611	-7	0	0	[illegible]
HIP_S1602	AGSLQPLA	GPNNQKRER	AGSLQPLAGPNNQKRER		SCG2	13923/334890	0.998	S1602	0.803	0.166872	-0.25	0.5	2	17	-1.4118	2	0	1	[illegible]
HIP_S1603	VELGGGPGA	DWWEKKPFS	VELGGGPGADWWEKKPFS		SCG1	7461/367830	0.999	S1603	0.803	0.036359	-0.9167	0.3333	-1	18	-0.7278	-1	2	0	[illegible]
HIP_S1604	EQCCTSICS	GEEDATSEV	EQCCTSICSGEEDATSEV		SCG1	7009/367830	0.999	S1604	0.803	0.053365	-0.2	0.3333	-5	18	-0.4	-5	0	0	[illegible]
HIP_S1605	SICSLYQLE	KDQLSDDVS	SICSLYQLEKDQLSDDVS		SCG2	28632/334890	0.991	S1605	0.803	-0.059376	-2.35	0	-3	18	-0.3778	-3	1	0	[illegible]
HIP_S1606	CCTSICSLY	NAVEVLKRE	CCTSICSLYNAVEVLKRE		IAPP	1855/45018	0.992	S1606	0.803	-0.072560	0.7	0.1667	0	18	0.4056	0	1	0	[illegible]
HIP_S1607	VEALYLVCG	GFEDELSEV	VEALYLVCGGFEDELSEV		CMGA	2920/247050	0.999	S1607	0.803	-0.027403	0.8667	0.3333	-5	18	0.5944	-5	2	0	[illegible]
HIP_S1608	GSHLVEALY	DNPGEDAP	GSHLVEALYDNPGEDAP		NPY	1728/49410	0.990	S1608	0.803	0.156788	-0.7167	0.1667	-3	17	-0.6706	-4	1	1	[illegible]
HIP_S1609	GSHLVEALY	PDNPGEDA	GSHLVEALYPDNPGEDA		NPY	1729/49410	0.990	S1609	0.803	0.156788	-0.7167	0.1667	-3	17	-0.6706	-4	1	1	[illegible]
HIP_S1610	ICSLYQLEN	EGNNQAPGE	ICSLYQLENEGNNQAPGE		CMGA	9863/247050	0.995	S1610	0.803	0.023117	-1.7667	0.1667	-3	18	-0.8944	-3	1	0	[illegible]
HIP_S1611	SLQPLAL	VQENQMDK	SLQPLALVQENQMDK		SCG1	19707/367830	0.990	S1611	0.803	0.149426	1.1	0	-1	15	-0.5333	-1	0	1	[illegible]
HIP_S1612	ICSLYQLEN	DLLMKVLEY	ICSLYQLENDLLMKVLEY		SCG2	14345/334890	0.998	S1612	0.803	-0.045950	0.15	0	-2	18	0.4056	-2	1	0	[illegible]
HIP_S1613	GSLQPLALE	INLITRQRY	GSLQPLALEINLITRQRY		NPY	3063/49410	0.975	S1613	0.803	-0.032515	1.15	0	1	18	-0.1	1	0	0	[illegible]
HIP_S1614	LVEALYLVC	GYPEEKKE	LVEALYLVCGYPEEKKE		CMGA	10092/247050	0.995	S1614	0.803	0.158787	1.2	0.3333	-2	17	-0.1353	-2	2	1	[illegible]
HIP_S1615	GSLQPLALE	DQLSDDVSK	GSLQPLALEDQLSDDVSK		SCG2	21161/334890	0.996	S1615	0.803	-0.020659	-0.1833	0	-3	18	-0.45	-3	0	0	[illegible]
HIP_S1616	ERGFFYTP	NNQKRERMD	ERGFFYTPNNQKRERMD		SCG2	28186/334890	0.992	S1616	0.803	0.014779	-2.35	0.1667	1	17	-2.0529	1	2	1	[illegible]
HIP_S1617	SHLVEALY	GIQEEDLRK	SHLVEALYGIQEEDLRK		SCG2	8512/334890	0.999	S1617	0.803	0.159509	0.8167	0.1667	-1	17	-0.5706	-2	1	1	[illegible]
HIP_S1618	EAEDLQVGQ	NTYGKRNAV	EAEDLQVGQNTYGKRNAV		IAPP	1787/45018	0.992	S1618	0.803	-0.101153	-0.8667	0.1667	-1	18	-1.1056	-1	1	0	[illegible]
HIP_S1619	GGGPGAGSL	REEEEEEEE	GGGPGAGSLREEEEEEEE		CMGA	8325/247050	0.996	S1619	0.803	-0.070426	-1.4833	0.3333	-7	18	-1.7389	-7	0	0	[illegible]
HIP_S1620	CTSICSLYQ	KPSPMPVSQ	CTSICSLYQKPSPMPVSQ		CMGA	16361/247050	0.984	S1620	0.803	0.189037	-1.2167	0.3333	1	18	-0.0833	1	1	0	[illegible]
HIP_S1621	SHLVEALYL	GKSGELEQ	SHLVEALYLGKSGELEQ		CMGA	13269/247050	0.990	S1621	0.803	0.239368	0.2	0.3333	-1	17	-0.2118	-2	1	0	[illegible]
HIP_S1622	LYQLENYCN	LNSEKNFPM	LYQLENYCNLNSEKNFPM		SCG2	10870/334890	0.999	S1622	0.803	-0.104302	-0.4667	0.1667	-1	18	-0.8222	-1	1	0	[illegible]
HIP_S1623	LCGSHLVEA	KRERMDEEQ	LCGSHLVEAKRERMDEEQ		SCG2	36442/334890	0.983	S1623	0.803	0.151248	-1.5667	0	-1	18	-1.1278	-2	0	0	[illegible]
HIP_S1624	CSLYQLENY	PAEDMARYY	CSLYQLENYPAEDMARYY		NPY	2994/49410	0.976	S1624	0.803	-0.114639	-1.9333	0.1667	-2	18	-0.7778	-2	1	0	[illegible]
HIP_S1625	GERGFFYTP	SGLTLALSL	GERGFFYTPSGLTLALSL		NPY	1340/49410	0.993	S1625	0.803	-0.125732	-0.1667	0.5	0	18	0.4167	0	3	0	[illegible]
HIP_S1626	LQVGQVELG	EKNFPMDMS	LQVGQVELGEKNFPMDMS		SCG2	27578/334890	0.992	S1626	0.803	-0.049707	-1.8333	0.1667	-2	18	-0.3056	-2	1	0	[illegible]
HIP_S1627	GAGSLQPLA	EDSSEEKHL	GAGSLQPLAEDSSEEKHL		SCG1	10361/367830	0.998	S1627	0.803	0.028174	-0.6333	0.3333	-2	18	-0.8	-3	0	0	[illegible]
HIP_S1628	CTSICSLYQ	PLQWKSSH	CTSICSLYQPLQWKSSH		SCG1	24403/367830	0.981	S1628	0.803	0.038399	-0.3833	0.1667	2	17	-0.2765	1	2	1	[illegible]
HIP_S1629	VCGERGFF	NLRQQAHKE	VCGERGFFNLRQQAHKE		SCG2	27828/334890	0.992	S1629	0.803	0.055627	0.1667	0.1667	2	17	-0.9706	1	2	1	[illegible]
HIP_S1630	HLVEALYLV	HAAGHSQEK	HLVEALYLVHAAGHSQEK		SCG1	26906/367830	0.975	S1630	0.803	0.028393	1.1833	0	2	18	-0.0722	-1	1	0	[illegible]
HIP_S1631	ERGFFYTPK	GGEDWNPVE	ERGFFYTPKGGEDWNPVE		SCG2	11234/334890	0.999	S1631	0.803	-0.118243	-1.75	0.5	-2	18	-1.3	-2	2	0	[illegible]
HIP_S1632	SICSLYQLE	SEDVNWGYE	SICSLYQLESEDVNWGYE		SCG1	22546/367830	0.985	S1632	0.803	-0.091015	-1.8333	0.1667	-4	18	-0.4722	-4	1	0	[illegible]
HIP_S1633	SHLVEALY	ERAHQQKKH	SHLVEALYERAHQQKKH		CMGA	27876/247050	0.932	S1633	0.803	0.228548	-0.3167	0	4	17	-1.3294	1	1	1	[illegible]
HIP_S1634	EQCCTSICS	EKTHSREKS	EQCCTSICSEKTHSREKS		SCG1	36816/367830	0.936	S1634	0.803	0.190334	-0.3167	0.1667	1	18	-1.2278	0	0	0	[illegible]
HIP_S1635	CCTSICSLY	SKGQPRSQE	CCTSICSLYSKGQPRSQE		SCG1	18747/367830	0.991	S1635	0.803	0.140608	-0.5667	0.5	1	18	-0.5722	1	1	0	[illegible]
HIP_S1636	HLVEALYLV	ELTAEKRLE	HLVEALYLVELTAEKRLE		CMGA	10303/247050	0.994	S1636	0.803	0.108146	1.05	0	-1	18	0.1889	-2	1	0	[illegible]
HIP_S1637	GSLQPLALE	AVVPQGLFR	GSLQPLALEAVVPQGLFR		CMGA	12127/247050	0.992	S1637	0.803	0.023721	2.05	0	0	18	0.5667	0	0	0	[illegible]
HIP_S1638	VELGGGPGA	EEGAPGKWQ	VELGGGPGAEEGAPGKWQ		SCG1	6176/367830	0.999	S1638	0.803	-0.089094	-1.2667	0.5	-2	18	-0.7111	-2	0	0	[illegible]
HIP_S1639	HLVEALYL	EPGETQNAF	HLVEALYLEPGETQNAF		SCG1	11152/367830	0.997	S1639	0.803	0.192681	0.1333	0.3333	-2	17	-0.1588	-3	1	1	[illegible]
HIP_S1640	SHLVEALYL	DEKKELEN	SHLVEALYLDEKKELEN		SCG1	25160/367830	0.980	S1640	0.803	0.212904	-0.7667	0	-2	17	-0.7588	-3	1	1	[illegible]
HIP_S1641	AGSLQPLAL	VGGEDWNPV	AGSLQPLALVGGEDWNPV		SCG2	4792/334890	1.000	S1641	0.803	0.158359	2.1333	0.3333	-2	18	0.1833	-2	0	1	[illegible]
HIP_S1642	TSICSLYQL	LEQEEERLS	TSICSLYQLLEQEEERLS		CMGA	11439/247050	0.993	S1642	0.803	-0.052654	-0.7	0	-3	18	-0.4278	-3	1	0	[illegible]
HIP_S1643	FVNQHLCGS	NTYGKRNAV	FVNQHLCGSNTYGKRNAV		IAPP	3133/45018	0.976	S1643	0.803	0.019457	-0.7	0.3333	3	18	-0.55	2	1	0	[illegible]
[illegible]	[illegible]	[illegible]	[illegible]		[illegible]	[illegible]	[illegible]	[illegible]	[illegible]	[illegible]	[illegible]	[illegible]	[illegible]	[illegible]	[illegible]	[illegible]	[illegible]	[illegible]	[illegible]

HIP_S2112	GGPGAGSLQ	SQECFETLR	GGPGAGSLQSQECFETLR		CMGA	14577/247050	0.988	S2112	0.803	-0.095436	-1.3833	0.3333	-1	18	-0.5167	-1	0	0
HIP_S2113	LVEALYLVC	SNQLPKAA	LVEALYLVCSNQLPKAA		SCG2	18950/334890	0.996	S2113	0.803	0.201701	0.45	0.1667	0	17	0.7882	0	1	0
HIP_S2114	SICSLYQL	PQGAWKELD	SICSLYQLPQGAWKELD		SCG1	10093/367830	0.998	S2114	0.803	0.109689	-1.0833	0.3333	-1	17	-0.2059	-1	2	1
HIP_S2115	SLYQLENYC	PSDSQVSEE	SLYQLENYCPSDSQVSEE		SCG1	7828/367830	0.998	S2115	0.803	0.061914	-1.3667	0.3333	-4	18	-0.9778	-4	1	0
HIP_S2116	LYLVCGERG	YNQEKVLPR	LYLVCGERGYNQEKVLPR		SCG2	24192/334890	0.994	S2116	0.803	0.013615	-2.7833	0.1667	1	18	-0.5333	1	1	0
HIP_S2117	GSHLVEALY	LSNALSKSS	GSHLVEALYLSNALSKSS		SCG1	15686/367830	0.994	S2117	0.803	0.089093	0.6333	0.1667	1	18	0.1778	0	1	0
HIP_S2118	GGPGAGSLQ	KTSYFPNPY	GGPGAGSLQKTSYFPNPY		SCG2	31707/334890	0.989	S2118	0.803	0.008299	-0.9833	0.3333	1	18	-0.7667	1	1	0
HIP_S2119	HLCGSHLVE	KGERGEDSS	HLCGSHLVEKGERGEDSS		SCG1	26525/367830	0.976	S2119	0.803	0.188746	-0.55	0.1667	0	18	-1.0056	-2	0	0
HIP_S2120	LQVGQVELG	YENLNDKDQ	LQVGQVELGYENLNDKDQ		SCG2	16945/334890	0.997	S2120	0.803	-0.134073	-1.4	0.1667	-3	18	-0.9833	-3	1	0
HIP_S2121	NQHLCGSHL	QINDEMKRS	NQHLCGSHLQINDEMKRS		SCG2	31164/334890	0.989	S2121	0.803	0.018956	-0.45	0.1667	2	18	-1.1833	0	0	0
HIP_S2122	GQVELGGGP	KTGEKPNGS	GQVELGGGPKTGEKPNGS		SCG2	9682/334890	0.999	S2122	0.803	-0.087247	-1.2333	0.6667	0	18	-1.1611	0	0	0
HIP_S2123	PGAGSLQPL	YTPQSLATL	PGAGSLQPLYTPQSLATL		SCG2	15792/334890	0.997	S2123	0.803	-0.053617	-0.8167	0.3333	0	18	0.1056	0	1	0
HIP_S2124	SICSLYQLE	IILEALRQA	SICSLYQLEIILEALRQA		SCG2	10040/334890	0.999	S2124	0.803	0.011933	1.6	0	-1	18	0.7444	-1	1	0
HIP_S2125	LVEALYLVC	SPETLISDL	LVEALYLVCSPETLISDL		NPY	1735/49410	0.990	S2125	0.803	0.181442	0.7667	0.3333	-3	18	1.1389	-3	1	0
HIP_S2126	GERGFFYT	LSFRARAYG	GERGFFYTLSFRARAYG		CMGA	15468/247050	0.986	S2126	0.803	-0.050668	1.1	0.1667	2	17	-0.3824	2	4	1
HIP_S2127	RGFFYTPKT	EAYPSKPDN	RGFFYTPKTEAYPSKPDN		NPY	3134/49410	0.974	S2127	0.803	0.100712	-1.5333	0.1667	1	18	-1.4111	1	2	0
HIP_S2128	HLVEALYL	SSQETDKL	HLVEALYLSSQETDKL		SCG2	17093/334890	0.997	S2128	0.803	0.243882	0.2	0.3333	-1	16	-0.2188	-2	1	0
HIP_S2129	VEQCCTSIC	YDWWEKKP	VEQCCTSICYDWWEKKP		SCG1	25488/367830	0.979	S2129	0.803	0.017809	0.0833	0.1667	-1	17	-0.6941	-1	3	1
HIP_S2130	VEALYLVCG	AEAASFQRN	VEALYLVCGAEAASFQRN		SCG2	9904/334890	0.999	S2130	0.803	-0.043957	1.0667	0.1667	-1	18	0.4167	-1	1	0
HIP_S2131	CTSICSLYQ	PQGAWKELD	CTSICSLYQPQGAWKELD		SCG1	8865/367830	0.998	S2131	0.803	0.127427	-1.0833	0.3333	-1	18	-0.3056	-1	1	0
HIP_S2132	VCGERGFF	PGEDAPAED	VCGERGFFPGEDAPAED		NPY	994/49410	0.995	S2132	0.803	0.079893	-0.05	0.5	-4	17	-0.6176	-4	2	1
HIP_S2133	EQCCTSICS	GEESEEERG	EQCCTSICSGEESEEERG		SCG1	6971/367830	0.999	S2133	0.803	0.051002	-0.2	0.3333	-5	18	-1.1611	-5	0	0
HIP_S2134	RGFFYTPKT	GLVDREKGL	RGFFYTPKTGLVDREKGL		CMGA	12477/247050	0.991	S2134	0.803	0.039129	0.2333	0.3333	2	18	-0.6611	2	1	0
HIP_S2135	ERGFFYTP	DEPQWSLYP	ERGFFYTPDEPQWSLYP		SCG1	21080/367830	0.988	S2135	0.803	-0.020964	-2.0333	0.3333	-2	17	-1.1353	-2	3	1
HIP_S2136	NQHLCGSHL	RLENVQKFP	NQHLCGSHLRLENVQKFP		SCG2	42947/334890	0.970	S2136	0.803	0.072890	-0.7333	0.1667	3	18	-0.7889	1	0	0
HIP_S2137	PGAGSLQPL	EDSLEAGLP	PGAGSLQPLEDSLEAGLP		CMGA	7923/247050	0.997	S2137	0.803	-0.090579	-1.5167	0.3333	-3	18	-0.1556	-3	0	0
HIP_S2138	CGERGFFYT	RSETHAAG	CGERGFFYTRSETHAAG		SCG1	32293/367830	0.956	S2138	0.803	0.171325	-1.3333	0.1667	1	17	-0.7176	0	3	1
HIP_S2139	GERGFFYT	WGYEKRNLA	GERGFFYTWGYEKRNLA		SCG1	25306/367830	0.979	S2139	0.803	0.032541	-0.3	0.1667	1	17	-1.0353	1	5	1
HIP_S2140	VEALYLVCG	KSSHFERRD	VEALYLVCGKSSHFERRD		SCG1	23823/367830	0.983	S2140	0.803	0.156860	0.1333	0.5	1	18	-0.3778	0	1	0
HIP_S2141	LVCGERGFF	IESHQVEKR	LVCGERGFFIESHQVEKR		IAPP	2054/45018	0.990	S2141	0.803	0.009014	0.9	0.3333	1	18	-0.3833	0	2	0
HIP_S2142	CCTSICSLY	TAEKRKRLG	CCTSICSLYTAEKRKRLG		SCG1	35163/367830	0.944	S2142	0.803	0.023177	-0.1167	0.1667	3	18	-0.2	3	1	0
HIP_S2143	LYQLENYCN	SQTQEEVRD	LYQLENYCNSQTQEEVRD		SCG2	11117/334890	0.999	S2143	0.803	0.063549	-1.2167	0.1667	-3	18	-1.4333	-3	1	0
HIP_S2144	LCGSHLVEA	SHLPERDSL	LCGSHLVEASHLPERDSL		SCG2	26391/334890	0.993	S2144	0.803	0.008608	0.3833	0.1667	0	18	-0.1167	-2	0	0
HIP_S2145	LVCGERGFF	QEEEQIEQ	LVCGERGFFQEEEQIEQ		SCG2	17698/334890	0.997	S2145	0.803	0.049372	-0.8833	0.1667	-4	17	-0.7471	-4	2	1
HIP_S2146	CTSICSLYQ	GPNNQKRE	CTSICSLYQGPNNQKRE		SCG2	13925/334890	0.998	S2146	0.803	0.163515	-1.0833	0.3333	1	17	-1.0706	1	1	1
HIP_S2147	NQHLCGSHL	GSEEYRAPR	NQHLCGSHLGSEEYRAPR		SCG1	18165/367830	0.992	S2147	0.803	0.166136	-0.8167	0.5	2	18	-1.2667	0	0	0
HIP_S2148	GSLQPLAL	ELENLAAMD	GSLQPLALELENLAAMD		SCG1	14023/367830	0.996	S2148	0.803	0.051920	1.0333	0	-3	17	0.3529	-3	0	1
HIP_S2149	VEALYLVC	EVDKRRTR	VEALYLVCEVDKRRTR		SCG1	23518/367830	0.983	S2149	0.803	0.131693	1.2833	0	1	16	-0.3375	1	1	0
HIP_S2150	LVEALYLV	GAGKPGAE	LVEALYLVGAGKPGAE		CMGA	4877/247050	0.998	S2150	0.803	0.184934	1.2833	0.3333	-1	16	0.6375	-1	1	0
HIP_S2151	QHLCGSHLV	LARMLVKYP	QHLCGSHLVLARMLVKYP		SCG2	29768/334890	0.990	S2151	0.803	0.059858	0.9833	0	4	18	0.4111	2	0	0
HIP_S2152	CTSICSLYQ	LKREPLNYL	CTSICSLYQLKREPLNYL		IAPP	5244/45018	0.919	S2152	0.803	0.042909	-0.9333	0	1	18	-0.0389	1	1	0
HIP_S2153	SHLVEALYL	APGEEEEEE	SHLVEALYLAPGEEEEEE		CMGA	262/247050	1.000	S2153	0.803	0.095332	1.0167	0.3333	-6	18	-0.7	-7	1	0
HIP_S2154	YLVCGERGF	QINDEMKRS	YLVCGERGFQINDEMKRS		SCG2	17479/334890	0.997	S2154	0.803	-0.010286	-0.7667	0.1667	0	18	-0.7556	0	1	1

[illegible]

HIP_S2623	EQCCTSICS	QEEDLRKES	EQCCTSICSQEEDLRKES		SCG2	16525/334890	0.997	S2623	0.802	0.039385	-0.7167	0.1667	-3	18	-1.1222	-3	0	0	0	[illegible]
HIP_S2624	LCGSHLVEA	EKTHSREKS	LCGSHLVEAEKTHSREKS		SCG1	44382/367830	0.892	S2624	0.802	0.221903	-0.9333	0	2	18	-0.9222	0	0	0	0	[illegible]
HIP_S2625	LYQLENYC	GSEEYRAPR	LYQLENYCGSEEYRAPR		SCG1	14819/367830	0.995	S2625	0.802	0.147429	-1.1667	0.3333	-1	17	-1.2529	-1	2	1	0	[illegible]
HIP_S2626	GERGFFYTP	LSGLTLALS	GERGFFYTPLSGLTLALS		NPY	1214/49410	0.993	S2626	0.802	-0.160578	-0.1667	0.5	0	18	0.4167	0	3	0	0	[illegible]
HIP_S2627	SICSLYQLE	THPPASLPS	SICSLYQLETHPPASLPS		CMGA	18726/247050	0.977	S2627	0.802	-0.125438	-1.45	0.1667	0	18	0	-1	1	0	0	[illegible]
HIP_S2628	ERGFFYTPK	NLAAMDLEL	ERGFFYTPKNLAAMDLEL		SCG1	11371/367830	0.997	S2628	0.802	0.016015	-0.6833	0.1667	-1	18	-0.2167	-1	2	0	0	[illegible]
HIP_S2629	VEQCCTSIC	KAAWIPHVE	VEQCCTSICKAAWIPHVE		SCG2	22995/334890	0.995	S2629	0.802	-0.005670	0.9833	0.1667	0	18	0.3833	-1	1	0	0	[illegible]
HIP_S2630	LVCGERGFF	KWQQQGDL	LVCGERGFFKWQQQGDL		SCG1	30668/367830	0.963	S2630	0.802	0.067177	-0.5167	0.1667	0	17	-0.4765	0	3	1	1	[illegible]
HIP_S2631	GIVEQCCTS	SEEDWMRII	GIVEQCCTSSEEDWMRII		SCG2	16113/334890	0.997	S2631	0.802	-0.026322	-1.1333	0.3333	-3	18	-0.0556	-3	0	0	0	[illegible]
HIP_S2632	LVEALYLV	TENVPRTRL	LVEALYLVTENVPRTRL		NPY	2884/49410	0.977	S2632	0.802	0.199143	-0.1667	0	0	17	0.3412	0	1	1	0	[illegible]
HIP_S2633	ICSLYQLE	DEPQWSLYP	ICSLYQLEDEPQWSLYP		SCG1	25073/367830	0.980	S2633	0.802	0.039944	-1.9667	0.1667	-3	17	-0.4353	-3	2	1	0	[illegible]
HIP_S2634	LVEALYLV	SEADLDHP	LVEALYLVSEADLDHP		SCG2	9925/334890	0.999	S2634	0.802	0.126657	0.7	0.1667	-3	16	0.3937	-4	1	0	0	[illegible]
HIP_S2635	VEALYLVC	QQKEEEEEM	VEALYLVCQQKEEEEEM		CMGA	16662/247050	0.983	S2635	0.802	0.184139	-0.0667	0	-5	17	-0.6471	-5	1	1	3	[illegible]
HIP_S2636	HLVEALYL	QMDKARRH	HLVEALYLQMDKARRH		SCG1	33020/367830	0.953	S2636	0.802	0.213924	0.2	0	3	16	-0.625	1	1	0	0	[illegible]
HIP_S2637	IVEQCCTSI	EDSKEAEKS	IVEQCCTSIEDSKEAEKS		CMGA	10278/247050	0.994	S2637	0.802	-0.028121	-0.8	0.3333	-3	18	-0.6611	-3	0	0	0	[illegible]
HIP_S2638	GSHLVEALY	QAPGEEEEE	GSHLVEALYQAPGEEEEE		CMGA	3237/247050	0.999	S2638	0.802	0.040296	0.1667	0.1667	-5	18	-0.9333	-6	1	0	3	[illegible]
HIP_S2639	LVEALYLVC	RNHNEGMVT	LVEALYLVCRNHNEGMVT		SCG1	33068/367830	0.953	S2639	0.802	0.153804	-0.1167	0	0	18	0.3389	-1	1	0	0	[illegible]
HIP_S2640	EQCCTSICS	GYPEEKKEE	EQCCTSICSGYPEEKKEE		CMGA	3048/247050	0.999	S2640	0.802	0.031240	0.4833	0.5	-3	18	-1.2444	-3	1	0	0	[illegible]
HIP_S2641	RGFFYTPKT	GKGRHHRGR	RGFFYTPKTGKGRHHRGR		SCG1	33344/367830	0.952	S2641	0.802	0.038067	-1.8167	0.5	8	18	-1.8056	6	1	0	0	[illegible]
HIP_S2642	LVEALYLV	GPPKNDDTP	LVEALYLVGPPKNDDTP		SCG2	8621/334890	0.999	S2642	0.802	0.180370	0.5167	0.5	-2	17	-0.2059	-2	1	1	0	[illegible]
HIP_S2643	LGGGPGAGS	PNGSVEPER	LGGGPGAGSPNGSVEPER		SCG2	6070/334890	0.999	S2643	0.802	-0.049677	-0.8167	0.6667	-1	18	-0.7778	-1	0	0	1	[illegible]
HIP_S2644	GSLQPLAL	FPSPEMIR	GSLQPLALFPSPEMIR		SCG2	13614/334890	0.998	S2644	0.802	0.189921	1.6333	0.3333	0	16	0.2562	0	1	0	0	[illegible]
HIP_S2645	CGSHLVEAL	RPRHHHGRS	CGSHLVEALRPRHHHGRS		SCG1	40061/367830	0.918	S2645	0.802	0.140521	-1.4167	0.1667	6	18	-0.9833	2	0	0	1	[illegible]
HIP_S2646	GSHLVEALY	EGHHRVQE	GSHLVEALYEGHHRVQE		SCG1	18532/367830	0.991	S2646	0.802	0.158889	-0.4667	0.1667	1	17	-0.7765	-2	1	1	0	[illegible]
HIP_S2647	PGAGSLQPL	LLLSLLGAV	PGAGSLQPLLLLSLLGAV		SCG1	8017/367830	0.998	S2647	0.802	0.117945	1.6833	0.1667	0	18	1.3833	0	0	0	2	[illegible]
HIP_S2648	FVNQHLCGS	SYFPNPYNQ	FVNQHLCGSSYFPNPYNQ		SCG2	7449/334890	0.999	S2648	0.802	0.020903	0.3333	0.5	1	18	-0.6889	0	2	0	0	[illegible]
HIP_S2649	CGERGFFYT	ERADEPQW	CGERGFFYTERADEPQW		SCG1	32210/367830	0.957	S2649	0.802	0.078667	-0.9	0	-2	17	-1.2882	-2	3	1	0	[illegible]
HIP_S2650	CSLYQLENY	QGDLQDTKE	CSLYQLENYQGDLQDTKE		SCG1	17748/367830	0.992	S2650	0.802	-0.082339	-2.6167	0.1667	-3	18	-1.25	-3	1	0	0	[illegible]
HIP_S2651	HLVEALYL	GAGKPGAE	HLVEALYLGAGKPGAE		CMGA	7507/247050	0.997	S2651	0.802	0.212457	1.2167	0.3333	0	16	0.175	-1	1	0	0	[illegible]
HIP_S2652	HLVEALYL	DSQVSEEVK	HLVEALYLDSQVSEEVK		SCG1	17289/367830	0.993	S2652	0.802	0.197742	-0.25	0.1667	-2	17	-0.1	-3	1	1	0	[illegible]
HIP_S2653	GSHLVEALY	YPKTPGRA	GSHLVEALYYPKTPGRA		SCG2	28117/334890	0.992	S2653	0.802	0.159152	-0.4167	0.1667	2	17	-0.4588	1	2	1	0	[illegible]
HIP_S2654	FVNQHLCGS	EESWDEEDK	FVNQHLCGSEESWDEEDK		SCG1	12567/367830	0.996	S2654	0.802	-0.072116	-1.0833	0.5	-4	18	-1.3722	-5	1	0	0	[illegible]
HIP_S2655	GGPGAGSLQ	WQAKREEEE	GGPGAGSLQWQAKREEEE		CMGA	6960/247050	0.997	S2655	0.802	-0.075173	-0.5167	0.1667	-2	18	-1.4944	-2	1	0	2	[illegible]
HIP_S2656	SICSLYQLE	EKGHPQEES	SICSLYQLEEKGHPQEES		SCG1	24420/367830	0.981	S2656	0.802	-0.031073	-1.8333	0.1667	-2	18	-1.0667	-3	1	0	0	[illegible]
HIP_S2657	ERGFFYTP	SSQGGSLPS	ERGFFYTPSSQGGSLPS		SCG1	18331/367830	0.992	S2657	0.802	-0.032098	-1.45	0.5	0	17	-0.6882	0	2	1	0	[illegible]
HIP_S2658	LYQLENYCN	SREDSLEAG	LYQLENYCNSREDSLEAG		CMGA	14926/247050	0.987	S2658	0.802	0.123104	-1.85	0.1667	-3	18	-0.9944	-3	1	0	0	[illegible]
HIP_S2659	YQLENYCN	EYNYDWWEK	YQLENYCNEYNYDWWEK		SCG1	26273/367830	0.977	S2659	0.802	0.029764	-1.7667	0	-3	17	-1.9176	-3	3	1	0	[illegible]
HIP_S2660	VEALYLVCG	ENSRDNPFK	VEALYLVCGENSRDNPFK		SCG2	14365/334890	0.998	S2660	0.802	0.098013	-0.25	0.3333	-1	18	-0.3833	-1	1	0	0	[illegible]
HIP_S2661	HLCGSHLVE	PEDLIEMLK	HLCGSHLVEPEDLIEMLK		SCG2	14559/334890	0.998	S2661	0.802	0.096798	-0.6833	0.1667	-1	18	0.0667	-3	0	0	0	[illegible]
HIP_S2662	EQCCTSICS	NFFPEYNYD	EQCCTSICSNFFPEYNYD		SCG1	12104/367830	0.997	S2662	0.802	-0.044623	1.3833	0.1667	-3	18	-0.55	-3	2	0	0	[illegible]
HIP_S2663	LCGSHLVEA	EEATNTHPP	LCGSHLVEAEEATNTHPP		CMGA	19722/247050	0.973	S2663	0.802	0.048808	-0.45	0	-1	18	-0.4611	-3	0	0	0	[illegible]
HIP_S2664	CSLYQLENY	ALLLCAGQV	CSLYQLENYALLLCAGQV		CMGA	6996/247050	0.997	S2664	0.802	0.014348	0.1833	0	-1	18	0.7778	-1	1	0	1	[illegible]
HIP_S2665	SHLVEALYL	KTPGRAGT	SHLVEALYLKTPGRAGT		SCG2	26130/334890	0.993	S2665	0.802	0.261845	0.0167	0.1667	2	17	-0.1059	1	1	1	0	[illegible]
[illegible]																				

HIP_S3134	LGGGPGAGS	RAYFMSDTR	LGGGPGAGSRAYFMSDTR		SCG1	19545/367830	0.990	S3134	0.802	0.071508	-0.5667	0.3333	1	18	-0.4222	1	2	0	1	0.000000	-1.5333	1	-0.1	0.700000	0.1111	0.5889	0	1	0	1	-1.5333	-0.3337	-3.0667	0	1	0	0	0.4269	1.0405	0.9723	0.1667	0.1823	0.9454	-0.2419	0.01	0.1533
HIP_S3135	GSLQPLALE	IQEEDLRKE	GSLQPLALEIQEEDLRKE		SCG2	20579/334890	0.996	S3135	0.802	-0.066105	-0.0667	0	-3	18	-0.7889	-3	0	0	0	0.000000	-1.5333	0	0.05	2.075000	0.3333	1.7417	0	0	0	0	0	-0.1161	0	0	0	1	0	0.4	1.3267	0.6817	0.1667	0.16	0.4647	-0.0267	0.0025	-0.0767
HIP_S3136	LVEALYLVC	GILKLQVF	LVEALYLVCGILKLQVF		IAPP	1804/45018	0.992	S3136	0.802	0.055433	3.0667	0.1667	0	17	1.8	0	1	1	0	0.000000	-0.8667	0	0.0833	2.000000	0.1111	1.8889	0	1	0	1	0	5.7926	-0.8667	0	0	0	0	0.3218	1.0436	0.7295	0.6667	0.1036	0.5322	0.9868	0.0069	-0.0722
HIP_S3137	SLQPLAL	ALSKSSA	SLQPLALALSKSSA		SCG1	22371/367830	0.986	S3137	0.802	0.092199	2.3667	0.1667	1	14	0.6	1	0	0	0	1.000000	-1.5333	0	0.0667	0.900000	0.1111	0.7889	0	0	0	0	0	1.867	0	1	0	0	0	0.2667	1.2667	0.67	0.8333	0.0711	0.4489	0.6311	0.0044	-0.1022
HIP_S3138	SHLVEALY	KTPGRAGTE	SHLVEALYKTPGRAGTE		SCG2	24741/334890	0.994	S3138	0.802	0.254743	-0.3167	0.1667	1	17	-0.5353	0	1	1	0	0.000000	-3.5	1	0.1667	0.000000	0.3333	-0.3333	1	0	1	0	-3.5	0.1056	-3.5	0	1	0	0	0.4269	1.0322	0.909	0.1667	0.1823	0.8262	-0.1352	0.0278	-0.5833
HIP_S3139	GPGAGSLQP	EHSQQKEEE	GPGAGSLQPEHSQQKEEE		CMGA	12380/247050	0.992	S3139	0.802	-0.100900	-1.4667	0.3333	-2	18	-1.7778	-3	0	0	1	0.000000	-2.0667	-1	0	0.000000	0.1111	-0.1111	1	0	1	0	2.0667	0.163	0	0	0	0	1	0.5218	1.0519	0.9745	-0.1667	0.2723	0.9496	-0.7653	0	0
HIP_S3140	HLVEALYLV	DSEEPVST	HLVEALYLVDSEEPVST		SCG1	15795/367830	0.994	S3140	0.802	0.165372	-0.1833	0.1667	-3	17	0.2	-4	1	1	0	0.000000	-4.8333	-2	0.1667	1.400000	0.3333	1.0667	0	0	0	0	9.6667	-0.1956	-4.8333	1	0	0	1	0.4103	1.1188	0.7923	0	0.1683	0.6278	-0.0752	0.0278	-0.8056
HIP_S3141	CSLYQLENY	HRGRGGEPR	CSLYQLENYHRGRGGEPR		SCG1	37432/367830	0.933	S3141	0.802	0.005373	-2.7333	0.1667	2	18	-1.4889	1	1	0	0	0.000000	0.0667	2	0.0333	0.000000	0.3333	-0.3333	0	1	0	1	0.1333	0.9111	0.0667	0	1	0	0	0.5321	0.9574	1.0868	-0.5	0.2831	1.1811	-1.4543	0.0011	0.0022
HIP_S3142	CGSHLVEAL	DEPQWSLYP	CGSHLVEALDEPQWSLYP		SCG1	16101/367830	0.994	S3142	0.802	0.105227	-1.0833	0.1667	-2	18	-0.2611	-3	0	0	0	0.000000	-3.5667	-1	0.1167	1.400000	0.3333	1.0667	1	0	1	0	3.5667	-1.1556	0	1	0	0	0	0.4551	1.2269	0.8445	-0.1667	0.2071	0.7132	-0.4931	0.0136	-0.4161
HIP_S3143	SHLVEALY	ENRQMAYEN	SHLVEALYENRQMAYEN		SCG2	25812/334890	0.993	S3143	0.802	0.233611	-1.2	0	-1	17	-0.8706	-2	1	1	0	0.000000	-5.2667	0	0.15	0.475000	0.3333	0.1417	0	0	0	0	0	-0.17	-5.2667	0	1	0	0	0.4103	1.1622	0.879	0	0.1683	0.7726	-0.4923	0.0225	-0.79
HIP_S3144	GSLQPLAL	HPQLFQNRM	GSLQPLALHPQLFQNRM		SCG2	21346/334890	0.995	S3144	0.802	0.169665	0.1833	0.1667	1	17	-0.2588	0	1	1	0	0.000000	-5.9	-1	0.1167	1.650000	0.1111	1.5389	1	0	1	0	5.9	0.2821	-5.9	1	0	0	0	0.3603	1.1105	0.8323	0.1667	0.1298	0.6927	0.066	0.0136	-0.6883
HIP_S3145	SLYQLENYC	NQAPGEEEE	SLYQLENYCNQAPGEEEE		CMGA	7609/247050	0.997	S3145	0.802	-0.098628	-1.25	0	-5	18	-1.3889	-5	1	0	2	0.000000	-0.9667	0	0.05	0.950000	0.1111	0.8389	0	0	0	0	0	-1.0486	-0.9667	0	1	0	0	0.4769	0.9905	1.0956	0.1667	0.2275	1.2004	-0.5962	0.0025	-0.0483
HIP_S3146	CGERGFFY	KEAEKSGEA	CGERGFFYKEAEKSGEA		CMGA	21490/247050	0.967	S3146	0.802	0.150612	-0.2167	0	-1	17	-1.0529	-1	3	1	0	0.000000	-3.3	0	0.1	0.000000	0.4444	-0.4444	0	0	0	0	0	0.0963	-9.9	0	1	0	0	0.3436	1.2338	0.7356	0.1667	0.1181	0.5412	-0.0744	0.01	-0.33
HIP_S3147	GERGFFYT	TTENENTK	GERGFFYTTTENENTK		SCG1	25755/367830	0.978	S3147	0.802	0.130018	-0.6833	0	-1	16	-1.575	-1	3	0	1	0.000000	-1.9	-1	0.1	0.000000	0.1111	-0.1111	0	0	0	0	1.9	0.0759	-5.7	0	1	0	0	0.5051	1.0369	0.8545	0	0.2552	0.7301	-0.3452	0.01	-0.19
HIP_S3148	GAGSLQPLA	GEKRDHHST	GAGSLQPLAGEKRDHHST		SCG1	20027/367830	0.989	S3148	0.802	0.143686	-0.6333	0.3333	2	18	-1.0667	0	0	0	0	0.000000	-3.9333	0	0.1167	1.900000	0.3333	1.5667	1	1	1	1	0	-0.9922	0	1	0	0	0	0.4718	1.0586	0.9712	0	0.2226	0.9431	-0.2988	0.0136	-0.4589
HIP_S3149	HLCGSHLVE	PRHHHGRSR	HLCGSHLVEPRHHHGRSR		SCG1	44205/367830	0.893	S3149	0.802	0.108391	-0.8	0.1667	7	18	-1.2611	2	0	0	1	0.000000	-4.6	2	0.0667	1.050000	0.2222	0.8278	1	0	1	0	-9.2	-0.6622	0	0	0	1	0	0.4269	1.0805	0.8456	-0.1667	0.1823	0.7151	-0.3415	0.0044	-0.3067
HIP_S3150	AGSLQPLAL	LSLLGAVG	AGSLQPLALLSLLGAVG		SCG1	18875/367830	0.991	S3150	0.802	0.038920	2.7	0.1667	0	17	1.4412	0	0	1	0	1.000000	-0.8667	0	0.0667	2.350000	0	2.35	0	0	0	0	0	6.345	0	1	0	0	0	0.2667	1.2417	0.6433	0.8333	0.0711	0.4139	0.72	0.0044	-0.0578
HIP_S3151	VEQCCTSIC	NGQNGERAT	VEQCCTSICNGQNGERAT		SCG2	9785/334890	0.999	S3151	0.802	0.119290	-0.2	0.3333	-1	18	-0.5833	-1	0	0	0	0.000000	-4.5333	0	0.1167	1.750000	0	1.75	0	1	0	1	0	-0.35	0	0	0	0	0	0.5218	0.8636	1.1028	0.1667	0.2723	1.2162	-0.1044	0.0136	-0.5289
HIP_S3152	SHLVEALY	SVFQELGKL	SHLVEALYSVFQELGKL		SCG2	15098/334890	0.998	S3152	0.802	0.008949	1.75	0.1667	0	17	0.4294	-1	2	1	0	0.000000	0.6333	0	0.0667	1.050000	0.2222	0.8278	0	0	0	0	0	1.4486	1.2667	0	1	0	0	0.3051	1.1386	0.7278	0.6667	0.0931	0.5297	0.534	0.0044	0.0422
HIP_S3153	LVEALYLVC	ITRQRYGKR	LVEALYLVCITRQRYGKR		NPY	4097/49410	0.955	S3153	0.802	0.137182	1.6333	0	3	18	0.0278	3	1	0	0	0.000000	-3.7333	1	0.1	3.125000	0.1111	3.0139	0	0	0	0	-3.7333	4.9227	-3.7333	0	0	0	0	0.3436	1.0522	0.7073	0.3333	0.1181	0.5003	0.5612	0.01	-0.3733

HIP_S3645	GSHLVEALY	MNKGDTEVM	GSHLVEALYMNKGDTEVM		CMGA	16461/247050	0.983	S3645	0.801	0.105566	-0.2	0	-1	18	-0.1722	-2	1	0	0	0.000000	-3.2667	1	0.1	2.475000	0.2222	2.2528	0	0	0	0	-3.2667	-0.4506	-3.2667	0	1	0	0	0.3436	1.1105	0.8556	0.3333	0.1181	0.7321	-0.0687	0.01	-0.3267
HIP_S3646	LVCGERGFF	NTKFEVRL	LVCGERGFFNTKFEVRL		SCG1	30588/367830	0.963	S3646	0.801	0.100168	-0.4833	0.1667	1	17	0.1176	1	3	1	0	0.000000	-4.4333	1	0.0667	1.400000	0.3333	1.0667	0	1	0	1	-4.4333	-0.5156	-13.3	1	0	0	0	0.4551	0.9036	1.0212	0.1667	0.2071	1.0428	-0.22	0.0044	-0.2956
HIP_S3647	LVEALYLVC	GQNGERAT	LVEALYLVCGQNGERAT		SCG2	16535/334890	0.997	S3647	0.801	0.212930	0.5167	0.1667	-1	17	0.2706	-1	1	1	0	0.000000	-5.9667	0	0.1833	2.000000	0	2	0	1	0	1	0	1.0333	-5.9667	0	0	0	0	0.4551	0.9436	0.9912	0.3333	0.2071	0.9824	0.2351	0.0336	-1.0939
HIP_S3648	SICSLYQLE	DKLAPVSKR	SICSLYQLEDKLAPVSKR		SCG2	36394/334890	0.983	S3648	0.801	-0.064512	-1.1333	0	1	18	-0.2056	1	1	0	0	0.000000	-0.1333	1	0	2.350000	0.3333	2.0167	0	0	0	0	-0.1333	-2.2856	-0.1333	0	0	1	0	0.4103	1.2105	0.7556	-0.1667	0.1683	0.571	-0.465	0	0
HIP_S3649	YLVCGERGF	PGEEEEEEE	YLVCGERGFPGEEEEEEE		CMGA	2331/247050	0.999	S3649	0.801	-0.064755	-1.2667	0.5	-7	18	-1.2944	-7	1	0	5	0.000000	-1.1333	-2	0.0667	0.000000	0.4444	-0.4444	1	2	1	1	2.2667	0.563	-1.1333	1	0	0	0	0.5551	0.8836	1.1278	-0.1667	0.3082	1.272	-0.7032	0.0044	-0.0756
HIP_S3650	GSLQPLAL	TSICSLYQ	GSLQPLALTSICSLYQ		INSA	139/3024	0.961	S3650	0.801	0.161647	2.0667	0.1667	0	16	0.6625	0	0	0	0	0.000000	-2.1333	0	0.1167	1.075000	0	1.075	0	0	0	0	0	2.2217	0	1	0	0	0	0.3333	1.1267	0.715	0.6667	0.1111	0.5112	0.6889	0.0136	-0.2489
HIP_S3651	GSHLVEALY	SSAPPITPE	GSHLVEALYSSAPPITPE		SCG1	10336/367830	0.998	S3651	0.801	0.129222	0.75	0.3333	-1	18	0.0056	-2	1	0	0	0.000000	-1.3667	0	0.1333	2.000000	0.1111	1.8889	0	0	0	0	0	1.4167	-1.3667	0	1	0	0	0.3718	1.1369	0.8545	0.5	0.1382	0.7301	0.2788	0.0178	-0.1822
HIP_S3652	LVCGERGFF	QAEGDSEG	LVCGERGFFQAEGDSEG		CMGA	12972/247050	0.991	S3652	0.801	0.012670	0	0.1667	-3	17	-0.3824	-3	2	1	0	0.000000	-3.4667	-1	0.0167	0.700000	0.3333	0.3667	0	1	0	1	3.4667	0	-6.9333	1	0	0	0	0.4167	1.1617	0.8467	0.3333	0.1736	0.7168	0	0.0003	-0.0578
HIP_S3653	GGPGAGSLQ	LLLSLLGAV	GGPGAGSLQLLLSLLGAV		SCG1	11165/367830	0.997	S3653	0.801	0.090646	1.8167	0.1667	0	18	1.2167	0	0	0	1	0.000000	3.9667	0	-0.1167	2.350000	0	2.35	0	0	0	0	0	4.2692	0	0	1	0	0	0.3333	1.185	0.6967	0.6667	0.1111	0.4853	0.6056	0.0136	-0.4628
HIP_S3654	PGAGSLQPL	PQSLATLES	PGAGSLQPLPQSLATLES		SCG2	9654/334890	0.999	S3654	0.801	-0.057228	-1.2	0.5	-1	18	-0.0222	-1	0	0	0	0.000000	-1.5333	0	0.0667	0.950000	0	0.95	2	0	1	0	0	-1.14	0	1	0	0	0	0.5667	0.91	1.1167	0.1667	0.3211	1.2469	-0.68	0.0044	-0.1022
HIP_S3655	SHLVEALY	EEQKLYTDD	SHLVEALYEEQKLYTDD		SCG2	17949/334890	0.997	S3655	0.801	0.149970	-1.0333	0	-3	17	-0.8706	-4	1	1	0	0.000000	-4.9333	-2	0.15	0.950000	0.4444	0.5056	0	0	0	0	9.8667	-0.5224	-4.9333	0	1	0	0	0.4385	1.3169	0.7545	0	0.1922	0.5693	-0.4531	0.0225	-0.74
HIP_S3656	CGERGFFY	VTALPVNSP	CGERGFFYVTALPVNSP		CMGA	15186/247050	0.986	S3656	0.801	0.023002	1.6	0	0	17	0.2235	0	3	1	0	0.000000	0.3333	0	0.05	0.000000	0	0	0	0	0	0	0	0	1	0	1	0	0	0.3051	1.1269	0.7045	0.6667	0.0931	0.4963	0.4882	0.0025	0.0167
HIP_S3657	GSLQPLAL	EEKHLEEPG	GSLQPLALEEKHLEEPG		SCG1	20933/367830	0.988	S3657	0.801	0.166536	-0.25	0	-2	17	-0.7294	-3	0	1	0	0.000000	-6.7667	-1	0.15	1.900000	0.3333	1.5667	0	0	0	0	6.7667	-0.3917	0	1	0	0	0	0.3718	1.3486	0.6745	0	0.1382	0.4549	-0.0929	0.0225	-1.015
HIP_S3658	GSHLVEAL	NYDWWEKKP	GSHLVEALNYDWWEKKP		SCG1	32439/367830	0.956	S3658	0.801	0.056474	-1.0333	0	0	17	-1.0176	-1	3	1	0	0.000000	-3.4667	0	0.05	2.000000	0.2222	1.7778	0	0	0	0	0	-1.837	-10.4	1	0	0	0	0.4103	1.1622	0.879	0	0.1683	0.7726	-0.4239	0.0025	-0.1733
HIP_S3659	GERGFFYTP	LKREPLNYL	GERGFFYTPLKREPLNYL		IAPP	3964/45018	0.959	S3659	0.801	-0.066931	-1.3667	0.1667	1	18	-0.7611	1	3	0	0	0.000000	-0.3333	2	-0.0167	1.650000	0.3333	1.3167	1	0	1	0	-0.6667	-1.7994	-1	0	0	0	1	0.4654	0.9608	0.9501	-0.1667	0.2166	0.9027	-0.636	0.0003	0.0056
HIP_S3660	GSLQPLAL	IKGYPGVQA	GSLQPLALIKGYPGVQA		SCG1	23529/367830	0.983	S3660	0.801	0.217809	1.6	0.1667	1	17	0.3706	1	1	1	0	1.000000	-3.0667	1	0.1333	0.950000	0.1111	0.8389	0	1	0	1	-3.0667	1.3422	-3.0667	1	0	0	0	0.3218	1.1086	0.7562	0.5	0.1036	0.5718	0.5149	0.0178	-0.4089
HIP_S3661	LQPLALEGS	LALEGSLQ	LQPLALEGSLALEGSLQ		INSC	92/5184	0.998	S3661	0.801	0.159437	0.7833	0.3333	-2	17	0.4941	-2	0	1	0	0.000000	4.7	1	-0.2	1.400000	0.2222	1.1778	0	1	0	1	4.7	0.9226	0	0	1	0	0	0.4167	1.1433	0.85	0.3333	0.1736	0.7225	0.3264	0.04	-0.94
HIP_S3662	GSHLVEALY	DPADASEAH	GSHLVEALYDPADASEAH		SCG1	9158/367830	0.998	S3662	0.801	0.088794	0.1667	0.1667	-2	18	-0.35	-4	1	0	0	0.000000	-2.5333	-1	0.1167	2.000000	0.3333	1.6667	1	0	1	0	2.5333	0.2778	-2.5333	0	1	0	0	0.3603	1.1355	0.859	0.3333	0.1298	0.7278	0.06	0.0136	-0.2956
HIP_S3663	HLCGSHLVE	NHPQESKGQ	HLCGSHLVENHPQESKGQ		SCG1	35197/367830	0.944	S3663	0.801	0.085844	-0.6333	0.1667	2	18	-1.15	-1	0	0	0	0.000000	-4.2667	1	0.0667	1.050000	0.1111	0.9389	1	0	1	0	-4.2667	-0.5946	0	0	0	1	0	0.4551	1.0219	0.9545	0	0.2071	0.911	-0.2882	0.0044	-0.2844
HIP_S3664	HLVEALYLV	VEDILNLLG	HLVEALYLVVEDILNLLG		SCG2	13134/334890	0.998	S3664	0.801	0.045612	0.65	0	-2	18	1.2667	-3	1	0	0	1.000000	-3.1667	-2	0.1	3.575000	0.2222	3.3528	0	0	0	0	6.3333	2.1793	-3.1667	1	0	0	0	0.3436	1.1622	0.6756	0.1667	0.1181	0.4565	0.2233	0.01	-0.3167
HIP_S3665	LYLVCGERG	EPQSAPKEN	LYLVCGERGEPQSAPKEN		SCG2	23191/334890	0.995	S3665	0.801	-0.104072	-2.8333	0.3333	-1	18	-0.8833	-1	0	0	0	0.000000	-0.0667	-1	-0.0167	0.625000	0.3333	0.2917	1	1	1	1	0.0667	-0.8264	0	0	0	0	1	0.6051	1.0369	1.0645	-0.5	0.3662	1.1331	-1.7145	0.0003	0.0011
HIP_S3666	PLALEGSLQ	NPYQGVSVP	PLALEGSLQNPYQGVSVP		SCG2	15780/334890	0.997	S3666	0.801	-0.101392	-1.15	0.3333	-1	18	-0.05	-1	1	0	0	0.000000	-1.9667	0	0	0.950000	0.1111	0.8389	1	0	1	0	0	-0.9647	-1.9667	0	1	0	0	0.5218	0.9119	1.1112	0.1667	0.2723	1.2347	-0.6001	0	0
HIP_S3667	CCTSICSLY	VLPRLPYGA	CCTSICSLYVLPRLPYGA		SCG2	13944/334890	0.998	S3667	0.801	-0.067259	1.35	0.3333	1	18	0.9111	1	1	0	0	0.000000	1.5667	0	0	3.125000	0.1111	3.0139	1	0	1	0	0	4.0687	1.5667	0	1	0	0	0.3885	1.0086	0.8545	0.5	0.1509	0.7301	0.5244	0	0
HIP_S3668	VEALYLVCG	ERATRLFEK	VEALYLVCGERATRLFEK		SCG2	26718/334890	0.993	S3668	0.801	0.055669	0.0167	0.1667	0	18	0.1611	0	1	0	0	0.000000	-4.1667	0	0.0167	0.625000	0.2222	0.4028	0	1	0	1	0	0.0067	-4.1667	0	0	0	1	0.4269	1.0938	0.869	0	0.1823	0.7551	0.0071	0.0003	-0.0694
HIP_S3669	GSLQPLAL	IQEEDLRKE	GSLQPLALIQEEDLRKE		SCG2	17268/334890	0.997	S3669	0.801	0.134232	1.15	0	-2	17	-0.6294	-2	0	1	0	1.000000	-3.9667	-1	0.1	0.950000	0.3333	0.6167	0	0	0	0	3.9667	0.7092	0	1	0	0	0	0.3333	1.2917	0.6417	0.5	0.1111	0.4117	0.3833	0.01	-0.3967
HIP_S3670	CGSHLVEAL	SLLVCLGAL	CGSHLVEALSLLVCLGAL		NPY	2352/49410	0.983	S3670	0.801	-0.037977	1.4833	0.1667	0	18	1.7056	-1	0	0	0	0.000000	1.5667	1	0.0167	2.450000	0.1111	2.3389	0	0	0	0	1.5667	3.4694	0	1	0	0	0	0.3333	1.2767	0.6833	0.5	0.1111	0.4669	0.4944	0.0003	0.0261
HIP_S3671	LCGSHLVEA	TEKWAEGGG	LCGSHLVEATEKWAEGGG		SCG1	35628/367830	0.942	S3671	0.801	0.191495	-0.9333	0	-1	18	-0.2056	-2	1	0	1	0.000000	-3.5333	1	0.1	0.000000	0.3333	-0.3333	0	0	0	0	-3.5333	0.3111	-3.5333	1	0	0	0	0.4385	1.2303	0.7512	-0.1667	0.1922	0.5642	-0.4092	0.01	-0.3533
HIP_S3672	SHLVEALYL	FPMDMSDD	SHLVEALYLFPMDMSDD		SCG2	8593/334890	0.999	S3672	0.801	0.015046	1.5667	0.1667	-3	17	0.1353	-4	2	1	0	1.000000	-1.0667	0	0.0667	0.700000	0.2222	0.4778	1	0	1	0	0	0.7485	-2.1333	1	0	0	0	0.3218	1.0986	0.7712	0.6667	0.1036	0.5947	0.5041	0.0044	-0.0711
HIP_S3673	EQCCTSICS	GDERILSIL	EQCCTSICSGDERILSIL		CMGA	7652/247050	0.997	S3673	0.801	0.095890	-0.2	0.3333	-2	18	0.3667	-2	0	0	0	0.000000	-4.5333	-2	0.1167	0.625000	0.3333	0.2917	0	1	0	1	9.0667	-0.0583	0	0	1	0	0	0.4936	0.9888	0.954	-0.1667	0.2436	0.9101	-0.0987	0.0136	-0.5289
HIP_S3674	IVEQCCTSI	EERGLEPGK	IVEQCCTSIEERGLEPGK		SCG1	28104/367830	0.972	S3674	0.801	-0.011729	-1.4167	0.1667	-2	18	-0.4333	-2	0	0	0	0.000000	-4.8333	0	0.0333	0.625000	0.3333	0.2917	0	0	0	0	4.8333	-0.4132	0	1	0	0	0	0.5051	1.1253	0.8362	-0.3333	0.2552	0.6992	-0.7156	0.0011	-0.1611
HIP_S3675	GERGFFYT	AAGSGRLQN	GERGFFYTAAGSGRLQN		SCG2	15651/334890	0.998	S3675	0.801	-0.057643	0.6667	0.1667	1	17	-0.6412	1	3	1	0	0.000000	0.8	0	0.0333	1.150000	0	1.15	0	1	0	1	0	0.7667	2.4	0	1	0	0	0.3885	1.0753	0.8812	0.5	0.1509	0.7764	0.259	0.0011	0.0267
HIP_S3676	LYQLENYCN	KRVPGQGSS	LYQLENYCNKRVPGQGSS		SCG2	42160/334890	0.972	S3676	0.801	0.068314	-1.0833	0	1	18	-1.0111	1	1	0	0	0.000000	-0.6333	2	0.05	0.625000	0.3333	0.2917	0	0	0	0	-1.2667	-0.316	-0.6333	0	1	0	0	0.4205	0.9694	0.9479	-0.1667	0.1768	0.8986	-0.4556	0.0025	-0.0317
HIP_S3677	SHLVEALY	IESHQVEKR	SHLVEALYIESHQVEKR		IAPP	2979/45018	0.978	S3677	0.801	0.201248	0.75	0.1667	1	17	-0.5529	-1	1	1	0	0.000000	-1.3667	-1	0.1167	0.000000	0.2222	-0.2222	0	0	0	0	1.3667	-0.1667	-1.3667	0	1	0	0	0.3718	1.1936	0.7462	0.3333	0.1382	0.5567	0.2788	0.0136	-0.1594
HIP_S3678	QHLCGSHLV	ELQDLALQG	QHLCGSHLVELQDLALQG		CMGA	14589/247050	0.988	S3678	0.801	0.145781	0.2667	0	0	18	0.1111	-2	0	0	0	0.000000	-2.6667	-1	0.1	0.950000	0.2222	0.7278	0	0	0	0	2.6667	0.1941	0	1	0	0	0	0.3718	1.2169	0.6878	0.1667	0.1382	0.4731	0.0991	0.01	-0.2667
HIP_S3679	SHLVEALY	EMKRSGQLG	SHLVEALYEMKRSGQLG		SCG2	33995/334890	0.986	S3679	0.801	0.220708	-0.2	0	1	17	-0.3824	0	1	1	0	0.000000	-3.2667	0	0.1	0.475000	0.4444	0.0306	0	0	0	0	0	-0.0061	-3.2667	0	1	0	0	0.3436	1.2505	0.719	0.1667	0.1181	0.5169	-0.0687	0.01	-0.3267
HIP_S3680	GERGFFYT	QVSEEVKTR	GERGFFYTQVSEEVKTR		SCG1	20878/367830	0.988	S3680	0.801	-0.002312	0.1167	0.1667	0	17	-1.0118	0	3	1	0	0.000000	-0.3	0	0.0667	1.750000	0.2222	1.5278	0	0	0	0	0	0.1782	-0.9	0	1	0	0	0.4385	1.0036	0.8578	0.3333	0.1922	0.7359	0.0512	0.0044	-0.02
HIP_S3681	HLCGSHLVE	GSQENHPQE	HLCGSHLVEGSQENHPQE		SCG1	16193/367830	0.994	S3681	0.801	0.178235	-0.0333	0.3333	0	18	-1.1278	-3	0	0	0	0.000000	-3.0667	1	0.15	1.050000	0.2222	0.8278	0	1	0	1	-3.0667	-0.0276	0	0	0	1	0	0.4833	1.0467	0.9033	0.1667	0.2336	0.816	-0.0161	0.0225	-0.46
HIP_S3682	ERGFFYTPK	QLSDDVSKV	ERGFFYTPKQLSDDVSKV		SCG2	18105/334890	0.997	S3682	0.801	-0.081619	-1.1167	0.3333	0	18	-0.7833	0	2	0	0	0.000000	1.9	-1	-0.05	0.700000	0.2222	0.4778	1	0	1	0	-1.9	-0.5335	3.8	0	0	1	0	0.5218	0.9386	1.0112	0	0.2723	1.0224	-0.5827	0.0025	-0.095
HIP_S3683	LVCGERGFF	ENIEKNEQ	LVCGERGFFENIEKNEQ		SCG2	16554/334890	0.997	S3683	0.801	0.012264	0.45	0.1667	-2	17	-0.7706	-2	2	1	0	0.000000	-2.5667	-1	0.0167	0.700000	0.4444	0.2556	0	1	0	1	2.5667	0.115	-5.1333	1	0	0	0	0.4167	1.0267	0.9117	0.3333	0.1736	0.8311	0.1875	0.0003	-0.0428
HIP_S3684	ERGFFYTPK	GQVTALPVN	ERGFFYTPKGQVTALPVN		CMGA	12203/247050	0.992	S3684	0.801	-0.091591	-0.9833	0.3333	1	18	-0.3333	1	2	0	0	0.000000	2.1667	-1	-0.0333	0.700000	0.1111	0.5889	1	1	1	1	-2.1667	-0.5791	4.3333	0	0	1	0	0.5385	0.8486	1.0612	0	0.2899	1.126	-0.5295	0.0011	-0.0722
HIP_S3685	GERGFFYTP	DPEGKGEQ	GERGFFYTPDPEGKGEQ		CMGA	14850/247050	0.987	S3685	0.801	-0.021059	-2.0333	0.3333	-2	17	-1.5941	-2	3	1	0	0.000000	-1.6667	-2	0.05	0.700000	0.2222	0.4778	2	0	1	0	3.3333	-0.9715	-5	0	0	0	1	0.5769	0.9205	1.0923	-0.3333	0.3328	1.1931	-1.1731	0.0025	-0.0833
HIP_S3686	GSLQPLAL	EESEEERG	GSLQPLALEESEEERG		SCG1	14827/367830	0.995	S3686	0.801	0.155822	0.2667	0.1667	-4	16	-1.0188	-4	0	0	1	0.000000	-5.7333	-2	0.1667	0.450000	0.3333	0.1167	0	0	0	0	11.4667	0.0311	0	1	0	0	0	0.4	1.3117	0.7233	0.1667	0.16	0.5232	0.1067	0.0278	-0.9556
HIP_S3687	CGERGFFY	KGRHHRGR	CGERGFFYKGRHHRGR		SCG1	33976/367830	0.949	S3687	0.801	0.228295	-0.75	0.1667	6	16	-1.6625	4	3	0	0	0.000000	-4.3667	2	0.1833	0.700000	0.3333	0.3667	0	1	0	1	-8.7333	-0.275	-13.1	0	1	0	0	0.3887	0.9941	0.9035	0	0.159	0.8162	-0.299	0.0336	-0.8006
HIP_S3688	LVEALYLVC	LPVDLDDIS	LVEALYLVCLPVDLDDIS		SCG2	11111/334890	0.999	S3688	0.801	-0.006523	2.8167	0.1667	-4	18	1.2611	-4	1	0	0	0.000000	-1.3667	0	0.0667	2.950000	0.1111	2.8389	1	0	1	0	0	7.9962	-1.3667	0	0	0	0	0.3218	1.0519	0.7378	0.6667	0.1036	0.5444	0.9064	0.0044	-0.0911
HIP_S3689	LYQLENYC	EEEEEEEAE	LYQLENYCEEEEEEEAE		CMGA	9876/247050	0.995	S3689	0.801	0.001411	-2.1333	0	-9	17	-1.7176	-9	1	1	5	0.000000	-2.7333	-3	0.1	0.625000	0.6667	-0.0417	0	0	0	0	8.2	0.0889	-2.7333	0	0	0	0	0.5436	1.2072	0.9323	-0.5	0.2955	0.8692	-1.1597	0.01	-0.2733
HIP_S3690	LVEALYLVC	RNYPSLEL	LVEALYLVCRNYPSLEL		SCG1	30981/367830	0.962	S3690	0.801	0.142092	0.2	0	-1	17	0.6882	-1	2	1	0	0.000000	-6.6	1	0.1	2.000000	0.1111	1.8889	0	0	0	0	-6.6	0.3778	-13.2	0	0	0	0	0.3821	1.0158	0.8801	0.1667	0.146	0.7746	0.0764	0.01	-0.66
HIP_S3691	HLVEALYL	SKEWEDSKR	HLVEALYLSKEWEDSKR		CMGA	18380/247050	0.978	S3691	0.801	0.212694	-0.3167	0.1667	0	17	-0.9353	-1	2	1	0	0.000000	-4.8333	0	0.1667	1.400000	0.3333	1.0667	0	0	0	0	0	-0.3278	-9.6667	1	0	0	0	0.4103	1.1588	0.7923	0	0.1683	0.6278	-0.1299	0.0278	-0.8056
HIP_S3692	LVCGERGFF	PQALPEPM	LVCGERGFFPQALPEPM		CMGA	10195/247050	0.995	S3692	0.801	0.006408	0.3167	0.3333	-1	17	0.1765	-1	2	1	0	0.000000	-2.8333	0	0.0333	1.650000	0.2222	1.4278	1	1	1	1	0	0.4521	-5.6667	1	0	0	0	0.4333	1.005	0.9767	0.5	0.1878	0.9539	0.1372	0.0011	-0.0944

[illegible]

HIP_S4156	TSICSLYQL	GGKSGELEQ	TSICSLYQLGGKSGELEQ		CMGA	9500/247050	0.995	S4156	0.801	0.187927	-0.95	0.3333	-1	18	-0.2833	-1	1	0	0	0.000000	-1.2333	1	0.1667	0.000000	0.1111	-0.1111	0	2	0	1	-1.2333	0.1056	-1.2333	1	0	0	0	0.5103	0.9088	1.049	0	0.2604	1.1003	-0.4847	0.0278	-0.2056
HIP_S4157	GSHLVEALY	ENGDESHL	GSHLVEALYENGDESHL		SCG2	12144/334890	0.998	S4157	0.801	0.175937	-0.5167	0.1667	-2	17	-0.6	-4	1	1	0	0.000000	-3.9	-1	0.1833	2.000000	0.3333	1.6667	0	1	0	1	3.9	-0.8611	-3.9	0	1	0	0	0.4551	1.0753	0.9778	0.1667	0.2071	0.9561	-0.2351	0.0036	-0.715
HIP_S4158	GSLQPLALE	ETQNAFLNE	GSLQPLALEETQNAFLNE		SCG1	23136/367830	0.984	S4158	0.801	0.108252	-0.9333	0	-3	18	-0.3556	-3	0	0	0	0.000000	-3.2667	0	0.1	0.950000	0.2222	0.7278	0	0	0	0	0	-0.6793	0	0	0	1	0	0.4667	1.285	0.7633	0	0.2178	0.5827	-0.4356	0.01	-0.3267
HIP_S4159	TSICSLYQL	PGETQNAFL	TSICSLYQLPGETQNAFL		SCG1	11604/367830	0.997	S4159	0.801	0.169822	-1.0833	0.3333	-1	18	0.15	-1	1	0	0	0.000000	-1.5	-1	0.15	0.000000	0.1111	-0.1111	1	1	1	1	1.5	0.1204	-1.5	1	0	0	0	0.5385	1.0019	1.0245	0	0.2899	1.0496	-0.5833	0.0225	-0.225
HIP_S4160	VCGERGFFY	IEMLKTGE	VCGERGFFYIEMLKTGE		SCG2	18515/334890	0.997	S4160	0.801	-0.009329	1.2	0	-1	17	0.0235	-1	3	1	0	0.000000	-0.4667	-1	0.05	2.775000	0.2222	2.5528	0	0	0	0	0.4667	3.0633	-1.4	0	1	0	0	0.3051	1.2019	0.6528	0.5	0.0931	0.4262	0.3662	0.0025	-0.0233
HIP_S4161	QHLCGSHLV	YLNYGEEG	QHLCGSHLVYLNYGEEG		SCG1	25003/367830	0.980	S4161	0.801	0.067888	0.6333	0	0	17	-0.4059	-2	2	1	0	0.000000	-1.9333	0	0.05	0.950000	0	0.95	0	0	0	0	0	0.6017	-3.8667	1	0	0	0	0.3436	1.0622	0.8123	0.3333	0.1181	0.6598	0.2176	0.0025	-0.0967
HIP_S4162	CCTSICSLY	AEFPDFYDS	CCTSICSLYAEFPDFYDS		SCG1	11596/367830	0.997	S4162	0.801	-0.124969	0.4667	0.1667	-3	18	0.3	-3	2	0	0	0.000000	-0.2	-1	-0.0167	2.525000	0.1111	2.4139	0	0	0	0	0.2	1.1265	-0.6	0	1	0	0	0.3718	1.2136	0.7678	0.3333	0.1382	0.5895	0.1735	0.0003	0.0033
HIP_S4163	GERGFFYT	QSAPKENK	GERGFFYTQSAPKENK		SCG2	27743/334890	0.992	S4163	0.801	0.102367	-0.2833	0.1667	1	16	-1.5063	1	3	0	0	0.000000	-1.1	0	0.0667	0.000000	0	0	0	0	0	0	0	0	-3.3	0	1	0	0	0.4385	1.0686	0.8845	0.3333	0.1922	0.7823	-0.1242	0.0044	-0.0733
HIP_S4164	GSHLVEALY	SMPVDNRNH	GSHLVEALYSMPVDNRNH		SCG1	11884/367830	0.997	S4164	0.801	0.104484	0.6333	0.3333	1	18	-0.5611	-1	1	0	0	0.000000	-1.6	0	0.1333	3.050000	0.1111	2.9389	1	0	1	0	0	1.8613	-1.6	0	1	0	0	0.3885	1.0569	0.8978	0.5	0.1509	0.8061	0.246	0.0178	-0.2133
HIP_S4165	AGSLQPLAL	DKQYSSHH	AGSLQPLALDKQYSSHH		SCG1	32231/367830	0.957	S4165	0.801	0.248317	-0.25	0	2	17	-0.6765	0	1	1	0	0.000000	-6.7667	0	0.15	0.450000	0.2222	0.2278	0	0	0	0	0	-0.0569	-6.7667	1	0	0	0	0.3436	1.2005	0.7423	0.1667	0.1181	0.551	-0.0859	0.0225	-1.015
HIP_S4166	HLVEALYL	SGFEDELSE	HLVEALYLSGFEDELSE		CMGA	2995/247050	0.999	S4166	0.801	0.124135	1.3167	0.3333	-4	17	0	-5	2	1	0	0.000000	-1.5667	0	0.15	1.400000	0.2222	1.1778	0	1	0	1	0	1.5507	-3.1333	1	0	0	0	0.3885	1.0203	0.8678	0.5	0.1509	0.7531	0.5115	0.0225	-0.235
HIP_S4167	EQCCTSICS	WWEKKPFS	EQCCTSICSWWEKKPFS		SCG1	28096/367830	0.972	S4167	0.801	0.039098	0.15	0.1667	0	17	-0.5882	0	2	1	0	0.000000	-3.8333	-1	-0.0167	0.625000	0.2222	0.4028	0	0	0	0	3.8333	0.0604	-7.6667	0	1	0	0	0.4487	1.0758	0.8551	0.3333	0.2013	0.7312	0.0673	0.0003	0.0639
HIP_S4168	HLVEALYL	NYPSLELDK	HLVEALYLNYPSLELDK		SCG1	10828/367830	0.990	S4168	0.801	0.120117	-0.0167	0.1667	-1	17	-0.0647	-2	2	1	0	0.000000	-4.2333	0	0.1167	2.350000	0	2.35	1	0	1	0	0	-0.0392	-8.4667	1	0	0	0	0.4269	0.9805	0.9823	0.3333	0.1823	0.9649	-0.0071	0.0136	-0.4939
HIP_S4169	EQCCTSICS	QDKQYSSHH	EQCCTSICSQDKQYSSHH		SCG1	36119/367830	0.939	S4169	0.801	0.180811	-0.7833	0.1667	1	18	-1.1667	-1	0	0	0	0.000000	-5.7	0	0.0833	0.625000	0.2222	0.4028	0	0	0	0	0	-0.3155	0	0	1	0	0	0.4487	1.0091	0.8951	-0.1667	0.2013	0.8013	-0.3515	0.0069	-0.475
HIP_S4170	VCGERGFFY	TPIESHQV	VCGERGFFYTPIESHQV		IAPP	1740/45018	0.993	S4170	0.801	0.132481	1.0833	0.1667	0	17	-0.1412	-1	3	1	0	0.000000	-0.7	0	0.1167	0.700000	0.2222	0.4778	0	0	1	0	0	0.5176	-2.1	0	1	0	0	0.3885	0.9836	0.8428	0.5	0.1509	0.7103	0.4208	0.0136	-0.0817
HIP_S4171	CTSICSLYQ	RGGKSGELE	CTSICSLYQRGGKSGELE		CMGA	11115/247050	0.993	S4171	0.801	0.187490	-1.05	0.3333	0	18	-0.4111	0	1	0	0	0.000000	-1.4333	1	0.1667	0.625000	0.2222	0.4028	0	2	0	1	-1.4333	-0.4229	-1.4333	0	1	0	0	0.5103	0.9088	1.049	0	0.2604	1.1003	-0.5358	0.0278	-0.2389
HIP_S4172	QPLALEGSL	GYEKRNLAR	QPLALEGSLGYEKRNLAR		SCG1	18582/367830	0.991	S4172	0.801	-0.032510	-0.4333	0.5	1	18	-0.7	1	1	0	0	0.000000	-2.6	-1	-0.0167	0.950000	0.3333	0.6167	0	2	0	1	2.6	-0.2672	-2.6	1	0	0	0	0.5385	0.9386	1.0578	0	0.2899	1.119	-0.2333	0.0003	0.0433
HIP_S4173	GERGFFYTP	NENTKFEV	GERGFFYTPNENTKFEV		SCG1	26486/367830	0.976	S4173	0.801	-0.001498	-2.35	0.1667	-1	17	-1.0824	-1	3	1	0	0.000000	-2.3	-1	0.0333	0.700000	0.1111	0.5889	1	0	1	0	2.3	-1.3839	-6.9	0	0	0	1	0.5885	0.8786	1.2078	-0.1667	0.3463	1.4588	-1.3829	0.0011	-0.0767
HIP_S4174	TSICSLYQL	FEVRLLRDP	TSICSLYQLFEVRLLRDP		SCG1	26349/367830	0.977	S4174	0.801	-0.041522	0.4167	0	0	18	0.25	0	2	0	0	1.000000	1.5	-1	0	0.700000	0.2222	0.4778	0	0	0	0	-1.5	0.1991	3	1	0	0	0	0.3718	1.2003	0.7045	0.3333	0.1382	0.4963	0.1549	0	0
HIP_S4175	LVEALYLV	PMYEENSRD	LVEALYLVPMYEENSRD		SCG2	13421/334890	0.998	S4175	0.801	0.023522	0.95	0.1667	-3	17	-0.2059	-3	2	1	0	0.000000	-2.5667	0	0.0667	2.475000	0.2222	2.2528	1	0	1	0	0	2.1401	-5.1333	1	0	0	0	0.3603	1.0288	0.8223	0.5	0.1298	0.6762	0.3422	0.0044	-0.1711
HIP_S4176	GERGFFYTP	SWDEEDKR	GERGFFYTPSWDEEDKR		SCG1	19173/367830	0.991	S4176	0.801	-0.088413	-1.4667	0.3333	-2	17	-1.8176	-2	4	1	0	0.000000	-0.5333	-1	0	0.700000	0.2222	0.4778	1	0	1	0	0.5333	-0.7007	-2.1333	1	0	0	1	0.5321	0.8774	1.0668	0	0.2831	1.1381	-0.7803	0	0
HIP_S4177	GGPGAGSLQ	SEEGEEDAT	GGPGAGSLQSEEGEEDAT		SCG1	13661/367830	0.996	S4177	0.801	-0.116217	-1.3833	0.3333	-5	18	-1.0833	-5	0	0	0	0.000000	-2.4333	-2	0.05	1.400000	0.2222	1.1778	0	0	0	0	4.8667	-1.6293	0	0	1	0	0	0.5333	1.1717	0.8933	-0.1667	0.2844	0.798	-0.7378	0.0025	-0.1217
HIP_S4178	SICSLYQLE	EKSQREDEE	SICSLYQLEEKSQREDEE		SCG1	28283/367830	0.971	S4178	0.801	-0.062472	-1.9	0.1667	-4	18	-1.4167	-4	1	0	0	0.000000	-1.6667	1	0.0667	1.900000	0.3333	1.5667	0	0	0	0	-1.6667	-2.9767	-1.6667	0	0	1	0	0.5051	1.2086	0.8445	-0.3333	0.2552	0.7132	-0.9597	0.0044	-0.1111
HIP_S4179	HLVEALYLV	DTEVMKQ	HLVEALYLVDTEVMKQ		CMGA	12395/247050	0.992	S4179	0.801	0.148769	-0.1667	0	-1	17	0.8882	-2	1	1	0	0.000000	-4.8	-2	0.15	2.450000	0.2222	2.2278	0	0	0	0	9.6	-0.3713	-4.8	1	0	0	0	0.4103	1.1238	0.7523	0	0.1683	0.566	-0.0684	0.0225	-0.72
HIP_S4180	GERGFFYTP	KSSHFERR	GERGFFYTPKSSHFERR		SCG1	31040/367830	0.961	S4180	0.801	0.032135	-1.5167	0.5	3	17	-1.4824	2	3	1	0	0.000000	-0.6333	1	0.0667	0.700000	0.1111	0.5889	1	0	1	0	-0.6333	-0.8921	-1.9	0	0	0	1	0.5603	0.8405	1.1156	-0.1667	0.3139	1.2447	-0.8497	0.0044	-0.0422
HIP_S4181	GGPGAGSLQ	WDEEDKRNY	GGPGAGSLQWDEEDKRNY		SCG1	24336/367830	0.982	S4181	0.801	-0.084086	-1.4	0.1667	-2	18	-1.6667	-2	1	0	0	0.000000	-2.4667	-2	-0.0167	1.400000	0.3333	1.0667	0	0	0	0	4.9333	-1.4933	-2.4667	0	1	0	0	0.4769	1.1272	0.8723	0	0.2275	0.7609	-0.6677	0.0003	0.0411
HIP_S4182	CGSHLVEAL	AAMDLELQK	CGSHLVEALAAMDLELQK		SCG1	17408/367830	0.993	S4182	0.801	-0.009300	1.2667	0	-1	18	0.3833	-2	0	0	0	1.000000	1.1333	1	-0.05	1.850000	0.2222	1.6278	0	0	0	0	1.1333	2.0619	0	1	0	0	0	0.2667	1.3933	0.6367	0.6667	0.0711	0.4053	0.3378	0.0025	-0.0567
HIP_S4183	SLQPLAL	DWWEKKPFS	SLQPLALDWWEKKPFS		SCG1	28559/367830	0.970	S4183	0.801	0.100500	0.6833	0	0	16	-0.5563	0	2	2	0	0.000000	-4.9	-1	0.05	0.950000	0.3333	0.6167	0	0	0	0	4.9	0.4214	-9.8	1	0	0	0	0.3154	1.1858	0.7301	0.6667	0.0995	0.5331	0.2155	0.0025	-0.245
HIP_S4184	CSLYQLENY	SNALSKSSA	CSLYQLENYSNALSKSSA		SCG1	24264/367830	0.982	S4184	0.801	-0.133447	-1.8	0.1667	0	18	-0.3889	0	1	0	0	0.000000	1.9333	1	0.0167	0.950000	0.1111	0.8389	0	0	0	0	1.9333	-1.51	1.9333	0	1	0	0	0.5051	1.0203	1.1045	0	0.2552	1.2199	-0.9092	0.0003	0.0322
HIP_S4185	VELGGGPGA	GPPKNDDTP	VELGGGPGAGPPKNDDTP		SCG2	3281/334890	1.000	S4185	0.801	-0.056400	-0.6333	0.8333	-2	18	-0.9556	-2	0	0	1	0.000000	-1.1333	0	0.0667	0.000000	0.1111	-0.1111	3	2	1	1	0	0.0704	0	1	0	0	0	0.6167	0.6933	1.37	0.1667	0.3803	1.8769	-0.3906	0.0044	-0.0756
HIP_S4186	CTSICSLY	EPGKGRHHR	CTSICSLYEPGKGRHHR		SCG1	21887/367830	0.986	S4186	0.801	0.180913	-0.6333	0.5	4	17	-0.9118	2	1	1	0	0.000000	-2.4	-1	0.1333	0.625000	0.2222	0.4028	1	1	1	1	2.4	-0.2551	-2.4	0	1	0	0	0.5385	0.9503	1.0612	0	0.2899	1.126	-0.341	0.0178	-0.32
HIP_S4187	GERGFFYT	RDNMNDNFL	GERGFFYTRDNMNDNFL		SCG1	31845/367830	0.958	S4187	0.801	0.130966	-1.7833	0	-1	17	-1.1	-1	3	1	0	0.000000	-4.1	0	0.1	0.700000	0.2222	0.4778	0	0	0	0	0	-0.852	-12.3	0	1	0	0	0.4487	0.9608	0.9735	-0.1667	0.2013	0.9476	-0.8002	0.01	-0.41
HIP_S4188	SHLVEALYL	LTLALSLL	SHLVEALYLLTLALSLL		NPY	2547/49410	0.981	S4188	0.801	0.019387	2.2	0	0	17	1.6412	-1	1	1	0	1.000000	0.2	0	0.05	1.400000	0.1111	1.2889	0	0	0	0	0	2.8356	0.2	1	0	0	0	0.3051	1.1753	0.6445	0.6667	0.0931	0.4154	0.6713	0.0025	0.01
HIP_S4189	GSHLVEALY	HPDLFQNRM	GSHLVEALYHPDLFQNRM		SCG2	19087/334890	0.996	S4189	0.801	0.104349	-0.6667	0.1667	1	18	-0.3833	-1	1	0	0	0.000000	-4.2	-1	0.1167	2.950000	0.2222	2.7278	1	0	1	0	4.2	-1.8185	-4.2	0	1	0	0	0.3987	1.0641	0.9001	0	0.159	0.8102	-0.2658	0.0136	-0.49
HIP_S4190	QCCTSICS	GALAEAYPS	QCCTSICSGALAEAYPS		NPY	1149/49410	0.994	S4190	0.801	-0.039707	1.9	0.3333	-1	17	0.4588	-1	0	1	0	0.000000	-0.3333	0	0.0167	0.450000	0.1111	0.3389	0	1	0	1	0	0.6439	0	0	1	0	0	0.3885	1.0253	0.8728	0.5	0.1509	0.7618	0.7381	0.0003	-0.0056
HIP_S4191	VCGERGFF	PRLPYGAGR	VCGERGFFPRLPYGAGR		SCG2	26165/334890	0.993	S4191	0.801	0.075387	0.4833	0.3333	2	17	-0.3059	2	3	1	0	0.000000	-2.5	1	0.0333	0.700000	0.2222	0.4778	1	1	1	1	-2.5	0.2309	-7.5	1	0	0	0	0.4051	0.9653	0.9378	0.3333	0.1641	0.8795	0.1958	0.0011	-0.0833
HIP_S4192	GSHLVEALY	LLGMESAAN	GSHLVEALYLLGMESAAN		SCG2	10833/334890	0.999	S4192	0.801	-0.025411	1.9167	0.1667	-1	18	0.5167	-2	1	0	0	0.000000	0.9667	0	0.0833	3.425000	0.1111	3.3139	0	1	0	1	0	6.3516	0.9667	0	1	0	0	0.3218	1.1453	0.7612	0.6667	0.1036	0.5794	0.6168	0.0069	0.0806
HIP_S4193	GERGFFYTP	IPPEDLIEM	GERGFFYTPIPPEDLIEM		SCG2	5014/334890	1.000	S4193	0.801	-0.188031	-0.3833	0.5	-3	18	-0.3222	-3	3	0	0	0.000000	1.6333	0	0.0167	1.825000	0.1111	1.7139	3	0	1	0	0	-0.657	4.9	0	0	0	1	0.5551	0.7736	1.1495	0.1667	0.3082	1.3213	-0.2128	0.0003	0.0272
HIP_S4194	VCGERGFF	PLQVRGYPE	VCGERGFFPLQVRGYPE		CMGA	10269/247050	0.994	S4194	0.801	0.008027	0.65	0.3333	0	17	-0.2882	0	2	1	[illegible]	[illegible]	[illegible]	[illegible]	[illegible]	[illegible]	[illegible]	[illegible]	[illegible]	[illegible]	[illegible]	[illegible]	[illegible]	[illegible]	[illegible]	[illegible]	[illegible]	[illegible]	[illegible]	[illegible]	[illegible]	[illegible]	[illegible]	[illegible]	[illegible]	[illegible]	[illegible]	[illegible]	[illegible]	[illegible]
HIP_S4195	ELGGGPGAG	PPKNDDTPN	ELGGGPGAGPPKNDDTPN		SCG2	9440/334890	0.999	S4195	0.801	-0.096642	-1.0167	0.6667	-2	18	-1.3833	-2	0	0	[illegible]	[illegible]	[illegible]	[illegible]	[illegible]	[illegible]	[illegible]	[illegible]	[illegible]	[illegible]	[illegible]	[illegible]	[illegible]	[illegible]	[illegible]	[illegible]	[illegible]	[illegible]	[illegible]	[illegible]	[illegible]	[illegible]	[illegible]	[illegible]	[illegible]	[illegible]	[illegible]	[illegible]	[illegible]	[illegible]
HIP_S4196	VCGERGFFY	IENLRQQA	VCGERGFFYIENLRQQA		SCG2	21469/334890	0.995	S4196	0.801	0.096882	0.3	0	0	17	-0.3647	0	3	1	[illegible]	[illegible]	[illegible]	[illegible]	[illegible]	[illegible]	[illegible]	[illegible]	[illegible]	[illegible]	[illegible]	[illegible]	[illegible]	[illegible]	[illegible]	[illegible]	[illegible]	[illegible]	[illegible]	[illegible]	[illegible]	[illegible]	[illegible]	[illegible]	[illegible]	[illegible]	[illegible]	[illegible]	[illegible]	[illegible]
HIP_S4197	HLCGSHLVE	LSLLVCLGA	HLCGSHLVELSLLVCLGA		NPY	2655/49410	0.980	S4197	0.801	-0.024585	1.8833	0.1667	1	18	1.4278	-1	0	0	[illegible]	[illegible]	[illegible]	[illegible]	[illegible]	[illegible]	[illegible]	[illegible]	[illegible]	[illegible]	[illegible]	[illegible]	[illegible]	[illegible]	[illegible]	[illegible]	[illegible]	[illegible]	[illegible]	[illegible]	[illegible]	[illegible]	[illegible]	[illegible]	[illegible]	[illegible]	[illegible]	[illegible]	[illegible]	[illegible]
HIP_S4198	SHLVEALYL	RQMAYENL	SHLVEALYLRQMAYENL		SCG2	31508/334890	0.989	S4198	0.801	0.203300	0.0333	0	0	17	-0.0118	-1	1	1	[illegible]	[illegible]	[illegible]	[illegible]	[illegible]	[illegible]	[illegible]	[illegible]	[illegible]	[illegible]	[illegible]	[illegible]	[illegible]	[illegible]	[illegible]	[illegible]	[illegible]	[illegible]	[illegible]	[illegible]	[illegible]	[illegible]	[illegible]	[illegible]	[illegible]	[illegible]	[illegible]	[illegible]	[illegible]	[illegible]
HIP_S4199	GSLQPLALE	NQMDKARRH	GSLQPLALENQMDKARRH		SCG1	43913/367830	0.895	S4199	0.801	0.093160	-0.5	0	2	18	-1.0833	1	0	0	[illegible]	[illegible]	[illegible]	[illegible]	[illegible]	[illegible]	[illegible]	[illegible]	[illegible]	[illegible]	[illegible]	[illegible]	[illegible]	[illegible]	[illegible]	[illegible]	[illegible]	[illegible]	[illegible]	[illegible]	[illegible]	[illegible]	[illegible]	[illegible]	[illegible]	[illegible]	[illegible]	[illegible]	[illegible]	[illegible]
HIP_S4200	LVEALYLV	EEDATSEVD	LVEALYLVEEDATSEVD		SCG1	15527/367830	0.994	S4200	0.801	0.102487	-0.6333	0	-6	17	0.2235	-6	1	1	[illegible]	[illegible]	[illegible]	[illegible]	[illegible]	[illegible]	[illegible]	[illegible]	[illegible]	[illegible]	[illegible]	[illegible]	[illegible]	[illegible]	[illegible]	[illegible]	[illegible]	[illegible]	[illegible]	[illegible]	[illegible]	[illegible]	[illegible]	[illegible]	[illegible]	[illegible]	[illegible]	[illegible]	[illegible]	[illegible]
HIP_S4201	LVCGERGFF	ELVARSETH	LVCGERGFFELVARSETH		SCG1	13385/367830	0.996	S4201	0.801	-0.021290	1.6167	0.1667	0	18	0.05	-1	2	0	[illegible]	[illegible]	[illegible]	[illegible]	[illegible]	[illegible]	[illegible]	[illegible]	[illegible]	[illegible]	[illegible]	[illegible]	[illegible]	[illegible]	[illegible]	[illegible]	[illegible]	[illegible]	[illegible]	[illegible]	[illegible]	[illegible]	[illegible]	[illegible]	[illegible]	[illegible]	[illegible]	[illegible]	[illegible]	[illegible]
HIP_S4202	CCTSICSLY	AVLALLLCA	CCTSICSLYAVLALLLCA		CMGA	9551/247050	0.995	S4202	0.801	-0.007009	1.9167	0.1667	0	18	2.1944	0	1	0	[illegible]	[illegible]	[illegible]	[illegible]	[illegible]	[illegible]	[illegible]	[illegible]	[illegible]	[illegible]	[illegible]	[illegible]	[illegible]	[illegible]	[illegible]	[illegible]	[illegible]	[illegible]	[illegible]	[illegible]	[illegible]	[illegible]	[illegible]	[illegible]	[illegible]	[illegible]	[illegible]	[illegible]	[illegible]	[illegible]
HIP_S4203	SICSLYQLE	NQMDKARRH	SICSLYQLENQMDKARRH		SCG1	39574/367830	0.921	S4203	0.801	-0.056427	-1.3833	0	2	18	-1.0111	1	1	0	[illegible]	[illegible]	[illegible]	[illegible]	[illegible]	[illegible]	[illegible]	[illegible]	[illegible]	[illegible]	[illegible]	[illegible]	[illegible]	[illegible]	[illegible]	[illegible]	[illegible]	[illegible]	[illegible]	[illegible]	[illegible]	[illegible]	[illegible]	[illegible]	[illegible]	[illegible]	[illegible]	[illegible]	[illegible]	[illegible]

HIP_54667	HLVEALYL	WIPHVENRQ	HLVEALYLWIPHVENRQ		SCG2	15506/334890	0.998	54667	0.800	0.012085	1.3833	0.1667	1	17	-0.1529	-1	2	1	0	1.000000	-1.4333	0	0.0667	3.575000	0	3.575	1	0	1	0	0	4.9454	-2.8667	1	0	0	0	0.3603	1.0488	0.8006	0.6667	0.1298	0.641	0.4984	0.0044	-0.0956
HIP_54668	GSLQPLAL	EDNRDSSM	GSLQPLALEDNRDSSM		CMGA	12665/247050	0.991	54668	0.800	0.154929	-0.1833	0	-2	16	-0.7063	-2	0	0	0	0.000000	-6.6333	-2	0.15	0.450000	0.3333	0.1167	0	0	0	0	13.2667	-0.0214	0	1	0	0	0	0.3718	1.2086	0.8112	0.1667	0.1382	0.658	-0.0682	0.0225	-0.995
HIP_54669	FVNQHLCGS	TRPRHHHGR	FVNQHLCGSTRPRHHHGR		SCG1	34715/367830	0.946	54669	0.800	0.035587	-0.9167	0.5	7	18	-1.3278	3	0	0	1	0.000000	-2.7	1	0.0167	0.000000	0.2222	-0.2222	1	1	1	1	-2.7	0.2037	0	0	1	0	0	0.5769	0.7905	1.1656	-0.1667	0.3328	1.3587	-0.5288	0.0003	-0.045
HIP_54670	VEALYLVC	EAGAPGEED	VEALYLVCEAGAPGEED		SCG1	8961/367830	0.998	54670	0.800	0.131474	1.4	0.1667	-5	17	0.1588	-5	1	1	0	0.000000	-4.2	-1	0.1333	1.075000	0.1111	0.9639	0	1	0	1	4.2	1.3494	-4.2	0	0	0	0	0.3885	1.1403	0.8012	0.3333	0.1509	0.6418	0.5438	0.0178	-0.56
HIP_54671	SICSLYQLE	EESEEGEED	SICSLYQLEEESEEGEED		SCG1	16962/367830	0.993	54671	0.800	-0.100150	-1.8333	0.1667	-8	18	-1.1667	-8	1	0	1	0.000000	-1.5333	-1	0.0667	1.900000	0.4444	1.4556	0	0	0	0	1.5333	-2.6685	-1.5333	0	0	1	0	0.5333	1.29	0.8167	-0.3333	0.2844	0.6669	-0.9778	0.0044	-0.1022
HIP_54672	GERGFFYTP	GAPGEEDIQ	GERGFFYTPGAPGEEDIQ		SCG1	2890/367830	1.000	54672	0.800	-0.171729	-0.6333	0.5	-3	18	-0.9389	-3	3	0	0	0.000000	1.1333	0	0.0333	0.700000	0	0.7	2	1	1	1	0	-0.4433	3.6	0	0	0	1	0.5551	0.8236	1.1778	0.1667	0.3082	1.3873	-0.3516	0.0011	0.0378
HIP_54673	FVNQHLCGS	NGDESHLPE	FVNQHLCGSNGDESHLPE		SCG2	9449/334890	0.999	54673	0.800	-0.063432	-1.0167	0.5	-1	18	-0.7944	-3	0	0	0	0.000000	-2.9	-1	0.0333	0.000000	0.2222	-0.2222	0	2	0	1	2.9	0.2259	0	0	1	0	0	0.5769	0.7522	1.2623	-0.1667	0.3328	1.5934	-0.5865	0.0011	-0.0967
HIP_54674	GSLQPLALE	NYPSLELDK	GSLQPLALENYPSLELDK		SCG1	23366/367830	0.984	54674	0.800	0.044648	-0.7167	0.1667	-2	18	-0.3944	-2	1	0	0	0.000000	-2.8333	1	0.0667	0.950000	0.1111	0.8389	1	0	1	0	-2.8333	-0.6012	-2.8333	0	0	1	0	0.4551	1.0869	0.9812	0.1667	0.2071	0.9627	-0.3262	0.0044	-0.1889
HIP_54675	VCGERGFF	EDVNWGYE	VCGERGFFEDVNWGYE		SCG1	16949/367830	0.993	54675	0.800	-0.002642	0.4	0.1667	-3	16	-0.5563	-3	2	0	0	0.000000	-2.6667	-2	0.0167	0.700000	0.4444	0.2556	0	1	0	1	5.3333	0.1022	-5.3333	1	0	0	0	0.3885	1.0819	0.8078	0.1667	0.1509	0.6526	0.1554	0.0003	-0.0444
HIP_54676	ICSLYQLEN	NNQAPGEEE	ICSLYQLENNNQAPGEEE		CMGA	15629/247050	0.985	54676	0.800	-0.027738	-2.2833	0	-4	18	-1.0667	-4	1	0	2	0.000000	-2.4333	1	0.05	0.450000	0.1111	0.3389	0	0	0	0	-2.4333	-0.7738	-2.4333	0	1	0	0	0.5333	0.9883	1.15	0	0.2844	1.3225	-1.2178	0.0025	-0.1217
HIP_54677	GERGFFYT	PGWQAKREE	GERGFFYTPGWQAKREE		CMGA	10394/247050	0.994	54677	0.800	0.059231	-0.35	0.3333	0	17	-1.4824	0	4	1	0	0.000000	-1.2333	0	0.1	1.150000	0	1.15	1	1	1	1	0	-0.4025	-4.9333	0	1	0	0	0.5103	0.8572	1.0656	0.3333	0.2604	1.1356	-0.1786	0.01	-0.1233
HIP_54678	LCGSHLVEA	GEETGSQEN	LCGSHLVEAGEETGSQEN		SCG1	14950/367830	0.995	54678	0.800	0.151838	-0.8167	0.1667	-3	18	-0.6444	-4	0	0	0	0.000000	-3.3	-1	0.1333	0.000000	0.3333	-0.3333	0	1	0	1	3.3	0.2722	0	1	0	0	0	0.4833	1.2567	0.8133	-0.1667	0.2336	0.6615	-0.3947	0.0178	-0.44
HIP_54679	LCGSHLVEA	ESEESNVSM	LCGSHLVEAESEESNVSM		SCG1	20124/367830	0.989	54679	0.800	0.142519	-0.8833	0.1667	-3	18	-0.1167	-4	0	0	0	0.000000	-3.4333	-1	0.1167	0.000000	0.4444	-0.4444	0	0	0	0	3.4333	0.3926	0	1	0	0	0	0.4667	1.3067	0.7633	-0.1667	0.2178	0.5827	-0.4122	0.0136	-0.4006
HIP_54680	VEALYLVC	QEESEEGEE	VEALYLVCQEESEEGEE		SCG1	16596/367830	0.993	54680	0.800	0.122076	0	0	-7	17	-0.6	-7	1	1	0	0.000000	-7	-2	0.15	0.625000	0.3333	0.2917	0	0	0	0	14	0	-7	0	0	0	0	0.4385	1.2519	0.7278	0	0.1922	0.5297	0	0.0225	-1.05
HIP_54681	VCGERGFFY	RQYWDEDL	VCGERGFFYRQYWDEDL		SCG2	28062/334890	0.992	54681	0.800	0.124898	-0.8333	0	-2	17	-0.8647	-2	5	1	0	0.000000	-4.5333	1	0.1	0.700000	0.2222	0.4778	0	0	0	0	-4.5333	-0.3981	-22.6667	0	1	0	0	0.3821	1.0958	0.8168	0.1667	0.146	0.6672	-0.3184	0.01	-0.4533
HIP_54682	FVNQHLCGS	ENGDESHLP	FVNQHLCGSENGDESHLP		SCG2	9544/334890	0.999	54682	0.800	-0.067971	-1.0167	0.5	-1	18	-0.7944	-3	0	0	0	0.000000	-2.9	-1	0.0333	0.000000	0.2222	-0.2222	0	2	0	1	2.9	0.2259	0	0	1	0	0	0.6051	0.8336	1.2345	-0.1667	0.3662	1.524	-0.6152	0.0011	-0.0967
HIP_54683	ERGFFYTPK	GAVGLAAVN	ERGFFYTPKGAVGLAAVN		SCG1	16185/367830	0.994	54683	0.800	-0.019362	-0.1	0.3333	1	18	0.1667	1	2	0	0	0.000000	3.9333	-1	-0.0833	0.700000	0.1111	0.5889	1	1	1	1	-3.9333	-0.0589	7.8667	0	0	1	0	0.4718	0.9053	1.0078	0.1667	0.2226	1.0157	-0.0472	0.0069	-0.3278
HIP_54684	EQCCTSICS	DTEKWAEGG	EQCCTSICSDTEKWAEGG		SCG1	16723/367830	0.993	54684	0.800	0.041559	-0.25	0.1667	-3	18	-0.6833	-3	0	0	0	0.000000	-4.6333	-2	0.0833	0.625000	0.3333	0.2917	0	0	0	0	9.2667	-0.0729	0	0	1	0	0	0.4769	1.0438	0.864	-0.1667	0.2275	0.7465	-0.1192	0.0069	-0.3861
HIP_54685	GSLQPLAL	KSGYPKTP	GSLQPLALKSGYPKTP		SCG2	23685/334890	0.994	54685	0.800	0.255455	0.7167	0.3333	2	16	-0.4563	2	1	0	0	0.000000	-4.8333	1	0.2	0.450000	0.1111	0.3389	0	1	0	1	-4.8333	0.2429	-4.8333	1	0	0	0	0.3885	1.0619	0.8778	0.3333	0.1509	0.7706	0.2784	0.04	-0.9667
HIP_54686	CGSHLVEAL	EAENEKDRA	CGSHLVEALEAENEKDRA		SCG1	27070/367830	0.975	54686	0.800	-0.018378	-0.5167	0	-2	18	-0.7833	-3	0	0	0	0.000000	-2.4333	-1	0.05	1.400000	0.3333	1.0667	0	0	0	0	2.4333	-0.5511	0	1	0	0	0	0.4	1.455	0.6733	0	0.16	0.4534	-0.2067	0.0025	-0.1217
HIP_54687	GERGFFYT	KELENLAAM	GERGFFYTKELENLAAM		SCG1	33257/367830	0.952	54687	0.800	0.064270	-0.4667	0	-1	17	-0.3824	-1	3	1	0	0.000000	-1.4667	0	0.05	0.700000	0.3333	0.3667	0	0	0	0	0	-0.1711	-4.4	0	1	0	0	0.4103	1.1472	0.769	0	0.1683	0.5913	-0.1915	0.0025	-0.0733
HIP_54688	SHLVEALYL	GMVTRCIIE	SHLVEALYLGMVTRCIIE		SCG1	13028/367830	0.996	54688	0.800	0.068391	2	0.1667	0	18	0.95	-1	1	0	0	0.000000	-0.2	0	0.0833	0.000000	0.1111	-0.1111	0	1	0	1	0	-0.2222	-0.2	1	0	0	0	0.3218	1.0636	0.7512	0.6667	0.1036	0.5642	0.6436	0.0069	-0.0167
HIP_54689	SLQPLALEG	EEERGLEPG	SLQPLALEGEEERGLEPG		SCG1	19847/367830	0.990	54689	0.800	-0.085755	-1.7667	0.1667	-4	18	-0.7611	-4	0	0	1	0.000000	-3.4667	-2	0.0167	0.950000	0.5556	0.3944	0	1	0	1	6.9333	-0.6969	0	0	0	0	1	0.55	1.3067	0.8267	-0.5	0.3025	0.6834	-0.9717	0.0003	-0.0578
HIP_54690	GAGSLQPLA	QQQGDLQDT	GAGSLQPLAQQQGDLQDT		SCG1	16253/367830	0.994	54690	0.800	0.026498	-1.0833	0.1667	-2	18	-0.7667	-2	0	0	1	0.000000	-4.8333	0	0.0833	1.900000	0	1.9	1	0	1	0	0	-2.0583	0	1	0	0	0	0.4833	1.1083	0.9367	0.3333	0.2336	0.8773	-0.5236	0.0069	-0.4028
HIP_54691	GSHLVEALY	TSEVDKRR	GSHLVEALYTSEVDKRR		SCG1	20584/367830	0.989	54691	0.800	0.182540	-0.1167	0.1667	1	17	-0.7529	0	1	1	0	0.000000	-3.1	-1	0.1667	3.050000	0.2222	2.8278	0	0	0	0	3.1	-0.3299	-3.1	0	1	0	0	0.4385	1.1519	0.8278	0.1667	0.1922	0.6853	-0.0512	0.0278	-0.5167
HIP_54692	LVEALYLV	EGSANRRPE	LVEALYLVEGSANRRPE		CMGA	10008/247050	0.995	54692	0.800	0.204022	0.3333	0.3333	-1	17	-0.2176	-1	1	1	0	0.000000	-3.8	-1	0.2	2.000000	0.1111	1.8889	0	1	0	1	3.8	0.6296	-3.8	1	0	0	0	0.4551	1.0319	0.8912	0.1667	0.2071	0.7942	0.1517	0.04	-0.76
HIP_54693	NQHLCGSHL	YLNYGEEGA	NQHLCGSHLYLNYGEEGA		SCG1	19376/367830	0.990	54693	0.800	-0.071850	-0.2	0.1667	0	18	-0.7111	-2	2	0	0	0.000000	-0.2667	0	-0.0167	0.625000	0	0.625	0	0	0	0	0	-0.125	-0.5333	1	0	0	0	0.4103	1.0188	0.929	0.1667	0.1683	0.863	-0.0821	0.0003	0.0044
HIP_54694	HLVEALYLV	EAGAPGEED	HLVEALYLVEAGAPGEED		SCG1	5399/367830	0.999	54694	0.800	0.104225	0.7667	0.1667	-4	18	0.0444	-5	1	0	0	0.000000	-2.9333	-1	0.1333	1.850000	0.1111	1.7389	0	1	0	1	2.9333	1.3331	-2.9333	1	0	0	0	0.3885	1.1403	0.8012	0.3333	0.1509	0.6418	0.2978	0.0178	-0.3911
HIP_54695	GSHLVEALY	LPDGLSVED	GSHLVEALYLPDGLSVED		SCG2	6029/334890	0.999	54695	0.800	0.039827	0.5	0.1667	-3	18	0.1611	-4	1	0	0	0.000000	-1.8667	-1	0.1167	2.950000	0.2222	2.7278	1	0	1	0	1.8667	1.3639	-1.8667	0	1	0	0	0.3603	1.1105	0.8323	0.3333	0.1298	0.6927	0.1801	0.0136	-0.2178
HIP_54696	GERGFFYTP	FRGGKSGEL	GERGFFYTPFRGGKSGEL		CMGA	11686/247050	0.993	54696	0.800	-0.119053	-0.95	0.3333	1	18	-0.7833	1	4	0	0	0.000000	0.5	1	0.0167	1.400000	0.1111	1.2889	1	1	1	1	0.5	-1.2244	2	0	0	0	1	0.5103	0.8572	1.0656	0	0.2604	1.1356	-0.4847	0.0003	0.0083
HIP_54697	CGERGFFY	LPVNSPMNK	CGERGFFYLPVNSPMNK		CMGA	13695/247050	0.989	54697	0.800	0.020086	1.7833	0.1667	1	17	-0.4118	1	3	1	0	0.000000	0.7	0	0.0667	0.000000	0	0	1	0	1	0	0	0	2.1	0	1	0	0	0.3218	1.0586	0.7712	0.6667	0.1036	0.5947	0.5739	0.0044	0.0467
HIP_54698	LVEALYLVC	LGIQEEDLR	LVEALYLVCLGIQEEDLR		SCG2	10839/334890	0.999	54698	0.800	0.007448	3.0667	0.1667	-3	18	0.6944	-3	1	0	0	0.000000	-0.8667	0	0.0833	2.950000	0	2.95	0	1	0	1	0	0.0467	-0.8667	0	0	0	0	0.3218	1.0436	0.7295	0.6667	0.1036	0.5322	0.9868	0.0069	-0.0722
HIP_54699	VEALYLVC	PQSEESWDE	VEALYLVCPQSEESWDE		SCG1	15174/367830	0.995	54699	0.800	0.196607	0.7667	0.3333	-5	17	-0.3588	-5	1	1	0	0.000000	-5.4667	0	0.1833	0.625000	0.2222	0.4028	1	0	1	0	0	0.3088	-5.4667	0	0	0	0	0.4551	0.9769	0.9345	0.3333	0.2071	0.8733	0.3489	0.0336	-1.0022
HIP_54700	CGSHLVEAL	DEMKRSGQL	CGSHLVEALDEMKRSGQL		SCG2	30867/334890	0.989	54700	0.800	0.034380	-0.5	0	0	18	-0.3444	-1	0	0	0	0.000000	-2.4	-1	0.05	1.400000	0.4444	0.9556	0	0	0	0	2.4	-0.4778	0	1	0	0	0	0.3718	1.3319	0.6912	0	0.1382	0.4777	-0.1859	0.0025	-0.12
HIP_54701	QHLCGSHLV	KPDNPGEDA	QHLCGSHLVKPDNPGEDA		NPY	3028/49410	0.975	54701	0.800	0.219768	-0.7	0.1667	0	18	-0.9167	-2	0	0	0	0.000000	-4.6	0	0.1667	0.950000	0.2222	0.7278	1	0	1	0	0	-0.5094	0	1	0	0	0	0.3987	0.9991	0.8735	-0.1667	0.159	0.7629	-0.2791	0.0278	-0.7667
HIP_54702	RGFFYTPKT	HESSSRGEA	RGFFYTPKTHESSSRGEA		SCG1	33179/367830	0.952	54702	0.800	0.005004	-2.2833	0.3333	2	18	-1.2889	1	1	0	1	0.000000	-0.4333	-2	-0.05	0.000000	0.2222	-0.2222	1	0	1	0	0.8667	0.5074	-0.4333	0	1	0	0	0.5603	0.9588	1.039	-0.5	0.3139	1.0795	-1.2793	0.0025	0.0217
HIP_54703	VEALYLVCG	TDKLAPVSK	VEALYLVCGTDKLAPVSK		SCG2	23678/334890	0.994	54703	0.800	0.095756	-0.3	0.1667	0	18	0.5833	0	1	0	0	0.000000	-4.8	0	0.0667	1.575000	0.2222	1.3528	0	1	0	1	0	-0.4058	-4.8	0	0	0	1	0.4654	0.9091	0.9468	-0.1667	0.2166	0.8964	-0.1396	0.0044	-0.32
HIP_54704	LVEALYLV	QNGQNGER	LVEALYLVQNGQNGER		SCG2	15968/334890	0.997	54704	0.800	0.218245	-0.1167	0.1667	-1	16	-0.375	-1	1	0	0	0.000000	-4.7	0	0.1833	1.400000	0	1.4	0	1	0	1	0	-0.1633	-4.7	1	0	0	0	0.4551	0.9436	0.9912	0.3333	0.2071	0.9824	-0.0531	0.0336	-0.8617
HIP_54705	CCTSICSLY	INSNQVKRV	CCTSICSLYINSNQVKRV		SCG2	19494/334890	0.996	54705	0.800	-0.038902	0.3167	0.3333	2	18	0.3	2	1	0	0	0.000000	-0.5	0	0.05	3.200000	0	3.2	0	0	0	0	0	1.0133	-0.5	0	1	0	0	0.4385	0.9453	0.9728	0.3333	0.1922	0.9464	0.1388	0.0025	-0.025
HIP_54706	YQLENYCN	EEEEEEEEA	YQLENYCNEEEEEEEEA		CMGA	10210/247050	0.995	54706	0.800	0.010200	-2.1333	0	-9	17	-2.1471	-9	1	1	6	0.000000	-2.7333	-3	0.1	0.000000	0.5556	-0.5556	0	0	0	0	8.2	1.1852	-2.7333	0	1	0	0	0.5436	1.2072	0.9323	-0.5	0.2955	0.8692	-1.1597	0.01	-0.2733
HIP_54707	YQLENYCN	EEEEEEEEA	YQLENYCNEEEEEEEEA		CMGA	10211/247050	0.995	54707	0.800	0.010200	-2.1333	0	-9	17	-2.1471	-9	1	1	6	0.000000	-2.7333	-3	0.1	0.000000	0.5556	-0.5556	0	0	0	0	8.2	1.1852	-2.7333	0	1	0	0	0.5436	1.2072	0.9323	-0.5	0.2955	0.8692	-1.1597	0.01	-0.2733
HIP_54708	VEALYLVCG	EAEAGEEAV	VEALYLVCGEAEAGEEAV		CMGA	7326/247050	0.997	54708	0.800	-0.059402	0.1833	0.1667	-5	18	0.5722	-5	1	0	0	0.000000	-3.8333	-2	0.0167	1.075000	0.2222	0.8528	0	1	0	1	7.6667	0.1563	-3.8333	0	0	0	1	0.4551	1.1753	0.8412	0	0.2071	0.7075	0.0834	0.0003	-0.0639
HIP_54709	ALYLVCGER	GRSRPDRSS	ALYLVCGERGRSRPDRSS		SCG1	30733/367830	0.963	54709	0.800	-0.056854	-2.35	0.5	2	18	-0.8333	2	0	0	0	0.000000	0.9	1	0.0167	1.050000	0.4444	0.6056	0	2	0	1	0.9	-1.4231	0	0	0	1	0	0.5769	0.8022	1.1256	-0.5	0.3328	1.2671	-1.3558	0.0003	0.015
[illegible]																																														

HIP_SS178	EDLQVGQVE	RSSQGGSLP	EDLQVGQVERSSQGGSLP		SCG1	26346/367830	0.977	SS178	0.800	-0.046596	-1.4833	0.3333	-2	18	-0.8167	-2	0	0	0	[illegible]
HIP_SS179	VEALYLVCG	REDEEEEEG	VEALYLVCGREDEEEEEG		SCG1	15013/367830	0.995	SS179	0.800	0.018608	-0.8667	0.1667	-7	18	-0.7944	-7	1	0	3	[illegible]
HIP_SS180	CGERGFFY	RADEPQWS	CGERGFFYRADEPQWS		SCG1	31391/367830	0.960	SS180	0.800	0.140779	-0.3167	0	-1	16	-1.1562	-1	3	0	0	[illegible]
HIP_SS181	AGSLQPLAL	AENEPQSA	AGSLQPLALAENEPQSA		SCG2	16717/334890	0.997	SS181	0.800	0.138822	0.7	0	-2	17	-0.2412	-2	0	1	0	[illegible]
HIP_SS182	SLYQLENYC	VEEQYTPQS	SLYQLENYCVEEQYTPQS		SCG2	20003/334890	0.996	SS182	0.800	-0.135404	-0.85	0	-3	18	-1	-3	1	0	0	[illegible]
HIP_SS183	CGERGFFYT	GLSAEPGWQ	CGERGFFYTGLSAEPGWQ		CMGA	3823/247050	0.999	SS183	0.800	-0.001816	0.5667	0.3333	-1	18	-0.4556	-1	3	0	0	[illegible]
HIP_SS184	VEALYLVC	QSLATLESV	VEALYLVCQSLATLESV		SCG2	16613/334890	0.997	SS184	0.800	0.179341	1.6667	0.1667	-2	17	1.1647	-2	1	1	0	[illegible]
HIP_SS185	HLVEALYL	IAEKFSQRG	HLVEALYLIAEKFSQRG		SCG1	24663/367830	0.981	SS185	0.800	0.039783	1.5167	0	1	17	0.1118	0	2	1	0	[illegible]
HIP_SS186	GERGFFYTP	RMLVKYPEI	GERGFFYTPRMLVKYPEI		SCG2	15562/334890	0.998	SS186	0.800	-0.067923	-0.4	0.1667	1	18	-0.4	1	3	0	0	[illegible]
HIP_SS187	RGFFYTPKT	RSSQGGSLP	RGFFYTPKTRSSQGGSLP		SCG1	36390/367830	0.938	SS187	0.800	0.004268	-2.05	0.5	3	18	-0.9167	3	1	0	0	[illegible]
HIP_SS188	QCCTSICSL	NQEKVLPRL	QCCTSICSLNQEKVLPRL		SCG2	26090/334890	0.993	SS188	0.800	0.158508	-0.8333	0.1667	1	18	0.0722	1	0	0	0	[illegible]
HIP_SS189	DLQVGQVEL	LKTSRKDVK	DLQVGQVELLKTSRKDVK		SCG1	40751/367830	0.914	SS189	0.800	0.088218	0.6167	0	1	18	-0.6444	1	0	0	0	[illegible]
HIP_SS190	TSICSLYQL	QQAHKEES	TSICSLYQLQQAHKEES		SCG2	30562/334890	0.990	SS190	0.800	0.086457	-1.0333	0	0	17	-0.7412	-1	1	1	0	[illegible]
HIP_SS191	HLVEALYL	EQIEQAIKE	HLVEALYLEQIEQAIKE		SCG2	16124/334890	0.997	SS191	0.800	0.122739	0.6333	0	-2	17	-0.0706	-3	1	1	0	[illegible]
HIP_SS192	QCCTSICSL	VRGYPEEKK	QCCTSICSLVRGYPEEKK		CMGA	15442/247050	0.986	SS192	0.800	0.098082	0.8	0.3333	1	18	-0.4667	1	1	0	0	[illegible]
HIP_SS193	EDLQVGQVE	YLNYGEEGA	EDLQVGQVEYLNYGEEGA		SCG1	8861/367830	0.998	SS193	0.800	-0.172226	-0.6333	0	-5	18	-0.7778	-5	2	0	0	[illegible]
HIP_SS194	LVEALYLVC	ESKGQPRS	LVEALYLVCESKGQPRS		SCG1	26706/367830	0.976	SS194	0.800	0.229534	0.3833	0.1667	0	17	0.0176	0	1	1	0	[illegible]
HIP_SS195	GERGFFYTP	TRLFEKPLD	GERGFFYTPTRLFEKPLD		SCG2	17991/334890	0.997	SS195	0.800	-0.130187	-0.8333	0.1667	0	18	-0.7833	0	4	0	0	[illegible]
HIP_SS196	GSLQPLAL	MSDTREEK	GSLQPLALMSDTREEK		SCG1	17758/367830	0.992	SS196	0.800	0.170980	1.1667	0.1667	-1	16	-0.725	-1	0	0	0	[illegible]
HIP_SS197	LVEALYLV	SQEKTHSR	LVEALYLVSQEKTHSR		SCG1	23149/367830	0.984	SS197	0.800	0.224138	-0.1833	0.1667	1	16	-0.2563	0	1	0	0	[illegible]
HIP_SS198	DLQVGQVEL	WLGAALSLI	DLQVGQVELWLGAALSLI		SCG2	10071/334890	0.999	SS198	0.800	-0.027477	1.1667	0.1667	-2	18	1.0556	-2	1	0	0	[illegible]
HIP_SS199	GERGFFYTP	IESHQVEKR	GERGFFYTPIESHQVEKR		IAPP	2556/45018	0.984	SS199	0.800	-0.142497	-0.5667	0.3333	1	18	-1.1667	0	3	0	0	[illegible]
HIP_SS200	VCGERGFFY	ERDSLSEE	VCGERGFFYERDSLSEE		SCG2	23499/334890	0.994	SS200	0.800	0.173559	-1.2	0	-3	17	-0.8294	-3	3	1	0	[illegible]
HIP_SS201	CGERGFFYT	LISDLLMR	CGERGFFYTLISDLLMR		NPY	2592/49410	0.981	SS201	0.800	-0.074240	1.3833	0.1667	0	17	0.3706	0	3	1	0	[illegible]
HIP_SS202	CSLYQLENY	LPSQKYPGP	CSLYQLENYLPSQKYPGP		CMGA	11454/247050	0.993	SS202	0.800	-0.151879	-1.15	0.3333	0	18	-0.8167	0	1	0	0	[illegible]
HIP_SS203	LVCGERGF	FLVHSSNNF	LVCGERGFFLVHSSNNF		IAPP	1772/45018	0.992	SS203	0.800	0.064128	1.45	0.1667	1	17	0.3706	0	2	1	0	[illegible]
HIP_SS204	LVEALYLVC	QENQMDKA	LVEALYLVCQENQMDKA		SCG1	21197/367830	0.988	SS204	0.800	0.174988	0	0	-2	17	0.0941	-2	1	1	0	[illegible]
HIP_SS205	VCGERGFFY	IKKEELVA	VCGERGFFYIKKEELVA		SCG1	33605/367830	0.951	SS205	0.800	0.132313	0.1667	0	0	17	0.1	0	3	1	0	[illegible]
HIP_SS206	LCGSHLVEA	KSQREDEEE	LCGSHLVEAKSQREDEEE		SCG1	28375/367830	0.971	SS206	0.800	0.142965	-0.95	0.1667	-3	18	-1.2222	-4	0	0	1	[illegible]
HIP_SS207	SLQPLAL	EDLEWERYR	SLQPLALEDLEWERYR		SCG1	22077/367830	0.986	SS207	0.800	0.124204	1.0333	0	-2	16	-0.8813	-2	1	2	0	[illegible]
HIP_SS208	VEALYLVCG	PEYGEEIK	VEALYLVCGPEYGEEIK		SCG1	17117/367830	0.993	SS208	0.800	0.009669	-0.0167	0.3333	-3	17	0.1118	-3	2	1	0	[illegible]
HIP_SS209	SICSLYQLE	MQESKAEGN	SICSLYQLEMQESKAEGN		CMGA	19910/247050	0.972	SS209	0.800	-0.131104	-1.3833	0	-2	18	-0.5944	-2	1	0	0	[illegible]
HIP_SS210	LVEALYLVC	GAPGEEDIQ	LVEALYLVCGAPGEEDIQ		SCG1	6131/367830	0.999	SS210	0.800	0.128726	1.7167	0.3333	-4	18	0.5111	-4	1	0	0	[illegible]
HIP_SS211	SHLVEALY	FFPEYNYDW	SHLVEALYFFPEYNYDW		SCG1	11435/367830	0.997	SS211	0.800	-0.015049	1.3833	0.1667	-2	17	-0.3059	-3	4	1	0	[illegible]
HIP_SS212	LVEALYLV	DRNYLNYGE	LVEALYLVDRNYLNYGE		SCG1	25717/367830	0.978	SS212	0.800	0.159422	-0.8	0	-2	17	-0.0529	-2	2	1	0	[illegible]
HIP_SS213	QVELGGGPG	LALEGSLQ	QVELGGGPGLALEGSLQ		INSC	45/5184	0.999	SS213	0.800	0.157735	1.1667	0.5	-2	17	0.1647	-2	0	1	1	[illegible]
HIP_SS214	GPGAGSLQP	GEEAVPEEE	GPGAGSLQPGEEAVPEEE		CMGA	3416/247050	0.999	SS214	0.800	-0.085248	-1.45	0.3333	-5	18	-0.9222	-5	0	0	1	[illegible]
HIP_SS215	LVCGERGFF	QLSDDVSK	LVCGERGFFQLSDDVSK		SCG2	14924/334890	0.998	SS215	0.800	0.028692	0.7833	0.3333	-1	17	-0.0412	-1	2	1	0	[illegible]
HIP_SS216	CGSHLVEAL	FLISGAEAA	CGSHLVEALFLISGAEAA		SCG2	12963/334890	0.998	SS216	0.800	-0.022472	2.2	0	-1	18	1.1111	-2	1	0	0	[illegible]
HIP_SS217	GAGSLQPLA	QLLHYRKKS	GAGSLQPLAQLLHYRKKS		SCG1	26955/367830	0.975	SS217	0.800	0.017258	1.35	0.1667	4	18	-0.5	3	0	0	0	[illegible]
HIP_SS218	TSICSLYQL	NQAPGEEEE	TSICSLYQLNQAPGEEEE		CMGA	10013/247050	0.995	SS218	0.800	-0.018383	-1.0333	0	-4	18	-0.7611	-4	1	0	2	[illegible]
HIP_SS219	RGFFYTPKT	ESGEETGSQ	RGFFYTPKTESGEETGSQ		SCG1	20400/367830	0.989	SS219	0.800	-0.080287	-1.8167	0.5	-1	18	-1.3667	-1	1	0	0	[illegible]
HIP_SS220	RGFFYTPKT	SGEETGSQE	RGFFYTPKTSGEETGSQE		SCG1	20401/367830	0.989	SS220	0.800	-0.080287	-1.8167	0.5	-1	18	-1.3667	-1	1	0	0	[illegible]

[illegible]

HIP_55689	CTSICSLYQ	GALAEAYPS	CTSICSLYQGALAEAYPS		NPY	1242/49410	0.993	55689	0.799	-0.110879	0.7	0.1667	-1	18	0.4333	-1	1	0	0	
HIP_55690	SICSLYQLE	SVEDILNLL	SICSLYQLESVEDILNLL		SCG2	15819/334890	0.997	55690	0.799	-0.176421	-0.55	0.1667	-3	18	0.75	-3	1	0	0	
HIP_55691	RGFFYTPKT	ENVQKFPSP	RGFFYTPKTENVQKFPSP		SCG2	30186/334890	0.990	55691	0.799	0.044389	-1.5	0.1667	2	18	-1.05	2	1	0	0	
HIP_55692	FVNQHLCGS	QKEEEEEMA	FVNQHLCGSQKEEEEEMA		CMGA	12559/247050	0.991	55692	0.799	-0.080320	-1.6	0.3333	-3	18	-1.0722	-4	0	0	3	
HIP_55693	LQPLALEGS	ENEPQSAPK	LQPLALEGSENEPQSAPK		SCG2	20906/334890	0.996	55693	0.799	-0.095105	-2.5333	0.3333	-2	18	-0.9278	-2	0	0	0	
HIP_55694	GGGPGAGSL	DQELESLSA	GGGPGAGSLDQELESLSA		CMGA	8915/247050	0.996	55694	0.799	-0.097616	-1.3167	0.3333	-3	18	-0.2778	-3	0	0	1	
HIP_55695	SLYQLENYC	AGSGRLQNG	SLYQLENYCAGSGRLQNG		SCG2	7756/334890	0.999	55695	0.799	-0.014241	-0.2833	0.3333	0	18	-0.65	0	1	0	0	
HIP_55696	GSLQPLAL	PSSKDVMEK	GSLQPLALPSSKDVMEK		CMGA	11864/247050	0.992	55696	0.799	0.230033	1.0333	0.5	0	17	-0.2941	0	0	1	0	
HIP_55697	VNQHLCGSH	PYYDPLQWK	VNQHLCGSHPYYDPLQWK		SCG1	15102/367830	0.995	55697	0.799	-0.035608	-1.4333	0.5	2	18	-0.9944	0	2	0	0	
HIP_55698	SLQPLALEG	SKEWEDSKR	SLQPLALEGSKEWEDSKR		CMGA	22719/247050	0.962	55698	0.799	-0.010296	-1.3833	0.3333	-1	18	-1.2167	-1	1	0	0	
HIP_55699	SHLVEALY	FLVHSSNN	SHLVEALYFLVHSSNN		IAPP	2386/45018	0.987	55699	0.799	-0.034398	2.5167	0	1	16	0.2375	-1	2	0	0	
HIP_55700	ERGFFYTPK	ESVFQELGK	ERGFFYTPKESVFQELGK		SCG2	21520/334890	0.995	55700	0.799	-0.089658	-1.05	0.3333	0	18	-0.8389	0	3	0	0	
HIP_55701	VEQCCTSIC	TGPNNQKRE	VEQCCTSICTGPNNQKRE		SCG2	7759/334890	0.999	55701	0.799	0.124659	0.5833	0.5	0	18	-0.9667	0	0	0	0	
HIP_55702	CGERGFFYT	NQEKAEKG	CGERGFFYTNQEKAEKG		SCG2	28455/334890	0.991	55702	0.799	0.138652	-1.6167	0	0	17	-1.3588	0	3	1	0	
HIP_55703	GSLQPLAL	LTEDEKK	GSLQPLALLTEDEKK		SCG1	19954/367830	0.990	55703	0.799	0.136203	1.5	0	-1	15	-0.5533	-1	0	1	0	
HIP_55704	VEALYLVC	FLVHSSNN	VEALYLVCFLVHSSNN		IAPP	2407/45018	0.986	55704	0.799	-0.001984	3.55	0	0	16	0.9062	-1	2	0	0	
HIP_55705	GSLQPLAL	GPGPQLRR	GSLQPLALGPGPQLRR		CMGA	11137/247050	0.993	55705	0.799	0.226225	1.1667	0.5	2	16	-0.3625	2	0	0	0	
HIP_55706	SHLVEALY	EAYPSKPDN	SHLVEALYEAYPSKPDN		NPY	3795/49410	0.962	55706	0.799	0.072004	0.2167	0	-1	17	-0.7706	-2	2	1	0	
HIP_55707	GERGFFYT	TDGARPQAL	GERGFFYTTDGARPQAL		CMGA	14213/247050	0.989	55707	0.799	0.164466	-0.6333	0.1667	0	17	-0.7059	0	3	1	0	
HIP_55708	YLVCGERGF	DNFLEGEEE	YLVCGERGFDNFLEGEEE		SCG1	6087/367830	0.999	55708	0.799	-0.126439	-1.05	0.1667	-5	18	-0.6444	-5	2	0	1	
HIP_55709	ICSLYQLEN	HWLGAALSL	ICSLYQLENHWLGAALSL		SCG2	15201/334890	0.998	55709	0.799	0.071128	-0.5833	0	0	18	0.65	-1	2	0	0	
HIP_55710	DLQVGQVEL	EDMARYYSA	DLQVGQVELEDMARYYSA		NPY	3370/49410	0.970	55710	0.799	0.025823	-0.1	0	-3	18	-0.4333	-3	0	0	0	
HIP_55711	GPGAGSLQP	EESNVSMAS	GPGAGSLQPEESNVSMAS		SCG1	18569/367830	0.991	55711	0.799	-0.030006	-1.5167	0.3333	-2	18	-0.45	-2	0	0	0	
HIP_55712	GPGAGSLQP	SEESNVSMA	GPGAGSLQPSEESNVSMA		SCG1	18570/367830	0.991	55712	0.799	-0.030006	-1.5167	0.3333	-2	18	-0.45	-2	0	0	0	
HIP_55713	VEALYLVC	THSREKSSQ	VEALYLVCTHSREKSSQ		SCG1	30557/367830	0.963	55713	0.799	0.233789	0.9667	0.1667	1	17	-0.3647	0	1	1	0	
HIP_55714	HLCGSHLV	PGWQAKRE	HLCGSHLVPGWQAKRE		CMGA	12093/247050	0.992	55714	0.799	0.211392	0.3167	0.3333	3	16	-0.6125	1	1	0	0	
HIP_55715	ERGFFYTPK	ELSEVLENQ	ERGFFYTPKELSEVLENQ		CMGA	13547/247050	0.990	55715	0.799	-0.096229	-1.1167	0.3333	-2	18	-0.9333	-2	2	0	0	
HIP_55716	NQHLCGSHL	SGEATDGAR	NQHLCGSHLSGEATDGAR		CMGA	9394/247050	0.995	55716	0.799	0.134456	-0.8167	0.5	1	18	-0.8167	-1	0	0	0	
HIP_55717	SHLVEALY	RYRGRGSEE	SHLVEALYRYRGRGSEE		SCG1	40698/367830	0.914	55717	0.799	0.167210	-1	0	1	17	-1.0941	0	2	1	0	
HIP_55718	VCGERGFF	NKPYALNSE	VCGERGFFNKPYALNSE		SCG2	24690/334890	0.994	55718	0.799	0.100720	-0.6333	0.3333	0	17	-0.5294	0	3	1	0	
HIP_55719	NQHLCGSHL	RGGEPRAYF	NQHLCGSHLRGGEPRAYF		SCG1	21913/367830	0.986	55719	0.799	0.186211	-0.9167	0.5	3	18	-0.8944	1	0	0	0	
HIP_55720	AGSLQPLAL	QKREMDE	AGSLQPLALQKREMDE		SCG2	32842/334890	0.987	55720	0.799	0.200367	-0.4167	0	0	17	-0.9588	0	0	1	0	
HIP_55721	GPGAGSLQP	DLFQNRMLS	GPGAGSLQPDLFQNRMLS		SCG2	16646/334890	0.997	55721	0.799	0.029248	0.3	0.1667	0	18	-0.3667	0	1	0	0	
HIP_55722	PGAGSLQPL	PYALNSEKN	PGAGSLQPLPYALNSEKN		SCG2	9536/334890	0.999	55722	0.799	-0.080754	-0.4	0.3333	0	18	-0.6333	0	1	0	0	
HIP_55723	SHLVEALY	LVKYPEIIN	SHLVEALYLVKYPEIIN		SCG2	22230/334890	0.995	55723	0.799	0.024792	1.4	0	0	17	0.4706	-1	2	1	0	
HIP_55724	SLQPLALEG	AEAYPSKPD	SLQPLALEGAEAYPSKPD		NPY	853/49410	0.995	55724	0.799	-0.040668	0	0.1667	-2	18	-0.5111	-2	1	0	0	
HIP_55725	QHLCGSHLV	LQNGQNGER	QHLCGSHLVLQNGQNGER		SCG2	23881/334890	0.994	55725	0.799	0.144828	0.2667	0	2	18	-0.8778	0	0	0	0	
HIP_55726	VCGERGFF	PERKLKHMQ	VCGERGFFPERKLKHMQ		SCG2	28574/334890	0.991	55726	0.799	0.120418	-0.7333	0.3333	3	17	-0.8765	2	2	1	0	
HIP_55727	GSHLVEALY	PGAEEAQDP	GSHLVEALYPGAEEAQDP		CMGA	1367/247050	1.000	55727	0.799	0.100005	0.6833	0.3333	-3	18	-0.5333	-4	1	0	0	
HIP_55728	ICSLYQLEN	LNQGSSQET	ICSLYQLENLNQGSSQET		SCG2	19220/334890	0.996	55728	0.799	-0.168027	-1.0667	0	-2	18	-0.6056	-2	1	0	0	
HIP_55729	GERGFFYTP	DVMEKREDS	GERGFFYTPDVMEKREDS		CMGA	10307/247050	0.994	55729	0.799	-0.072550	-0.1667	0.1667	-2	18	-1.3278	-2	3	0	0	
HIP_55730	YLVCGERGF	SKENIEKNE	YLVCGERGFSKENIEKNE		SCG2	25382/334890	0.993	55730	0.799	0.021188	-1.7167	0.3333	-1	18	-1.0222	-1	1	0	0	
HIP_55731	AGSLQPLAL	IESQTQEE	AGSLQPLALIESQTQEE		SCG2	10919/334890	0.999	55731	0.799	0.114503	1.6	0.1667	-3	17	-0.3412	-3	0	1	0	

HIP_S6200	VEQCCTSIC	RGWRPSSRE	VEQCCTSICRGWRPSSRE		CMGA	19717/247050	0.973	S6200	0.799	0.095365	0.0667	0.3333	1	18	-0.7667	1	1	0	0
HIP_S6201	CTSICSLY	TQQWPERKL	CTSICSLYTQQWPERKL		SCG2	30307/334890	0.990	S6201	0.799	0.135263	-1	0.1667	1	17	-0.5059	1	2	1	0
HIP_S6202	CTSICSLYQ	PLDSQSIYQ	CTSICSLYQPLDSQSIYQ		SCG2	13603/334890	0.998	S6202	0.799	-0.019096	-0.3833	0.1667	-1	18	-0.0278	-1	1	0	0
HIP_S6203	GIVEQCCTS	FPNPYNQEK	GIVEQCCTSFPNPYNQEK		SCG2	12054/334890	0.998	S6203	0.799	-0.110005	-0.2167	0.3333	-1	18	-0.8222	-1	1	0	0
HIP_S6204	SHLVEALY	SRERADEPQ	SHLVEALYSRERADEPQ		SCG1	27118/367830	0.975	S6204	0.799	0.241679	-0.75	0.1667	-1	17	-1.1059	-2	1	1	0
HIP_S6205	GIVEQCCTS	FQRNQLLQK	GIVEQCCTSFQRNQLLQK		SCG2	37619/334890	0.981	S6205	0.799	0.012361	-0.7	0.1667	1	18	-0.4	1	1	0	0
HIP_S6206	DLQVGQVEL	LSKSGYPKT	DLQVGQVELLSKSGYPKT		SCG2	31806/334890	0.988	S6206	0.799	0.096951	0.6	0.1667	0	18	-0.4444	0	0	0	0
HIP_S6207	GERGFFYTP	SSNNFGAIL	GERGFFYTPSSNNFGAIL		IAPP	2204/45018	0.989	S6207	0.799	-0.032344	-1.45	0.5	0	18	-0.1611	0	3	0	0
HIP_S6208	CCTSICSLY	EATNTHPPA	CCTSICSLYEATNTHPPA		CMGA	14716/247050	0.987	S6208	0.799	-0.073011	-0.1167	0.1667	0	18	0.0556	-1	1	0	0
HIP_S6209	GSLQPLAL	VEPERELDL	GSLQPLALVEPERELDL		SCG2	9863/334890	0.999	S6209	0.799	0.128197	1.4167	0.1667	-3	17	-0.0824	-3	0	1	0
HIP_S6210	HLVEALYLV	SLEAGLPLQ	HLVEALYLVSLEAGLPLQ		CMGA	9821/247050	0.995	S6210	0.799	0.130531	1.0333	0.1667	-1	18	0.9444	-2	1	0	0
HIP_S6211	VCGERGFFY	INLITRQR	VCGERGFFYINLITRQR		NPY	3234/49410	0.972	S6211	0.799	0.006764	1.5167	0	2	17	-0.1	2	3	0	0
HIP_S6212	CTSICSLYQ	EPMQESKAE	CTSICSLYQEPMQESKAE		CMGA	8718/247050	0.996	S6212	0.799	-0.034164	-0.7	0.1667	-2	18	-0.5778	-2	1	0	0
HIP_S6213	GGPGAGSLQ	SEDVNWGYE	GGPGAGSLQSEDVNWGYE		SCG1	18476/367830	0.991	S6213	0.799	-0.116754	-1.3833	0.3333	-3	18	-0.8389	-3	0	0	0
HIP_S6214	LVCGERGFF	HPQEESEE	LVCGERGFFHPQEESEE		SCG1	21125/367830	0.988	S6214	0.799	0.021371	-0.5167	0.3333	-3	17	-0.9294	-4	2	1	0
HIP_S6215	CSLYQLENY	PGEEEEEEE	CSLYQLENYPGEEEEEEE		CMGA	5488/247050	0.998	S6215	0.799	-0.071806	-2.3	0.3333	-8	18	-1.6833	-8	1	0	5
HIP_S6216	CGERGFFYT	EAHESSSR	CGERGFFYTEAHESSSR		SCG1	33810/367830	0.950	S6216	0.799	0.050737	-0.6833	0	0	17	-1.0588	-1	3	1	1
HIP_S6217	GSHLVEALY	AFLNERKQ	GSHLVEALYAFLNERKQ		SCG1	22240/367830	0.986	S6217	0.799	-0.023333	2.1167	0	1	17	-0.3588	0	2	1	0
HIP_S6218	VEALYLVCG	VTALPVNSP	VEALYLVCGVTALPVNSP		CMGA	7870/247050	0.997	S6218	0.799	-0.044226	1.9333	0.1667	-1	18	1.1611	-1	1	0	0
HIP_S6219	CCTSICSLY	QAENEPQSA	CCTSICSLYQAENEPQSA		SCG2	18525/334890	0.997	S6219	0.799	-0.080548	-0.5833	0.1667	-2	18	-0.2278	-2	1	0	0
HIP_S6220	YLVCGERGF	FREEARFQDK	YLVCGERGFREEARFQDK		SCG1	32459/367830	0.956	S6220	0.799	0.018952	-2.2667	0.1667	0	18	-1.0611	0	1	0	0
HIP_S6221	EQCCTSICS	QQGDLQDTK	EQCCTSICSQQGDLQDTK		SCG1	12162/367830	0.997	S6221	0.799	0.144984	-0.2	0.3333	-2	18	-0.8889	-2	0	0	0
HIP_S6222	GQVELGGG	VELGGGP	GQVELGGGVELGGGP		INSC	42/5184	1.000	S6222	0.799	0.146133	0.55	0.5	-2	15	0.0733	-2	0	1	2
HIP_S6223	GGGPGAGSL	YALNSEKNF	GGGPGAGSLYALNSEKNF		SCG2	6829/334890	0.999	S6223	0.799	0.149351	1.15	0.3333	0	18	-0.3833	0	1	0	1
HIP_S6224	CGERGFFYT	PSSREDSL	CGERGFFYTPSSREDSL		CMGA	10986/247050	0.994	S6224	0.799	0.166389	-0.4	0.5	-1	17	-0.8471	-1	3	1	0
HIP_S6225	QVGQVELGG	QEEEEDNRD	QVGQVELGGQEEEEDNRD		CMGA	6339/247050	0.998	S6225	0.799	-0.124970	-1.25	0.3333	-6	18	-1.7778	-6	0	0	2
HIP_S6226	GSHLVEALY	KPFSEDVN	GSHLVEALYKPFSEDVN		SCG1	20716/367830	0.988	S6226	0.799	0.128527	0.2667	0.1667	-1	17	-0.3176	-2	2	1	0
HIP_S6227	ICSLYQLEN	ELEKVAHQL	ICSLYQLENELEKVAHQL		CMGA	23877/247050	0.956	S6227	0.799	-0.027211	-1.0667	0	-1	18	-0.1111	-2	1	0	0
HIP_S6228	VCGERGFF	LFQNRMLSK	VCGERGFFLFQNRMLSK		SCG2	20245/334890	0.996	S6228	0.799	0.030747	1.3833	0.1667	2	17	-0.0235	2	3	1	0
HIP_S6229	LYQLENYCN	EEEEDNRD	LYQLENYCNEEEEDNRD		CMGA	13005/247050	0.991	S6229	0.799	0.024668	-2.1333	0	-6	17	-2.0882	-6	1	1	2
HIP_S6230	AGSLQPLA	QYWDEDLLM	AGSLQPLAQYWDEDLLM		SCG2	19606/334890	0.996	S6230	0.799	-0.000807	-0.2833	0.1667	-3	17	-0.1059	-3	2	1	0
HIP_S6231	HLCGSHLVE	WNPVEEKIE	HLCGSHLVEWNPVEEKIE		SCG2	19176/334890	0.996	S6231	0.799	0.015591	-0.25	0.1667	-1	18	-0.4722	-3	1	0	0
HIP_S6232	CGERGFFYT	MESAANQK	CGERGFFYTMESAANQK		SCG2	25229/334890	0.994	S6232	0.799	0.066325	-0.2667	0.1667	0	17	-0.7294	0	3	1	0
HIP_S6233	HLVEALYL	EEPVSTHQ	HLVEALYLEEPVSTHQ		SCG1	17933/367830	0.992	S6233	0.799	0.172519	-0.3833	0.1667	-1	16	-0.2	-3	1	0	0
HIP_S6234	SHLVEALYL	DRVAQLDQL	SHLVEALYLDRVAQLDQL		SCG1	28688/367830	0.970	S6234	0.799	0.188056	0.4167	0	-1	18	0.2056	-2	1	0	0
HIP_S6235	CGERGFFYT	VFQELGKL	CGERGFFYTVFQELGKL		SCG2	19892/334890	0.996	S6235	0.799	-0.063500	0.7167	0	0	17	0.0353	0	4	1	0
HIP_S6236	GERGFFYTP	GVQAPEDLE	GERGFFYTPGVQAPEDLE		SCG1	4411/367830	0.999	S6236	0.799	-0.193551	-0.55	0.3333	-3	18	-0.7222	-3	3	0	0
HIP_S6237	CSLYQLENY	GELFNPYYD	CSLYQLENYGELFNPYYD		SCG1	15994/367830	0.994	S6237	0.799	-0.165344	-1.4	0.1667	-3	18	-0.6833	-3	2	0	0
HIP_S6238	PGAGSLQPL	DRNYLNYGE	PGAGSLQPLDRNYLNYGE		SCG1	33021/367830	0.953	S6238	0.799	0.048861	-2.1333	0.1667	-1	18	-0.9222	-1	1	0	0
HIP_S6239	TSICSLYQL	ETLRGDERI	TSICSLYQLETLRGDERI		CMGA	19767/247050	0.973	S6239	0.799	0.023601	-0.2333	0	-1	18	-0.2667	-1	1	0	0
HIP_S6240	SLYQLENYC	LNQGSSQET	SLYQLENYCLNQGSSQET		SCG2	16189/334890	0.997	S6240	0.799	-0.133700	-0.9167	0	-2	18	-0.9278	-2	1	0	0
HIP_S6241	LVEALYLV	HGYGEESEE	LVEALYLVHGYGEESEE		SCG1	16542/367830	0.993	S6241	0.799	0.050891	0.3	0.1667	-4	17	-0.1941	-5	2	1	0
HIP_S6242	LGGGPGAGS	DNPFKRTNE	LGGGPGAGSDNPFKRTNE		SCG2	15956/334890	0.997	S6242	0.799	-0.043474	-1.3333	0.5	0	18	-1.15	0	1	0	1
HIP_S6243	CSLYQLENY	HGRSRPDRS	CSLYQLENYHGRSRPDRS		SCG1	38367/367830	0.927	S6243	0.799	-0.000364	-2.7333	0.1667	2	18	-1.5333	1	1	0	0
HIP_S6244	GERGFFYT	DIQGPTKAD	GERGFFYTDIQGPTKAD		SCG1	25968/367830	0.978	S6244	0.799	-0.006655	-0.2833	0	-1	17	-0.9412	-1	3	1	0
HIP_S6245	AGSLQPLA	QWPERKLKH	AGSLQPLAQWPERKLKH		SCG2	28083/334890	0.992	S6245	0.799	0.106151	-0.3333	0.3333	3	17	-0.9588	2	1	1	0
HIP_S6246	CGERGFFY	SEAHESSS	CGERGFFYSEAHESSS		SCG1	22093/367830	0.986	S6246	0.799	0.142814	0.3	0.1667	-1	16	-0.85	-2	3	0	1
HIP_S6247	CCTSICSLY	YGEEGAPG	CCTSICSLYYGEEGAPG		SCG1	15119/367830	0.995	S6247	0.799	-0.012596	-0.5833	0.3333	-2	17	0.1706	-2	2	1	0

HIP_56711	CGERGFFY	TEVMKQVE	CGERGFFYTEVMKQVE		CMGA	16960/247050	0.982	56711	0.798	0.144910	0.7167	0	-1	17	0.2176	-1	3	1	0
HIP_56712	SLYQLENYC	EYLNQEKAE	SLYQLENYCEYLNQEKAE		SCG2	18010/334890	0.997	56712	0.798	-0.159835	-0.55	0	-3	18	-1.1611	-3	2	0	0
HIP_56713	TSICSLYQL	ENEKDRAD	TSICSLYQLENEKDRAD		SCG1	26303/367830	0.977	56713	0.798	0.130099	-1.9167	0	-2	17	-0.9765	-2	1	1	0
HIP_56714	EQCCTSICS	KRNLARVPK	EQCCTSICSKRNLARVPK		SCG1	42888/367830	0.902	56714	0.798	0.114313	-0.95	0.1667	3	18	-0.5222	3	0	0	0
HIP_56715	EDLQVGQVE	EDEKKELEN	EDLQVGQVEEDEKKELEN		SCG1	24527/367830	0.981	56715	0.798	-0.095311	-2.2167	0	-6	18	-1.7056	-6	0	0	0
HIP_56716	PLALEGSLQ	TENVPRTRL	PLALEGSLQTENVPRTRL		NPY	3837/49410	0.961	56716	0.798	-0.093669	-1.3667	0.1667	0	18	-0.4222	0	0	0	0
HIP_56717	LCGSHLVEA	ATNTHPPAS	LCGSHLVEAATNTHPPAS		CMGA	21977/247050	0.965	56717	0.798	0.074321	0.0167	0	1	18	-0.0167	-1	0	0	0
HIP_56718	ERGFFYTPK	LLGAVGLAA	ERGFFYTPKLLGAVGLAA		SCG1	16143/367830	0.994	56718	0.798	-0.041021	0.1667	0.3333	1	18	0.55	1	2	0	0
HIP_56719	LVCGERGFF	PFKRTNEI	LVCGERGFFPFKRTNEI		SCG2	26000/334890	0.993	56719	0.798	0.069487	0.4167	0.3333	1	17	-0.1824	1	3	1	0
HIP_56720	SLYQLENYC	SHQVEKRKC	SLYQLENYCSHQVEKRKC		IAPP	4369/45018	0.948	56720	0.798	0.008785	-1.6333	0.1667	2	18	-1.1333	1	1	0	0
HIP_56721	HLVEALYLV	SSREDSLE	HLVEALYLVSSREDSLE		CMGA	14555/247050	0.988	56721	0.798	0.218678	0.1	0.3333	-2	17	0	-3	1	1	0
HIP_56722	AGSLQPLAL	ALPEPMQES	AGSLQPLALALPEPMQES		CMGA	9238/247050	0.996	56722	0.798	0.034289	2.2333	0.1667	-2	18	0.0944	-2	0	0	0
HIP_56723	VEQCCTSIC	REDSKEAEK	VEQCCTSICREDSKEAEK		CMGA	16578/247050	0.983	56723	0.798	0.063987	-0.8833	0.1667	-2	18	-0.9778	-2	0	0	0
HIP_56724	VEALYLVCG	VKYPEIINS	VEALYLVCGVKYPEIINS		SCG2	19192/334890	0.996	56724	0.798	-0.008527	0.8833	0.1667	-1	18	0.7611	-1	2	0	0
HIP_56725	ALYLVCGER	REDSKEAEK	ALYLVCGERREDSKEAEK		CMGA	24693/247050	0.952	56725	0.798	-0.070321	-3.3167	0.1667	-1	18	-1.05	-1	0	0	0
HIP_56726	CCTSICSLY	SANRRPEDQ	CCTSICSLYSANRRPEDQ		CMGA	12876/247050	0.991	56726	0.798	-0.042225	-0.1333	0.3333	0	18	-0.6333	0	1	0	0
HIP_56727	QHLCGSHLV	KNDDTPNRQ	QHLCGSHLVKNDDTPNRQ		SCG2	29668/334890	0.990	56727	0.798	0.243408	-1.0167	0	2	18	-1.3889	0	0	0	0
HIP_56728	GSLQPLALE	QGGSLPSEE	GSLQPLALEQGGSLPSEE		SCG1	10423/367830	0.998	56728	0.798	0.154241	-0.3667	0.3333	-3	18	-0.4056	-3	0	0	0
HIP_56729	CCTSICSLY	RPQSEESWD	CCTSICSLYRPQSEESWD		SCG1	23980/367830	0.982	56729	0.798	0.058427	-1.3167	0.3333	-2	18	-0.5778	-2	1	0	0
HIP_56730	GERGFFYT	TPQSLATLE	GERGFFYTTPQSLATLE		SCG2	19015/334890	0.996	56730	0.798	0.095618	-0.8333	0.1667	-1	17	-0.3882	-1	3	1	0
HIP_56731	SHLVEALYL	GVSVPLQQ	SHLVEALYLGVSVPLQQ		SCG2	9726/334890	0.999	56731	0.798	0.178026	1.55	0.3333	0	17	0.6471	-1	1	1	0
HIP_56732	LCGSHLVEA	PQSLATLES	LCGSHLVEAPQSLATLES		SCG2	15763/334890	0.997	56732	0.798	0.163474	-0.5667	0.3333	-1	18	0.3722	-2	0	0	0
HIP_56733	IVEQCCTSI	SQKYPGPQA	IVEQCCTSISQKYPGPQA		CMGA	24220/247050	0.955	56733	0.798	0.089444	-0.8667	0.3333	0	18	-0.2833	0	1	0	0
HIP_56734	VEALYLVC	ARFQDKQYS	VEALYLVCARFQDKQYS		SCG1	34473/367830	0.947	56734	0.798	0.116183	1.7667	0	0	17	-0.0529	0	2	1	0
HIP_56735	LVEALYLVC	PYALNSEK	LVEALYLVCPYALNSEK		SCG2	19521/334890	0.996	56735	0.798	0.060409	1.5667	0.1667	-1	17	0.6059	-1	2	1	0
HIP_56736	SLYQLENYC	NHLKATPIE	SLYQLENYCNHLKATPIE		IAPP	2752/45018	0.963	56736	0.798	-0.098673	-0.8667	0	0	18	-0.5611	-1	1	0	0
HIP_56737	LYQLENYCN	PSDSQVSEE	LYQLENYCNPSDSQVSEE		SCG1	9590/367830	0.998	56737	0.798	0.067724	-1.3667	0.3333	-4	18	-1.1278	-4	1	0	0
HIP_56738	RGFFYTPKT	EEDATSEVD	RGFFYTPKTEEDATSEVD		SCG1	25799/367830	0.978	56738	0.798	-0.074479	-2.7833	0.1667	-3	18	-1.1389	-3	1	0	0
HIP_56739	QVELGGGPG	NPYNQEKVL	QVELGGGPGNPYNQEKVL		SCG2	6432/334890	0.999	56739	0.798	-0.006624	-1.4667	0.6667	-1	18	-0.8333	-1	1	0	1
HIP_56740	SLYQLENYC	EISRNLQIP	SLYQLENYCEISRNLQIP		SCG2	19583/334890	0.996	56740	0.798	-0.057347	-0.35	0.1667	-1	18	-0.4667	-1	1	0	0
HIP_56741	CTSICSLYQ	IPLIFLISG	CTSICSLYQIPLIFLISG		SCG2	13226/334890	0.998	56741	0.798	-0.100355	0.95	0.1667	0	18	1.5167	0	1	0	0
HIP_56742	GSHLVEALY	TDGARPQA	GSHLVEALYTDGARPQA		CMGA	10861/247050	0.994	56742	0.798	0.192042	-0.05	0.1667	0	17	-0.3647	-1	1	1	0
HIP_56743	VEALYLVC	LQNGQNGER	VEALYLVCLQNGQNGER		SCG2	23503/334890	0.994	56743	0.798	0.190359	1.2167	0	-1	17	-0.2059	-1	1	1	0
HIP_56744	GSLQPLAL	SGLTLAL	GSLQPLALSGLTLAL		NPY	1872/49410	0.988	56744	0.798	0.201363	2	0.3333	0	15	1.2133	0	0	1	0
HIP_56745	HLVEALYL	GEEAVPEEE	HLVEALYLGEEAVPEEE		CMGA	7552/247050	0.997	56745	0.798	0.135583	-0.1833	0.1667	-5	17	-0.2412	-6	1	1	1
HIP_56746	YLVCGERGF	GNNQAPGEE	YLVCGERGFGNNQAPGEE		CMGA	8445/247050	0.996	56746	0.798	-0.019600	-1.5833	0.3333	-2	18	-0.8278	-2	1	0	0
HIP_56747	GAGSLQPLA	KEEEEEMAV	GAGSLQPLAKEEEEEMAV		CMGA	10691/247050	0.994	56747	0.798	0.007633	-1.15	0.1667	-4	18	-0.5	-4	0	0	3
HIP_56748	SLQPLAL	EDVNWGYEK	SLQPLALEDVNWGYEK		SCG1	21683/367830	0.987	56748	0.798	0.124163	1.1	0	-2	16	-0.5625	-2	1	2	0
HIP_56749	PLALEGSLQ	RGWRPSSRE	PLALEGSLQRGWRPSSRE		CMGA	21267/247050	0.967	56749	0.798	-0.016548	-1.05	0.3333	1	18	-1.0056	1	1	0	0
HIP_56750	QHLCGSHLV	GQVTALPVN	QHLCGSHLVGQVTALPVN		CMGA	11560/247050	0.993	56750	0.798	0.185584	0.85	0.1667	2	18	0.4167	0	0	0	0
HIP_56751	ERGFFYTPK	HLEEPGETQ	ERGFFYTPKHLEEPGETQ		SCG1	19211/367830	0.991	56751	0.798	0.041005	-1.5167	0.1667	-1	18	-1.4667	-2	2	0	0
HIP_56752	LVEALYLV	REDSLEAGL	LVEALYLVREDSLEAGL		CMGA	14948/247050	0.987	56752	0.798	0.146630	-0.8	0	-3	17	0.5882	-3	1	1	0
HIP_56753	TSICSLYQL	KRWSKMDQL	TSICSLYQLKRWSKMDQL		CMGA	30909/247050	0.906	56753	0.798	0.131505	-1.7167	0	2	18	-0.4333	2	2	0	0
HIP_56754	HLVEALYLV	SPEMIRAL	HLVEALYLVSPEMIRAL		SCG2	15656/334890	0.998	56754	0.798	0.205042	0.1333	0.3333	0	17	0.8941	-1	1	1	0
HIP_56755	GGPGAGSLQ	SEESWDEED	GGPGAGSLQSEESWDEED		SCG1	13662/367830	0.996	56755	0.798	-0.121053	-1.3833	0.3333	-6	18	-1.4111	-6	0	0	0
HIP_56756	RGFFYTPK	TQNAFLNER	RGFFYTPKTQNAFLNER		SCG1	34444/367830	0.947	56756	0.798	-0.033977	-2.3167	0.1667	2	17	-1.0353	2	2	1	0
HIP_56757	AGSLQPLA	GPQLRRGWR	AGSLQPLAGPQLRRGWR		CMGA	13734/247050	0.989	56757	0.798	0.189587	-0.25	0.5	3	17	-0.6824	3	0	1	0
HIP_56758	NQHLCGSHL	EEKIESQTQ	NQHLCGSHLEEKIESQTQ		SCG2	29234/334890	0.991	56758	0.798	0.036249	-1.85	0.1667	0	18	-1.2722	-2	0	0	0
HIP_56759	SLYQLENYC	PLQWKSSH	SLYQLENYCPLQWKSSH		SCG1	20690/367830	0.988	56759	0.798	-0.032371	-0.6	0.1667	1	17	-0.8647	0	2	1	0
HIP_56760	QVELGGG	VELGGGP	QVELGGGVELGGGP		INSC	39/5184	1.000	56760	0.798	0.143711	0.55	0.5	-2	14	0.1071	-2	0	0	2
HIP_56761	GSLQPLALE	NSPMNKGDT	GSLQPLALENSPMNKGDT		CMGA	12350/247050	0.992	56761	0.798	0.148224	-0.6333	0.3333	-1	18	-0.7	-1	0	0	0
HIP_56762	QCCTSICSL	KLAPVSKRF	QCCTSICSLKLAPVSKRF		SCG2	35191/334890	0.985	56762	0.798	0.079060	1.2	0.1667	3	18	0.4389	3	0	0	0
HIP_56763	CGERGFFY	GSLPSEEKG	CGERGFFYGSLPSEEKG		SCG1	12342/367830	0.997	56763	0.798	0.161981	1.15	0.3333	-1	17	-0.7706	-1	3	1	0
HIP_56764	HLVEALYL	VSKRFPVGP	HLVEALYLVSKRFPVGP		SCG2	26172/334890	0.993	56764	0.798	0.173901	0.9667	0.1667	2	17	0.4588	1	2	1	0
HIP_56765	CGERGFFYT	DSEGLSQG	CGERGFFYTDSEGLSQG		CMGA	16914/247050	0.982	56765	0.798	0.130978	-1.1667	0.1667	-2	17	-0.6941	-2	3	1	0
HIP_56766	TSICSLYQL	GEEAVPEE	TSICSLYQLGEEAVPEE		CMGA	7991/247050	0.997	56766	0.798	0.114331	-1.4	0.1667	-4	17	-0.4171	-4	1	1	0

HIP_S7222	HLVEALYL	QSSQAELKE	HLVEALYLQSSQAELKE		CMGA	12260/247050	0.992	S7222	0.798	0.212600	0.2	0.3333	-1	17
HIP_S7223	LVEALYLV	TGPNNQKR	LVEALYLVTGPNNQKR		SCG2	15072/334890	0.998	S7223	0.798	0.219096	0.6667	0.3333	1	16
HIP_S7224	GGGPGAGSL	QGSSQETDK	GGGPGAGSLQGSSQETDK		SCG2	7557/334890	0.999	S7224	0.798	-0.026053	-0.35	0.6667	-1	18
HIP_S7225	YLVCGERGF	QEEDLRKES	YLVCGERGFQEEDLRKES		SCG2	22453/334890	0.995	S7225	0.798	-0.006713	-2.1	0.1667	-2	18
HIP_S7226	PLALEGSLQ	PYQGVSVPL	PLALEGSLQPYQGVSVPL		SCG2	17763/334890	0.997	S7226	0.798	-0.102610	-1.15	0.3333	-1	18
HIP_S7227	LVEALYLV	PDNPGEDAP	LVEALYLVPDNPGEDAP		NPY	1914/49410	0.988	S7227	0.798	0.159895	-0.3167	0.1667	-4	17
HIP_S7228	HLCGSHLVE	PERELDLPV	HLCGSHLVEPERELDLPV		SCG2	26677/334890	0.993	S7228	0.798	0.153504	-0.85	0.1667	-1	18
HIP_S7229	LQPLALEGS	PNGSVEPER	LQPLALEGSPNGSVEPER		SCG2	10838/334890	0.999	S7229	0.798	-0.122911	-1.7	0.6667	-2	18
HIP_S7230	VEALYLVC	QKEPDLRLE	VEALYLVCQKEPDLRLE		SCG2	26654/334890	0.993	S7230	0.798	0.223431	-0.0667	0	-2	17
HIP_S7231	VEALYLV	QGAKERA	VEALYLVQGAKERA		CMGA	11495/247050	0.993	S7231	0.798	0.202172	0.7667	0.1667	0	14
HIP_S7232	EDLQVGQVE	QYWDEDLLM	EDLQVGQVEQYWDEDLLM		SCG2	15559/334890	0.998	S7232	0.798	-0.136297	-1.4167	0	-6	18
HIP_S7233	DLQVGQVEL	LLMKVLEYL	DLQVGQVELLLMKVLEYL		SCG2	14596/334890	0.998	S7233	0.798	0.008610	2.3333	0	-2	18
HIP_S7234	CTSICSLY	YDPLQWKSS	CTSICSLYYDPLQWKSS		SCG1	26385/367830	0.977	S7234	0.798	0.054989	-0.7833	0.3333	0	17
HIP_S7235	QPLALEGSL	GSGRLQNGQ	QPLALEGSLGSGRLQNGQ		SCG2	4982/334890	1.000	S7235	0.798	-0.002273	0.1667	0.8333	0	18
HIP_S7236	HLCGSHLVE	IAKRAMENM	HLCGSHLVEIAKRAMENM		SCG2	30142/334890	0.990	S7236	0.798	0.003569	1.15	0	2	18
HIP_S7237	VEALYLVC	PEIINSNQV	VEALYLVCPEIINSNQV		SCG2	14265/334890	0.998	S7237	0.798	0.161160	1.65	0.1667	-2	17
HIP_S7238	VEALYLVC	TGEKPNGSV	VEALYLVCTGEKPNGSV		SCG2	17256/334890	0.997	S7238	0.798	0.225233	0.9833	0.1667	-1	17
HIP_S7239	LVCGERGFF	GLSVEDILN	LVCGERGFFGLSVEDILN		SCG2	892/334890	1.000	S7239	0.798	-0.027553	1.3	0.5	-2	18
HIP_S7240	EQCCTSICS	EDSSEEKHL	EQCCTSICSEDSSEEKHL		SCG1	14535/367830	0.995	S7240	0.798	0.055497	-0.2667	0.3333	-3	18
HIP_S7241	ERGFFYTP	REEARFQDK	ERGFFYTPREEARFQDK		SCG1	37119/367830	0.934	S7241	0.798	0.027424	-2.5167	0.1667	0	17
HIP_S7242	GPGAGSLQP	PEDLEWERY	GPGAGSLQPPEDLEWERY		SCG1	14302/367830	0.995	S7242	0.798	-0.094126	-1.65	0.3333	-3	18
HIP_S7243	HLVEALYLV	ELTLNEKN	HLVEALYLVELTLNEKN		SCG1	17451/367830	0.993	S7243	0.798	0.097978	1.05	0	-1	17
HIP_S7244	QHLCGSHLV	TDDEDDIYK	QHLCGSHLVTDDEDDIYK		SCG2	16536/334890	0.997	S7244	0.798	0.151080	-0.4833	0	-2	18
HIP_S7245	ERGFFYTPK	EGENYQKG	ERGFFYTPKEGENYQKG		SCG1	28731/367830	0.970	S7245	0.798	-0.070176	-2.2667	0.3333	0	17
HIP_S7246	GERGFFYTP	ISGAEAASF	GERGFFYTPISGAEAASF		SCG2	8396/334890	0.999	S7246	0.798	-0.166264	-0.05	0.5	-1	18
HIP_S7247	TSICSLYQL	LQGAKERAH	TSICSLYQLLQGAKERAH		CMGA	12435/247050	0.991	S7247	0.798	0.026105	-0.1833	0.1667	2	18
HIP_S7248	SLYQLENYC	EVKTRHSEK	SLYQLENYCEVKTRHSEK		SCG1	45496/367830	0.885	S7248	0.798	0.025025	-0.9167	0	1	18
HIP_S7249	CGERGFFY	LSVEDILNL	CGERGFFYLSVEDILNL		SCG2	14953/334890	0.998	S7249	0.798	-0.020726	1.9167	0.1667	-2	17
HIP_S7250	AEDLQVGQV	GSEEYRAPR	AEDLQVGQVGSEEYRAPR		SCG1	6722/367830	0.999	S7250	0.798	-0.020107	-0.7333	0.5	-2	18
HIP_S7251	GERGFFYT	YFPNPYNQE	GERGFFYTYFPNPYNQE		SCG2	16836/334890	0.997	S7251	0.798	-0.072925	0.1167	0.1667	-1	17
HIP_S7252	DLQVGQVEL	DVVGGEDWN	DLQVGQVELDVVGGEDWN		SCG2	16651/334890	0.997	S7252	0.798	-0.053977	1.5667	0	-5	18
HIP_S7253	TSICSLYQ	YRGRGSEEY	TSICSLYQYRGRGSEEY		SCG1	31842/367830	0.958	S7253	0.798	0.141096	-1.2	0.1667	0	17
HIP_S7254	VEALYLVC	EQEEERLS	VEALYLVCEQEEERLS		CMGA	12543/247050	0.991	S7254	0.798	0.129881	0	0	-4	16
HIP_S7255	GERGFFYT	FETLRGDER	GERGFFYTFETLRGDER		CMGA	18868/247050	0.976	S7255	0.798	0.023805	-0.1	0	-1	17
HIP_S7256	AGSLQPLAL	SVFQELGK	AGSLQPLALSVFQELGK		SCG2	20025/334890	0.996	S7256	0.798	0.085725	2.6	0.1667	0	17
HIP_S7257	CGERGFFY	MRIILEALR	CGERGFFYMRIILEALR		SCG2	28400/334890	0.991	S7257	0.798	0.073777	1.0333	0	1	17
HIP_S7258	GAGSLQPLA	EMIRALEYI	GAGSLQPLAEMIRALEYI		SCG2	14329/334890	0.998	S7258	0.798	-0.056580	1.15	0.1667	-1	18
HIP_S7259	GGGPGAGSL	EGENYQKGE	GGGPGAGSLEGENYQKGE		SCG1	12146/367830	0.997	S7259	0.798	-0.046100	-0.8	0.5	-2	18
HIP_S7260	HLCGSHLV	PGWQAKREE	HLCGSHLVPGWQAKREE		CMGA	10711/247050	0.994	S7260	0.798	0.193258	0.3167	0.3333	2	17
HIP_S7261	TSICSLYQ	NTYGKRNAV	TSICSLYQNTYGKRNAV		IAPP	5038/45018	0.925	S7261	0.798	0.120615	-1.0833	0	2	17
HIP_S7262	SLYQLENYC	DEEQKLYTD	SLYQLENYCDEEQKLYTD		SCG2	20209/334890	0.996	S7262	0.798	0.039431	-2.1333	0	-4	18
HIP_S7263	AGSLQPLAL	VQAPEDLE	AGSLQPLALVQAPEDLE		SCG1	17457/367830	0.993	S7263	0.798	0.012710	1.9833	0	-3	17
HIP_S7264	VNQHLCGSH	ENELTLNEK	VNQHLCGSHENELTLNEK		SCG1	25370/367830	0.979	S7264	0.798	-0.120854	-2.4833	0.3333	0	18
HIP_S7265	CGERGFFY	APGEEEEE	CGERGFFYAPGEEEEE		CMGA	3145/247050	0.999	S7265	0.798	0.097239	0.6833	0.3333	-5	16
HIP_S7266	PGAGSLQPL	EPEYGEEIK	PGAGSLQPLEPEYGEEIK		SCG1	17423/367830	0.993	S7266	0.798	-0.023080	-1.65	0.3333	-3	18
HIP_S7267	CGERGFFYT	EIKGYPGV	CGERGFFYTEIKGYPGV		SCG1	37186/367830	0.934	S7267	0.798	0.097291	-0.35	0	0	17
HIP_S7268	CGSHLVEAL	EEPVSTHQE	CGSHLVEALEEPVSTHQE		SCG1	17130/367830	0.993	S7268	0.798	0.045814	-1.0833	0.1667	-2	18
HIP_S7269	PLALEGSLQ	EDEKKELEN	PLALEGSLQEDEKKELEN		SCG1	22295/367830	0.986	S7269	0.798	-0.060147	-1.8333	0.1667	-4	18
HIP_S7270	GSLQPLALE	VPKLDLKRQ	GSLQPLALEVPKLDLKRQ		SCG1	29623/367830	0.967	S7270	0.798	-0.002312	0.1333	0.1667	1	18
HIP_S7271	CGERGFFYT	LDRNYLNY	CGERGFFYTLDRNYLNY		SCG1	36367/367830	0.938	S7271	0.798	0.074938	-0.5667	0	0	17
HIP_S7272	QHLCGSHLV	YTDDEDDIY	QHLCGSHLVYTDDEDDIY		SCG2	16073/334890	0.997	S7272	0.798	0.101286	-0.1167	0	-3	18
HIP_S7273	GSLQPLAL	SMASLGE	GSLQPLALSMASLGE		SCG1	20294/367830	0.989	S7273	0.798	0.083771	2.05	0.1667	-1	15
HIP_S7274	PGAGSLQPL	SRDNPFKRT	PGAGSLQPLSRDNPFKRT		SCG2	37428/334890	0.981	S7274	0.798	0.095722	-1.6833	0.3333	2	18
HIP_S7275	HLCGSHLV	NPFKRTNE	HLCGSHLVNPFKRTNE		SCG2	23840/334890	0.994	S7275	0.798	0.211243	0.4167	0.1667	3	16
HIP_S7276	VEQCCTSIC	PYGAGRSRS	VEQCCTSICPYGAGRSRS		SCG2	13217/334890	0.998	S7276	0.798	0.058786	0.4833	0.5	1	18
HIP_S7277	SHLVEALYL	VQAPEDLE	SHLVEALYLVQAPEDLE		SCG1	15749/367830	0.994	S7277	0.798	0.001781	1.4667	0	-3	17
HIP_S7278	GAGSLQPLA	EEQKLYTDD	GAGSLQPLAEEQKLYTDD		SCG2	15278/334890	0.998	S7278	0.798	-0.000698	-1.0833	0.1667	-3	18
HIP_S7279	EQCCTSICS	AEAASFQRN	EQCCTSICSAEAASFQRN		SCG2	17691/334890	0.997	S7279	0.798	-0.058761	1.05	0.1667	-1	18

HIP_S7733	PLALEGSLQ	KGQPRSQEE	PLALEGSLQKGQPRSQEE		SCG1	24607/367830	0.981	S7733	0.797	-0.005152	-1.3833	0.3333	-1	18	-1.2111	-1	0	0	0	[illegible]
HIP_S7734	CTSICSLYQ	EKSQREDEE	CTSICSLYQEKSQREDEE		SCG1	24463/367830	0.981	S7734	0.797	0.137029	-1.5333	0.1667	-3	18	-1.3333	-3	1	0	0	[illegible]
HIP_S7735	YQLENYCN	WKSSHFERR	YQLENYCNWKSSHFERR		SCG1	39514/367830	0.921	S7735	0.797	0.109656	-1.3167	0.1667	2	17	-1.7412	1	2	1	0	[illegible]
HIP_S7736	GAGSLQPLA	RALEYIENL	GAGSLQPLARALEYIENL		SCG2	17374/334890	0.997	S7736	0.797	-0.057397	0.85	0.1667	-1	18	0.1167	-1	0	0	0	[illegible]
HIP_S7737	HLVEALYL	ASEEEPEYG	HLVEALYLASEEEPEYG		SCG1	32020/367830	0.997	S7737	0.797	0.109157	0.6333	0.1667	-4	17	-0.4059	-5	1	1	1	[illegible]
HIP_S7738	CCTSICSLY	NLRQQAHKE	CCTSICSLYNLRQQAHKE		SCG2	33714/334890	0.986	S7738	0.797	0.005953	-0.4167	0.1667	2	18	-0.4333	1	1	0	0	[illegible]
HIP_S7739	SHLVEALYL	KRNYPSLE	SHLVEALYLKRNYPSLE		SCG1	37360/367830	0.933	S7739	0.797	0.209960	-0.9333	0	1	17	-0.3941	0	2	1	0	[illegible]
HIP_S7740	SLQPLALE	EEEEEEATN	SLQPLALEEEEEEEATN		CMGA	12358/247050	0.992	S7740	0.797	0.024697	-1.4	0	-7	17	-1.1529	-7	0	1	5	[illegible]
HIP_S7741	HLCGSHLVE	IAEKFSQRG	HLCGSHLVEIAEKFSQRG		SCG1	31792/367830	0.959	S7741	0.797	-0.001314	1.2167	0	2	18	-0.2389	0	0	0	0	[illegible]
HIP_S7742	RGFFYTPKT	SDSQVSEEV	RGFFYTPKTSDSQVSEEV		SCG1	24967/367830	0.980	S7742	0.797	-0.080542	-1.8833	0.5	-1	18	-0.8611	-1	1	0	0	[illegible]
HIP_S7743	QHLCGSHLV	QMAYENLN	QHLCGSHLVQMAYENLN		SCG2	26242/334890	0.993	S7743	0.797	0.072882	0.8333	0	1	17	-0.2706	-1	1	1	0	[illegible]
HIP_S7744	CGERGFFY	WMRIILEAL	CGERGFFYWMRIILEAL		SCG2	28757/334890	0.991	S7744	0.797	0.071110	0.1333	0	0	17	0.5529	0	4	1	0	[illegible]
HIP_S7745	GERGFFYTP	LSEEDWMRI	GERGFFYTPLSEEDWMRI		SCG2	13057/334890	0.998	S7745	0.797	-0.189182	-0.6833	0.3333	-2	18	-0.7389	-2	3	0	0	[illegible]
HIP_S7746	GGPGAGSLQ	QDTKENREE	GGPGAGSLQQDTKENREE		SCG1	25188/367830	0.980	S7746	0.797	-0.116527	-1.3667	0.1667	-2	18	-1.7778	-2	0	0	0	[illegible]
HIP_S7747	LQPLALEGS	KEESSPDYN	LQPLALEGSKEESSPDYN		SCG2	24842/334890	0.994	S7747	0.797	-0.092356	-2.6	0.3333	-3	18	-1.0556	-3	0	0	0	[illegible]
HIP_S7748	TSICSLYQL	EARFQDKQ	TSICSLYQLEARFQDKQ		SCG1	41313/367830	0.911	S7748	0.797	0.093402	-1.2	0	0	17	-0.6059	0	2	1	0	[illegible]
HIP_S7749	VEQCCTSIC	DLFQNRMLS	VEQCCTSICDLFQNRMLS		SCG2	17084/334890	0.997	S7749	0.797	-0.029132	1.55	0.1667	-1	18	0.2333	-1	1	0	0	[illegible]
HIP_S7750	AGSLQPLAL	AGSLQPLAL	AGSLQPLALAGSLQPLAL		INSC	188/5184	0.998	S7750	0.797	0.169108	1.6667	0.3333	0	18	0.9667	0	0	0	0	[illegible]
HIP_S7751	AGSLQPLAL	DTLSKPSP	AGSLQPLALDTLSKPSP		CMGA	16695/247050	0.983	S7751	0.797	0.174408	1.5	0	0	17	-0.0235	0	0	1	0	[illegible]
HIP_S7752	QPLALEGSL	PGWQAKREE	QPLALEGSLPGWQAKREE		CMGA	3524/247050	0.999	S7752	0.797	-0.099913	-0.05	0.6667	-1	18	-0.9222	-1	1	0	0	[illegible]
HIP_S7753	VEALYLVC	AYEDVVGGE	VEALYLVCAYEDVVGGE		SCG2	20321/334890	0.996	S7753	0.797	0.047087	1.25	0	-4	17	0.7706	-4	2	1	0	[illegible]
HIP_S7754	VNQHLCGSH	GQEEEEDNR	VNQHLCGSHGQEEEEDNR		CMGA	9562/247050	0.995	S7754	0.797	-0.125866	-1.9667	0.5	-2	18	-1.8611	-4	0	0	2	[illegible]
HIP_S7755	LVCGERGFF	SLGYKEIR	LVCGERGFFSLGYKEIR		CMGA	9120/247050	0.996	S7755	0.797	0.058029	1.3	0.5	1	17	0.0706	1	3	1	0	[illegible]
HIP_S7756	GAGSLQPLA	YGEEIKGYP	GAGSLQPLAYGEEIKGYP		SCG1	10803/367830	0.997	S7756	0.797	0.004797	-0.2	0.3333	-1	18	-0.3833	-1	1	0	0	[illegible]
HIP_S7757	VEQCCTSIC	YPEEKKEEE	VEQCCTSICYPEEKKEEE		CMGA	8741/247050	0.996	S7757	0.797	-0.033056	-0.0333	0.3333	-4	18	-1.1389	-4	1	0	1	[illegible]
HIP_S7758	AGSLQPLAL	EEQIEQAI	AGSLQPLALEEQIEQAI		SCG2	16517/334890	0.997	S7758	0.797	0.128843	-0.1833	0	-3	17	0.1176	-3	0	1	0	[illegible]
HIP_S7759	HLVEALYLV	ASAIKKEE	HLVEALYLVASAIKKEE		SCG1	19363/367830	0.990	S7759	0.797	0.024664	1.5833	0.1667	0	17	0.3588	-1	1	1	0	[illegible]
HIP_S7760	TSICSLYQL	EEQYTPQS	TSICSLYQLEEQYTPQS		SCG2	25509/334890	0.993	S7760	0.797	0.121845	-1.9167	0	-2	17	-0.6412	-2	2	1	0	[illegible]
HIP_S7761	VEALYLVC	LEQEEERLS	VEALYLVCLEQEEERLS		CMGA	12652/247050	0.991	S7761	0.797	0.125271	1.2167	0	-4	17	0.0176	-4	1	1	1	[illegible]
HIP_S7762	SICSLYQL	AASFQRNQ	SICSLYQLAASFQRNQ		SCG2	21681/334890	0.995	S7762	0.797	-0.049843	0.3	0.1667	1	16	-0.075	1	2	0	0	[illegible]
HIP_S7763	LCGSHLVEA	LLLSLLGA	LCGSHLVEALLLSLLGA		SCG1	18026/367830	0.992	S7763	0.797	0.031144	2.3167	0	0	17	1.6353	-1	0	1	1	[illegible]
HIP_S7764	EDLQVGQVE	GEESEEERG	EDLQVGQVEGEESEEERG		SCG1	14144/367830	0.995	S7764	0.797	-0.102496	-1.7	0.1667	-7	18	-1.6278	-7	0	0	1	[illegible]
HIP_S7765	LVEALYLVC	YPGPQAEG	LVEALYLVCYPGPQAEG		CMGA	11195/247050	0.993	S7765	0.797	0.156107	1.2	0.3333	-2	17	0.5176	-2	2	1	0	[illegible]
HIP_S7766	SHLVEALY	QGVSVPLQQ	SHLVEALYQGVSVPLQQ		SCG2	11963/334890	0.998	S7766	0.797	0.174158	0.7667	0.1667	0	17	0.2176	-1	1	1	0	[illegible]
HIP_S7767	AEDLQVGQV	SFQRNQLLQ	AEDLQVGQVSFQRNQLLQ		SCG2	9700/334890	0.999	S7767	0.797	-0.068924	-0.2	0.3333	-1	18	-0.5167	-1	1	0	0	[illegible]
HIP_S7768	HLVEALYL	SALRHYINL	HLVEALYLSALRHYINL		NPY	3066/49410	0.975	S7768	0.797	0.056361	1.85	0.1667	2	17	0.5882	0	1	1	0	[illegible]
HIP_S7769	QHLCGSHLV	HHGRSRPDR	QHLCGSHLVHHGRSRPDR		SCG1	39377/367830	0.922	S7769	0.797	0.159717	-0.3333	0.1667	6	18	-1.2778	2	0	0	0	[illegible]
HIP_S7770	ERGFFYTPK	RMDEEQKLY	ERGFFYTPKRMDEEQKLY		SCG2	29219/334890	0.991	S7770	0.797	-0.010109	-2.05	0.1667	0	18	-1.5722	0	2	0	0	[illegible]
HIP_S7771	QCCTSICSL	EESSPDYNP	QCCTSICSLEESSPDYNP		SCG2	15202/334890	0.998	S7771	0.797	0.080697	-0.3833	0.3333	-3	18	-0.5611	-3	0	0	0	[illegible]
HIP_S7772	LGGGPGAGS	YPEEKKEEE	LGGGPGAGSYPEEKKEEE		CMGA	6454/247050	0.998	S7772	0.797	-0.087295	-0.9667	0.5	-3	18	-1.5	-3	1	0	2	[illegible]
HIP_S7773	LVCGERGFF	ERADEPQW	LVCGERGFFERADEPQW		SCG1	28881/367830	0.969	S7773	0.797	0.046873	-0.1667	0.1667	-2	17	-0.7	-2	2	1	0	[illegible]
HIP_S7774	LVCGERGFF	PDGLSVED	LVCGERGFFPDGLSVED		SCG2	8891/334890	0.999	S7774	0.797	0.083748	-0.05	0.5	-3	17	0.1176	-3	2	1	0	[illegible]
HIP_S7775	SICSLYQLE	LLQKEPDLR	SICSLYQLELLQKEPDLR		SCG2	15560/334890	0.998	S7775	0.797	-0.094396	0.15	0	-1	18	-0.2444	-1	1	0	0	[illegible]
HIP_S7776	VCGERGFF	GGKSGELE	VCGERGFFGGKSGELE		CMGA	11985/247050	0.992	S7776	0.797	0.158423	0.0833	0.5	-1	16	-0.35	-1	2	0	0	[illegible]
HIP_S7777	HLVEALYLV	ERAHQQKK	HLVEALYLVERAHQQKK		CMGA	26083/247050	0.944	S7777	0.797	0.208384	0.0833	0	3	17	-0.6235	1	1	1	0	[illegible]
HIP_S7778	PLALEGSLQ	GPPKNDDTP	PLALEGSLQGPPKNDDTP		SCG2	2592/334890	1.000	S7778	0.797	-0.026985	-0.6833	0.6667	-2	18	-0.9389	-2	0	0	0	[illegible]
HIP_S7779	YLVCGERGF	NAVEVLKRE	YLVCGERGFNAVEVLKRE		IAPP	1726/45018	0.993	S7779	0.797	0.070203	0.0667	0.1667	0	18	-0.0944	0	1	0	0	[illegible]
HIP_S7780	HLVEALYL	EEVKTRH	HLVEALYLEEVKTRH		SCG1	29112/367830	0.969	S7780	0.797	0.167084	0.5833	0	1	15	-0.38	-1	1	1	0	[illegible]
[illegible]																				

HIP_58244	LVEALYLVC	ATQRLANF	LVEALYLVCATQRLANF		IAPP	3031/45018	0.978	58244	0.797
HIP_58245	FVNQHLCGS	QECFETLRG	FVNQHLCGSQECFETLRG		CMGA	10741/247050	0.994	58245	0.797
HIP_58246	VELGGGPGA	TSYFPNPYN	VELGGGPGATSYFPNPYN		SCG2	6263/334890	0.999	58246	0.797
HIP_58247	ALYLVCGER	ENTKFEVRL	ALYLVCGERENTKFEVRL		SCG1	32878/367830	0.954	58247	0.797
HIP_58248	AGSLQPLAL	ECFETLRG	AGSLQPLALECFETLRG		CMGA	17243/247050	0.981	58248	0.797
HIP_58249	LVEALYLVC	LPYGAGRS	LVEALYLVCLPYGAGRS		SCG2	20219/334890	0.996	58249	0.797
HIP_58250	GSHLVEALY	SNTYGKRN	GSHLVEALYSNTYGKRN		IAPP	3151/45018	0.975	58250	0.797
HIP_58251	YLVCGERGF	KEPDLRLEN	YLVCGERGFKEPDLRLEN		SCG2	23309/334890	0.995	58251	0.797
HIP_58252	LVCGERGFF	EQINDEMK	LVCGERGFFEQINDEMK		SCG2	18365/334890	0.997	58252	0.797
HIP_58253	GERGFFYTP	RILEALRQ	GERGFFYTPRILEALRQ		SCG2	16168/334890	0.997	58253	0.797
HIP_58254	HLCGSHLVE	ARFQDKQYS	HLCGSHLVEARFQDKQYS		SCG1	38526/367830	0.926	58254	0.797
HIP_58255	HLVEALYLV	ESNVSMAS	HLVEALYLVESNVSMAS		SCG1	18761/367830	0.991	58255	0.797
HIP_58256	SICSLYQLE	AVEVLKREP	SICSLYQLEAVEVLKREP		IAPP	2654/45018	0.983	58256	0.797
HIP_58257	CSLYQLENY	TLSKPSPMP	CSLYQLENYTLSKPSPMP		CMGA	15945/247050	0.985	58257	0.797
HIP_58258	CGERGFFYT	HQEAENEK	CGERGFFYTHQEAENEK		SCG1	32539/367830	0.955	58258	0.797
HIP_58259	QCCTSICSL	QGDLQDTKE	QCCTSICSLQGDLQDTKE		SCG1	10173/367830	0.998	58259	0.797
HIP_58260	GSLQPLAL	PEYGEEIK	GSLQPLALPEYGEEIK		SCG1	20010/367830	0.989	58260	0.797
HIP_58261	SHLVEALYL	ETGSQENH	SHLVEALYLETGSQENH		SCG1	17733/367830	0.992	58261	0.797
HIP_58262	HLVEALYL	SEEEPEYG	HLVEALYLSEEEPEYG		SCG1	17826/367830	0.992	58262	0.797
HIP_58263	VNQHLCGSH	HYINLITRQ	VNQHLCGSHHYINLITRQ		NPY	2382/49410	0.983	58263	0.797
HIP_58264	VEALYLVCG	NGSVEPERE	VEALYLVCGNGSVEPERE		SCG2	4940/334890	1.000	58264	0.797
HIP_58265	AGSLQPLAL	RATRLFEK	AGSLQPLALRATRLFEK		SCG2	36023/334890	0.984	58265	0.797
HIP_58266	PGAGSLQPL	PVSKRFPVG	PGAGSLQPLPVSKRFPVG		SCG2	20712/334890	0.996	58266	0.797
HIP_58267	CGERGFFYT	DVKDKETT	CGERGFFYTDVKDKETT		SCG1	35448/367830	0.943	58267	0.797
HIP_58268	CGERGFFY	PERELDLPV	CGERGFFYPERELDLPV		SCG2	23400/334890	0.995	58268	0.797
HIP_58269	LVCGERGFF	QAPEDLEWE	LVCGERGFFQAPEDLEWE		SCG1	12412/367830	0.997	58269	0.797
HIP_58270	ERGFFYTPK	VGGEDWNPV	ERGFFYTPKVGGEDWNPV		SCG2	12102/334890	0.998	58270	0.797
HIP_58271	HLVEALYL	HMQFPPMYE	HLVEALYLHMQFPPMYE		SCG2	26587/334890	0.993	58271	0.797
HIP_58272	LVCGERGFF	GGSLPSEE	LVCGERGFFGGSLPSEE		SCG1	6113/367830	0.999	58272	0.797
HIP_58273	HLCGSHLVE	VNAAGSGRL	HLCGSHLVEVNAAGSGRL		SCG2	24809/334890	0.994	58273	0.797
HIP_58274	GERGFFYT	GESRSEAL	GERGFFYTGESRSEAL		CMGA	12739/247050	0.991	58274	0.797
HIP_58275	VELGGGPGA	SEEEPEYGE	VELGGGPGASEEEPEYGE		SCG1	7074/367830	0.999	58275	0.797
HIP_58276	LVCGERGFF	EEMAVVPQ	LVCGERGFFEEMAVVPQ		CMGA	14604/247050	0.988	58276	0.797
HIP_58277	CTSICSLYQ	PSLGYKEIR	CTSICSLYQPSLGYKEIR		CMGA	11813/247050	0.992	58277	0.797
HIP_58278	VEALYLVCG	QESKGQPR	VEALYLVCGQESKGQPR		SCG1	22287/367830	0.986	58278	0.797
HIP_58279	VEALYLVCG	ELQDLALQG	VEALYLVCGELQDLALQG		CMGA	8209/247050	0.996	58279	0.797
HIP_58280	CCTSICSLY	GGHSRERAD	CCTSICSLYGGHSRERAD		SCG1	12519/367830	0.997	58280	0.797
HIP_58281	LCGSHLVEA	PDNPGEDAP	LCGSHLVEAPDNPGEDAP		NPY	2235/49410	0.985	58281	0.797
HIP_58282	ELGGGPGAG	NPGEDAPAE	ELGGGPGAGNPGEDAPAE		NPY	303/49410	0.999	58282	0.797
HIP_58283	VNQHLCGSH	EEDIQGPTK	VNQHLCGSHEEDIQGPTK		SCG1	26954/367830	0.975	58283	0.797
HIP_58284	CCTSICSLY	PGEDAPAED	CCTSICSLYPGEDAPAED		NPY	488/49410	0.998	58284	0.797
HIP_58285	LVEALYLVC	NKPYALNS	LVEALYLVCNKPYALNS		SCG2	25577/334890	0.993	58285	0.797
HIP_58286	GSHLVEALY	GPQLRRGW	GSHLVEALYGPQLRRGW		CMGA	12351/247050	0.992	58286	0.797
HIP_58287	TSICSLYQ	GEQEHSQQ	TSICSLYQGEQEHSQQ		CMGA	11605/247050	0.993	58287	0.797
HIP_58288	ELGGGPGAG	GQEEEEDNR	ELGGGPGAGGQEEEEDNR		CMGA	2858/247050	0.999	58288	0.797
HIP_58289	LVEALYLV	FPNPYNQE	LVEALYLVFPNPYNQE		SCG2	13737/334890	0.998	58289	0.797
HIP_58290	LVEALYLV	SSEEKHLEE	LVEALYLVSSEEKHLEE		SCG1	15360/367830	0.995	58290	0.797
HIP_58291	VELGGGPGA	GAGRSRSNQ	VELGGGPGAGAGRSRSNQ		SCG2	8556/334890	0.999	58291	0.797

HIP_58755	QHLCGSHLV	PVSTHQEAE	QHLCGSHLVPVSTHQEAE		SCG1	12672/367830	0.996	58755	0.796	0.149643	1.1	0.3333	1	18	-0.4222	-2	0	0	0
HIP_58756	VEALYLVCG	RLQNGQNGE	VEALYLVCGRLQNGQNGE		SCG2	25401/334890	0.993	58756	0.796	0.025986	0.35	0.1667	-1	18	-0.2167	-1	1	0	0
HIP_58757	GSLQPLALE	EGEEEDATSE	GSLQPLALEEGEEEDATSE		SCG1	12025/367830	0.997	58757	0.796	0.078043	-0.8833	0.1667	-6	18	-0.7889	-6	0	0	0
HIP_58758	TSICSLYQ	EGNNQAPGE	TSICSLYQEGNNQAPGE		CMGA	10990/247050	0.994	58758	0.796	0.167775	-1.4	0.1667	-2	17	-0.8471	-2	1	1	0
HIP_58759	ICSLYQLEN	EEEEEEAE	ICSLYQLENEEEEEEAE		CMGA	14069/247050	0.989	58759	0.796	-0.030701	-2.2833	0	-8	17	-1.2176	-8	1	1	4
HIP_58760	VGQVELGGG	SGYPKTPGR	VGQVELGGGSGYPKTPGR		SCG2	8036/334890	0.999	58760	0.796	0.006714	-0.6167	0.8333	1	18	-0.6444	1	1	0	1
HIP_58761	CGSHLVEAL	RALEYIENL	CGSHLVEALRALEYIENL		SCG2	25982/334890	0.993	58761	0.796	-0.030491	0.5333	0	-1	18	0.3222	-2	0	0	0
HIP_58762	NQHLCGSHL	EEQKLYTDD	NQHLCGSHLEEQKLYTDD		SCG2	23385/334890	0.995	58762	0.796	0.047579	-1.7833	0.1667	-1	18	-1.3389	-3	0	0	0
HIP_58763	RGFFYTPKT	FLIVLSVAL	RGFFYTPKTFLIVLSVAL		IAPP	2216/45018	0.989	58763	0.796	0.098418	0.8167	0.1667	2	18	1.1444	2	2	0	0
HIP_58764	ELGGGPGAG	EENSRDNPF	ELGGGPGAGEENSRDNPF		SCG2	11644/334890	0.998	58764	0.796	-0.103580	-1.5833	0.3333	-3	18	-1.2833	-3	0	0	1
HIP_58765	GSLQPLAL	WNPVEEKI	GSLQPLALWNPVEEKI		SCG2	17745/334890	0.997	58765	0.796	0.167892	0.5667	0.1667	-1	16	-0.0813	-1	1	0	0
HIP_58766	EQCCTSICS	ERGLEPGKG	EQCCTSICSERGLEPGKG		SCG1	19482/367830	0.990	58766	0.796	0.149200	-0.3667	0.3333	-1	18	-0.65	-1	0	0	0
HIP_58767	VCGERGFF	EAEAGEEAV	VCGERGFFEAEAGEEAV		CMGA	12489/247050	0.991	58767	0.796	-0.012775	0	0.1667	-4	17	-0.0765	-4	2	1	0
HIP_58768	GGPGAGSLQ	RQQAHKEES	GGPGAGSLQRQQAHKEES		SCG2	37913/334890	0.981	58768	0.796	-0.037329	-2	0.1667	1	18	-1.4722	0	0	0	0
HIP_58769	SICSLYQL	SEEVKTRHS	SICSLYQLSEEVKTRHS		SCG1	28354/367830	0.971	58769	0.796	0.116675	-1.4667	0.1667	1	17	-0.5	0	1	1	0
HIP_58770	CGERGFFY	KVLEYLNQE	CGERGFFYKVLEYLNQE		SCG2	22063/334890	0.995	58770	0.796	0.008773	1.4	0	-1	17	-0.5529	-1	4	1	0
HIP_58771	VEALYLVCG	PAEDMARYY	VEALYLVCGPAEDMARYY		NPY	1230/49410	0.993	58771	0.796	-0.024028	0.5	0.3333	-2	18	0.2722	-2	1	0	0
HIP_58772	SLQPLAL	GLAAVNS	SLQPLALGLAAVNS		SCG1	15734/367830	0.994	58772	0.796	0.084808	2.4333	0.1667	0	14	1.0143	0	0	0	0
HIP_58773	QHLCGSHLV	EEEEEEEA	QHLCGSHLVEEEEEEEA		CMGA	10744/247050	0.994	58773	0.796	0.091535	-0.95	0	-5	17	-1.1471	-7	0	1	5
HIP_58774	QHLCGSHLV	EEEEEEEA	QHLCGSHLVEEEEEEEA		CMGA	10745/247050	0.994	58774	0.796	0.091535	-0.95	0	-5	17	-1.1471	-7	0	1	5
HIP_58775	ERGFFYTPK	RMLSKSGYP	ERGFFYTPKRMLSKSGYP		SCG2	22681/334890	0.995	58775	0.796	0.084321	-0.8333	0.1667	3	18	-0.9944	3	2	0	0
HIP_58776	IVEQCCTSI	EGDSEGLSQ	IVEQCCTSIEGDSEGLSQ		CMGA	5981/247050	0.998	58776	0.796	-0.034779	-0.7333	0.3333	-4	18	-0.1611	-4	0	0	0
HIP_58777	TSICSLYQL	RHQNLLKEL	TSICSLYQLRHQNLLKEL		CMGA	33615/247050	0.878	58777	0.796	0.144637	-2.0333	0	2	18	-0.1778	1	1	0	0
HIP_58778	GERGFFYT	KEEEEEMA	GERGFFYTKEEEEEMA		CMGA	18241/247050	0.978	58778	0.796	0.105414	-1.6833	0	-4	16	-1.4313	-4	3	0	3
HIP_58779	LVCGERGF	YNYDWWEKK	LVCGERGFYNYDWWEKK		SCG1	23885/367830	0.983	58779	0.796	0.041970	-1.3667	0.1667	0	17	-1.0706	0	4	1	0
HIP_58780	AEDLQVGQV	QDPEGKGEQ	AEDLQVGQVQDPEGKGEQ		CMGA	5997/247050	0.998	58780	0.796	-0.090867	-1.3833	0.3333	-4	18	-1.3444	-4	0	0	0
HIP_58781	CGERGFFY	ESRSEALAV	CGERGFFYESRSEALAV		CMGA	19652/247050	0.973	58781	0.796	0.218342	-0.75	0.1667	-1	17	-0.2059	-1	3	1	0
HIP_58782	GERGFFYTP	QDPEGKGE	GERGFFYTPQDPEGKGE		CMGA	15751/247050	0.985	58782	0.796	-0.020940	-2.0333	0.3333	-2	17	-1.5941	-2	3	1	0
HIP_58783	CCTSICSLY	RLQNGQNGE	CCTSICSLYRLQNGQNGE		SCG2	31224/334890	0.989	58783	0.796	-0.024561	-0.4167	0.1667	0	18	-0.3778	0	1	0	0
HIP_58784	GGPGAGSLQ	QELGKLTGP	GGPGAGSLQQELGKLTGP		SCG2	27527/334890	0.992	58784	0.796	-0.108883	-0.6167	0.1667	0	18	-0.4611	0	0	0	0
HIP_58785	PLALEGSLQ	QRNQLLQKE	PLALEGSLQQRNQLLQKE		SCG2	39784/334890	0.977	58785	0.796	-0.001926	-2	0.1667	0	18	-0.8278	0	0	0	0
HIP_58786	HLCGSHLVE	MDEEQKLYT	HLCGSHLVEMDEEQKLYT		SCG2	25718/334890	0.993	58786	0.796	0.002268	-0.3	0	-1	18	-0.6111	-3	0	0	0
HIP_58787	GAGSLQPLA	SRGEAGAPG	GAGSLQPLASRGEAGAPG		SCG1	16055/367830	0.994	58787	0.796	0.143314	-0.2833	0.5	0	18	-0.1944	0	0	0	0
HIP_58788	GERGFFYTP	IEAELEKVA	GERGFFYTPIEAELEKVA		CMGA	9796/247050	0.995	58788	0.796	-0.100344	-0.1333	0.1667	-2	18	-0.2833	-2	3	0	0
HIP_58789	QVGQVELGG	NPFKRTNEI	QVGQVELGGNPFKRTNEI		SCG2	6445/334890	0.999	58789	0.796	-0.068975	0.1167	0.5	0	18	-0.7444	0	1	0	0
HIP_58790	TSICSLYQ	SNQVKRVPG	TSICSLYQSNQVKRVPG		SCG2	32212/334890	0.988	58790	0.796	0.233901	-1.4667	0.1667	2	17	-0.3588	2	1	1	0
HIP_58791	HLVEALYL	ENQMDKAR	HLVEALYLENQMDKAR		SCG1	26143/367830	0.977	58791	0.796	0.218616	-0.7	0	0	16	-0.5813	-1	1	0	0
HIP_58792	ERGFFYTPK	EDLIEMLKT	ERGFFYTPKEDLIEMLKT		SCG2	24120/334890	0.994	58792	0.796	-0.084157	-1.5667	0.1667	-1	18	-0.6333	-1	2	0	0
HIP_58793	GAGSLQPLA	GGEPRAYFM	GAGSLQPLAGGEPRAYFM		SCG1	7446/367830	0.999	58793	0.796	0.134311	-0.05	0.5	0	18	-0.0389	0	0	0	0
HIP_58794	TSICSLYQL	EGSANRRP	TSICSLYQLEGSANRRP		CMGA	14260/247050	0.988	58794	0.796	0.204427	-0.95	0.3333	1	17	-0.5588	1	1	1	0
HIP_58795	ERGFFYTPK	VPEEEGPTV	ERGFFYTPKVPEEEGPTV		CMGA	11980/247050	0.992	58795	0.796	-0.132416	-1.1833	0.3333	-2	18	-0.9278	-2	2	0	1
HIP_58796	ALYLVCGER	KEEEEEMAV	ALYLVCGERKEEEEEMAV		CMGA	17993/247050	0.979	58796	0.796	-0.093405	-3.2167	0.1667	-4	18	-0.3944	-4	0	0	3
HIP_58797	SHLVEALYL	NVSMASLGE	SHLVEALYLNVSMASLGE		SCG1	16195/367830	0.994	58797	0.796	0.177699	1.0333	0.1667	-1	18	0.6278	-2	1	0	0

HIP_S9266	AEDLQVGQV	NNQAPGEEE	AEDLQVGQVNNQAPGEEE		CMGA	7382/247050	0.997	S9266	0.796	-0.121192	-1.7	0.1667	-5	18	-1.2	-5	0	0
HIP_S9267	SLQPLAL	AEFPDFY	SLQPLALAEFPDFY		SCG1	21543/367830	0.987	S9267	0.796	0.057172	1.75	0	-2	14	0.3429	-2	1	0
HIP_S9268	GGPGAGSLQ	VFQELGKLT	GGPGAGSLQVFQELGKLT		SCG2	13115/334890	0.998	S9268	0.796	-0.046701	0.5	0.1667	0	18	0.0389	0	1	0
HIP_S9269	QVGQVELGG	EEEEAEAGE	QVGQVELGGEEEEAEAGE		CMGA	7930/247050	0.997	S9269	0.796	-0.106635	-1.25	0.3333	-7	18	-0.9611	-7	0	0
HIP_S9270	VEQCCTSIC	EGHHRVQEN	VEQCCTSICEGHHRVQEN		SCG1	14415/367830	0.995	S9270	0.796	0.000240	-0.15	0.3333	0	18	-0.7444	-2	0	0
HIP_S9271	LGGGPGAGS	EWERYRGRG	LGGGPGAGSEWERYRGRG		SCG1	26040/367830	0.978	S9271	0.796	0.010815	-1.2167	0.3333	1	18	-1.2389	1	1	0
HIP_S9272	SHLVEALY	FLVLSVA	SHLVEALYFLVLSVA		IAPP	2387/45018	0.987	S9272	0.796	-0.041474	2.5667	0	0	16	1.8187	-1	2	0
HIP_S9273	GPGAGSLQP	LANFLVHSS	GPGAGSLQPLANFLVHSS		IAPP	1319/45018	0.995	S9273	0.796	-0.043329	0.1333	0.1667	1	18	0.2778	0	1	0
HIP_S9274	LCGSHLVE	EGENYQKGE	LCGSHLVEEGENYQKGE		SCG1	22510/367830	0.985	S9274	0.796	0.183907	-0.4833	0.1667	-2	17	-1.0059	-3	1	1
HIP_S9275	GSLQPLAL	GNNQAPGEE	GSLQPLALGNNQAPGEE		CMGA	11008/247050	0.994	S9275	0.796	0.205176	0.3333	0.1667	-2	17	-0.6588	-2	0	1
HIP_S9276	RGFFYTPKT	HPPASLPSQ	RGFFYTPKTHPPASLPSQ		CMGA	19789/247050	0.973	S9276	0.796	-0.039588	-2.1	0.5	3	18	-0.8333	2	1	0
HIP_S9277	YLVCGERGF	SQENHPQES	YLVCGERGFSQENHPQES		SCG1	21925/367830	0.986	S9277	0.796	-0.013923	-1.65	0.3333	-1	18	-1.15	-2	1	0
HIP_S9278	VEALYLVCG	NPYYDPLQ	VEALYLVCGNPYYDPLQ		SCG1	21608/367830	0.987	S9278	0.796	0.026349	-0.0167	0.3333	-2	17	0.1529	-2	3	1
HIP_S9279	ERGFFYTP	EEPSSKDVM	ERGFFYTPEEPSSKDVM		CMGA	15330/247050	0.986	S9279	0.796	-0.040430	-2.0333	0.3333	-2	17	-1.0529	-2	2	1
HIP_S9280	VNQHLCGSH	TYGKRNAVE	VNQHLCGSHTYGKRNAVE		IAPP	2795/45018	0.981	S9280	0.796	-0.075947	-1.1333	0.5	3	18	-0.8833	1	1	0
HIP_S9281	LYQLENYCN	QPTLLLSLL	LYQLENYCNQPTLLLSLL		SCG1	15502/367830	0.994	S9281	0.796	0.047465	-1.35	0.1667	-1	18	0.3278	-1	1	0
HIP_S9282	EQCCTSICS	EEQKLYTDD	EQCCTSICSEEQKLYTDD		SCG2	17039/334890	0.997	S9282	0.796	0.027545	-0.7167	0.1667	-4	18	-0.9389	-4	0	0
HIP_S9283	LVEALYLV	KQYSSHHTA	LVEALYLVKQYSSHHTA		SCG1	36131/367830	0.939	S9283	0.796	0.145937	-0.3333	0	2	17	0.0706	0	2	1
HIP_S9284	LVCGERGFF	RCIEVLSN	LVCGERGFFRCIEVLSN		SCG1	18783/367830	0.991	S9284	0.796	-0.011947	1.2833	0.1667	0	18	0.8056	0	2	0
HIP_S9285	GSLQPLAL	EPSSKDVME	GSLQPLALEPSSKDVME		CMGA	11059/247050	0.993	S9285	0.796	0.203847	0.5833	0.3333	-2	17	-0.2706	-2	0	1
HIP_S9286	GERGFFYTP	NGQNGERA	GERGFFYTPNGQNGERA		SCG2	19927/334890	0.996	S9286	0.796	-0.004970	-1.8333	0.3333	0	17	-1.4294	0	3	1
HIP_S9287	ICSLYQLEN	GETQNAFLN	ICSLYQLENGETQNAFLN		SCG1	16926/367830	0.993	S9287	0.796	0.019502	-1.3	0.1667	-2	18	-0.0611	-2	1	0
HIP_S9288	VNQHLCGSH	ESKAEGNNQ	VNQHLCGSHESKAEGNNQ		CMGA	20856/247050	0.969	S9288	0.796	-0.103293	-2.1	0.5	1	18	-1.3833	-1	0	0
HIP_S9289	SICSLYQLE	EALPDGLSV	SICSLYQLEEALPDGLSV		SCG2	16015/334890	0.997	S9289	0.796	-0.102843	-0.1833	0	-3	18	0.4722	-3	1	0
HIP_S9290	QHLCGSHLV	GLVDREKGL	QHLCGSHLVGLVDREKGL		CMGA	12617/247050	0.991	S9290	0.796	0.059496	2.0667	0.1667	2	18	-0.0667	0	0	0
HIP_S9291	PLALEGSLQ	QGDLQDTKE	PLALEGSLQQGDLQDTKE		SCG1	10372/367830	0.998	S9291	0.796	-0.109472	-1.3167	0.3333	-3	18	-0.85	-3	0	0
HIP_S9292	SHLVEALYL	KADTEKWA	SHLVEALYLKADTEKWA		SCG1	36122/367830	0.939	S9292	0.796	0.224876	0.1167	0	0	17	-0.2471	-1	1	1
HIP_S9293	GPGAGSLQP	EEENELTLN	GPGAGSLQPEEENELTLN		SCG1	20398/367830	0.989	S9293	0.796	-0.049128	-1.9667	0.1667	-4	18	-0.9556	-4	0	0
HIP_S9294	CGERGFFY	RSQEESEE	CGERGFFYRSQEESEE		SCG1	27452/367830	0.974	S9294	0.796	0.188318	-0.75	0.1667	-3	16	-1.6	-3	3	0
HIP_S9295	AGSLQPLAL	IPLIFLISG	AGSLQPLALIPLIFLISG		SCG2	17181/334890	0.997	S9295	0.796	0.023006	2.6833	0.1667	0	18	1.6556	0	0	0
HIP_S9296	GSLQPLAL	GEQEHSQQ	GSLQPLALGEQEHSQQ		CMGA	12220/247050	0.992	S9296	0.796	0.211923	0.3333	0.1667	-1	16	-0.9375	-2	0	0
HIP_S9297	YLVCGERGF	TDKLAPVSK	YLVCGERGFTDKLAPVSK		SCG2	32587/334890	0.988	S9297	0.796	0.007000	-1.7	0.1667	1	18	-0.0778	1	1	0
HIP_S9298	LVCGERGFF	NMNDNFLE	LVCGERGFFNMNDNFLE		SCG1	21734/367830	0.987	S9298	0.796	0.026548	0.0167	0.1667	-2	17	-0.1	-2	2	1
HIP_S9299	VELGGGPGA	SLGYKEIRK	VELGGGPGASLGYKEIRK		CMGA	5924/247050	0.998	S9299	0.796	-0.067579	0.4	0.6667	1	18	-0.3833	1	1	0
HIP_S9300	AGSLQPLAL	EEDIQGPT	AGSLQPLALEEDIQGPT		SCG1	19733/367830	0.990	S9300	0.796	0.128940	-0.1833	0	-3	17	-0.2059	-3	0	1
HIP_S9301	VEALYLVCG	MGILKLQVF	VEALYLVCGMGILKLQVF		IAPP	1353/45018	0.994	S9301	0.796	-0.043224	2.05	0.3333	0	18	1.5722	0	1	0
HIP_S9302	VCGERGFFY	LHYRKKSA	VCGERGFFYLHYRKKSA		SCG1	37672/367830	0.931	S9302	0.796	0.068802	0.6	0	4	17	-0.5765	3	4	1
HIP_S9303	VELGGGPGA	GEETGSQEN	VELGGGPGAGEETGSQEN		SCG1	6479/367830	0.999	S9303	0.796	-0.095413	-1.2667	0.5	-4	18	-0.9278	-4	0	0
HIP_S9304	LYQLENYCN	DQELGEYLA	LYQLENYCNDQELGEYLA		SCG2	19286/334890	0.996	S9304	0.796	0.016544	-2.1333	0	-4	18	-0.7111	-4	1	0
HIP_S9305	GSHLVEALY	TEVMKCIVE	GSHLVEALYTEVMKCIVE		CMGA	11451/247050	0.993	S9305	0.796	0.063682	0.7167	0	-1	18	0.5611	-2	1	0
HIP_S9306	SLYQLENYC	PRPQSEESW	SLYQLENYCPRPQSEESW		SCG1	18662/367830	0.991	S9306	0.796	0.098973	-1.6667	0.3333	-2	18	-1.3611	-2	1	0
HIP_S9307	VEQCCTSIC	EAASFQRNQ	VEQCCTSICEAASFQRNQ		SCG2	18572/334890	0.997	S9307	0.796	-0.056474	1.05	0.1667	-1	18	-0.2889	-1	0	0
HIP_S9308	LVCGERGFF	SLPSQKYP	LVCGERGFFSLPSQKYP		CMGA	10251/247050	0.994	S9308	0.796	0.040759	1.1	0.5	1	17	-0.1412	1	2	1
HIP_S9309	GGGPGAGSL	AYGFRGPGP	GGGPGAGSLAYGFRGPGP		CMGA	5967/247050	0.998	S9309	0.796	-0.010146	0.45	0.5	1	18	-0.2444	1	2	0
HIP_S9310	YLVCGERGF	LRLENVQKF	YLVCGERGFLRLENVQKF		SCG2	17448/334890	0.997	S9310	0.796	0.063240	0.1667	0.1667	1	18	-0.0611	1	1	0
HIP_S9311	QVELGGGPG	EEEEEEEEE	QVELGGGPGEEEEEEEEE		CMGA	5323/247050	0.998	S9311	0.796	-0.088676	-2.35	0.5	-10	18	-1.8722	-10	0	0
HIP_S9312	LVEALYLVC	EVLSNALSK	LVEALYLVCEVLSNALSK		SCG1	21692/367830	0.987	S9312	0.796	0.036562	2.5	0	-1	18	1.1333	-1	1	0
HIP_S9313	EQCCTSICS	PSPMPVSQE	EQCCTSICSPSPMPVSQE		CMGA	4838/247050	0.998	S9313	0.796	0.109151	0.3667	0.6667	-2	18	-0.2556	-2	0	0
HIP_S9314	ERGFFYTPK	ESLSAIEAE	ERGFFYTPKESLSAIEAE		CMGA	13752/247050	0.989	S9314	0.796	-0.101042	-1.1167	0.3333	-2	18	-0.5833	-2	2	0
HIP_S9315	GSLQPLAL	QGSSQETDK	GSLQPLALQGSSQETDK		SCG2	16274/334890	0.997	S9315	0.796	0.221767	0.7833	0.3333	-1	17	-0.8059	-1	0	1
HIP_S9316	HLVEALYL	IESQTQEEV	HLVEALYLIESQTQEEV		SCG2	10587/334890	0.999	S9316	0.796	0.111749	1.0833	0.1667	-3	17	-0.0529	-4	1	1
HIP_S9317	VEALYLVCG	PGEEEEEEE	VEALYLVCGPGEEEEEEE		CMGA	1733/247050	1.000	S9317	0.796	0.021962	0.1333	0.5	-8	18	-0.6333	-8	1	0
HIP_S9318	HLVEALY	MARYYSAL	HLVEALYMARYYSAL		NPY	4629/49410	0.943	S9318	0.796	0.100054	0.5833	0	1	15	0.4667	0	3	1
HIP_S9319	LVEALYLVC	ASFQRNQL	LVEALYLVCASFQRNQL		SCG2	21005/334890	0.996	S9319	0.796	0.066315	2.3833	0.1667	0	17	0.7	0	2	1
HIP_S9320	GERGFFY	PQAEGDS	GERGFFYPQAEGDS		CMGA	14761/247050	0.987	S9320	0.796	0.131746	0.1667	0.1667	-2	14	-1.1429	-2	3	0
HIP_S9321	DLQVGQVEL	FEDELSEVL	DLQVGQVELFEDELSEVL		CMGA	13377/247050	0.990	S9321	0.796	-0.008500	0.05	0	-6	18	0.0778	-6	1	0
HIP_S9322	GQVELGGGP	GSGRLQNGQ	GQVELGGGPGSGRLQNGQ		SCG2	8406/334890	0.999	S9322	0.796	-0.098786	-0.6667	1	0	18	-0.8556	0	0	0
HIP_S9323	LVEALYLV	YRASEEEPE	LVEALYLVYRASEEEPE		SCG1	26650/367830	0.976	S9323	0.796	0.038931	0	0.5	-4	17	-0.2118	-4	2	1
HIP_S9324	VEQCCTSIC	ESWDEEDKR	VEQCCTSICESWDEEDKR		SCG1	14229/367830	0.995	S9324	0.796	-0.029018	0.1667	0.3333	-4	18	-1.1056	-4	1	0
HIP_S9325	ICSLYQLEN	ARMLVKYPE	ICSLYQLENARMLVKYPE		SCG2	24961/334890	0.994	S9325	0.796	-0.045558	-0.6667	0	0	18	-0.0611	0	1	0
HIP_S9326	HLCGSHLVE	PQEESEESN	HLCGSHLVEPQEESEESN		SCG1	17596/367830	0.992	S9326	0.796	0.107325	-0.6833	0.1667	-3	18	-1.1667	-5	0	0
HIP_S9327	RGFFYTPKT	EKDRADQTV	RGFFYTPKTEKDRADQTV		SCG1	39865/367830	0.919	S9327	0.796	0.015497	-2.85	0.1667	1	18	-1.3667	1	1	0
HIP_S9328	HLCGSHLV	GSEEYRAPR	HLCGSHLVGSEEYRAPR		SCG1	21510/367830	0.987	S9328	0.796	0.232904	0.0167	0.3333	2	17	-0.6824	0	1	1
HIP_S9329	GERGFFYT	SWDEEDKR	GERGFFYTSWDEEDKR		SCG1	27219/367830	0.974	S9329	0.796	0.067123	-0.7333	0.1667	-2	16	-1.8313	-2	4	0
HIP_S9330	ERGFFYTPK	RLGLSGLTL	ERGFFYTPKRLGLSGLTL		NPY	3119/49410	0.974	S9330	0.796	-0.100355	-1.2167	0.3333	2	18	-0.1056	2	2	0
HIP_S9331	GPGAGSLQP	QKENGDESH	GPGAGSLQPQKENGDESH		SCG2	31513/334890	0.989	S9331	0.796	-0.012280	-2.0333	0.1667	-1	18	-1.6056	-2	0	0
HIP_S9332	LVEALYLV	PEPMQESKA	LVEALYLVPEPMQESKA		CMGA	10486/247050	0.994	S9332	0.796	0.185536	0	0.3333	-2	17	0.1235	-2	1	1
HIP_S9333	SLYQLENYC	VDKRRTRPR	SLYQLENYCVDKRRTRPR		SCG1	46187/367830	0.880	S9333	0.796	0.028948	-0.9167	0	3	18	-1.5167	3	1	0

HIP_S9777	HLCGSHLVE	EEQIEQAIK	HLCGSHLVEEEQIEQAIK		SCG2	22864/334890	0.995	S9777	0.795	0.068728	-1	0	-1	18	-0.4111	-3	0
HIP_S9778	ERGFFYTPK	PYALNSEKN	ERGFFYTPKPYALNSEKN		SCG2	17550/334890	0.997	S9778	0.795	-0.032353	-1.2167	0.3333	1	18	-1.2667	1	3
HIP_S9779	VEALYLVC	GGSLPSEEK	VEALYLVCGGSLPSEEK		SCG1	15042/367830	0.995	S9779	0.795	0.211868	1.4833	0.5	-2	17	0.2588	-2	1
HIP_S9780	VEQCCTSIC	WWEKKPFS	VEQCCTSICWWEKKPFS		SCG1	28720/367830	0.970	S9780	0.795	0.019904	0.15	0.1667	0	17	-0.2941	0	2
HIP_S9781	AGSLQPLAL	AGSLQPLA	AGSLQPLALAGSLQPLA		INSC	205/5184	0.998	S9781	0.795	0.175409	1.6667	0.3333	0	17	0.8	0	0
HIP_S9782	GERGFFY	NQAPGEE	GERGFFYNQAPGEE		CMGA	18126/247050	0.979	S9782	0.795	0.119148	-0.15	0	-2	14	-1.3357	-2	3
HIP_S9783	CGERGFFY	NVGSNTYGK	CGERGFFYNVGSNTYGK		IAPP	2428/45018	0.986	S9783	0.795	0.169861	0.7667	0.1667	1	17	-0.7235	1	3
HIP_S9784	QHLCGSHLV	QVRGYPEEK	QHLCGSHLVQVRGYPEEK		CMGA	25865/247050	0.945	S9784	0.795	0.193184	0.1667	0	2	18	-0.8222	0	0
HIP_S9785	LCGSHLVEA	PVSKRFPVG	LCGSHLVEAPVSKRFPVG		SCG2	23785/334890	0.994	S9785	0.795	0.115451	0.7167	0.3333	2	18	0.3667	1	0
HIP_S9786	ICSLYQLEN	EEPEYGEEI	ICSLYQLENEEPEYGEEI		SCG1	18921/367830	0.991	S9786	0.795	-0.035124	-1.9667	0.1667	-6	18	-0.7944	-6	1
HIP_S9787	CSLYQLENY	WQAKREEEE	CSLYQLENYWQAKREEEE		CMGA	11901/247050	0.992	S9787	0.795	-0.079793	-1.8167	0	-3	18	-1.6	-3	2
HIP_S9788	HLVEALYLV	APPITPECR	HLVEALYLVAPPITPECR		SCG1	12511/367830	0.997	S9788	0.795	0.115777	0.8833	0.3333	0	18	0.4944	-1	1
HIP_S9789	SICSLYQLE	EATNTHPPA	SICSLYQLEEATNTHPPA		CMGA	20415/247050	0.971	S9789	0.795	-0.145782	-0.9333	0	-1	18	-0.3611	-2	1
HIP_S9790	PLALEGSLQ	ESEEGEEDA	PLALEGSLQESEEGEEDA		SCG1	13927/367830	0.996	S9790	0.795	-0.105331	-1.3833	0.3333	-7	18	-0.9444	-7	0
HIP_S9791	RGFFYTPKT	ELQKIAEKF	RGFFYTPKTELQKIAEKF		SCG1	36804/367830	0.936	S9791	0.795	0.055170	-1.5667	0.1667	2	18	-0.7167	2	1
HIP_S9792	EALYLVCGE	GDERILSIL	EALYLVCGEGDERILSIL		CMGA	10329/247050	0.994	S9792	0.795	-0.085379	-1.4667	0.3333	-3	18	0.6278	-3	0
HIP_S9793	CGERGFFY	VGGEDWNP	CGERGFFYVGGEDWNP		SCG2	5782/334890	0.999	S9793	0.795	0.120416	1.2833	0.3333	-2	16	-0.725	-2	3
HIP_S9794	CTSICSLYQ	EKRDHHSTH	CTSICSLYQEKRDHHSTH		SCG1	48015/367830	0.867	S9794	0.795	0.190540	-2.15	0	3	18	-1.1278	0	1
HIP_S9795	CCTSICSLY	WERYRGRG	CCTSICSLYWERYRGRG		SCG1	37879/367830	0.930	S9795	0.795	0.054743	-1.2	0.1667	2	17	-0.4588	2	3
HIP_S9796	EQCCTSICS	TSRKDVKDK	EQCCTSICSTSRKDVKDK		SCG1	36489/367830	0.937	S9796	0.795	0.192618	0.0333	0.3333	1	18	-0.9889	1	0
HIP_S9797	SHLVEALY	ALEYIENLR	SHLVEALYALEYIENLR		SCG2	20539/334890	0.996	S9797	0.795	-0.000719	1.0667	0	-1	17	0.1412	-2	2
HIP_S9798	LVEALYLV	SQQKEEEEE	LVEALYLVSQQKEEEEE		CMGA	11106/247050	0.993	S9798	0.795	0.166892	-0.1833	0.1667	-5	17	-0.7294	-5	1
HIP_S9799	LGGGPGAGS	ENQMDKARR	LGGGPGAGSENQMDKARR		SCG1	26460/367830	0.976	S9799	0.795	-0.048149	-1.65	0.3333	1	18	-1.2222	1	0
HIP_S9800	TSICSLYQL	SEKSQREDE	TSICSLYQLSEKSQREDE		SCG1	29485/367830	0.967	S9800	0.795	0.194362	-1.5333	0.1667	-2	18	-1.1111	-2	1
HIP_S9801	CSLYQLENY	QSIYQLIEI	CSLYQLENYQSIYQLIEI		SCG2	17793/334890	0.997	S9801	0.795	-0.124357	-1.35	0.1667	-2	18	0.05	-2	2
HIP_S9802	SHLVEALY	EDMARYYSA	SHLVEALYEDMARYYSA		NPY	3608/49410	0.966	S9802	0.795	0.147055	-0.1333	0	-1	17	-0.2706	-2	1
HIP_S9803	CGERGFFY	DLPVDLDD	CGERGFFYDLPVDLDD		SCG2	11825/334890	0.998	S9803	0.795	0.075985	0.5	0.1667	-4	16	-0.3625	-4	3
HIP_S9804	LVEALYLVC	EGPTVVLN	LVEALYLVCEGPTVVLN		CMGA	10154/247050	0.995	S9804	0.795	0.188769	0.8333	0.3333	-2	17	1.2824	-2	1
HIP_S9805	PLALEGSLQ	KRFPVGPPK	PLALEGSLQKRFPVGPPK		SCG2	44357/334890	0.965	S9805	0.795	0.040623	-1.0167	0.1667	2	18	-0.3944	2	1
HIP_S9806	GERGFFYTP	EEPEYGEE	GERGFFYTPEEPEYGEE		SCG1	19386/367830	0.990	S9806	0.795	-0.054429	-2.0333	0.3333	-5	17	-1.6235	-5	3
HIP_S9807	LVEALYLV	DSQVSEEVK	LVEALYLVDSQVSEEVK		SCG1	18289/367830	0.992	S9807	0.795	0.181181	-0.1833	0.1667	-3	17	0.3353	-3	1
HIP_S9808	QCCTSICSL	RPRHHHGRS	QCCTSICSLRPRHHHGRS		SCG1	43195/367830	0.900	S9808	0.795	0.166797	-0.85	0.3333	6	18	-0.8833	3	0
HIP_S9809	LVEALYLV	EEKHLEEP	LVEALYLVEEKHLEEP		SCG1	23604/367830	0.983	S9809	0.795	0.173365	-0.7	0	-3	16	-0.1313	-4	1
HIP_S9810	HLVEALYLV	GAILSSTN	HLVEALYLVGAILSSTN		IAPP	1783/45018	0.992	S9810	0.795	0.028240	2.1	0.1667	0	17	1.0294	-1	1
HIP_S9811	VGQVELGG	EWERYRGRG	VGQVELGGEWERYRGRG		SCG1	23649/367830	0.983	S9811	0.795	0.024803	-0.8167	0.3333	0	17	-1.1471	0	2
HIP_S9812	LGGGPGAGS	PQGLFRGGK	LGGGPGAGSPQGLFRGGK		CMGA	11077/247050	0.993	S9812	0.795	-0.031421	-0.8167	0.6667	2	18	-0.3833	2	0
HIP_S9813	LVCGERGFF	RLQNGQNG	LVCGERGFFRLQNGQNG		SCG2	31978/334890	0.988	S9813	0.795	0.078749	0.1667	0.1667	1	17	-0.4824	1	2
HIP_S9814	IVEQCCTSI	GEEAVPEEE	IVEQCCTSIGEEAVPEEE		CMGA	4437/247050	0.999	S9814	0.795	-0.072259	-0.7333	0.3333	-6	18	-0.2111	-6	0
HIP_S9815	GPGAGSLQP	NYDWWEKK	GPGAGSLQPNYDWWEKK		SCG1	31073/367830	0.961	S9815	0.795	0.017414	-1.6	0.1667	0	17	-1.4412	0	2
HIP_S9816	SHLVEALYL	DVSKVIAY	SHLVEALYLDVSKVIAY		SCG2	21966/334890	0.995	S9816	0.795	0.205795	1.0333	0.1667	0	17	0.8118	-1	1
HIP_S9817	HLVEALYL	PSSREDSL	HLVEALYLPSSREDSL		CMGA	11848/247050	0.992	S9817	0.795	0.227640	0.5167	0.5	-1	16	-0.1438	-2	1
HIP_S9818	VEALYLVC	QVEKRKCNT	VEALYLVCQVEKRKCNT		IAPP	3594/45018	0.966	S9818	0.795	0.172033	1.2833	0	1	17	-0.0765	1	1
HIP_S9819	GSHLVEALY	LEGEEENEL	GSHLVEALYLEGEEENEL		SCG1	4954/367830	0.999	S9819	0.795	0.034752	0.7	0.1667	-5	18	-0.5222	-6	1
HIP_S9820	SLYQLENYC	GSSEDDLQE	SLYQLENYCGSSEDDLQE		SCG2	5511/334890	0.999	S9820	0.795	0.061826	-0.7167	0.5	-5	18	-1.0833	-5	1
HIP_S9821	LCGSHLVEA	QGAKERAHQ	LCGSHLVEAQGAKERAHQ		CMGA	20256/247050	0.971	S9821	0.795	0.150402	0.0667	0.1667	2	18	-0.5944	0	0
HIP_S9822	GSLQPLAL	AEDLQVGQ	GSLQPLALAEDLQVGQ		INSC	262/5184	0.997	S9822	0.795	0.081547	0.7	0	-2	16	0.1437	-2	0
HIP_S9823	VEALYLVCG	LISDLLMRE	VEALYLVCGLISDLLMRE		NPY	983/49410	0.995	S9823	0.795	-0.067531	2.3	0.3333	-2	18	1.1444	-2	1
HIP_S9824	CGSHLVEAL	NPYYDPLQ	CGSHLVEALNPYYDPLQ		SCG1	27736/367830	0.973	S9824	0.795	0.055108	-0.7167	0.1667	-1	17	-0.2529	-2	2
HIP_S9825	GSLQPLALE	GQEEEEDNR	GSLQPLALEGQEEEEDNR		CMGA	8667/247050	0.996	S9825	0.795	0.101308	-0.8833	0.1667	-5	18	-1.4444	-5	0
HIP_S9826	EQCCTSICS	NNQAPGEEE	EQCCTSICSNNQAPGEEE		CMGA	9556/247050	0.995	S9826	0.795	0.034854	-0.7167	0.1667	-4	18	-1.0278	-4	0
HIP_S9827	GPGAGSLQP	ETQQWPERK	GPGAGSLQPETQQWPERK		SCG2	28740/334890	0.991	S9827	0.795	-0.011179	-1.5	0.1667	0	18	-1.5944	0	0
HIP_S9828	GERGFFYTP	GQNGERAT	GERGFFYTPGQNGERAT		SCG2	10928/334890	0.996	S9828	0.795	-0.004858	-1.8333	0.3333	0	17	-1.2647	0	3
HIP_S9829	TSICSLYQ	SAEFPDFYD	TSICSLYQSAEFPDFYD		SCG1	17173/367830	0.993	S9829	0.795	-0.016432	-0.5833	0.1667	-3	17	-0.1824	-3	2
HIP_S9830	CGERGFFYT	LAVDGAGK	CGERGFFYTLAVDGAGK		CMGA	14200/247050	0.989	S9830	0.795	-0.038008	1.7667	0	0	17	0.0412	0	3
HIP_S9831	LVEALYLVC	EKGHPQEE	LVEALYLVCEKGHPQEE		SCG1	21578/367830	0.987	S9831	0.795	0.208556	0.45	0.1667	-2	17	-0.2235	-3	1
HIP_S9832	VCGERGFF	EEEEEATN	VCGERGFFEEEEEATN		CMGA	13835/247050	0.989	S9832	0.795	0.034684	-0.8833	0.1667	-5	16	-1.025	-5	2
HIP_S9833	GSLQPLALE	SNQVKRVPG	GSLQPLALESNQVKRVPG		SCG2	29751/334890	0.990	S9833	0.795	0.147916	-0.95	0.1667	1	18	-0.3556	1	0
HIP_S9834	SHLVEALYL	LESLSAIEA	SHLVEALYLLESLSAIEA		CMGA	9216/247050	0.996	S9834	0.795	0.112862	0.9667	0.1667	-2	18	0.8722	-3	1

HIP_60288	CGERGFFYT	ETHAAGHS	CGERGFFYTETHAAGHS		SCG1	34871/367830	0.945	60288	0.795	0.074563	-1.1	0	1	17	-0.6412	-1	3	1	0	0.000000	-2.7333	-1	0.05	0.450000	0.1111	0.3389	0	0	0	0	2.7333	-0.3728	-8.2	0	1	0	0	0.4769	1.0688	0.8456	-0.1667	0.2275	0.7151	-0.5246	0.0025	-0.1367
HIP_60289	TSICSLYQL	PEMIRALEY	TSICSLYQLPEMIRALEY		SCG2	15146/334890	0.998	60289	0.795	-0.019703	-0.7	0.1667	-1	18	0.2833	-1	1	0	0	0.000000	-0.7333	-1	0.0667	1.125000	0.1111	1.0139	1	0	1	0	0.7333	-0.7097	-0.7333	1	0	0	0	0.4551	1.1186	0.8745	0.1667	0.2071	0.7647	-0.3186	0.0044	-0.0489
HIP_60290	HLCGSHLVE	LNSEKNFPM	HLCGSHLVELNSEKNFPM		SCG2	23635/334890	0.994	60290	0.795	0.038704	0.6667	0.1667	1	18	-0.2833	-1	0	0	0	0.000000	-1.6667	1	0.0667	2.000000	0.2222	1.7778	0	0	0	0	-1.6667	1.1852	0	0	0	1	0	0.4	1.1067	0.8333	0.3333	0.16	0.6944	0.2667	0.0044	-0.1111
HIP_60291	GGGPGAGS	VCGERGFFY	GGGPGAGSVCGERGFFY		INSB	20/4968	0.999	60291	0.795	0.000818	1.15	0.5	0	17	-0.0235	0	0	1	1	0.000000	1.9	0	-0.0667	0.625000	0.2222	0.4028	0	2	0	1	0	0.4632	0	0	1	0	0	0.4718	0.8886	1.0445	0.3333	0.2226	1.091	0.5426	0.0044	-0.1267
HIP_60292	GGPGAGSLQ	PNGSVEPER	GGPGAGSLQPNGSVEPER		SCG2	8340/334890	0.999	60292	0.795	-0.049637	-1	0.5	-1	18	-0.95	-1	0	0	0	0.000000	-1.6667	0	0.0833	1.400000	0	1.4	1	1	1	1	0	-1.4	0	0	1	0	0	0.5667	0.825	1.21	0.1667	0.3211	1.4641	-0.5667	0.0069	-0.1389
HIP_60293	LCGSHLVEA	DVSKVIAYL	LCGSHLVEADVSKVIAYL		SCG2	26931/334890	0.992	60293	0.795	0.098767	0.4	0.1667	0	18	0.9556	-1	0	0	0	0.000000	-0.8667	0	0.0667	0.000000	0.3333	-0.3333	0	0	0	0	0	-0.1333	0	1	0	0	0	0.3718	1.1503	0.7512	0.1667	0.1382	0.5642	0.1487	0.0044	-0.0578
HIP_60294	YLVCGERGF	LKLQVFLIV	YLVCGERGFLKLQVFLIV		IAPP	2159/45018	0.989	60294	0.795	0.055399	0.2667	0.1667	1	18	1.2722	1	1	0	0	1.000000	1.9333	0	-0.0833	0.950000	0.3333	0.6167	0	1	0	1	0	0.1644	1.9333	1	0	0	0	0.3603	1.0572	0.819	0.1667	0.1298	0.6707	0.0961	0.0069	-0.1611
HIP_60295	SLYQLENYC	HSRERADEP	SLYQLENYCHSRERADEP		SCG1	37311/367830	0.933	60295	0.795	0.037952	-1.8	0.1667	-1	18	-1.5056	-2	1	0	0	0.000000	-2.0667	1	0.0667	0.950000	0.3333	0.6167	0	0	0	0	-2.0667	-1.11	-2.0667	0	0	0	0	0.4872	0.926	1.0646	-0.3333	0.2373	1.1334	-0.8769	0.0044	-0.1378
HIP_60296	HLVEALYL	DNPGEDAP	HLVEALYLDNPGEDAP		NPY	2415/49410	0.983	60296	0.795	0.198799	-0.3833	0.1667	-3	16	-0.4	-4	1	0	0	0.000000	-4.9667	-1	0.1667	0.000000	0.2222	-0.2222	1	0	1	0	4.9667	0.0852	-4.9667	1	0	0	0	0.4269	0.9805	0.9823	0.1667	0.1823	0.9649	-0.1637	0.0278	-0.8278
HIP_60297	HLVEALYL	PDNPGEDA	HLVEALYLPDNPGEDA		NPY	2416/49410	0.983	60297	0.795	0.198799	-0.3833	0.1667	-3	16	-0.4	-4	1	0	0	0.000000	-4.9667	-1	0.1667	0.000000	0.2222	-0.2222	1	0	1	0	4.9667	0.0852	-4.9667	1	0	0	0	0.4269	0.9805	0.9823	0.1667	0.1823	0.9649	-0.1637	0.0278	-0.8278
HIP_60298	LVCGERGFF	VPGQGSSED	LVCGERGFFVPGQGSSED		SCG2	435/334890	1.000	60298	0.795	-0.038808	1.2333	0.5	-2	18	-0.1667	-2	2	0	0	1.000000	-1	0	0.0667	1.750000	0.2222	1.5278	1	2	1	1	0	1.8843	-2	1	0	0	0	0.45	0.8383	1.0367	0.5	0.2025	1.0747	0.555	0.0044	-0.0667
HIP_60299	ICSLYQLEN	EPEYGEEI	ICSLYQLENEPEYGEEI		SCG1	22021/367830	0.986	60299	0.795	-0.004676	-1.9667	0.1667	-5	17	-0.6353	-5	2	1	0	0.000000	-1.8	-1	0.0667	0.000000	0.3333	-0.3333	1	0	1	0	1.8	0.6556	-3.6	0	1	0	0	0.55	1.1783	0.9667	-0.3333	0.3025	0.9344	-1.0817	0.0044	-0.12
HIP_60300	GERGFFYTP	YIENLRQQA	GERGFFYTPYIENLRQQA		SCG2	19165/334890	0.996	60300	0.795	-0.058860	-0.65	0.1667	0	18	-0.9167	0	4	0	0	0.000000	1.1	-1	-0.0667	0.700000	0.1111	0.5889	1	0	1	0	-1.1	-0.3828	4.4	0	0	0	1	0.4936	1.0055	0.9173	0	0.2436	0.8415	-0.3208	0.0044	-0.0733
HIP_60301	GSLQPLAL	GELFNPYY	GSLQPLALGELFNPYY		SCG1	15848/367830	0.994	60301	0.795	0.179394	1.55	0.1667	-1	16	0.1187	-1	1	0	0	0.000000	-3.1667	-1	0.1333	1.150000	0.1111	1.0389	0	1	0	1	3.1667	1.6103	-3.1667	1	0	0	0	0.35	1.2267	0.7333	0.5	0.1225	0.5378	0.5425	0.0178	-0.4222
HIP_60302	NQHLCGSHL	GPGPQLRRG	NQHLCGSHLGPGPQLRRG		CMGA	11673/247050	0.993	60302	0.795	0.153080	-0.4333	0.6667	4	18	-0.9778	2	0	0	0	0.000000	-0.7333	0	0.1667	0.625000	0	0.625	1	2	1	1	0	-0.2708	0	1	0	0	0	0.5551	0.7819	1.1878	0	0.3082	1.4109	-0.2406	0.0278	-0.1222
HIP_60303	SLYQLENYC	EEEEEMAV	SLYQLENYCEEEEEMAV		CMGA	13049/247050	0.990	60303	0.795	0.020599	-2.1333	0	-6	17	-0.7882	-6	1	1	3	0.000000	-2.7333	-3	0.1	0.950000	0.5556	0.3944	0	0	0	0	8.2	-0.8415	-2.7333	0	0	0	0	0.5436	1.2072	0.9323	-0.5	0.2955	0.8692	-1.1597	0.01	-0.2733
HIP_60304	ICSLYQLEN	KGEQEHSQQ	ICSLYQLENKGEQEHSQQ		CMGA	20491/247050	0.970	60304	0.795	0.080963	-1.8333	0.1667	-1	18	-1.3222	-2	1	0	0	0.000000	-1.5333	1	0.0833	0.000000	0.3333	-0.3333	0	1	0	1	-1.5333	0.6111	-1.5333	0	1	0	0	0.5218	1.0853	0.9912	-0.3333	0.2723	0.9824	-0.9566	0.0069	-0.1278
HIP_60305	HLCGSHLVE	AKTHWLGAA	HLCGSHLVEAKTHWLGAA		SCG2	38769/334890	0.979	60305	0.795	0.070948	0.2833	0	3	18	0.1833	0	0	0	0	0.000000	-2.4333	2	0.05	1.500000	0.2222	1.2778	0	0	0	0	-4.8667	0.362	0	0	0	1	0	0.3718	1.1953	0.7112	0.1667	0.1382	0.5057	0.1053	0.0025	-0.1217
HIP_60306	GPGAGSLQP	TYGKRNAVE	GPGAGSLQPTYGKRNAVE		IAPP	2534/45018	0.985	60306	0.795	-0.041645	-0.6167	0.3333	1	18	-0.8278	1	1	0	0	0.000000	-0.3667	0	0.0167	0.000000	0.1111	-0.1111	1	1	1	1	0	0.0685	-0.3667	0	0	0	1	0.5385	0.8886	1.0612	0.1667	0.2899	1.126	-0.3321	0.0003	-0.0061
HIP_60307	LYQLENYCN	QAPGEEEEE	LYQLENYCNQAPGEEEEE		CMGA	4980/247050	0.998	60307	0.795	-0.100442	-0.9333	0.1667	-6	18	-1.5389	-6	1	0	3	0.000000	-0.3333	0	0.0667	0.625000	0.1111	0.5139	1	0	1	0	0	-0.4796	-0.3333	0	1	0	0	0.4936	0.9738	1.089	0.1667	0.2436	1.1859	-0.4607	0.0044	-0.0222
HIP_60308	YLVCGERGF	EYRAPRPQS	YLVCGERGFEYRAPRPQS		SCG1	36353/367830	0.938	60308	0.795	0.036967	-1.9	0.1667	1	18	-0.9056	1	2	0	0	0.000000	-2.4	-1	-0.0333	0.450000	0.4444	0.0056	0	1	0	1	2.4	-0.0106	-4.8	1	0	0	0	0.4654	1.0458	0.9268	-0.3333	0.2166	0.8589	-0.8842	0.0011	0.08
HIP_60309	AGSLQPLAL	DNMNDNFL	AGSLQPLALDNMNDNFL		SCG1	20108/367830	0.989	60309	0.795	0.149160	0.7167	0	-2	17	-0.0176	-2	0	1	0	0.000000	-4.8333	-1	0.1	0.450000	0.1111	0.3389	0	0	0	0	4.8333	0.2429	0	1	0	0	0	0.3051	1.1569	0.7878	0.5	0.0931	0.6207	0.2187	0.01	-0.4833
HIP_60310	HLVEALYLV	EISRNLQI	HLVEALYLVEISRNLQI		SCG2	18403/334890	0.997	60310	0.795	0.173322	1.15	0.1667	0	17	0.6235	-1	1	1	0	0.000000	-2.1667	-1	0.1167	1.400000	0.2222	1.1778	0	0	0	0	2.1667	1.3544	-2.1667	1	0	0	0	0.3718	1.1286	0.7195	0.3333	0.1382	0.5177	0.4276	0.0136	-0.2528
HIP_60311	FVNQHLCGS	AWIPHVENR	FVNQHLCGSAWIPHVENR		SCG2	8189/334890	0.999	60311	0.795	0.141724	1.1167	0.3333	2	18	-0.2667	0	1	0	0	0.000000	1.3667	0	-0.15	0.450000	0	0.45	0	1	0	1	0	0.5025	1.3667	0	1	0	0	0.4269	0.9788	0.9406	0.5	0.1823	0.8848	0.4767	0.0225	-0.205
HIP_60312	LVEALYLVC	AGEEAVPE	LVEALYLVCAGEEAVPE		CMGA	7770/247050	0.997	60312	0.795	0.118649	1.4	0.1667	-4	17	0.8588	-4	1	1	0	0.000000	-4.2	-1	0.1333	2.450000	0.2222	2.2278	0	1	0	1	4.2	3.1189	-4.2	0	0	0	0	0.3885	1.1403	0.8012	0.3333	0.1509	0.6418	0.5438	0.0178	-0.56
HIP_60313	QCCTSICSL	PGQGSSEDD	QCCTSICSLPGQGSSEDD		SCG2	6646/334890	0.999	60313	0.795	0.130808	0	0.5	-3	18	-0.4444	-3	0	0	0	0.000000	-3.6667	0	0.1333	0.000000	0	0	1	1	1	1	0	0	0	1	0	0	0	0.5385	0.8836	1.1012	0.1667	0.2899	1.2125	0	0.0178	-0.4889
HIP_60314	SHLVEALYL	ISEADLDH	SHLVEALYLISEADLDH		SCG2	11797/334890	0.998	60314	0.795	0.117541	1.0833	0.1667	-2	17	0.2471	-4	1	1	0	1.000000	-2.0333	-1	0.1167	1.575000	0.2222	1.3528	0	0	0	0	2.0333	1.4655	-2.0333	1	0	0	0	0.3718	1.1686	0.7195	0.3333	0.1382	0.5177	0.4028	0.0136	-0.2372
HIP_60315	ICSLYQLEN	NTHPPASLP	ICSLYQLENNTHPPASLP		CMGA	27259/247050	0.936	60315	0.795	-0.007027	-1.7667	0	0	18	-0.3	-1	1	0	0	0.000000	-1.4	1	0	0.000000	0.1111	-0.1111	0	0	0	0	-1.4	0.1963	-1.4	0	1	0	0	0.5051	1.0003	1.0378	-0.1667	0.2552	1.0771	-0.8924	0	0
HIP_60316	VELGGGPGA	SEESWDEED	VELGGGPGASEESWDEED		SCG1	7085/367830	0.999	60316	0.795	-0.124409	-1.3333	0.5	-7	18	-1.1333	-7	0	0	1	0.000000	-2.5333	-2	0.0167	0.000000	0.2222	-0.2222	1	1	1	1	5.0667	0.2963	0	1	0	0	0	0.5667	1.0567	1.06	-0.1667	0.3211	1.1236	-0.7556	0.0003	-0.0422
HIP_60317	QVELGGGPG	PKTPGRAGT	QVELGGGPGPKTPGRAGT		SCG2	15782/334890	0.997	60317	0.795	-0.036118	-1.4333	0.6667	1	18	-0.7889	1	0	0	1	0.000000	-1.2667	1	-0.0667	0.000000	0.1111	-0.1111	2	2	1	1	-1.2667	0.1593	0	0	0	0	1	0.6385	0.6653	1.3178	-0.1667	0.4076	1.7367	-0.9151	0.0044	0.0844
HIP_60318	LVEALYLV	EEEEEGENY	LVEALYLVEEEEEGENY		SCG1	16859/367830	0.993	60318	0.795	0.094581	-0.6333	0	-7	17	-0.5529	-7	1	1	3	0.000000	-5.7333	-3	0.15	2.000000	0.5556	1.4444	0	0	0	0	17.2	-0.9148	-5.7333	1	0	0	0	0.4385	1.3186	0.6878	-0.1667	0.1922	0.4731	-0.2777	0.0225	-0.86
HIP_60319	SHLVEALYL	NQLPKAAW	SHLVEALYLNQLPKAAW		SCG2	29098/334890	0.991	60319	0.795	0.224691	0.5167	0	1	17	0.1529	0	1	1	0	0.000000	-3.1667	0	0.1	0.000000	0.1111	-0.1111	0	0	0	0	0	-0.0574	-3.1667	1	0	0	0	0.3718	1.1169	0.8245	0.5	0.1382	0.6798	0.1921	0.01	-0.3167
HIP_60320	SHLVEALY	EKSGEATDG	SHLVEALYEKSGEATDG		CMGA	17006/247050	0.979	60320	0.795	0.237680	-0.65	0.1667	-2	17	-0.5941	-3	1	1	0	0.000000	-4.1667	0	0.1667	0.000000	0.4444	-0.4444	0	0	0	0	0	0.2889	-4.1667	0	1	0	0	0.4103	1.1838	0.819	0	0.1683	0.6707	-0.2667	0.0278	-0.6944
HIP_60321	GGGPGAGSL	GQGSSEDDL	GGGPGAGSLGQGSSEDDL		SCG2	2123/334890	1.000	60321	0.795	-0.040267	-0.2833	0.6667	-3	18	-0.6333	-3	0	0	1	0.000000	-2.3	0	0.0667	0.000000	0	0	0	3	0	1	0	0	0	1	0	0	0	0.5833	0.785	1.1967	0.1667	0.3403	1.432	-0.1653	0.0044	-0.1533
HIP_60322	GAGSLQPLA	RKLKHMQFP	GAGSLQPLARKLKHMQFP		SCG2	40192/334890	0.977	60322	0.795	0.056783	-0.1	0.1667	4	18	-0.4222	3	0	0	0	0.000000	-2.8667	2	0.0333	1.900000	0.3333	1.5667	1	0	1	0	-5.7333	-0.1567	0	1	0	0	0	0.3603	1.1105	0.8323	0.1667	0.1298	0.6927	-0.036	0.0011	-0.0956
HIP_60323	HLCGSHLVE	SQREDEEEE	HLCGSHLVESQREDEEEE		SCG1	26659/367830	0.976	60323	0.795	0.090967	-0.7167	0.1667	-4	18	-1.4778	-6	0	0	2	0.000000	-4.4333	2	0.1167	1.050000	0.3333	0.7167	0	0	0	0	-8.8667	-0.5136	0	0	0	1	0	0.4385	1.1336	0.8045	0	0.1922	0.6472	-0.3142	0.0136	-0.5172
HIP_60324	VCGERGFF	PYGAGRSR	VCGERGFFPYGAGRSR		SCG2	21185/334890	0.996	60324	0.795	0.075213	0.3167	0.5	2	16	-0.5125	2	3	0	0	0.000000	-2.8333	0	0.0667	1.150000	0.2222	0.9278	1	2	1	1	0	0.2938	-8.5	1	0	0	0	0.4885	0.8319	1.1045	0.3333	0.2386	1.2199	0.1547	0.0044	-0.1889
HIP_60325	VGQVELGGG	SKSGYPKTP	VGQVELGGGSKSGYPKTP		SCG2	15661/334890	0.998	60325	0.795	-0.024167	-1.1167	0.8333	1	18	-0.6333	1	0	0	1	0.000000	-1.4333	1	-0.0667	0.000000	0.2222	-0.2222	0	3	0	1	-1.4333	0.2481	0	0	0	0	1	0.6218	0.6869	1.3012	-0.1667	0.3866	1.693	-0.6943	0.0044	0.0956
HIP_60326	VEALYLVC	FLIVLSVA	VEALYLVCFLIVLSVA		IAPP	2408/45018	0.986	60326	0.795	-0.019541	3.6	0	-1	16	2.4875	-1	2	0	0	0.000000	0.2	0	0	3.750000	0	3.75	0	0	0	0	0	13.5	0.4	0	0	0	0	0.2385	1.1603	0.5795	0.8333	0.0569	0.3358	0.8585	0	0
HIP_60327	EAEDLQVGQ	YNQEKVLPR	EAEDLQVGQYNQEKVLPR		SCG2	17820/334890	0.997	60327	0.795	-0.097639	-1.3333	0.1667	-2	18	-1.2167	-2	1	0	0	0.000000	-2.8667	0	-0.0333	0.950000	0.1111	0.8389	0	1	0	1	0	-1.1185	-2.8667	0	1	0	0	0.5218	0.9119	1.0712	0.1667	0.2723	1.1474	-0.6957	0.0011	0.0956
HIP_60328	GPGAGSLQP	EEKRFLGEG	GPGAGSLQPEEKRFLGEG		SCG1	34540/367830	0.947	60328	0.795	0.009290	-2.0333	0.1667	-1	18	-0.9	-1	0	0	0	0.000000	-3.2	-1	0.0333	0.000000	0.4444	-0.4444	1	0	1	0	3.2	0.9037	0	0	0	0	1	0.5218	1.1703	0.8978	-0.3333	0.2723	0.8061	-1.061	0.0011	-0.1067
HIP_60329	GGGPGAGSL	SSQGGSLPS	GGGPGAGSLSSQGGSLPS		SCG1	14998/367830	0.995	60329	0.795	-0.019818	-0.4167	0.6667	0	18	-0.2278	0	0	0	1	0.000000	-2.5667	0	0.0333	0.000000	0	0	0	1	0	1	0	0	0	1	0	0	0	0.55	0.885	1.0967	0.1667	0.3025	1.2027	-0.2292	0.0011	-0.0856
HIP_60330	LVEALYLV	EDNRDSSM	LVEALYLVEDNRDSSM		CMGA	13717/247050	0.989	60330	0.795	0.148815	-0.6333	0	-3	16	-0.0875	-3	1	0	0	0.000000	-5.7333	-2	0.15	1.400000	0.3333	1.0667	0	0	0	0	11.4667	-0.6756	-5.7333	1	0	0	0	0.4103	1.0972	0.8523	0	0.1683	0.7264	-0.2598	0.0225	-0.86
HIP_60331	LVCGERGFF	KDVEVISD	LVCGERGFFKDVEVISD		CMGA	10158/247050	0.995	60331	0.795	-0.032651	1.3833	0.1667	-1	18	0.8611	-1	2	0	0	0.000000	-0.7	1	-0.0333	1.750000	0.3333	1.4167	0	1	0	1	-0.7	1.9597	-1.4	1	0	0	0	0.3603	1.0038	0.8306	0.3333	0.1298	0.69	0.4984	0.0011	0.0233
HIP_60332	LVEALYLVC	ENENTKFE	LVEALYLVCENENTKFE		SCG1	20743/367830	0.988	60332	0.795	0.137118	0	0	-3	17	0	-3	1	1	0	0.000000	-7	-2	0.15	2.000000	0.2222	1.7778	0	0	0	0	14	0	-7	0	0	0	0	0.4385	1.1786	0.8245	0	0.1922	0.6798	0	0.0225	-1.05
HIP_60333	VELGGGPGA	PSEEKGHPQ	VELGGGPGAPSEEKGHPQ		SCG1	9893/367830	0.998	60333	0.795	-0.075866	-1.0167	0.6667	-1	18	-1.05	-2	0	0	1	0.000000	-1.9	-1	0.0333	0.000000	0.2222	-0.2222	2	1	1	1	1.9	0.2259	0	1	0	0	0	0.5833	0.9	1.19	0	0.3403	1.4161	-0.5931	0.0011	-0.0633
HIP_60334	EQCCTSICS	KGQPRSQEE	EQCCTSICSKGQPRSQEE		SCG1	25051/367830	0.980	60334	0.795	0.161245	-0.2667	0.3333	-1	18	-1.25	-1	0	0	0	0.000000	-4.6667	1	0.1167	0.625000	0.1111	0.5139	0	1	0	1	-4.6667	-0.137	0	0	1	0	0	0.4936	0.9222	0.994	0	0.2436	0.988	-0.1316	0.0136	-0.5444
HIP_60335	PGAGSLQPL	KGEQEHSQQ	PGAGSLQPLKGEQEHSQQ		CMGA	15941/247050	0.985	60335	0.795	0.038373	-1.5167	0.3333	0	18	-1.3722	-1	0	0	0	0.000000	-2.1667	0	0.0667	0.000000	0.2222	-0.2222	1	1	1	1	0	0.337	0	1	0	0	0	0.5385	1.0019	1.0245	-0.1667	0.2899	1.0496	-0.8167	0.0044	-0.1444

[illegible]

HIP_60799	VEALYLVC	GLSVEDILN	VEALYLVCGLSVEDILN		SCG2	9198/334890	0.999	60799	0.794	0.153085	2.1833	0.3333	-3	17	1.1824	-3	1	1
HIP_60800	PGAGSLQPL	GQNGERAT	PGAGSLQPLGQNGERAT		SCG2	16764/334890	0.997	60800	0.794	0.025153	-1.45	0.3333	0	17	-0.8	0	0	1
HIP_60801	GPGAGSLQP	YPGPQAEGD	GPGAGSLQPYPGPQAEGD		CMGA	5120/247050	0.998	60801	0.794	-0.101198	-0.7667	0.5	-2	18	-0.95	-2	1	0
HIP_60802	YLVCGERGF	KRSSPETLI	YLVCGERGFKRSSPETLI		NPY	5501/49410	0.918	60802	0.794	0.082769	-1.8833	0.3333	1	18	-0.2389	1	1	0
HIP_60803	CCTSICSLY	LNQGSSQET	CCTSICSLYLNQGSSQET		SCG2	18080/334890	0.997	60803	0.794	-0.105998	-0.25	0.1667	-1	18	-0.0389	-1	1	0
HIP_60804	CSLYQLEN	NWGYEKRNL	CSLYQLENNWGYEKRNL		SCG1	26799/367830	0.975	60804	0.794	-0.009192	-1.3333	0.1667	0	17	-1.1882	0	2	1
HIP_60805	VEALYLVC	RGEAGAPGE	VEALYLVCRGEAGAPGE		SCG1	24168/367830	0.982	60805	0.794	0.231296	0.35	0.1667	-2	17	0.2824	-2	1	1
HIP_60806	VEQCCTSIC	PLIFLISGA	VEQCCTSICPLIFLISGA		SCG2	13475/334890	0.998	60806	0.794	-0.048276	2.15	0.3333	-1	18	1.45	-1	1	0
HIP_60807	LCGSHLVEA	KEEEGSANR	LCGSHLVEAKEEEGSANR		CMGA	25896/247050	0.945	60807	0.794	0.187646	-1.4	0	-1	18	-0.7556	-2	0	0
HIP_60808	LVEALYLVC	SLLGAVGL	LVEALYLVCSLLGAVGL		SCG1	19761/367830	0.990	60808	0.794	0.041005	2.8833	0.1667	-1	17	2.0647	-1	1	1
HIP_60809	SICSLYQLE	HGYGEESEE	SICSLYQLEHGYGEESEE		SCG1	16569/367830	0.993	60809	0.794	-0.177036	-1.35	0.1667	-4	18	-0.8556	-5	2	0
HIP_60810	AGSLQPLAL	DSLEAGLP	AGSLQPLALDSLEAGLP		CMGA	12171/247050	0.992	60810	0.794	0.166175	1.4833	0.1667	-2	17	0.4882	-2	0	1
HIP_60811	GSHLVEALY	DSSEEKHL	GSHLVEALYDSSEEKHL		SCG1	18121/367830	0.992	60811	0.794	0.194225	-0.1333	0.3333	-1	17	-0.6471	-3	1	1
HIP_60812	FVNQHLCG	FPEYNYDWW	FVNQHLCGFPEYNYDWW		SCG1	18180/367830	0.992	60812	0.794	0.020173	0.6	0.3333	-1	17	-0.6471	-2	2	1
HIP_60813	GERGFFYT	SFQRNQLL	GERGFFYTSFQRNQLL		SCG2	27913/334890	0.992	60813	0.794	0.091974	-0.1167	0.1667	1	16	-0.6625	1	4	0
HIP_60814	HLVEALYL	PGAEEAQDP	HLVEALYLPGAEEAQDP		CMGA	4061/247050	0.999	60814	0.794	0.141375	1.0167	0.3333	-3	17	-0.2706	-4	1	1
HIP_60815	HLCGSHLVE	GSSEDDLQE	HLCGSHLVEGSSEDDLQE		SCG2	9238/334890	0.999	60815	0.794	0.143029	0.4167	0.5	-3	18	-0.6944	-5	0	0
HIP_60816	CSLYQLENY	GAEAASFQR	CSLYQLENYGAEAASFQR		SCG2	21292/334890	0.996	60816	0.794	-0.138275	-1.7333	0.1667	-1	18	-0.4611	-1	1	0
HIP_60817	NQHLCGSHL	QLIEISRNL	NQHLCGSHLQLIEISRNL		SCG2	26747/334890	0.993	60817	0.794	0.011176	0.7667	0.1667	2	18	-0.2056	0	0	0
HIP_60818	HLVEALYL	QENHPQES	HLVEALYLQENHPQES		SCG1	24894/367830	0.980	60818	0.794	0.209877	-0.7	0	-1	16	-0.8563	-3	1	0
HIP_60819	SLQPLAL	SLGYKEI	SLQPLALSLGYKEI		CMGA	10860/247050	0.994	60819	0.794	0.208740	2	0.3333	0	14	0.4071	0	1	0
HIP_60820	CCTSICSL	VALNHLKAT	CCTSICSLVALNHLKAT		IAPP	2643/45018	0.983	60820	0.794	0.039243	2.55	0.1667	2	17	1.0353	1	0	1
HIP_60821	VEALYLVCG	AVLALLLCA	VEALYLVCGAVLALLLCA		CMGA	6351/247050	0.998	60821	0.794	0.015702	2.6833	0.1667	-1	18	2.3556	-1	1	0
HIP_60822	HLVEALYL	EAEKSGEAT	HLVEALYLEAEKSGEAT		CMGA	14415/247050	0.988	60822	0.794	0.091349	0.1833	0	-2	17	-0.1941	-3	1	1
HIP_60823	FVNQHLCGS	QENQMDKAR	FVNQHLCGSQENQMDKAR		SCG1	26430/367830	0.976	60823	0.794	-0.041399	-1.5333	0.3333	1	18	-1.1278	0	0	0
HIP_60824	EQCCTSICS	GRHHRGRGG	EQCCTSICSGRHHRGRGG		SCG1	41105/367830	0.912	60824	0.794	0.125205	-0.3167	0.3333	4	18	-1.0444	2	0	0
HIP_60825	TSICSLYQL	RSEALAVDG	TSICSLYQLRSEALAVDG		CMGA	20939/247050	0.969	60825	0.794	0.201805	-1.6333	0.1667	-1	18	0.3556	-1	1	0
HIP_60826	CSLYQLENY	ARAYGFRGP	CSLYQLENYARAYGFRGP		CMGA	18834/247050	0.976	60826	0.794	-0.032730	-1.5333	0	1	18	-0.5611	1	2	0
HIP_60827	GAGSLQPLA	NPYYDPLQ	GAGSLQPLANPYYDPLQ		SCG1	22461/367830	0.985	60827	0.794	0.017169	-0.4	0.3333	-1	17	-0.4706	-3	2	1
HIP_60828	QVELGGGPG	EEEDNRDSS	QVELGGGPGEEEDNRDSS		CMGA	7417/247050	0.997	60828	0.794	-0.079019	-2.15	0.5	-5	18	-1.6278	-5	0	0
HIP_60829	PLALEGSLQ	PNNQKRERM	PLALEGSLQPNNQKRERM		SCG2	25519/334890	0.993	60829	0.794	-0.016227	-1.5167	0.3333	1	18	-1.2889	1	0	0
HIP_60830	SHLVEALY	SGLTLALSL	SHLVEALYSGLTLALSL		NPY	1568/49410	0.991	60830	0.794	0.159498	1.15	0.3333	0	17	1.1235	-1	1	1
HIP_60831	EQCCTSICS	RVPKLDLKR	EQCCTSICSRVPKLDLKR		SCG1	35735/367830	0.941	60831	0.794	0.088457	0.7167	0.3333	2	18	-0.4111	2	0	0
HIP_60832	GSLQPLALE	IKGYPGVQA	GSLQPLALEIKGYPGVQA		SCG1	21615/367830	0.987	60832	0.794	0.038228	0.3833	0.1667	0	18	0.1556	0	1	0
HIP_60833	SICSLYQLE	PQGAWKELD	SICSLYQLEPQGAWKELD		SCG1	14395/367830	0.995	60833	0.794	-0.059424	-1.45	0.3333	-2	18	-0.3889	-2	1	0
HIP_60834	CCTSICSLY	QELESLSAI	CCTSICSLYQELESLSAI		CMGA	12902/247050	0.991	60834	0.794	-0.088473	-0.25	0.1667	-2	18	0.7778	-2	1	0
HIP_60835	YLVCGERGF	RGSEEYRAP	YLVCGERGFRGSEEYRAP		SCG1	22407/367830	0.986	60835	0.794	0.053246	-1.3	0.5	0	18	-0.8389	0	1	0
HIP_60836	SLQPLALE	SYFPNPYNQ	SLQPLALESYFPNPYNQ		SCG2	25326/334890	0.993	60836	0.794	-0.025605	0.4667	0.1667	-1	17	-0.6176	-1	2	1
HIP_60837	ICSLYQLEN	WMRIILEAL	ICSLYQLENWMRIILEAL		SCG2	23856/334890	0.994	60837	0.794	-0.058280	-1.1167	0	-1	18	0.7444	-1	2	0
HIP_60838	ICSLYQLEN	INDEMKRSG	ICSLYQLENINDEMKRSG		SCG2	29280/334890	0.991	60838	0.794	-0.139132	-0.95	0	-1	18	-0.65	-1	1	0
HIP_60839	GIVEQCCTS	EESWDEEDK	GIVEQCCTSEESWDEEDK		SCG1	17474/367830	0.993	60839	0.794	-0.043420	-1.1333	0.3333	-6	18	-1.2111	-6	1	0
HIP_60840	CCTSICSL	FLVHSSN	CCTSICSLFLVHSSN		IAPP	2182/45018	0.989	60840	0.794	0.046771	2.7167	0.1667	1	15	1.0667	0	1	1
HIP_60841	VEALYLVCG	ALAVDGAGK	VEALYLVCGALAVDGAGK		CMGA	7416/247050	0.997	60841	0.794	0.006557	2.2833	0.1667	-1	18	1.1278	-1	1	0

HIP_61310	HLVEALYLV	VSTHQEAEN	HLVEALYLVVSTHQEAEN		SCG1	13326/367830	0.996	61310	0.794	0.077708
HIP_61311	GPGAGSLQP	DERILSILR	GPGAGSLQPDERILSILR		CMGA	23924/247050	0.956	61311	0.794	-0.034094
HIP_61312	GAGSLQPLA	PGETQNAFL	GAGSLQPLAPGETQNAFL		SCG1	6149/367830	0.999	61312	0.794	0.096330
HIP_61313	SLQPLAL	ASEAHESSS	SLQPLALASEAHESSS		SCG1	19203/367830	0.991	61313	0.794	0.164766
HIP_61314	SHLVEALY	GLPLQVRGY	SHLVEALYGLPLQVRGY		CMGA	8511/247050	0.996	61314	0.794	0.170264
HIP_61315	EQCCTSICS	NQGSSQETD	EQCCTSICSNQGSSQETD		SCG2	9445/334890	0.999	61315	0.794	0.111086
HIP_61316	GERGFFYT	ESSSRGEA	GERGFFYTESSSRGEA		SCG1	23959/367830	0.982	61316	0.794	0.168997
HIP_61317	GERGFFYT	KTGEKPNGS	GERGFFYTKTGEKPNGS		SCG2	27453/334890	0.992	61317	0.794	0.180101
HIP_61318	LVEALYLV	FPSPEMIR	LVEALYLVFPSPEMIR		SCG2	12876/334890	0.998	61318	0.794	0.136686
HIP_61319	GSHLVEALY	NFPMDMSD	GSHLVEALYNFPMDMSD		SCG2	12091/334890	0.998	61319	0.794	0.084272
HIP_61320	VEALYLVC	AVEVLKREP	VEALYLVCAVEVLKREP		IAPP	2836/45018	0.980	61320	0.794	0.036954
HIP_61321	VELGGGPGA	SWDEEDKRN	VELGGGPGASWDEEDKRN		SCG1	7042/367830	0.999	61321	0.794	-0.093673
HIP_61322	VCGERGFFY	IAYEDVVG	VCGERGFFYIAYEDVVG		SCG2	17344/334890	0.997	61322	0.794	-0.051485
HIP_61323	LVEALYLV	THYRASEEE	LVEALYLVTHYRASEEE		SCG1	26958/367830	0.975	61323	0.794	0.041043
HIP_61324	GGGPGAGS	NYDWWEKKP	GGGPGAGSNYDWWEKKP		SCG1	27599/367830	0.973	61324	0.794	0.032016
HIP_61325	GSHLVEALY	HLEEPGETQ	GSHLVEALYHLEEPGETQ		SCG1	22942/367830	0.985	61325	0.794	-0.018179
HIP_61326	GERGFFYTP	QGSSQETD	GERGFFYTPQGSSQETD		SCG2	10261/334890	0.999	61326	0.794	-0.054459
HIP_61327	CGERGFFYT	DDISEADLD	CGERGFFYTDDISEADLD		SCG2	18544/334890	0.997	61327	0.794	-0.014035
HIP_61328	GERGFFYT	EPRAYFMSD	GERGFFYTEPRAYFMSD		SCG1	30313/367830	0.964	61328	0.794	0.129746
HIP_61329	GSLQPLALE	ECFETLRGD	GSLQPLALEECFETLRGD		CMGA	16462/247050	0.983	61329	0.794	-0.070209
HIP_61330	VCGERGFFY	ESKAEGNN	VCGERGFFYESKAEGNN		CMGA	19699/247050	0.973	61330	0.794	0.200458
HIP_61331	LVEALYLV	PGETQNAFL	LVEALYLVPGETQNAFL		SCG1	14260/367830	0.995	61331	0.794	0.186146
HIP_61332	ICSLYQLEN	QEKTHSREK	ICSLYQLENQEKTHSREK		SCG1	50909/367830	0.844	61332	0.794	0.105380
HIP_61333	VGQVELGGG	LALSLLVCL	VGQVELGGGLALSLLVCL		NPY	791/49410	0.996	61333	0.794	0.160677
HIP_61334	EQCCTSICS	KRQYDRVAQ	EQCCTSICSKRQYDRVAQ		SCG1	43674/367830	0.897	61334	0.794	0.138258
HIP_61335	LVEALYLV	LSEEDWMRI	LVEALYLVLSEEDWMRI		SCG2	12982/334890	0.998	61335	0.794	0.115424
HIP_61336	HLCGSHLVE	SGQLGIQEE	HLCGSHLVESGQLGIQEE		SCG2	13836/334890	0.998	61336	0.794	0.145302
HIP_61337	VEALYLVCG	PGEDAPAED	VEALYLVCGPGEDAPAED		NPY	607/49410	0.997	61337	0.794	0.060402
HIP_61338	IVEQCCTSI	PYYDPLQWK	IVEQCCTSIPYYDPLQWK		SCG1	21577/367830	0.987	61338	0.794	-0.010236
HIP_61339	LVEALYLV	GETQNAFLN	LVEALYLVGETQNAFLN		SCG1	15737/367830	0.994	61339	0.794	0.193672
HIP_61340	GERGFFYTP	APGEEEEEE	GERGFFYTPAPGEEEEEE		CMGA	1957/247050	0.999	61340	0.794	-0.173190
HIP_61341	HLCGSHLVE	WPERKLKH	HLCGSHLVEWPERKLKH		SCG2	37071/334890	0.982	61341	0.794	0.141766
HIP_61342	GGPGAGSLQ	VLEYLNQEK	GGPGAGSLQVLEYLNQEK		SCG2	15433/334890	0.998	61342	0.794	-0.052442
HIP_61343	YLVCGERGF	EEEEEGENY	YLVCGERGFEEEEEGENY		SCG1	19539/367830	0.990	61343	0.794	-0.039276
HIP_61344	GGPGAGSLQ	TENENTKFE	GGPGAGSLQTENENTKFE		SCG1	23798/367830	0.983	61344	0.794	-0.128112
HIP_61345	LVEALYLVC	EEQKLYTD	LVEALYLVCEEQKLYTD		SCG2	19862/334890	0.996	61345	0.794	0.138354
HIP_61346	HLVEALYL	PSPEMIRA	HLVEALYLPSPEMIRA		SCG2	17267/334890	0.997	61346	0.794	0.229679
HIP_61347	HLVEALYLV	SQSIYQLI	HLVEALYLVSQSIYQLI		SCG2	18246/334890	0.997	61347	0.794	0.206449
HIP_61348	GSLQPLAL	QYTPQSLA	GSLQPLALQYTPQSLA		SCG2	28312/334890	0.991	61348	0.794	0.201475
HIP_61349	GSLQPLALE	ESEEGEEDA	GSLQPLALEESEEGEEDA		SCG1	16398/367830	0.994	61349	0.794	0.042913
HIP_61350	TSICSLYQL	GELFNPYYD	TSICSLYQLGELFNPYYD		SCG1	8703/367830	0.998	61350	0.794	-0.025418
HIP_61351	HLCGSHLVE	RNYLNYGEE	HLCGSHLVERNYLNYGEE		SCG1	40546/367830	0.915	61351	0.794	0.049151
HIP_61352	HLVEALYL	DDIYKANNI	HLVEALYLDDIYKANNI		SCG2	22239/334890	0.995	61352	0.794	0.120497

HIP_61821	CSLYQLENY	DVSKVIAYL	CSLYQLENYDVSKVIAYL		SCG2	26067/334890	0.993	61821	0.793	-0.091882	-1.4	0.1667	-1	18	0.2889	-1	1	0	0	0.000000	2.7333	0	0.0167	0.000000	0.3333	-0.3333	0	0	0	0	0	0.4667	2.7333	0	1	0	0	0.4769	1.0138	0.969	-0.1667	0.2275	0.9389	-0.6677	0.0003	0.0456
HIP_61822	ERGFFYTPK	DDVSKVIAY	ERGFFYTPKDDVSKVIAY		SCG2	28095/334890	0.992	61822	0.793	-0.017715	-1.5	0.1667	0	18	-0.4778	0	2	0	0	0.000000	1.1333	-3	-0.0667	0.700000	0.3333	0.3667	1	0	1	0	-3.4	-0.55	2.2667	0	0	1	0	0.4654	0.9208	0.9501	-0.3333	0.2166	0.9027	-0.6981	0.0044	-0.0756
HIP_61823	GSLQPLAL	YQLIEISRN	GSLQPLALYQLIEISRN		SCG2	28147/334890	0.992	61823	0.793	0.108281	1.4	0	0	17	0.1529	0	1	1	0	0.000000	-3.4667	0	0.05	0.950000	0.1111	0.8389	0	0	0	0	0	1.1744	-3.4667	1	0	0	0	0.3051	1.2469	0.6745	0.6667	0.0931	0.4549	0.4272	0.0025	-0.1733
HIP_61824	DLQVGQVEL	RSRSNQLPK	DLQVGQVELRSRSNQLPK		SCG2	39325/334890	0.978	61824	0.793	0.163824	-0.8833	0.1667	1	18	-0.9833	1	0	0	0	0.000000	-4.7667	3	0.1167	0.000000	0.3333	-0.3333	0	0	0	0	-14.3	0.2944	0	1	0	0	0	0.4103	1.1188	0.7923	-0.1667	0.1683	0.6278	-0.3624	0.0136	-0.5561
HIP_61825	PLALEGSLQ	EEEPEYGEE	PLALEGSLQEEEPEYGEE		SCG1	18897/367830	0.991	61825	0.793	-0.088056	-1.8333	0.1667	-7	18	-1.1611	-7	0	0	1	0.000000	-3.3333	-3	0.0333	0.950000	0.4444	0.5056	0	0	0	0	10	-0.9269	0	0	1	0	0	0.5333	1.29	0.8167	-0.3333	0.2844	0.6669	-0.9778	0.0011	-0.1111
HIP_61826	HLVEALYL	NKPYALNS	HLVEALYLNKPYALNS		SCG2	28467/334890	0.991	61826	0.793	0.224766	-0.45	0.1667	1	16	0.025	0	2	0	0	0.000000	-5.1	1	0.1667	0.000000	0.2222	-0.2222	1	0	1	0	-5.1	0.1	-10.2	1	0	0	0	0.4269	0.9805	0.9823	0.1667	0.1823	0.9649	-0.1921	0.0278	-0.85
HIP_61827	SLYQLENYC	LEALRQAEN	SLYQLENYCLEALRQAEN		SCG2	19389/334890	0.996	61827	0.793	-0.168696	-0.0333	0	-2	18	-0.6167	-2	1	0	0	0.000000	1.4667	-1	0	2.850000	0.2222	2.6278	0	0	0	0	-1.4667	-0.0876	1.4667	0	0	0	0	0.4103	1.1622	0.879	0.1667	0.1683	0.7726	-0.0137	0	0
HIP_61828	GPGAGSLQP	NNFGAILSS	GPGAGSLQPNNFGAILSS		IAPP	2694/45018	0.982	61828	0.793	-0.035362	-0.9167	0.1667	0	18	0.0444	0	1	0	0	0.000000	-0.9667	0	-0.0167	0.000000	0	0	1	0	1	0	0	0	-0.9667	0	0	0	1	0.4833	0.92	1.12	0.3333	0.2336	1.2544	-0.4431	0.0003	0.0161
HIP_61829	VCGERGFF	NRMLSKSGY	VCGERGFFNRMLSKSGY		SCG2	33680/334890	0.986	61829	0.793	0.087071	-0.15	0.1667	2	17	-0.3529	2	2	1	0	0.000000	-3.7667	1	0.0167	0.700000	0.2222	0.4778	0	1	0	1	-3.7667	-0.0717	-7.5333	1	0	0	0	0.3885	0.9653	0.9612	0.3333	0.1509	0.9238	-0.0583	0.0003	-0.0628
HIP_61830	CCTSICSLY	HESSSRGEA	CCTSICSLYHESSSRGEA		SCG1	27277/367830	0.974	61830	0.793	-0.018298	-0.9667	0.3333	0	18	-0.1944	-1	1	0	1	0.000000	-3.0667	-1	0.05	2.075000	0.1111	1.9639	0	0	0	0	3.0667	-1.8984	-3.0667	0	1	0	0	0.4769	1.0755	0.909	-0.1667	0.2275	0.8262	-0.461	0.0025	-0.1533
HIP_61831	AEDLQVGQV	LALEGSLQ	AEDLQVGQVLALEGSLQ		INSC	56/5184	0.999	61831	0.793	0.110735	1.6167	0.1667	-3	17	0.2706	-3	0	1	0	1.000000	3.0333	0	-0.1333	0.950000	0.1111	0.8389	0	1	0	1	0	1.3562	0	1	0	0	0	0.35	1.12	0.7733	0.6667	0.1225	0.598	0.5658	0.0178	-0.4044
HIP_61832	HLCGSHLVE	LQDTKENRE	HLCGSHLVELQDTKENRE		SCG1	31958/367830	0.958	61832	0.793	-0.000251	0.2167	0	0	18	-1.0889	-2	0	0	0	0.000000	-2.5667	0	0.05	2.000000	0.2222	1.7778	0	0	0	0	0	0.3852	0	0	0	1	0	0.3718	1.2169	0.6878	0.1667	0.1382	0.4731	0.0806	0.0025	-0.1283
HIP_61833	GERGFFYT	VSVPLQQKE	GERGFFYTVSVPLQQKE		SCG2	19130/334890	0.996	61833	0.793	-0.082863	1.4	0.1667	0	17	-0.5765	0	3	1	0	0.000000	2.2667	0	0.0167	1.650000	0	1.65	0	0	0	0	0	2.31	6.8	0	1	0	0	0.3718	0.9953	0.7778	0.5	0.1382	0.605	0.5205	0.0003	0.0378
HIP_61834	ERGFFYT	GEEEEEE	ERGFFYTGEEEEEE		CMGA	11161/247050	0.993	61834	0.793	0.091963	-1.1	0.1667	-6	14	-1.8714	-6	3	0	4	0.000000	-2.7333	-2	0.1333	0.000000	0.3333	-0.3333	0	1	0	1	5.4667	0.3667	-8.2	0	1	0	0	0.5218	1.0953	0.9078	-0.1667	0.2723	0.8241	-0.574	0.0178	-0.3644
HIP_61835	LVEALYLV	GAILSSTNV	LVEALYLVGAILSSTNV		IAPP	1784/45018	0.992	61835	0.793	0.021680	2.1	0.1667	-1	17	1.4647	-1	1	1	0	0.000000	-0.2667	0	0.0833	2.450000	0	2.45	0	1	0	1	0	5.145	-0.2667	1	0	0	0	0.3218	1.0686	0.7562	0.6667	0.1036	0.5718	0.6758	0.0069	-0.0222
HIP_61836	LYQLENYCN	REDEEEEEG	LYQLENYCNREDEEEEEG		SCG1	21111/367830	0.988	61836	0.793	0.031603	-2.3	0	-7	18	-1.9944	-7	1	0	3	0.000000	-3.0667	-1	0.1	0.625000	0.5556	0.0694	0	0	0	0	3.0667	-0.1597	-3.0667	0	1	0	0	0.4872	1.0444	0.9879	-0.5	0.2373	0.976	-1.1205	0.01	-0.3067
HIP_61837	GERGFFYTP	PGETQNAF	GERGFFYTPPGETQNAF		SCG1	14994/367830	0.995	61837	0.793	-0.028159	-1.5167	0.5	-1	17	-0.9059	-1	3	1	0	0.000000	-0.6333	-1	0.0833	0.700000	0.1111	0.5889	2	1	1	1	0.6333	-0.8931	-1.9	0	0	0	1	0.6218	0.8336	1.1912	-0.1667	0.3866	1.4188	-0.9431	0.0069	-0.0528
HIP_61838	LGGGPGAGS	AWIPHVENR	LGGGPGAGSAWIPHVENR		SCG2	7635/334890	0.999	61838	0.793	0.138494	1	0.3333	1	18	-0.3056	0	1	0	1	0.000000	1.6	0	-0.15	0.450000	0	0.45	0	1	0	1	0	0.45	1.6	0	1	0	0	0.3885	1.0503	0.8995	0.6667	0.1509	0.8091	0.3885	0.0225	-0.24
HIP_61839	LGGGPGAGS	YYDPLQWKS	LGGGPGAGSYYDPLQWKS		SCG1	20489/367830	0.989	61839	0.793	0.059141	-0.9167	0.3333	0	18	-0.6556	0	2	0	1	0.000000	-2.2333	-1	-0.1	0.000000	0.1111	-0.1111	0	1	0	1	2.2333	0.1019	-4.4667	0	1	0	0	0.4654	0.9691	1.0135	0	0.2166	1.0271	-0.4266	0.01	0.2233
HIP_61840	NQHLCGSHL	EDSKEAEKS	NQHLCGSHLEDSKEAEKS		CMGA	17220/247050	0.981	61840	0.793	0.101033	-1.3333	0.3333	0	18	-1.45	-2	0	0	0	0.000000	-2.5333	-2	0.1	0.625000	0.3333	0.2917	0	0	0	0	5.0667	-0.3889	0	1	0	0	0	0.4769	1.0755	0.909	-0.3333	0.2275	0.8262	-0.6359	0.01	-0.2533
HIP_61841	ALYLVCGER	PYYDPLQWK	ALYLVCGERPYYDPLQWK		SCG1	16369/367830	0.994	61841	0.793	0.011424	-2.1	0.3333	0	18	-0.4111	0	2	0	0	0.000000	1.4	0	-0.1167	1.050000	0.3333	0.7167	1	1	1	1	0	-1.505	2.8	0	0	1	0	0.5487	0.9408	1.0801	-0.3333	0.3011	1.1667	-1.1523	0.0136	-0.1633
HIP_61842	GERGFFYT	GQPRSQEE	GERGFFYTGQPRSQEE		SCG1	19560/367830	0.990	61842	0.793	0.160159	-0.7833	0.3333	-1	16	-1.6562	-1	3	0	0	0.000000	-2.1	0	0.15	0.000000	0.1111	-0.1111	1	1	1	1	0	0.087	-6.3	0	1	0	0	0.5385	0.8719	1.0778	0.1667	0.2899	1.1617	-0.4218	0.0225	-0.315
HIP_61843	SHLVEALYL	YRASEEEP	SHLVEALYLYRASEEEP		SCG1	33266/367830	0.952	61843	0.793	0.094959	0.3833	0	-2	17	-0.4882	-3	2	1	1	0.000000	-3.4333	1	0.05	0.000000	0.2222	-0.2222	0	0	0	0	-3.4333	-0.0852	-6.8667	1	0	0	0	0.3154	1.1858	0.7301	0.3333	0.0995	0.5331	0.1209	0.0025	-0.1717
HIP_61844	YLVCGERGF	HIAKRAMEN	YLVCGERGFHIAKRAMEN		SCG2	19675/334890	0.996	61844	0.793	0.171605	0.1667	0.1667	2	18	-0.3	1	1	0	0	0.000000	1.7333	-1	-0.1333	0.000000	0.3333	-0.3333	0	1	0	1	-1.7333	-0.0556	1.7333	1	0	0	0	0.3603	1.0455	0.8406	0.1667	0.1298	0.7067	0.06	0.0178	-0.2311
HIP_61845	LVEALYLVC	TLALSLLVC	LVEALYLVCTLALSLLVC		NPY	2947/49410	0.976	61845	0.793	0.021489	2.5667	0	-1	18	2.3056	-1	1	0	0	0.000000	-1.8667	0	0.05	2.950000	0	2.95	0	0	0	0	0	7.5717	-1.8667	0	0	0	0	0.3051	1.1603	0.6712	0.6667	0.0931	0.4504	0.7832	0.0025	-0.0933
HIP_61846	SHLVEALY	DQELGEYLA	SHLVEALYDQELGEYLA		SCG2	20605/334890	0.996	61846	0.793	0.167597	-1.0333	0	-3	17	-0.0882	-4	1	1	0	0.000000	-4.9333	-2	0.15	0.000000	0.3333	-0.3333	0	0	0	0	9.8667	0.3444	-4.9333	0	1	0	0	0.4103	1.2355	0.7823	0	0.1683	0.612	-0.4239	0.0225	-0.74
HIP_61847	RGFFYTPKT	NDKDQELGE	RGFFYTPKTNDKDQELGE		SCG2	31341/334890	0.989	61847	0.793	-0.024406	-2.85	0.1667	-1	18	-1.6111	-1	1	0	0	0.000000	-1.5667	-1	-0.0167	0.000000	0.4444	-0.4444	1	0	1	0	1.5667	1.2667	-1.5667	0	1	0	0	0.5321	0.8558	1.1268	-0.5	0.2831	1.2697	-1.5163	0.0003	0.0261
HIP_61848	CGERGFFYT	APGKWQQQ	CGERGFFYTAPGKWQQQ		SCG1	15072/367830	0.995	61848	0.793	0.081419	0.1	0.3333	1	17	-1.0706	1	3	1	1	0.000000	-0.3333	0	0.1	0.450000	0.1111	0.3389	1	1	1	1	0	0.0339	-1	0	1	0	0	0.4718	0.9286	1.0245	0.3333	0.2226	1.0496	0.0472	0.01	-0.0333
HIP_61849	CCTSICSLY	GQPRSQEES	CCTSICSLYGQPRSQEES		SCG1	10702/367830	0.997	61849	0.793	0.090414	-0.6333	0.5	-1	18	-0.55	-1	1	0	0	0.000000	-2.4	0	0.1333	2.075000	0.1111	1.9639	1	1	1	1	0	-1.2438	-2.4	0	1	0	0	0.5385	0.8836	1.1012	0.1667	0.2899	1.2125	-0.341	0.0178	-0.32
HIP_61850	HLVEALYLV	GKGEQEHS	HLVEALYLVGKGEQEHS		CMGA	13962/247050	0.989	61850	0.793	0.218683	0.3333	0.3333	0	17	-0.3294	-2	1	1	0	0.000000	-3.8	1	0.2167	1.400000	0.2222	1.1778	0	2	0	1	-3.8	0.3926	-3.8	1	0	0	0	0.4436	0.9005	0.969	0.1667	0.1968	0.9389	0.1479	0.0469	-0.8233
HIP_61851	QHLCGSHLV	DHPDLFQNR	QHLCGSHLVDHPDLFQNR		SCG2	24360/334890	0.994	61851	0.793	0.174850	-0.5833	0.1667	2	18	-0.75	-1	0	0	0	0.000000	-4.3667	-1	0.1167	0.950000	0.2222	0.7278	1	0	1	0	4.3667	-0.4245	0	1	0	0	0	0.3987	0.9991	0.8735	-0.1667	0.159	0.7629	-0.2326	0.0136	-0.5094
HIP_61852	SICSLYQLE	GPTKADTEK	SICSLYQLEGPTKADTEK		SCG1	11318/367830	0.997	61852	0.793	-0.057613	-0.9833	0.3333	-1	18	-0.65	-1	1	0	0	0.000000	0.1667	1	0.1	1.900000	0.2222	1.6778	1	1	1	1	0.1667	-1.6498	0.1667	0	0	1	0	0.5667	0.97	1.0333	0	0.3211	1.0678	-0.5572	0.01	0.0167
HIP_61853	EQCCTSICS	KLAPVSKRF	EQCCTSICSKLAPVSKRF		SCG2	34178/334890	0.986	61853	0.793	0.046587	1.3167	0.1667	2	18	0.0333	2	0	0	0	0.000000	-1.5	1	-0.0167	0.625000	0.1111	0.5139	0	0	0	0	-1.5	0.6766	0	0	1	0	0	0.3436	1.1122	0.774	0.3333	0.1181	0.599	0.4524	0.0003	0.025
HIP_61854	VEALYLVCG	MDKARRHPQ	VEALYLVCGMDKARRHPQ		SCG1	34206/367830	0.948	61854	0.793	0.047487	0.1333	0.1667	2	18	-0.3278	1	1	0	0	0.000000	-3.9333	0	0.0167	1.550000	0.2222	1.3278	0	1	0	1	0	0.177	-3.9333	0	0	0	1	0.3987	0.9708	0.8868	0	0.159	0.7864	0.0532	0.0003	-0.0656
HIP_61855	CTSICSLYQ	LPSQKYPGP	CTSICSLYQLPSQKYPGP		CMGA	11503/247050	0.993	61855	0.793	-0.022371	0.0667	0.3333	1	18	-0.2611	1	1	0	0	0.000000	0.8	0	0.0833	1.575000	0	1.575	1	0	1	0	0	0.105	0.8	0	1	0	0	0.4551	1.0169	0.9345	0.3333	0.2071	0.8733	0.0303	0.0069	0.0667
HIP_61856	GAGSLQPLA	PEPMQESKA	GAGSLQPLAPEPMQESKA		CMGA	7096/247050	0.997	61856	0.793	0.052172	-0.45	0.5	-1	18	-0.5667	-1	0	0	0	0.000000	-3.5667	-1	0.1167	2.375000	0.1111	2.2639	3	0	1	0	3.5667	-1.0188	0	1	0	0	0	0.5167	0.995	1.0767	0.1667	0.2669	1.1592	-0.2325	0.0136	-0.4161
HIP_61857	SHLVEALY	DKETTENEN	SHLVEALYDKETTENEN		SCG1	27185/367830	0.974	61857	0.793	0.199491	-1.1	0	-3	17	-1.2647	-4	1	1	0	0.000000	-5.0667	-1	0.15	0.000000	0.4444	-0.4444	0	0	0	0	5.0667	0.4889	-5.0667	0	1	0	0	0.3821	1.2208	0.7701	-0.1667	0.146	0.5931	-0.4203	0.0225	-0.76
HIP_61858	GERGFFYT	QGSSQETD	GERGFFYTQGSSQETD		SCG2	16314/334890	0.997	61858	0.793	0.158158	-0.65	0.3333	-2	16	-1.3688	-2	3	0	0	0.000000	-1.8333	0	0.15	0.000000	0	0	0	1	0	1	0	0	-5.5	0	1	0	0	0.5218	0.9103	1.0245	0.1667	0.2723	1.0496	-0.3392	0.0225	-0.275
HIP_61859	LYLVCGERG	PERELDLPV	LYLVCGERGPERELDLPV		SCG2	25676/334890	0.993	61859	0.793	-0.096889	-3	0.3333	-2	18	-0.1222	-2	0	0	0	0.000000	-0.4	0	-0.0167	0.625000	0.5556	0.0694	1	1	1	1	0	-0.2083	0	0	0	0	1	0.5769	1.0222	1.0523	-0.6667	0.3328	1.1074	-1.7308	0.0003	0.0067
HIP_61860	GSLQPLALE	YDSEEPVST	GSLQPLALEYDSEEPVST		SCG1	23486/367830	0.983	61860	0.793	0.022566	-0.5833	0.1667	-4	18	-0.45	-4	1	0	0	0.000000	-2.5667	0	0.0667	0.950000	0.3333	0.6167	0	0	0	0	0	-0.3597	-2.5667	0	0	1	0	0.4103	1.1838	0.819	0	0.1683	0.6707	-0.2393	0.0044	-0.1711
HIP_61861	VCGERGFF	QREDEEEE	VCGERGFFQREDEEEE		SCG1	25817/367830	0.978	61861	0.793	0.064802	-1.05	0.1667	-5	16	-1.5938	-5	2	0	2	0.000000	-5.5667	0	0.0667	0.700000	0.5556	0.1444	0	1	0	1	0	-0.1517	-11.1333	1	0	0	0	0.4551	1.0903	0.8878	0	0.2071	0.7882	-0.4779	0.0044	-0.3711
HIP_61862	HLCGSHLVE	PPEDLIEML	HLCGSHLVEPPEDLIEML		SCG2	13151/334890	0.998	61862	0.793	0.135920	-0.3667	0.3333	-2	18	0.1944	-4	0	0	0	0.000000	-3.7333	0	0.1333	1.050000	0.3333	0.7167	2	0	1	0	0	-0.2628	0	0	0	1	0	0.5	1.0867	0.92	0	0.25	0.8464	-0.1833	0.0178	-0.4978
HIP_61863	VNQHLCGSH	PERDSLSEE	VNQHLCGSHPERDSLSEE		SCG2	21775/334890	0.995	61863	0.793	-0.126294	-2.3333	0.5	-1	18	-1.2222	-3	0	0	0	0.000000	-1.7333	0	0.0167	0.950000	0.3333	0.6167	1	1	1	1	0	-1.4389	0	0	0	1	0	0.5769	0.9038	1.129	-0.5	0.3328	1.2746	-1.3462	0.0003	-0.0289
HIP_61864	CGERGFFY	ELDLPVQLD	CGERGFFYELDLPVQLD		SCG2	20309/334890	0.996	61864	0.793	0.082856	0.1833	0	-4	17	-0.1176	-4	3	1	0	0.000000	-2.5	-2	0.1	0.950000	0.2222	0.7278	0	0	0	0	5	0.1334	-7.5	0	1	0	0	0.3436	1.2088	0.709	0.1667	0.1181	0.5026	0.063	0.01	-0.25
HIP_61865	VNQHLCGSH	GEGHHRVQE	VNQHLCGSHGEGHHRVQE		SCG1	20194/367830	0.989	61865	0.793	-0.050019	-1.45	0.6667	3	18	-1.2278	-1	0	0	0	0.000000	0.0333	-1	0.0667	0.950000	0.1111	0.8389	0	3	0	1	-0.0333	-1.2164	0	0	0	1	0	0.6218	0.8053	1.2245	-0.3333	0.3866	1.4994	-0.9016	0.0044	0.0022
HIP_61866	GGGPGAGSL	NGSVEPERE	GGGPGAGSLNGSVEPERE		SCG2	4621/334890	1.000	61866	0.793	-0.081728	-0.35	0.6667	-2	18	-0.8833	-2	0	0	1	0.000000	-2.4333	0	0.05	1.050000	0	1.05	0	2	0	1	0	-0.3675	0	1	0	0	0	0.5667	0.7617	1.2433	0.1667	0.3211	1.5459	-0.1983	0.0025	-0.1217
HIP_61867	LYLVCGERG	REEARFQDK	LYLVCGERGREEARFQDK		SCG1	40608/367830	0.915	61867	0.793	-0.055983	-3.3167	0.1667	0	18	-1.0056	0	0	0	0	0.000000	-1.0333	-1	-0.0333	1.075000	0.5556	0.5194	0	1	0	1	1.0333	-1.7228	0	0	0	0	1	0.5603	1.1788	0.9223	-0.8333	0.3139	0.8507	-1.8582	0.0011	0.0344
HIP_61868	NQHLCGSHL	FPMDMSDQY	NQHLCGSHLFPMDMSDQY		SCG2	7512/334890	0.999	61868	0.793	-0.149920	0.4833	0.3333	-1	18	-0.6722	-3	1	0	0	1.000000	1.1	0	0	1.325000	0.1111	1.2139	1	0	1	0	0	0.5867	1.1	1	0	0	0	0.3885	1.0153	0.8878	0.3333	0.1509	0.7882	0.1878	0	0

HIP_62332	PLALEGSLQ	QELGKLTGP	PLALEGSLQQELGKLTGP		SCG2	24844/334890	0.994	62332	0.793	-0.118306	-0.6167	0.1667	-1	18	-0.1667	-1	0	0
HIP_62333	HLVEALYL	LQNGQNGER	HLVEALYLLQNGQNGER		SCG2	23570/334890	0.994	62333	0.793	0.168402	0.5167	0	0	17	-0.5647	-1	1	1
HIP_62334	ERGFFYTPK	EEGENYQK	ERGFFYTPKEEGENYQK		SCG1	28382/367830	0.971	62334	0.793	-0.081281	-2.2667	0.3333	-1	17	-1.9647	-1	2	1
HIP_62335	ALYLVCGER	ESSSRGEAG	ALYLVCGERESSSRGEAG		SCG1	28044/367830	0.972	62335	0.793	-0.119333	-2.25	0.5	-1	18	-0.3611	-1	0	0
HIP_62336	VCGERGFF	EEMAVVPQG	VCGERGFFEEMAVVPQG		CMGA	14988/247050	0.987	62336	0.793	-0.002511	0.0167	0.1667	-2	17	0.1824	-2	2	1
HIP_62337	GPGAGSLQP	YLKRLVNAA	GPGAGSLQPYLKRLVNAA		SCG2	29333/334890	0.991	62337	0.793	0.078518	-0.45	0.1667	2	18	-0.05	2	1	0
HIP_62338	GIVEQCCT	PQGLFRGGK	GIVEQCCTPQGLFRGGK		CMGA	13783/247050	0.989	62338	0.793	0.191010	-0.2	0.3333	1	17	-0.1471	1	1	1
HIP_62339	EDLQVGQVE	FFPEYNYDW	EDLQVGQVEFFPEYNYDW		SCG1	7999/367830	0.998	62339	0.793	-0.178097	0.2	0.1667	-5	18	-0.8722	-5	2	0
HIP_62340	VCGERGFFY	THWLGAAL	VCGERGFFYTHWLGAAL		SCG2	29802/334890	0.990	62340	0.793	0.069590	-0.0833	0	1	17	0.4824	0	4	1
HIP_62341	GPGAGSLQP	YETQQWPER	GPGAGSLQPYETQQWPER		SCG2	25058/334890	0.994	62341	0.793	-0.054457	-1.1333	0.1667	-1	18	-1.45	-1	1	0
HIP_62342	VCGERGFF	EATDGARPQ	VCGERGFFEATDGARPQ		CMGA	15661/247050	0.985	62342	0.793	0.016559	0.4667	0.1667	-1	17	-0.6235	-1	2	1
HIP_62343	LCGSHLVEA	VALNHLKA	LCGSHLVEAVALNHLKA		IAPP	3003/45018	0.978	62343	0.793	0.047944	2.05	0	2	17	0.7647	0	0	1
HIP_62344	CTSICSLY	EARFQDKQY	CTSICSLYEARFQDKQY		SCG1	39327/367830	0.922	62344	0.793	0.060495	-0.75	0.1667	0	17	-0.5529	0	2	1
HIP_62345	GSHLVEALY	AYGFRGPG	GSHLVEALYAYGFRGPG		CMGA	13938/247050	0.989	62345	0.793	0.042969	0.7333	0.1667	1	17	0.0235	0	3	1
HIP_62346	VCGERGFF	QKLYTDDED	VCGERGFFQKLYTDDED		SCG2	25191/334890	0.994	62346	0.793	0.028712	0.2667	0.1667	-3	17	-0.9471	-3	3	1
HIP_62347	SHLVEALY	DDIYKANNI	SHLVEALYDDIYKANNI		SCG2	25571/334890	0.993	62347	0.793	0.145356	0.3	0	-1	17	-0.2118	-2	2	1
HIP_62348	LVCGERGF	WGYEKRNLA	LVCGERGFWGYEKRNLA		SCG1	19280/367830	0.990	62348	0.793	-0.011487	-0.7833	0.3333	1	17	-0.4647	1	3	1
HIP_62349	LVCGERGFF	LEGQEEEED	LVCGERGFFLEGQEEEED		CMGA	2850/247050	0.999	62349	0.793	-0.066480	0.85	0.3333	-6	18	-0.7667	-6	2	0
HIP_62350	LVEALYLVC	KKELENLAA	LVEALYLVCKKELENLAA		SCG1	33545/367830	0.951	62350	0.793	0.184627	-0.1333	0	-1	18	0.6778	-1	1	0
HIP_62351	SLYQLENYC	VLALLLCAG	SLYQLENYCVLALLLCAG		CMGA	10280/247050	0.994	62351	0.793	-0.052408	1.25	0	-1	18	1.1833	-1	1	0
HIP_62352	GERGFFYT	PQEESEES	GERGFFYTPQEESEES		SCG1	23143/367830	0.984	62352	0.793	0.103149	-1.3	0.1667	-4	16	-1.6188	-4	3	0
HIP_62353	LQPLALEGS	KEEEGSANR	LQPLALEGSKEEEGSANR		CMGA	21014/247050	0.968	62353	0.793	-0.066799	-2.6	0.3333	-2	18	-1.0222	-2	0	0
HIP_62354	VEQCCTSIC	LVARSETHA	VEQCCTSICLVARSETHA		SCG1	19354/367830	0.990	62354	0.793	0.014351	2.6667	0.1667	0	18	0.3667	-1	0	0
HIP_62355	EDLQVGQVE	AYPSKPDNP	EDLQVGQVEAYPSKPDNP		NPY	1834/49410	0.989	62355	0.793	-0.200463	-0.65	0.1667	-3	18	-1.2056	-3	1	0
HIP_62356	ICSLYQLE	EEERLSKEW	ICSLYQLEEEERLSKEW		CMGA	22267/247050	0.963	62356	0.793	-0.004065	-2.2833	0	-3	17	-0.8706	-3	1	1
HIP_62357	GPGAGSLQP	IAYLKRLVN	GPGAGSLQPIAYLKRLVN		SCG2	14094/334890	0.998	62357	0.793	0.078374	0.6167	0.1667	2	18	0.1	2	1	0
HIP_62358	TSICSLYQL	PASLPSQKY	TSICSLYQLPASLPSQKY		CMGA	12892/247050	0.991	62358	0.793	0.037086	-0.2667	0.3333	1	18	-0.0222	1	1	0
HIP_62359	GAGSLQPLA	FPSPEMIRA	GAGSLQPLAFPSPEMIRA		SCG2	10578/334890	0.999	62359	0.793	-0.024742	0.7333	0.5	0	18	0.1944	0	1	0
HIP_62360	HLVEALYL	TRQRYGKR	HLVEALYLTRQRYGKR		NPY	5531/49410	0.917	62360	0.793	0.269406	-0.4	0	4	16	-0.8688	3	1	0
HIP_62361	CSLYQLENY	QQKKHSGFE	CSLYQLENYQQKKHSGFE		CMGA	31777/247050	0.897	62361	0.793	0.047979	-3.2	0	1	18	-1.3167	0	1	0
HIP_62362	SLQPLAL	EEVKTRH	SLQPLALEEVKTRH		SCG1	27348/367830	0.974	62362	0.793	0.138596	1.1	0	1	14	-0.5571	0	0	0
HIP_62363	EDLQVGQVE	RGGEPRAYF	EDLQVGQVERGGEPRAYF		SCG1	17016/367830	0.993	62363	0.793	0.005382	-1.35	0.3333	-2	18	-0.9611	-2	0	0
HIP_62364	ERGFFYTPK	LFEKPLDSQ	ERGFFYTPKLFEKPLDSQ		SCG2	21458/334890	0.995	62364	0.793	0.003414	-0.5167	0.1667	0	18	-0.9278	0	3	0
HIP_62365	CTSICSLYQ	KTHSREKSS	CTSICSLYQKTHSREKSS		SCG1	53159/367830	0.825	62365	0.793	0.112622	-1.4667	0	3	18	-0.8833	2	1	0
HIP_62366	CSLYQLENY	LMRESTENV	CSLYQLENYLMRESTENV		NPY	2609/49410	0.981	62366	0.793	-0.078986	-1.1833	0	-2	18	-0.5778	-2	1	0
HIP_62367	LYLVCGERG	QGSSQETDK	LYLVCGERGQGSSQETDK		SCG2	19586/334890	0.996	62367	0.793	-0.119097	-2.1833	0.5	-1	18	-0.9111	-1	0	0
HIP_62368	DLQVGQVEL	IEMLKTGEK	DLQVGQVELIEMLKTGEK		SCG2	22117/334890	0.995	62368	0.793	-0.046740	1.2333	0	-2	18	-0.2278	-2	0	0
HIP_62369	SHLVEALY	GLSAEPGWQ	SHLVEALYGLSAEPGWQ		CMGA	7007/247050	0.997	62369	0.793	0.153850	1.15	0.3333	-1	17	-0.0412	-2	1	1
HIP_62370	LYQLENYCN	LQVFLIVLS	LYQLENYCNLQVFLIVLS		IAPP	2176/45018	0.989	62370	0.793	-0.133698	0.3667	0	-1	18	0.9056	-1	2	0
HIP_62371	PGAGSLQPL	ETQQWPERK	PGAGSLQPLETQQWPERK		SCG2	29499/334890	0.991	62371	0.793	-0.004579	-1.5	0.1667	0	18	-1.3611	0	0	0
HIP_62372	ERGFFYTP	GWQAKREEE	ERGFFYTPGWQAKREEE		CMGA	10999/247050	0.994	62372	0.793	-0.101379	-1.4	0.3333	-1	17	-1.6647	-1	3	1
HIP_62373	PLALEGSLQ	WDEEDKRNY	PLALEGSLQWDEEDKRNY		SCG1	24869/367830	0.980	62373	0.793	-0.078219	-1.4	0.1667	-3	18	-1.3722	-3	1	0
HIP_62374	LVEALYL	EDVNWGYEK	LVEALYLEDVNWGYEK		SCG1	22614/367830	0.985	62374	0.793	0.102587	0.5833	0	-3	16	-0.2313	-3	2	2
HIP_62375	SICSLYQL	WAEGGGHSR	SICSLYQLWAEGGGHSR		SCG1	30630/367830	0.963	62375	0.793	-0.060308	-0.6	0	1	17	-0.2412	0	2	1
HIP_62376	LYQLENYCN	GRGSEEYRA	LYQLENYCNGRGSEEYRA		SCG1	16974/367830	0.993	62376	0.793	0.145620	-1.2667	0.3333	-1	18	-1.3111	-1	1	0
HIP_62377	GSHLVEALY	MESAANQK	GSHLVEALYMESAANQK		SCG2	18866/334890	0.996	62377	0.793	0.112956	0.3167	0.1667	0	17	-0.3118	-1	1	1
HIP_62378	CGERGFFY	LAKELTAEK	CGERGFFYLAKELTAEK		CMGA	21020/247050	0.968	62378	0.793	0.053531	1	0	0	17	-0.3706	0	3	1
HIP_62379	HLVEALYLV	HHTAEKRKR	HLVEALYLVHHTAEKRKR		SCG1	43281/367830	0.899	62379	0.793	0.116429	-0.0667	0	5	18	-0.6667	2	1	0

HIP_62843	PLALEGSLQ	AYFMSDTRE	PLALEGSLQAYFMSDTRE		SCG1	8059/367830	0.998	62843	0.792	0.020179
HIP_62844	ERGFFYTPK	PVGPPKNDD	ERGFFYTPKPVGPPKNDD		SCG2	16792/334890	0.997	62844	0.792	-0.117720
HIP_62845	VEALYLVC	DQLAKELTA	VEALYLVCDQLAKELTA		CMGA	16460/247050	0.983	62845	0.792	0.157186
HIP_62846	FVNQHLCGS	DSEEPVSTH	FVNQHLCGSDSEEPVSTH		SCG1	18056/367830	0.992	62846	0.792	-0.067336
HIP_62847	EQCCTSICS	EEKGHPQEE	EQCCTSICSEEKGHPQEE		SCG1	24375/367830	0.982	62847	0.792	0.064626
HIP_62848	LVCGERGFF	KHSGFEDEL	LVCGERGFFKHSGFEDEL		CMGA	21590/247050	0.966	62848	0.792	0.058353
HIP_62849	GERGFFYTP	EHLNQGSSQ	GERGFFYTPEHLNQGSSQ		SCG2	21097/334890	0.996	62849	0.792	-0.054831
HIP_62850	VCGERGFFY	AEKSGEAT	VCGERGFFYAEKSGEAT		CMGA	22453/247050	0.963	62850	0.792	0.131646
HIP_62851	PLALEGSLQ	HQQKKHSGF	PLALEGSLQHQQKKHSGF		CMGA	32228/247050	0.893	62851	0.792	-0.022234
HIP_62852	VEALYLVC	QAEGDSEGL	VEALYLVCQAEGDSEGL		CMGA	14107/247050	0.989	62852	0.792	0.118289
HIP_62853	VEQCCTSIC	AYFMSDTRE	VEQCCTSICAYFMSDTRE		SCG1	16429/367830	0.994	62853	0.792	-0.021771
HIP_62854	DLQVGQVEL	EEIKGYPGV	DLQVGQVELEEIKGYPGV		SCG1	29178/367830	0.968	62854	0.792	0.030904
HIP_62855	GSLQPLAL	QELESLSAI	GSLQPLALQELESLSAI		CMGA	14306/247050	0.988	62855	0.792	0.131809
HIP_62856	IVEQCCTSI	LLMRESTEN	IVEQCCTSILLMRESTEN		NPY	1311/49410	0.993	62856	0.792	0.084055
HIP_62857	QHLCGSHLV	GGKSGELEQ	QHLCGSHLVGGKSGELEQ		CMGA	14417/247050	0.988	62857	0.792	0.217160
HIP_62858	SICSLYQLE	QGSSQETDK	SICSLYQLEQGSSQETDK		SCG2	14245/334890	0.998	62858	0.792	-0.042210
HIP_62859	TSICSLYQL	KRVPGQGSS	TSICSLYQLKRVPGQGSS		SCG2	46637/334890	0.957	62859	0.792	0.135460
HIP_62860	HLVEALYL	AEGGGHSR	HLVEALYLAEGGGHSR		SCG1	19921/367830	0.990	62860	0.792	0.210145
HIP_62861	CGSHLVEAL	GRSRPQRSS	CGSHLVEALGRSRPQRSS		SCG1	30587/367830	0.963	62861	0.792	0.198035
HIP_62862	EQCCTSICS	YSALRHYIN	EQCCTSICSYSALRHYIN		NPY	3359/49410	0.970	62862	0.792	-0.013315
HIP_62863	ERGFFYTP	EYGEEIKGY	ERGFFYTPEYGEEIKGY		SCG1	17932/367830	0.992	62863	0.792	-0.107274
HIP_62864	HLVEALYL	EEERGLEP	HLVEALYLEEERGLEP		SCG1	23314/367830	0.984	62864	0.792	0.149487
HIP_62865	LVCGERGFF	SILRHQNL	LVCGERGFFSILRHQNL		CMGA	13349/247050	0.990	62865	0.792	-0.004934
HIP_62866	EAEDLQVGQ	YNYDWWEKK	EAEDLQVGQYNYDWWEKK		SCG1	16119/367830	0.994	62866	0.792	-0.040457
HIP_62867	DLQVGQVEL	DLQDTKENR	DLQVGQVELDLQDTKENR		SCG1	30502/367830	0.964	62867	0.792	0.042658
HIP_62868	GPGAGSLQP	GEDWNPVEE	GPGAGSLQPGEDWNPVEE		SCG2	10788/334890	0.999	62868	0.792	-0.031162
HIP_62869	LQPLALEGS	NWGYEKRNL	LQPLALEGSNWGYEKRNL		SCG1	15275/367830	0.995	62869	0.792	-0.058616
HIP_62870	HLVEALYL	HLEEPGET	HLVEALYLHLEEPGET		SCG1	26457/367830	0.976	62870	0.792	0.064156
HIP_62871	VCGERGFF	MYEENSRDN	VCGERGFFMYEENSRDN		SCG2	22700/334890	0.995	62871	0.792	0.018021
HIP_62872	GSLQPLAL	QKEEEEEMA	GSLQPLALQKEEEEEMA		CMGA	16455/247050	0.983	62872	0.792	0.189227
HIP_62873	EDLQVGQVE	YWDEDLLMK	EDLQVGQVEYWDEDLLMK		SCG2	19659/334890	0.996	62873	0.792	-0.127352
HIP_62874	SHLVEALY	KGQPRSQEE	SHLVEALYKGQPRSQEE		SCG1	28202/367830	0.971	62874	0.792	0.247689
HIP_62875	GPGAGSLQP	GAGRSRSNQ	GPGAGSLQPGAGRSRSNQ		SCG2	16816/334890	0.997	62875	0.792	-0.011441
HIP_62876	SLYQLENYC	FQDKQYSS	SLYQLENYCFQDKQYSS		SCG1	32505/367830	0.955	62876	0.792	-0.046802
HIP_62877	CGERGFFY	SHLPERDS	CGERGFFYSHLPERDS		SCG2	26062/334890	0.993	62877	0.792	0.085513
HIP_62878	TSICSLYQL	MPVSQECFE	TSICSLYQLMPVSQECFE		CMGA	7962/247050	0.997	62878	0.792	-0.107480
HIP_62879	QCCTSICSL	SEDVNWGYE	QCCTSICSLSEDVNWGYE		SCG1	16330/367830	0.994	62879	0.792	0.058836
HIP_62880	AGSLQPLAL	EDEEEEEG	AGSLQPLALEDEEEEEG		SCG1	17179/367830	0.993	62880	0.792	0.108352
HIP_62881	AGSLQPLAL	DEEEEEGE	AGSLQPLALDEEEEEGE		SCG1	17180/367830	0.993	62881	0.792	0.108352
HIP_62882	CGERGFFY	EVLENQSSQ	CGERGFFYEVLENQSSQ		CMGA	15384/247050	0.986	62882	0.792	-0.012653
HIP_62883	SICSLYQLE	TNEIVEEQY	SICSLYQLETNEIVEEQY		SCG2	24557/334890	0.994	62883	0.792	-0.113143
HIP_62884	QCCTSICSL	LEWERYRGR	QCCTSICSLLEWERYRGR		SCG1	30969/367830	0.962	62884	0.792	0.027182
HIP_62885	YLVCGERGF	DYETQQWPE	YLVCGERGFDYETQQWPE		SCG2	21630/334890	0.995	62885	0.792	-0.019848
HIP_62886	CGERGFFY	KENKPYAL	CGERGFFYKENKPYAL		SCG2	31817/334890	0.988	62886	0.792	0.213594
HIP_62887	HLVEALYL	EVRLLRDPA	HLVEALYLEVRLLRDPA		SCG1	27721/367830	0.973	62887	0.792	0.107488
HIP_62888	GGGPGAGSL	SDTREEKRF	GGGPGAGSLSDTREEKRF		SCG1	19684/367830	0.990	62888	0.792	-0.026321
HIP_62889	SICSLYQLE	GPTVVLNPH	SICSLYQLEGPTVVLNPH		CMGA	9648/247050	0.995	62889	0.792	-0.076908
HIP_62890	RGFFYTPKT	WMRIILEAL	RGFFYTPKTWMRIILEAL		SCG2	21644/334890	0.995	62890	0.792	0.071362
HIP_62891	TSICSLYQ	EEEEEEEAE	TSICSLYQEEEEEEEAE		CMGA	12770/247050	0.991	62891	0.792	0.028195
HIP_62892	VELGGGPGA	RQYDRVAQL	VELGGGPGARQYDRVAQL		SCG1	28648/367830	0.970	62892	0.792	0.008113
HIP_62893	PLALEGSLQ	ERATRLFEK	PLALEGSLQERATRLFEK		SCG2	40922/334890	0.975	62893	0.792	-0.055597
HIP_62894	CSLYQLENY	IFLISGAEA	CSLYQLENYIFLISGAEA		SCG2	12989/334890	0.998	62894	0.792	-0.026140
HIP_62895	CTSICSLYQ	GPQLRRGW	CTSICSLYQGPQLRRGW		CMGA	14058/247050	0.989	62895	0.792	0.147537

HIP_63354	IVEQCCTSI	GQEEEEDNR	IVEQCCTSIGQEEEEDNR		CMGA	4535/247050	0.999	63354	0.792	-0.051989	-0.7333	0.3333	-5	18	-1.0944	-5	0	0	2
HIP_63355	QHLCGSHL	NPYYDPLQW	QHLCGSHLNPYYDPLQW		SCG1	28868/367830	0.969	63355	0.792	0.027143	-1.1	0.3333	1	17	-0.8471	-1	2	1	0
HIP_63356	LYQLENYCN	RDSSMKLSF	LYQLENYCNRDSSMKLSF		CMGA	23405/247050	0.959	63356	0.792	0.185171	-1.85	0.1667	0	18	-0.6833	0	1	0	0
HIP_63357	SHLVEALY	DSKRWSKMD	SHLVEALYDSKRWSKMD		CMGA	22525/247050	0.962	63357	0.792	0.243255	-0.65	0.1667	1	17	-0.8882	0	2	1	0
HIP_63358	PGAGSLQP	FLVHSSNN	PGAGSLQPFLVHSSNN		IAPP	1623/85018	0.993	63358	0.792	0.087140	1.5833	0.1667	1	16	-0.2313	0	1	0	0
HIP_63359	LYQLENYCN	ASEAHESSS	LYQLENYCNASEAHESSS		SCG1	15132/367830	0.995	63359	0.792	-0.116344	-0.8	0.1667	-2	18	-0.9056	-3	1	0	1
HIP_63360	GERGFFYT	DSEEPVST	GERGFFYTDSEEPVST		SCG1	25708/367830	0.978	63360	0.792	0.119957	-1.1667	0.1667	-3	16	-0.9625	-3	3	0	0
HIP_63361	TSICSLYQL	DWWEKKPF	TSICSLYQLDWWEKKPF		SCG1	35164/367830	0.944	63361	0.792	0.054494	-1.05	0	0	17	-0.4647	0	3	1	0
HIP_63362	YLVCGERGF	ASLPSQKYP	YLVCGERGFASLPSQKYP		CMGA	13254/247050	0.990	63362	0.792	0.030146	0.45	0.3333	1	18	-0.2611	1	1	0	0
HIP_63363	GERGFFYT	ETTENENT	GERGFFYTETTENENT		SCG1	25464/367830	0.979	63363	0.792	0.101919	-0.6833	0	-3	16	-1.55	-3	3	0	0
HIP_63364	VELGGGPGA	EGEEENELT	VELGGGPGAEGEEENELT		SCG1	6399/367830	0.999	63364	0.792	-0.104091	-1.2667	0.5	-6	18	-0.8444	-6	0	0	2
HIP_63365	GERGFFYTP	RHYINLITR	GERGFFYTPRHYINLITR		NPY	4970/49410	0.933	63365	0.792	0.013101	-2.1	0.1667	3	18	-0.65	2	4	0	0
HIP_63366	ELGGGPGA	YDWWEKKPF	ELGGGPGAYDWWEKKPF		SCG1	16792/367830	0.993	63366	0.792	0.036048	-0.9833	0.3333	-1	17	-1.0471	-1	3	1	1
HIP_63367	PGAGSLQPL	FPPMYEENS	PGAGSLQPLFPPMYEENS		SCG2	5701/334890	0.999	63367	0.792	-0.159923	-0.2833	0.5	-2	18	-0.5556	-2	1	0	0
HIP_63368	QCCTSICSL	YPKTPGRAG	QCCTSICSLYPKTPGRAG		SCG2	37260/334890	0.982	63368	0.792	0.130447	-0.2167	0.3333	2	18	-0.1444	2	1	0	0
HIP_63369	ERGFFYTPK	SQENHPQE	ERGFFYTPKSQENHPQE		SCG1	32882/367830	0.954	63369	0.792	-0.081367	-2.3333	0.3333	0	17	-1.9647	-1	2	1	0
HIP_63370	CTSICSLYQ	REKSSQESG	CTSICSLYQREKSSQESG		SCG1	46762/367830	0.876	63370	0.792	0.182772	-2.15	0	0	18	-0.8611	0	1	0	0
HIP_63371	YQLENYCN	ENQSSQAEL	YQLENYCNENQSSQAEL		CMGA	16877/247050	0.982	63371	0.792	0.100901	-2.1333	0	-3	17	-1.4	-3	1	1	0
HIP_63372	GSLQPLALE	KLSFRARAY	GSLQPLALEKLSFRARAY		CMGA	27583/247050	0.934	63372	0.792	0.088372	0.2	0.1667	2	18	-0.0778	2	1	0	0
HIP_63373	LCGSHLVEA	QQWPERKL	LCGSHLVEAQQWPERKL		SCG2	41464/334890	0.974	63373	0.792	0.142567	-0.9	0	1	17	-0.5529	0	1	1	0
HIP_63374	HLVEALYLV	MSDDYETQ	HLVEALYLVMSDDYETQ		SCG2	13340/334890	0.998	63374	0.792	0.117585	0.7167	0.1667	-3	17	-0.0765	-4	1	1	0
HIP_63375	VEQCCTSIC	FFPEYNYDW	VEQCCTSICFFPEYNYDW		SCG1	11995/367830	0.997	63375	0.792	-0.078017	1.7	0.3333	-3	18	-0.1278	-3	2	0	0
HIP_63376	CGERGFFYT	SGFEDELSE	CGERGFFYTSGFEDELSE		CMGA	3604/247050	0.999	63376	0.792	-0.027397	0.4	0.3333	-4	18	-0.6722	-4	4	0	0
HIP_63377	HLCGSHLVE	KNDDTPNRQ	HLCGSHLVEKNDDTPNRQ		SCG2	40293/334890	0.976	63377	0.792	0.177233	-1.0667	0	1	18	-1.3889	-1	0	0	0
HIP_63378	SLQPLAL	KDVKDKE	SLQPLALKDVKDKE		SCG1	34821/367830	0.945	63378	0.792	0.211424	1.0333	0	0	14	-0.7643	0	0	0	0
HIP_63379	CTSICSLYQ	KGHPQEESE	CTSICSLYQKGHPQEESE		SCG1	28445/367830	0.971	63379	0.792	0.077842	-1.4167	0.1667	-1	18	-0.9833	-2	1	0	0
HIP_63380	YLVCGERGF	ENEPQSAPK	YLVCGERGFENEPQSAPK		SCG2	24940/334890	0.994	63380	0.792	0.000414	-2.1	0.1667	-1	18	-0.9389	-1	1	0	0
HIP_63381	SICSLYQLE	GGHSRERAD	SICSLYQLEGGHSRERAD		SCG1	19593/367830	0.990	63381	0.792	-0.096261	-1.2	0.3333	0	18	-0.7944	-1	1	0	0
HIP_63382	GERGFFYT	AQDPEGKGE	GERGFFYTAQDPEGKGE		CMGA	20709/247050	0.970	63382	0.792	0.044017	-0.7333	0	-2	17	-1.3941	-2	3	1	0
HIP_63383	DLQVGQVEL	GERATRLFE	DLQVGQVELGERATRLFE		SCG2	26022/334890	0.993	63383	0.792	0.195651	-0.65	0.1667	-2	18	-0.3944	-2	0	0	0
HIP_63384	SLYQLENYC	GGHSRERAD	SLYQLENYCGGHSRERAD		SCG1	15208/367830	0.995	63384	0.792	0.056608	-1.05	0.3333	0	18	-1.2667	-1	1	0	0
HIP_63385	LQPLALEGS	FQNRMLSKS	LQPLALEGSFQNRMLSKS		SCG2	16865/334890	0.997	63385	0.792	0.012776	-1.4833	0.3333	1	18	-0.2833	1	1	0	0
HIP_63386	ALYLVCGER	GEDSSEEKH	ALYLVCGERGEDSSEEKH		SCG1	27207/367830	0.974	63386	0.792	-0.124489	-2.6333	0.3333	-2	18	-0.9278	-3	0	0	0
HIP_63387	TSICSLYQL	EDVNWGYEK	TSICSLYQLEDVNWGYEK		SCG1	30539/367830	0.964	63387	0.792	0.026326	-0.6333	0	-2	18	-0.4889	-2	1	0	0
HIP_63388	GSHLVEALY	LSKPSPMPV	GSHLVEALYLSKPSPMPV		CMGA	17180/247050	0.981	63388	0.792	0.119881	0.5667	0.1667	1	18	0.2222	0	1	0	0
HIP_63389	CGSHLVEAL	YRGRGSEEY	CGSHLVEALYRGRGSEEY		SCG1	34500/367830	0.947	63389	0.792	0.099699	-0.6833	0.1667	0	18	-0.6667	-1	1	0	0
HIP_63390	GIVEQCCTS	ELEKVAHQL	GIVEQCCTSELEKVAHQL		CMGA	18902/247050	0.976	63390	0.792	-0.054012	-0.3667	0.1667	-1	18	0.0444	-2	0	0	0
HIP_63391	HLVEALYL	FEKPLDSQS	HLVEALYLFEKPLDSQS		SCG2	26891/334890	0.992	63391	0.792	0.121938	0.2833	0	-1	17	-0.0941	-2	2	1	0
HIP_63392	SICSLYQLE	PPKNDDTPN	SICSLYQLEPPKNDDTPN		SCG2	23640/334890	0.994	63392	0.792	-0.044938	-1.7167	0.3333	-2	18	-1.0389	-2	1	0	0
HIP_63393	VCGERGFFY	KGDTEVMK	VCGERGFFYKGDTEVMK		CMGA	18912/247050	0.976	63393	0.792	0.218471	-0.5833	0.1667	0	17	-0.4471	0	3	1	0
HIP_63394	GSLQPLAL	PEIINSNQ	GSLQPLALPEIINSNQ		SCG2	14359/334890	0.998	63394	0.792	0.137271	1.4667	0.1667	-1	16	-0.0313	-1	0	0	0
HIP_63395	CGERGFFYT	ATLESVFQ	CGERGFFYTATLESVFQ		SCG2	20433/334890	0.996	63395	0.792	-0.070434	0.95	0	-1	17	0.0824	-1	3	1	0
HIP_63396	LVEALYLVC	CATQRLAN	LVEALYLVCCATQRLAN		IAPP	3083/85018	0.977	63396	0.792	0.042585	2.35	0	0	17	1	0	1	1	0
HIP_63397	ELGGGPGAG	GPQLRRGWR	ELGGGPGAGGPQLRRGWR		CMGA	11109/247050	0.993	63397	0.792	-0.061835	-0.75	0.6667	2	18	-1	2	0	0	1
HIP_63398	TSICSLYQL	AIEAELEKV	TSICSLYQLAIEAELEKV		CMGA	15651/247050	0.985	63398	0.792	-0.074820	0.3	0	-2	18	0.5111	-2	1	0	0
HIP_63399	CTSICSLYQ	ASEEEPEYG	CTSICSLYQASEEEPEYG		SCG1	13333/367830	0.996	63399	0.792	-0.077828	-0.5833	0.1667	-4	18	-0.5611	-4	1	0	1
HIP_63400	VELGGGPGA	EDSLEAGLP	VELGGGPGAEDSLEAGLP		CMGA	5052/247050	0.998	63400	0.792	-0.142087	-1.3333	0.5	-4	18	-0.0444	-4	0	0	1
HIP_63401	QCCTSICSL	LTAEKRLEG	QCCTSICSLLTAEKRLEG		CMGA	15267/247050	0.986	63401	0.792	-0.004635	1.7333	0.1667	0	18	0.1611	0	0	0	0
HIP_63402	LVEALYLV	GYPEEKKEE	LVEALYLVGYPEEKKEE		CMGA	9840/247050	0.995	63402	0.792	0.145102	0.5667	0.3333	-3	17	-0.4882	-3	2	1	0
HIP_63403	QCCTSICSL	LCAGQVTAL	QCCTSICSLLCAGQVTAL		CMGA	12218/247050	0.992	63403	0.792	0.042622	2.2667	0.1667	0	18	1.2944	0	0	0	0
HIP_63404	SHLVEALYL	DGLSVEDI	SHLVEALYLDGLSVEDI		SCG2	11211/334890	0.999	63404	0.792	0.158491	1.0333	0.1667	-3	17	0.5529	-4	1	1	0

HIP_63865	SICSLYQL	LFQNRMLS	SICSLYQLLFQNRMLS		SCG2	25085/334890	0.994	63865	0.791	-0.036657	0.35	0	1	16	0.5125	1	2	[illegible]
HIP_63866	TSICSLYQL	PDGLSVEDI	TSICSLYQLPDGLSVEDI		SCG2	7687/334890	0.999	63866	0.791	0.112324	-1.0833	0.3333	-3	18	0.3722	-3	1	[illegible]
HIP_63867	VEALYLVCG	LRQQAHKEE	VEALYLVCGLRQQAHKEE		SCG2	26506/334890	0.993	63867	0.791	0.003385	0.35	0.1667	0	18	-0.2722	-1	1	[illegible]
HIP_63868	LYQLENYCN	PGWQAKRE	LYQLENYCNPGWQAKRE		CMGA	9668/247050	0.995	63868	0.791	0.023462	-0.8667	0.3333	0	17	-1.3529	0	2	[illegible]
HIP_63869	GSLQPLALE	PQEESEESN	GSLQPLALEPQEESEESN		SCG1	17836/367830	0.992	63869	0.791	0.077293	-1.0833	0.1667	-5	18	-1.1556	-5	0	[illegible]
HIP_63870	SLYQLENYC	EVLKREPLN	SLYQLENYCEVLKREPLN		IAPP	3568/45018	0.967	63870	0.791	-0.087854	0.3667	0	-1	18	-0.6944	-1	1	[illegible]
HIP_63871	PGAGSLQPL	EEEQIEQAI	PGAGSLQPLEEEQIEQAI		SCG2	19835/334890	0.996	63871	0.791	-0.045790	-1.9667	0.1667	-4	18	-0.5056	-4	0	[illegible]
HIP_63872	HLVEALYL	SGAEAASFQ	HLVEALYLSGAEAASFQ		SCG2	10860/334890	0.999	63872	0.791	0.163142	1.15	0.3333	-1	17	0.5059	-2	1	[illegible]
HIP_63873	ALYLVCGER	EPQWSLYPS	ALYLVCGEREPQWSLYPS		SCG1	28302/367830	0.971	63873	0.791	-0.119564	-2.8333	0.3333	-1	18	-0.2111	-1	1	[illegible]
HIP_63874	VEALYLVC	EEEEDNRD	VEALYLVCEEEEDNRD		CMGA	13403/247050	0.990	63874	0.791	0.114652	0	0	-6	16	-0.8438	-6	1	[illegible]
HIP_63875	PGAGSLQPL	LLVCLGALA	PGAGSLQPLLLVCLGALA		NPY	1594/49410	0.991	63875	0.791	0.105549	1.75	0.1667	0	18	1.4556	0	0	[illegible]
HIP_63876	VNQHLCGSH	DSEEPVSTH	VNQHLCGSHDSEEPVSTH		SCG1	24541/367830	0.981	63876	0.791	-0.117305	-2.0333	0.5	0	18	-0.9722	-3	0	[illegible]
HIP_63877	SHLVEALY	NAVEVLKRE	SHLVEALYNAVEVLKRE		IAPP	2972/45018	0.978	63877	0.791	0.021704	1.1333	0	0	17	-0.0059	-1	1	[illegible]
HIP_63878	SLQPLALEG	NQGSSQETD	SLQPLALEGNQGSSQETD		SCG2	13295/334890	0.998	63878	0.791	-0.094847	-1.25	0.3333	-3	18	-0.9333	-3	0	[illegible]
HIP_63879	LGGGPGAGS	NLITRQRYG	LGGGPGAGSNLITRQRYG		NPY	2139/49410	0.986	63879	0.791	0.115665	0.9	0.3333	2	18	-0.4944	2	0	[illegible]
HIP_63880	LVEALYLVC	ATCATQRL	LVEALYLVCATCATQRL		IAPP	3084/45018	0.977	63880	0.791	0.037563	2.35	0	0	17	1.1647	0	1	[illegible]
HIP_63881	GIVEQCCT	YDWWEKKPF	GIVEQCCTYDWWEKKPF		SCG1	33239/367830	0.952	63881	0.791	0.050730	-0.2333	0	-1	17	-0.6529	-1	3	[illegible]
HIP_63882	TSICSLYQL	KGERGEDSS	TSICSLYQLKGERGEDSS		SCG1	27047/367830	0.975	63882	0.791	0.213121	-1.4667	0.1667	-1	18	-0.7667	-1	1	[illegible]
HIP_63883	CGSHLVEAL	PQLRRGWRP	CGSHLVEALPQLRRGWRP		CMGA	24660/247050	0.952	63883	0.791	0.080112	0.1333	0.1667	3	18	-0.5278	2	0	[illegible]
HIP_63884	GSLQPLAL	PGPQAEGD	GSLQPLALPGPQAEGD		CMGA	9558/247050	0.995	63884	0.791	0.198347	0.9667	0.5	-2	16	-0.3625	-2	0	[illegible]
HIP_63885	GGPGAGSLQ	DWNPVEEKI	GGPGAGSLQDWNPVEEKI		SCG2	21424/334890	0.995	63885	0.791	-0.111250	-1.4	0.1667	-2	18	-0.7556	-2	1	[illegible]
HIP_63886	CGSHLVEAL	EEVKTRHSE	CGSHLVEALEEVKTRHSE		SCG1	35072/367830	0.944	63886	0.791	0.011087	-0.1167	0	0	18	-0.6222	-2	0	[illegible]
HIP_63887	CGERGFFYT	PGRAGTEA	CGERGFFYTPGRAGTEA		SCG2	23235/334890	0.995	63887	0.791	0.171820	-0.95	0.3333	0	17	-0.6	0	3	[illegible]
HIP_63888	LQPLALEGS	EDELSEVLE	LQPLALEGSEDELSEVLE		CMGA	12654/247050	0.991	63888	0.791	-0.146291	-2.5333	0.3333	-6	18	-0.1722	-6	0	[illegible]
HIP_63889	CGERGFFYT	LRLENVQK	CGERGFFYTLRLENVQK		SCG2	29120/334890	0.991	63889	0.791	-0.027390	0.65	0	1	17	-0.5765	1	3	[illegible]
HIP_63890	CSLYQLENY	SSQESGEET	CSLYQLENYSSQESGEET		SCG1	18403/367830	0.992	63890	0.791	-0.071362	-2.2333	0.3333	-4	18	-1.1833	-4	1	[illegible]
HIP_63891	EAEDLQVGQ	QKEEEEEMA	EAEDLQVGQQKEEEEEMA		CMGA	11203/247050	0.993	63891	0.791	-0.127851	-1.7667	0.1667	-7	18	-1.6278	-7	0	[illegible]
HIP_63892	LVCGERGFF	DQLAKELT	LVCGERGFFDQLAKELT		CMGA	16165/247050	0.984	63892	0.791	0.024075	0.3333	0.1667	-1	17	0.0941	-1	2	[illegible]
HIP_63893	LYLVCGERG	GGEDWNPVE	LYLVCGERGGGEDWNPVE		SCG2	15924/334890	0.997	63893	0.791	-0.116287	-2.1167	0.5	-3	18	-0.4944	-3	0	[illegible]
HIP_63894	GSHLVEALY	SSQAELKE	GSHLVEALYSSQAELKE		CMGA	13249/247050	0.990	63894	0.791	0.191396	-0.1333	0.3333	-1	17	-0.3529	-2	1	[illegible]
HIP_63895	ICSLYQLEN	GGHSRERAD	ICSLYQLENGGHSRERAD		SCG1	19817/367830	0.990	63895	0.791	-0.012597	-1.2	0.3333	0	18	-0.9444	-1	1	[illegible]
HIP_63896	QVELGGGPG	EPGKGRHHR	QVELGGGPGEPGKGRHHR		SCG1	18941/367830	0.991	63896	0.791	-0.049183	-1.3167	0.8333	3	18	-1.5222	1	0	[illegible]
HIP_63897	VELGGGPGA	LLMRESTEN	VELGGGPGALLMRESTEN		NPY	470/49410	0.988	63897	0.791	0.128832	1.55	0.3333	-2	18	-0.2167	-2	0	[illegible]
HIP_63898	GGGPGAGSL	RLPYGAGRS	GGGPGAGSLRLPYGAGRS		SCG2	24056/334890	0.994	63898	0.791	0.021337	0.05	0.5	2	18	-0.3722	2	1	[illegible]
HIP_63899	GPGAGSLQP	AALSLIPLI	GPGAGSLQPAALSLIPLI		SCG2	12223/334890	0.998	63899	0.791	0.124723	1.0167	0.1667	0	18	1.0278	0	0	[illegible]
HIP_63900	CGSHLVEAL	EEKHLEEPG	CGSHLVEALEEKHLEEPG		SCG1	32914/367830	0.954	63900	0.791	0.099194	-1.4667	0	-2	18	-0.6167	-4	0	[illegible]
HIP_63901	LVEALYLVC	EISRNLQI	LVEALYLVCEISRNLQI		SCG2	19085/334890	0.996	63901	0.791	0.148772	1.7833	0.1667	-1	17	0.9588	-1	1	[illegible]
HIP_63902	LYQLENYCN	EGQEEEED	LYQLENYCNEGQEEEED		CMGA	7267/247050	0.997	63902	0.791	0.038925	-1.6167	0.1667	-7	17	-1.8471	-7	1	[illegible]
HIP_63903	GSHLVEALY	EKRKCNTAT	GSHLVEALYEKRKCNTAT		IAPP	4900/45018	0.931	63903	0.791	0.211853	-1.2667	0	2	18	-0.6667	1	1	[illegible]
HIP_63904	VCGERGFF	TLNEKNFFP	VCGERGFFTLNEKNFFP		SCG1	21678/367830	0.987	63904	0.791	0.008113	0.8	0.1667	0	17	-0.2235	0	2	[illegible]
HIP_63905	ALYLVCGER	YGEEIKGYP	ALYLVCGERYGEEIKGYP		SCG1	24722/367830	0.981	63905	0.791	-0.093957	-2.2667	0.3333	-1	18	-0.2778	-1	1	[illegible]
HIP_63906	QHLCGSHLV	RADQTVLTE	QHLCGSHLVRADQTVLTE		SCG1	36991/367830	0.935	63906	0.791	0.201974	-0.2333	0	1	18	-0.1889	-1	0	[illegible]
HIP_63907	VCGERGFF	QGAKERAH	VCGERGFFQGAKERAH		CMGA	16975/247050	0.982	63907	0.791	0.083484	0.5167	0.3333	2	16	-0.7437	1	2	[illegible]
[illegible]																		

HIP_64376	GSHLVEALY	EPGETQNA	GSHLVEALYEPGETQNA		SCG1	15024/367830	0.995	64376	0.791	0.174903	-0.2	0.3333	-2	17	-0.6176	-3	1	1
HIP_64377	LCGSHLVEA	EKNFFPEYN	LCGSHLVEAEKNFFPEYN		SCG1	40127/367830	0.918	64377	0.791	0.172403	-1.4	0	-1	18	-0.3889	-2	1	0
HIP_64378	ERGFFYTP	NSNQVKRV	ERGFFYTPNSNQVKRV		SCG2	28576/334890	0.991	64378	0.791	0.016944	-1.9	0.3333	2	16	-1.1063	2	3	0
HIP_64379	GERGFFY	PGEEEEE	GERGFFYPGEEEEE		CMGA	11169/247050	0.993	64379	0.791	0.157412	-0.2	0.3333	-5	14	-1.7143	-5	3	0
HIP_64380	QHLCGSHLV	LGEKRDHH	QHLCGSHLVLGEKRDHH		SCG1	24366/367830	0.982	64380	0.791	0.172344	0.7833	0.1667	4	17	-0.8941	0	0	1
HIP_64381	EQCCTSICS	IFLISGAEA	EQCCTSICSIFLISGAEA		SCG2	15513/334890	0.998	64381	0.791	-0.011403	2.8833	0.1667	-2	18	0.9556	-2	1	0
HIP_64382	CSLYQLENY	ESEEGEEDA	CSLYQLENYESEEGEEDA		SCG1	23749/367830	0.983	64382	0.791	-0.072708	-2.6833	0.1667	-7	18	-1.3444	-7	1	0
HIP_64383	SHLVEALY	DEMKRSGQL	SHLVEALYDEMKRSGQL		SCG2	28932/334890	0.991	64383	0.791	0.160328	-0.1333	0	0	17	-0.5647	-1	1	1
HIP_64384	AGSLQPLAL	PGEEEEEE	AGSLQPLALPGEEEEEE		CMGA	7527/247050	0.997	64384	0.791	0.159355	0.65	0.3333	-6	17	-0.8412	-6	0	1
HIP_64385	VCGERGFF	GEAGAPGEE	VCGERGFFGEAGAPGEE		SCG1	12246/367830	0.997	64385	0.791	-0.000390	0.5167	0.3333	-3	17	-0.3647	-3	2	1
HIP_64386	GSLQPLAL	LTLNEKNFF	GSLQPLALLTLNEKNFF		SCG1	26476/367830	0.976	64386	0.791	0.055440	2.7167	0	0	17	0.2941	0	0	1
HIP_64387	GERGFFYT	PRSQEESEE	GERGFFYTPRSQEESEE		SCG1	25410/367830	0.979	64387	0.791	0.127360	-1.0167	0.3333	-3	17	-1.7882	-3	3	1
HIP_64388	HLCGSHLVE	KIESQTQEE	HLCGSHLVEKIESQTQEE		SCG2	34452/334890	0.985	64388	0.791	0.028090	0.2667	0	-1	18	-0.8444	-3	0	0
HIP_64389	CGERGFFY	LGAALSLIP	CGERGFFYLGAALSLIP		SCG2	14403/334890	0.998	64389	0.791	0.027947	1.5833	0.1667	0	17	0.8647	0	3	1
HIP_64390	CGERGFFYT	EEAQQPEG	CGERGFFYTEEAQQPEG		CMGA	18644/247050	0.977	64390	0.791	0.027312	-0.7333	0	-4	17	-1.2	-4	3	1
HIP_64391	VCGERGFF	RKQASAIKK	VCGERGFFRKQASAIKK		SCG1	44868/367830	0.889	64391	0.791	0.133954	-1.1167	0.1667	4	17	-0.5235	4	2	1
HIP_64392	GERGFFYT	RFQDKQYSS	GERGFFYTRFQDKQYSS		SCG1	39640/367830	0.920	64392	0.791	0.072046	-0.7333	0	1	17	-1.4235	1	4	1
HIP_64393	AGSLQPLAL	MAVVPQGLF	AGSLQPLALMAVVPQGLF		CMGA	16622/247050	0.983	64393	0.791	0.013590	2.8833	0	0	18	1.2167	0	0	0
HIP_64394	VEALYLVCG	KRSGQLGIQ	VEALYLVCGKRSGQLGIQ		SCG2	35210/334890	0.985	64394	0.791	0.129020	-0.4833	0.3333	1	18	0.3556	1	1	0
HIP_64395	RGFFYTPKT	SDTLSKPSP	RGFFYTPKTSDTLSKPSP		CMGA	25525/247050	0.947	64395	0.791	-0.060960	-1.8667	0.3333	2	18	-0.9667	2	1	0
HIP_64396	HLVEALYL	QVFLIVL	HLVEALYLQVFLIVL		IAPP	3026/45018	0.978	64396	0.791	0.053561	1.6333	0	0	15	1.9467	-1	2	1
HIP_64397	VEALYLVC	LEGEEENEL	VEALYLVCLEGEEENEL		SCG1	10510/367830	0.997	64397	0.791	0.119526	1.7333	0.1667	-6	17	0.1	-6	1	1
HIP_64398	VELGGGPGA	YGEEIKGYP	VELGGGPGAYGEEIKGYP		SCG1	9198/367830	0.998	64398	0.791	-0.086362	-0.9	0.5	-2	18	-0.4611	-2	1	0
HIP_64399	AGSLQPLAL	PRLPYGAG	AGSLQPLALPRLPYGAG		SCG2	29384/334890	0.991	64399	0.791	0.212497	1.1833	0.1667	1	17	0.2647	1	0	1
HIP_64400	LVEALYLV	SLGYKEIRK	LVEALYLVSLGYKEIRK		CMGA	11873/247050	0.992	64400	0.791	0.141593	1.55	0.3333	1	17	0.4	1	2	1
HIP_64401	HLVEALYLV	AEGDSEGL	HLVEALYLVAEGDSEGL		CMGA	7407/247050	0.997	64401	0.791	0.128731	0.7667	0.1667	-3	17	0.4176	-4	1	1
HIP_64402	CSLYQLEN	EEEEEATNT	CSLYQLENEEEEEATNT		CMGA	17680/247050	0.980	64402	0.791	-0.024320	-2.2833	0	-6	17	-1.3588	-6	0	1
HIP_64403	VELGGGPGA	ETTENENTK	VELGGGPGAETTENENTK		SCG1	9793/367830	0.998	64403	0.791	-0.125089	-0.85	0.3333	-3	18	-1.1333	-3	0	0
HIP_64404	LVEALYL	EDLEWERYR	LVEALYLEDLEWERYR		SCG1	24423/367830	0.981	64404	0.791	0.103883	0.5167	0	-3	16	-0.55	-3	2	2
HIP_64405	GERGFFYTP	GEQEHSQQ	GERGFFYTPGEQEHSQQ		CMGA	14832/247050	0.987	64405	0.791	-0.019117	-1.8333	0.3333	-1	17	-1.6882	-2	3	1
HIP_64406	GSLQPLALE	LLVCLGALA	GSLQPLALELLVCLGALA		NPY	2678/49410	0.980	64406	0.791	0.001713	2.3167	0	-1	18	1.5833	-1	0	0
HIP_64407	LVCGERGFF	LSEVLENQS	LVCGERGFFLSEVLENQS		CMGA	9738/247050	0.995	64407	0.791	-0.043813	0.7833	0.3333	-2	18	0.1944	-2	2	0
HIP_64408	QVELGGGPG	GYPKTPGRA	QVELGGGPGGYPKTPGRA		SCG2	10863/334890	0.999	64408	0.791	-0.025867	-0.95	0.8333	1	18	-0.8222	1	1	0
HIP_64409	GQVELGGG	ALAEAYPS	GQVELGGGALAEAYPS		NPY	775/49410	0.996	64409	0.791	0.161314	1.0333	0.5	-2	16	0.0875	-2	0	0
HIP_64410	EDLQVGQV	LALEGSLQ	EDLQVGQVLALEGSLQ		INSC	59/5184	0.999	64410	0.791	0.108730	1.6167	0.1667	-3	16	0.175	-3	0	0
HIP_64411	SHLVEALYL	SRNLQIPP	SHLVEALYLSRNLQIPP		SCG2	29737/334890	0.990	64411	0.791	0.245861	-0.4167	0.1667	1	17	0.0824	0	1	1
HIP_64412	VEALYLVCG	GVSVPLQQK	VEALYLVCGGVSVPLQQK		SCG2	7907/334890	0.999	64412	0.791	-0.001830	1.55	0.5	0	18	0.7556	0	1	0
HIP_64413	GSLQPLAL	GSLPSEEKG	GSLQPLALGSLPSEEKG		SCG1	14391/367830	0.995	64413	0.791	0.183622	2	0.3333	-1	17	-0.2471	-1	0	1
HIP_64414	LVCGERGFF	GELFNPYY	LVCGERGFFGELFNPYY		SCG1	17387/367830	0.993	64414	0.791	0.025296	0.85	0.3333	-1	17	0.1353	-1	3	1
HIP_64415	TSICSLYQL	NFLEGEEEN	TSICSLYQLNFLEGEEEN		SCG1	14857/367830	0.995	64415	0.791	-0.101077	0.35	0	-4	18	-0.4056	-4	2	0
HIP_64416	CTSICSLYQ	SRGEAGAPG	CTSICSLYQSRGEAGAPG		SCG1	23107/367830	0.984	64416	0.791	0.193464	-1.1167	0.3333	0	18	-0.1	0	1	0
HIP_64417	LYQLENYCN	LDQLLHYRK	LYQLENYCNLDQLLHYRK		SCG1	33906/367830	0.950	64417	0.791	-0.078081	-0.9167	0	1	18	-0.8333	0	1	0
HIP_64418	AGSLQPLAL	AEDLQVG	AGSLQPLALAEDLQVG		INSC	288/5184	0.997	64418	0.791	0.067564	0.7	0	-2	16	0.475	-2	0	2
HIP_64419	GSHLVEALY	SGEATDGA	GSHLVEALYSGEATDGA		CMGA	10223/247050	0.995	64419	0.791	0.172081	-0.0667	0.3333	-2	17	-0.0765	-3	1	1
HIP_64420	GSLQPLALE	RQQAHKEES	GSLQPLALERQQAHKEES		SCG2	38513/334890	0.980	64420	0.791	0.111831	-1.5667	0	0	18	-1.1778	-1	0	0
HIP_64421	HLVEALYL	GFEDELSE	HLVEALYLGFEDELSE		CMGA	9808/247050	0.995	64421	0.791	0.138997	0.8667	0.1667	-4	16	0.05	-5	2	0
HIP_64422	AGSLQPLAL	SLYQLEN	AGSLQPLALSLYQLEN		INSA	192/3024	0.918	64422	0.791	0.049307	1.85	0.1667	-1	16	0.2312	-1	1	2
HIP_64423	GPGAGSLQP	GPTKADTEK	GPGAGSLQPGPTKADTEK		SCG1	13608/367830	0.996	64423	0.791	0.003033	-0.6667	0.5	0	18	-1.0833	0	0	0

HIP_64887	EQCCTSICS	WDEDLLMKV	EQCCTSICSWDEDLLMKV		SCG2	21436/334890	0.995	64887	0.790	0.006239	-0.2833	0.1667	-3	18	0.0611	-3	1	0	0
HIP_64888	VCGERGFFY	VLENQSSQ	VCGERGFFYVLENQSSQ		CMGA	16609/247050	0.983	64888	0.790	-0.007665	1.4667	0	-1	17	-0.3176	-1	3	1	0
HIP_64889	FVNQHLCG	NYDWWEKK	FVNQHLCGNYDWWEKK		SCG1	30485/367830	0.964	64889	0.790	0.048783	-0.4	0.1667	1	16	-1.1688	0	2	0	0
HIP_64890	VEALYLVCG	DELSEVLEN	VEALYLVCGDELSEVLEN		CMGA	8789/247050	0.996	64890	0.790	-0.058795	0.5167	0.1667	-5	18	0.4778	-5	1	0	0
HIP_64891	SICSLYQLE	NRQMAYENL	SICSLYQLENRQMAYENL		SCG2	37065/334890	0.982	64891	0.790	-0.024253	-2.45	0	-1	18	-0.4222	-1	1	0	0
HIP_64892	AEDLQVGQV	VALNHLKA	AEDLQVGQVVALNHLKA		IAPP	1225/45018	0.995	64892	0.790	0.140063	1.6833	0.1667	0	17	0.2588	-1	0	1	0
HIP_64893	GGGPGAGSL	NQVKRVPGQ	GGGPGAGSLNQVKRVPGQ		SCG2	22971/334890	0.995	64893	0.790	0.025336	-0.0333	0.3333	2	18	-0.6278	2	0	0	1
HIP_64894	LGGGPGAGS	EEGEEDATS	LGGGPGAGSEEGEEDATS		SCG1	9898/367830	0.998	64894	0.790	-0.093894	-1.1333	0.5	-5	18	-0.9111	-5	0	0	1
HIP_64895	LGGGPGAGS	EGEEDATSE	LGGGPGAGSEGEEDATSE		SCG1	9899/367830	0.998	64895	0.790	-0.093894	-1.1333	0.5	-5	18	-0.9111	-5	0	0	1
HIP_64896	QHLCGSHLV	LELQKIAEK	QHLCGSHLVLELQKIAEK		SCG1	31000/367830	0.962	64896	0.790	0.033945	1.4833	0	2	18	-0.0667	0	0	0	0
HIP_64897	VGQVELGGG	YENLNDKDQ	VGQVELGGGYENLNDKDQ		SCG2	8089/334890	0.999	64897	0.790	0.006463	-1.5833	0.5	-3	18	-1.0444	-3	1	0	1
HIP_64898	GSLQPLAL	SALRHYINL	GSLQPLALSALRHYINL		NPY	3755/49410	0.963	64898	0.790	0.085835	2.3667	0.1667	2	17	0.6412	1	0	1	0
HIP_64899	ICSLYQLE	EEEGSANRR	ICSLYQLEEEEGSANRR		CMGA	24711/247050	0.952	64899	0.790	0.043692	-2.2833	0	-2	17	-0.9941	-2	1	1	2
HIP_64900	PGAGSLQPL	NVQKFPSPE	PGAGSLQPLNVQKFPSPE		SCG2	22905/334890	0.995	64900	0.790	-0.038442	-0.6833	0.1667	0	18	-0.5722	0	0	0	0
HIP_64901	GGGPGAG	VCGERGFF	GGGPGAGVCGERGFF		INSB	14/4968	0.999	64901	0.790	0.026861	1.2167	0.5	0	15	0.1133	0	0	1	1
HIP_64902	LQPLALEGS	GEQEHSQQK	LQPLALEGSGEQEHSQQK		CMGA	15501/247050	0.986	64902	0.790	-0.111339	-2.0167	0.5	-1	18	-1.2444	-2	0	0	0
HIP_64903	LYQLENYCN	VGSNTYGKR	LYQLENYCNVGSNTYGKR		IAPP	1900/45018	0.991	64903	0.790	-0.030118	0.1167	0.3333	1	18	-0.9889	1	1	0	0
HIP_64904	CCTSICSLY	SSQGGSLPS	CCTSICSLYSSQGGSLPS		SCG1	19772/367830	0.990	64904	0.790	0.104842	-0.5667	0.5	0	18	0.3833	0	1	0	0
HIP_64905	ICSLYQLEN	ESKAEGNNQ	ICSLYQLENESKAEGNNQ		CMGA	26645/247050	0.940	64905	0.790	0.041913	-1.9	0.1667	-2	18	-1.0444	-2	1	0	0
HIP_64906	GSLQPLALE	LEALRQAEN	GSLQPLALELEALRQAEN		SCG2	23753/334890	0.994	64906	0.790	-0.075091	-0.7	0	-2	18	-0.2167	-2	0	0	0
HIP_64907	LCGSHLVEA	GAPGKWQQQ	LCGSHLVEAGAPGKWQQQ		SCG1	17382/367830	0.993	64907	0.790	0.128808	0.3833	0.3333	1	18	-0.4278	0	0	0	1
HIP_64908	GSLQPLALE	EDIQGPTKA	GSLQPLALEEDIQGPTKA		SCG1	32702/367830	0.955	64908	0.790	0.005523	-0.0667	0	-2	18	-0.4111	-2	0	0	0
HIP_64909	CCTSICSLY	KSSAPPITP	CCTSICSLYKSSAPPITP		SCG1	27957/367830	0.972	64909	0.790	0.112685	-0.6333	0.5	1	18	0.4167	1	1	0	0
HIP_64910	CGERGFFYT	GLVDREKG	CGERGFFYTGLVDREKG		CMGA	14350/247050	0.988	64910	0.790	-0.071171	1.4	0.1667	0	17	-0.6412	0	3	1	0
HIP_64911	HLVEALYLV	IAYLKRLV	HLVEALYLVIAYLKRLV		SCG2	24330/334890	0.994	64911	0.790	0.006575	1.95	0	2	17	1.2941	1	2	1	0
HIP_64912	ICSLYQLEN	SEKSQREDE	ICSLYQLENSEKSQREDE		SCG1	35904/367830	0.960	64912	0.790	0.038866	-1.9	0.1667	-3	18	-1.4167	-3	1	0	0
HIP_64913	EDLQVGQVE	ENEKDRADQ	EDLQVGQVEENEKDRADQ		SCG1	28762/367830	0.970	64913	0.790	-0.103386	-2.2167	0	-5	18	-1.85	-5	0	0	0
HIP_64914	AGSLQPLAL	RSNQLPKA	AGSLQPLALRSNQLPKA		SCG2	31563/334890	0.989	64914	0.790	0.253042	0.1	0.1667	2	17	-0.2059	2	0	1	0
HIP_64915	GIVEQCCTS	SANRRPEDQ	GIVEQCCTSSANRRPEDQ		CMGA	15625/247050	0.985	64915	0.790	-0.074318	-0.25	0.3333	-1	18	-1.0444	-1	0	0	0
HIP_64916	SLQPLALEG	RYGKRSSPE	SLQPLALEGRYGKRSSPE		NPY	4940/49410	0.934	64916	0.790	-0.013055	-1.05	0.3333	1	18	-0.9944	1	1	0	0
HIP_64917	CGERGFFY	PKLDLKRQ	CGERGFFYPKLDLKRQ		SCG1	34460/367830	0.947	64917	0.790	0.180422	0.4333	0.1667	2	16	-0.9563	2	3	0	0
HIP_64918	TSICSLYQ	YYDPLQWKS	TSICSLYQYYDPLQWKS		SCG1	37527/367830	0.932	64918	0.790	0.016298	-1.1833	0	0	17	-0.5471	0	3	1	0
HIP_64919	PGAGSLQPL	HWLGAALSL	PGAGSLQPLHWLGAALSL		SCG2	15119/334890	0.998	64919	0.790	0.122185	-0.2667	0.1667	1	18	0.6	0	1	0	0
HIP_64920	GSHLVEALY	LFEKPLDS	GSHLVEALYLFEKPLDS		SCG2	21500/334890	0.995	64920	0.790	-0.020234	1.2333	0	-1	17	0.0882	-2	2	1	0
HIP_64921	CTSICSLYQ	KDRADQTVL	CTSICSLYQKDRADQTVL		SCG1	45333/367830	0.886	64921	0.790	0.164297	-2.15	0	0	18	-0.2	0	1	0	0
HIP_64922	ICSLYQLEN	ETTENENTK	ICSLYQLENETTENENTK		SCG1	30918/367830	0.962	64922	0.790	-0.035444	-1.35	0	-3	18	-1.1944	-3	1	0	0
HIP_64923	CGERGFFY	GLVDREKGL	CGERGFFYGLVDREKGL		CMGA	11581/247050	0.993	64923	0.790	-0.003331	1.9833	0.1667	0	17	-0.3765	0	3	1	0
HIP_64924	GERGFFYTP	EEKGHPQE	GERGFFYTPEEKGHPQE		SCG1	35257/367830	0.944	64924	0.790	0.011790	-2.4167	0.1667	-1	17	-1.7588	-2	3	1	0
HIP_64925	LCGSHLVEA	LIEISRNLQ	LCGSHLVEALIEISRNLQ		SCG2	25295/334890	0.994	64925	0.790	-0.026980	1.2167	0	0	18	0.5	-1	0	0	0
HIP_64926	QCCTSICSL	EHSQQKEEE	QCCTSICSLEHSQQKEEE		CMGA	16581/247050	0.988	64926	0.790	0.021583	-0.3333	0.3333	-2	18	-1.05	-3	0	0	1
HIP_64927	VEALYLVC	ATDGARPQA	VEALYLVCATDGARPQA		CMGA	17316/247050	0.981	64927	0.790	0.161951	1.35	0	-1	17	0.3941	-1	1	1	0
HIP_64928	PGAGSLQPL	NAVEVLKRE	PGAGSLQPLNAVEVLKRE		IAPP	1988/45018	0.991	64928	0.790	0.046659	0.2	0.1667	0	18	-0.2111	0	0	0	0
HIP_64929	AGSLQPLAL	IRKGESRSE	AGSLQPLALIRKGESRSE		CMGA	26786/247050	0.939	64929	0.790	0.151056	0.9167	0	1	18	-0.4833	1	0	0	0

[illegible]

HIP_65398	RGFFYTPKT	EDDIYKANN	RGFFYTPKTEDDIYKANN		SCG2	32256/334890	0.988	65398	0.790	-0.057644	-2.7833	0.1667	0	18	-1.3278	0
HIP_65399	SHLVEALY	PYQGVSVP	SHLVEALYPYQGVSVP		SCG2	23299/334890	0.995	65399	0.790	0.131582	-0.35	0.1667	0	16	0.25	-1
HIP_65400	VNQHLCGSH	PEDQELESL	VNQHLCGSHPEDQELESL		CMGA	12288/247050	0.992	65400	0.790	-0.145293	-2.1667	0.5	-2	18	-0.9111	-4
HIP_65401	CGSHLVEAL	KRERMDEEQ	CGSHLVEALKRERMDEEQ		SCG2	39715/334890	0.978	65401	0.790	0.112972	-1.6333	0	-1	18	-1.1278	-2
HIP_65402	ERGFFYTPK	YIENLRQQA	ERGFFYTPKYIENLRQQA		SCG2	24157/334890	0.994	65402	0.790	0.039917	-1.0833	0.1667	1	18	-1.1111	1
HIP_65403	LYLVCGERG	ENENTKFEV	LYLVCGERGENENTKFEV		SCG1	31880/367830	0.958	65403	0.790	-0.085394	-3.15	0.1667	-2	18	-0.6056	-2
HIP_65404	GPGAGSLQP	YIENLRQQA	GPGAGSLQPYIENLRQQA		SCG2	21264/334890	0.996	65404	0.790	0.024879	-0.2667	0.1667	0	18	-0.7111	0
HIP_65405	RGFFYTPKT	YFPNPYNQE	RGFFYTPKTYFPNPYNQE		SCG2	20441/334890	0.996	65405	0.790	0.008490	-1.05	0.3333	1	18	-1.3611	1
HIP_65406	PGAGSLQPL	LLHYRKKSA	PGAGSLQPLLLHYRKKSA		SCG1	24079/367830	0.982	65406	0.790	0.126976	0.5167	0.1667	4	18	-0.3944	3
HIP_65407	LVEALYLV	NWGYEKRN	LVEALYLVNWGYEKRN		SCG1	24983/367830	0.980	65407	0.790	0.157683	0.3167	0.1667	0	16	-0.2938	0
HIP_65408	RGFFYTPKT	GLEPGKGRH	RGFFYTPKTGLEPGKGRH		SCG1	36294/367830	0.938	65408	0.790	0.004959	-1.05	0.3333	4	18	-1.2	3
HIP_65409	DLQVGQVEL	LCAGQVTAL	DLQVGQVELLCAGQVTAL		CMGA	12862/247050	0.991	65409	0.790	0.001185	2.1	0	-2	18	0.8278	-2
HIP_65410	GIVEQCCT	PGEEEEEEE	GIVEQCCTPGEEEEEEE		CMGA	5057/247050	0.998	65410	0.790	0.065733	-0.2	0.3333	-8	17	-1.2294	-8
HIP_65411	RGFFYTPKT	AENEPQSAP	RGFFYTPKTAENEPQSAP		SCG2	27892/334890	0.992	65411	0.790	-0.016033	-1.9	0.1667	0	18	-1.2167	0
HIP_65412	VEALYLVC	YRASEEEPE	VEALYLVCYRASEEEPE		SCG1	32400/367830	0.956	65412	0.790	0.078073	1.0833	0	-4	17	-0.2882	-4
HIP_65413	VEALYLVC	SAEFPDFY	VEALYLVCSAEFPDFY		SCG1	21096/367830	0.988	65413	0.790	0.107786	1.3333	0.1667	-3	16	0.7625	-3
HIP_65414	GSLQPLAL	GDSEGLSQG	GSLQPLALGDSEGLSQG		CMGA	11777/247050	0.992	65414	0.790	0.207620	0.7833	0.3333	-2	17	-0.1529	-2
HIP_65415	PGAGSLQPL	SQEKTHSRE	PGAGSLQPLSQEKTHSRE		SCG1	32298/367830	0.956	65415	0.790	0.033025	-1.5167	0.3333	1	18	-1.2944	0
HIP_65416	GERGFFYTP	LGEYLARML	GERGFFYTPLGEYLARML		SCG2	16864/334890	0.997	65416	0.790	-0.146038	-0.6167	0.3333	0	18	-0.0778	0
HIP_65417	GGGPGAGSL	AASFQRNQL	GGGPGAGSLAASFQRNQL		SCG2	8790/334890	0.999	65417	0.790	0.026644	0.9	0.5	1	18	-0.2444	1
HIP_65418	ERGFFYTPK	KDVMEKRED	ERGFFYTPKKDVMEKRED		CMGA	21144/247050	0.968	65418	0.790	-0.051127	-1.5667	0.1667	0	18	-1.6944	0
HIP_65419	QPLALEGSL	EPEYGEEIK	QPLALEGSLEPEYGEEIK		SCG1	14777/367830	0.995	65419	0.790	-0.079015	-1	0.5	-4	18	-0.7389	-4
HIP_65420	SHLVEALY	EAKTHWLGA	SHLVEALYEAKTHWLGA		SCG2	35521/334890	0.984	65420	0.790	0.210351	-0.2167	0	1	17	-0.0235	-1
HIP_65421	VEQCCTSIC	DWMRIILEA	VEQCCTSICDWMRIILEA		SCG2	17919/334890	0.997	65421	0.790	-0.047773	0.6167	0.1667	-2	18	0.6556	-2
HIP_65422	GPGAGSLQP	SDDYETQQW	GPGAGSLQPSDDYETQQW		SCG2	18892/334890	0.996	65422	0.790	-0.018578	-1.5167	0.3333	-3	18	-1.35	-3
HIP_65423	PGAGSLQPL	QECFETLRG	PGAGSLQPLQECFETLRG		CMGA	16151/247050	0.984	65423	0.790	-0.107505	-0.9667	0.1667	-1	18	-0.3278	-1
HIP_65424	HLCGSHLVE	NQSSQAELK	HLCGSHLVENQSSQAELK		CMGA	22710/247050	0.962	65424	0.790	0.188867	-0.55	0.1667	1	18	-0.5944	-1
HIP_65425	SHLVEALY	AEAKTHWLG	SHLVEALYAEAKTHWLG		SCG2	31662/334890	0.989	65425	0.790	0.086813	0.7333	0	1	17	-0.0235	-1
HIP_65426	QCCTSICSL	DLELQKIAE	QCCTSICSLDLELQKIAE		SCG1	21232/367830	0.988	65426	0.790	-0.025723	0.3833	0.1667	-2	18	0.3333	-2
HIP_65427	SHLVEALYL	NLNDKDQE	SHLVEALYLNLNDKDQE		SCG2	22099/334890	0.995	65427	0.790	0.176360	0.5167	0	-2	17	-0.7353	-3
HIP_65428	LVEALYLVC	SGAEAASF	LVEALYLVCSGAEAASF		SCG2	13431/334890	0.998	65428	0.790	0.164803	1.85	0.3333	-2	17	1.2941	-2
HIP_65429	GSLQPLAL	YPKTPGRA	GSLQPLALYPKTPGRA		SCG2	34844/334890	0.985	65429	0.790	0.220694	0.4333	0.1667	2	16	-0.3312	2
HIP_65430	PGAGSLQPL	YETQQWPER	PGAGSLQPLYETQQWPER		SCG2	25863/334890	0.993	65430	0.790	-0.048594	-1.1333	0.1667	-1	18	-1.2167	-1
HIP_65431	ICSLYQLEN	EEEEEEAT	ICSLYQLENEEEEEEAT		CMGA	16521/247050	0.983	65431	0.790	-0.021353	-2.2833	0	-7	17	-1.0529	-7
HIP_65432	VELGGGPGA	YGAGRSRSN	VELGGGPGAYGAGRSRSN		SCG2	8966/334890	0.999	65432	0.790	-0.005792	-0.0167	0.5	1	18	-0.6278	1
HIP_65433	AGSLQPLAL	AAVLALLLC	AGSLQPLALAAVLALLLC		CMGA	16415/247050	0.983	65433	0.790	0.015330	2.8667	0	0	18	2	0
HIP_65434	LVEALYLVC	PEDQELES	LVEALYLVCPEDQELES		CMGA	11909/247050	0.992	65434	0.790	0.134398	0.3167	0.1667	-5	17	0.1882	-5
HIP_65435	EQCCTSICS	EDLEWERYR	EQCCTSICSEDLEWERYR		SCG1	21434/367830	0.987	65435	0.790	-0.036822	0.5	0.1667	-3	18	-1.0389	-3
HIP_65436	LCGSHLVEA	EGNNQAPGE	LCGSHLVEAEGNNQAPGE		CMGA	13195/247050	0.990	65436	0.790	0.189516	-0.8167	0.1667	-2	18	-0.55	-3
HIP_65437	ALYLVCGER	GGEDWNPVE	ALYLVCGERGGEDWNPVE		SCG2	17429/334890	0.997	65437	0.790	-0.132952	-2.1167	0.5	-3	18	-0.3722	-3
HIP_65438	GGGPGAGSL	LAAVNSMPV	GGGPGAGSLLAAVNSMPV		SCG1	9309/367830	0.998	65438	0.790	0.122112	1.6667	0.3333	0	18	0.7222	0
HIP_65439	RGFFYTPKT	NAAGSGRLQ	RGFFYTPKTNAAGSGRLQ		SCG2	26004/334890	0.993	65439	0.790	0.135982	-1.0167	0.1667	3	18	-0.7333	3
HIP_65440	SHLVEALYL	TREEKRFL	SHLVEALYLTREEKRFL		SCG1	36649/367830	0.937	65440	0.790	0.266509	-0.4	0	1	17	-0.3176	0
HIP_65441	HLVEALYLV	YQLIEISR	HLVEALYLVYQLIEISR		SCG2	25041/334890	0.994	65441	0.790	0.046649	0.95	0	0	17	0.7529	-1
HIP_65442	YLVCGERGF	TPECRQVLK	YLVCGERGFTPECRQVLK		SCG1	26242/367830	0.977	65442	0.790	-0.021529	-1.3167	0.3333	1	18	-0.2222	1
HIP_65443	EAEDLQVGQ	SQQKEEEEE	EAEDLQVGQSQQKEEEEE		CMGA	7043/247050	0.997	65443	0.790	-0.135264	-1.25	0.3333	-7	18	-2.0722	-7
HIP_65444	YLVCGERGF	CFETLRGDE	YLVCGERGFCFETLRGDE		CMGA	9869/247050	0.995	65444	0.790	-0.014198	-0.05	0.1667	-2	18	-0.2111	-2
HIP_65445	QHLCGSHLV	VNAAGSGRL	QHLCGSHLVVNAAGSGRL		SCG2	27058/334890	0.992	65445	0.790	0.032393	1.2167	0	3	18	0.2889	1

HIP_65909	CGERGFFY	ATQRLANF	CGERGFFYATQRLANF		IAPP	4038/45018	0.956	65909	0.789	0.148517	0.3167	0	1	16	-0.25	1	3	0	[illegible]
HIP_65910	VEQCCTSIC	YFMSDTREE	VEQCCTSICYFMSDTREE		SCG1	14408/367830	0.995	65910	0.789	-0.027147	1.6	0.1667	-3	18	-0.3	-3	2	0	[illegible]
HIP_65911	CGERGFFY	APGKWQQQ	CGERGFFYAPGKWQQQ		SCG1	19413/367830	0.990	65911	0.789	0.175193	0.6833	0.3333	1	16	-1.0938	1	3	0	[illegible]
HIP_65912	LVEALYLVC	GQGSSEDD	LVEALYLVCGQGSSEDD		SCG2	14488/334890	0.998	65912	0.789	0.182946	1.0333	0.3333	-4	17	0.1706	-4	1	1	[illegible]
HIP_65913	SICSLYQLE	GERGEDSSE	SICSLYQLEGERGEDSSE		SCG1	26135/367830	0.977	65913	0.789	-0.046242	-1.9333	0.1667	-4	18	-0.9	-4	1	0	[illegible]
HIP_65914	ICSLYQLEN	RAYGFRGPG	ICSLYQLENRAYGFRGPG		CMGA	29137/247050	0.921	65914	0.789	0.060920	-1.2	0	1	18	-0.3611	1	2	0	[illegible]
HIP_65915	CGERGFFYT	VGSNTYGKR	CGERGFFYTVGSNTYGKR		IAPP	2369/45018	0.987	65915	0.789	0.026696	0.6333	0.3333	2	18	-0.7778	2	3	0	[illegible]
HIP_65916	EQCCTSICS	ERKLKHMQF	EQCCTSICSERKLKHMQF		SCG2	43585/334890	0.968	65916	0.789	0.140464	-0.95	0.1667	2	18	-0.6278	1	0	0	[illegible]
HIP_65917	ICSLYQLEN	WSKMDQLAK	ICSLYQLENWSKMDQLAK		CMGA	27960/247050	0.931	65917	0.789	-0.010227	-1.4667	0.1667	0	18	-0.3889	0	2	0	[illegible]
HIP_65918	GSLQPLAL	AGSLQPLA	GSLQPLALAGSLQPLA		INSC	228/5184	0.997	65918	0.789	0.173846	1.6667	0.3333	0	16	0.7375	0	0	0	[illegible]
HIP_65919	CTSICSLYQ	KKELENLAA	CTSICSLYQKKELENLAA		SCG1	43951/367830	0.895	65919	0.789	0.130580	-2.05	0	0	18	-0.05	0	1	0	[illegible]
HIP_65920	GIVEQCCTS	PNGSVEPER	GIVEQCCTSPNGSVEPER		SCG2	10581/334890	0.999	65920	0.789	-0.007104	-0.75	0.5	-2	18	-0.5778	-2	0	0	[illegible]
HIP_65921	VCGERGFF	PLQQKENG	VCGERGFFPLQQKENG		SCG2	18938/334890	0.996	65921	0.789	0.030093	0.65	0.3333	0	16	-0.7875	0	2	0	[illegible]
HIP_65922	HLCGSHLVE	ENHPQESKG	HLCGSHLVEENHPQESKG		SCG1	46251/367830	0.879	65922	0.789	0.072067	-0.95	0	1	18	-1.15	-2	0	0	[illegible]
HIP_65923	HLCGSHLV	VALNHLKAT	HLCGSHLVVALNHLKAT		IAPP	3820/45018	0.962	65923	0.789	0.037077	2.4333	0	4	17	0.6353	1	0	1	[illegible]
HIP_65924	TSICSLYQL	YFMSDTREE	TSICSLYQLYFMSDTREE		SCG1	20570/367830	0.989	65924	0.789	-0.044961	0.4	0	-2	18	-0.3111	-2	3	0	[illegible]
HIP_65925	GERGFFYTP	TQRLANFLV	GERGFFYTPTQRLANFLV		IAPP	4478/45018	0.945	65925	0.789	-0.014899	-2.05	0.1667	1	18	-0.1444	1	3	0	[illegible]
HIP_65926	QHLCGSHLV	SRDNPFKRT	QHLCGSHLVSRDNPFKRT		SCG2	32795/334890	0.987	65926	0.789	0.255625	-0.6667	0.1667	4	18	-0.9444	2	0	0	[illegible]
HIP_65927	SLYQLENYC	DDTPNRQYW	SLYQLENYCDDTPNRQYW		SCG2	25319/334890	0.993	65927	0.789	0.078915	-1.6667	0	-2	18	-1.4889	-2	1	0	[illegible]
HIP_65928	EQCCTSICS	NGERATRLF	EQCCTSICSNGERATRLF		SCG2	17365/334890	0.997	65928	0.789	0.152269	-0.2	0.3333	0	18	-0.3333	0	0	0	[illegible]
HIP_65929	NQHLCGSHL	RMLSKSGYP	NQHLCGSHLRMLSKSGYP		SCG2	39060/334890	0.979	65929	0.789	0.053072	0.1667	0.1667	4	18	-0.6722	2	0	0	[illegible]
HIP_65930	ELGGGPGAG	REEEEEEEE	ELGGGPGAGREEEEEEEE		CMGA	9775/247050	0.995	65930	0.789	-0.100655	-1.75	0.3333	-8	18	-1.8889	-8	0	0	[illegible]
HIP_65931	ICSLYQLEN	NNFGAILSS	ICSLYQLENNNFGAILSS		IAPP	3403/45018	0.970	65931	0.789	-0.038492	-1.2333	0	-1	18	0.3278	-1	2	0	[illegible]
HIP_65932	CTSICSLYQ	QGLVDREKG	CTSICSLYQQGLVDREKG		CMGA	9587/247050	0.995	65932	0.789	-0.032113	-0.1833	0.1667	0	18	-0.3056	0	1	0	[illegible]
HIP_65933	TSICSLYQ	EEEDNRDSS	TSICSLYQEEEDNRDSS		CMGA	17665/247050	0.980	65933	0.789	0.072965	-1.9167	0	-4	17	-1.3765	-4	1	1	[illegible]
HIP_65934	CGERGFFY	RNQLLQKE	CGERGFFYRNQLLQKE		SCG2	33031/334890	0.987	65934	0.789	0.189569	-1.2	0	1	16	-1.05	1	3	0	[illegible]
HIP_65935	PLALEGSLQ	EGNNQAPGE	PLALEGSLQEGNNQAPGE		CMGA	7066/247050	0.997	65935	0.789	-0.078727	-1.3167	0.3333	-3	18	-0.8167	-3	0	0	[illegible]
HIP_65936	PGAGSLQPL	TLALSLLVC	PGAGSLQPLTLALSLLVC		NPY	1897/49410	0.988	65936	0.789	0.015927	0.6	0.1667	0	18	1.2944	0	0	0	[illegible]
HIP_65937	VEALYLVCG	PPKNDDTPN	VEALYLVCGPPKNDDTPN		SCG2	18499/334890	0.997	65937	0.789	0.096336	-0.1333	0.5	-2	18	-0.4611	-2	1	0	[illegible]
HIP_65938	LVCGERGFF	RGRGSEEY	LVCGERGFFRGRGSEEY		SCG1	35948/367830	0.940	65938	0.789	0.136221	-0.7	0.3333	0	17	-0.6824	0	2	1	[illegible]
HIP_65939	LVCGERGFF	ALEYIENL	LVCGERGFFALEYIENL		SCG2	15776/334890	0.997	65939	0.789	-0.016869	1.2167	0.1667	-2	17	0.5529	-2	3	1	[illegible]
HIP_65940	VCGERGFF	YGEEIKGY	VCGERGFFYGEEIKGY		SCG1	25655/367830	0.978	65940	0.789	0.057533	0	0.3333	-1	16	-0.3938	-1	3	0	[illegible]
HIP_65941	QVELGGGPG	AYPSKPDNP	QVELGGGPGAYPSKPDNP		NPY	627/49410	0.997	65941	0.789	0.079333	-0.5833	0.6667	-1	18	-1.0111	-1	1	0	[illegible]
HIP_65942	YLVCGERGF	KDQELGEYL	YLVCGERGFKDQELGEYL		SCG2	28380/334890	0.991	65942	0.789	0.014610	-2.1667	0.1667	-2	18	-0.4889	-2	1	0	[illegible]
HIP_65943	GERGFFYTP	EYNYDWWE	GERGFFYTPEYNYDWWE		SCG1	27731/367830	0.973	65943	0.789	-0.031812	-1.9833	0.1667	-3	17	-1.4824	-3	5	1	[illegible]
HIP_65944	SHLVEALY	WIPHVENRQ	SHLVEALYWIPHVENRQ		SCG2	17940/334890	0.997	65944	0.789	0.011583	1.05	0.1667	1	17	-0.4235	-1	2	1	[illegible]
HIP_65945	LGGGPGAGS	ASFQRNQLL	LGGGPGAGSASFQRNQLL		SCG2	9248/334890	0.999	65945	0.789	0.034288	0.7333	0.5	1	18	-0.1333	1	1	0	[illegible]
HIP_65946	PLALEGSLQ	GEPRAYFMS	PLALEGSLQGEPRAYFMS		SCG1	10317/367830	0.998	65946	0.789	-0.017622	-1	0.5	-1	18	-0.1222	-1	0	0	[illegible]
HIP_65947	SHLVEALY	LSLLGAVGL	SHLVEALYLSLLGAVGL		SCG1	23671/367830	0.983	65947	0.789	0.030959	1.85	0.1667	0	17	1.4353	-1	1	1	[illegible]
HIP_65948	AGSLQPLAL	EEPVSTHQ	AGSLQPLALEEPVSTHQ		SCG1	20163/367830	0.989	65948	0.789	0.156004	0.1333	0.1667	-1	17	-0.2294	-2	0	1	[illegible]
HIP_65949	VCGERGFFY	NIAYEDVV	VCGERGFFYNIAYEDVV		SCG2	19741/334890	0.996	65949	0.789	0.011508	1.1833	0	-2	17	0.3	-2	4	1	[illegible]
HIP_65950	IVEQCCTSI	AFLNERKQA	IVEQCCTSIAFLNERKQA		SCG1	14297/367830	0.995	65950	0.789	0.098192	1.9	0.1667	0	18	0.0556	0	1	0	[illegible]
HIP_65951	TSICSLYQL	EENELTLN	TSICSLYQLEENELTLN		SCG1	26301/367830	0.977	65951	0.789	0.113938	-1.9167	0	-3	17	-0.1824	-3	1	1	[illegible]
HIP_65952	GERGFFYT	AANQKTSY	GERGFFYTAANQKTSY		SCG2	29741/334890	0.990	65952	0.789	0.007755	0.15	0	1	16	-0.9563	1	3	0	[illegible]
HIP_65953	PGAGSLQPL	IAYLKRLVN	PGAGSLQPLIAYLKRLVN		SCG2	15224/334890	0.998	65953	0.789	0.081222	0.6167	0.1667	2	18	0.3333	2	1	0	[illegible]
HIP_65954	TSICSLYQL	EAVPEEEGP	TSICSLYQLEAVPEEEGP		CMGA	14015/247050	0.989	65954	0.789	-0.075787	0.25	0	-4	18	-0.2278	-4	1	0	[illegible]
HIP_65955	TSICSLYQL	SEDDLQEEE	TSICSLYQLSEDDLQEEE		SCG2	15348/334890	0.998	65955	0.789	0.076343	-1.4667	0.1667	-6	18	-0.7778	-6	1	0	[illegible]
HIP_65956	SHLVEALY	EEDWMRIIL	SHLVEALYEEDWMRIIL		SCG2	24241/334890	0.994	65956	0.789	0.143492	-1.0333	0	-2	17	0.2118	-3	2	1	[illegible]
[illegible]																			

HIP_66420	TSICSLYQL	AVPEEEGPT	TSICSLYQLAVPEEEGPT		CMGA	7074/247050	0.997	66420	0.789	-0.120207
HIP_66421	EALYLVCGE	SKRFPVGPP	EALYLVCGESKRFPVGPP		SCG2	37465/334890	0.981	66421	0.789	-0.010637
HIP_66422	TSICSLYQL	YGEEIKGY	TSICSLYQLYGEEIKGY		SCG1	24341/367830	0.982	66422	0.789	0.114813
HIP_66423	SHLVEALY	VSEEVKTRH	SHLVEALYVSEEVKTRH		SCG1	26519/367830	0.976	66423	0.789	0.182344
HIP_66424	GERGFFYT	GPTKADTE	GERGFFYTGPTKADTE		SCG1	16299/367830	0.994	66424	0.789	0.150598
HIP_66425	VNQHLCGSH	SEEEPEYGE	VNQHLCGSHSEEEPEYGE		SCG1	19627/367830	0.990	66425	0.789	-0.145227
HIP_66426	HLCGSHLV	ADEPQWSLY	HLCGSHLVADEPQWSLY		SCG1	26512/367830	0.976	66426	0.789	0.117970
HIP_66427	ICSLYQLEN	RPQSEESWD	ICSLYQLENRPQSEESWD		SCG1	33155/367830	0.953	66427	0.789	0.027222
HIP_66428	ERGFFYTPK	AEAASFQRN	ERGFFYTPKAEAASFQRN		SCG2	24071/334890	0.994	66428	0.789	0.041879
HIP_66429	YLVCGERGF	AGRSRSNQL	YLVCGERGFAGRSRSNQL		SCG2	28768/334890	0.991	66429	0.789	0.003323
HIP_66430	HLVEALYLV	FQRNQLLQ	HLVEALYLVFQRNQLLQ		SCG2	32625/334890	0.988	66430	0.789	0.145957
HIP_66431	GGGPGAGSL	RYYSALRHY	GGGPGAGSLRYYSALRHY		NPY	4584/49410	0.944	66431	0.789	0.093122
HIP_66432	CGSHLVEAL	SEEEPEYGE	CGSHLVEALSEEEPEYGE		SCG1	18464/367830	0.991	66432	0.789	0.056881
HIP_66433	AGSLQPLAL	GTEALPQG	AGSLQPLALGTEALPQG		SCG2	16354/334890	0.997	66433	0.789	0.205592
HIP_66434	LCGSHLVEA	ESEEGEEDA	LCGSHLVEAESEEGEEDA		SCG1	18974/367830	0.991	66434	0.789	0.079820
HIP_66435	ALYLVCGER	QESKGQPRS	ALYLVCGERQESKGQPRS		SCG1	40189/367830	0.917	66435	0.789	-0.064265
HIP_66436	EALYLVCGE	YWDEDLLMK	EALYLVCGEYWDEDLLMK		SCG2	13762/334890	0.998	66436	0.789	-0.057268
HIP_66437	LYQLENYCN	ERKQASAIK	LYQLENYCNERKQASAIK		SCG1	49530/367830	0.855	66437	0.789	0.180800
HIP_66438	CSLYQLENY	RMDEEQKLY	CSLYQLENYRMDEEQKLY		SCG2	35355/334890	0.984	66438	0.789	-0.051656
HIP_66439	VCGERGFF	KSSAPPITP	VCGERGFFKSSAPPITP		SCG1	31982/367830	0.958	66439	0.789	0.156865
HIP_66440	QHLCGSHLV	GKGEQEHSQ	QHLCGSHLVGKGEQEHSQ		CMGA	16213/247050	0.984	66440	0.789	0.220376
HIP_66441	SICSLYQLE	QSLATLESV	SICSLYQLEQSLATLESV		SCG2	18600/334890	0.997	66441	0.789	-0.185136
HIP_66442	AGSLQPLAL	PERELDLP	AGSLQPLALPERELDLP		SCG2	23966/334890	0.994	66442	0.789	0.225846
HIP_66443	AGSLQPLAL	VGQVELGGG	AGSLQPLALVGQVELGGG		INSC	212/5184	0.997	66443	0.789	0.122761
HIP_66444	AGSLQPLAL	PPITPECR	AGSLQPLALPPITPECR		SCG1	18069/367830	0.992	66444	0.789	0.188606
HIP_66445	ERGFFYTP	GEQEHSQQK	ERGFFYTPGEQEHSQQK		CMGA	16636/247050	0.983	66445	0.789	-0.014429
HIP_66446	VCGERGFFY	VNAAGSGR	VCGERGFFYVNAAGSGR		SCG2	25439/334890	0.993	66446	0.789	0.029513
HIP_66447	LGGGPGAGS	ENTKFEVRL	LGGGPGAGSENTKFEVRL		SCG1	23481/367830	0.984	66447	0.789	-0.047491
HIP_66448	ICSLYQLEN	QGAKERAHQ	ICSLYQLENQGAKERAHQ		CMGA	19541/247050	0.974	66448	0.789	-0.029266
HIP_66449	GSLQPLALE	QVSEEVKTR	GSLQPLALEQVSEEVKTR		SCG1	28414/367830	0.971	66449	0.789	0.039687
HIP_66450	LQPLALEGS	PYQGVSVPL	LQPLALEGSPYQGVSVPL		SCG2	13998/334890	0.998	66450	0.789	-0.054338
HIP_66451	CGSHLVEAL	EEEEEEEE	CGSHLVEALEEEEEEEE		CMGA	12813/247050	0.991	66451	0.789	0.000056
HIP_66452	CGSHLVEAL	EEEEEEEE	CGSHLVEALEEEEEEEE		CMGA	12814/247050	0.991	66452	0.789	0.000056
HIP_66453	CGSHLVEAL	EEEEEEEE	CGSHLVEALEEEEEEEE		CMGA	12815/247050	0.991	66453	0.789	0.000056
HIP_66454	LVCGERGFF	SEADLDHP	LVCGERGFFSEADLDHP		SCG2	18256/334890	0.997	66454	0.789	0.014347
HIP_66455	LCGSHLVEA	RSQEESEEG	LCGSHLVEARSQEESEEG		SCG1	33381/367830	0.952	66455	0.789	0.143802
HIP_66456	SHLVEALY	GEDWNPVEE	SHLVEALYGEDWNPVEE		SCG2	18509/334890	0.997	66456	0.789	0.176735
HIP_66457	LYQLENYCN	TLSKPSPMP	LYQLENYCNTLSKPSPMP		CMGA	15717/247050	0.985	66457	0.789	-0.049695
HIP_66458	QHLCGSHLV	LSDDVSKVI	QHLCGSHLVLSDDVSKVI		SCG2	20653/334890	0.996	66458	0.789	0.145168
HIP_66459	LYQLENYCN	EKRNLARVP	LYQLENYCNEKRNLARVP		SCG1	49531/367830	0.855	66459	0.789	0.180885
HIP_66460	YLVCGERGF	QEEVRDSKE	YLVCGERGFQEEVRDSKE		SCG2	24513/334890	0.994	66460	0.789	-0.047771
HIP_66461	GSLQPLAL	DEMKRSGQL	GSLQPLALDEMKRSGQL		SCG2	25426/334890	0.993	66461	0.789	0.125590
HIP_66462	HLVEALYL	EYIENLRQ	HLVEALYLEYIENLRQ		SCG2	27724/334890	0.992	66462	0.789	0.082912
HIP_66463	TSICSLYQL	PQAEGDSEG	TSICSLYQLPQAEGDSEG		CMGA	9366/247050	0.995	66463	0.789	-0.010465
HIP_66464	QHLCGSHLV	SKRWSKMDQ	QHLCGSHLVSKRWSKMDQ		CMGA	27545/247050	0.934	66464	0.789	0.236350
HIP_66465	GAGSLQPLA	DDYETQQWP	GAGSLQPLADDYETQQWP		SCG2	20391/334890	0.996	66465	0.789	-0.034810
HIP_66466	ERGFFYTPK	EALRQAENE	ERGFFYTPKEALRQAENE		SCG2	21955/334890	0.995	66466	0.789	0.039824
HIP_66467	GGGPGAGSL	EPQWSLYP	GGGPGAGSLEPQWSLYP		SCG1	20132/367830	0.989	66467	0.789	-0.010468
HIP_66468	ICSLYQLEN	EEEEDNRD	ICSLYQLENEEEEDNRD		CMGA	18825/247050	0.976	66468	0.789	-0.013501
HIP_66469	HLVEALYL	ENTKFEVR	HLVEALYLENTKFEVR		SCG1	28583/367830	0.970	66469	0.789	0.235571
HIP_66470	ICSLYQLE	EEEEMAVVP	ICSLYQLEEEEEMAVVP		CMGA	19358/247050	0.974	66470	0.789	-0.011058
HIP_66471	EQCCTSIC	EEEEEEATN	EQCCTSICEEEEEEATN		CMGA	12822/247050	0.991	66471	0.789	0.005156
HIP_66472	SHLVEALY	QKGERGEDS	SHLVEALYQKGERGEDS		SCG1	28201/367830	0.971	66472	0.789	0.239876
[illegible]	[illegible]	[illegible]	[illegible]		[illegible]	[illegible]	[illegible]	[illegible]	[illegible]	[illegible]

HIP_66931	SICSLYQLE	PLIFLISGA	SICSLYQLEPLIFLISGA		SCG2	14841/334890	0.998	66931	0.788	-0.113265	0.5833	0.1667	-1	18	1.2833	-1	2	0	0
HIP_66932	CGERGFFYT	LCAGQVTA	CGERGFFYTLCAGQVTA		CMGA	15517/247050	0.986	66932	0.788	-0.039956	1.4833	0	0	17	0.4	0	3	1	0
HIP_66933	QCCTSICSL	GEGHHRVQ	QCCTSICSLGEGHHRVQ		SCG1	19043/367830	0.991	66933	0.788	0.200589	0.2	0.5	2	17	-0.2647	0	0	1	0
HIP_66934	GSLQPLAL	ENYQKGER	GSLQPLALENYQKGER		SCG1	31899/367830	0.958	66934	0.788	0.174952	0.1833	0	0	16	-1.075	0	1	0	0
HIP_66935	LYLVCGERG	SYFPNPYNQ	LYLVCGERGSYFPNPYNQ		SCG2	16767/334890	0.997	66935	0.788	0.029393	-1.2833	0.3333	0	18	-0.5611	0	2	0	0
HIP_66936	ELGGGPGAG	EDQELESLS	ELGGGPGAGEDQELESLS		CMGA	8560/247050	0.996	66936	0.788	-0.117042	-1.5833	0.3333	-5	18	-0.7222	-5	0	0	1
HIP_66937	GAGSLQP	LALEGSL	GAGSLQPLALEGSL		INSC	156/5184	0.998	66937	0.788	0.115023	1.35	0.1667	-1	14	0.5286	-1	0	0	0
HIP_66938	LVEALYLVC	NFGAILSS	LVEALYLVCNFGAILSS		IAPP	2568/45018	0.984	66938	0.788	0.143693	1.5667	0.1667	-1	17	1.5706	-1	2	1	0
HIP_66939	QHLCGSHLV	LPERDSLSE	QHLCGSHLVLPERDSLSE		SCG2	15540/334890	0.998	66939	0.788	0.117329	0.5833	0.1667	0	18	-0.4111	-2	0	0	0
HIP_66940	GSLQPLAL	QEEVRDSK	GSLQPLALQEEVRDSK		SCG2	22059/334890	0.995	66940	0.788	0.144666	-0.1833	0	-1	16	-0.7563	-1	0	0	0
HIP_66941	ALYLVCGER	NPYYDPLQW	ALYLVCGERNPYYDPLQW		SCG1	20331/367830	0.989	66941	0.788	-0.099377	-2.4667	0.3333	-1	18	-0.3889	-1	2	0	0
HIP_66942	GPGAGSLQP	GGGHSRERA	GPGAGSLQPGGGHSRERA		SCG1	13059/367830	0.996	66942	0.788	0.078572	-0.4167	0.6667	2	18	-1.0556	1	0	0	1
HIP_66943	HLCGSHLVE	PGEDAPAED	HLCGSHLVEPGEDAPAED		NPY	1668/49410	0.990	66943	0.788	0.133110	-0.1667	0.3333	-3	18	-0.6	-5	0	0	0
HIP_66944	CGERGFFY	IYKANNIAY	CGERGFFYIYKANNIAY		SCG2	35321/334890	0.984	66944	0.788	0.103623	0.6	0	1	17	-0.1706	1	4	1	0
HIP_66945	SICSLYQLE	VNAAGSGRL	SICSLYQLEVNAAGSGRL		SCG2	19697/334890	0.996	66945	0.788	-0.099872	-0.1167	0	0	18	0.3722	0	1	0	0
HIP_66946	CSLYQLENY	EEGEEDATS	CSLYQLENYEEGEEDATS		SCG1	21272/367830	0.987	66946	0.788	-0.057694	-2.6167	0.1667	-6	18	-1.1889	-6	1	0	0
HIP_66947	CSLYQLENY	EGEEDATSE	CSLYQLENYEGEEDATSE		SCG1	21273/367830	0.987	66947	0.788	-0.057694	-2.6167	0.1667	-6	18	-1.1889	-6	1	0	0
HIP_66948	GSLQPLAL	ANQKTSYFP	GSLQPLALANQKTSYFP		SCG2	30223/334890	0.990	66948	0.788	0.205904	0.7	0	1	17	-0.2235	1	0	1	0
HIP_66949	ICSLYQLEN	ETGSQENHP	ICSLYQLENETGSQENHP		SCG1	19221/367830	0.991	66949	0.788	0.002621	-1.3	0.1667	-2	18	-1.0389	-3	1	0	0
HIP_66950	GSLQPLAL	VLSVALNHL	GSLQPLALVLSVALNHL		IAPP	2994/45018	0.978	66950	0.788	0.041745	2.7667	0.1667	1	17	1.2353	0	0	1	0
HIP_66951	CGSHLVEAL	RSSQGGSLP	CGSHLVEALRSSQGGSLP		SCG1	35027/367830	0.945	66951	0.788	0.185255	-0.6667	0.3333	1	18	-0.0444	0	0	0	0
HIP_66952	CTSICSLYQ	QWSLYPSD	CTSICSLYQQWSLYPSD		SCG1	26487/367830	0.976	66952	0.788	-0.001706	-1.0333	0.1667	-1	17	-0.1412	-1	2	1	0
HIP_66953	QHLCGSHLV	EKNEQINDE	QHLCGSHLVEKNEQINDE		SCG2	27417/334890	0.992	66953	0.788	0.212269	-1.0167	0	-1	18	-1.15	-3	0	0	0
HIP_66954	GSLQPLAL	SGELEQEEE	GSLQPLALSGELEQEEE		CMGA	9583/247050	0.995	66954	0.788	0.175618	0.7833	0.3333	-5	17	-0.6765	-5	0	1	1
HIP_66955	LYQLENYCN	LISGAEAAS	LYQLENYCNLISGAEAAS		SCG2	12496/334890	0.998	66955	0.788	-0.151419	0.8667	0.1667	-2	18	0.0944	-2	1	0	0
HIP_66956	CGERGFFY	MGILKLQV	CGERGFFYMGILKLQV		IAPP	2262/45018	0.988	66956	0.788	0.010096	1.7167	0.1667	1	16	0.525	1	3	0	0
HIP_66957	NQHLCGSHL	NFGAILSST	NQHLCGSHLNFGAILSST		IAPP	2274/45018	0.988	66957	0.788	0.004045	-0.2167	0.3333	2	18	0.1222	0	1	0	0
HIP_66958	FVNQHLCGS	KEELVARSE	FVNQHLCGSKEELVARSE		SCG1	30722/367830	0.963	66958	0.788	-0.087108	-1.6	0.3333	0	18	-0.4444	-1	0	0	0
HIP_66959	IVEQCCTSI	PEPMQESKA	IVEQCCTSIPEPMQESKA		CMGA	7316/247050	0.997	66959	0.788	-0.046518	-0.6167	0.5	-2	18	-0.2778	-2	0	0	0
HIP_66960	GPGAGSLQP	RDSKENIEK	GPGAGSLQPRDSKENIEK		SCG2	35176/334890	0.985	66960	0.788	0.055069	-1.6833	0.3333	0	18	-1.4278	0	0	0	0
HIP_66961	GSLQPLAL	ASEEEPE	GSLQPLALASEEEPE		SCG1	15918/367830	0.994	66961	0.788	0.124246	1.15	0.1667	-4	15	-0.5133	-4	0	1	1
HIP_66962	GGPGAGSLQ	RGWRPSSRE	GGPGAGSLQRGWRPSSRE		CMGA	25214/247050	0.949	66962	0.788	-0.019404	-1.05	0.3333	2	18	-1.3	2	1	0	0
HIP_66963	CTSICSLY	SQEKTHSRE	CTSICSLYSQEKTHSRE		SCG1	32961/367830	0.953	66963	0.788	0.172615	-1.0167	0.3333	1	17	-0.8647	0	1	1	0
HIP_66964	LVEALYLV	YDPLQWKS	LVEALYLVYDPLQWKS		SCG1	24431/367830	0.981	66964	0.788	0.121644	0.05	0.1667	-1	16	0.3187	-1	2	0	0
HIP_66965	GPGAGSLQP	AYFMSDTRE	GPGAGSLQPAYFMSDTRE		SCG1	10323/367830	0.998	66965	0.788	0.042692	0.3333	0.1667	-1	18	-0.6056	-1	2	0	0
HIP_66966	CGSHLVEAL	RDNPFKRTN	CGSHLVEALRDNPFKRTN		SCG2	43922/334890	0.967	66966	0.788	0.174512	-1.5667	0	2	18	-0.8167	1	0	0	0
HIP_66967	GIVEQCCT	GEQEHSQQK	GIVEQCCTGEQEHSQQK		CMGA	14115/247050	0.989	66967	0.788	0.172175	-0.5167	0.1667	-1	17	-1.1882	-2	0	1	0
HIP_66968	LVEALYLV	ETQNAFLNE	LVEALYLVETQNAFLNE		SCG1	21558/367830	0.987	66968	0.788	0.156260	-0.1667	0	-3	17	0.4118	-3	1	1	0
HIP_66969	SHLVEALY	LTLALSLLV	SHLVEALYLTLALSLLV		NPY	3235/49410	0.972	66969	0.788	0.004018	1.8667	0	0	17	1.6647	-1	1	1	0
HIP_66970	HLVEALYL	SMASLGEKR	HLVEALYLSMASLGEKR		SCG1	25069/367830	0.980	66970	0.788	0.036633	1.5333	0.1667	1	17	0.1765	0	1	1	0
HIP_66971	GGPGAGSLQ	ERATRLFEK	GGPGAGSLQERATRLFEK		SCG2	43173/334890	0.969	66971	0.788	-0.052927	-1.1167	0.1667	1	18	-0.7833	1	0	0	0
HIP_66972	GAGSLQPLA	QEEEQIEQA	GAGSLQPLAQEEEQIEQA		SCG2	16420/334890	0.997	66972	0.788	-0.011635	-1.0833	0.1667	-4	18	-0.7611	-4	0	0	1
HIP_66973	GSLQPLAL	SSSRGEAGA	GSLQPLALSSSRGEAGA		SCG1	22245/367830	0.986	66973	0.788	0.235664	1.1667	0.5	0	17	-0.0412	0	0	1	1

HIP_67442	VEALYLVCG	QALPEPMQE	VEALYLVCGQALPEPMQE		CMGA	6443/247050	0.998	67442	0.788	-0.067805	1.4	0.1667	-3	18	0.3	-3	1	0	0	[illegible]
HIP_67443	LYQLENYCN	LQIPPEDLI	LYQLENYCNLQIPPEDLI		SCG2	11906/334890	0.998	67443	0.788	-0.172897	0.4167	0	-3	18	-0.2	-3	1	0	0	[illegible]
HIP_67444	LVEALYLVC	QYWDEDLL	LVEALYLVCQYWDEDLL		SCG2	24572/334890	0.994	67444	0.788	0.047654	0.8	0	-4	17	0.6294	-4	3	1	0	[illegible]
HIP_67445	CSLYQLENY	PQAEGDSEG	CSLYQLENYPQAEGDSEG		CMGA	11983/247050	0.992	67445	0.788	-0.151881	-1.9333	0.1667	-4	18	-1.0667	-4	1	0	0	[illegible]
HIP_67446	NQHLCGSHL	DTEKWAEGG	NQHLCGSHLDTEKWAEGG		SCG1	30662/367830	0.963	67446	0.788	0.074620	-1.3167	0.1667	0	18	-1.0833	-2	0	0	0	[illegible]
HIP_67447	GAGSLQPLA	NEKNFFPEY	GAGSLQPLANEKNFFPEY		SCG1	28682/367830	0.970	67447	0.788	0.068418	-1.15	0.1667	-1	18	-0.5944	-1	0	0	0	[illegible]
HIP_67448	GERGFFYT	LSKSGYPKT	GERGFFYTLSKSGYPKT		SCG2	36454/334890	0.983	67448	0.788	0.099171	-0.0167	0.1667	2	17	-0.8706	2	3	1	0	[illegible]
HIP_67449	GGGPGAGSL	EEPVSTHQE	GGGPGAGSLEEPVSTHQE		SCG1	13782/367830	0.996	67449	0.788	-0.109118	-1	0.5	-2	18	-0.8278	-3	0	0	1	[illegible]
HIP_67450	QHLCGSHLV	EAVEEPSSK	QHLCGSHLVEAVEEPSSK		CMGA	19804/247050	0.973	67450	0.788	0.030056	1.2167	0	0	18	-0.4667	-2	0	0	0	[illegible]
HIP_67451	AGSLQPLAL	PSPEMIRA	AGSLQPLALPSPEMIRA		SCG2	19372/334890	0.996	67451	0.788	0.227284	0.9	0.5	0	17	0.2882	0	0	1	0	[illegible]
HIP_67452	VELGGGPGA	NTKFEVRLL	VELGGGPGANTKFEVRLL		SCG1	26933/367830	0.975	67452	0.788	-0.037741	-1.3833	0.3333	0	18	0.0889	0	1	0	1	[illegible]
HIP_67453	CGSHLVEAL	RRTRPRHHH	CGSHLVEALRRTRPRHHH		SCG1	50725/367830	0.846	67453	0.788	0.140534	-1.2667	0	7	18	-1.2056	3	0	0	1	[illegible]
HIP_67454	GPGAGSLQP	YQLIEISRN	GPGAGSLQPYQLIEISRN		SCG2	15101/334890	0.998	67454	0.788	-0.049055	-0.3833	0.1667	0	18	-0.4111	0	1	0	0	[illegible]
HIP_67455	GGPGAGSLQ	LEYIENLRQ	GGPGAGSLQLEYIENLRQ		SCG2	19252/334890	0.996	67455	0.788	-0.112964	-0.25	0.1667	-1	18	-0.5333	-1	1	0	0	[illegible]
HIP_67456	ELGGGPGAG	ERGFFYTP	ELGGGPGAGERGFFYTP		INSB	277/4968	0.956	67456	0.788	-0.055571	-1.2333	0.5	-1	17	-0.4647	-1	1	1	1	[illegible]
HIP_67457	VCGERGFF	REEEEEEE	VCGERGFFREEEEEEE		CMGA	16394/247050	0.983	67457	0.788	0.045970	-1.05	0.1667	-6	16	-1.5938	-6	2	0	5	[illegible]
HIP_67458	NQHLCGSHL	GPPKNDDTP	NQHLCGSHLGPPKNDDTP		SCG2	13065/334890	0.998	67458	0.788	0.117910	-0.6333	0.6667	1	18	-1.3778	-1	0	0	0	[illegible]
HIP_67459	EDLQVGQVE	LFEKPLDSQ	EDLQVGQVELFEKPLDSQ		SCG2	16568/334890	0.997	67459	0.788	-0.133976	0.05	0	-4	18	-0.6722	-4	1	0	0	[illegible]
HIP_67460	EDLQVGQVE	AAWIPHVEN	EDLQVGQVEAAWIPHVEN		SCG2	10483/334890	0.999	67460	0.788	-0.065549	-0.0167	0	-3	18	-0.3389	-4	1	0	0	[illegible]
HIP_67461	ERGFFYTP	QYSSHHTAE	ERGFFYTPQYSSHHTAE		SCG1	28856/367830	0.969	67461	0.788	-0.070969	-1.5333	0.3333	1	17	-1.2706	-1	3	1	0	[illegible]
HIP_67462	TSICSLYQL	MQPTLLLSL	TSICSLYQLMQPTLLLSL		SCG1	22031/367830	0.986	67462	0.788	0.037929	-0.7	0.1667	0	18	1	0	1	0	1	[illegible]
HIP_67463	GERGFFYT	DEKKELEN	GERGFFYTDEKKELEN		SCG1	35053/367830	0.944	67463	0.788	0.128946	-1.6833	0	-2	16	-1.6687	-2	3	0	0	[illegible]
HIP_67464	ALYLVCGER	YGEEGAPGK	ALYLVCGERYGEEGAPGK		SCG1	26160/367830	0.977	67464	0.788	-0.094158	-2.2667	0.3333	-1	18	-0.3778	-1	1	0	0	[illegible]
HIP_67465	CTSICSLYQ	LLGMESAAN	CTSICSLYQLLGMESAAN		SCG2	12583/334890	0.998	67465	0.788	-0.117469	1.0333	0.1667	-1	18	0.6167	-1	1	0	0	[illegible]
HIP_67466	SICSLYQL	AYLKRLVN	SICSLYQLAYLKRLVN		SCG2	27747/334890	0.992	67466	0.788	0.026092	0.55	0	2	16	0.5375	2	2	0	0	[illegible]
HIP_67467	GERGFFYT	EEMAVVPQG	GERGFFYTEEMAVVPQG		CMGA	20339/247050	0.971	67467	0.788	0.006932	-0.7167	0	-2	17	-0.3294	-2	3	1	0	[illegible]
HIP_67468	GGPGAGSLQ	TPECRQVLK	GGPGAGSLQTPECRQVLK		SCG1	29074/367830	0.969	67468	0.788	-0.073879	-1.05	0.3333	1	18	-0.5056	1	0	0	0	[illegible]
HIP_67469	LYQLENYCN	EVRLLRDPA	LYQLENYCNEVRLLRDPA		SCG1	37455/367830	0.932	67469	0.788	-0.073772	-1.0167	0	-1	18	-0.5833	-1	1	0	0	[illegible]
HIP_67470	VEALYLVCG	PQESKGQP	VEALYLVCGPQESKGQP		SCG1	20387/367830	0.989	67470	0.788	0.090710	-0.3833	0.3333	-1	17	-0.2176	-1	1	1	0	[illegible]
HIP_67471	HLVEALYL	ALEYIENL	HLVEALYLALEYIENL		SCG2	22256/334890	0.995	67471	0.788	0.040740	1.4	0	-2	16	0.7188	-3	2	0	0	[illegible]
HIP_67472	GERGFFYTP	EDLIEMLKT	GERGFFYTPEDLIEMLKT		SCG2	20631/334890	0.996	67472	0.788	-0.167540	-1.1333	0.1667	-2	18	-0.4389	-2	3	0	0	[illegible]
HIP_67473	VCGERGFFY	ILSILRHQ	VCGERGFFYILSILRHQ		CMGA	16566/247050	0.983	67473	0.788	-0.009586	1.9667	0.1667	2	17	0.4	1	3	1	0	[illegible]
HIP_67474	GIVEQCCTS	DKQYSSHHT	GIVEQCCTSDKQYSSHHT		SCG1	44486/367830	0.892	67474	0.788	0.080788	-1.65	0.1667	1	18	-0.8944	-1	1	0	0	[illegible]
HIP_67475	GERGFFY	ENQSSQA	GERGFFYENQSSQA		CMGA	20081/247050	0.972	67475	0.788	0.181402	-1.0333	0	-1	14	-1.3071	-1	3	0	0	[illegible]
HIP_67476	VEQCCTSIC	EDEEEEEGE	VEQCCTSICEDEEEEEGE		SCG1	16401/367830	0.994	67476	0.788	-0.032524	-0.7167	0.1667	-9	18	-1.15	-9	0	0	3	[illegible]
HIP_67477	QCCTSICS	YYDPLQWKS	QCCTSICSYYDPLQWKS		SCG1	34034/367830	0.949	67477	0.788	0.035616	0.0167	0.1667	0	17	-0.4	0	2	1	0	[illegible]
HIP_67478	GERGFFYTP	GWRPSSRED	GERGFFYTPGWRPSSRED		CMGA	17700/247050	0.980	67478	0.788	-0.054495	-1.5667	0.3333	0	18	-1.5167	0	4	0	0	[illegible]
HIP_67479	CGSHLVEAL	EDLRKESKD	CGSHLVEALEDLRKESKD		SCG2	32081/334890	0.988	67479	0.788	0.006598	-0.1833	0	-1	18	-0.8389	-2	0	0	0	[illegible]
HIP_67480	LVCGERGF	QEEERLSKE	LVCGERGFQEEERLSKE		CMGA	18870/247050	0.976	67480	0.788	-0.021709	-2.1	0.1667	-2	17	-1.0824	-2	1	1	1	[illegible]
HIP_67481	LVCGERGF	EQEEERLSK	LVCGERGFEQEEERLSK		CMGA	18871/247050	0.976	67481	0.788	-0.021709	-2.1	0.1667	-2	17	-1.0824	-2	1	1	1	[illegible]
HIP_67482	GIVEQCCTS	EESEEERGL	GIVEQCCTSEESEEERGL		SCG1	19011/367830	0.991	67482	0.788	-0.053858	-1.1333	0.3333	-5	18	-0.8111	-5	0	0	1	[illegible]
HIP_67483	GIVEQCCTS	ESEEERGLE	GIVEQCCTSESEEERGLE		SCG1	19012/367830	0.991	67483	0.788	-0.053858	-1.1333	0.3333	-5	18	-0.8111	-5	0	0	1	[illegible]
HIP_67484	LVEALYL	TPIESHQ	LVEALYLTPIESHQ		IAPP	2090/45018	0.990	67484	0.788	0.159654	1.4167	0.1667	-1	14	0.2714	-2	1	0	0	[illegible]
HIP_67485	SLYQLENYC	FPPMYEENS	SLYQLENYCFPPMYEENS		SCG2	6795/334890	0.999	67485	0.788	-0.134222	-0.45	0.3333	-3	18	-0.8278	-3	2	0	0	[illegible]
HIP_67486	FVNQHLCGS	RTNEIVEEQ	FVNQHLCGSRTNEIVEEQ		SCG2	26524/334890	0.993	67486	0.788	-0.057202	-1.2333	0.3333	-1	18	-0.6722	-2	0	0	0	[illegible]
HIP_67487	LGGGPGAGS	TPQSLATLE	LGGGPGAGSTPQSLATLE		SCG2	12402/334890	0.998	67487	0.788	-0.087003	-0.8667	0.5	-1	18	-0.0111	-1	0	0	1	[illegible]
HIP_67488	GERGFFYTP	HNEGMVTR	GERGFFYTPHNEGMVTR		SCG1	36085/367830	0.940	67488	0.788	-0.000085	-2.3	0.1667	1	17	-0.9706	0	3	1	0	[illegible]
HIP_67489	FVNQHLCGS	YPEEKKEEE	FVNQHLCGSYPEEKKEEE		CMGA	10578/247050	0.994	67489	0.788	-0.083375	-0.85	0.5	-2	18	-1.4611	-3	1	0	1	[illegible]
[illegible]																				

HIP_67953	VEALYLVC	SSMKLSFR	VEALYLVCSSMKLSFR		CMGA	16758/247050	0.982	67953	0.787	0.190246	1.8	0.3333	1	16	0.825	1	1	0	[illegible]
HIP_67954	EALYLVCG	EKNFFPEYN	EALYLVCGEKNFFPEYN		SCG1	30794/367830	0.963	67954	0.787	0.068896	-0.7667	0.1667	-2	17	-0.2529	-2	2	1	[illegible]
HIP_67955	TSICSLYQL	ENELTLNE	TSICSLYQLENELTLNE		SCG1	25692/367830	0.978	67955	0.787	0.076244	-1.9167	0	-3	17	-0.1824	-3	1	1	[illegible]
HIP_67956	CCTSICSLY	EVLKREPLN	CCTSICSLYEVLKREPLN		IAPP	3209/45018	0.974	67956	0.787	-0.055300	1.0333	0.1667	0	18	0.1944	0	1	0	[illegible]
HIP_67957	VEQCCTSIC	KQYSSHHTA	VEQCCTSICKQYSSHHTA		SCG1	44292/367830	0.893	67957	0.787	0.067533	-0.4167	0.1667	2	18	-0.4389	0	1	0	[illegible]
HIP_67958	PGAGSLQPL	LTLALSLLV	PGAGSLQPLLTLALSLLV		NPY	1901/49410	0.988	67958	0.787	0.010549	0.9333	0.1667	0	18	1.3667	0	0	0	[illegible]
HIP_67959	SHLVEALY	LGEGHHRVQ	SHLVEALYLGEGHHRVQ		SCG1	24591/367830	0.981	67959	0.787	0.206591	0.7	0.1667	2	17	-0.3471	-1	1	1	[illegible]
HIP_67960	PLALEGSLQ	ALQGAKERA	PLALEGSLQALQGAKERA		CMGA	14066/247050	0.989	67960	0.787	0.001072	0.2667	0.1667	0	18	-0.1778	0	0	0	[illegible]
HIP_67961	GQVELGGGP	GYPKTPGRA	GQVELGGGPGYPKTPGRA		SCG2	11123/334890	0.999	67961	0.787	-0.050147	-0.95	0.8333	1	18	-0.8222	1	1	0	[illegible]
HIP_67962	GIVEQCCTS	EEQIEQAIK	GIVEQCCTSEEQIEQAIK		SCG2	23034/334890	0.995	67962	0.787	-0.078968	-1.5833	0.1667	-3	18	-0.3222	-3	0	0	[illegible]
HIP_67963	QCCTSICS	QWSLYPSDS	QCCTSICSQWSLYPSDS		SCG1	30922/367830	0.962	67963	0.787	0.083721	0.1667	0.3333	-1	17	-0.1882	-1	2	1	[illegible]
HIP_67964	VEQCCTSIC	RDNMNDNFL	VEQCCTSICRDNMNDNFL		SCG1	28871/367830	0.969	67964	0.787	0.064760	-0.8833	0.1667	-2	18	-0.3222	-2	0	0	[illegible]
HIP_67965	LQVGQVEL	FLVHSSNN	LQVGQVELFLVHSSNN		IAPP	2679/45018	0.982	67965	0.787	0.011492	2.55	0	0	16	0.2562	-1	1	0	[illegible]
HIP_67966	EQCCTSICS	ENREEARFQ	EQCCTSICSENREEARFQ		SCG1	33403/367830	0.952	67966	0.787	0.140863	-0.8833	0.1667	-2	18	-1.0667	-2	0	0	[illegible]
HIP_67967	EQCCTSICS	NREEARFQD	EQCCTSICSNREEARFQD		SCG1	33404/367830	0.952	67967	0.787	0.140863	-0.8833	0.1667	-2	18	-1.0667	-2	0	0	[illegible]
HIP_67968	ERGFFYTPK	LSAIEAELE	ERGFFYTPKLSAIEAELE		CMGA	10896/247050	0.994	67968	0.787	-0.058913	-0.2333	0.3333	-2	18	-0.3278	-2	2	0	[illegible]
HIP_67969	LVEALYLVC	PDYNPYQG	LVEALYLVCPDYNPYQG		SCG2	22655/334890	0.995	67969	0.787	0.126548	0.6833	0.1667	-2	17	0.1529	-2	2	1	[illegible]
HIP_67970	VEQCCTSIC	LGEGHHRVQ	VEQCCTSICLGEGHHRVQ		SCG1	15931/367830	0.994	67970	0.787	-0.012065	1.0167	0.3333	1	18	-0.1667	-1	0	0	[illegible]
HIP_67971	GSLQPLALE	MDEEQKLYT	GSLQPLALEMDEEQKLYT		SCG2	27888/334890	0.992	67971	0.787	-0.025651	-0.5	0	-3	18	-0.6	-3	0	0	[illegible]
HIP_67972	LCGSHLV	LTEDEKKE	LCGSHLVLTEDEKKE		SCG1	25699/367830	0.978	67972	0.787	0.145109	0.7333	0	-1	15	-0.5867	-2	0	1	[illegible]
HIP_67973	HLVEALYLV	PVSTHQEAE	HLVEALYLVPVSTHQEAE		SCG1	11627/367830	0.997	67973	0.787	0.128814	1.4167	0.3333	-1	18	0.1556	-3	1	0	[illegible]
HIP_67974	GSLQPLAL	EGNNQAPG	GSLQPLALEGNNQAPG		CMGA	14108/247050	0.989	67974	0.787	0.215729	0.3333	0.1667	-1	16	-0.4813	-1	0	0	[illegible]
HIP_67975	SLQPLALEG	PQEEEEEEE	SLQPLALEGPQEEEEEEE		CMGA	5073/247050	0.998	67975	0.787	-0.103000	-0.9333	0.5	-8	18	-1.2833	-8	0	0	[illegible]
HIP_67976	LVEALYLV	EEENELTL	LVEALYLVEEENELTL		SCG1	21410/367830	0.987	67976	0.787	0.117564	-0.6333	0	-5	16	0.3875	-5	1	0	[illegible]
HIP_67977	VEQCCTSIC	ESGEETGSQ	VEQCCTSICESGEETGSQ		SCG1	7409/367830	0.999	67977	0.787	0.085427	0.25	0.5	-4	18	-0.5222	-4	0	0	[illegible]
HIP_67978	VEQCCTSIC	SGEETGSQE	VEQCCTSICSGEETGSQE		SCG1	7410/367830	0.999	67978	0.787	0.085427	0.25	0.5	-4	18	-0.5222	-4	0	0	[illegible]
HIP_67979	LVCGERGFF	NRRPEDQEL	LVCGERGFFNRRPEDQEL		CMGA	26395/247050	0.942	67979	0.787	0.098155	-1.2167	0.1667	-1	18	-0.9444	-1	2	0	[illegible]
HIP_67980	CGERGFFYT	RLFEKPLD	CGERGFFYTRLFEKPLD		SCG2	29766/334890	0.990	67980	0.787	-0.014470	0.4833	0	0	17	-0.5471	0	4	1	[illegible]
HIP_67981	ICSLYQLEN	EATDGARPQ	ICSLYQLENEATDGARPQ		CMGA	23193/247050	0.960	67981	0.787	-0.067382	-0.9333	0	-2	18	-0.6722	-2	1	0	[illegible]
HIP_67982	HLCGSHLVE	LENVQKFPS	HLCGSHLVELENVQKFPS		SCG2	29674/334890	0.990	67982	0.787	-0.020741	0.2167	0	1	18	-0.1556	-1	0	0	[illegible]
HIP_67983	GAGSLQPLA	SQEESEEGE	GAGSLQPLASQEESEEGE		SCG1	10615/367830	0.997	67983	0.787	-0.002580	-0.6333	0.3333	-5	18	-1.0278	-5	0	0	[illegible]
HIP_67984	CGERGFFY	WSKMDQLAK	CGERGFFYWSKMDQLAK		CMGA	25163/247050	0.949	67984	0.787	0.173148	-0.2167	0.1667	1	17	-0.6471	1	4	1	[illegible]
HIP_67985	DLQVGQVEL	GSEEYRAPR	DLQVGQVELGSEEYRAPR		SCG1	21431/367830	0.987	67985	0.787	0.207147	-0.0333	0.3333	-2	18	-0.9278	-2	0	0	[illegible]
HIP_67986	AGSLQPLAL	LQPLALEG	AGSLQPLALLQPLALEG		INSC	259/5184	0.997	67986	0.787	0.119139	1.35	0.1667	-1	17	0.7588	-1	0	1	[illegible]
HIP_67987	DLQVGQVEL	DSKENIEKN	DLQVGQVELDSKENIEKN		SCG2	31850/334890	0.988	67987	0.787	0.184066	-0.6167	0.1667	-3	18	-1.1111	-3	0	0	[illegible]
HIP_67988	GSHLVEALY	DRSSQGGS	GSHLVEALYDRSSQGGS		SCG1	30506/367830	0.964	67988	0.787	0.218054	-0.75	0.1667	0	17	-0.6059	-1	1	1	[illegible]
HIP_67989	RGFFYTPKT	RNLARVPKL	RGFFYTPKTRNLARVPKL		SCG1	42702/367830	0.903	67989	0.787	0.064914	-1.7333	0.1667	5	18	-0.6611	5	1	0	[illegible]
HIP_67990	HLCGSHLVE	SANRRPEDQ	HLCGSHLVESANRRPEDQ		CMGA	24064/247050	0.956	67990	0.787	0.094355	0.3333	0.1667	1	18	-1.1333	-1	0	0	[illegible]
HIP_67991	GSLQPLAL	TEKWAEGG	GSLQPLALTEKWAEGG		SCG1	32873/367830	0.954	67991	0.787	0.239064	0.2167	0	-1	16	-0.2875	-1	1	0	[illegible]
HIP_67992	GSLQPLALE	RHYINLITR	GSLQPLALERHYINLITR		NPY	6743/49410	0.860	67992	0.787	0.026149	-1.15	0	2	18	-0.0833	1	1	0	[illegible]
HIP_67993	VCGERGFFY	ALAVDGAG	VCGERGFFYALAVDGAG		CMGA	15440/247050	0.986	67993	0.787	-0.043108	1.95	0	-1	17	0.6647	-1	3	1	[illegible]
HIP_67994	YLVCGERGF	PTKADTEKW	YLVCGERGFPTKADTEKW		SCG1	27652/367830	0.973	67994	0.787	-0.023650	-1.3833	0.3333	0	18	-0.7611	0	1	0	[illegible]
HIP_67995	CGSHLVEAL	GPTKADTEK	CGSHLVEALGPTKADTEK		SCG1	16295/367830	0.994	67995	0.787	0.156631	-0.1	0.3333	0	18	-0.4556	-1	0	0	[illegible]
HIP_67996	LVEALYLVC	SVFQELGK	LVEALYLVCSVFQELGK		SCG2	20936/334890	0.996	67996	0.787	0.050128	2.7833	0.1667	-1	17	1.0588	-1	2	1	[illegible]
HIP_67997	VEALYLVC	ELQKIAEK	VEALYLVCELQKIAEK		SCG1	25858/367830	0.978	67997	0.787	0.136036	1.2167	0	-1	16	0.4563	-1	1	0	[illegible]
HIP_67998	HLCGSHLVE	HLNQGSSQE	HLCGSHLVEHLNQGSSQE		SCG2	36853/334890	0.982	67998	0.787	-0.018488	0.2667	0	1	18	-0.6778	-2	0	0	[illegible]
HIP_67999	ICSLYQLEN	SQGLVDREK	ICSLYQLENSQGLVDREK		CMGA	11647/247050	0.993	67999	0.787	0.016927	-1.3167	0.3333	-1	18	-0.5611	-1	1	0	[illegible]
HIP_68000	QHLCGSHLV	SQGLVDREK	QHLCGSHLVSQGLVDREK		CMGA	14885/247050	0.987	68000	0.787	0.207040	0.0167	0.3333	2	18	-0.4944	0	0	0	[illegible]
HIP_68001	CGSHLVEAL	LEALRQAEN	CGSHLVEALLEALRQAEN		SCG2	24519/334890	0.994	68001	0.787	-0.069196	0.7	0	-1	18	0.05	-2	0	0	[illegible]
HIP_68002	GERGFFYT	MLKTGEKPN	GERGFFYTMLKTGEKPN		SCG2	30748/334890	0.989	68002	0.787	-0.037213	0.4333	0	1	17	-1	1	3	1	[illegible]
HIP_68003	LVEALYLVC	TGEKPNGS	LVEALYLVCTGEKPNGS		SCG2	19476/334890	0.996	68003	0.787	0.207067	0.9833	0.1667	-1	17	0.2647	-1	1	1	[illegible]
HIP_68004	HLVEALYLV	NIAYEDVV	HLVEALYLVNIAYEDVV		SCG2	18283/334890	0.997	68004	0.787	0.018189	1.5833	0	-2	17	0.9706	-3	2	1	[illegible]
HIP_68005	HLVEALYLV	DASEAHES	HLVEALYLVDASEAHES		SCG1	18136/367830	0.992	68005	0.787	0.139914	0.7	0.1667	-2	17	0.1118	-4	1	1	[illegible]
HIP_68006	ERGFFYTPK	TLESVFQEL	ERGFFYTPKTLESVFQEL		SCG2	24228/334890	0.994	68006	0.787	-0.056118	-1.1	0.1667	-1	18	-0.4278	-1	2	0	[illegible]
HIP_68007	GGGPGAGSL	FRARAHGFR	GGGPGAGSLFRARAHGFR		CMGA	17968/247050	0.979	68007	0.787	0.113591	0.45	0.3333	3	18	-0.2667	3	1	0	[illegible]
HIP_68008	SHLVEALY	FPSPEMIR	SHLVEALYFPSPEMIR		SCG2	14030/334890	0.998	68008	0.787	0.110010	0.7833	0.3333	0	16	0.125	-1	2	0	[illegible]
HIP_68009	CTSICSLYQ	GHPQEESEE	CTSICSLYQGHPQEESEE		SCG1	8310/367830	0.998	68009	0.787	-0.000372	-1.0333	0.3333	-3	18	-0.9611	-4	1	0	[illegible]
HIP_68010	GSHLVEALY	QGSSEDDL	GSHLVEALYQGSSEDDL		SCG2	14382/334890	0.998	68010	0.787	0.172646	-0.0667	0.3333	-3	17	-0.4588	-4	1	1	[illegible]
[illegible]																			

HIP_68464	DLQVGQVEL	PSEEKGHPQ	DLQVGQVELPSEEKGHPQ		SCG1	22773/367830	0.985	68464	0.787	0.186951	-0.2333	0.3333
HIP_68465	GSLQPLAL	LPRLPYGAG	GSLQPLALLPRLPYGAG		SCG2	29385/334890	0.991	68465	0.787	0.200309	1.1833	0.1667
HIP_68466	AGSLQPLAL	SEKNFPMD	AGSLQPLALSEKNFPMD		SCG2	27109/334890	0.992	68466	0.787	0.243584	0.2	0.1667
HIP_68467	SLYQLENYC	DPADASEAH	SLYQLENYCDPADASEAH		SCG1	13221/367830	0.996	68467	0.787	-0.094514	-0.9333	0.1667
HIP_68468	CTSICSLYQ	FPNPYNQE	CTSICSLYQFPNPYNQE		SCG2	15046/334890	0.998	68468	0.787	-0.052839	-0.55	0.1667
HIP_68469	SICSLYQLE	IEISRNLQI	SICSLYQLEIEISRNLQI		SCG2	21252/334890	0.996	68469	0.787	-0.082113	0.3833	0
HIP_68470	VEALYLVCG	SRKDVKDKE	VEALYLVCGSRKDVKDKE		SCG1	39295/367830	0.922	68470	0.787	0.122329	-0.4833	0.3333
HIP_68471	PGAGSLQPL	TCATQRLAN	PGAGSLQPLTCATQRLAN		IAPP	2755/45018	0.981	68471	0.787	0.063558	0.3833	0.1667
HIP_68472	EALYLVCGE	EGQEEEEDN	EALYLVCGEEGQEEEEDN		CMGA	8502/247050	0.996	68472	0.787	-0.125917	-1.4667	0.3333
HIP_68473	QHLCGSHLV	PVSQECFET	QHLCGSHLVPVSQECFET		CMGA	9664/247050	0.995	68473	0.787	0.144816	1.1	0.3333
HIP_68474	LVEALYLV	SEVDKRRTR	LVEALYLVSEVDKRRTR		SCG1	28342/367830	0.971	68474	0.787	0.178119	1.1	0.1667
HIP_68475	GSLQPLALE	PQGAWKELD	GSLQPLALEPQGAWKELD		SCG1	16347/367830	0.994	68475	0.787	0.139759	-0.5667	0.3333
HIP_68476	NQHLCGSHL	PQLRRGWRP	NQHLCGSHLPQLRRGWRP		CMGA	27582/247050	0.934	68476	0.787	0.063005	-0.25	0.3333
HIP_68477	GERGFFYTP	EGNNQAPG	GERGFFYTPEGNNQAPG		CMGA	15635/247050	0.985	68477	0.787	-0.015770	-1.8333	0.3333
HIP_68478	VGQVELGGG	QYWDEDLLM	VGQVELGGGQYWDEDLLM		SCG2	7795/334890	0.999	68478	0.787	0.080169	-1.15	0.5
HIP_68479	EAEDLQVGQ	REEARFQDK	EAEDLQVGQREEARFQDK		SCG1	27629/367830	0.973	68479	0.787	-0.120474	-1.8667	0.1667
HIP_68480	GSLQPLAL	FGAILSST	GSLQPLALFGAILSST		IAPP	2466/45018	0.986	68480	0.787	0.063057	2.2667	0.1667
HIP_68481	VEALYLVC	KPGAEEAQD	VEALYLVCKPGAEEAQD		CMGA	16247/247050	0.984	68481	0.787	0.212416	0.7667	0.3333
HIP_68482	VEALYLVC	GMVTRCIIE	VEALYLVCGMVTRCIIE		SCG1	17186/367830	0.993	68482	0.787	0.081128	2.7	0.1667
HIP_68483	EQCCTSIC	EFPDFYDSE	EQCCTSICEFPDFYDSE		SCG1	16183/367830	0.994	68483	0.787	-0.032326	0.65	0.3333
HIP_68484	LVCGERGFF	SLELDKMA	LVCGERGFFSLELDKMA		SCG1	19226/367830	0.991	68484	0.787	0.000680	0.7833	0.3333
HIP_68485	QCCTSICSL	LGLSGLTLA	QCCTSICSLLGLSGLTLA		NPY	2194/49410	0.985	68485	0.787	-0.024238	2.1167	0.3333
HIP_68486	RGFFYTPKT	RLGELFNPY	RGFFYTPKTRLGELFNPY		SCG1	30312/367830	0.964	68486	0.787	-0.021926	-1.2167	0.3333
HIP_68487	VEQCCTSIC	HRGRGGEPR	VEQCCTSICHRGRGGEPR		SCG1	39409/367830	0.922	68487	0.787	0.085208	-0.3167	0.3333
HIP_68488	GGPGAGSLQ	ETGSQENHP	GGPGAGSLQETGSQENHP		SCG1	14044/367830	0.996	68488	0.787	-0.096683	-0.85	0.3333
HIP_68489	LVCGERGFF	REPLNYLPL	LVCGERGFFREPLNYLPL		IAPP	3764/45018	0.963	68489	0.787	0.097753	-0.7333	0.3333
HIP_68490	TSICSLYQ	IFLISGAEA	TSICSLYQIFLISGAEA		SCG2	20028/334890	0.996	68490	0.787	-0.038575	1.6833	0
HIP_68491	GSLQPLAL	ARYYSALR	GSLQPLALARYYSALR		NPY	5596/49410	0.915	68491	0.787	0.126016	0.9	0
HIP_68492	GGPGAGSLQ	EEEGSANR	GGPGAGSLQEEEGSANR		CMGA	19867/247050	0.972	68492	0.787	-0.054465	-1.8333	0.1667
HIP_68493	PLALEGSLQ	QDTKENREE	PLALEGSLQQDTKENREE		SCG1	25347/367830	0.979	68493	0.787	-0.113259	-1.3667	0.1667
HIP_68494	HLCGSHLVE	QEKVLPRLP	HLCGSHLVEQEKVLPRLP		SCG2	41176/334890	0.974	68494	0.787	0.137422	-1.0667	0
HIP_68495	GSLQPLALE	SQREDEEEE	GSLQPLALESQREDEEEE		SCG1	27308/367830	0.974	68495	0.787	0.072379	-1.1167	0.1667
HIP_68496	SICSLYQLE	LTGPNNQKR	SICSLYQLELTGPNNQKR		SCG2	20546/334890	0.996	68496	0.787	-0.142688	-0.0833	0.1667
HIP_68497	GAGSLQPLA	EEGSANRRP	GAGSLQPLAEEGSANRRP		CMGA	12058/247050	0.992	68497	0.787	0.079846	-0.5667	0.3333
HIP_68498	PGAGSLQPL	TGSQENHPQ	PGAGSLQPLTGSQENHPQ		SCG1	12820/367830	0.996	68498	0.787	-0.001342	-0.5333	0.5
HIP_68499	HLVEALYLV	NQVKRVPG	HLVEALYLVNQVKRVPG		SCG2	30590/334890	0.990	68499	0.787	0.197336	0.65	0
HIP_68500	ERGFFYTP	PQGAWKELD	ERGFFYTPPQGAWKELD		SCG1	16733/367830	0.993	68500	0.787	-0.032331	-1.5167	0.5
HIP_68501	VELGGGPGA	RPQSEESWD	VELGGGPGARPQSEESWD		SCG1	19209/367830	0.991	68501	0.787	-0.082918	-1.6333	0.5
HIP_68502	GERGFFYT	PKENKPYAL	GERGFFYTPKENKPYAL		SCG2	33760/334890	0.986	68502	0.787	0.153176	-1.3667	0.1667
HIP_68503	CGERGFFY	GEDWNPVEE	CGERGFFYGEDWNPVEE		SCG2	20395/334890	0.996	68503	0.787	0.169916	-0.5167	0.1667
HIP_68504	GERGFFYT	RLPYGAGRS	GERGFFYTRLPYGAGRS		SCG2	32272/334890	0.988	68504	0.787	0.057120	-0.25	0.1667
HIP_68505	PLALEGSLQ	EEKHLEEPG	PLALEGSLQEEKHLEEPG		SCG1	31374/367830	0.960	68505	0.787	-0.077911	-1.9	0.1667
HIP_68506	ERGFFYTP	EEGENYQK	ERGFFYTPEEGENYQK		SCG1	23310/367830	0.984	68506	0.787	-0.022730	-1.8333	0.3333

[illegible]

HIP_68975	LYQLENYCN	EEARFQDKQ	LYQLENYCNEEARFQDKQ		SCG1	35148/367830	0.944	68975	0.786	-0.018410	-1.25	0	-2	18	-1.5444	-2	1	0	0
HIP_68976	CTSICSLYQ	RNHNEGMVT	CTSICSLYQRNHNEGMVT		SCG1	49709/367830	0.854	68976	0.786	0.093849	-2.0333	0	0	18	-0.3889	0	1	0	0
HIP_68977	CCTSICSLY	SSSRGEAGA	CCTSICSLYSSSRGEAGA		SCG1	16032/367830	0.994	68977	0.786	0.116464	-0.1167	0.6667	0	18	0.2556	0	1	0	1
HIP_68978	ERGFFYTPK	DELSEVLEN	ERGFFYTPKDELSEVLEN		CMGA	17048/247050	0.982	68978	0.786	-0.077228	-1.5667	0.1667	-3	18	-0.9333	-3	2	0	0
HIP_68979	IVEQCCTSI	DEEDKRNYP	IVEQCCTSIDEEDKRNYP		SCG1	26241/367830	0.977	68979	0.786	-0.028631	-1.25	0.1667	-3	18	-1.0611	-3	0	0	0
HIP_68980	SHLVEALY	WSKMDQLAK	SHLVEALYWSKMDQLAK		CMGA	25930/247050	0.945	68980	0.786	0.207887	-0.2167	0.1667	1	17	-0.2471	0	2	1	0
HIP_68981	SICSLYQLE	EDDLQEEEQ	SICSLYQLEEDDLQEEEQ		SCG2	24600/334890	0.994	68981	0.786	-0.095652	-2.2833	0	-7	18	-1.0833	-7	0	0	1
HIP_68982	GQVELGGGP	QYWDEDLLM	GQVELGGGPQYWDEDLLM		SCG2	6388/334890	0.999	68982	0.786	0.056076	-1.35	0.5	-4	18	-0.4944	-4	2	0	1
HIP_68983	SICSLYQLE	NRNHNEGMV	SICSLYQLENRNHNEGMV		SCG1	43626/367830	0.897	68983	0.786	-0.006370	-2.45	0	0	18	-0.6278	-1	1	0	0
HIP_68984	GSHLVEALY	ESGEETIGS	GSHLVEALYESGEETIGS		SCG1	16359/367830	0.994	68984	0.786	0.171756	-0.0667	0.3333	-3	17	-0.5412	-4	1	1	0
HIP_68985	HLVEALYL	GAKERAHQ	HLVEALYLGAKERAHQ		CMGA	21960/247050	0.965	68985	0.786	0.243049	0.6333	0.1667	2	16	-0.375	0	1	0	0
HIP_68986	GERGFFYT	QELGEYLAR	GERGFFYTQELGEYLAR		SCG2	31142/334890	0.989	68986	0.786	0.046143	-0.4	0	-1	17	-0.7353	-1	3	1	0
HIP_68987	GSLQPLAL	DLKRQYD	GSLQPLALDLKRQYD		SCG1	38180/367830	0.929	68987	0.786	0.218508	0.9667	0	0	15	-0.6333	0	0	1	0
HIP_68988	VCGERGFF	DKMAHGYGE	VCGERGFFDKMAHGYGE		SCG1	34236/367830	0.948	68988	0.786	0.061596	-0.05	0.1667	0	17	-0.5294	-1	2	1	0
HIP_68989	QVGQVELGG	GDERILSIL	QVGQVELGGGDERILSIL		CMGA	8211/247050	0.996	68989	0.786	-0.089423	-0.7333	0.5	-2	18	0.2444	-2	0	0	1
HIP_68990	CGERGFFY	SQVSEEVK	CGERGFFYSQVSEEVK		SCG1	24749/367830	0.981	68990	0.786	0.151500	0.7	0.1667	-1	16	-0.6	-1	3	0	0
HIP_68991	VEQCCTSIC	EDELSEVLE	VEQCCTSICEDELSEVLE		CMGA	12988/247050	0.991	68991	0.786	-0.029805	-0.7167	0.1667	-6	18	0.0667	-6	0	0	0
HIP_68992	CGERGFFY	KRERMDEEQ	CGERGFFYKRERMDEEQ		SCG2	35847/334890	0.984	68992	0.786	0.204047	-1.2667	0	-1	17	-1.7941	-1	3	1	0
HIP_68993	SHLVEALY	EMLKTGEKP	SHLVEALYEMLKTGEKP		SCG2	30542/334890	0.990	68993	0.786	0.056791	1.0833	0	0	17	-0.4118	-1	1	1	0
HIP_68994	QCCTSICSL	EAEAGEEAV	QCCTSICSLEAEAGEEAV		CMGA	13641/247050	0.990	68994	0.786	-0.040643	0.05	0.1667	-4	18	0.2889	-4	0	0	0
HIP_68995	PGAGSLQPL	VCLGALAEA	PGAGSLQPLVCLGALAEA		NPY	1445/49410	0.992	68995	0.786	0.107384	1.5333	0.1667	-1	18	0.9389	-1	0	0	0
HIP_68996	CGSHLVEAL	HLPERDSLS	CGSHLVEALHLPERDSLS		SCG2	25260/334890	0.994	68996	0.786	-0.075571	0.1833	0.1667	0	18	-0.1167	-2	0	0	0
HIP_68997	GERGFFYT	PSSKDVME	GERGFFYTPSSKDVME		CMGA	13580/247050	0.990	68997	0.786	0.164428	-0.4	0.5	-1	16	-0.825	-1	3	0	0
HIP_68998	PGAGSLQPL	DWMRILEA	PGAGSLQPLDWMRILEA		SCG2	16330/334890	0.997	68998	0.786	0.014304	-0.6333	0.1667	-1	18	0.2889	-1	1	0	0
HIP_68999	ERGFFYT	GPQLRRG	ERGFFYTGPQLRRG		CMGA	17543/247050	0.980	68999	0.786	0.165316	-0.7833	0.3333	2	14	-1.1357	2	3	0	0
HIP_69000	VNQHLCGSH	ENQMDKARR	VNQHLCGSHENQMDKARR		SCG1	36933/367830	0.935	69000	0.786	-0.070662	-2.4833	0.3333	3	18	-1.5167	1	0	0	0
HIP_69001	NQHLCGSHL	PQGLFRGGK	NQHLCGSHLPQGLFRGGK		CMGA	16604/247050	0.983	69001	0.786	0.122688	-0.95	0.5	4	18	-0.7	2	0	0	0
HIP_69002	HLCGSHLVE	TEALPDGLS	HLCGSHLVETEALPDGLS		SCG2	25489/334890	0.993	69002	0.786	-0.028545	0.35	0	-1	18	0.1167	-3	0	0	0
HIP_69003	RGFFYTPKT	IINSNQVKR	RGFFYTPKTIINSNQVKR		SCG2	26380/334890	0.993	69003	0.786	0.101665	-0.1167	0.1667	4	18	-0.7778	4	1	0	0
HIP_69004	VEALYLVC	GHSRERADE	VEALYLVCGHSRERADE		SCG1	24232/367830	0.982	69004	0.786	0.219770	1.0167	0.3333	-1	17	-0.3882	-2	1	1	0
HIP_69005	GERGFFYTP	PEYGEEIK	GERGFFYTPPEYGEEIK		SCG1	25486/367830	0.979	69005	0.786	-0.074273	-1.6667	0.3333	-2	17	-1.1765	-2	4	1	0
HIP_69006	LYQLENYCN	CATQRLANF	LYQLENYCNCATQRLANF		IAPP	3077/45018	0.977	69006	0.786	-0.123327	0.2167	0	0	18	-0.3333	0	1	0	0
HIP_69007	QHLCGSHLV	VPGQGSSED	QHLCGSHLVVPGQGSSED		SCG2	9761/334890	0.999	69007	0.786	0.116567	1.1667	0.3333	0	18	-0.3944	-2	0	0	0
HIP_69008	PGAGSLQPL	EEKRFLGEG	PGAGSLQPLEEKRFLGEG		SCG1	37602/367830	0.932	69008	0.786	0.015070	-2.0333	0.1667	-1	18	-0.6667	-1	0	0	0
HIP_69009	RGFFYTPKT	DWMRILEA	RGFFYTPKTDWMRILEA		SCG2	24188/334890	0.994	69009	0.786	0.105310	-1.45	0.1667	1	18	-0.1889	1	2	0	0
HIP_69010	GGGPGAGSL	ENELTLNEK	GGGPGAGSLENELTLNEK		SCG1	20040/367830	0.989	69010	0.786	-0.088665	-1.3167	0.3333	-2	18	-0.7389	-2	0	0	1
HIP_69011	SICSLYQL	YGEESEEER	SICSLYQLYGEESEEER		SCG1	15069/367830	0.995	69011	0.786	-0.038501	-1.0333	0.1667	-4	17	-0.9588	-4	2	1	1
HIP_69012	LVEALYLV	DSEGLSQG	LVEALYLVDSEGLSQG		CMGA	14399/247050	0.988	69012	0.786	0.168065	-0.1833	0.1667	-3	16	0.4812	-3	1	0	0
HIP_69013	GGPGAGSL	EYNYDWWEK	GGPGAGSLEYNYDWWEK		SCG1	25050/367830	0.980	69013	0.786	-0.030474	-0.95	0.3333	-2	17	-1.2176	-2	2	1	0
HIP_69014	CSLYQLENY	AGQVTALPV	CSLYQLENYAGQVTALPV		CMGA	13867/247050	0.989	69014	0.786	-0.171952	-1.7333	0.1667	-1	18	0.3222	-1	1	0	0
HIP_69015	CGSHLVEAL	EYNYDWWE	CGSHLVEALEYNYDWWE		SCG1	33029/367830	0.953	69015	0.786	0.029633	-1.0333	0	-3	17	-0.6	-4	2	1	0
HIP_69016	HLVEALYL	DTEKWAEG	HLVEALYLDTEKWAEG		SCG1	25805/367830	0.978	69016	0.786	0.183247	-0.2333	0	-2	16	-0.325	-3	1	0	0
HIP_69017	QHLCGSHLV	DEDLLMKVL	QHLCGSHLVDEDLLMKVL		SCG2	22749/334890	0.995	69017	0.786	0.120274	-0.95	0	0	18	0.35	-2	0	0	0
HIP_69018	PLALEGSLQ	KNDDTPNRQ	PLALEGSLQKNDDTPNRQ		SCG2	40449/334890	0.976	69018	0.786	-0.024916	-1.9	0.1667	-1	18	-1.3778	-1	0	0	0
HIP_69019	HLVEALYLV	KRQYDRVA	HLVEALYLVKRQYDRVA		SCG1	41597/367830	0.909	69019	0.786	0.194929	-0.8667	0	2	17	-0.0941	1	2	1	0
HIP_69020	LYQLENYCN	PEGKGEQE	LYQLENYCNPEGKGEQE		CMGA	9524/247050	0.995	69020	0.786	0.098432	-1.3	0.3333	-3	17	-1.5765	-3	1	1	0
HIP_69021	VEALYLVC	VHSSNNFGA	VEALYLVCVHSSNNFGA		IAPP	3683/45018	0.965	69021	0.786	0.132133	1.7833	0.1667	0	17	0.7118	-1	1	1	0
HIP_69022	VNQHLCGSH	RESTENVPR	VNQHLCGSHRESTENVPR		NPY	5176/49410	0.927	69022	0.786	-0.064335	-2.2	0.5	2	18	-1.25	0	0	0	0
HIP_69023	FVNQHLCGS	GETQNAFLN	FVNQHLCGSGETQNAFLN		SCG1	17579/367830	0.992	69023	0.786	-0.055819	-0.55	0.5	0	18	-0.2667	-1	0	0	0
HIP_69024	AGSLQPLAL	SKPDNPGE	AGSLQPLALSKPDNPGE		NPY	3744/49410	0.963	69024	0.786	0.232446	0.5167	0.3333	-1	17	-0.5941	-1	0	1	0
HIP_69025	CCTSICSLY	MPVSQECFE	CCTSICSLYMPVSQECFE		CMGA	7954/247050	0.997	69025	0.786	-0.124959	1.0333	0.3333	-2	18	0.5944	-2	1	0	0
HIP_69026	SHLVEALY	QTQEEVRD	SHLVEALYQTQEEVRD		SCG2	22698/334890	0.995	69026	0.786	0.175874	-0.5667	0	-2	16	-0.8562	-3	1	0	0
HIP_69027	FVNQHLCGS	FQRNQLLQK	FVNQHLCGSFQRNQLLQK		SCG2	34162/334890	0.986	69027	0.786	0.022693	-0.65	0.3333	3	18	-0.5611	2	1	0	0
[illegible]																			

HIP_69486	VEQCCTSIC	EDLEWERYR	VEQCCTSICEDLEWERYR		SCG1	20843/367830	0.988	69486	0.786	-0.061495	0.5	0.1667	-3	18	-0.7611	-3	0	0
HIP_69487	VGQVELGGG	DNPFKRTNE	VGQVELGGGDNPFKRTNE		SCG2	13000/334890	0.998	69487	0.786	-0.056586	-1.6333	0.6667	-1	18	-1.0167	-1	1	0
HIP_69488	GPGAGSLQP	PMYEENSRD	GPGAGSLQPPMYEENSRD		SCG2	10711/334890	0.999	69488	0.786	-0.082966	-0.3833	0.3333	-2	18	-1.3	-2	1	0
HIP_69489	ALYLVCGER	RESTENVPR	ALYLVCGERRESTENVPR		NPY	5478/49410	0.918	69489	0.786	-0.078555	-2.8667	0.3333	0	18	-0.6667	0	0	0
HIP_69490	VEALYLVCG	SKPSPMPVS	VEALYLVCGSKPSPMPVS		CMGA	17564/247050	0.980	69490	0.786	0.133276	0	0.5	0	18	0.5611	0	1	0
HIP_69491	VEALYLVCG	HQEAENEKD	VEALYLVCGHQEAENEKD		SCG1	23505/367830	0.983	69491	0.786	-0.031990	-0.65	0.1667	-3	18	-0.6222	-4	1	0
HIP_69492	QPLALEGSL	YYSALRHYI	QPLALEGSLYYSALRHYI		NPY	2649/49410	0.980	69492	0.786	0.006469	-0.1333	0.5	1	18	0.0611	0	2	0
HIP_69493	VELGGGPGA	MPVDNRNHN	VELGGGPGAMPVDNRNHN		SCG1	2276/367830	1.000	69493	0.786	0.003304	0.7167	0.5	0	18	-0.7833	-1	0	0
HIP_69494	GGPGAGSL	NYDWWEKKP	GGPGAGSLNYDWWEKKP		SCG1	29995/367830	0.965	69494	0.786	0.008570	-0.95	0.3333	0	17	-1.2588	0	3	1
HIP_69495	LYQLENYCN	QVTALPVNS	LYQLENYCNQVTALPVNS		CMGA	14386/247050	0.988	69495	0.786	-0.123007	-0.3833	0	-1	18	-0.1444	-1	1	0
HIP_69496	CSLYQLENY	GSQENHPQE	CSLYQLENYGSQENHPQE		SCG1	18696/367830	0.991	69496	0.786	-0.036416	-2.1667	0.3333	-2	18	-1.5167	-3	1	0
HIP_69497	GSHLVEALY	EQIEQAIK	GSHLVEALYEQIEQAIK		SCG2	22707/334890	0.995	69497	0.786	0.108478	0.3	0	-1	17	-0.1588	-2	1	1
HIP_69498	VCGERGFF	TKENREEA	VCGERGFFTKENREEA		SCG1	33983/367830	0.949	69498	0.786	0.103320	-0.4833	0.1667	-1	16	-1.1125	-1	2	0
HIP_69499	LVCGERGFF	QGLVDREK	LVCGERGFFQGLVDREK		CMGA	12015/247050	0.992	69499	0.786	0.022620	0.85	0.3333	0	17	-0.2353	0	2	1
HIP_69500	LYQLENYCN	LGEYLARML	LYQLENYCNLGEYLARML		SCG2	13233/334890	0.998	69500	0.786	-0.082502	-0.4	0.1667	-1	18	-0.0611	-1	2	0
HIP_69501	LVEALYLV	GQGSSEDDL	LVEALYLVGQGSSEDDL		SCG2	13470/334890	0.998	69501	0.786	0.173665	0.4	0.3333	-4	17	0.2471	-4	1	1
HIP_69502	GGPGAGSLQ	AALSLIPLI	GGPGAGSLQAALSLIPLI		SCG2	15113/334890	0.998	69502	0.786	0.098266	1.15	0.1667	0	18	1.0944	0	0	0
HIP_69503	CCTSICSLY	LARMLVKYP	CCTSICSLYLARMLVKYP		SCG2	28745/334890	0.991	69503	0.786	-0.042678	0.4667	0.1667	2	18	0.9111	2	1	0
HIP_69504	FVNQHLCG	EEEEEEEAE	FVNQHLCGEEEEEEEEAE		CMGA	12006/247050	0.992	69504	0.786	-0.016396	-0.7667	0.1667	-7	17	-1.3824	-8	0	1
HIP_69505	CTSICSLYQ	QENQMDKA	CTSICSLYQQENQMDKA		SCG1	33164/367830	0.953	69505	0.786	0.141836	-1.9167	0	-1	17	-0.6765	-1	1	1
HIP_69506	VEQCCTSIC	QSAPKENKP	VEQCCTSICQSAPKENKP		SCG2	22522/334890	0.995	69506	0.786	0.012795	0.6167	0.3333	0	18	-0.7111	0	0	0
HIP_69507	LYLVCGERG	EYGEEIKGY	LYLVCGERGEYGEEIKGY		SCG1	23378/367830	0.984	69507	0.786	-0.093964	-2.2667	0.3333	-2	18	-0.5056	-2	1	0
HIP_69508	QCCTSICSL	TREEKRFLG	QCCTSICSLTREEKRFLG		SCG1	46515/367830	0.878	69508	0.786	0.231082	-0.5333	0.1667	1	18	-0.2444	1	0	0
HIP_69509	CTSICSLYQ	VRDSKENIE	CTSICSLYQVRDSKENIE		SCG2	34032/334890	0.986	69509	0.786	-0.035844	-0.8	0	-1	18	-0.4611	-1	1	0
HIP_69510	QHLCGSHLV	AEGGGHSR	QHLCGSHLVAEGGGHSR		SCG1	24699/367830	0.981	69510	0.786	0.193213	0.45	0.1667	3	17	-0.4824	0	0	1
HIP_69511	ICSLYQLEN	EKVAHQLQA	ICSLYQLENEKVAHQLQA		CMGA	30746/247050	0.907	69511	0.786	-0.039219	-1.0667	0	0	18	-0.2222	-1	1	0
HIP_69512	AGSLQPLAL	KRNAVEVLK	AGSLQPLALKRNAVEVLK		IAPP	5075/45018	0.924	69512	0.786	0.201721	-0.4167	0	2	18	0.1889	2	0	0
HIP_69513	CTSICSLY	KGYPGVQAP	CTSICSLYKGYPGVQAP		SCG1	35288/367830	0.943	69513	0.786	0.102515	-0.65	0.3333	1	17	0.1765	1	2	1
HIP_69514	CGERGFFY	IILEALRQA	CGERGFFYIILEALRQA		SCG2	22759/334890	0.995	69514	0.786	-0.046542	2.85	0	0	17	0.3941	0	3	1
HIP_69515	TSICSLYQL	YNQEKVLP	TSICSLYQLYNQEKVLP		SCG2	36282/334890	0.983	69515	0.786	0.108346	-1.55	0	0	17	-0.1059	0	2	1
HIP_69516	LCGSHLVEA	THSREKSSQ	LCGSHLVEATHSREKSSQ		SCG1	49661/367830	0.854	69516	0.786	0.166313	-0.3667	0.1667	2	18	-0.75	0	0	0
HIP_69517	EAEDLQVGQ	YYSALRHYI	EAEDLQVGQYYSALRHYI		NPY	1734/49410	0.990	69517	0.786	-0.086150	-0.5167	0.3333	-1	18	-0.5778	-2	2	0
HIP_69518	HLCGSHLVE	RDPADASEA	HLCGSHLVERDPADASEA		SCG1	32497/367830	0.955	69518	0.786	0.138938	-0.85	0.1667	-1	18	-0.4889	-3	0	0
HIP_69519	CGERGFFYT	LQIPPEDL	CGERGFFYTLQIPPEDL		SCG2	19315/334890	0.996	69519	0.786	-0.097349	0.9333	0	-2	17	-0.2529	-2	3	1
HIP_69520	HLVEALYL	EEEPEYGE	HLVEALYLEEEPEYGE		SCG1	23214/367830	0.984	69520	0.786	0.131209	-0.7	0	-5	16	-0.7125	-6	1	0
HIP_69521	EQCCTSICS	ELEQEEERL	EQCCTSICSELEQEEERL		CMGA	11925/247050	0.992	69521	0.786	-0.067576	0.5	0.1667	-5	18	-0.8444	-5	0	0
HIP_69522	CGERGFFYT	ENLRQQAH	CGERGFFYTENLRQQAH		SCG2	35371/334890	0.984	69522	0.786	0.091875	-0.4	0	1	17	-1.1059	0	3	1
HIP_69523	LVEALYLVC	RPDRSSQG	LVEALYLVCRPDRSSQG		SCG1	31007/367830	0.962	69523	0.786	0.222174	0.15	0.1667	0	17	-0.0176	0	1	1
HIP_69524	CTSICSLYQ	GPGPQLRR	CTSICSLYQGPGPQLRR		CMGA	12883/247050	0.991	69524	0.786	0.164299	-0.5667	0.5	2	17	-0.3824	2	1	1
HIP_69525	VNQHLCGSH	YNQEKVLPR	VNQHLCGSHYNQEKVLPR		SCG2	31245/334890	0.989	69525	0.786	-0.033604	-2.1167	0.3333	3	18	-0.9944	1	1	0
HIP_69526	TSICSLYQL	DLQEEEQIE	TSICSLYQLDLQEEEQIE		SCG2	18379/334890	0.997	69526	0.786	-0.039411	-0.7	0	-5	18	-0.4833	-5	1	0
HIP_69527	GQVELGGG	GFFYTPK	GQVELGGGGFFYTPK		INSB	102/4968	0.992	69527	0.786	0.078375	0.6667	0.6667	0	15	-0.1933	0	3	1
HIP_69528	LCGSHLVEA	NEKDRADQT	LCGSHLVEANEKDRADQT		SCG1	43799/367830	0.896	69528	0.786	0.190441	-1.4	0	-1	18	-0.9222	-2	0	0
HIP_69529	VCGERGFF	GAPGEEDIQ	VCGERGFFGAPGEEDIQ		SCG1	5948/367830	0.999	69529	0.786	-0.012828	0.8333	0.5	-3	17	-0.3882	-3	2	1
HIP_69530	PGAGSLQPL	NQGSSQET	PGAGSLQPLNQGSSQET		SCG2	16362/334890	0.997	69530	0.786	0.003166	-1.45	0.3333	-1	17	-0.9176	-1	0	1
HIP_69531	CGSHLVEAL	TREEKRFLG	CGSHLVEALTREEKRFLG		SCG1	47059/367830	0.874	69531	0.786	0.167890	-1.1	0	1	18	-0.3444	0	0	0
HIP_69532	GSLQPLALE	PSSKDVMEK	GSLQPLALEPSSKDVMEK		CMGA	13815/247050	0.989	69532	0.786	0.174361	-0.1833	0.5	-1	18	-0.4722	-1	0	0
HIP_69533	EQCCTSICS	FRGGKSGEL	EQCCTSICSFRGGKSGEL		CMGA	17703/247050	0.980	69533	0.786	0.077641	0.6833	0.3333	0	18	-0.2556	0	1	0

HIP_69997	VEQCCTSIC	EGNNQAPGE	VEQCCTSICEGNNQAPGE		CMGA	7671/247050	0.997	69997	0.785	0.073579	-0.2	0.3333	-3	18	-0.5778	-3	0
HIP_69998	LYQLENYCN	SQAELKEAV	LYQLENYCNSQAELKEAV		CMGA	11734/247050	0.993	69998	0.785	-0.057711	-0.8	0.1667	-2	18	-0.5611	-2	1
HIP_69999	SLQPLAL	ELGGGPGA	SLQPLALELGGGPGA		INSC	235/5184	0.997	69999	0.785	0.151575	1.55	0.1667	-1	15	0.4133	-1	0
HIP_70000	DLQVGQVEL	IENLRQQAH	DLQVGQVELIENLRQQAH		SCG2	26020/334890	0.993	70000	0.785	-0.018493	0.3333	0	-1	18	-0.5556	-2	0
HIP_70001	LVEALYLVC	MASLGEKR	LVEALYLVCMASLGEKR		SCG1	26296/367830	0.977	70001	0.785	0.043980	2.2333	0.1667	0	17	0.8059	0	1
HIP_70002	PLALEGSLQ	DRNYLNYGE	PLALEGSLQDRNYLNYGE		SCG1	38356/367830	0.927	70002	0.785	0.000509	-2	0.1667	-2	18	-0.7944	-2	1
HIP_70003	RGFFYTPKT	VDGAGKPGA	RGFFYTPKTVDGAGKPGA		CMGA	21060/247050	0.968	70003	0.785	-0.069154	-0.9833	0.3333	2	18	-0.55	2	1
HIP_70004	FVNQHLCGS	GEDWNPVEE	FVNQHLCGSGEDWNPVEE		SCG2	15089/334890	0.998	70004	0.785	-0.052231	-1.0167	0.5	-3	18	-0.7944	-4	1
HIP_70005	LVEALYLVC	APEDLEWE	LVEALYLVCAPEDLEWE		SCG1	15637/367830	0.994	70005	0.785	0.085997	1.2	0.1667	-5	17	0.4941	-5	1
HIP_70006	LYQLENYCN	LKREPLNYL	LYQLENYCNLKREPLNYL		IAPP	5702/45018	0.900	70006	0.785	-0.006364	-1.15	0	0	18	-0.7444	0	1
HIP_70007	VCGERGFF	EEEGSANR	VCGERGFFEEEGSANR		CMGA	18777/247050	0.976	70007	0.785	0.055793	-0.8833	0.1667	-2	16	-0.9	-2	2
HIP_70008	LVEALYLV	GSSEDDLQE	LVEALYLVGSSEDDLQE		SCG2	11934/334890	0.998	70008	0.785	0.161458	0.7833	0.5	-5	17	0.0647	-5	1
HIP_70009	PGAGSLQPL	WQAKREEEE	PGAGSLQPLWQAKREEEE		CMGA	10054/247050	0.995	70009	0.785	-0.021169	-0.65	0.1667	-2	18	-1.3278	-2	1
HIP_70010	VEALYLVCG	ECFETLRGD	VEALYLVCGECFETLRGD		CMGA	9341/247050	0.995	70010	0.785	-0.051347	1.35	0.1667	-3	18	0.45	-3	2
HIP_70011	GGPGAGSLQ	PAEDMARYY	GGPGAGSLQPAEDMARYY		NPY	2740/49410	0.979	70011	0.785	-0.184493	-0.6333	0.3333	-1	18	-0.6722	-1	0
HIP_70012	VNQHLCGSH	SSEEKHLEE	VNQHLCGSHSSEEKHLEE		SCG1	22586/367830	0.985	70012	0.785	-0.127570	-1.5833	0.6667	0	18	-1.2778	-3	0
HIP_70013	LQPLALEGS	RGGEPRAYF	LQPLALEGSRGGEPRAYF		SCG1	24027/367830	0.982	70013	0.785	-0.076160	-1.6667	0.6667	0	18	-0.4556	0	0
HIP_70014	IVEQCCT	VALNHLKA	IVEQCCTVALNHLKA		IAPP	3167/45018	0.975	70014	0.785	0.052512	2.35	0	1	15	0.72	0	0
HIP_70015	EAEDLQVGQ	SEEPVSTHQ	EAEDLQVGQSEEPVSTHQ		SCG1	15269/367830	0.995	70015	0.785	-0.128640	-1.25	0.3333	-4	18	-1.1944	-5	0
HIP_70016	GERGFFY	SGLTLAL	GERGFFYSGLTLAL		NPY	2255/49410	0.984	70016	0.785	0.164617	1.15	0.3333	0	14	0.4857	0	3
HIP_70017	GAGSLQPLA	QECFETLRG	GAGSLQPLAQECFETLRG		CMGA	15372/247050	0.986	70017	0.785	-0.058285	-0.0833	0.1667	-1	18	-0.1389	-1	1
HIP_70018	YLVCGERGF	PGETQNAFL	YLVCGERGFPGETQNAFL		SCG1	15364/367830	0.995	70018	0.785	-0.003288	-1.2667	0.5	-1	18	-0.0889	-1	1
HIP_70019	HLVEALYLV	DSKRWSKM	HLVEALYLVDSKRWSKM		CMGA	23398/247050	0.959	70019	0.785	0.253618	-0.25	0.1667	2	17	-0.1647	1	1
HIP_70020	SICSLYQLE	EEAEAGEEA	SICSLYQLEEEAEAGEEA		CMGA	17570/247050	0.980	70020	0.785	-0.137414	-1.4	0	-6	18	-0.4333	-6	1
HIP_70021	HLCGSHLV	FFPEYNYDW	HLCGSHLVFFPEYNYDW		SCG1	19344/367830	0.990	70021	0.785	0.005248	1.4667	0.1667	0	17	-0.1941	-2	3
HIP_70022	RGFFYTPK	GEKRDHHS	RGFFYTPKGEKRDHHS		SCG1	40914/367830	0.913	70022	0.785	-0.010452	-2.3333	0.3333	4	16	-1.8625	2	2
HIP_70023	NQHLCGSHL	PQEESEESN	NQHLCGSHLPQEESEESN		SCG1	19182/367830	0.991	70023	0.785	0.049661	-1.4667	0.3333	-2	18	-1.5944	-4	0
HIP_70024	CCTSICSLY	RERMDEEQK	CCTSICSLYRERMDEEQK		SCG2	37994/334890	0.981	70024	0.785	0.060563	-1.8	0.1667	-1	18	-0.9056	-1	1
HIP_70025	QCCTSICSL	AFLNERKQA	QCCTSICSLAFLNERKQA		SCG1	24610/367830	0.981	70025	0.785	0.056227	2.3167	0.1667	1	18	0.0722	1	1
HIP_70026	VCGERGFF	GMESAANQK	VCGERGFFGMESAANQK		SCG2	19151/334890	0.996	70026	0.785	0.036224	0.5333	0.3333	0	17	-0.3882	0	2
HIP_70027	GSLQPLAL	LPKAAWIPH	GSLQPLALLPKAAWIPH		SCG2	29402/334890	0.991	70027	0.785	0.188681	1.2833	0.1667	2	17	0.4471	1	0
HIP_70028	SICSLYQLE	KGRHHRGRG	SICSLYQLEKGRHHRGRG		SCG1	47989/367830	0.867	70028	0.785	0.072127	-2	0.1667	5	18	-1.1278	3	1
HIP_70029	LGGGPGAGS	RGWRPSSRE	LGGGPGAGSRGWRPSSRE		CMGA	21786/247050	0.965	70029	0.785	0.018505	-0.8667	0.5	2	18	-1.1278	2	1
HIP_70030	GPGAGSLQ	NPYYDPLQW	GPGAGSLQNPYYDPLQW		SCG1	29004/367830	0.969	70030	0.785	-0.012900	-1.15	0.3333	-1	17	-0.8765	-1	2
HIP_70031	GAGSLQPLA	YNPYQGVS	GAGSLQPLAYNPYQGVS		SCG2	25287/334890	0.994	70031	0.785	0.030173	-0.4	0.3333	0	17	-0.2176	0	2
HIP_70032	SHLVEALY	SKDVMEKRE	SHLVEALYSKDVMEKRE		CMGA	22075/247050	0.964	70032	0.785	0.253783	-0.65	0.1667	0	17	-0.7471	-1	1
HIP_70033	NQHLCGSHL	RNQLLQKEP	NQHLCGSHLRNQLLQKEP		SCG2	41982/334890	0.972	70033	0.785	0.092030	-1.95	0.1667	3	18	-1.1611	1	0
HIP_70034	GGGPGAGSL	GSSEDDLQE	GGGPGAGSLGSSEDDLQE		SCG2	3729/334890	1.000	70034	0.785	-0.062172	0.1	0.8333	-4	18	-0.8056	-4	0
HIP_70035	LVEALYLVC	RSSPETLIS	LVEALYLVCRSSPETLIS		NPY	3858/49410	0.961	70035	0.785	0.218325	0.7333	0.3333	-1	18	0.8278	-1	1
HIP_70036	HLCGSHLV	YGEESEEER	HLCGSHLVYGEESEEER		SCG1	18414/367830	0.992	70036	0.785	0.158354	-0.0667	0.1667	-2	17	-1.0471	-4	1
HIP_70037	YLVCGERGF	QEEEEDNR	YLVCGERGFQEEEEDNR		CMGA	16912/247050	0.982	70037	0.785	-0.024667	-2.1	0.1667	-4	17	-1.5176	-4	1
HIP_70038	GSLQPLALE	HVENRQMAY	GSLQPLALEHVENRQMAY		SCG2	39955/334890	0.977	70038	0.785	-0.016239	-0.0667	0	0	18	-0.4556	-1	0
HIP_70039	QVELGGGPG	EEEEEMAVV	QVELGGGPGEEEEEMAVV		CMGA	8237/247050	0.996	70039	0.785	-0.081347	-2.15	0.5	-6	18	-0.4222	-6	0
HIP_70040	SHLVEALY	NEIVEEQYT	SHLVEALYNEIVEEQYT		SCG2	20417/334890	0.996	70040	0.785	0.126883	0.3	0	-3	17	-0.3529	-4	1
HIP_70041	LYQLENYCN	KVLEYLNQE	LYQLENYCNKVLEYLNQE		SCG2	20089/334890	0.996	70041	0.785	-0.115528	0.3	0	-2	18	-0.7722	-2	1
HIP_70042	PGAGSLQPL	SEESNVSMA	PGAGSLQPLSEESNVSMA		SCG1	23635/367830	0.983	70042	0.785	-0.024255	-1.5167	0.3333	-2	18	-0.2167	-2	0
HIP_70043	PGAGSLQPL	EESNVSMAS	PGAGSLQPLEESNVSMAS		SCG1	23636/367830	0.983	70043	0.785	-0.024255	-1.5167	0.3333	-2	18	-0.2167	-2	0
HIP_70044	VCGERGFFY	LGMESAAN	VCGERGFFYLGMESAAN		SCG2	13983/334890	0.998	70044	0.785	0.001344	1.6	0.1667	-1	17	0.1041	-1	2

HIP_70508	GIVEQCCTS	EEAQDPEGK	GIVEQCCTSEEAQDPEGK		CMGA	15800/247050	0.985	70508	0.785	-0.099082	-0.7	0.1667	-4	18	-0.9333	-4	0	0	0
HIP_70509	VEALYLVCG	RRGWRPSSR	VEALYLVCGRRGWRPSSR		CMGA	27255/247050	0.935	70509	0.785	0.145218	-0.5167	0.3333	3	18	-0.4111	3	2	0	0
HIP_70510	AGSLQPLAL	RNQLLQKE	AGSLQPLALRNQLLQKE		SCG2	34271/334890	0.986	70510	0.785	0.221388	-0.35	0	1	17	-0.3588	1	0	1	0
HIP_70511	QVGQVELGG	EPEYGEEIK	QVGQVELGGEPEYGEEIK		SCG1	14829/367830	0.995	70511	0.785	-0.094712	-0.9333	0.5	-4	18	-0.9	-4	1	0	0
HIP_70512	HLCGSHLV	FRGGKSGEL	HLCGSHLVFRGGKSGEL		CMGA	22444/247050	0.963	70512	0.785	0.230350	0.45	0.1667	3	17	-0.0353	1	1	1	0
HIP_70513	ERGFFYTP	NWGYEKRNL	ERGFFYTPNWGYEKRNL		SCG1	25629/367830	0.979	70513	0.785	-0.055309	-1.4	0.3333	1	17	-1.4176	1	4	1	0
HIP_70514	CTSICSLY	WNPVEEKIE	CTSICSLYWNPVEEKIE		SCG2	21349/334890	0.995	70514	0.785	0.015142	-0.7167	0.3333	-2	17	-0.1176	-2	2	1	0
HIP_70515	CTSICSLY	YYDPLQWKS	CTSICSLYYDPLQWKS		SCG1	34720/367830	0.946	70515	0.785	0.013187	-0.7333	0.1667	0	17	-0.1941	0	3	1	1
HIP_70516	AEDLQVGQV	TPGRAGTEA	AEDLQVGQVTPGRAGTEA		SCG2	6842/334890	0.999	70516	0.785	-0.049146	-0.4	0.5	-2	18	-0.4778	-2	0	0	0
HIP_70517	LVCGERGFF	QIPPEDLI	LVCGERGFFQIPPEDLI		SCG2	13444/334890	0.998	70517	0.785	-0.009614	0.7667	0.3333	-2	17	0.3765	-2	2	1	0
HIP_70518	SICSLYQLE	EERGLEPGK	SICSLYQLEEERGLEPGK		SCG1	42749/367830	0.902	70518	0.785	-0.020114	-2.45	0	-2	18	-0.7111	-2	1	0	1
HIP_70519	YLVCGERGF	FGAILSSTN	YLVCGERGFFGAILSSTN		IAPP	1827/45018	0.992	70519	0.785	-0.078164	0.35	0.3333	0	18	0.55	0	2	0	0
HIP_70520	GERGFFYT	YGEEIKGY	GERGFFYTYGEEIKGY		SCG1	29436/367830	0.967	70520	0.785	0.087828	-0.7333	0.1667	-1	16	-0.9375	-1	4	0	0
HIP_70521	LCGSHLVEA	NTKFEVRLL	LCGSHLVEANTKFEVRLL		SCG1	44421/367830	0.892	70521	0.785	0.173432	-0.9333	0	1	18	0.3722	0	1	0	0
HIP_70522	VCGERGFFY	RKLKHMQF	VCGERGFFYRKLKHMQF		SCG2	42528/334890	0.971	70522	0.785	0.171791	-0.05	0	4	17	-0.4882	3	3	1	0
HIP_70523	GGPGAGSL	GFFYTPKT	GGPGAGSLGFFYTPKT		INSB	173/4968	0.980	70523	0.785	-0.007083	1.3	0.5	1	16	-0.0875	1	3	0	0
HIP_70524	QHLCGSHLV	QSIYQLIE	QHLCGSHLVQSIYQLIE		SCG2	23971/334890	0.994	70524	0.785	0.189530	0.8333	0.1667	1	17	0.2	-1	1	1	0
HIP_70525	QHLCGSHLV	EEEEEEEE	QHLCGSHLVEEEEEEEE		CMGA	13085/247050	0.990	70525	0.785	0.081265	-0.95	0	-6	17	-1.4588	-8	0	1	6
HIP_70526	QHLCGSHLV	EEEEEEEE	QHLCGSHLVEEEEEEEE		CMGA	13086/247050	0.990	70526	0.785	0.081265	-0.95	0	-6	17	-1.4588	-8	0	1	6
HIP_70527	QHLCGSHLV	EEEEEEEE	QHLCGSHLVEEEEEEEE		CMGA	13087/247050	0.990	70527	0.785	0.081265	-0.95	0	-6	17	-1.4588	-8	0	1	6
HIP_70528	GSLQPLALE	GTEALPDGL	GSLQPLALEGTEALPDGL		SCG2	14490/334890	0.998	70528	0.785	0.120328	-0.4167	0.1667	-3	18	0.15	-3	0	0	0
HIP_70529	AGSLQPLAL	ELEKVAHQ	AGSLQPLALELEKVAHQ		CMGA	19184/247050	0.975	70529	0.785	0.127122	1.0333	0	0	17	0.0529	-1	0	1	0
HIP_70530	VEQCCTSIC	EQYTPQSLA	VEQCCTSICEQYTPQSLA		SCG2	25750/334890	0.993	70530	0.785	-0.002049	-0.35	0.1667	-2	18	-0.0889	-2	1	0	0
HIP_70531	CCTSICSLY	LLGMESAAN	CCTSICSLYLLGMESAAN		SCG2	15044/334890	0.998	70531	0.785	-0.088902	1.4833	0.3333	-1	18	0.95	-1	1	0	0
HIP_70532	LQVGQVEL	YFPNPYNQE	LQVGQVELYFPNPYNQE		SCG2	23027/334890	0.995	70532	0.785	-0.032180	0.7333	0.1667	-2	17	-0.7	-2	2	1	0
HIP_70533	GSHLVEALY	PSDSQVSE	GSHLVEALYPSDSQVSE		SCG1	19176/367830	0.991	70533	0.785	0.180197	-0.2667	0.3333	-2	17	-0.3471	-3	1	1	0
HIP_70534	LVEALYLV	TGEKPNGSV	LVEALYLVTGEKPNGSV		SCG2	19159/334890	0.996	70534	0.785	0.205505	0.35	0.1667	-1	17	0.3647	-1	1	1	0
HIP_70535	SHLVEALYL	LPDGLSVE	SHLVEALYLLPDGLSVE		SCG2	15261/334890	0.998	70535	0.785	0.142966	0.8333	0.1667	-2	17	0.6235	-3	1	1	0
HIP_70536	PLALEGSLQ	QQGDLQDTK	PLALEGSLQQQGDLQDTK		SCG1	14582/367830	0.995	70536	0.785	-0.053444	-1.3167	0.3333	-2	18	-0.85	-2	0	0	1
HIP_70537	EAEDLQVGQ	AYLKRLVNA	EAEDLQVGQAYLKRLVNA		SCG2	7430/334890	0.999	70537	0.785	0.090101	0.7667	0.1667	-1	18	-0.3278	-1	1	0	0
HIP_70538	GGPGAGSL	ALAEAYPS	GGPGAGSLALAEAYPS		NPY	1363/49410	0.993	70538	0.785	0.153696	1.6667	0.3333	-1	16	0.225	-1	0	0	0
HIP_70539	AGSLQPLAL	EQKLYTDD	AGSLQPLALEQKLYTDD		SCG2	27948/334890	0.992	70539	0.785	0.208195	-0.25	0	-2	17	-0.4353	-2	0	1	0
HIP_70540	LYLVCGERG	GEEIKGYPG	LYLVCGERGGEEIKGYPG		SCG1	33099/367830	0.953	70540	0.785	-0.115797	-2.6333	0.3333	-1	18	-0.35	-1	0	0	0
HIP_70541	LVEALYLV	DWWEKKPF	LVEALYLVDWWEKKPF		SCG1	31179/367830	0.961	70541	0.785	0.069806	0.2333	0	-1	16	0.0875	-1	3	0	0
HIP_70542	GERGFFYTP	ESNVSMAS	GERGFFYTPESNVSMAS		SCG1	28428/367830	0.971	70542	0.785	-0.051759	-1.9	0.3333	-1	17	-0.4882	-1	3	1	0
HIP_70543	CSLYQLENY	QLAKELTAE	CSLYQLENYQLAKELTAE		CMGA	14570/247050	0.988	70543	0.785	-0.055202	-1.0333	0	-2	18	-0.4278	-2	1	0	0
HIP_70544	YLVCGERGF	EQKLYTDDE	YLVCGERGFEQKLYTDDE		SCG2	26934/334890	0.992	70544	0.785	-0.060475	-2.1667	0.1667	-3	18	-0.9111	-3	1	0	0
HIP_70545	PGAGSLQP	NGQNGERAT	PGAGSLQPNGQNGERAT		SCG2	20566/334890	0.996	70545	0.785	0.018727	-1.45	0.3333	0	17	-1.2294	0	0	1	0
HIP_70546	ERGFFYTP	GETQNAFLN	ERGFFYTPGETQNAFLN		SCG1	18838/367830	0.991	70546	0.785	-0.065795	-1.3667	0.3333	-1	17	-0.7706	-1	2	1	0
HIP_70547	VEQCCTSIC	EKGREHIAK	VEQCCTSICEKGREHIAK		SCG2	25885/334890	0.993	70547	0.785	0.173203	-0.2667	0.3333	1	18	-0.4944	0	0	0	0
HIP_70548	QPLALEGSL	NQSSQAELK	QPLALEGSLNQSSQAELK		CMGA	11782/247050	0.992	70548	0.785	-0.102631	-0.8667	0.5	-1	18	-0.5833	-1	0	0	0
HIP_70549	GSLQPLAL	ENGDESHL	GSLQPLALENGDESHL		SCG2	18132/334890	0.997	70549	0.785	0.203297	0.3333	0.1667	-2	16	-0.4813	-3	0	0	0
HIP_70550	DLQVGQVEL	GEKRDHHST	DLQVGQVELGEKRDHHST		SCG1	34703/367830	0.946	70550	0.785	0.213810	-0.55	0.1667	0	18	-1.2278	-2	0	0	0

HIP_71019	LYLVCGERG	EPRAYFMSD	LYLVCGERGEPRAYFMSD		SCG1	34043/367830	0.949	71019	0.784	-0.107797	-3	0.3333	-1	18	-0.25	-1	0	0	0
HIP_71020	HLVEALYL	AYEDVVGG	HLVEALYLAYEDVVGG		SCG2	26969/334890	0.992	71020	0.784	0.068531	0.55	0	-2	16	0.6562	-3	2	0	0
HIP_71021	GPGAGSLQP	EQCCTSICS	GPGAGSLQPEQCCTSICS		INSA	206/3024	0.902	71021	0.784	-0.133689	-0.9667	0.1667	-1	18	-0.0222	-1	0	0	0
HIP_71022	ALYLVCGER	EEDATSEVD	ALYLVCGEREEDATSEVD		SCG1	29476/367830	0.967	71022	0.784	-0.126091	-3.15	0.1667	-5	18	-0.3667	-5	0	0	0
HIP_71023	HLVEALYL	LDSQSIYQL	HLVEALYLLDSQSIYQL		SCG2	19185/334890	0.996	71023	0.784	0.156272	0.9667	0.1667	-1	17	0.4765	-2	1	1	0
HIP_71024	VEQCCTSIC	PERKLKHMQ	VEQCCTSICPERKLKHMQ		SCG2	33056/334890	0.987	71024	0.784	0.158350	-0.5667	0.3333	2	18	-0.5944	1	0	0	0
HIP_71025	GSLQPLALE	RILEALRQ	GSLQPLALERILEALRQ		SCG2	28928/334890	0.991	71025	0.784	-0.022222	1.1	0	0	18	0.3222	0	0	0	0
HIP_71026	LYQLENYCN	LEALRQAEN	LYQLENYCNLEALRQAEN		SCG2	22308/334890	0.995	71026	0.784	-0.171721	-0.0333	0	-2	18	-0.7667	-2	1	0	0
HIP_71027	VEALYLVCG	AENEKDRAD	VEALYLVCGAENEKDRAD		SCG1	17214/367830	0.993	71027	0.784	-0.045263	0.1833	0.1667	-3	18	-0.4	-3	1	0	0
HIP_71028	AEDLQVGQV	AWIPHVENR	AEDLQVGQVAWIPHVENR		SCG2	6762/334890	0.999	71028	0.784	0.099508	0.95	0.1667	-1	18	-0.3944	-2	1	0	0
HIP_71029	RGFFYTPKT	INDEMKRSG	RGFFYTPKTINDEMKRSG		SCG2	34136/334890	0.986	71029	0.784	0.000506	-1.45	0.1667	2	18	-1.1778	2	1	0	0
HIP_71030	CGSHLVEAL	EKIESQTQE	CGSHLVEALEKIESQTQE		SCG2	33801/334890	0.986	71030	0.784	-0.006311	-0.1333	0	-2	18	-0.5667	-3	0	0	0
HIP_71031	LCGSHLVE	DFYDSEEPV	LCGSHLVEDFYDSEEPV		SCG1	29344/367830	0.968	71031	0.784	-0.016563	0.4167	0	-4	17	-0.2529	-5	2	1	0
HIP_71032	CCTSICSLY	IENLRQQAH	CCTSICSLYIENLRQQAH		SCG2	25514/334890	0.993	71032	0.784	-0.091193	-0.1333	0.1667	1	18	0.0333	0	1	0	0
HIP_71033	VEQCCTSIC	EYLNQEKAE	VEQCCTSICEYLNQEKAE		SCG2	18591/334890	0.997	71033	0.784	-0.076754	0.8667	0.1667	-3	18	-0.5222	-3	1	0	0
HIP_71034	LVCGERGFF	PDLRLENV	LVCGERGFFPDLRLENV		SCG2	18768/334890	0.996	71034	0.784	0.020939	0.65	0.3333	-1	17	0.1471	-1	2	1	0
HIP_71035	CGSHLVEAL	IEISRNLQI	CGSHLVEALIEISRNLQI		SCG2	26486/334890	0.993	71035	0.784	-0.033147	1.2667	0	0	18	0.5389	-1	0	0	0
HIP_71036	FVNQHLCG	EEEEEEEEE	FVNQHLCGEEEEEEEEE		CMGA	12003/247050	0.992	71036	0.784	-0.021433	-0.7667	0.1667	-8	17	-1.6941	-9	0	1	7
HIP_71037	GSLQPLAL	AEAYPSKP	GSLQPLALAEAYPSKP		NPY	4235/49410	0.952	71037	0.784	0.086908	1.5833	0	0	16	-0.1375	0	1	0	0
HIP_71038	YLVCGERGF	EEPVSTHQE	YLVCGERGFEEPVSTHQE		SCG1	21589/367830	0.987	71038	0.784	-0.073373	-1.7833	0.3333	-2	18	-0.7167	-3	1	0	0
HIP_71039	QVELGGGPG	MARYYSALR	QVELGGGPGMARYYSALR		NPY	1737/49410	0.989	71039	0.784	0.144535	-0.5333	0.5	1	18	-0.2944	1	1	0	1
HIP_71040	QHLCGSHLV	DTREEKRFL	QHLCGSHLVDTREEKRFL		SCG1	37525/367830	0.932	71040	0.784	0.249301	-0.65	0	2	18	-0.7944	0	0	0	0
HIP_71041	GSLQPLALE	ENHPQESKG	GSLQPLALEENHPQESKG		SCG1	46083/367830	0.881	71041	0.784	0.036554	-1.35	0	-1	18	-1.1389	-2	0	0	0
HIP_71042	GIVEQCCTS	GEPRAYFMS	GIVEQCCTSGEPRAYFMS		SCG1	15584/367830	0.994	71042	0.784	0.021368	-0.75	0.5	-1	18	-0.0444	-1	0	0	0
HIP_71043	TSICSLYQL	EKSGEATDG	TSICSLYQLEKSGEATDG		CMGA	21974/247050	0.965	71043	0.784	0.197480	-1.5333	0.1667	-2	18	-0.4111	-2	1	0	0
HIP_71044	ELGGGPGAG	PEGKGEQEH	ELGGGPGAGPEGKGEQEH		CMGA	5436/247050	0.998	71044	0.784	-0.106110	-0.75	0.6667	-2	18	-1.3889	-3	0	0	1
HIP_71045	AGSLQPLA	GGPGAGSLQ	AGSLQPLAGGPGAGSLQ		INSC	171/5184	0.998	71045	0.784	0.153132	0.2667	0.6667	0	17	0.1765	0	0	1	0
HIP_71046	LYQLENYCN	ELQKIAEKF	LYQLENYCNELQKIAEKF		SCG1	36592/367830	0.937	71046	0.784	-0.025824	-0.9167	0	-1	18	-0.6611	-1	1	0	0
HIP_71047	CGERGFFYT	SNVSMASL	CGERGFFYTSNVSMASL		SCG1	26597/367830	0.976	71047	0.784	0.057286	0.1167	0.1667	0	17	0.1824	0	3	1	0
HIP_71048	CGERGFFY	GPTVVLNP	CGERGFFYGPTVVLNP		CMGA	14217/247050	0.989	71048	0.784	0.185582	0.2667	0.3333	0	16	0.15	0	3	0	0
HIP_71049	GSLQPLAL	SRSNQLPKA	GSLQPLALSRSNQLPKA		SCG2	30647/334890	0.990	71049	0.784	0.251605	0.55	0.3333	2	17	-0.3588	2	0	1	0
HIP_71050	ERGFFYTPK	QGAKERAHQ	ERGFFYTPKQGAKERAHQ		CMGA	21131/247050	0.968	71050	0.784	-0.056759	-1.3833	0.3333	3	18	-1.6222	2	2	0	0
HIP_71051	GSHLVEALY	YLNYGEEG	GSHLVEALYYLNYGEEG		SCG1	26038/367830	0.978	71051	0.784	0.011047	0.55	0	-2	17	-0.3353	-3	3	1	0
HIP_71052	LVCGERGFF	FPPMYEEN	LVCGERGFFFPPMYEEN		SCG2	12904/334890	0.998	71052	0.784	-0.014871	0.8	0.5	-2	17	-0.1765	-2	3	1	1
HIP_71053	QPLALEGSL	PYYDPLQWK	QPLALEGSLPYYDPLQWK		SCG1	16920/367830	0.993	71053	0.784	-0.013058	-0.2667	0.5	-1	18	-0.5778	-1	2	0	0
HIP_71054	EQCCTSICS	KRNYPSLEL	EQCCTSICSKRNYPSLEL		SCG1	44620/367830	0.891	71054	0.784	0.128784	-0.95	0.1667	0	18	-0.4889	0	1	0	0
HIP_71055	VCGERGFF	SGRLQNGQ	VCGERGFFSGRLQNGQ		SCG2	26721/334890	0.993	71055	0.784	0.148745	-0.0833	0.5	1	16	-0.5813	1	2	0	0
HIP_71056	GIVEQCCTS	GRGGEPRAY	GIVEQCCTSGRGGEPRAY		SCG1	22734/367830	0.985	71056	0.784	0.089692	-0.7167	0.5	0	18	-0.5556	0	0	0	0
HIP_71057	GQVELGGGP	EEEAEAGEE	GQVELGGGPEEEAEAGEE		CMGA	7928/247050	0.997	71057	0.784	-0.130695	-2.15	0.5	-7	18	-1.1111	-7	0	0	2
HIP_71058	VEALYLVCG	AEKGREHIA	VEALYLVCGAEKGREHIA		SCG2	29084/334890	0.991	71058	0.784	0.025170	0.1167	0.1667	0	18	0.2333	-1	1	0	0
HIP_71059	CGERGFFY	ATCATQRLA	CGERGFFYATCATQRLA		IAPP	4150/45018	0.954	71059	0.784	0.065075	1.3167	0	1	17	0.0176	1	3	1	0
HIP_71060	HLVEALYL	GAGKPGAEE	HLVEALYLGAGKPGAEE		CMGA	9010/247050	0.996	71060	0.784	0.168826	1.2167	0.3333	-1	17	-0.0412	-2	1	1	0
HIP_71061	IVEQCCTSI	GPQLRRGWR	IVEQCCTSIGPQLRRGWR		CMGA	14706/247050	0.987	71061	0.784	0.041474	-0.4167	0.5	2	18	-0.3778	2	0	0	0
HIP_71062	YLVCGERGF	SIYQLEIS	YLVCGERGFSIYQLEIS		SCG2	14200/334890	0.998	71062	0.784	-0.021990	0.05	0.3333	-1	18	0.5889	-1	2	0	0
HIP_71063	VEQCCTSIC	FERRDNMND	VEQCCTSICFERRDNMND		SCG1	32740/367830	0.954	71063	0.784	-0.007731	0.1667	0.1667	-2	18	-0.7833	-2	1	0	0
HIP_71064	EQCCTSICS	GAGRSRSNQ	EQCCTSICSGAGRSRSNQ		SCG2	13803/334890	0.998	71064	0.784	0.111152	1.2	0.5	1	18	-0.7722	1	0	0	0
HIP_71065	HLCGSHLVE	LDLPVDLDO	HLCGSHLVELDLPVDLDO		SCG2	20367/334890	0.996	71065	0.784	-0.072816	1.4333	0	-3	18	0.1778	-5	0	0	0
HIP_71066	EQCCTSICS	LEEPGETQN	EQCCTSICSLEEPGETQN		SCG1	19052/367830	0.991	71066	0.784	-0.080775	0.5	0.1667	-4	18	-0.7611	-4	0	0	0
HIP_71067	CSLYQLENY	WLGAALSLI	CSLYQLENYWLGAALSLI		SCG2	17552/334890	0.997	71067	0.784	-0.116359	-0.9667	0.1667	-1	18	0.7556	-1	2	0	0
HIP_71068	CGERGFFYT	NTYGKRNA	CGERGFFYTNTYGKRNA		IAPP	4945/45018	0.929	71068	0.784	0.094990	-0.7833	0	2	17	-1.1	2	4	1	0
HIP_71069	RGFFYTPKT	PMYEENSRD	RGFFYTPKTPMYEENSRD		SCG2	20037/334890	0.996	71069	0.784	0.023339	-1.2	0.3333	0	18	-1.5444	0	2	0	0
HIP_71070	HLVEALYL	TPIESHQV	HLVEALYLTPIESHQV		IAPP	2399/45018	0.986	71070	0.784	0.166311	1.4167	0.1667	0	16	0.3	-2	1	0	0
HIP_71071	LVEALYLV	SEADLDHPD	LVEALYLVSEADLDHPD		SCG2	14463/334890	0.998	71071	0.784	0.069479	0.7	0.1667	-4	17	0.1647	-5	1	1	0
HIP_71072	VCGERGFF	NQLPKAAW	VCGERGFFNQLPKAAW		SCG2	28528/334890	0.991	71072	0.784	0.060519	0.3333	0.1667	1	16	-0.1563	1	2	0	0
HIP_71073	LVCGERGFF	PITPECRQV	LVCGERGFFPITPECRQV		SCG1	15771/367830	0.994	71073	0.784	-0.008767	1.2333	0.3333	0	18	0.1722	0	2	0	0
HIP_71074	VCGERGFF	NEKDRADQ	VCGERGFFNEKDRADQ		SCG1	35378/367830	0.943	71074	0.784	0.105616	-0.95	0.1667	-1	16	-1.2875	-1	2	0	0
HIP_71075	LGGGPGAGS	GSNTYGKRN	LGGGPGAGSGSNTYGKRN		IAPP	2949/45018	0.979	71075	0.784	0.010008	-0.6833	0.6667	2	18	-0.9889	2	0	0	1
HIP_71076	AEDLQVGQV	PYYDPLQWK	AEDLQVGQVPYYDPLQWK		SCG1	14805/367830	0.995	71076	0.784	-0.031013	-0.65	0.3333	-2	18	-0.7889	-2	2	0	0
[illegible]																			

HIP_71530	SHLVEALY	NRMLSKSGY	SHLVEALYNRMLSKSGY		SCG2	41961/334890	0.972	71530	0.784	0.232805	-0.3	0	2	17	-0.2765	1	1	1
HIP_71531	GSLQPLAL	EESEESNV	GSLQPLALEESEESNV		SCG1	21351/367830	0.987	71531	0.784	0.156135	0.2667	0.1667	-4	16	-0.5	-4	0	0
HIP_71532	GGPGAGSLQ	NQEKVLPRL	GGPGAGSLQNQEKVLPRL		SCG2	36245/334890	0.983	71532	0.784	-0.033270	-1.8333	0.1667	1	18	-0.5889	1	0	0
HIP_71533	CGERGFFYT	SLIPLIFL	CGERGFFYTSLIPLIFL		SCG2	23725/334890	0.994	71533	0.784	-0.059731	1.3833	0.1667	0	17	1.0647	0	3	1
HIP_71534	HLVEALYL	EEEEEGEN	HLVEALYLEEEEEGEN		SCG1	22643/367830	0.985	71534	0.784	0.122051	-0.7	0	-6	16	-0.9688	-7	1	0
HIP_71535	LYQLENYCN	LSVEDILNL	LYQLENYCNLSVEDILNL		SCG2	11949/334890	0.998	71535	0.784	-0.165433	0.8167	0.1667	-3	18	0.1278	-3	1	0
HIP_71536	HLVEALYL	ESKDQLSDD	HLVEALYLESKDQLSDD		SCG2	24981/334890	0.994	71536	0.784	0.205672	-0.3167	0.1667	-3	17	-0.5765	-4	1	1
HIP_71537	SICSLYQLE	ELQQLALQG	SICSLYQLEELQQLALQG		CMGA	20486/247050	0.970	71537	0.784	-0.148393	-1.0667	0	-3	18	0.1944	-3	1	0
HIP_71538	QVELGGGPG	SSQETDKLA	QVELGGGPGSSQETDKLA		SCG2	10807/334890	0.999	71538	0.784	-0.094282	-1.25	0.8333	-2	18	-0.7389	-2	0	0
HIP_71539	CGERGFFY	RFLGEGHHR	CGERGFFYRFLGEGHHR		SCG1	39748/367830	0.920	71539	0.784	0.083681	1.0667	0	3	17	-0.8882	1	4	1
HIP_71540	EAEDLQVGQ	NPYQGVSVP	EAEDLQVGQNPYQGVSVP		SCG2	12040/334890	0.998	71540	0.784	-0.140627	-1.0167	0.3333	-3	18	-0.6889	-3	1	0
HIP_71541	GERGFFY	LSSTNVGSN	GERGFFYLSSTNVGSN		IAPP	3130/45018	0.976	71541	0.784	0.182562	1.0833	0.3333	0	16	-0.4375	0	3	2
HIP_71542	QHLCGSHLV	KKSAEFPDF	QHLCGSHLVKKSAEFPDF		SCG1	38354/367830	0.928	71542	0.784	0.213134	-0.6333	0.1667	2	18	-0.3667	0	0	0
HIP_71543	SICSLYQLE	TENENTKFE	SICSLYQLETENENTKFE		SCG1	33850/367830	0.950	71543	0.784	-0.091156	-1.8167	0	-3	18	-0.85	-3	1	0
HIP_71544	HLVEALYL	PSEEKGHP	HLVEALYLPSEEKGHP		SCG1	24645/367830	0.981	71544	0.784	0.230361	0.0667	0.3333	0	16	-0.5688	-2	1	0
HIP_71545	HLCGSHLVE	MDLELQKIA	HLCGSHLVEMDLELQKIA		SCG1	32092/367830	0.957	71545	0.784	-0.022184	1.1167	0	0	18	0.2556	-2	0	0
HIP_71546	QCCTSICSL	YRGRGSEEY	QCCTSICSLYRGRGSEEY		SCG1	36707/367830	0.936	71546	0.784	0.135109	-0.1167	0.3333	0	18	-0.5667	0	1	0
HIP_71547	GSHLVEALY	YRAPRPQS	GSHLVEALYYRAPRPQS		SCG1	43477/367830	0.898	71547	0.784	0.088690	0.05	0	2	17	-0.6824	1	2	1
HIP_71548	QVGQVELGG	GYPEEKKEE	QVGQVELGGGYPEEKKEE		CMGA	4713/247050	0.999	71548	0.784	-0.122472	-0.05	0.6667	-3	18	-1.3667	-3	1	0
HIP_71549	TSICSLYQL	FLGEGHHRV	TSICSLYQLFLGEGHHRV		SCG1	23237/367830	0.984	71549	0.784	-0.066433	0.8667	0.1667	2	18	0.1722	0	2	0
HIP_71550	SICSLYQLE	GSNTYGKRN	SICSLYQLEGSNTYGKRN		IAPP	3340/45018	0.972	71550	0.784	0.013352	-1.3167	0.3333	1	18	-0.7944	1	1	0
HIP_71551	GSHLVEALY	PQSLATLE	GSHLVEALYPQSLATLE		SCG2	18766/334890	0.996	71551	0.784	0.171042	-0.2667	0.3333	-1	17	0.2176	-2	1	1
HIP_71552	AGSLQPLAL	LTLNEKNF	AGSLQPLALLTLNEKNF		SCG1	28118/367830	0.972	71552	0.784	0.044708	2.7167	0	0	17	0.2353	0	0	1
HIP_71553	PLALEGSLQ	SETHAAGHS	PLALEGSLQSETHAAGHS		SCG1	25129/367830	0.980	71553	0.784	-0.078452	-0.9167	0.3333	0	18	-0.3111	-2	0	0
HIP_71554	PLALEGSLQ	GRGSEEYRA	PLALEGSLQGRGSEEYRA		SCG1	16486/367830	0.994	71554	0.784	0.042568	-0.9667	0.5	-1	18	-0.7611	-1	0	0
HIP_71555	CSLYQLENY	EGLSQGLVD	CSLYQLENYEGLSQGLVD		CMGA	13589/247050	0.990	71555	0.784	-0.149397	-1.4	0.1667	-3	18	-0.2278	-3	1	0
HIP_71556	GERGFFY	SLGYKEI	GERGFFYSLGYKEI		CMGA	12434/247050	0.991	71556	0.784	0.158151	1.15	0.3333	0	14	-0.4357	0	4	0
HIP_71557	CTSICSLY	SALRHYINL	CTSICSLYSALRHYINL		NPY	3889/49410	0.960	71557	0.784	-0.017274	1.0833	0.3333	2	17	0.6059	1	1	1
HIP_71558	CTSICSLYQ	SDDVSKVIA	CTSICSLYQSDDVSKVIA		SCG2	24158/334890	0.994	71558	0.784	0.097859	-1.4667	0.1667	-1	18	0.4667	-1	1	0
HIP_71559	QPLALEGSL	SQREDEEEE	QPLALEGSLSQREDEEEE		SCG1	18310/367830	0.992	71559	0.784	-0.115788	-1.0333	0.5	-6	18	-1.4667	-6	0	0
HIP_71560	IVEQCCTSI	FRGGKSGEL	IVEQCCTSIFRGGKSGEL		CMGA	16383/247050	0.983	71560	0.784	-0.037653	0.15	0.3333	0	18	0.1333	0	1	0
HIP_71561	GERGFFYTP	RHHRGRGGE	GERGFFYTPRHHRGRGGE		SCG1	45052/367830	0.888	71561	0.784	0.053394	-2.4167	0.1667	4	18	-1.7444	2	3	0
HIP_71562	LCGSHLVEA	EATNTHPPA	LCGSHLVEAEATNTHPPA		CMGA	25848/247050	0.945	71562	0.784	0.069663	0.0167	0	0	18	-0.1667	-2	0	0
HIP_71563	VELGGGPGA	GSNTYGKRN	VELGGGPGAGSNTYGKRN		IAPP	2322/45018	0.987	71563	0.784	-0.016930	-0.8167	0.6667	1	18	-0.8833	1	0	0
HIP_71564	HLVEALYL	SEEGEEDA	HLVEALYLSEEGEEDA		SCG1	21435/367830	0.987	71564	0.784	0.160898	-0.25	0.1667	-5	16	-0.4688	-6	1	0
HIP_71565	QVGQVELGG	GEETGSQEN	QVGQVELGGGEETGSQEN		SCG1	10666/367830	0.997	71565	0.784	-0.120214	-0.7333	0.5	-4	18	-1.0722	-4	0	0
HIP_71566	VCGERGFFY	LLKELQDL	VCGERGFFYLLKELQDL		CMGA	19199/247050	0.975	71566	0.784	0.008469	1.3333	0	-1	17	0.1765	-1	3	1
HIP_71567	AGSLQPLAL	GYPGVQAP	AGSLQPLALGYPGVQAP		SCG1	22873/367830	0.985	71567	0.784	0.220150	1.0167	0.3333	0	17	0.3471	0	1	1
HIP_71568	VEQCCTSIC	PSQKYPGPQ	VEQCCTSICPSQKYPGPQ		CMGA	13803/247050	0.989	71568	0.784	0.137683	0.05	0.5	0	18	-0.5833	0	0	0
HIP_71569	ELGGGPGAG	GEEIKGYPG	ELGGGPGAGGEEIKGYPG		SCG1	13151/367830	0.996	71569	0.784	-0.128250	-1.0667	0.5	-2	18	-0.6667	-2	0	0
HIP_71570	QCCTSICSL	KRERMDEEQ	QCCTSICSLKRERMDEEQ		SCG2	43378/334890	0.968	71570	0.784	0.169680	-1.0667	0.1667	-1	18	-1.0278	-1	0	0
HIP_71571	HLCGSHLVE	RQQAHKEES	HLCGSHLVERQQAHKEES		SCG2	41615/334890	0.973	71571	0.784	0.120541	-1.1667	0	2	18	-1.1889	-1	0	0
HIP_71572	GPGAGSLQP	GQNGERAT	GPGAGSLQPGQNGERAT		SCG2	20568/334890	0.996	71572	0.784	0.019137	-1.45	0.3333	0	17	-1.0471	0	0	1
HIP_71573	ELGGGPGAG	SNNFGAILS	ELGGGPGAGSNNFGAILS		IAPP	2226/45018	0.989	71573	0.784	-0.076381	-1.1333	0.5	-1	18	0.1333	-1	1	0
HIP_71574	LYQLENYCN	PGQGSSED	LYQLENYCNPGQGSSED		SCG2	12338/334890	0.998	71574	0.784	0.090820	-1.3	0.3333	-3	17	-1.2353	-3	1	1
HIP_71575	GAGSLQPLA	HRGRGGEPR	GAGSLQPLAHRGRGGEPR		SCG1	38740/367830	0.925	71575	0.784	0.065816	-0.6833	0.3333	3	18	-1.0278	2	0	0
HIP_71576	SLQPLAL	ASEEEPEYG	SLQPLALASEEEPEYG		SCG1	18817/367830	0.991	71576	0.784	0.127103	1.15	0.1667	-4	16	-0.5625	-4	0	2
HIP_71577	SLYQLENY	EEEATNTHP	SLYQLENYEEEATNTHP		CMGA	25724/247050	0.946	71577	0.784	-0.063762	-3.1333	0	-3	17	-1.4529	-4	1	1
[illegible]																		

HIP_72041	VEALYLVCG	QEAENEKDR	VEALYLVCGQEAENEKDR		SCG1	20532/367830	0.989	72041	0.783	-0.028508	0.1833	0.1667	-3	18	-0.6944	-3	1	0	0
HIP_72042	CGERGFFY	TKENREEA	CGERGFFYTKENREEA		SCG1	35268/367830	0.944	72042	0.783	0.193404	-0.6333	0	-1	16	-1.4563	-1	3	0	0
HIP_72043	EALYLVCGE	GGGHSRERA	EALYLVCGEGGGHSRERA		SCG1	11809/367830	0.997	72043	0.783	0.006622	-0.4333	0.6667	0	18	-0.4722	-1	0	0	1
HIP_72044	CGERGFF	PEGKGEQEH	CGERGFFPEGKGEQEH		CMGA	13683/247050	0.989	72044	0.783	0.120158	-0.05	0.5	-1	16	-1.5125	-2	2	2	0
HIP_72045	VCGERGFF	EEKKEEEG	VCGERGFFEEKKEEEG		CMGA	20949/247050	0.969	72045	0.783	0.076381	-0.95	0.1667	-3	16	-1.3875	-3	2	0	1
HIP_72046	SHLVEALY	KWAEGGGHS	SHLVEALYKWAEGGGHS		SCG1	40980/367830	0.913	72046	0.783	0.115216	0.2167	0	1	17	-0.4059	-1	2	1	1
HIP_72047	VEALYLVCG	EPMQESKAE	VEALYLVCGEPMQESKAE		CMGA	7437/247050	0.997	72047	0.783	-0.047171	0.5167	0.3333	-3	18	-0.0833	-3	1	0	0
HIP_72048	RGFFYTPK	APGKWQQQG	RGFFYTPKAPGKWQQQG		SCG1	30145/367830	0.965	72048	0.783	-0.040285	-1.0667	0.5	3	17	-1.3353	3	2	1	1
HIP_72049	GERGFFYT	GDLQDTKEN	GERGFFYTGDLQDTKEN		SCG1	16496/367830	0.994	72049	0.783	0.013176	0.1167	0.1667	-2	17	-1.4059	-2	3	1	0
HIP_72050	AEDLQVGQV	EEERGLEPG	AEDLQVGQVEEERGLEPG		SCG1	22300/367830	0.986	72050	0.783	-0.060051	-1.7	0.1667	-5	18	-0.9722	-5	0	0	1
HIP_72051	GERGFFYT	EEYRAPRP	GERGFFYTEEYRAPRP		SCG1	37928/367830	0.930	72051	0.783	0.076751	-1.25	0	0	16	-1.4938	0	4	0	0
HIP_72052	CGSHLVEAL	ENREEARFQ	CGSHLVEALENREEARFQ		SCG1	42583/367830	0.903	72052	0.783	0.134011	-1.5667	0	-1	18	-0.7611	-2	0	0	0
HIP_72053	CGSHLVEAL	NREEARFQD	CGSHLVEALNREEARFQD		SCG1	42584/367830	0.903	72053	0.783	0.134011	-1.5667	0	-1	18	-0.7611	-2	0	0	0
HIP_72054	CCTSICSLY	SEEVKTRH	CCTSICSLYSEEVKTRH		SCG1	29830/367830	0.966	72054	0.783	0.042665	-1.0167	0.3333	1	17	-0.2176	0	1	1	0
HIP_72055	ICSLYQLEN	EVISDTLSK	ICSLYQLENEVISDTLSK		CMGA	16372/247050	0.984	72055	0.783	-0.033752	0.3333	0	-2	18	0.0722	-2	1	0	0
HIP_72056	ELGGGPG	GFFYTPK	ELGGGPGGFFYTPK		INSB	121/4968	0.988	72056	0.783	0.044173	0.4667	0.6667	0	14	-0.3714	0	3	0	0
HIP_72057	CGERGFFYT	VNSMPVDN	CGERGFFYTVNSMPVDN		SCG1	19124/367830	0.991	72057	0.783	-0.030375	0.1167	0.1667	-1	17	-0.3118	-1	3	1	0
HIP_72058	CTSICSLYQ	KELDRNYLN	CTSICSLYQKELDRNYLN		SCG1	47982/367830	0.867	72058	0.783	0.062441	-0.7667	0	0	18	-0.55	0	1	0	0
HIP_72059	LVEALYLVC	SGQLGIQE	LVEALYLVCSGQLGIQE		SCG2	19021/334890	0.996	72059	0.783	0.188407	0.9667	0.3333	-2	17	0.9118	-2	1	1	0
HIP_72060	QPLALEGSL	GEKRDHHST	QPLALEGSLGEKRDHHST		SCG1	27186/367830	0.974	72060	0.783	-0.033901	-0.8667	0.5	1	18	-1.1278	-1	0	0	0
HIP_72061	VEALYLVC	NIAYEDVVG	VEALYLVCNIAYEDVVG		SCG2	22042/334890	0.995	72061	0.783	0.040128	2.2167	0	-3	17	1.0588	-3	2	1	0
HIP_72062	HLCGSHLVE	EEGSANRR	HLCGSHLVEEEGSANRR		CMGA	19014/247050	0.975	72062	0.783	0.185844	-0.4833	0.1667	1	17	-0.9235	-1	0	1	1
HIP_72063	CCTSICSLY	RYYSALRHY	CCTSICSLYRYYSALRHY		NPY	5766/49410	0.908	72063	0.783	0.034273	-0.9	0.1667	3	18	0.05	2	3	0	0
HIP_72064	VNQHLCGSH	GEEENELTL	VNQHLCGSHGEEENELTL		SCG1	19889/367830	0.990	72064	0.783	-0.126586	-1.9667	0.5	-2	18	-0.8389	-4	0	0	1
HIP_72065	AGSLQPLAL	TEVMKCIV	AGSLQPLALTEVMKCIV		CMGA	18651/247050	0.977	72065	0.783	0.153378	1.5667	0	0	17	1.0529	0	0	1	0
HIP_72066	VCGERGFF	NFPMDMSD	VCGERGFFNFPMDMSD		SCG2	19309/334890	0.996	72066	0.783	0.007595	0.4833	0.3333	-2	16	-0.175	-2	3	0	0
HIP_72067	LVEALYLVC	RGGEPRAY	LVEALYLVCRGGEPRAY		SCG1	27616/367830	0.973	72067	0.783	0.220566	0.8667	0.3333	0	17	0.2882	0	1	1	0
HIP_72068	EALYLVCGE	QECFETLRG	EALYLVCGEQECFETLRG		CMGA	14175/247050	0.989	72068	0.783	-0.169491	-0.9833	0.1667	-3	18	0.0222	-3	1	0	0
HIP_72069	VEALYLVC	ERAHQQKKH	VEALYLVCERAHQQKKH		CMGA	29994/247050	0.914	72069	0.783	0.223071	0.7167	0	3	17	-0.7	1	1	1	0
HIP_72070	GAGSLQPLA	LRQQAHKEE	GAGSLQPLALRQQAHKEE		SCG2	30798/334890	0.989	72070	0.783	-0.043591	-0.0333	0.1667	1	18	-0.8611	0	0	0	0
HIP_72071	SHLVEALYL	EGKGEQEH	SHLVEALYLEGKGEQEH		CMGA	18856/247050	0.976	72071	0.783	0.240312	-0.25	0.1667	-1	17	-0.7824	-3	1	1	0
HIP_72072	RGFFYTPKT	NKRLGLSGL	RGFFYTPKTNKRLGLSGL		NPY	6854/49410	0.854	72072	0.783	0.015978	-3.0167	0.1667	4	18	-0.5333	4	1	0	0
HIP_72073	EDLQVGQVE	SKRFPVGPP	EDLQVGQVESKRFPVGPP		SCG2	39090/334890	0.979	72073	0.783	-0.018546	-2	0.1667	-1	18	-0.7278	-1	1	0	0
HIP_72074	RGFFYTPKT	NLQIPPEDL	RGFFYTPKTNLQIPPEDL		SCG2	23506/334890	0.994	72074	0.783	-0.072002	-1.5667	0.1667	0	18	-0.7	0	1	0	0
HIP_72075	GPGAGSLQP	RAYGFRGPG	GPGAGSLQPRAYGFRGPG		CMGA	26551/247050	0.941	72075	0.783	0.077674	-0.8833	0.1667	2	18	-0.6444	2	1	0	0
HIP_72076	TSICSLYQ	EEERLSKEW	TSICSLYQEEERLSKEW		CMGA	20901/247050	0.969	72076	0.783	0.072646	-1.9167	0	-2	17	-0.9765	-2	1	1	1
HIP_72077	EDLQVGQVE	YFMSDTREE	EDLQVGQVEYFMSDTREE		SCG1	12444/367830	0.997	72077	0.783	-0.063043	0.1	0	-5	18	-1.0444	-5	2	0	0
HIP_72078	GGGPGAGSL	DRNYLNYGE	GGGPGAGSLDRNYLNYGE		SCG1	33670/367830	0.951	72078	0.783	0.025003	-1.4833	0.3333	-1	18	-0.9167	-1	1	0	1
HIP_72079	GAGSLQPLA	EGPTVVLNP	GAGSLQPLAEGPTVVLNP		CMGA	6670/247050	0.998	72079	0.783	0.095918	-0.25	0.5	-1	18	0.3	-1	0	0	0
HIP_72080	LCGSHLVEA	VRGYPEEKK	LCGSHLVEAVRGYPEEKK		CMGA	21246/247050	0.967	72080	0.783	0.108491	0.3	0.1667	1	18	-0.5667	0	1	0	0
HIP_72081	HLVEALYL	ESEEGEED	HLVEALYLESEEGEED		SCG1	20921/367830	0.988	72081	0.783	0.150500	-0.25	0.1667	-6	16	-0.8	-7	1	0	0
HIP_72082	HLVEALYL	EESEEGEE	HLVEALYLEESEEGEE		SCG1	20922/367830	0.988	72082	0.783	0.150500	-0.25	0.1667	-6	16	-0.8	-7	1	0	0
HIP_72083	VCGERGFF	ASAKKEEL	VCGERGFFASAKKEEL		SCG1	21051/367830	0.988	72083	0.783	0.000881	1.3333	0.3333	0	17	-0.0118	0	2	1	0
HIP_72084	CGSHLVEAL	LSLIPLIFL	CGSHLVEALLSLIPLIFL		SCG2	21930/334890	0.995	72084	0.783	-0.043061	1.4833	0.1667	0	18	1.8222	-1	0	0	0
HIP_72085	VEQCCTSIC	PEYNYDWWE	VEQCCTSICPEYNYDWWE		SCG1	17896/367830	0.992	72085	0.783	-0.037139	-0.0333	0.3333	-4	18	-0.6833	-4	1	0	0
HIP_72086	HLCGSHLVE	LALQGAKER	HLCGSHLVELALQGAKER		CMGA	19198/247050	0.975	72086	0.783	0.026578	2.3167	0	2	18	-0.0778	0	0	0	0
HIP_72087	IVEQCCTSI	DLFQNRMLS	IVEQCCTSIDLFQNRMLS		SCG2	17433/334890	0.997	72087	0.783	0.019263	1.0167	0.1667	-1	18	0.3444	-1	1	0	0
HIP_72088	CCTSICSLY	VDREKGLSA	CCTSICSLYVDREKGLSA		CMGA	23710/247050	0.957	72088	0.783	-0.053788	-0.35	0.1667	0	18	0.3	0	1	0	0

[illegible]

HIP_72552	SICSLYQLE	EEEEAEAG	SICSLYQLEEEEEAEAG		CMGA	19753/247050	0.973	72552	0.783	-0.065005	-2.2833	0	-6	17	-0.5647	-6	1	1
HIP_72553	HLCGSHLVE	RHYINLITR	HLCGSHLVERHYINLITR		NPY	6951/49410	0.850	72553	0.783	0.043860	-0.75	0	4	18	-0.0944	1	1	0
HIP_72554	CTSICSLY	LFQNRMLSK	CTSICSLYLFQNRMLSK		SCG2	30180/334890	0.990	72554	0.783	-0.004509	0.8	0.1667	2	17	0.3412	2	2	1
HIP_72555	EALYLVCGE	DERILSILR	EALYLVCGEDERILSILR		CMGA	24648/247050	0.952	72555	0.783	-0.105533	-2.15	0.1667	-2	18	0.4	-2	0	0
HIP_72556	GGPGAGSLQ	GREHIAKRA	GGPGAGSLQGREHIAKRA		SCG2	37654/334890	0.981	72556	0.783	0.044986	-1.4833	0.3333	3	18	-0.7667	2	0	0
HIP_72557	CGERGFFY	ASEEEPEY	CGERGFFYASEEEPEY		SCG1	23044/367830	0.984	72557	0.783	0.102365	0.3	0.1667	-4	16	-1.1188	-4	3	0
HIP_72558	GGGPGAGSL	ENENTKFEV	GGGPGAGSLENENTKFEV		SCG1	22408/367830	0.986	72558	0.783	-0.056096	-1.3167	0.3333	-2	18	-0.7722	-2	0	0
HIP_72559	TSICSLYQ	EEEEEATNT	TSICSLYQEEEEEATNT		CMGA	17662/247050	0.980	72559	0.783	0.062174	-1.9167	0	-5	17	-0.9941	-5	1	1
HIP_72560	HLVEALYL	RYRGRGSEE	HLVEALYLRYRGRGSEE		SCG1	45106/367830	0.887	72560	0.783	0.147028	-0.6667	0	1	17	-0.8235	0	2	1
HIP_72561	QCCTSICSL	LPVNSPMNK	QCCTSICSLLPVNSPMNK		CMGA	15710/247050	0.985	72561	0.783	-0.010788	1.9833	0.3333	1	18	0.2778	1	0	0
HIP_72562	RGFFYTPKT	LQVRGYPEE	RGFFYTPKTLQVRGYPEE		CMGA	16734/247050	0.983	72562	0.783	0.086717	-0.2833	0.1667	1	18	-0.9889	1	1	0
HIP_72563	ELGGGPGA	TPIESHQ	ELGGGPGATPIESHQ		IAPP	700/45018	0.998	72563	0.783	-0.086134	0.3333	0.5	-1	15	-0.66	-2	0	1
HIP_72564	RGFFYTPKT	KRLVNAAGS	RGFFYTPKTKRLVNAAGS		SCG2	41468/334890	0.974	72564	0.783	0.040336	-1.8	0.1667	4	18	-0.5	4	1	0
HIP_72565	GIVEQCCTS	NTKFEVRLL	GIVEQCCTSNTKFEVRLL		SCG1	41859/367830	0.907	72565	0.783	0.027869	-1.1833	0.1667	0	18	0.1833	0	1	0
HIP_72566	GERGFFYTP	EDILNLLGM	GERGFFYTPEDILNLLGM		SCG2	21195/334890	0.996	72566	0.783	-0.167891	-1.0167	0.1667	-2	18	0.0056	-2	3	0
HIP_72567	QCCTSICSL	PKNDDTPNR	QCCTSICSLPKNDDTPNR		SCG2	32922/334890	0.987	72567	0.783	0.171704	-0.5833	0.3333	0	18	-0.9056	0	0	0
HIP_72568	HLVEALYL	GAEEAQDPE	HLVEALYLGAEEAQDPE		CMGA	8409/247050	0.996	72568	0.783	0.099313	0.7	0.1667	-6	17	-0.3824	-5	1	1
HIP_72569	GPGAGSLQP	KSSQESGEE	GPGAGSLQPKSSQESGEE		SCG1	23344/367830	0.984	72569	0.783	-0.011538	-1.1333	0.5	-2	18	-1.3222	-2	0	0
HIP_72570	QPLALEGSL	GSSQETDKL	QPLALEGSLGSSQETDKL		SCG2	5175/334890	1.000	72570	0.783	-0.065296	0.1	0.8333	-2	18	-0.55	-2	0	0
HIP_72571	RGFFYTPKT	PGPQAEGDS	RGFFYTPKTPGPQAEGDS		CMGA	14755/247050	0.987	72571	0.783	-0.074398	-1.6333	0.6667	0	18	-1.1667	0	1	0
HIP_72572	LVEALYLV	YGAGRSRS	LVEALYLVYGAGRSRS		SCG2	24104/334890	0.994	72572	0.783	0.117866	1.1333	0.1667	1	16	0.3687	1	2	0
HIP_72573	CCTSICSLY	KGHPQEESE	CCTSICSLYKGHPQEESE		SCG1	30005/367830	0.965	72573	0.783	-0.005881	-0.9667	0.3333	-1	18	-0.65	-2	1	0
HIP_72574	SICSLYQL	MQFPPMYEE	SICSLYQLMQFPPMYEE		SCG2	19684/334890	0.996	72574	0.783	-0.121085	0.0333	0	-2	17	-0.0118	-2	2	1
HIP_72575	SHLVEALYL	SKVIAYLK	SHLVEALYLSKVIAYLK		SCG2	33200/334890	0.987	72575	0.783	0.217004	0.9667	0.1667	2	17	0.7647	1	1	1
HIP_72576	GSLQPLAL	EISRNLQIP	GSLQPLALEISRNLQIP		SCG2	22812/334890	0.995	72576	0.783	0.145971	1.6	0.1667	0	17	0.1353	0	0	1
HIP_72577	RGFFYTPKT	TLSKPSPMP	RGFFYTPKTTLSKPSPMP		CMGA	24512/247050	0.953	72577	0.783	0.018754	-0.65	0.3333	3	18	-0.7111	3	1	0
HIP_72578	SLYQLENYC	GEDWNPVEE	SLYQLENYCGEDWNPVEE		SCG2	15995/334890	0.997	72578	0.783	0.066087	-1.6167	0.1667	-5	18	-1.1111	-5	2	0
HIP_72579	SLYQLENYC	EDNRDSSM	SLYQLENYCEDNRDSSM		CMGA	19354/247050	0.974	72579	0.783	0.069553	-2.1333	0	-3	17	-1.2941	-3	1	1
HIP_72580	SHLVEALYL	DKMAHGYG	SHLVEALYLDKMAHGYG		SCG1	40809/367830	0.914	72580	0.783	0.220867	0.1333	0	1	17	-0.0235	-1	1	1
HIP_72581	LCGSHLVEA	APRPQSEES	LCGSHLVEAAPRPQSEES		SCG1	35727/367830	0.941	72581	0.783	0.064029	-0.3	0.1667	-1	18	-0.5444	-2	0	0
HIP_72582	HLCGSHLVE	LPLQVRGYP	HLCGSHLVELPLQVRGYP		CMGA	18605/247050	0.977	72582	0.783	-0.018040	1.75	0.1667	2	18	0.1167	0	0	0
HIP_72583	LCGSHLVEA	AGSGRLQNG	LCGSHLVEAAGSGRLQNG		SCG2	20075/334890	0.996	72583	0.783	0.130178	0.5167	0.3333	1	18	0.0167	0	0	0
HIP_72584	CGERGFFYT	LSAEPGWQ	CGERGFFYTLSAEPGWQ		CMGA	15858/247050	0.985	72584	0.783	-0.088609	0.9333	0.1667	-1	17	-0.4588	-1	3	1
HIP_72585	CTSICSLYQ	EKRKCNTAT	CTSICSLYQEKRKCNTAT		IAPP	6642/45018	0.851	72585	0.783	0.203084	-2.15	0	2	18	-0.5667	2	1	0
HIP_72586	FVNQHLCG	WWEKKPFSE	FVNQHLCGWWEKKPFSE		SCG1	32624/367830	0.955	72586	0.783	0.044999	0.1	0.1667	1	17	-0.7941	0	2	1
HIP_72587	LYQLENYC	EEEEMAVVP	LYQLENYCEEEEMAVVP		CMGA	17025/247050	0.982	72587	0.783	0.028248	-2.1333	0	-5	17	-0.3824	-5	1	1
HIP_72588	SLYQLENYC	DASEAHESS	SLYQLENYCDASEAHESS		SCG1	17750/367830	0.992	72588	0.783	-0.088764	-0.8	0.1667	-3	18	-0.9056	-4	1	0
HIP_72589	GGGPGAGSL	PEYNYDWWE	GGGPGAGSLPEYNYDWWE		SCG1	14544/367830	0.995	72589	0.783	-0.078650	-0.6333	0.5	-3	18	-1.0444	-3	1	0
HIP_72590	NQHLCGSHL	EVRDSKENI	NQHLCGSHLEVRDSKENI		SCG2	45321/334890	0.962	72590	0.783	0.029072	-0.6667	0.1667	1	18	-1.0556	-1	0	0
HIP_72591	YQLENYCN	YNPYQGVSV	YQLENYCNYNPYQGVSV		SCG2	26509/334890	0.993	72591	0.783	0.036763	-1.45	0.1667	-1	17	-0.8412	-1	3	1
HIP_72592	PLALEGSLQ	PVSKRFPVG	PLALEGSLQPVSKRFPVG		SCG2	21085/334890	0.996	72592	0.783	-0.073569	0.2167	0.5	1	18	0.1	1	0	0
HIP_72593	VNQHLCGSH	PGEEEEEEE	VNQHLCGSHPGEEEEEEE		CMGA	8205/247050	0.996	72593	0.783	-0.124548	-1.65	0.6667	-5	18	-1.7	-7	0	0
HIP_72594	PGAGSLQPL	GHSQEKTHS	PGAGSLQPLGHSQEKTHS		SCG1	26428/367830	0.976	72594	0.783	-0.028283	-0.95	0.5	2	18	-1.05	0	0	0
HIP_72595	AGSLQPLAL	APPITPEC	AGSLQPLALAPPITPEC		SCG1	17153/367830	0.993	72595	0.783	0.143693	1.3333	0.3333	-1	17	0.5	-1	0	1
HIP_72596	FVNQHLCGS	PEDLEWERY	FVNQHLCGSPEDLEWERY		SCG1	17327/367830	0.993	72596	0.783	-0.119273	-1.2167	0.5	-2	18	-0.9222	-3	0	0
HIP_72597	GERGFFYT	NAAGSGRL	GERGFFYTNAAGSGRL		SCG2	32156/334890	0.988	72597	0.783	0.023984	0.15	0	1	16	-0.4625	1	3	0
HIP_72598	SICSLYQL	EEEEEEEA	SICSLYQLEEEEEEEA		CMGA	14520/247050	0.988	72598	0.783	-0.008913	-1.9167	0	-8	17	-1.0588	-8	1	1
HIP_72599	SICSLYQL	EEEEEEEA	SICSLYQLEEEEEEEA		CMGA	14521/247050	0.988	72599	0.783	-0.008913	-1.9167	0	-8	17	-1.0588	-8	1	1
HIP_72600	PLALEGSLQ	FPVGPPKND	PLALEGSLQFPVGPPKND		SCG2	9620/334890	0.999	72600	0.783	-0.097231	0.8167	0.3333	-1	18	-0.3167	-1	1	0
HIP_72601	IVEQCCTSI	GENYQKGE	IVEQCCTSIGENYQKGE		SCG1	19384/367830	0.990	72601	0.783	0.034845	-0.7333	0.3333	-2	17	-0.6059	-2	1	1
HIP_72602	HLCGSHLV	SEEYRAPR	HLCGSHLVSEEYRAPR		SCG1	30602/367830	0.963	72602	0.783	0.208929	-0.5	0.1667	2	16	-0.7	0	1	0
HIP_72603	YLVCGERGF	EEAQDPEGK	YLVCGERGFEEAQDPEGK		CMGA	16737/247050	0.983	72603	0.783	-0.049712	-1.2167	0.1667	-3	18	-1.0222	-3	1	0
HIP_72604	VNQHLCGSH	TQEEVRDSK	VNQHLCGSHTQEEVRDSK		SCG2	31165/334890	0.989	72604	0.783	-0.104978	-2.0167	0.3333	1	18	-1.3222	-1	0	0
HIP_72605	DLQVGQVEL	KLKHMQFPP	DLQVGQVELKLKHMQFPP		SCG2	45856/334890	0.960	72605	0.783	0.098170	0.0833	0	1	18	-0.4222	0	0	0
HIP_72606	AEDLQVGQV	PQGLFRGGK	AEDLQVGQVPQGLFRGGK		CMGA	9715/247050	0.995	72606	0.783	-0.038318	-0.8667	0.5	0	18	-0.4722	0	0	0
HIP_72607	GGPGAGSLQ	GSQENHPQE	GGPGAGSLQGSQENHPQE		SCG1	13179/367830	0.996	72607	0.783	-0.051243	-0.8667	0.5	-1	18	-1.4111	-2	0	0
HIP_72608	QVGQVELGG	LIVLSVALN	QVGQVELGGLIVLSVALN		IAPP	1490/45018	0.994	72608	0.783	0.108761	2.5833	0.3333	-1	18	1.2389	-1	0	0
HIP_72609	HLVEALYL	GALAEAY	HLVEALYLGALAEAY		NPY	2132/49410	0.986	72609	0.783	0.061219	1.9167	0.1667	-1	15	0.8933	-2	1	1

HIP_73063	HLCGSHLVE	ERDSLSEED	HLCGSHLVEERDSLSEED		SCG2	31121/334890	0.989	73063	0.782	0.110535	-1.1667	0	-3	18	-0.9222	-5	0	0
HIP_73064	LCGSHLVEA	GDSEGLSQG	LCGSHLVEAGDSEGLSQG		CMGA	13515/247050	0.990	73064	0.782	0.194403	-0.3667	0.3333	-2	18	-0.0722	-3	0	0
HIP_73065	DLQVGQVEL	QSEESWDEE	DLQVGQVELQSEESWDEE		SCG1	21498/367830	0.987	73065	0.782	0.087696	-0.55	0.1667	-7	18	-1.2167	-7	0	0
HIP_73066	LGGGPGAGS	PEPMQESKA	LGGGPGAGSPEPMQESKA		CMGA	9660/247050	0.995	73066	0.782	-0.093051	-1.0167	0.6667	-1	18	-0.75	-1	0	0
HIP_73067	EDLQVGQVE	EKSSQESGE	EDLQVGQVEEKSSQESGE		SCG1	31970/367830	0.958	73067	0.782	-0.092688	-1.8333	0.1667	-5	18	-1.4667	-5	0	0
HIP_73068	CSLYQLENY	GQPRSQEES	CSLYQLENYGQPRSQEES		SCG1	19868/367830	0.990	73068	0.782	-0.031284	-2.3	0.3333	-2	18	-1.4389	-2	1	0
HIP_73069	QCCTSICSL	KNDDTPNRQ	QCCTSICSLKNDDTPNRQ		SCG2	39906/334890	0.977	73069	0.782	0.214602	-0.9	0.1667	0	18	-1.0111	0	0	0
HIP_73070	DLQVGQVEL	LQEEEQIEQ	DLQVGQVELLQEEEQIEQ		SCG2	20693/334890	0.996	73070	0.782	-0.047503	0.2167	0	-6	18	-0.8111	-6	0	0
HIP_73071	SHLVEALYL	KNDDTPNR	SHLVEALYLKNDDTPNR		SCG2	34363/334890	0.986	73071	0.782	0.255111	-0.7667	0	0	17	-0.9471	-1	1	1
HIP_73072	ALYLVCGER	YFPNPYNQE	ALYLVCGERYFPNPYNQE		SCG2	14942/334890	0.998	73072	0.782	-0.006021	-1.4167	0.3333	-1	18	-0.5889	-1	2	0
HIP_73073	LGGGPGAGS	RGRGGEPRA	LGGGPGAGSRGRGGEPRA		SCG1	41528/367830	0.909	73073	0.782	0.045862	-1.4667	0.5	2	18	-0.9333	2	0	0
HIP_73074	PGAGSLQPL	ENRQMAYEN	PGAGSLQPLENRQMAYEN		SCG2	36286/334890	0.983	73074	0.782	0.009541	-2.1333	0.1667	-1	18	-1.0278	-1	0	0
HIP_73075	GSHLVEALY	PVSTHQEAE	GSHLVEALYPVSTHQEAE		SCG1	12138/367830	0.997	73075	0.782	0.089788	1.0167	0.3333	-1	18	-0.3556	-3	1	0
HIP_73076	GAGSLQPLA	KVAHQLQAL	GAGSLQPLAKVAHQLQAL		CMGA	20699/247050	0.970	73076	0.782	-0.008866	1.0167	0.1667	2	18	0.3222	1	0	0
HIP_73077	EAEDLQVGQ	EGNNQAPGE	EAEDLQVGQEGNNQAPGE		CMGA	6810/247050	0.997	73077	0.782	-0.126721	-1.1833	0.3333	-5	18	-1.4556	-5	0	0
HIP_73078	PLALEGSLQ	NQEKVLPRL	PLALEGSLQNQEKVLPRL		SCG2	34647/334890	0.985	73078	0.782	-0.028505	-1.8333	0.1667	0	18	-0.2944	0	0	0
HIP_73079	HLVEALYL	EETGSQEN	HLVEALYLEETGSQEN		SCG1	25752/367830	0.978	73079	0.782	0.169067	-0.2333	0	-3	16	-0.625	-4	1	0
HIP_73080	CTSICSLYQ	LSNALSKSS	CTSICSLYQLSNALSKSS		SCG1	27860/367830	0.972	73080	0.782	-0.032833	-0.25	0.1667	1	18	0.2778	1	1	0
HIP_73081	HLCGSHLVE	LDQLLHYRK	HLCGSHLVELDQLLHYRK		SCG1	40048/367830	0.918	73081	0.782	0.016389	0.2167	0	3	18	-0.2944	0	0	0
HIP_73082	GSLQPLALE	GGGPGAGSL	GSLQPLALEGGGPGAGSL		INSC	219/5184	0.997	73082	0.782	0.164285	0.15	0.5	-1	18	0.2556	-1	0	0
HIP_73083	LVEALYLVC	LDHPDLFQN	LVEALYLVCLDHPDLFQN		SCG2	23722/334890	0.994	73083	0.782	0.023312	1.2667	0	-2	18	0.6056	-3	1	0
HIP_73084	LVCGERGFF	YPGPQAEG	LVCGERGFFYPGPQAEG		CMGA	15561/247050	0.986	73084	0.782	0.055152	0.3167	0.5	-1	17	-0.1882	-1	3	1
HIP_73085	GSLQPLAL	ENHPQESKG	GSLQPLALENHPQESKG		SCG1	31624/367830	0.959	73085	0.782	0.154286	-0.1333	0	0	17	-1	-1	0	1
HIP_73086	QVELGGGPG	LALSLLVCL	QVELGGGPGLALSLLVCL		NPY	1371/49410	0.992	73086	0.782	0.158697	1.1667	0.5	-1	18	1.3611	-1	0	0
HIP_73087	GERGFFYT	MDQLAKELT	GERGFFYTMDQLAKELT		CMGA	23366/247050	0.959	73087	0.782	0.042402	-0.7167	0	-1	17	-0.5294	-1	3	1
HIP_73088	RGFFYTPKT	PSSKDVMEK	RGFFYTPKTPSSKDVMEK		CMGA	22082/247050	0.964	73088	0.782	-0.038964	-1.5667	0.6667	2	18	-1.0778	2	1	0
HIP_73089	ERGFFYT	EEEEEAT	ERGFFYTEEEEEAT		CMGA	18873/247050	0.976	73089	0.782	0.071673	-1.6167	0	-5	14	-1.5143	-5	3	0
HIP_73090	HLVEALYLV	GELEQEEE	HLVEALYLVGELEQEEE		CMGA	6688/247050	0.998	73090	0.782	0.095853	1.1	0.1667	-5	17	-0.2353	-6	1	1
HIP_73091	TSICSLYQ	LLMRESTEN	TSICSLYQLLMRESTEN		NPY	3207/49410	0.973	73091	0.782	-0.017068	1.4167	0	-1	17	-0.1941	-1	1	1
HIP_73092	HLVEALYL	GPTVVLNP	HLVEALYLGPTVVLNP		CMGA	13186/247050	0.990	73092	0.782	0.212601	0.6	0.3333	0	16	0.8625	-1	1	0
HIP_73093	CGERGFFYT	KENKPYAL	CGERGFFYTKENKPYAL		SCG2	39002/334890	0.979	73093	0.782	0.163462	-1.6833	0	1	17	-0.8706	1	3	1
HIP_73094	ICSLYQLEN	AQDPEGKGE	ICSLYQLENAQDPEGKGE		CMGA	22369/247050	0.963	73094	0.782	-0.105620	-1.4	0	-3	18	-0.9167	-3	1	0
HIP_73095	LGGGPGAG	VCGERGFFY	LGGGPGAGVCGERGFFY		INSB	57/4968	0.997	73095	0.782	0.023277	1.2167	0.5	0	17	0.2471	0	0	1
HIP_73096	SLQPLAL	GARPQAL	SLQPLALGARPQAL		CMGA	21603/247050	0.966	73096	0.782	0.225331	1.05	0.1667	1	14	0.3357	1	0	0
HIP_73097	EQCCTSICS	DNRNHNEGM	EQCCTSICSDNRNHNEGM		SCG1	35808/367830	0.941	73097	0.782	0.147653	-0.8833	0.1667	-1	18	-1.1667	-2	0	0
HIP_73098	VCGERGFF	LGNKRLGL	VCGERGFFLGNKRLGL		NPY	3236/49410	0.972	73098	0.782	0.052268	0.85	0.3333	2	16	0.1375	2	2	0
HIP_73099	RGFFYTPKT	PVSKRFPVG	RGFFYTPKTPVSKRFPVG		SCG2	32916/334890	0.987	73099	0.782	0.002159	-0.7333	0.5	4	18	-0.5056	4	1	0
HIP_73100	GERGFFY	MRESTENVP	GERGFFYMRESTENVP		NPY	5253/49410	0.925	73100	0.782	0.152609	-0.3	0	-1	16	-1.0313	-1	3	2
HIP_73101	ICSLYQLEN	HSTHYRASE	ICSLYQLENHSTHYRASE		SCG1	44032/367830	0.894	73101	0.782	-0.020205	-1.3167	0.1667	1	18	-0.7889	-1	1	0
HIP_73102	VCGERGFF	APEDLEWER	VCGERGFFAPEDLEWER		SCG1	18370/367830	0.992	73102	0.782	-0.024870	0.3167	0.3333	-3	17	-0.7	-3	2	1
HIP_73103	FVNQHLCGS	TPGRAGTEA	FVNQHLCGSTPGRAGTEA		SCG2	16633/334890	0.997	73103	0.782	-0.035724	-0.2333	0.6667	1	18	-0.35	0	0	0
HIP_73104	QVGQVELGG	KGRHHRGRG	QVGQVELGGKGRHHRGRG		SCG1	42064/367830	0.906	73104	0.782	0.022608	-0.9667	0.5	5	18	-1.3611	3	0	0
HIP_73105	GSLQPLAL	EESSPDYN	GSLQPLALEESSPDYN		SCG2	20966/334890	0.996	73105	0.782	0.164168	0.2667	0.1667	-3	16	-0.725	-3	0	0
HIP_73106	VGQVELGGG	EEEEEEAEA	VGQVELGGGEEEEEEAEA		CMGA	8319/247050	0.996	73106	0.782	-0.072708	-1.95	0.5	-8	18	-0.9611	-8	0	0
HIP_73107	QHLCGSHLV	WKELDRNY	QHLCGSHLVWKELDRNY		SCG1	42699/367830	0.903	73107	0.782	0.178203	-0.5833	0	2	17	-0.8294	0	1	1
HIP_73108	CGERGFFY	WGYEKRNL	CGERGFFYWGYEKRNL		SCG1	32364/367830	0.956	73108	0.782	0.099160	0.2833	0.1667	1	16	-1.0125	1	5	0
HIP_73109	GGGPGAGSL	NQMDKARRH	GGGPGAGSLNQMDKARRH		SCG1	32389/367830	0.956	73109	0.782	0.027279	-0.4167	0.3333	3	18	-1.2056	2	0	0
HIP_73110	GSHLVEALY	SGELEQEE	GSHLVEALYSGELEQEE		CMGA	10463/247050	0.994	73110	0.782	0.140040	-0.0667	0.3333	-4	17	-0.6176	-5	1	1
HIP_73111	SHLVEALYL	SRDNPFKR	SHLVEALYLSRDNPFKR		SCG2	34715/334890	0.985	73111	0.782	0.274877	-0.4167	0.1667	2	17	-0.6412	1	1	1
HIP_73112	RGFFYTPKT	DQLSDDVSK	RGFFYTPKTDQLSDDVSK		SCG2	31333/334890	0.989	73112	0.782	0.014513	-1.5667	0.1667	0	18	-1.0556	0	1	0
HIP_73113	EQCCTSICS	PQSLATLES	EQCCTSICSPQSLATLES		SCG2	12504/334890	0.998	73113	0.782	0.096747	0.05	0.5	-2	18	0.0667	-2	0	0
HIP_73114	LVCGERGFF	EVRLLRDP	LVCGERGFFEVRLLRDP		SCG1	34679/367830	0.946	73114	0.782	0.041683	0.2333	0.1667	0	17	0.0882	0	2	1
HIP_73115	LYQLENYCN	QLDQLLHYR	LYQLENYCNQLDQLLHYR		SCG1	38332/367830	0.928	73115	0.782	-0.012593	-0.9167	0	0	18	-0.8111	-1	1	0
HIP_73116	CTSICSLYQ	SVFQELGK	CTSICSLYQSVFQELGK		SCG2	24009/334890	0.994	73116	0.782	-0.072656	0.8667	0.1667	0	17	0.2882	0	2	1
HIP_73117	RGFFYTPKT	NFGAILSST	RGFFYTPKTNFGAILSST		IAPP	3314/45018	0.972	73117	0.782	-0.063959	-1.2167	0.3333	2	18	-0.0444	2	2	0
HIP_73118	VEQCCTSIC	LGYKEIRKG	VEQCCTSICLGYKEIRKG		CMGA	16032/247050	0.984	73118	0.782	-0.026631	1.3833	0.3333	1	18	-0.1056	1	1	0
HIP_73119	CSLYQLENY	VLNPHPSLG	CSLYQLENYVLNPHPSLG		CMGA	14395/247050	0.988	73119	0.782	-0.075608	-0.6333	0	0	18	-0.1722	-1	1	0
HIP_73120	LVEALYLVC	GAKERAHQ	LVEALYLVCGAKERAHQ		CMGA	20966/247050	0.969	73120	0.782	0.198657	1.3333	0.1667	1	17	0.2294	0	1	1

HIP_73574	GSLQPLAL	GPQLRRGW	GSLQPLALGPQLRRGW		CMGA	16931/247050	0.982	73574	0.782	0.226083	0.65	0.3333	2	16	-0.3188	2	0	0	0
HIP_73575	LVEALYLV	YALNSEKN	LVEALYLVYALNSEKN		SCG2	24538/334890	0.994	73575	0.782	0.003577	1.8333	0	-1	16	0.3687	-1	2	0	0
HIP_73576	SICSLYQLE	HPQESKGQP	SICSLYQLEHPQESKGQP		SCG1	40804/367830	0.914	73576	0.782	-0.088819	-1.9167	0.1667	0	18	-0.9611	-1	1	0	0
HIP_73577	QCCTSICSL	KSGELEQEE	QCCTSICSLKSGELEQEE		CMGA	13846/247050	0.989	73577	0.782	0.160454	0.0667	0.5	-3	18	-0.4889	-3	0	0	0
HIP_73578	GGGPGAGSL	QECFETLRG	GGGPGAGSLQECFETLRG		CMGA	12161/247050	0.992	73578	0.782	-0.056796	-0.3167	0.3333	-1	18	-0.3222	-1	1	0	1
HIP_73579	ICSLYQLEN	NERKQASAI	ICSLYQLENNERKQASAI		SCG1	52167/367830	0.833	73579	0.782	0.105098	-2.45	0	0	18	-0.5333	0	1	0	0
HIP_73580	DLQVGQVEL	LFRGGKSGE	DLQVGQVELLFRGGKSGE		CMGA	24356/247050	0.954	73580	0.782	0.005963	1.1	0	-1	18	-0.3167	-1	1	0	0
HIP_73581	VELGGGPGA	ESTENVPRT	VELGGGPGAESTENVPRT		NPY	1792/49410	0.989	73581	0.782	-0.101203	-0.8667	0.5	-2	18	-0.6389	-2	0	0	1
HIP_73582	RGFFYTPKT	DVSKVIAYL	RGFFYTPKTDVSKVIAYL		SCG2	34957/334890	0.985	73582	0.782	0.022953	-1.05	0.3333	2	18	0.0833	2	1	0	0
HIP_73583	QHLCGSHLV	KIESQTQEE	QHLCGSHLVKIESQTQEE		SCG2	32653/334890	0.988	73583	0.782	0.168332	0.3167	0	0	18	-0.8444	-2	0	0	0
HIP_73584	ELGGGPGAG	EDELSEVLE	ELGGGPGAGEDELSEVLE		CMGA	11048/247050	0.993	73584	0.782	-0.124703	-1.5833	0.3333	-6	18	-0.4444	-6	0	0	1
HIP_73585	AGSLQPLAL	YKEIRKGE	AGSLQPLALYKEIRKGE		CMGA	28385/247050	0.928	73585	0.782	0.196512	0.1167	0	1	17	-0.4588	1	1	1	0
HIP_73586	GSHLVEALY	DIYKANNI	GSHLVEALYDIYKANNI		SCG2	29261/334890	0.991	73586	0.782	0.038049	0.6667	0	0	17	-0.0294	-1	2	1	0
HIP_73587	PLALEGSLQ	SAANQKTSY	PLALEGSLQSAANQKTSY		SCG2	20234/334890	0.996	73587	0.782	-0.017379	0.3833	0.3333	0	18	-0.4167	0	0	0	0
HIP_73588	VELGGGPGA	NRQYWDEDL	VELGGGPGANRQYWDEDL		SCG2	25246/334890	0.994	73588	0.782	-0.035121	-1.95	0.3333	-3	18	-0.9611	-3	1	0	1
HIP_73589	IVEQCCTSI	RLFEKPLDS	IVEQCCTSIRLFEKPLDS		SCG2	20586/334890	0.996	73589	0.782	0.024936	0.85	0.1667	-1	18	0.1278	-1	1	0	0
HIP_73590	GSLQPLAL	EEGPTVVL	GSLQPLALEEGPTVVL		CMGA	14485/247050	0.988	73590	0.782	0.180334	0.3333	0.1667	-2	16	0.5875	-2	0	0	0
HIP_73591	LGGGPGAGS	EESWDEEDK	LGGGPGAGSEESWDEEDK		SCG1	18094/367830	0.992	73591	0.782	-0.076731	-1.2	0.5	-5	18	-1.4111	-5	1	0	1
HIP_73592	LVCGERGFF	KAAWIPHV	LVCGERGFFKAAWIPHV		SCG2	32247/334890	0.988	73592	0.782	0.063375	0.8167	0.1667	2	17	0.5882	1	3	1	0
HIP_73593	LCGSHLVEA	QGDLQDTKE	LCGSHLVEAQGDLQDTKE		SCG1	21544/367830	0.987	73593	0.782	0.166620	-0.8167	0.1667	-2	18	-0.5833	-3	0	0	0
HIP_73594	LYLVCGERG	YETQQWPER	LYLVCGERGYETQQWPER		SCG2	27703/334890	0.992	73594	0.782	-0.017360	-2.3167	0.1667	-1	18	-1.0444	-1	1	0	0
HIP_73595	TSICSLYQL	PMPVSQECF	TSICSLYQLPMPVSQECF		CMGA	7892/247050	0.997	73595	0.782	-0.025157	-0.3833	0.3333	-1	18	0.4389	-1	1	0	0
HIP_73596	GGPGAGSLQ	PSSKDVMEK	GGPGAGSLQPSSKDVMEK		CMGA	12210/247050	0.992	73596	0.782	-0.026346	-0.6167	0.6667	0	18	-0.7667	0	0	0	0
HIP_73597	EQCCTSICS	SEEYRAPR	EQCCTSICSSEEYRAPR		SCG1	28663/367830	0.970	73597	0.782	0.112174	-0.2667	0.3333	-1	17	-0.8941	-1	1	1	0
HIP_73598	LYQLENYCN	SEADLDHPD	LYQLENYCNSEADLDHPD		SCG2	16293/334890	0.997	73598	0.782	-0.093015	-0.8	0.1667	-4	18	-1.1389	-5	1	0	0
HIP_73599	HLVEALYLV	LSEVLENQS	HLVEALYLVLSEVLENQS		CMGA	12612/247050	0.991	73599	0.782	0.068863	1.0333	0.1667	-2	18	0.5444	-3	1	0	0
HIP_73600	VEQCCTSIC	QNAFLNERK	VEQCCTSICQNAFLNERK		SCG1	31714/367830	0.959	73600	0.782	0.008642	0.1667	0.1667	0	18	-0.35	0	1	0	0
HIP_73601	LGGGPGAGS	EKNFFPEYN	LGGGPGAGSEKNFFPEYN		SCG1	33158/367830	0.953	73601	0.782	-0.031260	-1.7167	0.3333	-1	18	-0.7778	-1	1	0	1
HIP_73602	GERGFFYT	KGRHHRGRG	GERGFFYTKGRHHRGRG		SCG1	44801/367830	0.889	73602	0.782	0.182524	-1.3333	0.1667	6	17	-1.7765	4	3	1	0
HIP_73603	GGGPGAGSL	GGEPRAYF	GGGPGAGSLGGEPRAYF		SCG1	14432/367830	0.995	73603	0.782	0.018299	-0.2833	0.6667	0	17	-0.3471	0	0	1	1
HIP_73604	ERGFFYTPK	RIILEALRQ	ERGFFYTPKRIILEALRQ		SCG2	23236/334890	0.995	73604	0.782	0.037459	-0.2833	0.1667	2	18	-0.4389	2	2	0	0
HIP_73605	CSLYQLENY	PKLDLKRQY	CSLYQLENYPKLDLKRQY		SCG1	34553/367830	0.947	73605	0.782	-0.055482	-1.6667	0.1667	1	18	-1.0222	1	1	0	0
HIP_73606	EQCCTSICS	KGREHIAKR	EQCCTSICSKGREHIAKR		SCG2	39105/334890	0.979	73606	0.782	0.169448	-0.4333	0.3333	3	18	-0.8278	2	0	0	0
HIP_73607	SICSLYQLE	LCAGQVTAL	SICSLYQLELCAGQVTAL		CMGA	11884/247050	0.992	73607	0.782	0.003983	0.8167	0	-1	18	1	-1	1	0	0
HIP_73608	VCGERGFF	LRDPADASE	VCGERGFFLRDPADASE		SCG1	30712/367830	0.963	73608	0.782	0.025742	0.1667	0.1667	-2	17	-0.3824	-2	2	1	0
HIP_73609	LYQLENYCN	QREDEEEEE	LYQLENYCNQREDEEEEE		SCG1	25963/367830	0.978	73609	0.782	0.030498	-2.3	0	-7	18	-2.1667	-7	1	0	2
HIP_73610	LCGSHLVEA	GSQENHPQE	LCGSHLVEAGSQENHPQE		SCG1	22580/367830	0.985	73610	0.782	0.192454	-0.3667	0.3333	-1	18	-0.85	-3	0	0	0
HIP_73611	GSLQPLALE	ERGEDSSEE	GSLQPLALEERGEDSSEE		SCG1	25157/367830	0.980	73611	0.782	0.115505	-1.05	0.1667	-5	18	-1.1444	-5	0	0	0
HIP_73612	GSHLVEALY	YWDEDLLM	GSHLVEALYYWDEDLLM		SCG2	27532/334890	0.992	73612	0.782	0.028531	-0.2333	0	-3	17	0.0706	-4	3	1	0
HIP_73613	AGSLQPLAL	QLRRGWRP	AGSLQPLALQLRRGWRP		CMGA	32144/247050	0.894	73613	0.782	0.217795	0.8667	0	3	17	-0.4353	3	0	1	0
HIP_73614	VEALYLVCG	EEEEEEEE	VEALYLVCGEEEEEEEE		CMGA	12788/247050	0.991	73614	0.782	-0.011268	-0.7	0.1667	-9	17	-0.7588	-9	1	1	6
HIP_73615	VEALYLVCG	EEEEEEEE	VEALYLVCGEEEEEEEE		CMGA	12789/247050	0.991	73615	0.782	-0.011268	-0.7	0.1667	-9	17	-0.7588	-9	1	1	6
HIP_73616	VEALYLVCG	EEEEEEEE	VEALYLVCGEEEEEEEE		CMGA	12790/247050	0.991	73616	0.782	-0.011268	-0.7	0.1667	-9	17	-0.7588	-9	1	1	6
HIP_73617	GSLQPLAL	LQKEPDLRL	GSLQPLALLQKEPDLRL		SCG2	35815/334890	0.984	73617	0.782	0.186288	0.9667	0	0	17	-0.1294	0	0	1	0
HIP_73618	GIVEQCCT	QEEEEDNRD	GIVEQCCTQEEEEDNRD		CMGA	15469/247050	0.986	73618	0.782	0.045620	-1.0333	0	-6	17	-1.5824	-6	0	1	2
HIP_73619	LGGGPGAGS	AEAKTHWLG	LGGGPGAGSAEAKTHWLG		SCG2	15604/334890	0.998	73619	0.782	0.082593	0.1167	0.3333	1	18	-0.2222	0	0	0	1
HIP_73620	SLQPLAL	ETTENEN	SLQPLALETTENEN		SCG1	23460/367830	0.984	73620	0.782	0.190527	0.75	0	-3	14	-0.8286	-3	0	0	0
HIP_73621	QHLCGSHLV	PGAEEAQDP	QHLCGSHLVPGAEEAQDP		CMGA	8230/247050	0.996	73621	0.782	0.151400	0.7667	0.3333	-1	18	-0.6	-3	0	0	0

[illegible]

HIP_74085	GERGFFYTP	VMKDVEVI	GERGFFYTPVMKDVEVI		CMGA	13341/247050	0.990	74085	0.781	-0.093236	-0.2333	0.1667	0	18	0.6556	0	3	0	0
HIP_74086	HLVEALYL	SVFQELGKL	HLVEALYLSVFQELGKL		SCG2	21902/334890	0.995	74086	0.781	0.014634	2.0833	0.1667	0	17	0.7	-1	2	1	0
HIP_74087	DLQVGQVEL	SLYQLENYC	DLQVGQVELSLYQLENYC		INSA	219/3024	0.890	74087	0.781	-0.025140	1.0333	0.1667	-3	18	-0.1222	-3	1	0	0
HIP_74088	GPGAGSLQP	PSPMPVSQE	GPGAGSLQPPSPMPVSQE		CMGA	10542/247050	0.994	74088	0.781	-0.028545	-0.8833	0.6667	-1	18	-0.5778	-1	0	0	0
HIP_74089	VCGERGFF	EVRDSKENI	VCGERGFFEVRDSKENI		SCG2	30134/334890	0.990	74089	0.781	0.023634	0.2333	0.1667	-1	17	-0.6471	-1	2	1	0
HIP_74090	HLVEALYL	NTYGKRN	HLVEALYLNTYGKRN		IAPP	4939/45018	0.929	74090	0.781	0.194273	0.1333	0	2	15	-0.56	1	2	1	0
HIP_74091	ELGGGPGAG	LFQNRMLSK	ELGGGPGAGLFQNRMLSK		SCG2	8361/334890	0.999	74091	0.781	0.069668	0.6833	0.3333	1	18	-0.3	1	1	0	1
HIP_74092	QHLCGSHLV	AILSSTNV	QHLCGSHLVAILSSTNV		IAPP	3821/45018	0.962	74092	0.781	0.022521	2.4833	0	2	17	0.6882	0	0	1	0
HIP_74093	GSHLVEALY	EDMARYYS	GSHLVEALYEDMARYYS		NPY	3709/49410	0.964	74093	0.781	0.081659	-0.1333	0	-1	17	-0.4	-2	1	1	0
HIP_74094	EAEDLQVGQ	EGGGHSRER	EAEDLQVGQEGGGHSRER		SCG1	8067/367830	0.998	74094	0.781	-0.053541	-0.6667	0.5	-2	18	-1.6278	-3	0	0	1
HIP_74095	YLVCGERGF	DLRKESKDQ	YLVCGERGFDLRKESKDQ		SCG2	34879/334890	0.985	74095	0.781	0.028741	-1.05	0.1667	0	18	-1.1167	0	1	0	0
HIP_74096	LQPLALEGS	SKSGYPKTP	LQPLALEGSSKSGYPKTP		SCG2	31145/334890	0.989	74096	0.781	-0.062969	-1.7	0.6667	1	18	-0.6444	1	0	0	0
HIP_74097	LVEALYLVC	NEPQSAPK	LVEALYLVCNEPQSAPK		SCG2	23440/334890	0.995	74097	0.781	0.190291	0.3167	0.1667	-1	17	0.1588	-1	1	1	0
HIP_74098	PLALEGSLQ	GPTKADTEK	PLALEGSLQGPTKADTEK		SCG1	10282/367830	0.998	74098	0.781	-0.041753	-0.5333	0.5	-1	18	-0.7222	-1	0	0	0
HIP_74099	AEDLQVGQV	QQGDLQDTK	AEDLQVGQVQQGDLQDTK		SCG1	14121/367830	0.995	74099	0.781	-0.054130	-1.1833	0.3333	-3	18	-1.0611	-3	0	0	0
HIP_74100	GSLQPLAL	ENLAAMDLE	GSLQPLALENLAAMDLE		SCG1	20048/367830	0.989	74100	0.781	0.104007	1.0333	0	-3	17	0.3529	-3	0	1	0
HIP_74101	CSLYQLEN	EEEEEEEAT	CSLYQLENEEEEEEEAT		CMGA	17677/247050	0.980	74101	0.781	-0.039064	-2.2833	0	-8	17	-1.5235	-8	0	1	5
HIP_74102	VEQCCTSIC	DEEQKLYTD	VEQCCTSICDEEQKLYTD		SCG2	22123/334890	0.995	74102	0.781	0.004355	-0.7167	0.1667	-4	18	-0.6611	-4	0	0	0
HIP_74103	GERGFFYTP	NNQAPGEE	GERGFFYTPNNQAPGEE		CMGA	21420/247050	0.967	74103	0.781	-0.027804	-2.35	0.1667	-2	17	-1.4412	-2	3	1	0
HIP_74104	CGERGFFY	MKRSGQLGI	CGERGFFYMKRSGQLGI		SCG2	42218/334890	0.972	74104	0.781	0.169430	-0.3667	0	2	17	-0.3118	2	3	1	0
HIP_74105	ICSLYQLEN	EEEEEMAV	ICSLYQLENEEEEEMAV		CMGA	10614/247050	0.973	74105	0.781	-0.011686	-2.2833	0	-6	17	-0.6471	-6	1	1	3
HIP_74106	SICSLYQLE	RSEALAVDG	SICSLYQLERSEALAVDG		CMGA	26243/247050	0.943	74106	0.781	-0.039458	-2	0.1667	-2	18	0.2	-2	1	0	0
HIP_74107	HLCGSHLVE	LNDKDQELG	HLCGSHLVELNDKDQELG		SCG2	27346/334890	0.992	74107	0.781	-0.026272	0.2167	0	-1	18	-0.6111	-3	0	0	0
HIP_74108	CGERGFFY	SLIPLIFLI	CGERGFFYSLIPLIFLI		SCG2	21488/334890	0.995	74108	0.781	-0.017460	1.9667	0.1667	0	17	1.3706	0	3	1	0
HIP_74109	VEALYLVC	KWAEGGGHS	VEALYLVCKWAEGGGHS		SCG1	39556/367830	0.921	74109	0.781	0.110959	1.25	0	0	17	0.2235	-1	2	1	1
HIP_74110	VEALYLVCG	LISGAEAAS	VEALYLVCGLISGAEAAS		SCG2	11698/334890	0.998	74110	0.781	-0.065286	2.3	0.3333	-2	18	1.2944	-2	1	0	0
HIP_74111	GSLQPLAL	AWIPHVENR	GSLQPLALAWIPHVENR		SCG2	28536/334890	0.991	74111	0.781	0.038804	2.4667	0	1	17	0.0118	0	1	1	0
HIP_74112	VCGERGFFY	DYETQQWP	VCGERGFFYDYETQQWP		SCG2	29198/334890	0.991	74112	0.781	0.104432	-0.6667	0	-2	17	-0.9588	-2	4	1	0
HIP_74113	VEQCCTSIC	VALNHLKA	VEQCCTSICVALNHLKA		IAPP	3119/45018	0.976	74113	0.781	0.025242	2.6667	0.1667	1	17	0.7353	0	0	1	0
HIP_74114	PLALEGSLQ	EHSQQKEEE	PLALEGSLQEHSQQKEEE		CMGA	16534/247050	0.983	74114	0.781	-0.158626	-1.3333	0.3333	-3	18	-1.4167	-4	0	0	1
HIP_74115	EQCCTSICS	GELFNPYYD	EQCCTSICSGELFNPYYD		SCG1	9301/367830	0.998	74115	0.781	-0.020350	1.0167	0.3333	-3	18	-0.3222	-3	1	0	0
HIP_74116	GSLQPLAL	HWLGAALSL	GSLQPLALHWLGAALSL		SCG2	31408/334890	0.989	74116	0.781	0.049769	1.5167	0	1	17	0.9765	0	1	1	0
HIP_74117	VEALYLVCG	ENGDESHLP	VEALYLVCGENGDESHLP		SCG2	13740/334890	0.998	74117	0.781	0.075206	-0.1833	0.3333	-3	18	-0.0611	-4	1	0	0
HIP_74118	GIVEQCCTS	RDNPFKRTN	GIVEQCCTSRDNPFKRTN		SCG2	46602/334890	0.957	74118	0.781	0.074350	-1.75	0.1667	1	18	-1.0056	1	0	0	0
HIP_74119	VEALYLVC	YTDDEDDIY	VEALYLVCYTDDEDDIY		SCG2	24000/334890	0.994	74119	0.781	0.096439	0.8333	0	-6	17	-0.0471	-6	2	1	0
HIP_74120	ERGFFYTPK	GDTEVMKCI	ERGFFYTPKGDTEVMKCI		CMGA	19565/247050	0.973	74120	0.781	-0.101002	-1.8	0.3333	0	18	-0.5111	0	2	0	0
HIP_74121	CSLYQLENY	GRGGEPRAY	CSLYQLENYGRGGEPRAY		SCG1	24655/367830	0.981	74121	0.781	0.035013	-2.2667	0.3333	0	18	-1.0333	0	1	0	0
HIP_74122	GSLQPLAL	QEKVLPRLP	GSLQPLALQEKVLPRLP		SCG2	33154/334890	0.987	74122	0.781	0.224222	-0.25	0	1	17	0.0059	1	0	1	0
HIP_74123	EALYLVCGE	FPDFYDSEE	EALYLVCGEFPDFYDSEE		SCG1	9813/367830	0.998	74123	0.781	-0.209463	-0.6167	0.3333	-6	18	-0.2611	-6	2	0	0
HIP_74124	VEALYLVCG	HPQGAWKEL	VEALYLVCGHPQGAWKEL		SCG1	27217/367830	0.974	74124	0.781	0.026496	-0.3333	0.3333	0	18	0.2056	-1	1	0	0
HIP_74125	LVCGERGFF	ALQGAKER	LVCGERGFFALQGAKER		CMGA	18077/247050	0.979	74125	0.781	0.024436	1.2167	0.1667	1	17	-0.0647	1	2	1	0
HIP_74126	VEALYLVCG	ENQMDKAR	VEALYLVCGENQMDKAR		SCG1	27267/367830	0.974	74126	0.781	0.069273	-0.7	0.1667	-1	17	-0.2118	-1	1	1	0
HIP_74127	LVEALYLVC	PLQQKENG	LVEALYLVCPLQQKENG		SCG2	21855/334890	0.995	74127	0.781	0.150485	1.5333	0.1667	-1	17	0.1882	-1	1	1	0
HIP_74128	GERGFFYT	ELENLAAMD	GERGFFYTELENLAAMD		SCG1	28090/367830	0.972	74128	0.781	-0.050582	-0.4	0	-3	17	-0.3588	-3	3	1	0
HIP_74129	VEQCCTSIC	GYPEEKKEE	VEQCCTSICGYPEEKKEE		CMGA	7063/247050	0.997	74129	0.781	0.006708	0.4833	0.5	-3	18	-0.9667	-3	1	0	0
HIP_74130	GGGPGAGSL	SSQESGEET	GGGPGAGSLSSQESGEET		SCG1	11203/367830	0.997	74130	0.781	-0.082190	-0.4167	0.6667	-3	18	-0.9056	-3	0	0	1
HIP_74131	QVGQVELGG	EDQELESLS	QVGQVELGGEDQELESLS		CMGA	10797/247050	0.994	74131	0.781	-0.123856	-1.25	0.3333	-5	18	-0.6111	-5	0	0	0
HIP_74132	ICSLYQLEN	DVMEKREDS	ICSLYQLENDVMEKREDS		CMGA	16469/247050	0.983	74132	0.781	-0.044685	-0.1	0	-3	18	-0.8389	-3	1	0	0
HIP_74133	VEQCCTSIC	ENHPQESKG	VEQCCTSICENHPQESKG		SCG1	29278/367830	0.968	74133	0.781	-0.053172	-0.6667	0.1667	-1	18	-0.9	-2	0	0	0
HIP_74134	QHLCGSHLV	RMLVKYPEI	QHLCGSHLVRMLVKYPEI		SCG2	36244/334890	0.983	74134	0.781	0.058979	1	0	2	18	0.1556	1	0	0	0
HIP_74135	AGSLQPLAL	SQAELKEA	AGSLQPLALSQAELKEA		CMGA	18759/247050	0.976	74135	0.781	0.201733	1.15	0.1667	-1	17	0.0529	-1	0	1	0
HIP_74136	EQCCTSICS	VQKFPSPEM	EQCCTSICSVQKFPSPEM		SCG2	31006/334890	0.989	74136	0.781	-0.000986	0.5	0.1667	-1	18	-0.1833	-1	1	0	0
HIP_74137	CSLYQLENY	THPPASLPS	CSLYQLENYTHPPASLPS		CMGA	22964/247050	0.961	74137	0.781	-0.085614	-2.3	0.1667	0	18	-0.4722	-1	1	0	0
HIP_74138	GPGAGSLQP	RWSKMDQLA	GPGAGSLQPRWSKMDQLA		CMGA	27283/247050	0.936	74138	0.781	0.017905	-1.25	0.3333	1	18	-0.7056	1	1	0	0
HIP_74139	VCGERGFF	EGSANRRP	VCGERGFFEGSANRRP		CMGA	17038/247050	0.982	74139	0.781	0.147548	0.0833	0.5	1	16	-0.8438	1	2	0	0
HIP_74140	CSLYQLENY	SQESGEETG	CSLYQLENYSQESGEETG		SCG1	30285/367830	0.964	74140	0.781	-0.060480	-2.6833	0.1667	-4	18	-1.1611	-4	1	0	0
HIP_74141	CSLYQLENY	QESGEETGS	CSLYQLENYQESGEETGS		SCG1	30286/367830	0.964	74141	0.781	-0.060480	-2.6833	0.1667	-4	18	-1.1611	-4	1	0	0
HIP_74142	LQVGQVELG	GEEAVPEEE	LQVGQVELGGEEAVPEEE		CMGA	9084/247050	0.996	74142	0.781	-0.132005	-1.25	0.3333	-6	18	-0.4889	-6	0	0	1

HIP_74596	QPLALEGSL	FLISGAEAA	QPLALEGSLFLISGAEAA		SCG2	8323/334890	0.999	74596	0.781	0.094299	2.2833	0.3333	-2	18	0.8444	-2	1	0	0
HIP_74597	EQCCTSICS	PGEEEEEE	EQCCTSICSPGEEEEEE		CMGA	5467/247050	0.998	74597	0.781	0.049437	0.1167	0.5	-7	17	-1.1941	-7	0	1	4
HIP_74598	LVEALYLV	GAEAASFQ	LVEALYLVGAEAASFQ		SCG2	15211/334890	0.998	74598	0.781	0.147525	0.7667	0.1667	-2	16	1.05	-2	1	0	0
HIP_74599	LVEALYLVC	FQRNQLLQ	LVEALYLVCFQRNQLLQ		SCG2	34210/334890	0.986	74599	0.781	0.135207	0.8833	0	0	17	0.6588	0	2	1	0
HIP_74600	HLCGSHLVE	MSDDYETQQ	HLCGSHLVEMSDDYETQQ		SCG2	22539/334890	0.995	74600	0.781	-0.001739	0.35	0.1667	-2	18	-0.8444	-4	0	0	0
HIP_74601	SHLVEALYL	RPEDQELE	SHLVEALYLRPEDQELE		CMGA	19810/247050	0.973	74601	0.781	0.220697	-0.55	0.1667	-3	17	-0.6588	-4	1	1	0
HIP_74602	LVCGERGFF	YSALRHYI	LVCGERGFFYSALRHYI		NPY	4015/49410	0.957	74602	0.781	0.027228	0.8167	0.3333	2	17	0.3706	1	3	1	0
HIP_74603	HLVEALYLV	SEVLENQSS	HLVEALYLVSEVLENQSS		CMGA	12283/247050	0.992	74603	0.781	0.098082	1.1	0.1667	-2	18	0.2889	-3	1	0	0
HIP_74604	SLQPLALEG	GGGHSRERA	SLQPLALEGGGGHSRERA		SCG1	12929/367830	0.996	74604	0.781	0.037125	-0.2167	0.6667	1	18	-0.6944	0	0	0	2
HIP_74605	GERGFFYT	AIKEHLNQG	GERGFFYTAIKEHLNQG		SCG2	30110/334890	0.990	74605	0.781	-0.057348	0.5333	0	1	17	-0.7706	0	3	1	0
HIP_74606	VGQVELGGG	PYNQEKVLP	VGQVELGGGPYNQEKVLP		SCG2	11472/334890	0.999	74606	0.781	0.017994	-1.2667	0.6667	-1	18	-0.4056	-1	1	0	1
HIP_74607	VEALYLVCG	NGDESHLPE	VEALYLVCGNGDESHLPE		SCG2	13856/334890	0.998	74607	0.781	0.076480	-0.1833	0.3333	-3	18	-0.0611	-4	1	0	0
HIP_74608	LCGSHLVEA	SRSEALAVD	LCGSHLVEASRSEALAVD		CMGA	23452/247050	0.958	74608	0.781	0.198322	-0.6	0.3333	-1	18	0.3722	-2	0	0	0
HIP_74609	ICSLYQLEN	EAEAGEEAV	ICSLYQLENEAEAGEEAV		CMGA	19481/247050	0.974	74609	0.781	-0.106547	-1.4	0	-5	18	-0.1556	-5	1	0	0
HIP_74610	SHLVEALY	AAGSGRLQN	SHLVEALYAAGSGRLQN		SCG2	22204/334890	0.995	74610	0.781	0.090243	1.25	0.1667	1	17	-0.0529	0	1	1	0
HIP_74611	GSLQPLAL	EAGAPGEED	GSLQPLALEAGAPGEED		SCG1	14353/367830	0.995	74611	0.781	0.124305	1.2167	0.1667	-4	17	-0.3471	-4	0	1	0
HIP_74612	VELGGGPGA	SRGEAGAPG	VELGGGPGASRGEAGAPG		SCG1	17848/367830	0.992	74612	0.781	-0.028870	-0.9833	0.6667	-1	18	-0.2722	-1	0	0	1
HIP_74613	GERGFFYT	MAYENLNDK	GERGFFYTMAYENLNDK		SCG2	23102/334890	0.995	74613	0.781	-0.046822	0.5333	0	-1	17	-0.9941	-1	4	1	0
HIP_74614	QPLALEGSL	MAYENLNDK	QPLALEGSLMAYENLNDK		SCG2	7276/334890	0.999	74614	0.781	0.096162	0.8333	0.3333	-2	18	-0.4611	-2	1	0	0
HIP_74615	TSICSLYQ	EEEEEMAVV	TSICSLYQEEEEEMAVV		CMGA	17732/247050	0.980	74615	0.781	0.062756	-1.9167	0	-5	17	-0.1	-5	1	1	3
HIP_74616	LYQLENYCN	PASLPSQKY	LYQLENYCNPASLPSQKY		CMGA	12771/247050	0.991	74616	0.781	-0.058056	-0.4833	0.3333	0	18	-0.8	0	1	0	0
HIP_74617	GGGPGAGSL	EGHHRVQEN	GGGPGAGSLEGHHRVQEN		SCG1	23030/367830	0.984	74617	0.781	-0.055558	-0.75	0.5	1	18	-1.1056	-1	0	0	1
HIP_74618	VCGERGFF	DPADASEAH	VCGERGFFDPADASEAH		SCG1	19922/367830	0.990	74618	0.781	-0.004979	0.3167	0.3333	-2	17	-0.4235	-3	2	1	0
HIP_74619	QPLALEGSL	EGEEENELT	QPLALEGSLEGEEENELT		SCG1	11512/367830	0.997	74619	0.781	-0.112931	-0.8	0.5	-6	18	-0.8278	-6	0	0	1
HIP_74620	CSLYQLEN	EWERYRGRG	CSLYQLENEWERYRGRG		SCG1	48300/367830	0.865	74620	0.781	0.042780	-1.85	0	0	17	-1.5294	0	2	1	0
HIP_74621	SHLVEALY	VGGEDWNPV	SHLVEALYVGGEDWNPV		SCG2	12663/334890	0.998	74621	0.781	0.150624	1.2833	0.3333	-2	17	-0.0353	-3	1	1	0
HIP_74622	QVGQVELGG	IVLSVALNH	QVGQVELGGIVLSVALNH		IAPP	1503/45018	0.994	74622	0.781	0.130683	2.5833	0.3333	0	18	0.85	-1	0	0	0
HIP_74623	LYQLENYCN	VQKFPSPEM	LYQLENYCNVQKFPSPEM		SCG2	29720/334890	0.990	74623	0.781	-0.105523	-0.9167	0	-1	18	-0.6944	-1	2	0	0
HIP_74624	VCGERGFF	STHQEAENE	VCGERGFFSTHQEAENE		SCG1	27231/367830	0.974	74624	0.781	0.023930	0.0833	0.3333	-2	17	-0.9941	-3	2	1	0
HIP_74625	FVNQHLCGS	YGEEGAPGK	FVNQHLCGSYGEEGAPGK		SCG1	22572/367830	0.985	74625	0.781	-0.051107	-0.65	0.5	0	18	-0.6278	-1	1	0	0
HIP_74626	PGAGSLQPL	GSSEDDLQE	PGAGSLQPLGSSEDDLQE		SCG2	5398/334890	0.999	74626	0.781	-0.030232	-0.55	0.6667	-4	18	-0.8111	-4	0	0	0
HIP_74627	ALYLVCGER	SSEEKHLEE	ALYLVCGERSSEEKHLEE		SCG1	29453/367830	0.967	74627	0.781	-0.142628	-2.25	0.5	-2	18	-0.6944	-3	0	0	0
HIP_74628	CGSHLVEAL	ENKPYALNS	CGSHLVEALENKPYALNS		SCG2	41278/334890	0.974	74628	0.781	0.149109	-1.4667	0	0	18	-0.2389	-1	0	0	0
HIP_74629	DLQVGQVEL	PEPMQESKA	DLQVGQVELPEPMQESKA		CMGA	13773/247050	0.989	74629	0.781	0.156514	-0.3667	0.3333	-3	18	-0.7278	-3	0	0	0
HIP_74630	LYLVCGERG	LITRQRYGK	LYLVCGERGLITRQRYGK		NPY	3687/49410	0.964	74630	0.781	0.162470	-0.1333	0.1667	3	18	-0.35	3	0	0	0
HIP_74631	EAEDLQVGQ	SGYPKTPGR	EAEDLQVGQSGYPKTPGR		SCG2	13041/334890	0.998	74631	0.781	-0.121261	-0.3667	0.5	-1	18	-1.2944	-1	1	0	0
HIP_74632	SHLVEALY	FLEGEEENE	SHLVEALYFLEGEEENE		SCG1	20527/367830	0.989	74632	0.781	-0.045408	1.2333	0	-5	17	-0.5882	-6	2	1	1
HIP_74633	PGAGSLQPL	NDEMKRSGQ	PGAGSLQPLNDEMKRSGQ		SCG2	30632/334890	0.990	74633	0.781	-0.017981	-1.9667	0.1667	0	18	-1.1444	0	0	0	0
HIP_74634	HLVEALYL	KYPEIINSN	HLVEALYLKYPEIINSN		SCG2	31381/334890	0.989	74634	0.781	0.137268	-0.0833	0.1667	0	17	0.0176	-1	2	1	0
HIP_74635	LVCGERGFF	PLDSQSIY	LVCGERGFFPLDSQSIY		SCG2	19115/334890	0.996	74635	0.781	0.018520	0.65	0.3333	-1	17	0.2412	-1	2	1	0
HIP_74636	GSHLVEALY	KGQPRSQE	GSHLVEALYKGQPRSQE		SCG1	31517/367830	0.960	74636	0.781	0.218466	-0.5833	0.1667	1	17	-1.0176	0	1	1	0
HIP_74637	VEQCCTSIC	SEVDKRRTR	VEQCCTSICSEVDKRRTR		SCG1	30933/367830	0.962	74637	0.781	0.033067	1.0167	0.3333	1	18	-0.7778	1	0	0	0
HIP_74638	HLCGSHLVE	GFRGPGPQL	HLCGSHLVEGFRGPGPQL		CMGA	25826/247050	0.945	74638	0.781	0.129929	0.4	0.1667	2	18	-0.1444	0	1	0	0
HIP_74639	LALEGSLQ	NYDWWEKKP	LALEGSLQNYDWWEKKP		SCG1	36600/367830	0.937	74639	0.781	0.030512	-1.4667	0.1667	-1	17	-1.0588	-1	3	1	0
HIP_74640	AGSLQPLAL	LVCLGALAE	AGSLQPLALLVCLGALAE		NPY	3549/49410	0.967	74640	0.781	0.001953	3.3167	0	-1	18	1.4722	-1	0	0	0
HIP_74641	GGPGAGSLQ	NIAYEDVVG	GGPGAGSLQNIAYEDVVG		SCG2	9133/334890	0.999	74641	0.781	-0.074678	0.3833	0.1667	-2	18	0.0333	-2	1	0	0
HIP_74642	CGERGFFYT	NQKTSYFP	CGERGFFYTNQKTSYFP		SCG2	39764/334890	0.977	74642	0.781	0.157958	-1.6833	0	1	17	-0.8941	1	3	1	0
HIP_74643	LVEALYLV	DLKRQYDR	LVEALYLVDLKRQYDR		SCG1	38268/367830	0.928	74643	0.781	0.198161	0.5167	0	0	16	-0.2563	0	1	0	0
HIP_74644	HLCGSHLV	EEEEEEEE	HLCGSHLVEEEEEEEE		CMGA	13637/247050	0.990	74644	0.781	0.071573	-0.95	0	-7	17	-1.4588	-9	0	1	7
HIP_74645	LGGGPGAGS	PQEESEESN	LGGGPGAGSPQEESEESN		SCG1	13596/367830	0.996	74645	0.781	-0.117477	-1.3333	0.5	-4	18	-1.2778	-4	0	0	1
HIP_74646	GPGAGSLQ	EEEEEEATN	GPGAGSLQEEEEEEATN		CMGA	17088/247050	0.982	74646	0.781	-0.041472	-1.8333	0.1667	-6	17	-1.4647	-6	0	1	4
HIP_74647	GPGAGSLQP	VCLGALAEA	GPGAGSLQPVCLGALAEA		NPY	1693/49410	0.990	74647	0.781	0.105479	1.5333	0.1667	-1	18	0.7056	-1	0	0	0
HIP_74648	QHLCGSHLV	DLRKESKDQ	QHLCGSHLVDLRKESKDQ		SCG2	40835/334890	0.975	74648	0.781	0.205890	0.1	0	2	18	-1.1167	0	0	0	0
HIP_74649	GPGAGSLQP	EKAEKGREH	GPGAGSLQPEKAEKGREH		SCG2	41262/334890	0.974	74649	0.781	0.002857	-1.15	0.1667	1	18	-1.5389	0	0	0	0
HIP_74650	ERGFFYTPK	EENELTLN	ERGFFYTPKEENELTLN		SCG1	34225/367830	0.948	74650	0.781	-0.060352	-2.7833	0.1667	-2	17	-1.2294	-2	2	1	0
HIP_74651	QHLCGSHLV	PGRAGTEAL	QHLCGSHLVPGRAGTEAL		SCG2	25098/334890	0.994	74651	0.781	0.214198	-0.2833	0.3333	2	18	-0.0278	0	0	0	0
HIP_74652	CCTSICSLY	VPQGLFRGG	CCTSICSLYVPQGLFRGG		CMGA	15993/247050	0.984	74652	0.781	-0.066688	0.1333	0.3333	1	18	0.6778	1	1	0	0
HIP_74653	LYLVCGERG	YRGRGSEEY	LYLVCGERGYRGRGSEEY		SCG1	30832/367830	0.962	74653	0.781	-0.056983	-2.4333	0.3333	0	18	-0.8889	0	1	0	0
[illegible]																			

HIP_75107	GSLQPLAL	YALNSEKNF	GSLQPLALYALNSEKNF		SCG2	29124/334890	0.991	75107
HIP_75108	QHLCGSHLV	QNRMLSKSG	QHLCGSHLVQNRMLSKSG		SCG2	39033/334890	0.979	75108
HIP_75109	GSLQPLAL	SKEAEKSGE	GSLQPLALSKEAEKSGE		CMGA	21574/247050	0.966	75109
HIP_75110	EQCCTSICS	KSGEATDGA	EQCCTSICSKSGEATDGA		CMGA	15063/247050	0.987	75110
HIP_75111	LCGSHLVEA	PEPMQESKA	LCGSHLVEAPEPMQESKA		CMGA	15735/247050	0.985	75111
HIP_75112	QHLCGSHLV	YGEEGAPG	QHLCGSHLVYGEEGAPG		SCG1	25216/367830	0.980	75112
HIP_75113	GSLQPLALE	KIAEKFSQR	GSLQPLALEKIAEKFSQR		SCG1	43386/367830	0.899	75113
HIP_75114	CCTSICSLY	PQALPEPMQ	CCTSICSLYPQALPEPMQ		CMGA	13434/247050	0.990	75114
HIP_75115	LYQLENYC	DNFLEGEEE	LYQLENYCDNFLEGEEE		SCG1	19721/367830	0.990	75115
HIP_75116	HLVEALYL	NEGMVTRC	HLVEALYLNEGMVTRC		SCG1	25491/367830	0.979	75116
HIP_75117	SLQPLAL	GQVELGGG	SLQPLALGQVELGGG		INSC	263/5184	0.997	75117
HIP_75118	QPLALEGSL	RGRGGEPRA	QPLALEGSLRGRGGEPRA		SCG1	40760/367830	0.914	75118
HIP_75119	LYLVCGERG	QGSSEDDLQ	LYLVCGERGQGSSEDDLQ		SCG2	18416/334890	0.997	75119
HIP_75120	YQLENYCN	WDEEDKRNY	YQLENYCNWDEEDKRNY		SCG1	34574/367830	0.947	75120
HIP_75121	ELGGGPGAG	RGGEPRAYF	ELGGGPGAGRGGEPRAYF		SCG1	19483/367830	0.990	75121
HIP_75122	GAGSLQPLA	NQSSQAELK	GAGSLQPLANQSSQAELK		CMGA	15375/247050	0.986	75122
HIP_75123	IVEQCCTSI	GQPRSQEES	IVEQCCTSIGQPRSQEES		SCG1	9754/367830	0.998	75123
HIP_75124	SICSLYQLE	NFGAILSST	SICSLYQLENFGAILSST		IAPP	2301/45018	0.988	75124
HIP_75125	PLALEGSLQ	LIVLSVAL	PLALEGSLQLIVLSVAL		IAPP	2259/45018	0.988	75125
HIP_75126	CGSHLVEA	GEEEEEEE	CGSHLVEAGEEEEEEE		CMGA	11024/247050	0.994	75126
HIP_75127	LCGSHLVEA	SSHHTAEKR	LCGSHLVEASSHHTAEKR		SCG1	46700/367830	0.876	75127
HIP_75128	LGGGPGAGS	RGSEEYRAP	LGGGPGAGSRGSEEYRAP		SCG1	25158/367830	0.980	75128
HIP_75129	CGSHLVEAL	LRQQAHKEE	CGSHLVEALLRQQAHKEE		SCG2	40114/334890	0.977	75129
HIP_75130	RGFFYTPKT	KRWSKMDQL	RGFFYTPKTKRWSKMDQL		CMGA	33039/247050	0.884	75130
HIP_75131	CGERGFFY	NHPQESKG	CGERGFFYNHPQESKG		SCG1	35249/367830	0.944	75131
HIP_75132	SLYQLENYC	RLGELFNPY	SLYQLENYCRLGELFNPY		SCG1	17667/367830	0.992	75132
HIP_75133	EDLQVGQVE	STENVPRTR	EDLQVGQVESTENVPRTR		NPY	4528/49410	0.945	75133
HIP_75134	SICSLYQLE	SKRWSKMDQ	SICSLYQLESKRWSKMDQ		CMGA	33372/247050	0.881	75134
HIP_75135	HLVEALYLV	NMNDNFLEG	HLVEALYLVNMNDNFLEG		SCG1	23904/367830	0.983	75135
HIP_75136	QPLALEGSL	VFLIVLSV	QPLALEGSLVFLIVLSV		IAPP	1682/45018	0.993	75136
HIP_75137	VEALYLVC	SSKDVMEKR	VEALYLVCSSKDVMEKR		CMGA	22100/247050	0.964	75137
HIP_75138	HLCGSHLVE	EHIAKRAME	HLCGSHLVEEHIAKRAME		SCG2	40950/334890	0.975	75138
HIP_75139	DLQVGQVEL	SKGQPRSQE	DLQVGQVELSKGQPRSQE		SCG1	32185/367830	0.957	75139
HIP_75140	CGERGFFY	SEKNFPMD	CGERGFFYSEKNFPMD		SCG2	31221/334890	0.989	75140
HIP_75141	LVEALYLVC	GRAGTEAL	LVEALYLVCGRAGTEAL		SCG2	26186/334890	0.993	75141
HIP_75142	LVEALYL	SLGYKEI	LVEALYLSLGYKEI		CMGA	12782/247050	0.991	75142
HIP_75143	TSICSLYQ	WQAKREEEE	TSICSLYQWQAKREEEE		CMGA	20489/247050	0.970	75143
HIP_75144	HLVEALYL	HQEAENEK	HLVEALYLHQEAENEK		SCG1	33385/367830	0.952	75144
HIP_75145	QCCTSICSL	GGEDWNPVE	QCCTSICSLGGEDWNPVE		SCG2	12003/334890	0.998	75145
HIP_75146	LVEALYLVC	GRGGEPRA	LVEALYLVCGRGGEPRA		SCG1	28443/367830	0.971	75146
HIP_75147	GGGPGAGSL	YDPLQWKSS	GGGPGAGSLYDPLQWKSS		SCG1	21010/367830	0.988	75147
HIP_75148	LYLVCGERG	EEEEGENYQ	LYLVCGERGEEEEGENYQ		SCG1	30600/367830	0.963	75148
HIP_75149	HLVEALYL	LIFLISGAE	HLVEALYLLIFLISGAE		SCG2	22111/334890	0.995	75149
HIP_75150	GSLQPLAL	DTEKWAEG	GSLQPLALDTEKWAEG		SCG1	26050/367830	0.978	75150
HIP_75151	GPGAGSLQP	GPTVVLNPH	GPGAGSLQPGPTVVLNPH		CMGA	10358/247050	0.994	75151
HIP_75152	LVCGERGFF	SSAPPITPE	LVCGERGFFSSAPPITPE		SCG1	18101/367830	0.992	75152
HIP_75153	LYQLENYCN	TEKWAEGG	LYQLENYCNTEKWAEGG		SCG1	37149/367830	0.934	75153
HIP_75154	QVELGGGPG	NVPRTRLED	QVELGGGPGNVPRTRLED		NPY	1126/49410	0.994	75154
HIP_75155	SHLVEALYL	RYRGRGSE	SHLVEALYLRYRGRGSE		SCG1	48442/367830	0.864	75155
HIP_75156	QCCTSICSL	DNFLEGEEE	QCCTSICSLDNFLEGEEE		SCG1	17218/367830	0.993	75156
HIP_75157	LCGSHLVEA	LRHYINLIT	LCGSHLVEALRHYINLIT		NPY	6669/49410	0.864	75157
HIP_75158	EQCCTSICS	SGEETGSQE	EQCCTSICSSGEETGSQE		SCG1	11482/367830	0.997	75158
HIP_75159	EQCCTSICS	ESGEETGSQ	EQCCTSICSESGEETGSQ		SCG1	11483/367830	0.997	75159
HIP_75160	DLQVGQVEL	GSNTYGKRN	DLQVGQVELGSNTYGKRN		IAPP	3447/45018	0.969	75160
HIP_75161	RGFFYTPKT	LPSEEKGHP	RGFFYTPKTLPSEEKGHP		SCG1	29761/367830	0.966	75161
HIP_75162	CSLYQLENY	SKPDNPGED	CSLYQLENYSKPDNPGED		NPY	3474/49410	0.968	75162
HIP_75163	YLVCGERGF	EVISDTLSK	YLVCGERGFEVISDTLSK		CMGA	17127/247050	0.982	75163
HIP_75164	HLCGSHLVE	AGRSRSNQL	HLCGSHLVEAGRSRSNQL		SCG2	37908/334890	0.981	75164
HIP_75165	VEALYLVC	SVALNHLK	VEALYLVCSVALNHLK		IAPP	3385/45018	0.971	75165
HIP_75166	VCGERGFFY	KLKHMQFP	VCGERGFFYKLKHMQFP		SCG2	42529/334890	0.971	75166
HIP_75167	GSLQPLAL	WRPSSRED	GSLQPLALWRPSSRED		CMGA	25036/247050	0.950	75167
HIP_75168	HLCGSHLVE	GKLTGPNNQ	HLCGSHLVEGKLTGPNNQ		SCG2	33548/334890	0.987	75168
HIP_75169	HLVEALYLV	SREKSSQE	HLVEALYLVSREKSSQE		SCG1	33959/367830	0.949	75169
HIP_75170	IVEQCCTSI	TLISDLLMR	IVEQCCTSITLISDLLMR		NPY	2296/49410	0.984	75170
HIP_75171	VELGGGPGA	NEKDRADQT	VELGGGPGANEKDRADQT		SCG1	26291/367830	0.977	75171
HIP_75172	AEDLQVGQV	GSSQETDKL	AEDLQVGQVGSSQETDKL		SCG2	6507/334890	0.999	75172
HIP_75173	LVEALYLV	PGAEEAQDP	LVEALYLVPGAEEAQDP		CMGA	6883/247050	0.997	75173
HIP_75174	YLVCGERGF	LTAEKRLEG	YLVCGERGFLTAEKRLEG		CMGA	13428/247050	0.990	75174
HIP_75175	GSLQPLALE	ASEAHESSS	GSLQPLALEASEAHESSS		SCG1	25624/367830	0.979	75175
HIP_75176	ICSLYQLEN	QPTLLLSLL	ICSLYQLENQPTLLLSLL		SCG1	26647/367830	0.976	75176
HIP_75177	LGGGPGA	LALEGSLQ	LGGGPGALALEGSLQ		INSC	137/5184	0.998	75177
HIP_75178	GPGAGSLQP	EYIENLRQQ	GPGAGSLQPEYIENLRQQ		SCG2	21200/334890	0.996	75178
HIP_75179	EQCCTSICS	LLQKEPDLR	EQCCTSICSLLQKEPDLR		SCG2	22260/334890	0.995	75179
HIP_75180	YLVCGERGF	SAEFPDFYD	YLVCGERGFSAEFPDFYD		SCG1	15007/367830	0.995	75180
HIP_75181	GPGAGSLQP	KENIEKNEQ	GPGAGSLQPKENIEKNEQ		SCG2	35583/334890	0.984	75181
HIP_75182	QHLCGSHLV	THAAGHSQE	QHLCGSHLVTHAAGHSQE		SCG1	39962/367830	0.919	75182
HIP_75183	IVEQCCTSI	ARYYSALRH	IVEQCCTSIARYYSALRH		NPY	5352/49410	0.922	75183
HIP_75184	CTSICSLYQ	IPHVENRQM	CTSICSLYQIPHVENRQM		SCG2	25206/334890	0.994	75184
HIP_75185	GIVEQCCTS	DEPQWSLYP	GIVEQCCTSDEPQWSLYP		SCG1	22690/367830	0.985	75185
HIP_75186	LVCGERGFF	HTAEKRKR	LVCGERGFFHTAEKRKR		SCG1	41338/367830	0.910	75186
HIP_75187	VCGERGFF	HTAEKRKRL	VCGERGFFHTAEKRKRL		SCG1	41339/367830	0.910	75187
HIP_75188	QPLALEGSL	YGEESEEER	QPLALEGSLYGEESEEER		SCG1	10721/367830	0.997	75188
HIP_75189	HLVEALYL	KSQREDEEE	HLVEALYLKSQREDEEE		SCG1	30456/367830	0.964	75189
HIP_75190	GGGPGAGSL	GPTKADTEK	GGGPGAGSLGPTKADTEK		SCG1	15255/367830	0.995	75190
HIP_75191	QVGQVELGG	QGSSEDDLQ	QVGQVELGGQGSSEDDLQ		SCG2	5592/334890	0.999	75191
HIP_75192	GPGAGSLQP	IINSNQVKR	GPGAGSLQPIINSNQVKR		SCG2	22376/334890	0.995	75192
HIP_75193	LYQLENYCN	YNYDWWEK	LYQLENYCNYNYDWWEK		SCG1	34629/367830	0.946	75193
HIP_75194	NQHLCGSHL	FRGPGPQLR	NQHLCGSHLFRGPGPQLR		CMGA	25618/247050	0.947	75194
HIP_75195	AGSLQPLA	SEESWDEED	AGSLQPLASEESWDEED		SCG1	18818/367830	0.991	75195
HIP_75196	DLQVGQVEL	LLLCAGQVT	DLQVGQVELLLLCAGQVT		CMGA	14285/247050	0.988	75196
HIP_75197	SHLVEALY	GRGGEPRAY	SHLVEALYGRGGEPRAY		SCG1	29560/367830	0.967	75197
HIP_75198	ERGFFYTPK	SEGLSQGLV	ERGFFYTPKSEGLSQGLV		CMGA	18598/247050	0.977	75198
HIP_75199	ERGFFYTPK	EDVVGGEDW	ERGFFYTPKEDVVGGEDW		SCG2	24847/334890	0.994	75199
HIP_75200	GPGAGSLQP	SKENIEKNE	GPGAGSLQPSKENIEKNE		SCG2	33387/334890	0.987	75200
HIP_75201	CCTSICSLY	EISRNLQIP	CCTSICSLYEISRNLQIP		SCG2	23432/334890	0.995	75201
HIP_75202	ICSLYQLEN	IENLRQQAH	ICSLYQLENIENLRQQAH		SCG2	32989/334890	0.987	75202
HIP_75203	LVEALYLV	WDEDLLMKV	LVEALYLVWDEDLLMKV		SCG2	25032/334890	0.994	75203
HIP_75204	EQCCTSICS	QEEVRDSKE	EQCCTSICSQEEVRDSKE		SCG2	22805/334890	0.995	75204
HIP_75205	VEALYLVC	SEEKHLEE	VEALYLVCSEEKHLEE		SCG1	23880/367830	0.983	75205

HIP_75618	GSLQPLAL	GEEDATSE	GSLQPLALGEEDATSE		SCG1	20576/367830	0.989	75618	0.780	0.165213	0.3333	0.1667	-4	16	-0.45	-4	0	0	0
HIP_75619	CSLYQLENY	CRQVLKTSR	CSLYQLENYCRQVLKTSR		SCG1	45925/367830	0.882	75619	0.780	-0.073322	-2.3	0	2	18	-0.6222	2	1	0	0
HIP_75620	SLYQLENY	EEEEEEEEA	SLYQLENYEEEEEEEEA		CMGA	18587/247050	0.977	75620	0.780	-0.078751	-3.1333	0	-9	17	-1.9118	-9	1	1	6
HIP_75621	SLYQLENY	EEEEEEEEA	SLYQLENYEEEEEEEEA		CMGA	18588/247050	0.977	75621	0.780	-0.078751	-3.1333	0	-9	17	-1.9118	-9	1	1	6
HIP_75622	IVEQCCTSI	LFEKPLDSQ	IVEQCCTSILFEKPLDSQ		SCG2	14962/334890	0.998	75622	0.780	-0.033702	1.0167	0.1667	-2	18	0.1833	-2	1	0	0
HIP_75623	GIVEQCCTS	KYPEIINSN	GIVEQCCTSKYPEIINSN		SCG2	33421/334890	0.987	75623	0.780	-0.014664	-0.9667	0.3333	-1	18	-0.2389	-1	1	0	0
HIP_75624	LYLVCGERG	REDSLEAGL	LYLVCGERGREDSLEAGL		CMGA	26402/247050	0.942	75624	0.780	-0.066692	-3.3167	0.1667	-2	18	-0.1444	-2	0	0	0
HIP_75625	VEALYLVC	DSKEAEKSG	VEALYLVCDSKEAEKSG		CMGA	21745/247050	0.966	75625	0.780	0.229942	0.3833	0.1667	-2	17	-0.1765	-2	1	1	0
HIP_75626	CTSICSLY	GEEIKGYPG	CTSICSLYGEEIKGYPG		SCG1	24590/367830	0.981	75626	0.780	0.131900	-0.95	0.3333	-1	17	-0.0471	-1	1	1	0
HIP_75627	CGERGFFY	PGVQAPEDL	CGERGFFYPGVQAPEDL		SCG1	13331/367830	0.996	75627	0.780	0.133325	1.0833	0.3333	-2	17	-0.3706	-2	3	1	0
HIP_75628	DLQVGQVEL	ESKGQPRSQ	DLQVGQVELESKGQPRSQ		SCG1	43469/367830	0.898	75628	0.780	0.216891	-0.6167	0.1667	-1	18	-1.1611	-1	0	0	0
HIP_75629	VNQHLCGSH	PEPMQESKA	VNQHLCGSHPEPMQESKA		CMGA	17312/247050	0.981	75629	0.780	-0.114072	-1.85	0.6667	1	18	-1.0444	-1	0	0	0
HIP_75630	SLQPLAL	KWAEGGGHS	SLQPLALKWAEGGGHS		SCG1	42792/367830	0.902	75630	0.780	0.128163	1.0667	0	1	16	-0.275	0	1	2	1
HIP_75631	SLYQLENYC	PEYNYDWW	SLYQLENYCPEYNYDWW		SCG1	25483/367830	0.979	75631	0.780	-0.032274	-1.45	0.1667	-3	17	-1.1941	-3	2	1	0
HIP_75632	HLVEALYLV	LEGQEEEED	HLVEALYLVLEGQEEEED		CMGA	6564/247050	0.998	75632	0.780	0.052743	1.1	0.1667	-6	18	-0.4167	-7	1	0	2
HIP_75633	LQPLALEGS	EESSPDYNP	LQPLALEGSEESSPDYNP		SCG2	21893/334890	0.995	75633	0.780	-0.135596	-2.0833	0.5	-4	18	-0.9278	-4	0	0	0
HIP_75634	LYQLENYCN	LNEKNFFPE	LYQLENYCNLNEKNFFPE		SCG1	27864/367830	0.972	75634	0.780	-0.124112	-0.9167	0	-2	18	-0.9222	-2	1	0	0
HIP_75635	PGAGSLQPL	LPLQVRGYP	PGAGSLQPLLPLQVRGYP		CMGA	12774/247050	0.991	75635	0.780	-0.006575	0.7833	0.3333	1	18	0	1	0	0	0
HIP_75636	GSHLVEALY	DDIYKANN	GSHLVEALYDDIYKANN		SCG2	26203/334890	0.993	75636	0.780	0.099289	0.3	0	-1	17	-0.5	-2	2	1	0
HIP_75637	VEQCCTSIC	DNPFKRTNE	VEQCCTSICDNPFKRTNE		SCG2	22271/334890	0.995	75637	0.780	0.076903	-0.4	0.3333	-1	18	-0.7889	-1	1	0	0
HIP_75638	CGSHLVEAL	EGNNQAPGE	CGSHLVEALEGNNQAPGE		CMGA	13921/247050	0.989	75638	0.780	0.140086	-0.8833	0.1667	-2	18	-0.55	-3	0	0	0
HIP_75639	ELGGGPGAG	SYFPNPYNQ	ELGGGPGAGSYFPNPYNQ		SCG2	4193/334890	1.000	75639	0.780	0.008217	0.2833	0.5	-1	18	-0.8778	-1	2	0	1
HIP_75640	PLALEGSLQ	EMKRSGQLG	PLALEGSLQEMKRSGQLG		SCG2	44595/334890	0.965	75640	0.780	-0.046200	-1	0.1667	0	18	-0.4389	0	0	0	0
HIP_75641	GIVEQCCT	GEEEEEEEE	GIVEQCCTGEEEEEEEE		CMGA	10065/247050	0.995	75641	0.780	0.041468	-0.5167	0.1667	-9	17	-1.3412	-9	0	1	6
HIP_75642	ICSLYQLEN	LTLALSLLV	ICSLYQLENLTLALSLLV		NPY	2615/49410	0.981	75642	0.780	-0.060735	0.6167	0	-1	18	1.4167	-1	1	0	0
HIP_75643	EDLQVGQVE	EENELTLNE	EDLQVGQVEEENELTLNE		SCG1	27472/367830	0.974	75643	0.780	-0.115882	-2.2167	0	-7	18	-1.1	-7	0	0	1
HIP_75644	NQHLCGSHL	GEDWNPVEE	NQHLCGSHLGEDWNPVEE		SCG2	18144/334890	0.997	75644	0.780	0.088752	-1.2667	0.3333	-2	18	-1.15	-4	1	0	0
HIP_75645	GIVEQCCTS	EKRFLGEGH	GIVEQCCTSEKRFLGEGH		SCG1	51015/367830	0.843	75645	0.780	0.057798	-1.8167	0.1667	0	18	-0.4444	-1	1	0	0
HIP_75646	CCTSICSL	WGYEKRNLA	CCTSICSLWGYEKRNLA		SCG1	29592/367830	0.967	75646	0.780	0.017499	0.4833	0.3333	1	17	0.0647	1	2	1	0
HIP_75647	NQHLCGSHL	ELTAEKRLE	NQHLCGSHLELTAEKRLE		CMGA	26147/247050	0.943	75647	0.780	-0.041987	-0.1	0.1667	1	18	-0.8167	-1	0	0	0
HIP_75648	GSHLVEALY	MKCIVEVIS	GSHLVEALYMKCIVEVIS		CMGA	20458/247050	0.970	75648	0.780	-0.016823	0.8	0	0	18	1	-1	1	0	0
HIP_75649	VCGERGFF	EKGHPQEE	VCGERGFFEKGHPQEE		SCG1	30378/367830	0.964	75649	0.780	0.128017	-0.4333	0.3333	-1	16	-1.225	-2	2	0	0
HIP_75650	HLCGSHLVE	RYRGRGSEE	HLCGSHLVERYRGRGSEE		SCG1	51740/367830	0.837	75650	0.780	0.088727	-0.9667	0	2	18	-1.1222	0	1	0	0
HIP_75651	CGERGFF	NAVEVLK	CGERGFFNAVEVLK		IAPP	2693/45018	0.982	75651	0.780	0.014280	1.2833	0.1667	0	14	0.1714	0	2	0	0
HIP_75652	ICSLYQLEN	VCLGALAEA	ICSLYQLENVCLGALAEA		NPY	2089/49410	0.986	75652	0.780	0.019231	1.2167	0	-2	18	0.9889	-2	1	0	0
HIP_75653	EQCCTSICS	PECRQVLKT	EQCCTSICSPECRQVLKT		SCG1	24224/367830	0.982	75653	0.780	0.018724	0.6	0.3333	0	18	-0.25	0	0	0	0
HIP_75654	QCCTSICSL	WAEGGGHSR	QCCTSICSLWAEGGGHSR		SCG1	34403/367830	0.947	75654	0.780	0.044355	0.4833	0.1667	1	18	-0.1278	0	1	0	1
HIP_75655	HLVEALYL	YKEIRKGES	HLVEALYLYKEIRKGES		CMGA	28134/247050	0.930	75655	0.780	0.159145	-0.4	0	1	17	-0.4647	0	2	1	0
HIP_75656	EQCCTSICS	ELESLSAIE	EQCCTSICSELESLSAIE		CMGA	14304/247050	0.988	75656	0.780	-0.055512	0.5	0.1667	-4	18	0.25	-4	0	0	0
HIP_75657	CCTSICSL	DWWEKKPFS	CCTSICSLDWWEKKPFS		SCG1	33723/367830	0.950	75657	0.780	0.014263	0.0333	0.1667	0	17	-0.1588	0	2	1	0
HIP_75658	CSLYQLENY	AHQLQALRR	CSLYQLENYAHQLQALRR		CMGA	28704/247050	0.925	75658	0.780	-0.049275	-2.2	0	2	18	-0.6556	1	1	0	0
HIP_75659	SLQPLALE	TQQWPERKL	SLQPLALETQQWPERKL		SCG2	41167/334890	0.974	75659	0.780	0.182751	-0.9333	0	0	17	-0.8529	0	1	1	0
HIP_75660	CGSHLVEAL	QGAKERAHQ	CGSHLVEALQGAKERAHQ		CMGA	21258/247050	0.967	75660	0.780	0.093614	0	0.1667	2	18	-0.5944	0	0	0	0
HIP_75661	QCCTSICSL	GEKRDHHST	QCCTSICSLGEKRDHHST		SCG1	35114/367830	0.944	75661	0.780	0.225336	-0.3833	0.3333	2	18	-0.7611	0	0	0	0
HIP_75662	GSLQPLALE	EDVNWGYEK	GSLQPLALEEDVNWGYEK		SCG1	33091/367830	0.953	75662	0.780	-0.018064	-0.1167	0	-3	18	-0.7167	-3	0	0	0
HIP_75663	LGGGPGAGS	PYQGVSVPL	LGGGPGAGSPYQGVSVPL		SCG2	20047/334890	0.996	75663	0.780	-0.042132	-0.9667	0.5	0	18	0.2333	0	1	0	1
HIP_75664	GERGFFYT	GSGRLQNG	GERGFFYTGSGRLQNG		SCG2	20105/334890	0.996	75664	0.780	0.184542	-0.1333	0.5	1	16	-0.9313	1	3	0	0
HIP_75665	GPGAGSLQP	LSKSGYPKT	GPGAGSLQPLSKSGYPKT		SCG2	34462/334890	0.985	75665	0.780	-0.036375	-0.3667	0.3333	2	18	-0.7056	2	0	0	0
HIP_75666	DLQVGQVEL	ERMDEEQKL	DLQVGQVELERMDEEQKL		SCG2	36350/334890	0.983	75666	0.780	0.044054	-0.2667	0	-4	18	-1.0333	-4	0	0	0
HIP_75667	GSLQPLAL	PSKPDNPG	GSLQPLALPSKPDNPG		NPY	4165/49410	0.954	75667	0.780	0.237134	0.5167	0.3333	0	16	-0.625	0	0	0	0
HIP_75668	CSLYQLENY	NLQIPPEDL	CSLYQLENYNLQIPPEDL		SCG2	22752/334890	0.995	75668	0.780	-0.187995	-1.9167	0	-3	18	-0.4944	-3	1	0	0
HIP_75669	VNQHLCGSH	LEPGKGRHH	VNQHLCGSHLEPGKGRHH		SCG1	33529/367830	0.951	75669	0.780	-0.067038	-0.95	0.5	5	18	-1.1667	1	0	0	0
HIP_75670	VEALYLVCG	SKMDQLAKE	VEALYLVCGSKMDQLAKE		CMGA	19536/247050	0.974	75670	0.780	0.033845	0.5833	0.3333	-1	18	0.1944	-1	1	0	0
HIP_75671	QCCTSICSL	KGREHIAKR	QCCTSICSLKGREHIAKR		SCG2	41702/334890	0.973	75671	0.780	0.191483	-0.55	0.3333	4	18	-0.4222	3	0	0	0
HIP_75672	EAEDLQVGQ	LIVLSVALN	EAEDLQVGQLIVLSVALN		IAPP	1536/45018	0.994	75672	0.780	0.063847	2.1333	0.1667	-3	18	0.7611	-3	0	0	0
HIP_75673	VEQCCTSIC	GSQENHPQE	VEQCCTSICGSQENHPQE		SCG1	13068/367830	0.996	75673	0.780	0.116774	0.25	0.5	-2	18	-0.8778	-3	0	0	0
HIP_75674	CGERGFFYT	LSSTNVGSN	CGERGFFYTLSSTNVGSN		IAPP	2609/45018	0.983	75674	0.780	-0.005243	0.5	0.3333	0	18	-0.2889	0	3	0	0
HIP_75675	SICSLYQL	YDWWEKKPF	SICSLYQLYDWWEKKPF		SCG1	38128/367830	0.929	75675	0.780	-0.000696	-1.1167	0	0	17	-0.5	0	4	1	0
[illegible]	[illegible]	[illegible]	[illegible]	[illegible]	[illegible]	[illegible]	[illegible]	[illegible]	[illegible]	[illegible]	[illegible]	[illegible]	[illegible]	[illegible]	[illegible]	[illegible]	[illegible]	[illegible]	[illegible]

HIP_76129	EAEDLQVGQ	EGEEDATSE	EAEDLQVGQEGEEDATSE		SCG1	12833/367830	0.996	76129	0.779	-0.124256	-1.1833	0.3333	-8	18	-1.4278	-8	0	0	0
HIP_76130	GAGSLQPLA	SVALNHLKA	GAGSLQPLASVALNHLKA		IAPP	3176/45018	0.975	76130	0.779	-0.027813	1.5333	0.3333	2	18	0.4722	1	0	0	0
HIP_76131	TSICSLYQL	EPRAYFMSD	TSICSLYQLEPRAYFMSD		SCG1	34147/367830	0.948	76131	0.779	0.183985	-1.7667	0.1667	-1	18	-0.0667	-1	1	0	0
HIP_76132	QVELGGGPG	EQCCTSIC	QVELGGGPGEQCCTSIC		INSA	75/3024	0.985	76132	0.779	0.013242	-1.15	0.5	-2	17	0.0765	-2	0	1	1
HIP_76133	GGGPGAGSL	RSSPETLIS	GGGPGAGSLRSSPETLIS		NPY	3913/49410	0.959	76133	0.779	0.007284	-0.5833	0.6667	0	18	-0.1778	0	0	0	1
HIP_76134	CTSICSLYQ	EEEEEEEA	CTSICSLYQEEEEEEEA		CMGA	15995/247050	0.984	76134	0.779	0.022175	-1.9167	0	-7	17	-0.9706	-7	1	1	5
HIP_76135	CTSICSLYQ	EEEEEEEA	CTSICSLYQEEEEEEEA		CMGA	15996/247050	0.984	76135	0.779	0.022175	-1.9167	0	-7	17	-0.9706	-7	1	1	5
HIP_76136	VEALYLVC	TPNRQYWD	VEALYLVCTPNRQYWD		SCG2	23811/334890	0.994	76136	0.779	0.206052	0.7833	0.1667	-1	16	-0.25	-1	1	0	0
HIP_76137	QCCTSICSL	DELSEVLEN	QCCTSICSLDELSEVLEN		CMGA	15196/247050	0.986	76137	0.779	-0.031463	0.3833	0.1667	-4	18	0.1944	-4	0	0	0
HIP_76138	GERGFFYTP	SKAEGNNQA	GERGFFYTPSKAEGNNQA		CMGA	20244/247050	0.971	76138	0.779	-0.103673	-1.0833	0.3333	0	18	-1.2389	0	3	0	0
HIP_76139	LCGSHLVEA	EAEAGEEAV	LCGSHLVEAEAEAGEEAV		CMGA	19273/247050	0.974	76139	0.779	-0.016423	-0.45	0	-4	18	0.1889	-5	0	0	0
HIP_76140	CGERGFFY	KETTENEN	CGERGFFYKETTENEN		SCG1	35269/367830	0.944	76140	0.779	0.184446	-0.6333	0	-2	16	-1.55	-2	3	0	0
HIP_76141	GSLQPLAL	ERATRLFEK	GSLQPLALERATRLFEK		SCG2	39329/334890	0.978	76141	0.779	0.215050	0.5333	0	1	17	-0.3118	1	0	1	0
HIP_76142	CGERGFFY	EQKLYTDD	CGERGFFYEQKLYTDD		SCG2	31903/334890	0.988	76142	0.779	0.171280	-1.1	0	-2	16	-1.1313	-2	3	0	0
HIP_76143	VGQVELGGG	ESQTQEEVR	VGQVELGGGESQTQEEVR		SCG2	11371/334890	0.999	76143	0.779	-0.075047	-1.5	0.6667	-3	18	-0.8722	-3	0	0	1
HIP_76144	EALYLVCGE	QGLFRGGKS	EALYLVCGEQGLFRGGKS		CMGA	11582/247050	0.993	76144	0.779	-0.158131	-0.25	0.3333	0	18	0.0056	0	1	0	0
HIP_76145	SLYQLENYC	SVALNHLKA	SLYQLENYCSVALNHLKA		IAPP	3123/45018	0.976	76145	0.779	-0.110710	0.4833	0.1667	1	18	0.0111	0	1	0	0
HIP_76146	PGAGSLQPL	SRSNQLPKA	PGAGSLQPLSRSNQLPKA		SCG2	36926/334890	0.982	76146	0.779	0.089598	-1.2333	0.5	2	18	-0.6611	2	0	0	0
HIP_76147	CCTSICSLY	YSSHHTAE	CCTSICSLYYSSHHTAE		SCG1	26214/367830	0.977	76147	0.779	0.003455	-0.2	0.5	1	17	0.0294	-1	2	1	0
HIP_76148	CSLYQLENY	PSPMPVSQE	CSLYQLENYPSPMPVSQE		CMGA	13149/247050	0.990	76148	0.779	-0.067276	-2.05	0.5	-2	18	-0.6167	-2	1	0	0
HIP_76149	EQCCTSICS	KYPEIINSN	EQCCTSICSKYPEIINSN		SCG2	33998/334890	0.986	76149	0.779	0.072063	-0.1	0.3333	-1	18	-0.3556	-1	1	0	0
HIP_76150	HLVEALYL	LESVFQEL	HLVEALYLLESVFQEL		SCG2	17478/334890	0.997	76150	0.779	0.114845	0.9667	0.1667	-2	16	0.7937	-3	1	0	0
HIP_76151	SICSLYQLE	WSKMDQLAK	SICSLYQLEWSKMDQLAK		CMGA	27879/247050	0.932	76151	0.779	-0.081467	-1.4667	0.1667	0	18	-0.2389	0	2	0	0
HIP_76152	LVCGERGF	GEEEEEEEE	LVCGERGFGEEEEEEEE		CMGA	11343/247050	0.993	76152	0.779	-0.067611	-1.5833	0.3333	-8	17	-1.4059	-8	1	1	6
HIP_76153	CSLYQLENY	KIAEKFSQR	CSLYQLENYKIAEKFSQR		SCG1	32456/367830	0.956	76153	0.779	-0.001261	-0.9833	0	1	18	-0.8222	1	1	0	0
HIP_76154	DLQVGQVEL	THSREKSSQ	DLQVGQVELTHSREKSSQ		SCG1	46960/367830	0.875	76154	0.779	0.146300	-0.0333	0.1667	0	18	-1.1167	-1	0	0	0
HIP_76155	HLVEALYL	FQRNQLLQ	HLVEALYLFQRNQLLQ		SCG2	37870/334890	0.981	76155	0.779	0.174906	0.1833	0	1	16	0.0812	0	2	0	0
HIP_76156	QHLCGSHLV	SKMDQLAKE	QHLCGSHLVSKMDQLAKE		CMGA	26696/247050	0.940	76156	0.779	0.218163	0.3333	0.1667	2	18	-0.4667	0	0	0	0
HIP_76157	LQPLALEGS	PDYNPYQGV	LQPLALEGSPDYNPYQGV		SCG2	15890/334890	0.997	76157	0.779	-0.066652	-1.85	0.5	-2	18	-0.5056	-2	1	0	0
HIP_76158	HLCGSHLV	DPLQWKSSH	HLCGSHLVDPLQWKSSH		SCG1	31902/367830	0.958	76158	0.779	0.194477	0.5833	0.1667	3	17	-0.4529	0	1	1	0
HIP_76159	VCGERGFFY	LVKYPEII	VCGERGFFYLVKYPEII		SCG2	28260/334890	0.992	76159	0.779	0.013781	1.4	0	0	17	0.5235	0	4	1	0
HIP_76160	GGPGAGSLQ	FEDELSEVL	GGPGAGSLQFEDELSEVL		CMGA	15144/247050	0.986	76160	0.779	-0.157132	-0.7833	0.1667	-4	18	-0.1167	-4	1	0	0
HIP_76161	LVCGERGFF	GMESAANQ	LVCGERGFFGMESAANQ		SCG2	20081/334890	0.996	76161	0.779	0.046027	0.5333	0.3333	-1	17	0.0647	-1	2	1	0
HIP_76162	EALYLVCGE	QENQMDKAR	EALYLVCGEQENQMDKAR		SCG1	33438/367830	0.952	76162	0.779	-0.115220	-1.9833	0.1667	-2	18	-0.8222	-2	0	0	0
HIP_76163	CCTSICSLY	GGEDWNPVE	CCTSICSLYGGEDWNPVE		SCG2	10111/334890	0.999	76163	0.779	0.089989	-0.4333	0.5	-3	18	-0.05	-3	1	0	0
HIP_76164	LGGGPGAGS	QMAYENLND	LGGGPGAGSQMAYENLND		SCG2	6914/334890	0.999	76164	0.779	0.027952	0.1333	0.3333	-2	18	-0.5611	-2	1	0	1
HIP_76165	AEDLQVGQV	GAPGKWQQQ	AEDLQVGQVGAPGKWQQQ		SCG1	10466/367830	0.998	76165	0.779	-0.089876	0.0167	0.5	-1	18	-0.9056	-1	0	0	1
HIP_76166	CCTSICSLY	GKPGAEEAQ	CCTSICSLYGKPGAEEAQ		CMGA	13554/247050	0.990	76166	0.779	0.100826	-0.7	0.5	-1	18	-0.0556	-1	1	0	0
HIP_76167	CCTSICSL	FQGLFRGGK	CCTSICSLFQGLFRGGK		CMGA	16189/247050	0.984	76167	0.779	0.139204	0	0.5	2	17	0.3176	2	1	1	0
HIP_76168	EDLQVGQVE	ALEYIENLR	EDLQVGQVEALEYIENLR		SCG2	19441/334890	0.996	76168	0.779	-0.133344	-0.1167	0	-4	18	-0.45	-4	1	0	0
HIP_76169	CTSICSLYQ	QLDQLLHYR	CTSICSLYQQLDQLLHYR		SCG1	42494/367830	0.904	76169	0.779	0.047784	-0.7	0	1	18	-0.1056	0	1	0	0
HIP_76170	ERGFFYTPK	QNGERATR	ERGFFYTPKQNGERATR		SCG2	32649/334890	0.988	76170	0.779	-0.043156	-2.2667	0.3333	2	17	-1.7118	2	2	1	0
HIP_76171	PLALEGSL	FLIVLSVAL	PLALEGSLFLIVLSVAL		IAPP	1680/45018	0.993	76171	0.779	0.081564	2.2833	0.3333	-1	17	2.0588	-1	1	1	0
HIP_76172	QHLCGSHLV	NMNDNFLEG	QHLCGSHLVNMNDNFLEG		SCG1	27784/367830	0.973	76172	0.779	0.175598	-0.05	0	0	18	-0.3444	-2	0	0	0
HIP_76173	CGERGFFYT	LLLCAGQV	CGERGFFYTLLLCAGQV		CMGA	17142/247050	0.981	76173	0.779	-0.040375	2.0333	0	0	17	0.7824	0	3	1	1
HIP_76174	ERGFFYTP	LALQGAKER	ERGFFYTPLALQGAKER		CMGA	14861/247050	0.987	76174	0.779	0.036111	0.9667	0.1667	1	17	-0.6471	1	2	1	0
HIP_76175	LYQLENYCN	PPITPECRQ	LYQLENYCNPPITPECRQ		SCG1	15594/367830	0.994	76175	0.779	-0.064785	-0.1667	0.3333	-1	18	-0.9167	-1	1	0	0
HIP_76176	QHLCGSHLV	TNEIVEEQY	QHLCGSHLVTNEIVEEQY		SCG2	22049/334890	0.995	76176	0.779	0.156374	-0.4833	0	-1	18	-0.4222	-3	0	0	0
HIP_76177	GGGPGAGSL	YQLIEISRN	GGGPGAGSLYQLIEISRN		SCG2	11732/334890	0.998	76177	0.779	0.034925	0.2667	0.3333	0	18	-0.1722	0	1	0	1
HIP_76178	GIVEQCCTS	YQGVSVPLQ	GIVEQCCTSYQGVSVPLQ		SCG2	20139/334890	0.996	76178	0.779	-0.093017	-0.7	0.3333	-1	18	0.3278	-1	1	0	0
HIP_76179	CGERGFFY	SAPPITPE	CGERGFFYSAPPITPE		SCG1	23186/367830	0.984	76179	0.779	0.157986	0.6167	0.3333	-1	16	-0.3438	-1	3	0	0
HIP_76180	HLVEALYL	GIQEEDLR	HLVEALYLGIQEEDLR		SCG2	16327/334890	0.997	76180	0.779	0.176459	1.15	0.1667	-2	16	-0.075	-3	1	0	0
HIP_76181	LQVGQVELG	KRFPVGPPK	LQVGQVELGKRFPVGPPK		SCG2	44794/334890	0.964	76181	0.779	0.031536	-0.95	0.1667	2	18	-0.3222	2	1	0	0
[illegible]	[illegible]	[illegible]	[illegible]	[illegible]	[illegible]	[illegible]	[illegible]	[illegible]	[illegible]	[illegible]	[illegible]	[illegible]	[illegible]	[illegible]	[illegible]	[illegible]	[illegible]	[illegible]	[illegible]

HIP_76640	GSLQPLAL	MAYENLNDK	GSLQPLALMAYENLNDK		SCG2	28750/334890	0.991	76640	0.779	0.026268	1.9667	0	-1	17	-0.2824	-1	1	1	0
HIP_76641	ICSLYQLEN	AKTHWLGAA	ICSLYQLENAKTHWLGAA		SCG2	42241/334890	0.972	76641	0.779	-0.024096	-1	0	1	18	0.1167	0	1	0	0
HIP_76642	ICSLYQLEN	RGWRPSSRE	ICSLYQLENRGWRPSSRE		CMGA	28718/247050	0.925	76642	0.779	0.038493	-1.5	0.1667	1	18	-1.0833	1	2	0	0
HIP_76643	GPGAGSLQ	EQEEERLSK	GPGAGSLQEQEEERLSK		CMGA	23362/247050	0.959	76643	0.779	-0.004115	-1.8333	0.1667	-2	17	-1.4353	-2	0	1	1
HIP_76644	GPGAGSLQ	QEEERLSKE	GPGAGSLQQEEERLSKE		CMGA	23363/247050	0.959	76644	0.779	-0.004115	-1.8333	0.1667	-2	17	-1.4353	-2	0	1	1
HIP_76645	GSLQPLAL	QKIAEKFSQ	GSLQPLALQKIAEKFSQ		SCG1	42782/367830	0.902	76645	0.779	0.198407	1.0833	0	1	17	-0.1824	1	0	1	0
HIP_76646	AGSLQPLAL	ESRSEALA	AGSLQPLALESRSEALA		CMGA	21947/247050	0.965	76646	0.779	0.242327	0.1	0.1667	-1	17	0.1765	-1	0	1	0
HIP_76647	VEALYLVC	NRQMAYENL	VEALYLVCNRQMAYENL		SCG2	35089/334890	0.985	76647	0.779	0.213254	-0.1667	0	-1	17	0.1882	-1	1	1	0
HIP_76648	ERGFFYTP	EGEEENELT	ERGFFYTPEGEEENELT		SCG1	19047/367830	0.991	76648	0.779	-0.067340	-1.8333	0.3333	-5	17	-1.4529	-5	2	1	1
HIP_76649	EDLQVGQVE	KEEEEEMAV	EDLQVGQVEKEEEEEMAV		CMGA	21132/247050	0.968	76649	0.779	-0.093486	-2.2833	0	-7	18	-1.0667	-7	0	0	3
HIP_76650	VNQHLCGSH	GRGSEEYRA	VNQHLCGSHGRGSEEYRA		SCG1	26678/367830	0.976	76650	0.779	-0.041332	-1.6167	0.6667	2	18	-1.1778	0	0	0	0
HIP_76651	CSLYQLENY	DLQEEEQIE	CSLYQLENYDLQEEEQIE		SCG2	22949/334890	0.995	76651	0.779	-0.127737	-1.9167	0	-6	18	-1.1111	-6	1	0	1
HIP_76652	PGAGSLQPL	DDTPNRQYW	PGAGSLQPLDDTPNRQYW		SCG2	29637/334890	0.990	76652	0.779	-0.015600	-1.5	0.1667	-1	18	-1.2167	-1	0	0	0
HIP_76653	VEQCCTSIC	ENLRQQAHK	VEQCCTSICENLRQQAHK		SCG2	29231/334890	0.991	76653	0.779	-0.003743	0.5	0.1667	1	18	-0.6833	0	0	0	0
HIP_76654	TSICSLYQL	PSDSQVSEE	TSICSLYQLPSDSQVSEE		SCG1	18482/367830	0.991	76654	0.779	0.140672	-1.15	0.3333	-3	18	-0.35	-3	1	0	0
HIP_76655	HLCGSHLVE	ASLGEKRDH	HLCGSHLVEASLGEKRDH		SCG1	32963/367830	0.953	76655	0.779	-0.007059	1.55	0.1667	2	18	-0.6111	-1	0	0	0
HIP_76656	QHLCGSHLV	EEEEEEAT	QHLCGSHLVEEEEEEAT		CMGA	15802/247050	0.985	76656	0.779	0.101884	-0.95	0	-4	17	-0.9824	-6	0	1	4
HIP_76657	SICSLYQLE	LQVFLIVLS	SICSLYQLELQVFLIVLS		IAPP	2777/45018	0.981	76657	0.779	-0.102041	0.2167	0	-1	18	1.5278	-1	2	0	0
HIP_76658	HLCGSHLVE	GKSGELEQE	HLCGSHLVEGKSGELEQE		CMGA	19062/247050	0.975	76658	0.779	0.166747	-0.1	0.3333	-1	18	-0.6944	-3	0	0	0
HIP_76659	LQPLALEGS	RSSQGGSLP	LQPLALEGSRSSQGGSLP		SCG1	32262/367830	0.957	76659	0.779	-0.083425	-1.8	0.6667	0	18	-0.3111	0	0	0	0
HIP_76660	TSICSLYQ	EEPSSKDVM	TSICSLYQEEPSSKDVM		CMGA	18568/247050	0.977	76660	0.779	0.134321	-1.6	0.1667	-2	17	-0.4588	-2	1	1	0
HIP_76661	VELGGGPGA	SQREDEEEE	VELGGGPGASQREDEEEE		SCG1	17910/367830	0.992	76661	0.779	-0.099265	-1.5	0.5	-6	18	-1.4833	-6	0	0	3
HIP_76662	CGERGFF	PGEEEEEEE	CGERGFFPGEEEEEEE		CMGA	9904/247050	0.995	76662	0.779	0.053058	-0.05	0.5	-7	16	-1.7	-7	2	2	5
HIP_76663	LQVGQVEL	PRAYFMSDT	LQVGQVELPRAYFMSDT		SCG1	38668/367830	0.926	76663	0.779	0.077083	0.0333	0.1667	-1	17	-0.0471	-1	2	1	0
HIP_76664	RGFFYTPKT	GAKERAHQQ	RGFFYTPKTGAKERAHQQ		CMGA	26368/247050	0.942	76664	0.779	0.008767	-1.45	0.3333	4	18	-1.4667	3	1	0	0
HIP_76665	CGSHLVEAL	LYTDDEDDI	CGSHLVEALLYTDDEDDI		SCG2	17346/334890	0.997	76665	0.779	-0.127234	0.65	0	-5	18	-0.1667	-6	1	0	0
HIP_76666	GERGFFY	GQEEEED	GERGFFYGQEEEED		CMGA	16047/247050	0.984	76666	0.779	0.164929	-0.5167	0.1667	-5	14	-1.85	-5	3	0	2
HIP_76667	GERGFFY	EGQEEEE	GERGFFYEGQEEEE		CMGA	16048/247050	0.984	76667	0.779	0.164929	-0.5167	0.1667	-5	14	-1.85	-5	3	0	2
HIP_76668	LYQLENYCN	GSVEPEREL	LYQLENYCNGSVEPEREL		SCG2	8342/334890	0.999	76668	0.779	-0.070386	0.1167	0.3333	-3	18	-0.9056	-3	1	0	0
HIP_76669	GAGSLQPLA	LSLLVCLGA	GAGSLQPLALSLLVCLGA		NPY	3185/49410	0.973	76669	0.779	-0.039649	1.8	0.3333	0	18	1.5	0	0	0	0
HIP_76670	QHLCGSHL	SEEYRAPRP	QHLCGSHLSEEYRAPRP		SCG1	38673/367830	0.926	76670	0.779	0.134755	-1.3333	0.3333	2	17	-1.2059	0	1	1	0
HIP_76671	CTSICSLYQ	SMPVDNRNH	CTSICSLYQSMPVDNRNH		SCG1	20941/367830	0.988	76671	0.779	-0.025030	-0.25	0.3333	1	18	-0.4611	0	1	0	0
HIP_76672	VELGGGPGA	FVNQHLCGS	VELGGGPGAFVNQHLCGS		INSB	54/4968	0.997	76672	0.779	0.021790	0.55	0.3333	0	18	0.2778	-1	1	0	1
HIP_76673	EALYLVCG	EEEEEEEEE	EALYLVCGEEEEEEEEE		CMGA	12016/247050	0.992	76673	0.779	-0.043371	-0.7	0.1667	-10	17	-1.2118	-10	0	1	7
HIP_76674	CSLYQLENY	LSQGLVDRE	CSLYQLENYLSQGLVDRE		CMGA	16378/247050	0.984	76674	0.779	-0.185864	-1.4667	0.1667	-2	18	-0.4556	-2	1	0	0
HIP_76675	LCGSHLVEA	EEKKEEEGS	LCGSHLVEAEEKKEEEGS		CMGA	25681/247050	0.946	76675	0.779	0.145110	-1.4	0	-3	18	-1.0167	-4	0	0	1
HIP_76676	VCGERGFF	TLALSLLVC	VCGERGFFTLALSLLVC		NPY	2743/49410	0.979	76676	0.779	-0.017188	1.6833	0.1667	0	17	1.5118	0	2	1	0
HIP_76677	EDLQVGQVE	KRFPVGPPK	EDLQVGQVEKRFPVGPPK		SCG2	45840/334890	0.960	76677	0.779	-0.054454	-1.4	0	0	18	-0.9	0	1	0	0
HIP_76678	HLVEALYL	WLGAALSLI	HLVEALYLWLGAALSLI		SCG2	18807/334890	0.996	76678	0.779	0.036613	1.4667	0.1667	0	17	1.5765	-1	2	1	0
HIP_76679	HLVEALYLV	LARVPKLDL	HLVEALYLVLARVPKLDL		SCG1	32928/367830	0.954	76679	0.779	0.026771	1.3	0	1	18	0.9722	0	1	0	0
HIP_76680	QPLALEGSL	LSFRARAYG	QPLALEGSLLSFRARAYG		CMGA	9585/247050	0.995	76680	0.779	0.014424	1.4	0.5	1	18	0.1167	1	1	0	0
HIP_76681	GAGSLQPLA	AAMDLELQK	GAGSLQPLAAAMDLELQK		SCG1	19472/367830	0.990	76681	0.779	-0.008054	1.5833	0.1667	-1	18	0.1778	-1	0	0	1
HIP_76682	HLVEALYLV	GTEALPDG	HLVEALYLVGTEALPDG		SCG2	18726/334890	0.997	76682	0.779	0.190513	0.35	0.1667	-2	17	0.5353	-3	1	1	0
HIP_76683	CGERGFFYT	LDDISEADL	CGERGFFYTLDDISEADL		SCG2	23352/334890	0.995	76683	0.779	-0.051205	-0.4	0	-4	18	-0.2	-4	3	0	0
HIP_76684	ICSLYQLEN	LPLQVRGYP	ICSLYQLENLPLQVRGYP		CMGA	13374/247050	0.990	76684	0.779	-0.084737	0.4667	0.1667	0	18	0.05	0	1	0	0
HIP_76685	AGSLQPLAL	LQKEPDLR	AGSLQPLALLQKEPDLR		SCG2	35890/334890	0.984	76685	0.779	0.174380	0.9667	0	0	17	-0.2471	0	0	1	0
HIP_76686	VEALYLVCG	EQINDEMKR	VEALYLVCGEQINDEMKR		SCG2	18610/334890	0.997	76686	0.779	-0.029492	0.6333	0.1667	-2	18	-0.2444	-2	1	0	0
HIP_76687	SICSLYQL	EENELTLNE	SICSLYQLEENELTLNE		SCG1	28115/367830	0.972	76687	0.779	-0.003340	-1.9167	0	-4	17	-0.3471	-4	1	1	0
HIP_76688	QHLCGSHLV	ARVPKLDLK	QHLCGSHLVARVPKLDLK		SCG1	44261/367830	0.893	76688	0.779	0.086367	1.05	0	4	18	-0.0333	2	0	0	0
HIP_76689	VGQVELGGG	YEENSRDNP	VGQVELGGGYEENSRDNP		SCG2	13652/334890	0.998	76689	0.779	0.032387	-1.5833	0.5	-3	18	-1.2278	-3	1	0	1
HIP_76690	VNQHLCGSH	GLFRGGKSG	VNQHLCGSHGLFRGGKSG		CMGA	15409/247050	0.986	76690	0.779	-0.000290	0.3	0.5	4	18	-0.4611	2	1	0	0
HIP_76691	SLYQLENYC	ILRHQNLLK	SLYQLENYCILRHQNLLK		CMGA	22948/247050	0.961	76691	0.779	-0.073522	0.25	0	2	18	-0.3611	1	1	0	0
HIP_76692	ERGFFYTP	NPYQGVSVP	ERGFFYTPNPYQGVSVP		SCG2	27281/334890	0.992	76692	0.779	-0.058562	-1.6667	0.3333	0	17	-0.6294	0	3	1	0
HIP_76693	QCCTSICSL	ATNTHPPAS	QCCTSICSLATNTHPPAS		CMGA	21721/247050	0.966	76693	0.779	0.047962	0.5167	0.1667	1	18	0.0833	0	0	0	0
[illegible]																			

HIP_77151	VCGERGFF	QIPPEDLIE	VCGERGFFQIPPEDLIE		SCG2	12535/334890	0.998	77151	0.778	-0.054066	0.7667	0.3333	-3	17	-0.0529	-3	2	1	0	0.000000	-1.9333	0	0.0333	1.825000	0.2222	1.6028	1	1	1	1	0	1.2288	-3.8667	1	0	0	0	0.4333	0.9433	0.945	0.5	0.1878	0.893	0.3222	0.0011	-0.0644
HIP_77152	CGERGFFY	AELKEAVE	CGERGFFYAELKEAVE		CMGA	19463/247050	0.974	77152	0.778	0.015182	1.0667	0	-2	16	-0.3	-2	3	0	0	0.000000	-0.7333	-1	0.05	1.150000	0.3333	0.8167	0	0	0	0	0.7333	0.8711	-2.2	0	1	0	0	0.3051	1.2803	0.6678	0.5	0.0931	0.446	0.3255	0.0025	-0.0367
HIP_77153	VELGGGPGA	DTREEKRFL	VELGGGPGADTREEKRFL		SCG1	28379/367830	0.971	77153	0.778	-0.054009	-1.4833	0.3333	-1	18	-0.8	-1	0	0	1	0.000000	-2.8333	0	0	0.000000	0.3333	-0.3333	1	1	1	1	0	0.4944	0	1	0	0	0	0.5103	0.8988	1.0756	-0.1667	0.2604	1.157	-0.7569	0	0
HIP_77154	YLVCGERGF	MRIILEALR	YLVCGERGFMRIILEALR		SCG2	21066/334890	0.996	77154	0.778	0.035081	-0.0333	0.1667	1	18	0.6111	1	1	0	0	1.000000	1.3333	0	-0.0833	1.600000	0.3333	1.2667	0	1	0	1	0	-0.0422	1.3333	1	0	0	0	0.3603	1.0038	0.8306	0.1667	0.1298	0.69	-0.012	0.0069	-0.1111
HIP_77155	VGQVELGGG	QVFLIVLSV	VGQVELGGGQVFLIVLSV		IAPP	1077/45018	0.996	77155	0.778	0.126220	0.3833	0.5	-1	18	1.4889	-1	1	0	1	0.000000	1.5667	0	-0.2	0.950000	0.1111	0.8389	0	3	0	1	0	0.3216	1.5667	0	0	0	1	0.5167	0.8117	1.0967	0.3333	0.2669	1.2027	0.1981	0.04	-0.3133
HIP_77156	CTSICSLY	KFEVRLLRD	CTSICSLYKFEVRLLRD		SCG1	65959/367830	0.881	77156	0.778	0.064929	-0.4833	0.1667	1	17	0.2588	1	2	1	0	0.000000	-2.1	0	0.0333	1.325000	0.3333	0.9917	0	0	0	0	0	-0.4793	-4.2	0	1	0	0	0.4103	1.1422	0.809	0	0.1683	0.6544	-0.1983	0.0011	-0.07
HIP_77157	RGFFYTPK	GENYQKGER	RGFFYTPKGENYQKGER		SCG1	38178/367830	0.929	77157	0.778	-0.012128	-2.2667	0.3333	2	17	-1.8412	2	2	1	0	0.000000	-0.4	-2	0.0167	0.000000	0.2222	-0.2222	1	1	1	1	0.8	0.5037	-0.8	0	0	1	0	0.6051	0.8503	1.1978	-0.3333	0.3662	1.4348	-1.3716	0.0003	-0.0067
HIP_77158	VEQCCTSIC	QPRSQEESE	VEQCCTSICQPRSQEESE		SCG1	28584/367830	0.970	77158	0.778	0.070961	-0.5667	0.3333	-3	18	-0.9722	-3	0	0	0	0.000000	-5.2667	1	0.1	1.750000	0.1111	1.6389	1	0	1	1	-5.2667	-0.9287	0	0	0	0	0	0.4936	0.9338	0.9971	0	0.2436	0.9946	-0.2797	0.01	-0.5267
HIP_77159	QHLCGSHLV	EDILNLLGM	QHLCGSHLVEDILNLLGM		SCG2	24654/334890	0.994	77159	0.778	0.089811	0.3833	0	0	18	0.5611	-2	0	0	0	0.000000	-2.4333	-2	0.1	1.900000	0.2222	1.6778	0	0	0	0	4.8667	0.6431	0	1	0	0	0	0.3436	1.1655	0.6706	0	0.1181	0.4498	0.1317	0.01	-0.2433
HIP_77160	GERGFFYT	GERATRLFE	GERGFFYTGERATRLFE		SCG2	32498/334890	0.988	77160	0.778	0.163942	-1.2667	0.1667	0	17	-0.8176	0	3	1	0	0.000000	-3.0667	0	0.1333	0.700000	0.2222	0.4778	0	1	0	1	0	-0.6052	-9.2	0	1	0	0	0.4936	1.0138	0.9356	-0.1667	0.2436	0.8754	-0.6252	0.0178	-0.4089
HIP_77161	CGERGFFYT	RGDERILS	CGERGFFYTRGDERILS		CMGA	24442/247050	0.953	77161	0.778	0.181094	-1.2667	0.1667	0	17	-0.6824	0	3	1	0	0.000000	-3.0667	0	0.1333	0.000000	0.3333	-0.3333	0	1	0	1	0	0.4222	-9.2	0	1	0	0	0.4654	0.9324	0.9635	-0.1667	0.2166	0.9283	-0.5895	0.0178	-0.4089
HIP_77162	YLVCGERGF	EENELTLNE	YLVCGERGFEENELTLNE		SCG1	26551/367830	0.976	77162	0.778	-0.032567	-2.1	0.1667	-4	18	-0.6056	-4	1	0	0	0.000000	-2.8	-3	0.0167	0.000000	0.5556	-0.5556	0	1	0	1	8.4	1.1667	-2.8	1	0	0	0	0.5218	1.0686	1.0078	-0.3333	0.2723	1.0157	-1.0958	0.0003	-0.0467
HIP_77163	ICSLYQLEN	RLQNGQNGE	ICSLYQLENRLQNGQNGE		SCG2	42397/334890	0.971	77163	0.778	-0.020717	-1.2333	0	-1	18	-0.9444	-1	1	0	0	0.000000	-0.3333	2	0	0.000000	0.2222	-0.2222	0	0	0	0	-0.6667	0.2741	-0.3333	0	1	0	0	0.4385	1.1519	0.8645	0	0.1922	0.7473	-0.5408	0	0
HIP_77164	CGERGFFYT	QAEGDSEG	CGERGFFYTQAEGDSEG		CMGA	22675/247050	0.962	77164	0.778	0.043360	-0.7333	0	-3	17	-0.9706	-3	3	1	0	0.000000	-2	-1	0.05	0.000000	0.1111	-0.1111	0	0	0	0	2	0.0815	-6	0	1	0	0	0.4385	1.1869	0.8078	0.1667	0.1922	0.6526	-0.3215	0.0025	-0.1
HIP_77165	GIVEQCCT	EKNFFPEYN	GIVEQCCTEKNFFPEYN		SCG1	38388/367830	0.927	77165	0.778	0.130924	-1.1	0	-2	17	-0.5647	-2	2	1	0	0.000000	-5.0667	0	0.1	0.700000	0.2222	0.4778	0	0	0	0	0	-0.5256	-10.1333	0	1	0	0	0.5154	1.0124	0.9968	-0.3333	0.2656	0.9936	-0.5669	0.01	-0.5067
HIP_77166	SICSLYQL	GYGEESEEE	SICSLYQLGYGEESEEE		SCG1	5605/367830	0.999	77166	0.778	-0.030525	-0.5167	0.3333	-5	17	-0.7176	-5	2	1	1	0.000000	-0.3667	0	0.1167	1.900000	0.2222	1.6778	0	2	0	1	0	-0.8669	-0.7333	1	0	0	0	0.5103	0.9088	1.049	0.1667	0.2604	1.1003	-0.2636	0.0136	-0.0428
HIP_77167	RGFFYTPKT	SEESWDEED	RGFFYTPKTSEESWDEED		SCG1	29721/367830	0.966	77167	0.778	-0.099160	-2.3333	0.3333	-4	18	-1.7222	-4	1	0	0	0.000000	-0.5333	-3	0	0.000000	0.3333	-0.3333	1	0	1	0	1.6	0.7778	-0.5333	0	1	0	0	0.5885	1.0403	1.0112	-0.5	0.3463	1.0224	-1.3731	0	0
HIP_77168	SLYQLENYC	DKQYSSHH	SLYQLENYCDKQYSSHH		SCG1	44391/367830	0.892	77168	0.778	0.141410	-2.2	0	1	17	-1.4118	-1	2	1	0	0.000000	-2.8667	0	0.1	0.950000	0.3333	0.6167	0	0	0	0	0	-1.3567	-5.7333	0	0	0	0	0.4872	0.9777	1.0279	-0.3333	0.2373	1.0567	-1.0718	0.01	-0.2867
HIP_77169	HLCGSHLVE	MIRALEYIE	HLCGSHLVEMIRALEYIE		SCG2	31638/334890	0.989	77169	0.778	-0.028671	1.0667	0	0	18	0.3833	-2	0	0	0	0.000000	-0.8667	2	0	1.975000	0.2222	1.7528	0	0	0	0	-1.7333	1.8696	0	0	0	1	0	0.3051	1.1953	0.6195	0.3333	0.0931	0.3838	0.3255	0	0
HIP_77170	VEALYLVCG	DGAGKPGAE	VEALYLVCGDGAGKPGAE		CMGA	8696/247050	0.996	77170	0.778	0.001936	0.7	0.3333	-2	18	0.2778	-2	1	0	0	0.000000	-2.8	-1	0.05	0.625000	0.1111	0.5139	0	2	0	1	2.8	0.3597	-2.8	0	0	0	1	0.4436	0.9255	0.9956	0.1667	0.1968	0.9913	0.3105	0.0025	-0.14
HIP_77171	ERGFFYTP	EGGGHSRER	ERGFFYTPEGGGHSRER		SCG1	21177/367830	0.988	77171	0.778	0.009063	-1.3167	0.5	1	17	-1.6235	0	2	1	1	0.000000	-0.2333	-1	0.1	0.700000	0.1111	0.5889	1	2	1	1	0.2333	-0.7754	-0.4667	0	0	0	1	0.6218	0.8219	1.1878	-0.1667	0.3866	1.4109	-0.8187	0.01	-0.0233
HIP_77172	LVEALYLV	REEARFQD	LVEALYLVREEARFQD		SCG1	32117/367830	0.957	77172	0.778	0.179108	-0.8	0	-2	16	-0.1	-2	1	0	0	0.000000	-6.0667	-1	0.15	1.850000	0.3333	1.5167	0	0	0	0	6.0667	-1.2133	-6.0667	1	0	0	0	0.4103	1.2372	0.7156	-0.1667	0.1683	0.5121	-0.3282	0.0225	-0.91
HIP_77173	EQCCTSICS	NRMLSKSGY	EQCCTSICSNRMLSKSGY		SCG2	41841/334890	0.973	77173	0.778	0.039066	0.0167	0.1667	1	18	-0.3778	1	0	0	0	0.000000	-4.1	0	0.0333	1.575000	0.1111	1.4639	0	0	0	0	-4.1	0.0244	0	0	1	0	0	0.4103	0.9655	0.9406	0.1667	0.1683	0.8848	0.0068	0.0011	-0.1367
HIP_77174	FVNQHLCGS	TNTHPPASL	FVNQHLCGSTNTHPPASL		CMGA	19933/247050	0.972	77174	0.778	-0.041452	-0.6	0.3333	2	18	-0.2556	0	0	0	0	0.000000	-2.0667	0	0	0.000000	0	0	0	1	0	1	0	0	0	0	1	0	0	0.5885	0.7753	1.1812	0	0.3463	1.3951	-0.3531	0	0
HIP_77175	ERGFFYTP	SKEWEDSKR	ERGFFYTPSKEWEDSKR		CMGA	27270/247050	0.936	77175	0.778	0.022744	-1.9667	0.3333	0	17	-1.8647	0	3	1	0	0.000000	-1.5333	0	0.05	0.700000	0.3333	0.3667	1	0	1	0	0	-0.7211	-4.6	0	0	0	1	0.5603	0.9588	1.039	-0.3333	0.3139	1.0795	-1.1018	0.0025	-0.0767
HIP_77176	YQLENYCN	DPEGKGEQE	YQLENYCNDPEGKGEQE		CMGA	16347/247050	0.984	77176	0.778	0.082406	-1.8167	0.1667	-4	17	-2.0059	-4	1	1	0	0.000000	-2.1	-2	0.1167	0.000000	0.3333	-0.3333	1	0	1	0	4.2	0.6056	-2.1	0	1	0	0	0.5321	0.9691	1.0901	-0.3333	0.2831	1.1884	-0.9666	0.0136	-0.245
HIP_77177	VNQHLCGSH	YLARMLVKY	VNQHLCGSHYLARMLVKY		SCG2	21308/334890	0.996	77177	0.778	0.150701	-0.0167	0.3333	4	18	0.0222	2	1	0	0	0.000000	2.9	0	-0.15	0.950000	0.1111	0.8389	0	1	0	1	0	-0.014	2.9	0	0	1	0	0.4269	1.0155	0.9456	0.1667	0.1823	0.8942	-0.0071	0.0225	-0.435
HIP_77178	LYLVCGERG	DTEVMKCIV	LYLVCGERGDTEVMKCIV		CMGA	25380/247050	0.948	77178	0.778	-0.097155	-2.6833	0.1667	-1	18	0.55	-1	0	0	0	0.000000	0.2333	-2	-0.0333	1.675000	0.4444	1.2306	0	1	0	1	-0.4667	-3.302	0	0	0	0	1	0.5603	1.0655	0.959	-0.6667	0.3139	0.9196	-1.5034	0.0011	-0.0078
HIP_77179	CGSHLVEAL	DKDQELGEY	CGSHLVEALDKDQELGEY		SCG2	32564/334890	0.988	77179	0.778	0.101649	-1.4667	0	-3	18	-0.6167	-4	0	0	0	0.000000	-4.3333	0	0.1	1.400000	0.4444	0.9556	0	0	0	0	0	-1.4015	0	1	0	0	0	0.3821	1.2208	0.7701	-0.3333	0.146	0.5931	-0.5603	0.01	-0.4333
HIP_77180	SICSLYQL	GKGRHHRGR	SICSLYQLGKGRHHRGR		SCG1	34088/367830	0.949	77180	0.778	0.180655	-0.95	0.3333	6	17	-0.9882	4	1	1	0	0.000000	-1.2333	1	0.1667	1.900000	0.2222	1.6778	0	2	0	1	-1.2333	-1.5939	-1.2333	1	0	0	0	0.5103	0.9088	1.049	0	0.2604	1.1003	-0.4847	0.0278	-0.2056
HIP_77181	LCGSHLVEA	SLPSQKYPG	LCGSHLVEASLPSQKYPG		CMGA	19125/247050	0.975	77181	0.778	0.103450	0.65	0.3333	1	18	-0.1056	0	0	0	0	0.000000	-0.3667	1	0.0833	0.000000	0.1111	-0.1111	1	0	1	0	-0.3667	-0.0722	0	1	0	0	0	0.4167	1.115	0.8533	0.3333	0.1736	0.7282	0.2708	0.0069	-0.0306
HIP_77182	VCGERGFF	PADASEAHE	VCGERGFFPADASEAHE		SCG1	20314/367830	0.989	77182	0.778	-0.007634	0.3167	0.3333	-2	17	-0.4235	-3	2	1	0	0.000000	-2.8333	-1	0.0333	1.150000	0.2222	0.9278	1	1	1	1	2.8333	0.2938	-5.6667	1	0	0	0	0.4051	0.9903	0.9645	0.3333	0.1641	0.9302	0.1283	0.0011	-0.0944
HIP_77183	LCGSHLVEA	PDFYDSEE	LCGSHLVEAPDFYDSEE		SCG1	23501/367830	0.983	77183	0.778	0.025610	0.0333	0.1667	-4	17	-0.3941	-5	2	1	0	0.000000	-1.6	0	0.0667	0.000000	0.2222	-0.2222	1	0	1	0	0	-0.0074	-3.2	1	0	0	0	0.3885	1.1353	0.8212	0.1667	0.1509	0.6743	0.0129	0.0044	-0.1067
HIP_77184	FVNQHLCGS	HSGFEDELS	FVNQHLCGSHSGFEDELS		CMGA	11142/247050	0.993	77184	0.778	-0.110534	-0.5167	0.6667	-1	18	-0.4	-3	1	0	0	0.000000	-1.9	0	0	0.700000	0	0.7	0	2	0	1	0	-0.3617	-1.9	0	1	0	0	0.5769	0.7738	1.2023	-0.1667	0.3328	1.4455	-0.2981	0	0
HIP_77185	GQVELGG	ERGFFYTPK	GQVELGGERGFFYTPK		INSB	445/4968	0.905	77185	0.778	0.028372	-0.9	0.5	0	16	-0.6562	0	2	2	0	0.000000	-3.8	0	0.0167	0.700000	0.3333	0.3667	0	3	0	1	0	-0.33	-7.6	0	0	0	1	0.5551	0.8886	1.1078	-0.1667	0.3082	1.2273	-0.4996	0.0003	-0.0633
HIP_77186	QPLALEGSL	FQRNQLLQK	QPLALEGSLFQRNQLLQK		SCG2	31528/334890	0.989	77186	0.778	-0.016353	-0.4333	0.3333	1	18	-0.4778	1	1	0	0	1.000000	-2.6	1	-0.05	1.650000	0.2222	1.4278	0	1	0	1	-2.6	-0.6187	-2.6	1	0	0	0	0.4551	0.9886	0.9478	0.1667	0.2071	0.8984	-0.1972	0.0025	0.12
HIP_77187	VEALYLVC	QIPPEDLIE	VEALYLVCQIPPEDLIE		SCG2	16682/334890	0.997	77187	0.778	0.112876	1.65	0.1667	-4	17	0.6529	-4	1	1	0	0.000000	-3.7	0	0.1167	1.750000	0.1111	1.6389	1	0	1	0	0	2.7042	-3.7	0	0	0	0	0.3885	1.0236	0.8128	0.5	0.1509	0.6607	0.641	0.0136	-0.4317
HIP_77188	CGERGFFY	RMLVKYPE	CGERGFFYRMLVKYPE		SCG2	34695/334890	0.985	77188	0.778	0.061081	0.9167	0	1	16	-0.4313	1	3	0	0	0.000000	-1.0333	1	0.05	1.750000	0.2222	1.5278	0	0	0	0	-1.0333	1.4005	-3.1	0	1	0	0	0.2769	1.1572	0.6856	0.5	0.0767	0.4701	0.2538	0.0025	-0.0517
HIP_77189	CGERGFFY	LNPHPSLG	CGERGFFYLNPHPSLG		CMGA	18435/247050	0.977	77189	0.778	0.134822	0.5	0.1667	1	16	-0.3438	0	3	0	0	0.000000	-1.8667	0	0.1167	1.650000	0.1111	1.5389	1	0	1	0	0	0.7694	-5.6	0	1	0	0	0.3885	0.9936	0.9478	0.5	0.1509	0.8984	0.1942	0.0136	-0.2178
HIP_77190	LYQLENYCN	YNPYQGVS	LYQLENYCNYNPYQGVS		SCG2	27720/334890	0.992	77190	0.778	0.018008	-1.45	0.1667	-1	17	-0.8647	-1	3	1	0	0.000000	-1.3667	0	0.0667	0.625000	0.1111	0.5139	1	0	1	0	0	-0.7451	-4.1	0	1	0	0	0.5321	0.8291	1.2268	0	0.2831	1.505	-0.7715	0.0044	-0.0911
HIP_77191	NQHLCGSHL	NEPQSAPKE	NQHLCGSHLNEPQSAPKE		SCG2	26341/334890	0.993	77191	0.778	0.102435	-1.4667	0.3333	1	18	-1.3667	-1	0	0	0	0.000000	-2.8	-1	0.1	0.625000	0.1111	0.5139	1	0	1	0	2.8	-0.7537	0	1	0	0	0	0.5218	0.9786	1.0712	-0.1667	0.2723	1.1474	-0.7653	0.01	-0.28
HIP_77192	CCTSICSLY	KRRTRPRHH	CCTSICSLYKRRTRPRHH		SCG1	54256/367830	0.815	77192	0.778	0.111873	-1.8667	0.1667	7	18	-1.0222	5	1	0	0	0.000000	-4.8667	3	0.0833	2.075000	0.3333	1.7417	0	0	0	0	-14.6	-3.2511	-4.8667	0	1	0	0	0.4205	1.031	0.8879	-0.3333	0.1768	0.7885	-0.785	0.0069	-0.4056
HIP_77193	GAGSLQPLA	SSHHTAEKR	GAGSLQPLASSHHTAEKR		SCG1	31748/367830	0.959	77193	0.778	0.040987	-0.1333	0.5	3	18	-0.7944	1	0	0	0	0.000000	-2.9333	0	0.0667	1.900000	0	1.9	1	0	1	0	0	-0.2533	0	1	0	0	0	0.4551	0.9903	0.9978	0.1667	0.2071	0.9956	-0.0607	0.0044	-0.1956

HIP_77662	EALYLVCGE	DEPQWSLYP	EALYLVCGEDEPQWSLYP		SCG1	21181/367830	0.988	77662	0.778	-0.134493	-1.6667	0.3333	-4	18	-0.3056	-4	0	0	0	0.000000	-2.4	-1	0.0333	0.950000	0.3333	0.6167	1	1	1	1	2.4	-1.0278	0	0	0	1	0	0.5769	1.0222	1.0523	-0.5	0.3328	1.1074	-0.9615	0.0011	-0.08
HIP_77663	EAEDLQVGQ	EERGLEPGK	EAEDLQVGQEERGLEPGK		SCG1	27802/367830	0.973	77663	0.778	-0.119241	-1.8667	0.1667	-4	18	-1.4222	-4	0	0	0	0.000000	-3.9333	-1	0.0167	0.950000	0.3333	0.6167	0	1	0	1	3.9333	-1.1511	0	0	1	0	0	0.5218	1.1186	0.8945	-0.3333	0.2723	0.8001	-0.974	0.0003	-0.0656
HIP_77664	GSHLVEALY	LPSQKYPG	GSHLVEALYLPSQKYPG		CMGA	14323/247050	0.988	77664	0.778	0.106330	0.95	0.3333	1	17	-0.2882	0	1	1	0	0.000000	-0.9667	0	0.1333	2.950000	0.1111	2.8389	1	0	1	0	0	2.6969	-0.9667	0	1	0	0	0.3885	1.0736	0.8812	0.5	0.1509	0.7764	0.369	0.0178	-0.1289
HIP_77665	GERGFFYT	QAEGDSEGL	GERGFFYTQAEGDSEGL		CMGA	22091/247050	0.964	77665	0.778	0.013118	-0.7333	0	-3	17	-0.8941	-3	3	1	0	0.000000	-2	-1	0.05	1.150000	0.2222	0.9278	0	0	0	0	2	-0.6804	-6	0	1	0	0	0.4385	1.1869	0.8078	0.1667	0.1922	0.6526	-0.3215	0.0025	-0.1
HIP_77666	GSLQPLALE	VRDSKENIE	GSLQPLALEVRDSKENIE		SCG2	37431/334890	0.981	77666	0.778	-0.020850	-0.2833	0	-2	18	-0.6167	-2	0	0	0	0.000000	-1.9667	1	0.05	2.000000	0.3333	1.6667	0	0	0	0	-1.9667	-0.4722	0	0	0	1	0	0.3436	1.2272	0.7023	0	0.1181	0.4932	-0.0974	0.0025	-0.0983
HIP_77667	ERGFFYTPK	TCATQRLAN	ERGFFYTPKTCATQRLAN		IAPP	3555/45018	0.967	77667	0.778	0.055109	-0.4333	0.1667	2	18	-0.7389	2	2	0	0	0.000000	3.2667	-1	-0.1167	0.700000	0.1111	0.5889	1	0	1	0	-3.2667	-0.2552	6.5333	0	0	1	0	0.4936	0.9538	0.9856	0	0.2436	0.9715	-0.2139	0.0136	-0.3811
HIP_77668	GSHLVEALY	HTAEKRKRL	GSHLVEALYHTAEKRKRL		SCG1	45711/367830	0.883	77668	0.778	0.083295	0.3667	0	4	18	-0.7889	2	1	0	0	0.000000	-2.1333	0	0.05	2.000000	0.2222	1.7778	0	0	0	0	0	0.6519	-2.1333	0	1	0	0	0.3436	1.1788	0.7656	0.3333	0.1181	0.5862	0.126	0.0025	-0.1067
HIP_77669	LCGSHLVE	DEPQWSLYP	LCGSHLVEDEPQWSLYP		SCG1	31027/367830	0.962	77669	0.778	0.169381	-0.6833	0.1667	-2	17	-0.3824	-3	1	1	0	0.000000	-4.3667	-1	0.1167	0.000000	0.3333	-0.3333	1	0	1	0	4.3667	0.2278	-4.3667	0	0	1	0	0.4551	1.1619	0.8178	-0.1667	0.2071	0.6688	-0.311	0.0136	-0.5094
HIP_77670	SICSLYQLE	VRDSKENIE	SICSLYQLEVRDSKENIE		SCG2	34570/334890	0.985	77670	0.778	-0.134512	-1.1667	0	-2	18	-0.5444	-2	1	0	0	0.000000	-0.2	1	0	2.950000	0.3333	2.6167	0	0	0	0	-0.2	-3.0528	-0.2	0	0	1	0	0.4103	1.1705	0.7556	-0.1667	0.1683	0.571	-0.4786	0	0
HIP_77671	QCCTSICSL	GRLQNGQNG	QCCTSICSLGRLQNGQNG		SCG2	31458/334890	0.989	77671	0.778	0.123668	0.7333	0.3333	1	18	-0.3278	1	0	0	0	0.000000	-2.2	1	0.0667	0.000000	0.1111	-0.1111	0	1	0	1	-2.2	-0.0815	0	1	0	0	0	0.4269	0.9905	0.919	0.1667	0.1823	0.8445	0.3131	0.0044	-0.1467
HIP_77672	QPLALEGSL	FPEYNYDWW	QPLALEGSLFPEYNYDWW		SCG1	10942/367830	0.997	77672	0.778	-0.121385	0.05	0.5	-3	18	-0.5722	-3	2	0	0	1.000000	-1.6333	-1	-0.0333	1.650000	0.2222	1.4278	1	1	1	1	1.6333	0.0714	-3.2667	1	0	0	0	0.5	0.98	1.01	0.1667	0.25	1.0201	0.025	0.0011	0.0544
HIP_77673	GGPGAGSLQ	NTKFEVRLL	GGPGAGSLQNTKFEVRLL		SCG1	44287/367830	0.893	77673	0.778	-0.023078	-1.4333	0.1667	1	18	-0.1889	1	1	0	0	0.000000	-2.5333	1	0.0333	2.100000	0.1111	1.9889	0	0	0	0	-2.5333	-2.8507	-2.5333	0	1	0	0	0.5051	0.9553	1.0178	0	0.2552	1.036	-0.724	0.0011	-0.0844
HIP_77674	GSLQPLAL	TDDEDDIYK	GSLQPLALTDDEDDIYK		SCG2	22061/334890	0.995	77674	0.778	0.140245	0.2833	0	-4	17	-0.7059	-4	0	1	0	0.000000	-5.7	-2	0.15	0.950000	0.4444	0.5056	0	0	0	0	11.4	0.1432	0	1	0	0	0	0.3436	1.1538	0.739	0.1667	0.1181	0.5461	0.0974	0.0225	-0.855
HIP_77675	HLCGSHLVE	SAEPGWQAK	HLCGSHLVESAEPGWQAK		CMGA	26451/247050	0.941	77675	0.778	0.094646	0.3333	0.1667	1	18	-0.4333	-1	0	0	0	0.000000	-2.3333	0	0.0667	1.050000	0.2222	0.8278	0	0	0	0	0	0.2759	0	0	0	1	0	0.4	1.2717	0.7233	0.1667	0.16	0.5232	0.1333	0.0044	-0.1556
HIP_77676	CGSHLVEAL	GFRGPGPQL	CGSHLVEALGFRGPGPQL		CMGA	23192/247050	0.960	77676	0.778	0.101428	0	0.1667	1	18	0.1333	0	1	0	0	0.000000	-1.4	2	0.0833	1.400000	0.2222	1.1778	0	1	0	1	-2.8	0	-1.4	1	0	0	0	0.3885	1.1636	0.8178	0.1667	0.1509	0.6688	0	0.0069	-0.1167
HIP_77677	AEDLQVGQV	ENQMDKARR	AEDLQVGQVENQMDKARR		SCG1	28820/367830	0.970	77677	0.778	-0.068906	-1.7	0.1667	-1	18	-1.3111	-1	0	0	0	0.000000	-3.6	-1	0.0167	0.475000	0.1111	0.3639	0	1	0	1	3.6	-0.6186	0	1	0	0	0	0.55	0.9933	1.0433	0	0.3025	1.0885	-0.935	0.0003	-0.06
HIP_77678	GSHLVEALY	RGDERILS	GSHLVEALYRGDERILS		CMGA	20231/247050	0.971	77678	0.778	0.217869	-0.6833	0.1667	0	17	-0.2647	-1	1	1	0	0.000000	-4.2333	0	0.1833	2.000000	0.4444	1.5556	0	1	0	1	0	-1.063	-4.2333	0	1	0	0	0.3987	1.0524	0.8968	0	0.159	0.8042	-0.2725	0.0336	-0.7761
HIP_77679	LCGSHLVEA	PSLGYKEIR	LCGSHLVEAPSLGYKEIR		CMGA	19126/247050	0.975	77679	0.778	0.102995	0.65	0.3333	1	18	0.0278	0	0	0	0	0.000000	-0.3667	1	0.0833	0.000000	0.1111	-0.1111	1	0	1	0	-0.3667	-0.0722	0	1	0	0	0	0.4167	1.115	0.8533	0.3333	0.1736	0.7282	0.2708	0.0069	-0.0306
HIP_77680	CTSICSLY	FPPMYEENS	CTSICSLYFPPMYEENS		SCG2	13143/334890	0.998	77680	0.778	-0.029700	0.2167	0.5	-2	17	-0.0824	-2	3	1	0	0.000000	-0.7	0	0.0667	0.625000	0	0.625	2	0	1	0	0	0.1354	-2.1	0	1	0	0	0.4718	0.9103	1.0412	0.3333	0.2226	1.084	0.1022	0.0044	-0.0467
HIP_77681	HLVEALYL	GQLGIQEE	HLVEALYLGQLGIQEE		SCG2	16951/334890	0.997	77681	0.778	0.178510	1.0333	0.1667	-2	16	0.1812	-3	1	0	0	0.000000	-2.1333	0	0.1333	0.000000	0.1111	-0.1111	0	1	0	1	0	-0.1148	-2.1333	1	0	0	0	0.3885	1.0886	0.8145	0.5	0.1509	0.6634	0.4014	0.0178	-0.2844
HIP_77682	ICSLYQLEN	GMESAANQK	ICSLYQLENGMESAANQK		SCG2	25612/334890	0.993	77682	0.778	-0.072700	-0.8667	0.1667	-1	18	-0.45	-1	1	0	0	0.000000	0.4	0	0.0333	0.000000	0.2222	-0.2222	0	1	0	1	0	0.1926	0.4	0	1	0	0	0.4833	1.115	0.94	0	0.2336	0.8836	-0.4189	0.0011	0.0133
HIP_77683	SHLVEALY	EKVLPRLPY	SHLVEALYEKVLPRLPY		SCG2	40937/334890	0.975	77683	0.778	0.224526	0.1833	0	1	17	0.0118	0	1	1	0	0.000000	-2.5	0	0.1	0.000000	0.3333	-0.3333	0	0	0	0	0	-0.0611	-2.5	0	1	0	0	0.3436	1.2272	0.7023	0.1667	0.1181	0.4932	0.063	0.01	-0.25
HIP_77684	DLQVGQVEL	QGDLQDTKE	DLQVGQVELQGDLQDTKE		SCG1	20770/367830	0.988	77684	0.778	0.143487	-0.4833	0.1667	-4	18	-0.95	-4	0	0	0	0.000000	-3.9667	0	0.1333	0.950000	0.2222	0.7278	0	1	0	1	0	-0.3518	0	1	0	0	0	0.4551	1.0836	0.8545	0	0.2071	0.7301	-0.22	0.0178	-0.5289
HIP_77685	QPLALEGSL	GAGRSRSNQ	QPLALEGSLGAGRSRSNQ		SCG2	15126/334890	0.998	77685	0.778	-0.057952	0.6	0.6667	1	18	-0.7333	1	0	0	0	0.000000	-0.5333	0	0.0167	0.950000	0.2222	0.7278	0	3	0	1	0	0.4367	0	1	0	0	0	0.5167	0.8417	1.1433	0.3333	0.2669	1.3072	0.31	0.0003	-0.0089
HIP_77686	VEALYLVCG	PNNQKRER	VEALYLVCGPNNQKRER		SCG2	26733/334890	0.993	77686	0.778	0.112268	-0.3833	0.3333	1	17	-0.7882	1	1	1	0	0.000000	-4.9667	0	0.0833	0.625000	0	0.625	1	1	1	1	0	-0.2396	-4.9667	0	0	0	1	0.5385	0.7486	1.2578	0.1667	0.2899	1.5821	-0.2064	0.0069	-0.4139
HIP_77687	QPLALEGSL	YPEEKKEEE	QPLALEGSLYPEEKKEEE		CMGA	9621/247050	0.995	77687	0.778	-0.117913	-0.6333	0.5	-4	18	-1.3778	-4	1	0	1	0.000000	-3	-1	-0.0333	0.950000	0.3333	0.6167	1	1	1	1	3	-0.3906	-3	1	0	0	0	0.5385	0.9503	1.0612	0	0.2899	1.126	-0.341	0.0011	0.1
HIP_77688	CGSHLVEAL	ESGEETGSQ	CGSHLVEALESGEETGSQ		SCG1	17673/367830	0.992	77688	0.778	0.134045	-0.4333	0.3333	-3	18	-0.4944	-4	0	0	0	0.000000	-2.2667	0	0.15	1.400000	0.3333	1.0667	0	1	0	1	0	-0.4622	0	1	0	0	0	0.4833	1.1783	0.89	0	0.2336	0.7921	-0.2094	0.0225	-0.34
HIP_77689	CGSHLVEAL	SGEETGSQE	CGSHLVEALSGEETGSQE		SCG1	17674/367830	0.992	77689	0.778	0.134045	-0.4333	0.3333	-3	18	-0.4944	-4	0	0	0	0.000000	-2.2667	0	0.15	1.400000	0.3333	1.0667	0	1	0	1	0	-0.4622	0	1	0	0	0	0.4833	1.1783	0.89	0	0.2336	0.7921	-0.2094	0.0225	-0.34
HIP_77690	GERGFFYT	KARRHPQGA	GERGFFYTKARRHPQGA		SCG1	52474/367830	0.831	77690	0.778	0.108603	-0.9667	0	4	17	-1.3647	3	3	1	0	0.000000	-2.4667	2	0.05	1.150000	0.3333	0.8167	0	0	0	0	-4.9333	-0.7894	-7.4	0	1	0	0	0.3821	1.0908	0.8235	0	0.146	0.6781	-0.3693	0.0025	-0.1233
HIP_77691	CCTSICSLY	LSKSGYPKT	CCTSICSLYLSKSGYPKT		SCG2	33240/334890	0.987	77691	0.778	-0.017188	0.1333	0.3333	2	18	0.1444	2	1	0	0	0.000000	-0.8667	1	0.05	3.025000	0.1111	2.9139	0	0	0	0	-0.8667	0.3885	-0.8667	0	1	0	0	0.4103	1.0405	0.869	0.1667	0.1683	0.7551	0.0547	0.0025	-0.0433
HIP_77692	SHLVEALY	EEEEAEAG	SHLVEALYEEEEAEAG		CMGA	16188/247050	0.984	77692	0.778	0.092470	-1.0333	0	-5	16	-0.5937	-6	1	0	2	0.000000	-4.9333	-3	0.15	2.000000	0.5556	1.4444	0	0	0	0	14.8	-1.4926	-4.9333	0	1	0	0	0.4385	1.3836	0.7145	-0.1667	0.1922	0.5105	-0.4531	0.0225	-0.74
HIP_77693	LQPLALEGS	EKNEQINDE	LQPLALEGSEKNEQINDE		SCG2	27882/334890	0.992	77693	0.778	-0.102477	-2.6	0.3333	-4	18	-1.1389	-4	0	0	0	0.000000	-2.0667	1	-0.05	0.450000	0.4444	0.0056	0	1	0	1	-2.0667	-0.0144	0	0	1	0	0	0.5885	1.0019	1.1078	-0.5	0.3463	1.2273	-1.53	0.0025	0.1033
HIP_77694	GERGFFYTP	SGEETGSQ	GERGFFYTPSGEETGSQ		SCG1	18349/367830	0.992	77694	0.778	-0.046408	-1.3833	0.5	-2	17	-1.2	-2	3	1	0	0.000000	-0.3667	-1	0.0833	0.700000	0.2222	0.4778	1	1	1	1	0.3667	-0.6609	-1.1	0	0	0	1	0.6051	0.8719	1.1378	-0.1667	0.3662	1.2946	-0.8371	0.0069	-0.0306
HIP_77695	HLVEALYL	PGEEDIQG	HLVEALYLPGEEDIQG		SCG1	20598/367830	0.989	77695	0.778	0.200530	0.1333	0.3333	-3	16	-0.1563	-4	1	0	0	0.000000	-3.9333	-1	0.2	0.000000	0.3333	-0.3333	1	1	1	1	3.9333	-0.0444	-3.9333	1	0	0	0	0.4718	1.0336	0.9445	0.1667	0.2226	0.8921	0.0629	0.04	-0.7867
HIP_77696	CGERGFF	SSSRGEAGA	CGERGFFSSSRGEAGA		SCG1	23915/367830	0.983	77696	0.778	0.160359	0.4667	0.6667	0	16	-0.5188	0	2	2	1	0.000000	-2.5333	0	0.1167	0.700000	0.2222	0.4778	0	1	0	1	0	0.223	-5.0667	1	0	0	0	0.4833	0.8833	1.05	0.3333	0.2336	1.1025	0.2256	0.0136	-0.2956
HIP_77697	ELGGGPGAG	TPQSLATLE	ELGGGPGAGTPQSLATLE		SCG2	10208/334890	0.999	77697	0.778	-0.131310	-0.8	0.5	-2	18	-0.1611	-2	0	0	1	0.000000	-2.2667	0	0	0.450000	0	0.45	1	2	1	1	0	-0.36	0	0	0	0	1	0.5833	0.8267	1.1867	0.1667	0.3403	1.4082	-0.4667	0	0
HIP_77698	AEDLQVGQV	ASFQRNQLL	AEDLQVGQVASFQRNQLL		SCG2	8235/334890	0.999	77698	0.778	-0.027910	0.6833	0.3333	-1	18	-0.2222	-1	1	0	0	1.000000	1.1667	0	-0.0667	0.450000	0	0.45	0	1	0	1	0	0.3075	1.1667	1	0	0	0	0.4167	1.02	0.9067	0.5	0.1736	0.822	0.2847	0.0044	-0.0778

HIP_78173	VGQVELGGG	ESHLPERDS	VGQVELGGGESHLPERDS		SCG2	11378/334890	0.999	78173	0.777	-0.024757	-1.45	0.6667	-2	18	-0.7778	-3	0	0	1		
HIP_78174	HLVEALYLV	TLESVFQE	HLVEALYLVTLESVFQE		SCG2	20911/334890	0.996	78174	0.777	0.110210	1.05	0	-2	17	0.7294	-3	1	1	0		
HIP_78175	VGQVELGGG	QKTSYFPNP	VGQVELGGGQKTSYFPNP		SCG2	21176/334890	0.996	78175	0.777	-0.000355	-1.55	0.5	0	18	-0.5833	0	0	0	1		
HIP_78176	VNQHLCGSH	PNNQKRERM	VNQHLCGSHPNNQKRERM		SCG2	32628/334890	0.988	78176	0.777	-0.046574	-2.1667	0.5	4	18	-1.7056	2	0	0	0		
HIP_78177	LCGSHLVEA	VVPQGLFRG	LCGSHLVEAVVPQGLFRG		CMGA	17794/247050	0.980	78177	0.777	-0.020692	1.55	0.1667	1	18	0.7111	0	0	0	0		
HIP_78178	LVEALYLVC	QKGERGED	LVEALYLVCQKGERGED		SCG1	31026/367830	0.962	78178	0.777	0.211050	0.45	0.1667	-2	17	-0.2294	-2	1	1	0		
HIP_78179	CCTSICSLY	EKPLDSQSI	CCTSICSLYEKPLDSQSI		SCG2	28293/334890	0.991	78179	0.777	0.035483	-1.2167	0.3333	-1	18	0.1611	-1	1	0	0		
HIP_78180	RGFFYTPKT	QLSDDVSKV	RGFFYTPKTQLSDDVSKV		SCG2	30728/334890	0.989	78180	0.777	-0.013166	-1.1167	0.3333	1	18	-0.6278	1	1	0	0		
HIP_78181	GGGPGAGSL	ARYYSALRH	GGGPGAGSLARYYSALRH		NPY	4628/49410	0.943	78181	0.777	0.097609	-0.2333	0.3333	3	18	-0.3889	2	2	0	1		
HIP_78182	VGQVELGGG	DTPNRQYWD	VGQVELGGGDTPNRQYWD		SCG2	11780/334890	0.998	78182	0.777	-0.063000	-1.1667	0.6667	-2	18	-1.0778	-2	0	0	1		
HIP_78183	IVEQCCTSI	RAYGFRGPG	IVEQCCTSIRAYGFRGPG		CMGA	28161/247050	0.930	78183	0.777	0.066476	-0.1667	0.1667	1	18	0.0667	1	1	0	0		
HIP_78184	GERGFFYT	ASFQRNQL	GERGFFYTASFQRNQL		SCG2	28571/334890	0.991	78184	0.777	-0.045121	0.7667	0.1667	1	16	-0.7875	1	4	0	0		
HIP_78185	CGERGFFY	SMKLSFRAR	CGERGFFYSMKLSFRAR		CMGA	29293/247050	0.920	78185	0.777	0.194969	0.25	0.1667	3	17	-0.3647	3	3	1	0		
HIP_78186	LYQLENYCN	QQKEEEEEM	LYQLENYCNQQKEEEEEM		CMGA	20620/247050	0.970	78186	0.777	0.057523	-2.2	0	-5	18	-1.8333	-5	1	0	3		
HIP_78187	SHLVEALY	PMDMSDDYE	SHLVEALYPMDMSDDYE		SCG2	15921/334890	0.997	78187	0.777	0.106814	0.1833	0.1667	-4	17	-0.5353	-5	1	1	0		
HIP_78188	GSLQPLALE	LPLQVRGYP	GSLQPLALELPLQVRGYP		CMGA	17873/247050	0.979	78188	0.777	-0.046704	1.35	0.1667	0	18	0.1278	0	0	0	0		
HIP_78189	SLYQLENYC	LGMESAANQ	SLYQLENYCLGMESAANQ		SCG2	11140/334890	0.999	78189	0.777	-0.176194	0.5	0.1667	-2	18	-0.3444	-2	1	0	0		
HIP_78190	ERGFFYTPK	IEISRNLQI	ERGFFYTPKIEISRNLQI		SCG2	27817/334890	0.992	78190	0.777	0.029123	-0.1167	0.1667	1	18	-0.4889	1	2	0	0		
HIP_78191	LVEALYLVC	LLRDPADAS	LVEALYLVCLLRDPADAS		SCG1	30500/367830	0.964	78191	0.777	0.026354	2.2667	0	-2	18	0.9222	-2	1	0	0		
HIP_78192	ERGFFYTPK	PSPMPVSQE	ERGFFYTPKPSPMPVSQE		CMGA	17944/247050	0.979	78192	0.777	-0.117959	-1.7	0.6667	0	18	-0.9778	0	2	0	0		
HIP_78193	PGAGSLQPL	SGEATDGAR	PGAGSLQPLSGEATDGAR		CMGA	9705/247050	0.995	78193	0.777	-0.005459	-1	0.5	-1	18	-0.5056	-1	0	0	0		
HIP_78194	SHLVEALY	EEPSSKDV	SHLVEALYEEPSSKDV		CMGA	16320/247050	0.984	78194	0.777	0.148537	-0.7167	0.1667	-2	16	-0.5375	-3	1	0	0		
HIP_78195	EALYLVCGE	ENGDESHLP	EALYLVCGEENGDESHLP		SCG2	16695/334890	0.997	78195	0.777	-0.116608	-1.4667	0.3333	-4	18	-0.4889	-5	0	0	0		
HIP_78196	LCGSHLVEA	KNFFPEYN	LCGSHLVEAKNFFPEYN		SCG1	45655/367830	0.884	78196	0.777	0.095670	-0.35	0	0	17	-0.2059	-1	2	1	0		
HIP_78197	PLALEGSLQ	APAEDMARY	PLALEGSLQAPAEDMARY		NPY	1269/49410	0.993	78197	0.777	-0.116058	0.25	0.3333	-2	18	-0.2056	-2	0	0	0		
HIP_78198	HLCGSHLVE	ADASEAHES	HLCGSHLVEADASEAHES		SCG1	31031/367830	0.962	78198	0.777	-0.057285	0.7667	0	-1	18	-0.3722	-4	0	0	0		
HIP_78199	CGSHLVEAL	IPLIFLISG	CGSHLVEALIPLIFLISG		SCG2	22009/334890	0.995	78199	0.777	-0.054496	1.4667	0.1667	0	18	1.6278	-1	0	0	0		
HIP_78200	NQHLCGSHL	KRERMDEEQ	NQHLCGSHLKRERMDEEQ		SCG2	49792/334890	0.943	78200	0.777	0.142957	-2.0167	0.1667	1	18	-1.8333	-1	0	0	0		
HIP_78201	TSICSLYQL	SEEKHLEE	TSICSLYQLSEEKHLEE		SCG1	29958/367830	0.966	78201	0.777	0.148674	-1.4667	0.1667	-2	17	-0.6235	-3	1	1	0		
HIP_78202	VEALYLVC	DRADQTVLT	VEALYLVCDRADQTVLT		SCG1	37673/367830	0.931	78202	0.777	0.187874	0.7167	0	-2	17	0.5235	-2	1	1	0		
HIP_78203	SLYQLENYC	LQDLALQGA	SLYQLENYCLQDLALQGA		CMGA	19470/247050	0.974	78203	0.777	-0.131790	-0.9167	0	-2	18	0.0167	-2	1	0	0		
HIP_78204	CSLYQLENY	CIVEVISDT	CSLYQLENYCIVEVISDT		CMGA	11981/247050	0.992	78204	0.777	0.003846	0.4833	0	-3	18	0.4222	-3	1	0	0		
HIP_78205	RGFFYTPKT	MKLSFRARA	RGFFYTPKTMKLSFRARA		CMGA	25306/247050	0.948	78205	0.777	0.138534	-0.7333	0.1667	5	18	-0.5056	5	1	0	0		
HIP_78206	HLVEALYL	MAEAKTHWL	HLVEALYLMAEAKTHWL		SCG2	30116/334890	0.990	78206	0.777	0.054745	1.0833	0	1	17	0.3824	-1	1	1	0		
HIP_78207	GPGAGSLQP	KNEQINDEM	GPGAGSLQPKNEQINDEM		SCG2	33551/334890	0.987	78207	0.777	-0.026714	-2.0333	0.1667	-2	18	-1.2	-2	0	0	0		
HIP_78208	GIVEQCCTS	GKWQQQGDL	GIVEQCCTSGKWQQQGDL		SCG1	29650/367830	0.967	78208	0.777	-0.023081	-0.7	0.3333	-1	18	-0.6111	-1	1	0	1		
HIP_78209	GGGPGAGSL	GHSRERADE	GGGPGAGSLGHSRERADE		SCG1	20286/367830	0.989	78209	0.777	-0.063350	-0.3	0.6667	0	18	-1.1611	-1	0	0	1		
HIP_78210	GSLQPLALE	QDPEGKGEQ	GSLQPLALEQDPEGKGEQ		CMGA	18166/247050	0.978	78210	0.777	0.130694	-1.0833	0.1667	-3	18	-1.1333	-3	0	0	0		
HIP_78211	ICSLYQLEN	RGEAGAPGE	ICSLYQLENRGEAGAPGE		SCG1	34514/367830	0.947	78211	0.777	0.058125	-1.9333	0.1667	-2	18	-0.4833	-2	1	0	0		
HIP_78212	LYQLENYCN	PMPVSQECF	LYQLENYCNPMPVSQECF		CMGA	8340/247050	0.996	78212	0.777	-0.102785	-0.6	0.3333	-2	18	-0.3389	-2	1	0	0		
HIP_78213	EALYLVCGE	KENREEARF	EALYLVCGEKENREEARF		SCG1	41871/367830	0.907	78213	0.777	-0.073814	-2.05	0.1667	-2	18	-0.8278	-2	0	0	0		
HIP_78214	HLVEALYL	SGAEAASF	HLVEALYLSGAEAASF		SCG2	17995/334890	0.997	78214	0.777	0.200263	1.15	0.3333	-1	16	0.7562	-2	1	0	0		
HIP_78215	VEALYLVC	EPGWQAKR	VEALYLVCEPGWQAKR		CMGA	17858/247050	0.979	78215	0.777	0.194479	0.8333	0.3333	0	16	-0.0625	0	2	0	0		
HIP_78216	AGSLQPLAL	CTSICSLY	AGSLQPLALCTSICSLY		INSA	212/3024	0.896	78216	0.777	0.135097	1.7333	0.1667	0	17	1.0824	0	0	1	0		
HIP_78217	LCGSHLVEA	SKVIAYLKR	LCGSHLVEASKVIAYLKR		SCG2	42899/334890	0.970	78217	0.777	0.110862	0.3333	0.1667	3	18	0.45	2	0	0	0		
HIP_78218	VCGERGFF	VEVLKREPL	VCGERGFFVEVLKREPL		IAPP	3025/45018	0.978	78218	0.777	0.003817	1.6833	0.1667	0	17	0.1471	0	2	1	0		
HIP_78219	VEALYLVCG	KRTNEIVEE	VEALYLVCGKRTNEIVEE		SCG2	36566/334890	0.983	78219	0.777	0.081502	-0.4667	0.1667	-2	18	0.0389	-2	1	0	0		
HIP_78220	HLVEALYL	GARPQALPE	HLVEALYLGARPQALPE		CMGA	19371/247050	0.974	78220	0.777	0.188997	0.5333	0.1667	0	17	0.1	-1	1	1	0		
HIP_78221	GSLQPLALE	EHIAKRAME	GSLQPLALEEHIAKRAME		SCG2	40845/334890	0.975	78221	0.777	-0.035829	-0.0167	0	0	18	-0.2889	-1	0	0	0		
HIP_78222	CGSHLVEAL	WRPSSREDS	CGSHLVEALWRPSSREDS		CMGA	28068/247050	0.930	78222	0.777	0.066261	-0.8167	0.1667	0	18	-0.7056	-1	1	0	0		
HIP_78223	CGERGFFYT	KTPGRAGT	CGERGFFYTKTPGRAGT		SCG2	37799/334890	0.981	78223	0.777	0.177634	-0.9	0.1667	2	17	-0.7706	2	3	1	0		
HIP_78224	QVELGGGPG	AAWIPHVEN	QVELGGGPGAAWIPHVEN		SCG2	7037/334890	0.999	78224	0.777	0.110470	0.05	0.5	-1	18	-0.1444	-2	1	0	1		
HIP_78225	RGFFYTP	EEEEEAT	RGFFYTPEEEEEAT		CMGA	20427/247050	0.971	78225	0.777	-0.019482	-2.35	0.1667	-4	14	-1.3786	-4	3	0	3		
HIP_78226	VCGERGFF	NSMPVDNRN	VCGERGFFNSMPVDNRN		SCG1	27058/367830	0.975	78226	0.777	0.021391	0.4667	0.3333	0	17	-0.6647	0	2	1	0		
HIP_78227	EQCCTSICS	EPGKGRHH	EQCCTSICSEPGKGRHH		SCG1	23256/367830	0.984	78227	0.777	0.166982	0.1167	0.5	2	17	-1.0588	0	2	1	0		
HIP_78228	LVEALYLVC	WLGAALSL	LVEALYLVCWLGAALSL		SCG2	21892/334890	0.995	78228	0.777	0.067111	2.1667	0.1667	-1	17	1.8941	-1	2	1	0		

HIP_78684	GSHLVEALY	EEDIQGPT	GSHLVEALYEEDIQGPT		SCG1	26852/367830	0.975	78684	0.777
HIP_78685	CCTSICSLY	LYTDDEDDI	CCTSICSLYLYTDDEDDI		SCG2	13413/334890	0.998	78685	0.777
HIP_78686	IVEQCCTSI	QDTKENREE	IVEQCCTSIQDTKENREE		SCG1	25006/367830	0.980	78686	0.777
HIP_78687	IVEQCCTSI	GEKRDHHST	IVEQCCTSIGEKRDHHST		SCG1	33494/367830	0.951	78687	0.777
HIP_78688	VEQCCTSIC	QREDEEEEE	VEQCCTSICQREDEEEEE		SCG1	23932/367830	0.983	78688	0.777
HIP_78689	VGQVELGGG	GDESHLPER	VGQVELGGGGDESHLPER		SCG2	13236/334890	0.998	78689	0.777
HIP_78690	GSLQPLALE	NGSVEPERE	GSLQPLALENGSVEPERE		SCG2	16307/334890	0.997	78690	0.777
HIP_78691	CGERGFFY	SLELDKMA	CGERGFFYSLELDKMA		SCG1	27594/367830	0.973	78691	0.777
HIP_78692	VELGGGPGA	THSREKSSQ	VELGGGPGATHSREKSSQ		SCG1	24795/367830	0.981	78692	0.777
HIP_78693	GAGSLQPLA	GYGEESEEE	GAGSLQPLAGYGEESEEE		SCG1	3056/367830	1.000	78693	0.777
HIP_78694	CGSHLVEAL	LNLLGMESA	CGSHLVEALLNLLGMESA		SCG2	23377/334890	0.995	78694	0.777
HIP_78695	QVGQVELG	EEERLSKEW	QVGQVELGEEERLSKEW		CMGA	20958/247050	0.969	78695	0.777
HIP_78696	CGSHLVEAL	PQGLFRGGK	CGSHLVEALPQGLFRGGK		CMGA	18116/247050	0.979	78696	0.777
HIP_78697	CTSICSLYQ	FPDFYDSE	CTSICSLYQFPDFYDSE		SCG1	18276/367830	0.992	78697	0.777
HIP_78698	ERGFFYTPK	VKRVPGQGS	ERGFFYTPKVKRVPGQGS		SCG2	37082/334890	0.982	78698	0.777
HIP_78699	VNQHLCGSH	GENYQKGER	VNQHLCGSHGENYQKGER		SCG1	32238/367830	0.957	78699	0.777
HIP_78700	EQCCTSICS	YDRVAQLDQ	EQCCTSICSYDRVAQLDQ		SCG1	38817/367830	0.925	78700	0.777
HIP_78701	GPGAGSLQP	EEEATNTH	GPGAGSLQPEEEATNTH		CMGA	20397/247050	0.971	78701	0.777
HIP_78702	IVEQCCTSI	KYPEIINSN	IVEQCCTSIKYPEIINSN		SCG2	33620/334890	0.986	78702	0.777
HIP_78703	GIVEQCCTS	KTSRKDVKD	GIVEQCCTSKTSRKDVKD		SCG1	47711/367830	0.869	78703	0.777
HIP_78704	VEALYLVC	MAEAKTHWL	VEALYLVCMAEAKTHWL		SCG2	30191/334890	0.990	78704	0.777
HIP_78705	QVGQVELGG	YFPNPYNQE	QVGQVELGGYFPNPYNQE		SCG2	4356/334890	1.000	78705	0.777
HIP_78706	CCTSICSLY	LPVDLDDIS	CCTSICSLYLPVDLDDIS		SCG2	14076/334890	0.998	78706	0.777
HIP_78707	VELGGGPGA	RGSEEYRAP	VELGGGPGARGSEEYRAP		SCG1	20084/367830	0.989	78707	0.777
HIP_78708	RGFFYTPKT	QVTALPVNS	RGFFYTPKTQVTALPVNS		CMGA	23824/247050	0.957	78708	0.777
HIP_78709	CCTSICSLY	WWEKKPFS	CCTSICSLYWWEKKPFS		SCG1	37827/367830	0.930	78709	0.777
HIP_78710	VELGGGPGA	EGDSEGLSQ	VELGGGPGAEGDSEGLSQ		CMGA	8367/247050	0.996	78710	0.777
HIP_78711	ERGFFYTPK	REEKRFLG	ERGFFYTPKREEKRFLG		SCG1	49266/367830	0.857	78711	0.777
HIP_78712	YQLENYCN	EGDSEGLSQ	YQLENYCNEGDSEGLSQ		CMGA	14275/247050	0.988	78712	0.777
HIP_78713	VEALYLVCG	LQKEPDLRL	VEALYLVCGLQKEPDLRL		SCG2	29194/334890	0.991	78713	0.777
HIP_78714	AEDLQVGQV	TRQRYGKRS	AEDLQVGQVTRQRYGKRS		NPY	6716/49410	0.861	78714	0.777
HIP_78715	LCGSHLVEA	SLSEEDWMR	LCGSHLVEASLSEEDWMR		SCG2	19578/334890	0.996	78715	0.777
HIP_78716	GSLQPLAL	RMDEEQKLY	GSLQPLALRMDEEQKLY		SCG2	34515/334890	0.985	78716	0.777
HIP_78717	SHLVEALY	EVRDSKENI	SHLVEALYEVRDSKENI		SCG2	34545/334890	0.985	78717	0.777
HIP_78718	PLALEGSLQ	EGKGEQEHS	PLALEGSLQEGKGEQEHS		CMGA	16177/247050	0.984	78718	0.777
HIP_78719	LVCGERGFF	GSVEPERE	LVCGERGFFGSVEPERE		SCG2	8213/334890	0.999	78719	0.777
HIP_78720	CTSICSLY	NNQKRERMD	CTSICSLYNNQKRERMD		SCG2	38988/334890	0.979	78720	0.777
HIP_78721	GGPGAGSLQ	ANQKTSYFP	GGPGAGSLQANQKTSYFP		SCG2	35654/334890	0.984	78721	0.777
HIP_78722	ERGFFYTPK	WGYEKRNL	ERGFFYTPKWGYEKRNL		SCG1	28431/367830	0.971	78722	0.777
HIP_78723	TSICSLYQ	QMAYENLN	TSICSLYQQMAYENLN		SCG2	27524/334890	0.992	78723	0.777
HIP_78724	GAGSLQPLA	SLLVCLGAL	GAGSLQPLASLLVCLGAL		NPY	3183/49410	0.973	78724	0.777
HIP_78725	IVEQCCTSI	EPGETQNAF	IVEQCCTSIEPGETQNAF		SCG1	8290/367830	0.998	78725	0.777
HIP_78726	GSLQPLAL	GAILSSTN	GSLQPLALGAILSSTN		IAPP	2794/45018	0.981	78726	0.777
[illegible]	[illegible]	[illegible]	[illegible]		[illegible]	[illegible]	[illegible]	[illegible]	[illegible]

HIP_79195	FVNQHLCG	NNFGAILSS	FVNQHLCGNNFGAILSS		IAPP	3639/45018	0.966	79195	0.776	0.037035	0.2833	0.1667	[illegible]
HIP_79196	QHLCGSHLV	VSEEVKTR	QHLCGSHLVVSEEVKTR		SCG1	31886/367830	0.958	79196	0.776	0.161342	0.7833	0.1667	[illegible]
HIP_79197	LYLVCGERG	RGRGSEEYR	LYLVCGERGRGRGSEEYR		SCG1	44306/367830	0.893	79197	0.776	-0.039618	-2.9667	0.3333	[illegible]
HIP_79198	EALYLVCG	VFLIVLSVA	EALYLVCGVFLIVLSVA		IAPP	2441/45018	0.986	79198	0.776	0.009364	2.85	0.1667	[illegible]
HIP_79199	GERGFFYT	SQSIYQLI	GERGFFYTSQSIYQLI		SCG2	27726/334890	0.992	79199	0.776	0.153518	-0.7167	0.3333	[illegible]
HIP_79200	GSLQPLAL	DKLAPVSKR	GSLQPLALDKLAPVSKR		SCG2	40806/334890	0.975	79200	0.776	0.218820	0.9667	0	[illegible]
HIP_79201	ICSLYQLEN	SQREDEEEE	ICSLYQLENSQREDEEEE		SCG1	31170/367830	0.961	79201	0.776	-0.010022	-2	0.1667	[illegible]
HIP_79202	CSLYQLENY	ERKQASAIK	CSLYQLENYERKQASAIK		SCG1	57811/367830	0.781	79202	0.776	0.075818	-1.3667	0	[illegible]
HIP_79203	SLYQLENYC	LNPHPSLGY	SLYQLENYCLNPHPSLGY		CMGA	14249/247050	0.988	79203	0.776	-0.101444	-0.6	0.1667	[illegible]
HIP_79204	CGERGFFY	KNEQINDE	CGERGFFYKNEQINDE		SCG2	33235/334890	0.987	79204	0.776	0.188989	-1.1	0	[illegible]
HIP_79205	QCCTSICS	GPQLRRGWR	QCCTSICSGPQLRRGWR		CMGA	20898/247050	0.969	79205	0.776	0.204909	0.1167	0.5	[illegible]
HIP_79206	GERGFFYTP	PVDNRNHN	GERGFFYTPPVDNRNHN		SCG1	30051/367830	0.965	79206	0.776	-0.102550	-0.75	0.3333	[illegible]
HIP_79207	HLCGSHLVE	LSILRHQNL	HLCGSHLVELSILRHQNL		CMGA	21369/247050	0.967	79207	0.776	-0.009248	2	0.1667	[illegible]
HIP_79208	CTSICSLY	PSPMPVSQE	CTSICSLYPSPMPVSQE		CMGA	12097/247050	0.992	79208	0.776	0.126301	-0.3833	0.6667	[illegible]
HIP_79209	VGQVELGGG	ELDKMAHGY	VGQVELGGGELDKMAHGY		SCG1	12780/367830	0.996	79209	0.776	0.083167	-0.7333	0.5	[illegible]
HIP_79210	LVCGERGFF	FRARAYGF	LVCGERGFFFRARAYGF		CMGA	24685/247050	0.952	79210	0.776	0.057398	0.8833	0.1667	[illegible]
HIP_79211	SHLVEALY	LVCLGALAE	SHLVEALYLVCLGALAE		NPY	3434/49410	0.969	79211	0.776	-0.036810	2.4667	0	[illegible]
HIP_79212	LVEALYL	FLIVLSV	LVEALYLFLIVLSV		IAPP	2145/45018	0.975	79212	0.776	-0.020861	2.9	0	[illegible]
HIP_79213	LVEALYL	EEIKGYPG	LVEALYLEEIKGYPG		SCG1	28269/367830	0.971	79213	0.776	0.115021	0.6333	0	[illegible]
HIP_79214	ELGGGPGA	PEYNYDWWE	ELGGGPGAPEYNYDWWE		SCG1	16890/367830	0.993	79214	0.776	-0.090089	-1.1	0.5	[illegible]
HIP_79215	QCCTSICSL	IQGPTKADT	QCCTSICSLIQGPTKADT		SCG1	16871/367830	0.993	79215	0.776	0.031818	1.0167	0.3333	[illegible]
HIP_79216	VEALYLVC	QEHSQQKEE	VEALYLVCQEHSQQKEE		CMGA	22775/247050	0.961	79216	0.776	0.142485	0.05	0	[illegible]
HIP_79217	GIVEQCCTS	TENVPRTRL	GIVEQCCTSTENVPRTRL		NPY	4816/49410	0.928	79217	0.776	-0.055813	-1.1167	0.1667	[illegible]
HIP_79218	EQCCTSICS	STHYRASEE	EQCCTSICSSTHYRASEE		SCG1	31454/367830	0.960	79218	0.776	0.051582	0.25	0.3333	[illegible]
HIP_79219	SICSLYQLE	DQLSDDVSK	SICSLYQLEDQLSDDVSK		SCG2	29806/334890	0.990	79219	0.776	-0.161619	-1.0667	0	[illegible]
HIP_79220	ERGFFYTP	GSGRLQNG	ERGFFYTPGSGRLQNG		SCG2	16727/334890	0.997	79220	0.776	-0.002339	-0.8667	0.6667	[illegible]
HIP_79221	VEQCCTSIC	NAAGSGRLQ	VEQCCTSICNAAGSGRLQ		SCG2	25714/334890	0.993	79221	0.776	-0.037070	1.05	0.1667	[illegible]
HIP_79222	HLVEALYLV	KRDHHSTHY	HLVEALYLVKRDHHSTHY		SCG1	45423/367830	0.885	79222	0.776	0.227789	-0.8667	0	[illegible]
HIP_79223	VEALYLVC	EKSGEATDG	VEALYLVCEKSGEATDG		CMGA	21987/247050	0.965	79223	0.776	0.224458	0.3833	0.1667	[illegible]
HIP_79224	VEALYLVCG	QIPPEDLIE	VEALYLVCGQIPPEDLIE		SCG2	8085/334890	0.999	79224	0.776	-0.077832	0.95	0.3333	[illegible]
HIP_79225	NQHLCGSHL	PNGSVEPER	NQHLCGSHLPNGSVEPER		SCG2	18320/334890	0.997	79225	0.776	0.117130	-0.95	0.5	[illegible]
HIP_79226	CSLYQLENY	EKRNLARVP	CSLYQLENYEKRNLARVP		SCG1	57812/367830	0.781	79226	0.776	0.075856	-1.3667	0	[illegible]
HIP_79227	LYQLENYCN	LEQEEERLS	LYQLENYCNLEQEEERLS		CMGA	16025/247050	0.984	79227	0.776	-0.139353	-0.9167	0	[illegible]
HIP_79228	GGPGAGSLQ	SEVDKRRTR	GGPGAGSLQSEVDKRRTR		SCG1	44297/367830	0.893	79228	0.776	-0.033268	-0.1	0.3333	[illegible]
HIP_79229	AGSLQPLAL	LGGGPGAGS	AGSLQPLALLGGGPGAGS		INSC	261/5184	0.997	79229	0.776	0.179433	2.0667	0.3333	[illegible]
HIP_79230	GERGFFYT	KENKPYALN	GERGFFYTKENKPYALN		SCG2	40669/334890	0.976	79230	0.776	0.147994	-1.6833	0	[illegible]
HIP_79231	LYQLENYCN	KEAVEEPSS	LYQLENYCNKEAVEEPSS		CMGA	23246/247050	0.959	79231	0.776	-0.096731	-1.3167	0	[illegible]
HIP_79232	GSLQPLALE	YSALRHYIN	GSLQPLALEYSALRHYIN		NPY	5103/49410	0.930	79232	0.776	-0.027417	0.3	0.1667	[illegible]
HIP_79233	TSICSLYQL	YGKRNAVEV	TSICSLYQLYGKRNAVEV		IAPP	4887/45018	0.931	79233	0.776	0.150553	-1.1	0.1667	[illegible]
HIP_79234	VEALYLVC	YEENSRDNP	VEALYLVCYEENSRDNP		SCG2	28689/334890	0.991	79234	0.776	0.103504	0.3667	0	[illegible]
HIP_79235	GERGFFY	LQVFLIV	GERGFFYLQVFLIV		IAPP	3395/45018	0.970	79235	0.776	-0.007417	1.4667	0	[illegible]
HIP_79236	CSLYQLENY	SAANQKTSY	CSLYQLENYSAANQKTSY		SCG2	26649/334890	0.993	79236	0.776	-0.054095	-0.9167	0.1667	[illegible]
HIP_79237	CSLYQLENY	LQVRGYPEE	CSLYQLENYLQVRGYPEE		CMGA	15076/247050	0.987	79237	0.776	-0.089660	-0.6333	0	[illegible]
[illegible]													

HIP_79706	GSLQPLAL	EDWMRIIL	GSLQPLALEDWMRIIL		SCG2	27927/334890	0.982	79706	0.776	0.119668	0.25	0	-1	16	0.575	-1	1	0	0
HIP_79707	SLQPLALEG	ENELTLNEK	SLQPLALEGENELTLNEK		SCG1	28966/367830	0.969	79707	0.776	-0.139577	-1.7667	0.1667	-3	18	-0.6167	-3	0	0	0
HIP_79708	QHLCGSHLV	DNRDSSMKL	QHLCGSHLVDNRDSSMKL		CMGA	27183/247050	0.937	79708	0.776	0.244622	-1.1167	0	2	18	-0.6444	0	0	0	0
HIP_79709	ELGGGPGAG	EESEEERGL	ELGGGPGAGEESEEERGL		SCG1	14785/367830	0.995	79709	0.776	-0.123856	-1.1333	0.5	-5	18	-1.1611	-5	0	0	2
HIP_79710	ELGGGPGAG	ESEEERGLE	ELGGGPGAGESEEERGLE		SCG1	14786/367830	0.995	79710	0.776	-0.123856	-1.1333	0.5	-5	18	-1.1611	-5	0	0	2
HIP_79711	VEQCCTSIC	QVKRVPGQG	VEQCCTSICQVKRVPGQG		SCG2	41941/334890	0.973	79711	0.776	0.043060	0.5	0.1667	1	18	-0.0944	1	0	0	0
HIP_79712	NQHLCGSHL	QKLYTDDED	NQHLCGSHLQKLYTDDED		SCG2	34535/334890	0.985	79712	0.776	-0.034997	-0.6333	0.1667	-1	18	-1.3389	-3	1	0	0
HIP_79713	VEALYLVCG	GLSGLTLAL	VEALYLVCGGLSGLTLAL		NPY	1546/49410	0.991	79713	0.776	0.004924	1.4833	0.5	-1	18	1.6556	-1	1	0	0
HIP_79714	HLCGSHLV	EEEDNRDSS	HLCGSHLVEEEDNRDSS		CMGA	18882/247050	0.976	79714	0.776	0.120218	-0.95	0	-2	17	-1.2	-4	0	1	1
HIP_79715	ALYLVCGER	SRPDRSSQG	ALYLVCGERSRPDRSSQG		SCG1	38293/367830	0.928	79715	0.776	-0.083347	-2.55	0.5	1	18	-0.7778	1	0	0	0
HIP_79716	LGGGPGAGS	QEESEESNV	LGGGPGAGSQEESEESNV		SCG1	22557/367830	0.985	79716	0.776	-0.090637	-1.65	0.3333	-4	18	-0.9556	-4	0	0	1
HIP_79717	CGERGFFY	KRRTRPRHH	CGERGFFYKRRTRPRHH		SCG1	52112/367830	0.834	79717	0.776	0.216118	-1.4333	0	7	17	-1.9176	5	3	1	0
HIP_79718	FVNQHLCG	EGGGHSRER	FVNQHLCGEGGGHSRER		SCG1	21040/367830	0.988	79718	0.776	0.163432	0.2667	0.5	2	17	-1.0882	0	0	1	1
HIP_79719	GGPGAGSLQ	EENSRDNPF	GGPGAGSLQEENSRDNPF		SCG2	29685/334890	0.990	79719	0.776	-0.081315	-1.8333	0.1667	-2	18	-1.3056	-2	0	1	0
HIP_79720	NQHLCGSHL	ECFETLRGD	NQHLCGSHLECFETLRGD		CMGA	22658/247050	0.962	79720	0.776	-0.065644	0.2667	0.1667	0	18	-0.6389	-2	1	0	0
HIP_79721	HLCGSHLV	YGEEIKGYP	HLCGSHLVYGEEIKGYP		SCG1	27417/367830	0.974	79721	0.776	0.171664	-0.0667	0.1667	1	17	-0.2765	-1	1	1	0
HIP_79722	LVCGERGFF	KPLDSQSI	LVCGERGFFKPLDSQSI		SCG2	29397/334890	0.991	79722	0.776	0.051713	0.5833	0.3333	0	17	0.0882	0	2	1	0
HIP_79723	GAGSLQPLA	KQYSSHHTA	GAGSLQPLAKQYSSHHTA		SCG1	46938/367830	0.875	79723	0.776	0.051299	-0.7833	0.1667	3	18	-0.6167	1	1	0	0
HIP_79724	ICSLYQLE	EEEMAVVPQ	ICSLYQLEEEEMAVVPQ		CMGA	24182/247050	0.955	79724	0.776	-0.002284	-2.2833	0	-4	17	0.1176	-4	1	1	2
HIP_79725	GERGFFYT	ATNTHPPAS	GERGFFYTATNTHPPAS		CMGA	25524/247050	0.947	79725	0.776	0.053252	-0.2667	0	1	17	-0.8059	0	3	1	0
HIP_79726	IVEQCCTSI	WQQQGDLQD	IVEQCCTSIWQQQGDLQD		SCG1	27535/367830	0.973	79726	0.776	-0.055526	-0.8167	0.1667	-3	18	-0.4889	-3	1	0	1
HIP_79727	LALEGSLQ	IVLSVALNH	LALEGSLQIVLSVALNH		IAPP	2445/45018	0.986	79727	0.776	0.095929	2	0.1667	0	17	1.1647	-1	0	1	0
HIP_79728	CTSICSLY	EEEEDNRDS	CTSICSLYEEEEDNRDS		CMGA	18892/247050	0.976	79728	0.776	0.028322	-1.4667	0.1667	-5	17	-1.1824	-5	1	1	2
HIP_79729	IVEQCCTSI	EDWNPVEEK	IVEQCCTSIEDWNPVEEK		SCG2	23945/334890	0.994	79729	0.776	-0.057331	-0.8167	0.1667	-4	18	-0.5556	-4	1	0	0
HIP_79730	DLQVGQVEL	RSETHAAGH	DLQVGQVELRSETHAAGH		SCG1	44108/367830	0.894	79730	0.776	0.213627	-0.7167	0.1667	0	18	-0.6167	-2	0	0	0
HIP_79731	EQCCTSICS	HSEKSQRED	EQCCTSICSHSEKSQRED		SCG1	30968/367830	0.962	79731	0.776	0.015881	-0.2167	0.3333	-1	18	-1.3611	-2	0	0	0
HIP_79732	HLVEALYL	QPTLLLSLL	HLVEALYLQPTLLLSLL		SCG1	25887/367830	0.978	79732	0.776	0.193190	0.0833	0.1667	0	17	1.2824	-1	1	1	1
HIP_79733	HLVEALYLV	NLRQQAHK	HLVEALYLVNLRQQAHK		SCG2	41147/334890	0.974	79733	0.776	0.201517	0.4167	0	3	17	-0.1706	1	1	1	0
HIP_79734	GPGAGSLQP	FEKPLDSQS	GPGAGSLQPFEKPLDSQS		SCG2	32733/334890	0.987	79734	0.776	-0.089705	-0.9833	0.1667	-1	18	-0.7833	-1	1	0	0
HIP_79735	LYQLENYCN	DNMNDNFLE	LYQLENYCNDNMNDNFLE		SCG1	24096/367830	0.982	79735	0.776	-0.081690	-1.2333	0	-4	18	-1.0556	-4	1	0	0
HIP_79736	RGFFYTPKT	IEAELEKVA	RGFFYTPKTIEAELEKVA		CMGA	19951/247050	0.972	79736	0.776	0.074266	-0.5667	0.1667	0	18	-0.3222	0	1	0	0
HIP_79737	VEALYLVC	SREDSLEAG	VEALYLVCSREDSLEAG		CMGA	22220/247050	0.964	79737	0.776	0.223535	0.2833	0.1667	-3	17	0.2412	-3	1	1	0
HIP_79738	AGSLQPLAL	GQPRSQEE	AGSLQPLALGQPRSQEE		SCG1	24841/367830	0.980	79738	0.776	0.222035	0.65	0.3333	-1	17	-0.7412	-1	0	1	0
HIP_79739	SICSLYQLE	RSGQLGIQE	SICSLYQLERSGQLGIQE		SCG2	26828/334890	0.993	79739	0.776	0.002864	-1.4833	0.3333	-1	18	-0.2	-1	1	0	0
HIP_79740	YLVCGERGF	GVSVPLQQK	YLVCGERGFGVSVPLQQK		SCG2	20144/334890	0.996	79740	0.776	-0.086085	0.15	0.5	1	18	0.0944	1	1	0	0
HIP_79741	EAEDLQVGQ	APGEEEEEE	EAEDLQVGQAPGEEEEEE		CMGA	4461/247050	0.999	79741	0.776	-0.163105	0.0167	0.5	-9	18	-1.6278	-9	0	0	4
HIP_79742	VCGERGFF	NTATCATQ	VCGERGFFNTATCATQ		IAPP	3690/45018	0.964	79742	0.776	0.041370	0.4667	0.1667	0	16	0.0312	0	2	0	0
HIP_79743	GSHLVEALY	NLITRQRY	GSHLVEALYNLITRQRY		NPY	4557/49410	0.944	79743	0.776	0.025698	1.5167	0	2	17	-0.3118	1	1	1	0
HIP_79744	GSLQPLALE	EERLSKEWE	GSLQPLALEEERLSKEWE		CMGA	25984/247050	0.944	79744	0.776	0.075901	-1.5667	0	-3	18	-0.9389	-3	0	0	1
HIP_79745	CGERGFFY	REHIAKRAM	CGERGFFYREHIAKRAM		SCG2	43748/334890	0.967	79745	0.776	0.179064	-1.15	0	3	17	-0.6824	2	3	1	0
HIP_79746	GERGFFYT	LSVEDILNL	GERGFFYTLSVEDILNL		SCG2	22436/334890	0.995	79746	0.776	-0.096136	1.3333	0.1667	-2	17	0.2118	-2	3	1	0
HIP_79747	YLVCGERGF	DESHLPERD	YLVCGERGFDESHLPERD		SCG2	25301/334890	0.993	79747	0.776	-0.021916	-1.65	0.3333	-2	18	-0.95	-3	1	0	0
HIP_79748	TSICSLYQ	EENELTLNE	TSICSLYQEENELTLNE		SCG1	28708/367830	0.970	79748	0.776	0.057951	-1.9167	0	-4	17	-0.6118	-4	1	1	0
HIP_79749	LGGGPGAGS	YFMSDTREE	LGGGPGAGSYFMSDTREE		SCG1	10866/367830	0.997	79749	0.776	0.103434	0.6667	0.3333	-2	18	-0.6611	-2	2	0	1
HIP_79750	GQVELGGGP	YFPNPYNQE	GQVELGGGPYFPNPYNQE		SCG2	6409/334890	0.999	79750	0.776	0.052700	-0.4167	0.6667	-2	18	-1.0667	-2	2	0	1
HIP_79751	VEALYLVCG	DEMKRSGQL	VEALYLVCGDEMKRSGQL		SCG2	25915/334890	0.993	79751	0.776	0.005775	0.2	0.1667	-1	18	0.0389	-1	1	0	0
HIP_79752	VEALYLVCG	EEEEMAVV	VEALYLVCGEEEEMAVV		CMGA	16654/247050	0.983	79752	0.776	0.018333	0.7	0.1667	-5	17	0.7765	-5	1	1	2
HIP_79753	LQPLALEGS	EPEYGEEIK	LQPLALEGSEPEYGEEIK		SCG1	23598/367830	0.983	79753	0.776	-0.127796	-2.2167	0.5	-4	18	-0.7389	-4	1	0	0
HIP_79754	LYQLENYCN	RIILEALRQ	LYQLENYCNRIILEALRQ		SCG2	26320/334890	0.993	79754	0.776	-0.104907	0.3667	0	0	18	-0.2278	0	1	0	0
HIP_79755	SLYQLENYC	NLLKELQDL	SLYQLENYCNLLKELQDL		CMGA	13958/247050	0.989	79755	0.776	-0.141560	0.3	0	-2	18	-0.3611	-2	1	0	0
HIP_79756	SICSLYQLE	QWPERKLK	SICSLYQLEQWPERKLK		SCG2	38605/334890	0.979	79756	0.776	-0.062868	-1.5333	0.1667	1	17	-0.7824	1	2	1	0
HIP_79757	AGSLQPLA	GEEIKGYPG	AGSLQPLAGEEIKGYPG		SCG1	21603/367830	0.987	79757	0.776	0.117371	-0.5667	0.3333	-1	17	-0.3294	-1	0	1	0
HIP_79758	LVEALYLVC	THAAGHSQE	LVEALYLVCTHAAGHSQE		SCG1	32421/367830	0.956	79758	0.776	0.053516	1.4	0	0	18	0.4222	-2	1	0	0

HIP_80217	LVCGERGFF	EISRNLQI	LVCGERGFFEISRNLQI	SCG2	25822/334890	0.993	80217	0.775	0.042404	[illegible]
HIP_80218	ICSLYQLE	WPERKLKHM	ICSLYQLEWPERKLKHM	SCG2	44576/334890	0.965	80218	0.775	0.049230	[illegible]
HIP_80219	CGERGFF	GLSGLTL	CGERGFFGLSGLTL	NPY	2015/49410	0.987	80219	0.775	0.039882	[illegible]
HIP_80220	LGGGPGAGS	RLFEKPLDS	LGGGPGAGSRLFEKPLDS	SCG2	15478/334890	0.998	80220	0.775	0.086265	[illegible]
HIP_80221	GIVEQCCTS	NNFGAILSS	GIVEQCCTSNNFGAILSS	IAPP	3562/45018	0.967	80221	0.775	-0.035529	[illegible]
HIP_80222	GGPGAGSLQ	GEDWNPVEE	GGPGAGSLQGEDWNPVEE	SCG2	17914/334890	0.997	80222	0.775	-0.067954	[illegible]
HIP_80223	GQVELGGGP	YFMSDTREE	GQVELGGGPYFMSDTREE	SCG1	8249/367830	0.998	80223	0.775	0.093004	[illegible]
HIP_80224	RGFFYTPKT	LTGPNNQKR	RGFFYTPKTLTGPNNQKR	SCG2	33502/334890	0.987	80224	0.775	-0.030992	[illegible]
HIP_80225	HLVEALYL	MKLSFRARA	HLVEALYLMKLSFRARA	CMGA	27542/247050	0.934	80225	0.775	0.083522	[illegible]
HIP_80226	LQPLALEGS	NTYGKRNAV	LQPLALEGSNTYGKRNAV	IAPP	3420/45018	0.970	80226	0.775	0.030019	[illegible]
HIP_80227	SHLVEALY	EKIESQTQE	SHLVEALYEKIESQTQE	SCG2	33838/334890	0.986	80227	0.775	0.166301	[illegible]
HIP_80228	HLVEALYL	PGWQAKR	HLVEALYLPGWQAKR	CMGA	20034/247050	0.972	80228	0.775	0.218026	[illegible]
HIP_80229	CGERGFFY	LPRLPYGA	CGERGFFYLPRLPYGA	SCG2	31088/334890	0.989	80229	0.775	0.128253	[illegible]
HIP_80230	YLVCGERGF	PSSREDSLE	YLVCGERGFPSSREDSLE	CMGA	10051/247050	0.995	80230	0.775	-0.043389	[illegible]
HIP_80231	HLVEALYL	APEDLEWER	HLVEALYLAPEDLEWER	SCG1	18939/367830	0.991	80231	0.775	0.091257	[illegible]
HIP_80232	QCCTSICSL	ERGLEPGKG	QCCTSICSLERGLEPGKG	SCG1	31313/367830	0.960	80232	0.775	0.194381	[illegible]
HIP_80233	GSLQPLALE	AVLALLLCA	GSLQPLALEAVLALLLCA	CMGA	16951/247050	0.982	80233	0.775	0.005326	[illegible]
HIP_80234	GPGAGSLQP	GERATRLFE	GPGAGSLQPGERATRLFE	SCG2	28543/334890	0.991	80234	0.775	0.026576	[illegible]
HIP_80235	TSICSLYQL	FPMDMSDD	TSICSLYQLFPMDMSDD	SCG2	15415/334890	0.998	80235	0.775	-0.129360	[illegible]
HIP_80236	VCGERGFF	YNPYQGVS	VCGERGFFYNPYQGVS	SCG2	29434/334890	0.991	80236	0.775	0.047337	[illegible]
HIP_80237	HLVEALYL	DNFLEGEE	HLVEALYLDNFLEGEE	SCG1	25841/367830	0.978	80237	0.775	0.097475	[illegible]
HIP_80238	GPGAGSLQP	PGWQAKRE	GPGAGSLQPPGWQAKRE	CMGA	16287/247050	0.984	80238	0.775	-0.036842	[illegible]
HIP_80239	ERGFFYTP	PYNQEKVL	ERGFFYTPPYNQEKVL	SCG2	28447/334890	0.991	80239	0.775	-0.049321	[illegible]
HIP_80240	CTSICSLY	GYPGVQAPE	CTSICSLYGYPGVQAPE	SCG1	16676/367830	0.993	80240	0.775	0.003426	[illegible]
HIP_80241	LYQLENYCN	DLQDTKENR	LYQLENYCNDLQDTKENR	SCG1	35189/367830	0.944	80241	0.775	-0.051206	[illegible]
HIP_80242	YLVCGERGF	LFNPYYDPL	YLVCGERGFLFNPYYDPL	SCG1	16659/367830	0.993	80242	0.775	-0.015168	[illegible]
HIP_80243	TSICSLYQL	KARRHPQGA	TSICSLYQLKARRHPQGA	SCG1	60695/367830	0.748	80243	0.775	0.151423	[illegible]
HIP_80244	ERGFFYTP	GPQLRRGWR	ERGFFYTPGPQLRRGWR	CMGA	21508/247050	0.967	80244	0.775	0.026783	[illegible]
HIP_80245	GAGSLQPLA	EWERYRGR	GAGSLQPLAEWERYRGR	SCG1	36034/367830	0.940	80245	0.775	0.016293	[illegible]
HIP_80246	LVCGERGFF	ARRHPQGA	LVCGERGFFARRHPQGA	SCG1	48040/367830	0.867	80246	0.775	0.086094	[illegible]
HIP_80247	HLCGSHLVE	EQKLYTDDE	HLCGSHLVEEQKLYTDDE	SCG2	36693/334890	0.983	80247	0.775	0.084458	[illegible]
HIP_80248	SHLVEALY	SSQESGEET	SHLVEALYSSQESGEET	SCG1	25760/367830	0.978	80248	0.775	0.215314	[illegible]
HIP_80249	CGSHLVEAL	TVVLNPHPS	CGSHLVEALTVVLNPHPS	CMGA	19559/247050	0.973	80249	0.775	-0.028684	[illegible]
HIP_80250	FVNQHLCGS	PQGLFRGGK	FVNQHLCGSPQGLFRGGK	CMGA	18023/247050	0.979	80250	0.775	-0.025572	[illegible]
HIP_80251	GSLQPLAL	LKATPIESH	GSLQPLALLKATPIESH	IAPP	4875/45018	0.932	80251	0.775	0.087748	[illegible]
HIP_80252	HLVEALYL	PVGPPKNDD	HLVEALYLPVGPPKNDD	SCG2	13650/334890	0.998	80252	0.775	0.116869	[illegible]
HIP_80253	SHLVEALY	PMYEENSR	SHLVEALYPMYEENSR	SCG2	22680/334890	0.995	80253	0.775	0.021459	[illegible]
HIP_80254	VEALYLVCG	ATLESVFQE	VEALYLVCGATLESVFQE	SCG2	11028/334890	0.999	80254	0.775	-0.074820	[illegible]
HIP_80255	ICSLYQLEN	LKRQYDRVA	ICSLYQLENLKRQYDRVA	SCG1	56953/367830	0.790	80255	0.775	-0.001368	[illegible]
HIP_80256	AGSLQPLA	NLITRQRYG	AGSLQPLANLITRQRYG	NPY	4289/49410	0.951	80256	0.775	0.013490	[illegible]
HIP_80257	GERGFFYT	YEKRNLARV	GERGFFYTYEKRNLARV	SCG1	47700/367830	0.869	80257	0.775	0.099104	[illegible]
HIP_80258	GERGFFYT	QEEEQIEQA	GERGFFYTQEEEQIEQA	SCG2	29842/334890	0.990	80258	0.775	0.077779	[illegible]
HIP_80259	SICSLYQLE	LYTDDEDDI	SICSLYQLELYTDDEDDI	SCG2	15439/334890	0.998	80259	0.775	-0.169461	[illegible]
HIP_80260	GSLQPLAL	ETTENEN	GSLQPLALETTENEN	SCG1	26369/367830	0.977	80260	0.775	0.194054	[illegible]
HIP_80261	VNQHLCGSH	DSSMKLSFR	VNQHLCGSHDSSMKLSFR	CMGA	23995/247050	0.956	80261	0.775	-0.067375	[illegible]
HIP_80262	SHLVEALY	KGDTEVMKC	SHLVEALYKGDTEVMKC	CMGA	22007/247050	0.964	80262	0.775	0.253608	[illegible]
HIP_80263	GGGPGAGSL	VFLVLSV	GGGPGAGSLVFLVLSV	IAPP	1835/45018	0.992	80263	0.775	0.102917	[illegible]
HIP_80264	GGPGAGSLQ	LGYKEIRKG	GGPGAGSLQLGYKEIRKG	CMGA	17668/247050	0.980	80264	0.775	-0.078915	[illegible]
HIP_80265	GAGSLQPLA	EEATNTHPP	GAGSLQPLAEEATNTHPP	CMGA	17568/247050	0.980	80265	0.775	-0.064636	[illegible]
HIP_80266	GGPGAGSL	NPGEDAPAE	GGPGAGSLNPGEDAPAE	NPY	1398/49410	0.992	80266	0.775	-0.103287	[illegible]
HIP_80267	EQCCTSIC	GGSLPSEEK	EQCCTSICGGSLPSEEK	SCG1	15468/367830	0.994	80267	0.775	0.162049	[illegible]
HIP_80268	LVEALYLVC	DRVAQLDQ	LVEALYLVCDRVAQLDQ	SCG1	32741/367830	0.954	80268	0.775	0.136862	[illegible]
HIP_80269	GIVEQCCTS	EGNNQAPGE	GIVEQCCTSEGNNQAPGE	CMGA	12577/247050	0.991	80269	0.775	-0.027733	[illegible]
[illegible]										

HIP_B0728	VCGERGFF	YWDEDLLMK	VCGERGFFYWDEDLLMK		SCG2	29013/334890	0.991	B0728	0.775	0.021101	[illegible]
HIP_B0729	CCTSICSLY	GHSRERADE	CCTSICSLYGHSRERADE		SCG1	21180/367830	0.988	B0729	0.775	0.005384	[illegible]
HIP_B0730	GAGSLQPLA	ALQGAKERA	GAGSLQPLAALQGAKERA		CMGA	19545/247050	0.974	B0730	0.775	-0.032776	[illegible]
HIP_B0731	ERGFFYTP	EEENELTL	ERGFFYTPEEENELTL		SCG1	31801/367830	0.959	B0731	0.775	-0.026591	[illegible]
HIP_B0732	SLYQLENYC	AKTHWLGAA	SLYQLENYCAKTHWLGAA		SCG2	40423/334890	0.976	B0732	0.775	-0.026036	[illegible]
HIP_B0733	SLQPLALEG	SQKYPGPQA	SLQPLALEGSQKYPGPQA		CMGA	29848/247050	0.915	B0733	0.775	0.009654	[illegible]
HIP_B0734	VCGERGFF	ELEQEEER	VCGERGFFELEQEEER		CMGA	17037/247050	0.982	B0734	0.775	-0.014258	[illegible]
HIP_B0735	NQHLCGSHL	AVDGAGKPG	NQHLCGSHLAVDGAGKPG		CMGA	27167/247050	0.937	B0735	0.775	0.004112	[illegible]
HIP_B0736	VNQHLCGSH	RDSSMKLSF	VNQHLCGSHRDSSMKLSF		CMGA	29608/247050	0.917	B0736	0.775	-0.038383	[illegible]
HIP_B0737	GSHLVEALY	DGAGKPGA	GSHLVEALYDGAGKPGA		CMGA	15342/247050	0.986	B0737	0.775	0.161612	[illegible]
HIP_B0738	VGQVELGGG	RSGQLGIQE	VGQVELGGGRSGQLGIQE		SCG2	17748/334890	0.997	B0738	0.775	-0.063251	[illegible]
HIP_B0739	SICSLYQL	VIAYLKRL	SICSLYQLVIAYLKRL		SCG2	27796/334890	0.992	B0739	0.775	-0.007185	[illegible]
HIP_B0740	ERGFFYTP	DSKRWSKMD	ERGFFYTPDSKRWSKMD		CMGA	29303/247050	0.920	B0740	0.775	0.041045	[illegible]
HIP_B0741	RGFFYTPKT	GRGGEPRAY	RGFFYTPKTGRGGEPRAY		SCG1	36076/367830	0.940	B0741	0.775	-0.003592	[illegible]
HIP_B0742	HLCGSHLVE	APPITPECR	HLCGSHLVEAPPITPECR		SCG1	24561/367830	0.981	B0742	0.775	0.013892	[illegible]
HIP_B0743	LGGGPGAGS	APGEEEEEE	LGGGPGAGSAPGEEEEEE		CMGA	5075/247050	0.998	B0743	0.775	-0.133219	[illegible]
HIP_B0744	SHLVEALY	SQETDKLA	SHLVEALYSQETDKLA		SCG2	25677/334890	0.993	B0744	0.775	0.187565	[illegible]
HIP_B0745	GSLQPLAL	NRMLSKSGY	GSLQPLALNRMLSKSGY		SCG2	42596/334890	0.971	B0745	0.775	0.207110	[illegible]
HIP_B0746	YQLENYCN	QESKAEGNN	YQLENYCNQESKAEGNN		CMGA	21044/247050	0.968	B0746	0.775	0.128077	[illegible]
HIP_B0747	EAEDLQVGQ	EPGETQNAF	EAEDLQVGQEPGETQNAF		SCG1	7960/367830	0.998	B0747	0.775	-0.119784	[illegible]
HIP_B0748	TSICSLYQL	YGEEGAPG	TSICSLYQLYGEEGAPG		SCG1	25597/367830	0.979	B0748	0.775	0.090170	[illegible]
HIP_B0749	NQHLCGSHL	APPITPECR	NQHLCGSHLAPPITPECR		SCG1	24298/367830	0.982	B0749	0.775	-0.055541	[illegible]
HIP_B0750	FVNQHLCGS	YPKTPGRAG	FVNQHLCGSYPKTPGRAG		SCG2	36042/334890	0.984	B0750	0.775	0.015199	[illegible]
HIP_B0751	GSHLVEALY	QKEPDLRL	GSHLVEALYQKEPDLRL		SCG2	35124/334890	0.985	B0751	0.775	0.202987	[illegible]
HIP_B0752	CGERGFFYT	MSDDYETQ	CGERGFFYTMSDDYETQ		SCG2	25387/334890	0.993	B0752	0.775	0.024849	[illegible]
HIP_B0753	CGERGFFYT	SLSEEDWM	CGERGFFYTSLSEEDWM		SCG2	15841/334890	0.997	B0753	0.775	-0.027345	[illegible]
HIP_B0754	ALYLVCGER	DERILSILR	ALYLVCGERDERILSILR		CMGA	29255/247050	0.920	B0754	0.775	-0.078705	[illegible]
HIP_B0755	GERGFFY	VALNHLK	GERGFFYVALNHLK		IAPP	3839/45018	0.962	B0755	0.775	-0.024175	[illegible]
HIP_B0756	CSLYQLENY	DDVSKVIAY	CSLYQLENYDDVSKVIAY		SCG2	37935/334890	0.981	B0756	0.775	-0.070651	[illegible]
HIP_B0757	TSICSLYQL	WLGAALSL	TSICSLYQLWLGAALSL		SCG2	20548/334890	0.996	B0757	0.775	-0.059203	[illegible]
HIP_B0758	ELGGGPGAG	WQAKREEEE	ELGGGPGAGWQAKREEEE		CMGA	5666/247050	0.998	B0758	0.775	0.010017	[illegible]
HIP_B0759	VEALYLVCG	SQREDEEEE	VEALYLVCGSQREDEEEE		SCG1	25711/367830	0.978	B0759	0.775	0.048095	[illegible]
HIP_B0760	HLCGSHLVE	VRDSKENIE	HLCGSHLVEVRDSKENIE		SCG2	40772/334890	0.975	B0760	0.775	0.017405	[illegible]
HIP_B0761	ERGFFYTPK	MNKGDTEVM	ERGFFYTPKMNKGDTEVM		CMGA	22347/247050	0.963	B0761	0.775	-0.063122	[illegible]
HIP_B0762	ICSLYQLEN	LRQQAHKEE	ICSLYQLENLRQQAHKEE		SCG2	44277/334890	0.966	B0762	0.775	-0.077223	[illegible]
HIP_B0763	LCGSHLVEA	RGRGGEPRA	LCGSHLVEARGRGGEPRA		SCG1	46203/367830	0.880	B0763	0.775	0.183395	[illegible]
HIP_B0764	LQPLALEGS	EERLSKEWE	LQPLALEGSEERLSKEWE		CMGA	23863/247050	0.956	B0764	0.775	-0.127014	[illegible]
HIP_B0765	QVELGGGPG	DTPNRQYWD	QVELGGGPGDTPNRQYWD		SCG2	14457/334890	0.998	B0765	0.775	-0.081827	[illegible]
HIP_B0766	GSLQPLAL	EEATNTHPP	GSLQPLALEEATNTHPP		CMGA	19738/247050	0.973	B0766	0.775	0.102014	[illegible]
HIP_B0767	QCCTSICS	QWPERKLKH	QCCTSICSQWPERKLKH		SCG2	39169/334890	0.978	B0767	0.775	0.129021	[illegible]
HIP_B0768	VEALYLVCG	RNLQIPPED	VEALYLVCGRNLQIPPED		SCG2	27900/334890	0.992	B0768	0.775	0.002685	[illegible]
HIP_B0769	CGERGFF	GPQLRRGWR	CGERGFFGPQLRRGWR		CMGA	22246/247050	0.964	B0769	0.775	0.165384	[illegible]
HIP_B0770	VEALYLVC	KGDTEVMKC	VEALYLVCKGDTEVMKC		CMGA	21803/247050	0.965	B0770	0.775	0.241276	[illegible]
HIP_B0771	VEALYLVCG	EARFQDKQ	VEALYLVCGEARFQDKQ		SCG1	37140/367830	0.934	B0771	0.775	0.038765	[illegible]
HIP_B0772	CCTSICSLY	DLIEMLKTG	CCTSICSLYDLIEMLKTG		SCG2	24289/334890	0.994	B0772	0.775	-0.068956	[illegible]
HIP_B0773	QHLCGSHLV	HSREKSSQE	QHLCGSHLVHSREKSSQE		SCG1	48306/367830	0.865	B0773	0.775	0.215148	[illegible]
HIP_B0774	LYLVCGERG	QESKGQPRS	LYLVCGERGQESKGQPRS		SCG1	45279/367830	0.886	B0774	0.775	-0.044743	[illegible]
HIP_B0775	SLYQLENYC	DNMNDNFLE	SLYQLENYCDNMNDNFLE		SCG1	24152/367830	0.982	B0775	0.775	-0.093657	[illegible]
[illegible]											

HIP_81239	LGGGPGAGS	ERGLEPGKG	LGGGPGAGSERGLEPGKG		SCG1	28885/367830	0.969	81239	0.774	-0.060273
HIP_81240	GERGFFYT	DQLAKELTA	GERGFFYTDQLAKELTA		CMGA	25450/247050	0.948	81240	0.774	0.050034
HIP_81241	QPLALEGSL	EGENYQKGE	QPLALEGSLEGENYQKGE		SCG1	17951/367830	0.992	81241	0.774	-0.092129
HIP_81242	VELGGGPGA	QNAFLNERK	VELGGGPGAQNAFLNERK		SCG1	20193/367830	0.989	81242	0.774	-0.058699
HIP_81243	VELGGGPGA	TRQRYGKRS	VELGGGPGATRQRYGKRS		NPY	6016/49410	0.898	81243	0.774	0.028100
HIP_81244	RGFFYTPKT	LNDKDQELG	RGFFYTPKTLNDKDQELG		SCG2	31811/334890	0.988	81244	0.774	-0.037010
HIP_81245	VCGERGFF	NALSKSSAP	VCGERGFFNALSKSSAP		SCG1	27935/367830	0.972	81245	0.774	0.022252
HIP_81246	RGFFYTPKT	RELDLPVDL	RGFFYTPKTRELDLPVDL		SCG2	36594/334890	0.983	81246	0.774	0.029265
HIP_81247	HLVEALYLV	TGSQENHP	HLVEALYLVTGSQENHP		SCG1	23174/367830	0.984	81247	0.774	0.210443
HIP_81248	VGQVELGGG	PPMYEENSR	VGQVELGGGPPMYEENSR		SCG2	6677/334890	0.999	81248	0.774	-0.015739
HIP_81249	VEALYLVCG	DLIEMLKTG	VEALYLVCGDLIEMLKTG		SCG2	15457/334890	0.998	81249	0.774	-0.045284
HIP_81250	VEALYLVCG	SEKSQREDE	VEALYLVCGSEKSQREDE		SCG1	28101/367830	0.972	81250	0.774	0.097283
HIP_81251	YLVCGERGF	SGELEQEEE	YLVCGERGFSGELEQEEE		CMGA	7442/247050	0.997	81251	0.774	-0.101320
HIP_81252	VNQHLCGSH	GEPRAYFMS	VNQHLCGSHGEPRAYFMS		SCG1	27932/367830	0.972	81252	0.774	-0.067505
HIP_81253	LQPLALEGS	KEEEEEMAV	LQPLALEGSKEEEEEMAV		CMGA	19356/247050	0.974	81253	0.774	-0.094524
HIP_81254	EALYLVCG	VALNHLKAT	EALYLVCGVALNHLKAT		IAPP	2577/45018	0.984	81254	0.774	0.052958
HIP_81255	GGGPGAGSL	RNQLLQKEP	GGGPGAGSLRNQLLQKEP		SCG2	39139/334890	0.979	81255	0.774	-0.024945
HIP_81256	LVEALYLV	AWKELDRNY	LVEALYLVAWKELDRNY		SCG1	37328/367830	0.933	81256	0.774	0.051511
HIP_81257	AGSLQPLA	GQNGERATR	AGSLQPLAGQNGERATR		SCG2	24671/334890	0.994	81257	0.774	0.182917
HIP_81258	GAGSLQPLA	DTKENREEA	GAGSLQPLADTKENREEA		SCG1	29267/367830	0.968	81258	0.774	0.052430
HIP_81259	SHLVEALY	ARFQDKQYS	SHLVEALYARFQDKQYS		SCG1	45542/367830	0.884	81259	0.774	0.108470
HIP_81260	TSICSLYQL	YPGVQAPE	TSICSLYQLYPGVQAPE		SCG1	14928/367830	0.995	81260	0.774	0.036882
HIP_81261	YLVCGERGF	GLVDREKGL	YLVCGERGFGLVDREKGL		CMGA	15685/247050	0.985	81261	0.774	-0.011752
HIP_81262	GERGFFYTP	GDLQDTKE	GERGFFYTPGDLQDTKE		SCG1	19936/367830	0.990	81262	0.774	-0.157185
HIP_81263	CGERGFFY	GEDWNPVE	CGERGFFYGEDWNPVE		SCG2	24815/334890	0.994	81263	0.774	0.169904
HIP_81264	LVEALYL	PDNPGED	LVEALYLPDNPGED		NPY	3347/49410	0.970	81264	0.774	0.194445
HIP_81265	LQPLALEGS	KTSRKDVKD	LQPLALEGSKTSRKDVKD		SCG1	38770/367830	0.925	81265	0.774	-0.047752
HIP_81266	VEALYLVC	LVHSSNNFG	VEALYLVCLVHSSNNFG		IAPP	4017/45018	0.957	81266	0.774	0.023825
HIP_81267	GERGFFYTP	SLGEKRDH	GERGFFYTPSLGEKRDH		SCG1	23787/367830	0.983	81267	0.774	-0.116191
HIP_81268	GAGSLQPLA	NQLLQKEPD	GAGSLQPLANQLLQKEPD		SCG2	20399/334890	0.996	81268	0.774	-0.101837
HIP_81269	NQHLCGSHL	PERKLKHMQ	NQHLCGSHLPERKLKHMQ		SCG2	45100/334890	0.963	81269	0.774	0.172071
HIP_81270	ICSLYQLE	ITRQRYGKR	ICSLYQLEITRQRYGKR		NPY	7340/49410	0.826	81270	0.774	0.056768
HIP_81271	GSLQPLAL	ESNVSMASL	GSLQPLALESNVSMASL		SCG1	27836/367830	0.973	81271	0.774	0.209005
HIP_81272	CTSICSLY	WQAKREEEE	CTSICSLYWQAKREEEE		CMGA	19552/247050	0.974	81272	0.774	-0.025382
HIP_81273	HLCGSHLV	ELTLNEKN	HLCGSHLVELTLNEKN		SCG1	30976/367830	0.962	81273	0.774	0.121402
HIP_81274	SICSLYQL	PGEEEEEE	SICSLYQLPGEEEEEE		CMGA	8558/247050	0.996	81274	0.774	0.030048
HIP_81275	GSHLVEALY	RPEDQELE	GSHLVEALYRPEDQELE		CMGA	19730/247050	0.973	81275	0.774	0.175794
HIP_81276	VCGERGFF	TNEIVEEQY	VCGERGFFTNEIVEEQY		SCG2	25302/334890	0.993	81276	0.774	0.037557
HIP_81277	SLYQLENYC	KERAHQQKK	SLYQLENYCKERAHQQKK		CMGA	37568/247050	0.829	81277	0.774	0.150235
HIP_81278	LGGGPGAGS	DSSEEKHLE	LGGGPGAGSDSSEEKHLE		SCG1	15739/367830	0.994	81278	0.774	-0.085511
HIP_81279	ERGFFYTPK	RLEGQEEEE	ERGFFYTPKRLEGQEEEE		CMGA	18431/247050	0.977	81279	0.774	-0.081867
HIP_81280	CGSHLVEAL	SALRHYIN	CGSHLVEALSALRHYIN		NPY	4842/49410	0.937	81280	0.774	0.006142
HIP_81281	QHLCGSHLV	SQENHPQE	QHLCGSHLVSQENHPQE		SCG1	31046/367830	0.961	81281	0.774	0.214842
HIP_81282	LVCGERGFF	PMPVSQEC	LVCGERGFFPMPVSQEC		CMGA	12020/247050	0.992	81282	0.774	-0.007323
HIP_81283	GGGPGAGSL	PQSLATLES	GGGPGAGSLPQSLATLES		SCG2	13119/334890	0.998	81283	0.774	-0.096239
HIP_81284	HLVEALYL	PLDSQSIY	HLVEALYLPLDSQSIY		SCG2	22946/334890	0.995	81284	0.774	0.180577
HIP_81285	VEALYLVC	RVPGQGSSE	VEALYLVCRVPGQGSSE		SCG2	29780/334890	0.990	81285	0.774	0.170547
HIP_81286	LGGGPGAGS	AALSLIPLI	LGGGPGAGSAALSLIPLI		SCG2	14728/334890	0.998	81286	0.774	0.154877
HIP_81287	ALYLVCGE	EEEEEEEEE	ALYLVCGEEEEEEEEEEE		CMGA	15715/247050	0.985	81287	0.774	-0.093813
HIP_81288	SHLVEALYL	DDTPNRQY	SHLVEALYLDDTPNRQY		SCG2	29223/334890	0.991	81288	0.774	0.195257
HIP_81289	GQVELGGGP	LLMRESTEN	GQVELGGGPLLMRESTEN		NPY	1588/49410	0.991	81289	0.774	0.141759
HIP_81290	GPGAGSLQP	RGYPEEKKE	GPGAGSLQPRGYPEEKKE		CMGA	23506/247050	0.958	81290	0.774	-0.020786

HIP_81750	GERGFFYT	AVVPQGLFR	GERGFFYTAVVPQGLFR		CMGA	19783/247050	0.973	81750	0.774	-0.035202	1.8333	0	1	17	0.0941	1	3	1	0	[illegible]
HIP_81751	SICSLYQLE	EEARFQDKQ	SICSLYQLEEEARFQDKQ		SCG1	41922/367830	0.907	81751	0.774	-0.099695	-1.4	0	-2	18	-0.9222	-2	1	0	1	[illegible]
HIP_81752	TSICSLYQL	MDKARRHPQ	TSICSLYQLMDKARRHPQ		SCG1	55993/367830	0.799	81752	0.774	0.107343	-1.0833	0	3	18	-0.75	2	1	0	0	[illegible]
HIP_81753	SHLVEALYL	AEAGEEAV	SHLVEALYLAEAGEEAV		CMGA	18917/247050	0.976	81753	0.774	0.029450	1.0667	0	-3	17	0.4294	-4	1	1	0	[illegible]
HIP_81754	CGSHLVEAL	RESTENVPR	CGSHLVEALRESTENVPR		NPY	5944/49410	0.902	81754	0.774	0.173894	-1.1167	0.1667	0	18	-0.5667	-1	0	0	0	[illegible]
HIP_81755	SICSLYQLE	GYKEIRKGE	SICSLYQLEGYKEIRKGE		CMGA	27822/247050	0.932	81755	0.774	-0.068973	-1.4667	0.1667	0	18	-0.6778	0	2	0	0	[illegible]
HIP_81756	GSLQPLALE	SAIEAELEK	GSLQPLALESAIEAELEK		CMGA	16300/247050	0.984	81756	0.774	-0.070434	1.2667	0.1667	-3	18	0.0056	-3	0	0	0	[illegible]
HIP_81757	DLQVGQVEL	EKNEQINDE	DLQVGQVELEKNEQINDE		SCG2	34804/334890	0.985	81757	0.774	0.120425	-1.0667	0	-5	18	-1.2389	-5	0	0	0	[illegible]
HIP_81758	CGSHLVEAL	GWQAKREEE	CGSHLVEALGWQAKREEE		CMGA	17203/247050	0.981	81758	0.774	0.041214	-0.45	0.1667	-1	18	-0.7611	-2	1	0	1	[illegible]
HIP_81759	LVCGERGFF	NALSKSSA	LVCGERGFFNALSKSSA		SCG1	28792/367830	0.970	81759	0.774	0.033291	1.2167	0.1667	1	17	0.2882	1	2	1	0	[illegible]
HIP_81760	NQHLCGSHL	EKNFFPEYN	NQHLCGSHLEKNFFPEYN		SCG1	45598/367830	0.884	81760	0.774	0.109008	-1.85	0.1667	1	18	-1.0944	-1	1	0	0	[illegible]
HIP_81761	YLVCGERGF	GGGHSRER	YLVCGERGFGGGHSRER		SCG1	20656/367830	0.988	81761	0.774	0.066920	-0.55	0.6667	2	17	-0.8529	1	1	1	1	[illegible]
HIP_81762	GERGFFYT	ITPECRQVL	GERGFFYTITPECRQVL		SCG1	22706/367830	0.985	81762	0.774	-0.050786	0.5	0.1667	0	17	-0.2353	0	3	1	0	[illegible]
HIP_81763	GERGFFY	ATQRLAN	GERGFFYATQRLAN		IAPP	4755/45018	0.936	81763	0.774	0.148925	0.3167	0	1	14	-0.6643	1	3	0	0	[illegible]
HIP_81764	QCCTSICSL	NLNDKDQEL	QCCTSICSLNLNDKDQEL		SCG2	22306/334890	0.995	81764	0.774	0.001373	0.3833	0.1667	-2	18	-0.4056	-2	0	0	0	[illegible]
HIP_81765	FVNQHLCG	GEGHHRVQE	FVNQHLCGGEGHHRVQE		SCG1	20696/367830	0.988	81765	0.774	0.157044	0.2667	0.5	2	17	-0.9	-1	0	1	0	[illegible]
HIP_81766	VEALYLVCG	ENLNDKDQE	VEALYLVCGENLNDKDQE		SCG2	15799/334890	0.997	81766	0.774	-0.063362	0.5167	0.1667	-4	18	-0.5278	-4	1	0	0	[illegible]
HIP_81767	PLALEGSLQ	LALQGAKER	PLALEGSLQLALQGAKER		CMGA	14708/247050	0.987	81767	0.774	0.106757	1.4833	0.1667	0	18	-0.0667	0	0	0	0	[illegible]
HIP_81768	LQPLALEGS	AFLNERKQA	LQPLALEGSAFLNERKQA		SCG1	18583/367830	0.991	81768	0.774	0.146909	0.6167	0.3333	0	18	-0.2944	0	1	0	0	[illegible]
HIP_81769	VCGERGFF	RATRLFEKP	VCGERGFFRATRLFEKP		SCG2	38219/334890	0.980	81769	0.774	0.053968	0.3	0.1667	2	17	-0.4	2	2	1	0	[illegible]
HIP_81770	GSLQPLAL	SEDDLQEEE	GSLQPLALSEDDLQEEE		SCG2	19408/334890	0.996	81770	0.774	0.121477	0.2667	0.1667	-6	17	-0.8588	-6	0	1	1	[illegible]
HIP_81771	GAGSLQPLA	GEQEHSQQK	GAGSLQPLAGEQEHSQQK		CMGA	13370/247050	0.990	81771	0.774	0.094379	-0.5667	0.3333	0	18	-1.1833	-1	0	0	0	[illegible]
HIP_81772	GAGSLQPLA	QVEKRKCNT	GAGSLQPLAQVEKRKCNT		IAPP	4632/45018	0.940	81772	0.774	0.006350	0.2	0.1667	2	18	-0.6833	2	0	0	0	[illegible]
HIP_81773	SICSLYQLE	VEPERELDL	SICSLYQLEVEPERELDL		SCG2	20774/334890	0.996	81773	0.774	-0.210711	-0.6833	0.1667	-4	18	-0.2	-4	1	0	0	[illegible]
HIP_81774	HLCGSHLVE	ELESLSAIE	HLCGSHLVEELESLSAIE		CMGA	21889/247050	0.965	81774	0.774	-0.010882	0.2167	0	-2	18	0.2778	-4	0	0	0	[illegible]
HIP_81775	TSICSLYQL	QGSSQETD	TSICSLYQLQGSSQETD		SCG2	19128/334890	0.996	81775	0.774	0.170635	-0.95	0.3333	-2	17	-0.5412	-2	1	1	0	[illegible]
HIP_81776	DLQVGQVEL	SLYQLENY	DLQVGQVELSLYQLENY		INSA	249/3024	0.866	81776	0.774	-0.018320	1.0333	0.1667	-3	17	-0.2765	-3	1	1	0	[illegible]
HIP_81777	HLVEALYL	RPEDQELES	HLVEALYLRPEDQELES		CMGA	20291/247050	0.971	81777	0.774	0.196929	-0.55	0.1667	-3	17	-0.6588	-4	1	1	0	[illegible]
HIP_81778	VCGERGFF	PGAEEAQDP	VCGERGFFPGAEEAQDP		CMGA	8081/247050	0.997	81778	0.774	0.000068	0.8333	0.5	-3	17	-0.6176	-3	2	1	0	[illegible]
HIP_81779	GERGFFYT	KGDTEVMKC	GERGFFYTKGDTEVMKC		CMGA	24858/247050	0.951	81779	0.774	0.169539	-1.1667	0.1667	0	17	-0.7353	0	3	1	0	[illegible]
HIP_81780	PGAGSLQP	LIVLSVALN	PGAGSLQPLIVLSVALN		IAPP	2371/45018	0.987	81780	0.774	0.120590	1.8667	0.1667	0	17	1.1235	0	0	1	0	[illegible]
HIP_81781	CTSICSLYQ	EEERGLEP	CTSICSLYQEEERGLEP		SCG1	32637/367830	0.955	81781	0.774	0.070379	-1.9167	0	-3	17	-0.6176	-3	1	1	1	[illegible]
HIP_81782	GPGAGSLQP	SNTYGKRN	GPGAGSLQPSNTYGKRN		IAPP	4817/45018	0.934	81782	0.774	0.049408	-1.05	0.3333	2	17	-1.2765	2	1	1	0	[illegible]
HIP_81783	ELGGGPGAG	AFLNERKQA	ELGGGPGAGAFLNERKQA		SCG1	13922/367830	0.996	81783	0.774	0.140789	1.5667	0.3333	0	18	-0.5667	0	1	0	1	[illegible]
HIP_81784	FVNQHLCGS	EEMAVVPQG	FVNQHLCGSEEMAVVPQG		CMGA	14198/247050	0.989	81784	0.774	-0.094376	-0.6333	0.3333	-1	18	0.0833	-2	0	0	0	[illegible]
HIP_81785	PLALEGSLQ	SKENIEKNE	PLALEGSLQSKENIEKNE		SCG2	35246/334890	0.985	81785	0.774	-0.054955	-1.45	0.3333	-2	18	-1.0111	-2	0	0	0	[illegible]
HIP_81786	GERGFFY	RSSPETLIS	GERGFFYRSSPETLIS		NPY	5021/49410	0.932	81786	0.774	0.228024	-0.3	0.3333	0	16	-0.5563	0	3	2	0	[illegible]
HIP_81787	SHLVEALY	AWKELDRNY	SHLVEALYAWKELDRNY		SCG1	42959/367830	0.901	81787	0.774	0.088949	0.2167	0	0	17	-0.6294	-1	2	1	0	[illegible]
HIP_81788	VEALYLVCG	QNLLKELQD	VEALYLVCGQNLLKELQD		CMGA	13174/247050	0.990	81788	0.774	-0.054334	0.5167	0.1667	-2	18	0.2833	-2	1	0	0	[illegible]
HIP_81789	VGQVELGGG	EEEAEAGEE	VGQVELGGGEEEAEAGEE		CMGA	10200/247050	0.995	81789	0.774	-0.087648	-1.95	0.5	-7	18	-0.7889	-7	0	0	2	[illegible]
HIP_81790	CSLYQLENY	LLQKEPDLR	CSLYQLENYLLQKEPDLR		SCG2	23990/334890	0.994	81790	0.774	-0.073894	-0.7	0	-1	18	-0.7167	-1	1	0	0	[illegible]
HIP_81791	ALYLVCGER	QYTPQSLAT	ALYLVCGERQYTPQSLAT		SCG2	29014/334890	0.991	81791	0.774	-0.030704	-2.3167	0.1667	0	18	-0.0056	0	1	0	0	[illegible]
HIP_81792	GSLQPLAL	NSNQVKRV	GSLQPLALNSNQVKRV		SCG2	31495/334890	0.989	81792	0.774	0.252829	0.2667	0.1667	2	16	-0.275	2	0	0	0	[illegible]

[illegible]

HIP_82261	YLVCGERGF	CIVEVISDT	YLVCGERGFCIVEVISDT		CMGA	13061/247050	0.990	82261	0.773	0.093299	1.5167	0.1667	-2	18	0.8111	-2	1
HIP_82262	VEALYLVC	EEGENYQK	VEALYLVCEEGENYQK		SCG1	24985/367830	0.980	82262	0.773	0.165369	0.5167	0.1667	-3	16	-0.475	-3	1
HIP_82263	VEALYLVCG	GSANRRPED	VEALYLVCGGSANRRPED		CMGA	8572/247050	0.996	82263	0.773	0.021253	1.15	0.5	-1	18	-0.3	-1	1
HIP_82264	HLCGSHLVE	PLDSQSIYQ	HLCGSHLVEPLDSQSIYQ		SCG2	26287/334890	0.993	82264	0.773	0.044055	0.5333	0.1667	0	18	-0.1944	-2	0
HIP_82265	GAGSLQPLA	EPRAHFMSD	GAGSLQPLAEPRAHFMSD		SCG1	28229/367830	0.971	82265	0.773	0.065331	-0.9333	0.3333	-1	18	-0.2333	-1	0
HIP_82266	GSLQPLAL	QYDRVAQL	GSLQPLALQYDRVAQL		SCG1	37553/367830	0.932	82266	0.773	0.178599	0.1833	0	0	16	0.025	0	1
HIP_82267	LQPLALEGS	TGEKPNGSV	LQPLALEGSTGEKPNGSV		SCG2	24914/334890	0.994	82267	0.773	-0.106511	-1.55	0.5	-1	18	-0.4	-1	0
HIP_82268	CGERGFFYT	PLQVRGYP	CGERGFFYTPLQVRGYP		CMGA	22065/247050	0.964	82268	0.773	0.041032	-0.0833	0.1667	1	17	-0.4471	1	2
HIP_82269	PGAGSLQP	SNTYGKRNA	PGAGSLQPSNTYGKRNA		IAPP	4818/45018	0.934	82269	0.773	0.049911	-1.05	0.3333	2	17	-1.1471	2	1
HIP_82270	ERGFFYTPK	NKGDTEVM	ERGFFYTPKNKGDTEVM		CMGA	23548/247050	0.958	82270	0.773	-0.066814	-2.3333	0.3333	0	17	-1.1588	0	2
HIP_82271	QVELGGGPG	QGAKERAHQ	QVELGGGPGQGAKERAHQ		CMGA	9749/247050	0.995	82271	0.773	0.005663	-0.75	0.6667	1	18	-1.1722	0	0
HIP_82272	NQHLCGSHL	YPGVQAPED	NQHLCGSHLYPGVQAPED		SCG1	14940/367830	0.995	82272	0.773	-0.045340	-0.5833	0.5	0	18	-0.7722	-2	1
HIP_82273	QVGQVELGG	VIAYLKRLV	QVGQVELGGVIAYLKRLV		SCG2	14684/334890	0.998	82273	0.773	0.125696	2.25	0.3333	1	18	0.7278	1	1
HIP_82274	GERGFFYT	RNYPSLELD	GERGFFYTRNYPSLELD		SCG1	43379/367830	0.899	82274	0.773	0.068353	-1.4167	0	-1	17	-0.9588	-1	4
HIP_82275	GSLQPLAL	EPGETQNA	GSLQPLALEPGETQNA		SCG1	22784/367830	0.985	82275	0.773	0.202800	0.65	0.3333	-2	16	-0.5	-2	0
HIP_82276	HLVEALYL	EDIQGPT	HLVEALYLEDIQGPT		SCG1	30649/367830	0.963	82276	0.773	0.136343	0.6333	0	-2	15	0.0467	-3	1
HIP_82277	SHLVEALYL	VSMASLGE	SHLVEALYLVSMASLGE		SCG1	24400/367830	0.981	82277	0.773	0.024512	1.9333	0.1667	-1	17	0.8706	-2	1
HIP_82278	ELGGGPGAG	DSEEPVSTH	ELGGGPGAGDSEEPVSTH		SCG1	16696/367830	0.993	82278	0.773	-0.115134	-1.1333	0.5	-3	18	-0.8278	-4	0
HIP_82279	AGSLQPLAL	IVLSVALN	AGSLQPLALIVLSVALN		IAPP	3765/45018	0.963	82279	0.773	0.020518	3.65	0	0	17	1.5706	0	0
HIP_82280	SHLVEALY	NEPQSAPK	SHLVEALYNEPQSAPK		SCG2	25945/334890	0.993	82280	0.773	0.185689	-0.7167	0.1667	0	16	-0.7375	-1	1
HIP_82281	SICSLYQLE	HYRASEEEP	SICSLYQLEHYRASEEEP		SCG1	42701/367830	0.903	82281	0.773	-0.060526	-2.0333	0	-2	18	-0.8556	-3	2
HIP_82282	HLVEALYLV	LGAVGLAA	HLVEALYLVLGAVGLAA		SCG1	24038/367830	0.982	82282	0.773	0.016318	1.9833	0.1667	0	17	1.7647	-1	1
HIP_82283	CGERGFF	EGGGHSRE	CGERGFFEGGGHSRE		SCG1	22723/367830	0.985	82283	0.773	0.152445	0.15	0.5	0	15	-1.16	-1	2
HIP_82284	AGSLQPLAL	SRNLQIPP	AGSLQPLALSRNLQIPP		SCG2	34677/334890	0.985	82284	0.773	0.233514	0.1	0.1667	1	17	0.0882	1	0
HIP_82285	IVEQCCTSI	EEPGETQNA	IVEQCCTSIEEPGETQNA		SCG1	21680/367830	0.987	82285	0.773	-0.058163	-0.9333	0.3333	-4	18	-0.4833	-4	0
HIP_82286	HLVEALYL	NGDESHLP	HLVEALYLNGDESHLP		SCG2	24266/334890	0.994	82286	0.773	0.223335	-0.1833	0.1667	-1	16	-0.2063	-3	1
HIP_82287	SLYQLENYC	MDKARRHPQ	SLYQLENYCMDKARRHPQ		SCG1	52579/367830	0.830	82287	0.773	0.013878	-1.3	0	2	18	-1.3778	1	1
HIP_82288	ICSLYQLEN	GPTVVLNPH	ICSLYQLENGPTVVLNPH		CMGA	11805/247050	0.992	82288	0.773	-0.022528	-0.9833	0.3333	0	18	0.1778	-1	1
HIP_82289	QCCTSICSL	NIEKNEQIN	QCCTSICSLNIEKNEQIN		SCG2	28386/334890	0.991	82289	0.773	0.031078	0.5	0.1667	-1	18	-0.3278	-1	0
HIP_82290	YLVCGERGF	FPDFYDSEE	YLVCGERGFFPDFYDSEE		SCG1	13376/367830	0.996	82290	0.773	-0.167953	-0.7333	0.3333	-4	18	-0.4944	-4	2
HIP_82291	GERGFFYT	VEPERELDL	GERGFFYTVEPERELDL		SCG2	19823/334890	0.996	82291	0.773	-0.028011	-0.0167	0.1667	-3	17	-0.7941	-3	3
HIP_82292	LCGSHLVEA	KSGEATDGA	LCGSHLVEAKSGEATDGA		CMGA	21229/247050	0.968	82292	0.773	0.199906	-0.4333	0.3333	-1	18	-0.0778	-2	0
HIP_82293	NQHLCGSHL	QNAFLNERK	NQHLCGSHLQNAFLNERK		SCG1	46717/367830	0.876	82293	0.773	0.024129	-0.9	0.1667	3	18	-1.0278	1	1
HIP_82294	GSLQPLALE	RESTENVPR	GSLQPLALERESTENVPR		NPY	6041/49410	0.897	82294	0.773	0.184836	-1.1167	0.1667	-1	18	-0.8333	-1	0
HIP_82295	CGSHLVEAL	SANRRPEDQ	CGSHLVEALSANRRPEDQ		CMGA	24327/247050	0.954	82295	0.773	0.050421	-0.0667	0.1667	0	18	-0.8556	-1	0
HIP_82296	LYLVCGERG	FEKPLDSQS	LYLVCGERGFEKPLDSQS		SCG2	29797/334890	0.990	82296	0.773	-0.036709	-2.1667	0.1667	-1	18	-0.3778	-1	1
HIP_82297	ERGFFYTPK	GQEEEEDN	ERGFFYTPKGQEEEEDN		CMGA	16620/247050	0.983	82297	0.773	-0.125073	-2.2667	0.3333	-4	17	-2.0706	-4	2
HIP_82298	GIVEQCCTS	PKTPGRAGT	GIVEQCCTSPKTPGRAGT		SCG2	38918/334890	0.979	82298	0.773	0.043960	-0.8667	0.3333	1	18	-0.4	1	0
HIP_82299	QVGQVELGG	LALSLLVCL	QVGQVELGGLALSLLVCL		NPY	1760/49410	0.989	82299	0.773	0.126670	2.0667	0.3333	1	18	1.5111	-1	0
HIP_82300	GERGFFY	EEEATNT	GERGFFYEEEATNT		CMGA	20951/247050	0.969	82300	0.773	0.126743	-1.0333	0	-3	14	-1.2929	-3	3
HIP_82301	YLVCGERGF	QALPEPMQE	YLVCGERGFQALPEPMQE		CMGA	11388/247050	0.993	82301	0.773	0.026094	0	0.1667	-2	18	-0.3611	-2	1
HIP_82302	AGSLQPLAL	TLESVFQE	AGSLQPLALTLESVFQE		SCG2	25150/334890	0.994	82302	0.773	0.136051	1.5	0	-2	17	0.4412	-2	0
HIP_82303	GGGPGAGSL	PPITPECRQ	GGGPGAGSLPPITPECRQ		SCG1	15481/367830	0.994	82303	0.773	-0.070501	0.65	0.6667	0	18	-0.4889	0	0
HIP_82304	GSLQPLAL	AEKGREHIA	GSLQPLALAEKGREHIA		SCG2	39328/334890	0.978	82304	0.773	0.206032	0.6333	0	1	17	-0.2353	0	0
HIP_82305	RGFFYTPKT	GQVTALPVN	RGFFYTPKTGQVTALPVN		CMGA	21900/247050	0.965	82305	0.773	-0.033852	-0.9833	0.3333	2	18	-0.1778	2	1
HIP_82306	LQPLALEGS	FPNPYNQEK	LQPLALEGSFPNPYNQEK		SCG2	12133/334890	0.998	82306	0.773	-0.089369	-1.1667	0.5	-1	18	-0.9	-1	1
HIP_82307	VEALYLVCG	ARPQALPEP	VEALYLVCGARPQALPEP		CMGA	19292/247050	0.974	82307	0.773	0.008131	0.3333	0.3333	-1	18	0.3444	-1	1
HIP_82308	GERGFFYT	LLCAGQVTA	GERGFFYTLLCAGQVTA		CMGA	18853/247050	0.976	82308	0.773	-0.046794	1.8167	0	0	17	0.4765	0	3

[illegible]

HIP_82772	RGFFYTPKT	SEALAVDGA	RGFFYTPKTSEALAVDGA		CMGA	23228/247050	0.959	82772	0.773	-0.077775	-1.45	0.3333	0	18	-0.1278	0	1	0	0	[illegible]
HIP_82773	GSLQPLALE	FRGPGPQLR	GSLQPLALEFRGPGPQLR		CMGA	26350/247050	0.942	82773	0.773	0.097263	0	0.1667	1	18	-0.3611	1	1	0	0	[illegible]
HIP_82774	EQCCTSICS	LKTSRKDVK	EQCCTSICSLKTSRKDVK		SCG1	46445/367830	0.878	82774	0.773	0.047923	0.9	0.1667	2	18	-0.5833	2	0	0	0	[illegible]
HIP_82775	RGFFYTPKT	ECRQVLKTS	RGFFYTPKTECRQVLKTS		SCG1	49452/367830	0.856	82775	0.773	0.065163	-1.95	0.1667	3	18	-0.7722	3	1	0	0	[illegible]
HIP_82776	GIVEQCCTS	QWKSSHFER	GIVEQCCTSQWKSSHFER		SCG1	50111/367830	0.850	82776	0.773	0.035557	-1.2167	0.1667	1	18	-0.75	0	1	0	0	[illegible]
HIP_82777	HLVEALYLV	APGEEEEE	HLVEALYLVAPGEEEEE		CMGA	6965/247050	0.997	82777	0.773	0.095412	1.0833	0.3333	-5	17	-0.2412	-6	1	1	3	[illegible]
HIP_82778	GERGFFYT	NVSMASLGE	GERGFFYTNVSMASLGE		SCG1	22500/367830	0.985	82778	0.773	-0.053312	0.1167	0.1667	-1	17	-0.1471	-1	3	1	0	[illegible]
HIP_82779	GERGFFYT	LQGAKERAH	GERGFFYTLQGAKERAH		CMGA	20916/247050	0.969	82779	0.773	0.076101	0.1167	0.1667	2	17	-0.9882	1	3	1	0	[illegible]
HIP_82780	LVCGERGFF	LAAVNSMP	LVCGERGFFLAAVNSMP		SCG1	22073/367830	0.986	82780	0.773	0.013493	2.1	0.1667	0	17	0.8765	0	2	1	0	[illegible]
HIP_82781	GSLQPLAL	QNGQNGER	GSLQPLALQNGQNGER		SCG2	26735/334890	0.993	82781	0.773	0.235210	0.3333	0.1667	0	16	-0.9938	0	0	0	0	[illegible]
HIP_82782	VEALYLVCG	WQAKREEE	VEALYLVCGWQAKREEE		CMGA	18298/247050	0.978	82782	0.773	0.000073	0.6167	0.1667	-2	17	-0.3765	-2	2	1	1	[illegible]
HIP_82783	ALYLVCGER	GEEENELTL	ALYLVCGERGEEENELTL		SCG1	30247/367830	0.965	82783	0.773	-0.129417	-2.6333	0.3333	-4	18	-0.2556	-4	0	0	1	[illegible]
HIP_82784	VNQHLCGSH	RGSEEYRAP	VNQHLCGSHRGSEEYRAP		SCG1	32936/367830	0.954	82784	0.773	-0.052011	-1.6833	0.6667	2	18	-1.2444	0	0	0	0	[illegible]
HIP_82785	HLVEALYLV	TAEKRLEG	HLVEALYLVTAEKRLEG		CMGA	22109/247050	0.964	82785	0.773	0.160906	0.7167	0	0	17	0.1588	-1	1	1	0	[illegible]
HIP_82786	DLQVGQVEL	LGEGHHRV	DLQVGQVELLGEGHHRV		SCG1	24734/367830	0.981	82786	0.773	0.092619	0.7333	0.1667	0	17	-0.3294	-2	0	1	0	[illegible]
HIP_82787	CSLYQLENY	NAVEVLKRE	CSLYQLENYNAVEVLKRE		IAPP	3301/45018	0.972	82787	0.773	-0.041696	-0.9667	0	-1	18	-0.4833	-1	1	0	0	[illegible]
HIP_82788	GPGAGSLQP	ESNVSMASL	GPGAGSLQPESNVSMASL		SCG1	26102/367830	0.977	82788	0.773	-0.079662	-1.5167	0.3333	-1	18	-0.0444	-1	0	0	0	[illegible]
HIP_82789	ERGFFYTPK	DLPVDLDDI	ERGFFYTPKDLPVDLDDI		SCG2	21658/334890	0.995	82789	0.773	-0.149511	-1.25	0.3333	-3	18	-0.5333	-3	2	0	0	[illegible]
HIP_82790	AEDLQVGQV	AEAKTHWLG	AEDLQVGQVAEAKTHWLG		SCG2	14518/334890	0.998	82790	0.773	0.019649	0.0667	0.1667	-1	18	-0.3111	-2	0	0	0	[illegible]
HIP_82791	CSLYQLENY	EEEEEEAE	CSLYQLENYEEEEEEAE		CMGA	21365/247050	0.967	82791	0.773	-0.052484	-3.1333	0	-8	17	-1.5588	-8	1	1	4	[illegible]
HIP_82792	VELGGGPGA	EHSQQKEEE	VELGGGPGAEHSQQKEEE		CMGA	11386/247050	0.993	82792	0.773	-0.120176	-1.2833	0.5	-3	18	-1.4333	-4	0	0	2	[illegible]
HIP_82793	GSLQPLALE	SGAEAASFQ	GSLQPLALESGAEAASFQ		SCG2	13666/334890	0.998	82793	0.773	0.055218	0.45	0.3333	-2	18	0.1444	-2	0	0	0	[illegible]
HIP_82794	HLVEALYL	SKEWEDSK	HLVEALYLSKEWEDSK		CMGA	24986/247050	0.950	82794	0.773	0.222897	-0.3167	0.1667	-1	16	-0.7125	-2	2	0	0	[illegible]
HIP_82795	GIVEQCCTS	WQAKREEEE	GIVEQCCTSWQAKREEEE		CMGA	13392/247050	0.990	82795	0.773	-0.037523	-0.2667	0.1667	-3	18	-1.1222	-3	1	0	2	[illegible]
HIP_82796	GAGSLQPL	VALNHLKAT	GAGSLQPLVALNHLKAT		IAPP	2802/45018	0.981	82796	0.773	0.106103	1.4167	0.1667	2	17	0.4	1	0	1	0	[illegible]
HIP_82797	LYQLENYCN	REKGLSAEP	LYQLENYCNREKGLSAEP		CMGA	33688/247050	0.878	82797	0.773	0.154880	-2.3667	0	-1	18	-1.0611	-1	1	0	0	[illegible]
HIP_82798	PGAGSLQPL	VVPQGLFRG	PGAGSLQPLVVPQGLFRG		CMGA	13834/247050	0.989	82798	0.773	-0.007270	0.9167	0.3333	1	18	0.3167	1	0	0	0	[illegible]
HIP_82799	EAEDLQVGQ	KSGYPKTPG	EAEDLQVGQKSGYPKTPG		SCG2	21954/334890	0.995	82799	0.773	-0.017212	-0.8	0.5	-1	18	-1.2611	-1	1	0	0	[illegible]
HIP_82800	LYQLENYCN	VQENQMDKA	LYQLENYCNVQENQMDKA		SCG1	31549/367830	0.960	82800	0.773	-0.138458	-0.85	0	-2	18	-1.1111	-2	1	0	0	[illegible]
HIP_82801	YLVCGERGF	SSRGEAGAP	YLVCGERGFSSRGEAGAP		SCG1	34158/367830	0.948	82801	0.773	0.042607	-1.3667	0.5	0	18	-0.2889	0	1	0	0	[illegible]
HIP_82802	QHLCGSHLV	VLNPHPSLG	QHLCGSHLVVLNPHPSLG		CMGA	23128/247050	0.960	82802	0.773	0.029883	1.55	0	3	18	0.2167	0	0	0	0	[illegible]
HIP_82803	SLQPLALEG	QEKVLPRLP	SLQPLALEGQEKVLPRLP		SCG2	43115/334890	0.969	82803	0.773	-0.065939	-1.8333	0.1667	0	18	-0.1889	0	0	0	0	[illegible]
HIP_82804	QCCTSICSL	IAKRAMENM	QCCTSICSLIAKRAMENM		SCG2	35603/334890	0.984	82804	0.773	0.023408	1.3167	0.1667	1	18	0.3611	1	0	0	0	[illegible]
HIP_82805	CGERGFFYT	ENRQMAYE	CGERGFFYTENRQMAYE		SCG2	39116/334890	0.979	82805	0.773	0.145417	-1.7833	0	-1	17	-1.1059	-1	3	1	0	[illegible]
HIP_82806	GSLQPLALE	GKRSSPETL	GSLQPLALEGKRSSPETL		NPY	5874/49410	0.904	82806	0.773	0.155595	-1.1167	0.1667	0	18	-0.5	0	0	0	0	[illegible]
HIP_82807	AEDLQVGQV	WIPHVENRQ	AEDLQVGQVWIPHVENRQ		SCG2	7073/334890	0.999	82807	0.773	-0.051950	0.3833	0.3333	-1	18	-0.6889	-2	1	0	0	[illegible]
HIP_82808	HLVEALYL	EDWNPVEE	HLVEALYLEDWNPVEE		SCG2	27924/334890	0.992	82808	0.773	0.099302	-0.2667	0	-4	16	-0.4	-5	2	0	0	[illegible]
HIP_82809	LVCGERGF	LLMRESTEN	LVCGERGFLLMRESTEN		NPY	2837/49410	0.978	82809	0.773	0.099621	1.2333	0.1667	-1	17	-0.1471	-1	1	1	0	[illegible]
HIP_82810	EQCCTSICS	EKAEKGREH	EQCCTSICSEKAEKGREH		SCG2	44566/334890	0.965	82810	0.773	0.057104	0.1	0.1667	0	18	-1.2167	-1	0	0	0	[illegible]
HIP_82811	HLVEALYL	KEESSPDY	HLVEALYLKEESSPDY		SCG2	32430/334890	0.988	82811	0.773	0.209081	-0.7667	0	-2	16	-0.5938	-3	1	0	0	[illegible]
HIP_82812	CGSHLVEAL	LSDDVSKVI	CGSHLVEALLSDDVSKVI		SCG2	26447/334890	0.993	82812	0.773	-0.018003	0.2667	0.1667	-1	18	0.6889	-2	0	0	0	[illegible]
HIP_82813	HLVEALYL	RWSKMDQLA	HLVEALYLRWSKMDQLA		CMGA	27419/247050	0.935	82813	0.773	0.168347	0.0167	0.1667	1	17	-0.0118	0	2	1	0	[illegible]
HIP_82814	GGGPGAGSL	SSQAELKEA	GGGPGAGSLSSQAELKEA		CMGA	11674/247050	0.993	82814	0.773	-0.067448	-0.4167	0.6667	-1	18	-0.4111	-1	0	0	1	[illegible]

HIP_83283	NQHLCGSHL	EEEEEATN	NQHLCGSHLEEEEEATN		CMGA	20132/247050	0.972	83283	0.772	0.016066	-1.7833	0.1667	-3	17
HIP_83284	SHLVEALY	LEGQEEEED	SHLVEALYLEGQEEEED		CMGA	10443/247050	0.994	83284	0.772	0.081259	0.7	0.1667	-6	17
HIP_83285	CGERGFFY	QREDIEEE	CGERGFFYQREDIEEE		SCG1	34196/367830	0.948	83285	0.772	0.160444	-1.2	0	-5	16
HIP_83286	CGSHLVEAL	QIPPEDLIE	CGSHLVEALQIPPEDLIE		SCG2	15691/334890	0.997	83286	0.772	-0.065300	0.25	0.1667	-3	18
HIP_83287	CCTSICSLY	SKMDQLAKE	CCTSICSLYSKMDQLAKE		CMGA	24641/247050	0.952	83287	0.772	-0.013339	-0.1833	0.3333	0	18
HIP_83288	SLYQLENYC	LIEISRNLQ	SLYQLENYCLIEISRNLQ		SCG2	28069/334890	0.992	83288	0.772	-0.128913	0.4167	0	-1	18
HIP_83289	LVCGERGFF	ASAIKKEE	LVCGERGFFASAIKKEE		SCG1	23936/367830	0.983	83289	0.772	-0.029012	1.3333	0.3333	0	17
HIP_83290	LVCGERGF	QWSLYPSDS	LVCGERGFQWSLYPSDS		SCG1	27292/367830	0.974	83290	0.772	-0.049849	-1.2167	0.3333	-1	17
HIP_83291	SHLVEALYL	DPADASEA	SHLVEALYLDPADASEA		SCG1	24120/367830	0.982	83291	0.772	0.151645	0.5	0.1667	-3	17
HIP_83292	GGPGAGSLQ	SDDYETQQW	GGPGAGSLQSDDYETQQW		SCG2	26390/334890	0.993	83292	0.772	-0.059411	-1.3833	0.3333	-3	18
HIP_83293	CTSICSLYQ	QQAHKEES	CTSICSLYQQQAHKEES		SCG2	37535/334890	0.981	83293	0.772	0.037178	-1.0333	0	0	17
HIP_83294	QHLCGSHLV	RVAQLDQLL	QHLCGSHLVRVAQLDQLL		SCG1	42872/367830	0.902	83294	0.772	0.075260	1.05	0	2	18
HIP_83295	PGAGSLQPL	GPNNQKRE	PGAGSLQPLGPNNQKRE		SCG2	23920/334890	0.994	83295	0.772	0.038031	-1.1333	0.5	1	17
HIP_83296	VNQHLCGS	GEEEEEEEE	VNQHLCGSGEEEEEEEE		CMGA	11353/247050	0.993	83296	0.772	-0.103966	-1.0167	0.5	-7	17
HIP_83297	ALYLVCGER	HRGRGGEPR	ALYLVCGERHRGRGGEPR		SCG1	45309/367830	0.886	83297	0.772	-0.016748	-2.75	0.3333	3	18
HIP_83298	HLVEALYLV	GEAGAPGE	HLVEALYLVGEAGAPGE		SCG1	19996/367830	0.989	83298	0.772	0.155497	0.7667	0.1667	-2	17
HIP_83299	ERGFFYTPK	SLLVCLGAL	ERGFFYTPKSLLVCLGAL		NPY	3720/49410	0.963	83299	0.772	-0.004469	0.1	0.3333	1	18
HIP_83300	GERGFFYT	TQNAFLNE	GERGFFYTTQNAFLNE		SCG1	37072/367830	0.935	83300	0.772	0.118414	-1.15	0	-1	16
HIP_83301	CCTSICSLY	RTNEIVEEQ	CCTSICSLYRTNEIVEEQ		SCG2	33383/334890	0.987	83301	0.772	0.045351	-1.1667	0.1667	-2	18
HIP_83302	ERGFFYTP	ALAEAYPSK	ERGFFYTPALAEAYPSK		NPY	2892/49410	0.977	83302	0.772	0.017465	0.6333	0.1667	0	17
HIP_83303	GERGFFYTP	NDNFLEGE	GERGFFYTPNDNFLEGE		SCG1	33900/367830	0.950	83303	0.772	-0.024173	-2.35	0.1667	-3	17
HIP_83304	CTSICSLYQ	SEVLENQSS	CTSICSLYQSEVLENQSS		CMGA	15658/247050	0.985	83304	0.772	-0.095737	-0.1833	0.1667	-2	18
HIP_83305	DLQVGQVEL	KEEEEEMAV	DLQVGQVELKEEEEEMAV		CMGA	21949/247050	0.965	83305	0.772	0.097716	-1.0667	0	-6	18
HIP_83306	GIVEQCCT	DTEKWAEGG	GIVEQCCTDTEKWAEGG		SCG1	31495/367830	0.960	83306	0.772	0.107870	-0.5667	0	-3	17
HIP_83307	FVNQHLCGS	KTHWLGAAL	FVNQHLCGSKTHWLGAAL		SCG2	40401/334890	0.976	83307	0.772	0.035753	-1.0833	0.3333	3	18
HIP_83308	CTSICSLYQ	EPGKGRHH	CTSICSLYQEPGKGRHH		SCG1	30585/367830	0.963	83308	0.772	0.185769	-1.0833	0.3333	3	17
HIP_83309	GPGAGSLQ	EEEEEEEEE	GPGAGSLQEEEEEEEEE		CMGA	17082/247050	0.982	83309	0.772	-0.055608	-1.8333	0.1667	-9	17
HIP_83310	EALYLVCG	GGGHSRERA	EALYLVCGGGGHSRERA		SCG1	16567/367830	0.993	83310	0.772	0.149189	0.85	0.6667	1	17
HIP_83311	CCTSICSLY	ERRDNMNDN	CCTSICSLYERRDNMNDN		SCG1	47851/367830	0.868	83311	0.772	0.084843	-1.8	0.1667	-1	18
HIP_83312	LVEALYLV	KRNYPSLEL	LVEALYLVKRNYPSLEL		SCG1	43919/367830	0.895	83312	0.772	0.177223	-0.8667	0	0	17
HIP_83313	AGSLQPLAL	IESHQVEK	AGSLQPLALIESHQVEK		IAPP	3373/45018	0.971	83313	0.772	0.151603	1.6	0.1667	0	17
HIP_83314	GGGPGAGSL	EDSKRWSK	GGGPGAGSLEDSKRWSK		CMGA	22623/247050	0.962	83314	0.772	0.006987	-0.8667	0.5	1	17
HIP_83315	GERGFFYTP	KSSAPPIT	GERGFFYTPKSSAPPIT		SCG1	36113/367830	0.940	83315	0.772	-0.008413	-1.5167	0.5	1	17
HIP_83316	YQLENYCN	PGEEEEEE	YQLENYCNPGEEEEEE		CMGA	9650/247050	0.995	83316	0.772	0.036542	-1.3	0.3333	-7	16
HIP_83317	ERGFFYTP	NQGSSQETD	ERGFFYTPNQGSSQETD		SCG2	22435/334890	0.995	83317	0.772	-0.046253	-1.8333	0.3333	-2	17
HIP_83318	CGERGFFYT	ATNTHPPA	CGERGFFYTATNTHPPA		CMGA	26517/247050	0.941	83318	0.772	0.062938	-0.2667	0	1	17
HIP_83319	CGSHLVEAL	PQSLATLES	CGSHLVEALPQSLATLES		SCG2	22838/334890	0.995	83319	0.772	0.104455	-0.6333	0.3333	-1	18
HIP_83320	CTSICSLY	EEEEEATNT	CTSICSLYEEEEEATNT		CMGA	19264/247050	0.974	83320	0.772	0.028816	-1.4667	0.1667	-5	17
HIP_83321	TSICSLYQL	ERGEDSSEE	TSICSLYQLERGEDSSEE		SCG1	26722/367830	0.976	83321	0.772	0.154018	-1.5667	0.1667	-4	18
HIP_83322	VEALYLVC	KVLEYLNQE	VEALYLVCKVLEYLNQE		SCG2	30934/334890	0.989	83322	0.772	0.056942	2.4333	0	-2	17
HIP_83323	LCGSHLVEA	FQNRMLSK	LCGSHLVEAFQNRMLSK		SCG2	47126/334890	0.955	83323	0.772	0.134049	-0.2833	0	2	17
HIP_83324	VEALYLVCG	EDIQGPTKA	VEALYLVCGEDIQGPTKA		SCG1	24592/367830	0.981	83324	0.772	-0.021856	0.6333	0.1667	-2	18
HIP_83325	CSLYQLENY	NTATCATQR	CSLYQLENYNTATCATQR		IAPP	4658/45018	0.939	83325	0.772	-0.075046	-1.7833	0	0	18

HIP_83794	CGERGFFYT	NRQMAYEN	CGERGFFYTNRQMAYEN		SCG2	41254/334890	0.974	83794	0.772	0.140694	-1.7833	0	0	17	-1.1059	0	3	1	0
HIP_83795	TSICSLYQL	HPQEESEES	TSICSLYQLHPQEESEES		SCG1	28563/367830	0.970	83795	0.772	0.025304	-1.55	0.1667	-3	18	-0.9111	-4	1	0	0
HIP_83796	CGERGFFYT	MDQLAKEL	CGERGFFYTMDQLAKEL		CMGA	25564/247050	0.947	83796	0.772	0.033563	-0.7167	0	-1	17	-0.3412	-1	3	1	0
HIP_83797	ALYLVCGER	YPEEKKEEE	ALYLVCGERYPEEKKEEE		CMGA	17619/247050	0.980	83797	0.772	-0.108942	-2.4667	0.3333	-3	18	-1.2111	-3	1	0	1
HIP_83798	LGGGPGAGS	GPTVVLNPH	LGGGPGAGSGPTVVLNPH		CMGA	11321/247050	0.993	83798	0.772	-0.072558	-0.35	0.6667	1	18	0.1333	0	0	0	1
HIP_83799	LVEALYLV	GAKERAHQ	LVEALYLVGAKERAHQ		CMGA	22318/247050	0.963	83799	0.772	0.213842	0.7	0.1667	1	16	0.0875	0	1	0	0
HIP_83800	QLENYCN	VALNHLKA	QLENYCNVALNHLKA		IAPP	3598/45018	0.966	83800	0.772	0.001592	1.25	0	1	15	-0.28	0	1	1	0
HIP_83801	VELGGGPGA	EQCCTSICS	VELGGGPGAEQCCTSICS		INSA	95/3024	0.979	83801	0.772	-0.132594	-0.7833	0.3333	-2	18	0.3222	-2	0	0	1
HIP_83802	QVELGGGPG	PSQKYPGPQ	QVELGGGPGPSQKYPGPQ		CMGA	14487/247050	0.988	83802	0.772	-0.068291	-1.3833	0.8333	0	18	-1.1333	0	0	0	1
HIP_83803	HLCGSHLVE	RVPGQGSSE	HLCGSHLVERVPGQGSSE		SCG2	38128/334890	0.980	83803	0.772	0.070214	0.4333	0.1667	1	18	-0.45	-1	0	0	0
HIP_83804	SHLVEALY	LNERKQASA	SHLVEALYLNERKQASA		SCG1	41905/367830	0.907	83804	0.772	0.208825	0.1833	0	1	17	-0.4412	0	1	1	0
HIP_83805	DLQVGQVEL	SEGLSQGLV	DLQVGQVELSEGLSQGLV		CMGA	14262/247050	0.988	83805	0.772	0.168578	-0.0333	0.3333	-3	18	0.2222	-3	0	0	0
HIP_83806	CCTSICSL	GSEEYRAPR	CCTSICSLGSEEYRAPR		SCG1	24208/367830	0.982	83806	0.772	0.155805	0.1333	0.5	0	17	-0.2824	0	1	1	0
HIP_83807	LCGSHLVEA	GRAGTEALP	LCGSHLVEAGRAGTEALP		SCG2	34730/334890	0.985	83807	0.772	0.129884	-0.1	0.1667	0	18	0.25	-1	0	0	0
HIP_83808	QCCTSICSL	ASEAHESSS	QCCTSICSLASEAHESSS		SCG1	21257/367830	0.987	83808	0.772	0.000598	0.5	0.3333	-1	18	0.0111	-2	0	0	1
HIP_83809	PLALEGSLQ	EGHHRVQEN	PLALEGSLQEGHHRVQEN		SCG1	29588/367830	0.967	83809	0.772	-0.129170	-1.2667	0.3333	0	18	-0.9833	-2	0	0	0
HIP_83810	AEDLQVGQV	FPPMYEENS	AEDLQVGQVFPPMYEENS		SCG2	5069/334890	1.000	83810	0.772	-0.183824	-0.0167	0.5	-4	18	-0.6389	-4	1	0	0
HIP_83811	VGQVELGGG	AAWIPHVEN	VGQVELGGGAAWIPHVEN		SCG2	8705/334890	0.999	83811	0.772	0.114018	0.25	0.5	-1	18	0.1778	-2	1	0	1
HIP_83812	SLQPLALEG	PYGAGRSRS	SLQPLALEGPYGAGRSRS		SCG2	23707/334890	0.994	83812	0.772	-0.089058	-0.5667	0.5	1	18	-0.5056	1	1	0	0
HIP_83813	SLQPLAL	SLYPSDSQ	SLQPLALSLYPSDSQ		SCG1	29871/367830	0.966	83813	0.772	0.071357	1.85	0.1667	-1	15	-0.08	-1	1	1	0
HIP_83814	GERGFFYT	PVGPPKNDD	GERGFFYTPVGPPKNDD		SCG2	11779/334890	0.998	83814	0.772	-0.041701	0.5	0.3333	-1	17	-1.2118	-1	3	1	0
HIP_83815	SHLVEALYL	KENIEKNE	SHLVEALYLKENIEKNE		SCG2	34899/334890	0.985	83815	0.772	0.223901	-0.7667	0	-1	17	-0.7176	-2	1	1	0
HIP_83816	VELGGGPGA	YYSALRHYI	VELGGGPGAYYSALRHYI		NPY	2590/49410	0.981	83816	0.772	0.004318	-0.6	0.5	1	18	0.0444	0	2	0	1
HIP_83817	ELGGGPGAG	WGYEKRNLA	ELGGGPGAGWGYEKRNLA		SCG1	15899/367830	0.994	83817	0.772	-0.004497	-0.2667	0.5	0	18	-0.7722	0	2	0	1
HIP_83818	PLALEGSLQ	NVPRTRLED	PLALEGSLQNVPRTRLED		NPY	2576/49410	0.981	83818	0.772	-0.154453	-0.2333	0.3333	-1	18	-0.5778	-1	0	0	0
HIP_83819	QCCTSICS	EESWDEEDK	QCCTSICSEESWDEEDK		SCG1	26847/367830	0.975	83819	0.772	0.074842	-0.2667	0.3333	-5	17	-1.2	-5	1	1	0
HIP_83820	LVEALYLV	LITRQRYGK	LVEALYLVLITRQRYGK		NPY	4581/49410	0.944	83820	0.772	0.024024	2.3833	0	2	17	0.3706	2	1	1	0
HIP_83821	GSLQPLALE	FEVRLLRDP	GSLQPLALEFEVRLLRDP		SCG1	35816/367830	0.941	83821	0.772	-0.042301	0.9333	0	-1	18	0.0222	-1	1	0	0
HIP_83822	HLVEALY	CNTATCAT	HLVEALYCNTATCAT		IAPP	4166/45018	0.953	83822	0.772	0.205216	0.4333	0	0	15	0.5733	-1	1	1	0
HIP_83823	QPLALEGSL	SKRFPVGPP	QPLALEGSLSKRFPVGPP		SCG2	41808/334890	0.973	83823	0.772	0.015641	-1.1	0.5	1	18	-0.2222	1	1	0	0
HIP_83824	GGPGAGSLQ	ALQGAKERA	GGPGAGSLQALQGAKERA		CMGA	20551/247050	0.970	83824	0.772	0.002603	0.2667	0.1667	1	18	-0.4722	1	0	0	0
HIP_83825	CGERGFFYT	NNFGAILS	CGERGFFYTNNFGAILS		IAPP	4726/45018	0.937	83825	0.772	0.073680	-0.5667	0	0	17	0.1176	0	4	1	0
HIP_83826	VELGGGPGA	QYSSHHTAE	VELGGGPGAQYSSHHTAE		SCG1	20320/367830	0.989	83826	0.772	-0.068607	-0.9667	0.5	0	18	-0.6722	-2	1	0	1
HIP_83827	LVCGERGFF	VCLGALAE	LVCGERGFFVCLGALAE		NPY	2477/49410	0.982	83827	0.772	0.004735	2.6167	0.1667	-1	17	1.2529	-1	2	1	0
HIP_83828	GSLQPLALE	ELEQEEERL	GSLQPLALEELEQEEERL		CMGA	20453/247050	0.970	83828	0.772	-0.049420	-0.1833	0	-5	18	-0.8056	-5	0	0	1
HIP_83829	VCGERGFF	MAYENLNDK	VCGERGFFMAYENLNDK		SCG2	20195/334890	0.996	83829	0.772	0.037415	1.2667	0.1667	-1	17	-0.4824	-1	3	1	0
HIP_83830	AEDLQVGQV	NQEKAEKGR	AEDLQVGQVNQEKAEKGR		SCG2	29630/334890	0.990	83830	0.772	-0.034385	-1.7	0.1667	-1	18	-1.4056	-1	0	0	0
HIP_83831	CGERGFFY	PPITPECR	CGERGFFYPPITPECR		SCG1	23684/367830	0.983	83831	0.772	0.149876	0.9333	0.3333	0	16	-0.5312	0	3	0	0
HIP_83832	QCCTSICSL	EGMVTRCII	QCCTSICSLEGMVTRCII		SCG1	20233/367830	0.989	83832	0.772	0.046004	0.5833	0.3333	0	18	1.0278	0	0	0	0
HIP_83833	LCGSHLVEA	GLVDREKGL	LCGSHLVEAGLVDREKGL		CMGA	17783/247050	0.980	83833	0.772	-0.016384	1.6833	0.1667	0	18	0.2111	-1	0	0	0
HIP_83834	VEALYLVC	SEEERGLE	VEALYLVCSEEERGLE		SCG1	26092/367830	0.977	83834	0.772	0.148517	0.45	0.1667	-4	16	-0.025	-4	1	0	1
HIP_83835	VEALYLVC	ESEEERGL	VEALYLVCESEEERGL		SCG1	26093/367830	0.977	83835	0.772	0.148517	0.45	0.1667	-4	16	-0.025	-4	1	0	1
HIP_83836	SICSLYQLE	LNLLGMESA	SICSLYQLELNLLGMESA		SCG2	19682/334890	0.996	83836	0.772	-0.124209	0.15	0	-2	18	0.6444	-2	1	0	0

[illegible]

HIP_84305	CGERGFFYT	QKENGDES	CGERGFFYTQKENGDES		SCG2	38723/334890	0.979	84305	0.771	0.134112	-1.6833	0	-2	17	-1.4882	-2	3
HIP_84306	GSHLVEALY	IESQTQEE	GSHLVEALYIESQTQEE		SCG2	18972/334890	0.996	84306	0.771	0.042015	0.75	0.1667	-3	17	-0.5941	-4	1
HIP_84307	VCGERGFF	LHYRKKSAE	VCGERGFFLHYRKKSAE		SCG1	38241/367830	0.928	84307	0.771	0.094675	0.75	0.1667	3	17	-0.7059	2	3
HIP_84308	HLCGSHLVE	MQPTLLLSL	HLCGSHLVEMQPTLLLSL		SCG1	33181/367830	0.952	84308	0.771	0.047142	0.2167	0.1667	1	18	0.7611	-1	0
HIP_84309	GGPGAGSLQ	TALPVNSPM	GGPGAGSLQTALPVNSPM		CMGA	16236/247050	0.984	84309	0.771	0.011326	0.7333	0.1667	0	18	0.0889	0	0
HIP_84310	GERGFFYT	ELTLNEKNF	GERGFFYTELTLNEKNF		SCG1	33610/367830	0.951	84310	0.771	-0.039126	0.0667	0	-1	17	-0.7882	-1	3
HIP_84311	GGPGAGSLQ	LLMKVLEYL	GGPGAGSLQLLMKVLEYL		SCG2	20369/334890	0.996	84311	0.771	0.093102	1.5	0.1667	0	18	0.5944	0	0
HIP_84312	VGQVELGGG	LTGPNNQKR	VGQVELGGGLTGPNNQKR		SCG2	11307/334890	0.999	84312	0.771	0.035985	0.25	0.6667	1	18	-0.7889	1	0
HIP_84313	HLCGSHLV	GENYQKGE	HLCGSHLVGENYQKGE		SCG1	29643/367830	0.967	84313	0.771	0.223592	-0.4333	0.1667	1	16	-0.8313	-1	1
HIP_84314	VEALYLVC	KNDDTPNRQ	VEALYLVCKNDDTPNRQ		SCG2	36520/334890	0.983	84314	0.771	0.233549	-0.0667	0	-1	17	-0.7471	-1	1
HIP_84315	GSLQPLAL	ELFNPYYD	GSLQPLALELFNPYYD		SCG1	31307/367830	0.960	84315	0.771	0.054692	2.0833	0	-2	16	-0.075	-2	1
HIP_84316	CTSICSLYQ	NNQKRERM	CTSICSLYQNNQKRERM		SCG2	43667/334890	0.968	84316	0.771	0.184925	-1.9167	0	2	17	-1.1059	2	1
HIP_84317	GGPGAGSLQ	EGHHRVQEN	GGPGAGSLQEGHHRVQEN		SCG1	34178/367830	0.948	84317	0.771	-0.117185	-1.2667	0.3333	1	18	-1.2778	-1	0
HIP_84318	CTSICSLYQ	YNQEKVLP	CTSICSLYQYNQEKVLP		SCG2	40730/334890	0.975	84318	0.771	0.075234	-1.55	0	0	17	-0.1824	0	2
HIP_84319	VELGGGPGA	DNRNHNEGM	VELGGGPGADNRNHNEGM		SCG1	30191/367830	0.965	84319	0.771	-0.062272	-1.95	0.3333	-1	18	-1.1444	-2	0
HIP_84320	YLVCGERGF	KATPIESHQ	YLVCGERGFKATPIESHQ		IAPP	4663/45018	0.939	84320	0.771	0.017785	-0.8167	0.1667	1	18	-0.4278	0	1
HIP_84321	CTSICSLYQ	PGWQAKRE	CTSICSLYQPGWQAKRE		CMGA	15954/247050	0.985	84321	0.771	0.077391	-0.65	0.3333	1	17	-0.6059	1	2
HIP_84322	SICSLYQLE	DLQEEEQIE	SICSLYQLEDLQEEEQIE		SCG2	24492/334890	0.994	84322	0.771	-0.163840	-1.0667	0	-6	18	-0.6389	-6	1
HIP_84323	VEALYLVC	SRNLQIPPE	VEALYLVCSRNLQIPPE		SCG2	35090/334890	0.985	84323	0.771	0.231843	0.2833	0.1667	-1	17	0.2824	-1	1
HIP_84324	AEDLQVGQV	EEPVSTHQE	AEDLQVGQVEEPVSTHQE		SCG1	18147/367830	0.992	84324	0.771	-0.129772	-1.3833	0.3333	-4	18	-0.9167	-5	0
HIP_84325	CTSICSLYQ	RQYDRVAQ	CTSICSLYQRQYDRVAQ		SCG1	53715/367830	0.820	84325	0.771	0.107102	-1.7167	0	1	17	-0.5059	1	2
HIP_84326	EALYLVCGE	EPGWQAKRE	EALYLVCGEEPGWQAKRE		CMGA	14174/247050	0.989	84326	0.771	-0.068953	-1.15	0.5	-2	18	-0.7	-2	1
HIP_84327	GSLQPLAL	QLPKAAWI	GSLQPLALQLPKAAWI		SCG2	29371/334890	0.991	84327	0.771	0.205103	1.35	0.1667	1	16	0.5562	1	0
HIP_84328	FVNQHLCGS	GEPRAYFMS	FVNQHLCGSGEPRAYFMS		SCG1	23667/367830	0.983	84328	0.771	-0.024221	-0.7	0.6667	1	18	-0.2056	0	0
HIP_84329	EALYLVCGE	PGKWQQQGD	EALYLVCGEPGKWQQQGD		SCG1	25266/367830	0.979	84329	0.771	-0.047879	-1.2167	0.5	-2	18	-0.7667	-2	1
HIP_84330	AGSLQPLAL	RSEALAVD	AGSLQPLALRSEALAVD		CMGA	23287/247050	0.959	84330	0.771	0.235185	0.1	0.1667	-1	17	0.4706	-1	0
HIP_84331	LVEALYL	PASLPSQ	LVEALYLPASLPSQ		CMGA	15364/247050	0.986	84331	0.771	0.185705	0.95	0.3333	-1	14	0.7071	-1	1
HIP_84332	VCGERGFF	APGEEEEEE	VCGERGFFAPGEEEEEE		CMGA	7399/247050	0.997	84332	0.771	-0.034316	0.8333	0.5	-6	17	-1.0412	-6	2
HIP_84333	LVEALYL	EEEEEMA	LVEALYLEEEEEMA		CMGA	18419/247050	0.977	84333	0.771	0.128328	-0.7	0	-6	14	-0.0857	-6	1
HIP_84334	LQVGQVEL	EEARFQDKQ	LQVGQVELEEARFQDKQ		SCG1	40114/367830	0.918	84334	0.771	0.039083	-0.1167	0	-2	17	-0.9529	-2	1
HIP_84335	EALYLVCGE	TSYFPNPYN	EALYLVCGETSYFPNPYN		SCG2	20164/334890	0.996	84335	0.771	-0.173411	-0.7	0.3333	-2	18	-0.2278	-2	2
HIP_84336	QHLCGSHLV	DERILSILR	QHLCGSHLVDERILSILR		CMGA	25680/247050	0.946	84336	0.771	0.184597	-1.1167	0	2	18	0.1667	0	0
HIP_84337	SLQPLALEG	SQGGSLPSE	SLQPLALEGSQGGSLPSE		SCG1	16582/367830	0.993	84337	0.771	-0.071391	-0.8	0.5	-2	18	-0.2556	-2	0
HIP_84338	LYQLENYCN	LKELQDLAL	LYQLENYCNLKELQDLAL		CMGA	23973/247050	0.956	84338	0.771	-0.123160	-0.9833	0	-2	18	-0.2167	-2	1
HIP_84339	VEALYLVC	VIAYLKRLV	VEALYLVCVIAYLKRLV		SCG2	29533/334890	0.990	84339	0.771	0.014030	3.5	0	1	17	1.6529	1	2
HIP_84340	AGSLQPLAL	YEDVVGGE	AGSLQPLALYEDVVGGE		SCG2	27407/334890	0.992	84340	0.771	0.094296	0.1833	0	-3	17	0.2647	-3	1
HIP_84341	VCGERGFF	SKVIAYLKR	VCGERGFFSKVIAYLKR		SCG2	37715/334890	0.981	84341	0.771	0.084135	0.7833	0.3333	3	17	0.2	3	2
HIP_84342	ELGGGPGA	FLVHSSNN	ELGGGPGAFLVHSSNN		IAPP	1616/45018	0.993	84342	0.771	0.115360	1.7667	0.3333	0	16	-0.1313	-1	1
HIP_84343	HLVEALYL	NEQINDEM	HLVEALYLNEQINDEM		SCG2	26819/334890	0.993	84343	0.771	0.175011	-0.7	0	-3	16	-0.325	-4	1
HIP_84344	VELGGGPGA	TLNEKNFFP	VELGGGPGATLNEKNFFP		SCG1	12253/367830	0.997	84344	0.771	-0.059583	-0.1	0.3333	-1	18	-0.2333	-1	0
HIP_84345	LYQLENYCN	EVMKCIVEV	LYQLENYCNEVMKCIVEV		CMGA	20990/247050	0.968	84345	0.771	-0.104509	0.05	0	-2	18	0.2278	-2	1
HIP_84346	VELGGGPGA	KEWEDSKRW	VELGGGPGAKEWEDSKRW		CMGA	20465/247050	0.970	84346	0.771	-0.017160	-1.4167	0.3333	-1	18	-1.2389	-1	1
HIP_84347	CGERGFFY	HSSNNFGAI	CGERGFFYHSSNNFGAI		IAPP	4694/45018	0.938	84347	0.771	0.187540	-0.0833	0.3333	1	17	-0.3	0	1

HIP_84816	SICSLYQL	STHYRASEE	SICSLYQLSTHYRASEE		SCG1	37587/367830	0.932	84816	0.771	0.002618	-0.95	0.1667	0	17	-0.4882	-1	2	1	0
HIP_84817	ICSLYQLEN	PRLPYGAGR	ICSLYQLENPRLPYGAGR		SCG2	40113/334890	0.977	84817	0.771	-0.011808	-0.9167	0.1667	1	18	-0.3722	1	1	0	0
HIP_84818	LQVGQVELG	PEGKGEQEH	LQVGQVELGPEGKGEQEH		CMGA	10730/247050	0.994	84818	0.771	-0.085523	-0.9333	0.5	-2	18	-1.0444	-3	0	0	0
HIP_84819	VELGGGPGA	ASFQRNQLL	VELGGGPGAASFQRNQLL		SCG2	8640/334890	0.999	84819	0.771	0.002494	0.6	0.5	0	18	-0.0278	0	1	0	1
HIP_84820	HLVEALYL	SKPDNPGED	HLVEALYLSKPDNPGED		NPY	4106/49410	0.955	84820	0.771	0.210039	0	0.3333	-2	17	-0.7588	-3	1	1	0
HIP_84821	CTSICSLY	QEESEESNV	CTSICSLYQEESEESNV		SCG1	30802/367830	0.963	84821	0.771	0.048501	-1.4667	0.1667	-4	17	-0.5118	-4	1	1	0
HIP_84822	TSICSLY	FLVHSSNN	TSICSLYFLVHSSNN		IAPP	3293/45018	0.973	84822	0.771	-0.007164	2.0833	0.1667	1	15	0.4133	0	2	1	0
HIP_84823	TSICSLYQL	TSYFPNPY	TSICSLYQLTSYFPNPY		SCG2	32355/334890	0.988	84823	0.771	0.050259	-0.6333	0.1667	0	17	-0.0294	0	3	1	0
HIP_84824	SHLVEALYL	RNLQIPPE	SHLVEALYLRNLQIPPE		SCG2	40586/334890	0.976	84824	0.771	0.216538	0.35	0	0	17	-0.0765	-1	1	1	0
HIP_84825	LYQLENYC	PGEEEEEE	LYQLENYCPGEEEEEE		CMGA	9651/247050	0.995	84825	0.771	0.033072	-1.3	0.3333	-7	16	-1.625	-7	1	0	4
HIP_84826	GSLQPLAL	SSMKLSFRA	GSLQPLALSSMKLSFRA		CMGA	21925/247050	0.965	84826	0.771	0.192021	1.6167	0.3333	2	17	0.3765	2	0	1	0
HIP_84827	GSLQPLAL	GRSRPDRSS	GSLQPLALGRSRPDRSS		SCG1	36252/367830	0.939	84827	0.771	0.245657	0.6167	0.3333	2	17	-0.8529	2	0	1	0
HIP_84828	PLALEGSLQ	QLPKAAWIP	PLALEGSLQQLPKAAWIP		SCG2	24417/334890	0.994	84828	0.771	-0.113921	-0.3	0.3333	0	18	0.2111	0	0	0	0
HIP_84829	LYLVCGERG	QKEPDLRLE	LYLVCGERGQKEPDLRLE		SCG2	41453/334890	0.974	84829	0.771	-0.064865	-3.2167	0.1667	-1	18	-0.6778	-1	0	0	0
HIP_84830	GERGFFY	NFLVHSSNN	GERGFFYNFLVHSSNN		IAPP	4376/45018	0.948	84830	0.771	0.048788	1.2333	0	1	16	-0.5625	0	4	2	0
HIP_84831	EQCCTSICS	RDPADASEA	EQCCTSICSRDPADASEA		SCG1	26240/367830	0.977	84831	0.771	0.101597	-0.5667	0.3333	-3	18	-0.5167	-3	0	0	0
HIP_84832	EQCCTSICS	SLIPLIFLI	EQCCTSICSSLIPLIFLI		SCG2	22363/334890	0.995	84832	0.771	-0.030569	2.2833	0.3333	-1	18	1.5556	-1	0	0	0
HIP_84833	VEALYLVCG	YINLITRQR	VEALYLVCGYINLITRQR		NPY	4040/49410	0.956	84833	0.771	0.006213	1	0.1667	1	18	0.55	1	2	0	0
HIP_84834	LVEALYLVC	SQAELKEA	LVEALYLVCSQAELKEA		CMGA	19448/247050	0.974	84834	0.771	0.156288	1.3333	0.1667	-2	17	0.6765	-2	1	1	0
HIP_84835	YLVCGERGF	ILSSTNVGS	YLVCGERGFILSSTNVGS		IAPP	2783/45018	0.981	84835	0.771	-0.006594	0.9	0.3333	0	18	0.4833	0	1	0	0
HIP_84836	HLCGSHLVE	ETHAAGHSQ	HLCGSHLVEETHAAGHSQ		SCG1	50637/367830	0.866	84836	0.771	0.046842	-0.4833	0	2	18	-0.4722	-2	0	0	0
HIP_84837	LVEALYL	SLSAIEA	LVEALYLSLSAIEA		CMGA	14743/247050	0.987	84837	0.771	0.166135	1.4167	0.3333	-2	14	1.3857	-2	1	0	0
HIP_84838	LVEALYLV	FLISGAEAA	LVEALYLVFLISGAEAA		SCG2	24911/334890	0.994	84838	0.771	-0.031083	2.9667	0	-2	17	1.6824	-2	2	1	0
HIP_84839	CSLYQLENY	EEVKTRHSE	CSLYQLENYEEVKTRHSE		SCG1	47228/367830	0.873	84839	0.771	-0.057556	-1.85	0	-1	18	-1.2889	-2	1	0	0
HIP_84840	QPLALEGSL	LIFLISGAE	QPLALEGSLLIFLISGAE		SCG2	13880/334890	0.998	84840	0.771	0.070985	2.2833	0.3333	-2	18	1.1056	-2	1	0	0
HIP_84841	LGGGPGAGS	EESEEGEED	LGGGPGAGSEESEEGEED		SCG1	18669/367830	0.991	84841	0.771	-0.114057	-1.2	0.5	-7	18	-1.3611	-7	0	0	1
HIP_84842	HLVEALYLV	RRHPQGAWK	HLVEALYLVRRHPQGAWK		SCG1	53915/367830	0.818	84842	0.771	0.162354	-0.9167	0	4	18	-0.3944	2	1	0	0
HIP_84843	GGPGAGSLQ	EDWMRIILE	GGPGAGSLQEDWMRIILE		SCG2	29786/334890	0.990	84843	0.771	-0.102781	-1.4	0.1667	-2	18	-0.1722	-2	1	0	0
HIP_84844	NQHLCGSHL	LQQKENGDE	NQHLCGSHLLQQKENGDE		SCG2	32830/334890	0.987	84844	0.771	-0.098511	-0.5667	0.1667	0	18	-1.4444	-2	0	0	0
HIP_84845	VEALYLVCG	RDPADASEA	VEALYLVCGRDPADASEA		SCG1	26776/367830	0.975	84845	0.771	0.074386	-0.55	0.3333	-3	18	0.1722	-3	1	0	0
HIP_84846	CGERGFFYT	LENQSSQA	CGERGFFYTLENQSSQA		CMGA	25076/247050	0.950	84846	0.771	0.030645	-0.4	0	-1	17	-0.7471	-1	3	1	0
HIP_84847	LGGGPGAGS	KENKPYALN	LGGGPGAGSKENKPYALN		SCG2	40254/334890	0.976	84847	0.771	0.006428	-1.7167	0.3333	1	18	-0.8	1	0	0	1
HIP_84848	EQCCTSICS	KPFSEDVNW	EQCCTSICSKPFSEDVNW		SCG1	31078/367830	0.961	84848	0.771	0.031628	0.5833	0.3333	-2	18	-0.4444	-2	1	0	0
HIP_84849	ICSLYQLEN	QDTKENREE	ICSLYQLENQDTKENREE		SCG1	41070/367830	0.912	84849	0.771	0.002272	-1.8167	0	-3	18	-1.5611	-3	1	0	0
HIP_84850	VEALYLVCG	IAKRAMENM	VEALYLVCGIAKRAMENM		SCG2	25607/334890	0.993	84850	0.771	-0.016384	1.45	0.1667	0	18	0.6444	0	1	0	0
HIP_84851	SICSLYQL	EEARFQDKQ	SICSLYQLEEARFQDKQ		SCG1	39877/367830	0.919	84851	0.771	-0.030592	-1.0333	0	-1	17	-0.7706	-1	2	1	0
HIP_84852	GSLQPLALE	ELTLNEKNF	GSLQPLALEELTLNEKNF		SCG1	34485/367830	0.947	84852	0.771	-0.048664	0.2833	0	-2	18	-0.2667	-2	0	0	0
HIP_84853	HLVEALYLV	TPIESHQV	HLVEALYLVTPIESHQV		IAPP	2636/45018	0.983	84853	0.771	0.132845	1.4833	0.1667	0	17	0.5294	-2	1	1	0
HIP_84854	GSLQPLAL	SNQVKRVP	GSLQPLALSNQVKRVP		SCG2	31902/334890	0.988	84854	0.771	0.237657	0.2667	0.1667	2	16	-0.1562	2	0	0	0
HIP_84855	GERGFFYTP	PKNDDTPN	GERGFFYTPPKNDDTPN		SCG2	34255/334890	0.986	84855	0.771	0.001750	-2.1	0.3333	-1	17	-1.6824	-1	3	1	0
HIP_84856	RGFFYTPKT	NLLGMESAA	RGFFYTPKTNLLGMESAA		SCG2	26114/334890	0.993	84856	0.771	0.106676	-0.35	0.1667	1	18	-0.1444	1	1	0	0
HIP_84857	DLQVGQVEL	NYGEEGAPG	DLQVGQVELNYGEEGAPG		SCG1	27414/367830	0.974	84857	0.771	0.084317	-0.1167	0.1667	-4	18	-0.6222	-4	1	0	0
HIP_84858	EQCCTSICS	LQQKENGDE	EQCCTSICSLQQKENGDE		SCG2	20782/334890	0.996	84858	0.771	-0.094257	0.5	0.1667	-3	18	-1.0444	-3	0	0	0

HIP_8S327	PLALEGSLQ	NYDWWEKK	PLALEGSLQNYDWWEKK		SCG1	40008/367830	0.918	8S327	0.770	-0.014634	-1.4667	0.1667	-1	17	-1.0588	-1	2	1
HIP_8S328	CGERGFF	ATNTHPP	CGERGFFATNTHPP		CMGA	22210/247050	0.964	8S328	0.770	0.037432	0.4667	0.1667	1	14	-0.7286	0	2	0
HIP_8S329	QVGQVELGG	LFQNRMLSK	QVGQVELGGLFQNRMLSK		SCG2	13637/334890	0.998	8S329	0.770	0.056337	1.0167	0.3333	1	18	-0.1889	1	1	0
HIP_8S330	ELGGGPGAG	KGEQEHSQQ	ELGGGPGAGKGEQEHSQQ		CMGA	15683/247050	0.985	8S330	0.770	-0.086446	-1.1333	0.5	-1	18	-1.5167	-2	0	0
HIP_8S331	EAEDLQVGQ	GGEPRAYFM	EAEDLQVGQGGEPRAYFM		SCG1	9866/367830	0.998	8S331	0.770	-0.063194	-0.6667	0.5	-3	18	-0.7389	-3	0	0
HIP_8S332	QVGQVELGG	PPEDLIEML	QVGQVELGGPPEDLIEML		SCG2	9638/334890	0.999	8S332	0.770	-0.147664	-0.6167	0.6667	-4	18	0.0444	-4	0	0
HIP_8S333	ALYLVCGER	EDWMRILE	ALYLVCGEREDWMRILE		SCG2	30374/334890	0.990	8S333	0.770	-0.062815	-2.7167	0.1667	-2	18	0.2889	-2	1	0
HIP_8S334	SICSLYQLE	EALAVDGAG	SICSLYQLEEALAVDGAG		CMGA	17900/247050	0.979	8S334	0.770	-0.112442	-0.1833	0	-3	18	0.5722	-3	1	0
HIP_8S335	CGERGFFY	GWQAKRE	CGERGFFYGWQAKRE		CMGA	23881/247050	0.956	8S335	0.770	0.157841	-0.0833	0.1667	1	15	-1.1267	1	4	1
HIP_8S336	GPGAGSLQP	ERGEDSSEE	GPGAGSLQPERGEDSSEE		SCG1	23175/367830	0.984	8S336	0.770	-0.035207	-1.6167	0.3333	-4	18	-1.5056	-4	0	0
HIP_8S337	EALYLVCGE	PSPEMIRAL	EALYLVCGEPSPEMIRAL		SCG2	17035/334890	0.997	8S337	0.770	-0.087868	-0.9	0.6667	-2	18	0.4111	-2	0	0
HIP_8S338	LYLVCGERG	ESKAEGNNQ	LYLVCGERGESKAEGNNQ		CMGA	28634/247050	0.926	8S338	0.770	-0.101594	-2.7667	0.3333	-1	18	-0.9222	-1	0	0
HIP_8S339	AGSLQPLAL	AEKGREHI	AGSLQPLALAEKGREHI		SCG2	39945/334890	0.977	8S339	0.770	0.205520	0.6333	0	1	17	-0.2353	0	0	1
HIP_8S340	SHLVEALY	EDSKEAEK	SHLVEALYEDSKEAEK		CMGA	19171/247050	0.975	8S340	0.770	0.160838	-0.5833	0.1667	-2	16	-1	-3	1	0
HIP_8S341	PLALEGSLQ	SKSGYPKTP	PLALEGSLQSKSGYPKTP		SCG2	40793/334890	0.975	8S341	0.770	0.053433	-1	0.5	1	18	-0.6444	1	0	0
HIP_8S342	VGQVELGGG	QVLKTSRK	VGQVELGGGQVLKTSRK		SCG1	20528/367830	0.989	8S342	0.770	0.185796	0.55	0.5	2	17	-0.3353	2	0	1
HIP_8S343	GAGSLQPLA	EKNFPMDMS	GAGSLQPLAEKNFPMDMS		SCG2	33545/334890	0.987	8S343	0.770	0.062217	-1.15	0.1667	-1	18	-0.3167	-1	1	0
HIP_8S344	EQCCTSICS	QLIEISRNL	EQCCTSICSQLIEISRNL		SCG2	25583/334890	0.993	8S344	0.770	-0.027721	1.8333	0.1667	-1	18	0.1944	-1	0	0
HIP_8S345	VELGGGPGA	DEEDKRNYP	VELGGGPGADEEDKRNYP		SCG1	27051/367830	0.975	8S345	0.770	-0.062089	-1.7833	0.3333	-3	18	-1.4278	-3	0	0
HIP_8S346	VELGGGPGA	SEVDKRRTR	VELGGGPGASEVDKRRTR		SCG1	26374/367830	0.977	8S346	0.770	-0.022924	-0.05	0.5	1	18	-1.0333	1	0	0
HIP_8S347	AEDLQVGQV	SEEKGHPQE	AEDLQVGQVSEEKGHPQE		SCG1	24788/367830	0.981	8S347	0.770	-0.064608	-1.25	0.3333	-3	18	-1.35	-4	0	0
HIP_8S348	GERGFFYT	PGRAGTEAL	GERGFFYTPGRAGTEAL		SCG2	29939/334890	0.990	8S348	0.770	0.166716	-0.95	0.3333	0	17	-0.5235	0	3	1
HIP_8S349	TSICSLYQL	ENQSSQAE	TSICSLYQLENQSSQAE		CMGA	24441/247050	0.953	8S349	0.770	0.156208	-1.9167	0	-2	17	-0.5765	-2	1	1
HIP_8S350	SLQPLAL	LDRNYLN	SLQPLALLDRNYLN		SCG1	39346/367830	0.922	8S350	0.770	0.185199	0.8667	0	0	14	-0.1	0	0	0
HIP_8S351	CCTSICSLY	DVSKVIAYL	CCTSICSLYDVSKVIAYL		SCG2	26127/334890	0.993	8S351	0.770	-0.037001	0.2667	0.3333	0	18	1.1778	0	1	0
HIP_8S352	VEALYLVCG	QDTKENRE	VEALYLVCGQDTKENRE		SCG1	28614/367830	0.970	8S352	0.770	0.072227	-0.2333	0.1667	-2	17	-0.6765	-2	1	1
HIP_8S353	SICSLYQLE	SLSEEDWMR	SICSLYQLESLSEEDWMR		SCG2	16980/334890	0.997	8S353	0.770	-0.209107	-0.1667	0.3333	-3	18	-0.3944	-3	1	0
HIP_8S354	FVNQHLCGS	RPDRSSQGG	FVNQHLCGSRPDRSSQGG		SCG1	40616/367830	0.915	8S354	0.770	0.001075	-1.3833	0.5	2	18	-1.0056	1	0	0
HIP_8S355	QVELGGGPG	YWDEDLLMK	QVELGGGPGYWDEDLLMK		SCG2	13575/334890	0.998	8S355	0.770	0.090532	-1.35	0.5	-3	18	-0.5167	-3	2	0
HIP_8S356	NQHLCGSHL	YDWWEKKP	NQHLCGSHLYDWWEKKP		SCG1	45166/367830	0.887	8S356	0.770	0.040726	-0.9833	0.1667	2	17	-1.4118	0	3	1
HIP_8S357	LCGSHLVEA	TDDEDDIYK	LCGSHLVEATDDEDDIYK		SCG2	27548/334890	0.992	8S357	0.770	0.103602	-0.8667	0	-4	18	-0.5944	-5	0	0
HIP_8S358	GAGSLQPLA	GPQAEGDSE	GAGSLQPLAGPQAEGDSE		CMGA	8024/247050	0.997	8S358	0.770	0.053731	-0.25	0.5	-3	18	-0.6056	-3	0	0
HIP_8S359	CGSHLVEAL	AGLPLQVRG	CGSHLVEALAGLPLQVRG		CMGA	18604/247050	0.977	8S359	0.770	-0.028806	1.2167	0.1667	1	18	0.6333	0	0	0
HIP_8S360	HLCGSHLVE	NRMLSKSGY	HLCGSHLVENRMLSKSGY		SCG2	48936/334890	0.948	8S360	0.770	0.064971	-0.2667	0	3	18	-0.35	1	0	0
HIP_8S361	DLQVGQVEL	EHLNQGSSQ	DLQVGQVELEHLNQGSSQ		SCG2	38735/334890	0.979	8S361	0.770	-0.011197	0.2667	0	-2	18	-0.7667	-2	0	0
HIP_8S362	LVEALYLVC	LLGAVGLA	LVEALYLVCLLGAVGLA		SCG1	25663/367830	0.978	8S362	0.770	0.024353	2.95	0.1667	-1	17	2.2176	-1	1	1
HIP_8S363	VEQCCTSIC	VQKFPSPEM	VEQCCTSICVQKFPSPEM		SCG2	33036/334890	0.987	8S363	0.770	-0.022369	0.5	0.1667	-1	18	0.0944	-1	1	0
HIP_8S364	GPGAGSLQP	SEGLSQGLV	GPGAGSLQPSEGLSQGLV		CMGA	10370/247050	0.994	8S364	0.770	-0.037028	-1	0.5	-1	18	-0.0389	-1	0	0
HIP_8S365	QPLALEGSL	DWMRILEA	QPLALEGSLDWMRILEA		SCG2	11696/334890	0.998	8S365	0.770	0.004962	0.0167	0.3333	-2	18	0.4167	-2	1	0
HIP_8S366	HLCGSHLVE	LQWKSSHFE	HLCGSHLVELQWKSSHFE		SCG1	41591/367830	0.909	8S366	0.770	-0.004483	0.65	0	2	18	-0.3278	-1	1	0
HIP_8S367	LYQLENYCN	EVISDTLSK	LYQLENYCNEVISDTLSK		CMGA	21224/247050	0.968	8S367	0.770	-0.106029	0.4833	0	-2	18	-0.4	-2	1	0
HIP_8S368	VEALYLVCG	EKVAHQLQA	VEALYLVCGEKVAHQLQA		CMGA	22751/247050	0.962	8S368	0.770	0.012697	0.5167	0.1667	0	18	0.5056	-1	1	0
HIP_8S369	CGERGFFYT	AAMDLELQ	CGERGFFYTAAMDLELQ		SCG1	26773/367830	0.976	8S369	0.770	-0.046461	1.05	0	-2	17	-0.0059	-2	3	1
[illegible]																		

HIP_85838	HLVEALYL	ENEPQSAP	HLVEALYLENEPQSAP		SCG2	29093/334890	0.991	85838	0.770	0.173091	-0.7	0	-2	16	-0.425	-3	1	0	0
HIP_85839	GSHLVEALY	EVKTRHSE	GSHLVEALYEVKTRHSE		SCG1	41687/367830	0.908	85839	0.770	0.152829	0.1833	0	1	17	-0.6765	-1	1	1	0
HIP_85840	CGERGFFYT	LTLALSLL	CGERGFFYTLTLALSLL		NPY	4070/49410	0.956	85840	0.770	-0.071465	1.2833	0	0	17	0.9765	0	3	1	0
HIP_85841	VCGERGFF	NFLEGEEEN	VCGERGFFNFLEGEEEN		SCG1	15757/367830	0.994	85841	0.770	-0.066918	1.3833	0.1667	-4	17	-0.6647	-4	3	1	1
HIP_85842	TSICSLYQL	SGELEQEE	TSICSLYQLSGELEQEE		CMGA	11571/247050	0.993	85842	0.770	0.109976	-0.95	0.3333	-4	17	-0.4353	-4	1	1	0
HIP_85843	SICSLYQL	EAASFQRNQ	SICSLYQLEAASFQRNQ		SCG2	33695/334890	0.986	85843	0.770	-0.092613	-0.15	0	0	17	-0.2765	0	2	1	0
HIP_85844	SLYQLENYC	NPYNQEKV	SLYQLENYCNPYNQEKV		SCG2	30225/334890	0.990	85844	0.770	0.000448	-1.45	0.1667	-1	17	-1.2	-1	2	1	0
HIP_85845	GERGFFY	EDSKEAE	GERGFFYEDSKEAE		CMGA	21571/247050	0.966	85845	0.770	0.166425	-0.5833	0.1667	-3	14	-1.5286	-3	3	0	0
HIP_85846	QVGQVELGG	AYFMSDTRE	QVGQVELGGAYFMSDTRE		SCG1	10956/367830	0.997	85846	0.770	0.089961	1.05	0.3333	-2	18	-0.4056	-2	2	0	0
HIP_85847	PGAGSLQPL	ALNHLKATP	PGAGSLQPLALNHLKATP		IAPP	3560/45018	0.967	85847	0.770	0.051469	0.1333	0.1667	2	18	-0.0333	1	0	0	0
HIP_85848	LVEALYL	GFRGPGP	LVEALYLGFRGPGP		CMGA	24162/247050	0.955	85848	0.770	0.213484	0.7	0.1667	0	14	0.4643	0	2	0	0
HIP_85849	CGSHLVEAL	LGYKEIRKG	CGSHLVEALLGYKEIRKG		CMGA	22129/247050	0.964	85849	0.770	-0.028294	0.7	0.1667	2	18	-0.0778	1	1	0	0
HIP_85850	GIVEQCCTS	PDRSSQGGS	GIVEQCCTSPDRSSQGGS		SCG1	39219/367830	0.923	85850	0.770	0.026156	-1.4333	0.3333	-1	18	-0.6389	-1	0	0	0
HIP_85851	YQLENYCN	EPRAYFMSD	YQLENYCNEPRAYFMSD		SCG1	36560/367830	0.937	85851	0.770	0.141981	-1.9833	0.1667	-2	17	-1.1176	-2	2	1	0
HIP_85852	PGAGSLQPL	SAAVLALLL	PGAGSLQPLSAAVLALLL		CMGA	13715/247050	0.989	85852	0.770	-0.027584	0.25	0.3333	0	18	1.3944	0	0	0	1
HIP_85853	LVEALYLVC	LRGDERIL	LVEALYLVCLRGDERIL		CMGA	20352/247050	0.971	85853	0.770	0.149647	1.5667	0.1667	-1	17	0.8824	-1	1	1	0
HIP_85854	RGFFYTPK	SGYPKTPG	RGFFYTPKSGYPKTPG		SCG2	32518/334890	0.988	85854	0.770	-0.067581	-1.45	0.5	3	16	-1.0938	3	3	0	0
HIP_85855	SLQPLALEG	TSEVDKRRT	SLQPLALEGTSEVDKRRT		SCG1	34746/367830	0.946	85855	0.770	-0.122103	-0.85	0.3333	0	18	-0.8056	0	0	0	0
HIP_85856	LVEALYLV	LQEEEQIEQ	LVEALYLVLQEEEQIEQ		SCG2	21259/334890	0.996	85856	0.770	0.065895	0.5833	0	-5	17	0.0353	-5	1	1	1
HIP_85857	VELGGGPGA	EKSQREDEE	VELGGGPGAEKSQREDEE		SCG1	21575/367830	0.987	85857	0.770	-0.108196	-1.4	0.5	-4	18	-1.5056	-4	0	0	1
HIP_85858	CCTSICSLY	AVEEPSSKD	CCTSICSLYAVEEPSSKD		CMGA	17445/247050	0.981	85858	0.770	-0.109310	0.7	0.1667	-2	18	0.0333	-2	1	0	0
HIP_85859	LCGSHLVEA	QKTSYFPNP	LCGSHLVEAQKTSYFPNP		SCG2	47964/334890	0.952	85859	0.770	0.184659	-0.9333	0	1	18	-0.3278	0	0	0	0
HIP_85860	GIVEQCCTS	NEQINDEMK	GIVEQCCTSNEQINDEMK		SCG2	27276/334890	0.992	85860	0.770	-0.106672	-1.5833	0.1667	-3	18	-0.7611	-3	0	0	0
HIP_85861	VELGGGPGA	YLNQEKAEK	VELGGGPGAYLNQEKAEK		SCG2	10264/334890	0.999	85861	0.770	0.026933	-0.2	0.3333	-1	18	-0.8	-1	1	0	1
HIP_85862	ICSLYQLEN	PPITPECRQ	ICSLYQLENPPITPECRQ		SCG1	21745/367830	0.987	85862	0.770	-0.117707	-0.3167	0.3333	-1	18	-0.4444	-1	1	0	0
HIP_85863	EQCCTSICS	SRDNPFKRT	EQCCTSICSSRDNPFKRT		SCG2	41487/334890	0.974	85863	0.770	0.216202	-0.4333	0.3333	1	18	-0.9722	1	0	0	0
HIP_85864	GQVELGGGP	FGAILSS	GQVELGGGPFGAILSS		IAPP	961/45018	0.996	85864	0.770	0.081976	0.3	0.6667	-1	16	0.5437	-1	1	2	1
HIP_85865	LCGSHLVEA	SEGLSQGLV	LCGSHLVEASEGLSQGLV		CMGA	16453/247050	0.983	85865	0.770	0.181795	-0.3667	0.3333	-1	18	0.5889	-2	0	0	0
HIP_85866	CGERGFFY	DLDDISEAD	CGERGFFYDLDDISEAD		SCG2	26226/334890	0.993	85866	0.770	0.053217	0.1833	0	-5	17	-0.6	-5	3	1	0
HIP_85867	CSLYQLENY	VPRTRLEDP	CSLYQLENYVPRTRLEDP		NPY	4148/49410	0.954	85867	0.770	-0.152701	-1.7	0.1667	-1	18	-0.8722	-1	1	0	0
HIP_85868	AGSLQPLAL	EEAQDPEG	AGSLQPLALEEAQDPEG		CMGA	18697/247050	0.977	85868	0.770	0.080050	0.7	0	-4	17	-0.5294	-4	0	1	0
HIP_85869	LYQLENYCN	RKKSAEFPD	LYQLENYCNRKKSAEFPD		SCG1	53689/367830	0.820	85869	0.770	0.131354	-2.4333	0	0	18	-1.3111	0	1	0	0
HIP_85870	AGSLQPLAL	RGPGPQLR	AGSLQPLALRGPGPQLR		CMGA	23774/247050	0.957	85870	0.770	0.241910	0.4833	0.3333	2	17	-0.2353	2	0	1	0
HIP_85871	SLQPLALE	FLVHSSNNF	SLQPLALEFLVHSSNNF		IAPP	3449/45018	0.969	85871	0.770	-0.009444	2.15	0	0	17	0.3294	-1	1	1	0
HIP_85872	FVNQHLCGS	GGHSRERAD	FVNQHLCGSGGHSRERAD		SCG1	27569/367830	0.973	85872	0.770	-0.044273	-0.45	0.6667	2	18	-0.95	0	0	0	0
HIP_85873	PGAGSLQPL	SSEEKHLEE	PGAGSLQPLSSEEKHLEE		SCG1	21595/367830	0.987	85873	0.770	-0.030653	-1.0667	0.5	-2	18	-0.9889	-3	0	0	0
HIP_85874	ERGFFYTP	PNNQKRERM	ERGFFYTPPNNQKRERM		SCG2	36110/334890	0.983	85874	0.770	0.011529	-2.0333	0.3333	2	17	-1.9412	2	2	1	0
HIP_85875	SICSLYQLE	DELSEVLEN	SICSLYQLEDELSEVLEN		CMGA	21558/247050	0.966	85875	0.770	-0.153847	-1.0667	0	-5	18	-0.1	-5	1	0	0
HIP_85876	VEALYLVCG	AELEKVAHQ	VEALYLVCGAELEKVAHQ		CMGA	13761/247050	0.989	85876	0.770	-0.047081	1.4	0.1667	-1	18	0.5056	-2	1	0	0
HIP_85877	VGQVELGGG	SVFQELGKL	VGQVELGGGSVFQELGKL		SCG2	11226/334890	0.999	85877	0.770	0.132268	0.8333	0.6667	-1	18	0.3389	-1	1	0	1
HIP_85878	IVEQCCTSI	GYGEESEEE	IVEQCCTSIGYGEESEEE		SCG1	9167/367830	0.998	85878	0.770	-0.099490	0.15	0.5	-6	18	-0.5944	-6	1	0	1
HIP_85879	VCGERGFF	VSEEVKTR	VCGERGFFVSEEVKTR		SCG1	27965/367830	0.972	85879	0.770	0.012164	0.85	0.3333	0	16	-0.3125	0	2	0	0
HIP_85880	LVEALYLVC	SPEMIRAL	LVEALYLVCSPEMIRAL		SCG2	25013/334890	0.994	85880	0.770	0.193337	0.7667	0.3333	-1	17	1.2294	-1	1	1	0
HIP_85881	AGSLQPLAL	YINLITRQ	AGSLQPLALYINLITRQ		NPY	5156/49410	0.928	85881	0.770	0.103030	1.5167	0	1	17	0.4706	1	1	1	0
HIP_85882	GIVEQCCTS	FLVHSSNN	GIVEQCCTSFLVHSSNN		IAPP	2479/45018	0.985	85882	0.770	0.091809	1.9667	0.1667	0	17	0.2235	-1	1	1	0
HIP_85883	PGAGSLQPL	LLCAGQVTA	PGAGSLQPLLLCAGQVTA		CMGA	12621/247050	0.991	85883	0.770	0.112357	1.4667	0.1667	0	18	0.8	0	0	0	1
HIP_85884	AEDLQVGQV	ENENTKFEV	AEDLQVGQVENENTKFEV		SCG1	25444/367830	0.979	85884	0.770	-0.074316	-1.7	0.1667	-4	18	-0.8611	-4	0	0	0
HIP_85885	GSLQPLAL	GKWQQQGD	GSLQPLALGKWQQQGD		SCG1	37195/367830	0.934	85885	0.770	0.219690	0.7	0.1667	0	16	-0.7938	0	1	0	1
HIP_85886	PGAGSLQPL	SEEGEEDAT	PGAGSLQPLSEEGEEDAT		SCG1	23311/367830	0.984	85886	0.770	-0.072361	-1.5167	0.3333	-5	18	-0.9167	-5	0	0	0
HIP_85887	CGSHLVEAL	RPSSREDSL	CGSHLVEALRPSSREDSL		CMGA	25152/247050	0.949	85887	0.770	0.157645	-0.8	0.3333	0	18	-0.4444	-1	0	0	0
HIP_85888	HLVEALYL	SIYQLIEI	HLVEALYLSIYQLIEI		SCG2	26930/334890	0.992	85888	0.770	0.080662	1.45	0.1667	-1	16	1.1	-2	2	0	0
HIP_85889	LVEALYLVC	RQAENEPQ	LVEALYLVCRQAENEPQ		SCG2	36974/334890	0.982	85889	0.770	0.149526	0.7167	0	-2	17	-0.1471	-2	1	1	0
HIP_85890	SLYQLENYC	WPERKLKH	SLYQLENYCWPERKLKH		SCG2	38910/334890	0.979	85890	0.770	0.057406	-1.3833	0.1667	2	17	-1.2647	1	2	1	0
HIP_85891	YLVCGERGF	RMDEEQKLY	YLVCGERGFRMDEEQKLY		SCG2	35183/334890	0.985	85891	0.770	0.011428	-1.3667	0.1667	-1	18	-0.8222	-1	1	0	0
HIP_85892	GSLQPLAL	EEDWMRII	GSLQPLALEEDWMRII		SCG2	30155/334890	0.990	85892	0.770	0.163378	-0.1833	0	-2	16	0.1187	-2	1	0	0
HIP_85893	GERGFFY	LGNKRLG	GERGFFYLGNKRLG		NPY	4227/49410	0.952	85893	0.770	0.170554	0.7	0.1667	2	14	-0.6857	2	3	0	0
HIP_85894	SICSLYQL	PSSREDSL	SICSLYQLPSSREDSL		CMGA	15519/247050	0.986	85894	0.770	0.168102	-0.7	0.5	-1	16	-0.2188	-1	1	0	0
HIP_85895	EDLQVGQVE	PERKLKHMQ	EDLQVGQVEPERKLKHMQ		SCG2	43029/334890	0.969	85895	0.770	-0.030515	-2.0667	0.1667	0	18	-1.3389	-1	0	0	0

[illegible]

HIP_86349	VNQHLCGSH	YGFRGPGPQ	VNQHLCGSHYGFRGPGPQ		CMGA	17447/247050	0.981	86349	0.769	-0.042606	-0.55	0.5	3	18	-0.8333	1	2	0	0
HIP_86350	VEALYLVC	LSEVLENQS	VEALYLVCLSEVLENQS		CMGA	17032/247050	0.982	86350	0.769	0.133703	1.6667	0.1667	-3	17	0.6882	-3	1	1	0
HIP_86351	CGSHLVEAL	RDNMNDNFL	CGSHLVEALRDNMNDNFL		SCG1	45057/367830	0.888	86351	0.769	0.108436	-1.5667	0	-1	18	-0.2944	-2	0	0	0
HIP_86352	TSICSLYQL	AGEEAVPEE	TSICSLYQLAGEEAVPEE		CMGA	10019/247050	0.995	86352	0.769	-0.024289	-0.5167	0.1667	-4	18	-0.0389	-4	1	0	0
HIP_86353	VEALYLVC	EDSKRWSK	VEALYLVCEDSKRWSK		CMGA	21651/247050	0.966	86353	0.769	0.179248	0.45	0.1667	0	16	-0.3937	0	1	0	0
HIP_86354	EQCCTSICS	KDVKDKETT	EQCCTSICSKDVKDKETT		SCG1	43992/367830	0.895	86354	0.769	0.037165	0.5	0.1667	-1	18	-0.9278	-1	0	0	0
HIP_86355	GQVELGG	NPGEDAPA	GQVELGGNPGEDAPA		NPY	2086/49410	0.986	86355	0.769	-0.071348	-0.4167	0.6667	-3	15	-0.7133	-3	0	1	0
HIP_86356	LVEALYLVC	TRLFEKPL	LVEALYLVCTRLFEKPL		SCG2	35650/334890	0.984	86356	0.769	0.129402	1.5167	0	0	17	0.9118	0	2	1	0
HIP_86357	SLQPLAL	EAENEKDRA	SLQPLALEAENEKDRA		SCG1	31192/367830	0.961	86357	0.769	0.094588	0.7	0	-2	16	-0.9375	-2	0	2	0
HIP_86358	GGPGAGSLQ	SSHHTAEKR	GGPGAGSLQSSHHTAEKR		SCG1	44710/367830	0.890	86358	0.769	-0.054667	-0.8833	0.5	3	18	-1.15	1	0	0	0
HIP_86359	VNQHLCGSH	EKGHPQEES	VNQHLCGSHEKGHPQEES		SCG1	35216/367830	0.944	86359	0.769	-0.088560	-2.0333	0.5	1	18	-1.5556	-2	0	0	0
HIP_86360	SLQPLALE	KNFFPEYNY	SLQPLALEKNFFPEYNY		SCG1	48200/367830	0.866	86360	0.769	0.076605	-0.4167	0	-1	17	-0.5412	-1	2	1	0
HIP_86361	LYQLENYCN	DLLMRESTE	LYQLENYCNDLLMRESTE		NPY	3769/49410	0.962	86361	0.769	-0.138354	0.3	0	-3	18	-0.75	-3	1	0	0
HIP_86362	SICSLYQL	GEEAVPEE	SICSLYQLGEEAVPEE		CMGA	14533/247050	0.988	86362	0.769	0.112034	-1.4	0.1667	-4	16	-0.1125	-4	1	0	0
HIP_86363	GGGPGAGSL	LQVFLIVLS	GGGPGAGSLLQVFLIVLS		IAPP	1718/45018	0.993	86363	0.769	0.032308	1.1833	0.3333	0	18	1.3333	0	1	0	1
HIP_86364	TSICSLYQL	GEESEEER	TSICSLYQLGEESEEER		SCG1	23496/367830	0.983	86364	0.769	0.126258	-1.4	0.1667	-4	17	-0.9235	-4	1	1	1
HIP_86365	GAGSLQPLA	GALAEAYPS	GAGSLQPLAGALAEAYPS		NPY	2562/49410	0.981	86365	0.769	-0.070983	1.5333	0.3333	-1	18	0.3389	-1	0	0	0
HIP_86366	TSICSLYQ	SEEERGLEP	TSICSLYQSEEERGLEP		SCG1	31783/367830	0.959	86366	0.769	0.134594	-1.4667	0.1667	-3	17	-0.8118	-3	1	1	1
HIP_86367	SLQPLALE	WWEKKPFSE	SLQPLALEWWEKKPFSE		SCG1	45530/367830	0.884	86367	0.769	0.031028	-0.5333	0	-1	17	-0.7294	-1	2	1	0
HIP_86368	GIVEQCCTS	SRGEAGAPG	GIVEQCCTSSRGEAGAPG		SCG1	29296/367830	0.968	86368	0.769	0.062434	-0.7833	0.5	-1	18	-0.1778	-1	0	0	0
HIP_86369	TSICSLYQL	EEEEEGEN	TSICSLYQLEEEEEGEN		SCG1	29410/367830	0.967	86369	0.769	0.052181	-1.9167	0	-6	17	-1.0235	-6	1	1	3
HIP_86370	GGGPGAGSL	TRQRYGKRS	GGGPGAGSLTRQRYGKRS		NPY	7083/49410	0.843	86370	0.769	0.063799	-1.0167	0.3333	4	18	-1.2722	4	0	0	1
HIP_86371	GPGAGSLQP	ALNSEKNFP	GPGAGSLQPALNSEKNFP		SCG2	20598/334890	0.996	86371	0.769	0.014624	0.1333	0.1667	0	18	-0.6389	0	0	0	0
HIP_86372	CTSICSLYQ	QLGIQEEDL	CTSICSLYQQLGIQEEDL		SCG2	9500/334890	0.999	86372	0.769	-0.104825	-0.1833	0.1667	-3	18	0.0222	-3	1	0	0
HIP_86373	TSICSLYQL	DLDHPOLFQ	TSICSLYQLDLDHPOLFQ		SCG2	32043/334890	0.988	86373	0.769	0.018097	-0.7	0	-2	18	-0.05	-3	1	0	0
HIP_86374	PLALEGSLQ	VCLGALAEA	PLALEGSLQVCLGALAEA		NPY	2408/49410	0.983	86374	0.769	0.077760	1.6667	0.1667	-2	18	1.0667	-2	0	0	0
HIP_86375	NQHLCGSHL	PLQQKENGD	NQHLCGSHLPLQQKENGD		SCG2	28501/334890	0.991	86375	0.769	-0.030587	-0.25	0.3333	1	18	-1.3389	-1	0	0	0
HIP_86376	YLVCGERGF	HGYGEESEE	YLVCGERGFHGYGEESEE		SCG1	19318/367830	0.990	86376	0.769	-0.070423	-1.1667	0.3333	-3	18	-0.9389	-4	2	0	0
HIP_86377	CGERGFFYT	WLGAALSL	CGERGFFYTWLGAALSL		SCG2	22738/334890	0.995	86377	0.769	-0.060533	0.55	0.1667	0	17	0.6	0	4	1	0
HIP_86378	NQHLCGSHL	EEEEEEAT	NQHLCGSHLEEEEEEAT		CMGA	19672/247050	0.973	86378	0.769	0.005459	-1.7833	0.1667	-4	17	-1.4353	-6	0	1	4
HIP_86379	GGPGAGSLQ	EEEEEEEA	GGPGAGSLQEEEEEEEA		CMGA	18259/247050	0.978	86379	0.769	-0.084085	-1.8333	0.1667	-7	17	-1.4471	-7	0	1	5
HIP_86380	GGPGAGSLQ	EEEEEEEA	GGPGAGSLQEEEEEEEA		CMGA	18260/247050	0.978	86380	0.769	-0.084085	-1.8333	0.1667	-7	17	-1.4471	-7	0	1	5
HIP_86381	VGQVELGGG	GEEGAPGKW	VGQVELGGGGEEGAPGKW		SCG1	18377/367830	0.992	86381	0.769	-0.069494	-1.4333	0.6667	-2	18	-0.5111	-2	0	0	2
HIP_86382	QVGQVELGG	LVARSETHA	QVGQVELGGLVARSETHA		SCG1	15682/367830	0.994	86382	0.769	0.136390	2.1333	0.3333	0	18	-0.0333	-1	0	0	0
HIP_86383	SLYQLENYC	KDKETTENE	SLYQLENYCKDKETTENE		SCG1	45187/367830	0.887	86383	0.769	0.108613	-2.2667	0	-3	18	-1.6944	-3	1	0	0
HIP_86384	SICSLYQLE	KRLVNAAGS	SICSLYQLEKRLVNAAGS		SCG2	45552/334890	0.961	86384	0.769	-0.065888	-1.3	0	1	18	0.1778	1	1	0	0
HIP_86385	AGSLQPLAL	QKIAEKFS	AGSLQPLALQKIAEKFS		SCG1	45389/367830	0.885	86385	0.769	0.195279	1.0833	0	1	17	0.1294	1	0	1	0
HIP_86386	DLQVGQVEL	ENREEARFQ	DLQVGQVELENREEARFQ		SCG1	45770/367830	0.883	86386	0.769	0.162193	-1.1667	0	-3	18	-1.1278	-3	0	0	0
HIP_86387	DLQVGQVEL	NREEARFQD	DLQVGQVELNREEARFQD		SCG1	45771/367830	0.883	86387	0.769	0.162193	-1.1667	0	-3	18	-1.1278	-3	0	0	0
HIP_86388	EAEDLQVGQ	GPQAEGDSE	EAEDLQVGQGPQAEGDSE		CMGA	6888/247050	0.997	86388	0.769	-0.137695	-0.8667	0.5	-6	18	-1.3056	-6	0	0	0
HIP_86389	LYQLENYC	SSPDYNPYQ	LYQLENYCSSPDYNPYQ		SCG2	19893/334890	0.996	86389	0.769	0.102635	-0.9167	0.5	-2	17	-1.2294	-2	2	1	0
HIP_86390	SICSLYQL	SQEKTHSRE	SICSLYQLSQEKTHSRE		SCG1	38762/367830	0.925	86390	0.769	0.127030	-1.4667	0.1667	1	17	-0.9529	0	1	1	0
HIP_86391	GPGAGSLQP	EYLNQEKAE	GPGAGSLQPEYLNQEKAE		SCG2	21189/334890	0.996	86391	0.769	-0.031901	-0.3833	0.1667	-2	18	-1.1222	-2	1	0	0
[illegible]	[illegible]	[illegible]	[illegible]		[illegible]	[illegible]	[illegible]	[illegible]	[illegible]	[illegible]	[illegible]	[illegible]	[illegible]	[illegible]	[illegible]	[illegible]	[illegible]	[illegible]	[illegible]

HIP_86860	GAGSLQPLA	GYKEIRKGE	GAGSLQPLAGYKEIRKGE		CMGA	24550/247050	0.953	86860	0.769	0.064112	-0.2667	0.3333	1	18	-0.6889	1	1	0	0	0.000000	-3.2	1	0.0667	1.900000	0.2222	1.6778	1	1	1	1	-3.2	-0.4474	-3.2	1	0	0	0	0.4436	0.9772	0.999	0.1667	0.1968	0.9979	-0.1183	0.0044	-0.2133		
HIP_86861	VEALYLVC	VALNHLKA	VEALYLVCVALNHLKA		IAPP	3983/45018	0.958	86861	0.769	0.006009	3.3833	0	1	16	1.2688	0	1	0	0	0.000000	-0.2333	0	0	3.050000	0	3.05	0	0	0	0	0	10.3192	-0.2333	0	0	0	0	0.2385	1.1986	0.5945	0.8333	0.0569	0.3534	0.8068	0	0		
HIP_86862	GSLQPLALE	EQKLYTDDE	GSLQPLALEEQKLYTDDE		SCG2	37179/334890	0.982	86862	0.769	0.057474	-1.4667	0	-4	18	-0.9	-4	0	0	0	0.000000	-4.3333	1	0.1	1.900000	0.3333	1.5667	0	0	0	0	-4.3333	-2.2978	0	0	0	1	0	0.4385	1.3169	0.7545	-0.1667	0.1922	0.5693	-0.6431	0.01	-0.4333		
HIP_86863	EAEDLQVGQ	EEQYTPQSL	EAEDLQVGQEEQYTPQSL		SCG2	24947/334890	0.994	86863	0.769	-0.084180	-1.7	0.1667	-5	18	-1.2611	-5	1	0	0	0.000000	-3.6	-2	0.0167	0.950000	0.2222	0.7278	0	1	0	1	7.2	-1.2372	-3.6	0	1	0	0	0.55	1.1333	0.9067	-0.1667	0.3025	0.822	-0.935	0.0003	-0.06		
HIP_86864	AGSLQPLAL	TRPRHHHG	AGSLQPLALTRPRHHHG		SCG1	42515/367830	0.904	86864	0.769	0.251546	0.4333	0.1667	5	17	-0.7412	2	0	1	1	0.000000	-5.4	1	0.1667	0.450000	0.2222	0.2278	1	0	1	0	-5.4	0.0987	0	1	0	0	0	0.3885	1.0786	0.8412	0.3333	0.1509	0.7075	0.1683	0.0278	-0.9		
HIP_86865	VEALYLVC	QVSEEVKTR	VEALYLVCQVSEEVKTR		SCG1	31168/367830	0.961	86865	0.769	0.176656	1.7333	0.1667	-1	17	0.2059	-1	1	1	0	0.000000	-3.5333	0	0.1167	1.675000	0.2222	1.4528	0	0	0	0	0	2.5181	-3.5333	0	0	0	0	0.3718	1.0586	0.7645	0.5	0.1382	0.5844	0.6444	0.0136	-0.4122		
HIP_86866	CGERGFFYT	PQALPEPM	CGERGFFYTPQALPEPM		CMGA	20998/247050	0.968	86866	0.769	0.031442	-0.4167	0.1667	-1	17	-0.4118	-1	3	1	0	0.000000	-1.3667	0	0.0667	0.950000	0	0.95	1	0	1	0	0	-0.3958	-4.1	0	1	0	0	0.4551	1.0303	0.9378	0.3333	0.2071	0.8795	-0.1896	0.0044	-0.0911		
HIP_86867	ERGFFYTPK	SKEWEDSK	ERGFFYTPKSKEWEDSK		CMGA	30617/247050	0.908	86867	0.769	-0.046042	-2.4	0.3333	0	17	-1.8294	0	3	1	0	0.000000	-0.6667	-1	0	0.700000	0.3333	0.3667	1	0	1	0	0.6667	-0.88	-2	0	0	1	0	0.5603	0.9588	1.039	-0.5	0.3139	1.0795	-1.3446	0	0		
HIP_86868	QVELGGGPG	QSAPKENKP	QVELGGGPGQSAPKENKP		SCG2	13861/334890	0.998	86868	0.769	0.030907	-0.8167	0.6667	0	18	-1.2611	0	0	0	1	0.000000	-0.0333	0	-0.1167	0.000000	0	0	1	2	1	1	0	0	0	0	0	0	1	0.5833	0.8217	1.2267	0.1667	0.3403	1.5047	-0.4764	0.0136	0.0039		
HIP_86869	YLVCGERGF	IKEHLNQGS	YLVCGERGFIKEHLNQGS		SCG2	36489/334890	0.983	86869	0.769	-0.037418	-0.8333	0.1667	1	18	-0.4056	0	1	0	0	1.000000	-0.2667	-1	-0.0333	1.125000	0.4444	0.6806	0	1	0	1	0.2667	-0.5671	-0.2667	1	0	0	0	0.4269	1.0555	0.854	-0.1667	0.1823	0.7293	-0.3558	0.0011	0.0089		

HIP_87371	CCTSICSLY	RHPQGAWKE	CCTSICSLYRHPQGAWKE		SCG1	47808/367830	0.869	87371	0.768	0.027533	-1.2667	0.3333	2	18	-0.4167	1	1	0
HIP_87372	PGAGSLQPL	LLQKEPDLR	PGAGSLQPLLLQKEPDLR		SCG2	20268/334890	0.996	87372	0.768	0.019009	0.4667	0.1667	0	18	-0.4444	0	0	0
HIP_87373	GSLQPLALE	LGAALSLIP	GSLQPLALELGAALSLIP		SCG2	22495/334890	0.995	87373	0.768	-0.061082	1.2167	0.1667	-1	18	1.1167	-1	0	0
HIP_87374	CCTSICSLY	YPSLELDK	CCTSICSLYYPSLELDK		SCG1	25749/367830	0.978	87374	0.768	-0.047821	-0.3333	0.5	-1	17	0.3059	-1	2	1
HIP_87375	GSLQPLAL	GTEALPDG	GSLQPLALGTEALPDG		SCG2	22717/334890	0.995	87375	0.768	0.204323	0.8	0.1667	-2	16	0.15	-2	0	0
HIP_87376	VEALYLVCG	EMAVVPQGL	VEALYLVCGEMAVVPQGL		CMGA	13682/247050	0.989	87376	0.768	-0.061050	1.0833	0.1667	-2	18	1.2222	-2	1	0
HIP_87377	PLALEGSLQ	LGYKEIRKG	PLALEGSLQLGYKEIRKG		CMGA	16099/247050	0.984	87377	0.768	-0.097571	0.2667	0.3333	1	18	-0.3444	1	1	0
HIP_87378	QVGQVELGG	DTEVMKCIV	QVGQVELGGDTEVMKCIV		CMGA	14456/247050	0.988	87378	0.768	-0.136103	-0.7833	0.3333	-2	18	0.3444	-2	0	0
HIP_87379	LVEALYLV	ERELDLPVD	LVEALYLVERELDLPVD		SCG2	30085/334890	0.990	87379	0.768	0.157859	-0.8	0	-4	17	0.5	-4	1	1
HIP_87380	RGFFYTPKT	ELEQEEERL	RGFFYTPKTELEQEEERL		CMGA	24718/247050	0.952	87380	0.768	-0.017901	-1.5667	0.1667	-2	18	-1.4111	-2	1	0
HIP_87381	VNQHLCGSH	GESRSEALA	VNQHLCGSHGESRSEALA		CMGA	19517/247050	0.974	87381	0.768	-0.081125	-1.5167	0.6667	1	18	-0.5667	-1	0	0
HIP_87382	FVNQHLCGS	NRDSSMKLS	FVNQHLCGSNRDSSMKLS		CMGA	30844/247050	0.906	87382	0.768	0.005788	-1.7	0.3333	2	18	-0.5667	1	0	0
HIP_87383	LQPLALEGS	KPYALNSEK	LQPLALEGSKPYALNSEK		SCG2	27943/334890	0.992	87383	0.768	-0.043228	-1.9167	0.5	0	18	-0.5278	0	1	0
HIP_87384	QPLALEGSL	TPQSLATLE	QPLALEGSLTPQSLATLE		SCG2	13220/334890	0.998	87384	0.768	-0.142240	-0.5333	0.5	-2	18	0.1111	-2	0	0
HIP_87385	SICSLYQL	PEYGEEIK	SICSLYQLPEYGEEIK		SCG1	31951/367830	0.958	87385	0.768	0.057198	-1.2333	0.1667	-2	16	-0.3125	-2	2	0
HIP_87386	SLQPLALEG	NSRDNPFKR	SLQPLALEGNSRDNPFKR		SCG2	44913/334890	0.963	87386	0.768	0.005462	-1.4833	0.3333	1	18	-1.0889	1	0	0
HIP_87387	LYQLENYC	GGEPRAYFM	LYQLENYCGGEPRAYFM		SCG1	24296/367830	0.982	87387	0.768	0.149381	-1.1	0.3333	-1	17	-0.4824	-1	1	1
HIP_87388	PLALEGSLQ	FKRTNEIVE	PLALEGSLQFKRTNEIVE		SCG2	43392/334890	0.968	87388	0.768	-0.060633	-1.0167	0.1667	-1	18	-0.2611	-1	1	0
HIP_87389	SLQPLALEG	QRNQLLQKE	SLQPLALEGQRNQLLQKE		SCG2	45542/334890	0.961	87389	0.768	-0.016968	-1.9333	0.1667	0	18	-0.8278	0	0	0
HIP_87390	ALYLVCGER	RGRGGEPRA	ALYLVCGERRGRGGEPRA		SCG1	49173/367830	0.858	87390	0.768	-0.039560	-2.9667	0.3333	2	18	-0.6444	2	0	0
HIP_87391	LVEALYLV	SEEGEEDA	LVEALYLVSEEGEEDA		SCG1	24597/367830	0.981	87391	0.768	0.136023	-0.1833	0.1667	-6	16	-0.0063	-6	1	0
HIP_87392	GSHLVEALY	IFLISGAE	GSHLVEALYIFLISGAE		SCG2	26269/334890	0.993	87392	0.768	-0.054519	2.5667	0	-1	17	1.0059	-2	2	1
HIP_87393	QHLCGSHLV	LGAALSLIP	QHLCGSHLVLGAALSLIP		SCG2	25373/334890	0.993	87393	0.768	0.034810	1.6667	0.1667	2	18	1.1056	0	0	0
HIP_87394	EDLQVGQVE	LIVLSVALN	EDLQVGQVELIVLSVALN		IAPP	2670/45018	0.983	87394	0.768	0.012707	1.6167	0	-3	18	0.8944	-3	0	0
HIP_87395	LVEALYLVC	EESSPDYN	LVEALYLVCEESSPDYN		SCG2	25324/334890	0.993	87395	0.768	0.148316	0.45	0.1667	-4	17	0.0471	-4	1	1
HIP_87396	VELGGGPGA	SQGGSLPSE	VELGGGPGASQGGSLPSE		SCG1	14345/367830	0.995	87396	0.768	-0.069618	-0.8167	0.6667	-2	18	-0.2722	-2	0	0
HIP_87397	HLCGSHLVE	EDLIEMLKT	HLCGSHLVEEDLIEMLKT		SCG2	32026/334890	0.988	87397	0.768	-0.025913	0.2167	0	-1	18	0.1167	-3	0	0
HIP_87398	SICSLYQLE	PMQESKAEG	SICSLYQLEPMQESKAEG		CMGA	19757/247050	0.973	87398	0.768	-0.189061	-1.0667	0.1667	-2	18	-0.4889	-2	1	0
HIP_87399	CGERGFFYT	LIEMLKTG	CGERGFFYTLIEMLKTG		SCG2	32097/334890	0.988	87399	0.768	-0.064194	0.9333	0	0	17	0.1647	0	3	1
HIP_87400	QVELGGGPG	MLGNKRLGL	QVELGGGPGMLGNKRLGL		NPY	1687/49410	0.990	87400	0.768	0.115698	0.4833	0.6667	1	18	-0.0889	1	0	0
HIP_87401	RGFFYTPKT	QTVLTEDEK	RGFFYTPKTQTVLTEDEK		SCG1	31383/367830	0.960	87401	0.768	-0.055225	-1.0333	0.1667	0	18	-1.0444	0	1	0
HIP_87402	SICSLYQLE	ISDTLSKPS	SICSLYQLEISDTLSKPS		CMGA	23023/247050	0.960	87402	0.768	-0.168526	-0.5	0.1667	-1	18	0.05	-1	1	0
HIP_87403	PLALEGSLQ	NGQNGERAT	PLALEGSLQNGQNGERAT		SCG2	22408/334890	0.995	87403	0.768	-0.036219	-1.3167	0.3333	-1	18	-0.8222	-1	0	0
HIP_87404	RGFFYTPKT	LGELFNPYY	RGFFYTPKTLGELFNPYY		SCG1	34767/367830	0.946	87404	0.768	-0.107306	-1.05	0.3333	1	18	-0.4833	1	1	0
HIP_87405	ICSLYQLEN	HPQESKGQP	ICSLYQLENHPQESKGQP		SCG1	47512/367830	0.871	87405	0.768	-0.006802	-1.9167	0.1667	0	18	-1.1111	-1	1	0
HIP_87406	VCGERGFF	KGYPGVQAP	VCGERGFFKGYPGVQAP		SCG1	40578/367830	0.915	87406	0.768	0.086607	-0.0667	0.3333	1	17	-0.1882	1	3	1
HIP_87407	DLQVGQVEL	AEEAQDPEG	DLQVGQVELAEEAQDPEG		CMGA	19739/247050	0.973	87407	0.768	-0.042401	-0.1167	0	-6	18	-0.7944	-6	0	0
HIP_87408	ERGFFYTPK	LEYLNQEKA	ERGFFYTPKLEYLNQEKA		SCG2	28695/334890	0.991	87408	0.768	-0.021323	-1.2	0.1667	0	18	-1.1167	0	3	0
HIP_87409	LVCGERGFF	ELGKLTGP	LVCGERGFFELGKLTGP		SCG2	24751/334890	0.994	87409	0.768	0.054949	0.85	0.3333	0	17	0.2588	0	2	1
HIP_87410	ICSLYQLEN	ETQNAFLNE	ICSLYQLENETQNAFLNE		SCG1	40183/367830	0.917	87410	0.768	-0.007373	-1.8167	0	-3	18	-0.4333	-3	1	0
HIP_87411	GERGFFY	LGALAEAYP	GERGFFYLGALAEAYP		NPY	3085/49410	0.974	87411	0.768	0.027240	1.5833	0.1667	-1	16	0.1062	-1	3	2
HIP_87412	VEALYLVCG	LRKESKDQL	VEALYLVCGLRKESKDQL		SCG2	41401/334890	0.974	87412	0.768	0.031285	0.2833	0.1667	0	18	-0.05	0	1	0
HIP_87413	EQCCTSICS	HQEAENEKD	EQCCTSICSHQEAENEKD		SCG1	30610/367830	0.963	87413	0.768	-0.065177	-0.6667	0.1667	-3	18	-1.3111	-4	0	0

HIP_87882	CGERGFFYT	IQEEDLRK	CGERGFFYTIQEEDLRK		SCG2	36195/334890	0.983	87882	0.768	0.021819	-0.2833	0	-1	17	-0.9882	-1	3	1	0
HIP_87883	TSICSLYQL	LRGDERILS	TSICSLYQLLRGDERILS		CMGA	18321/247050	0.978	87883	0.768	0.051314	-0.35	0.1667	0	18	0.1333	0	1	0	0
HIP_87884	VEALYLVC	SFRARAYGF	VEALYLVCSFRARAYGF		CMGA	28992/247050	0.922	87884	0.768	0.185171	1.3333	0.1667	1	17	0.7765	1	2	1	0
HIP_87885	RGFFYTPKT	DEDDIYKAN	RGFFYTPKTDEDDIYKAN		SCG2	38021/334890	0.981	87885	0.768	-0.019935	-2.7833	0.1667	-1	18	-1.3278	-1	1	0	0
HIP_87886	AEDLQVGQV	LIFLISGAE	AEDLQVGQVLIFLISGAE		SCG2	12654/334890	0.998	87886	0.768	0.054516	1.9	0.1667	-3	18	0.8944	-3	1	0	0
HIP_87887	LVCGERGFF	IAKRAMEN	LVCGERGFFIAKRAMEN		SCG2	31225/334890	0.989	87887	0.768	0.019019	1.2667	0.1667	1	17	0.1118	1	2	1	0
HIP_87888	CGSHLVEAL	PVDNRNHNE	CGSHLVEALPVDNRNHNE		SCG1	29972/367830	0.966	87888	0.768	0.002461	0.2	0.1667	0	18	-0.8	-2	0	0	0
HIP_87889	LVCGERGFF	GLSAEPGW	LVCGERGFFGLSAEPGW		CMGA	9472/247050	0.995	87889	0.768	0.009477	1.3	0.5	-1	17	0.3118	-1	2	1	0
HIP_87890	VCGERGFFY	EWEDSKR	VCGERGFFYEWEDSKR		CMGA	26026/247050	0.944	87890	0.768	0.114717	-0.6	0	-1	16	-1.15	-1	4	2	0
HIP_87891	GGPGAGSLQ	WWEKKPFS	GGPGAGSLQWWEKKPFS		SCG1	40165/367830	0.918	87891	0.768	0.012045	-0.9667	0.1667	1	17	-0.8588	1	2	1	0
HIP_87892	GPGAGSLQP	GKLTGPNNQ	GPGAGSLQPGKLTGPNNQ		SCG2	29268/334890	0.991	87892	0.768	-0.035415	-0.3	0.3333	1	18	-0.9333	1	0	0	0
HIP_87893	HLCGSHLV	EEVKTRHS	HLCGSHLVEEVKTRHS		SCG1	41994/367830	0.906	87893	0.768	0.154799	0.3333	0	3	16	-0.575	0	0	0	0
HIP_87894	YLVCGERGF	EDKRNYPSL	YLVCGERGFEDKRNYPSL		SCG1	43672/367830	0.897	87894	0.768	0.040535	-2.1667	0.1667	0	18	-0.8667	0	1	0	0
HIP_87895	GERGFFY	LANFLVHSS	GERGFFYLANFLVHSS		IAPP	4415/45018	0.946	87895	0.768	0.046489	1.0667	0	1	16	0.225	0	4	2	0
HIP_87896	PLALEGSL	FLVHSSNN	PLALEGSLFLVHSSNN		IAPP	1967/45018	0.991	87896	0.768	0.112729	2.2333	0.3333	0	16	0.3687	-1	1	0	0
HIP_87897	CGERGFFYT	NRRPEDQEL	CGERGFFYTNRRPEDQEL		CMGA	32446/247050	0.890	87897	0.768	0.136816	-1.95	0	-1	18	-1.5	-1	3	0	0
HIP_87898	LVEALYLVC	IRKGESRSE	LVEALYLVCIRKGESRSE		CMGA	29597/247050	0.917	87898	0.768	0.122701	1.1	0	0	18	0.1056	0	1	0	0
HIP_87899	QPLALEGSL	KFPSPEMIR	QPLALEGSLKFPSPEMIR		SCG2	23484/334890	0.994	87899	0.768	-0.057648	-0.0167	0.5	0	18	-0.1833	0	1	0	0
HIP_87900	GGGPGAGSL	QAIKEHLNQ	GGGPGAGSLQAIKEHLNQ		SCG2	18405/334890	0.997	87900	0.768	0.096085	0.9	0.3333	1	18	-0.5444	0	0	0	1
HIP_87901	RGFFYTPKT	SKPDNPGED	RGFFYTPKTSKPDNPGED		NPY	5225/49410	0.926	87901	0.768	-0.052893	-2.0833	0.5	0	18	-1.6556	0	1	0	0
HIP_87902	LVCGERGFF	GKLTGPNN	LVCGERGFFGKLTGPNN		SCG2	31999/334890	0.988	87902	0.768	0.083778	0.7833	0.3333	1	17	-0.1706	1	2	1	0
HIP_87903	HLVEALYL	ELGEYLAR	HLVEALYLELGEYLAR		SCG2	23567/334890	0.994	87903	0.768	0.193394	1.0333	0.1667	-1	16	0.35	-2	1	0	0
HIP_87904	CSLYQLENY	AQQPEGKGE	CSLYQLENYAQQPEGKGE		CMGA	26212/247050	0.943	87904	0.768	-0.139518	-2.25	0	-3	18	-1.2389	-3	1	0	0
HIP_87905	DLQVGQVEL	DSLSEEDWM	DLQVGQVELDSLSEEDWM		SCG2	22897/334890	0.995	87905	0.768	-0.016752	0.6667	0.1667	-6	18	-0.5111	-6	0	0	0
HIP_87906	QHLCGSHLV	EEDNRDSS	QHLCGSHLVEEDNRDSS		CMGA	21775/247050	0.965	87906	0.768	0.130988	-0.95	0	-1	17	-1.2	-3	0	1	0
HIP_87907	GSLQPLAL	FYDSEEP	GSLQPLALFYDSEEP		SCG1	33978/367830	0.949	87907	0.768	0.062625	1.2333	0	-3	15	-0.3	-3	2	1	0
HIP_87908	GSLQPLAL	RDSKENIEK	GSLQPLALRDSKENIEK		SCG2	35381/334890	0.984	87908	0.768	0.247016	0.1	0.1667	0	17	-0.9235	0	0	1	0
HIP_87909	NQHLCGSHL	KLTGPNNQK	NQHLCGSHLKLTGPNNQK		SCG2	50495/334890	0.940	87909	0.768	0.064044	-0.1667	0.1667	4	18	-1.2056	2	0	0	0
HIP_87910	QHLCGSHLV	EKSGEATDG	QHLCGSHLVEKSGEATDG		CMGA	24323/247050	0.954	87910	0.768	0.229226	-0.5667	0.1667	0	18	-0.65	-2	0	0	0
HIP_87911	RGFFYTPKT	AIKEHLNQG	RGFFYTPKTAIKEHLNQG		SCG2	21729/334890	0.988	87911	0.768	0.125817	-0.6333	0.1667	3	18	-0.8556	2	1	0	0
HIP_87912	GGGPGAGSL	YSALRHYIN	GGGPGAGSLYSALRHYIN		NPY	2986/49410	0.976	87912	0.768	0.032256	0.3833	0.5	2	18	-0.1833	1	1	0	1
HIP_87913	SLYQLENY	WPERKLKHM	SLYQLENYWPERKLKHM		SCG2	43137/334890	0.969	87913	0.768	-0.071357	-2.3833	0.1667	2	17	-1.3	1	2	1	0
HIP_87914	HLVEALYLV	MDKARRHP	HLVEALYLVMDKARRHP		SCG1	46107/367830	0.880	87914	0.768	0.179415	0.2	0	3	17	-0.2294	1	1	1	0
HIP_87915	GERGFFYTP	LGEKRDHH	GERGFFYTPLGEKRDHH		SCG1	31023/367830	0.962	87915	0.768	-0.130636	-0.6167	0.3333	2	17	-1.4824	0	3	1	0
HIP_87916	HLVEALYL	DDYETQQWP	HLVEALYLDDYETQQWP		SCG2	30871/334890	0.989	87916	0.768	0.091250	-0.3333	0	-3	17	-0.7412	-4	2	1	0
HIP_87917	VELGGGPGA	AASFQRNQL	VELGGGPGAAASFQRNQL		SCG2	8843/334890	0.999	87917	0.768	-0.017806	0.4333	0.5	0	18	-0.1389	0	1	0	2
HIP_87918	TSICSLYQL	SEVLENQSS	TSICSLYQLSEVLENQSS		CMGA	17219/247050	0.981	87918	0.768	-0.050514	-0.1833	0.1667	-2	18	-0.05	-2	1	0	0
HIP_87919	EAEDLQVGQ	NSNQVKRVP	EAEDLQVGQNSNQVKRVP		SCG2	24628/334890	0.994	87919	0.768	-0.090224	-1.25	0.3333	-1	18	-1.1667	-1	0	0	0
HIP_87920	VEALYLVC	ANNIAYEDV	VEALYLVCANNIAYEDV		SCG2	27963/334890	0.992	87920	0.768	0.146187	0.8833	0	-3	17	0.7353	-3	1	1	0
HIP_87921	ERGFFYTP	DSQVSEEVK	ERGFFYTPDSQVSEEVK		SCG1	32186/367830	0.957	87921	0.768	-0.047051	-1.9	0.3333	-2	17	-1.0294	-2	2	1	0
HIP_87922	ELGGGPGAG	PSEEKGHPQ	ELGGGPGAGPSEEKGHPQ		SCG1	17317/367830	0.993	87922	0.768	-0.109900	-0.8167	0.6667	-1	18	-1.3056	-2	0	0	1
HIP_87923	AGSLQPLAL	LRKESKDQ	AGSLQPLALLRKESKDQ		SCG2	45563/334890	0.961	87923	0.768	0.160325	0.8	0	1	17	-0.6529	1	0	1	0
HIP_87924	EQCCTSICS	QEEEQIEQA	EQCCTSICSQEEEQIEQA		SCG2	24526/334890	0.994	87924	0.768	0.016594	-0.7167	0.1667	-5	18	-0.8611	-5	0	0	1
HIP_87925	LGGGPGAGS	FPVGPPKND	LGGGPGAGSFPVGPPKND		SCG2	9347/334890	0.999	87925	0.768	-0.010862	1	0.5	0	18	-0.4389	0	1	0	1
HIP_87926	VEALYLVCG	RNYPSLEL	VEALYLVCGRNYPSLEL		SCG1	42269/367830	0.905	87926	0.768	0.054264	-0.5	0.1667	-1	17	0.6412	-1	2	1	0
HIP_87927	VEALYLVCG	GAGKPGAEE	VEALYLVCGGAGKPGAEE		CMGA	6627/247050	0.998	87927	0.768	0.021708	1.2167	0.5	-2	18	0.2778	-2	1	0	0
HIP_87928	HLCGSHLVE	HNEGMVTRC	HLCGSHLVEHNEGMVTRC		SCG1	56391/367830	0.795	87928	0.768	0.098465	-0.95	0	2	18	-0.2222	-1	0	0	0
HIP_87929	QVGQVELGG	GNNQAPGEE	QVGQVELGGGNNQAPGEE		CMGA	11088/247050	0.993	87929	0.768	-0.113900	-0.7333	0.5	-3	18	-0.9778	-3	0	0	1
[illegible]																			

HIP_88393	CSLYQLENY	LEKVAHQLQ	CSLYQLENYLEKVAHQLQ		CMGA	29808/247050	0.916	88393	0.768	-0.131745	-1.9833	0	0	18	-0.4333	-1	1	0	0
HIP_88394	SHLVEALY	QIPPEDLIE	SHLVEALYQIPPEDLIE		SCG2	16582/334890	0.997	88394	0.768	0.097777	0.6167	0.1667	-3	17	0.0235	-4	1	1	0
HIP_88395	GSHLVEALY	KNEQINDE	GSHLVEALYKNEQINDE		SCG2	33742/334890	0.986	88395	0.768	0.181468	-1.1	0	-2	17	-0.9412	-3	1	1	0
HIP_88396	LYQLENYCN	GSLPSEEKG	LYQLENYCNGSLPSEEKG		SCG1	14942/367830	0.995	88396	0.768	-0.053558	0.05	0.3333	-2	18	-0.9778	-2	1	0	0
HIP_88397	LVCGERGFF	IVLSVAL	LVCGERGFFIVLSVAL		IAPP	2873/45018	0.980	88397	0.768	0.019033	2.95	0.1667	0	16	1.8	0	2	2	0
HIP_88398	GERGFFYTP	QEAENEKD	GERGFFYTPQEAENEKD		SCG1	30858/367830	0.962	88398	0.768	-0.114074	-1.4667	0.1667	-3	17	-1.7588	-3	3	1	0
HIP_88399	LVCGERGF	AWIPHVENR	LVCGERGFAWIPHVENR		SCG2	22118/334890	0.995	88399	0.768	0.099219	0.55	0.1667	1	17	-0.1294	0	2	1	0
HIP_88400	IVEQCCTSI	QLPKAAWIP	IVEQCCTSIQLPKAAWIP		SCG2	24441/334890	0.994	88400	0.768	-0.062770	0.2833	0.3333	0	18	0.5611	0	0	0	0
HIP_88401	PGAGSLQPL	DFYDSEEPV	PGAGSLQPLDFYDSEEPV		SCG1	18705/367830	0.991	88401	0.768	-0.056624	-0.55	0.1667	-4	18	-0.5333	-4	2	0	0
HIP_88402	VGQVELGGG	QMDKARRHP	VGQVELGGGQMDKARRHP		SCG1	22763/367830	0.985	88402	0.768	0.100123	-1.05	0.5	2	18	-0.9667	1	0	0	1
HIP_88403	LYQLENYCN	LGELFNPYY	LYQLENYCNLGELFNPYY		SCG1	18722/367830	0.991	88403	0.768	-0.127847	-0.4	0.1667	-2	18	-0.4278	-2	1	0	0
HIP_88404	EDLQVGQVE	KTHWLGAAL	EDLQVGQVEKTHWLGAAL		SCG2	45001/334890	0.963	88404	0.768	-0.083210	-1.7667	0	-1	18	-0.2	-2	1	0	0
HIP_88405	PLALEGSLQ	SDDYETQQW	PLALEGSLQSDDYETQQW		SCG2	25973/334890	0.993	88405	0.768	-0.052897	-1.3833	0.3333	-4	18	-0.9889	-4	1	0	0
HIP_88406	VEALYLVC	SHHTAEKRK	VEALYLVCSHHTAEKRK		SCG1	49598/367830	0.855	88406	0.768	0.166929	0.55	0.1667	3	17	-0.3765	1	1	1	0
HIP_88407	EAEDLQVGQ	SRPDRSSQG	EAEDLQVGQSRPDRSSQG		SCG1	26330/367830	0.977	88407	0.768	-0.085584	-1.1	0.5	-2	18	-1.5833	-2	0	0	0
HIP_88408	LYQLENYC	QDPEGKGEQ	LYQLENYCQDPEGKGEQ		CMGA	19166/247050	0.975	88408	0.768	0.096508	-1.8167	0.1667	-3	17	-1.5765	-3	1	1	0
HIP_88409	GERGFFYTP	PITPECRQ	GERGFFYTPPITPECRQ		SCG1	27787/367830	0.973	88409	0.768	-0.120539	-0.2333	0.3333	0	17	-0.8941	0	3	1	0
HIP_88410	LCGSHLVE	AILSSTNVG	LCGSHLVEAILSSTNVG		IAPP	3823/45018	0.962	88410	0.767	0.027368	2.4333	0	0	17	0.8529	-1	0	1	0
HIP_88411	DLQVGQVEL	EQKLYTDDE	DLQVGQVELEQKLYTDDE		SCG2	36137/334890	0.983	88411	0.767	0.091788	-1.0667	0	-5	18	-1	-5	0	0	0
HIP_88412	RGFFYTPKT	QGAWKELD	RGFFYTPKTQGAWKELD		SCG1	33078/367830	0.953	88412	0.767	-0.060956	-1.3833	0.3333	1	17	-1.0353	1	2	1	0
HIP_88413	RGFFYTPKT	VVGGEDWNP	RGFFYTPKTVVGGEDWNP		SCG2	22330/334890	0.995	88413	0.767	-0.011752	0.3	0.3333	0	18	-0.7167	0	1	0	0
HIP_88414	QVELGGGPG	SVFQELGKL	QVELGGGPGSVFQELGKL		SCG2	10616/334890	0.999	88414	0.767	0.110348	0.6333	0.6667	-1	18	0.0167	-1	1	0	1
HIP_88415	LGGGPGAGS	EKGHPQEES	LGGGPGAGSEKGHPQEES		SCG1	27465/367830	0.974	88415	0.767	-0.046972	-1.2	0.5	-1	18	-1.2611	-2	0	0	1
HIP_88416	QHLCGSHL	NYDWWEKKP	QHLCGSHLNYDWWEKKP		SCG1	45642/367830	0.884	88416	0.767	0.055896	-1.4167	0.1667	2	17	-1.4118	0	3	1	0
HIP_88417	CGERGFFYT	PMQESKAE	CGERGFFYTPMQESKAE		CMGA	23134/247050	0.960	88417	0.767	0.066950	-0.4	0.1667	-1	17	-0.9294	-1	3	1	0
HIP_88418	VGQVELGGG	WEDSKRWSK	VGQVELGGGWEDSKRWSK		CMGA	14954/247050	0.987	88418	0.767	0.067564	-1.5167	0.5	0	18	-1.0611	0	1	0	1
HIP_88419	ERGFFYTP	SEESWDEED	ERGFFYTPSEESWDEED		SCG1	28251/367830	0.971	88419	0.767	-0.072782	-1.9	0.3333	-6	17	-1.7588	-6	3	1	0
HIP_88420	GERGFFYT	AWKELDRNY	GERGFFYTAWKELDRNY		SCG1	44105/367830	0.894	88420	0.767	0.007006	-0.3667	0	0	17	-1.2176	0	4	1	0
HIP_88421	IVEQCCTSI	SSSRGEAGA	IVEQCCTSISSSRGEAGA		SCG1	18821/367830	0.991	88421	0.767	0.050542	0.1	0.6667	-1	18	0.1167	-1	0	0	1
HIP_88422	HLVEALYL	QLPKAAWIP	HLVEALYLQLPKAAWIP		SCG2	26672/334890	0.993	88422	0.767	0.129839	0.8333	0.1667	1	17	0.5765	0	1	1	0
HIP_88423	HLVEALYL	LSDDVSKV	HLVEALYLLSDDVSKV		SCG2	25731/334890	0.993	88423	0.767	0.172487	0.9667	0.1667	-1	16	0.5687	-2	1	0	0
HIP_88424	LGGGPGAGS	SGQLGIQEE	LGGGPGAGSSGQLGIQEE		SCG2	12298/334890	0.998	88424	0.767	-0.105253	-0.6833	0.6667	-2	18	-0.3389	-2	0	0	1
HIP_88425	CGSHLVEAL	KGDTEVMKC	CGSHLVEALKGDTEVMKC		CMGA	25444/247050	0.948	88425	0.767	0.185257	-0.95	0.1667	0	18	0.05	-1	0	0	0
HIP_88426	VGQVELGGG	ESSPDYNPY	VGQVELGGGESSPDYNPY		SCG2	14247/334890	0.998	88426	0.767	-0.087601	-1.05	0.8333	-3	18	-0.7944	-3	0	0	1
HIP_88427	PLALEGSLQ	QQKKHSGFE	PLALEGSLQQQKKHSGFE		CMGA	38068/247050	0.822	88427	0.767	0.017582	-1.9	0.1667	1	18	-0.9167	0	0	0	1
HIP_88428	SICSLYQL	GEESEEERG	SICSLYQLGEESEEERG		SCG1	23292/367830	0.984	88428	0.767	0.065039	-1.4	0.1667	-6	17	-0.9059	-6	1	1	1
HIP_88429	LVCGERGFF	DIYKANNI	LVCGERGFFDIYKANNI		SCG2	31818/334890	0.988	88429	0.767	0.038257	0.8167	0.1667	0	17	0.1412	0	3	1	0
HIP_88430	AEDLQVGQV	SSHFERRDN	AEDLQVGQVSSHFERRDN		SCG1	24979/367830	0.980	88430	0.767	-0.107273	-0.75	0.5	-1	18	-1.2167	-2	1	0	0
HIP_88431	EQCCTSIC	EESWDEEDK	EQCCTSICEESWDEEDK		SCG1	26848/367830	0.975	88431	0.767	0.054435	-0.2667	0.3333	-6	17	-1.3588	-6	1	1	0
HIP_88432	AGSLQPLAL	RVPGQGSS	AGSLQPLALRVPGQGSS		SCG2	36958/334890	0.982	88432	0.767	0.213392	1.25	0.1667	1	17	0.0529	1	0	1	0
HIP_88433	TSICSLYQ	EGDSEGLSQ	TSICSLYQEGDSEGLSQ		CMGA	17924/247050	0.979	88433	0.767	0.147991	-1.4	0.1667	-3	17	-0.5235	-3	1	1	0
HIP_88434	LQPLALEGS	PERDSLSEE	LQPLALEGSPERDSLSEE		SCG2	27586/334890	0.992	88434	0.767	-0.125905	-2.3833	0.5	-4	18	-0.8056	-4	0	0	0
HIP_88435	QVELGGGPG	RQYWDEDLL	QVELGGGPGRQYWDEDLL		SCG2	21080/334890	0.996	88435	0.767	0.025674	-1.95	0.5	-3	18	-0.85	-3	2	0	1
HIP_88436	GSHLVEALY	KREPLNYLP	GSHLVEALYKREPLNYLP		IAPP	5741/45018	0.899	88436	0.767	0.204172	-1.2667	0	1	18	-0.4389	0	1	0	0
HIP_88437	SHLVEALY	KHMQFPPMY	SHLVEALYKHMQFPPMY		SCG2	47056/334890	0.956	88437	0.767	0.119311	-0.15	0	2	17	-0.2176	0	2	1	0
HIP_88438	CTSICSLY	YDSEEPVST	CTSICSLYYDSEEPVST		SCG1	32437/367830	0.956	88438	0.767	0.021674	-0.65	0.3333	-3	17	-0.1059	-3	2	1	0
HIP_88439	GSLQPLALE	EGMVTRCII	GSLQPLALEEGMVTRCII		SCG1	26315/367830	0.977	88439	0.767	0.019055	0.0167	0.1667	-1	18	0.6611	-1	0	0	0
HIP_88440	VNQHLCGSH	NGERATRLF	VNQHLCGSHNGERATRLF		SCG2	34438/334890	0.986	88440	0.767	-0.045843	-1.9667	0.5	2	18	-0.7111	1	0	0	0
HIP_88441	EQCCTSICS	SMPVDNRNH	EQCCTSICSSMPVDNRNH		SCG1	20126/367830	0.989	88441	0.767	-0.008490	0.95	0.5	0	18	-0.6556	-1	0	0	0
HIP_88442	SICSLYQL	SQGLVDREK	SICSLYQLSQGLVDREK		CMGA	17375/247050	0.981	88442	0.767	0.089947	-0.95	0.3333	0	17	-0.2294	0	1	1	0
HIP_88443	EQCCTSICS	NLLKELQDL	EQCCTSICSNLLKELQDL		CMGA	16030/247050	0.984	88443	0.767	-0.057151	1.7167	0.1667	-2	18	0	-2	0	0	0
HIP_88444	RGFFYTPKT	LGLSGLTLA	RGFFYTPKTLGLSGLTLA		NPY	3883/49410	0.960	88444	0.767	0.017789	0.1667	0.3333	2	18	0.4	2	1	0	0
HIP_88445	EQCCTSICS	ENSRDNPFK	EQCCTSICSENSRDNPFK		SCG2	27211/334890	0.992	88445	0.767	0.129773	-0.2667	0.3333	-1	18	-1.0722	-1	0	0	0
HIP_88446	QCCTSICSL	EQIEQAIKE	QCCTSICSLEQIEQAIKE		SCG2	28269/334890	0.992	88446	0.767	0.005142	0.5	0.1667	-2	18	-0.0333	-2	0	0	0
HIP_88447	LVEALYLVC	ERMDEEQK	LVEALYLVCERMDEEQK		SCG2	33802/334890	0.986	88447	0.767	0.142412	0.7333	0	-3	17	-0.2765	-3	1	1	0
HIP_88448	IVEQCCTSI	WWEKKPFS	IVEQCCTSIWWEKKPFS		SCG1	36835/367830	0.936	88448	0.767	0.041316	-0.3833	0.1667	0	17	-0.1765	0	2	1	0
HIP_88449	FVNQHLCGS	ESWDEEDKR	FVNQHLCGSESWDEEDKR		SCG1	23109/367830	0.984	88449	0.767	-0.085388	-0.65	0.5	-2	18	-1.4278	-3	1	0	0

HIP_88904	EQCCTSICS	SANRRPEDQ	EQCCTSICSSANRRPEDQ		CMGA	18621/247050	0.977	88904	0.767	0.024769	0.6167	0.3333	-1	18	-1.1611	-1	0	0	0
HIP_88905	YQLENYCN	ENEKDRADQ	YQLENYCNENEKDRADQ		SCG1	36374/367830	0.938	88905	0.767	0.090971	-2.1333	0	-3	17	-2.2294	-3	1	1	0
HIP_88906	CTSICSLY	EEEEEEAEA	CTSICSLYEEEEEEAEA		CMGA	18877/247050	0.976	88906	0.767	0.010256	-1.4667	0.1667	-7	17	-0.6588	-7	1	1	4
HIP_88907	GERGFFYT	YRASEEEPE	GERGFFYTYRASEEEPE		SCG1	40237/367830	0.917	88907	0.767	0.003929	-0.5333	0	-3	17	-1.5059	-3	4	1	1
HIP_88908	EALYLVCGE	DRNYLNYGE	EALYLVCGEDRNYLNYGE		SCG1	42264/367830	0.905	88908	0.767	-0.046500	-2.15	0.1667	-3	18	-0.5722	-3	1	0	0
HIP_88909	VEALYLVC	SQREDEEEE	VEALYLVCSQREDEEEE		SCG1	34283/367830	0.948	88909	0.767	0.186580	0.2833	0.1667	-6	17	-0.8412	-6	1	1	2
HIP_88910	CGERGFFYT	LIVLSVA	CGERGFFYTLIVLSVA		IAPP	3714/45018	0.964	88910	0.767	-0.036091	2.2167	0	0	16	1.175	0	3	2	0
HIP_88911	VCGERGFF	KVLPRLPYG	VCGERGFFKVLPRLPYG		SCG2	32079/334890	0.988	88911	0.767	0.024969	1.55	0.1667	2	17	0.1176	2	2	1	0
HIP_88912	VCGERGFF	LVCLGALAE	VCGERGFFLVCLGALAE		NPY	2720/49410	0.979	88912	0.767	0.003866	2.6167	0.1667	-1	17	1.2529	-1	2	1	0
HIP_88913	CCTSICSLY	LNDKDQELG	CCTSICSLYLNDKDQELG		SCG2	24963/334890	0.994	88913	0.767	-0.119487	-0.25	0.1667	-2	18	-0.1111	-2	1	0	0
HIP_88914	CGERGFFYT	YYSALRHY	CGERGFFYTYYSALRHY		NPY	5933/49410	0.902	88914	0.767	0.049517	-0.4333	0.1667	2	17	-0.5588	1	5	1	0
HIP_88915	CGERGFFY	EKRNLARVP	CGERGFFYEKRNLARVP		SCG1	49336/367830	0.857	88915	0.767	0.215297	-1.2667	0	2	17	-0.8059	2	3	1	0
HIP_88916	LGGGPGAGS	THWLGAALS	LGGGPGAGSTHWLGAALS		SCG2	21867/334890	0.995	88916	0.767	0.019834	-0.7	0.3333	1	18	0.3556	0	1	0	1
HIP_88917	GERGFFYTP	HPQESKGQ	GERGFFYTPHPQESKGQ		SCG1	41318/367830	0.911	88917	0.767	-0.023301	-1.9833	0.3333	1	17	-1.6	0	3	1	0
HIP_88918	GSLQPLAL	LGGGPGAG	GSLQPLALLGGGPGAG		INSC	306/5184	0.996	88918	0.767	0.182750	2.0667	0.3333	0	16	0.5562	0	0	0	1
HIP_88919	SLYQLENYC	SNVSMASLG	SLYQLENYCSNVSMASLG		SCG1	23852/367830	0.983	88919	0.767	-0.069614	-0.4	0.1667	-1	18	0.0889	-1	1	0	0
HIP_88920	HLVEALYL	WMRIILEAL	HLVEALYLWMRIILEAL		SCG2	36298/334890	0.983	88920	0.767	0.050937	0.4667	0	0	17	1.2235	-1	2	1	0
HIP_88921	TSICSLYQL	SDSQVSEEV	TSICSLYQLSDSQVSEEV		SCG1	24754/367830	0.981	88921	0.767	0.155210	-1.0167	0.3333	-3	18	-0.0278	-3	1	0	0
HIP_88922	ICSLYQLEN	LSDDVSKVI	ICSLYQLENLSDDVSKVI		SCG2	28181/334890	0.992	88922	0.767	-0.159080	-0.6167	0.1667	-2	18	0.3444	-2	1	0	0
HIP_88923	AEDLQVGQV	QENHPQESK	AEDLQVGQVQENHPQESK		SCG1	28726/367830	0.970	88923	0.767	-0.071956	-1.7	0.1667	-2	18	-1.5222	-3	0	0	0
HIP_88924	LVCGERGFF	KRNLARVP	LVCGERGFFKRNLARVP		SCG1	50838/367830	0.845	88924	0.767	0.111689	-1.1167	0.1667	3	17	-0.0529	3	2	1	0
HIP_88925	GSLQPLALE	KQASAIKKE	GSLQPLALEKQASAIKKE		SCG1	57571/367830	0.783	88925	0.767	0.083191	-0.5833	0	1	18	-0.4444	1	0	0	0
HIP_88926	CGSHLVEAL	GDSEGLSQG	CGSHLVEALGDSEGLSQG		CMGA	15464/247050	0.986	88926	0.767	0.156786	-0.4333	0.3333	-2	18	-0.0722	-3	0	0	0
HIP_88927	PGAGSLQPL	SEESWDEED	PGAGSLQPLSEESWDEED		SCG1	23576/367830	0.983	88927	0.767	-0.081497	-1.5167	0.3333	-6	18	-1.2444	-6	0	0	0
HIP_88928	VCGERGFF	GLSQGLVDR	VCGERGFFGLSQGLVDR		CMGA	12168/247050	0.992	88928	0.767	0.013572	1.3	0.5	0	17	0.1294	0	2	1	0
HIP_88929	GGPGAGSLQ	TQEEVRDSK	GGPGAGSLQTQEEVRDSK		SCG2	36709/334890	0.983	88929	0.767	-0.099178	-1.3667	0.1667	-1	18	-1.2	-1	0	0	0
HIP_88930	VELGGGPGA	YDSEEPVST	VELGGGPGAYDSEEPVST		SCG1	15598/367830	0.994	88930	0.767	-0.104144	-0.9667	0.5	-4	18	-0.4667	-4	1	0	1
HIP_88931	ERGFFYTP	EGQEEEEDN	ERGFFYTPEGQEEEEDN		CMGA	14299/247050	0.988	88931	0.767	-0.079147	-1.8333	0.3333	-6	17	-2.0471	-6	2	1	2
HIP_88932	VEALYLVCG	GEEGAPGK	VEALYLVCGGEEGAPGK		SCG1	25293/367830	0.979	88932	0.767	0.083647	-0.1833	0.3333	-2	17	0.1882	-2	1	1	0
HIP_88933	HLVEALYL	PGKGRHHRG	HLVEALYLPGKGRHHRG		SCG1	37932/367830	0.930	88933	0.767	0.238452	0.0667	0.3333	5	17	-0.7471	2	1	1	0
HIP_88934	ERGFFYTPK	SSSRGEAG	ERGFFYTPKSSSRGEAG		SCG1	35734/367830	0.941	88934	0.767	-0.086389	-1.4333	0.6667	1	17	-1.1588	1	2	1	1
HIP_88935	GSHLVEALY	LEYIENLR	GSHLVEALYLEYIENLR		SCG2	30200/334890	0.990	88935	0.767	-0.025624	0.55	0	-1	17	0.0118	-2	2	1	0
HIP_88936	SLQPLAL	FPEYNYDW	SLQPLALFPEYNYDW		SCG1	31254/367830	0.961	88936	0.767	0.143822	1.1833	0.1667	-2	15	-0.3667	-2	2	1	0
HIP_88937	VEQCCTSIC	GYEKRNLA	VEQCCTSICGYEKRNLA		SCG1	30530/367830	0.964	88937	0.767	0.067082	0.1667	0.3333	0	17	-0.2235	0	1	1	0
HIP_88938	LYQLENYC	EGQEEEED	LYQLENYCEGQEEEED		CMGA	12786/247050	0.991	88938	0.767	0.031736	-1.6167	0.1667	-7	16	-1.7437	-7	1	0	2
HIP_88939	GAGSLQPLA	GFRGPGPQL	GAGSLQPLAGFRGPGPQL		CMGA	20360/247050	0.971	88939	0.767	0.045749	0.3167	0.3333	1	18	-0.0722	1	1	0	0
HIP_88940	LGGGPGAGS	MLSKSGYPK	LGGGPGAGSMLSKSGYPK		SCG2	21837/334890	0.995	88940	0.767	0.076782	0.9167	0.5	2	18	-0.3222	2	0	0	1
HIP_88941	HLVEALYL	QLSDDVSK	HLVEALYLQLSDDVSK		SCG2	28646/334890	0.991	88941	0.767	0.199186	0.9667	0.1667	-1	16	0.0875	-2	1	0	0
HIP_88942	SICSLYQL	WPERKLKH	SICSLYQLWPERKLKH		SCG2	41668/334890	0.973	88942	0.767	0.126815	-1.1667	0.1667	3	16	-0.5938	2	2	0	0
HIP_88943	VCGERGFF	IPHVENRQM	VCGERGFFIPHVENRQM		SCG2	27946/334890	0.992	88943	0.767	0.010579	0.8167	0.3333	1	17	-0.3353	0	2	1	0
HIP_88944	VNQHLCGSH	RGLEPGKGR	VNQHLCGSHRGLEPGKGR		SCG1	38489/367830	0.927	88944	0.767	-0.034134	-0.9167	0.5	4	18	-1.0833	2	0	0	0
HIP_88945	VGQVELGGG	SDSQVSEEV	VGQVELGGGSDSQVSEEV		SCG1	13835/367830	0.996	88945	0.767	-0.094093	-1.05	0.8333	-4	18	-0.2444	-4	0	0	1
HIP_88946	ERGFFYTPK	QENHPQES	ERGFFYTPKQENHPQES		SCG1	42405/367830	0.904	88946	0.767	-0.051974	-2.7833	0.1667	0	17	-1.9647	-1	2	1	0
HIP_88947	GSLQPLALE	NRQMAYENL	GSLQPLALENRQMAYENL		SCG2	44444/334890	0.965	88947	0.767	0.107366	-1.5667	0	-1	18	-0.4944	-1	0	0	0
HIP_88948	CTSICSLYQ	ESLSAIEAE	CTSICSLYQESLSAIEAE		CMGA	16387/247050	0.983	88948	0.767	-0.060067	-0.25	0.1667	-3	18	0.3333	-3	1	0	0
HIP_88949	GGGPGAGSL	KTHWLGAAL	GGGPGAGSLKTHWLGAAL		SCG2	40890/334890	0.975	88949	0.767	0.048212	-0.8667	0.3333	2	18	0.1833	1	1	0	1
HIP_88950	YLVCGERGF	EESWDEED	YLVCGERGFEESWDEED		SCG1	27409/367830	0.974	88950	0.767	-0.045159	-1.65	0.3333	-6	17	-1.1471	-6	2	1	0
HIP_88951	GIVEQCCTS	PMPVSQECF	GIVEQCCTSPMPVSQECF		CMGA	10787/247050	0.994	88951	0.767	-0.154427	-0.05	0.5	-2	18	0.2889	-2	0	0	0
[illegible]																			

HIP_89415	EQCCTSICS	GEQEHSQQ	EQCCTSICSGEQEHSQQ		CMGA	15296/247050	0.986	89415	0.767	0.121788	-0.2	0.3333	-2	17	-1.1294	-3	0	1	0
HIP_89416	GERGFFYT	EDATSEVD	GERGFFYTEDATSEVD		SCG1	35357/367830	0.943	89416	0.767	0.027861	-0.7333	0	-4	16	-0.9187	-4	3	0	0
HIP_89417	EQCCTSICS	PRLPYGAGR	EQCCTSICSPRLPYGAGR		SCG2	38514/334890	0.980	89417	0.767	0.071518	0.65	0.3333	1	18	-0.3333	1	0	0	0
HIP_89418	LVEALYLV	EYIENLRQ	LVEALYLVEYIENLRQ		SCG2	30579/334890	0.990	89418	0.767	0.048681	1.0667	0	-2	16	0.3312	-2	2	0	0
HIP_89419	GGPGAGSLQ	AVVPQGLFR	GGPGAGSLQAVVPQGLFR		CMGA	17729/247050	0.980	89419	0.767	0.115658	1.6167	0.1667	1	18	0.2722	1	0	0	0
HIP_89420	SICSLYQLE	LSQGLVDRE	SICSLYQLELSQGLVDRE		CMGA	19772/247050	0.973	89420	0.767	-0.189454	-0.6167	0.1667	-2	18	0.0167	-2	1	0	0
HIP_89421	IVEQCCTSI	SIYQLIEIS	IVEQCCTSISIYQLIEIS		SCG2	15899/334890	0.997	89421	0.767	-0.056329	0.9	0.3333	-2	18	0.95	-2	1	0	0
HIP_89422	VELGGGPGA	ASEEEPEYG	VELGGGPGAASEEEPEYG		SCG1	9747/367830	0.998	89422	0.767	-0.150126	-0.45	0.5	-5	18	-0.7333	-5	0	0	2
HIP_89423	LGGGPGAGS	PTKADTEKW	LGGGPGAGSPTKADTEKW		SCG1	31875/367830	0.958	89423	0.767	-0.067600	-0.9333	0.5	0	18	-0.8722	0	0	0	1
HIP_89424	GERGFFYT	QETDKLAPV	GERGFFYTQETDKLAPV		SCG2	37241/334890	0.982	89424	0.767	0.119955	-1.15	0	-1	17	-0.7118	-1	3	1	0
HIP_89425	CCTSICSLY	EESEESNV	CCTSICSLYEESEESNV		SCG1	27146/367830	0.975	89425	0.767	0.021023	-1.0167	0.3333	-4	17	-0.1588	-4	1	1	0
HIP_89426	HLVEALYLV	LHYRKKSA	HLVEALYLVLHYRKKSA		SCG1	46028/367830	0.881	89426	0.767	0.060009	1	0	4	17	0.0941	2	2	1	0
HIP_89427	LCGSHLVEA	GKLTGPNNQ	LCGSHLVEAGKLTGPNNQ		SCG2	37429/334890	0.981	89427	0.767	0.137246	0.3333	0.1667	1	18	-0.3056	0	0	0	0
HIP_89428	LVCGERGFF	FNPYYDP	LVCGERGFFFNPYYDP		SCG1	30461/367830	0.964	89428	0.767	0.035789	0.4833	0.3333	-1	16	-0.1687	-1	3	2	1
HIP_89429	GERGFFY	QSSQAEL	GERGFFYQSSQAEL		CMGA	22900/247050	0.961	89429	0.767	0.205216	-0.1333	0.3333	-1	14	-0.7857	-1	3	0	0
HIP_89430	EALYLVC	VGSNTYGK	EALYLVCVGSNTYGK		IAPP	3272/45018	0.973	89430	0.767	0.214539	2.25	0.3333	0	15	0.3	0	1	1	0
HIP_89431	GSLQPLAL	QVLKTSRK	GSLQPLALQVLKTSRK		SCG1	44780/367830	0.890	89431	0.767	0.105622	2.3167	0	3	16	-0.15	3	0	0	0
HIP_89432	VEALYLVCG	GEEDIQGP	VEALYLVCGGEEDIQGP		SCG1	22803/367830	0.985	89432	0.767	0.061094	-0.1833	0.3333	-4	17	0.1882	-4	1	1	0
HIP_89433	GERGFFY	QGLVDRE	GERGFFYQGLVDRE		CMGA	17398/247050	0.981	89433	0.767	0.136386	0.7	0.1667	-1	14	-0.85	-1	3	0	0
HIP_89434	AEDLQVGQV	PEPMQESKA	AEDLQVGQVPEPMQESKA		CMGA	10512/247050	0.994	89434	0.767	-0.100831	-1.0667	0.5	-3	18	-0.8389	-3	0	0	0
HIP_89435	EQCCTSICS	VEEPSSKDV	EQCCTSICSVEEPSSKDV		CMGA	17793/247050	0.980	89435	0.767	-0.071574	0.5667	0.1667	-3	18	-0.3611	-3	0	0	0
HIP_89436	SHLVEALY	GGSLPSEEK	SHLVEALYGGSLPSEEK		SCG1	24399/367830	0.982	89436	0.767	0.204837	0.45	0.5	-1	17	-0.3706	-2	1	1	0
HIP_89437	IVEQCCTSI	LVKYPEIIN	IVEQCCTSILVKYPEIIN		SCG2	21542/334890	0.995	89437	0.767	-0.010626	1.1833	0.1667	-1	18	0.7167	-1	1	0	0
HIP_89438	HLVEALYLV	QVKRVPGQ	HLVEALYLVQVKRVPGQ		SCG2	43607/334890	0.968	89438	0.767	0.200287	0.5833	0	2	17	0.2706	1	1	1	0
HIP_89439	CCTSICSLY	ATCATQRLA	CCTSICSLYATCATQRLA		IAPP	3902/45018	0.960	89439	0.767	-0.063459	0.8833	0.1667	1	18	0.8056	1	1	0	0
HIP_89440	GAGSLQPLA	IENLRQQAH	GAGSLQPLAIENLRQQAH		SCG2	27258/334890	0.992	89440	0.766	-0.081611	0.25	0.1667	1	18	-0.3944	0	0	0	0
HIP_89441	QVGQVELGG	AEPGWQAKR	QVGQVELGGAEPGWQAKR		CMGA	10287/247050	0.994	89441	0.766	-0.122768	-0.05	0.5	0	18	-0.7889	0	0	0	0
HIP_89442	QVGQVELGG	GEEEEEEE	QVGQVELGGGEEEEEEE		CMGA	10995/247050	0.994	89442	0.766	-0.111598	-0.7333	0.5	-8	17	-1.4353	-8	0	1	6
HIP_89443	GSLQPLAL	NVGSNTYGK	GSLQPLALNVGSNTYGK		IAPP	3459/45018	0.969	89443	0.766	0.170174	1.6167	0.1667	1	17	-0.2	1	0	1	0
HIP_89444	SLQPLAL	AGAPGEE	SLQPLALAGAPGEE		SCG1	22394/367830	0.986	89444	0.766	0.066308	2.1	0.1667	-2	14	0.1071	-2	0	0	0
HIP_89445	DLQVGQV	ERGFFYT	DLQVGQVERGFFYT		INSB	430/4968	0.910	89445	0.766	0.021319	-1.35	0.3333	-1	14	-0.25	-1	1	0	0
HIP_89446	ICSLYQLEN	RVPKLDLKR	ICSLYQLENRVPKLDLKR		SCG1	48487/367830	0.864	89446	0.766	0.013799	-0.85	0.1667	2	18	-0.45	2	1	0	0
HIP_89447	GPGAGSLQP	EQEHSQQK	GPGAGSLQPEQEHSQQK		CMGA	27365/247050	0.935	89447	0.766	-0.005189	-1.9667	0.1667	0	17	-1.6765	-1	0	1	0
HIP_89448	CGSHLVEAL	GFEDELSEV	CGSHLVEALGFEDELSEV		CMGA	12179/247050	0.992	89448	0.766	-0.024562	0.1667	0.1667	-4	18	0.2111	-5	1	0	0
HIP_89449	ALYLVCGER	DYETQQWPE	ALYLVCGERDYETQQWPE		SCG2	31674/334890	0.989	89449	0.766	-0.059655	-2.7833	0.1667	-3	18	-0.8667	-3	1	0	0
HIP_89450	VCGERGFF	LKRQYDRVA	VCGERGFFLKRQYDRVA		SCG1	48690/367830	0.862	89450	0.766	0.085758	0.1	0.1667	2	17	-0.4647	2	3	1	0
HIP_89451	AGSLQPLA	PNGSVEPER	AGSLQPLAPNGSVEPER		SCG2	16075/334890	0.997	89451	0.766	0.109302	-0.25	0.5	-1	17	-0.6059	-1	0	1	0
HIP_89452	NQHLCGSHL	WQAKREEEE	NQHLCGSHLWQAKREEEE		CMGA	23265/247050	0.959	89452	0.766	-0.043123	-0.4667	0.1667	0	18	-1.6389	-2	1	0	2
HIP_89453	EALYLVCGE	REEARFQDK	EALYLVCGEREEARFQDK		SCG1	45870/367830	0.882	89453	0.766	-0.095533	-2.15	0.1667	-2	18	-0.8278	-2	0	0	0
HIP_89454	CCTSICSLY	QEESEEGE	CCTSICSLYQEESEEGE		SCG1	30167/367830	0.965	89454	0.766	-0.009203	-1.4667	0.1667	-5	17	-0.5882	-5	1	1	0
HIP_89455	VEALYLV	GAGKPGA	VEALYLVGAGKPGA		CMGA	15258/247050	0.986	89455	0.766	0.195263	1.2833	0.3333	0	14	0.7071	0	1	0	0
HIP_89456	CGSHLVEAL	VLNPHPSLG	CGSHLVEALVLNPHPSLG		CMGA	22176/247050	0.964	89456	0.766	-0.038814	1.1	0	1	18	0.4944	-1	0	0	0
HIP_89457	LVEALYLV	DTPNRQYW	LVEALYLVDTPNRQYW		SCG2	26706/334890	0.993	89457	0.766	0.206482	0.15	0.1667	-1	16	-0.1688	-1	1	0	0
HIP_89458	LCGSHLVEA	ITRQRYGKR	LCGSHLVEAITRQRYGKR		NPY	7607/49410	0.809	89458	0.766	0.099410	0.3	0	4	18	-0.5889	3	0	0	0
HIP_89459	GSHLVEALY	RPQALPEP	GSHLVEALYRPQALPEP		CMGA	25203/247050	0.949	89459	0.766	0.188763	-0.8833	0.1667	0	17	-0.3706	-1	1	1	0
HIP_89460	HLVEALYL	VENRQMAYE	HLVEALYLVENRQMAYE		SCG2	28529/334890	0.991	89460	0.766	0.146324	0.5833	0	-1	17	-0.1471	-2	1	1	0
HIP_89461	RGFFYTPKT	SEGLSQGLV	RGFFYTPKTSEGLSQGLV		CMGA	24131/247050	0.955	89461	0.766	-0.080732	-1.8167	0.5	1	18	-0.2833	1	1	0	0
HIP_89462	RGFFYTPKT	LGIQEEDLR	RGFFYTPKTLGIQEEDLR		SCG2	22956/334890	0.995	89462	0.766	-0.009413	0.2833	0.3333	0	18	-0.7944	0	1	0	0

[illegible]

HIP_89926	SLQPLALEG	DEEQKLYTD	SLQPLALEGDEEQKLYTD		SCG2	29307/334890	0.991	89926	0.766	-0.093330	-1.7667	0.1667	-4	18	-0.9	-4	0	0	0
HIP_89927	VGQVELGGG	HFERRDNMN	VGQVELGGGHFERRDNMN		SCG1	21169/367830	0.988	89927	0.766	0.126103	-0.85	0.5	0	18	-0.9944	-1	1	0	1
HIP_89928	CCTSICSLY	RRDNMNDNF	CCTSICSLYRRDNMNDNF		SCG1	51255/367830	0.841	89928	0.766	0.095796	-1.8	0.1667	0	18	-0.5333	0	1	0	0
HIP_89929	GSLQPLAL	EADLDHPDL	GSLQPLALEADLDHPDL		SCG2	25879/334890	0.993	89929	0.766	0.074177	0.7	0	-3	17	-0.1471	-4	0	1	0
HIP_89930	EAEDLQVGQ	GYEKRNLAR	EAEDLQVGQGYEKRNLAR		SCG1	26205/367830	0.977	89930	0.766	-0.105279	-0.8167	0.3333	-1	18	-1.3389	-1	1	0	0
HIP_89931	IVEQCCTSI	HQQKKHSGF	IVEQCCTSIHQQKKHSGF		CMGA	37729/247050	0.827	89931	0.766	0.008624	-1.2	0.1667	3	18	-0.55	1	0	0	0
HIP_89932	HLVEALYL	RGEAGAPGE	HLVEALYLRGEAGAPGE		SCG1	33441/367830	0.952	89932	0.766	0.224474	-0.35	0.1667	-1	17	-0.0765	-2	1	1	0
HIP_89933	EQCCTSICS	RNLQIPPED	EQCCTSICSRNLQIPPED		SCG2	35834/334890	0.984	89933	0.766	-0.001232	0.3333	0.1667	-2	18	-0.5944	-2	0	0	0
HIP_89934	CGSHLVEAL	EEKKEEEGS	CGSHLVEALEEKKEEEGS		CMGA	27565/247050	0.934	89934	0.766	0.092891	-1.4667	0	-3	18	-1.0167	-4	0	0	1
HIP_89935	GIVEQCCTS	ELESLSAIE	GIVEQCCTSELESLSAIE		CMGA	18950/247050	0.976	89935	0.766	-0.101865	-0.3667	0.1667	-4	18	0.3667	-4	0	0	0
HIP_89936	SHLVEALY	GQNGERAT	SHLVEALYGQNGERAT		SCG2	27265/334890	0.992	89936	0.766	0.202831	-0.5167	0.1667	0	16	-0.6187	-1	1	0	0
HIP_89937	ERGFFYTP	EEEGPTVV	ERGFFYTPEEEGPTVV		CMGA	24449/247050	0.953	89937	0.766	-0.016537	-2.35	0.1667	-3	16	-0.7	-3	3	0	1
HIP_89938	YLVCGERGF	ENQMDKAR	YLVCGERGFENQMDKAR		SCG1	39508/367830	0.921	89938	0.766	0.001337	-2.1	0.1667	0	17	-0.9118	0	1	1	0
HIP_89939	GERGFFYT	RVPGQGSSE	GERGFFYTRVPGQGSSE		SCG2	33138/334890	0.987	89939	0.766	0.014376	-0.1833	0.1667	0	17	-0.9706	0	3	1	0
HIP_89940	VEQCCTSIC	WMRIILEAL	VEQCCTSICWMRIILEAL		SCG2	33869/334890	0.986	89940	0.766	-0.036822	0.45	0.1667	-1	18	1.0611	-1	1	0	0
HIP_89941	VELGGGPGA	NGDESHLPE	VELGGGPGANGDESHLPE		SCG2	14801/334890	0.998	89941	0.766	-0.087922	-1.2667	0.5	-3	18	-0.7278	-4	0	0	1
HIP_89942	EAEDLQVGQ	TENVPRTRL	EAEDLQVGQTENVPRTRL		NPY	3641/49410	0.965	89942	0.766	-0.164947	-1.2333	0.1667	-2	18	-1.0611	-2	0	0	0
HIP_89943	SLYQLENYC	KANNIAYED	SLYQLENYCKANNIAYED		SCG2	36567/334890	0.983	89943	0.766	-0.037684	-1.3167	0	-2	18	-0.8278	-2	1	0	0
HIP_89944	YLVCGERGF	EEVRDSKEN	YLVCGERGFEEVRDSKEN		SCG2	31958/334890	0.988	89944	0.766	-0.031858	-0.8167	0.1667	-2	18	-1.0722	-2	1	0	0
HIP_89945	HLVEALYL	ENLNDKDQ	HLVEALYLENLNDKDQ		SCG2	29578/334890	0.990	89945	0.766	0.157940	0.5167	0	-2	16	-0.7313	-3	1	0	0
HIP_89946	SLYQLENYC	LEAGLPLQV	SLYQLENYCLEAGLPLQV		CMGA	20646/247050	0.970	89946	0.766	-0.154345	-0.0333	0	-2	18	0.2556	-2	1	0	0
HIP_89947	HLVEALYLV	VPRTRLEDP	HLVEALYLVVPRTRLEDP		NPY	4297/49410	0.950	89947	0.766	0.135204	0.8	0.1667	0	18	0.0944	-1	1	0	0
HIP_89948	LYQLENYCN	DRNYLNYG	LYQLENYCNDRNYLNYG		SCG1	45473/367830	0.885	89948	0.766	0.134748	-2.3	0	-1	17	-1.2176	-1	2	1	0
HIP_89949	CGSHLVEAL	SRSEALAVD	CGSHLVEALSRSEALAVD		CMGA	25476/247050	0.947	89949	0.766	0.160314	-0.6667	0.3333	-1	18	0.3722	-2	0	0	0
HIP_89950	VEALYLVCG	IVEEQYTPQ	VEALYLVCGIVEEQYTPQ		SCG2	16809/334890	0.997	89950	0.766	-0.075838	1.9167	0.1667	-3	18	0.3444	-3	1	0	0
HIP_89951	PGAGSLQPL	SSKDVMEKR	PGAGSLQPLSSKDVMEKR		CMGA	28290/247050	0.929	89951	0.766	0.076684	-1.1333	0.5	1	18	-0.7611	1	0	0	0
HIP_89952	GGGPGAGSL	PMYEENSRD	GGGPGAGSLPMYEENSRD		SCG2	10858/334890	0.999	89952	0.766	-0.024175	0.2667	0.5	-2	18	-1.0611	-2	1	0	1
HIP_89953	IVEQCCTSI	ESVFQELGK	IVEQCCTSIESVFQELGK		SCG2	19373/334890	0.996	89953	0.766	-0.116119	0.4833	0.3333	-2	18	0.2722	-2	1	0	0
HIP_89954	VNQHLCGSH	YGEEGAPGK	VNQHLCGSHYGEEGAPGK		SCG1	32078/367830	0.957	89954	0.766	-0.107890	-1.6	0.5	1	18	-0.9611	-1	1	0	0
HIP_89955	ALYLVCGER	DSKEAEKSG	ALYLVCGERDSKEAEKSG		CMGA	29978/247050	0.914	89955	0.766	-0.090971	-2.7667	0.3333	-1	18	-0.6722	-1	0	0	0
HIP_89956	QHLCGSHLV	SPETLISDL	QHLCGSHLVSPETLISDL		NPY	3763/49410	0.962	89956	0.766	0.203103	-0.1833	0.3333	0	18	0.2444	-2	0	0	0
HIP_89957	NQHLCGSHL	PSPEMIRAL	NQHLCGSHLPSPEMIRAL		SCG2	25115/334890	0.994	89957	0.766	0.131189	-0.7	0.6667	2	18	-0.25	0	0	0	0
HIP_89958	SLYQLENYC	VVGGEDWNP	SLYQLENYCVVGGEDWNP		SCG2	14706/334890	0.998	89958	0.766	-0.169357	0.95	0.1667	-3	18	-0.5111	-3	1	0	0
HIP_89959	EALYLVCGE	KGQPRSQEE	EALYLVCGEKGQPRSQEE		SCG1	36406/367830	0.938	89959	0.766	-0.059647	-1.5333	0.3333	-2	18	-0.9889	-2	0	0	0
HIP_89960	CCTSICS	GGGHSRERA	CCTSICSGGGHSRERA		SCG1	25443/367830	0.979	89960	0.766	0.196787	0.8333	0.6667	2	16	-0.3875	1	0	2	1
HIP_89961	SHLVEALYL	ATLESVFQ	SHLVEALYLATLESVFQ		SCG2	29540/334890	0.990	89961	0.766	0.035417	1.8667	0	-1	17	0.7471	-2	1	1	0
HIP_89962	GIVEQCCTS	EKTHSREKS	GIVEQCCTSEKTHSREKS		SCG1	57053/367830	0.789	89962	0.766	0.051267	-1.1833	0.1667	1	18	-1.1111	0	0	0	0
HIP_89963	VEQCCTSIC	TALPVNSPM	VEQCCTSICTALPVNSPM		CMGA	19183/247050	0.975	89963	0.766	-0.034860	1.85	0.1667	-1	18	0.6222	-1	0	0	0
HIP_89964	AEDLQVGQV	REEARFQDK	AEDLQVGQVREEARFQDK		SCG1	38200/367830	0.928	89964	0.766	-0.057554	-1.8667	0.1667	-2	18	-1.2611	-2	0	0	0
HIP_89965	LQPLALEGS	TSEVDKRRT	LQPLALEGSTSEVDKRRT		SCG1	35741/367830	0.941	89965	0.766	-0.126122	-1.6167	0.5	0	18	-0.8056	0	0	0	0
HIP_89966	GSHLVEALY	KENKPYAL	GSHLVEALYKENKPYAL		SCG2	39316/334890	0.978	89966	0.766	0.214578	-1.1	0	1	17	-0.4529	0	1	1	0
HIP_89967	GGGPGAG	GERGFFYTP	GGGPGAGGERGFFYTP		INSB	444/4968	0.905	89967	0.766	-0.012607	-1.2333	0.5	0	16	-0.5375	0	1	2	1
HIP_89968	LVEALYL	EEEEEEE	LVEALYLEEEEEEE		CMGA	18353/247050	0.978	89968	0.766	0.108477	-0.7	0	-8	14	-0.85	-8	1	0	5
HIP_89969	LVEALYL	EEEEEEE	LVEALYLEEEEEEE		CMGA	18354/247050	0.978	89969	0.766	0.108477	-0.7	0	-8	14	-0.85	-8	1	0	5
HIP_89970	LVEALYL	EEEEEEE	LVEALYLEEEEEEE		CMGA	18355/247050	0.978	89970	0.766	0.108477	-0.7	0	-8	14	-0.85	-8	1	0	5
HIP_89971	LVEALYL	EEEEEEE	LVEALYLEEEEEEE		CMGA	18356/247050	0.978	89971	0.766	0.108477	-0.7	0	-8	14	-0.85	-8	1	0	5
HIP_89972	LVEALYL	EEEEEEE	LVEALYLEEEEEEE		CMGA	18357/247050	0.978	89972	0.766	0.108477	-0.7	0	-8	14	-0.85	-8	1	0	5
HIP_89973	RGFFYTPKT	LTLALSLLV	RGFFYTPKTLTLALSLLV		NPY	4199/49410	0.953	89973	0.766	0.079313	0.1167	0.1667	2	18	0.8889	2	1	0	0

[illegible]

HIP_90437	ERGFFYTPK	PEEKKEEE	ERGFFYTPKPEEKKEEE		CMGA	25561/247050	0.947	90437	0.766	-0.081187	-2.4667	0.3333	-2	17	-2.1882	-2	2	1
HIP_90438	EDLQVGQVE	GERGEDSSE	EDLQVGQVEGERGEDSSE		SCG1	31025/367830	0.962	90438	0.766	-0.085085	-1.8667	0.1667	-6	18	-1.4778	-6	0	0
HIP_90439	QHLCGSHLV	GHPQEESEE	QHLCGSHLVGHPQEESEE		SCG1	22200/367830	0.986	90439	0.766	0.155136	-0.0667	0.3333	-1	18	-1.1278	-4	0	0
HIP_90440	AGSLQPLAL	YPSLELDK	AGSLQPLALYPSLELDK		SCG1	30020/367830	0.965	90440	0.766	0.191139	0.95	0.3333	-1	17	0.1	-1	1	1
HIP_90441	QCCTSICSL	SLEAGLPLQ	QCCTSICSLSLEAGLPLQ		CMGA	18671/247050	0.977	90441	0.766	0.027881	0.8333	0.3333	-1	18	0.7444	-1	0	0
HIP_90442	CGERGFFYT	EKPLDSQS	CGERGFFYTEKPLDSQS		SCG2	37151/334890	0.982	90442	0.766	0.128091	-1.3667	0.1667	-1	17	-0.9706	-1	3	1
HIP_90443	GSLQPLALE	IEKNEQIND	GSLQPLALEIEKNEQIND		SCG2	37356/334890	0.982	90443	0.766	-0.049692	-0.1333	0	-3	18	-0.6944	-3	0	0
HIP_90444	EQCCTSICS	EDATSEVDK	EQCCTSICSEDATSEVDK		SCG1	27788/367830	0.973	90444	0.766	-0.048687	0.1667	0.1667	-4	18	-0.5944	-4	0	0
HIP_90445	HLVEALYLV	INSNQVKR	HLVEALYLVINSNQVKR		SCG2	30284/334890	0.990	90445	0.766	0.147573	1.15	0.1667	2	17	0.1529	1	1	1
HIP_90446	EQCCTSICS	LAAMDLELQ	EQCCTSICSLAAMDLELQ		SCG1	23568/367830	0.983	90446	0.766	-0.020303	2.2667	0.1667	-3	18	0.5056	-3	0	0
HIP_90447	LCGSHLVEA	EEEEDNRD	LCGSHLVEAEEEEDNRD		CMGA	21907/247050	0.965	90447	0.766	0.064740	-1.3333	0	-5	17	-1.2235	-6	0	1
HIP_90448	YLVCGERGF	DLRLENVQK	YLVCGERGFDLRLENVQK		SCG2	37097/334890	0.982	90448	0.766	-0.031898	-1.05	0.1667	0	18	-0.4111	0	1	0
HIP_90449	LGGGPGAGS	FEDELSEVL	LGGGPGAGSFEDELSEVL		CMGA	11484/247050	0.993	90449	0.766	-0.106191	-0.6	0.3333	-4	18	0.0556	-4	1	0
HIP_90450	DLQVGQVEL	REEEEEEE	DLQVGQVELREEEEEEEE		CMGA	21046/247050	0.968	90450	0.766	0.047769	-1.1667	0	-9	18	-1.7167	-9	0	0
HIP_90451	GSLQPLAL	RGEDSSEEK	GSLQPLALRGEDSSEEK		SCG1	32942/367830	0.954	90451	0.766	0.223626	0.1667	0.1667	-2	17	-1.0294	-2	0	1
HIP_90452	CGERGFFYT	LSEEDWMR	CGERGFFYTLSEEDWMR		SCG2	27508/334890	0.992	90452	0.766	-0.006505	0.05	0.1667	-2	17	-0.8059	-2	3	1
HIP_90453	LYQLENYCN	TPNRQYWD	LYQLENYCNTPNRQYWD		SCG2	27567/334890	0.992	90453	0.766	0.091392	-1.35	0.1667	-1	17	-1.5294	-1	1	1
HIP_90454	GERGFFYT	FSEDVNWG	GERGFFYTFSEDVNWG		SCG1	32243/367830	0.957	90454	0.766	0.020914	-0.1167	0.1667	-2	16	-0.675	-2	4	0
HIP_90455	QCCTSICSL	RRGWRPSSR	QCCTSICSLRRGWRPSSR		CMGA	35055/247050	0.861	90455	0.766	0.198899	-0.65	0.3333	4	18	-0.6944	4	1	0
HIP_90456	HLVEALY	DNPGEDAPA	HLVEALYDNPGEDAPA		NPY	3972/49410	0.958	90456	0.766	0.201822	-0.7167	0.1667	-3	16	-0.525	-4	1	2
HIP_90457	GAGSLQPLA	GIQEEDLRK	GAGSLQPLAGIQEEDLRK		SCG2	12770/334890	0.998	90457	0.766	-0.043971	0.7667	0.3333	-1	18	-0.5556	-1	0	0
HIP_90458	LCGSHLVEA	SKRWSKMDQ	LCGSHLVEASKRWSKMDQ		CMGA	36612/247050	0.842	90458	0.766	0.184848	-1.1167	0.1667	2	18	-0.65	1	1	0
HIP_90459	PGAGSLQPL	EQINDEMKR	PGAGSLQPLEQINDEMKR		SCG2	31885/334890	0.988	90459	0.766	-0.063968	-0.6333	0.1667	-1	18	-1.0222	-1	0	0
HIP_90460	CTSICSLY	EEEEEEEAE	CTSICSLYEEEEEEEAE		CMGA	18640/247050	0.977	90460	0.766	0.002075	-1.4667	0.1667	-8	17	-0.9706	-8	1	1
HIP_90461	GERGFFY	NFLVHSS	GERGFFYNFLVHSS		IAPP	4185/45018	0.953	90461	0.766	0.023270	1.2333	0	1	14	-0.1429	0	4	0
HIP_90462	VCGERGFF	TYGKRNA	VCGERGFFTYGKRNA		IAPP	4686/45018	0.938	90462	0.766	0.086356	0.4667	0.3333	2	15	-0.6	2	3	1
HIP_90463	CGERGFF	TYGKRNAV	CGERGFFTYGKRNAV		IAPP	4687/45018	0.938	90463	0.766	0.086356	0.4667	0.3333	2	15	-0.6	2	3	1
HIP_90464	VEALYLVC	NIEKNEQIN	VEALYLVCNIEKNEQIN		SCG2	28523/334890	0.991	90464	0.766	0.133075	1.3333	0	-2	17	-0.0235	-2	1	1
HIP_90465	GGGPGAGSL	WQQQGDLQD	GGGPGAGSLWQQQGDLQD		SCG1	27045/367830	0.975	90465	0.766	-0.031033	-0.8833	0.3333	-2	18	-0.9611	-2	1	0
HIP_90466	SLQPLALEG	SQECFETLR	SLQPLALEGSQECFETLR		CMGA	20665/247050	0.970	90466	0.766	-0.141736	-1.3167	0.3333	-2	18	-0.2222	-2	0	0
HIP_90467	VNQHLCGSH	WDEDLLMKV	VNQHLCGSHWDEDLLMKV		SCG2	29829/334890	0.990	90467	0.766	-0.086936	-2.05	0.3333	0	18	-0.3167	-2	1	0
HIP_90468	GERGFFYT	SSQESGEE	GERGFFYTSSQESGEE		SCG1	30878/367830	0.962	90468	0.766	0.142948	-0.7167	0.3333	-3	16	-1.375	-3	3	0
HIP_90469	VELGGGPGA	NFPMDMSDD	VELGGGPGANFPMDMSDD		SCG2	8967/334890	0.999	90469	0.766	-0.138588	-0.4167	0.5	-4	18	-0.3722	-4	1	0
HIP_90470	CGERGFFY	PQWSLYPS	CGERGFFYPQWSLYPS		SCG1	37521/367830	0.932	90470	0.766	0.139784	-0.2833	0.1667	0	16	-0.5438	0	4	0
HIP_90471	GERGFFYTP	PSLELDKM	GERGFFYTPPSLELDKM		SCG1	24736/367830	0.981	90471	0.766	-0.139977	-0.3667	0.5	-1	17	-0.6235	-1	3	1
HIP_90472	GGPGAGSLQ	LEALRQAEN	GGPGAGSLQLEALRQAEN		SCG2	26214/334890	0.993	90472	0.766	-0.070459	0.2667	0.1667	-1	18	-0.5111	-1	0	0
HIP_90473	VEALYLV	FGAILSS	VEALYLVFGAILSS		IAPP	3097/45018	0.976	90473	0.766	0.020842	1.8167	0.1667	-1	14	1.7071	-1	2	0
HIP_90474	SICSLYQLE	ARSETHAAG	SICSLYQLEARSETHAAG		SCG1	44097/367830	0.894	90474	0.766	-0.117680	-1.1167	0.1667	0	18	-0.1667	-1	1	0
HIP_90475	GERGFFY	GDSEGLS	GERGFFYGDSEGLS		CMGA	20419/247050	0.971	90475	0.766	0.194201	-0.0667	0.3333	-2	14	-0.7214	-2	3	0
HIP_90476	EQCCTSICS	LLMKVLEYL	EQCCTSICSLLMKVLEYL		SCG2	24166/334890	0.994	90476	0.766	0.018244	2.6167	0.1667	-1	18	0.85	-1	0	0
HIP_90477	VEALYLVC	NGQNGERA	VEALYLVCNGQNGERA		SCG2	28283/334890	0.991	90477	0.765	0.212002	0.5167	0.1667	-1	16	-0.125	-1	1	0
HIP_90478	ELGGGPGAG	NPYYDPLQW	ELGGGPGAGNPYYDPLQW		SCG1	22870/367830	0.985	90478	0.765	-0.053721	-0.9	0.5	-2	18	-0.8278	-2	2	0
HIP_90479	VELGGGPGA	RYYSALRHY	VELGGGPGARYYSALRHY		NPY	4907/49410	0.936	90479	0.765	0.076382	-1.2167	0.3333	2	18	-0.4556	1	2	0
HIP_90480	QPLALEGSL	SEEKGHPQE	QPLALEGSLSEEKGHPQE		SCG1	25987/367830	0.978	90480	0.765	-0.092710	-0.8667	0.5	-2	18	-1.1389	-3	0	0
HIP_90481	GQVELGGGP	REDSKEAEK	GQVELGGGPREDSKEAEK		CMGA	17406/247050	0.981	90481	0.765	-0.093754	-2.3167	0.5	-2	18	-1.5278	-2	0	0
HIP_90482	LVEALYLVC	ELQDLALQ	LVEALYLVCELQDLALQ		CMGA	20080/247050	0.972	90482	0.765	0.109190	1.2167	0	-3	17	1.0882	-3	1	1
HIP_90483	SHLVEALYL	ADLDHPDL	SHLVEALYLADLDHPDL		SCG2	29042/334890	0.991	90483	0.765	0.030014	1.4	0	-2	17	0.1588	-4	1	1
HIP_90484	HLVEALYLV	HPSLGYKE	HLVEALYLVHPSLGYKE		CMGA	21351/247050	0.967	90484	0.765	0.172738	0.1833	0.3333	1	17	0.1588	-1	1	1
HIP_90485	GGGPGAGSL	SEVDKRRTR	GGGPGAGSLSEVDKRRTR		SCG1	35836/367830	0.941	90485	0.765	0.017604	0.4167	0.5	2	18	-1.1389	2	0	0
HIP_90486	DLQVGQVEL	DATSEVDKR	DLQVGQVELDATSEVDKR		SCG1	37798/367830	0.931	90486	0.765	0.036033	0.35	0	-3	18	-0.7111	-3	0	0
HIP_90487	TSICSLYQL	TPGRAGTE	TSICSLYQLTPGRAGTE		SCG2	22768/334890	0.995	90487	0.765	0.166577	-0.6167	0.3333	0	17	-0.1471	0	1	1
HIP_90488	SLYQLENYC	EDLLMKVLE	SLYQLENYCEDLLMKVLE		SCG2	29793/334890	0.990	90488	0.765	-0.124558	-0.9167	0	-3	18	-0.0389	-3	1	0
HIP_90489	GIVEQCCTS	SPMNKGDTE	GIVEQCCTSSPMNKGDTE		CMGA	13823/247050	0.989	90489	0.765	-0.115150	0.0833	0.5	-2	18	-0.6222	-2	0	0
HIP_90490	IVEQCCTSI	FEDELSEVL	IVEQCCTSIFEDELSEVL		CMGA	16610/247050	0.983	90490	0.765	-0.096747	-0.2	0.1667	-5	18	0.5278	-5	1	0
HIP_90491	CCTSICSLY	MKVLEYLNQ	CCTSICSLYMKVLEYLNQ		SCG2	29496/334890	0.991	90491	0.765	-0.078477	0.65	0.1667	0	18	0.5667	0	1	0
HIP_90492	LVEALYL	AEGNNQA	LVEALYLAEGNNQA		CMGA	17139/247050	0.981	90492	0.765	0.156797	0.7	0.1667	-2	14	0.1286	-2	1	0

HIP_90948	CGSHLVEA	EEEEEEEEE	CGSHLVEAEEEEEEEEE		CMGA	18349/247050	0.978	90948	0.765	-0.004501	-1.3333	0	-9	17	-1.5941	-10	0	1	7	[illegible]	[illegible]
HIP_90949	GSHLVEALY	DVSKVIAY	GSHLVEALYDVSKVIAY		SCG2	27503/334890	0.992	90949	0.765	0.141625	0.7	0.1667	0	17	0.5647	-1	1	1	0	[illegible]	[illegible]
HIP_90950	EAEDLQVG	EEEEEEEEA	EAEDLQVGEEEEEEEEA		CMGA	15769/247050	0.985	90950	0.765	-0.099937	-1.7	0.1667	-11	17	-1.8118	-11	0	1	6	[illegible]	[illegible]
HIP_90951	EAEDLQVG	EEEEEEEEA	EAEDLQVGEEEEEEEEA		CMGA	15770/247050	0.985	90951	0.765	-0.099937	-1.7	0.1667	-11	17	-1.8118	-11	0	1	6	[illegible]	[illegible]
HIP_90952	EAEDLQVGQ	SALRHYINL	EAEDLQVGQSALRHYINL		NPY	1436/49410	0.992	90952	0.765	-0.042026	0.85	0.3333	-1	18	-0.4167	-2	0	0	0	[illegible]	[illegible]
HIP_90953	RGFFYTPKT	LKRLVNAAG	RGFFYTPKTLKRLVNAAG		SCG2	45256/334890	0.962	90953	0.765	0.002661	-1.8	0.1667	4	18	-0.2444	4	1	0	0	[illegible]	[illegible]
HIP_90954	LVCGERGFF	CIVEVISD	LVCGERGFFCIVEVISD		CMGA	12545/247050	0.991	90954	0.765	-0.005281	2.7333	0.1667	-2	17	1.1412	-2	2	1	0	[illegible]	[illegible]
HIP_90955	VEALYLVCG	EETGSQEN	VEALYLVCGEETGSQEN		SCG1	27542/367830	0.973	90955	0.765	0.021048	-0.2333	0.1667	-4	17	-0.2529	-4	1	1	0	[illegible]	[illegible]
HIP_90956	CGSHLVEAL	PGEEEEEEE	CGSHLVEALPGEEEEEEE		CMGA	11233/247050	0.993	90956	0.765	0.051887	-0.5667	0.3333	-7	18	-1.0167	-8	0	0	5	[illegible]	[illegible]
HIP_90957	EQCCTSICS	SALRHYIN	EQCCTSICSSALRHYIN		NPY	4651/49410	0.942	90957	0.765	0.003741	1.8333	0.3333	1	17	-0.0294	0	0	1	0	[illegible]	[illegible]
HIP_90958	LQPLALEGS	PSEEKGHPQ	LQPLALEGSPSEEKGHPQ		SCG1	29449/367830	0.967	90958	0.765	-0.123109	-1.7667	0.6667	-1	18	-1.0333	-2	0	0	0	[illegible]	[illegible]
HIP_90959	LVCGERGFF	LDSQSIYQ	LVCGERGFFLDSQSIYQ		SCG2	24618/334890	0.994	90959	0.765	0.003231	0.7833	0.3333	-1	17	0.1294	-1	2	1	0	[illegible]	[illegible]
HIP_90960	GERGFFYTP	GSANRRPE	GERGFFYTPGSANRRPE		CMGA	18804/247050	0.976	90960	0.765	-0.107339	-0.5	0.5	1	17	-1.4	1	3	1	0	[illegible]	[illegible]
HIP_90961	LGGGPGAGS	DSQVSEEVK	LGGGPGAGSDSQVSEEVK		SCG1	20783/367830	0.988	90961	0.765	-0.144889	-1.2	0.5	-2	18	-0.55	-2	0	0	1	[illegible]	[illegible]
HIP_90962	SLYQLENYC	LSSTNVGSN	SLYQLENYCLSSTNVGSN		IAPP	2592/45018	0.984	90962	0.765	-0.097480	-0.0167	0.3333	-1	18	-0.35	-1	1	0	0	[illegible]	[illegible]
HIP_90963	VEALYLVCG	EPDLRLENV	VEALYLVCGEPDLRLENV		SCG2	22321/334890	0.995	90963	0.765	0.022695	-0.3833	0.3333	-3	18	0.3778	-3	1	0	0	[illegible]	[illegible]
HIP_90964	TSICSLYQL	DLKRQYDRV	TSICSLYQLDLKRQYDRV		SCG1	57940/367830	0.779	90964	0.765	0.128974	-0.7667	0	1	18	-0.5111	1	1	0	0	[illegible]	[illegible]
HIP_90965	QCCTSICSL	VSKRFPVGP	QCCTSICSLVSKRFPVGP		SCG2	42021/334890	0.972	90965	0.765	0.093051	0.8333	0.3333	2	18	0.4667	2	0	0	0	[illegible]	[illegible]
HIP_90966	VNQHLCGSH	KSSHFERRD	VNQHLCGSHKSSHFERRD		SCG1	43415/367830	0.899	90966	0.765	-0.039046	-1.65	0.6667	4	18	-1.4444	1	0	0	0	[illegible]	[illegible]
HIP_90967	GERGFFYTP	SSHHTAEK	GERGFFYTPSSHHTAEK		SCG1	38893/367830	0.924	90967	0.765	-0.057928	-1.4	0.5	2	17	-1.2412	0	3	1	0	[illegible]	[illegible]
HIP_90968	IVEQCCTSI	GDSEGLSQG	IVEQCCTSIGDSEGLSQG		CMGA	8797/247050	0.996	90968	0.765	0.003336	-0.2833	0.5	-3	18	0.0111	-3	0	0	0	[illegible]	[illegible]
HIP_90969	ICSLYQLEN	ADEPQWSLY	ICSLYQLENADEPQWSLY		SCG1	41567/367830	0.909	90969	0.765	-0.085490	-1.4	0	-3	18	-0.4167	-3	1	0	0	[illegible]	[illegible]
HIP_90970	GERGFFYT	SDSQVSEE	GERGFFYTSDSQVSEE		SCG1	30589/367830	0.963	90970	0.765	0.143290	-0.7167	0.3333	-3	16	-1.0875	-3	3	0	0	[illegible]	[illegible]
HIP_90971	VEQCCTSIC	HESSSRGEA	VEQCCTSICHESSSRGEA		SCG1	29875/367830	0.966	90971	0.765	-0.026453	-0.2167	0.3333	-1	18	-0.4444	-2	0	0	1	[illegible]	[illegible]
HIP_90972	ELGGGPGAG	FLIVLSVA	ELGGGPGAGFLIVLSVA		IAPP	1937/45018	0.991	90972	0.765	0.108203	2.0167	0.3333	-1	17	1.3412	-1	1	1	1	[illegible]	[illegible]
HIP_90973	NQHLCGSHL	PDLFQNRML	NQHLCGSHLPDLFQNRML		SCG2	33480/334890	0.987	90973	0.765	-0.020215	-0.25	0.3333	2	18	-0.4889	0	1	0	0	[illegible]	[illegible]
HIP_90974	EQCCTSICS	ETQQWPERK	EQCCTSICSETQQWPERK		SCG2	31137/334890	0.989	90974	0.765	0.101667	-0.25	0.1667	-1	18	-1.2722	-1	0	0	0	[illegible]	[illegible]
HIP_90975	LVEALYLVC	QIPPEDLI	LVEALYLVCQIPPEDLI		SCG2	22503/334890	0.995	90975	0.765	0.116275	1.65	0.1667	-3	17	1.0824	-3	1	1	0	[illegible]	[illegible]
HIP_90976	GERGFFYT	SKEAEKSGE	GERGFFYTSKEAEKSGE		CMGA	29221/247050	0.921	90976	0.765	0.146956	-1.2333	0.1667	-1	17	-1.3941	-1	3	1	0	[illegible]	[illegible]
HIP_90977	SHLVEALYL	AGLPLQVR	SHLVEALYLAGLPLQVR		CMGA	19389/247050	0.974	90977	0.765	0.092030	1.9167	0.1667	1	17	0.7176	0	1	1	0	[illegible]	[illegible]
HIP_90978	DLQVGQVEL	DRNYLNYGE	DLQVGQVELDRNYLNYGE		SCG1	47201/367830	0.873	90978	0.765	0.170017	-1.1667	0	-3	18	-0.8944	-3	1	0	0	[illegible]	[illegible]
HIP_90979	DLQVGQVEL	ITRQRYGKR	DLQVGQVELITRQRYGKR		NPY	7446/49410	0.818	90979	0.765	0.094718	0.6333	0	2	18	-0.9556	2	0	0	0	[illegible]	[illegible]
HIP_90980	LYLVCGERG	GEYLARMLV	LYLVCGERGGEYLARMLV		SCG2	32448/334890	0.988	90980	0.765	-0.093194	-2.2667	0.3333	0	18	0.5556	0	1	0	0	[illegible]	[illegible]
HIP_90981	VELGGGPGA	NNQAPGEEE	VELGGGPGANNQAPGEEE		CMGA	14082/247050	0.989	90981	0.765	-0.124680	-1.7833	0.3333	-4	18	-1.0056	-4	0	0	2	[illegible]	[illegible]
HIP_90982	LYQLENYCN	NTYGKRNA	LYQLENYCNNTYGKRNA		IAPP	5877/45018	0.893	90982	0.765	0.040859	-1.3	0	1	17	-1.3235	1	2	1	0	[illegible]	[illegible]
HIP_90983	GIVEQCCTS	KAAWIPHVE	GIVEQCCTSKAAWIPHVE		SCG2	31050/334890	0.989	90983	0.765	0.040341	0.1167	0.1667	0	18	0.2222	-1	1	0	0	[illegible]	[illegible]
HIP_90984	SLQPLALEG	LIVLSVALN	SLQPLALEGLIVLSVALN		IAPP	2436/45018	0.986	90984	0.765	0.080104	2.0667	0.1667	-1	18	1.4	-1	0	0	0	[illegible]	[illegible]
HIP_90985	FVNQHLCGS	GDSEGLSQG	FVNQHLCGSGDSEGLSQG		CMGA	14265/247050	0.988	90985	0.765	-0.047419	-0.5667	0.6667	-1	18	-0.4222	-2	0	0	0	[illegible]	[illegible]
HIP_90986	EALYLVCGE	TQNAFLNER	EALYLVCGETQNAFLNER		SCG1	34519/367830	0.947	90986	0.765	-0.135578	-1.5167	0.1667	-2	18	-0.1889	-2	0	0	0	[illegible]	[illegible]
HIP_90987	VCGERGFF	GEAGAPGE	VCGERGFFGEAGAPGE		SCG1	24259/367830	0.982	90987	0.765	0.027865	0.5167	0.3333	-2	16	-0.1688	-2	2	0	0	[illegible]	[illegible]
HIP_90988	LVEALYLV	AHESSSRGE	LVEALYLVAHESSSRGE		SCG1	36135/367830	0.939	90988	0.765	0.045988	0.3	0	-1	17	0.0647	-2	1	1	1	[illegible]	[illegible]
HIP_90989	SLYQLENYC	LEEPGETQN	SLYQLENYCLEEPGETQN		SCG1	32384/367830	0.956	90989	0.765	-0.135712	-0.9167	0	-4	18	-1.1222	-4	1	0	0	[illegible]	[illegible]
HIP_90990	AGSLQPLAL	VKYPEIIN	AGSLQPLALVKYPEIIN		SCG2	40036/334890	0.977	90990	0.765	0.099396	1.4	0	0	17	0.4765	0	1	1	0	[illegible]	[illegible]
HIP_90991	EQCCTSICS	KSQREDEEE	EQCCTSICSKSQREDEEE		SCG1	33728/367830	0.950	90991	0.765	0.097125	-0.3333	0.3333	-4	18	-1.5278	-4	0	0	1	[illegible]	[illegible]
HIP_90992	CGSHLVEAL	SEEYRAPR	CGSHLVEALSEEYRAPR		SCG1	41634/367830	0.909	90992	0.765	0.167587	-0.95	0.1667	0	17	-0.5706	-1	1	1	0	[illegible]	[illegible]
HIP_90993	SICSLYQLE	ISEADLDHP	SICSLYQLEISEADLDHP		SCG2	26394/334890	0.993	90993	0.765	-0.198184	-0.5	0.1667	-3	18	-0.0722	-4	1	0	0	[illegible]	[illegible]
HIP_90994	AGSLQPLA	TLISDLLMR	AGSLQPLATLISDLLMR		NPY	3823/49410	0.961	90994	0.765	-0.015157	1.9333	0.1667	0	17	0.7765	0	0	1	0	[illegible]	[illegible]
HIP_90995	CSLYQLENY	PMPVSQECF	CSLYQLENYPMPVSQECF		CMGA	15868/247050	0.985	90995	0.765	-0.184522	-1.6	0.3333	-2	18	-0.1889	-2	1	0	0	[illegible]	[illegible]
HIP_90996	SICSLYQL	PEPMQESK	SICSLYQLPEPMQESK		CMGA	18315/247050	0.978	90996	0.765	0.153811	-1.2833	0.3333	-1	16	-0.5188	-1	1	0	0	[illegible]	[illegible]
HIP_90997	LYLVCGERG	RGPGPQLRR	LYLVCGERGRGPGPQLRR		CMGA	28774/247050	0.924	90997	0.765	-0.036055	-2.4833	0.5	3	18	-0.7222	3	0	0	0	[illegible]	[illegible]
HIP_90998	VELGGGPGA	HWLGAALSL	VELGGGPGAHWLGAALSL		SCG2	15078/334890	0.998	90998	0.765	0.122564	-0.0833	0.3333	0	18	0.7111	-1	1	0	1	[illegible]	[illegible]
HIP_90999	CGERGFFYT	VSTHQEAE	CGERGFFYTVSTHQEAE		SCG1	24666/367830	0.981	90999	0.765	-0.070149	0.5833	0.1667	-1	17	-0.7	-2	3	1	0	[illegible]	[illegible]
HIP_91000	LQVGQVELG	LIVLSVALN	LQVGQVELGLIVLSVALN		IAPP	2437/45018	0.986	91000	0.765	0.079989	2.0667	0.1667	-1	18	1.4722	-1	0	0	0	[illegible]	[illegible]
[illegible]	[illegible]	[illegible]	[illegible]	[illegible]	[illegible]	[illegible]	[illegible]	[illegible]	[illegible]	[illegible]	[illegible]	[illegible]	[illegible]	[illegible]	[illegible]	[illegible]	[illegible]	[illegible]	[illegible]	[illegible]	[illegible]

HIP_91459	SHLVEALYL	QLAKELTA	SHLVEALYLQLAKELTA		CMGA	26823/247050	0.939	91459	0.765	0.084599	1.4	0	0	17	0.4824	-1	1	1	0
HIP_91460	HLCGSHLVE	EEYRAPRP	HLCGSHLVEEEYRAPRP		SCG1	48973/367830	0.860	91460	0.765	0.089742	-0.6333	0	1	17	-0.9118	-1	1	1	1
HIP_91461	VCGERGFF	NTHPPASLP	VCGERGFFNTHPPASLP		CMGA	28087/247050	0.930	91461	0.765	0.058837	-0.3667	0.1667	1	17	-0.2294	0	2	1	0
HIP_91462	LVEALYLVC	DSKEAEKS	LVEALYLVCDSKEAEKS		CMGA	24719/247050	0.952	91462	0.765	0.215688	0.3833	0.1667	-2	17	0.0706	-2	1	1	0
HIP_91463	SLYQLENYC	GSNTYGKRN	SLYQLENYCGSNTYGKRN		IAPP	3709/45018	0.964	91463	0.765	0.134092	-1.1667	0.3333	1	18	-1.2667	1	1	0	0
HIP_91464	VEALYLVCG	AKELTAEKR	VEALYLVCGAKELTAEKR		CMGA	26436/247050	0.941	91464	0.765	0.003151	0.1167	0.1667	0	18	0.1389	0	1	0	0
HIP_91465	ICSLYQLEN	GFRGPGPQL	ICSLYQLENGFRGPGPQL		CMGA	24273/247050	0.954	91465	0.765	-0.041104	-0.8833	0.1667	0	18	-0.2111	0	2	0	0
HIP_91466	CGERGFFYT	PRLPYGAG	CGERGFFYTPRLPYGAG		SCG2	40030/334890	0.977	91466	0.765	0.104165	-0.25	0.1667	1	17	-0.4059	1	3	1	0
HIP_91467	EDLQVGQVE	EGNNQAPGE	EDLQVGQVEEGNNQAPGE		CMGA	17985/247050	0.979	91467	0.765	-0.099642	-1.7	0.1667	-5	18	-1.3222	-5	0	0	0
HIP_91468	EALYLVCGE	PPEDLIEML	EALYLVCGEPPEDLIEML		SCG2	18333/334890	0.997	91468	0.765	-0.130884	-1.35	0.5	-5	18	0.4278	-5	0	0	0
HIP_91469	NQHLCGSHL	EVLKREPLN	NQHLCGSHLEVLKREPLN		IAPP	5748/45018	0.899	91469	0.765	0.023755	0.7167	0.1667	2	18	-0.7333	0	0	0	0
HIP_91470	GERGFFYT	STNVGSNTY	GERGFFYTSTNVGSNTY		IAPP	4616/45018	0.940	91470	0.765	0.146062	-0.7	0.1667	0	17	-0.7471	0	3	1	0
HIP_91471	GSHLVEALY	QELGEYLA	GSHLVEALYQELGEYLA		SCG2	27167/334890	0.992	91471	0.765	0.086930	0.1833	0	-2	17	0.0941	-3	1	1	0
HIP_91472	EQCCTSICS	ELQDLALQG	EQCCTSICSELQDLALQG		CMGA	17726/247050	0.980	91472	0.765	-0.050279	0.5	0.1667	-3	18	0.0833	-3	0	0	0
HIP_91473	EAEDLQVG	EEEEEEEEE	EAEDLQVGEEEEEEEEE		CMGA	15768/247050	0.985	91473	0.765	-0.099535	-1.7	0.1667	-12	17	-2.1235	-12	0	1	7
HIP_91474	ELGGGPGA	FPNPYNQEK	ELGGGPGAFPNPYNQEK		SCG2	17337/334890	0.997	91474	0.765	-0.079791	-0.4167	0.5	-1	17	-1.2176	-1	2	1	1
HIP_91475	GAGSLQPL	WGYEKRNLA	GAGSLQPLWGYEKRNLA		SCG1	27080/367830	0.975	91475	0.765	-0.095408	-0.65	0.3333	1	17	-0.5706	1	2	1	0
HIP_91476	GERGFFYTP	TREEKRFL	GERGFFYTPTREEKRFL		SCG1	49778/367830	0.853	91476	0.765	0.031938	-2.05	0.1667	1	17	-1.2235	1	3	1	0
HIP_91477	VELGGGPGA	ESKAEGNNQ	VELGGGPGAESKAEGNNQ		CMGA	17411/247050	0.981	91477	0.765	-0.110873	-1.4	0.5	-2	18	-0.9833	-2	0	0	1
HIP_91478	NQHLCGS	AILSSTN	NQHLCGSAILSSTN		IAPP	2554/45018	0.984	91478	0.765	0.171958	1.9	0.3333	1	14	-0.0571	0	0	0	0
HIP_91479	SICSLYQL	EEPSSKDVM	SICSLYQLEEPSSKDVM		CMGA	20881/247050	0.969	91479	0.765	0.063582	-1.6	0.1667	-2	17	-0.1941	-2	1	1	0
HIP_91480	HLCGSHLVE	DISEADLDH	HLCGSHLVEDISEADLDH		SCG2	25300/334890	0.994	91480	0.765	-0.010373	0.7833	0.1667	-2	18	-0.2611	-5	0	0	0
HIP_91481	VEQCCTSIC	KRERMDEEQ	VEQCCTSICKRERMDEEQ		SCG2	44872/334890	0.963	91481	0.765	0.094535	-0.95	0.1667	-2	18	-1.1556	-2	0	0	0
HIP_91482	CGERGFFYT	INDEMKRS	CGERGFFYTINDEMKRS		SCG2	38567/334890	0.980	91482	0.765	0.043663	-0.2833	0	0	17	-0.9412	0	3	1	0
HIP_91483	YLVCGERGF	PVNSPMNKG	YLVCGERGFPVNSPMNKG		CMGA	20349/247050	0.971	91483	0.765	-0.033281	-0.5	0.3333	1	18	-0.3333	1	1	0	0
HIP_91484	CCTSICSLY	IEAELEKVA	CCTSICSLYIEAELEKVA		CMGA	18790/247050	0.976	91484	0.765	-0.104370	0.75	0.1667	-2	18	0.7722	-2	1	0	0
HIP_91485	SHLVEALY	DEDDIYKAN	SHLVEALYDEDDIYKAN		SCG2	29730/334890	0.990	91485	0.765	0.129263	-1.0333	0	-3	17	-0.6824	-4	1	1	0
HIP_91486	EQCCTSICS	QKIAEKFSQ	EQCCTSICSQKIAEKFSQ		SCG1	47447/367830	0.871	91486	0.765	0.033616	0.55	0.1667	0	18	-0.4056	0	0	0	0
HIP_91487	CGSHLVEAL	SLELDKMAH	CGSHLVEALSLELDKMAH		SCG1	34308/367830	0.948	91487	0.765	-0.017046	0.2667	0.1667	0	18	0.2556	-2	0	0	0
HIP_91488	LYQLENYCN	VIAYLKRL	LYQLENYCNVIAYLKRL		SCG2	29639/334890	0.990	91488	0.765	-0.037393	1.3667	0	1	17	0.1118	1	2	1	0
HIP_91489	AGSLQPLAL	QVRGYPEE	AGSLQPLALQVRGYPEE		CMGA	29544/247050	0.918	91489	0.765	0.182873	0.9333	0	-1	17	-0.3176	-1	0	1	0
HIP_91490	HLCGSHLV	EEQYTPQSL	HLCGSHLVEEQYTPQSL		SCG2	33333/334890	0.987	91490	0.765	0.171419	-0.95	0	0	17	-0.4647	-2	1	1	0
HIP_91491	CGERGFFY	DDEDDIYK	CGERGFFYDDEDDIYK		SCG2	30877/334890	0.989	91491	0.765	0.128472	-1.0333	0	-4	16	-1.2625	-4	3	0	0
HIP_91492	LYQLENYCN	LIPLIFLIS	LYQLENYCNLIPLIFLIS		SCG2	20262/334890	0.996	91492	0.765	-0.146239	0.7333	0.1667	-1	18	1.0444	-1	1	0	0
HIP_91493	LGGGPGAGS	YGEESEEER	LGGGPGAGSYGEESEEER		SCG1	18653/367830	0.991	91493	0.765	-0.101044	-0.7667	0.5	-4	18	-1.2944	-4	1	0	2
HIP_91494	GAGSLQPLA	ETHAAGHSQ	GAGSLQPLAETHAAGHSQ		SCG1	35369/367830	0.943	91494	0.765	-0.070131	-0.5667	0.1667	1	18	-0.4	-1	0	0	0
HIP_91495	GERGFFYT	RDSKENIEK	GERGFFYTRDSKENIEK		SCG2	41750/334890	0.973	91495	0.765	0.157262	-1.3333	0.1667	0	17	-1.6353	0	3	1	0
HIP_91496	SICSLYQL	IFLISGAEA	SICSLYQLIFLISGAEA		SCG2	26143/334890	0.993	91496	0.765	-0.046773	1.6833	0	-1	17	1.3353	-1	2	1	0
HIP_91497	QVGQVELGG	LIFLISGAE	QVGQVELGGLIFLISGAE		SCG2	14933/334890	0.998	91497	0.765	0.083775	2.35	0.3333	-2	18	0.9444	-2	1	0	0
HIP_91498	SICSLYQLE	LPVNSPMNK	SICSLYQLELPVNSPMNK		CMGA	17283/247050	0.981	91498	0.765	-0.108606	0.5333	0.1667	0	18	-0.0167	0	1	0	0
HIP_91499	GSLQPLALE	SGRLQNGQN	GSLQPLALESGRLQNGQN		SCG2	33382/334890	0.987	91499	0.765	0.165219	-0.6	0.3333	0	18	-0.7167	0	0	0	0
HIP_91500	GERGFFYT	ENIEKNEQI	GERGFFYTENIEKNEQI		SCG2	34358/334890	0.986	91500	0.765	0.018071	-0.2833	0	-2	17	-1.2412	-2	3	1	0
HIP_91501	QCCTSICSL	TENVPRTRL	QCCTSICSLTENVPRTRL		NPY	5092/49410	0.930	91501	0.765	0.126937	-0.3667	0.1667	1	18	-0.0556	1	0	0	0

HIP_91970	ERGFFYT	PGPQLRR	ERGFFYTPGPQLRR		CMGA	20611/247050	0.970	91970	0.764	0.176390	-0.4667	0.5	2	14	-1.2214	2	3	0	0
HIP_91971	SLYQLENYC	LNLLGMESA	SLYQLENYCLNLLGMESA		SCG2	21539/334890	0.995	91971	0.764	-0.168942	0.3	0	-2	18	0.1722	-2	1	0	0
HIP_91972	SLQPLALEG	PGAEEAQDP	SLQPLALEGPGAEEAQDP		CMGA	5483/247050	0.998	91972	0.764	-0.189140	-0.05	0.5	-4	18	-0.5889	-4	0	0	0
HIP_91973	CGERGFFY	ISEADLDH	CGERGFFYISEADLDH		SCG2	24425/334890	0.994	91973	0.764	0.085581	0.75	0.1667	-2	16	-0.4	-3	3	0	0
HIP_91974	GGPGAGSLQ	DDIYKANNI	GGPGAGSLQDDIYKANNI		SCG2	34176/334890	0.986	91974	0.764	-0.110473	-0.5	0.1667	-1	18	-0.5722	-1	1	0	0
HIP_91975	SICSLYQLE	SKPSPMPVS	SICSLYQLESKPSPMPVS		CMGA	28019/247050	0.931	91975	0.764	-0.007289	-1.5833	0.3333	0	18	-0.0167	0	1	0	0
HIP_91976	QPLALEGSL	ENELTLNEK	QPLALEGSLENELTLNEK		SCG1	27059/367830	0.975	91976	0.764	-0.122557	-1.3167	0.3333	-3	18	-0.6167	-3	0	0	0
HIP_91977	HLVEALYLV	YRKKSAEF	HLVEALYLVYRKKSAEF		SCG1	52594/367830	0.830	91977	0.764	0.154382	-0.5	0	2	17	0.0176	1	2	1	0
HIP_91978	VEALYLVC	CNTATCATQ	VEALYLVCCNTATCATQ		IAPP	4379/45018	0.947	91978	0.764	0.185107	1.4667	0	-1	17	0.8824	-1	1	1	0
HIP_91979	LQVGQVELG	EYLARMLVK	LQVGQVELGEYLARMLVK		SCG2	24898/334890	0.994	91979	0.764	0.002200	-0.1833	0.1667	0	18	0.3889	0	1	0	0
HIP_91980	QCCTSICSL	LLGMESAAN	QCCTSICSLLLGMESAAN		SCG2	19487/334890	0.996	91980	0.764	-0.045147	2.1167	0.3333	-1	18	0.8278	-1	0	0	1
HIP_91981	LYQLENYC	GDERILSIL	LYQLENYCGDERILSIL		CMGA	18891/247050	0.976	91981	0.764	0.088947	-1.6167	0.1667	-2	17	0.0529	-2	1	1	0
HIP_91982	VGQVELGGG	GEQEHSQQK	VGQVELGGGGEQEHSQQK		CMGA	14347/247050	0.988	91982	0.764	-0.065045	-1.4333	0.6667	-1	18	-1.2333	-2	0	0	2
HIP_91983	LQPLALEGS	EGEEENELT	LQPLALEGSEGEEENELT		SCG1	24467/367830	0.981	91983	0.764	-0.141306	-2.0167	0.5	-6	18	-0.8278	-6	0	0	1
HIP_91984	LYQLENYCN	FQNRMLSK	LYQLENYCNFQNRMLSK		SCG2	42154/334890	0.972	91984	0.764	0.004293	-1.0833	0	1	17	-0.8353	1	2	1	0
HIP_91985	VNQHLCGSH	QKENGDESH	VNQHLCGSHQKENGDESH		SCG2	41640/334890	0.973	91985	0.764	-0.095803	-2.55	0.3333	1	18	-1.6611	-2	0	0	0
HIP_91986	VEALYLVCG	SPMPVSQEC	VEALYLVCGSPMPVSQEC		CMGA	10036/247050	0.995	91986	0.764	-0.026668	0.9667	0.5	-2	18	0.6611	-2	1	0	0
HIP_91987	ICSLYQLEN	GAALSLIPL	ICSLYQLENGAALSLIPL		SCG2	18870/334890	0.996	91987	0.764	-0.121647	0	0.1667	-1	18	1.0389	-1	1	0	0
HIP_91988	DLQVGQVEL	KSAEFPDFY	DLQVGQVELKSAEFPDFY		SCG1	42501/367830	0.904	91988	0.764	0.069121	0.2667	0.1667	-3	18	-0.3111	-3	0	0	0
HIP_91989	GGGPGAGSL	GDSEGLSQG	GGGPGAGSLGDSEGLSQG		CMGA	11277/247050	0.993	91989	0.764	-0.037160	-0.35	0.6667	-2	18	-0.4611	-2	0	0	1
HIP_91990	EQCCTSICS	LNEKNFFPE	EQCCTSICSLNEKNFFPE		SCG1	25217/367830	0.980	91990	0.764	-0.074395	0.5	0.1667	-2	18	-0.4111	-2	0	0	0
HIP_91991	GSLQPLAL	ELDKMAH	GSLQPLALELDKMAH		SCG1	35870/367830	0.941	91991	0.764	0.132744	1.0333	0	0	15	0.02	-1	0	1	0
HIP_91992	HLVEALYL	LDSQSIYQ	HLVEALYLLDSQSIYQ		SCG2	26503/334890	0.993	91992	0.764	0.172548	0.9667	0.1667	-1	16	0.2687	-2	1	0	0
HIP_91993	ALYLVCGER	DQTVLTEDE	ALYLVCGERDQTVLTEDE		SCG1	35198/367830	0.944	91993	0.764	-0.145119	-2.6833	0.1667	-4	18	-0.25	-4	0	0	0
HIP_91994	GERGFFYTP	LKHMQFPPM	GERGFFYTPLKHMQFPPM		SCG2	35393/334890	0.984	91994	0.764	-0.057976	-1.15	0.1667	2	18	-0.5667	1	2	0	0
HIP_91995	NQHLCGSHL	DAPAEDMAR	NQHLCGSHLDAPAEDMAR		NPY	3737/49410	0.963	91995	0.764	-0.074679	-0.5833	0.3333	0	18	-0.7667	-2	0	0	0
HIP_91996	PLALEGSLQ	DLEWERYRG	PLALEGSLQDLEWERYRG		SCG1	36615/367830	0.937	91996	0.764	-0.099845	-0.6167	0.1667	-2	18	-0.8278	-2	1	0	0
HIP_91997	QPLALEGSL	ALEYIENLR	QPLALEGSLALEYIENLR		SCG2	10676/334890	0.999	91997	0.764	0.006425	0.7833	0.3333	-2	18	0.0556	-2	1	0	0
HIP_91998	PLALEGSLQ	SSHHTAEKR	PLALEGSLQSSHHTAEKR		SCG1	44722/367830	0.890	91998	0.764	-0.064562	-0.8833	0.5	2	18	-0.8556	0	0	0	0
HIP_91999	CTSICSLYQ	PYALNSEK	CTSICSLYQPYALNSEK		SCG2	29226/334890	0.991	91999	0.764	-0.111674	-0.35	0.1667	0	17	-0.1647	0	2	1	0
HIP_92000	EAEDLQVGQ	FYDSEEPVS	EAEDLQVGQFYDSEEPVS		SCG1	13525/367830	0.996	92000	0.764	-0.112149	-0.2833	0.1667	-6	18	-0.8944	-6	2	0	0
HIP_92001	HLVEALYL	GGEDWNPV	HLVEALYLGGEDWNPV		SCG2	24246/334890	0.994	92001	0.764	0.213016	0.3333	0.3333	-2	16	-0.0125	-3	1	0	0
HIP_92002	EDLQVGQVE	DEEQKLYTD	EDLQVGQVEDEEQKLYTD		SCG2	33318/334890	0.987	92002	0.764	-0.099098	-2.2167	0	-6	18	-1.4056	-6	0	0	0
HIP_92003	GERGFFYT	MSDTREEK	GERGFFYTMSDTREEK		SCG1	37352/367830	0.933	92003	0.764	0.059614	-0.2667	0.1667	-1	16	-1.4812	-1	3	0	0
HIP_92004	RGFFYTPKT	KVLPRLPYG	RGFFYTPKTKVLPRLPYG		SCG2	36026/334890	0.984	92004	0.764	0.138161	-0.35	0.1667	4	18	-0.5	4	1	0	0
HIP_92005	SLYQLENYC	GQVTALPVN	SLYQLENYCGQVTALPVN		CMGA	12122/247050	0.992	92005	0.764	-0.052762	-0.3333	0.1667	-1	18	0.0278	-1	1	0	0
HIP_92006	DLQVGQVEL	FEVRLLRDP	DLQVGQVELFEVRLLRDP		SCG1	37190/367830	0.934	92006	0.764	-0.018400	1.3333	0	-2	18	-0.0778	-2	1	0	0
HIP_92007	EDLQVGQVE	RVPKLDLKR	EDLQVGQVERVPKLDLKR		SCG1	42168/367830	0.906	92007	0.764	-0.130531	-0.7833	0.1667	0	18	-0.8778	0	0	0	0
HIP_92008	GERGFFY	ALNHLKA	GERGFFYALNHLKA		IAPP	4898/45018	0.931	92008	0.764	0.065370	1.0667	0	2	14	-0.2786	1	3	0	0
HIP_92009	CGSHLVEAL	QEHSQQKEE	CGSHLVEALQEHSQQKEE		CMGA	32943/247050	0.885	92009	0.764	-0.001426	-1.35	0	-1	18	-1.15	-3	0	0	0
HIP_92010	ICSLYQLEN	KRKCNTATC	ICSLYQLENKRKCNTATC		IAPP	7513/45018	0.800	92010	0.764	0.083586	-2.5833	0	2	18	-0.4667	2	1	0	0
HIP_92011	QCCTSICSL	HQEAENEKD	QCCTSICSLHQEAENEKD		SCG1	35764/367830	0.941	92011	0.764	-0.017199	-0.7833	0.1667	-2	18	-0.9056	-3	0	0	0
HIP_92012	RGFFYTPKT	PFSEDVNW	RGFFYTPKTPFSEDVNW		SCG1	32580/367830	0.955	92012	0.764	-0.076045	-0.9667	0.5	0	17	-0.8412	0	2	1	0
HIP_92013	VELGGGPGA	PSSREDSLE	VELGGGPGAPSSREDSLE		CMGA	10224/247050	0.995	92013	0.764	-0.088533	-0.5667	0.8333	-3	18	-0.6722	-3	0	0	1
HIP_92014	VEALYLVC	VKYPEIINS	VEALYLVCVKYPEIINS		SCG2	41101/334890	0.975	92014	0.764	0.106057	1.5833	0	-1	17	0.8294	-1	2	1	0
HIP_92015	CCTSICSLY	KTHSREKSS	CCTSICSLYKTHSREKSS		SCG1	56774/367830	0.792	92015	0.764	0.036191	-1.0167	0.1667	3	18	-0.55	2	1	0	0
HIP_92016	ALYLVCGER	QEEEQIEQA	ALYLVCGERQEEEQIEQA		SCG2	34270/334890	0.986	92016	0.764	-0.114648	-3.15	0.1667	-4	18	-0.6556	-4	0	0	1
HIP_92017	LVCGERGFF	EKIESQTQ	LVCGERGFFEKIESQTQ		SCG2	37189/334890	0.982	92017	0.764	0.048594	0.3833	0.1667	-1	17	-0.4471	-1	2	1	0

HIP_92481	QCCTSICS	GESRSEALA	QCCTSICSGESRSEALA		CMGA	17198/247050	0.981	92481	0.764	0.178488	0.25	0.5	-1	17	0.0059	-1	0	1	0	[illegible]
HIP_92482	HLVEALYL	KEWEDSKR	HLVEALYLKEWEDSKR		CMGA	31505/247050	0.900	92482	0.764	0.173248	-0.3333	0	0	16	-0.9438	-1	2	0	0	[illegible]
HIP_92483	HLCGSHLV	EGNNQAPGE	HLCGSHLVEGNNQAPGE		CMGA	19880/247050	0.972	92483	0.764	0.207241	-0.4333	0.1667	0	17	-0.6706	-2	0	1	0	[illegible]
HIP_92484	LVEALYLV	ADEPQWSL	LVEALYLVADEPQWSL		SCG1	29907/367830	0.966	92484	0.764	0.084561	0.25	0	-3	16	0.5375	-3	1	0	0	[illegible]
HIP_92485	EAEDLQVGQ	LLMRESTEN	EAEDLQVGQLLMRESTEN		NPY	1946/49410	0.987	92485	0.764	0.065819	1.6333	0.1667	-4	18	-0.8389	-4	0	0	0	[illegible]
HIP_92486	CTSICSLYQ	PTLLLSLLG	CTSICSLYQPTLLLSLLG		SCG1	25058/367830	0.980	92486	0.764	-0.064032	0.0833	0.1667	0	18	1.2056	0	1	0	1	[illegible]
HIP_92487	LVEALYLVC	RGDERILS	LVEALYLVCRGDERILS		CMGA	23803/247050	0.957	92487	0.764	0.222907	0.35	0.1667	-1	17	0.6118	-1	1	1	0	[illegible]
HIP_92488	GAGSLQPLA	ADEPQWSLY	GAGSLQPLAADEPQWSLY		SCG1	27893/367830	0.972	92488	0.764	-0.080237	-0.2	0.1667	-2	18	-0.2778	-2	0	0	0	[illegible]
HIP_92489	ALYLVCGER	GYPKTPGRA	ALYLVCGERGYPKTPGRA		SCG2	32181/334890	0.988	92489	0.764	-0.077773	-1.95	0.5	2	18	-0.3444	2	1	0	0	[illegible]
HIP_92490	GSHLVEALY	AYEDVVGG	GSHLVEALYAYEDVVGG		SCG2	32083/334890	0.988	92490	0.764	0.007028	0.2167	0	-2	17	0.3235	-3	2	1	0	[illegible]
HIP_92491	AEDLQVGQV	QEEVRDSKE	AEDLQVGQVQEEVRDSKE		SCG2	26097/334890	0.993	92491	0.764	-0.110870	-1.7	0.1667	-4	18	-1.2722	-4	0	0	0	[illegible]
HIP_92492	VELGGGPGA	PNNQKRER	VELGGGPGAPNNQKRER		SCG2	29661/334890	0.990	92492	0.764	-0.028981	-1.4667	0.5	1	17	-1.4941	1	0	1	1	[illegible]
HIP_92493	TSICSLYQL	TKADTEKWA	TSICSLYQLTKADTEKWA		SCG1	51212/367830	0.842	92493	0.764	0.092978	-0.6333	0	0	18	-0.3333	0	1	0	0	[illegible]
HIP_92494	LVCGERGF	DTEKWAEGG	LVCGERGFDTEKWAEGG		SCG1	36577/367830	0.937	92494	0.764	-0.020837	-1.6333	0.1667	-2	17	-0.6176	-2	2	1	0	[illegible]
HIP_92495	TSICSLYQ	YSALRHYIN	TSICSLYQYSALRHYIN		NPY	6272/49410	0.885	92495	0.764	0.025048	-0.2167	0.1667	2	17	-0.0471	1	2	1	0	[illegible]
HIP_92496	VEALYLVC	PQSAPKEN	VEALYLVCPQSAPKEN		SCG2	28253/334890	0.992	92496	0.764	0.203734	0.7667	0.3333	-1	16	-0.0687	-1	1	0	0	[illegible]
HIP_92497	VCGERGFFY	PPASLPS	VCGERGFFYPPASLPS		CMGA	21280/247050	0.967	92497	0.764	0.160976	0.4833	0.3333	0	16	0.0875	0	3	2	0	[illegible]
HIP_92498	LVCGERGF	PQWSLYPSD	LVCGERGFPQWSLYPSD		SCG1	26945/367830	0.975	92498	0.764	-0.111780	-1.35	0.3333	-1	17	-0.3353	-1	2	1	0	[illegible]
HIP_92499	QCCTSICSL	QSLATLESV	QCCTSICSLQSLATLESV		SCG2	24966/334890	0.994	92499	0.764	0.027378	0.8333	0.3333	-1	18	0.7944	-1	0	0	0	[illegible]
HIP_92500	EALYLVCG	GPGPQLRR	EALYLVCGGPGPQLRR		CMGA	15135/247050	0.986	92500	0.764	0.154912	0.65	0.6667	1	16	-0.1125	1	1	0	0	[illegible]
HIP_92501	RGFFYTPKT	KIAEKFSQR	RGFFYTPKTKIAEKFSQR		SCG1	41665/367830	0.908	92501	0.764	0.145749	-0.6333	0.1667	4	18	-1.0278	4	1	0	0	[illegible]
HIP_92502	LVEALYLV	MQPTLLLSL	LVEALYLVMQPTLLLSL		SCG1	26891/367830	0.975	92502	0.764	0.110125	0.5833	0.1667	-1	17	1.6059	-1	1	1	1	[illegible]
HIP_92503	LCGSHLVEA	KPGAEEAQD	LCGSHLVEAKPGAEEAQD		CMGA	20495/247050	0.970	92503	0.764	0.161476	-0.5667	0.3333	-2	18	-0.45	-3	0	0	0	[illegible]
HIP_92504	PLALEGSL	FNPYYDPLQ	PLALEGSLFNPYYDPLQ		SCG1	22802/367830	0.985	92504	0.764	-0.051955	0.05	0.5	-2	17	-0.1647	-2	3	1	0	[illegible]
HIP_92505	SHLVEALY	ARMLVKYPE	SHLVEALYARMLVKYPE		SCG2	43189/334890	0.969	92505	0.764	0.087790	0.5833	0	1	17	0.1	0	1	1	0	[illegible]
HIP_92506	EQCCTSICS	TVVLNPHPS	EQCCTSICSTVVLNPHPS		CMGA	20032/247050	0.972	92506	0.764	-0.025448	2.3167	0.1667	0	18	0.1944	-1	0	0	0	[illegible]
HIP_92507	VCGERGFF	ATPIESHQV	VCGERGFFATPIESHQV		IAPP	3249/45018	0.973	92507	0.764	0.015182	0.7833	0.3333	0	17	0.0412	-1	2	1	0	[illegible]
HIP_92508	GSLQPLALE	CGERGFFY	GSLQPLALECGERGFFY		INSB	388/4968	0.923	92508	0.764	0.050791	0.1167	0.1667	-1	17	0.0824	-1	0	1	0	[illegible]
HIP_92509	GGGPGAGSL	WKSSHFERR	GGGPGAGSLWKSSHFERR		SCG1	44223/367830	0.893	92509	0.764	0.045165	-0.5	0.5	3	18	-1.0056	2	1	0	1	[illegible]
HIP_92510	HLVEALYL	EAQLDHPQL	HLVEALYLEAQLDHPQL		SCG2	26932/334890	0.992	92510	0.764	0.071756	0.1833	0	-3	17	0	-5	1	1	0	[illegible]
HIP_92511	GSLQPLAL	PMDMSDDY	GSLQPLALPMDMSDDY		SCG2	21145/334890	0.996	92511	0.764	0.128974	1.0333	0.1667	-3	16	-0.2188	-3	0	0	0	[illegible]
HIP_92512	VELGGGPGA	HGRSRPQRS	VELGGGPGAHGRSRPQRS		SCG1	39171/367830	0.923	92512	0.764	-0.019268	-1.3833	0.5	2	18	-1.15	1	0	0	1	[illegible]
HIP_92513	AGSLQPLA	NGQNGERAT	AGSLQPLANGQNGERAT		SCG2	24673/334890	0.994	92513	0.764	0.165123	-0.5667	0.3333	0	17	-0.7824	0	0	1	0	[illegible]
HIP_92514	SLQPLAL	QMDKARRH	SLQPLALQMDKARRH		SCG1	42505/367830	0.904	92514	0.764	0.165846	0.7167	0	3	15	-0.8067	2	0	1	0	[illegible]
HIP_92515	LVEALYLVC	APGKWQQQ	LVEALYLVCAPGKWQQQ		SCG1	26493/367830	0.976	92515	0.764	0.169422	1.7167	0.3333	0	17	0.2235	0	1	1	1	[illegible]
HIP_92516	SHLVEALY	RSQEESEEG	SHLVEALYRSQEESEEG		SCG1	38566/367830	0.926	92516	0.764	0.221117	-0.75	0.1667	-3	17	-1.1294	-4	1	1	0	[illegible]
HIP_92517	RGFFYTPKT	PLQQKENGD	RGFFYTPKTPLQQKENGD		SCG2	34180/334890	0.986	92517	0.764	-0.028048	-1.25	0.3333	1	18	-1.5056	1	1	0	0	[illegible]
HIP_92518	LCGSHLVEA	VNSPMNKGD	LCGSHLVEAVNSPMNKGD		CMGA	22562/247050	0.962	92518	0.764	0.049853	0.4	0.1667	0	18	-0.1611	-1	0	0	0	[illegible]
HIP_92519	LYQLENYCN	GFEDELSEV	LYQLENYCNGFEDELSEV		CMGA	11253/247050	0.993	92519	0.764	-0.093006	-0.5667	0.1667	-5	18	-0.6056	-5	2	0	0	[illegible]
HIP_92520	VEALYLVCG	LPVDLDDIS	VEALYLVCGLPVDLDDIS		SCG2	11722/334890	0.998	92520	0.764	-0.106129	2.1167	0.3333	-4	18	1.0278	-4	1	0	0	[illegible]
HIP_92521	CCTSICSLY	VLENQSSQA	CCTSICSLYVLENQSSQA		CMGA	20799/247050	0.969	92521	0.764	-0.081281	1.0333	0.1667	-1	18	0.3556	-1	1	0	0	[illegible]
HIP_92522	YLVCGERGF	ESEEGEEDA	YLVCGERGFESEEGEEDA		SCG1	27237/367830	0.974	92522	0.764	-0.061755	-1.65	0.3333	-6	18	-0.9556	-6	1	0	0	[illegible]
HIP_92523	VEALYLVCG	GDERILSI	VEALYLVCGGDERILSI		CMGA	17734/247050	0.980	92523	0.764	0.086817	-0.1833	0.3333	-2	17	0.8941	-2	1	1	0	[illegible]
HIP_92524	LQPLALEGS	DSEGLSQGL	LQPLALEGSDSEGLSQGL		CMGA	20432/247050	0.970	92524	0.764	-0.126703	-2.0833	0.5	-3	18	-0.1056	-3	0	0	0	[illegible]
HIP_92525	PLALEGSLQ	LSLLVCLGA	PLALEGSLQLSLLVCLGA		NPY	3223/49410	0.972	92525	0.764	-0.052429	1.05	0.3333	-1	18	1.4389	-1	0	0	0	[illegible]
HIP_92526	VEALYLVCG	ISGAEAASF	VEALYLVCGISGAEAASF		SCG2	13953/334890	0.998	92526	0.764	-0.039593	1.6	0.5	-2	18	1.2389	-2	1	0	0	[illegible]
HIP_92527	ERGFFYTPK	LYTDDEDDI	ERGFFYTPKLYTDDEDDI		SCG2	22180/334890	0.995	92527	0.764	-0.047572	-0.7333	0.1667	-4	18	-1.1944	-4	3	0	0	[illegible]
HIP_92528	VNQHLCGSH	LANFLVHSS	VNQHLCGSHLANFLVHSS		IAPP	2879/45018	0.980	92528	0.764	-0.010016	-0.3833	0.3333	1	18	0.2222	0	1	0	0	[illegible]
[illegible]	[illegible]	[illegible]	[illegible]	[illegible]	[illegible]	[illegible]	[illegible]	[illegible]	[illegible]	[illegible]	[illegible]	[illegible]	[illegible]	[illegible]	[illegible]	[illegible]	[illegible]	[illegible]	[illegible]	[illegible]

HIP_92992	EALYLVCGE	SRPDRSSQG	EALYLVCGESRPDRSSQG		SCG1	37479/367830	0.932	92992	0.763	-0.056214	-1.3833	0.5	-1	18	-0.7222	-1	0	0	0	[illegible]
HIP_92993	LVEALYL	TRQRYGK	LVEALYLTRQRYGK		NPY	6678/49410	0.863	92993	0.763	0.266821	-0.4	0	2	14	-0.4429	2	1	0	0	[illegible]
HIP_92994	DLQVGQVEL	NEGMVTRCI	DLQVGQVELNEGMVTRCI		SCG1	29923/367830	0.966	92994	0.763	0.195443	-0.4833	0.1667	-2	18	0.1167	-2	0	0	0	[illegible]
HIP_92995	HLVEALYL	YWDEDLLM	HLVEALYLYWDEDLLM		SCG2	34375/334890	0.986	92995	0.763	0.068154	0.1	0	-3	16	0.3875	-4	3	0	0	[illegible]
HIP_92996	VGQVELGGG	IAYEDVVGG	VGQVELGGGIAYEDVVGG		SCG2	11185/334890	0.999	92996	0.763	0.116175	0.6333	0.5	-3	18	0.5111	-3	1	0	1	[illegible]
HIP_92997	LVCGERGFF	RKQASAIK	LVCGERGFFRKQASAIK		SCG1	53501/367830	0.822	92997	0.763	0.122559	-1.1167	0.1667	3	17	-0.0706	3	2	1	0	[illegible]
HIP_92998	HLVEALYLV	YTDDEDDI	HLVEALYLVYTDDEDDI		SCG2	28321/334890	0.991	92998	0.763	0.080111	0.2	0	-5	17	-0.0824	-6	2	1	0	[illegible]
HIP_92999	VELGGGPGA	SRDNPFKRT	VELGGGPGASRDNPFKRT		SCG2	35999/334890	0.984	92999	0.763	-0.002189	-1.5	0.5	1	18	-0.95	1	0	0	1	[illegible]
HIP_93000	FVNQHLCGS	LGEGHHRVQ	FVNQHLCGSLGEGHHRVQ		SCG1	28642/367830	0.970	93000	0.763	-0.072125	0.2	0.5	3	18	-0.4889	0	0	0	0	[illegible]
HIP_93001	AEDLQVGQV	DEEEEEGEN	AEDLQVGQVDEEEEEGEN		SCG1	25114/367830	0.980	93001	0.763	-0.082429	-1.7	0.1667	-9	18	-1.6	-9	0	0	3	[illegible]
HIP_93002	QPLALEGSL	NSPMNKGDT	QPLALEGSLNSPMNKGDT		CMGA	13934/247050	0.989	93002	0.763	-0.106122	-0.55	0.6667	-1	18	-0.7	-1	0	0	0	[illegible]
HIP_93003	PGAGSLQP	WERYRGRGS	PGAGSLQPWERYRGRGS		SCG1	53448/367830	0.823	93003	0.763	0.068750	-1.7	0.1667	2	17	-1.3824	2	2	1	0	[illegible]
HIP_93004	PGAGSLQPL	CIVEVISDT	PGAGSLQPLCIVEVISDT		CMGA	11863/247050	0.992	93004	0.763	0.106486	1.65	0.1667	-2	18	0.6944	-2	0	0	0	[illegible]
HIP_93005	HLVEALYL	TNEIVEEQ	HLVEALYLTNEIVEEQ		SCG2	27668/334890	0.992	93005	0.763	0.178925	-0.2333	0	-3	16	-0.0063	-4	1	0	0	[illegible]
HIP_93006	CGERGFFY	KFEVRLLR	CGERGFFYKFEVRLLR		SCG1	46760/367830	0.876	93006	0.763	0.135855	-0.05	0	2	16	-0.2375	2	4	0	0	[illegible]
HIP_93007	SLQPLALEG	MYEENSRDN	SLQPLALEGMYEENSRDN		SCG2	19733/334890	0.996	93007	0.763	-0.074024	-0.5	0.1667	-3	18	-1.0444	-3	1	0	0	[illegible]
HIP_93008	LQPLALEGS	RPDRSSQGG	LQPLALEGSRPDRSSQGG		SCG1	42549/367830	0.904	93008	0.763	-0.063143	-2.3833	0.5	0	18	-0.9222	0	0	0	0	[illegible]
HIP_93009	LCGSHLVEA	AEEAQDPEG	LCGSHLVEAAEEAQDPEG		CMGA	23071/247050	0.960	93009	0.763	-0.033209	-0.45	0	-4	18	-0.4278	-5	0	0	0	[illegible]
HIP_93010	LVEALYLV	KLDLKRQYD	LVEALYLVKLDLKRQYD		SCG1	41363/367830	0.910	93010	0.763	0.139661	0.5167	0	0	17	0.0176	0	1	1	0	[illegible]
HIP_93011	GQVELGGGP	QSAPKENKP	GQVELGGGPQSAPKENKP		SCG2	14511/334890	0.998	93011	0.763	0.001154	-0.8167	0.6667	0	18	-1.2611	0	0	0	1	[illegible]
HIP_93012	SLQPLALEG	EMLKTGEKP	SLQPLALEGEMLKTGEKP		SCG2	21806/334890	0.995	93012	0.763	0.029184	0.35	0.1667	-1	18	-0.4667	-1	0	0	0	[illegible]
HIP_93013	AEDLQVGQV	STENVPRTR	AEDLQVGQVSTENVPRTR		NPY	3843/49410	0.961	93013	0.763	-0.048681	-0.7833	0.3333	-1	18	-0.8889	-1	0	0	0	[illegible]
HIP_93014	ELGGGPGAG	PGPQAEGDS	ELGGGPGAGPGPQAEGDS		CMGA	9283/247050	0.996	93014	0.763	-0.097997	-0.4333	0.8333	-3	18	-0.8333	-3	0	0	1	[illegible]
HIP_93015	QVELGGGPG	REDSKEAEK	QVELGGGPGREDSKEAEK		CMGA	18427/247050	0.977	93015	0.763	-0.056352	-2.3167	0.5	-2	18	-1.5278	-2	0	0	1	[illegible]
HIP_93016	GIVEQCCTS	WKSSHFERR	GIVEQCCTSWKSSHFERR		SCG1	52622/367830	0.829	93016	0.763	0.040948	-0.7667	0.3333	2	18	-0.8056	1	1	0	0	[illegible]
HIP_93017	QCCTSICSL	KEIRKGESR	QCCTSICSLKEIRKGESR		CMGA	39820/247050	0.796	93017	0.763	0.127352	0.4333	0.1667	2	18	-0.5833	2	0	0	0	[illegible]
HIP_93018	EALYLVC	QVFLIVLS	EALYLVCQVFLIVLS		IAPP	4206/45018	0.952	93018	0.763	0.041559	2.3333	0	-1	15	2.02	-1	2	1	0	[illegible]
HIP_93019	LVCGERGFF	GAEEAQDP	LVCGERGFFGAEEAQDP		CMGA	16508/247050	0.983	93019	0.763	0.015840	0.5167	0.3333	-3	17	-0.3	-3	2	1	0	[illegible]
HIP_93020	CCTSICSL	PGKWQQQG	CCTSICSLPGKWQQQG		SCG1	36221/367830	0.939	93020	0.763	0.206572	-0.0667	0.5	1	16	-0.2625	1	1	0	1	[illegible]
HIP_93021	LVEALYL	LQDTKEN	LVEALYLLQDTKEN		SCG1	33218/367830	0.952	93021	0.763	0.148357	0.5167	0	-2	14	-0.1571	-2	1	0	0	[illegible]
HIP_93022	CTSICSLYQ	HTAEKRKRL	CTSICSLYQHTAEKRKRL		SCG1	57808/367830	0.781	93022	0.763	0.050867	-0.5167	0	4	18	-0.6889	3	1	0	0	[illegible]
HIP_93023	ELGGGPGAG	WIPHVENRQ	ELGGGPGAGWIPHVENRQ		SCG2	7532/334890	0.999	93023	0.763	0.001606	0.5	0.5	0	18	-0.75	-1	1	0	1	[illegible]
HIP_93024	GSLQPLAL	SSPDYNPY	GSLQPLALSSPDYNPY		SCG2	26716/334890	0.993	93024	0.763	0.224734	1.0333	0.5	-1	16	-0.4688	-1	0	0	0	[illegible]
HIP_93025	VEALYLVC	EDELSEVL	VEALYLVCEDELSEVL		CMGA	20351/247050	0.971	93025	0.763	0.110845	0	0	-5	16	0.7812	-5	1	0	0	[illegible]
HIP_93026	AEDLQVGQV	DTEKWAEGG	AEDLQVGQVDTEKWAEGG		SCG1	27102/367830	0.975	93026	0.763	-0.079991	-1.2333	0.1667	-4	18	-0.8556	-4	0	0	0	[illegible]
HIP_93027	DLQVGQVEL	NQMDKARRH	DLQVGQVELNQMDKARRH		SCG1	55736/367830	0.801	93027	0.763	0.129641	-0.1	0	1	18	-1.1833	0	0	0	0	[illegible]
HIP_93028	HLCGSHLVE	YNPYQGVS	HLCGSHLVEYNPYQGVS		SCG2	40441/334890	0.976	93028	0.763	0.095472	-0.3167	0.1667	1	17	-0.2941	-1	2	1	0	[illegible]
HIP_93029	QHLCGSHL	EEEEEEEA	QHLCGSHLEEEEEEEA		CMGA	19661/247050	0.973	93029	0.763	-0.016740	-1.7833	0.1667	-6	17	-1.6	-8	0	1	6	[illegible]
HIP_93030	QHLCGSHL	EEEEEEEEA	QHLCGSHLEEEEEEEEA		CMGA	19662/247050	0.973	93030	0.763	-0.016740	-1.7833	0.1667	-6	17	-1.6	-8	0	1	6	[illegible]
HIP_93031	VEALYLVCG	KSGEATDGA	VEALYLVCGKSGEATDGA		CMGA	18947/247050	0.976	93031	0.763	0.136295	0.2	0.5	-2	18	0.3056	-2	1	0	0	[illegible]
HIP_93032	QPLALEGSL	PNPYNQEK	QPLALEGSLPNPYNQEK		SCG2	23522/334890	0.994	93032	0.763	-0.047113	-0.6833	0.6667	-1	17	-1.1176	-1	1	1	0	[illegible]
HIP_93033	LVEALYLVC	ETLRGDER	LVEALYLVCETLRGDER		CMGA	21303/247050	0.967	93033	0.763	0.118248	1.6833	0	-2	17	0.1471	-2	1	1	0	[illegible]
HIP_93034	AGSLQPLA	GRSRSNQLP	AGSLQPLAGRSRSNQLP		SCG2	34319/334890	0.986	93034	0.763	0.212036	-0.2833	0.5	2	17	-0.6412	2	0	1	0	[illegible]
HIP_93035	CGSHLVEAL	GAWKELDRN	CGSHLVEALGAWKELDRN		SCG1	29381/367830	0.968	93035	0.763	-0.042549	0.4333	0.1667	0	18	-0.3556	-1	1	0	0	[illegible]
HIP_93036	GAGSLQPLA	LSGLTLALS	GAGSLQPLALSGLTLALS		NPY	2915/49410	0.977	93036	0.763	-0.029549	1.1	0.5	0	18	1.0444	0	0	0	0	[illegible]
HIP_93037	GGPGAGSLQ	ESRSEALAV	GGPGAGSLQESRSEALAV		CMGA	30125/247050	0.913	93037	0.763	-0.054005	-1.55	0.3333	-1	18	-0.1889	-1	0	0	0	[illegible]
HIP_93038	GGGPGAGSL	QVFLIVLSV	GGGPGAGSLQVFLIVLSV		IAPP	1986/45018	0.991	93038	0.763	0.049404	1.0167	0.3333	0	18	1.3556	0	1	0	1	[illegible]
HIP_93039	SLYQLENYC	FSEDVNWG	SLYQLENYCFSEDVNWG		SCG1	25386/367830	0.979	93039	0.763	-0.127721	-0.6333	0.1667	-3	17	-0.5529	-3	2	1	0	[illegible]
HIP_93040	LQPLALEGS	SETHAAGHS	LQPLALEGSSETHAAGHS		SCG1	32546/367830	0.955	93040	0.763	-0.110087	-1.6167	0.5	0	18	-0.3111	-2	0	0	0	[illegible]
HIP_93041	LQPLALEGS	ENSRDNPFK	LQPLALEGSENSRDNPFK		SCG2	32914/334890	0.987	93041	0.763	-0.094331	-2.0833	0.5	-1	18	-1.0333	-1	0	0	0	[illegible]
HIP_93042	QPLALEGSL	APAEDMARY	QPLALEGSLAPAEDMARY		NPY	790/49410	0.996	93042	0.763	-0.058673	0.7667	0.5	-2	18	-0.2056	-2	0	0	0	[illegible]
HIP_93043	IVEQCCTSI	YGEEGAPGK	IVEQCCTSIYGEEGAPGK		SCG1	25774/367830	0.978	93043	0.763	-0.055371	-0.3667	0.3333	-2	18	-0.1944	-2	1	0	0	[illegible]
HIP_93044	SHLVEALY	SSMKLSFR	SHLVEALYSSMKLSFR		CMGA	22302/247050	0.963	93044	0.763	0.176009	0.7667	0.3333	2	16	0.1562	1	1	0	0	[illegible]
[illegible]																				

HIP_93503	GAGSLQPLA	ALSKSSAPP	GAGSLQPLAALSKSSAPP		SCG1	31508/367830	0.960	93503	0.763	-0.029613	1.4667	0.3333	1	18	0.1333	1	0	0	0	
HIP_93504	RGFFYTPKT	RARAYGFRG	RGFFYTPKTRARAYGFRG		CMGA	38736/247050	0.812	93504	0.763	0.079086	-2.2333	0.1667	5	18	-0.9278	5	1	0	0	
HIP_93505	GERGFFYT	EAGLPLQVR	GERGFFYTEAGLPLQVR		CMGA	21447/247050	0.967	93505	0.763	0.066117	-0.2167	0.1667	0	17	-0.3	0	3	1	0	
HIP_93506	AEDLQVGQ	NWGYEKRNL	AEDLQVGQNWGYEKRNL		SCG1	29455/367830	0.967	93506	0.763	-0.030373	-0.75	0.3333	-1	17	-1.3118	-1	2	1	0	
HIP_93507	FVNQHLCGS	PSSKDVMEK	FVNQHLCGSPSSKDVMEK		CMGA	18205/247050	0.978	93507	0.763	-0.032446	-0.3167	0.8333	1	18	-0.5556	0	0	0	0	
HIP_93508	QHLCGSHLV	LYPSDSQVS	QHLCGSHLVLYPSDSQVS		SCG1	30884/367830	0.962	93508	0.763	0.030724	0.95	0.1667	1	18	-0.0611	-1	1	0	0	
HIP_93509	VCGERGFFY	VEVLKREP	VCGERGFFYVEVLKREP		IAPP	4396/45018	0.947	93509	0.763	-0.010386	1.5333	0	0	17	-0.1529	0	3	1	0	
HIP_93510	VEALYLVCG	EIVEEQYTP	VEALYLVCGEIVEEQYTP		SCG2	15410/334890	0.998	93510	0.763	-0.085690	1.9167	0.1667	-4	18	0.3444	-4	1	0	0	
HIP_93511	AEDLQVGQ	EEEEEEAEA	AEDLQVGQEEEEEEAEA		CMGA	17600/247050	0.980	93511	0.763	-0.078647	-1.7	0.1667	-9	17	-1.5	-9	0	1	4	
HIP_93512	LQVGQVELG	EDWNPVEEK	LQVGQVELGEDWNPVEEK		SCG2	26985/334890	0.992	93512	0.763	-0.110597	-1.3333	0.1667	-4	18	-0.8333	-4	1	0	0	
HIP_93513	LVEALYLV	GQLGIQEE	LVEALYLVGQLGIQEE		SCG2	18273/334890	0.997	93513	0.763	0.137532	1.1	0.1667	-3	16	0.6438	-3	1	0	0	
HIP_93514	GSLQPLALE	EAVEEPSSK	GSLQPLALEEAVEEPSSK		CMGA	24849/247050	0.951	93514	0.763	-0.054271	0.7667	0	-3	18	-0.4556	-3	0	0	0	
HIP_93515	CGSHLVEAL	SSAPPITPE	CGSHLVEALSSAPPITPE		SCG1	26662/367830	0.976	93515	0.763	0.030979	0.3833	0.3333	-1	18	0.2167	-2	0	0	0	
HIP_93516	DLQVGQVEL	VNQHLCGSH	DLQVGQVELVNQHLCGSH		INSB	557/4968	0.851	93516	0.763	0.038008	0.2833	0	0	18	-0.1389	-2	0	0	0	
HIP_93517	VCGERGFF	EARFQDKQ	VCGERGFFEARFQDKQ		SCG1	43822/367830	0.896	93517	0.763	0.053387	-0.1667	0.1667	0	16	-0.8938	0	3	0	0	
HIP_93518	GSLQPLALE	PEMIRALEY	GSLQPLALEPEMIRALEY		SCG2	27133/334890	0.992	93518	0.763	-0.048274	-0.1833	0.1667	-2	18	0.0556	-2	0	0	0	
HIP_93519	VCGERGFF	YFMSDTREE	VCGERGFFYFMSDTREE		SCG1	22647/367830	0.985	93519	0.763	0.012112	1.4333	0.1667	-2	17	-0.5647	-2	4	1	0	
HIP_93520	FVNQHLCGS	SRNLQIPPE	FVNQHLCGSSRNLQIPPE		SCG2	37298/334890	0.982	93520	0.763	-0.053057	-1.25	0.5	1	18	-0.4889	0	0	0	0	
HIP_93521	VCGERGFF	QAPGEEEE	VCGERGFFQAPGEEEE		CMGA	15850/247050	0.985	93521	0.763	-0.016416	0.3167	0.3333	-4	16	-0.8875	-4	2	0	2	
HIP_93522	QCCTSICSL	PDYNPYQGV	QCCTSICSLPDYNPYQGV		SCG2	31139/334890	0.989	93522	0.763	0.067638	-0.15	0.3333	-1	18	-0.1389	-1	1	0	0	
HIP_93523	RGFFYTPKT	EDLIEMLKT	RGFFYTPKTEDLIEMLKT		SCG2	39794/334890	0.977	93523	0.763	-0.031610	-1.5667	0.1667	0	18	-0.4778	0	1	0	0	
HIP_93524	ERGFFYTPK	VHSSNNFGA	ERGFFYTPKVHSSNNFGA		IAPP	4167/45018	0.953	93524	0.763	-0.005880	-1	0.3333	2	18	-0.7611	1	2	0	0	
HIP_93525	GGPGAGSLQ	YGEESEEER	GGPGAGSLQYGEESEEER		SCG1	21727/367830	0.987	93525	0.763	-0.162620	-0.95	0.3333	-4	18	-1.4667	-4	1	0	1	
HIP_93526	HLVEALYLV	KDKETTENE	HLVEALYLVKDKETTENE		SCG1	44334/367830	0.893	93526	0.763	0.200670	-0.7667	0	-2	18	-0.7278	-3	1	0	0	
HIP_93527	SICSLYQLE	LNERKQASA	SICSLYQLELNERKQASA		SCG1	46317/367830	0.879	93527	0.763	-0.117865	-1.0667	0	0	18	-0.4222	0	1	0	0	
HIP_93528	CGERGFFY	ELESLSAI	CGERGFFYELESLSAI		CMGA	24985/247050	0.950	93528	0.763	0.109514	0.1833	0	-2	16	0.2062	-2	3	0	0	
HIP_93529	GSHLVEALY	KPLDSQSI	GSHLVEALYKPLDSQSI		SCG2	31248/334890	0.989	93529	0.763	0.149555	0.4333	0.1667	0	17	-0.0824	-1	1	1	0	
HIP_93530	VCGERGFF	DLQDTKEN	VCGERGFFDLQDTKEN		SCG1	33707/367830	0.950	93530	0.763	0.018562	0.3333	0.1667	-2	16	-0.925	-2	2	0	0	
HIP_93531	EAEDLQVGQ	EGKGEQEHS	EAEDLQVGQEGKGEQEHS		CMGA	15374/247050	0.986	93531	0.763	-0.116729	-1.25	0.3333	-4	18	-1.7111	-5	0	0	0	
HIP_93532	LVEALYLV	AGTEALPDG	LVEALYLVAGTEALPDG		SCG2	18152/334890	0.997	93532	0.763	0.105461	1.2333	0.1667	-3	17	0.8294	-3	1	1	0	
HIP_93533	QCCTSICS	TQQWPERKL	QCCTSICSTQQWPERKL		SCG2	43905/334890	0.967	93533	0.763	0.189878	-0.25	0.1667	1	17	-0.7118	1	1	1	0	
HIP_93534	VCGERGFFY	LVNAAGSG	VCGERGFFYLVNAAGSG		SCG2	29964/334890	0.990	93534	0.763	-0.004259	1.4667	0	0	17	0.5118	0	3	1	0	
HIP_93535	CGERGFFY	SDLLMREST	CGERGFFYSDLLMREST		NPY	4763/49410	0.939	93535	0.763	0.158918	0.6333	0.1667	-1	17	-0.3706	-1	3	1	0	
HIP_93536	HLVEALYL	RSSPETLIS	HLVEALYLRSSPETLIS		NPY	5069/49410	0.931	93536	0.763	0.236486	0.0333	0.3333	0	17	0.2941	-1	1	1	0	
HIP_93537	GPGAGSLQP	LLCAGQVTA	GPGAGSLQPLLCAGQVTA		CMGA	14771/247050	0.987	93537	0.763	0.110397	1.4667	0.1667	0	18	0.5667	0	0	0	0	
HIP_93538	RGFFYTPKT	PLDSQSIYQ	RGFFYTPKTPLDSQSIYQ		SCG2	37517/334890	0.981	93538	0.763	-0.009469	-1.25	0.3333	1	18	-0.7889	1	1	0	0	
HIP_93539	GGGPGAGSL	YEENSRDNP	GGGPGAGSLYEENSRDNP		SCG2	28951/334890	0.991	93539	0.763	-0.027429	-0.95	0.3333	-2	18	-1.3611	-2	1	0	1	
HIP_93540	VNQHLCGSH	DEEQKLYTD	VNQHLCGSHDEEQKLYTD		SCG2	34100/334890	0.986	93540	0.763	-0.100809	-2.4833	0.3333	-1	18	-1.3167	-3	0	0	0	
HIP_93541	EDLQVGQVE	RNLARVPKL	EDLQVGQVERNLARVPKL		SCG1	49255/367830	0.857	93541	0.763	-0.107348	-1.1667	0	0	18	-0.5611	0	0	0	0	
HIP_93542	QVGQVELGG	QMAYENLND	QVGQVELGGQMAYENLND		SCG2	7969/334890	0.999	93542	0.763	0.004745	0.5333	0.3333	-3	18	-0.6	-3	1	0	0	
HIP_93543	LYQLENYCN	QGAWKELD	LYQLENYCNQGAWKELD		SCG1	19556/367830	0.990	93543	0.763	-0.036893	-0.7333	0.1667	-2	17	-0.9765	-2	2	1	0	
HIP_93544	HLCGSHLVE	PVGPPKNDD	HLCGSHLVEPVGPPKNDD		SCG2	16652/334890	0.997	93544	0.763	0.055384	1.1167	0.3333	0	18	-0.6778	-2	0	0	0	
HIP_93545	GERGFFYT	KRFLGEGHH	GERGFFYTKRFLGEGHH		SCG1	53471/367830	0.822	93545	0.763	0.097039	-0.8	0	3	17	-1.0412	1	4	1	0	

HIP_94014	SHLVEALY	DQELESLS	SHLVEALYDQELESLS		CMGA	21037/247050	0.968	94014	0.762	0.105746	-1.0333	0	-3	16	-0.2	-4	1	0
HIP_94015	QHLCGSHLV	PEYNYDWW	QHLCGSHLVPEYNYDWW		SCG1	34869/367830	0.945	94015	0.762	0.171032	-0.2667	0.1667	0	17	-0.7824	-2	1	1
HIP_94016	SLYQLENYC	MSDDYETQQ	SLYQLENYCMSDDYETQQ		SCG2	22842/334890	0.995	94016	0.762	-0.115397	-0.7833	0.1667	-4	18	-1.2333	-4	1	0
HIP_94017	CCTSICSLY	KHMQFPPMY	CCTSICSLYKHMQFPPMY		SCG2	47957/334890	0.952	94017	0.762	-0.012819	-0.5833	0.1667	2	18	0.2056	1	1	0
HIP_94018	SLQPLAL	LPSEEKGH	SLQPLALLPSEEKGH		SCG1	28199/367830	0.971	94018	0.762	0.189489	1.8	0.3333	0	15	-0.3867	-1	0	1
HIP_94019	VNQHLCGSH	AENEKDRAD	VNQHLCGSHAENEKDRAD		SCG1	32139/367830	0.957	94019	0.762	-0.126300	-1.6	0.3333	0	18	-1.4667	-2	0	0
HIP_94020	EALYLVCGE	GRGSEEYRA	EALYLVCGEGRGSEEYRA		SCG1	26846/367830	0.975	94020	0.762	-0.030419	-1.1167	0.5	-2	18	-0.5389	-2	0	0
HIP_94021	LGGGPGAGS	EDSKEAEK	LGGGPGAGSEDSKEAEK		CMGA	19612/247050	0.973	94021	0.762	-0.057661	-1.2	0.5	-2	17	-1.1529	-2	0	1
HIP_94022	GSLQPLALE	DLDHPDLFQ	GSLQPLALEDLDHPDLFQ		SCG2	33407/334890	0.987	94022	0.762	-0.025300	-0.1833	0	-3	18	-0.2778	-4	0	0
HIP_94023	LQVGQVELG	PQGAWKELD	LQVGQVELGPQGAWKELD		SCG1	19861/367830	0.990	94023	0.762	-0.094029	-0.9333	0.5	-2	18	-0.3889	-2	0	0
HIP_94024	GERGFFYT	PYALNSEK	GERGFFYTPYALNSEK		SCG2	32220/334890	0.988	94024	0.762	-0.031110	-0.05	0.1667	0	16	-0.8875	0	4	0
HIP_94025	VEALYLVC	EDSKEAEK	VEALYLVCEDSKEAEK		CMGA	20952/247050	0.969	94025	0.762	0.157826	0.45	0.1667	-3	16	-0.3312	-3	1	0
HIP_94026	AGSLQPLAL	DELSEVLE	AGSLQPLALDELSEVLE		CMGA	19611/247050	0.973	94026	0.762	0.083328	1.0333	0	-4	17	0.3353	-4	0	1
HIP_94027	HLCGSHLVE	ERELDLPVD	HLCGSHLVEERELDLPVD		SCG2	38808/334890	0.979	94027	0.762	0.087048	-1.1667	0	-2	18	-0.2833	-4	0	0
HIP_94028	RGFFYTPKT	TLRGDERIL	RGFFYTPKTTLRGDERIL		CMGA	29861/247050	0.955	94028	0.762	0.045529	-1.2667	0.1667	2	18	-0.6944	2	1	0
HIP_94029	DLQVGQVEL	KPGAEEAQD	DLQVGQVELKPGAEEAQD		CMGA	20410/247050	0.971	94029	0.762	0.142934	-0.2333	0.3333	-4	18	-0.8167	-4	0	0
HIP_94030	QVGQVELGG	MYEENSRDN	QVGQVELGGMYEENSRDN		SCG2	13469/334890	0.998	94030	0.762	-0.018513	0.0167	0.3333	-3	18	-1.2056	-3	1	0
HIP_94031	ERGFFYTPK	ALLLCAGQV	ERGFFYTPKALLLCAGQV		CMGA	19708/247050	0.973	94031	0.762	0.087106	0.5333	0.1667	1	18	0.4167	1	2	0
HIP_94032	VNQHLCGSH	FEKPLDSQS	VNQHLCGSHFEKPLDSQS		SCG2	34656/334890	0.985	94032	0.762	-0.106174	-1.5	0.3333	1	18	-0.8889	-1	1	0
HIP_94033	DLQVGQVEL	HPQESKGQP	DLQVGQVELHPQESKGQP		SCG1	47052/367830	0.874	94033	0.762	0.106962	-0.6333	0.1667	-1	18	-1.1333	-2	0	0
HIP_94034	LGGGPGAGS	LAAVNSMPV	LGGGPGAGSLAAVNSMPV		SCG1	19366/367830	0.990	94034	0.762	0.124981	1.3333	0.3333	0	18	0.7222	0	0	0
HIP_94035	EQCCTSICS	EFPDFYDSE	EQCCTSICSEFPDFYDSE		SCG1	20684/367830	0.988	94035	0.762	-0.045930	0.65	0.3333	-5	18	-0.5222	-5	1	0
HIP_94036	SLQPLAL	PGWQAKREE	SLQPLALPGWQAKREE		CMGA	19877/247050	0.972	94036	0.762	0.203098	1.0833	0.3333	0	16	-0.7938	0	1	2
HIP_94037	SHLVEALYL	DTLSKPSP	SHLVEALYLDTLSKPSP		CMGA	26735/247050	0.960	94037	0.762	0.192119	0.9833	0	0	17	-0.0294	-1	1	1
HIP_94038	GPGAGSLQP	REHIAKRAM	GPGAGSLQPREHIAKRAM		SCG2	53683/334890	0.920	94038	0.762	-0.022138	-2.0833	0.1667	3	18	-0.7056	2	0	0
HIP_94039	QVGQVELGG	KGEQEHSQQ	QVGQVELGGKGEQEHSQQ		CMGA	17584/247050	0.980	94039	0.762	-0.105083	-0.8	0.5	-1	18	-1.4056	-2	0	0
HIP_94040	TSICSLYQL	LDHPDLFQN	TSICSLYQLLDHPDLFQN		SCG2	37531/334890	0.981	94040	0.762	-0.068307	-0.65	0	-1	18	-0.05	-2	1	0
HIP_94041	GSHLVEALY	LNYGEEGA	GSHLVEALYLNYGEEGA		SCG1	31363/367830	0.960	94041	0.762	-0.024190	0.55	0	-2	17	-0.1529	-3	2	1
HIP_94042	EQCCTSICS	PRAYFMSDT	EQCCTSICSPRAYFMSDT		SCG1	39881/367830	0.919	94042	0.762	0.064159	0.3167	0.3333	-1	18	-0.1778	-1	1	0
HIP_94043	DLQVGQVEL	HLPERDSLS	DLQVGQVELHLPERDSLS		SCG2	33351/334890	0.987	94043	0.762	-0.031284	0.5833	0.1667	-2	18	-0.4833	-3	0	0
HIP_94044	SHLVEALYL	GHSRERAD	SHLVEALYLGHSRERAD		SCG1	35515/367830	0.942	94044	0.762	0.245325	0.3167	0.3333	1	17	-0.5882	-1	1	1
HIP_94045	LYLVCGERG	MARYYSALR	LYLVCGERGMARYYSALR		NPY	4409/49410	0.948	94045	0.762	0.117251	-1.5333	0.1667	2	18	0.0611	2	1	0
HIP_94046	YLVCGERGF	GSSQETDKL	YLVCGERGFGSSQETDKL		SCG2	18849/334890	0.996	94046	0.762	-0.000804	-0.6833	0.6667	-1	18	-0.5611	-1	1	0
HIP_94047	VEALYLVC	GQNGERAT	VEALYLVCGQNGERAT		SCG2	29363/334890	0.991	94047	0.762	0.211921	0.5167	0.1667	-1	16	0.05	-1	1	0
HIP_94048	RGFFYTPK	EGENYQKG	RGFFYTPKEGENYQKG		SCG1	43571/367830	0.897	94048	0.762	-0.054338	-2.2667	0.3333	1	16	-1.675	1	2	0
HIP_94049	GSHLVEALY	MASLGEKR	GSHLVEALYMASLGEKR		SCG1	34494/367830	0.947	94049	0.762	0.005182	1.2	0.1667	1	17	-0.0706	0	1	1
HIP_94050	EQCCTSICS	LQDTKENRE	EQCCTSICSLQDTKENRE		SCG1	29220/367830	0.968	94050	0.762	-0.065140	0.5	0.1667	-2	18	-1.1167	-2	0	0
HIP_94051	ERGFFYTP	QMDKARRHP	ERGFFYTPQMDKARRHP		SCG1	49158/367830	0.858	94051	0.762	-0.041427	-1.45	0.1667	3	17	-1.6118	2	2	1
HIP_94052	VEALYLVC	AELKEAVEE	VEALYLVCAELKEAVEE		CMGA	21285/247050	0.967	94052	0.762	0.034026	2.1	0	-4	17	0.5412	-4	1	0
HIP_94053	VGQVELGGG	LYQLENY	VGQVELGGGLYQLENY		INSA	130/3024	0.963	94053	0.762	0.108094	-0.3667	0.5	-2	16	-0.1188	-2	1	2
HIP_94054	LVEALYLV	ALSKSSA	LVEALYLVALSKSSA		SCG1	32736/367830	0.954	94054	0.762	0.050323	1.9167	0.1667	0	15	1.1933	0	1	1
HIP_94055	QVELGGGP	VALNHLKAT	QVELGGGPVALNHLKAT		IAPP	2569/45018	0.984	94055	0.762	0.179418	1.2333	0.5	1	17	0.1353	0	0	1
HIP_94056	GERGFFYTP	PRPQSEES	GERGFFYTPPRPQSEES		SCG1	36194/367830	0.939	94056	0.762	-0.009402	-1.8833	0.5	-1	17	-1.5647	-1	2	1
HIP_94057	DLQVGQVEL	QNLLKELQD	DLQVGQVELQNLLKELQD		CMGA	24051/247050	0.956	94057	0.762	-0.003479	0.2167	0	-3	18	-0.4667	-3	0	0
HIP_94058	AGSLQPLA	EEEEEEEAE	AGSLQPLAEEEEEEEAE		CMGA	17707/247050	0.980	94058	0.762	-0.009135	-1.0833	0.1667	-8	17	-1.2529	-8	0	1
HIP_94059	VEALYLVC	NDNFLEGE	VEALYLVCNDNFLEGE		SCG1	31980/367830	0.958	94059	0.762	0.139080	0	0	-4	16	0.2625	-4	2	0
HIP_94060	SLQPLAL	TLISDLL	SLQPLALTLISDLL		NPY	4475/49410	0.946	94060	0.762	0.042249	2.8333	0	-1	14	1.3	-1	0	0
HIP_94061	HLVEALYL	GKPGAEEAQ	HLVEALYLGKPGAEEAQ		CMGA	21175/247050	0.968	94061	0.762	0.203683	0.0667	0.3333	-1	17	-0.2235	-2	1	1
[illegible]	[illegible]	[illegible]	[illegible]		[illegible]	[illegible]	[illegible]	[illegible]	[illegible]	[illegible]	[illegible]	[illegible]	[illegible]	[illegible]	[illegible]	[illegible]	[illegible]	[illegible]

HIP_94525	GPGAGSLQP	HPDLFQNRM	GPGAGSLQPHPDLFQNRM		SCG2	36336/334890	0.983	94525	0.762	-0.091441	-1.6	0.3333	1	18	-0.8	0	0	0	0	0.000000	-2.3333	-1	0	0.950000	0.1111	0.8389	2	0	1	0	2.3333	-1.3422	0	0	0	0	1	0.5103	0.9322	1.0556	-0.1667	0.2604	1.1146	-0.8164	0	0
HIP_94526	GSLQPLALE	NEQINDEMK	GSLQPLALENEQINDEMK		SCG2	34702/334890	0.985	94526	0.762	0.052622	-1.4	0	-3	18	-0.8389	-3	0	0	0	0.000000	-4.2	0	0.1	2.075000	0.2222	1.8528	0	0	0	0	0	-2.5939	0	0	0	1	0	0.4667	1.2583	0.8633	0	0.2178	0.7453	-0.6533	0.01	-0.42
HIP_94527	VEQCCTSIC	SQEKTHSR	VEQCCTSICSQEKTHSR		SCG1	36801/367830	0.936	94527	0.762	0.133208	-0.2667	0.3333	1	17	-0.7765	0	0	1	0	0.000000	-4.6667	-1	0.1	1.750000	0.2222	1.5278	0	0	0	0	4.6667	-0.4074	0	0	0	0	0	0.5051	1.0536	0.9162	0	0.2552	0.8393	-0.1347	0.01	-0.4667
HIP_94528	QVGQVELGG	REDSKEAEK	QVGQVELGGREDSKEAEK		CMGA	21557/247050	0.966	94528	0.762	-0.110220	-1.4167	0.3333	-2	18	-1.3778	-2	0	0	0	0.000000	-4.8333	-1	-0.0167	1.050000	0.4444	0.6056	0	2	0	1	4.8333	-0.8579	0	0	0	0	1	0.5103	0.9755	1.009	-0.3333	0.2604	1.018	-0.7229	0.0003	0.0806
HIP_94529	RGFFYTPKT	AGEEAVPEE	RGFFYTPKTAGEEAVPEE		CMGA	19484/247050	0.974	94529	0.762	-0.123196	-1.3833	0.3333	-2	18	-0.8722	-2	1	0	0	0.000000	1.3667	-2	-0.0333	0.450000	0.3333	0.1167	1	1	1	1	-2.7333	-0.1614	1.3667	0	1	0	0	0.5385	0.9803	1.0478	-0.1667	0.2899	1.0979	-0.7449	0.0011	-0.0456
HIP_94530	GERGFFY	YPEEKKE	GERGFFYYPEEKKE		CMGA	25943/247050	0.945	94530	0.762	0.130257	-0.35	0.1667	-1	14	-1.8357	-1	4	0	0	0.000000	-3.5667	-1	0.1167	0.700000	0.3333	0.3667	1	0	1	0	3.5667	-0.1283	-14.2667	0	1	0	0	0.4269	1.0872	0.879	0.1667	0.1823	0.7726	-0.1494	0.0136	-0.4161
HIP_94531	GERGFFY	IVLSVALNH	GERGFFYIVLSVALNH		IAPP	4195/45018	0.952	94531	0.762	-0.038226	2.8	0	1	16	0.6438	0	3	2	0	0.000000	2.7333	0	0	2.100000	0	2.1	0	0	0	0	0	5.88	8.2	0	1	0	0	0.2385	1.1436	0.5962	0.8333	0.0569	0.3554	0.6677	0	0
HIP_94532	EALYLVCGE	AWIPHVENR	EALYLVCGEAWIPHVENR		SCG2	13515/334890	0.998	94532	0.762	0.072864	0.6667	0.1667	-1	18	0.0389	-2	1	0	0	0.000000	2.2667	1	-0.1333	1.400000	0.1111	1.2889	0	1	0	1	2.2667	0.8593	2.2667	0	0	1	0	0.4269	1.0972	0.864	0.3333	0.1823	0.7465	0.2846	0.0178	-0.3022
HIP_94533	LYQLENYCN	PPMYEENS	LYQLENYCNPPMYEENS		SCG2	17064/334890	0.997	94533	0.762	-0.077744	-0.6	0.3333	-3	17	-1.2	-3	2	1	0	0.000000	0.3333	0	0.0833	0.625000	0.1111	0.5139	2	0	1	0	0	-0.3083	0.6667	0	1	0	0	0.5103	0.8422	1.169	0.1667	0.2604	1.3665	-0.3062	0.0069	0.0278
HIP_94534	VCGERGFF	EKPLDSQSI	VCGERGFFEKPLDSQSI		SCG2	36132/334890	0.983	94534	0.762	0.112478	-0.6333	0.3333	-1	17	-0.3412	-1	2	1	0	0.000000	-4.7333	0	0.0833	0.700000	0.4444	0.2556	1	1	1	1	0	-0.1619	-9.4667	1	0	0	0	0.4718	1.0003	0.9778	0	0.2226	0.9561	-0.2988	0.0069	-0.3944
HIP_94535	TSICSLYQ	DERILSILR	TSICSLYQDERILSILR		CMGA	33295/247050	0.882	94535	0.762	0.158856	-2.0833	0	0	17	0.2059	0	1	1	0	0.000000	-3.5	-1	0.1	0.950000	0.3333	0.6167	0	0	0	0	3.5	-1.2847	-3.5	0	1	0	0	0.4487	1.1641	0.8235	-0.3333	0.2013	0.6781	-0.9348	0.01	-0.35
HIP_94536	VELGGGPGA	RDNMNDNFL	VELGGGPGARDNMNDNFL		SCG1	30556/367830	0.963	94536	0.762	-0.100910	-1.95	0.3333	-2	18	-0.5778	-2	0	0	1	0.000000	-3.7667	0	0	0.475000	0.2222	0.2528	1	1	1	1	0	-0.4929	0	1	0	0	0	0.5103	0.8722	1.1756	-0.1667	0.2604	1.3821	-0.995	0	0
HIP_94537	VELGGGPGA	PSLGYKEIR	VELGGGPGAPSLGYKEIR		CMGA	13077/247050	0.990	94537	0.762	-0.074398	0.2	0.6667	0	18	-0.2556	0	0	0	1	0.000000	0.5333	0	-0.0167	0.000000	0	0	2	1	1	1	0	0	0	1	0	0	0	0.5167	0.865	1.15	0.3333	0.2669	1.3225	0.1033	0.0003	-0.0089
HIP_94538	EQCCTSICS	DFYDSEEP	EQCCTSICSDFYDSEEP		SCG1	31377/367830	0.960	94538	0.762	-0.033363	0.7	0.1667	-5	17	-0.7176	-5	2	1	0	0.000000	-2.7333	-1	-0.0167	0.625000	0.2222	0.4028	0	0	0	0	2.7333	0.2819	-5.4667	0	1	0	0	0.3821	1.0241	0.8318	0.1667	0.146	0.6919	0.2674	0.0003	0.0456
HIP_94539	GAGSLQPLA	GPNNQKRE	GAGSLQPLAGPNNQKRE		SCG2	22985/334890	0.995	94539	0.762	0.120264	-0.25	0.5	1	17	-1.1706	1	0	1	0	0.000000	-3.1667	0	0.1333	1.900000	0	1.9	2	1	1	1	0	-0.475	0	1	0	0	0	0.5167	0.8433	1.21	0.3333	0.2669	1.4641	-0.1292	0.0178	-0.4222
HIP_94540	GSLQPLAL	FNPYYDP	GSLQPLALFNPYYDP		SCG1	34571/367830	0.947	94540	0.762	0.157465	1.1833	0.1667	-1	15	-0.2067	-1	2	1	0	1.000000	-3.9	0	0.1167	1.150000	0	1.15	1	0	1	0	0	1.3608	-7.8	1	0	0	0	0.35	1.0817	0.89	0.6667	0.1225	0.7921	0.4142	0.0136	-0.455
HIP_94541	CTSICSLYQ	LLLSLLGA	CTSICSLYQLLLSLLGA		SCG1	30816/367830	0.963	94541	0.762	-0.010516	1.7333	0	0	17	1.5176	0	1	1	1	0.000000	4.1333	0	-0.05	1.575000	0	1.575	0	0	0	0	0	2.73	4.1333	0	1	0	0	0.3051	1.2219	0.6478	0.6667	0.0931	0.4197	0.5289	0.0025	-0.2067
HIP_94542	LQPLALEGS	NYDWWEKKP	LQPLALEGSNYDWWEKKP		SCG1	33065/367830	0.953	94542	0.762	0.010797	-2.1667	0.3333	-1	18	-1.0889	-1	2	0	0	0.000000	-1.2	0	-0.1	0.450000	0.2222	0.2278	0	1	0	1	0	-0.4935	-2.4	0	1	0	0	0.5603	0.9205	1.1356	-0.3333	0.3139	1.2897	-1.2139	0.01	0.12
HIP_94543	GSLQPLALE	AEEAQDPEG	GSLQPLALEAEEAQDPEG		CMGA	23067/247050	0.960	94543	0.762	-0.074147	-0.5167	0	-5	18	-0.6944	-5	0	0	0	0.000000	-2.4333	-1	0.05	1.850000	0.3333	1.5167	0	0	0	0	2.4333	-0.7836	0	0	0	1	0	0.4	1.455	0.6733	0	0.16	0.4534	-0.2067	0.0025	-0.1217
HIP_94544	TSICSLYQ	FERRDNMND	TSICSLYQFERRDNMND		SCG1	52833/367830	0.828	94544	0.762	0.093634	-1.0333	0	-1	17	-1.0647	-1	2	1	0	0.000000	-1.4	0	0.05	0.000000	0.4444	-0.4444	0	0	0	0	0	0.4593	-2.8	0	1	0	0	0.4103	1.1938	0.7723	0	0.1683	0.5965	-0.4239	0.0025	-0.07
HIP_94545	GGPGAGSLQ	EKSQREDEE	GGPGAGSLQEKSQREDEE		SCG1	38575/367830	0.926	94545	0.762	-0.100223	-1.45	0.3333	-3	18	-1.7833	-3	0	0	0	0.000000	-2.5667	0	0.05	1.400000	0.2222	1.1778	0	0	0	0	0	-1.7078	0	0	1	0	0	0.5051	1.0903	0.9212	-0.1667	0.2552	0.8485	-0.7324	0.0025	-0.1283
HIP_94546	LVCGERGFF	EKPNGSVE	LVCGERGFFEKPNGSVE		SCG2	36133/334890	0.983	94546	0.762	0.112480	-0.6333	0.3333	-1	17	-0.3353	-1	2	1	0	0.000000	-4.7333	0	0.0833	0.700000	0.4444	0.2556	1	1	1	1	0	-0.1619	-9.4667	1	0	0	0	0.4718	1.0003	0.9778	0	0.2226	0.9561	-0.2988	0.0069	-0.3944
HIP_94547	GERGFFYT	EEKIESQTQ	GERGFFYTEEKIESQTQ		SCG2	40640/334890	0.976	94547	0.762	0.114703	-1.6833	0	-2	17	-1.3882	-2	3	1	0	0.000000	-3.9	-1	0.1	0.700000	0.4444	0.2556	0	0	0	0	3.9	-0.4302	-11.7	0	1	0	0	0.4769	1.1822	0.809	-0.3333	0.2275	0.6544	-0.8028	0.01	-0.39
HIP_94548	LVCGERGFF	DLIEMLKT	LVCGERGFFDLIEMLKT		SCG2	25471/334890	0.993	94548	0.762	-0.009670	1.6667	0.1667	-1	17	0.5706	-1	2	1	0	0.000000	-0.1333	-1	-0.0333	0.700000	0.4444	0.2556	0	1	0	1	0.1333	0.4259	-0.2667	1	0	0	0	0.3218	1.0503	0.7628	0.5	0.1036	0.5819	0.5363	0.0011	0.0044
HIP_94549	YLVCGERGF	EEEEAEAG	YLVCGERGFEEEEAEAG		CMGA	22111/247050	0.964	94549	0.762	-0.028799	-2.1	0.1667	-5	17	-0.6529	-5	1	1	2	0.000000	-2.8	-4	0.0167	0.000000	0.6667	-0.6667	0	1	0	1	11.2	1.4	-2.8	1	0	0	0	0.5218	1.2086	0.8712	-0.5	0.2723	0.7589	-1.0958	0.0003	-0.0467
HIP_94550	CTSICSLY	DPLQWKSSH	CTSICSLYDPLQWKSSH		SCG1	35995/367830	0.940	94550	0.762	0.035296	0.0667	0.3333	1	17	-0.2765	0	2	1	0	0.000000	-1	-1	0.05	0.625000	0.1111	0.5139	1	0	1	0	1	0.0343	-2	0	1	0	0	0.4269	1.0022	0.9223	0.1667	0.1823	0.8507	0.0285	0.0025	-0.05
HIP_94551	GPGAGSLQP	EAKTHWLGA	GPGAGSLQPEAKTHWLGA		SCG2	46041/334890	0.959	94551	0.762	-0.002336	-1.15	0.1667	1	18	-0.4611	0	0	0	0	0.000000	-1.4333	0	-0.0167	0.000000	0.2222	-0.2222	1	0	1	0	0	0.2556	0	0	0	0	1	0.4551	1.1603	0.8845	0	0.2071	0.7823	-0.5234	0.0003	0.0239
HIP_94552	CCTSICSLY	MDKARRHPQ	CCTSICSLYMDKARRHPQ		SCG1	51884/367830	0.836	94552	0.762	0.001459	-0.6333	0.1667	3	18	-0.4889	2	1	0	0	0.000000	-2.4	0	0.0333	3.000000	0.2222	2.7778	0	0	0	0	0	-1.7593	-2.4	0	1	0	0	0.3821	1.0608	0.8368	0	0.146	0.7002	-0.242	0.0011	-0.08
HIP_94553	QVGQVELGG	YFMSDTREE	QVGQVELGGYFMSDTREE		SCG1	12905/367830	0.996	94553	0.762	0.094113	1.0667	0.3333	-3	18	-0.7	-3	2	0	0	0.000000	0.1333	0	-0.1667	1.050000	0.1111	0.9389	0	2	0	1	0	1.0015	0.2667	0	0	0	1	0.4051	0.9536	0.9345	0.5	0.1641	0.8733	0.4321	0.0278	-0.0222
HIP_94554	PLALEGSLQ	LNERKQASA	PLALEGSLQLNERKQASA		SCG1	41029/367830	0.912	94554	0.762	-0.132069	-0.6167	0.1667	0	18	-0.4944	0	0	0	0	0.000000	-0.9	-1	-0.0167	1.900000	0.3333	1.5667	0	0	0	0	0.9	-0.9661	0	0	1	0	0	0.4667	1.115	0.9133	0.1667	0.2178	0.8342	-0.2878	0.0003	0.015
HIP_94555	TSICSLYQL	QYTPQSLA	TSICSLYQLQYTPQSLA		SCG2	43562/334890	0.968	94555	0.762	0.095380	-1.0833	0	0	17	0.1	0	2	1	0	0.000000	-1.5	0	0.05	0.000000	0	0	0	0	0	0	0	0	-3	1	0	0	0	0.4769	1.0655	0.8723	0.1667	0.2275	0.7609	-0.5167	0.0025	-0.075
HIP_94556	GIVEQCCTS	VPQGLFRGG	GIVEQCCTSVPQGLFRGG		CMGA	18729/247050	0.977	94556	0.762	-0.120728	0.0167	0.3333	0	18	0.2667	0	0	0	0	0.000000	-0.6333	0	0	1.050000	0	1.05	1	0	1	0	0	0.0175	0	0	1	0	0	0.5218	0.8986	1.0112	0.1667	0.2723	1.0224	0.0087	0	0
HIP_94557	LYQLENYC	ENELTLNEK	LYQLENYCENELTLNEK		SCG1	36488/367830	0.938	94557	0.762	0.077206	-2.1333	0	-3	17	-1.0294	-3	1	1	0	0.000000	-2.7333	-2	0.1	0.625000	0.3333	0.2917	0	0	0	0	5.4667	-0.6222	-2.7333	0	0	0	0	0.5436	1.0672	1.069	-0.3333	0.2955	1.1427	-1.1597	0.01	-0.2733
HIP_94558	CGERGFFYT	EEQIEQAI	CGERGFFYTEEQIEQAI		SCG2	35263/334890	0.985	94558	0.762	0.076262	-1.6167	0	-3	17	-0.5529	-3	3	1	0	0.000000	-3.7667	-2	0.1	1.125000	0.2222	0.9028	0	0	0	0	7.5333	-1.4595	-11.3	0	1	0	0	0.5051	1.1969	0.8212	-0.1667	0.2552	0.6743	-0.8166	0.01	-0.3767
HIP_94559	AEDLQVGQV	SPDYNPYQG	AEDLQVGQVSPDYNPYQG		SCG2	18844/334890	0.996	94559	0.762	-0.047150	-0.9333	0.5	-3	18	-0.9944	-3	1	0	0	0.000000	-2.0667	-1	0.05	0.000000	0.1111	-0.1111	1	1	1	1	2.0667	0.1037	-2.0667	1	0	0	0	0.5385	0.8436	1.1012	0	0.2899	1.2125	-0.5026	0.0025	-0.1033
HIP_94560	EDLQVGQ	ERGFFYTP	EDLQVGQERGFFYTP		INSB	487/4968	0.890	94560	0.762	0.019119	-1.35	0.3333	-2	15	-0.8533	-2	2	1	0	0.000000	-2.9	0	0.05	0.700000	0.2222	0.4778	0	2	0	1	0	-0.645	-5.8	0	1	0	0	0.5385	0.9503	1.0212	-0.1667	0.2899	1.0428	-0.7269	0.0025	-0.145
HIP_94561	VEQCCTSIC	ELQKIAEKF	VEQCCTSICELQKIAEKF		SCG1	32810/367830	0.954	94561	0.762	-0.036392	0.5	0.1667	-1	18	0.1278	-1	0	0	0	0.000000	-2.1333	-1	0.0333	1.750000	0.2222	1.5278	0	0	0	0	2.1333	0.7639	0	0	0	0	0	0.4285	1.1369	0.7995	0.1667	0.1922	0.6392	0.2192	0.0011	-0.1044
HIP_94562	EQCCTSICS	RKLKHMQFP	EQCCTSICSRKLKHMQFP		SCG2	55009/334890	0.910	94562	0.762	0.091435	0.2667	0.1667	3	18	-0.5222	2	0	0	0	0.000000	-3.6	2	0.0333	0.625000	0.3333	0.2917	0	0	0	0	-7.2	0.0778	0	0	0	1	0	0.3821	1.0408	0.8151	0	0.146	0.6644	0.1019	0.0011	-0.12
HIP_94563	GSLQPLALE	NPVEEKIES	GSLQPLALENPVEEKIES		SCG2	20297/334890	0.996	94563	0.762	-0.044167	0.2	0.1667	-3	18	-0.4556	-3	0	0	0	0.000000	-1	1	0.0667	0.950000	0.2222	0.7278	1	0	1	0	-1	0.1456	0	0	0	1	0	0.4167	1.0933	0.9133	0.3333	0.1736	0.8342	0.0833	0.0044	-0.0667
HIP_94564	VNQHLCGSH	EEDLRKESK	VNQHLCGSHEEDLRKESK		SCG2	38554/334890	0.980	94564	0.762	-0.093638	-2.4833	0.3333	1	18	-1.5222	-1	0	0	0	0.000000	-2.0333	-3	0	1.900000	0.3333	1.5667	0	1	0	1	6.1	-3.8906	0	0	0	1	0	0.5603	1.0605	0.999	-0.6667	0.3139	0.9979	-1.3913	0	0
HIP_94565	VEALYLVCG	QNAFLNER	VEALYLVCGQNAFLNER		SCG1	36044/367830	0.940	94565	0.762	0.021612	0.1833	0.1667	-1	17	0.2941	-1	2	1	0	0.000000	-3.8333	0	0.0167	1.325000	0	1.325	0	1	0	1	0	0.2429	-7.6667	0	0	0	1	0.4551	0.9686	1.0178	0.3333	0.2071	1.036	0.0834	0.0003	-0.0639
HIP_94566	TSICSLYQ	DREKGLSAE	TSICSLYQDREKGLSAE		CMGA	33061/247050	0.884	94566	0.762	0.183266	-2.0833	0	-1	17	-0.6353	-1	1	1	0	0.000000	-3.5	-1	0.1	0.000000	0.4444	-0.4444	0	0	0	0	3.5	0.9259	-3.5	0	1	0	0	0.4487	1.1641	0.8235	-0.3333	0.2013	0.6781	-0.9348	0.01	-0.35
HIP_94567	EDLQVGQV	IVLSVALNH	EDLQVGQVIVLSVALNH		IAPP	2827/45018	0.980	94567	0.762	0.059081	2.1333	0.1667	-1	17	0.7412	-2	0	1	0	1.000000	4.0667	0	-0.1333	4.200000	0	4.2	0	1	0	1	0	8.96	0	1	0	0	0	0.35	1.0183	0.7417	0.6667	0.1225	0.5501	0.7467	0.0178	-0.5422
HIP_94568	GAGSLQPLA	SLIPLIFLI	GAGSLQPLASLIPLIFLI		SCG2	25628/334890	0.993	94568	0.762	-0.032305	1.9167	0.3333	0	18	1.6556	0	0	0	0	0.000000	1.1667	0	0	1.900000	0	1.9	1	0	1	0	0	3.6417	0	1	0	0	0	0.35	1.0833	0.8083	0.6667	0.1225	0.6534	0.6708	0	0
HIP_94569	CGSHLVEAL	KHMQFPPMY	CGSHLVEALKHMQFPPMY		SCG2	53639/334890	0.920	94569	0.762	0.019450	-0.5167	0	2	18	-0.0167	0	0	0	0	0.000000	-2.4333	2	0	1.400000	0.2222	1.1778	0	0	0	0	-4.8667	-0.6085	0	1	0	0	0	0.3436	1.2505	0.719	0	0.1181	0.5169	-0.1775	0	0
HIP_94570	VCGERGFF	MLGNKRLG	VCGERGFFMLGNKRLG		NPY	3917/49410	0.959	94570	0.762	-0.002264	1.75	0.3333	2	16	0.0187	2	2	0	0	1.000000	0.0333	0	0	1.175000	0.2222	0.9528	0	2	0	1	0	1.6674	0.0667	1	0	0	0	0.3667	0.9833	0.8833	0.6667	0.1344	0.7803	0.6417	0	0
HIP_94571	QCCTSICSL	SKAEGNNQA	QCCTSICSLSKAEGNNQA		CMGA	32625/247050	0.888	94571	0.762	0.115317	0.4333	0.3333	0	18	-0.3056	0	0	0	0	0.000000	-2.8	1	0.05	0.000000	0.2222	-0.2222	0	0	0	0	-2.8	-0.0963	0	1	0	0	0	0.4103	1.0655	0.8956	0.1667	0.1683	0.8022	0.1778	0.0025	-0.14
HIP_94572	GIVEQCCTS	RSRSNQLPK	GIVEQCCTSRSRSNQLPK		SCG2	54482/334890	0.914	94572	0.762	0.092976	-1.4667	0.3333	2	18	-0.8056	2	0	0	0	0.000000	-3.6	2	0.05	0.000000	0.2222	-0.2222	0	0	0	0	-7.2	0.3259	0	0	1	0	0	0.5154	0.9158	1.0125	-0.3333	0.2656	1.0271	-0.7559	0.0025	-0.18

[illegible]

HIP_95036	GGPGAGSLQ	WERYRGRG	GGPGAGSLQWERYRGRG		SCG1	55183/367830	0.806	95036	0.761	0.028033	-1.5667	0.1667	2	17	-1.2882	2	2	1
HIP_95037	QHLCGSHLV	GEQEHGQQ	QHLCGSHLVGEQEHGQQ		CMGA	21943/247050	0.965	95037	0.761	0.214450	-0.4333	0.1667	1	17	-1.1	-2	0	1
HIP_95038	VELGGGPGA	VCGERGFFY	VELGGGPGAVCGERGFFY		INSB	101/4968	0.992	95038	0.761	-0.047231	1.0167	0.5	-1	18	0.2944	-1	0	0
HIP_95039	GQVELGGG	LYQLENYC	GQVELGGGLYQLENYC		INSA	121/3024	0.967	95039	0.761	0.086844	-0.3667	0.5	-2	16	-0.225	-2	1	0
HIP_95040	GSLQPLAL	TRLEDPAMW	GSLQPLALTRLEDPAMW		NPY	5778/49410	0.908	95040	0.761	0.172892	1.3333	0	-1	17	-0.0176	-1	0	1
HIP_95041	GERGFFY	SSQAELK	GERGFFYSSQAELK		CMGA	24868/247050	0.951	95041	0.761	0.206159	-0.1333	0.3333	0	14	-0.8143	0	3	0
HIP_95042	HLVEALYL	GPTKADTE	HLVEALYLGPTKADTE		SCG1	28004/367830	0.972	95042	0.761	0.219965	0.6	0.3333	-1	16	-0.1938	-2	1	0
HIP_95043	VELGGGPGA	QREDEEEEE	VELGGGPGAQREDEEEEE		SCG1	25587/367830	0.979	95043	0.761	-0.102827	-1.95	0.3333	-7	18	-1.6333	-7	0	0
HIP_95044	SICSLYQLE	QVLKTSRKD	SICSLYQLEQVLKTSRKD		SCG1	37126/367830	0.934	95044	0.761	-0.034948	0.2167	0	1	18	-0.45	1	1	0
HIP_95045	CTSICSLYQ	GEEENELT	CTSICSLYQGEEENELT		SCG1	25473/367830	0.979	95045	0.761	0.106427	-1.4	0.1667	-4	17	-0.5059	-4	1	1
HIP_95046	LQPLALEGS	EGEEDATSE	LQPLALEGSEGEEDATSE		SCG1	26121/367830	0.977	95046	0.761	-0.143447	-2.0167	0.5	-6	18	-0.7889	-6	0	0
HIP_95047	LQPLALEGS	EEGEEDATS	LQPLALEGSEEGEEDATS		SCG1	26122/367830	0.977	95047	0.761	-0.143447	-2.0167	0.5	-6	18	-0.7889	-6	0	0
HIP_95048	TSICSLY	FLIVLSV	TSICSLYFLIVLSV		IAPP	3643/45018	0.965	95048	0.761	-0.022663	2.1333	0.1667	0	14	2.1214	0	2	0
HIP_95049	ERGFFYTPK	GSEEYRAP	ERGFFYTPKGSEEYRAP		SCG1	37103/367830	0.934	95049	0.761	-0.092752	-1.8167	0.5	0	17	-1.4176	0	2	1
HIP_95050	VEALYLVCG	TENENTKF	VEALYLVCGTENENTKF		SCG1	30597/367830	0.963	95050	0.761	0.066139	-0.2333	0.1667	-2	17	-0.0824	-2	1	1
HIP_95051	LYLVCGERG	ALEYIENLR	LYLVCGERGALEYIENLR		SCG2	27155/334890	0.992	95051	0.761	0.090198	-1.05	0.1667	-1	18	0.1	-1	1	0
HIP_95052	SLYQLENYC	PDFYDSEE	SLYQLENYCPDFYDSEE		SCG1	21894/367830	0.986	95052	0.761	-0.109426	-0.7667	0.1667	-5	17	-1.1	-5	3	1
HIP_95053	VEQCCTSIC	PKNDDTPNR	VEQCCTSICPKNDDTPNR		SCG2	35109/334890	0.985	95053	0.761	0.103101	-0.4667	0.3333	-1	18	-1.0333	-1	0	0
HIP_95054	QHLCGSHLV	EEGPTVVL	QHLCGSHLVEEGPTVVL		CMGA	20655/247050	0.970	95054	0.761	0.189966	-0.4333	0.1667	0	17	0.3353	-2	0	1
HIP_95055	GIVEQCCT	NPYYDPLQ	GIVEQCCTNPYYDPLQ		SCG1	36162/367830	0.939	95055	0.761	0.058340	-0.35	0.1667	-2	16	-0.4312	-2	2	0
HIP_95056	ELGGGPGAG	IFLISGAEA	ELGGGPGAGIFLISGAEA		SCG2	14932/334890	0.998	95056	0.761	0.088053	2.0167	0.3333	-2	18	0.7222	-2	1	0
HIP_95057	PLALEGSLQ	PLQQKENGD	PLALEGSLQPLQQKENGD		SCG2	18854/334890	0.996	95057	0.761	-0.191605	-0.3	0.3333	-2	18	-0.9	-2	0	0
HIP_95058	FVNQHLCG	LGALAEAYP	FVNQHLCGLGALAEAYP		NPY	2774/49410	0.979	95058	0.761	-0.043040	1.85	0.3333	0	17	0.5235	-1	0	1
HIP_95059	VELGGGPGA	GELFNPYYD	VELGGGPGAGELFNPYYD		SCG1	7894/367830	0.998	95059	0.761	-0.167169	-0.05	0.5	-3	18	-0.3	-3	1	0
HIP_95060	DLQVGQVEL	LEGEEENE	DLQVGQVELLEGEEENE		SCG1	19845/367830	0.990	95060	0.761	-0.035220	0.7333	0.1667	-7	17	-0.9412	-7	0	1
HIP_95061	GERGFFY	EGSANRRPE	GERGFFYEGSANRRPE		CMGA	24368/247050	0.954	95061	0.761	0.214988	-0.0667	0.3333	0	16	-1.5625	0	3	2
HIP_95062	VELGGGPGA	EEEPEYGEE	VELGGGPGAEEEPEYGEE		SCG1	24710/367830	0.981	95062	0.761	-0.097572	-1.7833	0.3333	-7	18	-1.1778	-7	0	0
HIP_95063	QHLCGSHLV	PEGKGEQE	QHLCGSHLVPEGKGEQE		CMGA	19217/247050	0.975	95063	0.761	0.201994	-0.1167	0.3333	0	17	-1.0059	-2	0	1
HIP_95064	GERGFFYT	GHSRERAD	GERGFFYTGHSRERAD		SCG1	38457/367830	0.927	95064	0.761	0.131030	-0.6	0.3333	1	16	-1.4875	0	3	0
HIP_95065	YLVCGERGF	RELDLPVDL	YLVCGERGFRELDLPVDL		SCG2	33116/334890	0.987	95065	0.761	-0.021518	-1.05	0.1667	-2	18	0.1222	-2	1	0
HIP_95066	GGPGAGSLQ	SQETDKLAP	GGPGAGSLQSQETDKLAP		SCG2	34067/334890	0.986	95066	0.761	-0.078225	-1.3833	0.3333	-1	18	-0.7667	-1	0	0
HIP_95067	ERGFFYTP	PKNDDTPN	ERGFFYTPPKNDDTPN		SCG2	36939/334890	0.982	95067	0.761	-0.000389	-2.1	0.3333	-1	16	-1.7625	-1	3	0
HIP_95068	GIVEQCCTS	GAEEAQDPE	GIVEQCCTSGAEEAQDPE		CMGA	12459/247050	0.991	95068	0.761	-0.140399	-0.1833	0.3333	-5	18	-0.6167	-5	0	0
HIP_95069	VELGGGPGA	TQNAFLNER	VELGGGPGATQNAFLNER		SCG1	25863/367830	0.978	95069	0.761	-0.104822	-1.3167	0.3333	-1	18	-0.4278	-1	0	0
HIP_95070	CTSICSLY	SEEEPEYGE	CTSICSLYSEEEPEYGE		SCG1	28076/367830	0.972	95070	0.761	0.050909	-1.0167	0.3333	-5	17	-0.7	-5	1	1
HIP_95071	CGERGFFYT	YIENLRQQ	CGERGFFYTYIENLRQQ		SCG2	41587/334890	0.973	95071	0.761	0.014939	0.0833	0	0	17	-0.8353	0	4	1
HIP_95072	CGERGFFYT	SKEWEDSK	CGERGFFYTSKEWEDSK		CMGA	30110/247050	0.913	95072	0.761	0.148096	-1.2333	0.1667	-1	17	-1.3824	-1	4	1
HIP_95073	SHLVEALY	SEEKGHPQ	SHLVEALYSEEKGHPQ		SCG1	34390/367830	0.947	95073	0.761	0.171791	-0.5833	0.1667	0	16	-0.975	-2	1	0
HIP_95074	LYLVCGERG	KSSQESGEE	LYLVCGERGKSSQESGEE		SCG1	35421/367830	0.943	95074	0.761	-0.129955	-2.3167	0.5	-2	18	-0.9167	-2	0	0
HIP_95075	ALYLVCGER	QKGERGEDS	ALYLVCGERQKGERGEDS		SCG1	40807/367830	0.914	95075	0.761	-0.109769	-2.7	0.3333	-1	18	-0.9778	-1	0	0
HIP_95076	LQVGQVEL	WWEKKPFS	LQVGQVELWWEKKPFS		SCG1	49247/367830	0.858	95076	0.761	0.078713	-0.1333	0	0	16	-0.475	0	2	0
HIP_95077	AGSLQPLAL	PGKGRHHR	AGSLQPLALPGKGRHHR		SCG1	41125/367830	0.912	95077	0.761	0.247151	0.5833	0.3333	5	17	-0.7647	3	0	1
HIP_95078	SLQPLAL	FLEGEEEN	SLQPLALFLEGEEEN		SCG1	29858/367830	0.966	95078	0.761	0.010541	2.0833	0	-4	15	-0.2667	-4	1	1
HIP_95079	YQLENYCN	GESRSEALA	YQLENYCNGESRSEALA		CMGA	18201/247050	0.978	95079	0.761	0.143318	-1.1667	0.3333	-2	17	-0.9647	-2	1	1
HIP_95080	ALYLVCGER	RPQSEESWD	ALYLVCGERRPQSEESWD		SCG1	39160/367830	0.923	95080	0.761	-0.131164	-3	0.3333	-2	18	-0.9	-2	0	0
HIP_95081	GSHLVEALY	RSSQGGSL	GSHLVEALYRSSQGGSL		SCG1	38821/367830	0.925	95081	0.761	0.225682	-0.3	0.3333	1	17	-0.1765	0	1	1
HIP_95082	GGGPGAG	ERGFFYTP	GGGPGAGERGFFYTP		INSB	495/4968	0.886	95082	0.761	0.011205	-1.2333	0.5	0	15	-0.5467	0	2	1
HIP_95083	LYQLENYCN	KRLGELFNP	LYQLENYCNKRLGELFNP		SCG1	58769/367830	0.770	95083	0.761	0.051681	-1.15	0	0	18	-0.75	0	1	0

HIP_95547	DLQVGQVEL	SKSSAPPIT	DLQVGQVELSKSSAPPIT		SCG1	44263/367830	0.893	95547	0.761	0.203665	-0.1667	0.3333	-1	18	-0.1278
HIP_95548	YLVCGERGF	FNPYYDPL	YLVCGERGFFNPYYDPL		SCG1	22531/367830	0.985	95548	0.761	-0.103102	-0.7333	0.3333	-1	17	-0.1765
HIP_95549	GPGAGSLQP	EEEGSANR	GPGAGSLQPEEEGSANR		CMGA	25518/247050	0.947	95549	0.761	-0.021199	-1.9667	0.1667	-2	17	-1.2353
HIP_95550	NQHLCGSHL	RALEYIENL	NQHLCGSHLRALEYIENL		SCG2	40522/334890	0.976	95550	0.761	-0.005243	0.15	0.1667	1	18	-0.3833
HIP_95551	QVGQVELGG	DKQYSSHHT	QVGQVELGGDKQYSSHHT		SCG1	41799/367830	0.908	95551	0.761	-0.020138	-1.3167	0.3333	1	18	-1.1333
HIP_95552	DLQVGQVEL	PQSEESWDE	DLQVGQVELPQSEESWDE		SCG1	26708/367830	0.976	95552	0.761	0.109859	-0.2333	0.3333	-6	18	-1.1111
HIP_95553	SHLVEALYL	MLSKSGYP	SHLVEALYLMLSKSGYP		SCG2	34587/334890	0.985	95553	0.761	0.088592	1.8667	0.1667	1	17	0.3235
HIP_95554	VEALYLVCG	EEPVSTHQ	VEALYLVCGEEPVSTHQ		SCG1	27383/367830	0.974	95554	0.761	0.027104	-0.3833	0.3333	-2	17	0.1471
HIP_95555	EALYLVCGE	GEYLARMLV	EALYLVCGEGEYLARMLV		SCG2	26455/334890	0.993	95555	0.761	-0.150652	-1.1	0.3333	-2	18	0.7333
HIP_95556	HLCGSHLV	EESNVSMAS	HLCGSHLVEESNVSMAS		SCG1	32827/367830	0.954	95556	0.761	0.161904	-0.5	0.1667	0	17	0.1

[illegible]

HIP_96058	RGFFYTPK	RGEDSSEE	RGFFYTPKRGEDSSEE		SCG1	40501/367830	0.915	96058	0.760	-0.075373	-2.4333	0.3333	-1	16	-1.7063	-1	2	0	0
HIP_96059	QVGQVELGG	EEPEYGEEI	QVGQVELGGEEPEYGEEI		SCG1	19166/367830	0.991	96059	0.760	-0.143006	-0.9333	0.5	-6	18	-0.8778	-6	0	0	0
HIP_96060	VNQHLCGS	YDWWEKKPF	VNQHLCGSYDWWEKKPF		SCG1	35766/367830	0.941	96060	0.760	0.043570	-0.7333	0.3333	3	17	-1.0353	0	3	1	0
HIP_96061	VGQVELGGG	GEEENELTL	VGQVELGGGGEEENELTL		SCG1	18182/367830	0.992	96061	0.760	-0.091400	-1.4333	0.6667	-5	18	-0.4111	-5	0	0	3
HIP_96062	EQCCTSICS	QGAKERAHQ	EQCCTSICSQGAKERAHQ		CMGA	20133/247050	0.972	96062	0.760	0.080581	0.6833	0.3333	1	18	-0.9	0	0	0	0
HIP_96063	ERGFFYTPK	AAVLALLLC	ERGFFYTPKAAVLALLLC		CMGA	19780/247050	0.973	96063	0.760	0.087486	0.2667	0.1667	1	18	0.9444	1	2	0	1
HIP_96064	ICSLYQLEN	RPRHHHGRS	ICSLYQLENRPRHHHGRS		SCG1	59960/367830	0.756	96064	0.760	0.115025	-2.3	0.1667	5	18	-1.3278	2	1	0	1
HIP_96065	LYQLENYC	FLIVLSVAL	LYQLENYCFLIVLSVAL		IAPP	3677/45018	0.965	96065	0.760	-0.057794	1.4667	0	-1	17	1.4765	-1	2	1	0
HIP_96066	FVNQHLCG	NWGYEKRN	FVNQHLCGNWGYEKRN		SCG1	37382/367830	0.933	96066	0.760	0.069777	0.1833	0.3333	2	16	-1.175	1	2	0	0
HIP_96067	SLQPLALEG	EAASFQRNQ	SLQPLALEGEAASFQRNQ		SCG2	23770/334890	0.994	96067	0.760	0.011793	0	0.1667	-1	18	-0.5278	-1	0	0	0
HIP_96068	QCCTSICSL	ENIEKNEQI	QCCTSICSLENIEKNEQI		SCG2	28561/334890	0.991	96068	0.760	0.000387	0.5	0.1667	-2	18	-0.3278	-2	0	0	0
HIP_96069	ERGFFYTPK	HWLGAALSL	ERGFFYTPKHWLGAALSL		SCG2	28470/334890	0.991	96069	0.760	0.112570	-1.0833	0.1667	2	18	-0.0333	1	3	0	0
HIP_96070	LYQLENYCN	EAVPEEEGP	LYQLENYCNEAVPEEEGP		CMGA	18413/247050	0.978	96070	0.760	-0.141337	0.0333	0	-5	18	-1.0056	-5	1	0	1
HIP_96071	CSLYQLENY	QVSEEVKTR	CSLYQLENYQVSEEVKTR		SCG1	36612/367830	0.937	96071	0.760	-0.111249	-1.4	0.1667	-1	18	-0.8778	-1	1	0	0
HIP_96072	HLVEALYL	RSNQLPKAA	HLVEALYLRSNQLPKAA		SCG2	39760/334890	0.977	96072	0.760	0.221036	-0.4167	0.1667	2	17	-0.0588	1	1	1	0
HIP_96073	LVCGERGFF	ALRHYINL	LVCGERGFFALRHYINL		NPY	5463/49410	0.918	96073	0.760	0.045964	1.05	0.1667	2	17	0.5118	1	2	1	0
HIP_96074	LYLVCGERG	PGPQLRRGW	LYLVCGERGPGPQLRRGW		CMGA	23945/247050	0.956	96074	0.760	-0.068372	-2	0.6667	2	18	-0.5222	2	0	0	0
HIP_96075	QCCTSICSL	RKLKHMQFP	QCCTSICSLRKLKHMQFP		SCG2	57260/334890	0.890	96075	0.760	0.119473	0.15	0.1667	4	18	-0.1167	3	0	0	0
HIP_96076	HLVEALYLV	NFLVHSS	HLVEALYLVNFLVHSS		IAPP	4426/45018	0.946	96076	0.760	0.047096	1.6333	0	1	16	1.0062	-1	2	2	0
HIP_96077	QCCTSICS	RAYFMSDTR	QCCTSICSRAYFMSDTR		SCG1	50963/367830	0.844	96077	0.760	0.063114	0.3667	0.1667	1	17	-0.1529	1	2	1	0
HIP_96078	YLVCGERGF	GAKERAHQQ	YLVCGERGFGAKERAHQQ		CMGA	27995/247050	0.931	96078	0.760	0.011760	-0.7667	0.3333	2	18	-0.8722	1	1	0	0
HIP_96079	IVEQCCTSI	DYETQQWPE	IVEQCCTSIDYETQQWPE		SCG2	30708/334890	0.989	96079	0.760	-0.053920	-0.8833	0.1667	-4	18	-0.6833	-4	1	0	0
HIP_96080	AGSLQPLAL	DKLAPVSK	AGSLQPLALDKLAPVSK		SCG2	43651/334890	0.968	96080	0.760	0.212588	0.9667	0	1	17	0.2824	1	0	1	0
HIP_96081	HLCGSHLV	GPQLRRGWR	HLCGSHLVGPQLRRGWR		CMGA	25263/247050	0.949	96081	0.760	0.234052	-0.1167	0.3333	5	17	-0.5765	3	0	1	0
HIP_96082	VNQHLCGSH	QVFLIVLSV	VNQHLCGSHQVFLIVLSV		IAPP	2796/45018	0.981	96082	0.760	0.011893	-0.15	0.3333	2	18	1.0611	0	1	0	0
HIP_96083	SICSLYQLE	VSVPLQQKE	SICSLYQLEVSVPLQQKE		SCG2	24767/334890	0.994	96083	0.760	-0.104665	0.7333	0.1667	-1	18	0.0056	-1	1	0	0
HIP_96084	VNQHLCGSH	QEEEQIEQA	VNQHLCGSHQEEEQIEQA		SCG2	31671/334890	0.989	96084	0.760	-0.127510	-2.4833	0.3333	-2	18	-1.2389	-4	0	0	1
HIP_96085	CGERGFFYT	PPASLPSQ	CGERGFFYTPPASLPSQ		CMGA	20744/247050	0.969	96085	0.760	0.067112	-0.1	0.3333	0	17	-0.4118	0	3	1	0
HIP_96086	TSICSLYQL	AEAKTHWL	TSICSLYQLAEAKTHWL		SCG2	44297/334890	0.965	96086	0.760	0.017669	-0.15	0	1	17	0.1588	0	1	1	0
HIP_96087	GSLQPLAL	ENLAAMDL	GSLQPLALENLAAMDL		SCG1	30212/367830	0.965	96087	0.760	0.131611	1.0333	0	-2	16	0.5938	-2	0	1	0
HIP_96088	SICSLYQL	WIPHVENR	SICSLYQLWIPHVENR		SCG2	29960/334890	0.990	96088	0.760	-0.045226	0.1667	0.1667	1	16	-0.0188	0	2	0	0
HIP_96089	GERGFFYT	EEAVPEEE	GERGFFYTEEAVPEEE		CMGA	23261/247050	0.959	96089	0.760	-0.013082	-0.7333	0	-5	16	-1.1438	-5	3	0	1
HIP_96090	HLCGSHLVE	EEEDNRDS	HLCGSHLVEEEEDNRDS		CMGA	24062/247050	0.956	96090	0.760	0.058210	-1	0	-3	17	-1.3588	-5	0	1	2
HIP_96091	VELGGGPGA	TDGARPQAL	VELGGGPGATDGARPQAL		CMGA	13081/247050	0.990	96091	0.760	-0.087239	-0.8	0.5	-1	18	-0.2056	-1	0	0	1
HIP_96092	HLCGSHLV	GEEDATSEV	HLCGSHLVGEEDATSEV		SCG1	28186/367830	0.972	96092	0.760	0.164015	-0.4333	0.1667	-2	17	-0.1882	-4	0	1	0
HIP_96093	YLVCGERGF	NIEKNEQIN	YLVCGERGFNIEKNEQIN		SCG2	31115/334890	0.989	96093	0.760	-0.026802	-0.7667	0.1667	-1	18	-0.7056	-1	1	0	0
HIP_96094	EQCCTSICS	HTAEKRKRL	EQCCTSICSHTAEKRKRL		SCG1	53774/367830	0.820	96094	0.760	0.042203	0.6833	0.1667	3	18	-0.8833	2	0	0	0
HIP_96095	HLCGSHLVE	LCAGQVTAL	HLCGSHLVELCAGQVTAL		CMGA	23205/247050	0.960	96095	0.760	0.010714	2.1	0	1	18	0.9167	-1	0	0	0
HIP_96096	QHLCGSHLV	TGPNNQKR	QHLCGSHLVTGPNNQKR		SCG2	31626/334890	0.989	96096	0.760	0.223285	0.35	0.3333	4	17	-1.0824	2	0	1	0
HIP_96097	IVEQCCTSI	NYQKGERGE	IVEQCCTSINYQKGERGE		SCG1	42760/367830	0.902	96097	0.760	-0.014158	-0.8833	0.1667	-1	18	-0.8222	-1	1	0	0
HIP_96098	GGPGAGSLQ	SLIPLIFLI	GGPGAGSLQSLIPLIFLI		SCG2	24511/334890	0.994	96098	0.760	-0.010369	1.1667	0.3333	0	18	1.3	0	0	0	0
HIP_96099	LGGGPGAGS	NDNFLEGEE	LGGGPGAGSNDNFLEGEE		SCG1	26713/367830	0.976	96099	0.760	-0.087018	-1.65	0.3333	-4	18	-0.7556	-4	1	0	1
HIP_96100	VEALYLV	EGNNQAP	VEALYLVEGNNQAP		CMGA	19971/247050	0.972	96100	0.760	0.202304	-0.1167	0.1667	-2	14	-0.0857	-2	1	0	0
HIP_96101	ERGFFYTPK	EEEEGENY	ERGFFYTPKEEEEGENY		SCG1	38916/367830	0.924	96101	0.760	-0.066393	-2.7833	0.1667	-4	17	-1.9412	-4	2	1	2
HIP_96102	QVELGGGPG	EEDLRKESK	QVELGGGPGEEDLRKESK		SCG2	24131/334890	0.994	96102	0.760	-0.084960	-2.15	0.5	-2	18	-1.4167	-2	0	0	1
HIP_96103	LVEALYLVC	KCIVEVISD	LVEALYLVCKCIVEVISD		CMGA	24193/247050	0.955	96103	0.760	0.021310	2.2667	0	-2	18	1.5278	-2	1	0	0
HIP_96104	YLVCGERGF	QVRGYPEEK	YLVCGERGFQVRGYPEEK		CMGA	28545/247050	0.926	96104	0.760	0.011642	-0.9833	0.1667	0	18	-0.8222	0	1	0	0
HIP_96105	EDLQVGQVE	FQRNQLLQK	EDLQVGQVEFQRNQLLQK		SCG2	44008/334890	0.966	96105	0.760	-0.108231	-1.3333	0	-1	18	-0.9833	-1	1	0	0
HIP_96106	LQVGQVEL	LEGEEENEL	LQVGQVELLEGEEENEL		SCG1	20802/367830	0.988	96106	0.760	-0.018491	0.7333	0.1667	-6	17	-0.5118	-6	0	1	1
HIP_96107	GSHLVEALY	DNMNDNFL	GSHLVEALYDNMNDNFL		SCG1	31004/367830	0.962	96107	0.760	0.096910	-0.1333	0	-2	17	-0.2706	-3	1	1	0
HIP_96108	LVEALYLVC	EKRKCNTAT	LVEALYLVCEKRKCNTAT		IAPP	6113/45018	0.879	96108	0.760	0.214373	-0.2333	0	1	18	0.1611	1	1	0	0
HIP_96109	QVELGGGPG	ELFNPYYDP	QVELGGGPGELFNPYYDP		SCG1	16219/367830	0.994	96109	0.760	0.121236	0.1167	0.5	-3	18	-0.6611	-3	1	0	1
HIP_96110	QHLCGSHLV	KEIRKGESR	QHLCGSHLVKEIRKGESR		CMGA	37987/247050	0.824	96110	0.760	0.205705	0.3167	0	4	18	-0.9611	2	0	0	0
HIP_96111	VCGERGFF	LLLCAGQVT	VCGERGFFLLLCAGQVT		CMGA	17449/247050	0.981	96111	0.760	0.013873	2.7667	0.1667	0	17	1.1059	0	2	1	1
HIP_96112	YQLENYCN	EDQELESLS	YQLENYCNEDQELESLS		CMGA	22411/247050	0.963	96112	0.760	0.030520	-2.1333	0	-5	17	-1.2824	-5	1	1	0
HIP_96113	VEQCCTSIC	EAQDPEGKG	VEQCCTSICEAQDPEGKG		CMGA	18922/247050	0.976	96113	0.760	-0.082062	0.1667	0.1667	-3	18	-0.6	-3	0	0	0
HIP_96114	GIVEQCCTS	DSQVSEEVK	GIVEQCCTSDSQVSEEVK		SCG1	27844/367830	0.973	96114	0.760	-0.095716	-1.1333	0.3333	-3	18	-0.35	-3	0	0	0
HIP_96115	NQHLCGSHL	PTKADTEKW	NQHLCGSHLPTKADTEKW		SCG1	44052/367830	0.894	96115	0.760	0.115910	-1.0667	0.1667	2	18	-1.1889	0	0	0	0

[illegible]

HIP_96569	GSHLVEAL	EEEEEEEEA	GSHLVEALEEEEEEEEA		CMGA	20142/247050	0.971	96569	0.760	-0.009493	-1.4	0	-8	17	-1.2059	-9	0	1	[illegible]
HIP_96570	GSHLVEAL	EEEEEEEEA	GSHLVEALEEEEEEEEA		CMGA	20143/247050	0.971	96570	0.760	-0.009493	-1.4	0	-8	17	-1.2059	-9	0	1	[illegible]
HIP_96571	GSLQPLAL	GRLQNGQN	GSLQPLALGRLQNGQN		SCG2	36395/334890	0.983	96571	0.760	0.225131	1.3833	0.1667	1	16	-0.5375	1	0	0	[illegible]
HIP_96572	PLALEGSLQ	KWQQQGDLQ	PLALEGSLQKWQQQGDLQ		SCG1	48898/367830	0.860	96572	0.760	-0.039833	-1.4667	0.1667	-1	18	-0.8611	-1	1	0	[illegible]
HIP_96573	HLVEALY	FLVHSSNNF	HLVEALYFLVHSSNNF		IAPP	4389/45018	0.947	96573	0.760	-0.025890	2.5167	0	1	16	0.4625	-1	2	2	[illegible]
HIP_96574	HLVEALYLV	NIEKNEQI	HLVEALYLVNIEKNEQI		SCG2	30464/334890	0.990	96574	0.760	0.147750	0.7	0	-1	17	0.0706	-2	1	1	[illegible]
HIP_96575	PLALEGSLQ	RALEYIENL	PLALEGSLQRALEYIENL		SCG2	25076/334890	0.994	96575	0.760	-0.022710	0.1	0.1667	-2	18	0.0556	-2	0	0	[illegible]
HIP_96576	PLALEGSLQ	RTNEIVEEQ	PLALEGSLQRTNEIVEEQ		SCG2	37781/334890	0.981	96576	0.760	-0.089382	-1.5333	0.1667	-3	18	-0.5889	-3	0	0	[illegible]
HIP_96577	VEALYLVC	EESNVSMA	VEALYLVCEESNVSMA		SCG1	31558/367830	0.959	96577	0.760	0.154848	0.45	0.1667	-3	16	0.7063	-3	1	0	[illegible]
HIP_96578	QHLCGSHLV	LQKIAEKFS	QHLCGSHLVLQKIAEKFS		SCG1	46724/367830	0.876	96578	0.760	0.115853	0.2	0	3	18	0.0278	1	0	0	[illegible]
HIP_96579	LYLVCGERG	ENRQMAYEN	LYLVCGERGENRQMAYEN		SCG2	44146/334890	0.966	96579	0.760	-0.066232	-1.3167	0.1667	-1	18	-0.8556	-1	0	0	[illegible]
HIP_96580	VCGERGFFY	REKSSQES	VCGERGFFYREKSSQES		SCG1	50051/367830	0.851	96580	0.760	0.209066	-1.2667	0	0	17	-1.1235	0	3	1	[illegible]
HIP_96581	VEQCCTSIC	EFPDFYDSE	VEQCCTSICEFPDFYDSE		SCG1	20019/367830	0.989	96581	0.760	-0.067290	0.65	0.3333	-5	18	-0.2444	-5	1	0	[illegible]
HIP_96582	GGPGAGSLQ	DLIEMLKTG	GGPGAGSLQDLIEMLKTG		SCG2	27929/334890	0.992	96582	0.760	0.009847	0.7167	0.1667	-1	18	0.0056	-1	0	0	[illegible]
HIP_96583	CGERGFFY	DDIYKANN	CGERGFFYDDIYKANN		SCG2	37139/334890	0.982	96583	0.760	0.114742	0.3	0	-1	16	-0.9312	-1	4	0	[illegible]
HIP_96584	GPGAGSLQP	KVIAYLKRL	GPGAGSLQPKVIAYLKRL		SCG2	35947/334890	0.984	96584	0.760	0.080486	0.5833	0.1667	3	18	0.0778	3	0	0	[illegible]
HIP_96585	YLVCGERGF	NDKDQELGE	YLVCGERGFNDKDQELGE		SCG2	35062/334890	0.985	96585	0.760	-0.006416	-2.1667	0.1667	-3	18	-1.0167	-3	1	0	[illegible]
HIP_96586	LVEALYLVC	YLKRLVNA	LVEALYLVCYLKRLVNA		SCG2	43459/334890	0.968	96586	0.760	0.102832	1.5167	0	1	17	1.1588	1	2	1	[illegible]
HIP_96587	LQPLALEGS	LFQNRMLSK	LQPLALEGSLFQNRMLSK		SCG2	20909/334890	0.996	96587	0.760	0.112048	-0.2667	0.3333	1	18	-0.0278	1	1	0	[illegible]
HIP_96588	VEQCCTSIC	NGDISHLPE	VEQCCTSICNGDISHLPE		SCG2	18208/334890	0.997	96588	0.760	0.068618	-0.2	0.3333	-3	18	-0.4722	-4	0	0	[illegible]
HIP_96589	CGSHLVEAL	AEDMARYYS	CGSHLVEALAEDMARYYS		NPY	5641/49410	0.913	96589	0.760	-0.030218	-0.5167	0	-1	18	-0.0667	-2	0	0	[illegible]
HIP_96590	CGSHLVEAL	PSDSQVSEE	CGSHLVEALPSDSQVSEE		SCG1	24715/367830	0.981	96590	0.760	0.115407	-0.6333	0.3333	-3	18	-0.3111	-4	0	0	[illegible]
HIP_96591	ICSLYQLEN	RGRGGEPRA	ICSLYQLENRGRGGEPRA		SCG1	54696/367830	0.811	96591	0.760	0.122202	-2.1	0.1667	1	18	-0.8889	1	1	0	[illegible]
HIP_96592	ELGGGPGAG	SEDVNWGYE	ELGGGPGAGSEDVNWGYE		SCG1	20115/367830	0.989	96592	0.760	-0.156364	-1.1333	0.5	-4	18	-0.8167	-4	0	0	[illegible]
HIP_96593	TSICSLYQL	ATPIESHQV	TSICSLYQLATPIESHQV		IAPP	3108/45018	0.976	96593	0.760	-0.066537	-0.25	0.1667	0	18	0.2611	-1	1	0	[illegible]
HIP_96594	SICSLYQL	IPLIFLISG	SICSLYQLIPLIFLISG		SCG2	26801/334890	0.993	96594	0.760	-0.095636	0.95	0.1667	0	17	1.7235	0	2	1	[illegible]
HIP_96595	VELGGGPGA	PQSEESWDE	VELGGGPGAPQSEESWDE		SCG1	16978/367830	0.993	96595	0.760	-0.117674	-1.0167	0.6667	-5	18	-1.0278	-5	0	0	[illegible]
HIP_96596	SHLVEALYL	GHSQEKTH	SHLVEALYLGHSQEKTH		SCG1	36977/367830	0.935	96596	0.760	0.244425	0.3167	0.3333	2	17	-0.6235	-1	1	1	[illegible]
HIP_96597	LYQLENYCN	YDSEEPVS	LYQLENYCNYDSEEPVS		SCG1	31949/367830	0.958	96597	0.760	-0.027647	-1.3167	0.1667	-4	17	-1.0176	-4	2	1	[illegible]
HIP_96598	GGGPGAGS	EDSKRWSKM	GGGPGAGSEDSKRWSKM		CMGA	27082/247050	0.937	96598	0.760	0.003360	-1.2	0.5	1	17	-1.3235	1	0	1	[illegible]
HIP_96599	VEALYLVCG	SHFERRDN	VEALYLVCGSHFERRDN		SCG1	35486/367830	0.942	96599	0.760	0.012195	0.85	0.3333	0	17	-0.3294	-1	2	1	[illegible]
HIP_96600	CCTSICSLY	RDHHSTHYR	CCTSICSLYRDHHSTHYR		SCG1	57472/367830	0.785	96600	0.760	0.042085	-1.5833	0.1667	4	18	-0.7056	1	1	0	[illegible]
HIP_96601	RGFFYTPKT	DRNYLNYG	RGFFYTPKTDRNYLNYG		SCG1	53630/367830	0.821	96601	0.760	0.037194	-2.95	0.1667	2	17	-1.2765	2	2	1	[illegible]
HIP_96602	GSLQPLAL	SLELDKM	GSLQPLALSLELDKM		SCG1	31506/367830	0.960	96602	0.760	0.156566	1.4833	0.1667	-1	15	0.3133	-1	0	1	[illegible]
HIP_96603	EAEDLQVGQ	REDSKEAEK	EAEDLQVGQREDSKEAEK		CMGA	23260/247050	0.959	96603	0.760	-0.115356	-1.8667	0.1667	-4	18	-1.8556	-4	0	0	[illegible]
HIP_96604	LVEALYLVC	ENLRQQAH	LVEALYLVCENLRQQAH		SCG2	33509/334890	0.987	96604	0.760	0.145168	1.2167	0	0	17	0.1882	-1	1	1	[illegible]
HIP_96605	VEALYLVCG	EALAVQGAG	VEALYLVCGEALAVQGAG		CMGA	13853/247050	0.989	96605	0.760	-0.069512	1.4	0.1667	-3	18	1.15	-3	1	0	[illegible]
HIP_96606	YQLENYCN	TPNRQYWDE	YQLENYCNTPNRQYWDE		SCG2	28286/334890	0.991	96606	0.760	0.088503	-1.35	0.1667	-2	17	-1.9588	-2	1	1	[illegible]
HIP_96607	CGERGFFY	MNKGDTEVM	CGERGFFYMNKGDTEVM		CMGA	31236/247050	0.903	96607	0.760	0.148993	-0.2	0	-1	17	-0.5588	-1	2	1	[illegible]
HIP_96608	DLQVGQVEL	DTLSKPSPM	DLQVGQVELDTLSKPSPM		CMGA	28294/247050	0.929	96608	0.760	0.051953	0.6833	0	-2	18	-0.3111	-2	0	0	[illegible]
HIP_96609	ALYLVCGER	SSPDYNPYQ	ALYLVCGERSSPDYNPYQ		SCG2	29095/334890	0.991	96609	0.760	-0.140642	-1.9333	0.6667	-1	18	-0.6389	-1	0	0	[illegible]
HIP_96610	QCCTSICS	DTEKWAEGG	QCCTSICSDTEKWAEGG		SCG1	37233/367830	0.934	96610	0.760	0.096965	-0.25	0.1667	-2	17	-0.5176	-2	1	1	[illegible]
HIP_96611	CGERGFFY	DLIEMLKT	CGERGFFYDLIEMLKT		SCG2	33526/334890	0.987	96611	0.760	0.010261	1.5167	0	-1	16	0.025	-1	2	0	[illegible]
HIP_96612	VCGERGFF	KEEEGSAN	VCGERGFFKEEEGSAN		CMGA	27566/247050	0.934	96612	0.760	0.086954	-0.95	0.1667	-2	16	-0.8625	-2	2	0	[illegible]
HIP_96613	LQVGQVELG	REEARFQDK	LQVGQVELGREEARFQDK		SCG1	51634/367830	0.838	96613	0.760	-0.072302	-1.9333	0.1667	-1	18	-0.9778	-1	0	0	[illegible]
HIP_96614	LYQLENYCN	CRQVLKTSR	LYQLENYCNCRQVLKTSR		SCG1	54317/367830	0.815	96614	0.760	-0.016042	-1.3	0	2	18	-0.7722	2	1	0	[illegible]
HIP_96615	PLALEGSLQ	EKSSQESGE	PLALEGSLQEKSSQESGE		SCG1	38553/367830	0.926	96615	0.760	-0.079979	-1.45	0.3333	-3	18	-0.9611	-3	0	0	[illegible]
HIP_96616	LQPLALEGS	RGSEEYRAP	LQPLALEGSRGSEEYRAP		SCG1	33895/367830	0.950	96616	0.760	-0.099589	-1.7333	0.6667	-1	18	-0.8278	-1	0	0	[illegible]
HIP_96617	EAEDLQVGQ	WDEEDKRNY	EAEDLQVGQWDEEDKRNY		SCG1	27794/367830	0.973	96617	0.760	-0.109520	-1.2667	0.1667	-5	18	-2.0111	-5	1	0	[illegible]
HIP_96618	LYQLENYCN	GLTLALSLL	LYQLENYCNGLTLALSLL		NPY	2573/49410	0.981	96618	0.760	-0.096162	0.0667	0.1667	-1	18	0.6889	-1	1	0	[illegible]
HIP_96619	NQHLCGSHL	LGMESAANQ	NQHLCGSHLLGMESAANQ		SCG2	23944/334890	0.994	96619	0.760	-0.081945	0.85	0.3333	1	18	-0.3833	-1	0	0	[illegible]
HIP_96620	QVELGGGPG	DSKEAEKSG	QVELGGGPGDSKEAEKSG		CMGA	17454/247050	0.981	96620	0.760	-0.069329	-1.7667	0.6667	-2	18	-1.15	-2	0	0	[illegible]
HIP_96621	YQLENYCN	EEGSANRR	YQLENYCNEEGSANRR		CMGA	22707/247050	0.962	96621	0.760	0.132726	-1.6167	0.1667	-1	16	-1.825	-1	1	0	[illegible]
HIP_96622	SLYQLENYC	GEAGAPGEE	SLYQLENYCGEAGAPGEE		SCG1	17458/367830	0.993	96622	0.760	-0.084614	-0.7333	0.1667	-4	18	-0.75	-4	1	0	[illegible]

HIP_97080	VCGERGFF	DQLLHYRK	VCGERGFFDQLLHYRK		SCG1	41092/367830	0.912	97080	0.759	0.043253	0.3333	0.1667	2	16	-0.55	1	2	0
HIP_97081	GSHLVEALY	QVLKTSRK	GSHLVEALYQVLKTSRK		SCG1	44399/367830	0.892	97081	0.759	0.044425	1.4667	0	3	17	-0.2882	2	1	1
HIP_97082	ERGFFYT	AILSSTNVG	ERGFFYTAILSSTNVG		IAPP	4132/45018	0.954	97082	0.759	-0.041214	1.8167	0	0	16	0.2062	0	3	2
HIP_97083	LVEALYLVC	QKTSYFPN	LVEALYLVCQKTSYFPN		SCG2	41073/334890	0.975	97083	0.759	0.213818	0.4	0	0	17	0.4	0	1	1
HIP_97084	PLALEGSLQ	QALPEPMQE	PLALEGSLQQALPEPMQE		CMGA	13947/247050	0.989	97084	0.759	-0.056986	0.2667	0.1667	-3	18	-0.35	-3	0	0
HIP_97085	EALYLVCGE	SKENIEKNE	EALYLVCGESKENIEKNE		SCG2	36872/334890	0.982	97085	0.759	-0.102615	-1.6	0.3333	-3	18	-0.7889	-3	0	0
HIP_97086	LCGSHLVEA	RPDRSSQGG	LCGSHLVEARPDRSSQGG		SCG1	51973/367830	0.835	97086	0.759	0.226323	-1.1833	0.1667	1	18	-0.6556	0	0	0
HIP_97087	CGERGFFY	LDQLLHYR	CGERGFFYLDQLLHYR		SCG1	40661/367830	0.914	97087	0.759	0.105311	0.1833	0	1	16	-0.4125	0	3	0
HIP_97088	GSLQPLAL	EAENEKD	GSLQPLALEAENEKD		SCG1	33457/367830	0.951	97088	0.759	0.094507	0.7	0	-3	15	-0.8467	-3	0	1
HIP_97089	AEDLQVGQV	PMYEENSRD	AEDLQVGQVPMYEENSRD		SCG2	11112/334890	0.999	97089	0.759	-0.090747	-0.1167	0.3333	-4	18	-1.15	-4	1	0
HIP_97090	SICSLYQL	WKSSHFER	SICSLYQLWKSSHFER		SCG1	53124/367830	0.825	97090	0.759	0.146854	-1.1	0.1667	2	16	-0.4125	1	2	0
HIP_97091	SHLVEALYL	ENRQMAYE	SHLVEALYLENRQMAYE		SCG2	40332/334890	0.976	97091	0.759	0.243092	-0.8667	0	-1	17	-0.4412	-2	1	1
HIP_97092	VEALYLVC	NRMLSKSGY	VEALYLVCNRMLSKSGY		SCG2	46911/334890	0.956	97092	0.759	0.207233	0.7333	0	1	17	0.3529	1	1	1
HIP_97093	GAGSLQPLA	RNQLLQKEP	GAGSLQPLARNQLLQKEP		SCG2	42313/334890	0.972	97093	0.759	0.047576	-1.25	0.1667	1	18	-0.6611	1	0	0
HIP_97094	LCGSHLVEA	EKSGEATDG	LCGSHLVEAEKSGEATDG		CMGA	30373/247050	0.911	97094	0.759	0.189043	-0.95	0.1667	-2	18	-0.3722	-3	0	0
HIP_97095	GERGFFYT	AVLALLLCA	GERGFFYTAVLALLLCA		CMGA	21537/247050	0.966	97095	0.759	-0.046127	1.7667	0	0	17	1.3	0	3	1
HIP_97096	LVCGERGF	EGQEEEEDN	LVCGERGFEGQEEEEDN		CMGA	15462/247050	0.986	97096	0.759	-0.076934	-1.5833	0.3333	-6	17	-1.4059	-6	1	1
HIP_97097	VEALYLVCG	LSFRARAY	VEALYLVCGLSFRARAY		CMGA	19634/247050	0.973	97097	0.759	-0.015304	2.0167	0.3333	1	17	0.8353	1	2	1
HIP_97098	GERGFFYT	QDPEGKGE	GERGFFYTQDPEGKGE		CMGA	25960/247050	0.944	97098	0.759	0.126857	-1.3	0.1667	-2	16	-1.5937	-2	3	0
HIP_97099	QCCTSICSL	EKGHPQEES	QCCTSICSLEKGHPQEES		SCG1	34467/367830	0.947	97099	0.759	0.192549	-0.3833	0.3333	-1	18	-0.7722	-2	0	0
HIP_97100	SLQPLAL	DGARPQALP	SLQPLALDGARPQALP		CMGA	21608/247050	0.966	97100	0.759	0.202753	1.2167	0.1667	0	16	-0.025	0	0	2
HIP_97101	GAGSLQPLA	GYEKRNLA	GAGSLQPLAGYEKRNLA		SCG1	32744/367830	0.954	97101	0.759	0.051031	-0.2	0.3333	1	17	-0.4118	1	1	1
HIP_97102	SLQPLALEG	EEEDNRDS	SLQPLALEGEEEDNRDS		CMGA	22979/247050	0.960	97102	0.759	-0.091074	-1.7667	0.1667	-5	17	-1.3471	-5	0	1
HIP_97103	VGQVELGGG	SSEDDLQEE	VGQVELGGGSSEDDLQEE		SCG2	14716/334890	0.998	97103	0.759	-0.109877	-1.05	0.8333	-6	18	-0.8444	-6	0	0
HIP_97104	GGPGAGSLQ	LALEGSL	GGPGAGSLQLALEGSL		INSC	369/5184	0.995	97104	0.759	0.126276	1.4833	0.1667	-1	16	0.4125	-1	0	2
HIP_97105	LVEALYLVC	IQEEDLRK	LVEALYLVCIQEEDLRK		SCG2	30289/334890	0.990	97105	0.759	0.093419	1.3333	0	-2	17	0.3059	-2	1	1
HIP_97106	VCGERGFF	AEGDSEGLS	VCGERGFFAEGDSEGLS		CMGA	16876/247050	0.982	97106	0.759	0.013469	0.5167	0.3333	-3	17	-0.2235	-3	2	1
HIP_97107	AEDLQVGQV	KRNLARVPK	AEDLQVGQVKRNLARVPK		SCG1	56981/367830	0.790	97107	0.759	0.021847	-1.9333	0.1667	2	18	-0.6944	2	0	0
HIP_97108	HLVEALYL	LDRNYLNY	HLVEALYLLDRNYLNY		SCG1	45590/367830	0.884	97108	0.759	0.199615	0.35	0	0	16	-0.0375	-1	1	0
HIP_97109	CCTSICSLY	LGELFNPYY	CCTSICSLYLGELFNPYY		SCG1	24581/367830	0.981	97109	0.759	-0.094492	0.2667	0.3333	-1	18	0.6111	-1	1	0
HIP_97110	SLYQLENYC	ELGEYLARM	SLYQLENYCELGEYLARM		SCG2	19693/334890	0.996	97110	0.759	-0.058460	-0.4	0.1667	-2	18	-0.3167	-2	1	0
HIP_97111	CCTSICSLY	RLEGQEEEE	CCTSICSLYRLEGQEEEE		CMGA	21501/247050	0.967	97111	0.759	-0.098743	-0.4167	0.1667	-4	18	-0.55	-4	1	0
HIP_97112	SICSLYQL	EGSANRRPE	SICSLYQLEGSANRRPE		CMGA	20321/247050	0.971	97112	0.759	0.138660	-0.95	0.3333	0	17	-0.7235	0	1	1
HIP_97113	LYLVCGERG	GHSQEKTHS	LYLVCGERGGHSQEKTHS		SCG1	39803/367830	0.920	97113	0.759	-0.060001	-2.1333	0.5	2	18	-0.8778	0	0	0
HIP_97114	GERGFFYT	PQAEGDSEG	GERGFFYTPQAEGDSEG		CMGA	20946/247050	0.969	97114	0.759	0.005397	-0.4167	0.1667	-3	17	-1.2118	-3	3	1
HIP_97115	VCGERGFF	EDQELESL	VCGERGFFEDQELESL		CMGA	23547/247050	0.958	97115	0.759	0.049324	-0.8833	0.1667	-4	16	-0.45	-4	2	0
HIP_97116	VNQHLCGSH	KSSAPPITP	VNQHLCGSHKSSAPPITP		SCG1	42970/367830	0.901	97116	0.759	-0.064457	-1.65	0.6667	3	18	-0.4889	1	0	0
HIP_97117	LCGSHLVEA	QAEGDSEGL	LCGSHLVEAQAEGDSEGL		CMGA	26798/247050	0.939	97117	0.759	0.021331	-0.45	0	-3	18	-0.1	-4	0	0
HIP_97118	LYLVCGERG	QECFETLRG	LYLVCGERGQECFETLRG		CMGA	24959/247050	0.951	97118	0.759	-0.048362	-2.15	0.1667	-1	18	-0.1556	-1	1	0
HIP_97119	VNQHLCGSH	SRKDVKDKE	VNQHLCGSHSRKDVKDKE		SCG1	54349/367830	0.814	97119	0.759	-0.022122	-2.2667	0.5	3	18	-1.5222	1	0	0
HIP_97120	HLCGSHLVE	YDRVAQLDQ	HLCGSHLVEYDRVAQLDQ		SCG1	54150/367830	0.816	97120	0.759	0.058207	-0.8	0	0	18	-0.3778	-2	1	0
HIP_97121	VEALYLVC	NLRQQAHKE	VEALYLVCNLRQQAHKE		SCG2	43861/334890	0.967	97121	0.759	0.185268	1.05	0	1	17	-0.2647	0	1	1
HIP_97122	GPGAGSLQP	DKLAPVSKR	GPGAGSLQPDKLAPVSKR		SCG2	50081/334890	0.942	97122	0.759	0.018097	-0.8167	0.1667	2	18	-0.6389	2	0	0
HIP_97123	VNQHLCGSH	EEKIESQTQ	VNQHLCGSHEEKIESQTQ		SCG2	40550/334890	0.976	97123	0.759	-0.116287	-2.55	0.3333	0	18	-1.25	-2	0	0
HIP_97124	GIVEQCCTS	NVPRTRLED	GIVEQCCTSNVPRTRLED		NPY	3815/49410	0.961	97124	0.759	-0.089003	0.0167	0.3333	-1	18	-0.5	-1	0	0
HIP_97125	CTSICSLYQ	YPGVQAPE	CTSICSLYQYPGVQAPE		SCG1	20971/367830	0.988	97125	0.759	0.000178	-0.7167	0.3333	-1	17	0.0176	-1	2	1
HIP_97126	SICSLYQLE	DSLEAGLPL	SICSLYQLEDSLEAGLPL		CMGA	21650/247050	0.966	97126	0.759	-0.181575	-0.6167	0.1667	-3	18	0.45	-3	1	0
HIP_97127	LYQLENYCN	SSHFERRD	LYQLENYCNSSHFERRD		SCG1	38947/367830	0.924	97127	0.759	0.013696	-1.1833	0.3333	0	17	-1.4412	-1	2	1
HIP_97128	CCTSICSLY	LDLKRQYDR	CCTSICSLYLDLKRQYDR		SCG1	38961/367830	0.924	97128	0.759	-0.046669	0.9667	0.1667	1	18	-0.2722	1	1	0
HIP_97129	GERGFFY	AEAGEEA	GERGFFYAEAGEEA		CMGA	23851/247050	0.957	97129	0.759	0.019287	0.7333	0	-3	14	-0.7143	-3	3	0
HIP_97130	HLVEALYLV	PGKGRHHR	HLVEALYLVPGKGRHHR		SCG1	40426/367830	0.916	97130	0.759	0.237844	0.1333	0.3333	5	17	-0.4765	2	1	1
HIP_97131	GSLQPLALE	EEEEAEAG	GSLQPLALEEEEEAEAG		CMGA	22430/247050	0.963	97131	0.759	0.034544	-1.4	0	-6	17	-0.6412	-6	0	1
HIP_97132	TSICSLYQL	SQSIYQLI	TSICSLYQLSQSIYQLI		SCG2	31067/334890	0.989	97132	0.759	0.168894	-1.0167	0.3333	0	17	0.6118	0	1	1
HIP_97133	GERGFFYT	KRNLARVPK	GERGFFYTKRNLARVPK		SCG1	57251/367830	0.787	97133	0.759	0.148511	-1.85	0	4	17	-1.0176	4	3	1
HIP_97134	CGERGFF	LIVLSVALN	CGERGFFLIVLSVALN		IAPP	3278/45018	0.973	97134	0.759	0.016755	2.95	0.1667	0	16	1.3187	0	2	2
HIP_97135	LQVGQVELG	ITRQRYGKR	LQVGQVELGITRQRYGKR		NPY	6449/49410	0.876	97135	0.759	-0.022664	-0.1333	0.1667	3	18	-0.7833	3	0	0
HIP_97136	LGGGPGAGS	VCGERGFF	LGGGPGAGSVCGERGFF		INSB	184/4968	0.978	97136	0.759	-0.013186	1.15	0.5	0	17	0.2765	0	0	1
HIP_97137	YLVCGERGF	EHLNQGSSQ	YLVCGERGFEHLNQGSSQ		SCG2	31873/334890	0.988	97137	0.759	0.067185	-0.8333	0.1667	0	18	-0.6778	-1	1	0

HIP_97591	GERGFFYT	RSGQLGIQE	GERGFFYTRSGQLGIQE		SCG2	33940/334890	0.986	97591	0.759	0.155731	-0.8167	0.3333	0	17	-0.7941	0	3	1	0	0.000000	-2.1667	1	0.15	1.650000	0.1111	1.5389	0	1	0	1	-2.1667	-1.2568	-6.5	0	1	0	0	0.4936	0.8955	1.0123	0	0.2436	1.0248	-0.4031	0.0225	-0.325
HIP_97592	PLALEGSLQ	ESWDEEDKR	PLALEGSLQESWDEEDKR		SCG1	28811/367830	0.970	97592	0.759	-0.143007	-0.95	0.3333	-4	18	-1.3444	-4	1	0	0	0.000000	-1.5667	-1	0	0.950000	0.3333	0.6167	0	0	0	0	1.5667	-0.5858	-1.5667	0	1	0	0	0.5051	1.0903	0.9212	0.1667	0.2552	0.8485	-0.4799	0	0
HIP_97593	QCCTSICSL	EKSSQESGE	QCCTSICSLEKSSQESGE		SCG1	39936/367830	0.919	97593	0.759	0.188791	-0.45	0.3333	-2	18	-0.5944	-2	0	0	0	0.000000	-4.5667	0	0.1	0.000000	0.2222	-0.2222	0	0	0	0	0	0.1	0	0	1	0	0	0.4769	1.0755	0.909	-0.1667	0.2275	0.8262	-0.2146	0.01	-0.4567
HIP_97594	GGPGAGSLQ	RPRHHHGRS	GGPGAGSLQRPRHHHGRS		SCG1	59067/367830	0.766	97594	0.759	0.050241	-1.85	0.3333	6	18	-1.5444	3	0	0	1	0.000000	-3.3667	2	0.05	1.400000	0.2222	1.1778	1	0	1	0	-6.7333	-2.1789	0	0	1	0	0	0.4936	0.9705	1.0023	-0.1667	0.2436	1.0046	-0.9131	0.0025	-0.1683
HIP_97595	LYQLENYCN	PVSQECFET	LYQLENYCNPVSQECFET		CMGA	11430/247050	0.993	97595	0.759	-0.103559	-0.0833	0.3333	-3	18	-0.5889	-3	1	0	0	0.000000	1.3667	0	0.0833	0.625000	0.1111	0.5139	1	0	1	0	0	-0.0428	1.3667	0	1	0	0	0.4936	0.8572	1.099	0.1667	0.2436	1.2077	-0.0411	0.0069	0.1139
HIP_97596	GIVEQCCTS	QDKQYSSHH	GIVEQCCTSQDKQYSSHH		SCG1	58475/367830	0.774	97596	0.759	0.053780	-1.65	0.1667	1	18	-1.05	-1	0	0	0	0.000000	-3.9667	0	0.0333	0.000000	0.2222	-0.2222	0	0	0	0	0	0.3667	0	0	1	0	0	0.5154	0.9674	0.9768	-0.3333	0.2656	0.9541	-0.8504	0.0011	-0.1322
HIP_97597	VCGERGFF	SRSEALAVD	VCGERGFFSRSEALAVD		CMGA	26119/247050	0.943	97597	0.759	0.128155	-0.15	0.5	-1	17	0.1176	-1	2	1	0	0.000000	-3.7667	1	0.1	1.150000	0.3333	0.8167	0	1	0	1	-3.7667	-0.1225	-7.5333	1	0	0	0	0.4551	0.9203	1.0012	0.1667	0.2071	1.0023	-0.0683	0.01	-0.3767
HIP_97598	SHLVEALYL	GREHIAKR	SHLVEALYLGREHIAKR		SCG2	39243/334890	0.978	97598	0.759	0.268098	-0.35	0.1667	3	17	-0.3	1	1	1	0	0.000000	-4.9	0	0.1833	0.000000	0.3333	-0.3333	0	1	0	1	0	0.1167	-4.9	1	0	0	0	0.4269	1.1088	0.8423	0	0.1823	0.7095	-0.1494	0.0136	-0.8883
HIP_97599	CGERGFF	SALRHYI	CGERGFFSALRHYI		NPY	4468/49410	0.946	97599	0.759	0.014865	1.6667	0.3333	2	14	-0.0286	1	2	0	0	0.000000	-0.1333	0	-0.0167	0.700000	0.3333	0.3667	0	1	0	1	0	0.6111	-0.2667	1	0	0	0	0.35	1.075	0.8433	0.6667	0.1225	0.7112	0.5833	0.0003	0.0022
HIP_97600	LVEALYL	APGEEEE	LVEALYLAPGEEEE		CMGA	11596/247050	0.993	97600	0.759	0.130167	1.0167	0.3333	-5	14	-0.1143	-5	1	0	2	1.000000	-2.1667	0	0.15	0.450000	0.2222	0.2278	1	1	1	1	0	0.2316	-2.1667	1	0	0	0	0.4051	1.0236	0.9312	0.5	0.1641	0.867	0.4119	0.0225	-0.325
HIP_97601	HLCGSHLV	QENHPQESK	HLCGSHLVQENHPQESK		SCG1	40571/367830	0.915	97601	0.759	0.208146	-0.95	0	2	17	-1.1941	-1	0	1	0	0.000000	-5.1	-1	0.15	1.050000	0.1111	0.9389	0	0	0	0	5.1	-0.8919	0	1	0	0	0	0.4385	1.1119	0.8645	0	0.1922	0.7473	-0.4165	0.0225	-0.765
HIP_97602	GFFYTPKT	FNPYYDPLQ	GFFYTPKTFNPYYDPLQ		SCG1	33743/367830	0.950	97602	0.759	-0.063319	-1.4167	0.3333	0	17	-0.7471	0	3	1	0	0.000000	1.3	-1	-0.05	0.000000	0.1111	-0.1111	2	0	1	0	-1.3	0.1574	3.9	0	1	0	0	0.5385	0.8103	1.1778	0	0.2899	1.3873	-0.7628	0.0025	-0.065
HIP_97603	QPLALEGSL	SSQAELKEA	QPLALEGSLSSQAELKEA		CMGA	11907/247050	0.992	97603	0.759	-0.136395	-0.4167	0.6667	-2	18	-0.2889	-2	0	0	0	0.000000	-2.5667	0	0.0333	1.400000	0.1111	1.2889	0	1	0	1	0	-0.537	0	1	0	0	0	0.55	0.885	1.0967	0.1667	0.3025	1.2027	-0.2292	0.0011	-0.0856
HIP_97604	DLQVGQVEL	KEIRKGESR	DLQVGQVELKEIRKGESR		CMGA	40218/247050	0.790	97604	0.759	0.116555	0.2667	0	0	18	-1.05	0	0	0	0	0.000000	-2.4667	1	0.05	0.000000	0.4444	-0.4444	0	1	0	1	-2.4667	-0.1185	0	1	0	0	0	0.3718	1.2469	0.6428	0	0.1382	0.4132	0.0991	0.0025	-0.1233
HIP_97605	AGSLQPLAL	ATRLFEKP	AGSLQPLALATRLFEKP		SCG2	42555/334890	0.971	97605	0.759	0.167149	1	0	1	17	0.1706	1	0	1	0	1.000000	-4.2667	1	0.1	1.850000	0.1111	1.7389	0	0	0	0	-4.2667	1.7389	0	1	0	0	0	0.3051	1.2253	0.6978	0.5	0.0931	0.487	0.3051	0.01	-0.4267
HIP_97606	CCTSICSLY	YDPLQWKS	CCTSICSLYYDPLQWKS		SCG1	38342/367830	0.928	97606	0.759	-0.022497	-0.7833	0.3333	0	17	0.0294	0	2	1	0	0.000000	-2.7	-1	0.05	3.025000	0.1111	2.9139	1	0	1	0	2.7	-2.2825	-5.4	0	1	0	0	0.4654	0.9558	0.9901	0	0.2166	0.9804	-0.3646	0.0025	-0.135
HIP_97607	VCGERGFF	SEVLENQSS	VCGERGFFSEVLENQSS		CMGA	18719/247050	0.977	97607	0.759	0.002130	0.85	0.3333	-2	17	-0.2882	-2	2	1	0	0.000000	-1.7667	-1	0.0333	0.700000	0.3333	0.3667	0	1	0	1	1.7667	0.3117	-3.5333	1	0	0	0	0.4167	1.045	0.8567	0.3333	0.1736	0.7339	0.3542	0.0011	-0.0589
HIP_97608	SLQPLAL	AMDLELQKI	SLQPLALAMDLELQKI		SCG1	37189/367830	0.934	97608	0.759	0.056036	1.6	0	-1	16	0.5438	-1	0	2	0	1.000000	-3.0667	-1	0.05	1.425000	0.2222	1.2028	0	0	0	0	3.0667	1.9244	0	1	0	0	0	0.2385	1.2869	0.6378	0.6667	0.0569	0.4068	0.3815	0.0025	-0.1533
HIP_97609	CTSICSLYQ	QGSSQETD	CTSICSLYQQGSSQETD		SCG2	23139/334890	0.995	97609	0.759	0.146980	-0.95	0.3333	-2	17	-0.6176	-2	1	1	0	0.000000	-1.2333	0	0.15	0.625000	0	0.625	0	1	0	1	0	-0.5938	-1.2333	0	1	0	0	0.5218	0.9736	1.0112	0.1667	0.2723	1.0224	-0.4957	0.0225	-0.185
HIP_97610	NQHLCGSHL	EKVLPRLPY	NQHLCGSHLEKVLPRLPY		SCG2	53762/334890	0.919	97610	0.759	0.017815	-0.5667	0.1667	3	18	-0.5056	1	0	0	0	0.000000	-1	0	0.0333	1.575000	0.2222	1.3528	0	0	0	0	0	-0.7666	0	1	0	0	0	0.4103	1.1188	0.7923	-0.1667	0.1683	0.6278	-0.2325	0.0011	-0.0333
HIP_97611	VEALYLVC	EEDWMRII	VEALYLVCEEDWMRII		SCG2	32646/334890	0.988	97611	0.759	0.136753	0	0	-3	16	0.6562	-3	2	0	0	0.000000	-7	-3	0.15	2.000000	0.3333	1.6667	0	0	0	0	21	0	-14	0	0	0	0	0.4103	1.2372	0.7156	-0.1667	0.1683	0.5121	0	0.0225	-1.05
HIP_97612	LQPLALEGS	EEEEEGENY	LQPLALEGSEEEEEGENY		SCG1	30158/367830	0.965	97612	0.759	-0.107765	-2.5333	0.3333	-7	18	-1.2667	-7	0	0	3	0.000000	-1.9333	-2	-0.05	0.450000	0.5556	-0.1056	0	1	0	1	3.8667	0.2674	0	0	1	0	0	0.6167	1.2233	0.9433	-0.6667	0.3803	0.8899	-1.5622	0.0025	0.0967
HIP_97613	QVGQVELGG	KNFPMDMSD	QVGQVELGGKNFPMDMSD		SCG2	23253/334890	0.995	97613	0.759	-0.057756	-0.2667	0.3333	-2	18	-0.5389	-2	1	0	0	0.000000	-2.5333	1	-0.0667	1.050000	0.2222	0.8278	0	2	0	1	-2.5333	-0.2207	-2.5333	0	0	0	1	0.4718	0.8653	1.0945	0.1667	0.2226	1.1979	-0.1258	0.0044	0.1689
HIP_97614	AEDLQVGQV	WDEDLLMKV	AEDLQVGQVWDEDLLMKV		SCG2	25710/334890	0.993	97614	0.759	-0.058899	-1.2667	0.1667	-4	18	-0.1111	-4	1	0	0	1.000000	-2.7333	-2	-0.0333	0.000000	0.3333	-0.3333	0	1	0	1	5.4667	0.4222	-2.7333	1	0	0	0	0.4936	1.0372	0.9223	0	0.2436	0.8507	-0.6252	0.0011	0.0911
HIP_97615	LVEALYLVC	KGEQEHSQ	LVEALYLVCKGEQEHSQ		CMGA	24422/247050	0.953	97615	0.759	0.217747	0.45	0.1667	-1	17	-0.1765	-2	1	1	0	0.000000	-6.1	0	0.1833	2.000000	0.2222	1.7778	0	1	0	1	0	0.8	-6.1	0	0	0	0	0.4269	1.0688	0.8423	0	0.1823	0.7095	0.1921	0.0136	-1.1183
HIP_97616	HLCGSHLVE	NEIVEEQYT	HLCGSHLVENEIVEEQYT		SCG2	30092/334890	0.990	97616	0.759	-0.061969	0.3333	0	-2	18	-0.4222	-4	0	0	0	0.000000	-2.3333	0	0.05	2.100000	0.2222	1.8778	0	0	0	0	0	0.6259	0	0	0	1	0	0.4	1.1883	0.7517	0.1667	0.16	0.565	0.1333	0.0025	-0.1167
HIP_97617	PGAGSLQPL	GERGEDSSE	PGAGSLQPLGERGEDSSE		SCG1	28082/367830	0.972	97617	0.759	-0.016563	-1.6167	0.3333	-3	18	-1.1	-3	0	0	0	0.000000	-2.3667	0	0.0667	0.000000	0.2222	-0.2222	1	1	1	1	0	0.3593	0	0	1	0	0	0.5385	1.0019	1.0245	-0.1667	0.2899	1.0496	-0.8705	0.0044	-0.1578
HIP_97618	LQVGQVELG	DEMKRSGQL	LQVGQVELGDEMKRSGQL		SCG2	35694/334890	0.984	97618	0.759	-0.090425	-0.8667	0.1667	-1	18	-0.5389	-1	0	0	0	0.000000	-1.6667	-1	-0.0333	0.950000	0.4444	0.5056	0	1	0	1	1.6667	-0.4381	0	0	0	0	1	0.4551	1.1736	0.8312	-0.1667	0.2071	0.6908	-0.3944	0.0011	0.0556
HIP_97619	VNQHLCGSH	ELQKIAEKF	VNQHLCGSHELQKIAEKF		SCG1	41191/367830	0.911	97619	0.759	-0.048017	-1.2667	0.3333	2	18	-0.5278	0	0	0	0	0.000000	0.4	-1	-0.05	0.950000	0.2222	0.7278	0	1	0	1	-0.4	-0.9219	0	0	0	1	0	0.5218	1.0403	0.9712	-0.1667	0.2723	0.9431	-0.6609	0.0025	-0.02
HIP_97620	HLVEALYLV	DRSSQGGS	HLVEALYLVDRSSQGGS		SCG1	41167/367830	0.911	97620	0.759	0.236906	-0.35	0.1667	0	17	-0.0647	-1	1	1	0	0.000000	-5.1667	0	0.1667	1.400000	0.2222	1.1778	0	0	0	0	0	-0.4122	-5.1667	1	0	0	0	0.3821	1.0374	0.8201	0	0.146	0.6726	-0.1337	0.0278	-0.8611
HIP_97621	LVCGERGF	ALAEAYPSK	LVCGERGFALAEAYPSK		NPY	3579/49410	0.966	97621	0.759	0.103239	0.8833	0.1667	0	17	0.1529	0	1	1	0	1.000000	3.1667	-1	-0.1333	2.100000	0.3333	1.7667	0	1	0	1	-3.1667	1.5606	3.1667	1	0	0	0	0.3218	1.1536	0.8045	0.5	0.1036	0.6472	0.2843	0.0178	-0.4222
HIP_97622	SICSLYQLE	RILSILRHQ	SICSLYQLERILSILRHQ		CMGA	26510/247050	0.941	97622	0.759	-0.023648	0.1	0	2	18	0.2667	1	1	0	0	0.000000	2.3333	2	-0.05	1.900000	0.2222	1.6778	0	0	0	0	4.6667	0.1678	2.3333	0	0	1	0	0.3718	1.2203	0.6828	0.1667	0.1382	0.4662	0.0372	0.0025	-0.1167
HIP_97623	IVEQCCTSI	LQVFLIVLS	IVEQCCTSILQVFLIVLS		IAPP	3142/45018	0.975	97623	0.759	-0.025480	1.25	0.1667	-1	18	1.8056	-1	1	0	0	1.000000	0.5	0	-0.0667	2.275000	0	2.275	0	0	0	0	0	2.8438	0.5	1	0	0	0	0.4	1.03	0.7683	0.5	0.16	0.5903	0.5	0.0044	-0.0333
HIP_97624	ELGGGPGAG	YPKTPGRAG	ELGGGPGAGYPKTPGRAG		SCG2	32287/334890	0.988	97624	0.759	-0.018874	-0.9667	0.5	1	18	-0.7833	1	1	0	1	0.000000	-2.6	1	-0.05	0.450000	0.1111	0.3389	1	2	1	1	-2.6	-0.3276	-2.6	0	0	0	1	0.5269	0.8438	1.1656	0	0.2776	1.3587	-0.5094	0.0025	0.13
HIP_97625	VEALYLVC	ELQDLALQG	VEALYLVCELQDLALQG		CMGA	21524/247050	0.966	97625	0.759	0.097325	1.2167	0	-3	17	0.8412	-3	1	1	0	0.000000	-4.5667	-1	0.1	2.525000	0.2222	2.3028	0	0	0	0	4.5667	2.8017	-4.5667	0	0	0	0	0.3718	1.2169	0.6878	0.3333	0.1382	0.4731	0.4524	0.01	-0.4567
HIP_97626	GGGPGAGS	SNTYGKRNA	GGGPGAGSSNTYGKRNA		IAPP	4941/45018	0.929	97626	0.759	0.016574	-0.7333	0.5	2	17	-1.1412	2	1	1	1	0.000000	-1.8667	0	0.0167	0.000000	0	0	0	1	0	1	0	0	-1.8667	0	1	0	0	0.55	0.8417	1.18	0.1667	0.3025	1.3924	-0.4033	0.0003	-0.0311
HIP_97627	GERGFFYTP	QEHSQQKE	GERGFFYTPQEHSQQKE		CMGA	31268/247050	0.902	97627	0.759	-0.011117	-2.3	0.1667	0	17	-1.8941	-1	3	1	0	0.000000	-2.2	-1	-0.0167	0.700000	0.1111	0.5889	1	0	1	0	2.2	-1.3544	-6.6	0	0	0	1	0.5603	1.0105	1.0023	-0.3333	0.3139	1.0046	-1.2886	0.0003	0.0367
HIP_97628	PLALEGSLQ	SIYQLIEIS	PLALEGSLQSIYQLIEIS		SCG2	16390/334890	0.997	97628	0.759	-0.105917	0.3167	0.3333	-2	18	0.6	-2	1	0	0	0.000000	0.9667	0	-0.05	0.950000	0.1111	0.8389	0	0	0	0	0	0.2656	0.9667	0	1	0	0	0.4385	1.0186	0.8762	0.3333	0.1922	0.7676	0.1388	0.0025	-0.0483
HIP_97629	QCCTSICS	YGFRGPGPQ	QCCTSICSYGFRGPGPQ		CMGA	25979/247050	0.944	97629	0.759	0.048480	1.2167	0.3333	1	17	-0.2765	1	2	1	0	0.000000	-1.7	0	0.0167	0.000000	0.1111	-0.1111	0	1	0	1	0	-0.1352	-3.4	0	1	0	0	0.4269	0.9372	0.9306	0.3333	0.1823	0.8661	0.5194	0.0003	-0.0283
HIP_97630	RGFFYTPKT	HYRKKSAEF	RGFFYTPKTHYRKKSAEF		SCG1	52094/367830	0.834	97630	0.759	0.129307	-2.5333	0.1667	5	18	-1.3333	4	2	0	0	0.000000	-0.9333	0	-0.1167	0.000000	0.3333	-0.3333	1	0	1	0	0	0.8444	-1.8667	0	1	0	0	0.5038	0.9144	1.0179	-0.5	0.2539	1.0362	-1.2764	0.0136	0.1089
HIP_97631	LVCGERGF	FLISGAEAA	LVCGERGFFLISGAEAA		SCG2	25045/334890	0.994	97631	0.759	0.051812	1.5	0.1667	-1	17	0.9588	-1	2	1	0	1.000000	4.4	-1	-0.1333	1.650000	0.2222	1.4278	0	1	0	1	-4.4	2.1417	8.8	1	0	0	0	0.3218	1.0503	0.7628	0.5	0.1036	0.5819	0.4827	0.0178	-0.5867
HIP_97632	HLVEALYL	LDLKRQYD	HLVEALYLLDLKRQYD		SCG1	39345/367830	0.922	97632	0.759	0.053658	1.7333	0	0	16	-0.2	-1	1	0	0	1.000000	-0.7333	-1	0.05	0.950000	0.3333	0.6167	0	0	0	0	0.7333	1.0689	-0.7333	1	0	0	0	0.2769	1.2072	0.6356	0.5	0.0767	0.404	0.48	0.0025	-0.0367
HIP_97633	GPGAGSLQP	GHSRERADE	GPGAGSLQPGHSRERADE		SCG1	29996/367830	0.965	97633	0.759	-0.065863	-0.95	0.5	0	18	-1.4	-1	0	0	0	0.000000	-1.0333	0	0.0333	0.000000	0.1111	-0.1111	1	1	1	1	0	0.1056	0	0	0	0	1	0.5385	0.8836	1.1012	0	0.2899	1.2125	-0.5115	0.0011	-0.0344
HIP_97634	HLVEALYL	ERLSKEWED	HLVEALYLERLSKEWED		CMGA	27376/247050	0.935	97634	0.759	0.150564	0.35	0	-2	17	-0.6412	-3	1	1	0	0.000000	-3.5	0	0.1	1.400000	0.3333	1.0667	0	0	0	0	0	0.3733	-3.5	1	0	0	0	0.3436	1.2422	0.6756	0.1667	0.1181	0.4565	0.1203	0.01	-0.35
HIP_97635	ERGFFYTP	WPERKLKH	ERGFFYTPWPERKLKH		SCG2	40764/334890	0.975	97635	0.759	-0.011552	-1.6	0.3333	3	16	-1.5062	2	4	0	0	0.000000	-0.8	-1	0	0.700000	0.2222	0.4778	2	0	1	0	0.8	-0.7644	-3.2	0	0	0	1	0.5769	0.9205	1.0923	0	0.3328	1.1931	-0.9231	0	0
HIP_97636	GPGAGSLQ	EEGSANRRP	GPGAGSLQEEGSANRRP		CMGA	25589/247050	0.947	97636	0.759	0.025778	-1.3167	0.3333	0	17	-1.2941	0	0	1	0	0.000000	-2.3	-2	0.0667	0.450000	0.2222	0.2278	0	1	0	1	4.6	-0.2999	0	0	1	0	0	0.55	1.1217	0.9433	-0.1667	0.3025	0.8899	-0.7242	0.0044	-0.1533
HIP_97637	VELGGGPGA	GGGHSRER	VELGGGPGAGGGHSRER		SCG1	20662/367830	0.988	97637	0.759	0.014335	-0.2333	0.8333	1	17	-0.8588	0	0	1	2	0.000000	-0.3333	0	0.1	0.000000	0	0	1	4	1	1	0	0	0	1	0	0	0	0.6167	0.67	1.3633	0.1667	0.3803	1.8587	-0.1439	0.01	-0.0333
HIP_97638	GPGAGSLQP	MLKTGEKPN	GPGAGSLQPMLKTGEKPN		SCG2	33012/334890	0.987	97638	0.759	0.048718	0.0833	0.1667	1	18	-0.8278	1	0	0	0	0.000000	1.0333	1	-0.0667	0.475000	0.1111	0.3639	1	0	1	0	1.0333	0.0303	0	0	0	0	1	0.3885	1.0836	0.8345	0.3333	0.1509	0.6964	0.0324	0.0044	-0.0689

HIP_98102	SICSLYQLE	LGEKRDHHS	SICSLYQLELGEKRDHHS		SCG1	37605/367830	0.932	98102	0.758	-0.151094	-0.55	0.1667	1	18	-0.8056	-1	1	0
HIP_98103	VNQHLCGSH	AYGFRGPGP	VNQHLCGSHAYGFRGPGP		CMGA	19720/247050	0.973	98103	0.758	-0.077508	-0.7167	0.5	3	18	-0.5389	1	2	0
HIP_98104	ERGFFYTPK	DEPQWSLY	ERGFFYTPKDEPQWSLY		SCG1	42299/367830	0.905	98104	0.758	-0.074703	-2.4667	0.3333	-1	17	-1.2706	-1	2	1
HIP_98105	SLYQLENYC	LLGMESAAN	SLYQLENYCLLGMESAAN		SCG2	18837/334890	0.996	98105	0.758	-0.161793	0.8167	0.1667	-2	18	0.0611	-2	1	0
HIP_98106	ERGFFYTP	IFLISGAEA	ERGFFYTPIFLISGAEA		SCG2	23968/334890	0.994	98106	0.758	0.009508	1.25	0.1667	-1	17	0.4765	-1	3	1
HIP_98107	GGPGAGSLQ	MRESTENVP	GGPGAGSLQMRESTENVP		NPY	6385/49410	0.879	98107	0.758	-0.141234	-1.1	0.1667	-1	18	-0.7722	-1	0	0
HIP_98108	CGERGFFYT	GAPGEEDI	CGERGFFYTGAPGEEDI		SCG1	16488/367830	0.994	98108	0.758	0.014554	0.1	0.3333	-3	17	-0.5471	-3	3	1
HIP_98109	HLVEALYLV	VGGEDWNP	HLVEALYLVVGGEDWNP		SCG2	18393/334890	0.997	98109	0.758	0.123520	1.6833	0.3333	-2	17	0.2353	-3	1	1
HIP_98110	GIVEQCCTS	SWDEEDKRN	GIVEQCCTSSWDEEDKRN		SCG1	32616/367830	0.955	98110	0.758	-0.072007	-0.7	0.3333	-3	18	-1.2667	-3	1	0
HIP_98111	QCCTSICS	TQNAFLNER	QCCTSICSTQNAFLNER		SCG1	43504/367830	0.898	98111	0.758	0.155999	-0.25	0.1667	0	17	-1.2706	0	1	1
HIP_98112	ERGFFYTP	LIVLSVALN	ERGFFYTPLIVLSVALN		IAPP	3356/45018	0.971	98112	0.758	0.019529	1.4833	0.1667	0	17	0.9059	0	2	1
HIP_98113	LVCGERGFF	RHSEKSQR	LVCGERGFFRHSEKSQR		SCG1	52915/367830	0.827	98113	0.758	0.102820	-0.55	0.3333	3	17	-1.0235	2	2	1
HIP_98114	SICSLYQLE	INDEMKRSG	SICSLYQLEINDEMKRSG		SCG2	39284/334890	0.978	98114	0.758	-0.147587	-0.95	0	-1	18	-0.5	-1	1	0
HIP_98115	VEQCCTSIC	RDNPFKRTN	VEQCCTSICRDNPFKRTN		SCG2	46801/334890	0.956	98115	0.758	0.145176	-0.8833	0.1667	1	18	-0.8444	1	0	0
HIP_98116	SICSLYQLE	APGKWQQQG	SICSLYQLEAPGKWQQQG		SCG1	25340/367830	0.979	98116	0.758	-0.158818	-0.5667	0.3333	0	18	-0.6222	0	1	0
HIP_98117	GQVELGGGP	GSEEYRAPR	GQVELGGGPGSEEYRAPR		SCG1	23182/367830	0.984	98117	0.758	-0.115262	-1.1833	0.8333	-1	18	-1.1389	-1	0	0
HIP_98118	GPGAGSLQP	SLIPLIFLI	GPGAGSLQPSLIPLIFLI		SCG2	24261/334890	0.994	98118	0.758	0.010028	1.0333	0.3333	0	18	1.2333	0	0	0
HIP_98119	GERGFFY	SPMPVSQEC	GERGFFYSPMPVSQEC		CMGA	20944/247050	0.969	98119	0.758	0.152236	0.6333	0.3333	-1	16	-0.4812	-1	3	2
HIP_98120	AGSLQPLAL	DLDHPDLF	AGSLQPLALDLDHPDLF		SCG2	29898/334890	0.990	98120	0.758	0.105447	1.0333	0	-2	17	0.2235	-3	0	1
HIP_98121	LCGSHLVE	EAASFQRNQ	LCGSHLVEEAASFQRNQ		SCG2	43234/334890	0.969	98121	0.758	0.014123	0.7667	0	0	17	-0.3824	-1	1	1
HIP_98122	GERGFFYTP	GGGHSRER	GERGFFYTPGGGHSRER		SCG1	24931/367830	0.980	98122	0.758	0.016249	-0.8	0.6667	2	17	-1.4412	1	3	1
HIP_98123	LCGSHLVEA	SDLLMREST	LCGSHLVEASDLLMREST		NPY	6869/49410	0.937	98123	0.758	0.041682	0.3333	0.1667	-1	18	0.2167	-2	0	0
HIP_98124	GIVEQCCTS	PDYNPYQGV	GIVEQCCTSPDYNPYQGV		SCG2	30478/334890	0.990	98124	0.758	-0.059781	-0.9	0.3333	-2	18	-0.4278	-2	1	0
HIP_98125	LYQLENYCN	EERLSKEW	LYQLENYCNEERLSKEW		CMGA	30708/247050	0.907	98125	0.758	0.072675	-2.3	0	-2	17	-1.3706	-2	1	1
HIP_98126	GERGFFYTP	PQRSSQGG	GERGFFYTPPQRSSQGG		SCG1	45558/367830	0.884	98126	0.758	0.025068	-2.2	0.3333	0	17	-1.3118	0	3	1
HIP_98127	HLCGSHLVE	AGTEALPDG	HLCGSHLVEAGTEALPDG		SCG2	21418/334890	0.995	98127	0.758	0.020283	0.8667	0.1667	-1	18	0.0278	-3	0	0
HIP_98128	SLYQLENYC	TEALPDGLS	SLYQLENYCTEALPDGLS		SCG2	28078/334890	0.992	98128	0.758	-0.160682	-0.7833	0	-3	18	-0.2722	-3	1	0
HIP_98129	CTSICSLYQ	SNQLPKAA	CTSICSLYQSNQLPKAA		SCG2	38383/334890	0.980	98129	0.758	0.137934	-1.4667	0.1667	1	17	0.0176	1	1	1
HIP_98130	VNQHLCGSH	SRDNPFKRT	VNQHLCGSHSRDNPFKRT		SCG2	48410/334890	0.950	98130	0.758	-0.018728	-2.2	0.5	4	18	-1.35	2	0	0
HIP_98131	SLYQLENYC	GEEAVPEE	SLYQLENYCGEEAVPEE		CMGA	17196/247050	0.981	98131	0.758	0.047536	-1.6167	0.1667	-5	17	-0.8118	-5	1	1
HIP_98132	GGPGAGSLQ	ECFETLRGD	GGPGAGSLQECFETLRGD		CMGA	16612/247050	0.983	98132	0.758	-0.078190	0.2167	0.1667	-2	18	-0.4944	-2	1	0
HIP_98133	VEALYLVC	APGEEDIQG	VEALYLVCAPGEEDIQG		SCG1	23113/367830	0.984	98133	0.758	0.166177	1.7167	0.3333	-4	17	0.3176	-4	1	1
HIP_98134	LYQLENYC	LGELFNPYY	LYQLENYCLGELFNPYY		SCG1	21565/367830	0.987	98134	0.758	-0.085597	-0.4	0.1667	-2	17	-0.2471	-2	2	1
HIP_98135	GGGPGAGSL	LLMKVLEYL	GGGPGAGSLLLMKVLEYL		SCG2	19164/334890	0.996	98135	0.758	0.152817	2.0167	0.3333	0	18	0.7667	0	0	0
HIP_98136	LVEALYLV	YEENSRDNP	LVEALYLVYEENSRDNP		SCG2	34380/334890	0.986	98136	0.758	0.083239	-0.2667	0	-3	17	-0.5235	-3	2	1
HIP_98137	VEALYLVC	EMIRALEYI	VEALYLVCEMIRALEYI		SCG2	31489/334890	0.989	98137	0.758	0.044927	2.2333	0	-2	17	1.1294	-2	1	1
HIP_98138	EQCCTSICS	DDIYKANNI	EQCCTSICSDDIYKANNI		SCG2	32185/334890	0.988	98138	0.758	0.009449	0.6167	0.1667	-2	18	-0.3167	-2	1	0
HIP_98139	QVGQVELGG	AAMDLELQK	QVGQVELGGAAMDLELQK		SCG1	16208/367830	0.994	98139	0.758	0.120660	1.4167	0.3333	-2	18	-0.0444	-2	0	0
HIP_98140	ERGFFYTP	AVVPQGLFR	ERGFFYTPAVVPQGLFR		CMGA	19434/247050	0.974	98140	0.758	0.026589	1.1	0.1667	1	17	0.0235	1	2	1
HIP_98141	VCGERGFF	QNRMLSKSG	VCGERGFFQNRMLSKSG		SCG2	45520/334890	0.961	98141	0.758	0.107199	-1.05	0.1667	2	17	-0.4824	2	2	1
HIP_98142	VEALYLV	EDVNWGY	VEALYLVEDVNWGY		SCG1	30927/367830	0.962	98142	0.758	0.096744	0.65	0	-3	14	0.2929	-3	1	0
HIP_98143	GERGFFYT	NKGDTEVMK	GERGFFYTNKGDTEVMK		CMGA	28585/247050	0.926	98143	0.758	0.161080	-1.1667	0.1667	0	17	-1.0882	0	3	1
HIP_98144	VEALYLVC	EKVLPRLPY	VEALYLVCEKVLPRLPY		SCG2	43518/334890	0.968	98144	0.758	0.211351	1.2167	0	0	17	0.6412	0	1	1
HIP_98145	EAEDLQVGQ	KRQYDRVAQ	EAEDLQVGQKRQYDRVAQ		SCG1	52891/367830	0.827	98145	0.758	0.006921	-1.9333	0.1667	-1	18	-1.4889	-1	1	0
HIP_98146	GIVEQCCTS	DWMRIILEA	GIVEQCCTSDWMRIILEA		SCG2	25567/334890	0.993	98146	0.758	-0.007564	-0.25	0.1667	-2	18	0.4944	-2	1	0
HIP_98147	QHLCGSHLV	QGLVDREKG	QHLCGSHLVQGLVDREKG		CMGA	21055/247050	0.968	98147	0.758	0.155816	0.7833	0.1667	2	18	-0.4722	0	0	0
HIP_98148	QVELGGGPG	FQDKQYSSH	QVELGGGPGFQDKQYSSH		SCG1	18901/367830	0.991	98148	0.758	0.042256	-1.1	0.5	0	18	-1.1056	-1	1	0
HIP_98149	SHLVEALY	QGGSLPSEE	SHLVEALYQGGSLPSEE		SCG1	27263/367830	0.974	98149	0.758	0.196503	0	0.3333	-2	17	-0.3471	-3	1	1
HIP_98150	LYQLENYC	QMAYENLND	LYQLENYCQMAYENLND		SCG2	26297/334890	0.993	98150	0.758	-0.160831	-0.35	0	-3	17	-0.8412	-3	2	1
HIP_98151	LQPLALEGS	DFYDSEEPV	LQPLALEGSDFYDSEEPV		SCG1	18059/367830	0.992	98151	0.758	0.047872	-1.1167	0.3333	-5	18	-0.4056	-5	2	0
HIP_98152	ALYLVCGER	EESEESNVS	ALYLVCGEREESEESNVS		SCG1	39200/367830	0.923	98152	0.758	-0.128756	-2.7	0.3333	-4	18	-0.5167	-4	0	0
HIP_98153	CGERGFFY	NVSMASLG	CGERGFFYNVSMASLG		SCG1	34786/367830	0.946	98153	0.758	0.152587	0.7	0.1667	0	16	0.2625	0	3	0
HIP_98154	QVGQVELGG	SVFQELGKL	QVGQVELGGSVFQELGKL		SCG2	9119/334890	0.999	98154	0.758	0.013800	1.5333	0.5	-1	18	0.1667	-1	1	0

[illegible]

HIP_98613	SLYQLENYC	VHSSNNFGA	SLYQLENYCVHSSNNFGA		IAPP	4311/45018	0.949	98613	0.758	-0.175604	-0.35	0.1667	0	18	-0.4	-1	1	0	0
HIP_98614	LVEALYLV	PQESKGQP	LVEALYLVPQESKGQP		SCG1	34479/367830	0.947	98614	0.758	0.200838	-0.3167	0.1667	-1	16	-0.125	-1	1	0	0
HIP_98615	VEALYLVCG	KHMQFPPMY	VEALYLVCGKHMQFPPMY		SCG2	43106/334890	0.969	98615	0.758	0.032447	0.1833	0.1667	1	18	0.3667	0	1	0	0
HIP_98616	RGFFYTPKT	RKQASAIKK	RGFFYTPKTRKQASAIKK		SCG1	64486/367830	0.699	98616	0.758	0.060105	-3.0167	0.1667	6	18	-1.1056	6	1	0	0
HIP_98617	LQPLALEGS	EYNYDWWEK	LQPLALEGSEYNYDWWEK		SCG1	29970/367830	0.966	98617	0.758	-0.025098	-2.1667	0.3333	-3	18	-1.05	-3	2	0	0
HIP_98618	CTSICSLYQ	SEESNVSM	CTSICSLYQSEESNVSM		SCG1	36546/367830	0.937	98618	0.758	0.130225	-1.4667	0.1667	-2	17	-0.0353	-2	1	1	0
HIP_98619	GSLQPLAL	DIYKANNI	GSLQPLALDIYKANNI		SCG2	38862/334890	0.979	98619	0.758	0.107446	1.5167	0	0	16	0.125	0	1	0	0
HIP_98620	VEALYLVCG	GLSAEPGWQ	VEALYLVCGGLSAEPGWQ		CMGA	9215/247050	0.996	98620	0.758	-0.025017	1.4833	0.5	-2	18	0.5333	-2	1	0	0
HIP_98621	LGGGPGAGS	LIVLSVAL	LGGGPGAGSLIVLSVAL		IAPP	2771/45018	0.981	98621	0.758	0.133991	2.1833	0.3333	0	17	1.5588	0	0	1	1
HIP_98622	QHLCGSHLV	SEEEPEYG	QHLCGSHLVSEEEPEYG		SCG1	32242/367830	0.957	98622	0.758	0.166463	-0.5	0.1667	-2	17	-0.8765	-4	0	1	1
HIP_98623	CCTSICSLY	EEPSSKDV	CCTSICSLYEEPSSKDV		CMGA	21644/247050	0.966	98623	0.758	0.035750	-1.15	0.3333	-2	17	-0.0706	-2	1	1	0
HIP_98624	GERGFFY	TRQRYGKRS	GERGFFYTRQRYGKRS		NPY	7164/49410	0.838	98624	0.758	0.226661	-0.7333	0	4	16	-1.7875	4	4	2	0
HIP_98625	SLYQLENYC	ADTEKWAEG	SLYQLENYCADTEKWAEG		SCG1	32500/367830	0.955	98625	0.758	-0.133539	-0.7833	0	-3	18	-0.9222	-3	1	0	0
HIP_98626	YQLENYCN	YPEEKKEE	YQLENYCNYPEEKKEE		CMGA	23459/247050	0.958	98626	0.758	-0.020520	-1.45	0.1667	-3	16	-2.1875	-3	2	0	0
HIP_98627	ELGGGPGA	LGLSGLTLA	ELGGGPGALGLSGLTLA		NPY	1361/49410	0.993	98627	0.758	-0.016584	1.1667	0.5	-1	17	0.8	-1	0	1	1
HIP_98628	VEQCCTSIC	LEALRQAEN	VEQCCTSICLEALRQAEN		SCG2	29365/334890	0.991	98628	0.758	-0.067046	1.3833	0.1667	-2	18	0.0222	-2	0	0	0
HIP_98629	VEALYLVC	ELESLSAIE	VEALYLVCELESLSAIE		CMGA	20820/247050	0.969	98629	0.758	0.066981	1.2167	0	-4	17	1.0176	-4	1	1	0
HIP_98630	SLYQLENYC	EEDKRNYP	SLYQLENYCEEDKRNYP		SCG1	42047/367830	0.906	98630	0.758	0.073811	-2.1333	0	-2	17	-1.7118	-2	1	1	0
HIP_98631	GPGAGSLQP	LAAVNSMPV	GPGAGSLQPLAAVNSMPV		SCG1	23999/367830	0.982	98631	0.758	0.088488	1.0167	0.1667	0	18	0.4833	0	0	0	0
HIP_98632	SICSLYQL	NLITRQRYG	SICSLYQLNLITRQRYG		NPY	5444/49410	0.919	98632	0.758	-0.049476	0.6333	0	2	17	-0.1118	2	1	1	0
HIP_98633	GSLQPLALE	TRHSEKSQR	GSLQPLALETRHSEKSQR		SCG1	65200/367830	0.689	98633	0.758	0.086024	-1.05	0	2	18	-1.2222	1	0	0	0
HIP_98634	QHLCGSHL	FNPYYDPL	QHLCGSHLFNPYYDPL		SCG1	30484/367830	0.964	98634	0.758	-0.041732	-0.4167	0.3333	1	16	-0.45	-1	2	0	0
HIP_98635	LYQLENYCN	VDLDDISEA	LYQLENYCNVDLDDISEA		SCG2	24789/334890	0.994	98635	0.758	-0.160031	0.3667	0	-5	18	-0.3889	-5	1	0	0
HIP_98636	QVGQVELGG	EEENELTLN	QVGQVELGGEEENELTLN		SCG1	27655/367830	0.973	98636	0.758	-0.099366	-1.25	0.3333	-5	18	-0.7556	-5	0	0	1
HIP_98637	LVEALYLV	TATCATQRL	LVEALYLVTATCATQRL		IAPP	4227/45018	0.952	98637	0.758	0.093984	1.1833	0	0	17	0.9765	0	1	1	0
HIP_98638	SLQPLALEG	QRYGKRSSP	SLQPLALEGQRYGKRSSP		NPY	7736/49410	0.803	98638	0.758	0.022374	-1.5667	0.1667	2	18	-0.9944	2	1	0	0
HIP_98639	AGSLQPLA	PPMYEENSR	AGSLQPLAPPMYEENSR		SCG2	22405/334890	0.995	98639	0.758	0.027164	0.45	0.5	-1	17	-0.7941	-1	1	1	0
HIP_98640	GGPGAGSLQ	EKWAEGGGH	GGPGAGSLQEKWAEGGGH		SCG1	48385/367830	0.864	98640	0.758	-0.076398	-1.4667	0.1667	0	18	-0.9056	-1	1	0	1
HIP_98641	ERGFFYTP	SRGEAGAPG	ERGFFYTPSRGEAGAPG		SCG1	33111/367830	0.953	98641	0.758	-0.012491	-1.55	0.5	0	17	-0.8471	0	2	1	0
HIP_98642	LGGGPGAGS	PMYEENSRD	LGGGPGAGSPMYEENSRD		SCG2	12157/334890	0.998	98642	0.758	-0.036588	-0.0667	0.5	-2	18	-1.0611	-2	1	0	1
HIP_98643	QVELGGGPG	QRYGKRSSP	QVELGGGPGQRYGKRSSP		NPY	4707/49410	0.941	98643	0.758	0.070561	-1.95	0.5	2	18	-1.3056	2	1	0	1
HIP_98644	HLVEALYL	GEAGAPGE	HLVEALYLGEAGAPGE		SCG1	28114/367830	0.972	98644	0.758	0.182839	0.7	0.1667	-2	16	0.2	-3	1	0	0
HIP_98645	GERGFFYT	LNSEKNFP	GERGFFYTLNSEKNFP		SCG2	32262/334890	0.988	98645	0.758	0.029079	0.05	0.1667	0	16	-0.9625	0	3	0	0
HIP_98646	CGERGFFY	VSQECFET	CGERGFFYVSQECFET		CMGA	20676/247050	0.970	98646	0.758	0.112622	0.7	0.1667	-2	16	-0.2813	-2	3	0	0
HIP_98647	CGSHLVEAL	EGMVTRCII	CGSHLVEALEGMVTRCII		SCG1	30838/367830	0.962	98647	0.758	0.013269	0.0167	0.1667	0	18	0.9278	-1	0	0	0
HIP_98648	CTSICSLY	LEWERYRGR	CTSICSLYLEWERYRGR		SCG1	48323/367830	0.865	98648	0.758	0.029260	0.1833	0.1667	1	17	-0.5647	1	2	1	0
HIP_98649	SICSLYQLE	KLYTDDEDD	SICSLYQLEKLYTDDEDD		SCG2	26963/334890	0.992	98649	0.758	-0.147148	-0.7667	0	-5	18	-0.8278	-5	2	0	0
HIP_98650	LVCGERGFF	EKVLPRLP	LVCGERGFFEKVLPRLP		SCG2	41484/334890	0.974	98650	0.758	0.053608	0.3333	0.1667	1	17	0.2353	1	2	1	0
HIP_98651	HLVEALYL	NPHPSLGYK	HLVEALYLNPHPSLGYK		CMGA	28392/247050	0.928	98651	0.758	0.192799	-0.3333	0.1667	2	17	-0.1824	0	1	1	0
HIP_98652	TSICSLYQ	EEGPTVVL	TSICSLYQEEGPTVVL		CMGA	20052/247050	0.972	98652	0.758	0.136326	-1.4	0.1667	-2	16	0.3875	-2	1	0	0
HIP_98653	QPLALEGSL	WQQQGDLQD	QPLALEGSLWQQQGDLQD		SCG1	26259/367830	0.977	98653	0.758	-0.085195	-0.8833	0.3333	-3	18	-0.8389	-3	1	0	1
HIP_98654	VCGERGFF	PGRAGTEA	VCGERGFFPGRAGTEA		SCG2	33110/334890	0.987	98654	0.758	0.137904	-0.2167	0.5	0	16	-0.25	0	2	0	0
HIP_98655	LGGGPGAGS	MARYYSALR	LGGGPGAGSMARYYSALR		NPY	4487/49410	0.946	98655	0.758	0.100623	-0.0333	0.3333	2	18	-0.1056	2	1	0	1

HIP_99124	GSLQPLAL	EWEDSKRW	GSLQPLALEWEDSKRW		CMGA	26505/247050	0.941	99124	0.757	0.125718	0.25	0	-1	16	-0.9125	-1	1
HIP_99125	SHLVEALY	ECFETLRGD	SHLVEALYECFETLRGD		CMGA	24587/247050	0.953	99125	0.757	0.016358	1.0167	0	-2	17	-0.1294	-3	2
HIP_99126	QHLCGSHLV	GAVGLAAVN	QHLCGSHLVGAVGLAAVN		SCG1	32248/367830	0.957	99126	0.757	0.066975	1.7333	0.1667	2	18	0.9167	0	0
HIP_99127	HLCGSHLVE	SQQKEEEEE	HLCGSHLVESQQKEEEEE		CMGA	23438/247050	0.959	99127	0.757	0.107578	-0.55	0.1667	-3	18	-1.4444	-5	0
HIP_99128	EDLQVGQVE	EEEEEEEE	EDLQVGQVEEEEEEEEE		CMGA	21729/247050	0.966	99128	0.757	-0.095036	-2.2167	0	-11	17	-1.9824	-11	0
HIP_99129	EDLQVGQVE	EEEEEEEE	EDLQVGQVEEEEEEEEE		CMGA	21730/247050	0.966	99129	0.757	-0.095036	-2.2167	0	-11	17	-1.9824	-11	0
HIP_99130	EDLQVGQVE	EEEEEEEE	EDLQVGQVEEEEEEEEE		CMGA	21731/247050	0.966	99130	0.757	-0.095036	-2.2167	0	-11	17	-1.9824	-11	0
HIP_99131	GSLQPLAL	DRSSQGGSL	GSLQPLALDRSSQGGSL		SCG1	41735/367830	0.908	99131	0.757	0.245800	0.1	0.1667	0	17	-0.2353	0	0
HIP_99132	GIVEQCCTS	KPDNPGEDA	GIVEQCCTSKPDNPGEDA		NPY	4822/49410	0.938	99132	0.757	-0.026899	-1.3333	0.3333	-3	18	-0.8278	-3	0
HIP_99133	PGAGSLQPL	HPQEESEES	PGAGSLQPLHPQEESEES		SCG1	30423/367830	0.964	99133	0.757	-0.119067	-1.6	0.3333	-3	18	-1.2667	-4	0
HIP_99134	HLCGSHLV	AFLNERKQA	HLCGSHLVAFLNERKQA		SCG1	42350/367830	0.905	99134	0.757	0.019487	2.2	0	3	17	-0.1176	1	1
HIP_99135	GIVEQCCTS	RLENVQKFP	GIVEQCCTSRLENVQKFP		SCG2	51476/334890	0.934	99135	0.757	-0.002380	-0.5333	0.1667	0	18	-0.2722	0	0
HIP_99136	CSLYQLENY	ETQNAFLNE	CSLYQLENYETQNAFLNE		SCG1	44510/367830	0.892	99136	0.757	-0.062033	-2.6667	0	-3	18	-0.7556	-3	1
HIP_99137	GIVEQCCTS	KNFPMDMSD	GIVEQCCTSKNFPMDMSD		SCG2	37854/334890	0.981	99137	0.757	-0.038681	-0.6	0.1667	-2	18	-0.3	-2	1
HIP_99138	VGQVELGGG	IFLISGAEA	VGQVELGGGIFLISGAEA		SCG2	16002/334890	0.997	99138	0.757	0.115703	1.65	0.5	-2	18	1.0056	-2	1
HIP_99139	EALYLVC	SVALNHLKA	EALYLVCSVALNHLKA		IAPP	4578/45018	0.942	99139	0.757	0.079416	2.6167	0.1667	1	16	0.9563	0	1
HIP_99140	HLVEALYLV	NTHPPASL	HLVEALYLVNTHPPASL		CMGA	29497/247050	0.918	99140	0.757	0.180759	-0.1167	0	1	17	0.4588	-1	1
HIP_99141	EQCCTSICS	RMLVKYPEI	EQCCTSICSRMLVKYPEI		SCG2	37600/334890	0.981	99141	0.757	-0.020882	1.2333	0.1667	0	18	0.1278	0	0
HIP_99142	HLVEALYL	EALPDGLSV	HLVEALYLEALPDGLSV		SCG2	30543/334890	0.990	99142	0.757	0.007659	1.4	0	-2	17	0.7765	-3	1
HIP_99143	VGQVELGG	FPNPYNQEK	VGQVELGGFPNPYNQEK		SCG2	22227/334890	0.995	99143	0.757	-0.029512	0.1167	0.5	-1	17	-0.9176	-1	2
HIP_99144	ICSLYQLEN	EAGLPLQVR	ICSLYQLENEAGLPLQVR		CMGA	21380/247050	0.967	99144	0.757	-0.143329	-0.8833	0.1667	-1	18	0.1167	-1	1
HIP_99145	CSLYQLENY	LRKESKDQL	CSLYQLENYLRKESKDQL		SCG2	46756/334890	0.957	99145	0.757	-0.074467	-2.15	0	0	18	-1.1	0	1
HIP_99146	VEALYLVC	RGRGSEEYR	VEALYLVCRGRGSEEYR		SCG1	46239/367830	0.879	99146	0.757	0.220950	0.1833	0.1667	0	17	-0.4647	0	1
HIP_99147	GGGPGAGSL	GTEALPDGL	GGGPGAGSLGTEALPDGL		SCG2	16035/334890	0.997	99147	0.757	-0.089222	-0.3333	0.5	-2	18	0.0278	-2	0
HIP_99148	GPGAGSLQP	ESKDQLSDD	GPGAGSLQPESKDQLSDD		SCG2	33610/334890	0.986	99148	0.757	-0.032607	-1.5833	0.3333	-3	18	-1.2389	-3	0
HIP_99149	ELGGGPGAG	PDFYDSEEP	ELGGGPGAGPDFYDSEEP		SCG1	12801/367830	0.996	99149	0.757	-0.118309	-0.2167	0.5	-5	18	-1	-5	2
HIP_99150	VEALYLVC	DREKGLSA	VEALYLVCDREKGLSA		CMGA	27647/247050	0.933	99150	0.757	0.187663	-0.1667	0	-1	16	0.2812	-1	1
HIP_99151	ERGFFYTP	SSEEKHLE	ERGFFYTPSSEEKHLE		SCG1	32251/367830	0.957	99151	0.757	-0.050980	-1.45	0.5	-1	16	-1.3625	-2	3
HIP_99152	HLCGSHLVE	GHPQEESEE	HLCGSHLVEGHPQEESEE		SCG1	25535/367830	0.979	99152	0.757	0.042711	-0.1167	0.3333	-2	18	-1.1278	-5	0
HIP_99153	PGAGSLQPL	GTEALPDGL	PGAGSLQPLGTEALPDGL		SCG2	17565/334890	0.997	99153	0.757	-0.065798	-0.9833	0.3333	-2	18	0.0222	-2	0
HIP_99154	VCGERGFF	YPGVQAPE	VCGERGFFYPGVQAPE		SCG1	30598/367830	0.963	99154	0.757	0.034579	0.3167	0.5	-1	16	-0.15	-1	3
HIP_99155	HLVEALYL	KKEEEGSAN	HLVEALYLKKEEEGSAN		CMGA	32503/247050	0.890	99155	0.757	0.213946	-0.8333	0	-1	17	-0.6941	-2	1
HIP_99156	GERGFFY	YSALRHYIN	GERGFFYYSALRHYIN		NPY	6371/49410	0.880	99156	0.757	0.118549	0.6667	0.1667	2	16	-0.5625	1	4
HIP_99157	ALYLVCGER	QSSQAELKE	ALYLVCGERQSSQAELKE		CMGA	25578/247050	0.947	99157	0.757	-0.138053	-2.25	0.5	-1	18	-0.4167	-1	0
HIP_99158	EAEDLQVGQ	ALEYIENLR	EAEDLQVGQALEYIENLR		SCG2	12672/334890	0.998	99158	0.757	-0.080498	0.4	0.1667	-4	18	-0.5833	-4	1
HIP_99159	HLVEALYL	LEEPGET	HLVEALYLLEEPGET		SCG1	32976/367830	0.953	99159	0.757	0.098100	0.5167	0	-3	15	0	-4	1
HIP_99160	ELGGGPGAG	QEEVRDSKE	ELGGGPGAGQEEVRDSKE		SCG2	25517/334890	0.993	99160	0.757	-0.133581	-1.5833	0.3333	-3	18	-1.3333	-3	0
HIP_99161	QHLCGSHLV	GMVTRCIIE	QHLCGSHLVGMVTRCIIE		SCG1	32249/367830	0.957	99161	0.757	0.066780	1.75	0.1667	2	18	0.65	0	0
HIP_99162	CSLYQLENY	ILSSTNVGS	CSLYQLENYILSSTNVGS		IAPP	3527/45018	0.968	99162	0.757	-0.101107	-0.1333	0.1667	-1	18	0.0944	-1	1
HIP_99163	GSLQPLAL	AVNSMPVDN	GSLQPLALAVNSMPVDN		SCG1	34979/367830	0.945	99163	0.757	0.022912	1.9833	0	-1	17	0.3588	-1	0
HIP_99164	LYQLENYCN	AWIPHVEN	LYQLENYCNAWIPHVEN		SCG2	26569/334890	0.993	99164	0.757	-0.075887	0.5167	0	-1	17	-0.5118	-2	2
HIP_99165	GFFYTPKT	QWSLYPSDS	GFFYTPKTQWSLYPSDS		SCG1	37640/367830	0.931	99165	0.757	-0.044407	-1.9	0.3333	0	17	-0.7294	0	2
HIP_99166	CGERGFFY	KARRHPQG	CGERGFFYKARRHPQG		SCG1	56893/367830	0.791	99166	0.757	0.166421	-0.3833	0	4	16	-1.3625	3	3
HIP_99167	GSLQPLALE	EDDLQEEEQ	GSLQPLALEEDDLQEEEQ		SCG2	29252/334890	0.991	99167	0.757	-0.003901	-1.4	0	-7	18	-1.1556	-7	0
HIP_99168	RGFFYTPKT	EMIRALEYI	RGFFYTPKTEMIRALEYI		SCG2	36776/334890	0.982	99168	0.757	0.113950	-0.55	0.1667	1	18	-0.2111	1	1
HIP_99169	SLQPLAL	LLGAVGLA	SLQPLALLLGAVGLA		SCG1	31609/367830	0.959	99169	0.757	0.037350	2.7667	0.1667	0	15	1.7133	0	0
HIP_99170	GERGFFY	EEEGSANRR	GERGFFYEEEGSANRR		CMGA	27492/247050	0.934	99170	0.757	0.156233	-1.0333	0	-1	16	-1.6812	-1	3
HIP_99171	AEDLQVGQV	EDSSEEKHL	AEDLQVGQVEDSSEEKHL		SCG1	25965/367830	0.978	99171	0.757	-0.080941	-1.25	0.3333	-4	18	-1.0722	-5	0

HIP_99635	LVEALYLVC	IEQAIKEH	LVEALYLVCIEQAIKEH		SCG2	31214/334890	0.989	99635	0.757	0.083697	1.3333	0	-1	17	0.7353	-2	1	1	0
HIP_99636	SICSLYQL	IVLSVAL	SICSLYQLIVLSVAL		IAPP	3982/45018	0.958	99636	0.757	0.000593	1.9167	0	0	15	1.98	0	1	1	0
HIP_99637	EQCCTSIC	GEQEHSQQK	EQCCTSICGEQEHSQQK		CMGA	19946/247050	0.972	99637	0.757	0.145741	-0.2	0.3333	-1	17	-1.3118	-2	0	1	0
HIP_99638	QVELGGGPG	IAYEDVVGG	QVELGGGPGIAYEDVVGG		SCG2	13372/334890	0.998	99638	0.757	0.112406	0.4333	0.5	-3	18	0.1889	-3	1	0	1
HIP_99639	GIVEQCCTS	ERGEDSSEE	GIVEQCCTSERGEDSSEE		SCG1	29237/367830	0.968	99639	0.757	-0.031775	-1.2333	0.3333	-5	18	-1.0667	-5	0	0	0
HIP_99640	GGPGAGSLQ	RRTRPRHHH	GGPGAGSLQRRTRPRHHH		SCG1	65358/367830	0.687	99640	0.757	0.061674	-1.7	0.1667	7	18	-1.7667	4	0	0	1
HIP_99641	VEALYLVC	GEETGSQE	VEALYLVCGEETGSQE		SCG1	29606/367830	0.967	99641	0.757	0.156817	0.5167	0.1667	-4	16	-0.05	-4	1	0	0
HIP_99642	LGGGPGAGS	TALPVNSPM	LGGGPGAGSTALPVNSPM		CMGA	13753/247050	0.989	99642	0.757	0.091180	0.9167	0.3333	0	18	0.2611	0	0	0	1
HIP_99643	LGGGPGAGS	EEMAVVPQG	LGGGPGAGSEEMAVVPQG		CMGA	16511/247050	0.983	99643	0.757	-0.095025	-0.75	0.3333	-2	18	0.0444	-2	0	0	1
HIP_99644	CGSHLVEA	TSYFPNPYN	CGSHLVEATSYFPNPYN		SCG2	40721/334890	0.975	99644	0.757	0.078847	-0.05	0.1667	0	17	-0.4176	-1	2	1	0
HIP_99645	VEALYLVC	LAAVNSMPV	VEALYLVCLAAVNSMPV		SCG1	36694/367830	0.937	99645	0.757	0.019266	2.9833	0	-1	17	1.6059	-1	1	1	0
HIP_99646	VEQCCTSIC	GGKSGELEQ	VEQCCTSICGGKSGELEQ		CMGA	18086/247050	0.979	99646	0.757	0.144043	0.25	0.5	-2	18	-0.2722	-2	0	0	0
HIP_99647	IVEQCCTSI	SGEETGSQE	IVEQCCTSISGEETGSQE		SCG1	15458/367830	0.994	99647	0.757	-0.022242	-0.2833	0.5	-4	18	-0.4111	-4	0	0	0
HIP_99648	IVEQCCTSI	ESGEETGSQ	IVEQCCTSIESGEETGSQ		SCG1	15459/367830	0.994	99648	0.757	-0.022242	-0.2833	0.5	-4	18	-0.4111	-4	0	0	0
HIP_99649	GPGAGSLQP	VMEKREDSK	GPGAGSLQPVMEKREDSK		CMGA	23010/247050	0.960	99649	0.757	0.012973	0.2167	0.1667	0	18	-1.1444	0	0	0	0
HIP_99650	IVEQCCTSI	RLQNGQNGE	IVEQCCTSIRLQNGQNGE		SCG2	43340/334890	0.968	99650	0.757	-0.048429	-0.2	0.1667	-1	18	-0.5167	-1	0	0	0
HIP_99651	GSLQPLALE	QAEGDSEGL	GSLQPLALEQAEGDSEGL		CMGA	26093/247050	0.944	99651	0.757	-0.035264	-0.5167	0	-4	18	-0.3667	-4	0	0	0
HIP_99652	GSLQPLAL	YPEEKKEE	GSLQPLALYPEEKKEE		CMGA	23978/247050	0.956	99652	0.757	0.165182	0.5	0.1667	-2	16	-1.1125	-2	1	0	0
HIP_99653	SHLVEALY	KLAPVSKRF	SHLVEALYKLAPVSKRF		SCG2	45302/334890	0.962	99653	0.757	0.067548	1	0	3	17	0.1588	2	1	1	0
HIP_99654	IVEQCCTSI	RQAENEPQS	IVEQCCTSIRQAENEPQS		SCG2	42395/334890	0.971	99654	0.757	-0.065851	-0.5333	0.1667	-2	18	-0.7167	-2	0	0	0
HIP_99655	HLVEALYLV	FSEDVNW	HLVEALYLVFSEDVNW		SCG1	33933/367830	0.949	99655	0.757	0.148302	0.8667	0.1667	-2	16	0.525	-3	2	2	0
HIP_99656	SHLVEALY	DDYETQQWP	SHLVEALYDDYETQQWP		SCG2	36122/334890	0.983	99656	0.757	0.111630	-0.6667	0	-3	17	-1.0118	-4	2	1	0
HIP_99657	GSLQPLAL	KNEQINDEM	GSLQPLALKNEQINDEM		SCG2	35958/334890	0.984	99657	0.757	0.194762	-0.25	0	-2	17	-0.6824	-2	0	1	0
HIP_99658	QCCTSICSL	QEEERLSK	QCCTSICSLQEEERLSK		CMGA	26049/247050	0.944	99658	0.757	0.088756	-0.8333	0.1667	-1	17	-0.5529	-1	0	1	1
HIP_99659	GERGFFYT	KEPQLRLEN	GERGFFYTKEPQLRLEN		SCG2	39832/334890	0.977	99659	0.757	0.112277	-1.3667	0.1667	-1	17	-1.2706	-1	3	1	0
HIP_99660	IVEQCCTSI	SQEESEEGE	IVEQCCTSISQEESEEGE		SCG1	24507/367830	0.981	99660	0.757	-0.083882	-0.8	0.3333	-6	18	-0.7389	-6	0	0	0
HIP_99661	SLYQLENYC	SGEATDGA	SLYQLENYCSGEATDGA		CMGA	16444/247050	0.983	99661	0.757	0.088522	-1.1667	0.3333	-3	17	-0.5588	-3	1	1	0
HIP_99662	SLYQLENYC	LGAALSLIP	SLYQLENYCLGAALSLIP		SCG2	20647/334890	0.996	99662	0.757	-0.152925	0.4833	0.1667	-1	18	0.7167	-1	1	0	0
HIP_99663	AEDLQVGQV	GESRSEALA	AEDLQVGQVGESRSEALA		CMGA	12178/247050	0.992	99663	0.757	-0.040222	-0.7333	0.5	-3	18	-0.3611	-3	0	0	0
HIP_99664	DLQVGQVEL	TLLLSLLGA	DLQVGQVELTLLLSLLGA		SCG1	32747/367830	0.954	99664	0.757	-0.028436	1.9	0	-2	18	1.1389	-2	0	0	1
HIP_99665	SLYQLENYC	MRSAAVLAL	SLYQLENYCMRSAAVLAL		CMGA	28189/247050	0.929	99665	0.757	-0.029842	-0.95	0.1667	0	18	0.5556	0	1	0	0
HIP_99666	SHLVEALY	SEAHESSSR	SHLVEALYSEAHESSSR		SCG1	38743/367830	0.925	99666	0.757	0.197007	0.3	0.1667	0	17	-0.6647	-2	1	1	1
HIP_99667	VEALYLVC	FGAILSS	VEALYLVCFGAILSS		IAPP	3914/45018	0.960	99667	0.757	0.039475	2.45	0.1667	-1	15	1.76	-1	2	1	0
HIP_99668	EQCCTSICS	SLGEKRDHH	EQCCTSICSSLGEKRDHH		SCG1	23320/367830	0.984	99668	0.757	0.057256	1.4667	0.5	1	18	-0.9167	-1	0	0	0
HIP_99669	VNQHLCGSH	EKKEEEGSA	VNQHLCGSHEKKEEEGSA		CMGA	34556/247050	0.867	99669	0.757	-0.073560	-2.6167	0.3333	0	18	-1.4056	-2	0	0	1
HIP_99670	GGPGAGSLQ	EAEAGEEAV	GGPGAGSLQEAEAGEEAV		CMGA	21429/247050	0.967	99670	0.757	-0.166795	-0.95	0.1667	-4	18	-0.3722	-4	0	0	0
HIP_99671	GAGSLQPLA	AVDGAGKPG	GAGSLQPLAAVDGAGKPG		CMGA	22405/247050	0.963	99671	0.757	-0.044581	1.0833	0.1667	0	18	0.1167	0	0	0	0
HIP_99672	EQCCTSICS	EKNEQINDE	EQCCTSICSEKNEQINDE		SCG2	33832/334890	0.986	99672	0.757	0.083835	-0.7833	0.1667	-4	18	-1.1778	-4	0	0	0
HIP_99673	CSLYQLENY	LVARSETHA	CSLYQLENYLVARSETHA		SCG1	29459/367830	0.967	99673	0.757	0.027224	0.25	0	0	18	-0.2722	-1	1	0	0
HIP_99674	ALYLVCGER	KLSFRARAY	ALYLVCGERKLSFRARAY		CMGA	28622/247050	0.926	99674	0.757	-0.022345	-1.55	0.3333	3	18	0.0889	3	1	0	0
HIP_99675	ELGGGPGAG	EEDIQGPTK	ELGGGPGAGEEDIQGPTK		SCG1	29936/367830	0.966	99675	0.757	-0.106571	-1.5833	0.3333	-3	18	-0.9778	-3	0	0	1
HIP_99676	EALYLVCGE	DNRNHNEGM	EALYLVCGEDNRNHNEGM		SCG1	47068/367830	0.874	99676	0.757	-0.084132	-2.15	0.1667	-2	18	-0.9056	-3	0	0	0
HIP_99677	RGFFYTPKT	MDLELQKIA	RGFFYTPKTMDLELQKIA		SCG1	39672/367830	0.920	99677	0.757	0.108141	-0.6667	0.1667	1	18	-0.3389	1	1	0	0
